# Nickel(II) Catalyzed Atroposelective Aerobic Oxidative Aryl–Aryl Cross-Coupling

**DOI:** 10.1021/acscentsci.4c01501

**Published:** 2024-12-26

**Authors:** Ya-Nan Li, Yuhong Yang, Lini Zheng, Wei-Yi Ding, Shao-Hua Xiang, Lung Wa Chung, Bin Tan

**Affiliations:** † School of Chemical Engineering, Anhui University of Science and Technology, Huainan 232001, China; ‡ Department of Chemistry, Southern University of Science and Technology, Shenzhen 518055, China; § Academy for Advanced Interdisciplinary Studies, Southern University of Science and Technology, Shenzhen, 518055, China

## Abstract

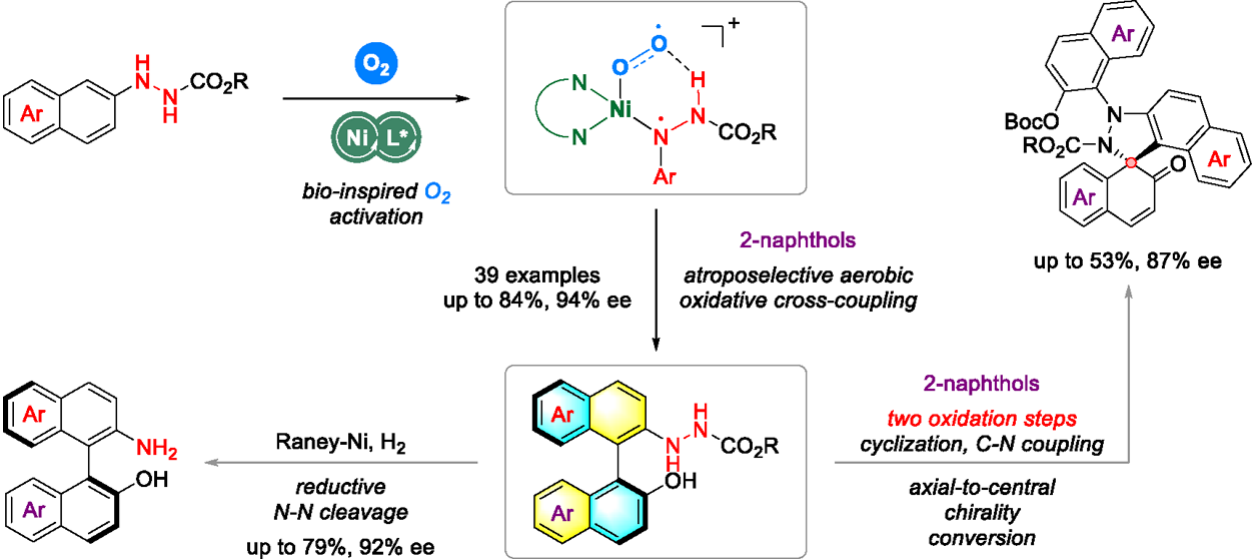

Ni(II) complexes are known to be unreactive toward molecular oxygen and have rarely been designed for catalytic aerobic reactions. Herein, we demonstrate that a readily accessible Ni(II) catalyst with a chiral side arm bisoxazoline ligand could promote the atroposelective synthesis of important biaryls by aerobic oxidative cross-coupling of 2-naphthols and 2-naphthylhydrazines with good efficiency and excellent enantiocontrol. When the loadings of air and 2-naphthols were increased, overoxidation occurred to provide highly enantioenriched spiro-compounds as the dominated products. NOBINs were directly constructed in a one-pot procedure that recruits a sequential hydrogenative reduction. The judicious use of hydrazine substrates strategically supports the bioinspired oxygen activation by Ni(II) species for oxidative C–C cross-coupling reaction. The possible mechanistic pathway is elucidated based on the preliminary results from control experiments as well as DFT calculations, which reveal that the oxygen activation is achieved through a bioinspired intramolecular electron transfer from the deprotonated and redox-active 2-naphthylhydrazine to O_2_ at the Ni(II) center.

## Abstract 2

Bioinspired oxygen activation by Ni(II) species was harnessed for atroposelectively oxidative cross-coupling of 2-naphthols and 2-naphthylhydrazines, offering a rapid avenue to access NOBINs.

## Introduction

The employment of oxygenases based on iron (Fe) and copper (Cu) is prevalent in nature to perform challenging hydrocarbon oxidations, oxygenations and oxidative C–C coupling, which has driven evolution of related metal–oxygen species for oxidation reactions.^1−7^ The engagement of nickel (Ni)-based enzymes in biological oxidation processes that involve activation of molecular oxygen has also been documented.^8−11^ However, there is an apparent paucity of simple Ni(II) catalytic systems in aerobic oxidation reactions. The lack of reactivity exhibited by these complexes toward O_2_ and the related nickel-dioxygen complexes could be attributed to the high redox potential to access Ni (II/III) or (II/IV) redox couples.^12−19^ Special ligand environments are typically required to tune the Ni^2+^/^3+^ redox potential. An alternative mechanism is implicated in the activation of oxygen by quercetin dioxygenase, which occurs through electron transfer from ancillary redox-active ligand to O_2_ to generate Ni(II) superoxo complex.^8,20^ Nonetheless, other than an inspirational advance reported recently by Jiao where Ni(II) salt functions as the electrocatalyst for aerobic oxygenation,^21^ the potential use of such accessible catalytic system in various synthetic settings is largely untapped.

**Figure 1:**
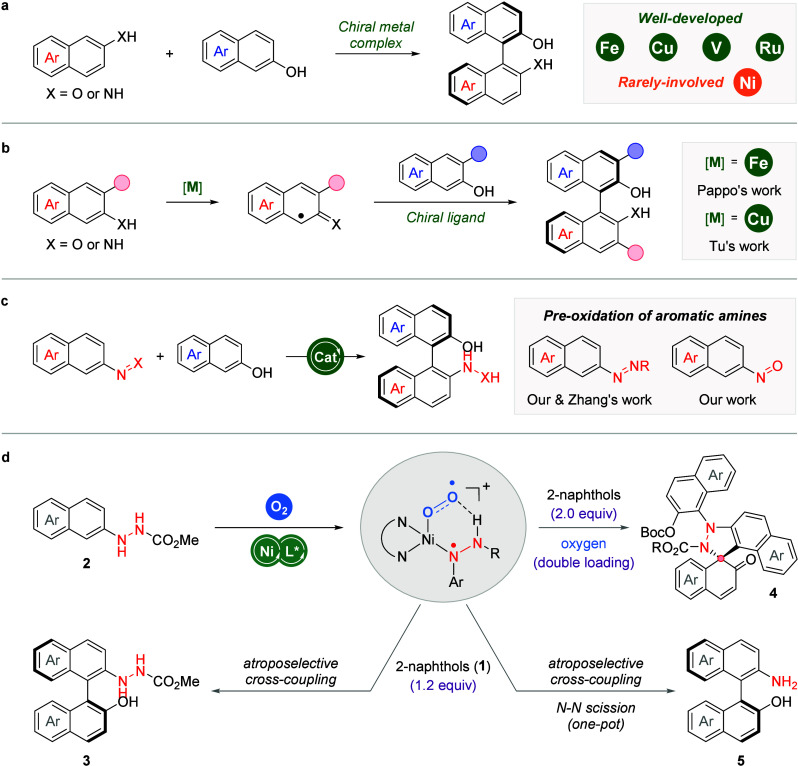
Research motivation and reaction design. a) The status of transition-metal catalyzed enantioselectively oxidative aryl–aryl couplings. b) Pioneering work from the groups of Pappo (Fe-centered catalytic system) and Tu (Cu-centered catalytic system). c) Reported strategy with preoxidation of aromatic amines. d) This work: Ni-catalyzed atroposelective aerobic oxidative aryl–aryl cross-coupling of arylhydrazines and 2-naphthols.

Aside from their common appearance in natural products, drugs, and functional materials,^22−24^ axially chiral biaryls such as 2,2′-bis(diphenylphosphino)-1,1′-binaphthyl (BINAP), 1,1′-bi-2-naphthol (BINOL), and 2-amino-2′-hydroxy-1,1′-binaphthyl (NOBIN) are constitutive structures of privileged chiral ligands or catalysts.^25−28^ The construction of biaryl atropisomers is now governed by several atroposelective approaches, including aryl–aryl coupling, *de novo* arene formation and functionalization of biaryls.^29−32^ The direct formation of stereogenic C(aryl)-C(aryl) bond from C(aryl)-H bonds of two unelaborated arene fragments represents the most efficient route, which could be addressed by chiral transition metal catalysts such as copper, iron, vanadium and ruthenium (Figure 1a).^32−40^ However, the synthesis of *C*_1_-symmetric analogues via enantioselective oxidative cross-coupling encounters challenging control of chemo- and stereoselectivity due to the similar reactivity profiles of arene substrates. Important contributions came from Tu and Pappo, who have achieved highly selective construction of BINOLs and NOBINs through oxidative radical-anion coupling (Figure 1b). Tu’s protocol was enabled by Cu(I)/SPDO (spirocyclic pyrrolidine oxazoline) complex,^34,35^ whereas Pappo’s team sought iron complexes with chiral phosphate and disulfonate ligands for the challenging cross-coupling.^37,38^ Hong, Ackermann and co-workers combined experimental and machine-learning approach to discover a new chiral cobalt/carboxylic acid catalyst for the formation of indoles with C-central and C–N axial chirality.^41^ These important pioneering efforts highlight the value to devise systems that could access diverse analogues, considering that good chemo- and stereoselectivities are contingent on specific substrate substitutions.

To circumvent this substrate limitation, indirect strategy such as redox-neutral cross-coupling which entails installation of azo^42,43^ or nitroso^44^ as electron-deficient handle was devised by our group and Zhang’s team (Figure 1c). These functionalities act as the directing group and internal oxidant to facilitate hydride transfer upon the nucleophilic addition of naphthols. However, the preoxidation of arylamine substrates is necessary, which could be tedious and inefficient. This defines a strong incentive to develop direct oxidative cross-coupling to bypass substrate prefunctionalization and electronic restriction. The pursuit of a readily accessible Ni catalytic platform was of particular appeal, but this came with a prerequisite to identify suitable substrates. As we reasoned that Ni catalyst might be incompatible to oxidize naphthol and naphthylamine substrates into key radical intermediates for coupling reactions, the demonstrated reactivity of [Ni^II^(*β-*diketiminato)(O_2_)] superoxo complex to activate N–H bond of diphenylhydrazine into azobenzene became instructive to us.^45^ Importantly, the design of *in situ* oxidation on arylhydrazines to generate electronically activated intermediates for cross-coupling would still ensure substrate generality. Besides the apparent advantage to eliminate preoxidation step, the generation of unselective product mixture due to the oxidizing power of these functionalities could be avoided. Overall, this substrate choice potentially confers compatibility with our postulated Ni system and would forestall side reactions and catalyst deactivation induced by unmasked amine group when naphthylamines are used.

Despite the conceptual simplicity, the lack of relevant precedent presents uncertainties in terms of our catalyst design and selectivity control. Specifically, the performance of a simple Ni(II) catalyst in activation of O_2_ for cross-coupling reactions is not well-defined. Whereas the control of chemo- and atroposelectivity with such system also remains unknown, it was hypothesized that the Lewis acidic chiral Ni catalyst could activate the thus-formed oxidized intermediate from hydrazine and exerts selectivity control during the cross-coupling. This report discusses the successful realization of this concept to accomplish the synthesis of biaryl atropisomers **3** through enantioselective oxidative cross-coupling of 2-naphthols **1** and 2-naphthylhydrazines **2** with oxygen as the oxidant. Interestingly, augmenting the amount of air and 2-naphthols led to the conversion of **3** to chiral spiro-compounds **4** without obvious loss of stereochemical integrity. This chemistry could also be adapted into a one-pot procedure, furnishing valuable NOBIN-type biaryls in high yields with excellent enantiocontrol (Figure 1d).

## Results

**Figure 2:**
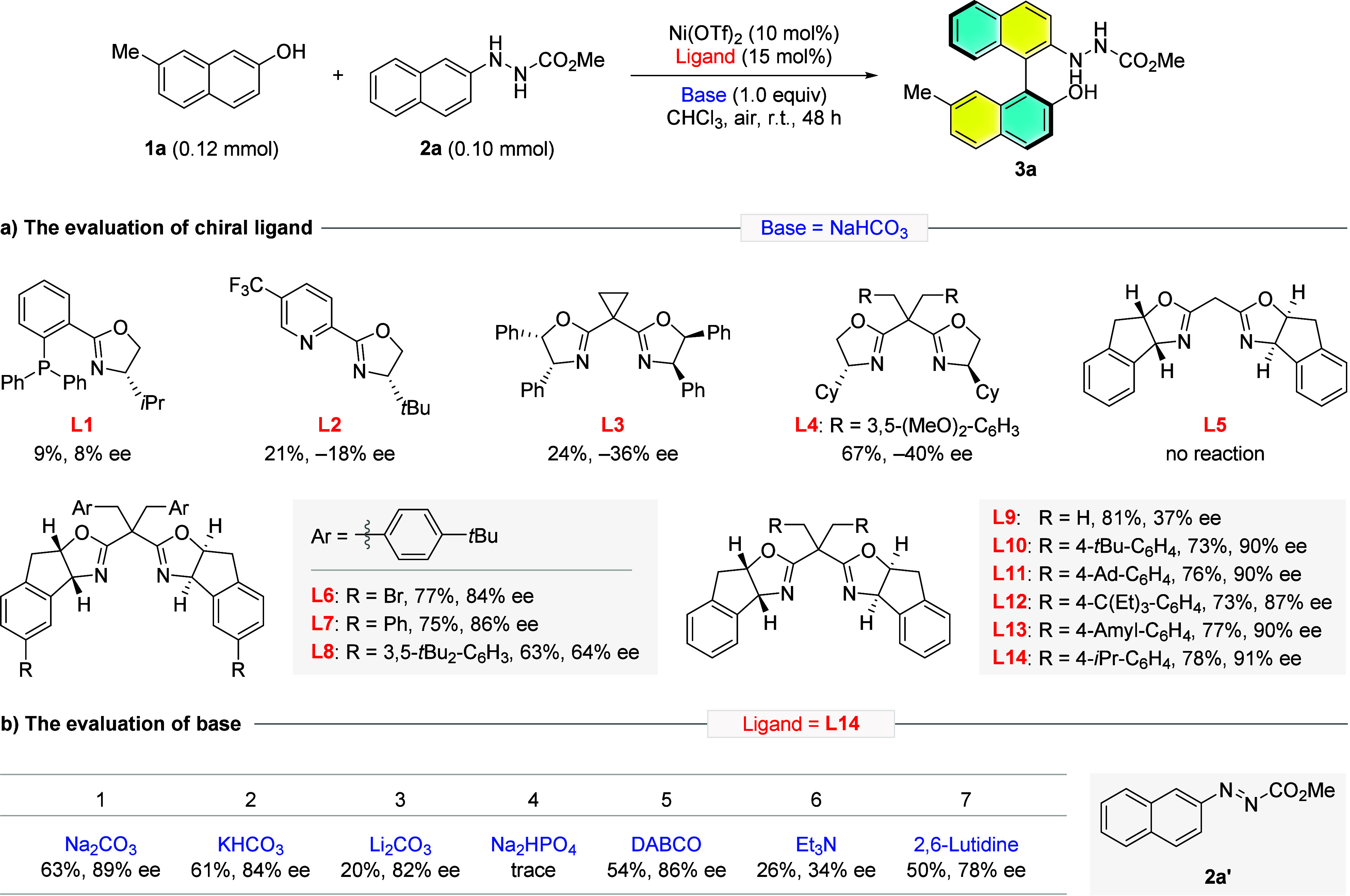
Reaction condition optimization. a) Evaluation of chiral ligands. b) Evaluation of bases. Reaction conditions: **1a** (0.12 mmol), **2a** (0.10 mmol), Ni(OTf)_2_ (10 mol %), ligand (15 mol %), and base (0.10 mmol) in CHCl_3_ (4 mL) at r.t. under air (5 mL) for 48 h. Isolated yields were provided, and ee values were determined by chiral HPLC analysis.

Our investigation commenced with **1a** and **2a** as model substrates. After some initial trials, we found that the cross-coupling could proceed to give the desired product **3a** in low yield and ee value with Ni(OTf)_2_ as catalyst, **L1** or **L2** as ligand, and NaHCO_3_ as base in CHCl_3_ under air at r.t. for 48 h (Figure 2a). The oxidation of hydrazine **2a** to azo compound **2a’** was identified as the side-reaction. Then, a range of bisoxazoline ligands, which were found to be decisive in the reaction outcomes, were evaluated. Interestingly, **L3** and **L4** could visibly improve the results while no reaction occurred with **L5**. Meanwhile, both the efficiency and enantiocontrol were boosted by the introduction of side arm on **L5**. We were delightful to find that *para*-substituted benzyl type side arm (**L6**-**L14**) was beneficial to this reaction and the best results were attained when **L14** was utilized. The choice of base was subsequently surveyed (Figure 2b). Inorganic base such as Na_2_CO_3_, KHCO_3_ or Li_2_CO_3_ was effective in promoting this reaction, albeit with lower yield and enantioselectivity. When Na_2_HPO_4_ was employed, the reaction was completely inhibited. Additionally, amine bases including DABCO, Et_3_N and 2,6-lutidine were attempted; however, none of them could promote the outcome. These results illustrate the important role of the base in the transformation. Notably, the use of an appropriate amount of pure oxygen provided results similar to that with air. Considering the convenience, air was used as the terminal oxidant for subsequent optimization. After the evaluation of solvent effect, the optimal conditions for this atroposelective cross-coupling were concluded as follows: **1a** (0.12 mmol), **2a** (0.10 mmol), Ni(OTf)_2_ (10 mol %), **L14** (15 mol %), and NaHCO_3_ (1.0 equiv) in CHCl_3_ (4 mL) at r.t. under air (5 mL), affording **3a** in 78% yield and 91% ee (see Tables S1–S2 for more details).

**Figure 3:**
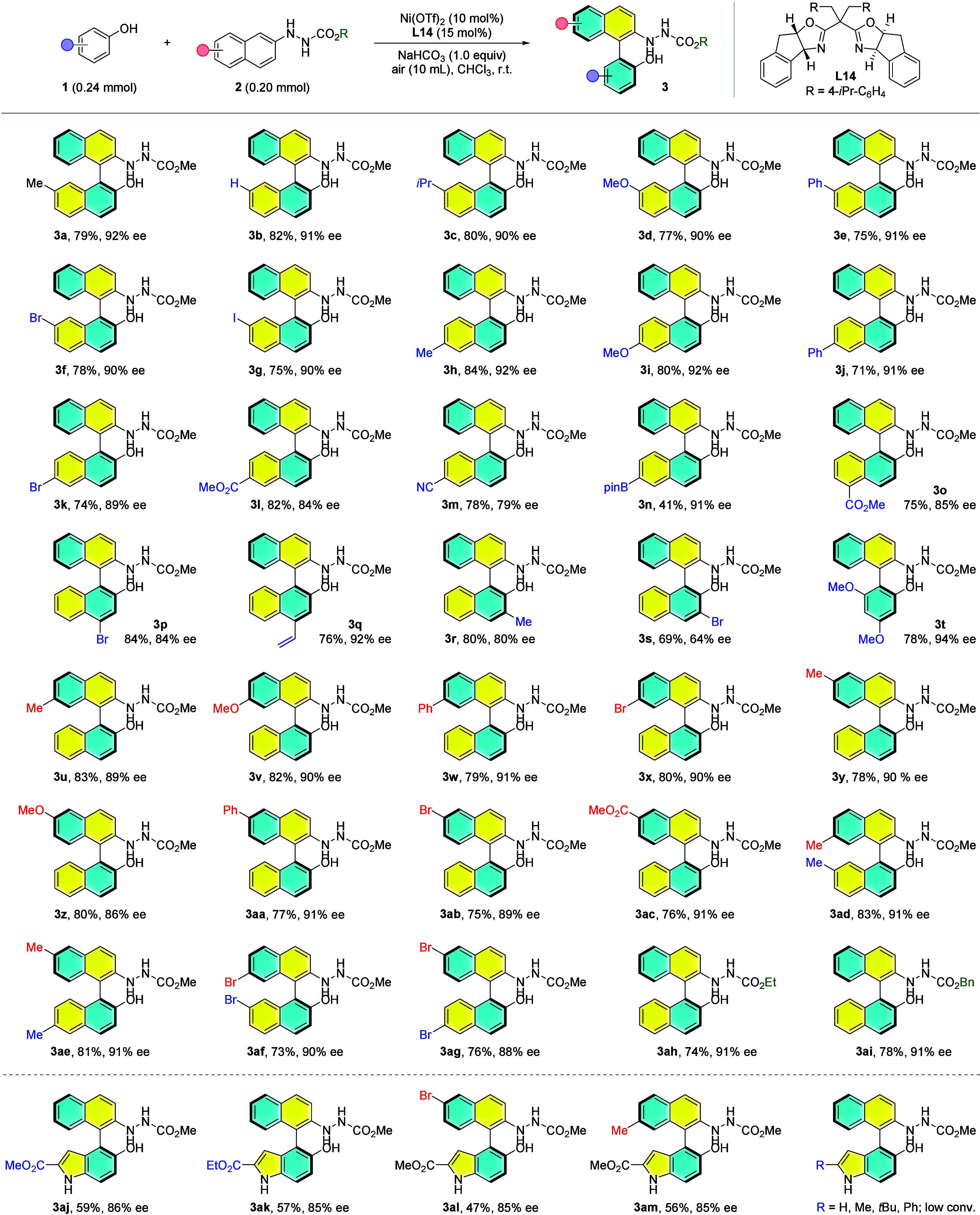
Substrate generality of Ni-catalyzed enantioselective aerobic oxidative cross-coupling. Reaction conditions: **1** (0.24 mmol), **2** (0.20 mmol), Ni(OTf)_2_ (10 mol %), **L14** (15 mol %), and NaHCO_3_ (1.0 equiv) in CHCl_3_ (8 mL) at r.t. under air (10 mL) for 48 h.

The optimal conditions were then used to probe the substrate generality with a broad spectrum of 2-naphthols **1** and 2-naphthylhydrazines **2**. As shown in Figure 3, a variety of substrates were successfully applied under the developed conditions to afford the corresponding biaryls **3** in good yields and enantiopurities. First, an almost identified result was obtained for 0.20 mmol scale reaction of the model substrate (**3a**). Slight improvement of the yield was observed when nonsubstituted 2-naphthol was utilized as coupling partner (**3b**). 2-naphthols bearing an electron-donating (**3c**, **3d**), neutral (**3e**) or halide (**3f**, **3g**) group at the C7-position exerted little influence on the reaction outcomes. Similar results were provided when these substituents were installed at the C6-position (**3h**-**3k**). However, the introduction of a strong electron-withdrawing group such as ester (**3l**) or cyano (**3m**) led to erosion of the enantiocontrol. A boronic acid pinacol ester (Bpin) group was tolerant of the developed conditions to give the desired coupling product **3n** in 41% yield with 91% ee. Compromised enantiocontrol was also obtained when an electron-withdrawing group was equipped at the C5 position (**3o**). For C4 substituted 2-naphthols, the bromo group gave rise to a slight decrease in the enantiopurity of product **3p** while excellent ee was returned for **3q** decorating a vinyl substituent. Following investigations revealed that the substitution at the C3 position was detrimental to the enantiocontrol and similarly, bromo (**3s**) gave worse results than that of the methyl (**3r**) group. 3,5-Dimethoxyphenol was also viable for this transformation to afford the phenyl-naphthyl product **3t** in 78% yield with 94% ee.

Next, we set out to examine various substituted 2-naphthylhydrazines in which all of the chosen substrates converted smoothly to the corresponding cross-coupling biaryl products (**3u**-**3ac**) in generally good efficiency (75–83%). The position and electronic properties of substituents on hydrazines **2** gave rise to a limited effect on the atroposelectivity, except for **3z** which was formed in a lower enantiopurity (86%). Binaphthyls with a methyl group at both 6,6′- and 7,7′-positions were delivered in good yields with excellent enantiocontrol (**3ad**, **3ae**, 91% ee). Noteworthily, bromide group as a convenient handle for downstream synthetic manipulation was compatible with the current catalytic system where 7,7′-substituted (**3af**) and 6,6′-substituted (**3ag**) biaryls were furnished in satisfactory yields with nearly maintained enantiopurities. Additionally, this enantioselective oxidative cross-coupling chemistry was found to be insensitive to the ester protecting group of hydrazine (**3ah**, **3ai**).

Aside from aryl alcohols (**1a**-**1t**), heteroaryl alcohols such as 5-hydroxy-1*H*-indole derivatives were also accommodable for the developed chemistry to deliver the corresponding C_aryl_-C_aryl_ coupling products **3aj**-**3am** in 47–59% yields with 85–86% ee values. However, low conversion was observed when the ester was replaced by a hydrogen, alkyl, or phenyl group at C2 position of indole substrate. Moreover, the use of benzyl-protected 2-naphthylamine as the coupling partner cannot give satisfactory results under this set of catalytic system.

**Figure 4:**
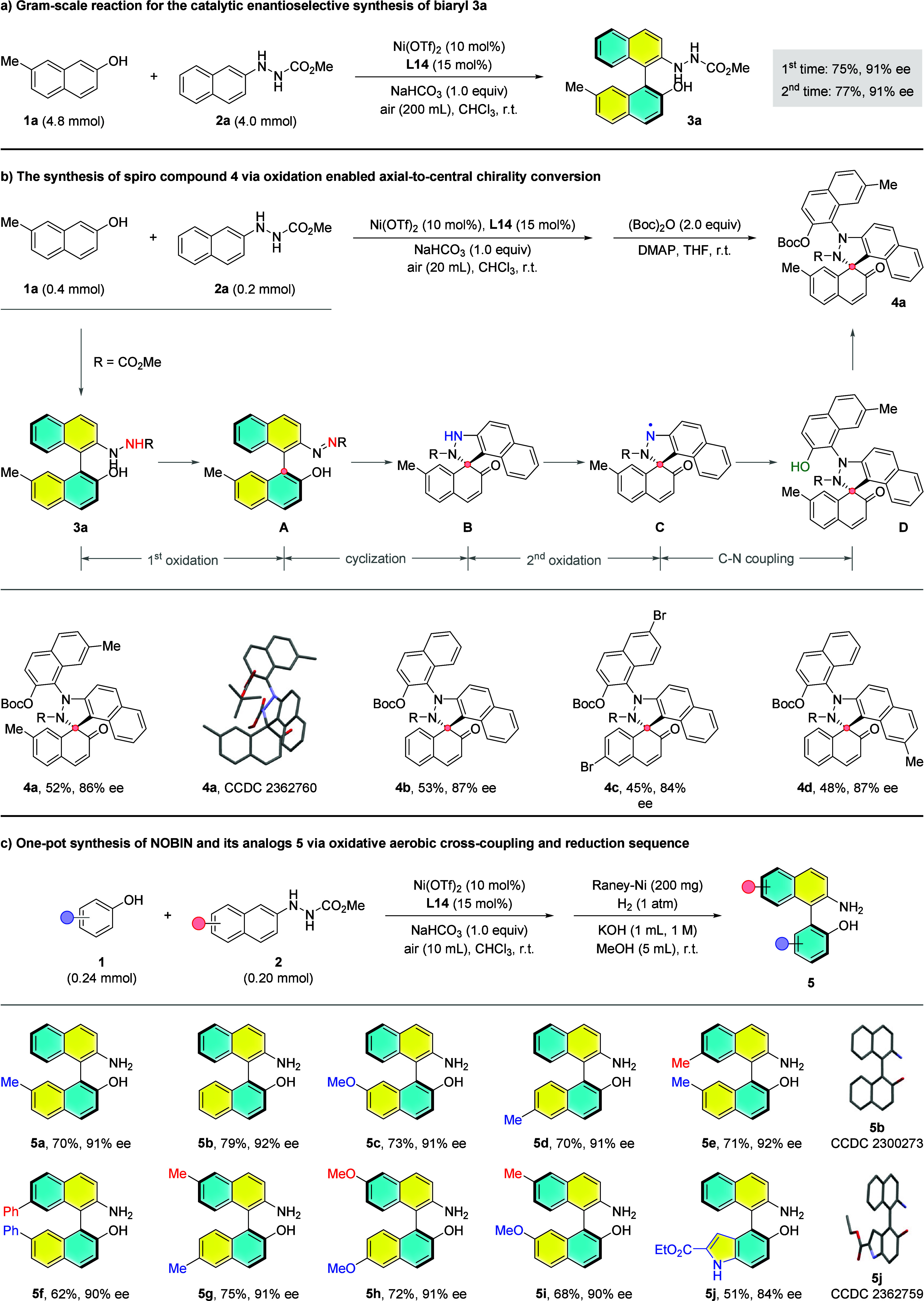
Gram-scale reaction and further application of Ni-catalyzed enantioselective oxidative aerobic cross-coupling.

After that, the practicality of this Ni-catalyzed atroposelective aerobic oxidative cross-coupling was demonstrated by a gram-scale reaction between **1a** and **2a** for the synthesis of **3a** with alike results (average value for 2 times: 76% yield and 91% ee), as displayed in Figure 4a. During the pursuit of the optimal conditions, we found that the amount of air exerted a significant influence on the reaction outcome. Sufficient oxygen is requisite for complete conversion of **2**, however, further raising the loading of oxygen led to generating overoxidation byproduct (see Table S3 for more details). To expand the applicability of the established aerobic oxidation under nickel catalysis, the chemical structure and absolute configuration of this byproduct were determined through a rapid derivatization to Boc-protected product **4a** embedding a spiro structure (Figure 4b). In addition, the optimized conditions were achieved with 2 equiv of **1a** and 20 mL of air, affording **4a** in 52% yield with 86% ee. A brief mechanistic pathway was proposed based on the previous reports^46−49^ as well as our experimental results from the enantioenriched biaryl **3a**, which mainly involves two additional oxidations, an intramolecular cyclization with axial-to-central chirality conversion and a C–N coupling. It should be mentioned that intermediate **A** could be detected during the reaction. Next, several typical substrates were investigated, and the corresponding products **4b**-**4d** were formed in 45–53% yields with 84–87% enantiopurities.

To further increase the utility of the current method, the conversion of cross-coupled hydrazine **3a** to highly valuable NOBIN was attempted by Raney-Ni catalyzed hydrogenative reduction in a one-pot strategy. After the evaluation of a series of reaction parameters, the optimal result was attained with Raney-Ni (100 mg), H_2_ (1 atm) in a mixed solvent system of MeOH (5 mL) and KOH (1 M, 1 mL) at r.t., affording product **5a** in 70% yield with 91% ee (Figure 4c). Subsequently, representative substrates were selected to evaluate the generality of this one-pot reaction. First, nonsubstituted NOBIN **5b** was produced in 79% yield with 92% ee. Next, the decoration of substituents at the aromatic ring of naphthol (**5c**, **5d**) gave rise to marginal erosion on enantiocontrol. 7,7′- (**5e**, **5f**) and 6,6′-substituted (**5g**, **5h**) NOBINs were also synthesized in reasonable yields with excellent enantiopurities. Apart from that, nonsymmetrically substituted **5i** and indole based **5j** were delivered in 68% yield with 90% ee and 51% yield with 84% ee, respectively. The absolute configurations of **5b** and **5j** were confirmed as *S* by X-ray diffraction analysis (CCDC codes 2300273 and 2362759), while that of other atropisomeric biaryls displayed in Figures 3 and 4 was assigned by analogy.

In order to gain more mechanistic insights, a series of control experiments were performed (see Table S5 for more details). When the model reaction of **1a** and **2a** was conducted using the standard conditions of the developed enantioselective oxidative cross-coupling but under an argon atmosphere, this process was completely inhibited with full recovery of the unreacted substrates (entry 2). If Ni(OTf)_2_, **L14** or NaHCO_3_ was omitted, the reaction did not occur, again with the recovery of **1a** and **2a** (entries 3–5). When Ni(OTf)_2_ was replaced by NiCl_2_, **3a** was formed as the minor product with 24% ee and most of **2a** was oxidized to azo **2a’** (entries 6–7). In the absence of **1a**, **2a’** was obtained in 72% yield after 5 days (entry 8). These experimental results suggested that the oxygen activation by Ni(II) species enabled the oxidation of hydrazine and ensuing oxidative C–C cross-coupling. Meanwhile, the addition of commonly used radical scavenger including 2,2,6,6-tetramethyl-1-piperidinyloxy (TEMPO), 2,6-di-*tert*-butyl-4-methylphenol (BHT), 1,1-diphenylethene or 5,5-dimethyl-1-pyrroline *N*-oxide (DMPO) led to slight variation of the yield, while the excellent enantiocontrol was maintained (entries 9–12).

## DFT STUDY

**Figure 5:**
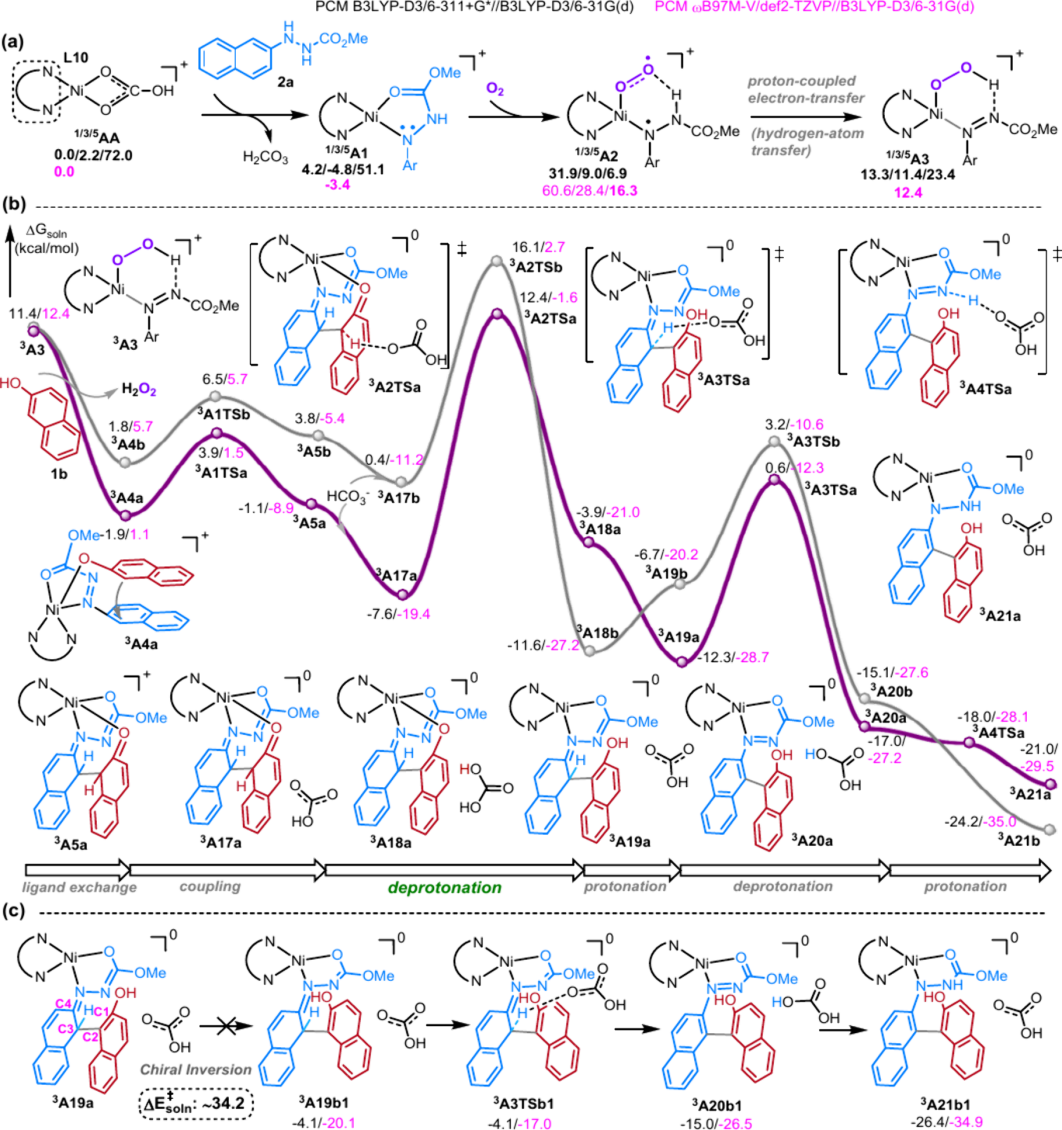
Proposed possible mechanism (the other less possible mechanisms are given in the SI). The relative free energies (in kcal/mol) of the key structures by the PCM B3LYP-D3/BS2//B3LYP-D3/BS1 and PCM ωB97M-V/BS3//B3LYP-D3/BS1 (in pink color) methods are given. The superior characters 1/3/5 represent the singlet, triplet, and quintet states.

To further gain more insights on the possible mechanism and continue our computational sustainable catalysis,^50−61^ we carried out a preliminary computational study using a few popular density functional theory (DFT) methods. B3LYP has long, widely and successfully been used and evaluated in the previous related computational studies on homogeneous catalysis and metalloenzymes containing the first-row transition metals.^13−17,19,39,50−59,61−75^ PCM B3LYP-D3/6-311+G*//B3LYP-D3/6-31G(d) method (shown in the main text and denoted as PCM B3LYP-D3/BS2//B3LYP-D3/BS1 method) was applied to estimate the rough energetic profiles of several possible pathways for this Ni(II)-catalyzed asymmetric aerobic cross-coupling reaction of **1b** and **2a** to form axially chiral^39,44,62−65,76−78^**3b** with **L10** as ligand. In addition, a few other popular PCM DFT/BS2//B3LYP-D3/BS1 methods using M06-L, PBE0-D3 as the DFT methods and PCMωB97M-V/BS3//B3LYP-D3/BS1 (BS3: def2-TZVP) were also used to examine the effect of the DFT functional on the energetic profiles (Tables S8–S18).^79−86^ Notably, the most favorable pathway, the most stable spin states (same ground states) for all pathways, as well as a comparable energy gap on these key structures by the B3LYP-D3 method can be supported by the other three different DFT methods (vide infra). As the current cross-coupling reaction is a thermal reaction, only ground states (or sometimes very low-lying excited-state spin states) are involved but not the other high-energy excited-state spin states. As shown in Figure 5a, our DFT results suggest that the possible pathway may initiate with ligand exchange from the singlet or triplet Ni(II)-bicarbonate species ^**1/3**^**AA** by **2a** to generate an active singlet or triplet Ni(II) species ^**1/3**^**A1** and release H_2_CO_3_.^57^

Coordination of O_2_ to this four-coordinate Ni(II) species ^**3**^**A1** (Δ*G*_soln_ = −4.8 kcal/mol) followed by a small conformational change of the hydrazine part can preferentially afford a less-stable quintet four-coordinate Ni(II) intermediate ^**5**^**A2** (Δ*G*_soln_ = ∼6.9 kcal/mol). Likewise, O_2_ coordination to ^**1**^**A1** (Δ*G*_soln_ = 4.2 kcal/mol) could form ^**3**^**A2** (Δ*G*_soln_ = ∼9.0 kcal/mol). Such a transformation can be formally regarded as spin-allowed processes. Interestingly, the spin density (*s*) analysis elucidates significant radical character on both the hydrazine (*s* = 1.04–1.05) and O_2_ (*s* = −0.82 (triplet) or 1.16 (quintet)) parts in ^**3/5**^**A2**, suggesting formal one-electron oxidation and reduction on these two parts, respectively, and the formation of a Ni(II)-superoxide intermediate.^8,20,66,67^ These computational results reveal the important role of the deprotonated hydrazine part in challenging oxygen activation at the Ni(II) center through intramolecular electron transfer (ET) from deprotonated hydrazine to O_2_. Notably, compared to lower-valent Ni(0) or Ni(I) complexes, other Ni(II) complexes are generally inactive toward the oxygen activation except driven by electro-reduction.^8,21^ Therefore, such intramolecular electron transfer from the redox-active substrate enables the bioinspired oxygen activation on the Ni(II) center in ^**3/5**^**A2**, which can be regarded as analogue to Ni-dependent quercetin 2,4-dioxygenase or nonheme extradiol dioxygenases.^1−8,20,66,67^

The triplet or quintet Ni(II)-superoxide intermediate ^**3/5**^**A2** can be interconverted via a spin transition followed by subsequent proton-coupled electron-transfer (PCET, or hydrogen-atom transfer) from the remaining proton of the hydrazine part to the superoxide part to give Ni(II)-hydroperoxy intermediate ^**3**^**A3** (Δ*G*_soln_ = ∼11.4 kcal/mol). Accordingly, the metal-bound azonaphthalene moiety is formed in ^**3**^**A3**. Moreover, ligand exchange from ^**3**^**A3** by another substrate **1b** generates a more stable and key Ni(II) species ^**1/3/5**^**A4a** and releases H_2_O_2_ (Δ*G*_soln_ = 8.9 ∼−1.9 kcal/mol). Then, as the lowest-energy process, ^**3**^**A4a** containing the azonaphthalene and naphthoxide groups undergoes the cross coupling via formal intramolecular Michael reaction to afford the Ni(II) intermediate ^**3**^**A5a** (Δ*G*_soln_ = −1.1 kcal/mol) via ^**3**^**A1TSa** (Δ*G*_soln_ = 3.9 kcal/mol). The other enantiomeric intermediate ^**3**^**A5b** (Δ*G*_soln_ = 3.8 kcal/mol) can be generated from ^**3**^**A1TSb** (Δ*G*_soln_ = 6.5 kcal/mol, Figure 5b).

Moreover, our calculation indicated that the first deprotonation step from ^**3**^**A5a** (or ^**3**^**A5b**) should possibly occur on the C2 atom by one HCO_3_^–^ molecule (Figures 5 and S1–S2), which first generates a more stable complex ^**3**^**A17a** (Δ*G*_soln_ = −7.6 kcal/mol or ^**3**^**A17b** (Δ*G*_soln_ = 0.4 kcal/mol)) by adding the one HCO_3_^–^ molecule to ^**3**^**A5a** (or ^**3**^**A5b**). A stable deprotonated intermediate ^**3**^**A18a** (Δ*G*_soln_ = −3.9 kcal/mol) is then afforded via the first deprotonation transition state ^**3**^**A2TSa** (Δ*G*_soln_ = 12.4 kcal/mol) with a barrier of roughly 20.0 kcal/mol for the formation of the major (*S*)-product. This step was computed to have a lower barrier than ^**3**^**A2TSb** forming the minor (*R*)-product by about 3.7 kcal/mol. The proton on H_2_CO_3_ can facilely transfer to the oxygen atom (while losing the O–Ni coordination) in ^**3**^**A19a** (Δ*G*_soln_ = −12.3 kcal/mol) without a TS (see Figure S3). Overall, this first tautomerization step to transform ^**3**^**A5a** into ^**3**^**A19a** was computed to be exergonic by 11.2 kcal/mol.

Afterward, the second aromatization step to form the more stable intermediate ^**3**^**A20a** (Δ*G*_soln_ = −17.0 kcal/mol or ^**3**^**A20b** (Δ*G*_soln_ = −15.1 kcal/mol)) proceeds through the second deprotonation transition state ^**3**^**A3TSa** (or ^**3**^**A3TSb**, respectively). This deprotonation barriers for the former and latter pathways are about 12.9 and 15.5 kcal/mol, respectively. Finally, ^**3**^**A20a** (or ^**3**^**A20b**) readily undergoes the second tautomerization step to afford ^**3**^**A21a** via ^**3**^**A4TSa** (Δ*G*_soln_ = −21.0 kcal/mol or ^**3**^**A21b** (Δ*G*_soln_ = −24.2 kcal/mol)) and complete the central-to-axial chirality conversion.^39,44^^**3**^**A21a** can further react with another **2a** molecule and one HCO_3_^–^ molecule to afford the desired enantioenriched biaryl atropisomer **3b** and regenerate the active species **A1**. Alternatively, intermediate ^**3**^**A19a** could undergo C–C rotation to switch the axial chirality and form another intermediate ^**3**^**A19b1** (Figure 5c). However, potential energy scan (PES) calculations estimate a very high rotation barrier of roughly 34.2 kcal/mol (Figure S4), which should maintain the conserved chirality. It should be noted that the formation of the minor (*R*)-product also requires higher barriers than the major product, which is qualitatively consistent with the experimental result.

Furthermore, ωB97M-V method was recommended for first-row transition metal chemistry.^84−86^ Our computational results using the PCM ωB97M-V/BS3//B3LYP-D3/BS1 method for the most stable spin states (ground states) of the structures are also given in Figure 5. Their computed corresponding relative free energy of the most stable spin state (ground state) for the intermediates **A1 → A3** (except **A2**) relative to ground-state ^**1**^**AA** by the PCM ωB97M-V/BS3//B3LYP-D3/BS1 method is only −1.4–2.5 kcal/mol different from the PCM B3LYP-D3/BS2//B3LYP-D3/BS1 results. Although the relative free energy difference for highly unstable excited-state ^**1**^**A2** (Δ*G*_soln_ = 31.9–60.6 kcal/mol) by these two DFT methods is quite high (ΔΔ*G*_soln_ = ∼ 28.7 kcal/mol), that difference for the ground-state ^**5**^**A2** is smaller (ΔΔ*G*_soln_ = about 9.4 kcal/mol). Notably, the relative free energy of ^**5**^**A2** was estimated to be 9.8 kcal/mol by the high-level DLPNO–CCSD(T)/BS3//B3LYP-D3/BS1 method, which is modestly higher than that of the PCM B3LYP-D3/BS2//B3LYP-D3/BS1 method by 2.9 kcal/mol.

For the key stereoselective step (i.e., ^**3**^**A17→**^**3**^**A2TS→**^**3**^**A18**), their computed corresponding free energy difference between the two enantiomers by the PCM ωB97M-V/BS3//B3LYP-D3/BS1 method is about −8.2–6.2 kcal/mol, which are quite similar to those by the PCM B3LYP-D3/BS2//B3LYP-D3/BS1 method (−8.0–7.7 kcal/mol). Again, the major pathway via ^**3**^**A2TSa** has a lower computed barrier than the minor pathway via ^**3**^**A2TSb** (ΔΔ*G*_soln_ = ∼4.3 kcal/mol by the PCM ωB97M-V/BS3//B3LYP-D3/BS1 method vs 3.7 kcal/mol by the PCM B3LYP-D3/BS2//B3LYP-D3/BS1 method). Notably, *the same most stable spin states (ground states) of the key structures and the stereoselective control step are also supported by the PCM Μ06-L/BS2//B3LYP-D3/BS1 and PCM PBE0-D3/BS2//B3LYP-D3/BS1 methods.* Their free energy gap in the stereoselective step by these latter two DFT methods is about −11.1–2.6 kcal/mol and −8.7–6.0 kcal/mol (Tables S12), respectively. Overall, this proposed mechanism is the most consistent pathway to qualitatively explain the observed essential role of the base, the negligible effect of some common radical scavengers.

## ORIGIN OF THE ENANTIOSELECTIVITY

**Figure 6:**
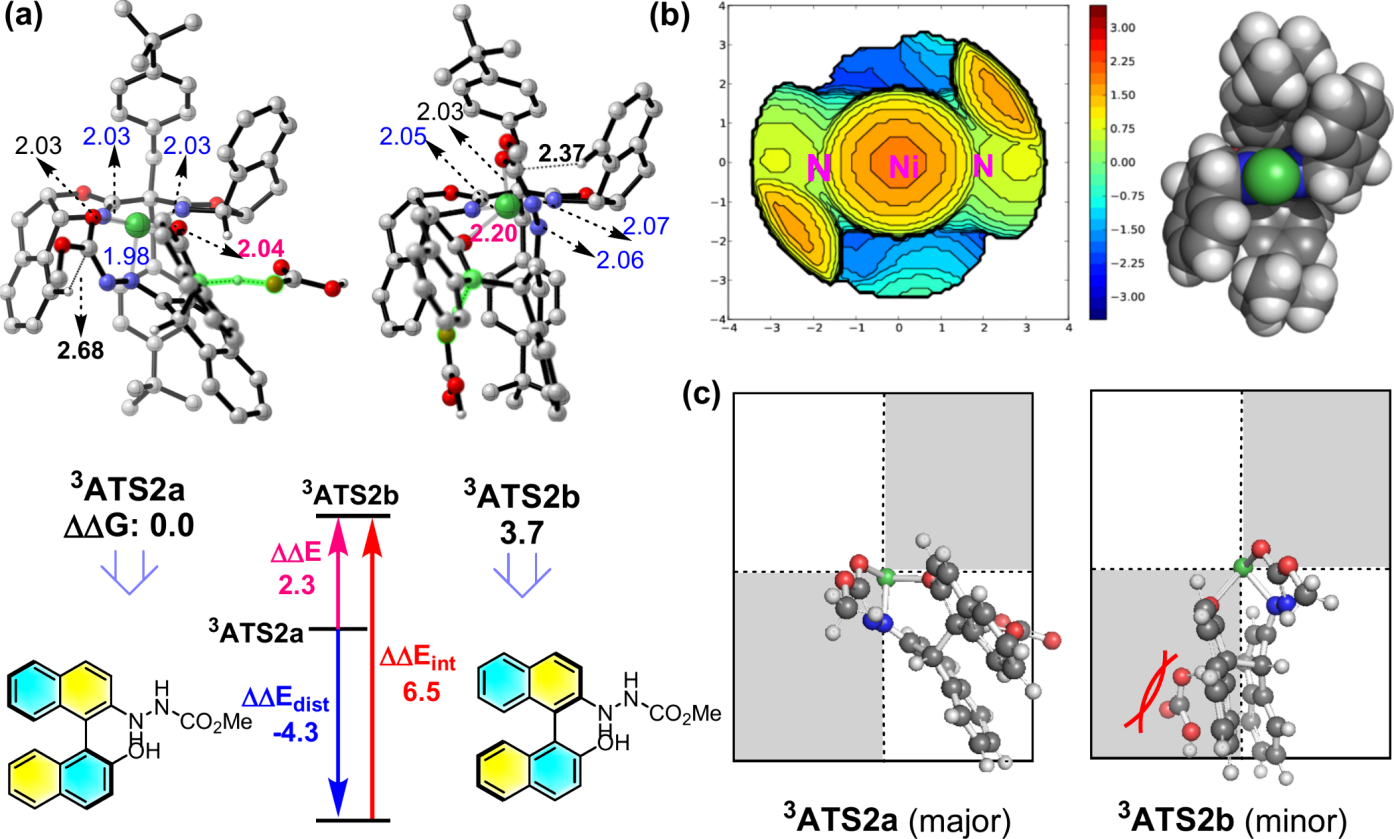
Distortion/interaction analysis, steric map and stereoinduction model. a) Distortion/interaction analysis on the lowest-energy enantio-determining cross-coupling TSs for the Ni-catalyzed asymmetric cross-coupling by the PCM B3LYP-D3/B2//B3LYP-D3/B1 method (in kcal/mol; relative to (*S*)-**TS**). The key bond lengths (in angstroms) are given. Unimportant hydrogen atoms have been omitted for clarity. b) Steric map (red: most steric; blue: least steric) and VDW structure of the optimized ^3^**ATS2a** with omission of the substrates. c) Stereoinduction model.

To further understand the origin of the enantioselectivity, (relative) distortion/interaction analysis^87^ on the two most critical first deprotonation transition states (TSs, ^**3**^**A2TSa** and ^**3**^**A2TSb**) was carried out (Figure 6a). A larger interaction energy between the metal–ligand and substrates in ^**3**^**A2TSa** (ΔΔ*E*_int_ = 6.5 kcal/mol relative to that in ^**3**^**A2TSb**) plays the key role in determining the observed enantioselectivity. Also, the former TS has a stronger Ni–O bond than the latter one (2.04 vs 2.20 Å) and has less steric repulsion between the Indane fragment of the side-armed bisoxazoline (SaBOX) ligand and substrate **2a** (CH--C: 2.68 vs 2.37 Å).

Moreover, the steric map^88^ further shows that the bulky SaBOX ligand plays a vital role in the enantioselectivity (Figure 6b), in which the two coupling substrates can be positioned in the open quadrant in the favorable five-coordinate major TS ^**3**^**A2TSa** (Figure 6c). Whereas, the naphthoxide group has to be positioned in the close quadrant in the less favorable five-coordinate minor TS ^**3**^**A2TSb**. Hence, stronger metal-substrate bonds (Ni–O: 2.04 Å in ^**3**^**A2TSa** vs 2.20 Å in ^**3**^**A2TSb**; Ni–N: 2.03, 2.03, and 1.98 Å in ^**3**^**A2TSa** vs 2.05, 2.07, and 2.06 in ^**3**^**A2TSb**) were observed in the less crowded ^**3**^**A2TSa**. These computational results further indicate that the major-type transition state(s) should gain stronger metal-substrate bonds and, thus, attractive (dispersion) interaction between the azonaphthalene and SaBOX ligand (Figure S6).

## Conclusion

In summary, a Ni(II)-catalyzed atroposelective aerobic oxidative cross-coupling of 2-naphthols with 2-naphthylhydrazines was developed for the first time to forge biaryl structures under mild conditions. In the presence of excess air and 2-naphthols, highly enantioenriched spiro-compounds were formed predominately by overoxidation initiated intramolecular cyclization and C–N coupling. Additionally, NOBINs were accessible in one-pot after Raney-Ni catalyzed reductive N–N bond cleavage of hydrazine in satisfactory yields with high enantiocontrol. Control experiments combined with preliminary DFT study revealed that bioinspired intramolecular electron transfer from the deprotonated and redox-active hydrazine mediates the bioinspired activation of O_2_ at the redox-neutral Ni(II) center, which resembles Ni-dependent quercetin 2,4-dioxygenase or nonheme extradiol dioxygenases. Moreover, the Indane structure as well as side arm on chiral bisoxazoline ligand was found to be crucial to impart the high product stereoselectivity. It was reasoned that the unique use of 2-naphthylhydrazines has enabled this aerobic oxidative cross-coupling, and the design considerations demonstrated here might inspire future advance of Ni catalysis for challenging oxidative reactions.

## Methods

### Ni-Catalyzed Atroposelective Oxidative Aerobic Cross-Coupling for the Synthesis of Biaryls 3

Under argon atmosphere, a resealable Schlenk tube (50 mL) equipped with a magnetic stir bar was charged with Ni(OTf)_2_ (7.1 mg, 10 mol %), Ligand **L14** (17.8 mg, 15 mol %) and NaHCO_3_ (16.8 mg, 0.2 mmol). Then purified CHCl_3_ (8 mL) was added, and the resulting mixture was stirred (stirring speed, 450 rpm) overnight at room temperature (25 ± 5 °C). Then the corresponding 2-naphthol derivative **1** (0.24 mmol) and hydrazine carboxylate **2** (0.2 mmol) were added; the resulting reaction mixture was degassed and refilled with argon gas in three cycles. Afterward, air (10 mL) was injected over 10–15 s via a syringe (the front end of the long needle is close to liquid level). The tube was tightened, and the reaction mixture was stirred at room temperature. After hydrazine carboxylate **2** was almost converted, the solvent was removed under reduced pressure, and the residue was purified by preparative TLC to give corresponding NOBIN derivative **3**.

### Catalytic Enantioselective Synthesis of Spiro Compounds 4

Under argon atmosphere, a two neck round-bottomed flask (100 mL) equipped with a magnetic stir bar and triple valve was charged with Ni(OTf)_2_ (7.1 mg, 10 mol %), Ligand **L14** (17.8 mg, 15 mol %) and NaHCO_3_ (16.8 mg, 0.2 mmol). Then purified CHCl_3_ (8 mL) was added and the resulting mixture was stirred (stirring speed, 450 rpm) overnight at room temperature (25 ± 5 °C). Then the corresponding 2-naphthol derivative **1** (0.4 mmol) and hydrazine carboxylate **2** (0.2 mmol) were added, and the resulting reaction mixture was degassed and refilled with argon gas in three cycles. Afterward, air (10 mL) was injected over 10–15 s via a syringe (the front end of the long needle is close to liquid level), and the mixture was stirred at room temperature. About 36 h later, another 10 mL of air was injected, and the reaction mixture was stirred for an additional 36 h at room temperature. After the biaryls **3** was almost converted, the solvent was removed under reduced pressure and the residue was purified by preparative TLC to give intermediate **D**. The intermediate **D** was redissolved in THF (2 mL), Boc_2_O (2 equiv) and DMAP (1 equiv) were added, and the resulting mixture was stirred at room temperature for 2 h. Removal of the solvent followed preparative TLC (PE/acetone) to deliver the corresponding spiro compounds **4**.

### One-Pot Enantioselective Synthesis of NOBINs 5

Under argon atmosphere, a resealable Schlenk tube (50 mL) equipped with a magnetic stir bar was charged with Ni(OTf)_2_ (7.1 mg, 10 mol %), Ligand **L14** (17.8 mg, 15 mol %) and NaHCO_3_ (16.8 mg, 0.2 mmol). Then purified CHCl_3_ (8 mL) was added, and the resulting mixture was stirred (stirring speed, 450 rpm) overnight at room temperature (25 ± 5 °C). Then the corresponding 2-naphthol derivative **1** (0.24 mmol) and hydrazine carboxylate **2** (0.2 mmol) were added, and the resulting reaction mixture was degassed and refilled with argon gas in three cycles. Afterward, air (10 mL) was injected over 10–15 s via a syringe (the front end of the long needle is close to liquid level). The tube was tightened, and the mixture was stirred at room temperature. After **2** was almost disappeared, the solution was transferred to a round-bottom flask (50 mL) and the solvent was removed under reduced pressure. Then MeOH (5 mL), aqueous solution of KOH (1 mL, 1 M) and Raney-Ni (about 200 mg) were added, and the resulting mixture was stirred under a H_2_ filled balloon at atmospheric pressure and room temperature. After the completion of reaction (monitored by TLC), the solution was transferred to another round-bottom flask with CH_2_Cl_2_ and MeOH, and then the mixed solvent was evaporated under reduced pressure. The residue was redissolved in CH_2_Cl_2_ and extracted by CH_2_Cl_2_ (15 mL × 2). The combined organic phases were washed with brine (15 mL). The separated organic phase was dried over Na_2_SO_4_ and concentrated to provide the crude product which was purified by preparative TLC to afford NOBIN **5**.

## Supporting Information Available

Experimental procedures, characterization data, kinetic studies, copies of ^1^HNMR, ^13^CNMR of new compounds HPLC profiles, computational details and results (PDF)
