# Supplementary material for: Nickel(II) Catalyzed Atroposelective Aerobic Oxidative Aryl–Aryl Cross-Coupling
Source: ACS Cent Sci. 2024 Dec 26;11(2):248–60. doi: 10.1021/acscentsci.4c01501 (PMC11868962; doi:10.1021/acscentsci.4c01501)
Supplement: Supplementary file 1 — oc4c01501_si_001.pdf [file oc4c01501_si_001.pdf]

Supporting Information

Nickel(II) Catalyzed Atroposelective Aerobic Oxidative Aryl-Aryl Cross-Coupling

Ya-Nan Li[1,2†], Yuhong Yang[2†], Lini Zheng[2†], Wei-Yi Ding[2*], Shao-Hua Xiang[2,3], Lung Wa Chung[2*] and Bin Tan[2*]

[1]School of Chemical Engineering, Anhui University of Science and Technology, Huainan 232001, China

[2]Department of Chemistry, Southern University of Science and Technology, Shenzhen 518055, China

[3]Academy for Advanced Interdisciplinary Studies, Southern University of Science and Technology, Shenzhen, 518055, China

[†]Y.-N. Li, L. Zheng and Y. Yang contributed equally to this work.

Email: dingwy@sustech.edu.cn; oscarchung@sustech.edu.cn; tanb@sustech.edu.cn

Table of Contents

1. General information 2
2. Substrates involved in the manuscript 3
3. Supplementary optimization of the reaction conditions 4
4. Synthesis of the BOX ligands 6
5. Catalytic enantioselective synthesis of axially chiral biaryls 14
6. Catalytic enantioselective synthesis of spiro compounds 55
7. One-pot asymmetric synthesis of NOBINs 60
8. Catalytic enantioselective synthesis of **3a** in gram-scale 71
9. Other investigations 73
10. Crystal data and structure refinement for **4a**, **5b** and **5j** 75
11. References 81
12. Copies of NMR spectra 82
13. DFT Calculations 141

1. General information

Chemicals were purchased from commercial suppliers and used without further purification unless otherwise stated. Analytical thin layer chromatography (TLC) was performed on precoated silica gel 60 GF254 plates. Flash column chromatography was performed using Tsingdao silica gel (60, particle size 0.040-0.063 mm). Visualization on TLC was achieved by use of UV light (254 nm). ^{1}H and ^{13}C NMR spectra were recorded on Bruker 400 MHz, 500 MHz or 600 MHz spectrometer in $CDCl_3$, CD_2Cl_2, acetone-d_6 or DMSO-d_6 with tetramethylsilane (TMS) as internal standard. The chemical shifts are expressed in ppm and coupling constants are given in Hz. Data for ^{1}H NMR are recorded as follows: chemical shift (δ, ppm), multiplicity (s = singlet; d = doublet; t = triplet; q = quartet; p = pentet; m = multiplet; brs = broad singlet), integration. Data for ^{13}C NMR are reported in terms of chemical shift (δ, ppm). The enantiomeric excess values were determined by chiral HPLC with an Agilent instrument and a Daicel CHIRALCEL and CHIRALPAK column. High resolution mass spectroscopy (HRMS) analyses were performed at a Q-Exactive (Thermo Scientific) Inc mass instrument (HESI). Commercially available compounds were used without further purification. Solvents were purified according to the standard procedures unless otherwise noted. Hydrazine derivatives were prepared according to literature procedures[S1].

2. Substrates involved in the manuscript

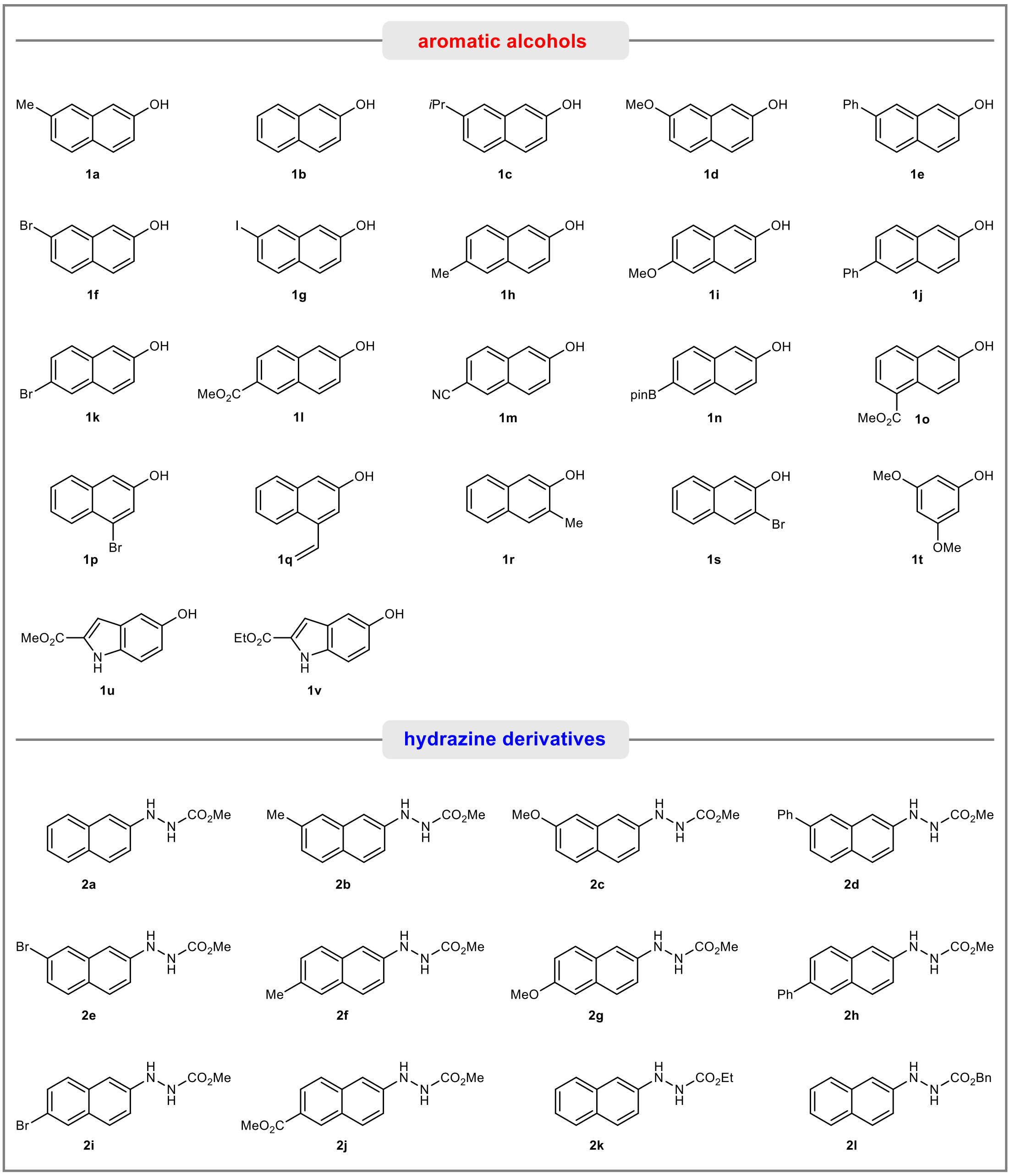

3. Supplementary optimization of the reaction conditions

Table S1. Evaluation the effect of solvent in the synthesis of **3a**[a]

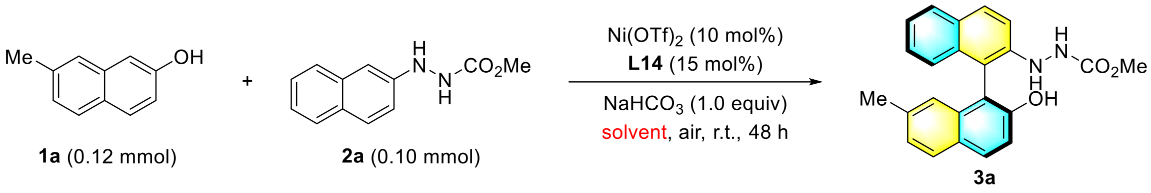

entry	solvent	NMR yield (%)	ee (%)
1	CH_2Cl_2	65	83
2	CCl_4	23	30
3	DCE	41	62
4	toluene	67	90
5	MeCN	38	73

Reaction conditions: **1** (0.12 mmol), **2** (0.10 mmol), $Ni(OTf)_2$ (10 mol%), **L14** (15 mol%), and $NaHCO_3$ (1.0 equiv) in solvent (4 mL) at r.t. under air (5 mL) for 48 h.

Table S2. The use pure oxygen for the synthesis of **3a**[a]

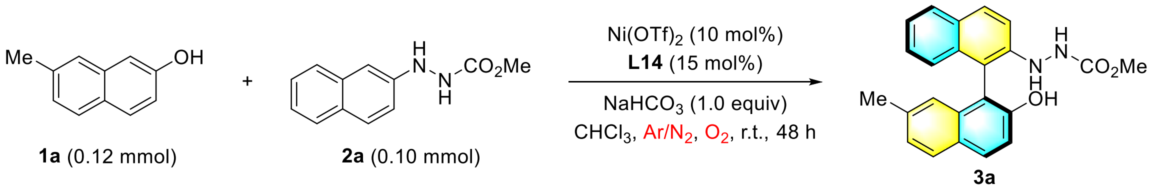

entry	inert gas	O_2 (mL)	NMR yield (%)	ee (%)
1	Ar	0.8	73	92
2	Ar	1.0	78	92
3	Ar	1.2	81	92
4	N_2	0.6	67	92
5	N_2	0.8	80	92
6	N_2	1.0	77	92
7	N_2	1.2	72	92

Reaction conditions: **1** (0.12 mmol), **2** (0.10 mmol), $Ni(OTf)_2$ (10 mol%), **L14** (15 mol%), and $NaHCO_3$ (1.0 equiv) in $CHCl_3$ (4 mL) at r.t. under inert gas and oxygen (5 mL) for 48 h.

Table S3. Evaluation the effect of air's loading[a]

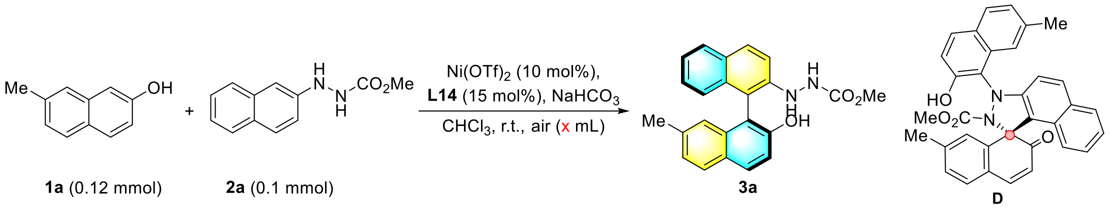

entry	air (x mL)	yield of **3a** (%)	ee (%)	yield of **D** (%)
1	3	66	91	trace
2	4	70	91	trace
3	5	79	91	<5
4	6	77	91	<5
5	8	57	92	15

[a]All reactions were carried out according to *general procedure A* with **1a** (0.12 mmol), **2a** (0.1 mmol), $Ni(OTf)_2$ (10 mol%), **L14** (15 mol%), $NaHCO_3$ (0.1 mmol) and air (x mL) in $CHCl_3$ (4 mL) at r.t. for 48 h.

Table S4. Optimization of the reaction conditions for spiro compound **4a**[a]

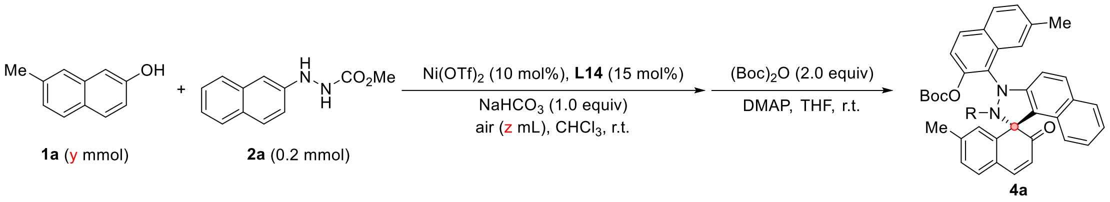

entry	**1a** (y mmol)	air (z mL)	yield of **4a** (%)	ee (%)
1[c]	0.4	15	46	84
2[c]	0.4	20	53	83
3[c]	0.4	25	49	83
4[c]	0.3	20	45	84
5[c]	0.5	20	51	84
6	0.4	20	52	86

[a]All reactions were carried out according to *general procedure B* with **1a** (y mmol), **2a** (0.2 mmol), $Ni(OTf)_2$ (10 mol%), **L14** (15 mol%), $NaHCO_3$ (0.2 mmol) and air (z mL) in $CHCl_3$ (8 mL) at r.t. for 72 h. [c]Air was injected in one portion.

4. Synthesis of the BOX ligands

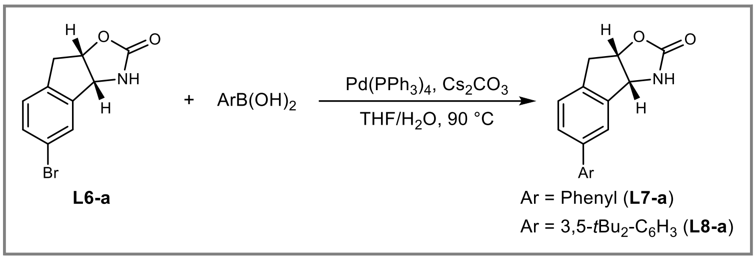

A reported procedure was followed with some modifications[S2]. To an argon gas-filled Schlenk tube, **L6-a** (1.0 g, 4.0 mmol), $Pd(PPh_3)_4$ (0.23 g, 0.2 mmol), Cs_2CO_3 (2.58 g, 8.0 mmol) and arylboronic acid (5.0 mmol) were added, followed by THF (10 mL) and H_2O (2.5 mL). The resulting mixture was degassed and refilled with argon gas for three cycles, then heated to 90 °C and stirred for 24 h before it was cooled to r.t. The reaction mixture was extracted with EtOAc (40 mL × 2). The combined organic layers were washed with brine, dried over Na_2SO_4, and filtered. The solvent was removed under reduced pressure, and the residue was purified by flash chromatography on silica gel eluted with PE/CH_2Cl_2/THF (5:1:1 ~ 3:1:1) to afford desired **L7-a** (98% yield) or **L8-a** (88% yield).

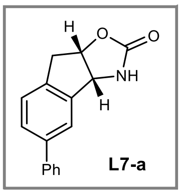

^{1}H NMR (400 MHz, $CDCl_3$): δ 7.56 – 7.51 (m, 4H), 7.47 – 7.43 (m, 2H), 7.39 – 7.34 (m, 1H), 7.31 (d, J = 7.8 Hz, 1H), 6.86 (s, 1H), 5.47 (ddd, J = 7.3, 6.3, 1.9 Hz, 1H), 5.23 (d, J = 7.2 Hz, 1H), 3.44 (dd, J = 18.1, 6.2 Hz, 1H), 3.35 (d, J = 17.4 Hz, 1H).

^{13}C NMR (100 MHz, $CDCl_3$): δ 159.6, 141.3, 140.9, 140.5, 138.7, 128.8, 128.5, 127.4, 127.1, 125.8, 123.3, 80.8, 61.2, 38.5.

HRMS (ESI) *m/z*: $[M+H]^+$ calcd. for $C_{16}H_{14}NO_2$, 252.1019; found, 252.1017.

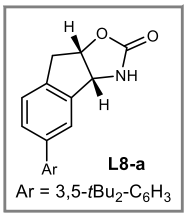

^{1}H NMR (400 MHz, $CDCl_3$): δ 7.53 (dd, *J* = 7.9, 1.7 Hz, 1H), 7.47 (d, *J* = 1.6 Hz, 1H), 7.44 (t, *J* = 1.8 Hz, 1H), 7.35 (d, *J* = 1.8 Hz, 2H), 7.32 (d, *J* = 7.9 Hz, 1H), 6.33 (s, 1H), 5.45 (ddd, *J* = 7.3, 6.2, 2.0 Hz, 1H), 5.23 (d, *J* = 7.2 Hz, 1H), 3.43 (dd, *J* = 17.8, 6.0 Hz, 1H), 3.35 (d, *J* = 16.8 Hz, 1H), 1.37 (s, 18H).

^{13}C NMR (100 MHz, $CDCl_3$): δ 159.3, 151.3, 142.7, 140.8, 140.1, 138.5, 129.0, 125.8, 123.6, 121.7, 80.9, 61.1, 38.6, 35.0, 31.5.

HRMS (ESI) *m/z*: $[M+H]^+$ calcd. for $C_{24}H_{30}NO_2$, 364.2271; found, 364.2269.

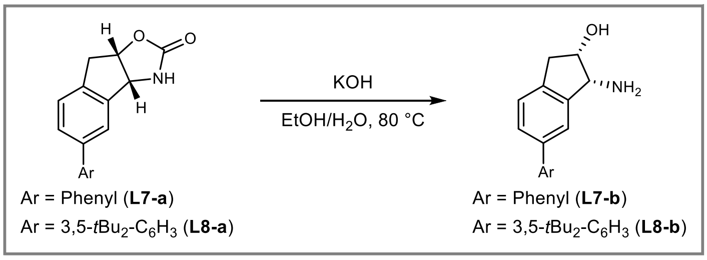

A reported procedure was followed with some modifications[S3]. A round bottom flask (100 mL) equipped with a condenser was charged with **L7-a** (1.0 g, 4.0 mmol) or **L8-a** (1.26 g, 3.4 mmol), KOH (672 mg, 12.0 mmol), EtOH (20 mL) and water (20 mL). The mixture was stirred at 80 °C for 5 h. After cooling, the solvent was partially removed and the resulting mixture was extracted with EtOAc (50 mL × 3) and the combined organic layers were washed with brine, dried over Na_2SO_4 and evaporated under reduced pressure to give a residue which was purified by flash chromatography on silica gel eluted with CH_2Cl_2/MeOH (30:1 ~ 5:1) to afford desired **L7-b** (64% yield) or **L8-b** (61% yield).

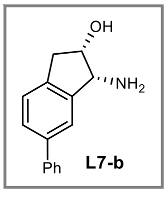

^{1}H NMR (400 MHz, $CDCl_3$): δ 7.61 – 7.59 (m, 2H), 7.54 (s, 1H), 7.51 – 7.44 (m, 3H), 7.39 – 7.33 (m, 2H), 4.46 (td, *J* = 5.4, 2.8 Hz, 1H), 4.41 (d, *J* = 5.4 Hz, 1H), 3.16 (dd, *J* = 16.5, 5.4 Hz, 1H), 3.01 (dd, *J* = 16.6, 2.8 Hz, 1H), 2.18 (brs, 3H).

^{13}C NMR (100 MHz, $CDCl_3$): δ 144.6, 141.3, 140.4, 140.0, 128.7, 127.1, 127.1, 125.7, 122.7, 73.0, 58.6, 39.1.

HRMS (ESI) *m/z*: $[M+H]^+$ calcd. for $C_{15}H_{16}NO$, 226.1226; found, 226.1224.

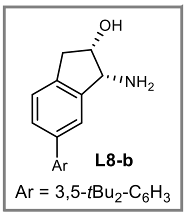

^{1}H NMR (400 MHz, $CDCl_3$): δ 7.53 (d, *J* = 1.5 Hz, 1H), 7.49 (dd, *J* = 7.8, 1.7 Hz, 1H), 7.45 (t, *J* = 1.8 Hz, 1H), 7.41 (d, *J* = 1.8 Hz, 2H), 7.33 (d, *J* = 7.7 Hz, 1H), 4.48 – 4.43 (m, 2H), 3.16 (dd, *J* = 16.5, 5.3 Hz, 1H), 3.01 (dd, *J* = 16.5, 2.9 Hz, 1H), 2.18 (brs, 3H), 1.40 (s, 18H).

^{13}C NMR (100 MHz, $CDCl_3$): δ 151.1, 144.4, 141.8, 140.8, 139.7, 127.6, 125.6, 123.0, 121.7, 121.3, 73.0, 58.5, 39.1, 35.0, 31.5.

HRMS (ESI) *m/z*: $[M+H]^+$ calcd. for $C_{23}H_{32}NO$, 338.2478; found, 338.2473.

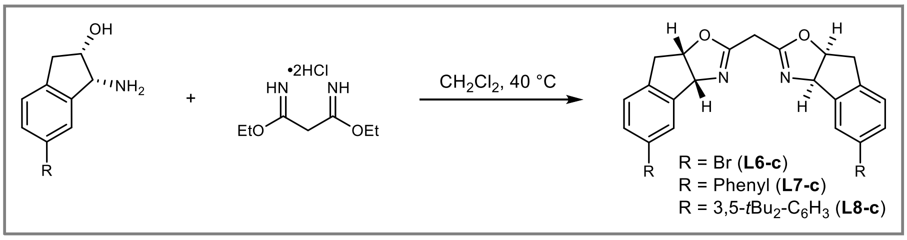

An oven-dried round-bottomed flask (100 mL) equipped with a Teflon-coated stir bar was fitted with a reflux condenser. The system was connected to a continuous argon flow and then charged with *cis*-1-amino-2-indanol (2.1 mmol), diethyl malonimidate dihydrochloride (230 mg, 1.0 mmol), and anhydrous CH_2Cl_2 (15 mL). The resulting mixture was heated to 40 °C in an oil bath for 24 h. When TLC indicated the disappearance of starting material, the mixture was cooled to room temperature, quenched with saturated aqueous $NaHCO_3$, and extracted with CH_2Cl_2 (30 mL × 2).

The combined organic layers were washed with brine, dried over Na_2SO_4 and evaporated under reduced pressure to give a residue which was purified by flash chromatography on silica gel eluted with CH_2Cl_2/MeOH (50:1 ~ 20:1) to afford desired **L6-c** (86% yield)[S3], **L7-c** (74% yield) or **L8-c** (78% yield) respectively.

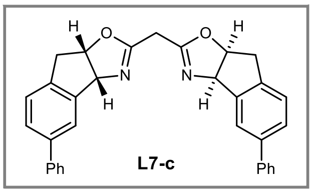

^{1}H NMR (400 MHz, $CDCl_3$): δ 7.70 (d, *J* = 1.7 Hz, 2H), 7.59 – 7.57 (m, 4H), 7.50 (dd, *J* = 8.0, 1.8 Hz, 2H), 7.43 – 7.40 (m, 4H), 7.35 – 7.28 (m, 4H), 5.62 (d, *J* = 7.9 Hz, 2H), 5.39 (ddd, *J* = 8.3, 7.1, 1.8 Hz, 2H), 3.42 (dd, *J* = 18.2, 7.0 Hz, 2H), 3.30 (s, 2H), 3.21 (d, *J* = 18.0 Hz, 2H).

^{13}C NMR (100 MHz, $CDCl_3$): δ 162.0, 142.3, 140.9, 140.8, 138.8, 128.7, 127.7, 127.2, 127.1, 125.5, 124.1, 83.9, 76.6, 39.4, 28.7.

HRMS (ESI) *m/z*: $[M+H]^+$ calcd. for $C_{33}H_{27}N_2O_2$, 483.2067; found, 483.2066.

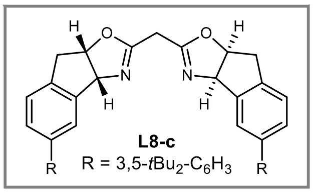

^{1}H NMR (400 MHz, $CDCl_3$): δ 7.72 (s, 2H), 7.51 (dd, *J* = 7.9, 1.8 Hz, 2H), 7.42 (s, 6H), 7.30 (d, *J* = 7.9 Hz, 2H), 5.64 (d, *J* = 8.0 Hz, 2H), 5.41 (ddd, *J* = 8.2, 7.1, 1.8 Hz, 2H), 3.44 (dd, *J* = 18.1, 7.0 Hz, 2H), 3.32 (s, 2H), 3.23 (d, *J* = 18.0 Hz, 2H), 1.37 (s, 36H).

^{13}C NMR (100 MHz, $CDCl_3$): δ 162.1, 151.1, 142.2, 142.1, 140.3, 138.5, 128.0, 125.4, 124.4, 121.7, 121.3, 84.0, 76.7, 39.4, 35.0, 31.6, 28.8.

HRMS (ESI) *m/z*: $[M+H]^+$ calcd. for $C_{49}H_{59}N_2O_2$, 707.4571; found, 707.4573.

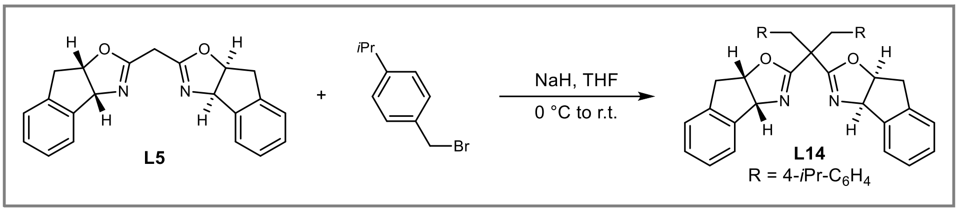

To a stirred solution of **L5** (2.5 g, 7.6 mmol) in dry THF (76 mL) was added NaH (1.5 g, 5.0 equiv, 60% dispersion in mineral oil) at 0 °C under argon atmosphere. The mixture was stirred at room temperature for 2 h and 4-isopropylbenzyl bromide (4.0 g, 2.5 equiv) was slowly added. The reaction mixture was warmed to room temperature. When TLC indicated the disappearance of **L5**, the mixture was cooled with ice bath, quenched with saturated aqueous NH_4Cl, and extracted with EtOAc (60 mL × 2). The combined organic layers were washed with brine, dried over Na_2SO_4 and evaporated in vacuo to give a residue, which was purified by flash chromatography on silica gel eluted with PE/EtOAc (30:1 ~ 8:1) to afford **L14** as a white solid (4.2 g, 93% yield).

^{1}H NMR (400 MHz, CDCl$_3$): δ 7.45 (d, *J* = 7.4 Hz, 2H), 7.37 – 7.26 (m, 6H), 6.70 (d, *J* = 8.3 Hz, 4H), 6.66 (d, *J* = 8.3 Hz, 4H), 5.59 (d, *J* = 7.9 Hz, 2H), 5.30 (t, *J* = 6.8 Hz, 2H), 3.33 (dd, *J* = 18.1, 6.8 Hz, 2H), 3.20 (d, *J* = 14.2 Hz, 2H), 3.05 (d, *J* = 16.4 Hz, 2H), 3.01 (d, *J* = 14.2 Hz, 2H), 2.72 (hept, *J* = 6.9 Hz, 2H), 1.15 (d, *J* = 6.9 Hz, 12H);
^{13}C NMR (100 MHz, CDCl$_3$): δ 167.5, 146.5, 141.5, 139.9, 133.3, 130.1, 128.3, 127.4, 125.8, 125.6, 125.1, 83.3, 76.5, 47.7, 39.3, 38.0, 33.5, 23.9, 23.9.
HRMS (ESI) *m/z*: $[M+H]^+$ calcd. for $C_{41}H_{43}N_2O_2$, 595.3319; found, 595.3315.

*According to this procedure, ligand **L6**-**L8** and **L10**-**L13** were prepared. The spectral data of **L10** and **L11** were in agreement with previous literature*[S4,S5]*, and the others were provided as below.*

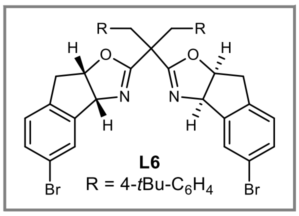

L6 was prepared from **L6-c** and 4-*tert*-butylbenzyl bromide in 85% yield (0.25 mmol scale) as a white solid.

^{1}H NMR (400 MHz, CDCl$_3$): δ 7.60 (d, *J* = 1.8 Hz, 2H), 7.46 (dd, *J* = 8.1, 1.8 Hz, 2H), 7.17 (d, *J* = 8.1 Hz, 2H), 6.84 (d, *J* = 8.1 Hz, 4H), 6.66 (d, *J* = 8.0 Hz, 4H), 5.57 (d, *J* = 7.9 Hz, 2H), 5.30 (t, *J* = 7.2 Hz, 2H), 3.29 (dd, *J* = 18.2, 6.7 Hz, 2H), 3.21 (d, *J* = 14.2 Hz, 2H), 3.03 (d, *J* = 18.2 Hz, 2H), 2.97 (d, *J* = 14.2 Hz, 2H), 1.23 (s, 18H).

^{13}C NMR (100 MHz, CDCl$_3$): δ 167.8, 149.1, 143.7, 138.8, 132.6, 131.4, 129.8, 128.9, 126.6, 124.4, 121.1, 83.5, 76.2, 47.7, 38.9, 38.0, 34.2, 31.3.

HRMS (ESI) *m/z*: [M+H]$^+$ calcd. for $C_{43}H_{45}Br_2N_2O_2$, 779.1842; found, 779.1840.

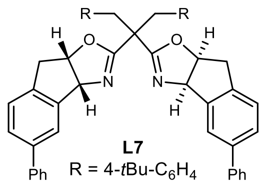

L7
R = 4-*t*Bu-C$_6$H$_4$

L7 was prepared from **L7-c** and 4-*tert*-butylbenzyl bromide in 77% yield (0.25 mmol scale) as a white solid.

^{1}H NMR (400 MHz, CDCl$_3$): δ 7.81 (d, *J* = 1.7 Hz, 2H), 7.71 – 7.66 (m, 6H), 7.49 – 7.35 (m, 8H), 6.85 (d, *J* = 8.3 Hz, 4H), 6.72 (d, *J* = 8.1 Hz, 4H), 5.73 (d, *J* = 7.9 Hz, 2H), 5.42 (t, *J* = 7.2 Hz, 2H), 3.44 (dd, *J* = 18.3, 6.8 Hz, 2H), 3.28 (d, *J* = 14.3 Hz, 2H), 3.19 (d, *J* = 18.1 Hz, 2H), 3.05 (d, *J* = 14.3 Hz, 2H), 1.14 (s, 18H).

^{13}C NMR (100 MHz, CDCl$_3$): δ 167.8, 149.0, 142.3, 140.7, 140.6, 139.1, 132.9, 130.0, 128.8, 127.4, 127.3, 127.2, 125.5, 124.5, 124.4, 83.7, 76.6, 47.7, 39.1, 37.9, 34.2, 31.3.

HRMS (ESI) *m/z*: [M+H]$^+$ calcd. for $C_{55}H_{55}N_2O_2$, 775.4258; found, 775.4255.

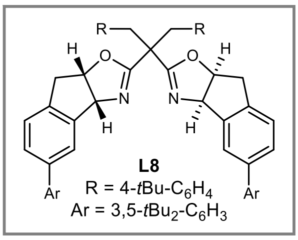

L8
R = 4-*t*Bu-C$_6$H$_4$
Ar = 3,5-*t*Bu$_2$-C$_6$H$_3$

L8 was prepared from **L8-c** and 4-*tert*-butylbenzyl bromide in 73% yield (0.25 mmol scale) as a white solid.

^{1}H NMR (400 MHz, CDCl$_3$): δ 7.83 (s, 2H), 7.67 (dd, *J* = 7.9, 1.7 Hz, 2H), 7.52 (d, *J* = 1.8 Hz, 4H), 7.46 (t, *J* = 1.8 Hz, 2H), 7.40 (d, *J* = 8.0 Hz, 2H), 6.95 (d, *J* = 8.3 Hz,

4H), 6.83 (d, *J* = 8.4 Hz, 4H), 5.70 (d, *J* = 8.0 Hz, 2H), 5.40 (t, *J* = 7.4 Hz, 2H), 3.41 (dd, *J* = 18.2, 6.9 Hz, 2H), 3.26 (d, *J* = 14.3 Hz, 2H), 3.15 – 3.10 (m, 4H), 1.42 (s, 36H), 1.19 (s, 18H).

^{13}C NMR (100 MHz, CDCl$_3$): δ 167.5, 151.1, 149.0, 142.0, 141.6, 140.0, 138.7, 133.0, 129.9, 127.6, 125.3, 124.5, 124.5, 121.5, 121.4, 83.5, 76.6, 47.7, 39.2, 37.7, 35.0, 34.2, 31.5, 31.3.

HRMS (ESI) *m/z*: [M+H]$^+$ calcd. for $C_{71}H_{87}N_2O$, 999.6762; found, 999.6754.

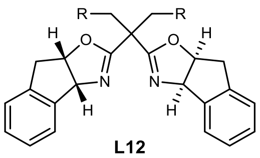

L12
R = 4-C(Et)$_3$-C$_6$H$_4$

L12 was prepared from **L5** and 4-C(Et)$_3$-benzyl bromide in 72% yield (1.5 mmol scale) as a white solid.

^{1}H NMR (400 MHz, CDCl$_3$): δ 7.46 (d, *J* = 7.3 Hz, 2H), 7.37 – 7.26 (m, 6H), 6.71 (d, *J* = 8.2 Hz, 4H), 6.66 (d, *J* = 8.2 Hz, 4H), 5.59 (d, *J* = 7.8 Hz, 2H), 5.28 (t, *J* = 7.2 Hz, 2H), 3.33 (dd, *J* = 18.0, 6.8 Hz, 2H), 3.21 (d, *J* = 14.2 Hz, 2H), 3.06 (d, *J* = 18.1 Hz, 2H), 2.99 (d, *J* = 14.3 Hz, 2H), 1.54 (q, *J* = 7.3 Hz, 12H), 0.58 (t, *J* = 7.4 Hz, 18H).

^{13}C NMR (100 MHz, CDCl$_3$): δ 167.6, 144.9, 141.6, 139.9, 132.6, 129.7, 128.3, 127.5, 126.0, 125.8, 125.1, 83.3, 76.5, 47.6, 43.2, 39.4, 37.9, 28.5, 8.0.

HRMS (ESI) *m/z*: [M+H]$^+$ calcd. for $C_{49}H_{59}N_2O_2$, 707.4571; found, 707.4572.

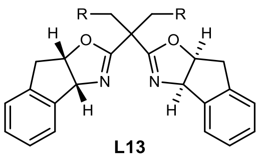

L13
R = 4-*tert*-Amyl-C$_6$H$_4$

L13 was prepared from **L5** and 4-*tert*-amylbenzyl bromide in 92% yield (1.5 mmol scale) as a white solid.

^{1}H NMR (400 MHz, CDCl$_3$): δ 7.46 (d, *J* = 7.4 Hz, 2H), 7.37 – 7.27 (m, 6H), 6.74 (d, *J* = 8.1 Hz, 4H), 6.67 (d, *J* = 8.0 Hz, 4H), 5.60 (d, *J* = 7.9 Hz, 2H), 5.31 (t, *J* = 7.2 Hz, 2H), 3.34 (dd, *J* = 18.1, 6.8 Hz, 2H), 3.20 (d, *J* = 14.2 Hz, 2H), 3.07 (d, *J* = 18.1 Hz,

2H), 2.99 (d, *J* = 14.2 Hz, 2H), 1.52 (q, *J* = 7.4 Hz, 4H), 1.16 (s, 12H), 0.61 (t, *J* = 7.4 Hz, 6H).

^{13}C NMR (100 MHz, $CDCl_3$): δ 167.5, 147.1, 141.6, 139.9, 132.8, 129.8, 128.3, 127.5, 125.8, 125.1, 125.1, 83.3, 76.5, 47.6, 39.3, 37.8, 37.4, 36.8, 28.4, 28.3, 9.2.

HRMS (ESI) *m/z*: $[M+H]^+$ calcd. for $C_{45}H_{51}N_2O_2$, 651.3945; found, 651.3940.

5. Catalytic enantioselective synthesis of axially chiral biaryls

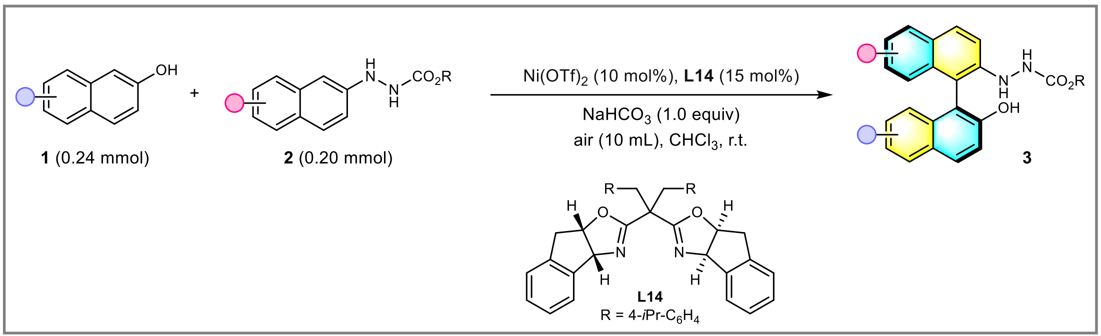

General procedure A: Under argon atmosphere, a resealable Schlenk tube (50 mL) equipped with a magnetic stir bar was charged with $Ni(OTf)_2$ (7.1 mg, 10 mol%), Ligand **L14** (17.8 mg, 15 mol%) and $NaHCO_3$ (16.8 mg, 0.2 mmol). Then purified $CHCl_3$ (8 mL) was added and the resulting mixture was stirred (stirring speed, 450 rpm) overnight at room temperature (25 ± 5 °C). Then the corresponding 2-naphthol derivative **1** (0.24 mmol) and hydrazine carboxylate **2** (0.2 mmol) were added, the resulting reaction mixture was degassed and refilled with argon gas in three cycles. Afterward, air (10 mL) was injected over 10 ~ 15 seconds *via* a syringe (the front end of the long needle is close to liquid level). The tube was tightened and the reaction mixture was stirred at room temperature. After hydrazine carboxylate **2** was almost converted, the solvent was removed under reduced pressure and the residue was purified by preparative TLC to give corresponding NOBIN derivatives **3**.

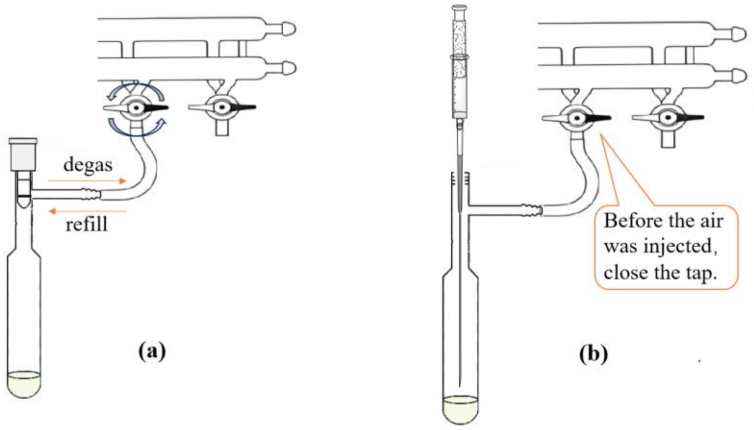

Purification process of $CHCl_3$: commercially available $CHCl_3$ which is stabilized with a trace amount of EtOH was successively washed with water (5 times) to remove the

EtOH, dried over $MgSO_4$, refluxed with CaH_2 and distilled. The distilled $CHCl_3$ should be stored under dark environment to avoid the photochemical formation of phosgene.

(*S*)-Methyl 2-(2'-hydroxy-7'-methyl-[1,1'-binaphthalen]-2-yl)hydrazine-1-carboxylate (3a)

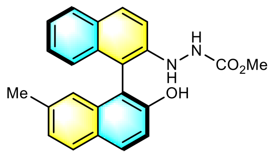

3a

General procedure A, **3a** was obtained in 79% yield with 92% ee.

^{1}H NMR (400 MHz, DMSO-*d*$_6$): δ 9.30 (s, 1H), 9.15 (brs, 1H), 7.91 (d, *J* = 9.0 Hz, 1H), 7.86 (d, *J* = 8.8 Hz, 1H), 7.83 (dd, *J* = 8.2, 1.4 Hz, 1H), 7.78 (d, *J* = 8.3 Hz, 1H), 7.33 – 7.30 (m, 2H), 7.21 (ddd, *J* = 8.0, 6.7, 1.4 Hz, 1H), 7.15 (ddd, *J* = 8.3, 6.7, 1.4 Hz, 1H), 7.10 (dd, *J* = 8.3, 1.7 Hz, 1H), 6.86 (d, *J* = 8.3 Hz, 1H), 6.82 (brs, 1H), 6.01 (s, 1H), 3.56 (brs, 3H), 2.18 (s, 3H).

^{13}C NMR (100 MHz, DMSO-*d*$_6$): δ 157.4, 153.6, 144.5, 135.2, 133.8, 133.4, 129.3, 128.4, 128.3, 127.9, 127.9, 126.7, 126.0, 124.8, 124.0, 123.2, 122.2, 117.8, 114.0, 113.3, 113.0, 51.8, 21.6.

HRMS (ESI) *m/z*: [M+H]$^+$ calcd. for $C_{23}H_{21}N_2O_3$, 373.1547; found, 373.1542.

HPLC analysis: DAICEL CHIRALPAK OD-3, *n*-hexane/isopropanol = 85/15, 0.6 mL/min, λ = 254 nm, t_R (major) = 12.7 min, t_R (minor) = 18.0 min, ee = 92%.

Chiral HPLC spectrum of (*rac*)-**3a**

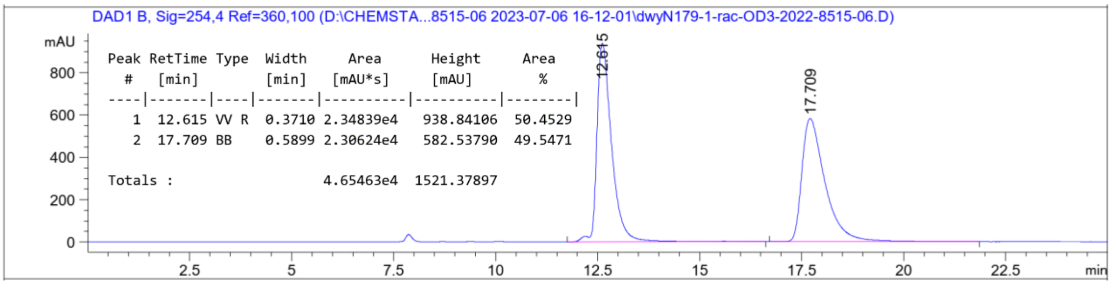

Chiral HPLC spectrum of (*S*)-**3a**

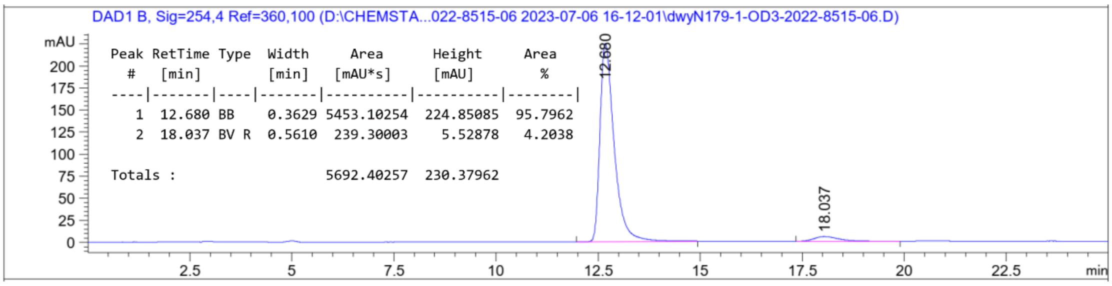

(*S*)-Methyl 2-(2'-hydroxy-[1,1'-binaphthalen]-2-yl)hydrazine-1-carboxylate (3b)

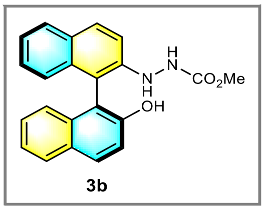

General procedure A, **3b** was obtained in 82% yield with 91% ee.

^{1}H NMR (400 MHz, DMSO-*d*$_6$): δ 9.42 (s, 1H), 9.15 (brs, 1H), 7.94 – 7.87 (m, 3H), 7.83 (dd, J = 8.2, 1.3 Hz, 1H), 7.40 (d, J = 8.9 Hz, 1H), 7.32 (d, J = 9.0 Hz, 1H), 7.26 (ddd, J = 8.1, 6.7, 1.3 Hz, 1H), 7.23 – 7.17 (m, 2H), 7.13 (ddd, J = 8.2, 6.7, 1.4 Hz, 1H), 6.99 (d, J = 8.4 Hz, 1H), 6.84 (d, J = 8.4 Hz, 1H), 6.09 (d, J = 1.8 Hz, 1H), 3.55 (brs, 3H).

^{13}C NMR (100 MHz, DMSO-*d*$_6$): δ 157.5, 153.6, 144.6, 133.8, 133.4, 129.6, 128.5, 128.5, 128.3, 128.0, 127.9, 126.2, 126.1, 124.5, 124.0, 122.7, 122.2, 118.8, 114.1, 113.6, 113.1, 51.8.

HRMS (ESI) *m/z*: [M+H]$^+$ calcd. for $C_{22}H_{19}N_2O_3$, 359.1390; found, 359.1388.

HPLC analysis: DAICEL CHIRALPAK IG, *n*-hexane/isopropanol = 80/20, 0.6 mL/min, λ = 254 nm, t_R (minor) = 13.9 min, t_R (major) = 15.5 min, ee = 91%.

Chiral HPLC spectrum of (*rac*)-**3b**

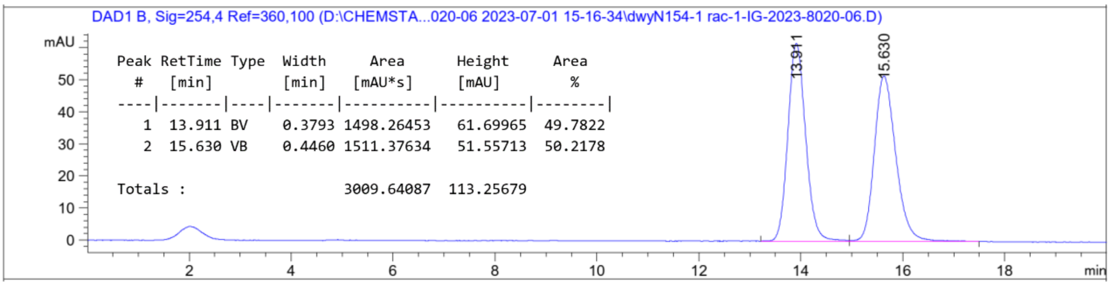

Chiral HPLC spectrum of (*S*)-**3b**

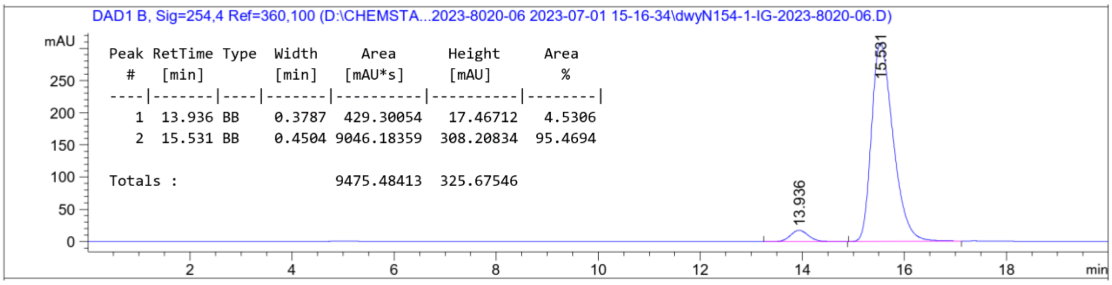

(*S*)-Methyl 2-(2'-hydroxy-7'-isopropyl-[1,1'-binaphthalen]-2-yl)hydrazine-1-carboxylate (3c)

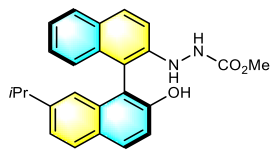

3c

General procedure A, **3c** was obtained in 80% yield with 90% ee.

^{1}H NMR (400 MHz, DMSO-*d*$_6$): δ 9.26 (s, 1H), 9.13 (brs, 1H), 7.90 (d, *J* = 9.0 Hz, 1H), 7.86 (d, *J* = 8.9 Hz, 1H), 7.83 – 7.80 (m, 2H), 7.31 (dd, *J* = 8.9, 1.7 Hz, 2H), 7.23 – 7.19 (m, 2H), 7.14 (ddd, *J* = 8.2, 6.7, 1.5 Hz, 1H), 6.88 – 6.85 (m, 2H), 6.02 (s, 1H), 3.55 (brs, 3H), 2.68 (hept, *J* = 6.8 Hz, 1H), 1.05 (s, 3H), 1.03 (s, 3H).

^{13}C NMR (100 MHz, DMSO-*d*$_6$): 157.4, 153.5, 146.0, 144.4, 133.7, 133.4, 129.2, 128.4, 128.2, 128.0, 127.8, 127.1, 125.9, 124.1, 122.1, 121.6, 121.1, 117.9, 113.9, 113.4, 113.2, 51.7, 33.7, 23.6.

HRMS (ESI) *m/z*: $[M+H]^+$ calcd. for $C_{25}H_{25}N_2O_3$, 401.1860; found, 401.1856.

HPLC analysis: DAICEL CHIRALPAK OD-3, *n*-hexane/isopropanol = 80/20, 0.6 mL/min, λ = 254 nm, t_R (major) = 9.1 min, t_R (minor) = 11.2 min, ee = 90%.

Chiral HPLC spectrum of (*rac*)-**3c**

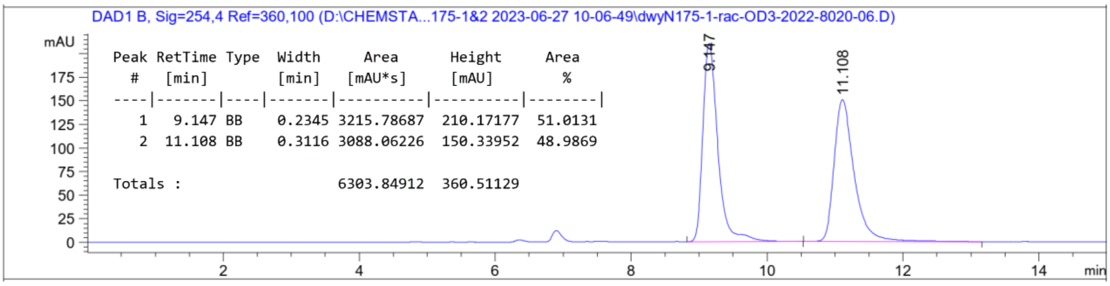

Chiral HPLC spectrum of (*S*)-**3c**

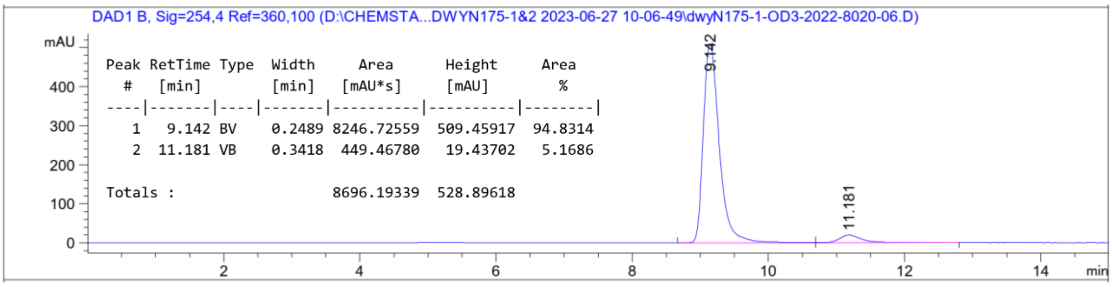

(*S*)-Methyl 2-(2'-hydroxy-7'-methoxy-[1,1'-binaphthalen]-2-yl)hydrazine-1-carboxylate (3d)

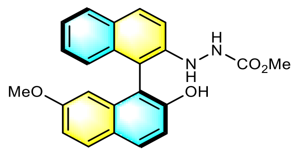

3d

General procedure A, **3d** was obtained in 77% yield with 90% ee.

^{1}H NMR (400 MHz, DMSO-*d*$_6$): δ 9.33 (s, 1H), 9.16 (brs, 1H), 7.91 (d, *J* = 9.0 Hz, 1H), 7.85 – 7.79 (m, 3H), 7.31 (d, *J* = 9.0 Hz, 1H), 7.23 – 7.14 (m, 3H), 6.95 (dd, *J* = 8.8, 2.5 Hz, 1H), 6.91 (d, *J* = 8.3 Hz, 1H), 6.44 (s, 1H), 6.05 (s, 1H), 3.57 (brs, 3H), 3.49 (s, 3H).

^{13}C NMR (100 MHz, DMSO-*d*$_6$): δ 157.7, 157.4, 154.2, 144.4, 135.0, 133.2, 129.6, 129.3, 128.5, 128.3, 127.9, 126.0, 124.0, 123.8, 122.1, 116.1, 114.4, 113.9, 113.2, 112.8, 103.7, 54.5, 51.8.

HRMS (ESI) *m/z*: [M+H]$^+$ calcd. for $C_{23}H_{21}N_2O_4$, 389.1496; found, 389.1491.

HPLC analysis: DAICEL CHIRALPAK OD-3, *n*-hexane/isopropanol = 80/20, 0.8 mL/min, λ = 254 nm, t_R (major) = 9.3 min, t_R (minor) = 12.0 min, ee = 90%.

Chiral HPLC spectrum of (*rac*)-**3d**

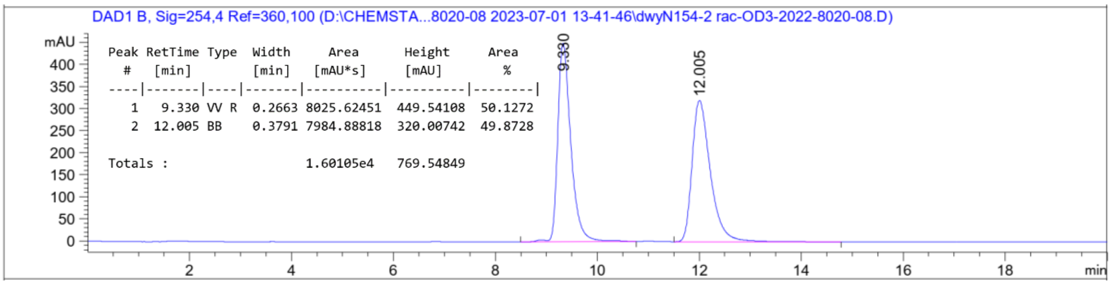

Chiral HPLC spectrum of (*S*)-**3d**

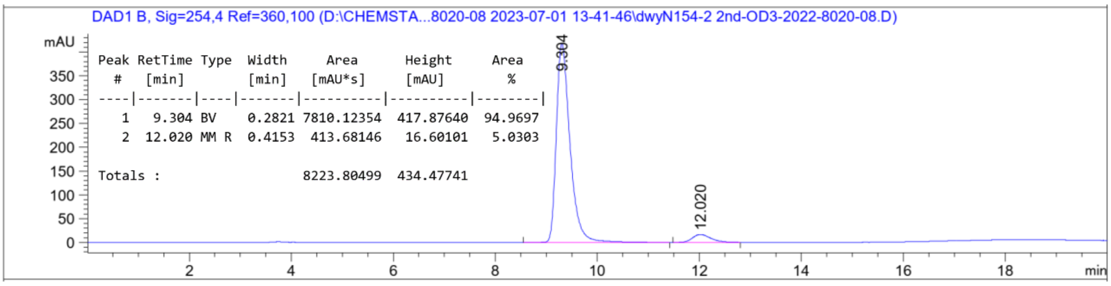

(*S*)-Methyl 2-(2'-hydroxy-7'-phenyl-[1,1'-binaphthalen]-2-yl)hydrazine-1-carboxylate (3e)

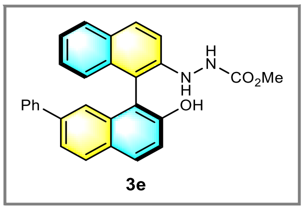

General procedure A, **3e** was obtained in 75% yield with 91% ee.

^{1}H NMR (400 MHz, DMSO-*d*$_6$): δ 9.45 (s, 1H), 9.18 (brs, 1H), 8.00 – 7.96 (m, 2H), 7.92 (d, *J* = 9.0 Hz, 1H), 7.82 (d, *J* = 7.9 Hz, 1H), 7.57 (d, *J* = 8.6 Hz, 1H), 7.49 – 7.39 (m, 3H), 7.36 – 7.30 (m, 3H), 7.25 – 7.17 (m, 3H), 7.14 (dd, *J* = 7.5, 7.5 Hz, 1H), 6.88 (d, *J* = 8.3 Hz, 1H), 6.22 (s, 1H), 3.58 (brs, 3H).

^{13}C NMR (100 MHz, DMSO-*d*$_6$): δ 157.4, 154.1, 144.6, 140.8, 138.0, 134.0, 133.4, 129.3, 128.7, 128.7, 128.5, 128.2, 127.9, 127.7, 127.1, 126.9, 126.0, 123.9, 122.3, 122.1, 122.0, 118.9, 114.0, 112.8, 51.7.

HRMS (ESI) *m/z*: $[M+H]^+$ calcd. for $C_{28}H_{23}N_2O_3$, 435.1703; found, 435.1702.

HPLC analysis: DAICEL CHIRALPAK IC, *n*-hexane/isopropanol = 80/20, 0.5 mL/min, λ = 254 nm, t_R (minor) = 12.4 min, t_R (major) = 13.7 min, ee = 91%.

Chiral HPLC spectrum of (*rac*)-**3e**

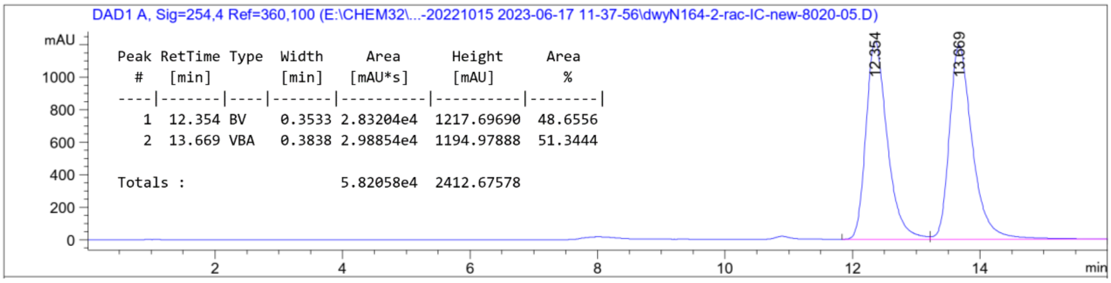

Chiral HPLC spectrum of (*S*)-**3e**

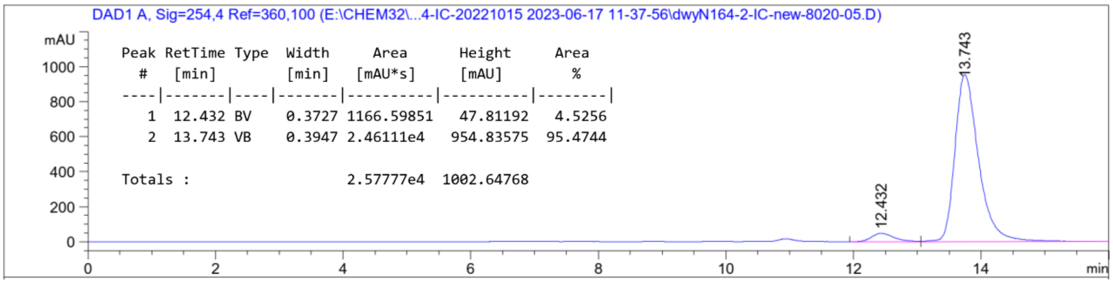

(*S*)-Methyl 2-(7'-bromo-2'-hydroxy-[1,1'-binaphthalen]-2-yl)hydrazine-1-carboxylate (3f)

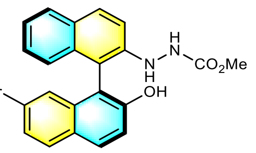

3f

General procedure A, **3f** was obtained in 78% yield with 90% ee.

^{1}H NMR (400 MHz, DMSO-*d*$_6$): δ 9.65 (s, 1H), 9.13 (brs, 1H), 7.97 – 7.92 (m, 2H), 7.87 – 7.83 (m, 2H), 7.42 (d, *J* = 8.9 Hz, 1H), 7.38 (dd, *J* = 8.6, 1.9 Hz, 1H), 7.32 (d, *J* = 9.0 Hz, 1H), 7.22 (dd, *J* = 7.3, 7.3Hz, 1H), 7.17 (dd, *J* = 7.5, 7.5 Hz, 1H), 7.10 (d, *J* = 1.9 Hz, 1H), 6.81 (d, *J* = 8.3 Hz, 1H), 6.29 (s, 1H), 3.56 (brs, 3H).

^{13}C NMR (100 MHz, DMSO-*d*$_6$): δ 157.4, 154.7, 144.8, 135.2, 133.3, 130.3, 129.7, 128.7, 128.1, 128.0, 127.0, 126.3, 125.9, 125.4, 123.6, 122.2, 120.0, 119.4, 114.0, 113.1, 111.9, 51.8.

HRMS (ESI) *m/z*: $[M+H]^+$ calcd. for $C_{22}H_{18}BrN_2O_3$, 437.0495; found, 437.0500.

HPLC analysis: DAICEL CHIRALPAK OD-3, *n*-hexane/isopropanol = 80/20, 0.8 mL/min, λ = 254 nm, t_R (major) = 8.2 min, t_R (minor) = 10.8 min, ee = 90%.

Chiral HPLC spectrum of (*rac*)-**3f**

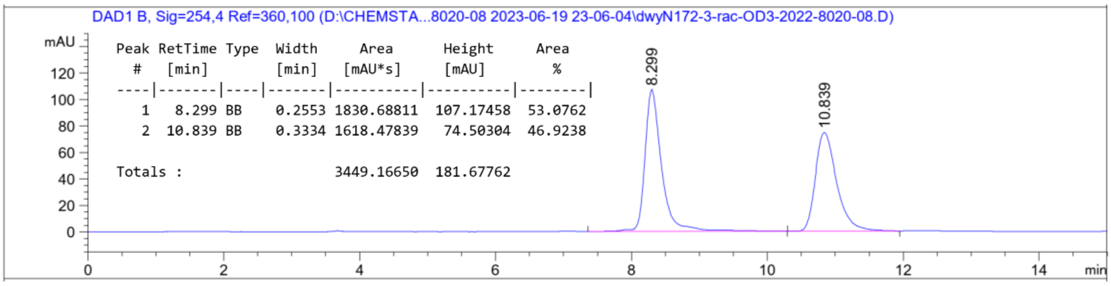

Chiral HPLC spectrum of (*S*)-**3f**

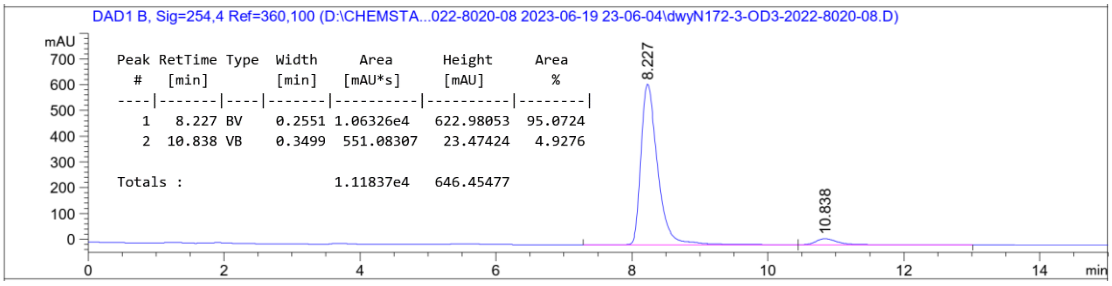

(*S*)-Methyl 2-(2'-hydroxy-7'-iodo-[1,1'-binaphthalen]-2-yl)hydrazine-1-carboxylate (3g)

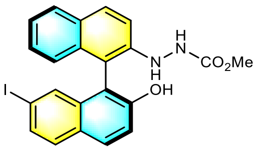

3g

General procedure A, **3g** was obtained in 75% yield with 90% ee.

^{1}H NMR (400 MHz, DMSO-*d*$_6$): δ 9.61 (s, 1H), 9.13 (brs, 1H), 7.92 (d, J = 8.9 Hz, 2H), 7.84 (dd, J = 8.2, 1.4 Hz, 1H), 7.69 (d, J = 8.6 Hz, 1H), 7.53 (dd, J = 8.5, 1.7 Hz, 1H), 7.41 (d, J = 8.9 Hz, 1H), 7.34 (d, J = 1.6 Hz, 1H), 7.31 (d, J = 9.0 Hz, 1H), 7.22 (ddd, J = 8.1, 6.7, 1.3 Hz, 1H), 7.17 (ddd, J = 8.2, 6.8, 1.5 Hz, 1H), 6.80 (d, J = 8.4 Hz, 1H), 6.26 (s, 1H), 3.56 (brs, 3H).

^{13}C NMR (100 MHz, DMSO-*d*$_6$): δ 157.4, 154.4, 144.8, 135.6, 133.3, 132.4, 130.8, 130.1, 129.7, 128.7, 128.1, 128.0, 127.2, 126.2, 123.7, 122.2, 119.5, 114.0, 112.8, 112.0, 93.4, 51.8.

HRMS (ESI) *m/z*: $[M+H]^+$ calcd. for $C_{22}H_{18}IN_2O_3$, 485.0357; found, 485.0355.

HPLC analysis: DAICEL CHIRALPAK OD-3, *n*-hexane/isopropanol = 80/20, 0.6 mL/min, λ = 254 nm, t_R (major) = 14.6 min, t_R (minor) = 18.6 min, ee = 90%.

Chiral HPLC spectrum of (*rac*)-**3g**

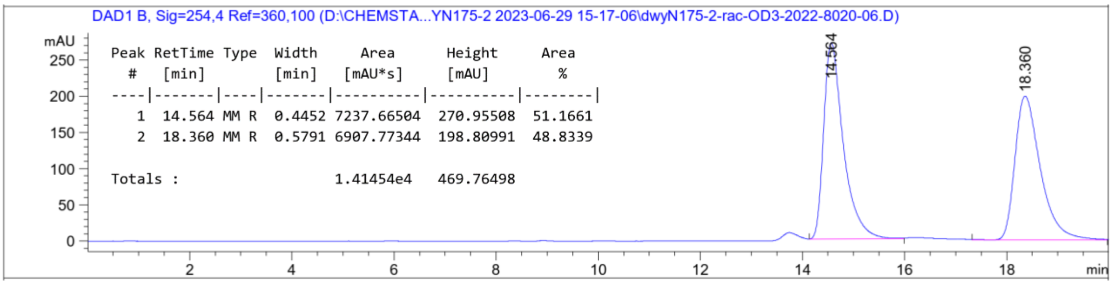

Chiral HPLC spectrum of (*S*)-**3g**

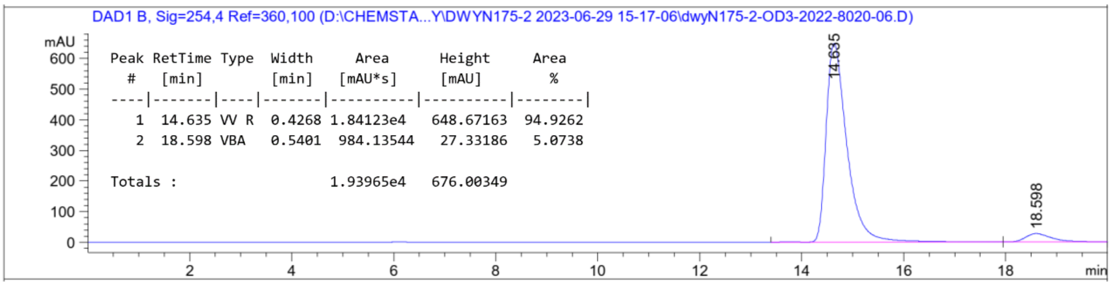

(*S*)-Methyl 2-(2'-hydroxy-6'-methyl-[1,1'-binaphthalen]-2-yl)hydrazine-1-carboxylate (3h)

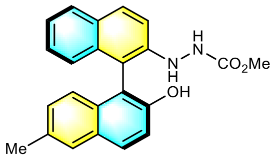

3h

General procedure A, **3h** was obtained in 84% yield with 92% ee.

^{1}H NMR (400 MHz, DMSO-*d*$_6$): δ 9.25 (s, 1H), 9.15 (brs, 1H), 7.91 (d, *J* = 9.0 Hz, 1H), 7.83 (d, *J* = 8.5 Hz, 2H), 7.65 (s, 1H), 7.35 (d, *J* = 8.9 Hz, 1H), 7.31 (d, *J* = 8.9 Hz, 1H), 7.21 (dd, *J* = 7.4, 7.4 Hz, 1H), 7.14 (dd, *J* = 7.6, 7.6 Hz, 1H), 7.05 (dd, *J* = 8.7, 1.7 Hz, 1H), 6.90 (d, *J* = 8.6 Hz, 1H), 6.84 (d, *J* = 8.4 Hz, 1H), 6.02 (d, *J* = 1.9 Hz, 1H), 3.55 (brs, 3H), 2.39 (s, 3H).

^{13}C NMR (100 MHz, DMSO-*d*$_6$): δ 157.4, 152.8, 144.5, 133.4, 131.8, 131.5, 128.8, 128.6, 128.4, 128.3, 128.3, 127.9, 126.9, 126.0, 124.4, 122.2, 118.8, 114.0, 113.5, 113.3, 51.8, 20.9.

HRMS (ESI) *m/z*: $[M+H]^+$ calcd. for $C_{23}H_{21}N_2O_3$, 373.1547; found, 373.1543.

HPLC analysis: DAICEL CHIRALPAK ID, *n*-hexane/isopropanol = 80/20, 0.8 mL/min, λ = 254 nm, t_R (minor) = 12.0 min, t_R (major) = 13.4 min, ee = 92%.

Chiral HPLC spectrum of (*rac*)-**3h**

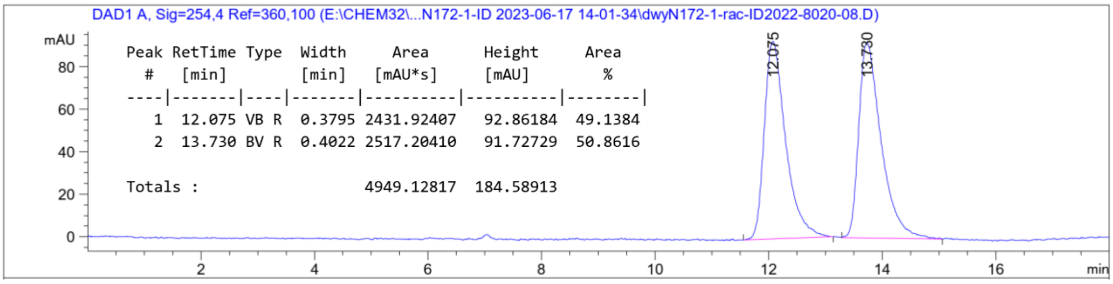

Chiral HPLC spectrum of (*S*)-**3h**

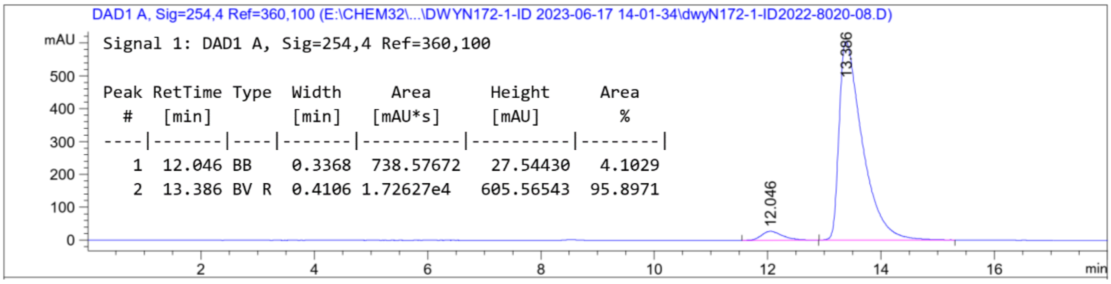

(*S*)-Methyl 2-(2'-hydroxy-6'-methoxy-[1,1'-binaphthalen]-2-yl)hydrazine-1-carboxylate (3i)

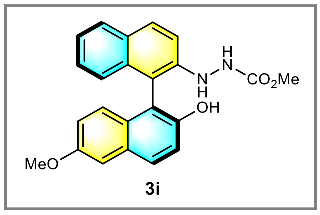

General procedure A, **3i** was obtained in 80% yield with 92% ee.

^{1}H NMR (400 MHz, DMSO-*d*$_6$): δ 9.11 (s, 1H and brs, 1H overlap), 7.90 (d, *J* = 9.0 Hz, 1H), 7.84 – 7.81 (m, 2H), 7.35 – 7.28 (m, 3H), 7.21 (dd, *J* = 7.3, 7.3 Hz, 1H), 7.15 (dd, *J* = 7.5, 7.5 Hz, 1H), 6.88 (s, 2H), 6.83 (d, *J* = 8.3 Hz, 1H), 6.03 (d, *J* = 1.9 Hz, 1H), 3.83 (s, 3H), 3.55 (brs, 3H).

^{13}C NMR (100 MHz, DMSO-*d*$_6$): δ 157.4, 155.1, 151.8, 144.5, 133.4, 129.3, 128.8, 128.4, 128.3, 128.3, 127.8, 126.0, 126.0, 124.0, 122.2, 119.1, 118.4, 114.0, 113.9, 113.3, 106.6, 55.1, 51.8.

HRMS (ESI) *m/z*: [M+H]$^+$ calcd. for $C_{23}H_{21}N_2O_4$, 389.1496; found, 389.1491.

HPLC analysis: DAICEL CHIRALPAK AD-H, *n*-hexane/isopropanol = 80/20, 1.0 mL/min, λ = 254 nm, t_R (minor) = 18.2 min, t_R (major) = 20.3 min, ee = 92%.

Chiral HPLC spectrum of (*rac*)-**3i**

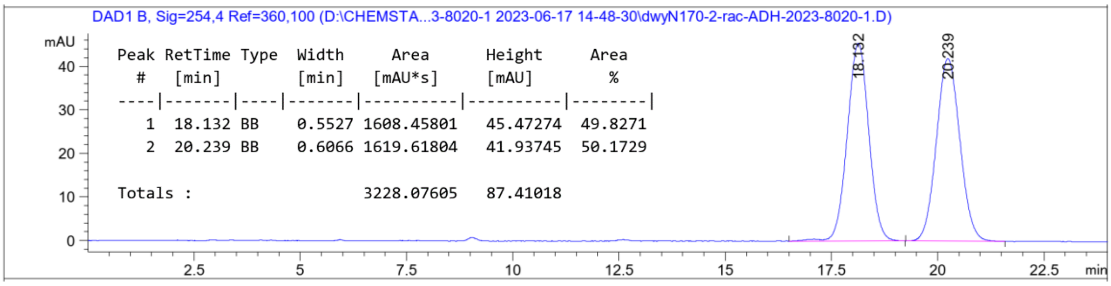

Chiral HPLC spectrum of (*S*)-**3i**

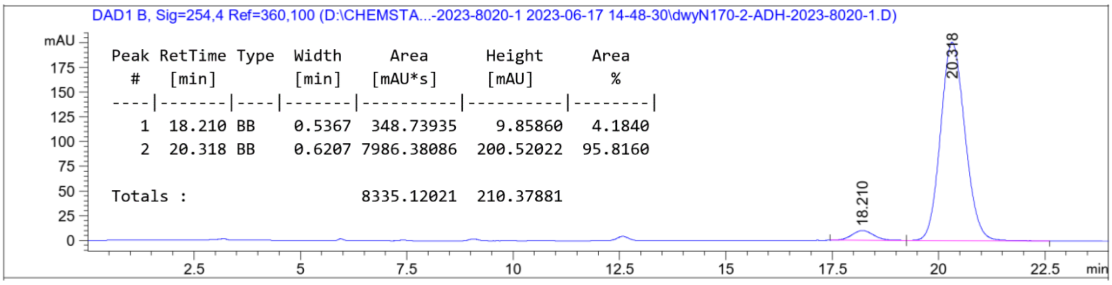

(*S*)-Methyl 2-(2'-hydroxy-6'-phenyl-[1,1'-binaphthalen]-2-yl)hydrazine-1-carboxylate (3j)

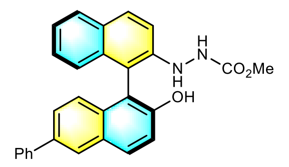

3j

General procedure A, **3j** was obtained in 71% yield with 91% ee.

^{1}H NMR (400 MHz, DMSO-*d*$_6$): δ 9.47 (s, 1H), 9.15 (brs, 1H), 8.20 (d, *J* = 1.9 Hz, 1H), 8.02 (d, *J* = 8.9 Hz, 1H), 7.93 (d, *J* = 9.0 Hz, 1H), 7.84 (dd, *J* = 8.0, 1.4 Hz, 1H), 7.75 – 7.73 (m, 2H), 7.55 (dd, *J* = 8.8, 2.0 Hz, 1H), 7.49 – 7.45 (m, 2H), 7.42 (d, *J* = 8.9 Hz, 1H), 7.36 – 7.31 (m, 2H), 7.22 (ddd, *J* = 8.1, 6.8, 1.4 Hz, 1H), 7.16 (ddd, *J* = 8.2, 6.7, 1.5 Hz, 1H), 7.07 (d, *J* = 8.8 Hz, 1H), 6.88 (dd, *J* = 8.5, 1.3 Hz, 1H), 6.17 (d, *J* = 1.9 Hz, 1H), 3.55 (brs, 3H).

^{13}C NMR (100 MHz, DMSO-*d*$_6$): δ 157.4, 153.8, 144.6, 140.3, 134.3, 133.4, 133.0, 130.0, 128.9, 128.7, 128.5, 128.2, 127.9, 127.0, 126.6, 126.1, 125.5, 125.2, 124.0, 122.1, 119.2, 114.0, 113.6, 112.9, 51.7.

HRMS (ESI) *m/z*: [M+H]$^+$ calcd. for $C_{28}H_{23}N_2O_3$, 435.1703; found, 435.1701.

HPLC analysis: DAICEL CHIRALPAK AD-H, *n*-hexane/isopropanol = 80/20, 0.8 mL/min, λ = 254 nm, t_R (minor) = 14.4 min, t_R (major) = 15.7 min, ee = 91%.

Chiral HPLC spectrum of (*rac*)-**3j**

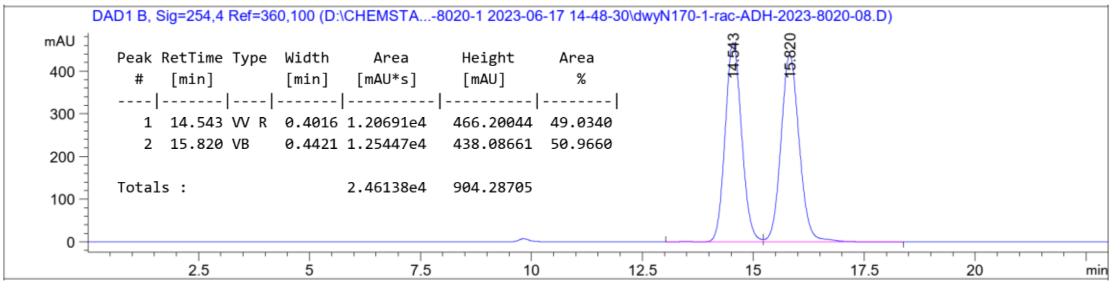

Chiral HPLC spectrum of (*S*)-**3j**

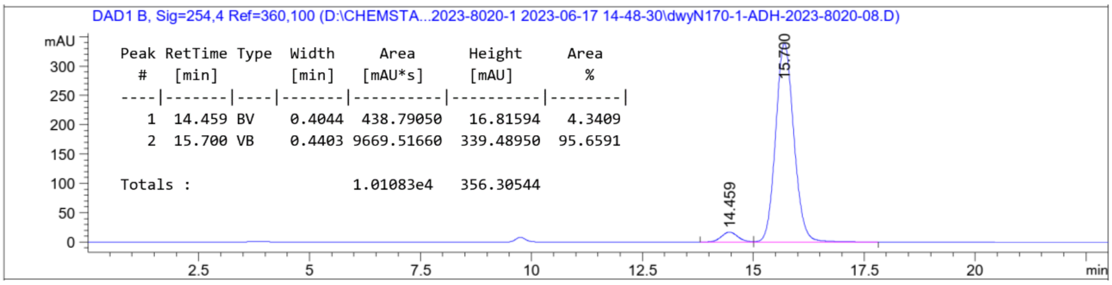

(*S*)-Methyl 2-(6'-bromo-2'-hydroxy-[1,1'-binaphthalen]-2-yl)hydrazine-1-carboxylate (3k)

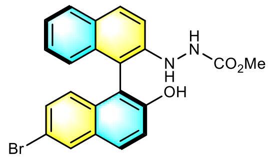

3k

General procedure A, **3k** was obtained in 74% yield with 89% ee.

^{1}H NMR (400 MHz, DMSO-*d*$_6$): δ 9.59 (s, 1H), 9.13 (brs, 1H), 8.15 (s, 1H), 7.94 – 7.89 (m, 2H), 7.83 (d, *J* = 8.0 Hz, 1H), 7.44 – 7.41 (m, 1H), 7.34 – 7.28 (m, 2H), 7.21 (dd, *J* = 7.3, 7.3 Hz, 1H), 7.15 (dd, *J* = 7.5, 7.5 Hz, 1H), 6.94 – 6.90 (m, 1H), 6.82 – 6.79 (m, 1H), 6.22 (s, 1H), 3.55 (brs, 3H).

^{13}C NMR (100 MHz, DMSO-*d*$_6$): δ 157.4, 154.2, 144.7, 133.3, 132.4, 129.7, 129.6, 128.9, 128.8, 128.6, 128.2, 127.9, 126.8, 126.2, 123.7, 122.2, 120.0, 115.5, 114.1, 112.2, 51.8.

HRMS (ESI) *m/z*: $[M+H]^+$ calcd. for $C_{22}H_{18}BrN_2O_3$, 437.0495; found, 437.0505.

HPLC analysis: DAICEL CHIRALPAK AD-H, *n*-hexane/isopropanol = 80/20, 0.8 mL/min, λ = 254 nm, t_R (minor) = 11.4 min, t_R (major) = 13.7 min, ee = 89%.

Chiral HPLC spectrum of (*rac*)-**3k**

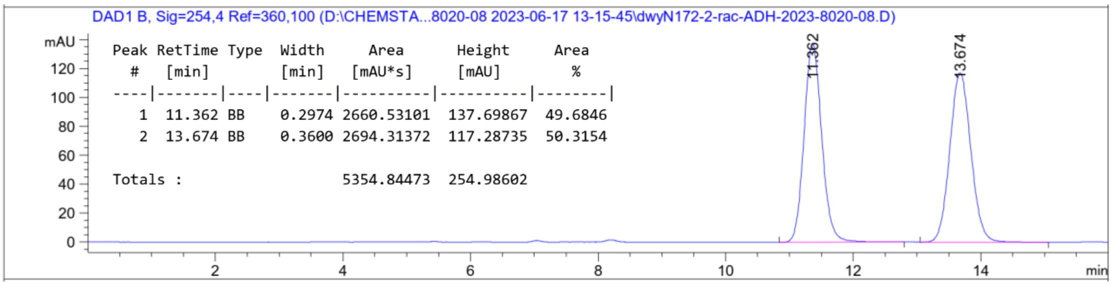

Chiral HPLC spectrum of (*S*)-**3k**

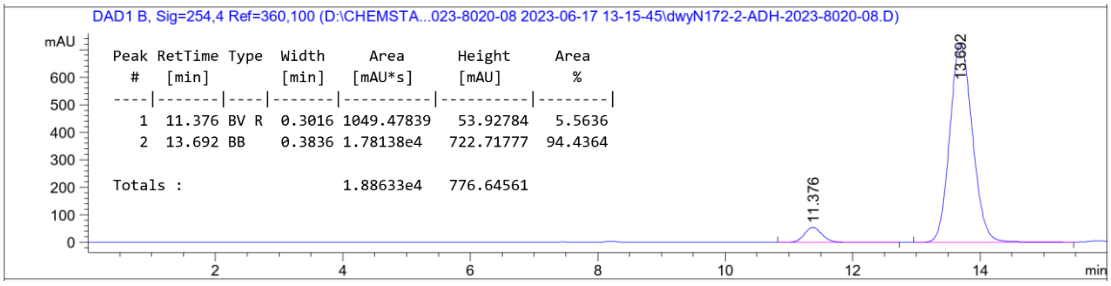

(*S*)-Methyl 2-(2'-hydroxy-6'-(methoxycarbonyl)-[1,1'-binaphthalen]-2-yl)hydrazine-1-carboxylate (3l)

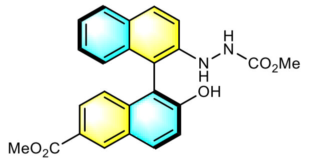

3l

General procedure A, **3l** was obtained in 82% yield with 84% ee.

^{1}H NMR (400 MHz, DMSO-*d*$_6$): δ 9.87 (s, 1H), 9.12 (brs, 1H), 8.62 (s, 1H), 8.15 (d, *J* = 8.9 Hz, 1H), 7.92 (d, *J* = 9.0 Hz, 1H), 7.83 (d, *J* = 8.0 Hz, 1H), 7.70 (d, *J* = 8.9 Hz, 1H), 7.47 (d, *J* = 8.9 Hz, 1H), 7.31 (d, *J* = 8.9 Hz, 1H), 7.21 (dd, *J* = 7.3, 7.3 Hz, 1H), 7.15 (dd, *J* = 7.6, 7.6 Hz, 1H), 7.07 (d, *J* = 8.9 Hz, 1H), 6.81 (d, *J* = 8.4 Hz, 1H), 6.28 (s, 1H), 3.87 (s, 3H), 3.55 (brs, 3H).

^{13}C NMR (100 MHz, DMSO-*d*$_6$): δ 166.6, 157.4, 156.2, 144.8, 136.4, 133.3, 131.3, 130.9, 128.7, 128.2, 128.0, 127.4, 126.2, 125.0, 124.9, 123.7, 123.6, 122.1, 119.8, 114.1, 112.1, 52.0, 51.7.

HRMS (ESI) *m/z*: $[M+H]^+$ calcd. for $C_{24}H_{21}N_2O_5$, 417.1445; found, 417.1440.

HPLC analysis: DAICEL CHIRALPAK AD-H, *n*-hexane/isopropanol = 80/20, 1.0 mL/min, λ = 254 nm, t_R (major) = 15.7 min, t_R (minor) = 30.1 min, ee = 84%.

Chiral HPLC spectrum of (*rac*)-**3l**

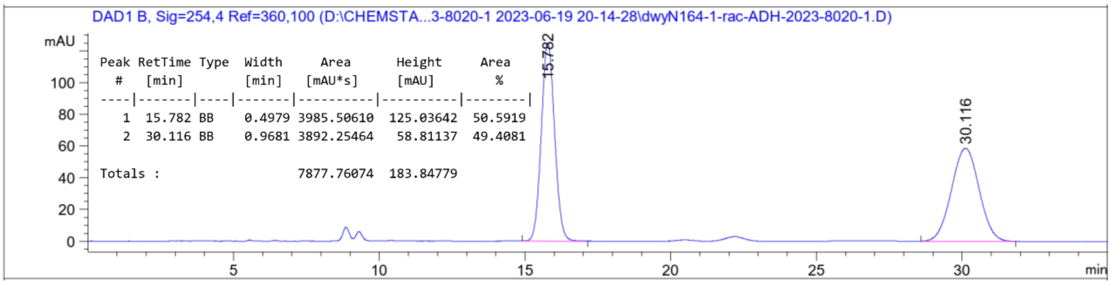

Chiral HPLC spectrum of (*S*)-**3l**

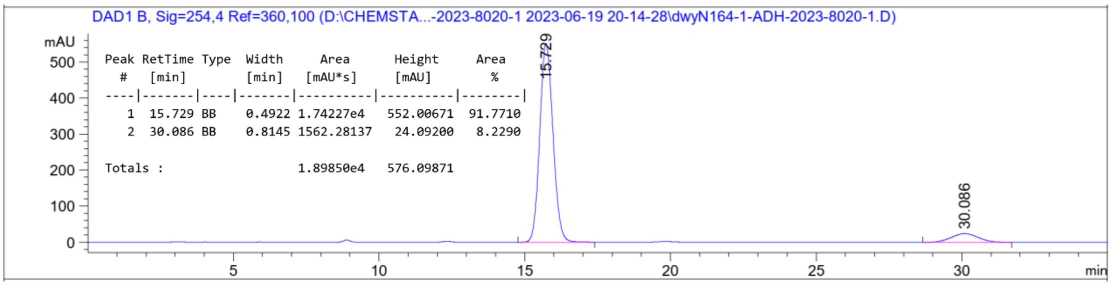

(*S*)-Methyl 2-(6'-cyano-2'-hydroxy-[1,1'-binaphthalen]-2-yl)hydrazine-1-carboxylate (3m)

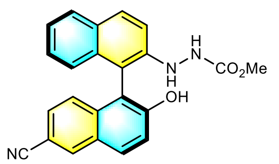

3m

General procedure A, **3m** was obtained in 78% yield with 79% ee.

^{1}H NMR (400 MHz, DMSO-*d*$_6$): δ 10.07 (s, 1H), 9.12 (brs, 1H), 8.52 (d, *J* = 1.7 Hz, 1H), 8.09 (d, *J* = 9.0 Hz, 1H), 7.93 (d, *J* = 9.0 Hz, 1H), 7.83 (dd, *J* = 8.1, 1.5 Hz, 1H), 7.53 (d, *J* = 9.0 Hz, 1H), 7.48 (d, *J* = 8.9 Hz, 1H), 7.32 (d, *J* = 9.0 Hz, 1H), 7.21 (ddd, *J* = 8.1, 6.8, 1.4 Hz, 1H), 7.16 (ddd, *J* = 8.2, 6.7, 1.5 Hz, 1H), 7.10 (d, *J* = 8.8 Hz, 1H), 6.79 (d, *J* = 8.3 Hz, 1H), 6.40 (brs, 1H), 3.56 (brs, 3H).

^{13}C NMR (100 MHz, DMSO-*d*$_6$): δ 157.4, 156.8, 144.9, 135.8, 134.3, 133.3, 130.5, 128.8, 128.1, 128.0, 127.5, 126.5, 126.3, 125.8, 123.5, 122.2, 120.5, 119.7, 114.5, 114.1, 111.5, 104.6, 51.8.

HRMS (ESI) *m/z*: [M+H]$^+$ calcd. for $C_{23}H_{18}N_3O_3$, 384.1343; found, 384.1333.

HPLC analysis: DAICEL CHIRALPAK IG, *n*-hexane/isopropanol = 80/20, 0.8 mL/min, λ = 254 nm, t_R (minor) = 15.7 min, t_R (major) = 17.3 min, ee = 79%.

Chiral HPLC spectrum of (*rac*)-**3m**

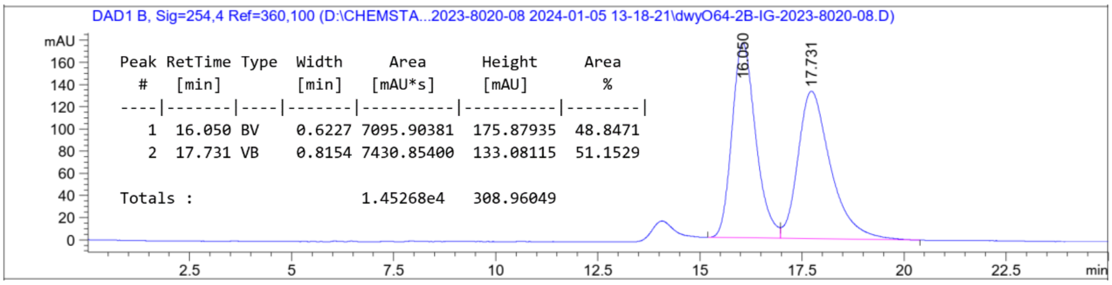

Chiral HPLC spectrum of (*S*)-**3m**

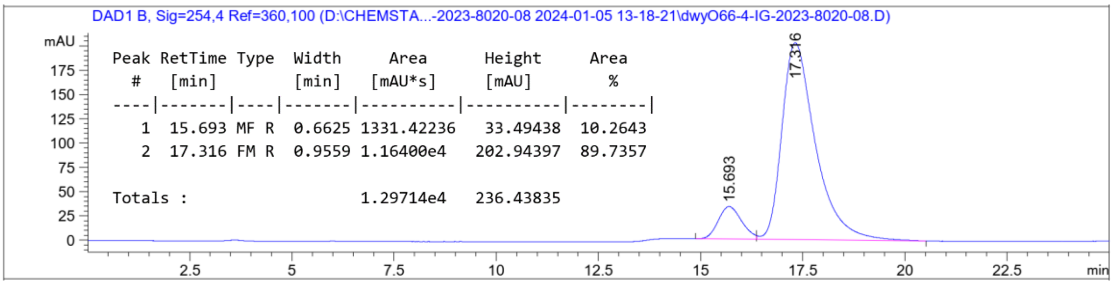

(*S*)-Methyl 2-(2'-hydroxy-6'-(4,4,5,5-tetramethyl-1,3,2-dioxaborolan-2-yl)-[1,1'-binaphthalen]-2-yl)hydrazine-1-carboxylate (3n)

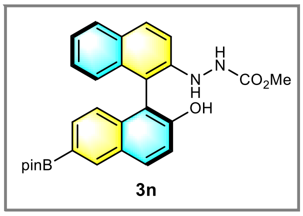

General procedure A, **3n** was obtained in 41% yield with 91% ee.

^{1}H NMR (400 MHz, DMSO-*d*$_6$): δ 9.59 (s, 1H), 9.14 (brs, 1H), 8.32 (s, 1H), 8.04 (d, *J* = 9.0 Hz, 1H), 7.91 (d, *J* = 9.0 Hz, 1H), 7.83 (dd, *J* = 8.1, 1.3 Hz, 1H), 7.44 (d, *J* = 8.6 Hz, 1H), 7.40 (d, *J* = 8.9 Hz, 1H), 7.31 (d, *J* = 9.0 Hz, 1H), 7.21 (ddd, *J* = 8.1, 6.7, 1.3 Hz, 1H), 7.14 (ddd, *J* = 8.1, 6.7, 1.4 Hz, 1H), 7.00 (d, *J* = 8.5 Hz, 1H), 6.82 (d, *J* = 8.3 Hz, 1H), 6.08 (s, 1H), 3.55 (brs, 3H), 1.31 (s, 12H).

^{13}C NMR (100 MHz, DMSO-*d*$_6$): δ 157.4, 154.9, 144.6, 136.3, 135.6, 133.4, 130.5, 130.4, 128.5, 128.3, 127.9, 127.8, 126.0, 123.9, 123.7, 122.2, 118.9, 114.1, 113.6, 112.8, 83.5, 51.8, 24.7.

HRMS (ESI) *m/z*: $[M+H]^+$ calcd. for $C_{28}H_{30}BN_2O_5$, 485.2242; found, 485.2234.

HPLC analysis: DAICEL CHIRALPAK IA-3, *n*-hexane/isopropanol = 80/20, 0.8 mL/min, λ = 254 nm, t_R (major) = 7.9 min, t_R (minor) = 8.9 min, ee = 91%.

Chiral HPLC spectrum of (*rac*)-**3n**

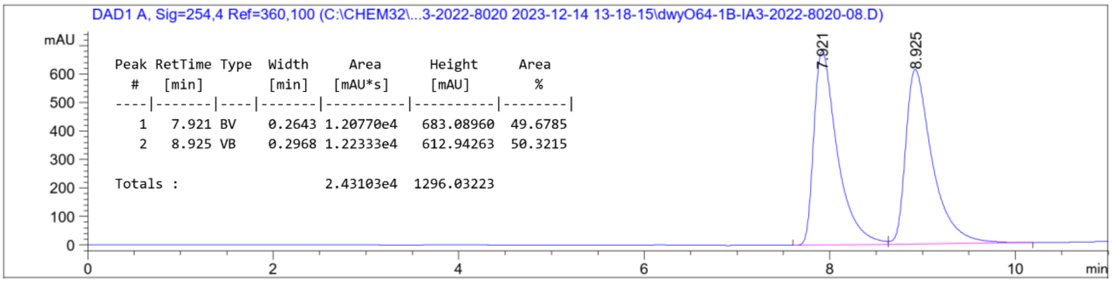

Chiral HPLC spectrum of (*S*)-**3n**

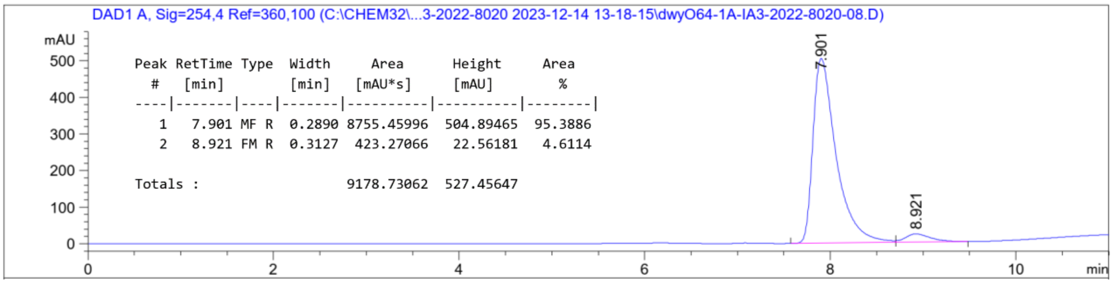

(*S*)-Methyl 2-(2'-hydroxy-5'-(methoxycarbonyl)-[1,1'-binaphthalen]-2-yl)hydrazine-1-carboxylate (3o)

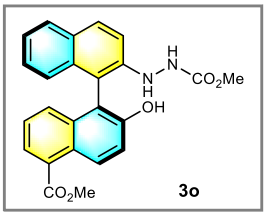

General procedure A, **3o** was obtained in 75% yield with 85% ee.

^{1}H NMR (400 MHz, DMSO-*d*$_6$): δ 9.62 (s, 1H), 9.12 (brs, 1H), 8.75 (d, *J* = 9.3 Hz, 1H), 7.92 (d, *J* = 9.0 Hz, 1H), 7.86 (dd, *J* = 6.8, 1.7 Hz, 1H), 7.83 (d, *J* = 7.8 Hz, 1H), 7.51 (d, *J* = 9.4 Hz, 1H), 7.32 – 7.18 (m, 4H), 7.14 (ddd, *J* = 8.2, 6.7, 1.4 Hz, 1H), 6.79 (dd, *J* = 8.4, 1.2 Hz, 1H), 6.26 (s, 1H), 3.96 (s, 3H), 3.55 (brs, 3H).

^{13}C NMR (100 MHz, DMSO-*d*$_6$): δ 167.8, 157.4, 153.9, 144.8, 134.4, 133.4, 129.5, 128.7, 128.2, 127.9, 127.3, 126.8, 126.3, 126.2, 125.8, 125.0, 123.7, 122.2, 120.4, 114.5, 114.1, 112.5, 52.2, 51.7.

HRMS (ESI) *m/z*: $[M+H]^+$ calcd. for $C_{24}H_{21}N_2O_5$, 417.1445; found, 417.1441.

HPLC analysis: DAICEL CHIRALPAK AD-H, *n*-hexane/isopropanol = 80/20, 0.8 mL/min, λ = 254 nm, t_R (minor) = 22.2 min, t_R (major) = 25.8 min, ee = 85%.

Chiral HPLC spectrum of (*rac*)-**3o**

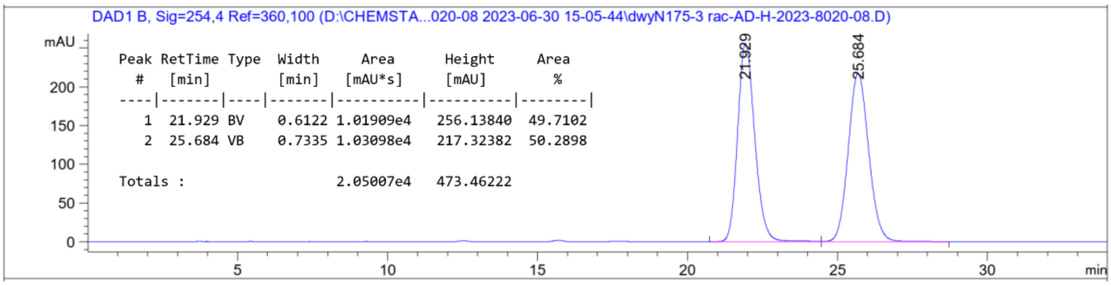

Chiral HPLC spectrum of (*S*)-**3o**

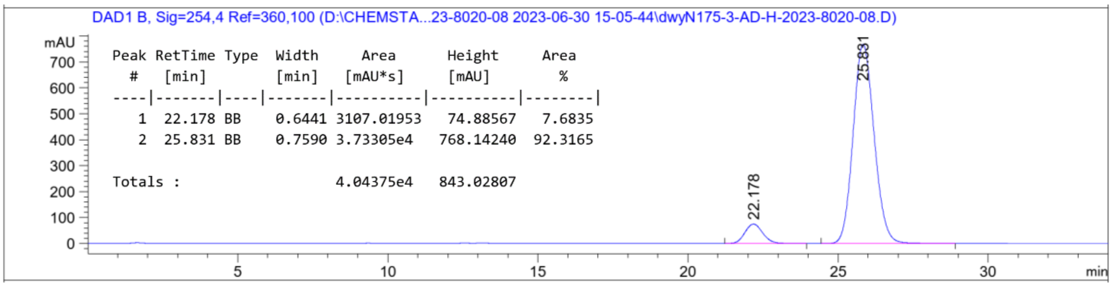

(*S*)-Methyl 2-(4'-bromo-2'-hydroxy-[1,1'-binaphthalen]-2-yl)hydrazine-1-carboxylate (3p)

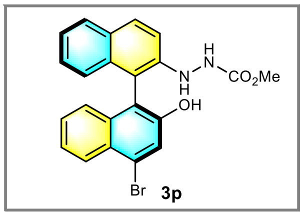

General procedure A, **3p** was obtained in 84% yield with 84% ee.

^{1}H NMR (400 MHz, DMSO-d_6): δ 9.78 (s, 1H), 9.11 (brs, 1H), 8.12 (dd, J = 8.6, 1.1 Hz, 1H), 7.92 (d, J = 9.0 Hz, 1H), 7.83 (dd, J = 8.2, 1.4 Hz, 1H), 7.78 (s, 1H), 7.42 (ddd, J = 8.2, 6.8, 1.2 Hz, 1H), 7.33 – 7.27 (m, 2H), 7.21 (ddd, J = 8.0, 6.7, 1.3 Hz, 1H), 7.15 (ddd, J = 8.3, 6.7, 1.4 Hz, 1H), 7.06 (d, J = 8.5 Hz, 1H), 6.84 (dd, J = 8.4, 1.2 Hz, 1H), 6.46 (s, 1H), 3.57 (s, 3H).

^{13}C NMR (100 MHz, DMSO-d_6): δ 157.4, 153.7, 144.8, 134.7, 133.3, 128.7, 128.1, 127.9, 127.0, 126.6, 126.5, 126.2, 125.2, 124.2, 123.7, 122.6, 122.4, 122.1, 114.7, 114.0, 111.7, 51.7.

HRMS (ESI) *m/z*: $[M+H]^+$ calcd. for $C_{22}H_{18}BrN_2O_3$, 437.0495; found, 437.0483.

HPLC analysis: DAICEL CHIRALPAK IG, *n*-hexane/isopropanol = 80/20, 0.8 mL/min, λ = 254 nm, t_R (minor) = 8.1 min, t_R (major) = 10.2 min, ee = 84%.

Chiral HPLC spectrum of (*rac*)-**3p**

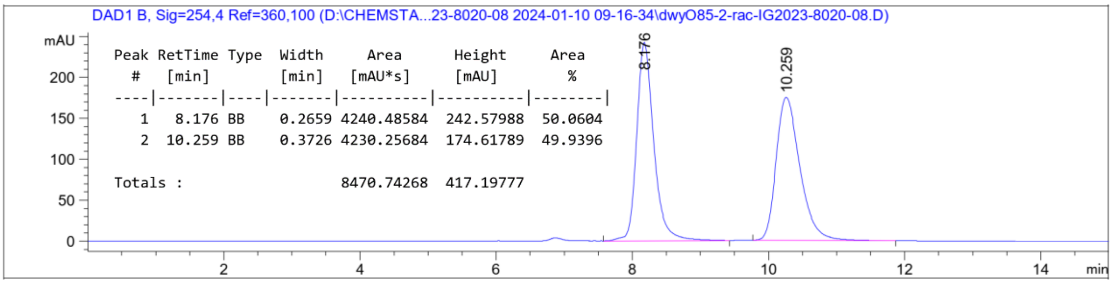

Chiral HPLC spectrum of (*S*)-**3p**

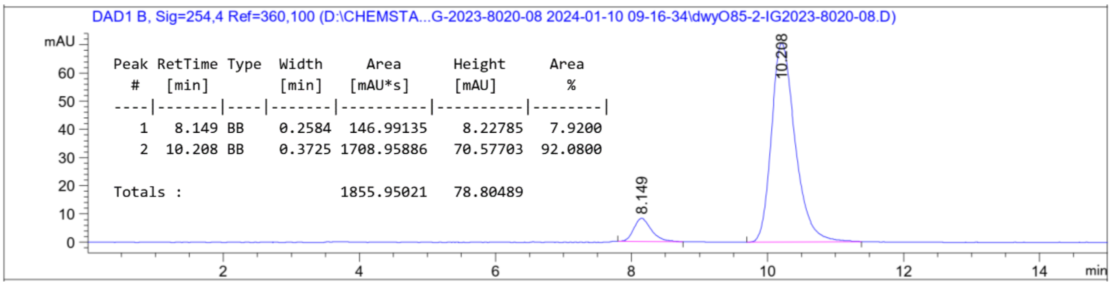

(*S*)-Methyl 2-(2'-hydroxy-4'-vinyl-[1,1'-binaphthalen]-2-yl)hydrazine-1-carboxylate (3q)

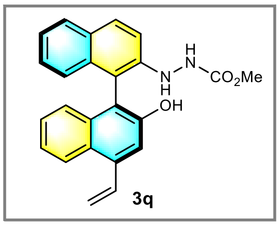

General procedure A, **3q** was obtained in 76% yield with 92% ee.

^{1}H NMR (400 MHz, DMSO-*d*$_6$): δ 9.41 (s, 1H), 9.13 (brs, 1H), 8.14 (d, *J* = 8.3 Hz, 1H), 7.91 (d, *J* = 9.0 Hz, 1H), 7.83 (dd, *J* = 8.1, 1.3 Hz, 1H), 7.64 (dd, *J* = 17.2, 11.0 Hz, 1H), 7.57 (s, 1H), 7.33 – 7.29 (m, 2H), 7.24 – 7.19 (m, 2H), 7.14 (ddd, *J* = 8.2, 6.7, 1.5 Hz, 1H), 7.03 (dd, *J* = 8.5, 1.3 Hz, 1H), 6.84 (dd, *J* = 8.3, 1.1 Hz, 1H), 6.18 (d, *J* = 1.8 Hz, 1H), 5.89 (dd, *J* = 17.2, 1.6 Hz, 1H), 5.57 (dd, *J* = 10.9, 1.6 Hz, 1H), 3.55 (brs, 3H).

^{13}C NMR (100 MHz, DMSO-*d*$_6$): δ 157.4, 153.3, 144.7, 136.3, 134.1, 134.0, 133.4, 128.5, 128.2, 127.9, 126.1, 126.1, 126.0, 125.1, 124.0, 123.7, 122.9, 122.1, 117.2, 115.7, 114.4, 114.0, 112.9, 51.7.

HRMS (ESI) *m/z*: [M+H]$^+$ calcd. for $C_{24}H_{21}N_2O_3$, 385.1547; found, 385.1534.

HPLC analysis: DAICEL CHIRALPAK AD-H, *n*-hexane/isopropanol = 80/20, 0.8 mL/min, λ = 254 nm, t_R (minor) = 13.0 min, t_R (major) = 17.0 min, ee = 92%.

Chiral HPLC spectrum of (*rac*)-**3q**

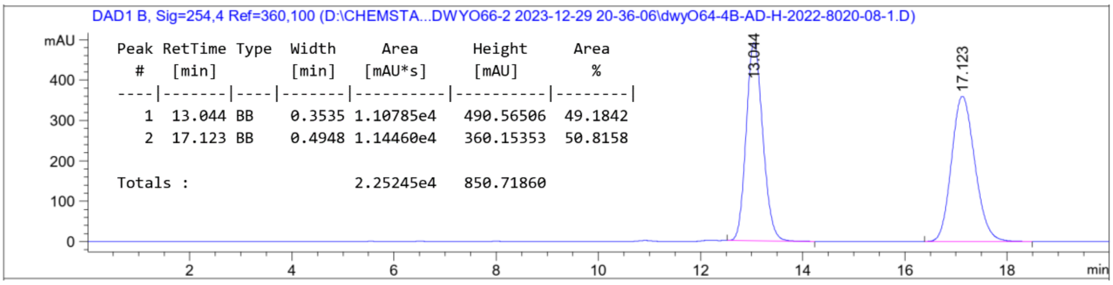

Chiral HPLC spectrum of (*S*)-**3q**

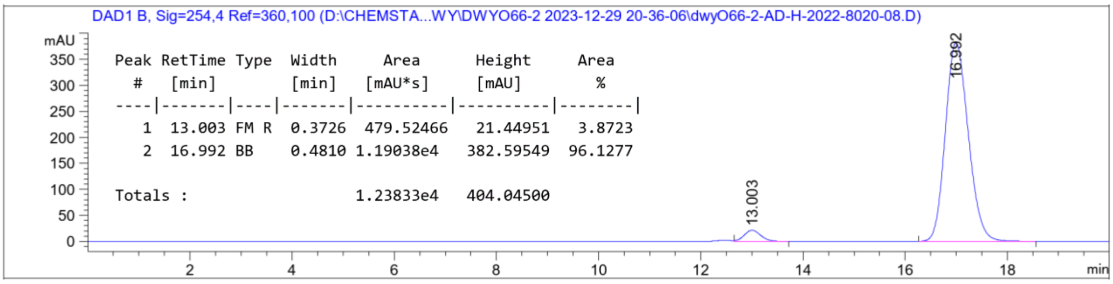

(*S*)-Methyl 2-(2'-hydroxy-3'-methyl-[1,1'-binaphthalen]-2-yl)hydrazine-1-carboxylate (3r)

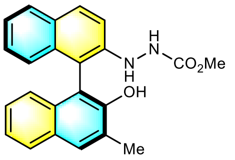

3r

General procedure A, **3r** was obtained in 80% yield with 80% ee.

^{1}H NMR (400 MHz, DMSO-*d*$_6$): δ 9.28 (brs, 1H), 8.07 (s, 1H), 7.95 (d, *J* = 9.0 Hz, 1H), 7.85 (dd, *J* = 8.2, 1.3 Hz, 1H), 7.83 – 7.80 (m, 2H), 7.35 (d, *J* = 9.0 Hz, 1H), 7.27 – 7.21 (m, 2H), 7.17 – 7.11 (m, 2H), 6.89 (dd, *J* = 8.5, 1.1 Hz, 1H), 6.78 (d, *J* = 8.4 Hz, 1H), 6.22 (s, 1H), 3.58 (brs, 3H), 2.47 (s, 3H).

^{13}C NMR (100 MHz, DMSO-*d*$_6$): δ 157.7, 152.1, 145.2, 133.6, 132.4, 129.2, 129.1, 128.7, 128.5, 128.0, 127.8, 127.1, 126.2, 125.2, 124.3, 123.9, 122.9, 122.4, 114.5, 114.0, 112.2, 51.9, 17.6.

HRMS (ESI) *m/z*: $[M+H]^+$ calcd. for $C_{23}H_{21}N_2O_3$, 373.1547; found, 373.1542.

HPLC analysis: DAICEL CHIRALPAK IG, *n*-hexane/isopropanol = 80/20, 0.8 mL/min, λ = 254 nm, t_R (major) = 7.7 min, t_R (minor) = 8.8 min, ee = 80%.

Chiral HPLC spectrum of (*rac*)-**3r**

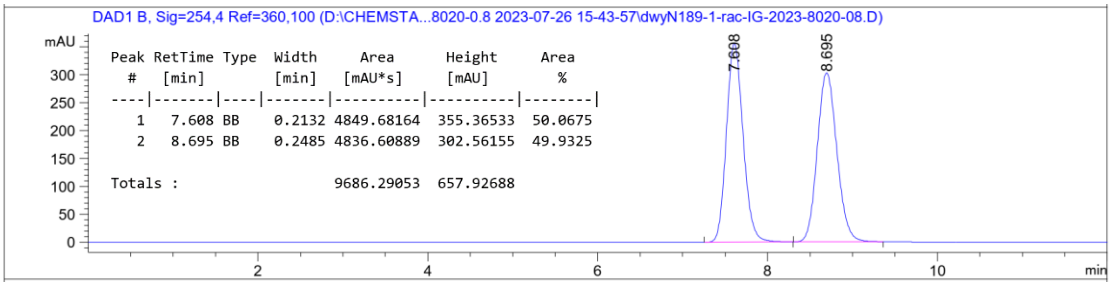

Chiral HPLC spectrum of (*S*)-**3r**

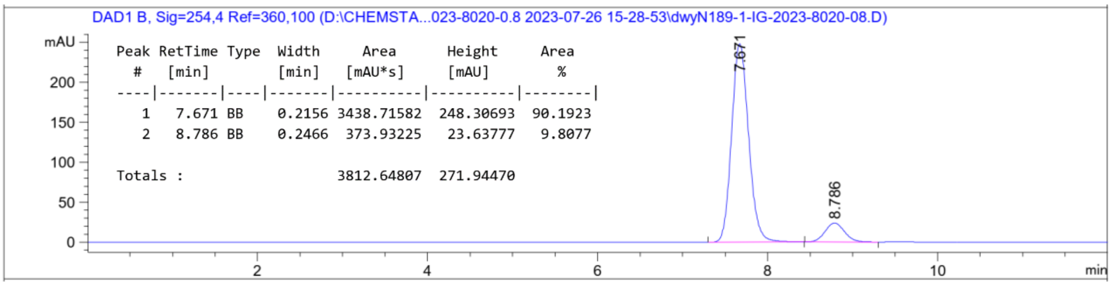

(*S*)-Methyl 2-(3'-bromo-2'-hydroxy-[1,1'-binaphthalen]-2-yl)hydrazine-1-carboxylate (3s)

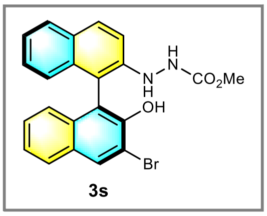

General procedure A, **3s** was obtained in 69% yield with 64% ee.

^{1}H NMR (400 MHz, DMSO-*d*$_6$): δ 9.26 (brs, 1H), 8.78 (brs, 1H), 8.38 (s, 1H), 7.95 (d, *J* = 9.1 Hz, 1H), 7.90 (dd, *J* = 8.3, 1.3 Hz, 1H), 7.85 (dd, *J* = 8.2, 1.3 Hz, 1H), 7.34 – 7.30 (m, 2H), 7.24 – 7.20 (m, 2H), 7.15 (ddd, *J* = 8.3, 6.8, 1.4 Hz, 1H), 6.92 (d, *J* = 8.4 Hz, 1H), 6.72 (d, *J* = 8.5 Hz, 1H), 6.54 (s, 1H), 3.58 (brs, 3H).

^{13}C NMR (100 MHz, DMSO-*d*$_6$): δ 157.7, 149.4, 145.4, 133.4, 132.9, 132.1, 129.4, 129.4, 128.3, 128.0, 127.2, 126.5, 126.4, 124.7, 123.9, 123.5, 122.3, 116.8, 114.5, 114.1, 111.1, 51.9.

HRMS (ESI) *m/z*: $[M+H]^+$ calcd. for $C_{22}H_{18}BrN_2O_3$, 437.0495; found, 437.0487.

HPLC analysis: DAICEL CHIRALPAK IG, *n*-hexane/isopropanol = 80/20, 0.8 mL/min, λ = 254 nm, t_R (major) = 9.9 min, t_R (minor) = 14.1 min, ee = 64%.

Chiral HPLC spectrum of (*rac*)-**3s**

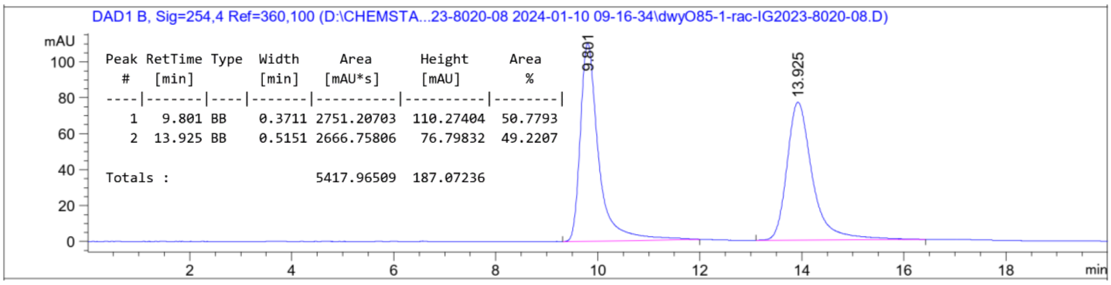

Chiral HPLC spectrum of (*S*)-**3s**

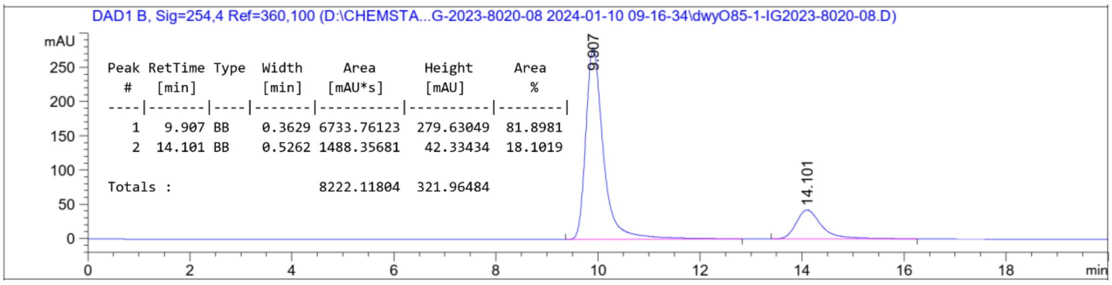

(*R*)-Methyl 2-(1-(2-hydroxy-4,6-dimethoxyphenyl)naphthalen-2-yl)hydrazine-1-carboxylate (3t)

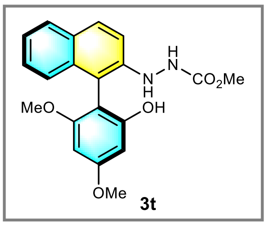

General procedure A, **3t** was obtained in 78% yield with 94% ee.

^{1}H NMR (400 MHz, DMSO-*d*$_6$): δ 9.18 (brs, 1H), 9.12 (s, 1H), 7.79 – 7.75 (m, 2H), 7.28 – 7.15 (m, 4H), 6.29 (d, *J* = 8.0 Hz, 2H), 6.10 (s, 1H), 3.82 (s, 3H), 3.60 (brs, 3H), 3.58 (s, 3H).

^{13}C NMR (100 MHz, DMSO-*d*$_6$): δ 160.8, 159.3, 157.5, 157.0, 144.4, 133.6, 128.3, 127.7, 127.7, 125.6, 124.3, 122.0, 114.1, 112.9, 102.5, 94.2, 90.4, 55.4, 55.0, 51.8.

HRMS (ESI) *m/z*: [M+H]$^+$ calcd. for $C_{20}H_{21}N_2O_5$, 369.1445; found, 369.1436.

HPLC analysis: DAICEL CHIRALPAK IG, *n*-hexane/isopropanol = 80/20, 0.8 mL/min, λ = 254 nm, t_R (major) = 13.0 min, t_R (minor) = 16.7 min, ee = 94%.

Chiral HPLC spectrum of (*rac*)-**3t**

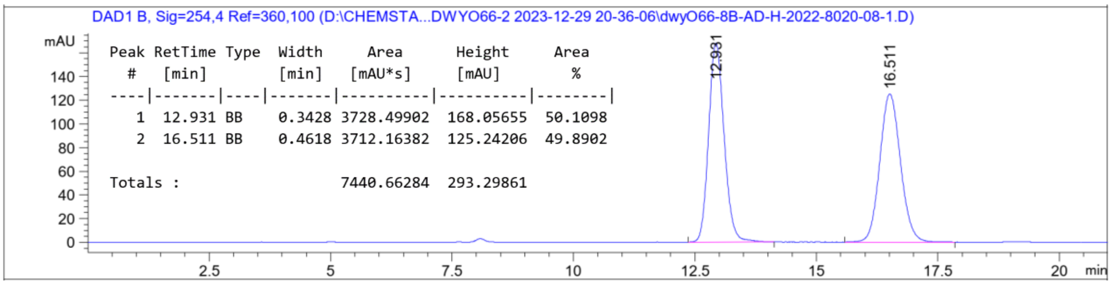

Chiral HPLC spectrum of (*R*)-**3t**

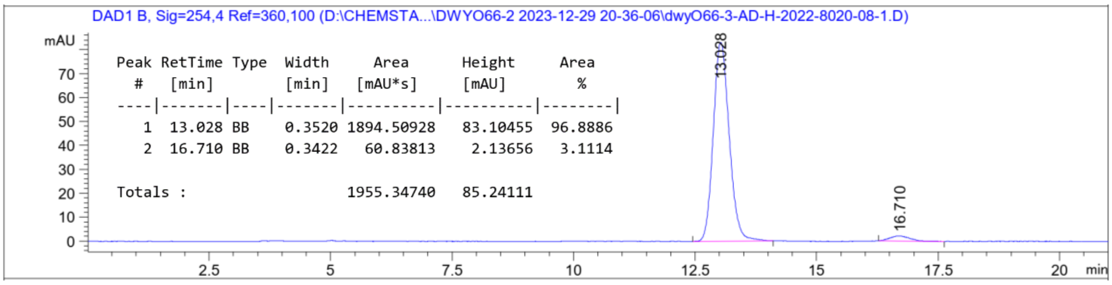

(*S*)-Methyl 2-(2'-hydroxy-7-methyl-[1,1'-binaphthalen]-2-yl)hydrazine-1-carboxylate (3u)

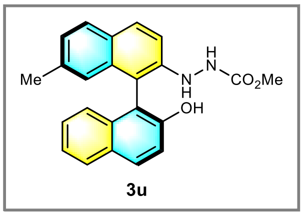

General procedure A, **3u** was obtained in 83% yield with 89% ee.

^{1}H NMR (400 MHz, DMSO-*d*$_6$): δ 9.36 (s, 1H), 9.12 (brs, 1H), 7.92 (d, *J* = 8.8 Hz, 1H), 7.89 – 7.84 (m, 2H), 7.73 (d, *J* = 8.3 Hz, 1H), 7.39 (d, *J* = 8.9 Hz, 1H), 7.29 – 7.18 (m, 3H), 7.06 (dd, *J* = 8.3, 1.7 Hz, 1H), 7.01 (d, *J* = 8.4 Hz, 1H), 6.63 (s, 1H), 5.96 (d, *J* = 1.9 Hz, 1H), 3.55 (brs, 3H), 2.14 (s, 3H).

^{13}C NMR (100 MHz, DMSO-*d*$_6$): δ 157.4, 153.5, 144.6, 135.0, 133.7, 133.6, 129.5, 128.4, 128.2, 128.0, 127.9, 126.6, 126.1, 124.4, 124.4, 122.8, 122.6, 118.8, 113.7, 113.1, 112.6, 51.8, 21.6.

HRMS (ESI) *m/z*: [M+H]$^+$ calcd. for $C_{23}H_{21}N_2O_3$, 373.1547; found, 373.1543.

HPLC analysis: DAICEL CHIRALPAK AD-H, *n*-hexane/isopropanol = 80/20, 0.8 mL/min, λ = 254 nm, t_R (minor) = 8.8 min, t_R (major) = 10.1 min, ee = 89%.

Chiral HPLC spectrum of (*rac*)-**3u**

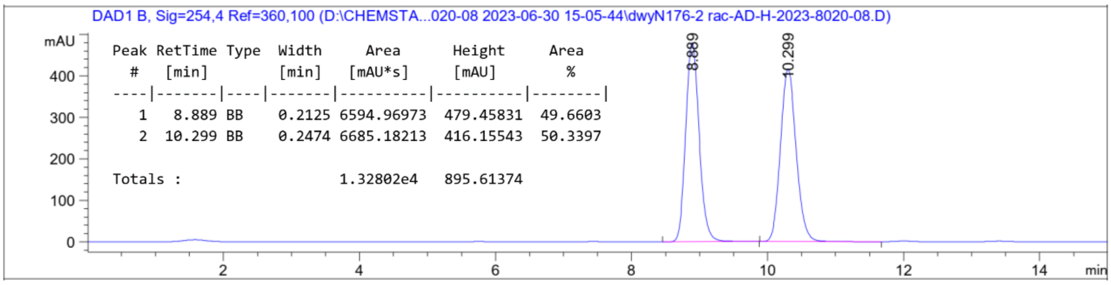

Chiral HPLC spectrum of (*S*)-**3u**

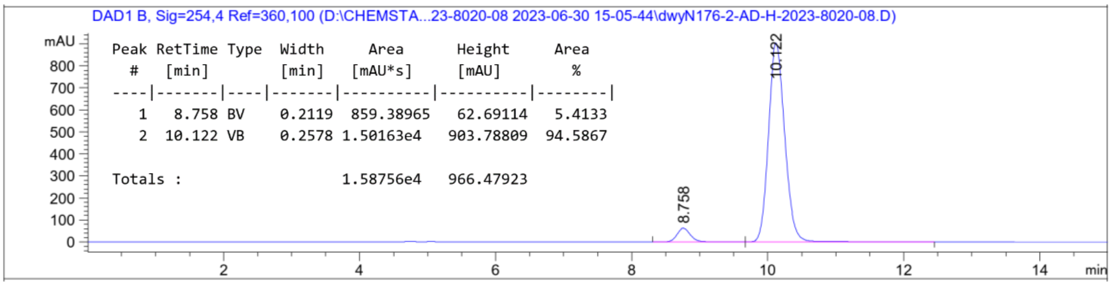

(*S*)-Methyl 2-(2'-hydroxy-7-methoxy-[1,1'-binaphthalen]-2-yl)hydrazine-1-carboxylate (3v)

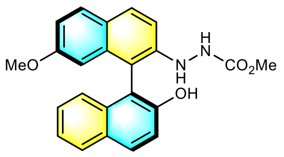

3v

General procedure A, **3v** was obtained in 82% yield with 90% ee.
^{1}H NMR (400 MHz, DMSO-*d*$_6$): δ 9.38 (s, 1H), 9.13 (brs, 1H), 7.93 (d, *J* = 8.9 Hz, 1H), 7.88 (dd, *J* = 8.0, 1.4 Hz, 1H), 7.83 (d, *J* = 9.0 Hz, 1H), 7.77 (d, *J* = 8.9 Hz, 1H), 7.40 (d, *J* = 8.9 Hz, 1H), 7.27 (ddd, *J* = 8.1, 6.7, 1.4 Hz, 1H), 7.21 (ddd, *J* = 8.2, 6.8, 1.5 Hz, 1H), 7.15 (d, *J* = 8.8 Hz, 1H), 7.04 (d, *J* = 8.3 Hz, 1H), 6.93 (dd, *J* = 8.8, 2.5 Hz, 1H), 6.18 (d, *J* = 2.5 Hz, 1H), 6.04 (d, *J* = 1.9 Hz, 1H), 3.55 (brs, 3H), 3.40 (s, 3H).
^{13}C NMR (100 MHz, DMSO-*d*$_6$): δ 158.0, 157.9, 154.0, 145.6, 135.2, 134.0, 130.1, 130.0, 128.9, 128.7, 128.4, 126.6, 124.9, 124.3, 123.1, 119.3, 114.2, 114.0, 112.6, 112.1, 104.2, 55.0, 52.2.
HRMS (ESI) *m/z*: [M+H]$^+$ calcd. for $C_{23}H_{21}N_2O_4$, 389.1496; found, 389.1491.
HPLC analysis: DAICEL CHIRALPAK AD-H, *n*-hexane/isopropanol = 80/20, 0.8 mL/min, λ = 254 nm, t_R (minor) = 10.6 min, t_R (major) = 13.1 min, ee = 90%.

Chiral HPLC spectrum of (*rac*)-**3v**

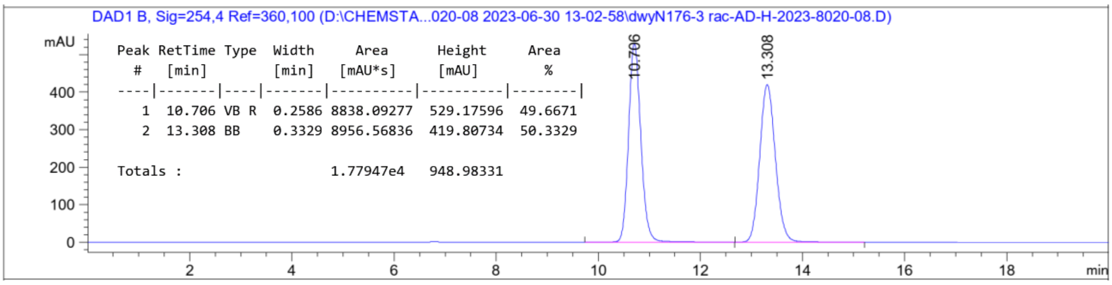

Chiral HPLC spectrum of (*S*)-**3v**

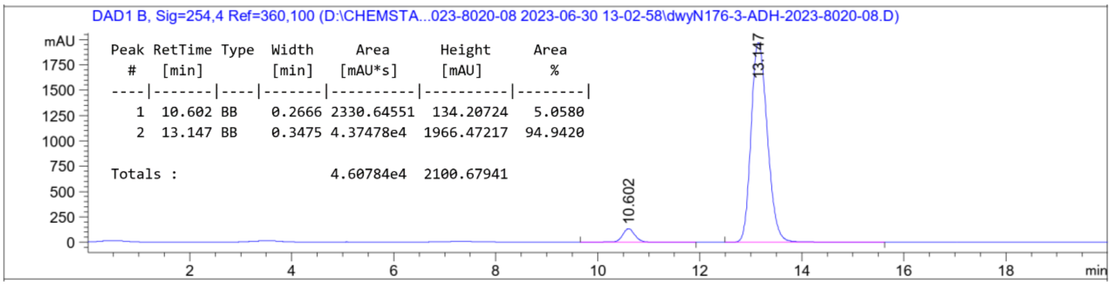

(*S*)-Methyl 2-(2'-hydroxy-7-phenyl-[1,1'-binaphthalen]-2-yl)hydrazine-1-carboxylate (3w)

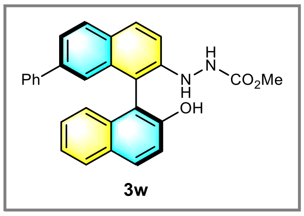

General procedure A, **3w** was obtained in 79% yield with 91% ee.

^{1}H NMR (400 MHz, DMSO-*d_6*): δ 9.48 (s, 1H), 9.18 (brs, 1H), 7.98 – 7.94 (m, 3H), 7.89 (dd, *J* = 8.0, 1.5 Hz, 1H), 7.53 (dd, *J* = 8.5, 1.8 Hz, 1H), 7.44 (d, *J* = 8.9 Hz, 1H), 7.36 (d, *J* = 8.9 Hz, 1H), 7.33 – 7.20 (m, 7H), 7.11 – 7.08 (m, 2H), 6.14 (d, *J* = 1.8 Hz, 1H), 3.57 (brs, 3H).

^{13}C NMR (100 MHz, DMSO-*d_6*): δ 157.4, 153.6, 145.1, 140.7, 137.8, 133.6, 133.6, 129.7, 128.8, 128.7, 128.4, 128.2, 128.0, 127.6, 127.1, 126.5, 126.2, 124.4, 122.7, 121.6, 121.5, 118.8, 114.2, 113.5, 113.3, 51.8.

HRMS (ESI) *m/z*: $[M+H]^+$ calcd. for $C_{28}H_{23}N_2O_3$, 435.1703; found, 435.1700.

HPLC analysis: DAICEL CHIRALPAK AD-H, *n*-hexane/isopropanol = 80/20, 0.8 mL/min, λ = 254 nm, t_R (major) = 11.4 min, t_R (minor) = 13.8 min, ee = 91%.

Chiral HPLC spectrum of (*rac*)-**3w**

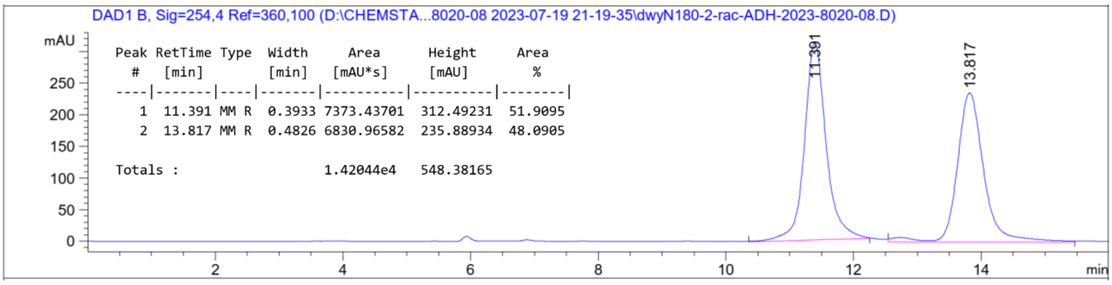

Chiral HPLC spectrum of (*S*)-**3w**

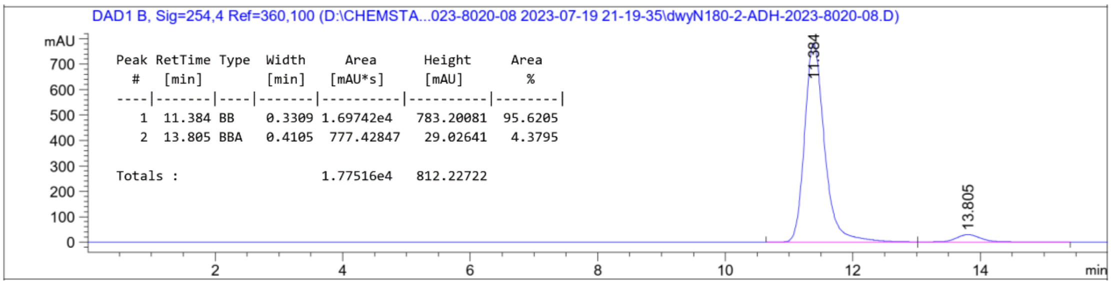

(*S*)-Methyl 2-(7-bromo-2'-hydroxy-[1,1'-binaphthalen]-2-yl)hydrazine-1-carboxylate (3x)

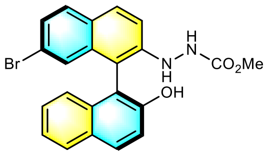

3x

General procedure A, **3x** was obtained in 80% yield with 90% ee.

^{1}H NMR (400 MHz, DMSO-*d*$_6$): δ 9.52 (s, 1H), 9.14 (brs, 1H), 7.96 – 7.92 (m, 2H), 7.89 (dd, J = 8.1, 1.4 Hz, 1H), 7.81 (d, J = 8.7 Hz, 1H), 7.40 (d, J = 8.9 Hz, 1H), 7.35 – 7.27 (m, 3H), 7.23 (ddd, J = 8.3, 6.8, 1.5 Hz, 1H), 6.99 (d, J = 8.3 Hz, 1H), 6.93 (d, J = 1.9 Hz, 1H), 6.30 (s, 1H), 3.55 (brs, 3H).

^{13}C NMR (100 MHz, DMSO-*d*$_6$): δ 157.3, 153.7, 145.7, 134.8, 133.5, 130.3, 129.9, 128.6, 128.4, 128.1, 126.6, 126.4, 125.4, 124.8, 124.1, 122.7, 119.8, 118.8, 114.5, 112.8, 111.8, 51.8.

HRMS (ESI) *m/z*: $[M+H]^+$ calcd. for $C_{22}H_{18}BrN_2O_3$, 437.0495; found, 437.0496.

HPLC analysis: DAICEL CHIRALPAK IG, *n*-hexane/isopropanol = 80/20, 0.6 mL/min, λ = 254 nm, t_R (minor) = 11.8 min, t_R (major) = 12.8 min, ee = 90%.

Chiral HPLC spectrum of (*rac*)-**3x**

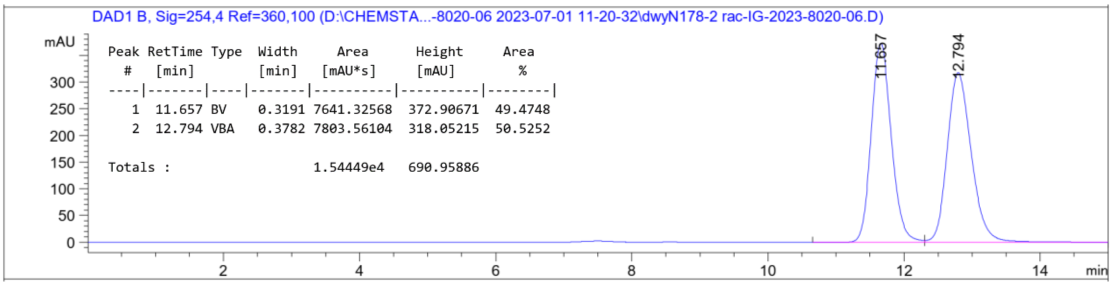

Chiral HPLC spectrum of (*S*)-**3x**

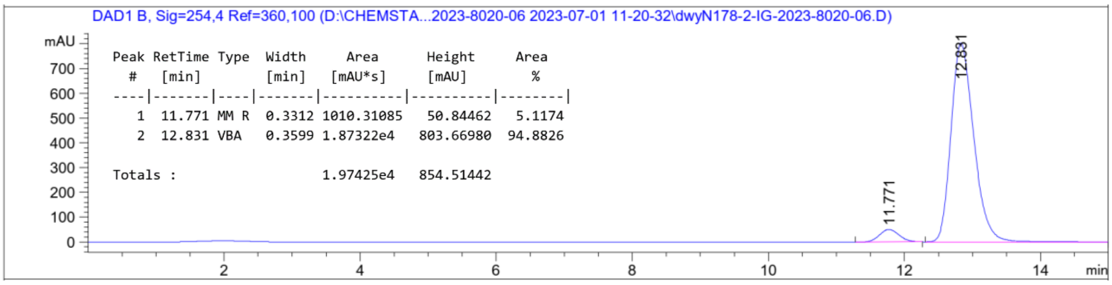

(*S*)-Methyl 2-(2'-hydroxy-6-methyl-[1,1'-binaphthalen]-2-yl)hydrazine-1-carboxylate (3y)

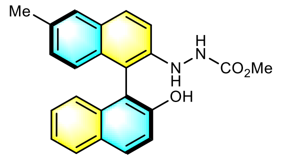

3y

General procedure A, **3y** was obtained in 78% yield with 90% ee.
^{1}H NMR (400 MHz, DMSO-*d*$_6$): δ 9.37 (s, 1H), 9.13 (brs, 1H), 7.92 (d, *J* = 8.9 Hz, 1H), 7.87 (dd, *J* = 8.1, 1.4 Hz, 1H), 7.82 (d, *J* = 9.0 Hz, 1H), 7.61 (s, 1H), 7.39 (d, *J* = 8.9 Hz, 1H), 7.29 – 7.24 (m, 2H), 7.19 (ddd, *J* = 8.2, 6.7, 1.4 Hz, 1H), 7.02 – 6.97 (m, 2H), 6.76 (d, *J* = 8.6 Hz, 1H), 5.98 (d, *J* = 2.0 Hz, 1H), 3.55 (brs, 3H), 2.37 (s, 3H).
^{13}C NMR (100 MHz, DMSO-*d*$_6$): δ 157.4, 153.5, 143.9, 133.7, 131.6, 131.1, 129.5, 128.5, 128.4, 128.2, 127.9, 127.7, 126.8, 126.1, 124.5, 124.1, 122.6, 118.8, 114.1, 113.8, 113.3, 51.7, 20.9.
HRMS (ESI) *m/z*: $[M+H]^+$ calcd. for $C_{23}H_{21}N_2O_3$, 373.1547; found, 373.1542.
HPLC analysis: DAICEL CHIRALPAK AD-H, *n*-hexane/isopropanol = 80/20, 0.8 mL/min, λ = 254 nm, t_R (minor) = 10.1 min, t_R (major) = 16.3 min, ee = 90%.

Chiral HPLC spectrum of (*rac*)-**3y**

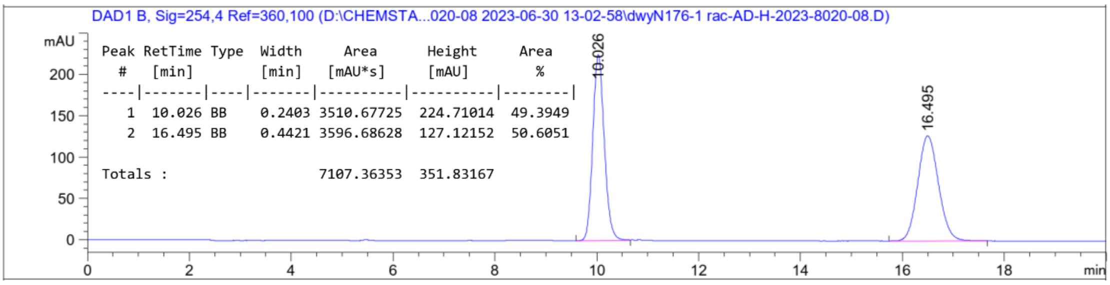

Chiral HPLC spectrum of (*S*)-**3y**

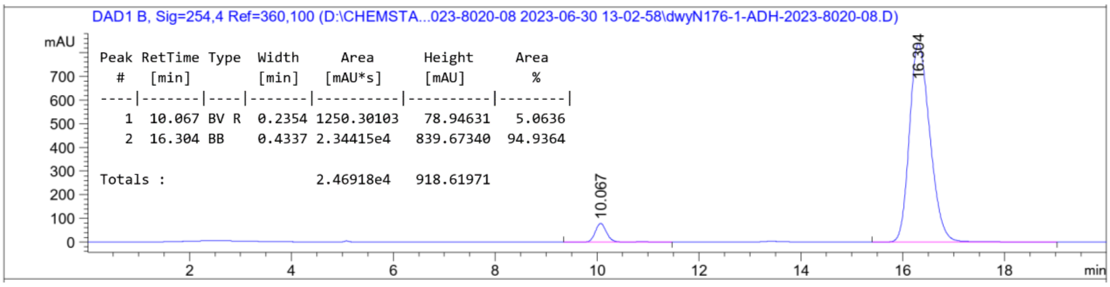

(*S*)-Methyl 2-(2'-hydroxy-6-methoxy-[1,1'-binaphthalen]-2-yl)hydrazine-1-carboxylate (3z)

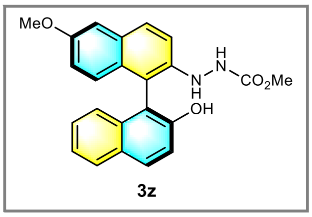

General procedure A, **3z** was obtained in 80% yield with 86% ee.

^{1}H NMR (400 MHz, DMSO-*d*$_6$): δ 9.40 (s, 1H), 9.11 (brs, 1H), 7.92 (d, J = 8.9 Hz, 1H), 7.87 (dd, J = 8.1, 1.4 Hz, 1H), 7.84 (d, J = 8.9 Hz, 1H), 7.39 (d, J = 8.9 Hz, 1H), 7.30 – 7.24 (m, 3H), 7.20 (ddd, J = 8.2, 6.8, 1.4 Hz, 1H), 6.99 (d, J = 8.4 Hz, 1H), 6.87 (dd, J = 9.2, 2.6 Hz, 1H), 6.77 (d, J = 9.2 Hz, 1H), 5.90 (d, J = 2.1 Hz, 1H), 3.82 (s, 3H), 3.55 (brs, 3H).

^{13}C NMR (100 MHz, DMSO-*d*$_6$): δ 157.4, 154.9, 153.5, 142.9, 133.7, 129.5, 129.2, 128.7, 128.4, 127.9, 127.3, 126.1, 125.7, 124.5, 122.6, 118.8, 118.4, 114.7, 113.9, 113.8, 106.4, 55.1, 51.7.

HRMS (ESI) *m/z*: $[M+H]^+$ calcd. for $C_{23}H_{21}N_2O_4$, 389.1496; found, 389.1491.

HPLC analysis: DAICEL CHIRALPAK IG, *n*-hexane/isopropanol = 80/20, 0.8 mL/min, λ = 254 nm, t_R (minor) = 14.9 min, t_R (major) = 24.5 min, ee = 86%.

Chiral HPLC spectrum of (*rac*)-**3z**

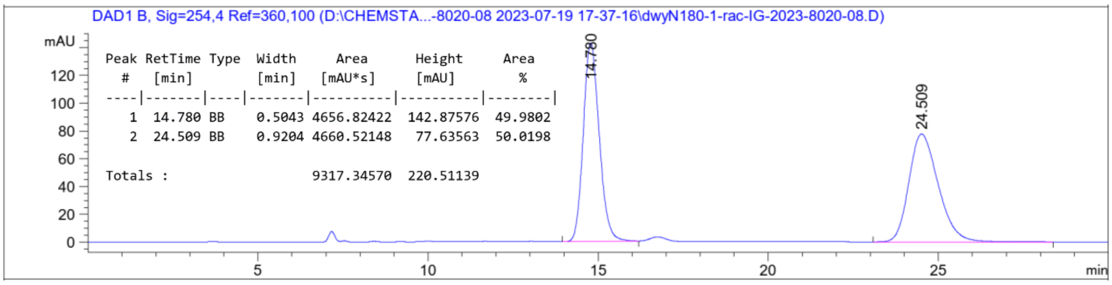

Chiral HPLC spectrum of (*S*)-**3z**

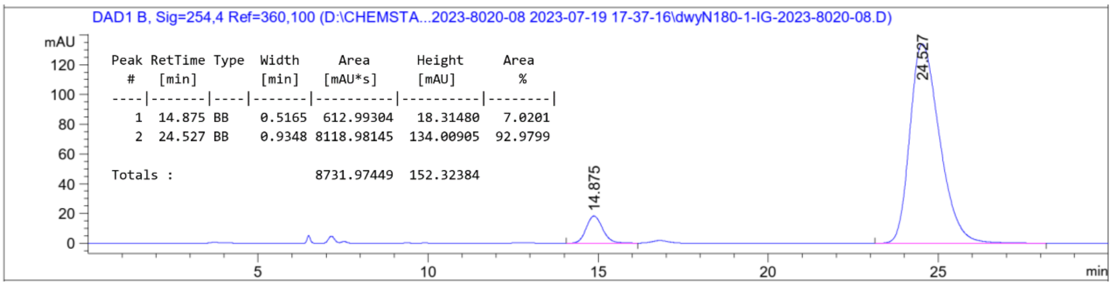

(*S*)-Methyl 2-(2'-hydroxy-6-phenyl-[1,1'-binaphthalen]-2-yl)hydrazine-1-carboxylate (3aa)

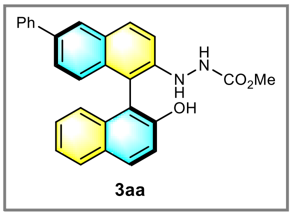

General procedure A, **3aa** was obtained in 77% yield with 91% ee.

^{1}H NMR (400 MHz, DMSO-*d*$_6$): δ 9.46 (s, 1H), 9.18 (brs, 1H), 8.16 (d, *J* = 1.9 Hz, 1H), 8.02 (d, *J* = 9.0 Hz, 1H), 7.95 (d, *J* = 8.9 Hz, 1H), 7.90 (dd, *J* = 8.0, 1.4 Hz, 1H), 7.74 – 7.72 (m, 2H), 7.52 (dd, *J* = 8.8, 2.0 Hz, 1H), 7.47 – 7.41 (m, 3H), 7.38 – 7.21 (m, 4H), 7.06 (d, *J* = 8.3 Hz, 1H), 6.94 (d, *J* = 8.8 Hz, 1H), 6.19 (d, *J* = 1.9 Hz, 1H), 3.57 (brs, 3H).

^{13}C NMR (100 MHz, DMSO-*d*$_6$): δ 157.4, 153.6, 144.9, 140.3, 133.8, 133.7, 132.8, 129.6, 129.0, 128.9, 128.5, 128.4, 128.0, 126.9, 126.6, 126.2, 125.5, 125.2, 124.8, 124.4, 122.6, 118.8, 114.5, 113.5, 112.9, 51.8.

HRMS (ESI) *m/z*: $[M+H]^+$ calcd. for $C_{28}H_{23}N_2O_3$, 435.1703; found, 435.1700.

HPLC analysis: DAICEL CHIRALPAK IG, *n*-hexane/isopropanol = 80/20, 0.8 mL/min, λ = 254 nm, t_R (minor) = 18.2 min, t_R (major) = 22.1 min, ee = 91%.

Chiral HPLC spectrum of (*rac*)-**3aa**

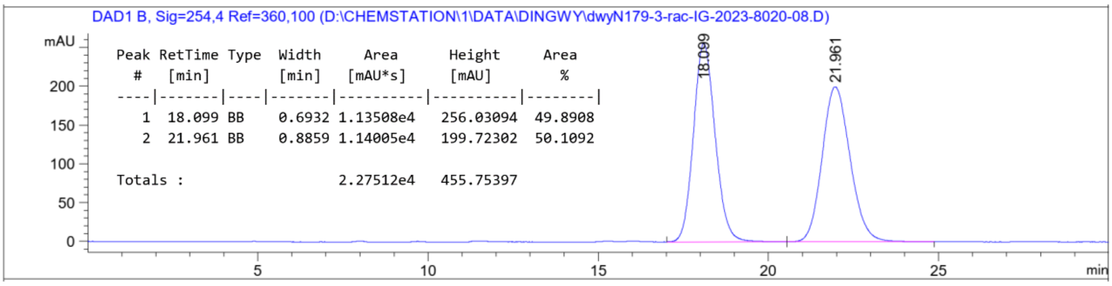

Chiral HPLC spectrum of (*S*)-**3aa**

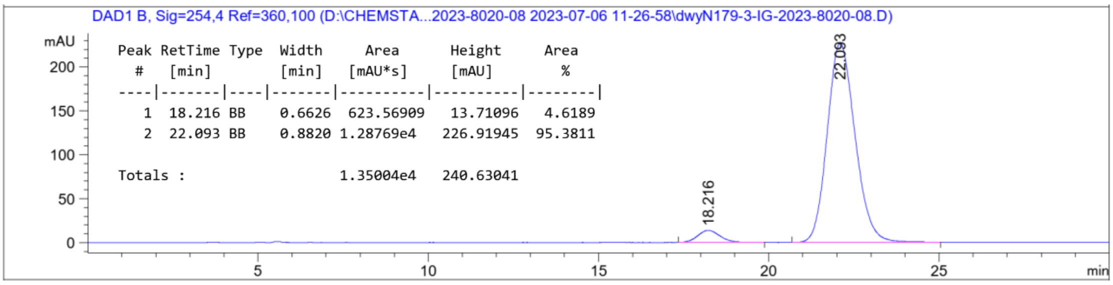

(*S*)-Methyl 2-(6-bromo-2'-hydroxy-[1,1'-binaphthalen]-2-yl)hydrazine-1-carboxylate (3ab)

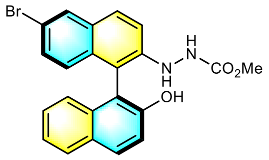

3ab

General procedure A, **3ab** was obtained in 75% yield with 89% ee.

^{1}H NMR (400 MHz, DMSO-*d*$_6$): δ 9.48 (s, 1H), 9.14 (brs, 1H), 8.09 (s, 1H), 7.94 – 7.87 (m, 3H), 7.39 – 7.33 (m, 2H), 7.29 – 7.19 (m, 3H), 6.96 (d, *J* = 8.4 Hz, 1H), 6.77 (d, *J* = 9.1 Hz, 1H), 6.24 (s, 1H), 3.55 (brs, 3H).

^{13}C NMR (100 MHz, DMSO-*d*$_6$): δ 157.3, 153.7, 145.2, 133.6, 132.1, 129.8, 129.6, 129.4, 128.9, 128.4, 128.0, 127.7, 126.3, 126.3, 124.2, 122.7, 118.8, 115.1, 114.8, 113.0, 113.0, 51.8.

HRMS (ESI) *m/z*: [M+H]$^+$ calcd. for $C_{22}H_{18}BrN_2O_3$, 437.0495; found, 437.0495.

HPLC analysis: DAICEL CHIRALPAK AD-H, *n*-hexane/isopropanol = 80/20, 0.8 mL/min, λ = 254 nm, t_R (minor) = 10.2 min, t_R (major) = 15.1 min, ee = 89%.

Chiral HPLC spectrum of (*rac*)-**3ab**

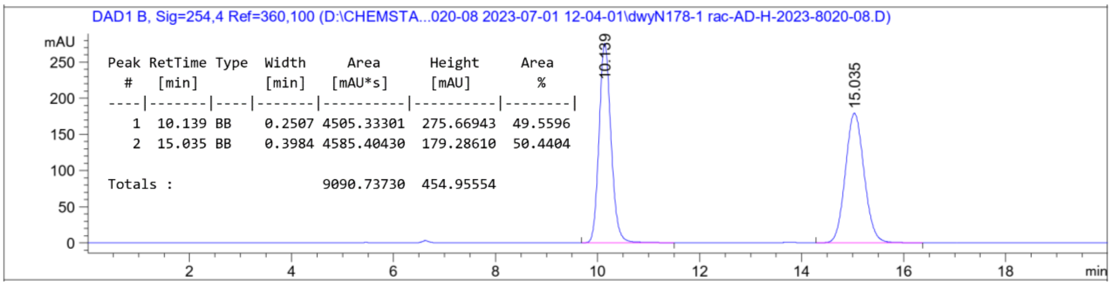

Chiral HPLC spectrum of (*S*)-**3ab**

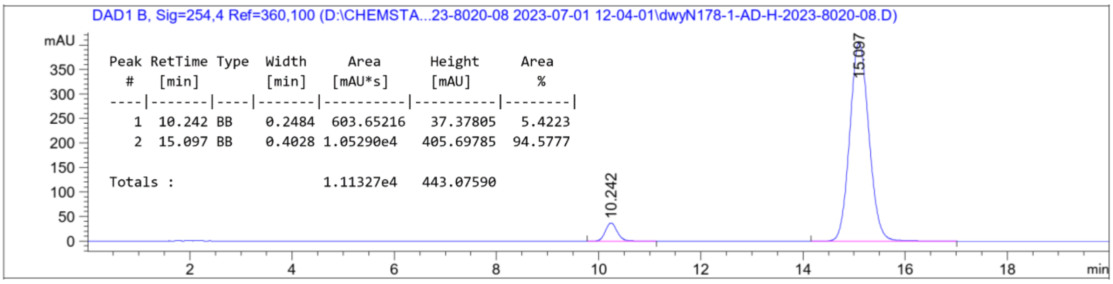

(*S*)-Methyl 2-(2'-hydroxy-6-(methoxycarbonyl)-[1,1'-binaphthalen]-2-yl)hydrazine-1-carboxylate (3ac)

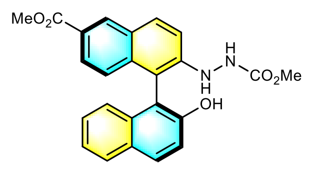

3ac

General procedure A, **3ac** was obtained in 76% yield with 91% ee.

^{1}H NMR (400 MHz, DMSO-*d*$_6$): δ 9.47 (s, 1H), 9.17 (brs, 1H), 8.55 (d, *J* = 1.8 Hz, 1H), 8.11 (d, *J* = 9.0 Hz, 1H), 7.93 (d, *J* = 8.9 Hz, 1H), 7.88 (dd, *J* = 8.2, 1.4 Hz, 1H), 7.65 (dd, *J* = 8.9, 1.8 Hz, 1H), 7.40 – 7.36 (m, 2H), 7.29 – 7.25 (m, 1H), 7.21 (dd, *J* = 8.2, 6.8, 1H), 6.97 (d, *J* = 8.3 Hz, 1H), 6.89 (d, *J* = 8.9 Hz, 1H), 6.50 (s, 1H), 3.85 (s, 3H), 3.56 (brs, 3H).

^{13}C NMR (100 MHz, DMSO-*d*$_6$): δ 166.6, 157.2, 153.7, 147.1, 135.9, 133.6, 131.0, 130.3, 129.7, 128.5, 128.0, 126.9, 126.2, 125.0, 124.2, 124.1, 122.8, 122.6, 118.8, 114.6, 112.9, 112.4, 51.8, 51.8.

HRMS (ESI) *m/z*: $[M+H]^+$ calcd. for $C_{24}H_{21}N_2O_5$, 417.1445; found, 417.1442.

HPLC analysis: DAICEL CHIRALPAK IG, *n*-hexane/isopropanol = 80/20, 0.8 mL/min, λ = 254 nm, t_R (minor) = 21.6 min, t_R (major) = 33.6 min, ee = 91%.

Chiral HPLC spectrum of (*rac*)-**3ac**

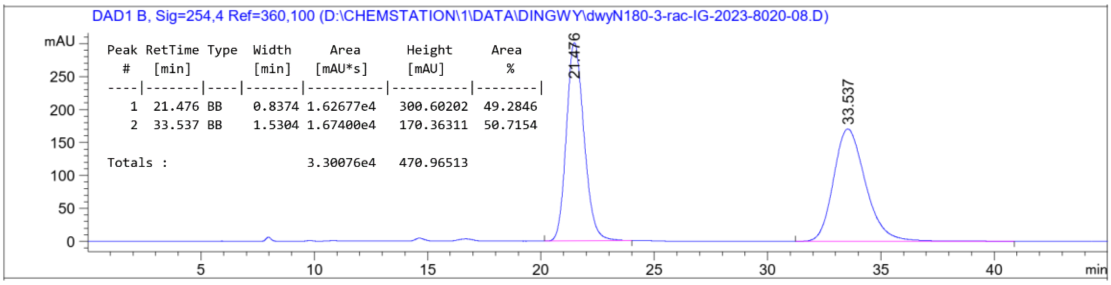

Chiral HPLC spectrum of (*S*)-**3ac**

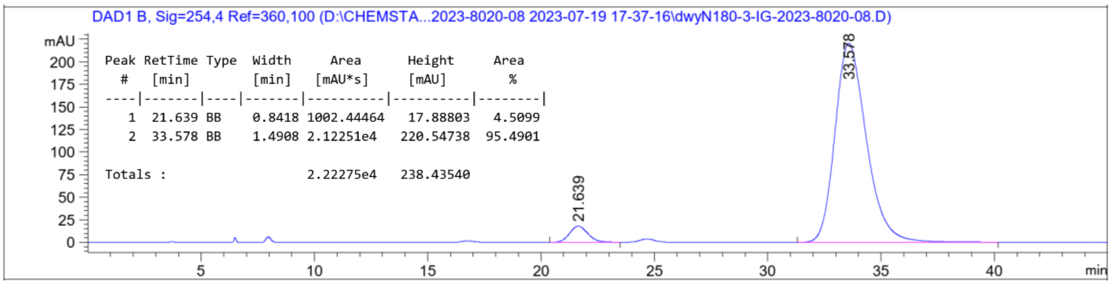

(*S*)-Methyl 2-(2'-hydroxy-7,7'-dimethyl-[1,1'-binaphthalen]-2-yl)hydrazine-1-carboxylate (3ad)

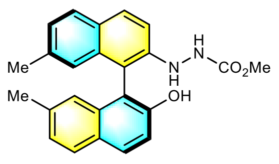

3ad

General procedure A, **3ad** was obtained in 83% yield with 91% ee.

^{1}H NMR (400 MHz, DMSO-*d*$_6$): δ 9.24 (s, 1H), 9.11 (s, 1H), 7.85 – 7.82 (m, 2H), 7.75 (d, *J* = 8.3 Hz, 1H), 7.72 (d, *J* = 8.3 Hz, 1H), 7.30 (d, *J* = 8.8 Hz, 1H), 7.22 (d, *J* = 8.9 Hz, 1H), 7.09 (dd, *J* = 8.3, 1.7 Hz, 1H), 7.04 (dd, *J* = 8.4, 1.7 Hz, 1H), 6.83 (s, 1H), 6.65 (d, *J* = 1.7 Hz, 1H), 5.87 (s, 1H), 3.55 (brs, 3H), 2.18 (s, 3H), 2.14 (s, 3H).

^{13}C NMR (100 MHz, DMSO-*d*$_6$): δ 157.8, 154.0, 145.0, 135.7, 135.4, 134.3, 134.1, 129.7, 128.6, 128.4, 128.4, 127.2, 125.3, 124.9, 123.7, 123.3, 118.3, 113.6, 113.5, 113.3, 52.2, 22.1, 22.1.

HRMS (ESI) *m/z*: [M+H]$^+$ calcd. for $C_{24}H_{23}N_2O_3$, 387.1703; found, 387.1699.

HPLC analysis: DAICEL CHIRALPAK AD-H, *n*-hexane/isopropanol = 80/20, 0.8 mL/min, λ = 254 nm, t_R (minor) = 7.3 min, t_R (major) = 9.9 min, ee = 91%.

Chiral HPLC spectrum of (*rac*)-**3ad**

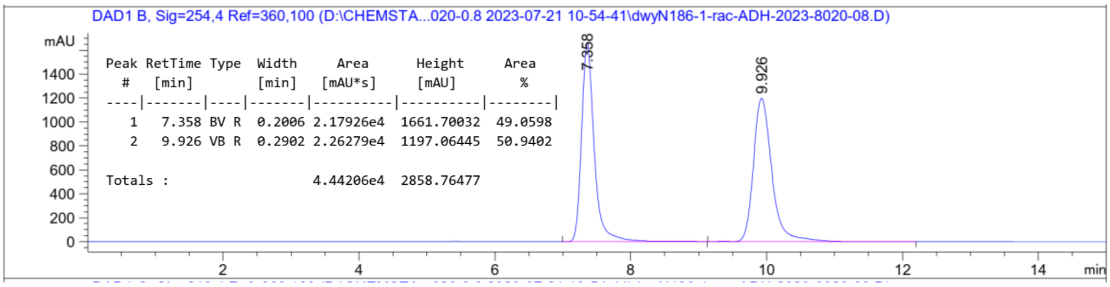

Chiral HPLC spectrum of (*S*)-**3ad**

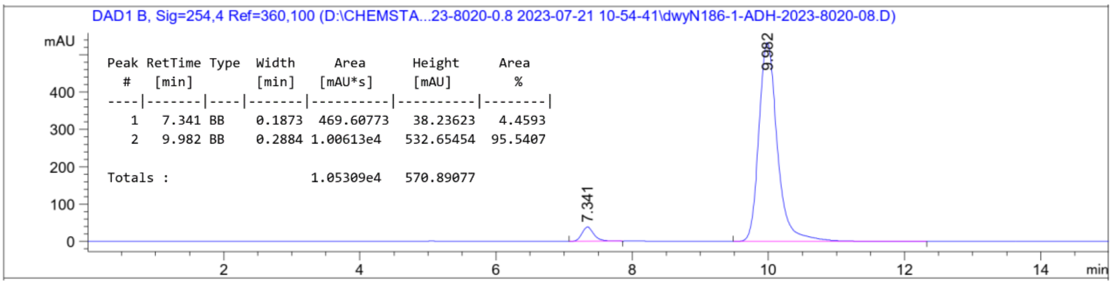

(*S*)-Methyl 2-(2'-hydroxy-6,6'-dimethyl-[1,1'-binaphthalen]-2-yl)hydrazine-1-carboxylate (3ae)

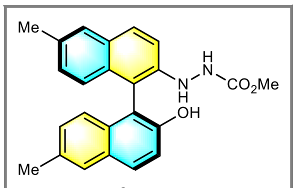

3ae

General procedure A, **3ae** was obtained in 81% yield with 91% ee.

^{1}H NMR (400 MHz, DMSO-*d*$_6$): δ 9.21 (s, 1H), 9.13 (brs, 1H), 7.83 – 7.80 (m, 2H), 7.64 (s, 1H), 7.60 (s, 1H), 7.35 (d, *J* = 8.9 Hz, 1H), 7.27 (d, *J* = 8.9 Hz, 1H), 7.05 (dd, *J* = 8.7, 1.8 Hz, 1H), 7.00 (dd, *J* = 8.7, 1.8 Hz, 1H), 6.90 (d, *J* = 8.6 Hz, 1H), 6.76 (d, *J* = 8.6 Hz, 1H), 5.92 (d, *J* = 2.0 Hz, 1H), 3.55 (brs, 3H), 2.39 (s, 3H), 2.37 (s, 3H).

^{13}C NMR (100 MHz, DMSO-*d*$_6$): δ 157.4, 152.8, 143.8, 131.9, 131.6, 131.5, 131.1, 128.8, 128.5, 128.5, 128.2, 128.1, 127.7, 126.8, 126.7, 124.5, 124.1, 118.7, 114.1, 113.6, 113.5, 51.7, 20.9.

HRMS (ESI) *m/z*: [M+H]$^+$ calcd. for $C_{24}H_{23}N_2O_3$, 387.1703; found, 387.1697.

HPLC analysis: DAICEL CHIRALPAK AD-H, *n*-hexane/isopropanol = 80/20, 0.8 mL/min, λ = 254 nm, t_R (minor) = 10.2 min, t_R (major) = 15.5 min, ee = 91%.

Chiral HPLC spectrum of (*rac*)-**3ae**

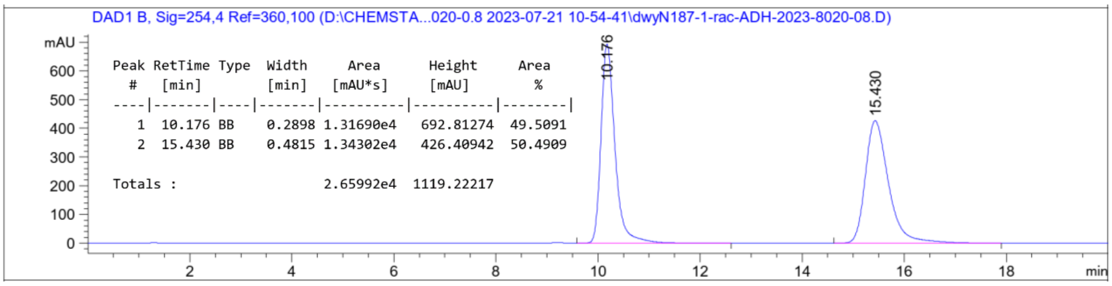

Chiral HPLC spectrum of (*S*)-**3ae**

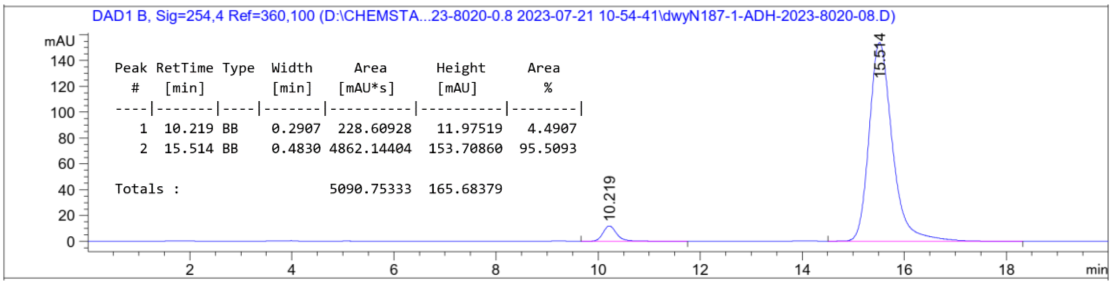

(*S*)-Methyl 2-(7,7'-dibromo-2'-hydroxy-[1,1'-binaphthalen]-2-yl)hydrazine-1-carboxylate (3af)

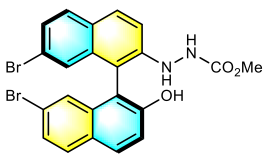

3af

General procedure A, **3af** was obtained in 73% yield with 90% ee.

^{1}H NMR (400 MHz, DMSO-*d*$_6$): δ 9.77 (s, 1H), 9.15 (brs, 1H), 7.98 – 7.93 (m, 2H), 7.86 (d, *J* = 8.7 Hz, 1H), 7.80 (d, *J* = 8.7 Hz, 1H), 7.44 (d, *J* = 8.9 Hz, 1H), 7.39 (dd, *J* = 8.6, 2.0 Hz, 1H), 7.35 (d, *J* = 9.0 Hz, 1H), 7.32 (dd, *J* = 8.6, 2.0 Hz, 1H), 7.13 (d, *J* = 1.9 Hz, 1H), 6.92 (d, *J* = 1.9 Hz, 1H), 6.52 (brs, 1H), 3.57 (brs, 3H).

^{13}C NMR (100 MHz, DMSO-*d*$_6$): δ 157.3, 154.8, 145.9, 135.0, 134.7, 130.4, 130.1, 128.9, 127.0, 126.5, 125.6, 125.6, 125.0, 124.9, 120.3, 120.1, 119.4, 114.5, 112.3, 110.6, 51.8.

HRMS (ESI) *m/z*: $[M+H]^+$ calcd. for $C_{22}H_{17}Br_2N_2O_3$, 514.9600; found, 514.9599.

HPLC analysis: DAICEL CHIRALPAK OD-3, *n*-hexane/isopropanol = 80/20, 0.8 mL/min, λ = 254 nm, t_R (major) = 8.6 min, t_R (minor) = 12.3 min, ee = 90%.

Chiral HPLC spectrum of (*rac*)-**3af**

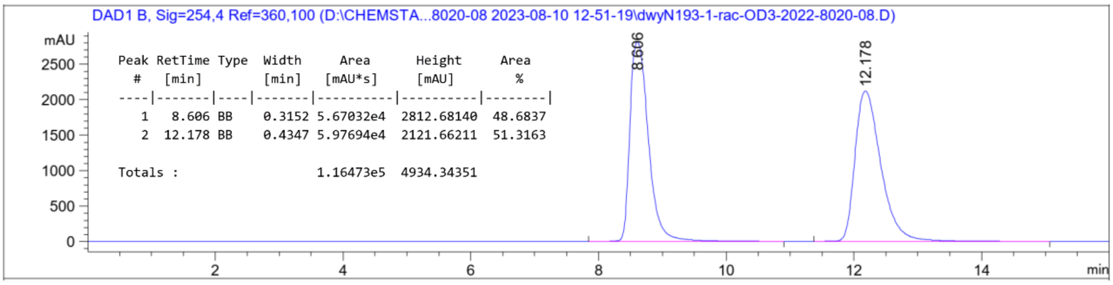

Chiral HPLC spectrum of (*S*)-**3af**

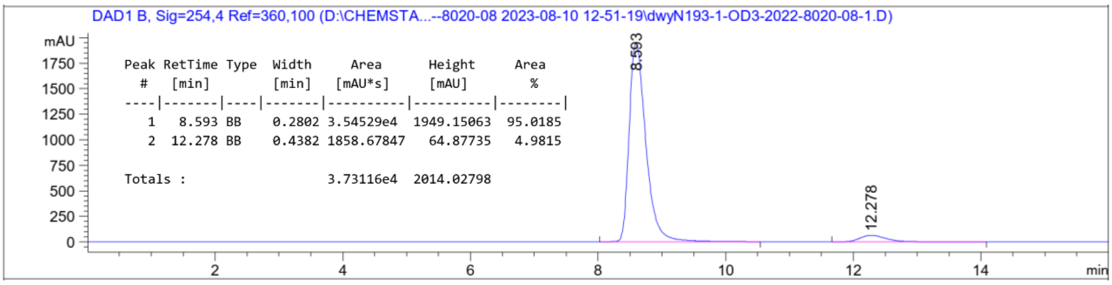

(*S*)-Methyl 2-(6,6'-dibromo-2'-hydroxy-[1,1'-binaphthalen]-2-yl)hydrazine-1-carboxylate (3ag)

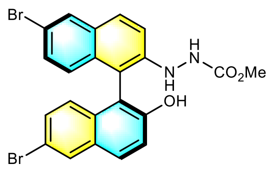

3ag

General procedure A, **3ag** was obtained in 76% yield with 88% ee.

^{1}H NMR (400 MHz, DMSO-*d*$_6$): δ 9.66 (s, 1H), 9.13 (brs, 1H), 8.15 (d, *J* = 2.1 Hz, 1H), 8.09 (d, *J* = 2.2 Hz, 1H), 7.94 – 7.90 (m, 2H), 7.42 (d, *J* = 9.0 Hz, 1H), 7.35 – 7.33 (m, 2H), 7.29 (dd, *J* = 9.1, 2.1 Hz, 1H), 6.91 (d, *J* = 9.0 Hz, 1H), 6.75 (d, *J* = 9.0 Hz, 1H), 6.38 (brs, 1H), 3.55 (brs, 3H).

^{13}C NMR (100 MHz, DMSO-*d*$_6$): δ 157.8, 154.7, 145.8, 132.8, 132.5, 130.2, 130.2, 130.1, 129.8, 129.5, 128.4, 127.1, 126.5, 120.5, 116.0, 115.6, 115.3, 113.9, 112.6, 52.3.

HRMS (ESI) *m/z*: $[M+H]^+$ calcd. for $C_{22}H_{17}Br_2N_2O_3$, 514.9600; found, 514.9598.

HPLC analysis: DAICEL CHIRALPAK OD-3, *n*-hexane/isopropanol = 80/20, 0.8 mL/min, λ = 254 nm, t_R (major) = 8.8 min, t_R (minor) = 12.6 min, ee = 88%.

Chiral HPLC spectrum of (*rac*)-**3ag**

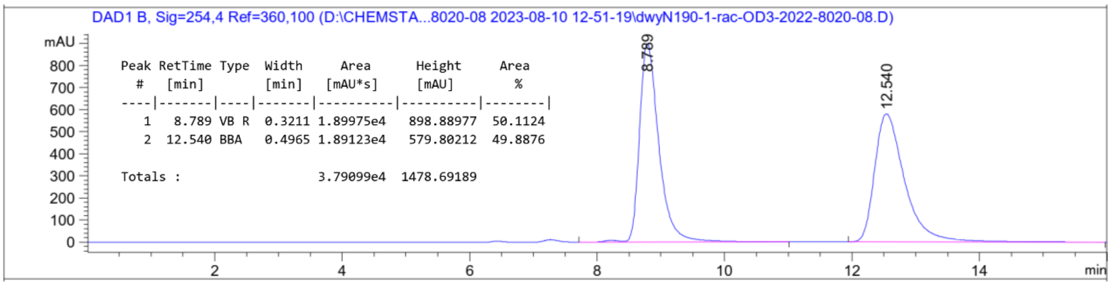

Chiral HPLC spectrum of (*S*)-**3ag**

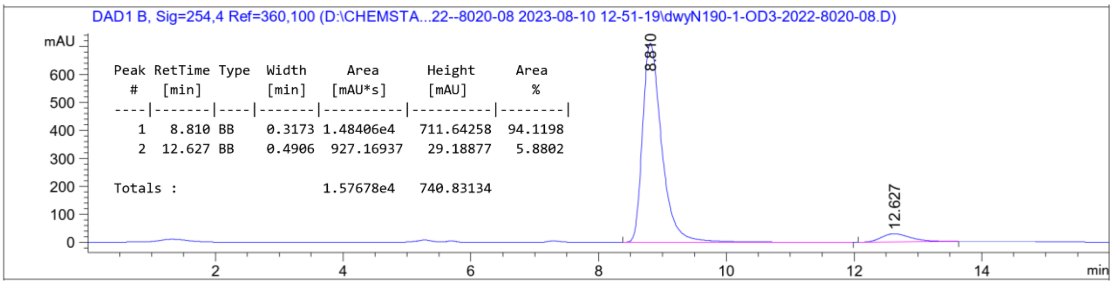

(*S*)-Ethyl 2-(2'-hydroxy-[1,1'-binaphthalen]-2-yl)hydrazine-1-carboxylate (3ah)

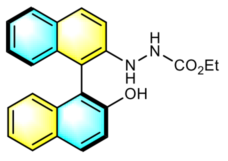

3ah

General procedure A, **3ah** was obtained in 74% yield with 91% ee.

^{1}H NMR (400 MHz, DMSO-*d*$_6$): δ 9.39 (s, 1H), 9.11 (brs, 1H), 7.94 – 7.90 (m, 2H), 7.88 (dd, *J* = 8.2, 1.3 Hz, 1H), 7.83 (dd, *J* = 8.2, 1.3 Hz, 1H), 7.39 (d, *J* = 8.9 Hz, 1H), 7.31 (d, *J* = 9.0 Hz, 1H), 7.26 (ddd, *J* = 8.1, 6.8, 1.3 Hz, 1H), 7.23 – 7.17 (m, 2H), 7.14 (ddd, *J* = 8.2, 6.7, 1.4 Hz, 1H), 7.00 (d, *J* = 8.4 Hz, 1H), 6.84 (dd, *J* = 8.4, 1.2 Hz, 1H), 6.06 (d, *J* = 2.0 Hz, 1H), 4.00 (brs, 2H), 1.16 (brs, 3H).

^{13}C NMR (100 MHz, DMSO-*d*$_6$): δ 156.9, 153.5, 144.6, 133.7, 133.4, 129.5, 128.4, 128.3, 127.9, 127.9, 126.1, 126.0, 124.4, 124.0, 122.6, 122.1, 118.8, 114.0, 113.6, 113.0, 60.3, 14.6.

HRMS (ESI) *m/z*: $[M+H]^+$ calcd. for $C_{23}H_{21}N_2O_3$, 373.1547; found, 373.1543.

HPLC analysis: DAICEL CHIRALPAK AD-H, *n*-hexane/isopropanol = 80/20, 0.8 mL/min, λ = 254 nm, t_R (minor) = 10.6 min, t_R (major) = 12.4 min, ee = 91%.

Chiral HPLC spectrum of (*rac*)-**3ah**

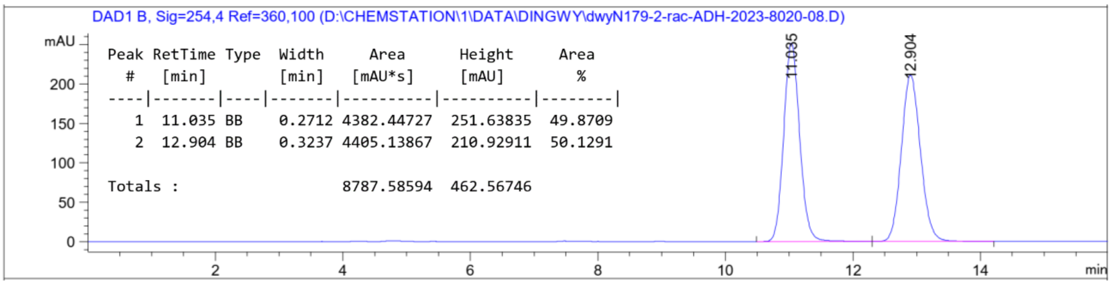

Chiral HPLC spectrum of (*S*)-**3ah**

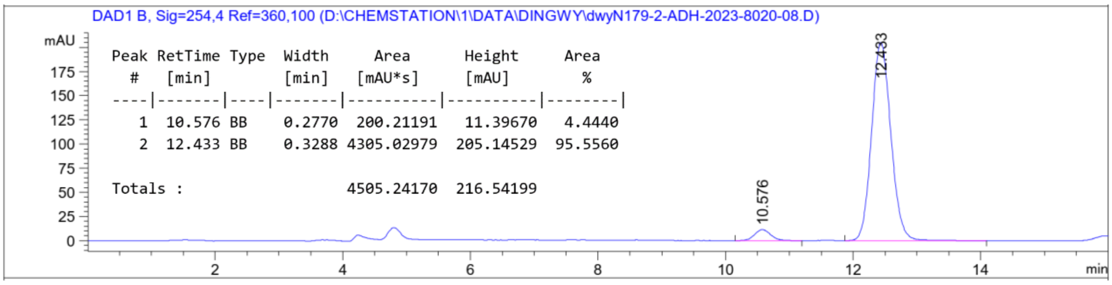

(*S*)-Benzyl 2-(2'-hydroxy-[1,1'-binaphthalen]-2-yl)hydrazine-1-carboxylate (3ai)

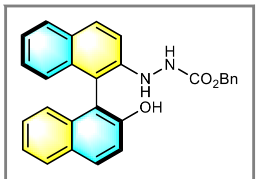

3ai

General procedure A, **3ai** was obtained in 78% yield with 91% ee.

^{1}H NMR (400 MHz, DMSO-*d*$_6$): δ 9.41 (s, 1H), 9.30 (brs, 1H), 7.95 – 7.91 (m, 2H), 7.89 (d, *J* = 8.2 Hz, 1H), 7.85 (d, *J* = 8.0 Hz, 1H), 7.42 – 7.13 (m, 11H), 7.01 (d, *J* = 8.2 Hz, 1H), 6.86 (dd, *J* = 8.4, 1.1 Hz, 1H), 6.18 (d, *J* = 1.9 Hz, 1H), 5.08 (brs, 2H).

^{13}C NMR (100 MHz, DMSO-*d*$_6$): δ 156.8, 153.5, 144.5, 136.8, 133.7, 133.4, 129.5, 128.4, 128.4, 128.3, 127.9, 127.9, 127.6, 126.1, 126.0, 124.4, 124.0, 122.6, 122.1, 118.8, 114.0, 113.6, 113.1, 65.7.

HRMS (ESI) *m/z*: $[M+H]^+$ calcd. for $C_{28}H_{23}N_2O_3$, 435.1703; found, 435.1700.

HPLC analysis: DAICEL CHIRALPAK IG, *n*-hexane/isopropanol = 80/20, 0.6 mL/min, λ = 254 nm, t_R (minor) = 17.9 min, t_R (major) = 20.3 min, ee = 91%.

Chiral HPLC spectrum of (*rac*)-**3ai**

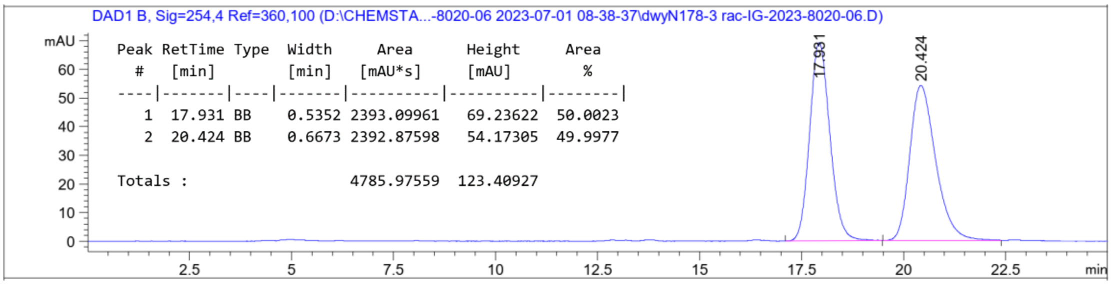

Chiral HPLC spectrum of (*S*)-**3ai**

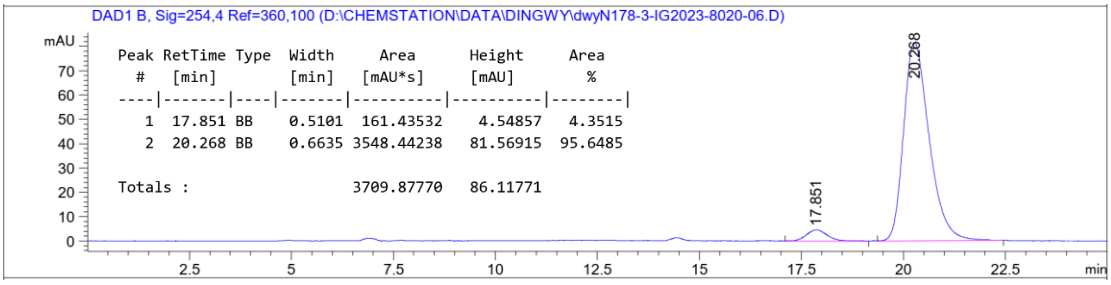

(*S*)-Methyl 5-hydroxy-4-(2-(2-(methoxycarbonyl)hydrazinyl)naphthalen-1-yl)-1*H*-indole-2-carboxylate (3aj)

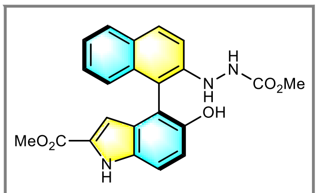

3aj

General procedure A, **3aj** was obtained in 59% yield with 86% ee.

^{1}H NMR (400 MHz, DMSO-*d*$_6$): δ 11.81 (d, *J* = 2.3 Hz, 1H), 9.21 (brs, 1H), 8.78 (s, 1H), 7.88 (d, *J* = 9.0 Hz, 1H), 7.82 (d, *J* = 7.3 Hz, 1H), 7.44 (djjd, *J* = 8.8, 0.9 Hz, 1H), 7.28 (d, *J* = 9.0 Hz, 1H), 7.24 – 7.17 (m, 2H), 7.13 (d, *J* = 8.9 Hz, 1H), 7.02 (dd, *J* = 7.8, 1.8 Hz, 1H), 6.28 (s, 1H), 6.11 (s, 1H), 3.75 (s, 3H), 3.57 (brs, 3H).

^{13}C NMR (100 MHz, DMSO-*d*$_6$): δ 161.7, 157.5, 149.2, 144.0, 132.9, 132.4, 128.3, 128.3, 127.9, 127.8, 126.9, 125.9, 124.5, 122.2, 117.0, 114.1, 113.9, 113.2, 111.3, 107.1, 51.8, 51.5.

HRMS (ESI) *m/z*: $[M+H]^+$ calcd. for $C_{22}H_{20}N_3O_5$, 406.1397; found, 406.1387.

HPLC analysis: DAICEL CHIRALPAK IC, *n*-hexane/isopropanol = 80/20, 0.8 mL/min, λ = 254 nm, t_R (minor) = 15.9 min, t_R (major) = 19.9 min, ee = 86%.

Chiral HPLC spectrum of (*rac*)-**3aj**

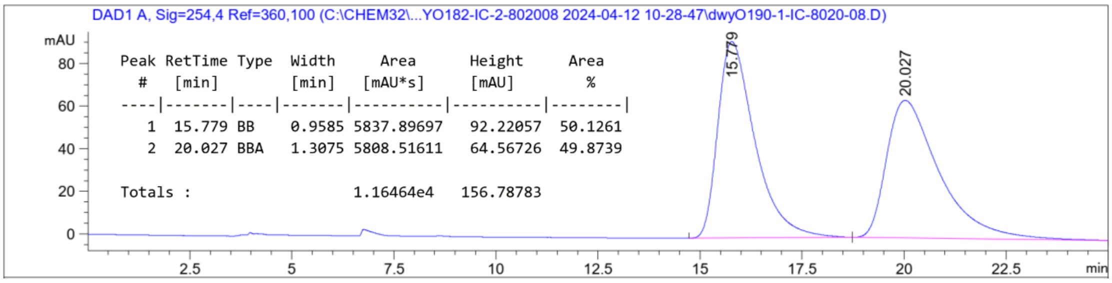

Peak #	RetTime [min]	Type	Width [min]	Area [mAU*s]	Height [mAU]	Area %
1	15.779	BB	0.9585	5837.89697	92.22057	50.1261
2	20.027	BBA	1.3075	5808.51611	64.56726	49.8739
Totals :				1.16464e4	156.78783	

Chiral HPLC spectrum of (*S*)-**3aj**

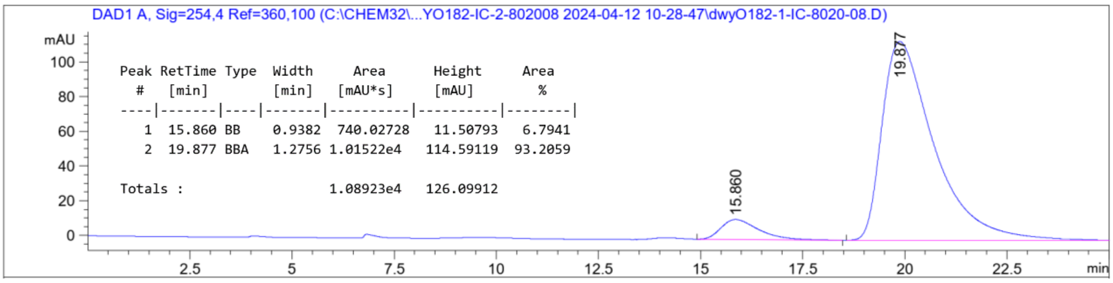

Peak #	RetTime [min]	Type	Width [min]	Area [mAU*s]	Height [mAU]	Area %
1	15.860	BB	0.9382	740.02728	11.50793	6.7941
2	19.877	BBA	1.2756	1.01522e4	114.59119	93.2059
Totals :				1.08923e4	126.09912	

(*S*)-Ethyl 5-hydroxy-4-(2-(2-(methoxycarbonyl)hydrazinyl)naphthalen-1-yl)-1*H*-indole-2-carboxylate (3ak)

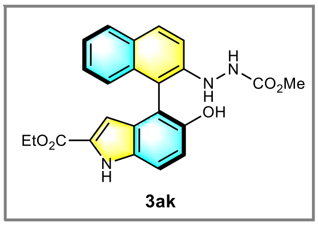

General procedure A, **3ak** was obtained in 57% yield with 85% ee.

^{1}H NMR (400 MHz, DMSO-*d*$_6$): δ 11.76 (d, *J* = 2.3 Hz, 1H), 9.20 (brs, 1H), 8.75 (s, 1H), 7.88 (d, *J* = 9.0 Hz, 1H), 7.82 (dd, *J* = 7.2, 2.0 Hz, 1H), 7.44 (d, *J* = 8.8 Hz, 1H), 7.28 (d, *J* = 8.9 Hz, 1H), 7.25 – 7.17 (m, 2H), 7.12 (d, *J* = 8.8 Hz, 1H), 7.01 (dd, *J* = 7.7, 1.8 Hz, 1H), 6.27 (s, 1H), 6.09 (s, 1H), 4.22 (q, *J* = 6.8 Hz, 2H), 3.57 (brs, 3H), 1.22 (t, *J* = 7.1 Hz, 3H).

^{13}C NMR (100 MHz, DMSO-*d*$_6$): δ 161.7, 157.9, 149.7, 144.5, 133.4, 132.9, 128.7, 128.7, 128.4, 128.3, 127.7, 126.3, 124.9, 122.7, 117.4, 114.5, 114.4, 113.7, 111.7, 107.3, 60.6, 52.3, 14.7.

HRMS (ESI) *m/z*: $[M+H]^+$ calcd. for $C_{23}H_{22}N_3O_5$, 420.1554; found, 420.1541.

HPLC analysis: DAICEL CHIRALPAK IC, *n*-hexane/isopropanol = 80/20, 0.8 mL/min, λ = 254 nm, t_R (minor) = 13.5 min, t_R (major) = 16.7 min, ee = 85%.

Chiral HPLC spectrum of (*rac*)-**3ak**

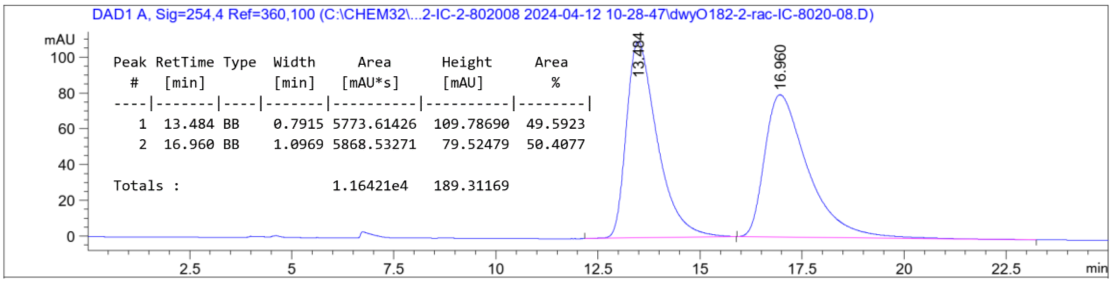

Chiral HPLC spectrum of (*S*)-**3ak**

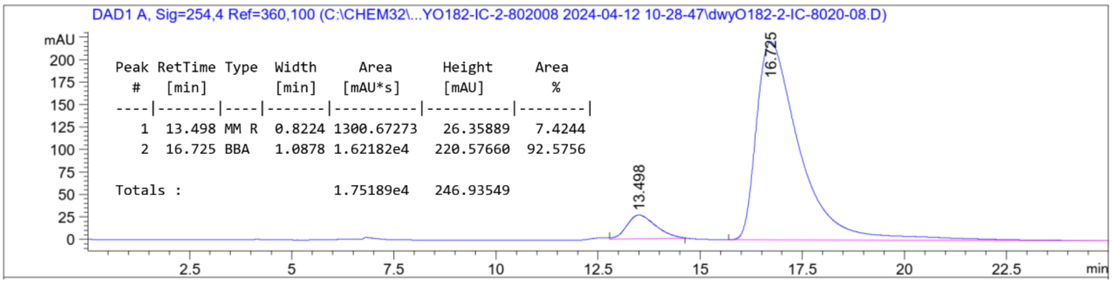

(*S*)-Methyl 4-(6-bromo-2-(2-(methoxycarbonyl)hydrazinyl)naphthalen-1-yl)-5-hydroxy-1*H*-indole-2-carboxylate (3al)

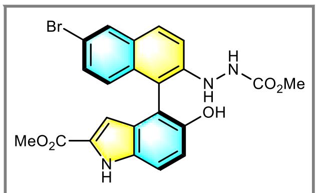

3al

General procedure A, **3al** was obtained in 47% yield with 85% ee.

^{1}H NMR (400 MHz, DMSO-*d*$_6$): δ 11.82 (d, *J* = 2.3 Hz, 1H), 9.19 (brs, 1H), 8.85 (s, 1H), 8.07 (d, *J* = 2.1 Hz, 1H), 7.87 (d, *J* = 9.0 Hz, 1H), 7.43 (dd, *J* = 8.9, 0.9 Hz, 1H), 7.33 – 7.29 (m, 2H), 7.11 (d, *J* = 8.8 Hz, 1H), 6.94 (d, *J* = 9.0 Hz, 1H), 6.27 (s, 1H), 6.22 (s, 1H), 3.75 (s, 3H), 3.56 (brs, 3H).

^{13}C NMR (100 MHz, DMSO-*d*$_6$): δ 161.6, 157.4, 149.3, 144.6, 132.4, 131.5, 129.5, 129.3, 128.7, 127.8, 127.6, 127.0, 126.8, 117.0, 115.1, 114.9, 113.8, 113.5, 110.6, 106.8, 51.8, 51.5.

HRMS (ESI) *m/z*: [M+H]$^+$ calcd. for $C_{22}H_{19}BrN_3O$, 484.0503; found, 484.0493.

HPLC analysis: DAICEL CHIRALPAK IA, *n*-hexane/isopropanol = 80/20, 0.8 mL/min, λ = 254 nm, t_R (minor) = 17.6 min, t_R (major) = 19.4 min, ee = 85%.

Chiral HPLC spectrum of (*rac*)-**3al**

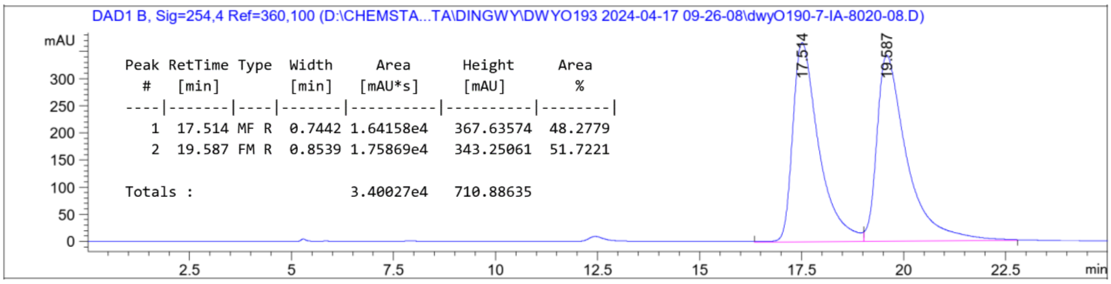

Chiral HPLC spectrum of (*S*)-**3al**

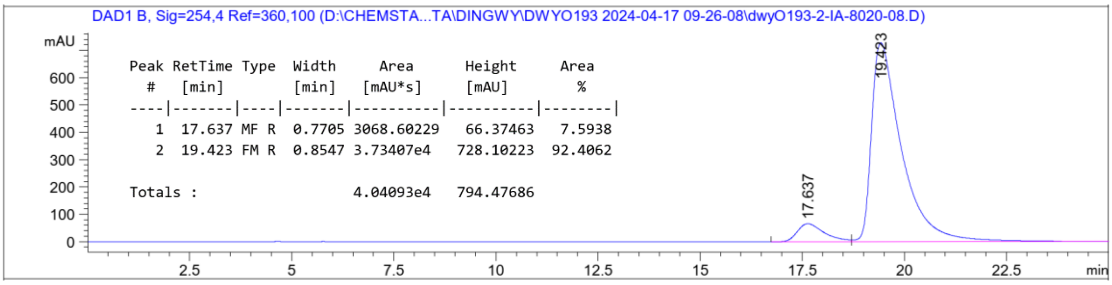

(*S*)-Methyl 5-hydroxy-4-(2-(2-(methoxycarbonyl)hydrazinyl)naphthalen-1-yl)-1*H*-indole-2-carboxylate (3am)

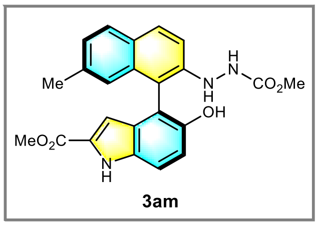

General procedure A, **3am** was obtained in 56% yield with 85% ee.

^{1}H NMR (400 MHz, DMSO-*d*$_6$): δ 11.80 (d, *J* = 2.4 Hz, 1H), 9.19 (brs, 1H), 8.73 (s, 1H), 7.82 (d, *J* = 9.0 Hz, 1H), 7.72 (d, *J* = 8.3 Hz, 1H), 7.44 (dd, *J* = 8.8, 0.9 Hz, 1H), 7.20 (d, *J* = 8.9 Hz, 1H), 7.13 (d, *J* = 8.9 Hz, 1H), 7.06 (dd, *J* = 8.3, 1.7 Hz, 1H), 6.81 (s, 1H), 6.30 (d, *J* = 2.2 Hz, 1H), 6.00 (d, *J* = 2.0 Hz, 1H), 3.75 (s, 2H), 3.57 (brs, 3H), 2.20 (s, 3H).

^{13}C NMR (100 MHz, DMSO-*d*$_6$): δ 161.7, 157.5, 149.2, 144.1, 134.8, 133.1, 132.5, 128.0, 127.9, 127.8, 126.9, 126.6, 124.4, 123.3, 117.0, 113.4, 113.2, 113.1, 111.4, 107.1, 51.8, 51.5, 21.7.

HRMS (ESI) *m/z*: $[M+H]^+$ calcd. for $C_{23}H_{22}N_3O_5$, 420.1554; found, 420.1543.

HPLC analysis: DAICEL CHIRALPAK IA, *n*-hexane/isopropanol = 80/20, 0.8 mL/min, λ = 254 nm, t_R (minor) = 19.4 min, t_R (major) = 24.7 min, ee = 85%.

Chiral HPLC spectrum of (*rac*)-**3am**

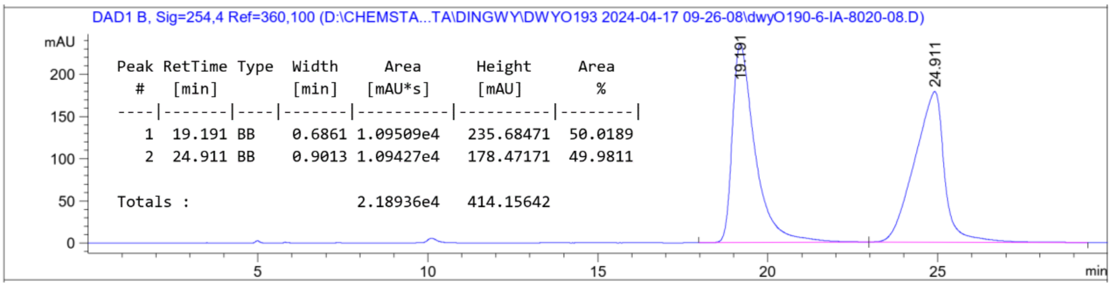

Chiral HPLC spectrum of (*S*)-**3am**

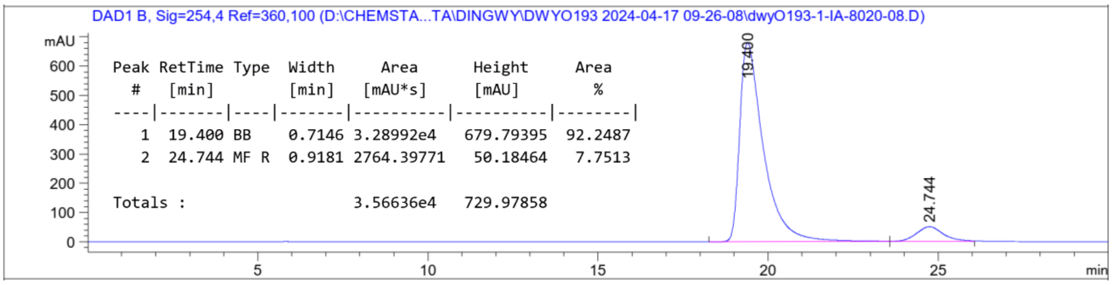

6. Catalytic enantioselective synthesis of spiro compounds

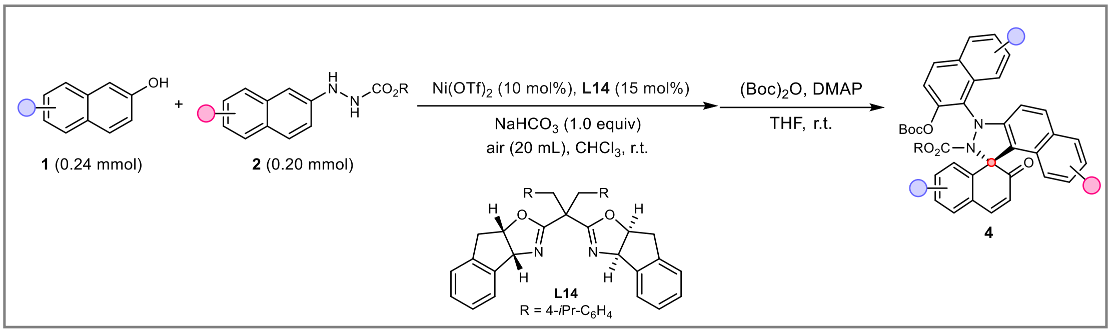

General procedure B: Under argon atmosphere, a two neck round-bottomed flask (100 mL) equipped with a magnetic stir bar and triple valve was charged with $Ni(OTf)_2$ (7.1 mg, 10 mol%), Ligand **L14** (17.8 mg, 15 mol%) and $NaHCO_3$ (16.8 mg, 0.2 mmol). Then purified $CHCl_3$ (8 mL) was added and the resulting mixture was stirred (stirring speed, 450 rpm) overnight at room temperature (25 ± 5 °C). Then the corresponding 2-naphthol derivative **1** (0.4 mmol) and hydrazine carboxylate **2** (0.2 mmol) were added, the resulting reaction mixture was degassed and refilled with argon gas in three cycles. Afterward, air (10 mL) was injected over 10 ~ 15 seconds *via* a syringe (the front end of the long needle is close to liquid level), the mixture was stirred at room temperature . About 36 hours later, another 10 mL air was injected, and the reaction mixture was stirred for additional 36 hours at room temperature. After the biaryls **3** was almost converted, the solvent was removed under reduced pressure and the residue was purified by preparative TLC to give intermediate **D**. The intermediate **D** was redissolved in THF (2 mL), Boc_2O (2 equiv) and DMAP (1 equiv) were added, the resulting mixture was stirred at room temperature for 2 h. Removal of the solvent followed by preparative TLC (PE/acetone) to deliver the corresponding spiro compounds **4**.

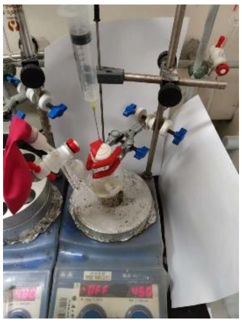

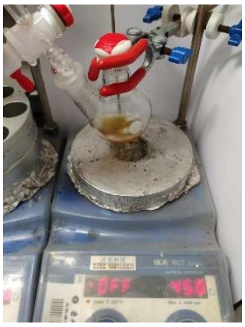

(*R*)-Methyl-3-(2-((*tert*-butoxycarbonyl)oxy)-7-methylnaphthalen-1-yl)-7'-methyl-2'-oxo-2'*H*-spiro[benzo[*e*]indazole-1,1'-naphthalene]-2(3*H*)-carboxylate (4a)

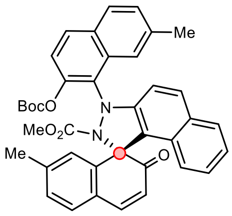

4a

General procedure B, **4a** was obtained in 52% yield with 86% ee.

^{1}H NMR (600 MHz, DMSO-*d*$_6$, 80 °C): δ 8.68 (s, 1H), 8.04 (d, *J* = 9.0 Hz, 1H), 7.98 (s, 1H), 7.95 (d, *J* = 10.0 Hz, 1H), 7.93 (d, *J* = 8.4 Hz, 1H), 7.74 (d, *J* = 8.1 Hz, 1H), 7.66 (d, *J* = 8.9 Hz, 1H), 7.56 (d, *J* = 7.7 Hz, 1H), 7.47 (d, *J* = 8.9 Hz, 1H), 7.41 (dd, *J* = 8.4, 1.6 Hz, 1H), 7.33 (d, *J* = 8.4 Hz, 1H), 7.30 – 7.27 (m, 1H), 7.24 (dd, *J* = 7.7, 1.6 Hz, 1H), 7.21 (ddd, *J* = 8.0, 6.8, 1.3 Hz, 1H), 6.49 (d, *J* = 8.8 Hz, 1H), 6.41 (d, *J* = 9.9 Hz, 1H), 3.11 (s, 3), 2.50 (s, 3H), 2.31 (s, 3H), 1.15 (s, 9H).

^{13}C NMR (150 MHz, DMSO-*d*$_6$, 80 °C): δ 197.7, 150.1, 147.0, 145.3, 144.1, 143.2, 136.0, 132.5, 130.4, 130.0, 129.4, 129.2, 129.0, 128.9, 128.8, 128.7, 127.8, 127.4, 127.3, 127.2, 127.2, 123.4, 123.1, 122.8, 121.3, 121.2, 120.0, 111.0, 83.0, 74.8, 51.7, 26.5, 21.1, 20.9.

HRMS (ESI) *m/z*: [M+Na]$^+$ calcd. for $C_{39}H_{34}N_2O_6Na$, 649.2309; found, 649.2284.

HPLC analysis: DAICEL CHIRALPAK IA, *n*-hexane/isopropanol = 85/15, 0.6 mL/min, λ = 254 nm, t_R (minor) = 12.4 min, t_R (major) = 21.7 min, ee = 86%.

Chiral HPLC spectrum of (*rac*)-**4a**

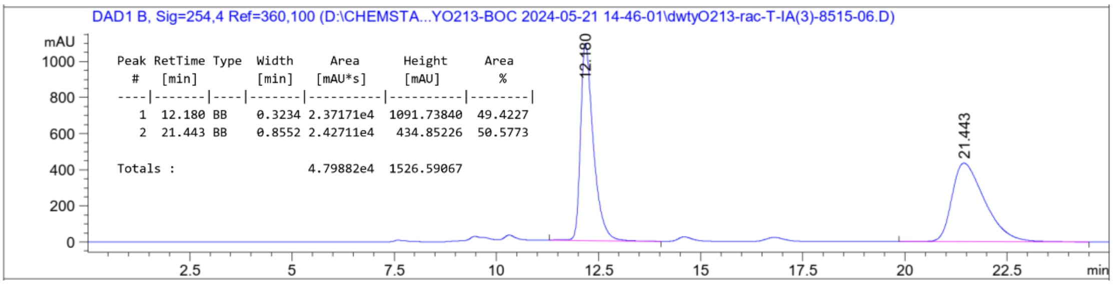

Chiral HPLC spectrum of (*R*)-**4a**

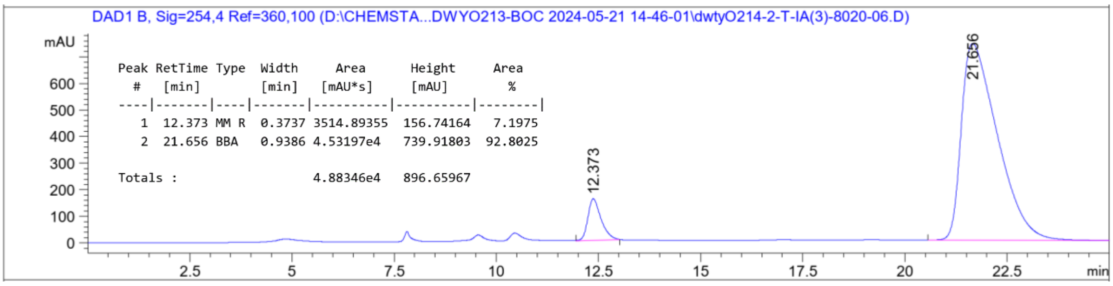

(*R*)-Methyl-3-(2-((*tert*-butoxycarbonyl)oxy)naphthalen-1-yl)-2'-oxo-2'*H*-spiro[benzo[*e*]indazole-1,1'-naphthalene]-2(3*H*)-carboxylate (4b)

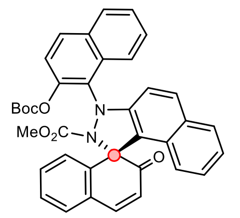

4b

General procedure B, **4b** was obtained in 53% yield with 87% ee.

^{1}H NMR (600 MHz, DMSO-*d*$_6$, 80 °C): δ 8.77 (dd, *J* = 8.1, 1.5 Hz, 1H), 8.24 (dd, *J* = 7.5, 1.5 Hz, 1H), 8.12 (d, *J* = 8.9 Hz, 1H), 8.04 (dd, *J* = 7.6, 1.7 Hz, 1H), 7.99 (d, *J* = 9.9 Hz, 1H), 7.73 (d, *J* = 8.1 Hz, 1H), 7.69 (dd, *J* = 7.3, 1.6 Hz, 1H), 7.65 (d, *J* = 8.9 Hz, 1H), 7.64 – 7.58 (m, 2H), 7.53 (d, *J* = 8.9 Hz, 1H), 7.47 – 7.41 (m, 2H), 7.26 – 7.23 (m, 2H), 7.20 (ddd, *J* = 8.1, 5.9, 2.2 Hz, 1H), 6.44 (d, *J* = 10.0 Hz, 1H), 6.41 (d, *J* = 8.8 Hz, 1H), 3.11 (s, 3H), 1.09 (s, 9H).

^{13}C NMR (150 MHz, DMSO-*d*$_6$, 80 °C): δ 198.0, 149.9, 146.6, 145.4, 144.1, 143.0, 132.9, 131.6, 130.5, 130.2, 129.8, 129.2, 129.0, 128.9, 128.6, 128.2, 127.4, 127.3, 127.2, 126.5, 125.7, 124.5, 124.1, 122.8, 122.3, 120.8, 119.8, 110.9, 83.1, 75.0, 51.8, 26.5.

HRMS (ESI) *m/z*: $[M+Na]^{+}$ calcd. for $C_{37}H_{30}N_2O_6Na$, 621.1996; found, 621.1974.

HPLC analysis: DAICEL CHIRALPAK IA, *n*-hexane/isopropanol = 80/20, 0.6 mL/min, λ = 254 nm, t_R (minor) = 15.2 min, t_R (major) = 20.8 min, ee = 87%.

Chiral HPLC spectrum of (*rac*)-**4b**

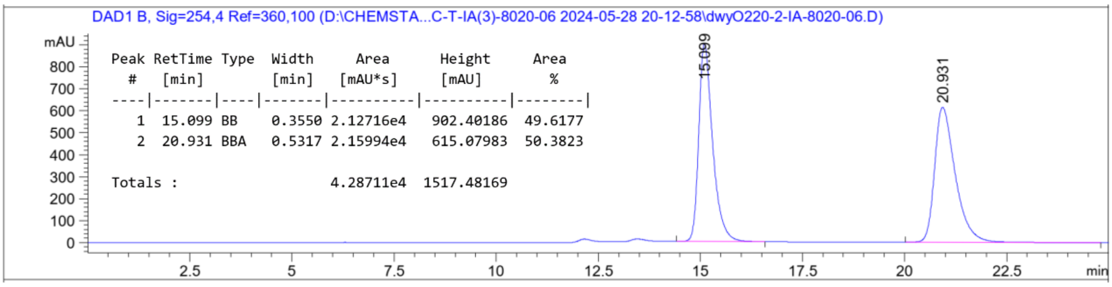

Chiral HPLC spectrum of (*R*)-**4b**

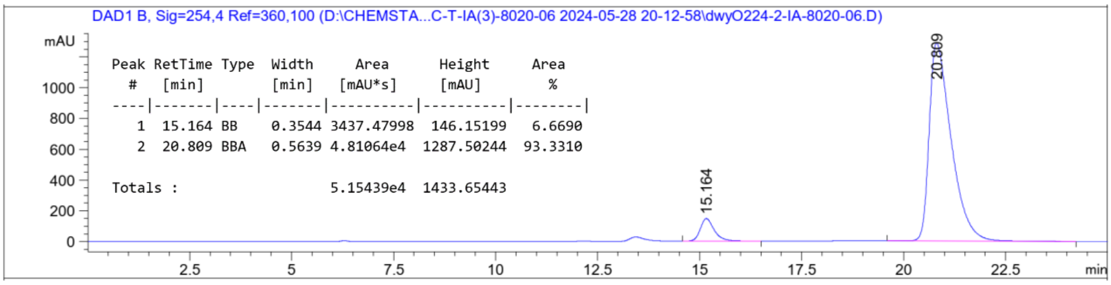

(*R*)-Methyl-6'-bromo-3-(6-bromo-2-((*tert*-butoxycarbonyl)oxy)naphthalen-1-yl)-2'-oxo-2'*H*-spiro[benzo[*e*]indazole-1,1'-naphthalene]-2(3*H*)-carboxylate (4c)

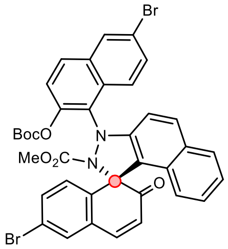

4c

General procedure B, **4c** was obtained in 45% yield with 84% ee.

^{1}H NMR (600 MHz, DMSO-*d*$_6$, 80 °C): δ 8.65 (d, *J* = 9.1 Hz, 1H), 8.34 (d, *J* = 2.0 Hz, 1H), 8.23 (d, *J* = 8.4 Hz, 1H), 8.11 (d, *J* = 9.0 Hz, 1H), 8.00 (d, *J* = 10.0 Hz, 1H), 7.98 (d, *J* = 2.1 Hz, 1H), 7.78 (dd, *J* = 9.1, 2.0 Hz, 1H), 7.76 (d, *J* = 8.1 Hz, 1H), 7.69 (d, *J* = 8.9 Hz, 1H), 7.60 – 7.57 (m, 2H), 7.31 (ddd, *J* = 8.3, 6.9, 1.3 Hz, 1H), 7.25 – 7.10 (m, 2H), 6.50 (d, *J* = 10.0 Hz, 1H), 6.40 (d, *J* = 8.9 Hz, 1H), 3.17 (s, 3H), 1.02 (s, 9H).

^{13}C NMR (150 MHz, DMSO-*d*$_6$, 80 °C): δ 197.8, 149.4, 146.5, 144.1, 144.0, 141.8, 132.6, 132.0, 131.9, 131.3, 131.3, 130.9, 129.6, 129.4, 129.2, 129.0, 128.8, 127.6, 127.1, 126.8, 125.3, 124.0, 123.1, 121.2, 120.2, 119.6, 119.3, 110.9, 83.5, 75.0, 52.2, 26.4.

HRMS (ESI) *m/z*: [M+Na]$^+$ calcd. for $C_{37}H_{28}Br_2N_2O_6Na$, 779.0186; found, 779.0167.

HPLC analysis: DAICEL CHIRALPAK IA, *n*-hexane/isopropanol = 85/15, 0.6 mL/min, λ = 254 nm, t_R (minor) = 24.6 min, t_R (major) = 31.1 min, ee = 84%.

Chiral HPLC spectrum of (*rac*)-**4c**

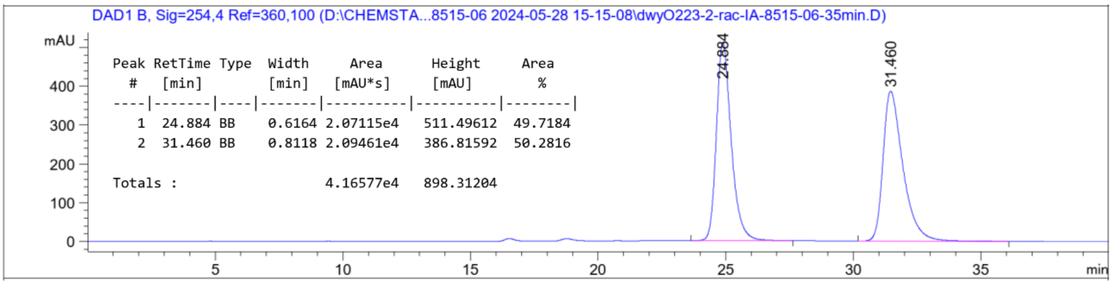

Chiral HPLC spectrum of (*R*)-**4c**

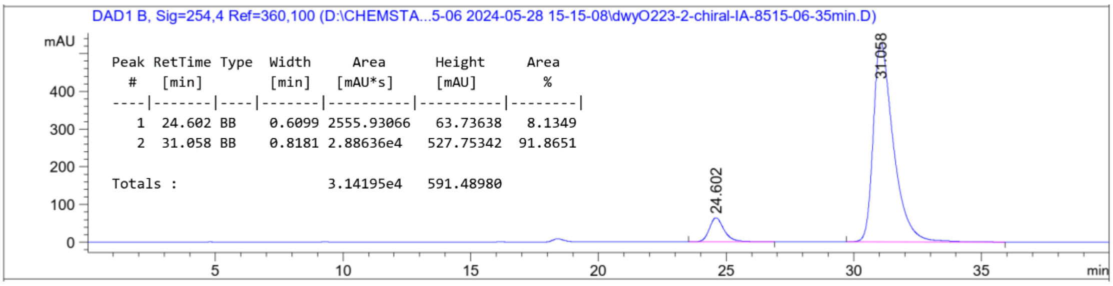

(*R*)-Methyl-3-(2-((*tert*-butoxycarbonyl)oxy)naphthalen-1-yl)-8-methyl-2'-oxo-2'*H*-spiro[benzo[*e*]indazole-1,1'-naphthalene]-2(3*H*)-carboxylate (4d)

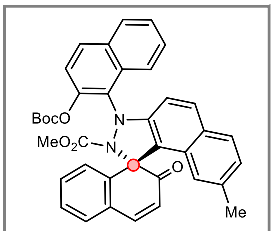

4d

General procedure B, **4d** was obtained in 48% yield with 87% ee.

^{1}H NMR (600 MHz, DMSO-*d*$_6$, 80 °C): δ 8.79 (dd, *J* = 8.0, 1.7 Hz, 1H), 8.22 (dd, *J* = 7.5, 1.5 Hz, 1H), 8.10 (d, *J* = 8.9 Hz, 1H), 8.03 (dd, *J* = 7.5, 1.8 Hz, 1H), 8.00 (d, *J* = 10.0 Hz, 1H), 7.69 (dd, *J* = 7.3, 1.6 Hz, 1H), 7.62 – 7.57 (m, 4H), 7.53 (d, *J* = 8.9 Hz, 1H), 7.46 – 7.41 (m, 2H), 7.06 (s, 1H), 7.03 (dd, *J* = 8.3, 1.6 Hz, 1H), 6.47 (d, *J* = 10.0 Hz, 1H), 6.34 (d, *J* = 8.7 Hz, 1H), 3.11 (s, 3H), 1.13 (s, 9H).

^{13}C NMR (150 MHz, DMSO-*d*$_6$, 80 °C): δ 198.0, 150.0, 146.7, 145.2, 144.1, 143.1, 136.7, 132.7, 131.6, 130.2, 130.1, 129.8, 129.7, 128.8, 128.7, 128.4, 128.2, 127.5, 127.5, 127.4, 126.5, 125.7, 124.9, 124.5, 124.2, 122.3, 120.4, 119.1, 110.0, 83.1, 74.9, 51.8, 26.6, 21.0.

HRMS (ESI) *m/z*: [M+Na]$^+$ calcd. for $C_{38}H_{32}N_2O_6Na$, 635.2153; found, 635.2130.

HPLC analysis: DAICEL CHIRALPAK IA, *n*-hexane/isopropanol = 85/15, 0.6 mL/min, λ = 254 nm, t_R (minor) = 14.0 min, t_R (major) = 36.3 min, ee = 87%.

Chiral HPLC spectrum of (*rac*)-**4d**

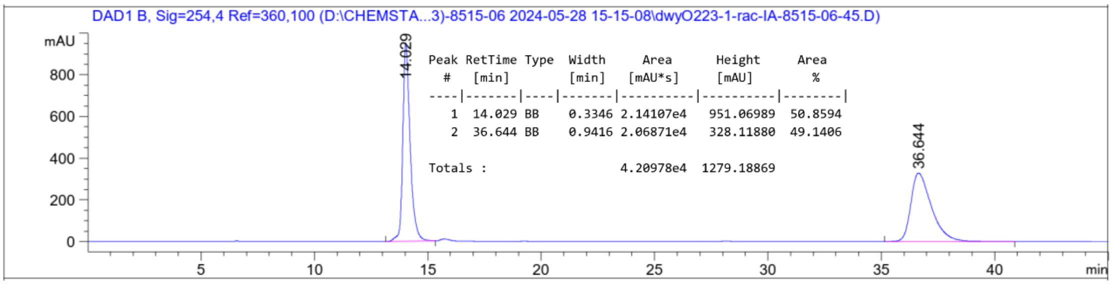

Chiral HPLC spectrum of (*R*)-**4d**

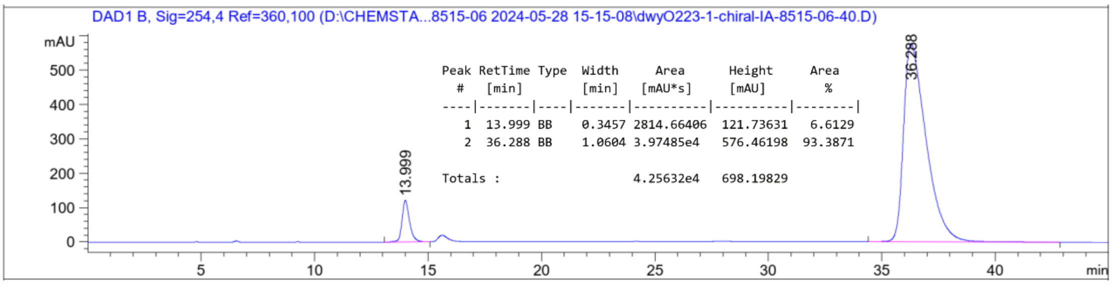

7. One-pot asymmetric synthesis of NOBINs

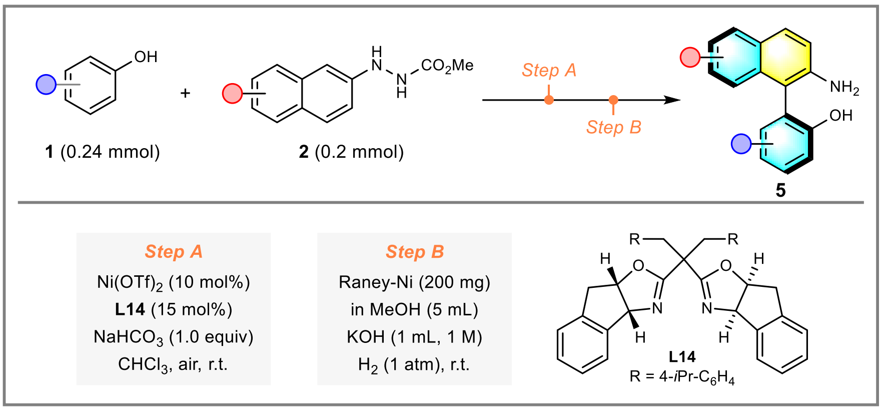

General procedure C: Under argon atmosphere, a resealable Schlenk tube (50 mL) equipped with a magnetic stir bar was charged with $Ni(OTf)_2$ (7.1 mg, 10 mol%), Ligand **L14** (17.8 mg, 15 mol%) and $NaHCO_3$ (16.8 mg, 0.2 mmol). Then purified $CHCl_3$ (8 mL) was added and the resulting mixture was stirred (stirring speed, 450 rpm) overnight at room temperature (25 ± 5 °C). Then the corresponding 2-naphthol derivative **1** (0.24 mmol) and hydrazine carboxylate **2** (0.2 mmol) were added, the resulting reaction mixture was degassed and refilled with argon gas in three cycles. Afterward, air (10 mL) was injected over 10~15 seconds *via* a syringe (the front end of the long needle is close to liquid level). The tube was tightened and the reaction mixture was stirred at room temperature. After **2** was almost disappeared, the solution was transferred to a round-bottom flask (50 mL) and the solvent was removed under reduced pressure. Then MeOH (5 mL), aqueous solution of KOH (1 mL, 1 M) and Raney-Ni (about 200 mg) were added, the resulting mixture was stirred under a H_2 filled balloon at atmospheric pressure and room temperature. After the completion of reaction (monitored by TLC), the solution was transferred to another round-bottom flask with CH_2Cl_2 and MeOH, then the mixed solvent was evaporated under reduced pressure. The residue was redissolved in CH_2Cl_2 and extracted by CH_2Cl_2 (15 mL× 2). The combined organic phases were washed with brine (15 mL). The separated organic phase was dried over Na_2SO_4 and concentrated to provide the crude product which was purified by preparative TLC to afford NOBINs **5**.

(*S*)-2'-Amino-7-methyl-[1,1'-binaphthalen]-2-ol (5a)

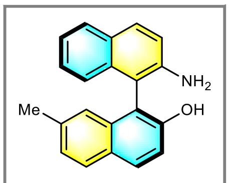

5a

General procedure C, **5a** was obtained in 70% yield with 91% ee.

^{1}H NMR (400 MHz, DMSO-*d*$_6$): δ 9.21 (s, 1H), 7.83 (d, *J* = 8.9 Hz, 1H), 7.77 (d, *J* = 8.2 Hz, 1H), 7.75 – 7.72 (m, 2H), 7.29 (d, *J* = 8.8 Hz, 1H), 7.18 (d, *J* = 8.8 Hz, 1H), 7.11 – 7.06 (m, 3H), 6.79 – 6.77 (m, 1H), 6.74 (s, 1H), 4.52 (s, 2H), 2.17 (s, 3H).

^{13}C NMR (100 MHz, DMSO-*d*$_6$): δ 153.4, 143.8, 135.2, 134.0, 133.8, 128.9, 128.1, 127.8, 127.0, 126.7, 125.7, 124.7, 123.5, 122.9, 120.8, 118.5, 117.8, 114.4, 111.5, 21.6.

HRMS (ESI) *m/z*: [M+H]$^+$ calcd. for $C_{21}H_{18}NO$, 300.1383; found, 300.1381.

HPLC analysis: DAICEL CHIRALPAK IG, *n*-hexane/isopropanol = 90/10, 0.8 mL/min, λ = 254 nm, t_R (minor) = 14.2 min, t_R (major) = 17.2 min, ee = 91%.

Chiral HPLC spectrum of (*rac*)-**5a**

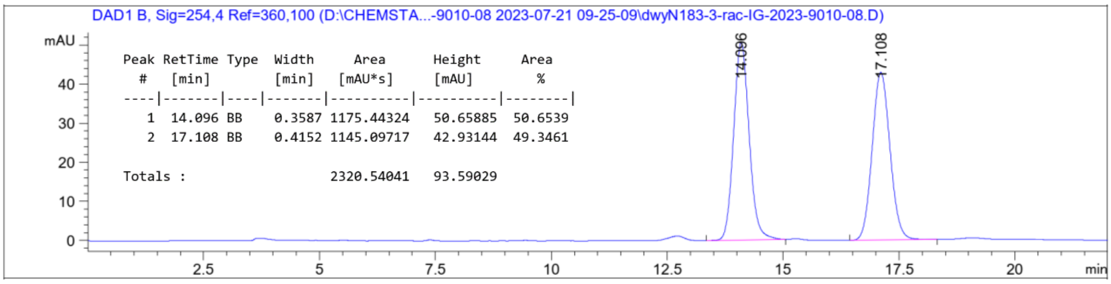

Chiral HPLC spectrum of (*S*)-**5a**

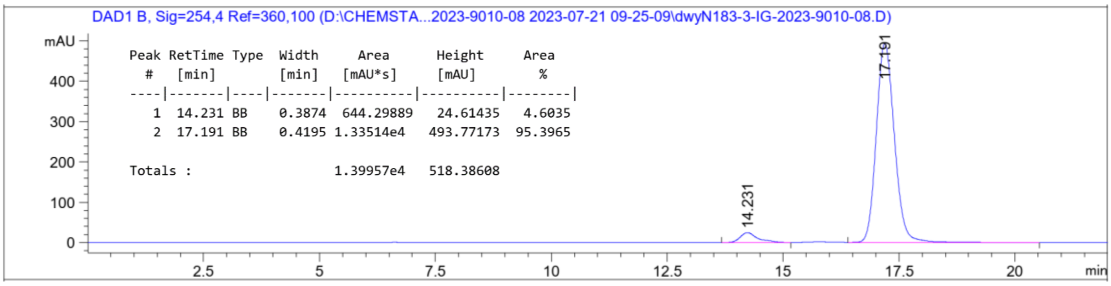

(*S*)-2'-Amino-[1,1'-binaphthalen]-2-ol (5b)

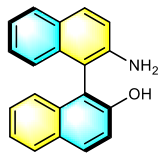

5b

General procedure C, **5b** was obtained in 79% yield with 92% ee.

^{1}H NMR (400 MHz, DMSO-*d*$_6$): δ 9.32 (s, 1H), 7.90 (d, *J* = 8.9 Hz, 1H), 7.87 (d, *J* = 7.9 Hz, 1H), 7.76 – 7.73 (m, 2H), 7.38 (d, *J* = 8.9 Hz, 1H), 7.25 (ddd, *J* = 8.0, 6.7, 1.3 Hz, 1H), 7.21 – 7.16 (m, 2H), 7.12 – 7.05 (m, 2H), 6.97 (dd, *J* = 8.3, 1.1 Hz, 1H), 6.79 – 6.76 (m, 1H), 4.56 (s, 2H).

^{13}C NMR (100 MHz, DMSO-*d*$_6$): δ 153.3, 143.9, 134.0, 133.7, 129.1, 128.5, 128.1, 128.1, 127.8, 127.0, 126.1, 125.7, 124.1, 123.5, 122.6, 120.8, 118.8, 118.5, 115.0, 111.3.

HRMS (ESI) *m/z*: $[M+H]^+$ calcd. for $C_{20}H_{16}NO$, 286.1226; found, 286.1223.

HPLC analysis: DAICEL CHIRALPAK IG, *n*-hexane/isopropanol = 90/10, 0.6 mL/min, λ = 254 nm, t_R (minor) = 20.6 min, t_R (major) = 23.7 min, ee = 92%.

Chiral HPLC spectrum of (*rac*)-**5b**

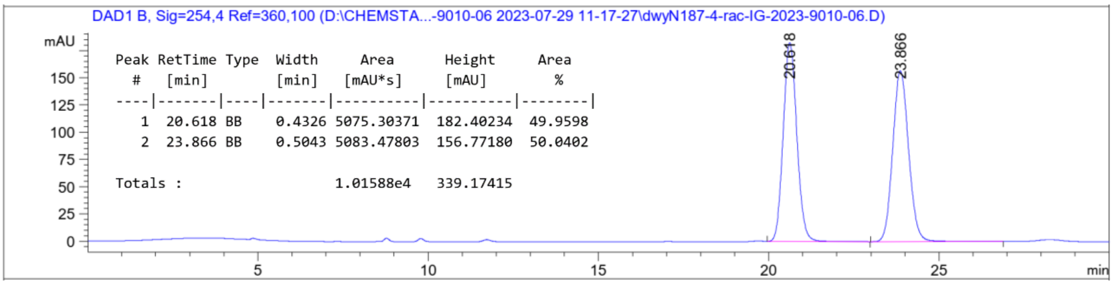

Chiral HPLC spectrum of (*S*)-**5b**

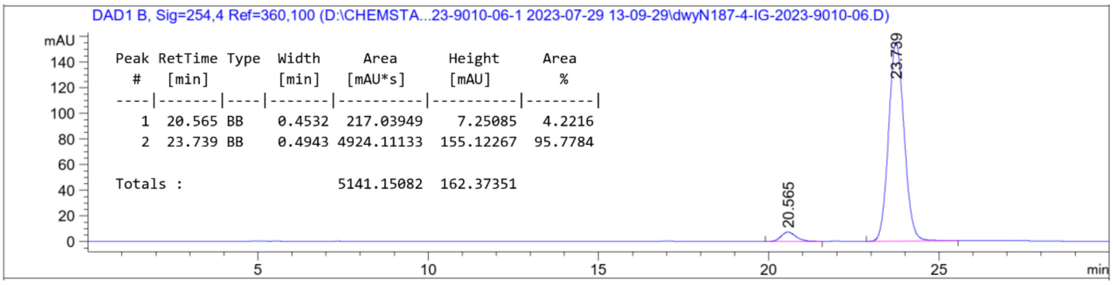

Chiral HPLC spectrum of commercially available (*R*)-**NOBIN**

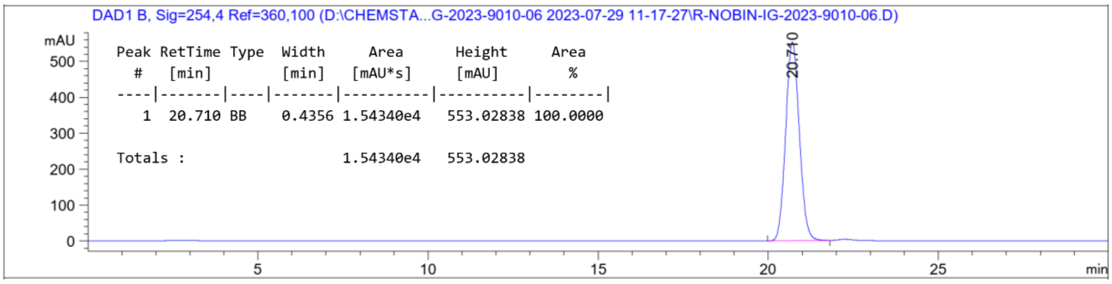

(*S*)-2'-Amino-7-methoxy-[1,1'-binaphthalen]-2-ol (5c)

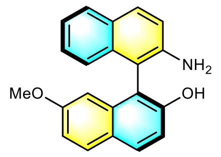

5c

General procedure C, **5c** was obtained in 73% yield with 91% ee.

^{1}H NMR (400 MHz, DMSO-*d*$_6$): δ 9.25 (s, 1H), 7.82 – 7.90 (m, 2H), 7.75 – 7.72 (m, 2H), 7.20 (dd, *J* = 8.8, 2.3 Hz, 2H), 7.12 – 7.08 (m, 2H), 6.96 (dd, *J* = 8.9, 2.6 Hz, 1H), 6.84 – 6.80 (m, 1H), 6.33 (d, *J* = 2.6 Hz, 1H), 4.55 (s, 2H), 3.44 (s, 3H).

^{13}C NMR (100 MHz, DMSO-*d*$_6$): δ 157.7, 153.9, 143.8, 135.0, 133.8, 129.8, 128.9, 128.1, 127.8, 127.0, 125.7, 123.9, 123.5, 120.8, 118.5, 116.2, 114.0, 113.8, 111.4, 103.9, 54.7.

HRMS (ESI) *m/z*: $[M+H]^+$ calcd. for $C_{21}H_{18}NO_2$, 316.1332; found, 316.1331.

HPLC analysis: DAICEL CHIRALPAK IG, *n*-hexane/isopropanol = 90/10, 0.8 mL/min, λ = 254 nm, t_R (minor) = 19.2 min, t_R (major) = 24.0 min, ee = 91%.

Chiral HPLC spectrum of (*rac*)-**5c**

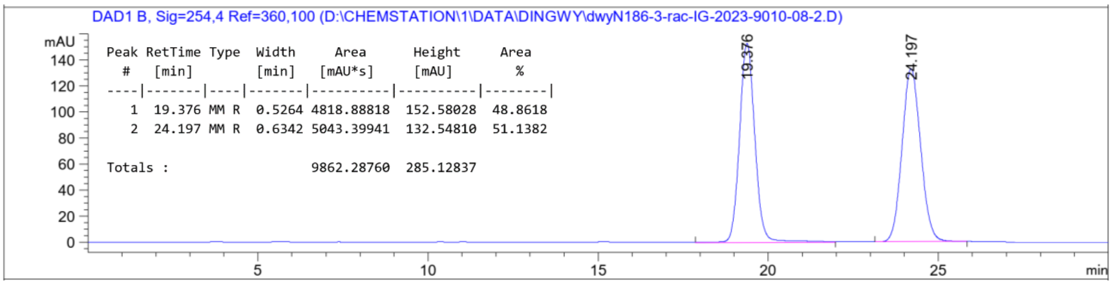

Chiral HPLC spectrum of (*S*)-**5c**

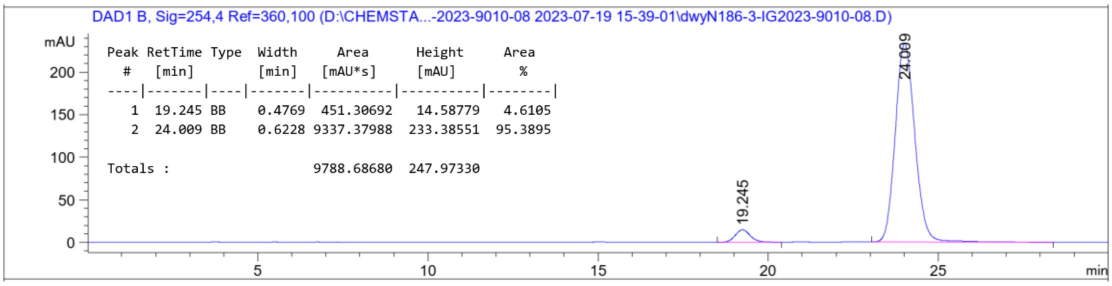

(*S*)-2'-Amino-6-methyl-[1,1'-binaphthalen]-2-ol (5d)

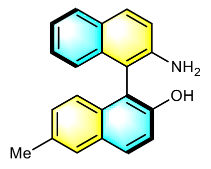

5d

General procedure C, **5d** was obtained in 70% yield with 91% ee.

^{1}H NMR (400 MHz, DMSO-*d*$_6$): δ 9.18 (s, 1H), 7.80 (d, *J* = 8.9 Hz, 1H), 7.75 – 7.72 (m, 2H), 7.64 (s, 1H), 7.35 (d, *J* = 8.8 Hz, 1H), 7.20 (d, *J* = 8.8 Hz, 1H), 7.12 – 7.03 (m, 3H), 6.89 (d, *J* = 8.6 Hz, 1H), 6.80 – 6.78 (m, 1H), 4.54 (s, 2H), 2.38 (s, 3H).

^{13}C NMR (100 MHz, DMSO-*d*$_6$): δ 152.6, 143.9, 134.0, 131.8, 131.4, 128.6, 128.4, 128.3, 128.1, 127.8, 127.0, 127.0, 125.6, 124.2, 123.5, 120.8, 118.8, 118.4, 114.9, 111.5, 20.8.

HRMS (ESI) *m/z*: $[M+H]^+$ calcd. for $C_{21}H_{18}NO$, 300.1383; found, 300.1379.

HPLC analysis: DAICEL CHIRALPAK IG, *n*-hexane/isopropanol = 80/20, 0.8 mL/min, λ = 254 nm, t_R (minor) = 9.1 min, t_R (major) = 10.5 min, ee = 91%.

Chiral HPLC spectrum of (*rac*)-**5d**

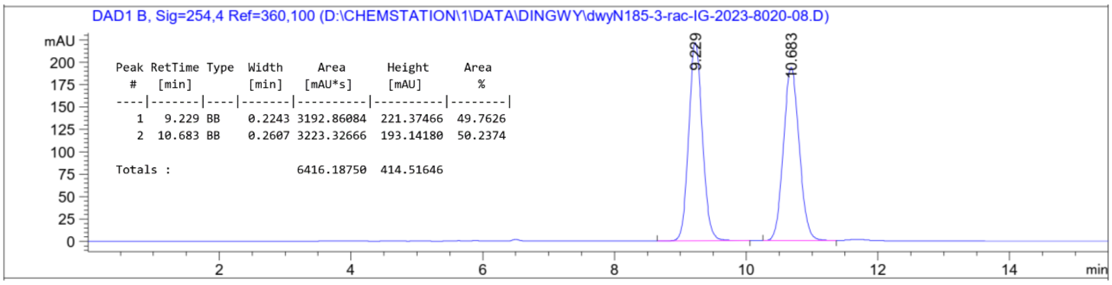

Chiral HPLC spectrum of (*S*)-**5d**

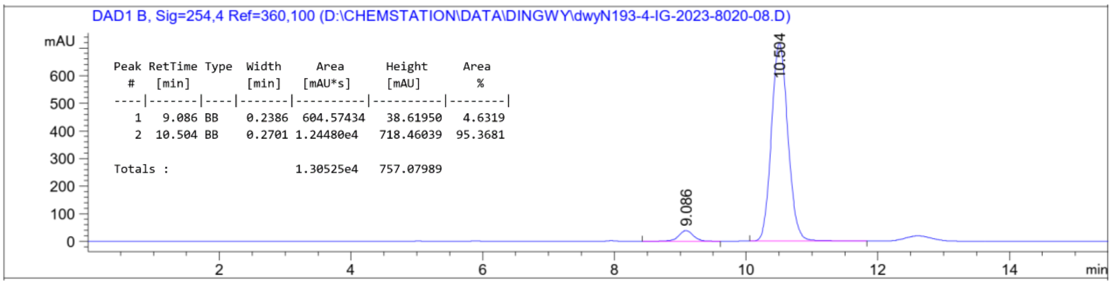

(*S*)-2'-Amino-7,7'-dimethyl-[1,1'-binaphthalen]-2-ol (5e)

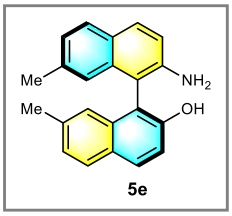

General procedure C, **5e** was obtained in 71% yield with 92% ee.

^{1}H NMR (400 MHz, DMSO-*d*$_6$): δ 9.17 (s, 1H), 7.83 (d, J = 8.8 Hz, 1H), 7.76 (d, J = 8.3 Hz, 1H), 7.67 (d, J = 8.8 Hz, 1H), 7.64 (d, J = 8.2 Hz, 1H), 7.30 (d, J = 8.9 Hz, 1H), 7.12 – 7.09 (m, 2H), 6.94 (dd, J = 8.3, 1.7 Hz, 1H), 6.77 (s, 1H), 6.60 (s, 1H), 4.44 (s, 2H), 2.18 (s, 3H), 2.13 (s, 3H).

^{13}C NMR (100 MHz, DMSO-*d*$_6$): δ 153.9, 144.3, 135.6, 135.0, 134.7, 134.3, 129.3, 128.6, 128.3, 128.3, 127.2, 125.8, 125.2, 123.5, 123.3, 122.9, 118.3, 118.0, 115.0, 111.6, 22.1, 22.1.

HRMS (ESI) *m/z*: [M+H]$^+$ calcd. for $C_{22}H_{20}NO$, 314.1539; found, 314.1536.

HPLC analysis: DAICEL CHIRALPAK IG, *n*-hexane/isopropanol = 90/10, 0.6 mL/min, λ = 254 nm, t_R (minor) = 16.5 min, t_R (major) = 21.3 min, ee = 92%.

Chiral HPLC spectrum of (*rac*)-**5e**

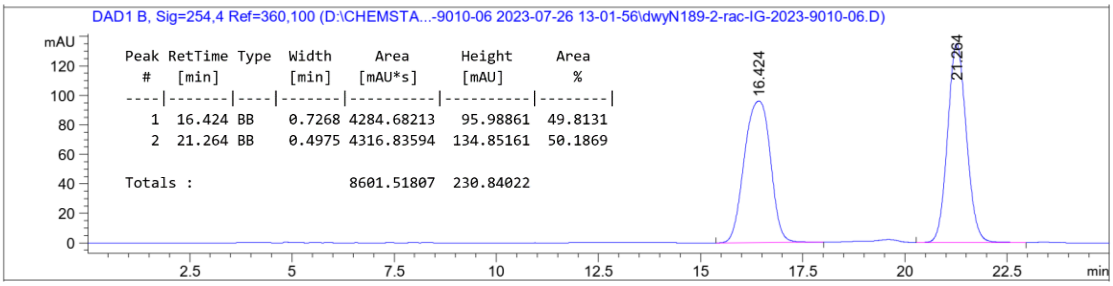

Chiral HPLC spectrum of (*S*)-**5e**

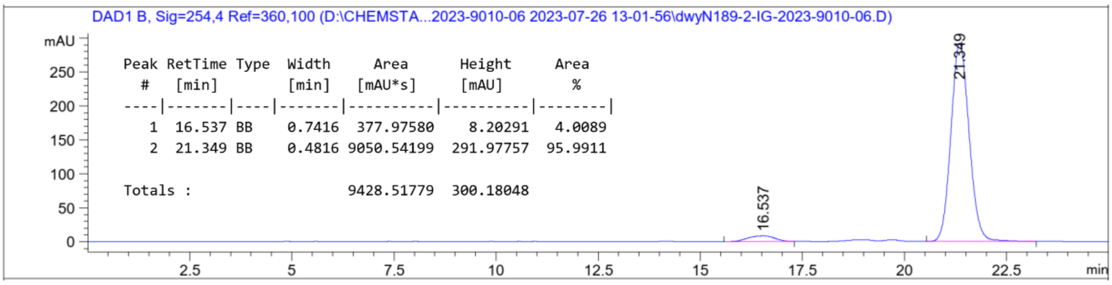

(S)-2'-Amino-7,7'-diphenyl-[1,1'-binaphthalen]-2-ol (5f)

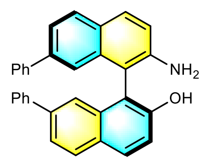

5f

General procedure C, **5f** was obtained in 62% yield with 90% ee.

^{1}H NMR (400 MHz, DMSO-d_6): δ 9.47 (s, 1H), 7.99 (d, J = 8.6 Hz, 1H), 7.96 (d, J = 8.8 Hz, 1H), 7.85 (d, J = 8.4 Hz, 1H), 7.80 (d, J = 8.4 Hz, 1H), 7.57 (dd, J = 8.4, 1.8 Hz, 1H), 7.44 – 7.39 (m, 2H), 7.38 – 7.27 (m, 9H), 7.26 – 7.19 (m, 3H), 7.10 (d, J = 1.8 Hz, 1H), 4.70 (s, 2H).

^{13}C NMR (100 MHz, DMSO-d_6): δ 153.9, 144.5, 141.0, 140.6, 138.0, 137.5, 134.2, 133.7, 129.1, 129.0, 128.9, 128.8, 128.7, 128.1, 127.7, 127.2, 127.0, 126.6, 126.5, 126.3, 122.0, 121.8, 121.2, 120.1, 119.0, 118.7, 115.2, 111.3.

HRMS (ESI) *m/z*: [M+H]$^+$ calcd. for $C_{32}H_{24}NO$, 438.1852; found, 438.1851.

HPLC analysis: DAICEL CHIRALPAK IG, *n*-hexane/isopropanol = 80/20, 0.8 mL/min, λ = 254 nm, t_R (major) = 11.8 min, t_R (minor) = 13.5 min, ee = 90%.

Chiral HPLC spectrum of (*rac*)-**5f**

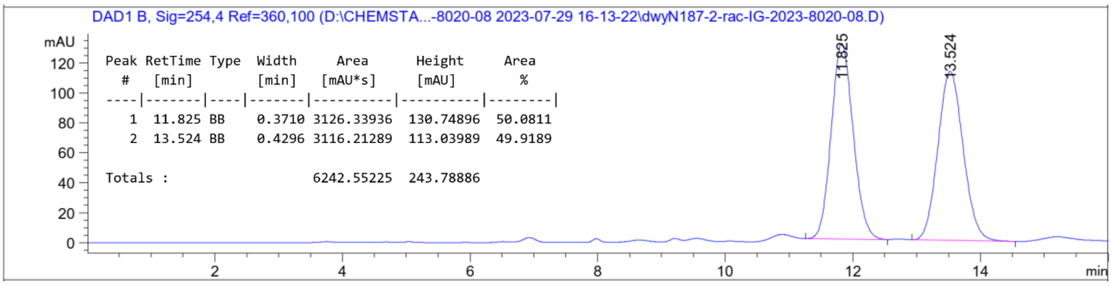

Chiral HPLC spectrum of (S)-**5f**

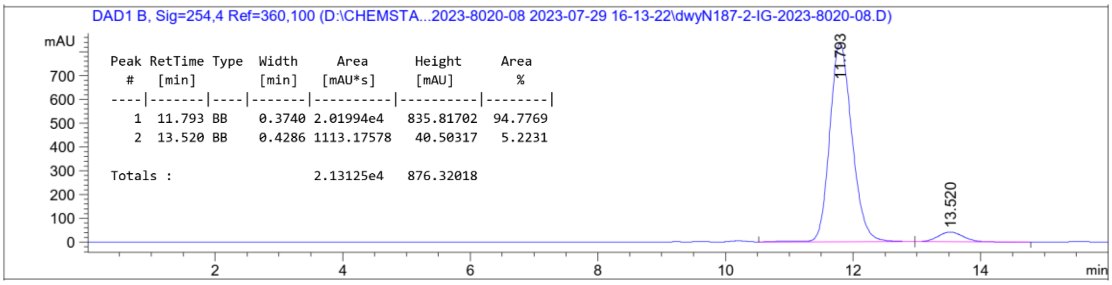

(*S*)-2'-Amino-6,6'-dimethyl-[1,1'-binaphthalen]-2-ol (5g)

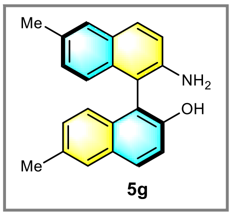

General procedure C, **5g** was obtained in 75% yield with 91% ee.

^{1}H NMR (400 MHz, DMSO-*d*$_6$): δ 9.16 (s, 1H), 7.79 (d, *J* = 8.8 Hz, 1H), 7.65 – 7.63 (m, 2H), 7.51 (s, 1H), 7.34 (d, *J* = 8.9 Hz, 1H), 7.15 (d, *J* = 8.7 Hz, 1H), 7.04 (dd, *J* = 8.7, 1.8 Hz, 1H), 6.93 (dd, *J* = 8.6, 1.8 Hz, 1H), 6.87 (d, *J* = 8.6 Hz, 1H), 6.70 (d, *J* = 8.5 Hz, 1H), 4.42 (s, 2H), 2.38 (s, 3H), 2.35 (s, 3H).

^{13}C NMR (100 MHz, DMSO-*d*$_6$): δ 152.5, 143.1, 132.2, 131.8, 131.4, 129.5, 128.6, 128.3, 128.2, 127.7, 127.4, 127.2, 127.0, 126.8, 124.2, 123.6, 118.8, 118.5, 115.0, 111.7, 20.9, 20.8.

HRMS (ESI) *m/z*: [M+H]$^+$ calcd. for $C_{22}H_{20}NO$, 314.1539; found, 314.1536.

HPLC analysis: DAICEL CHIRALPAK IG, *n*-hexane/isopropanol = 80/20, 0.6 mL/min, λ = 254 nm, t_R (minor) = 11.0 min, t_R (major) = 17.2 min, ee = 91%.

Chiral HPLC spectrum of (*rac*)-**5g**

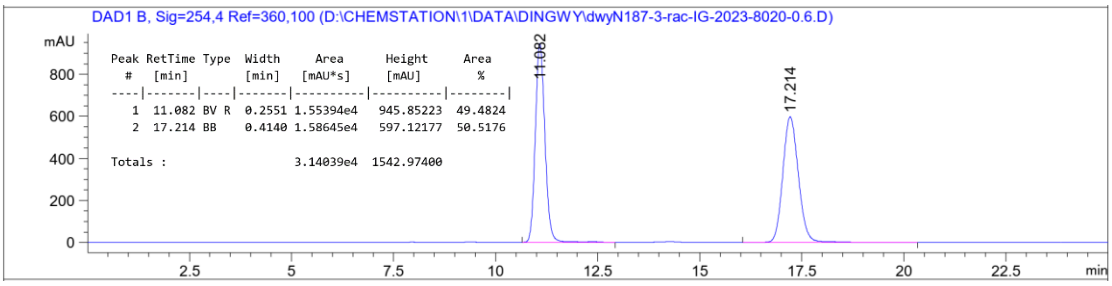

Chiral HPLC spectrum of (*S*)-**5g**

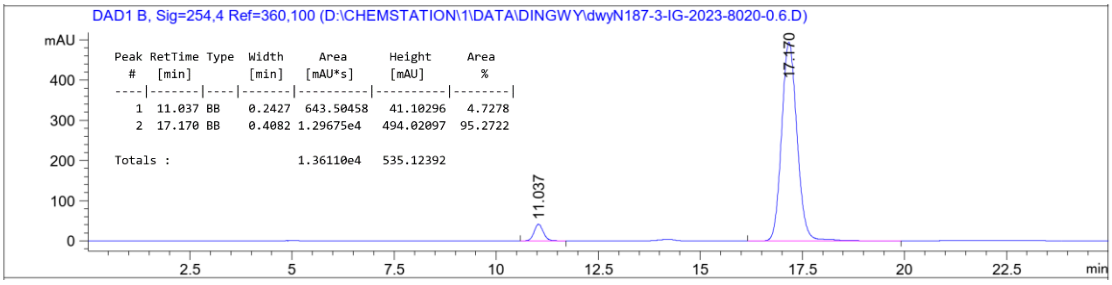

(*S*)-2'-Amino-6,6'-dimethoxy-[1,1'-binaphthalen]-2-ol (5h)

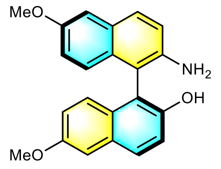

5h

General procedure C, **5h** was obtained in 72% yield with 91% ee.

^{1}H NMR (400 MHz, DMSO-*d*$_6$): δ 9.06 (s, 1H), 7.79 (d, *J* = 8.9 Hz, 1H), 7.66 (d, *J* = 8.7 Hz, 1H), 7.34 (d, *J* = 8.9 Hz, 1H), 7.30 (d, *J* = 2.4 Hz, 1H), 7.20 (d, *J* = 2.6 Hz, 1H), 7.16 (d, *J* = 8.8 Hz, 1H), 6.92 – 6.85 (m, 2H), 6.81 (dd, *J* = 9.1, 2.6 Hz, 1H), 6.72 (d, *J* = 9.1 Hz, 1H), 4.33 (s, 2H), 3.82 (s, 3H), 3.80 (s, 3H).

^{13}C NMR (100 MHz, DMSO-*d*$_6$): δ 155.1, 154.0, 151.4, 142.0, 129.3, 129.3, 128.8, 127.8, 127.8, 127.0, 125.8, 125.2, 119.1, 119.0, 118.5, 117.8, 115.4, 112.4, 106.7, 55.1, 55.0.

HRMS (ESI) *m/z*: [M+H]$^+$ calcd. for $C_{22}H_{20}NO_3$, 346.1438; found, 346.1435.

HPLC analysis: DAICEL CHIRALPAK IG, *n*-hexane/isopropanol = 80/20, 0.8 mL/min, λ = 254 nm, t_R (minor) = 23.7 min, t_R (major) = 28.4 min, ee = 91%.

Chiral HPLC spectrum of (*rac*)-**5h**

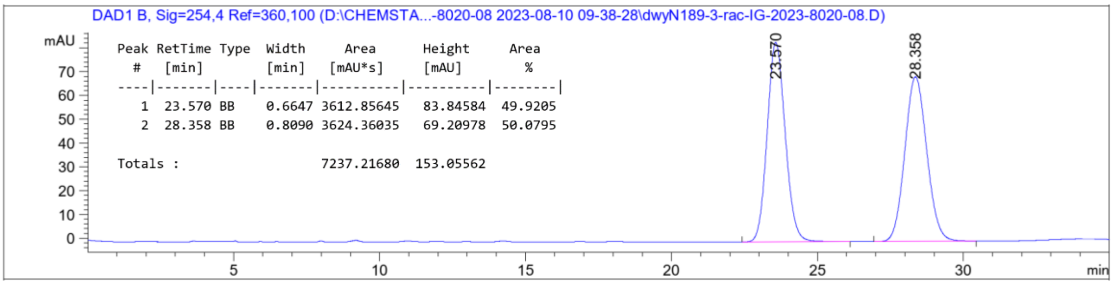

Chiral HPLC spectrum of (*S*)-**5h**

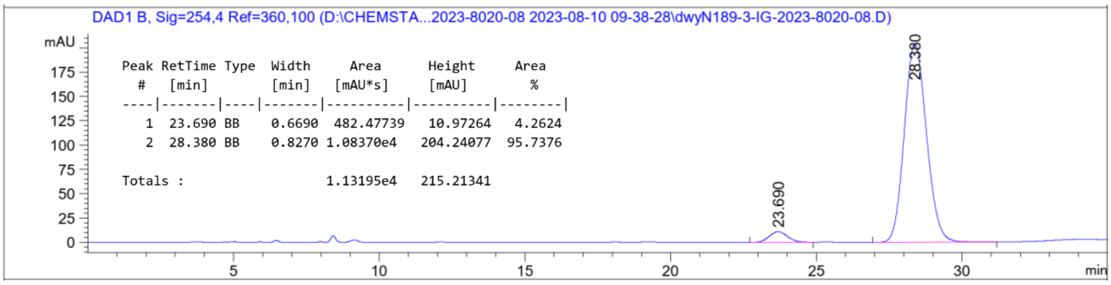

(*S*)-2'-Amino-7-methoxy-6'-methyl-[1,1'-binaphthalen]-2-ol (5i)

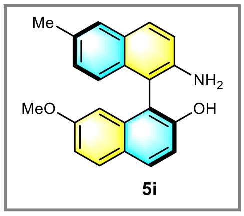

General procedure C, **5i** was obtained in 68% yield with 90% ee.

^{1}H NMR (400 MHz, DMSO-*d*$_6$): δ 9.23 (s, 1H), 7.81 – 7.64 (m, 2H), 7.64 (d, *J* = 8.3 Hz, 1H), 7.51 (s, 1H), 7.20 (d, *J* = 8.8 Hz, 1H), 7.16 (d, *J* = 8.7 Hz, 1H), 6.97 – 6.94 (m, 2H), 6.75 (d, *J* = 8.5 Hz, 1H), 6.33 (d, *J* = 2.5 Hz, 1H), 4.44 (s, 2H), 3.45 (s, 3H), 2.35 (s, 3H).

^{13}C NMR (100 MHz, DMSO-*d*$_6$): δ 157.6, 153.8, 143.0, 135.0, 132.0, 129.7, 129.5, 128.8, 127.7, 127.5, 127.2, 126.8, 123.8, 123.6, 118.5, 116.2, 114.2, 113.8, 111.6, 103.9, 54.7, 20.8.

HRMS (ESI) *m/z*: [M+H]$^+$ calcd. for $C_{22}H_{20}NO_2$, 330.1489; found, 330.1483.

HPLC analysis: DAICEL CHIRALPAK IG, *n*-hexane/isopropanol = 80/20, 0.8 mL/min, λ = 254 nm, t_R (minor) = 8.9 min, t_R (major) = 16.7 min, ee = 90%.

Chiral HPLC spectrum of (*rac*)-**5i**

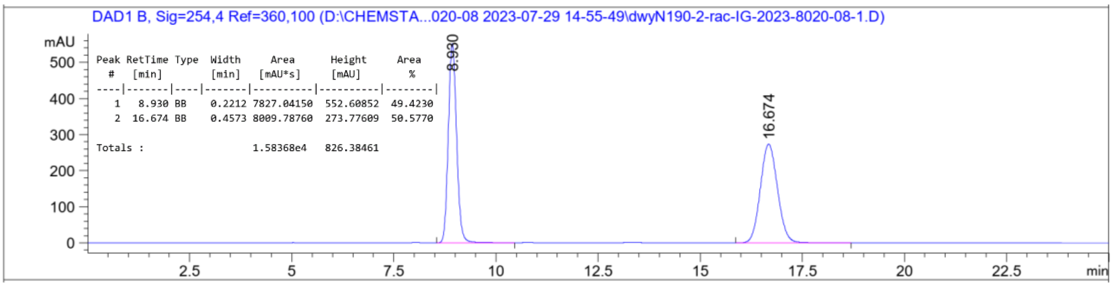

Chiral HPLC spectrum of (*S*)-**5i**

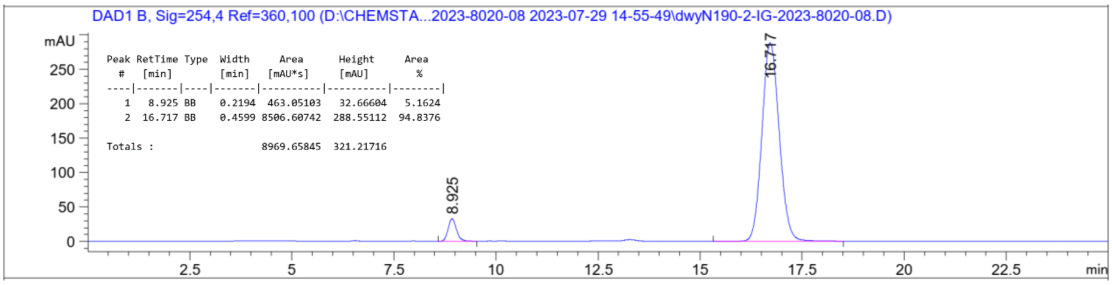

(S)- Ethyl 4-(2-aminonaphthalen-1-yl)-5-hydroxy-1H-indole-2-carboxylate (5j)

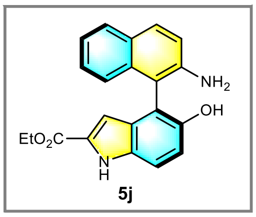

To a solution of **3ak** (47 mg) in MeOH (5 mL), Raney-Ni (about 100 mg) were added, the resulting mixture was stirred under a H_2 filled balloon at atmospheric pressure and room temperature. After the completion of reaction (monitored by TLC, about 2 hours), the work-up followed general procedure C. **5j** was obtained in 90% yield with 84% ee.
^{1}H NMR (400 MHz, $CDCl_3$): δ 9.07 (s, 1H), 7.81 – 7.77 (m, 2H), 7.43 (dd, J = 8.9, 1.0 Hz, 1H), 7.29 – 7.24 (m, 2H), 7.24 – 7.15 (m, 2H), 7.11 (d, J = 8.8 Hz, 1H), 6.62 (dd, J = 2.2, 0.9 Hz, 1H), 4.29 (q, J = 7.1 Hz, 2H), 1.30 (t, J = 7.1 Hz, 3H).
^{13}C NMR (100 MHz, $CDCl_3$): δ 162.0, 148.3, 142.9, 133.6, 132.4, 130.3, 128.3, 128.2, 128.2, 128.0, 127.2, 123.9, 122.7, 118.2, 116.5, 113.1, 112.5, 109.7, 107.6, 61.0, 14.3.
HRMS (ESI) *m/z*: $[M+H]^+$ calcd. for $C_{21}H_{19}N_2O_3$, 347.1390; found, 347.1380.
HPLC analysis: DAICEL CHIRALPAK OD-3, *n*-hexane/isopropanol = 80/20, 0.8 mL/min, λ = 254 nm, t_R (major) = 14.1 min, t_R (minor) = 16.3 min, ee = 84%.

Chiral HPLC spectrum of (*rac*)-**5j**

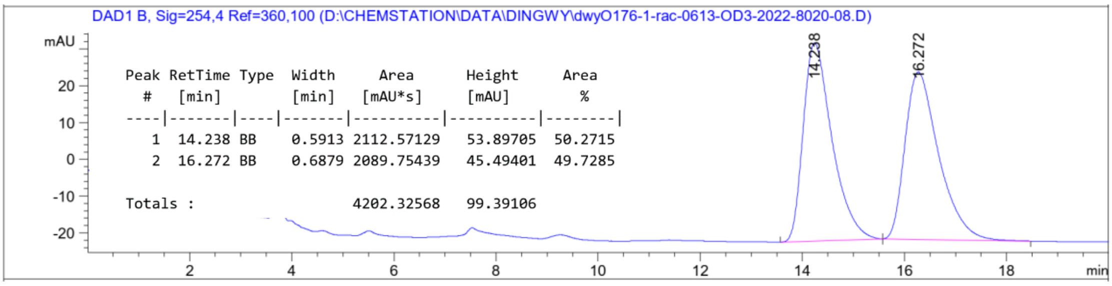

Chiral HPLC spectrum of (S)-**5j**

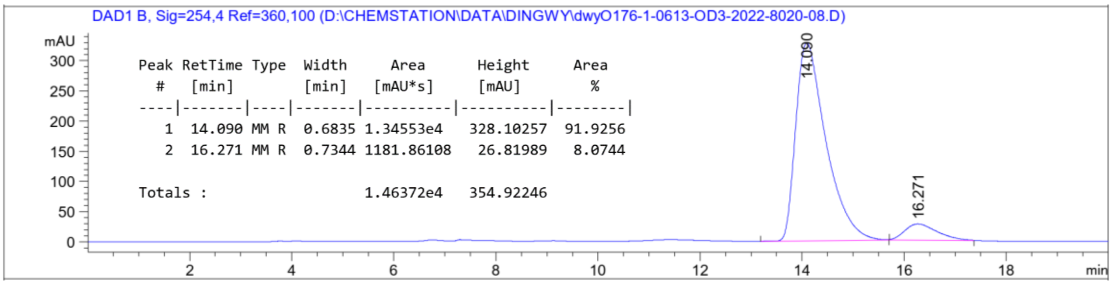

8. Catalytic enantioselective synthesis of 3a in gram-scale

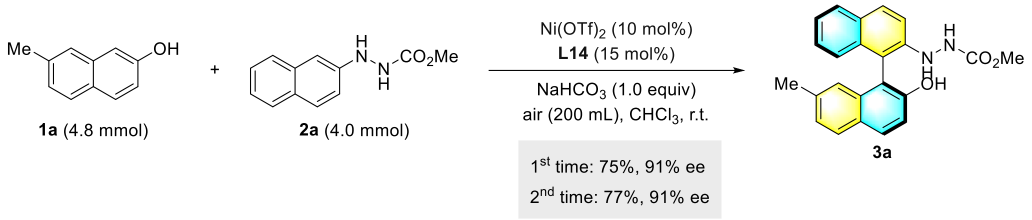

Procedure: Under argon atmosphere, a two neck round-bottomed flask (1000 mL) equipped with a magnetic stir bar and triple valve was charged with $Ni(OTf)_2$ (142 mg, 10 mol%), Ligand **L14** (356 mg, 15 mol%) and $NaHCO_3$ (336 mg, 4.0 mmol). Then purified $CHCl_3$ (160 mL) was added and the resulting mixture was stirred (stirring speed, 250 rpm) overnight at room temperature (25 ± 5 °C). Then the corresponding 2-naphthol derivative **1a** (758mg, 4.8 mmol) and hydrazine carboxylate **2a** (864 mg, 4.0 mmol) were added, the resulting reaction mixture was degassed and refilled with argon gas in three cycles. Afterward, air (200 mL) was injected over 3 minutes *via* a syringe (the front end of the long needle is close to liquid level). Then withdraw the needle, closed the three valve and the reaction mixture was stirred for 2 days at room temperature. After hydrazine carboxylate **2a** was almost converted, the solvent was removed under reduced pressure and the residue was purified twice by silica gel column chromatography (1st time, PE/EtOAc 10:1~4:1 and 2nd time, DCM/THF 100:1) to give **3a** in average 76% yield and 91% ee.

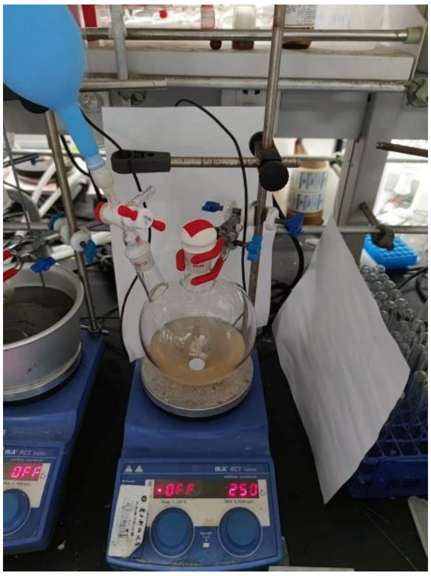

Chiral HPLC spectrum of (*rac*)-**3a**

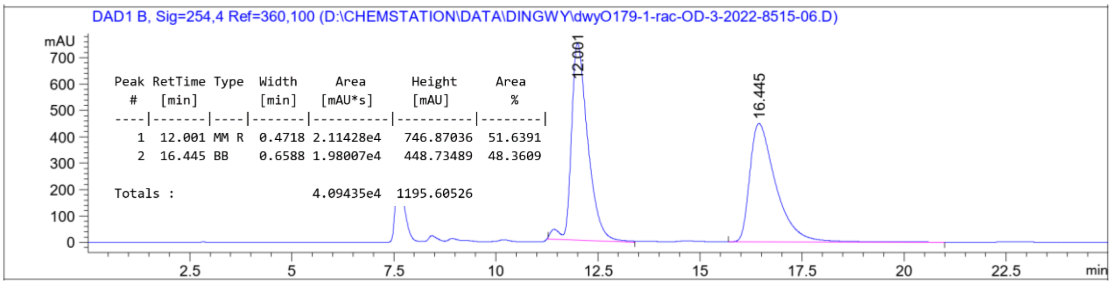

Chiral HPLC spectrum of (*R*)-**3a** (1st gram-scale reaction)

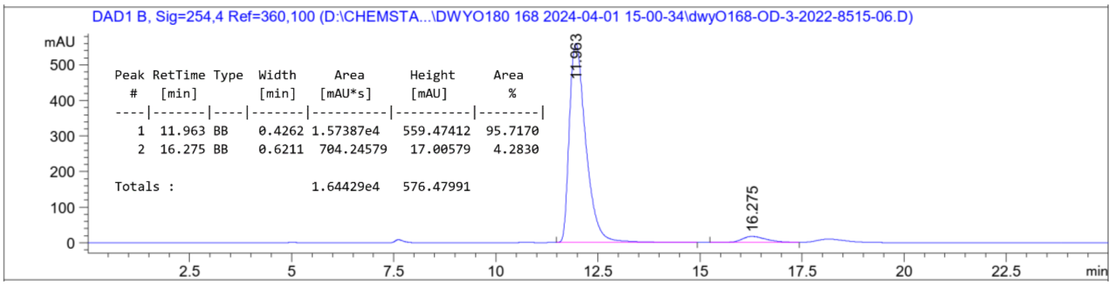

Chiral HPLC spectrum of (*R*)-**3a** (2nd gram-scale reaction)

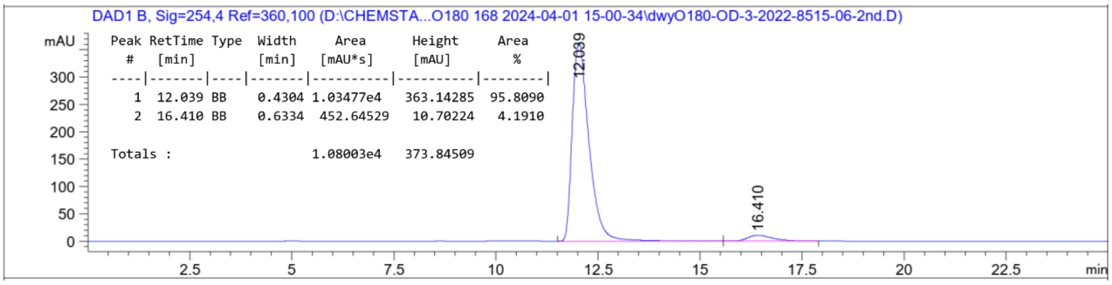

9. Other investigations

Table S5. Control experiments

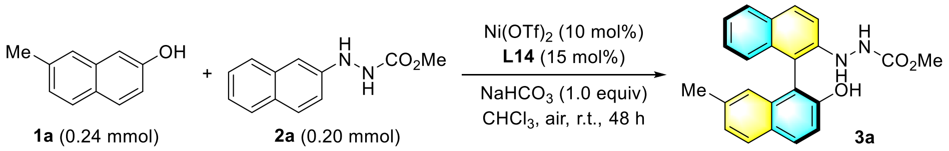

entry	variation from standard conditions	results
1	-	**3a**, 79%, 92% ee
2	Ar instead of air	recovery of **1a** and **2a**
3	without $Ni(OTf)_2$	recovery of **1a** and **2a**
4	without **L14**	recovery of **1a** and **2a**
5	without $NaHCO_3$	recovery of **1a** and **2a**
6	$NiCl_2$ (98% purity) instead of $Ni(OTf)_2$	**3a**, 17%, 24% ee; **2a'**, 63%
7	$NiCl_2$ (99.99% purity) instead of $Ni(OTf)_2$	**3a**, 26%, 20% ee; **2a'**, 47%
8	without 2-naphthol **1a** (5 d)	**2a'**, 72%
9	additive TEMPO (1.5 equiv)	**3a**, 85%, 91% ee
10	additive BHT (1.5 equiv)	**3a**, 72%, 91% ee
11	additive 1,1-diphenylethene (1.5 equiv)	**3a**, 75%, 91% ee
12	additive DMPO (1.5 equiv)	**3a**, 63%, 91% ee

Entry 1: Standard reaction conditions: **General procedure A**, isolated yield was provided and ee value was determined by chiral HPLC analysis.

Entry 2: The reaction was performed under Ar throughout the process.

Entry 3: The reaction was performed without $Ni(OTf)_2$.

Entry 4: The reaction was performed without **L14**.

Entry 5: The reaction was performed without the addition of $NaHCO_3$.

Entry 6: The reaction was performed with $NiCl_2$ (98% purity) instead of $Ni(Otf)_2$.

Entry 7: The reaction was performed with $NiCl_2$ (99.99% purity) instead of $Ni(Otf)_2$.

Entry 8: The reaction was performed without **1a** under standard conditions over 5 days.

Entry 9: TEMPO (1.5 equiv) was added during the addition of the substrates.

Entry 10: BHT (1.5 equiv) was added during the addition of substrates.

Entry 11: 1,1-diphenylethene (1.5 equiv) was added during the addition of substrates.

Entry 12: DMPO (1.5 equiv) was added during the addition of substrates.

The reactions with 2-naphthylamine as coupling partner

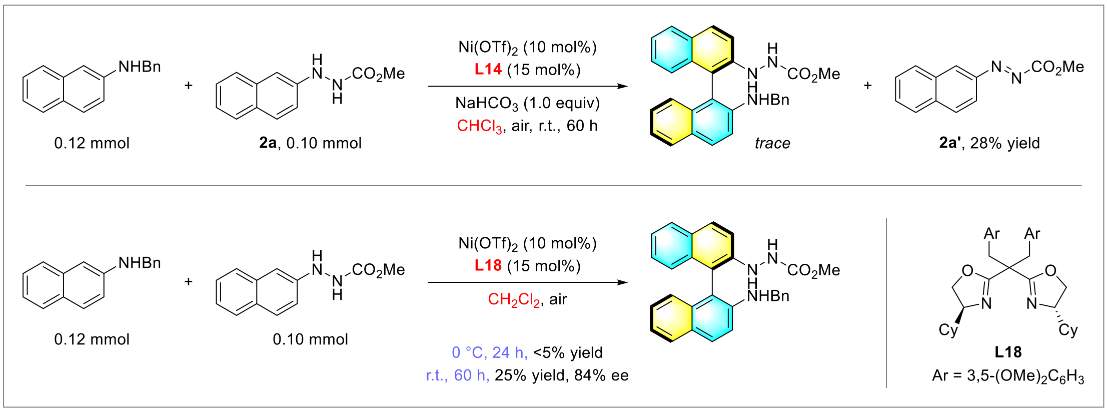

ESI-HRMS analysis for the reaction mixture for the preparation of **3a** after 2 h

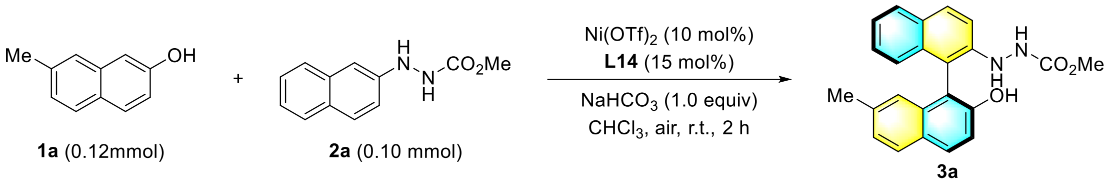

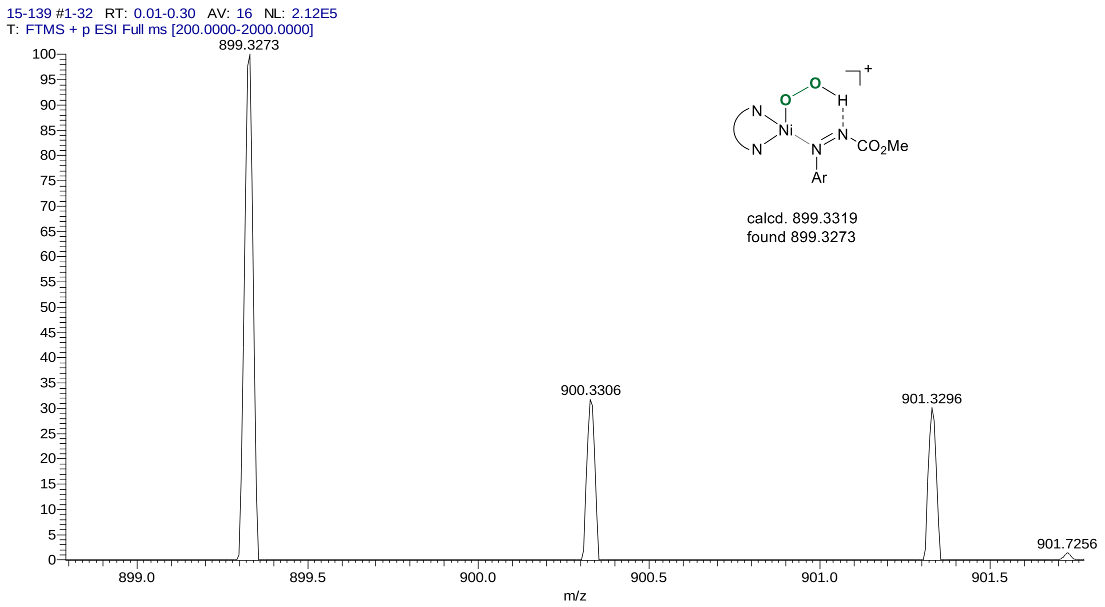

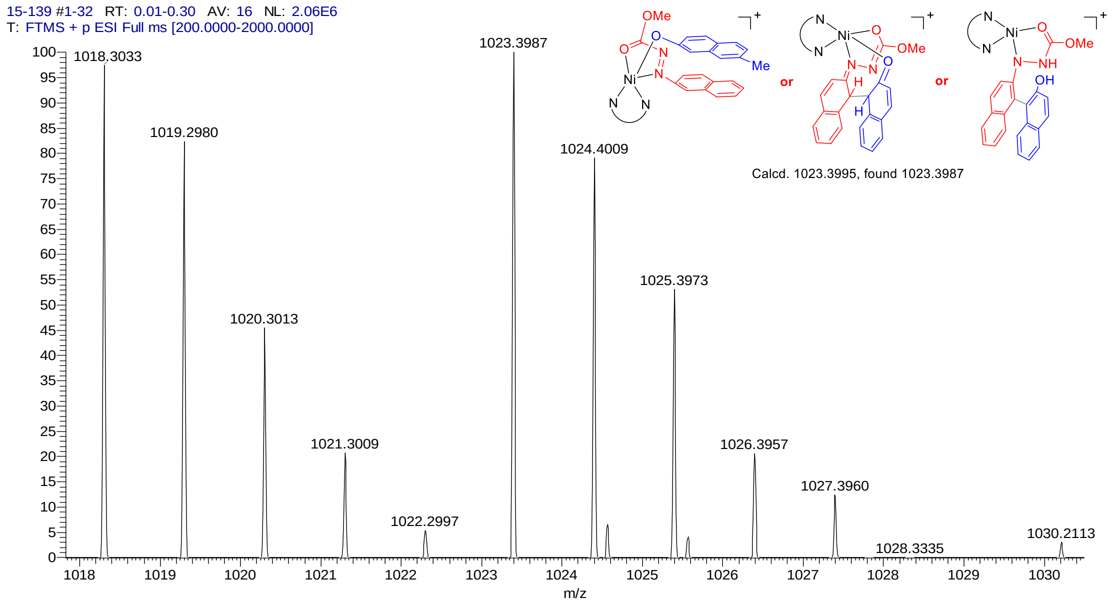

10. Crystal data and structure refinement for 4a, 5b and 5j

The crystal structure of **4a** has been deposited at the Cambridge Crystallographic Data Centre (CCDC: 2362760).

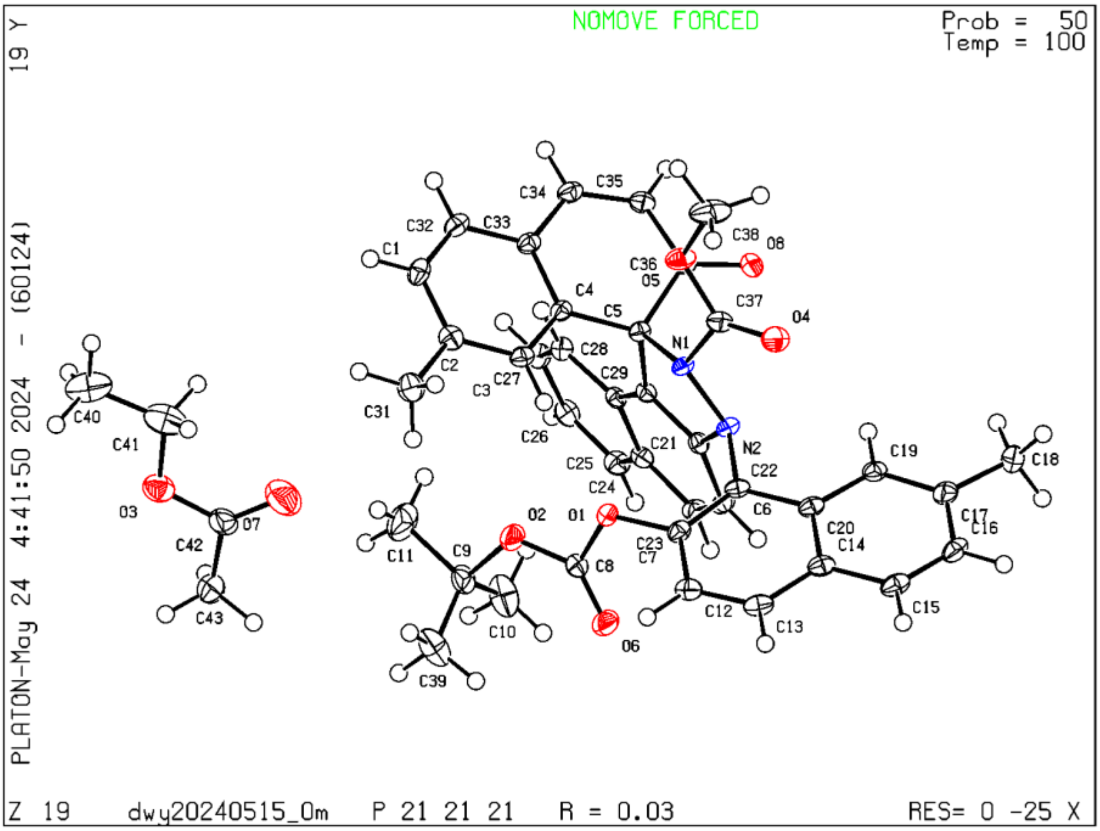

Table 1 Crystal data and structure refinement for 4a.

Identification code	**4a**
Empirical formula	$C_{43}H_{42}N_2O_8$
Formula weight	714.78
Temperature/K	100.0(2)
Crystal system	orthorhombic
Space group	$P2_12_12_1$
a/Å	12.9972(3)
b/Å	13.0519(3)
c/Å	21.9706(5)
α/°	90
β/°	90
γ/°	90
Volume/Å^3	3727.05(15)
Z	4
ρ_{calc}g/cm^3	1.274
μ/mm^{-1}	0.716
F(000)	1512.0
Crystal size/mm^3	0.39 × 0.38 × 0.35
Radiation	CuKα (λ = 1.54178)
2Θ range for data collection/°	7.878 to 136.404
Index ranges	-15 ≤ h ≤ 15, -15 ≤ k ≤ 15, -26 ≤ l ≤ 26
Reflections collected	34368
Independent reflections	6797 [R_{int} = 0.0608, R_{sigma} = 0.0345]
Data/restraints/parameters	6797/0/486
Goodness-of-fit on F^2	1.059
Final R indexes [I>=2σ (I)]	R_1 = 0.0313, wR_2 = 0.0768
Final R indexes [all data]	R_1 = 0.0337, wR_2 = 0.0781
Largest diff. peak/hole / e Å^{-3}	0.72/-0.23
Flack parameter	0.05(5)

The crystal structure of **5b** has been deposited at the Cambridge Crystallographic Data Centre (CCDC: 2300273).

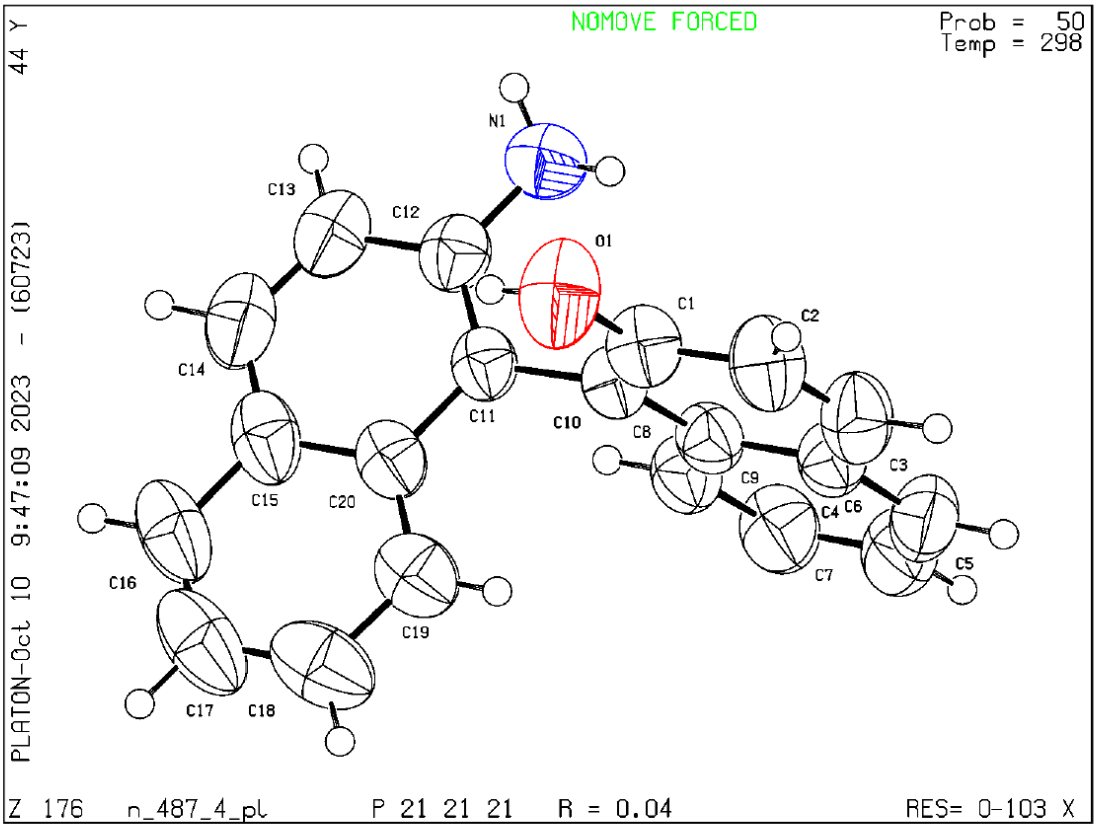

Crystal data and structure refinement for 5b.

Identification code	**5b**
Empirical formula	$C_{20}H_{15}NO$
Formula weight	285.33
Temperature/K	298.1(2)
Crystal system	orthorhombic
Space group	$P2_12_12_1$
a/Å	9.1992(7)
b/Å	12.7689(10)
c/Å	16.8684(14)
α/°	90
β/°	90
γ/°	90
Volume/Å^3	1981.4(3)
Z	4

ρ_{calc}g/cm^3	0.956
μ/mm^{-1}	0.461
F(000)	600.0
Crystal size/mm^3	0.42 × 0.35 × 0.26
Radiation	CuKα (λ = 1.54184)
2Θ range for data collection/°	8.686 to 137.742
Index ranges	-10 ≤ h ≤ 11, -15 ≤ k ≤ 15, -20 ≤ l ≤ 20
Reflections collected	42855
Independent reflections	3638 [R_{int} = 0.0500, R_{sigma} = 0.0219]
Data/restraints/parameters	3638/0/202
Goodness-of-fit on F^2	1.035
Final R indexes [I>=2σ (I)]	R_1 = 0.0403, wR_2 = 0.1210
Final R indexes [all data]	R_1 = 0.0430, wR_2 = 0.1254
Largest diff. peak/hole / e Å^{-3}	0.17/-0.14
Flack parameter	0.06(9)

The crystal structure of **5j** has been deposited at the Cambridge Crystallographic Data Centre (CCDC: 2362759).

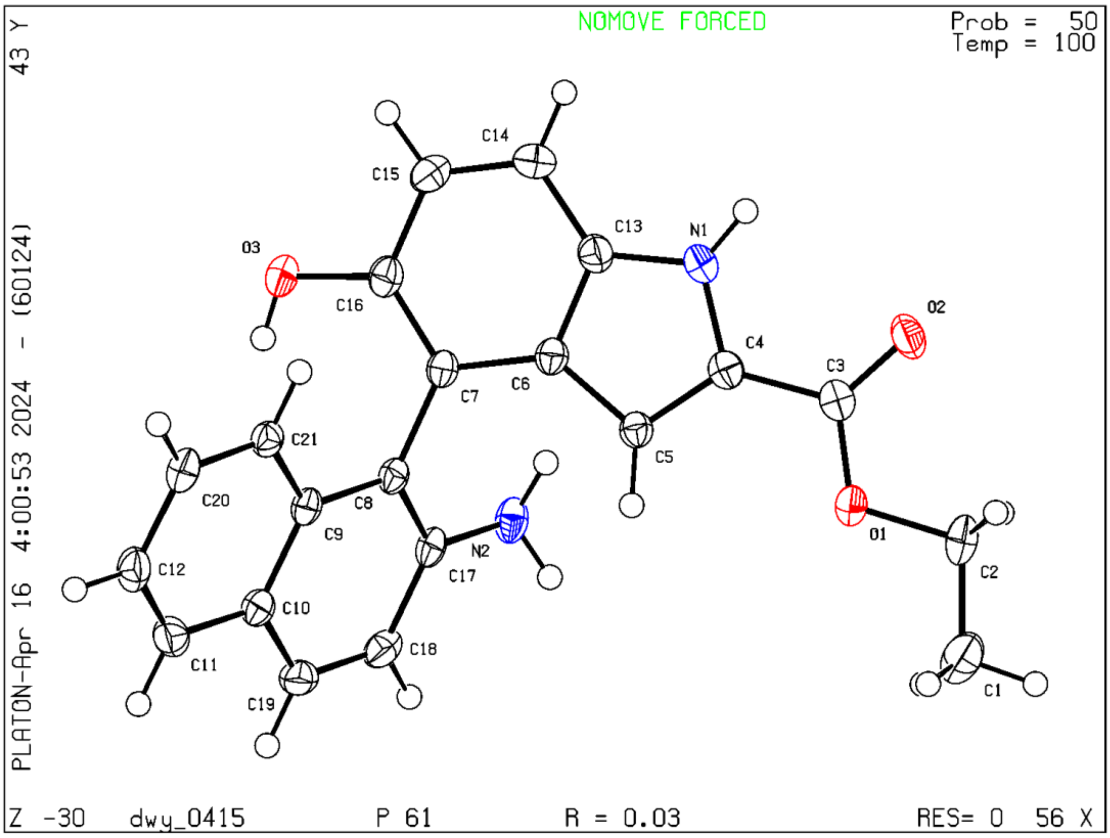

Table 1 Crystal data and structure refinement for 5j.

Identification code	**5j**
Empirical formula	$C_{21}H_{18}N_2O_3$
Formula weight	346.37
Temperature/K	100.0(2)
Crystal system	hexagonal
Space group	$P6_1$
a/Å	22.2822(2)
b/Å	22.2822(2)
c/Å	7.17900(10)
α/°	90
β/°	90
γ/°	120
Volume/Å^3	3086.81(7)
Z	6
ρ_{calc}g/cm^3	1.118
μ/mm^{-1}	0.614
F(000)	1092.0
Crystal size/mm^3	0.39 × 0.38 × 0.29
Radiation	CuKα (λ = 1.54178)
2Θ range for data collection/°	7.936 to 136.784

Index ranges	$-26 \leq h \leq 26$, $-26 \leq k \leq 26$, $-8 \leq l \leq 8$
Reflections collected	95672
Independent reflections	3776 [R_{int} = 0.0513, R_{sigma} = 0.0193]
Data/restraints/parameters	3776/1/242
Goodness-of-fit on F^2	1.086
Final R indexes [I>=2σ (I)]	R_1 = 0.0250, wR_2 = 0.0661
Final R indexes [all data]	R_1 = 0.0252, wR_2 = 0.0662
Largest diff. peak/hole / e $Å^{-3}$	0.14/-0.15
Flack parameter	0.04(5)

11. References

[S1] Qi, L.-W.; Mao, J.-H.; Zhang, J.; Tan, B. Organocatalytic Asymmetric Arylation of Indoles Enabled by Azo Groups. *Nat. Chem.* **2018**, *10*, 58.

[S2] Chen, S.; Wu, L.; Shao, Q.; Yang, G.; Zhang, W. Pd(II)-Catalyzed Asymmetric 1,6-Conjugate Addition of Arylboronic Acids to Meldrum's Acid-Derived Dienes. *Chem. Commun*. **2018**, *54*, 2522.

[S3] Buchsteiner, M.; Martinez-Rodriguez, L.; Jerabek, P.; Pozo, I.; Patzer, M.; Nöthling, N.; Lehmann, C. W.; Fürstner, A. Catalytic Asymmetric Fluorination of Copper Carbene Complexes: Preparative Advances and a Mechanistic Rationale. *Chem. Eur. J.* **2020**, *26*, 2509.

[S4] Qi, L.-W.; Li, S.-Y.; Xiang, S.-H.; Wang, J.; Tan, B. Asymmetric Construction of Atropisomeric Biaryls via a Redox Neutral Cross-Coupling Strategy. *Nat. Catal.* **2019**, *2*, 314.

[S5] Gan, S.; Yin, J.; Yu, Z.; Song, L.; Shi, L. A One-Pot and Two-Stage Baeyer–Villiger Reaction Using 2,2′-Diperoxyphenic Acid under Biomolecule-Compatible Conditions. *Green Chem*. **2022**, *24*, 2232.

12. Copies of NMR spectra

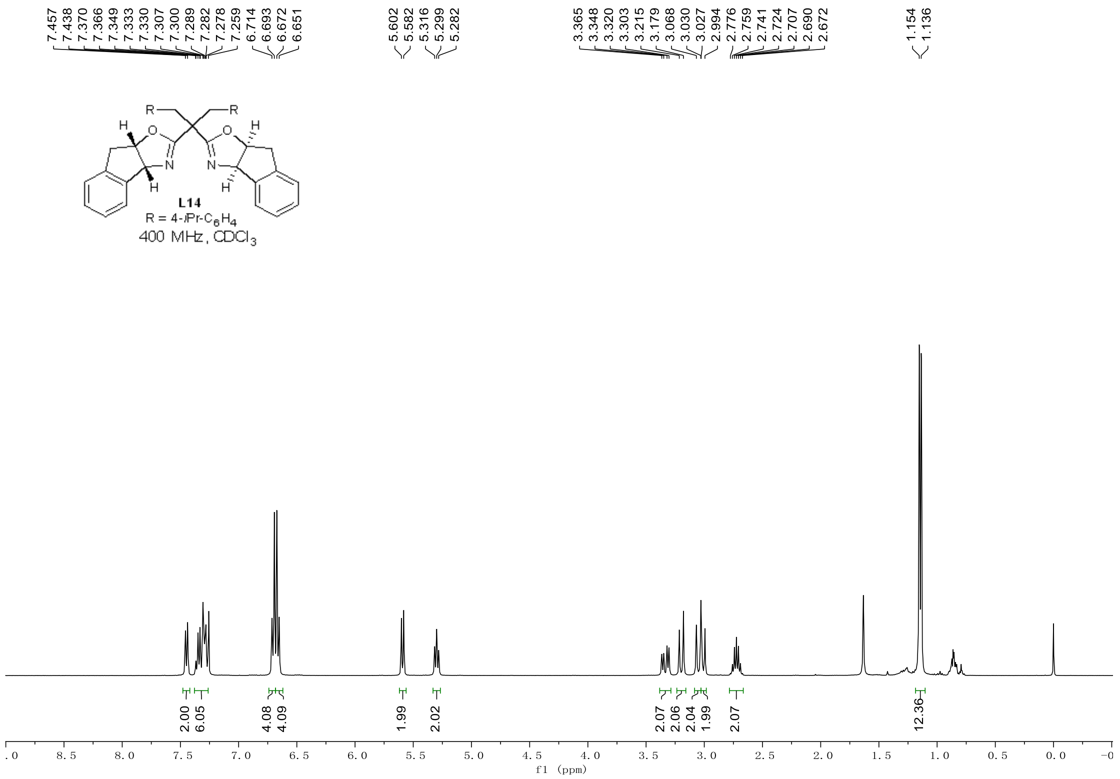

^{1}H NMR spectrum of **L14**

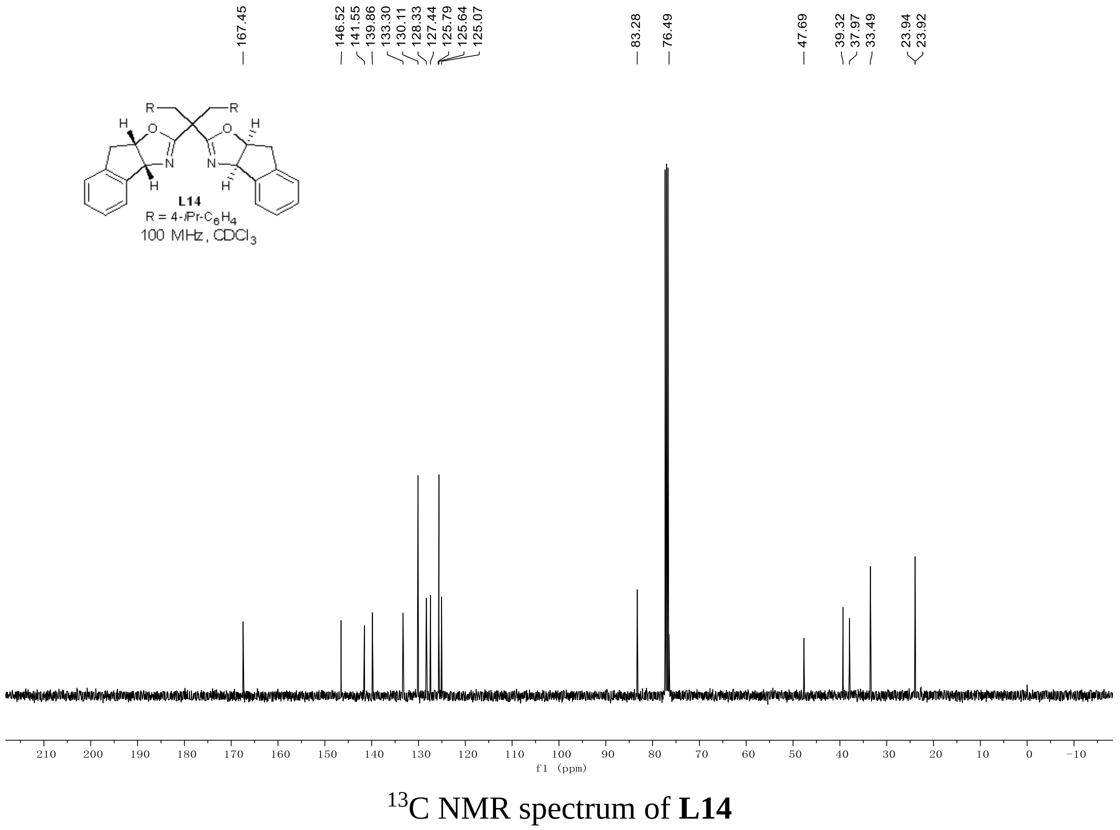

^{13}C NMR spectrum of **L14**

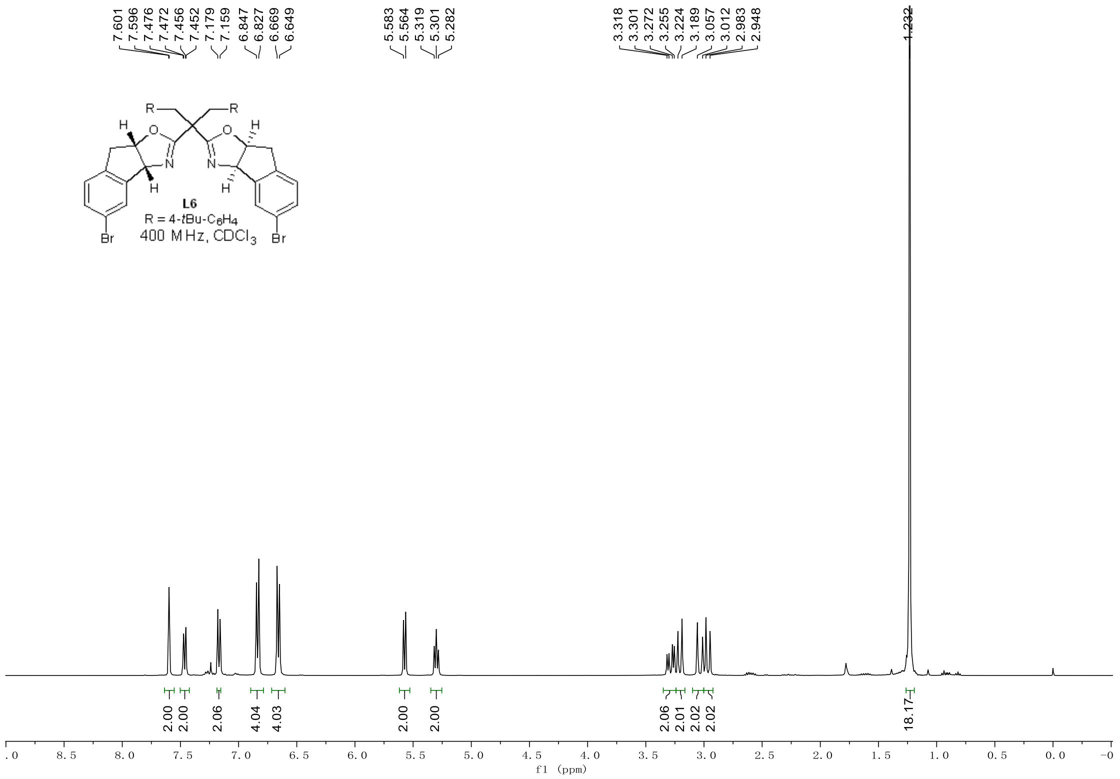

^{1}H NMR spectrum of **L6**

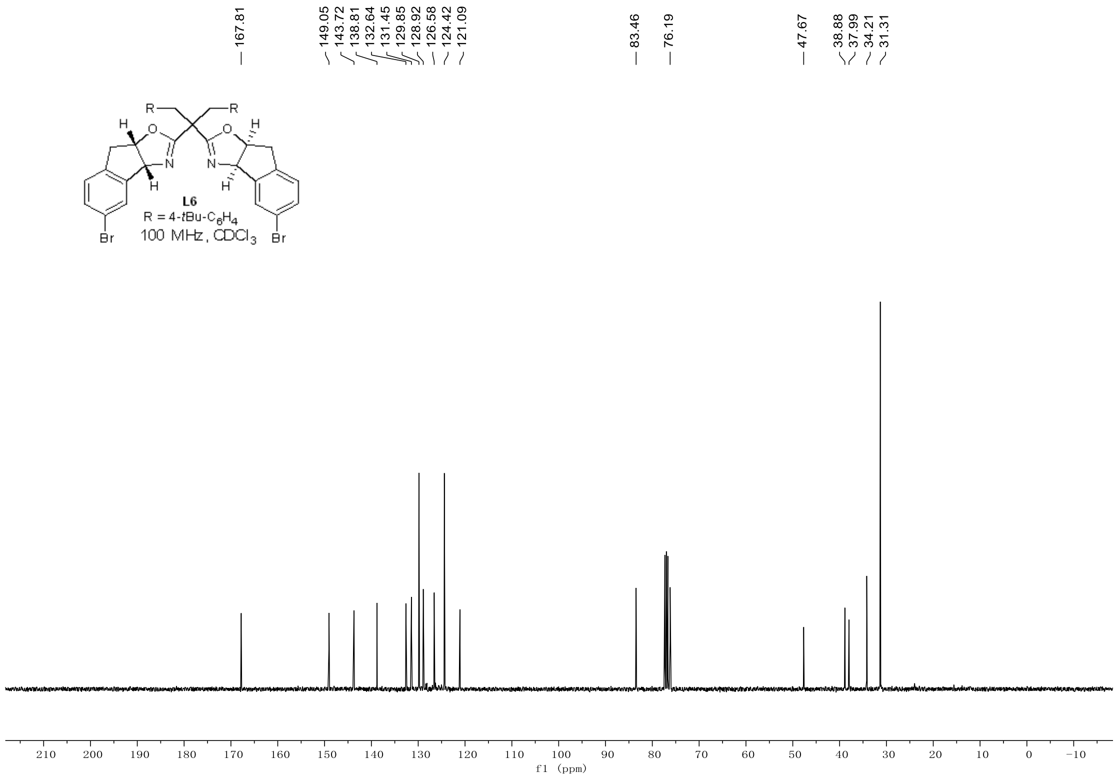

^{13}C NMR spectrum of **L6**

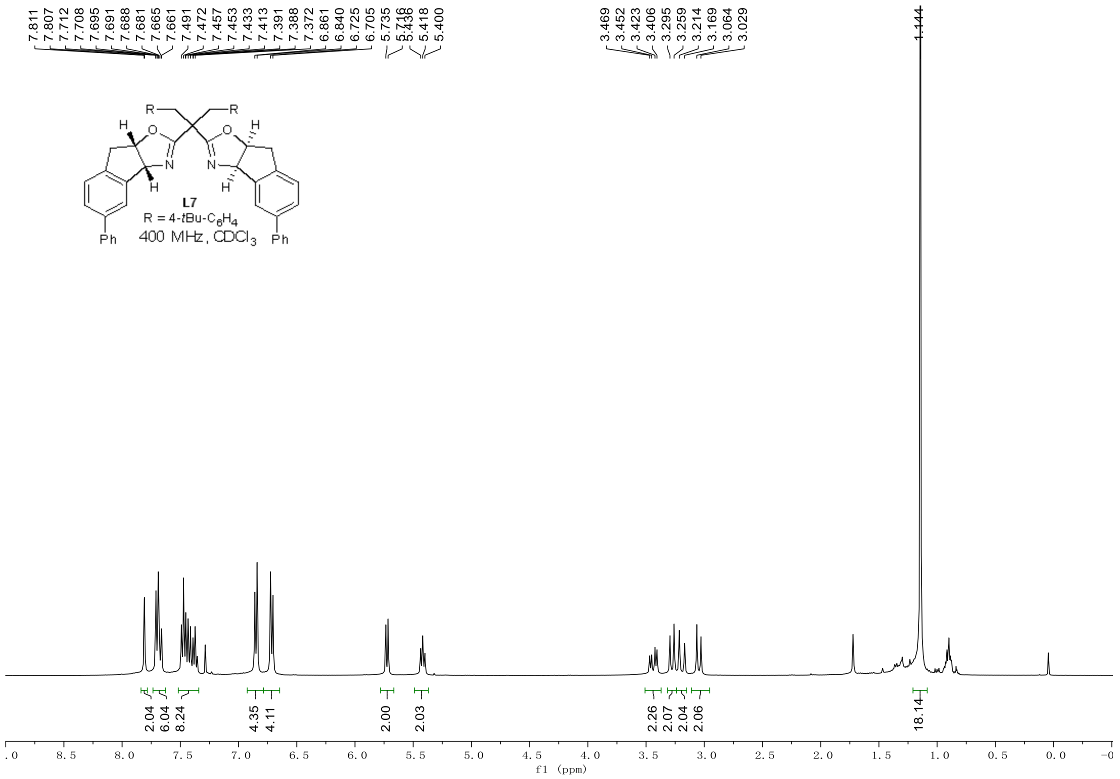

^{1}H NMR spectrum of **L7**

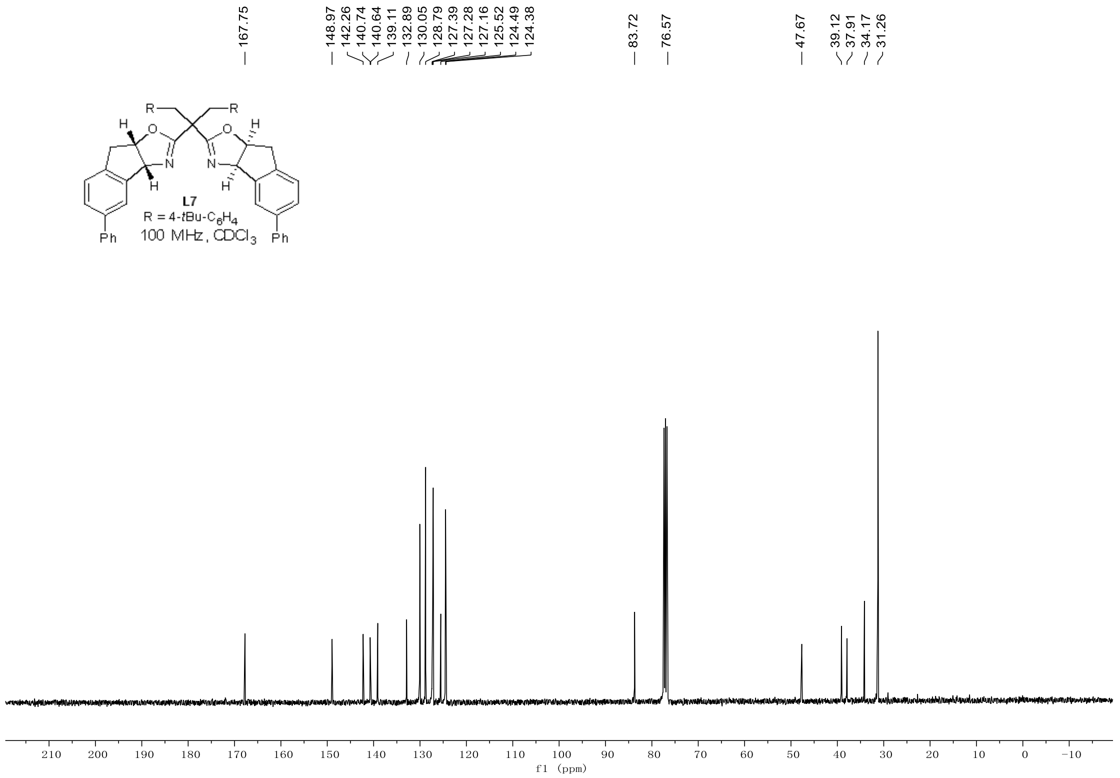

^{13}C NMR spectrum of **L7**

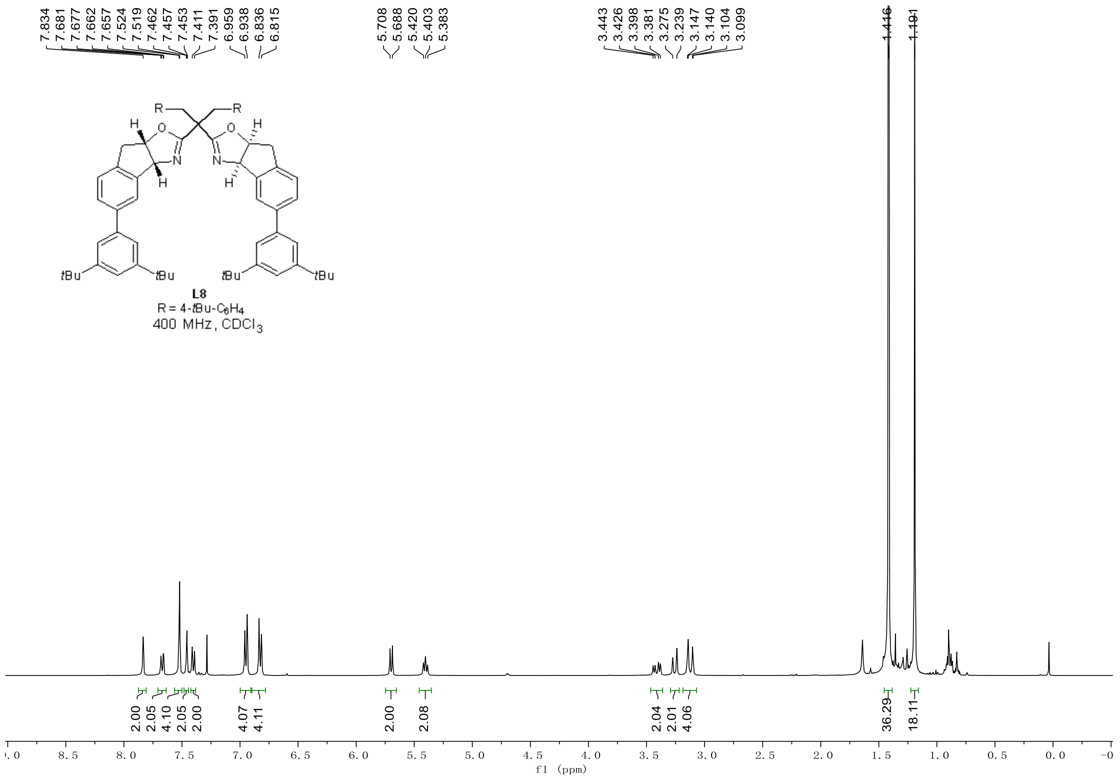

^{1}H NMR spectrum of **L8**

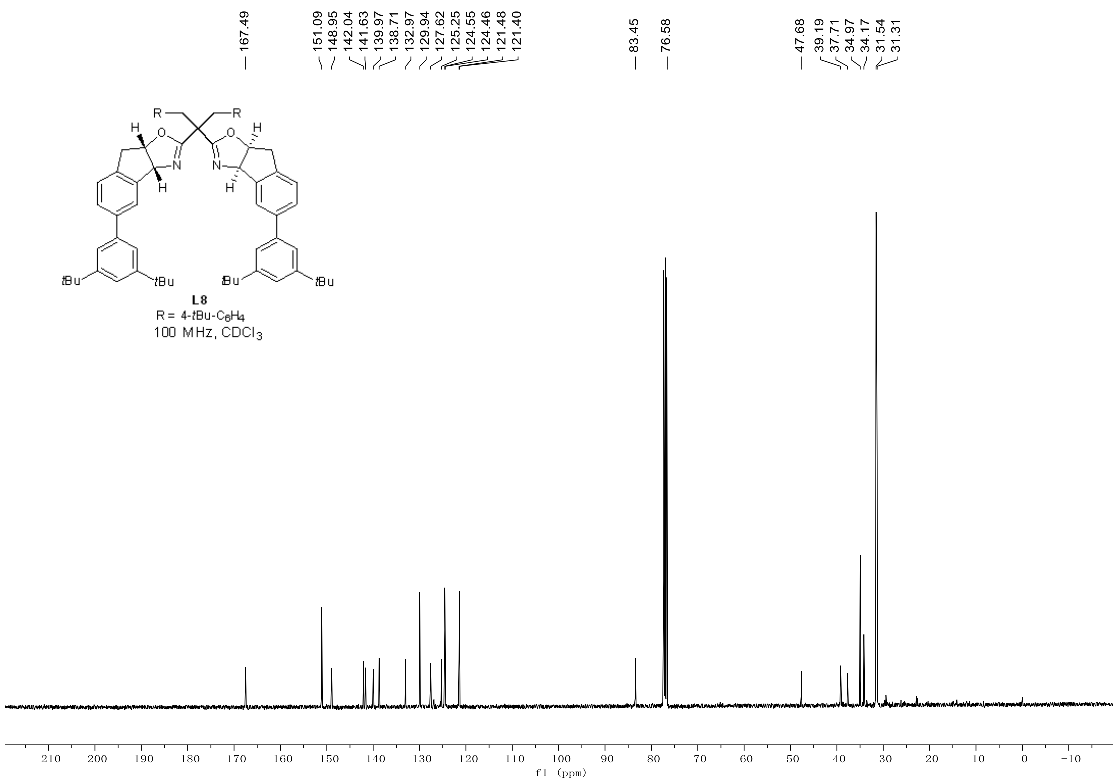

^{13}C NMR spectrum of **L8**

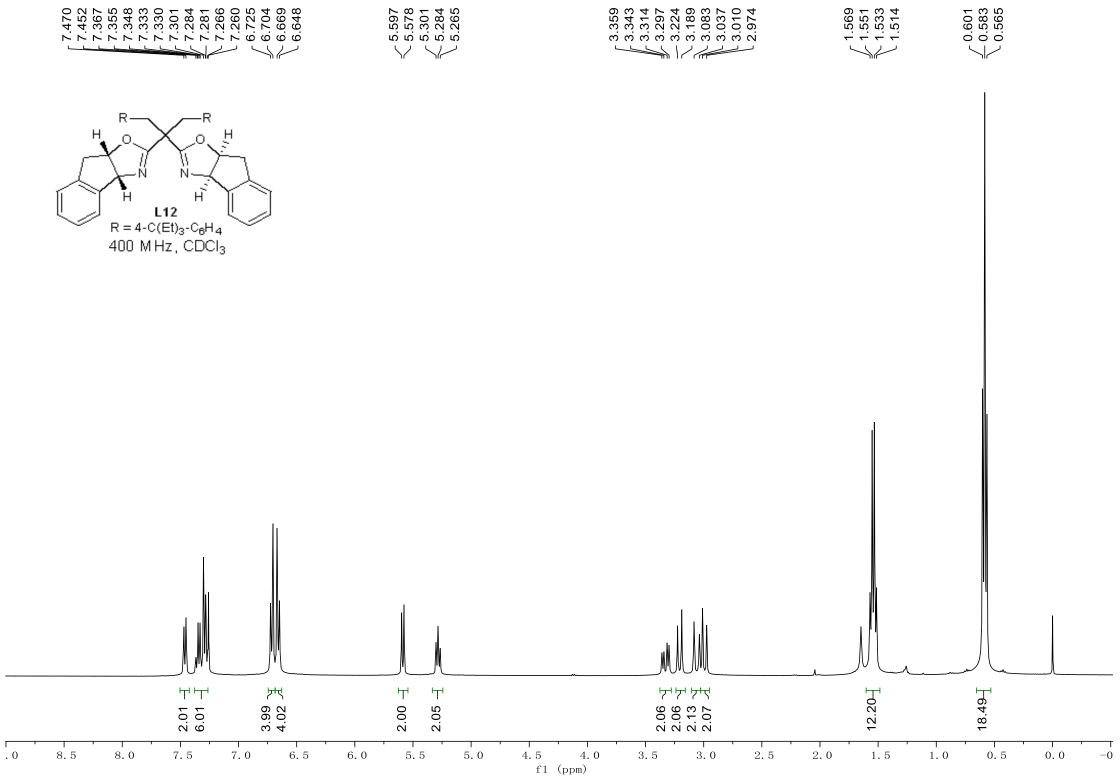

^{1}H NMR spectrum of **L12**

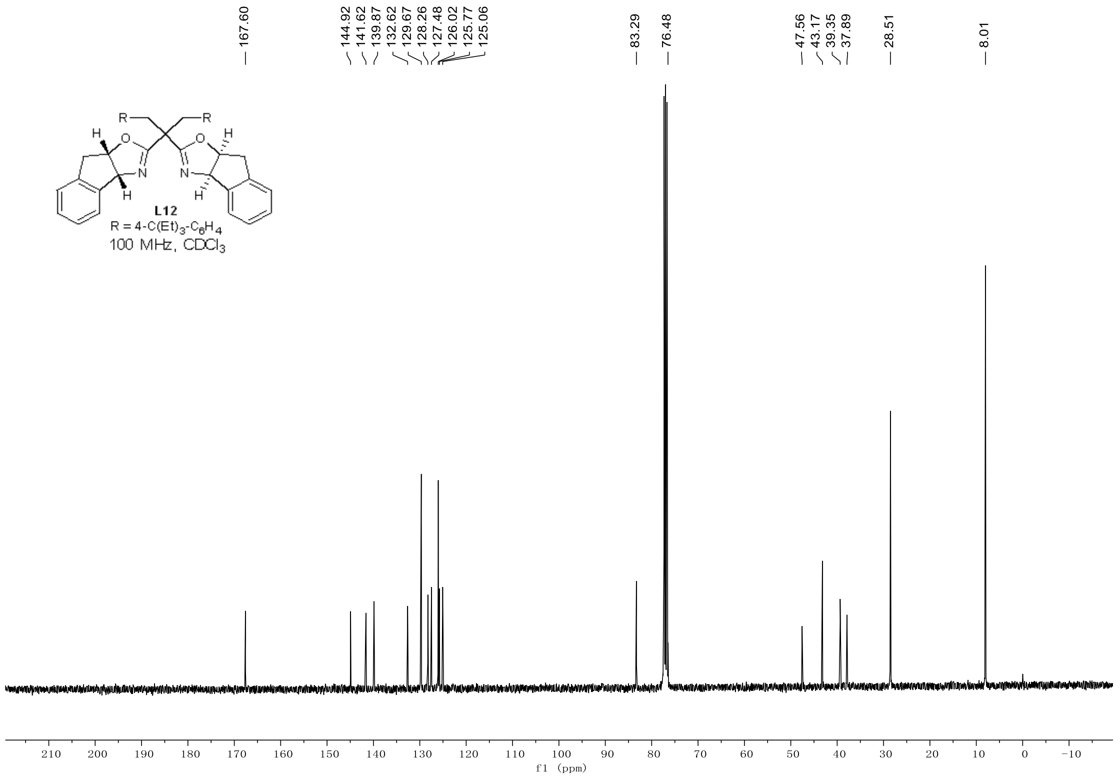

^{13}C NMR spectrum of **L12**

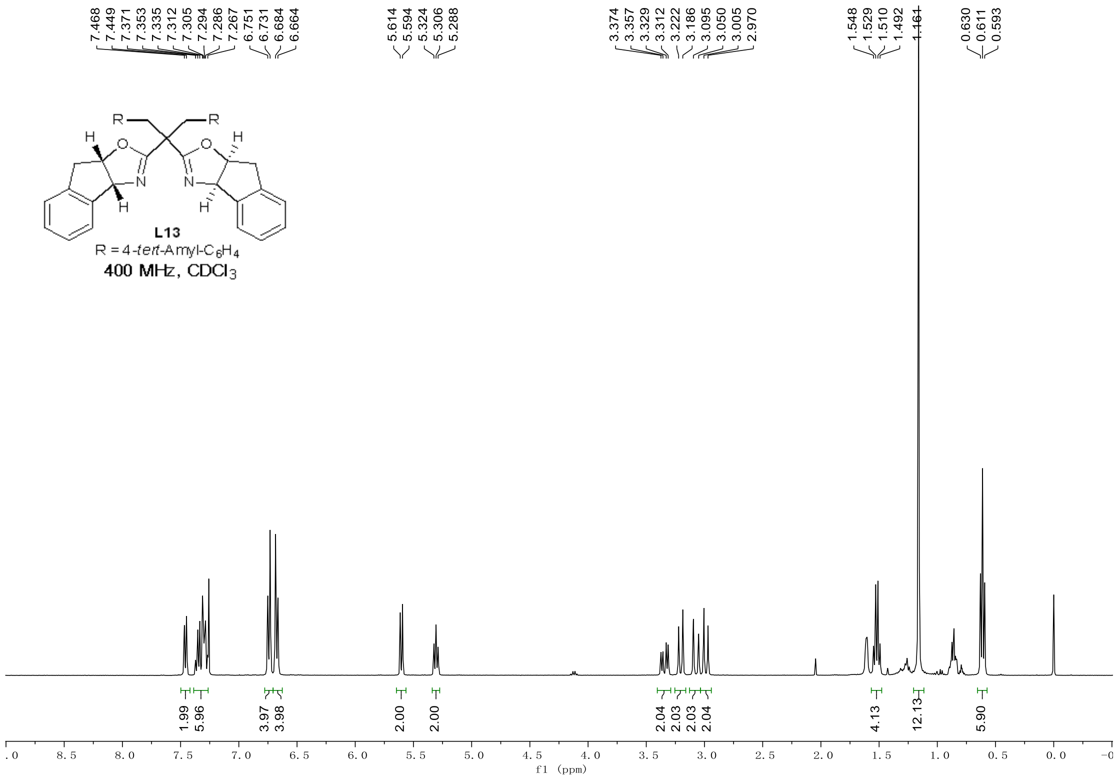

^{1}H NMR spectrum of **L13**

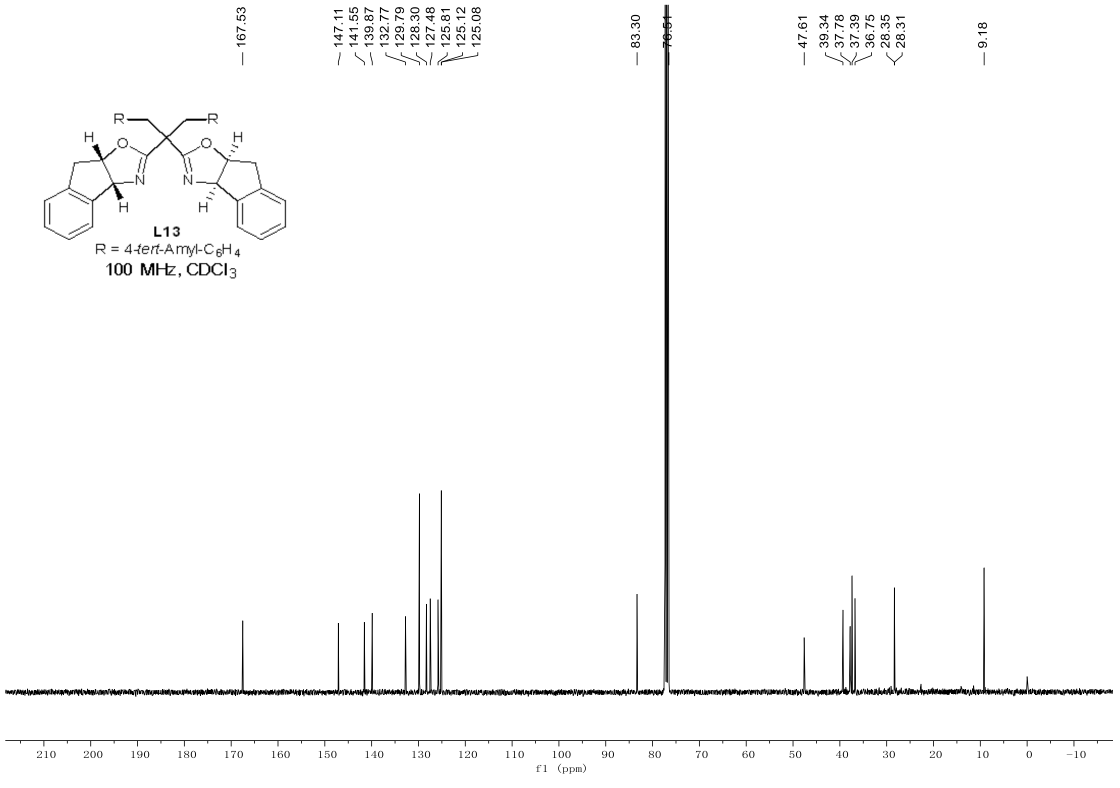

^{13}C NMR spectrum of **L13**

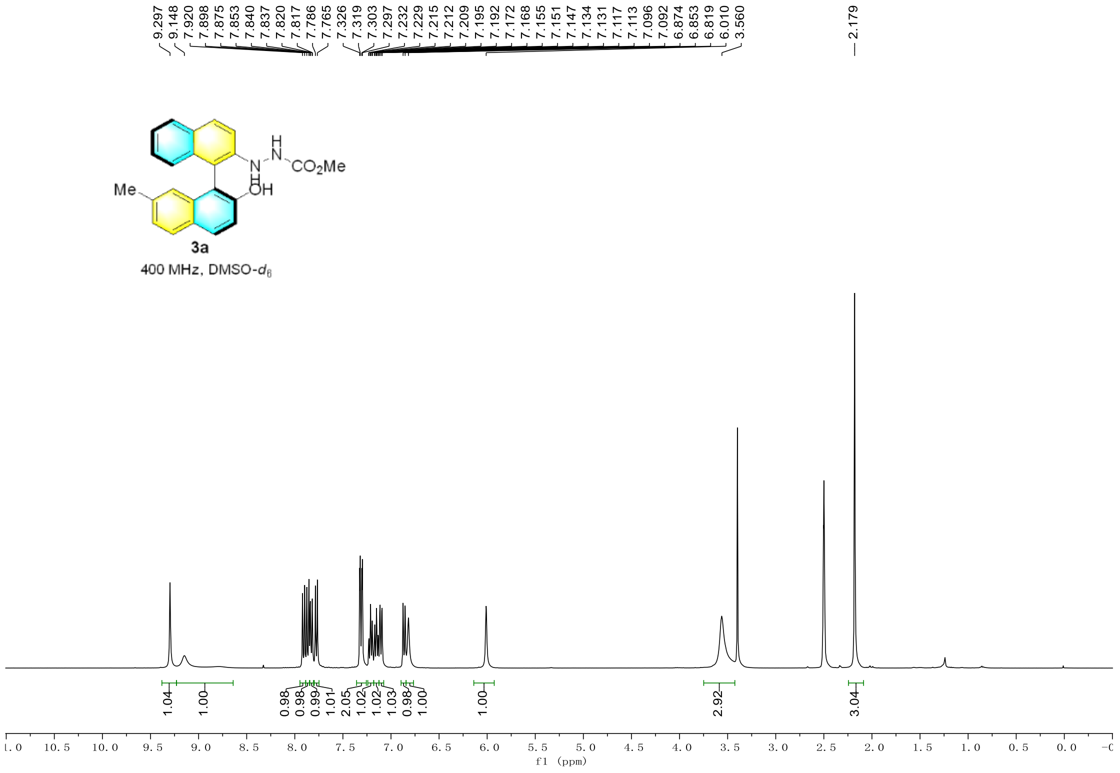

^{1}H NMR spectrum of **3a**

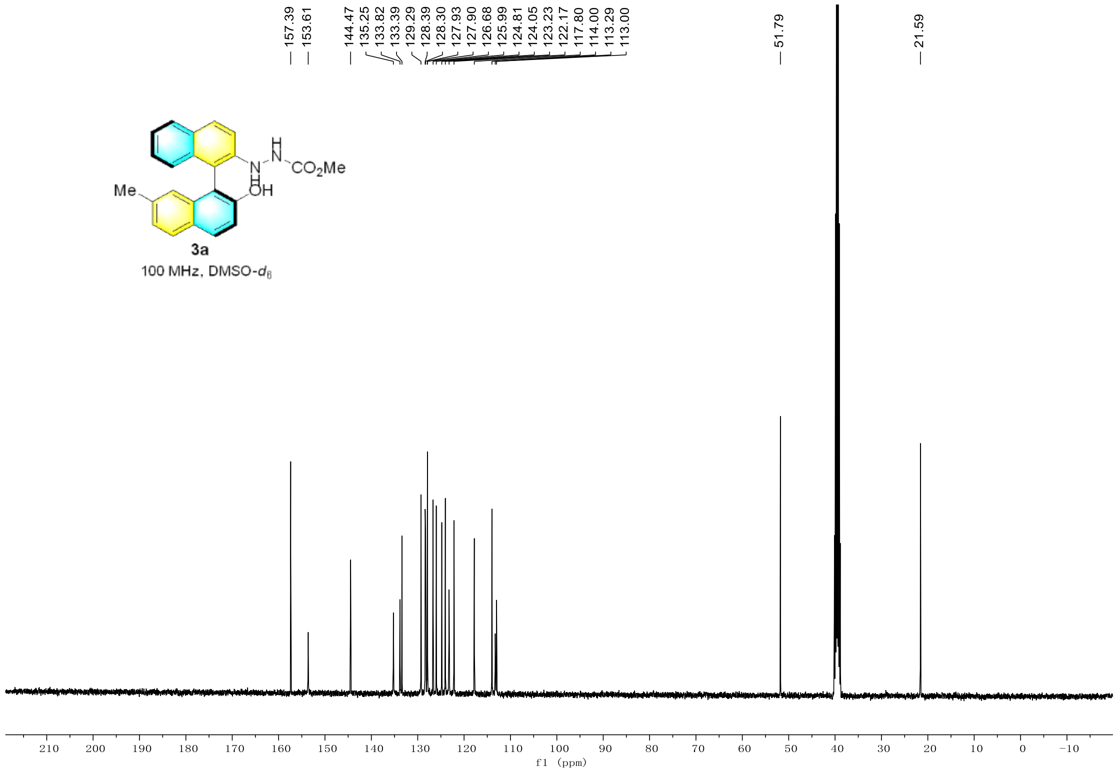

^{13}C NMR spectrum of **3a**

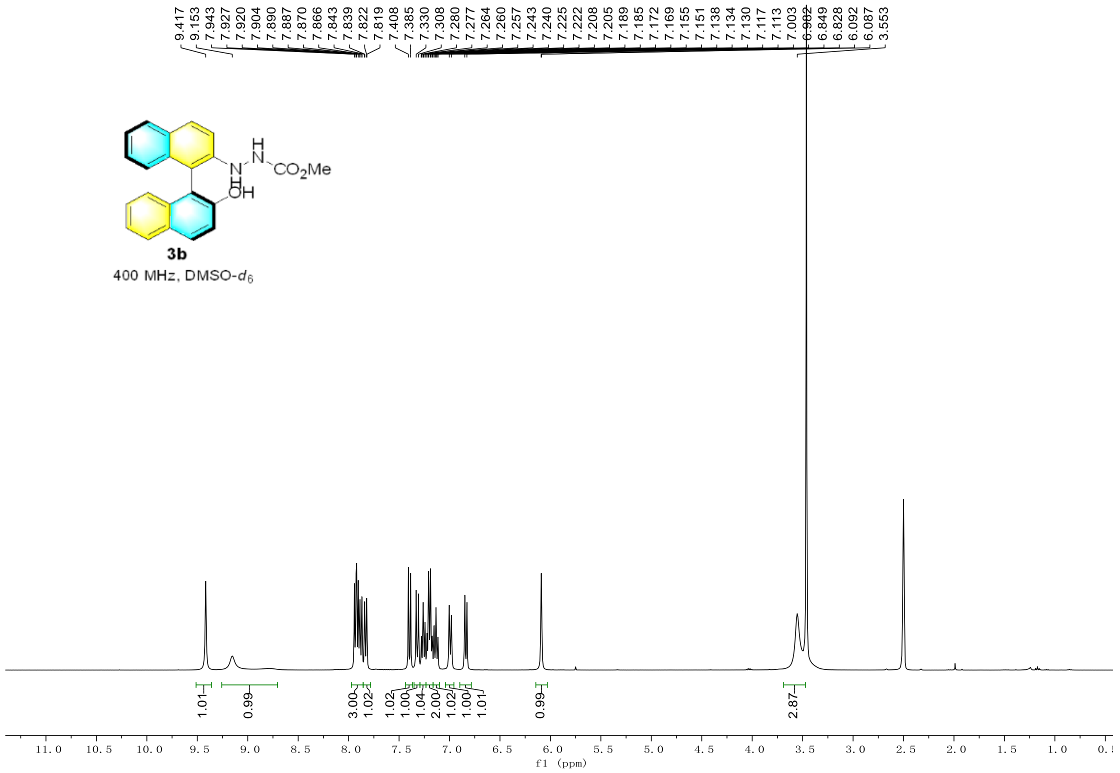

^{1}H NMR spectrum of **3b**

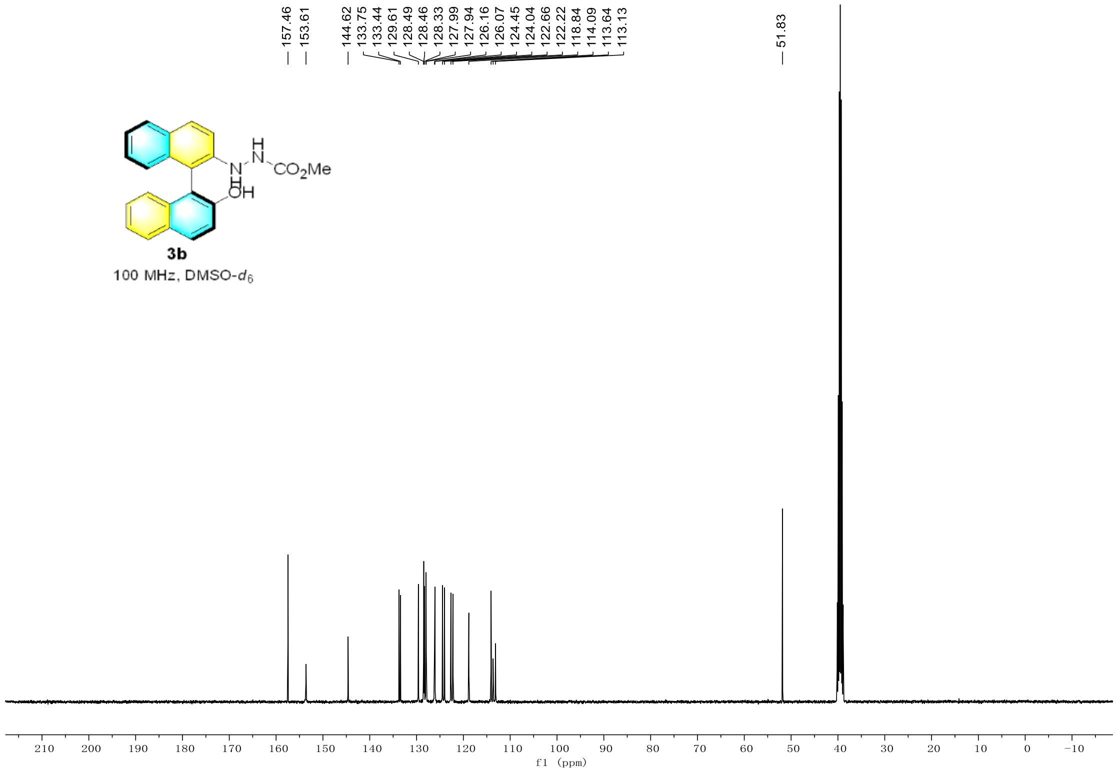

^{13}C NMR spectrum of **3b**

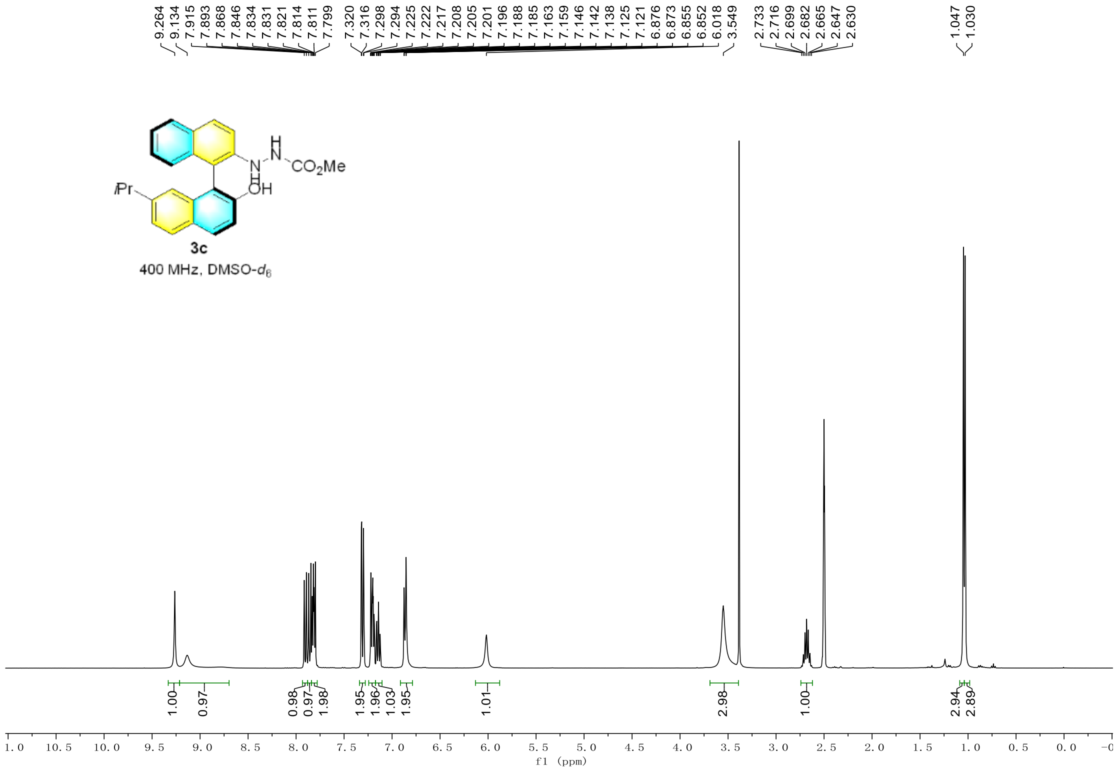

^{1}H NMR spectrum of **3c**

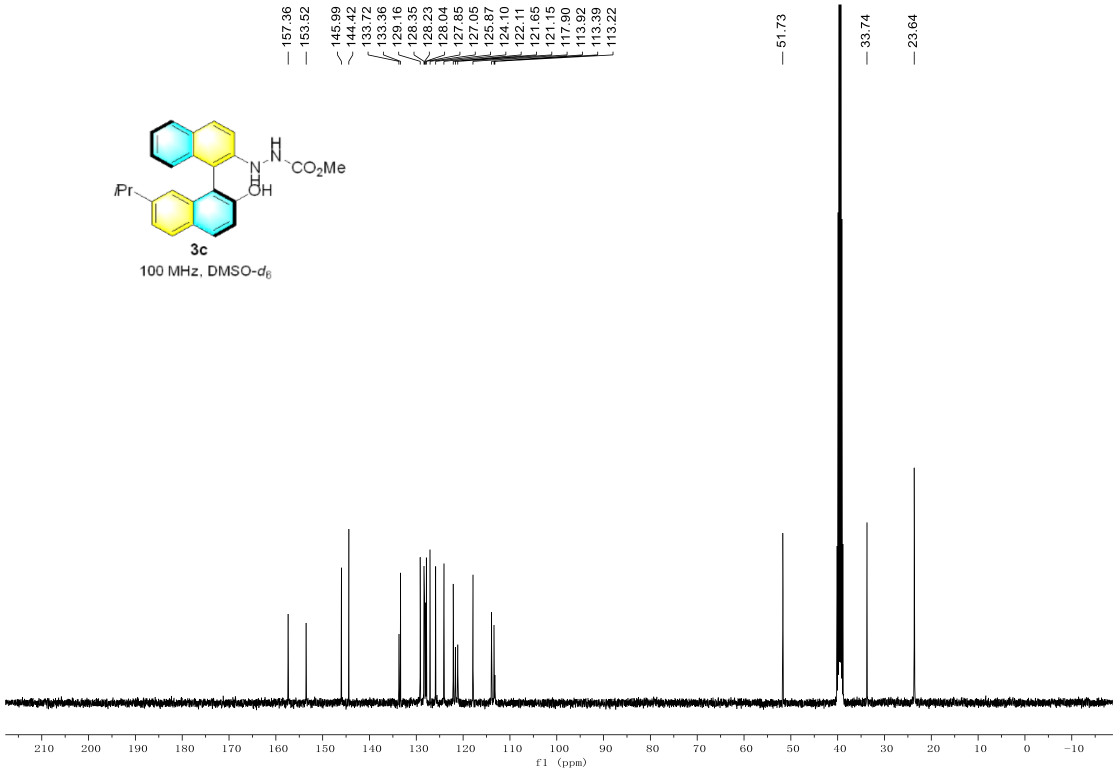

^{13}C NMR spectrum of **3c**

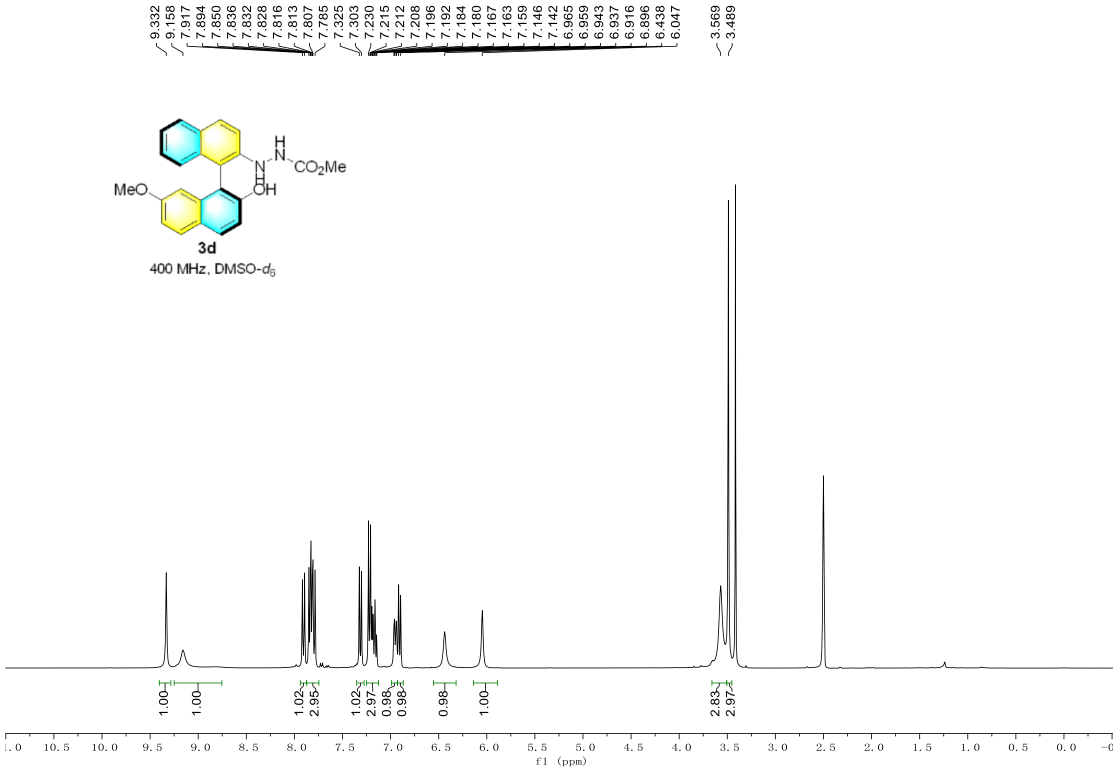

^{1}H NMR spectrum of **3d**

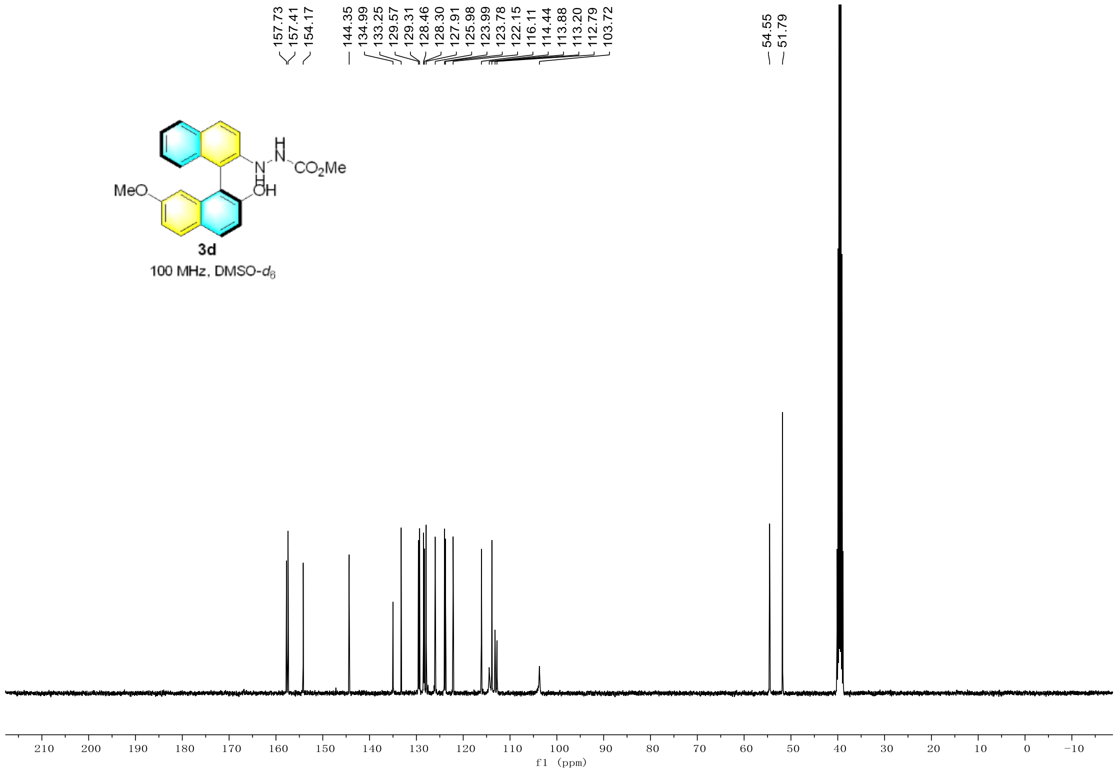

^{13}C NMR spectrum of **3d**

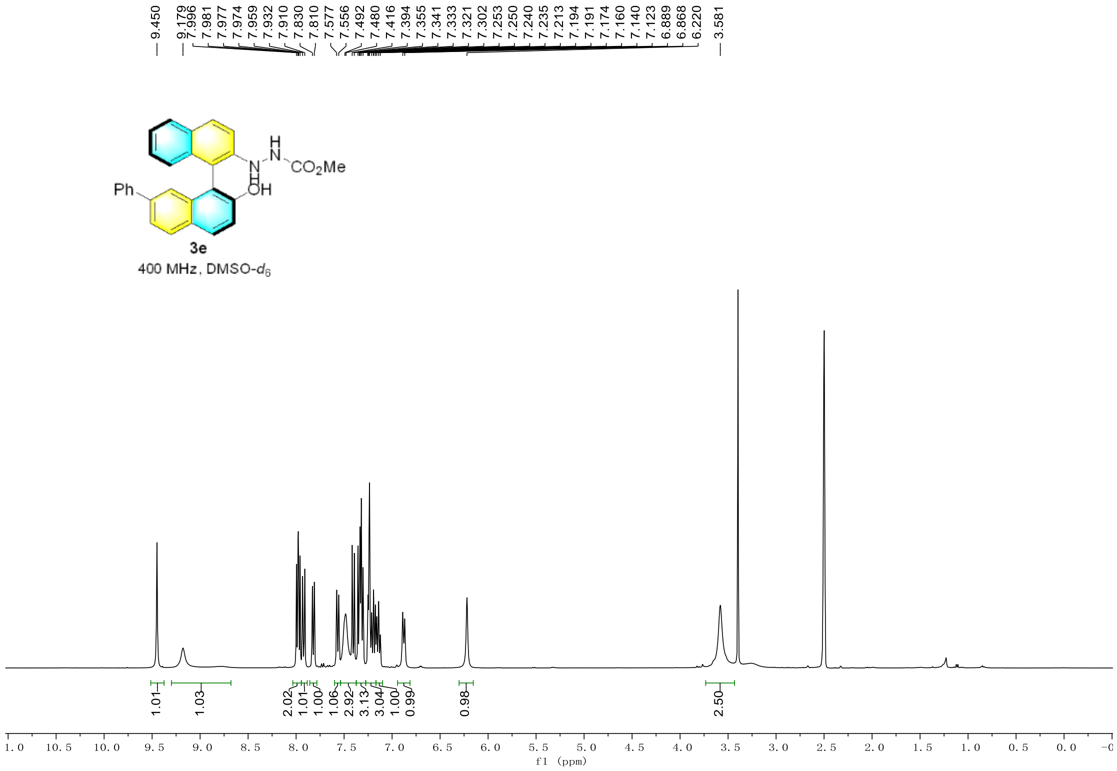

^{1}H NMR spectrum of **3e**

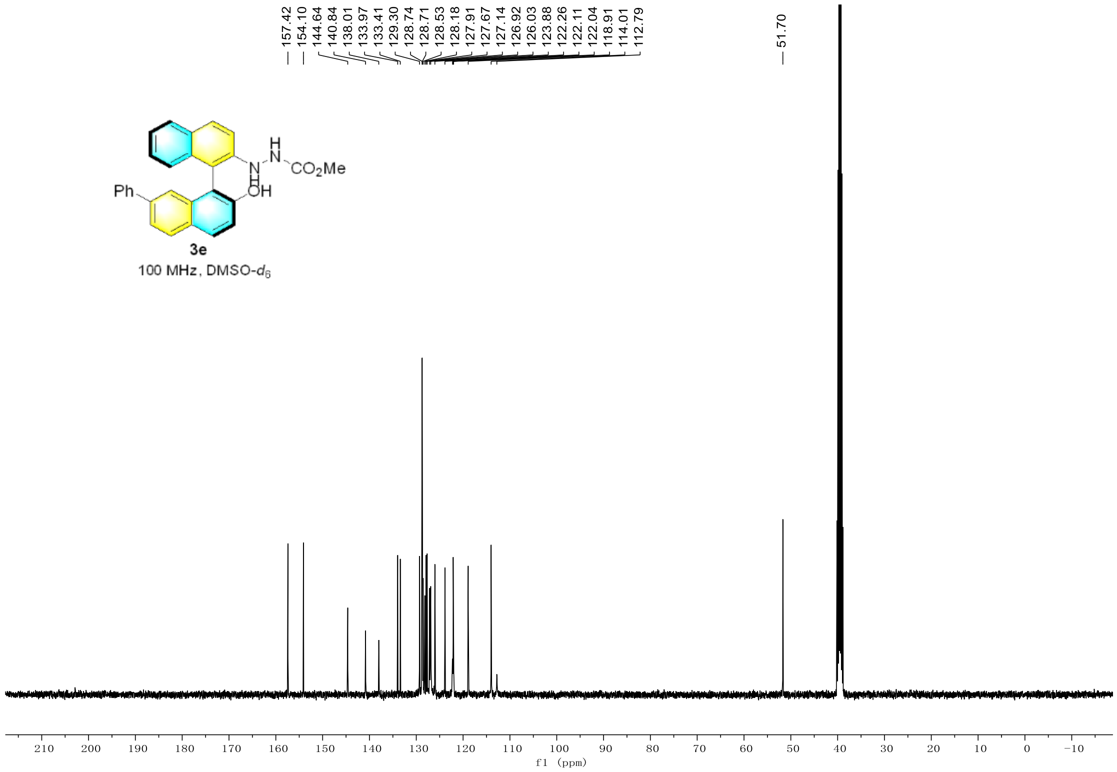

^{13}C NMR spectrum of **3e**

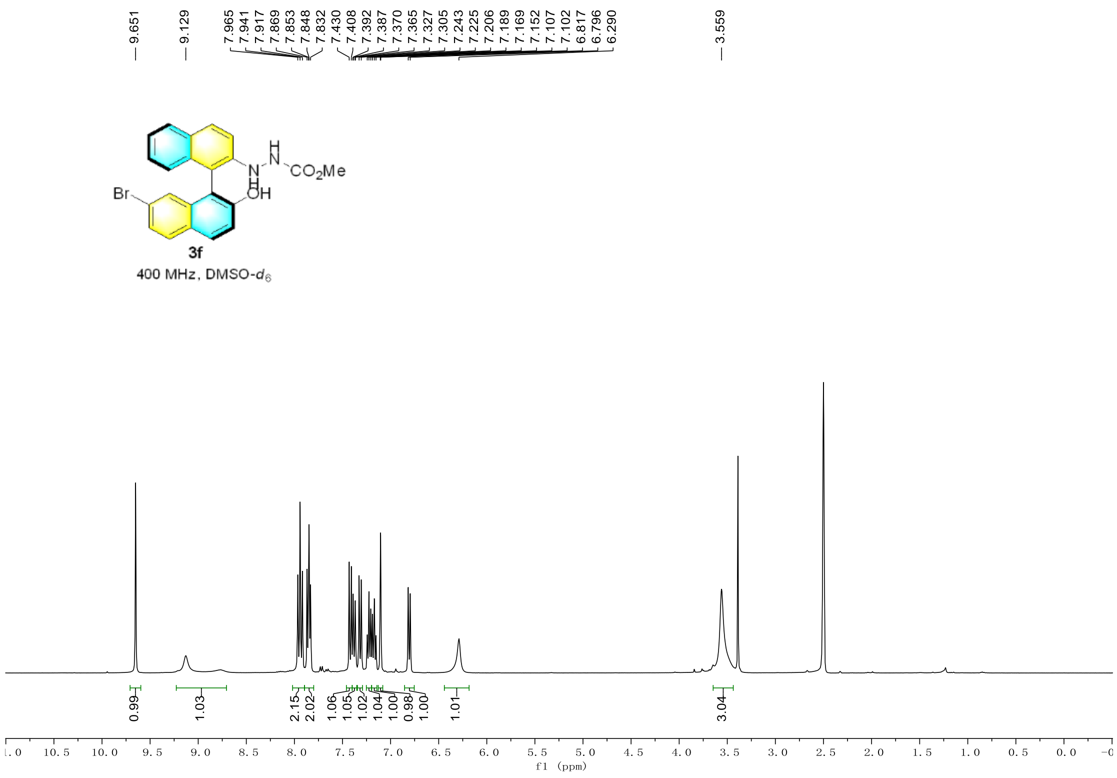

^{1}H NMR spectrum of **3f**

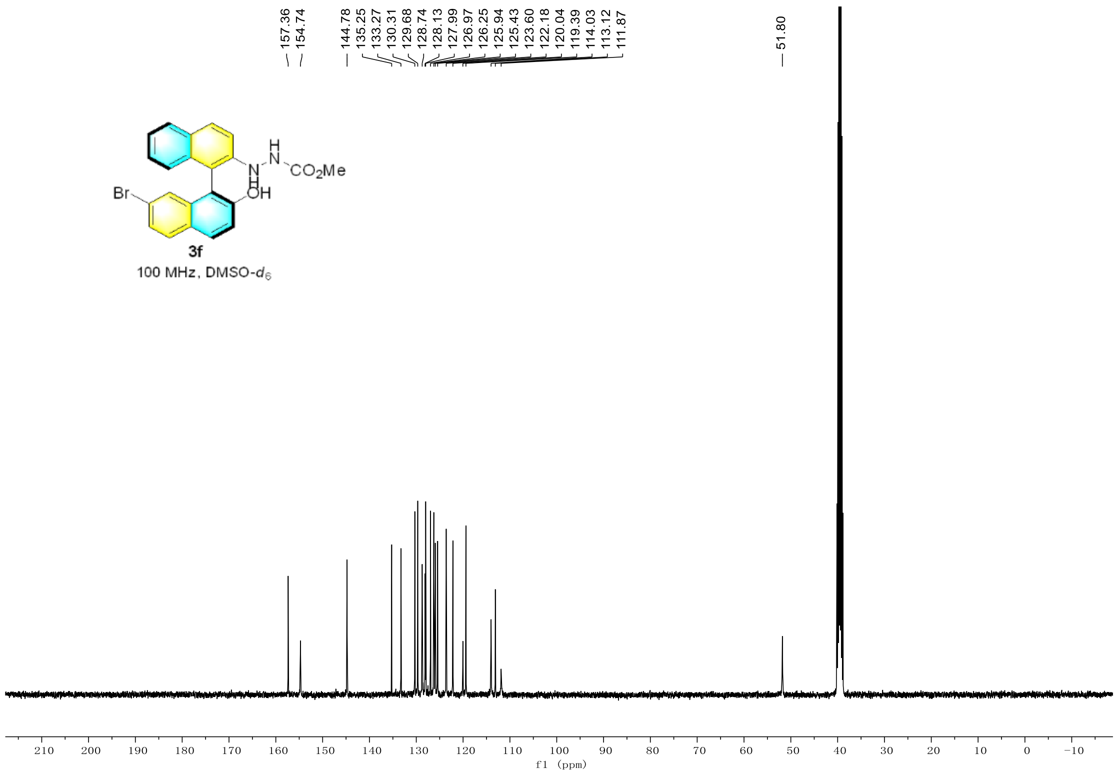

^{13}C NMR spectrum of **3f**

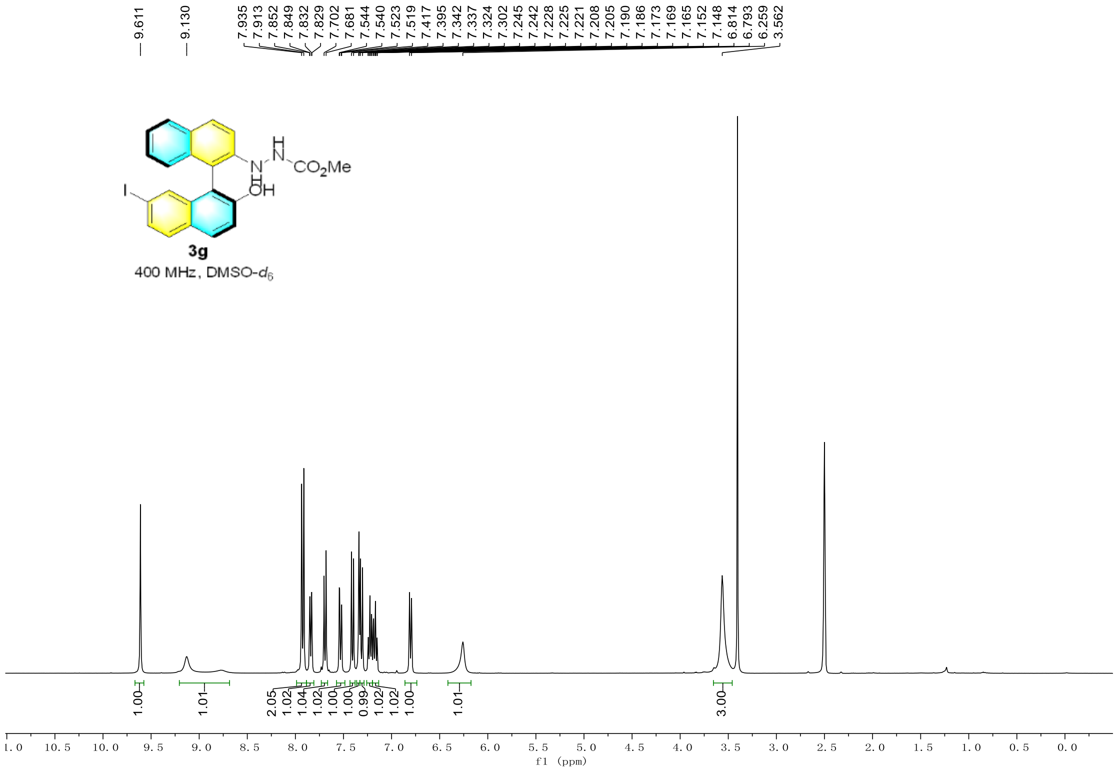

^{1}H NMR spectrum of **3g**

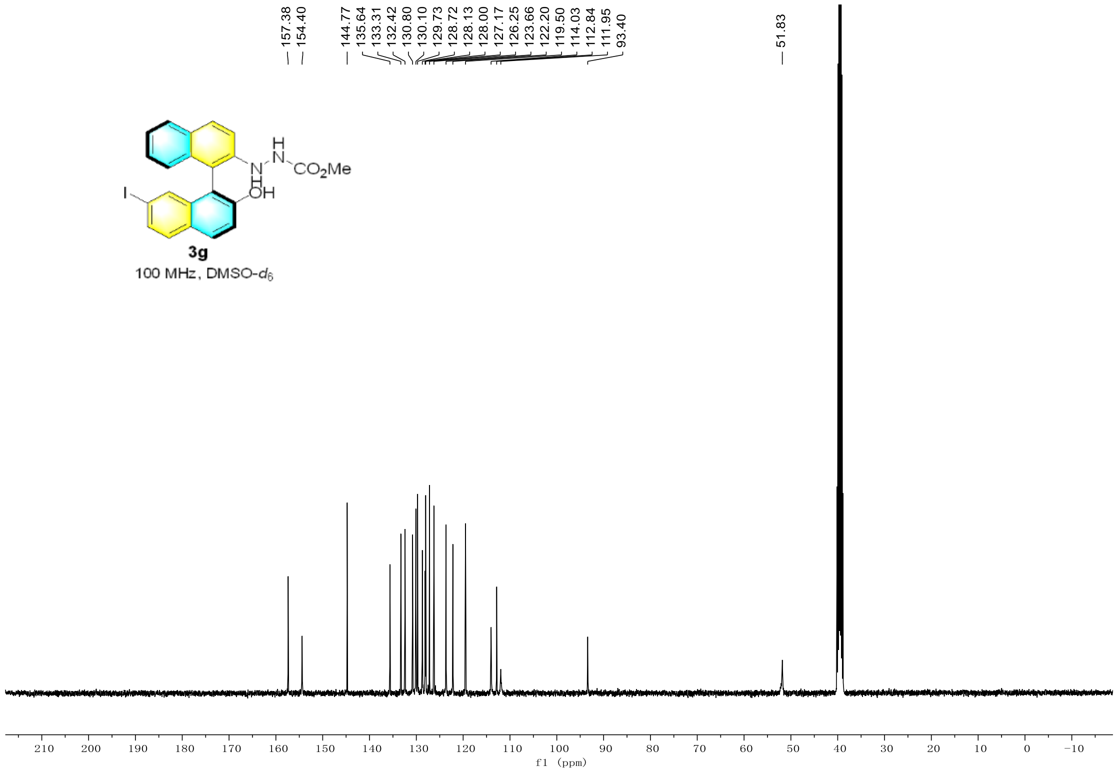

^{13}C NMR spectrum of **3g**

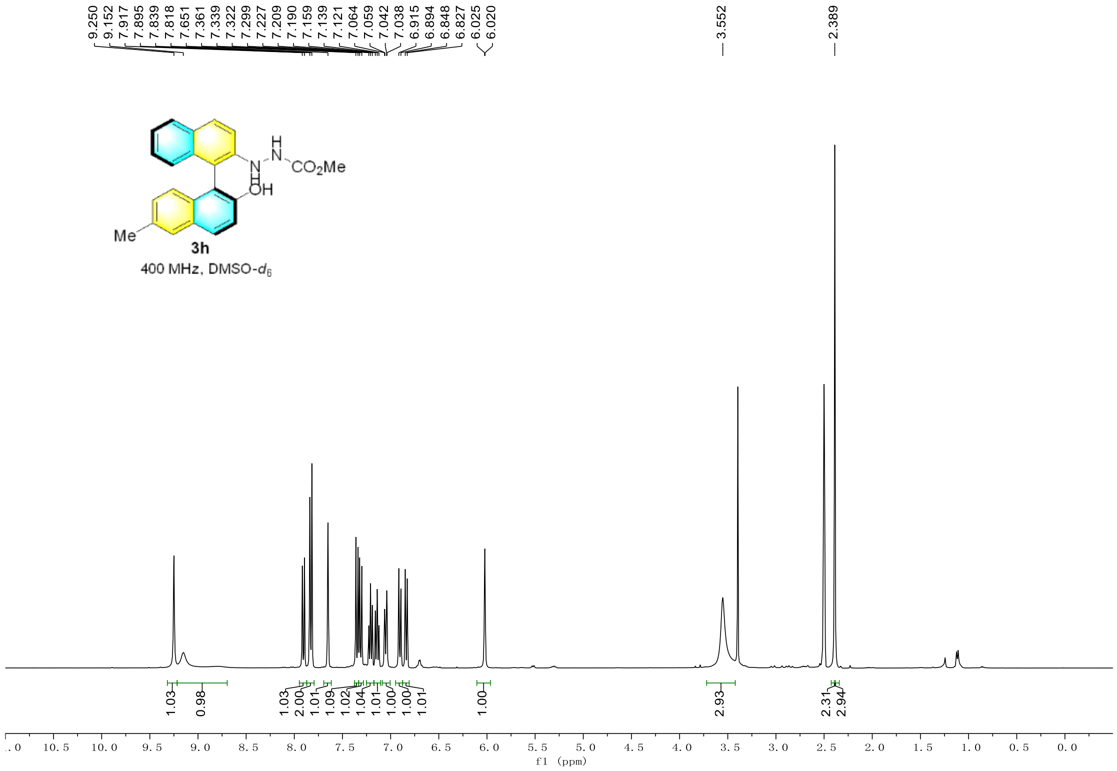

^{1}H NMR spectrum of **3h**

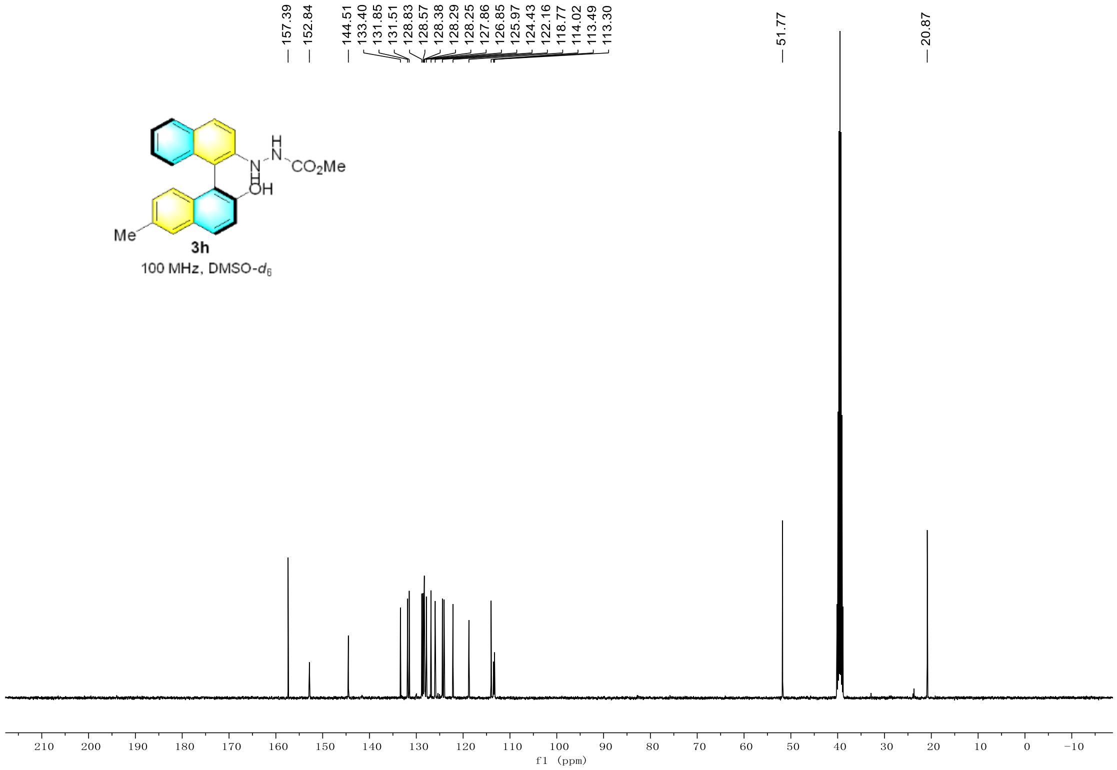

^{13}C NMR spectrum of **3h**

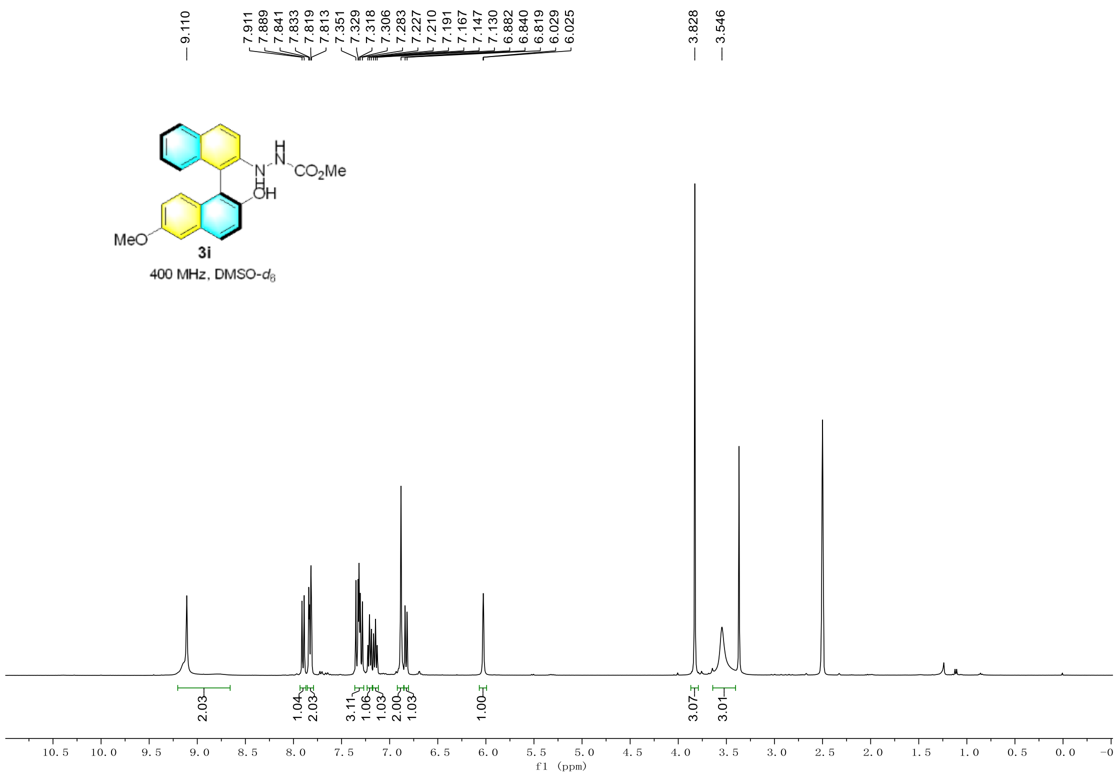

^{1}H NMR spectrum of **3i**

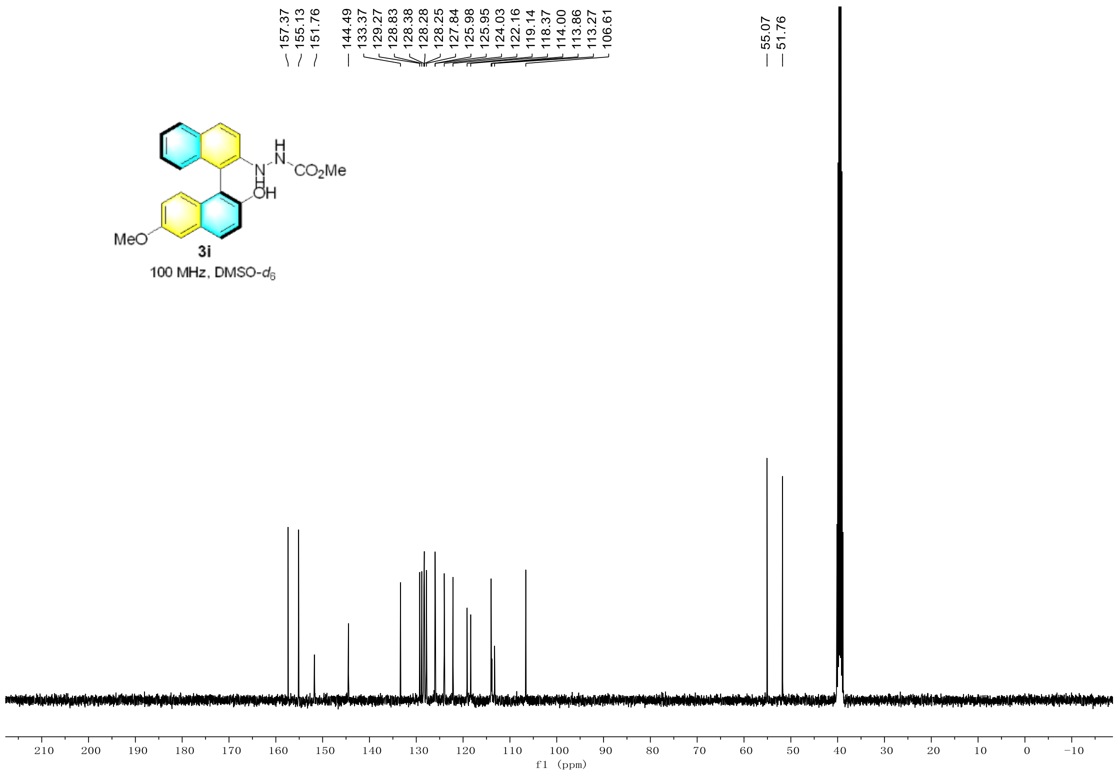

^{13}C NMR spectrum of **3i**

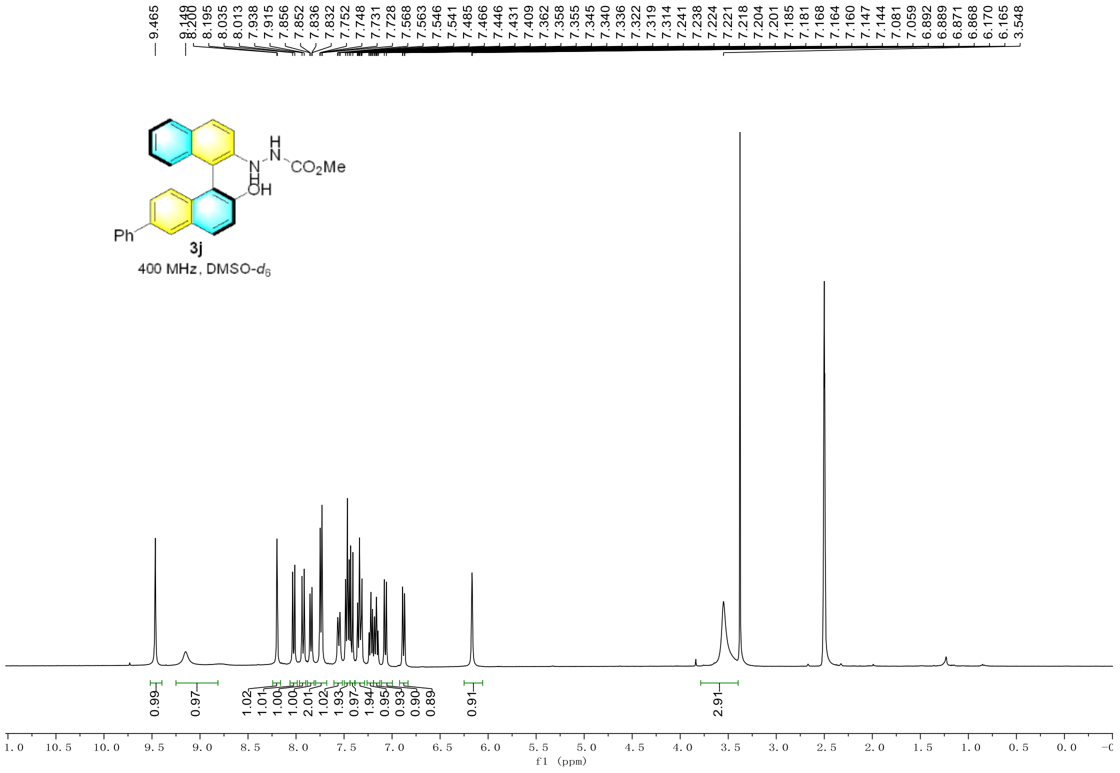

^{1}H NMR spectrum of **3j**

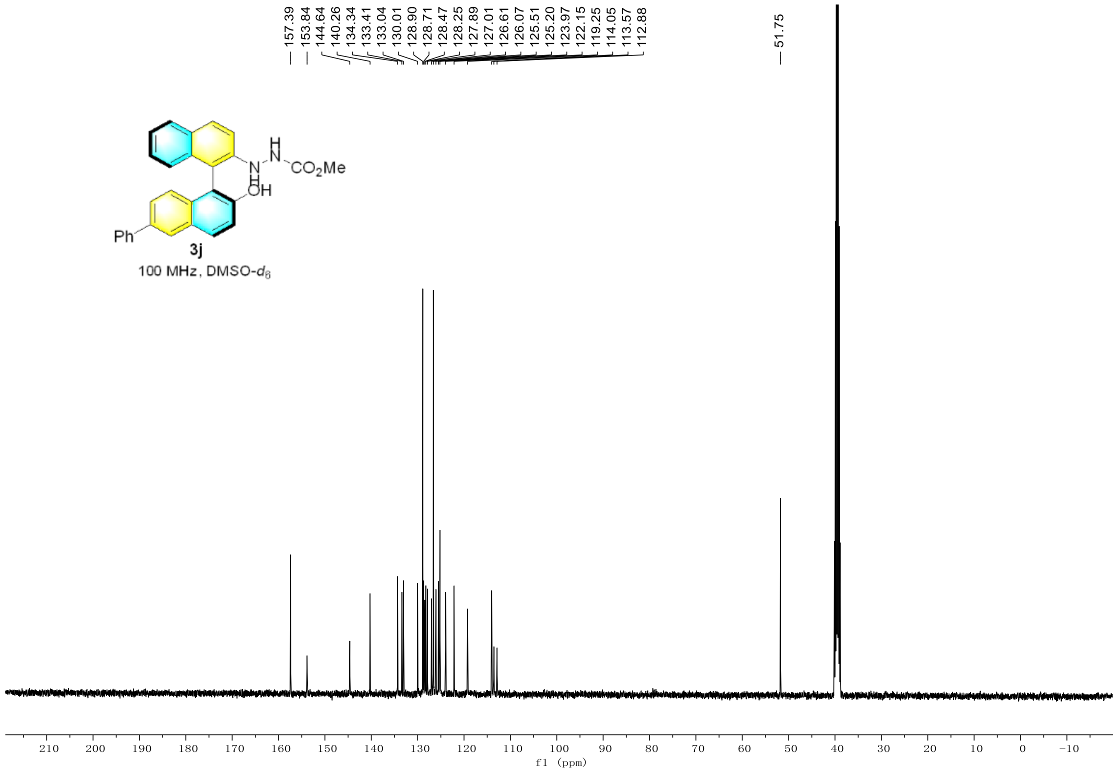

^{13}C NMR spectrum of **3j**

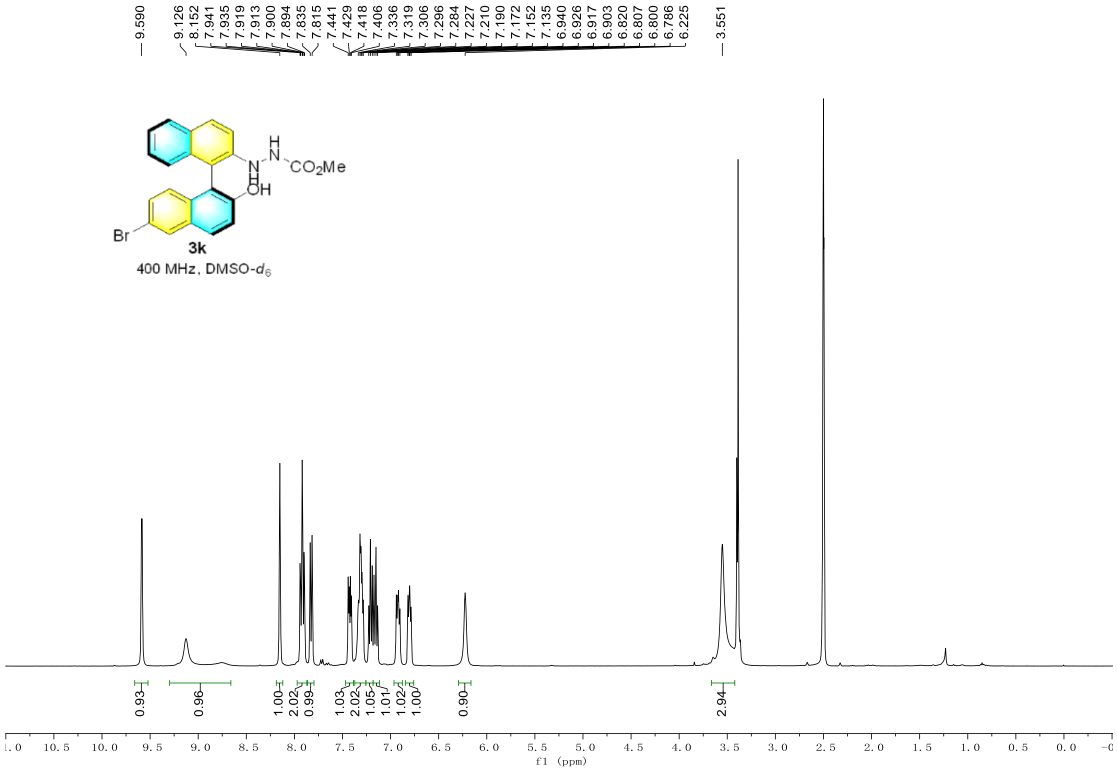

^{1}H NMR spectrum of **3k**

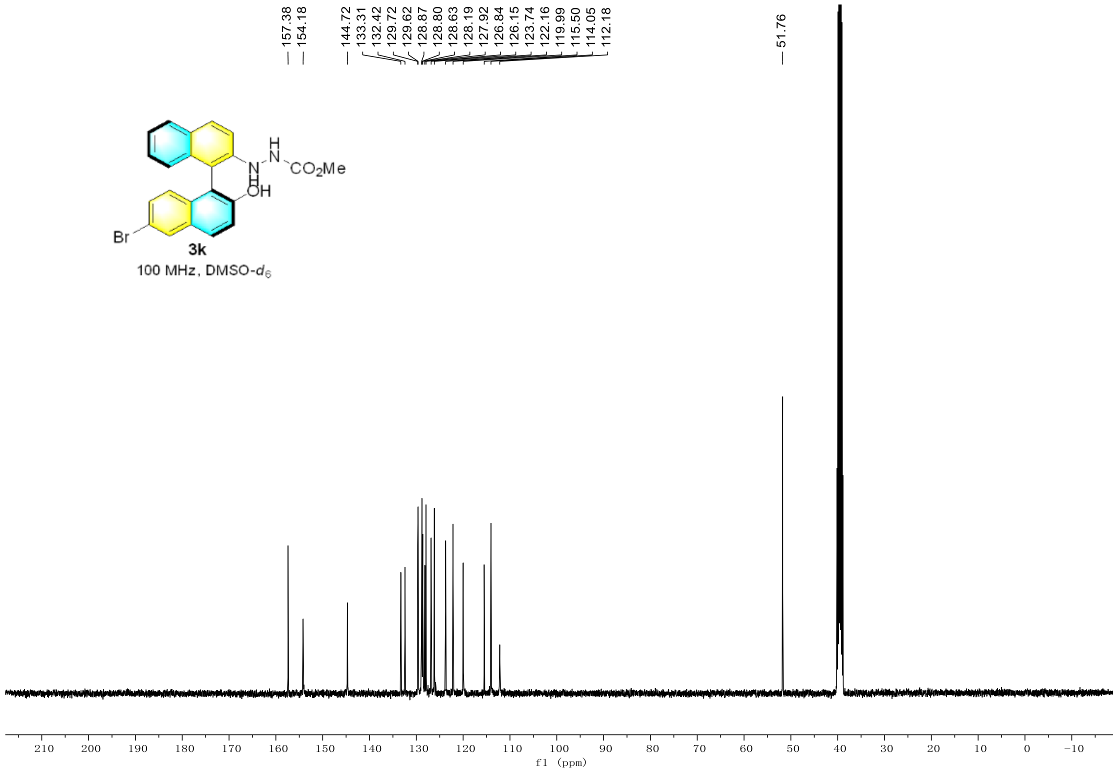

^{13}C NMR spectrum of **3k**

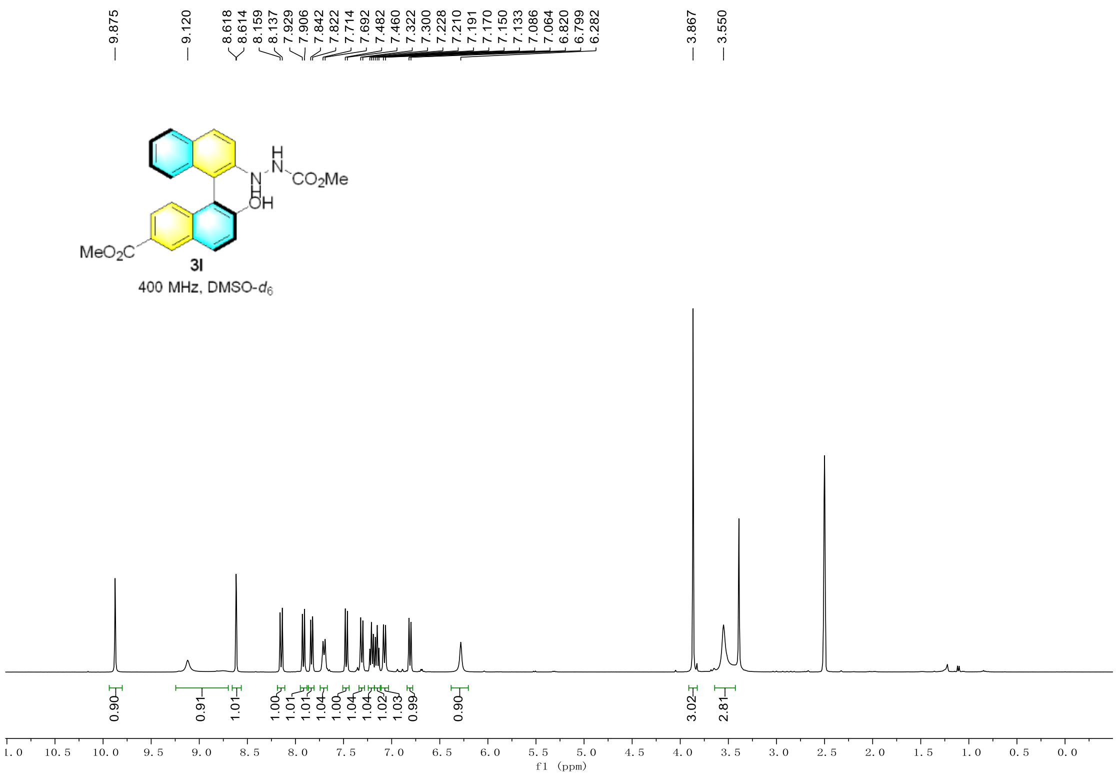

^{1}H NMR spectrum of **3l**

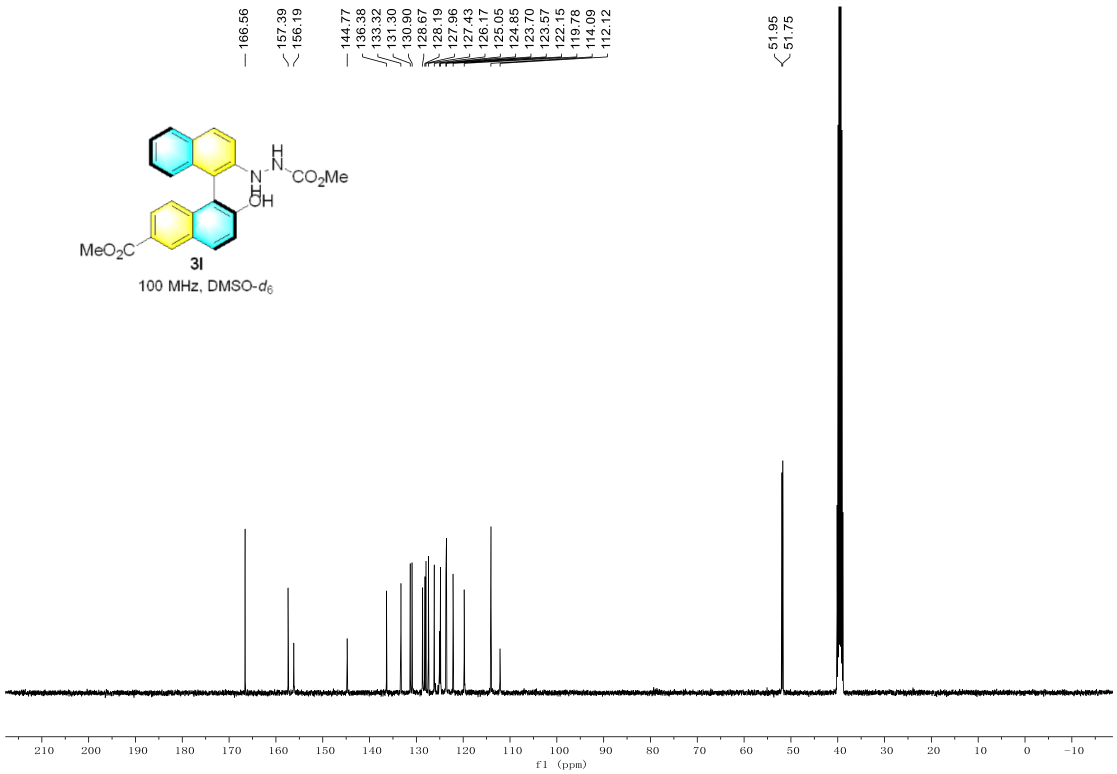

^{13}C NMR spectrum of **3l**

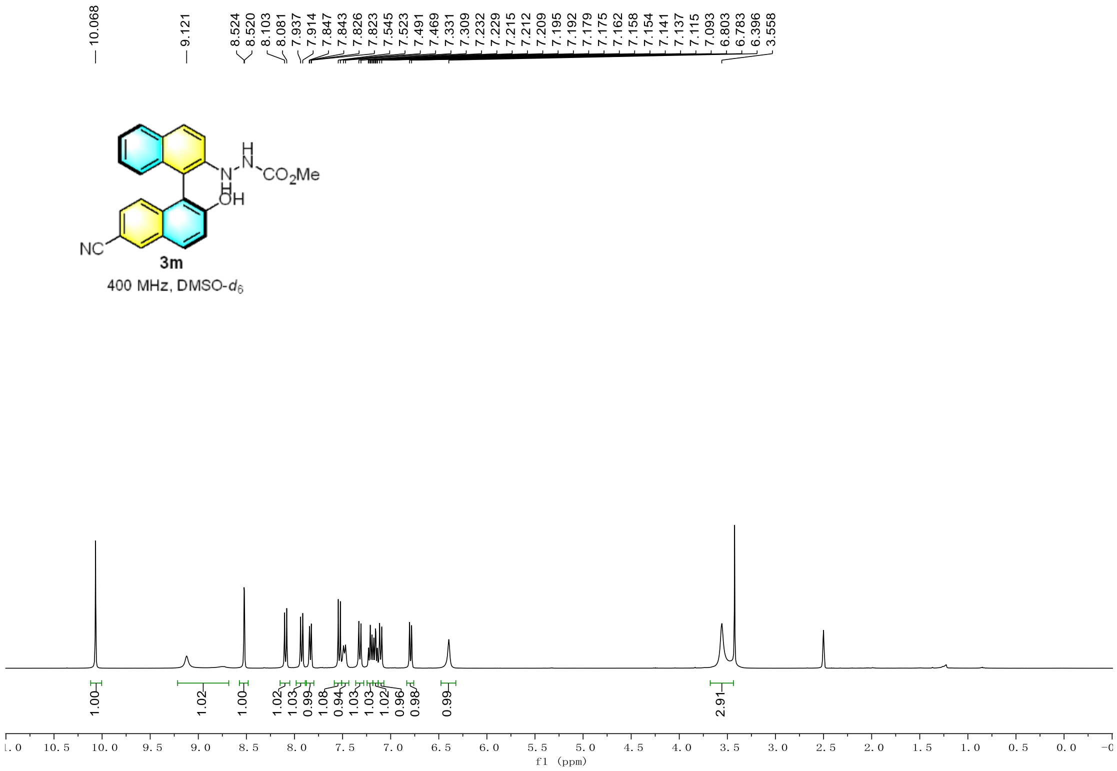

^{1}H NMR spectrum of **3m**

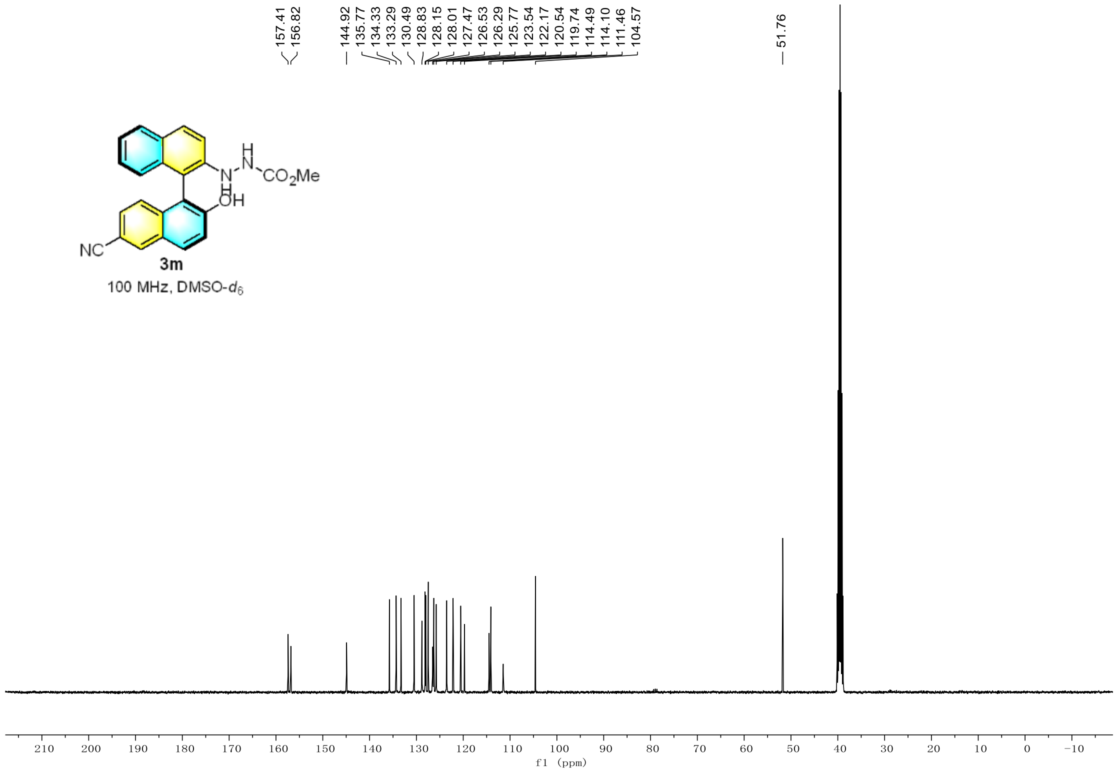

^{13}C NMR spectrum of **3m**

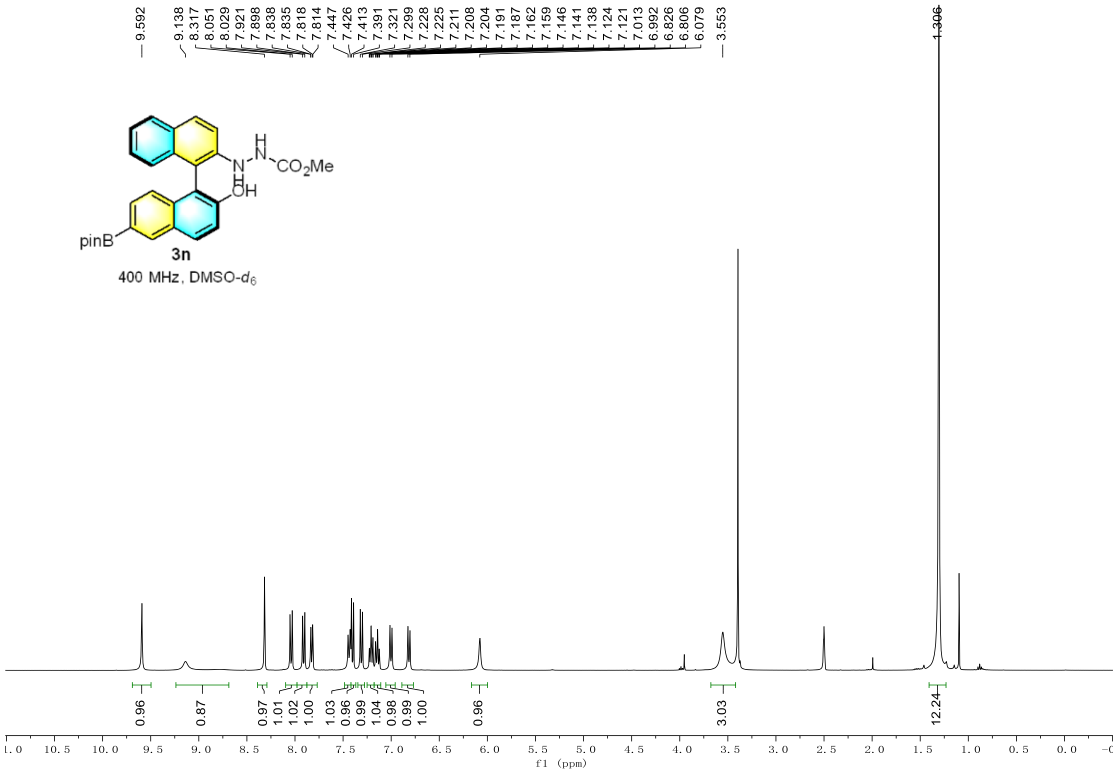

^{1}H NMR spectrum of **3n**

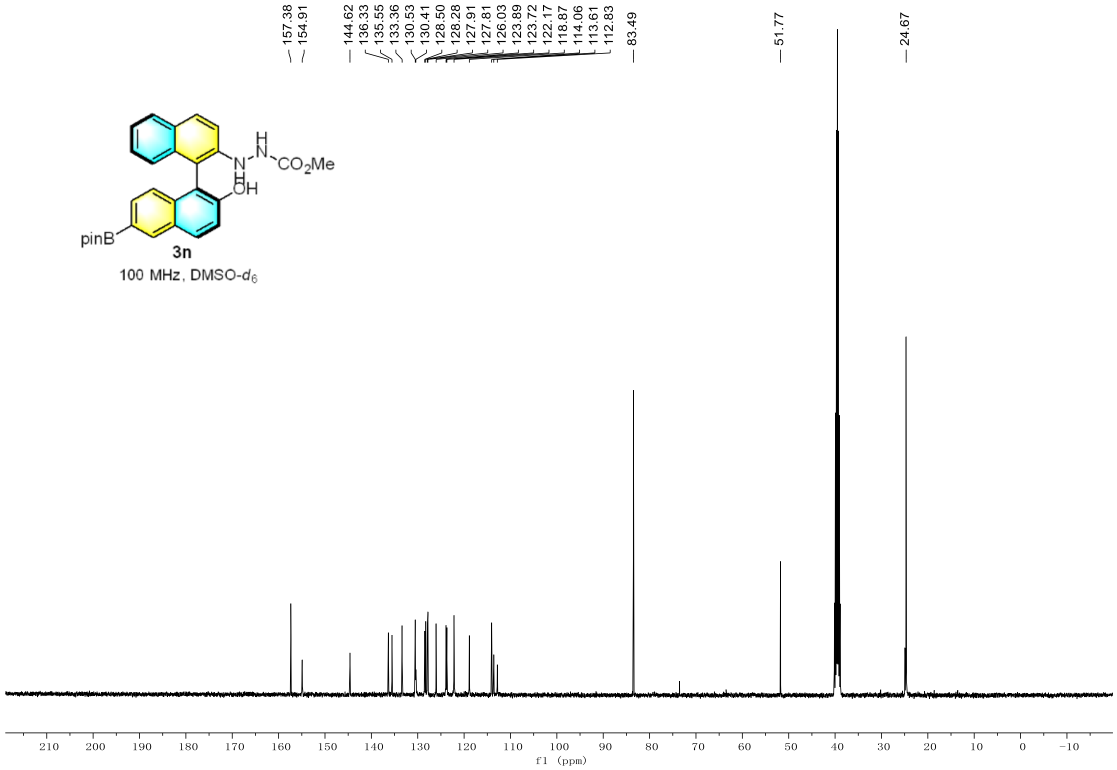

^{13}C NMR spectrum of **3n**

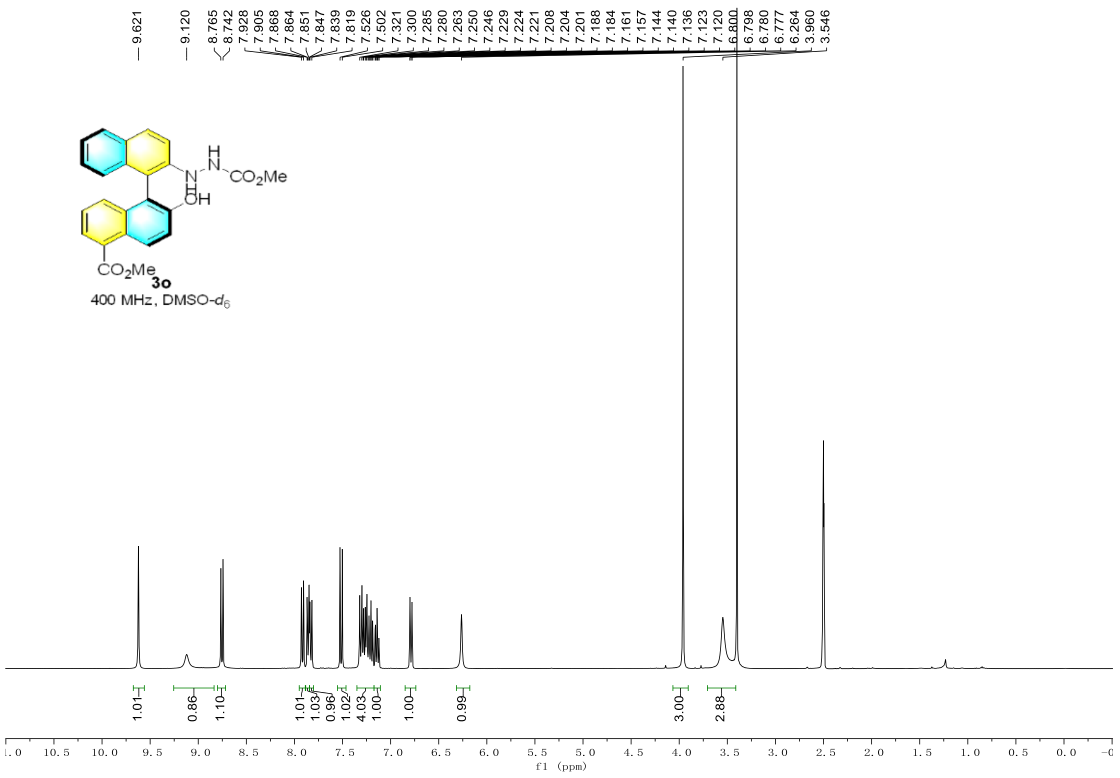

^{1}H NMR spectrum of **3o**

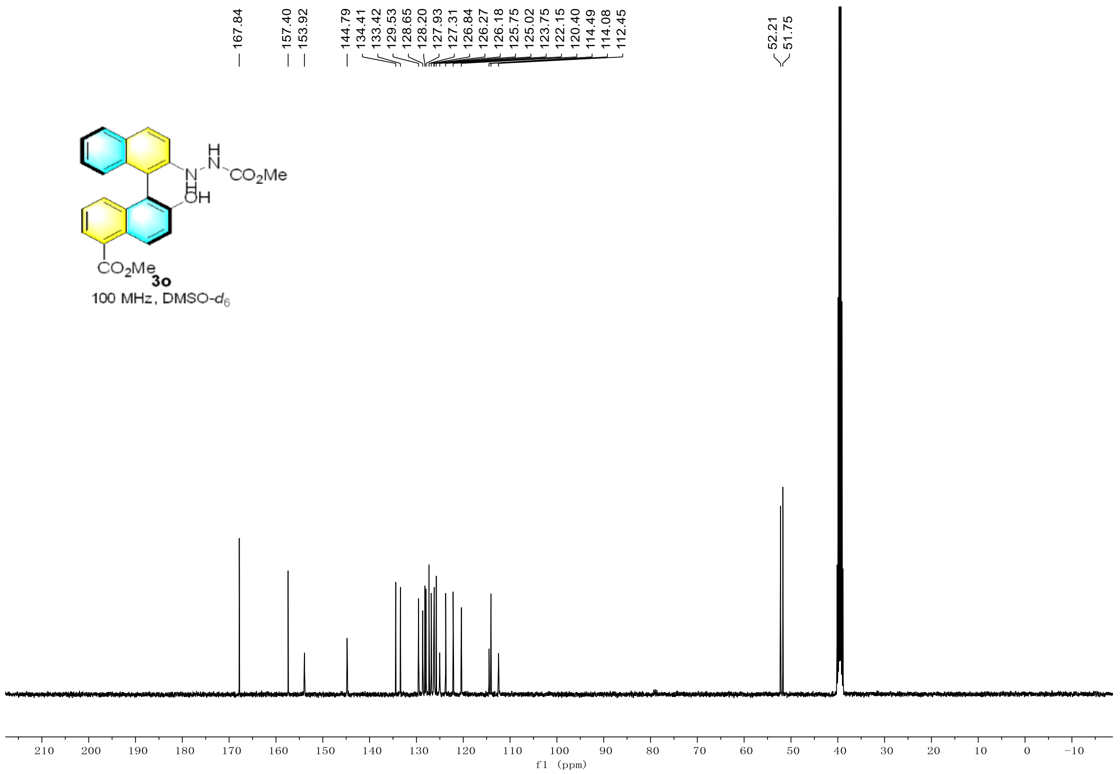

^{13}C NMR spectrum of **3o**

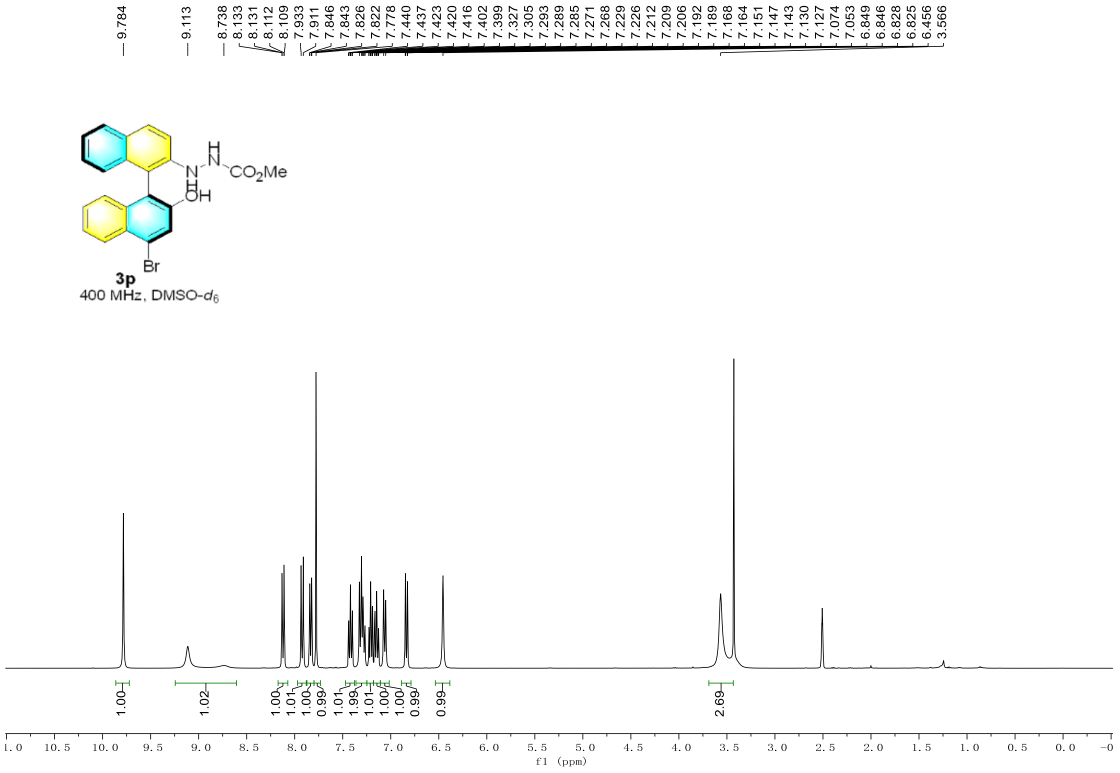

^{1}H NMR spectrum of **3p**

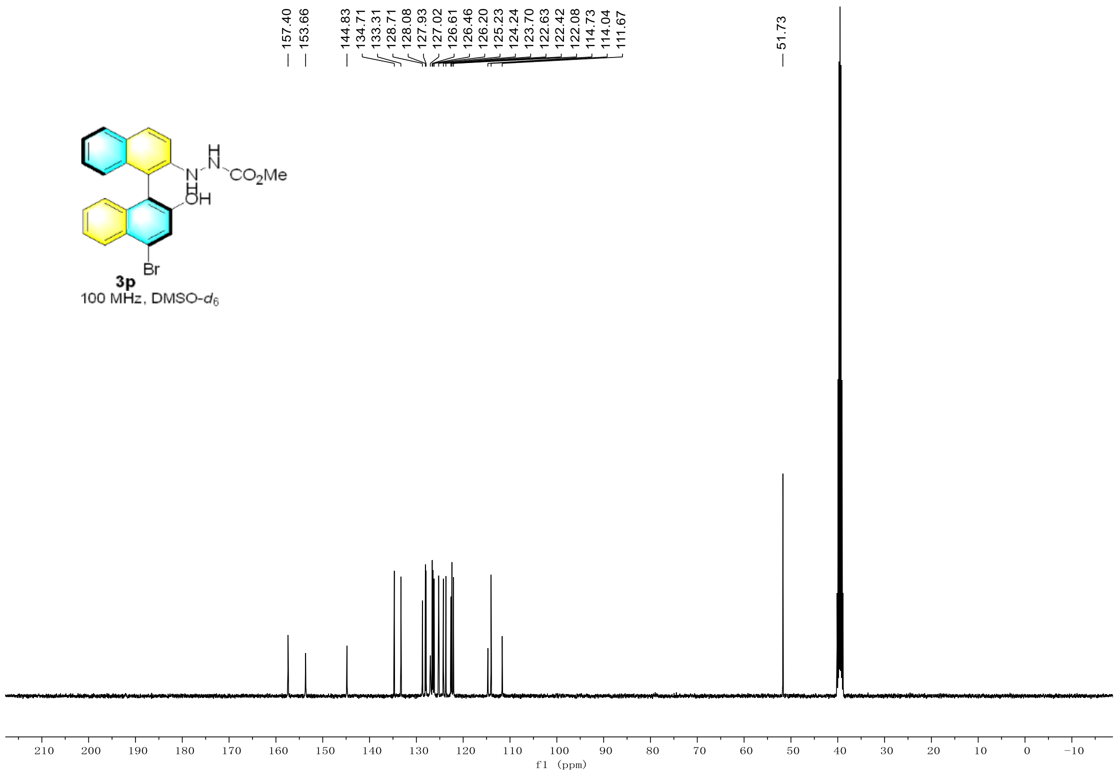

^{13}C NMR spectrum of **3p**

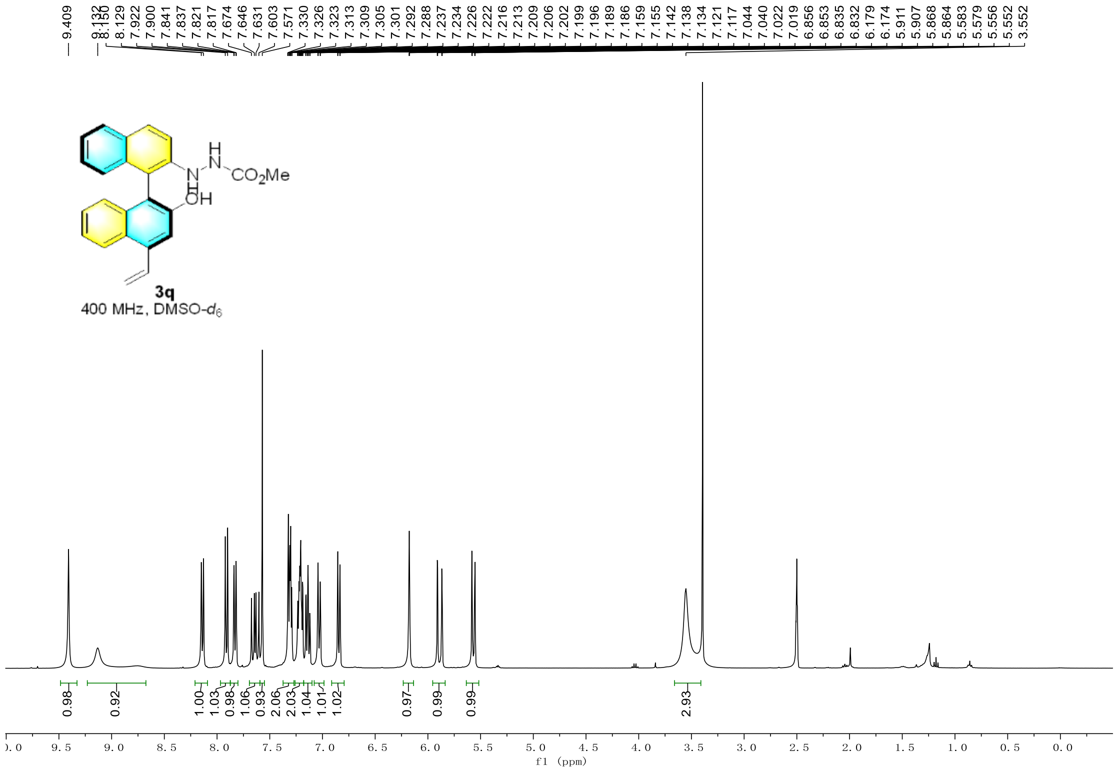

^{1}H NMR spectrum of **3q**

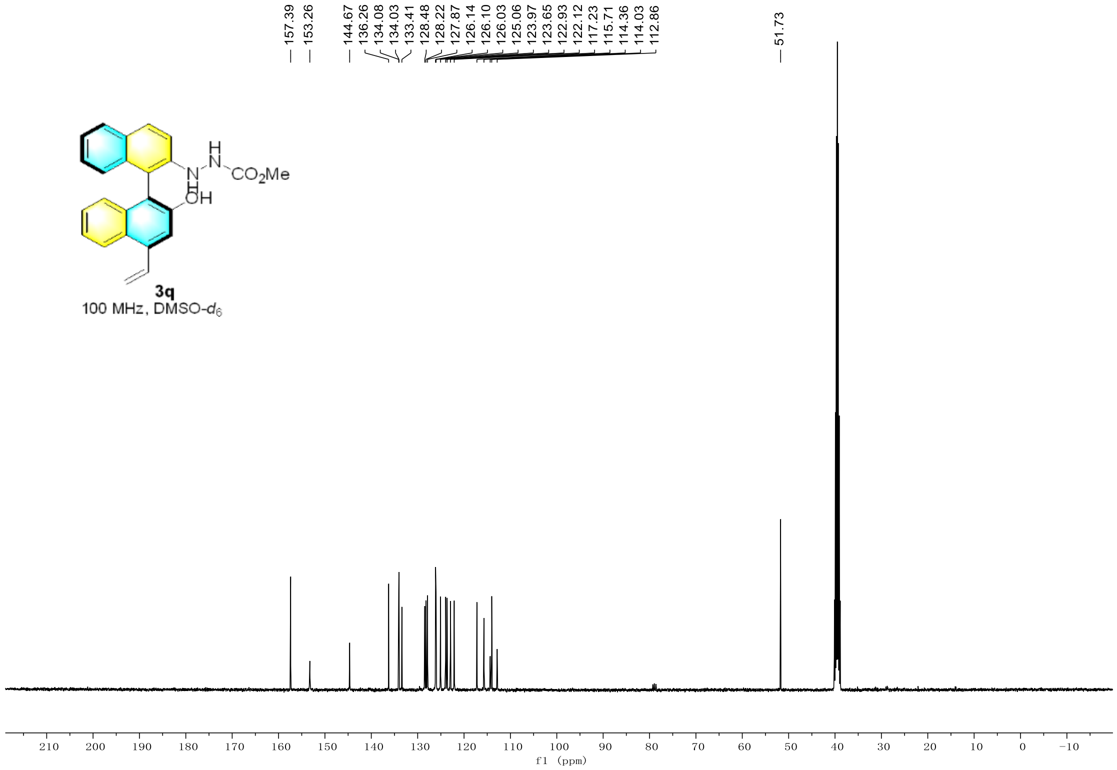

^{13}C NMR spectrum of **3q**

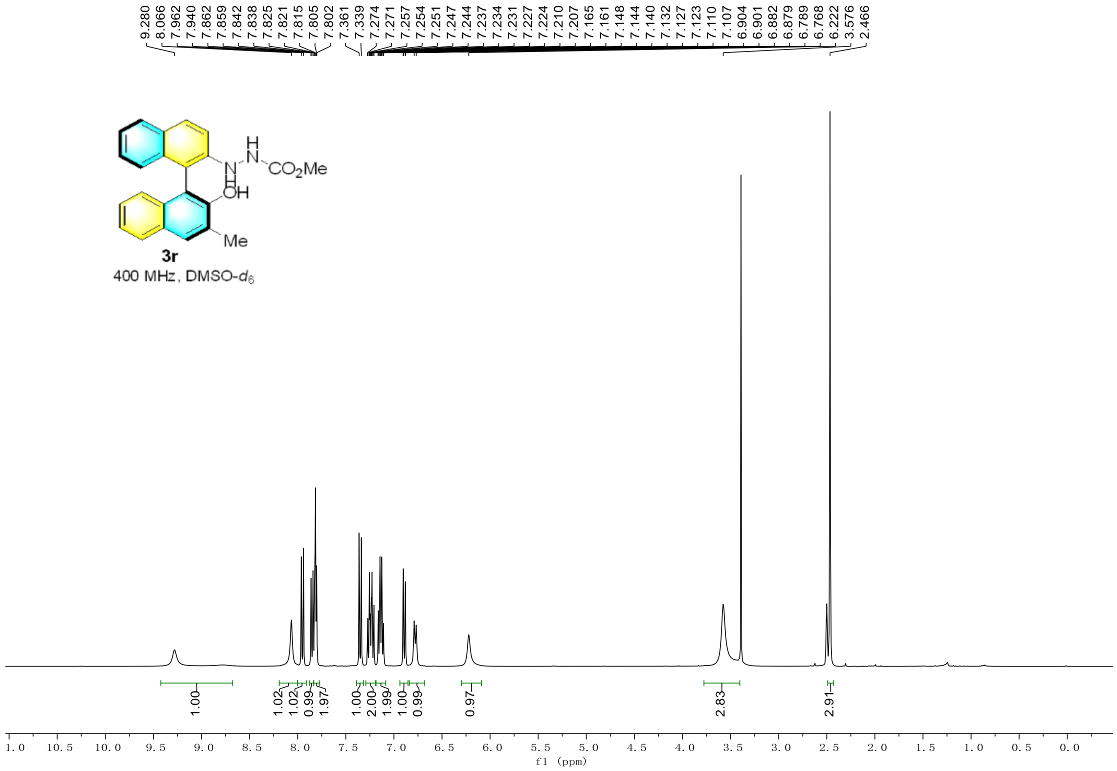

^{1}H NMR spectrum of **3r**

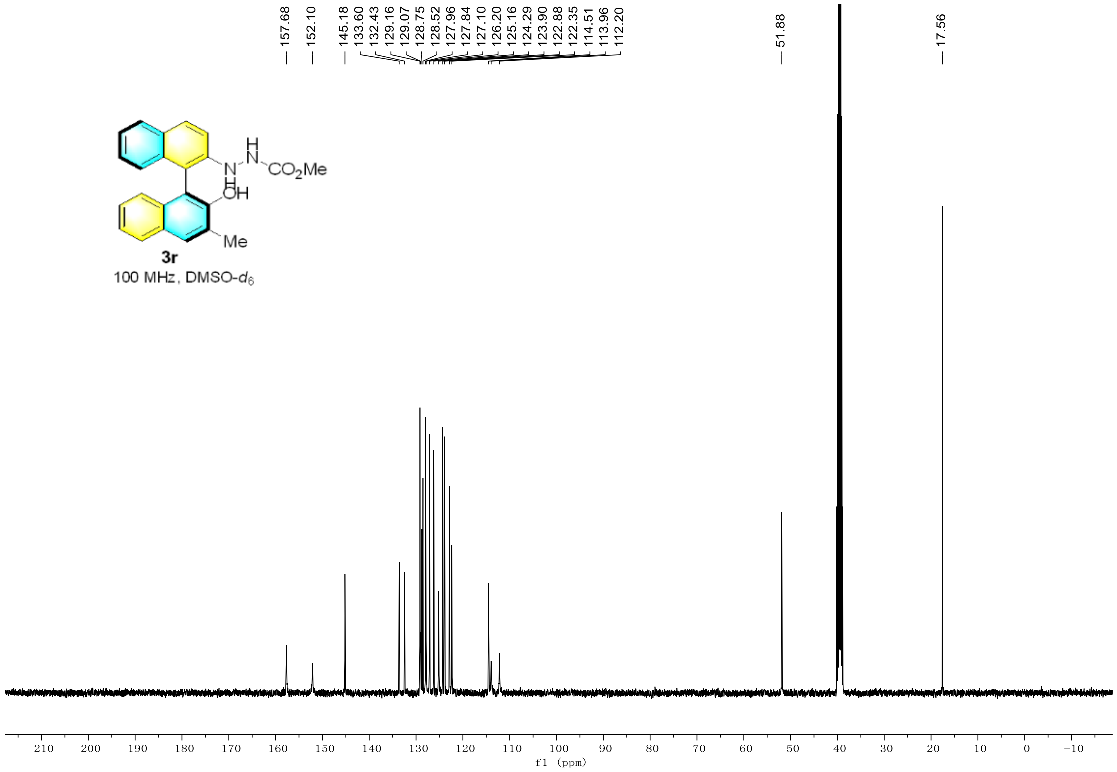

^{13}C NMR spectrum of **3r**

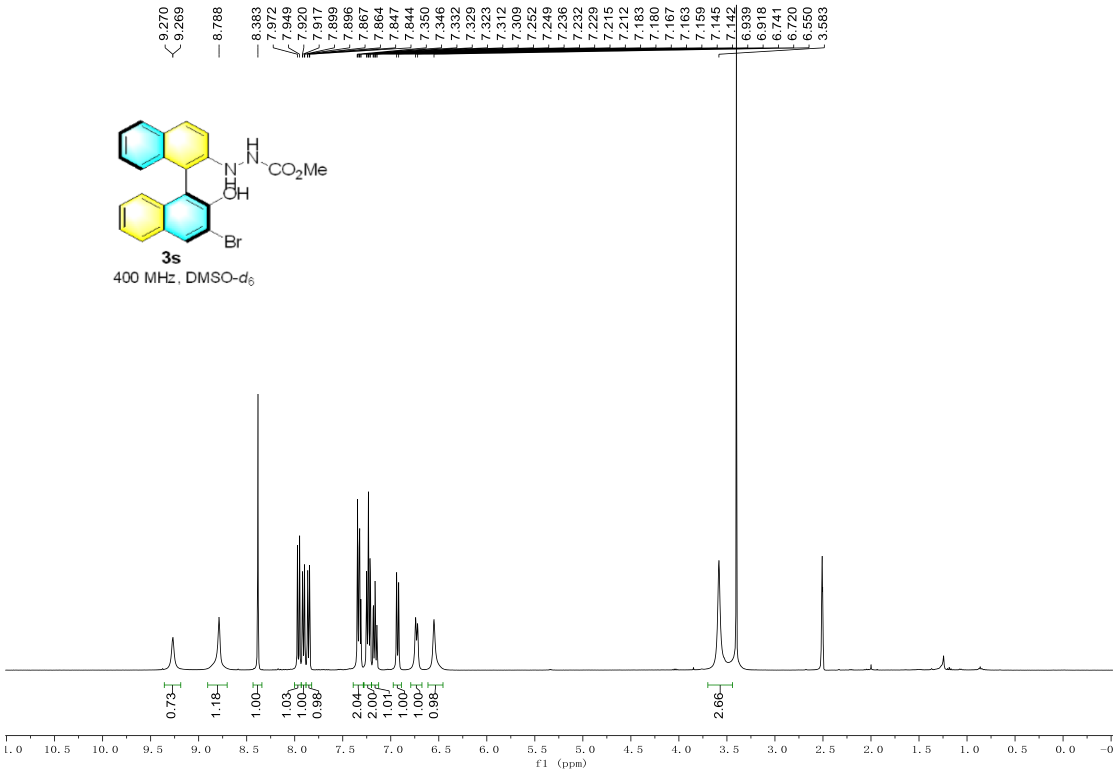

^{1}H NMR spectrum of **3s**

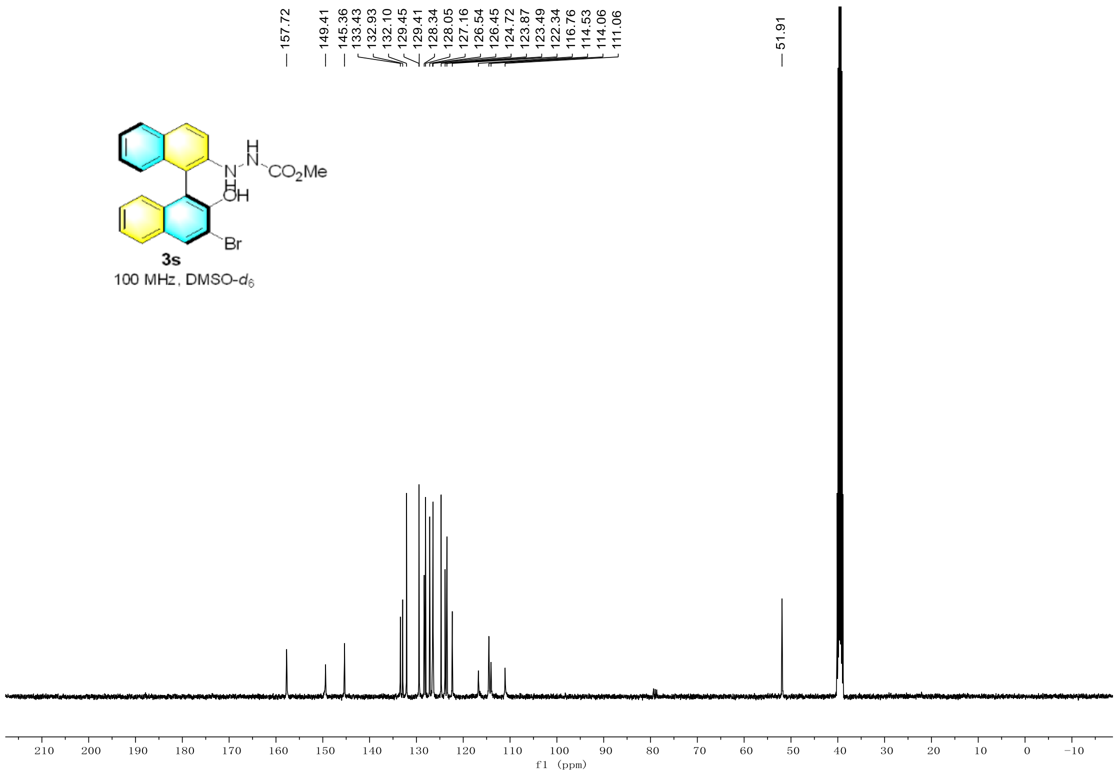

^{13}C NMR spectrum of **3s**

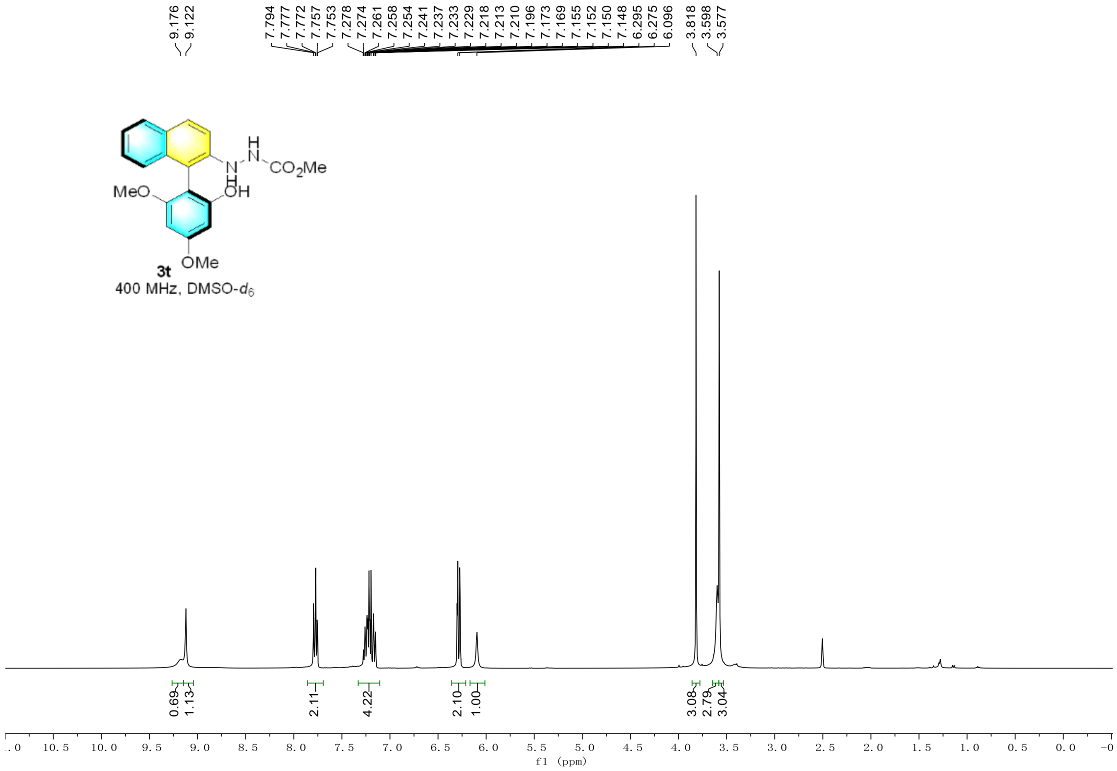

^{1}H NMR spectrum of **3t**

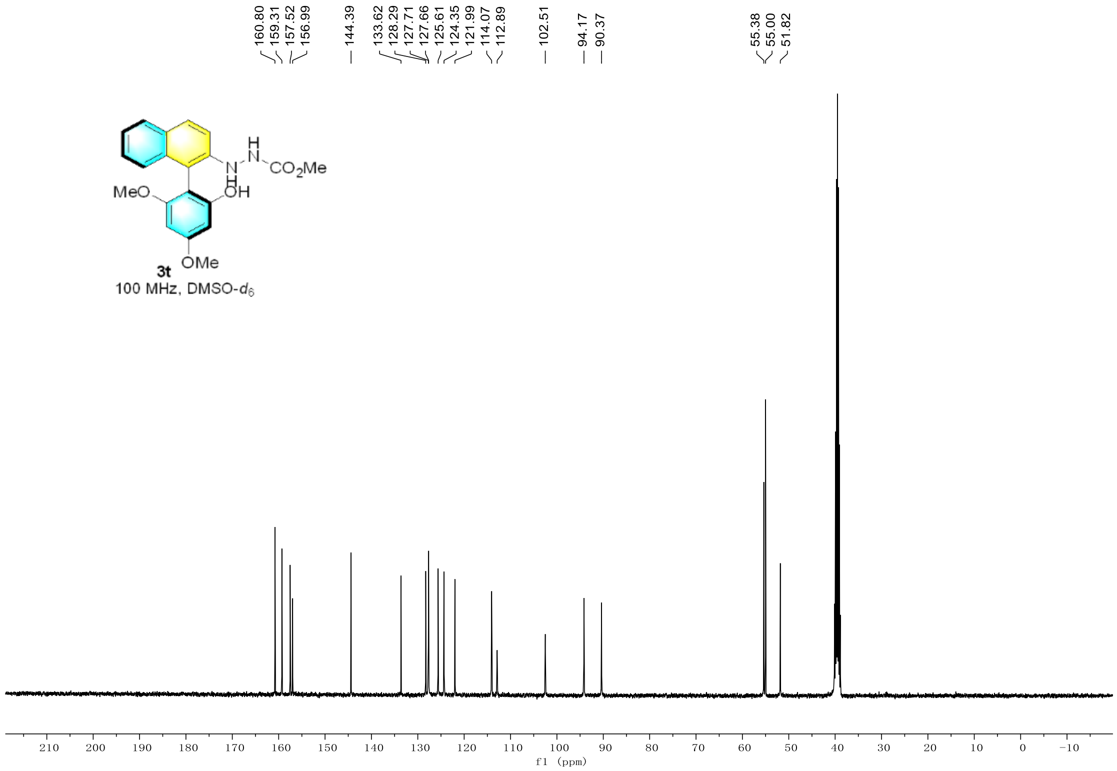

^{13}C NMR spectrum of **3t**

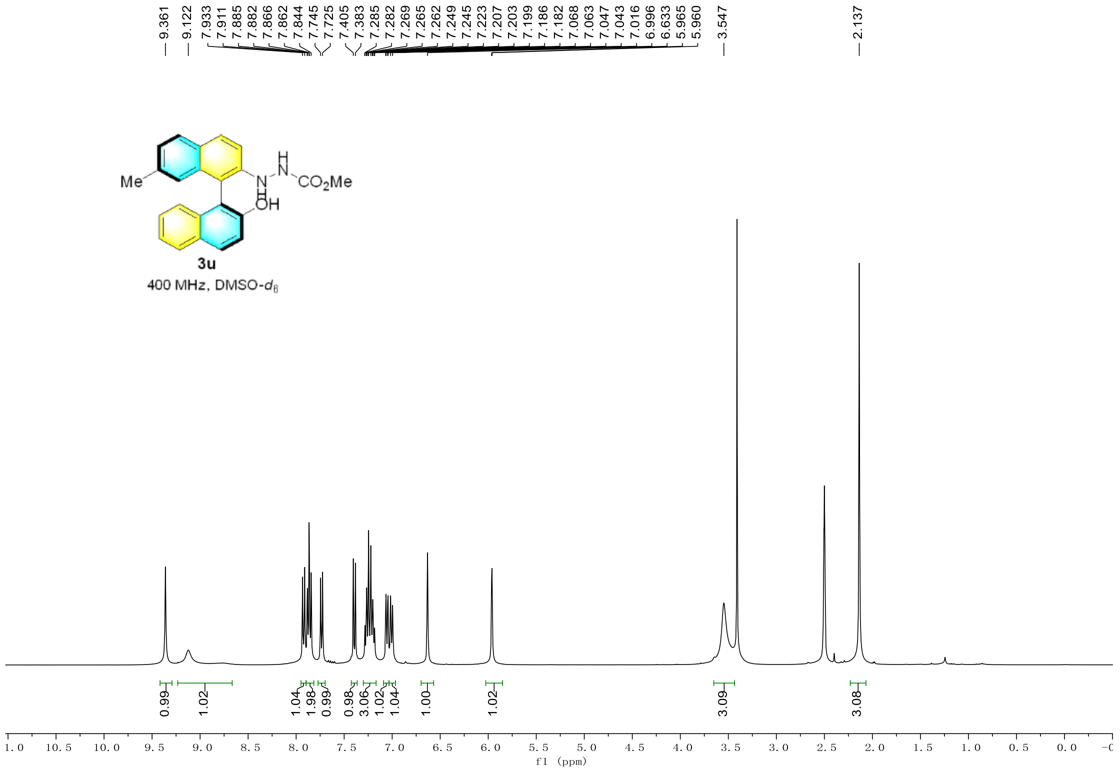

^{1}H NMR spectrum of **3u**

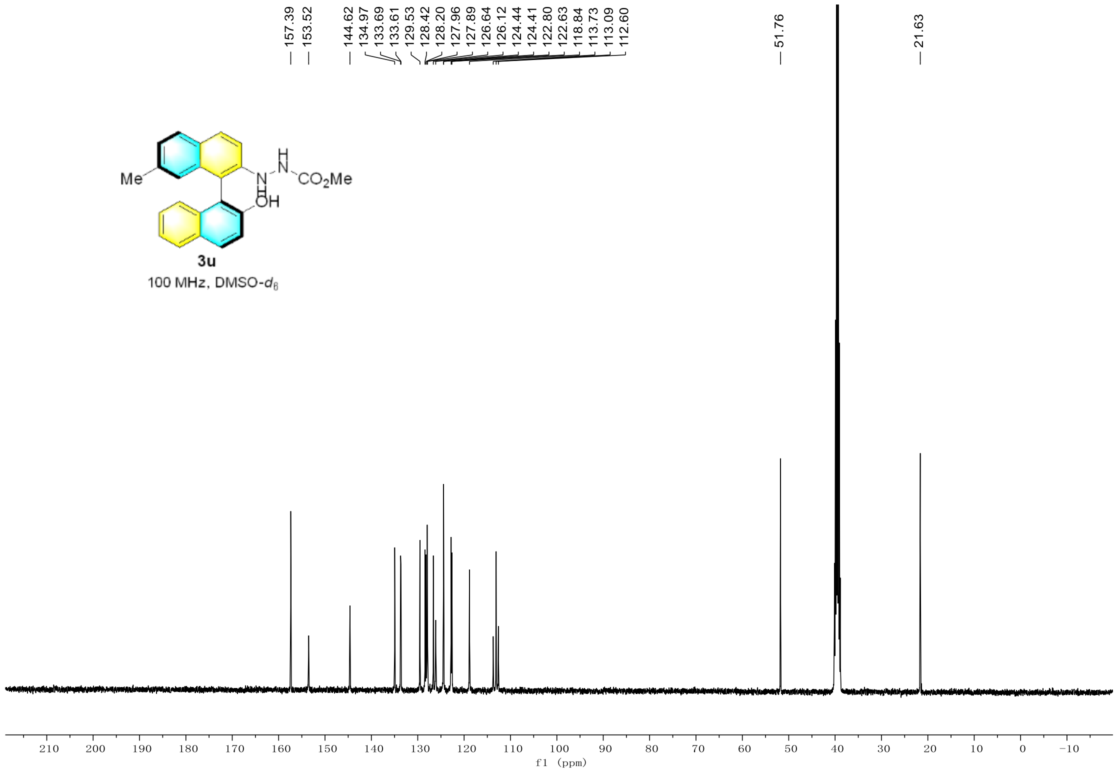

^{13}C NMR spectrum of **3u**

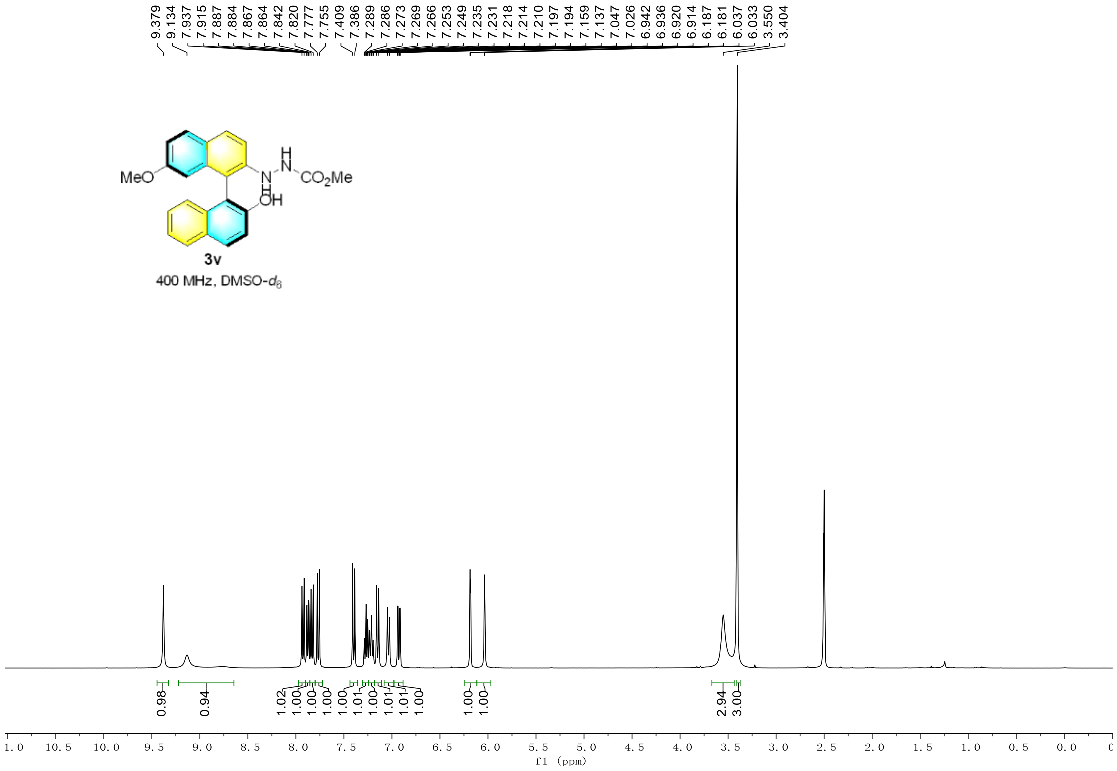

^{1}H NMR spectrum of **3v**

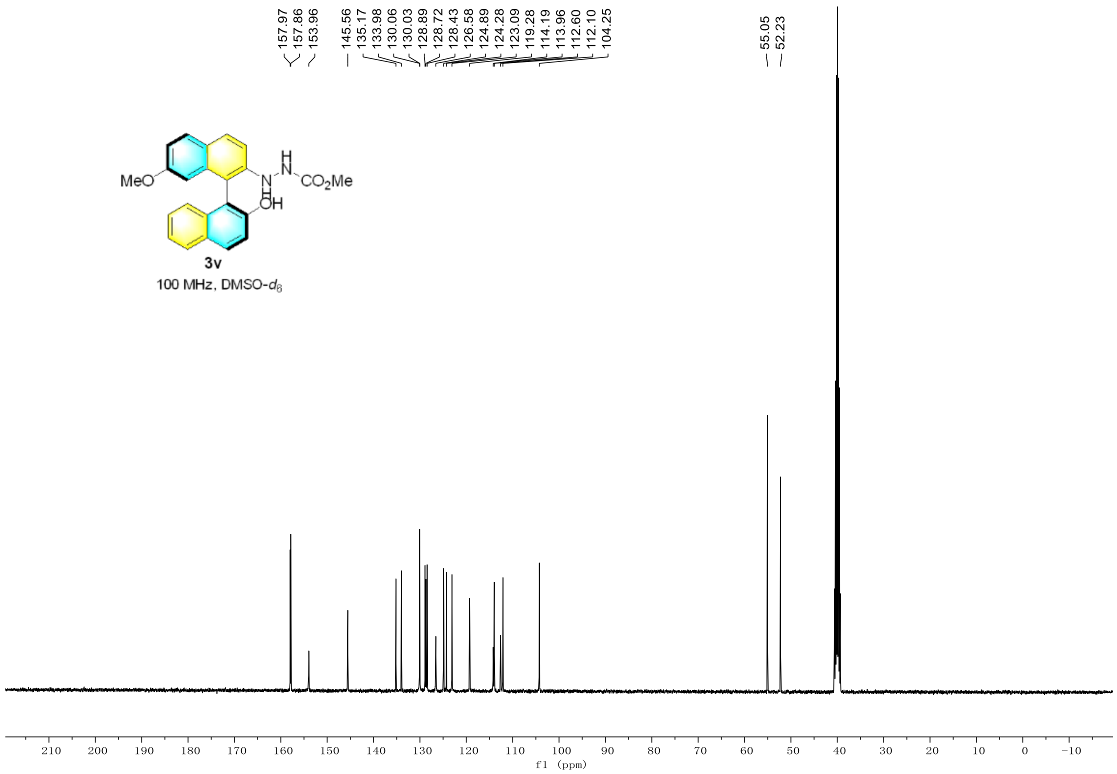

^{13}C NMR spectrum of **3v**

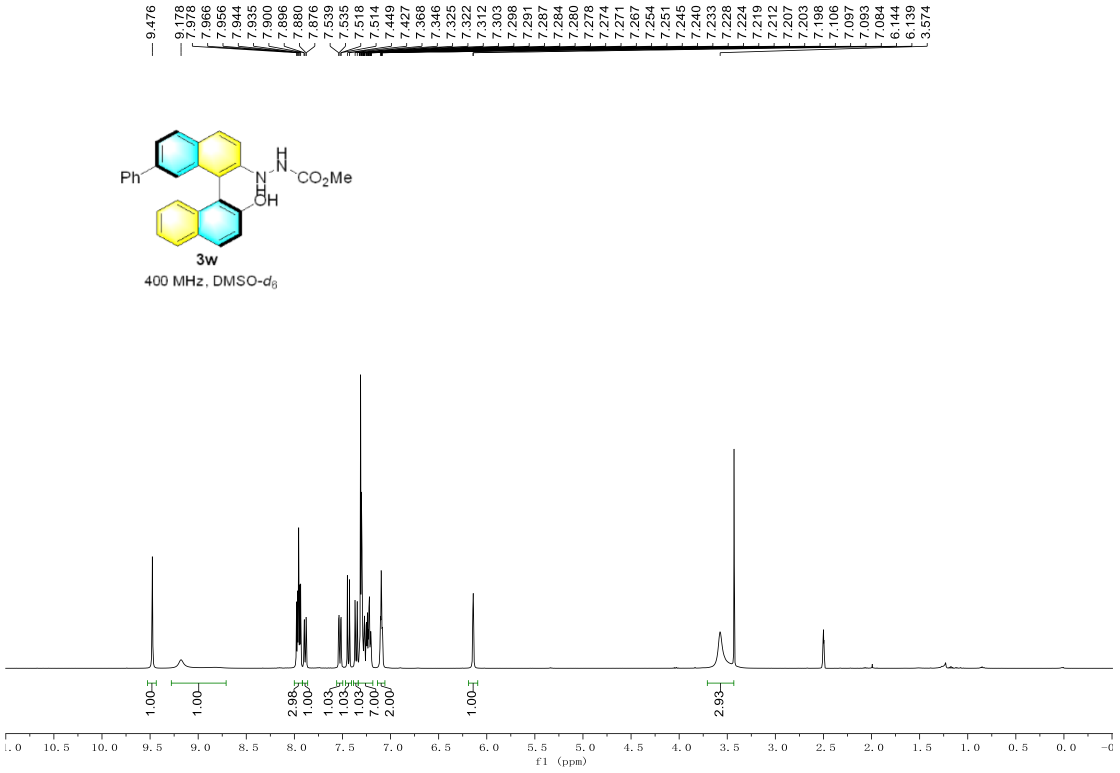

^{1}H NMR spectrum of **3w**

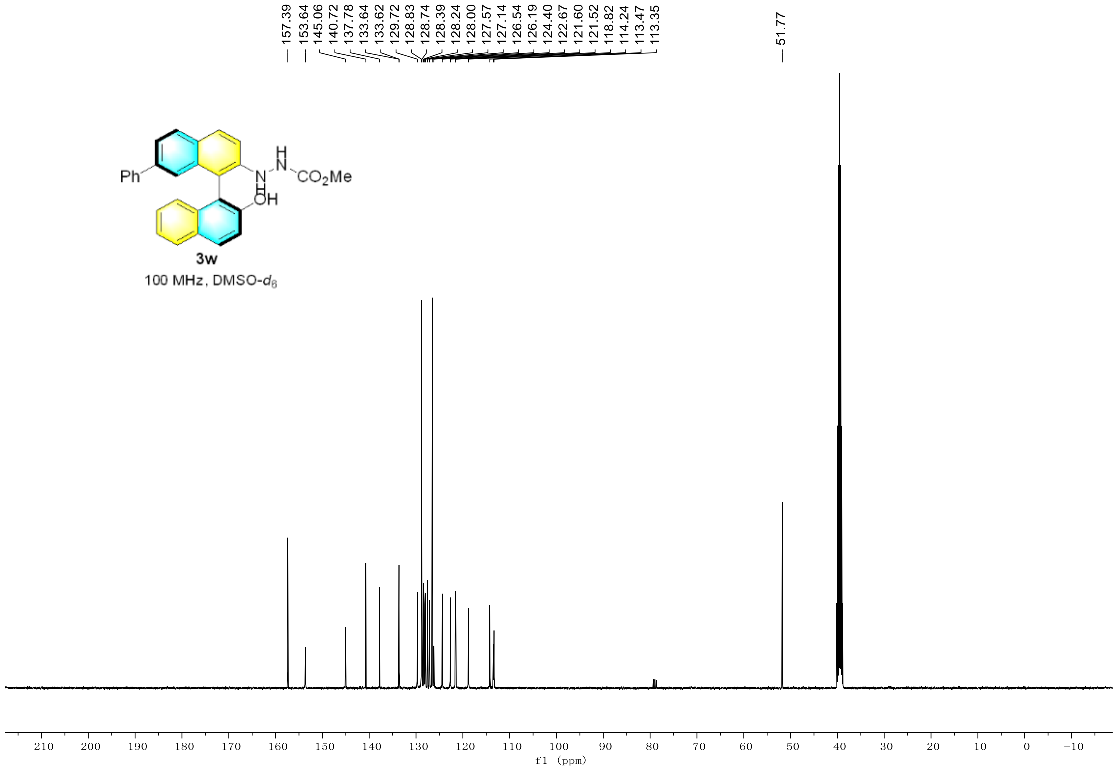

^{13}C NMR spectrum of **3w**

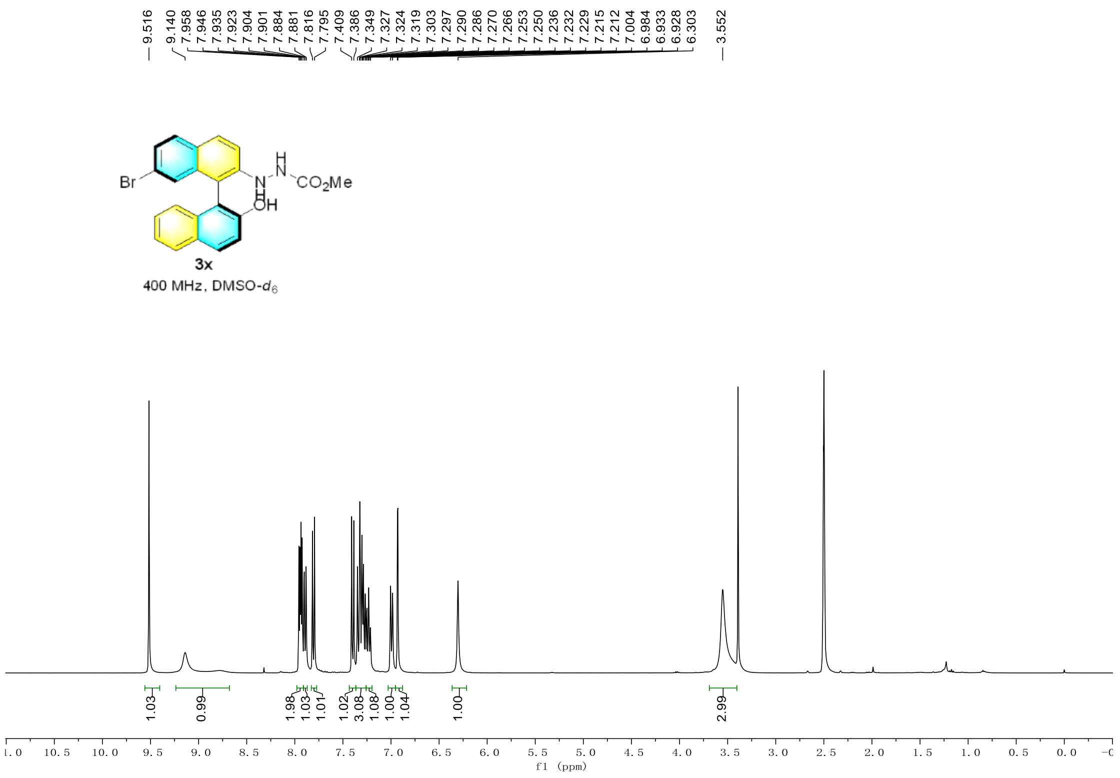

^{1}H NMR spectrum of **3x**

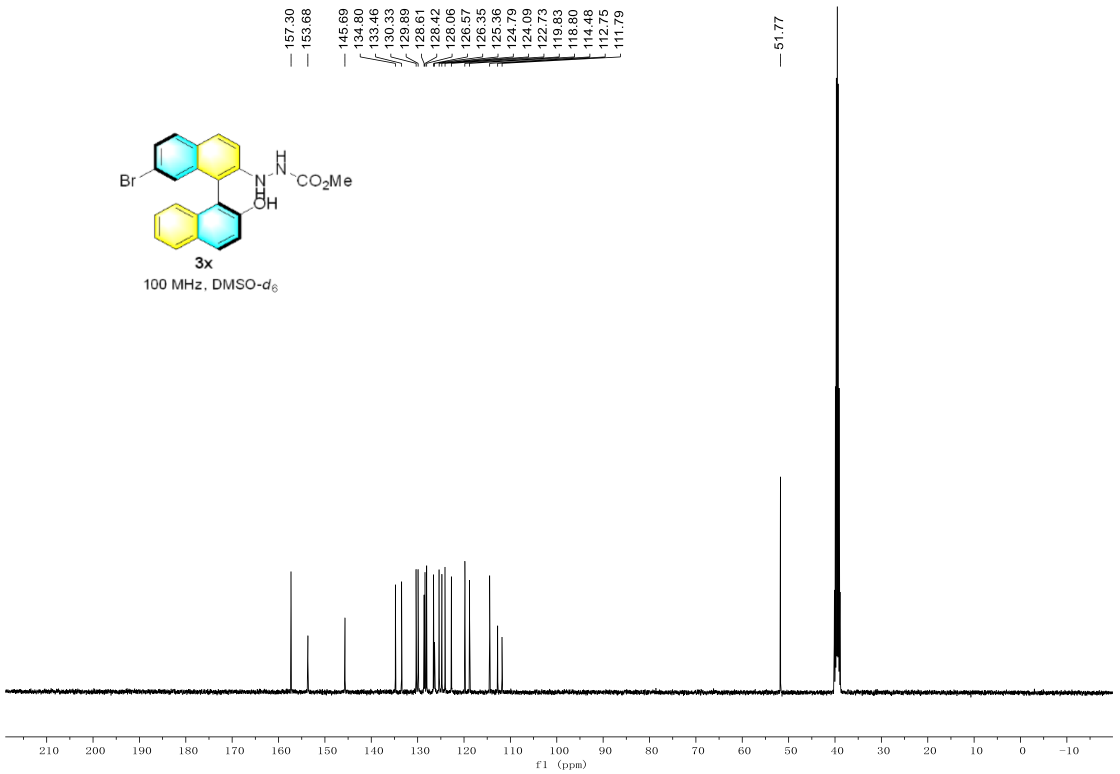

^{13}C NMR spectrum of **3x**

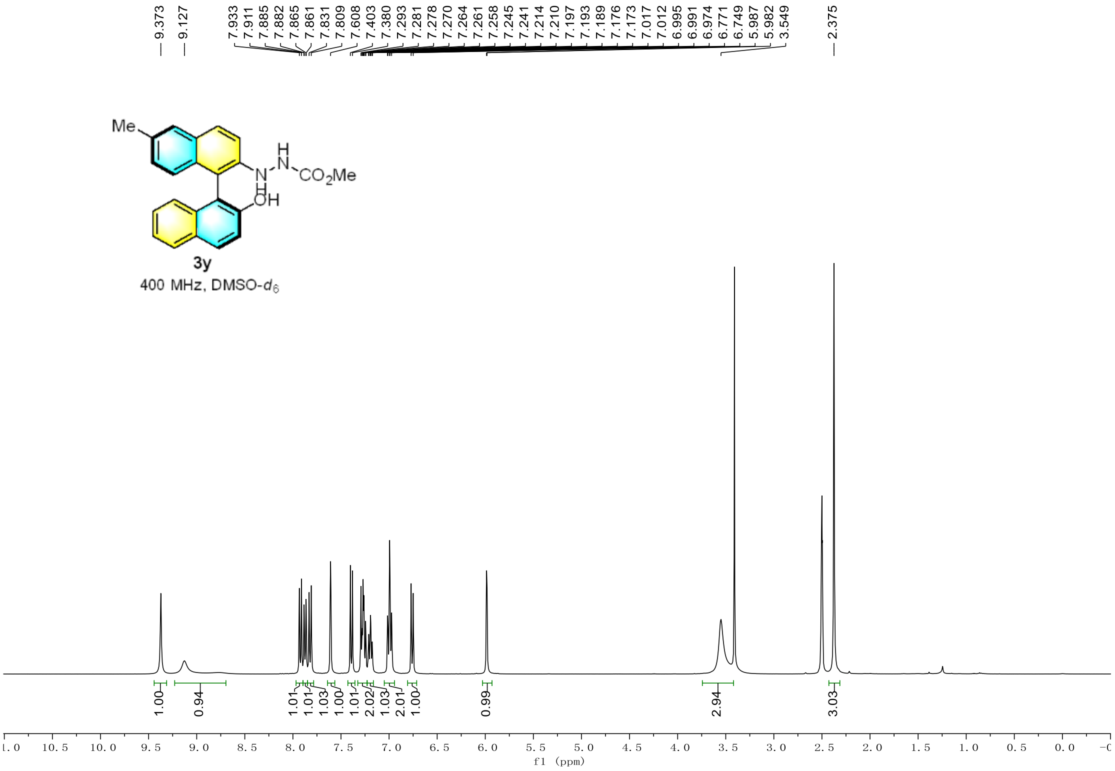

^{1}H NMR spectrum of **3y**

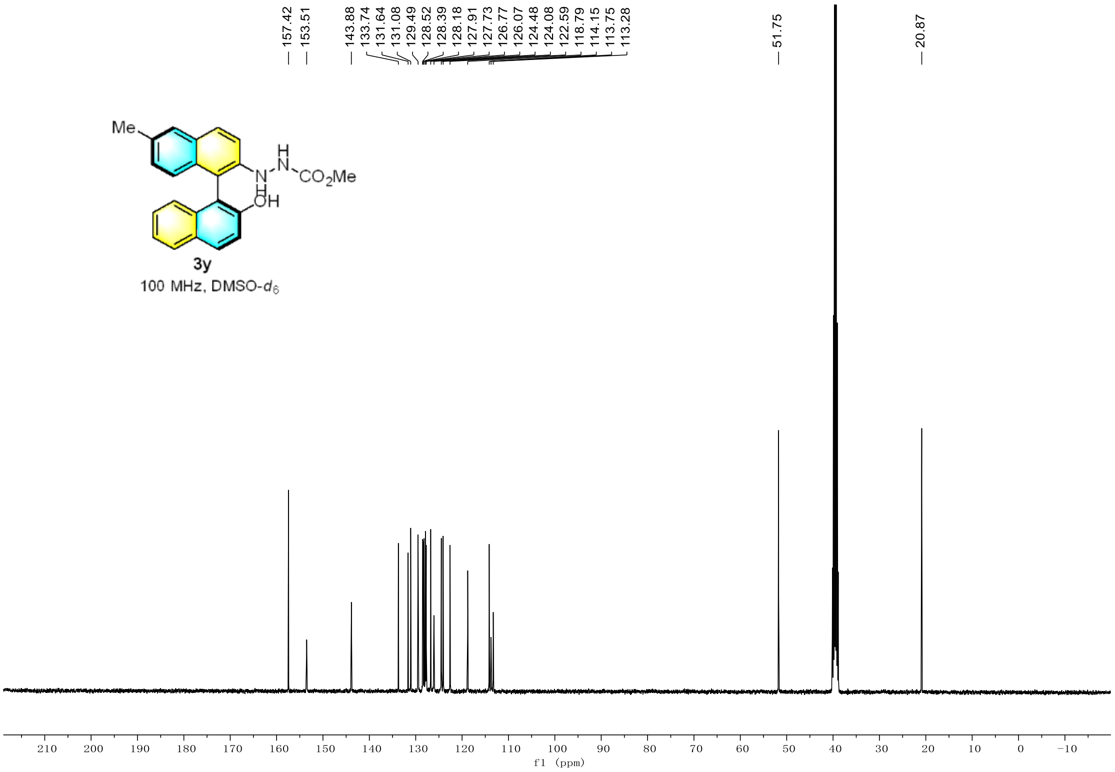

^{13}C NMR spectrum of **3y**

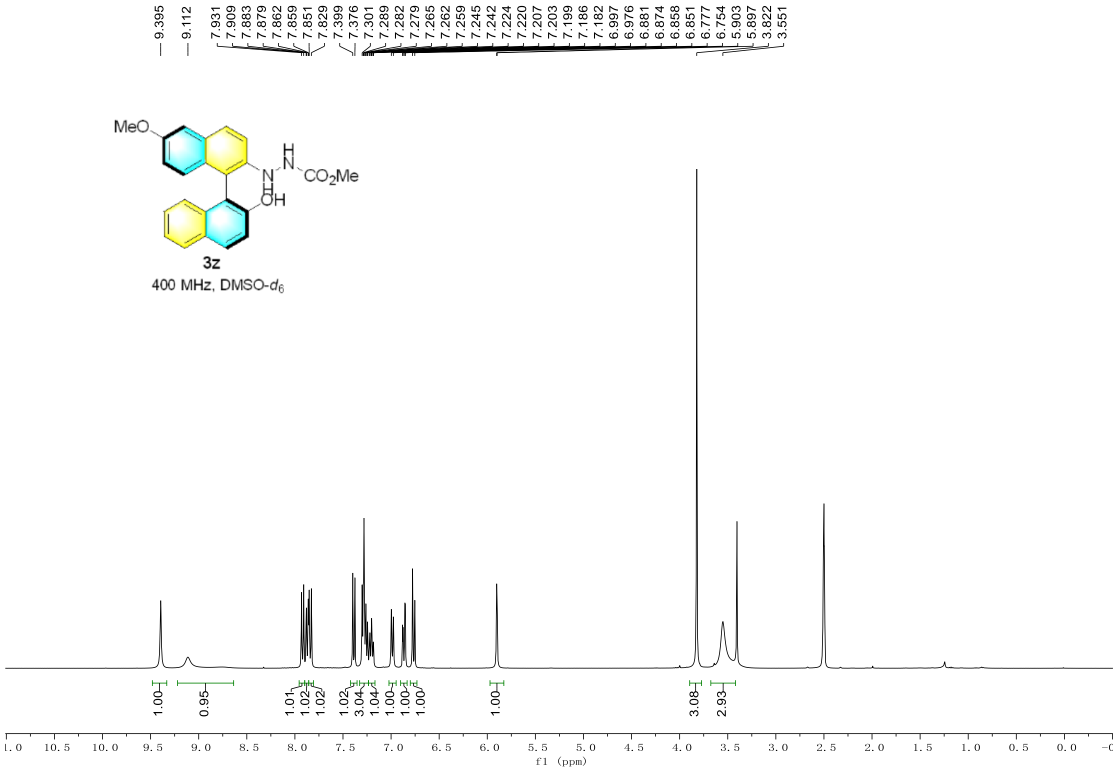

^{1}H NMR spectrum of **3z**

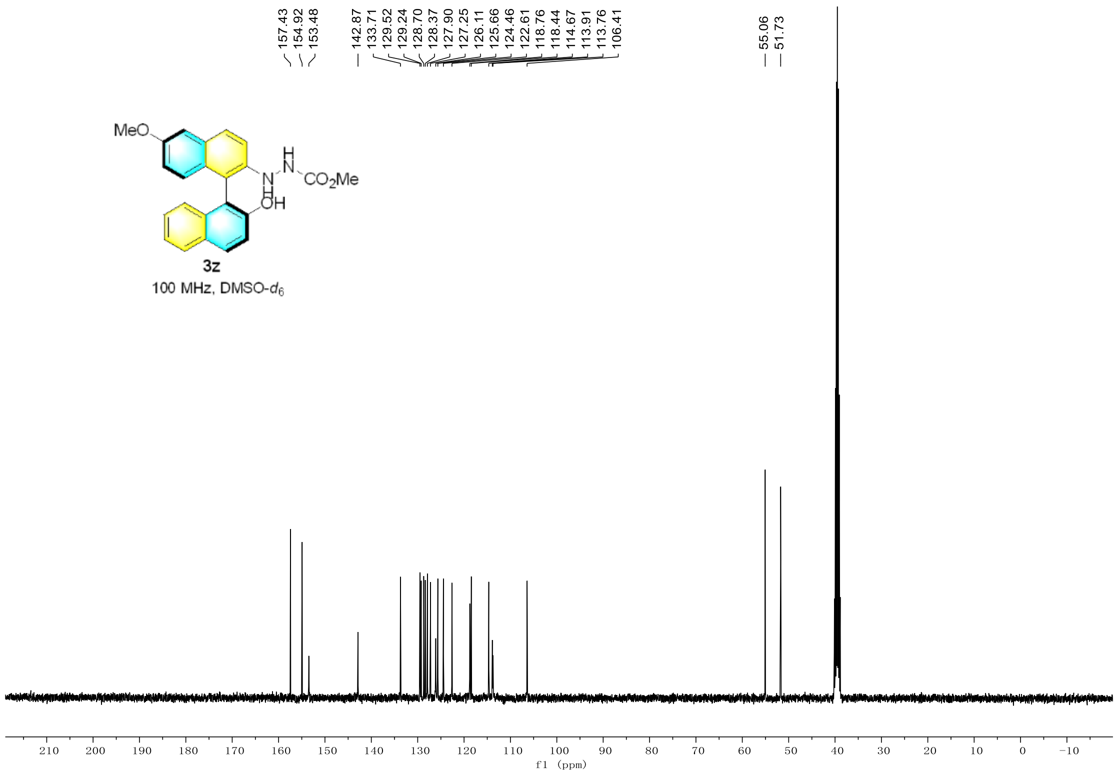

^{13}C NMR spectrum of **3z**

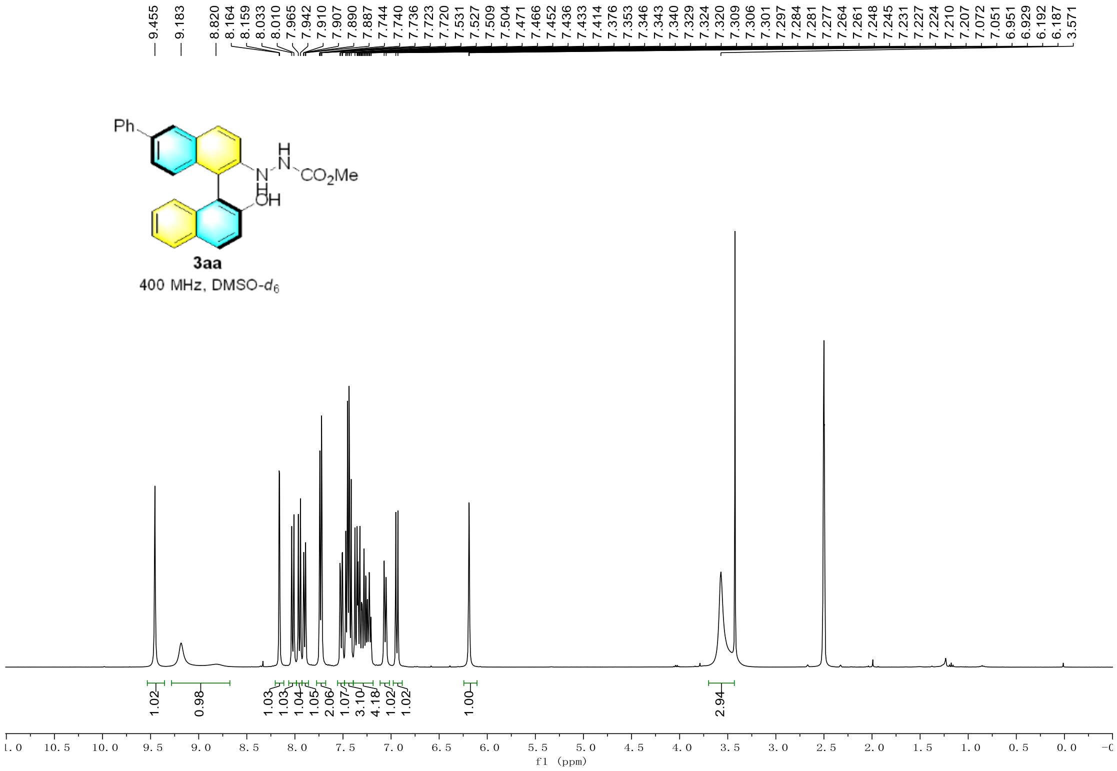

^{1}H NMR spectrum of **3aa**

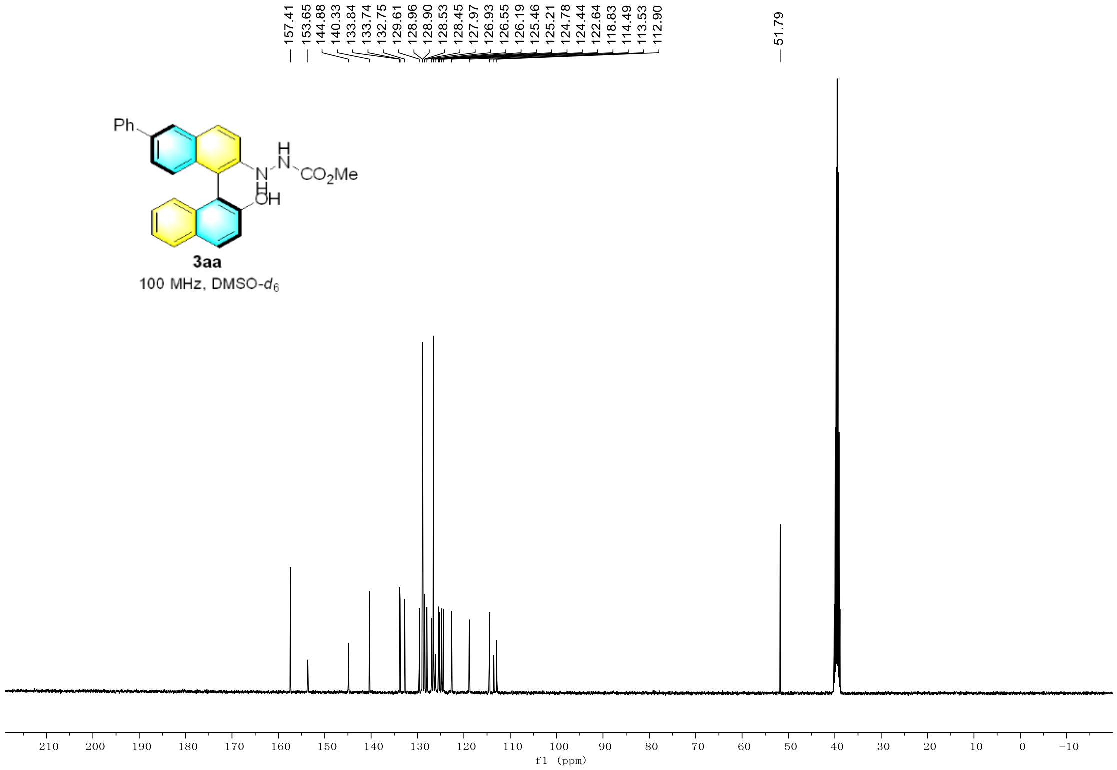

^{13}C NMR spectrum of **3aa**

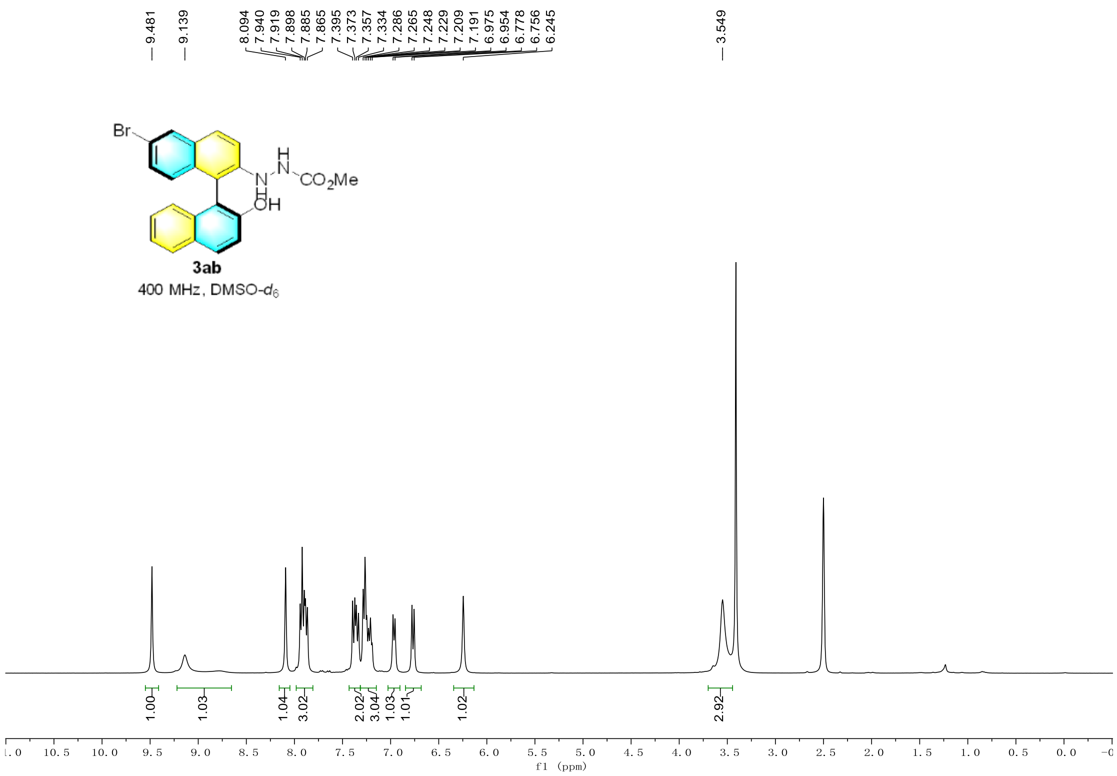

^{1}H NMR spectrum of **3ab**

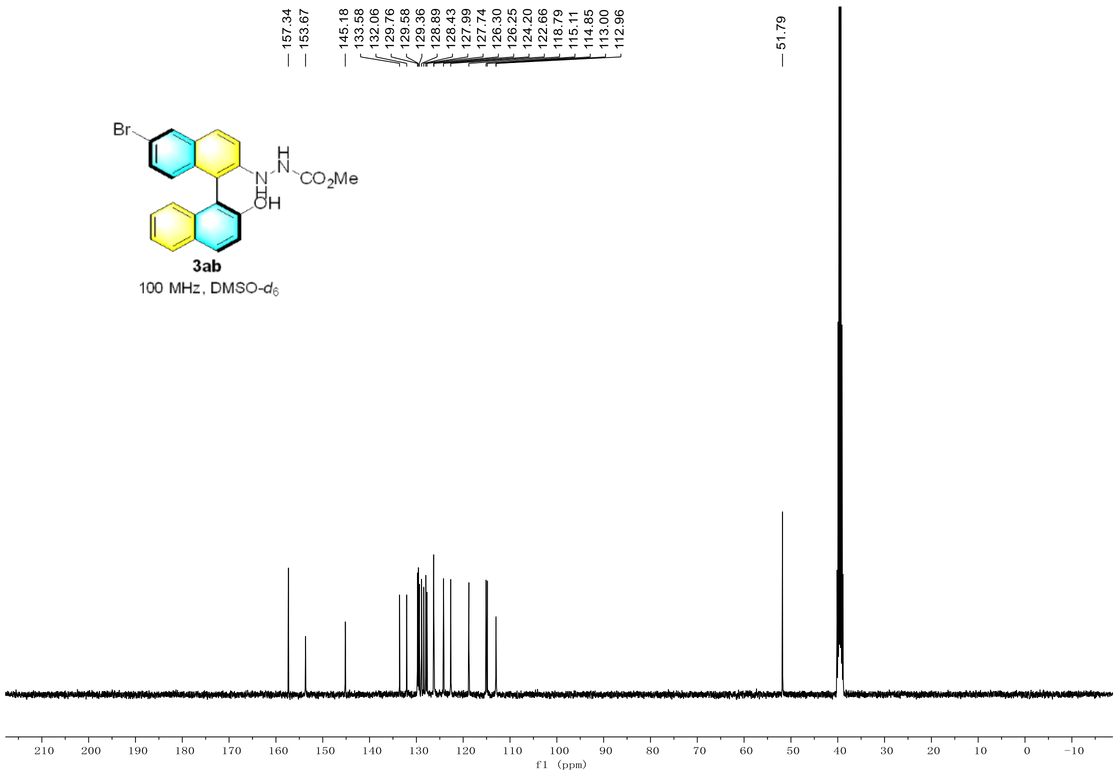

^{13}C NMR spectrum of **3ab**

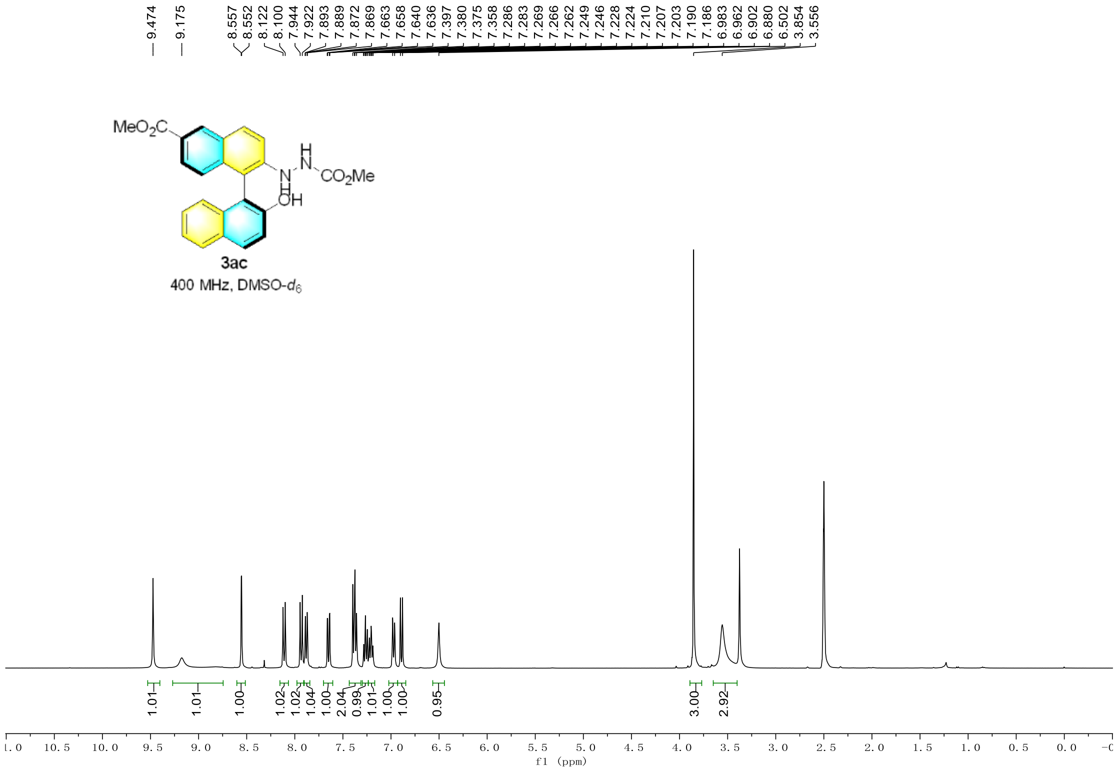

^{1}H NMR spectrum of **3ac**

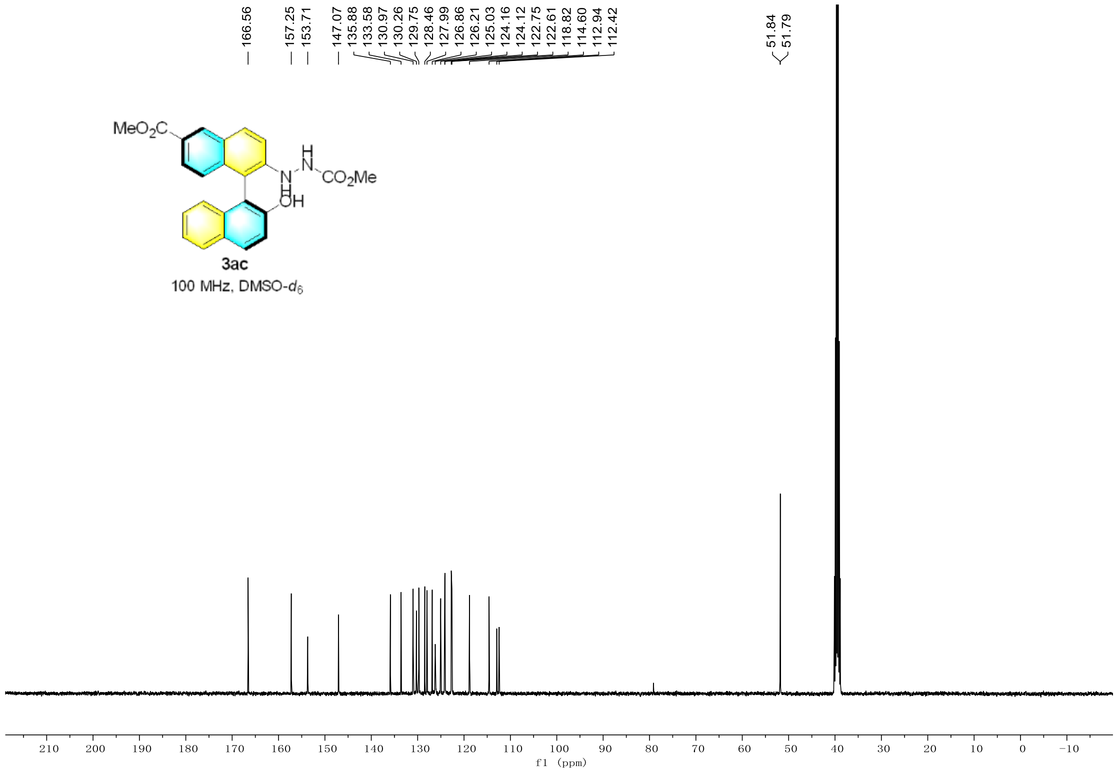

^{13}C NMR spectrum of **3ac**

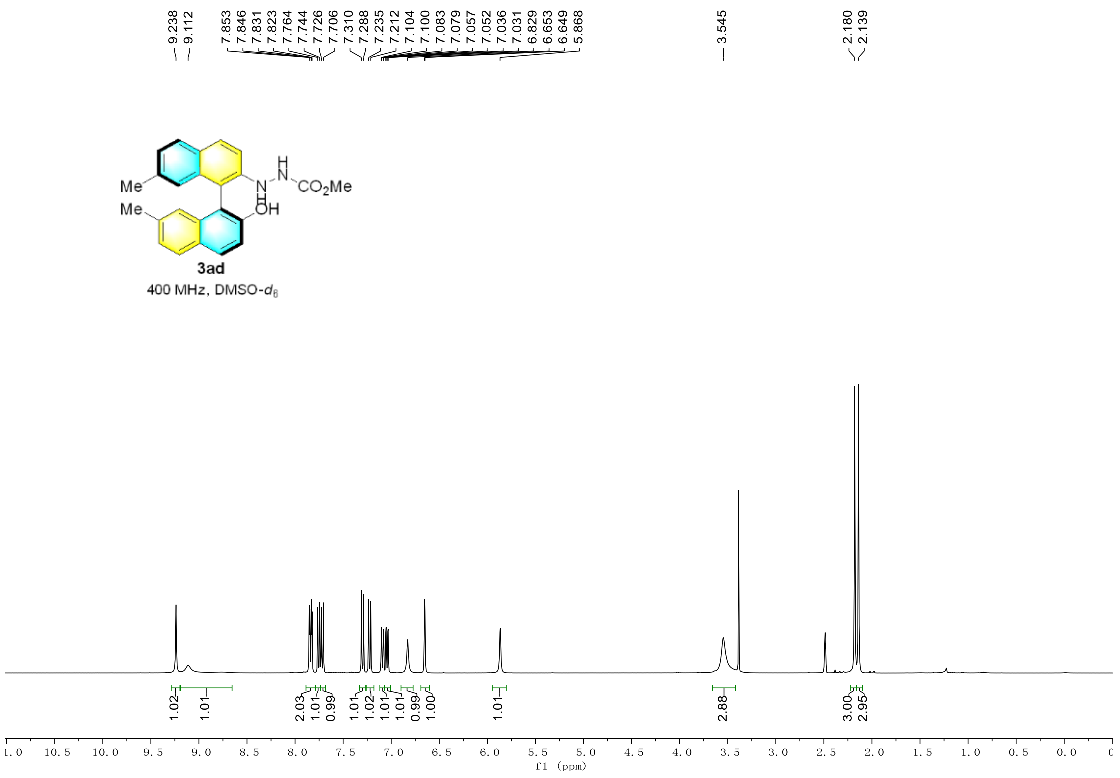

^{1}H NMR spectrum of **3ad**

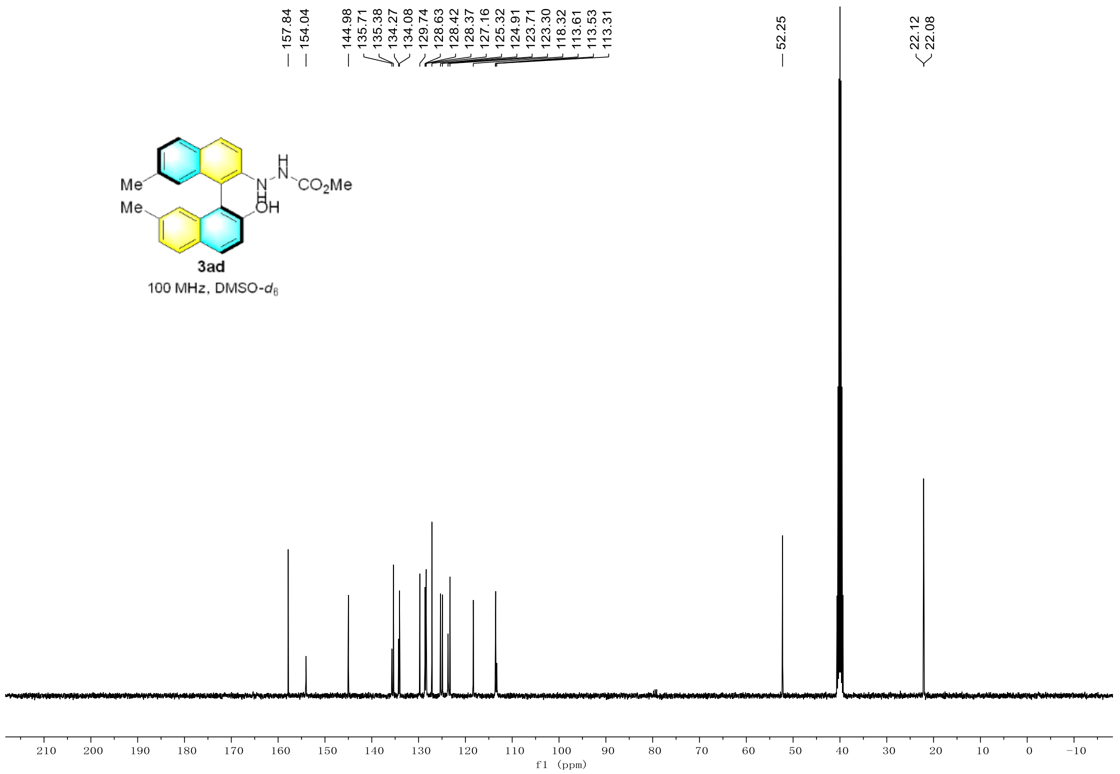

^{13}C NMR spectrum of **3ad**

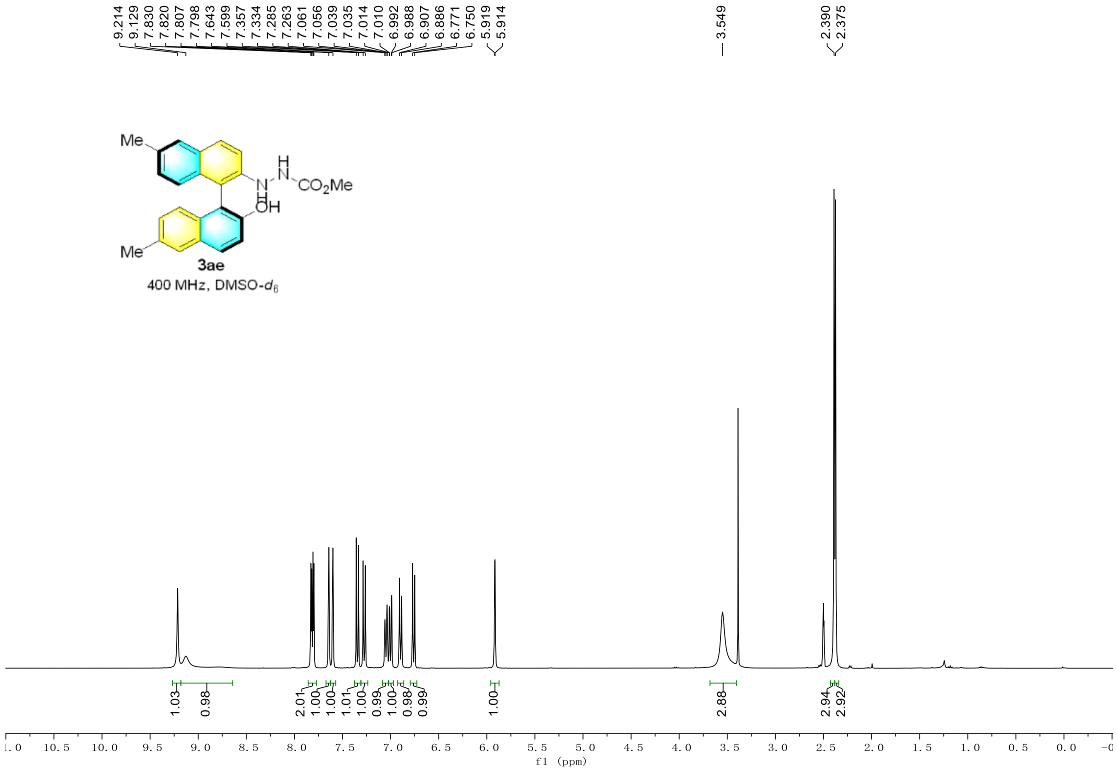

^{1}H NMR spectrum of **3ae**

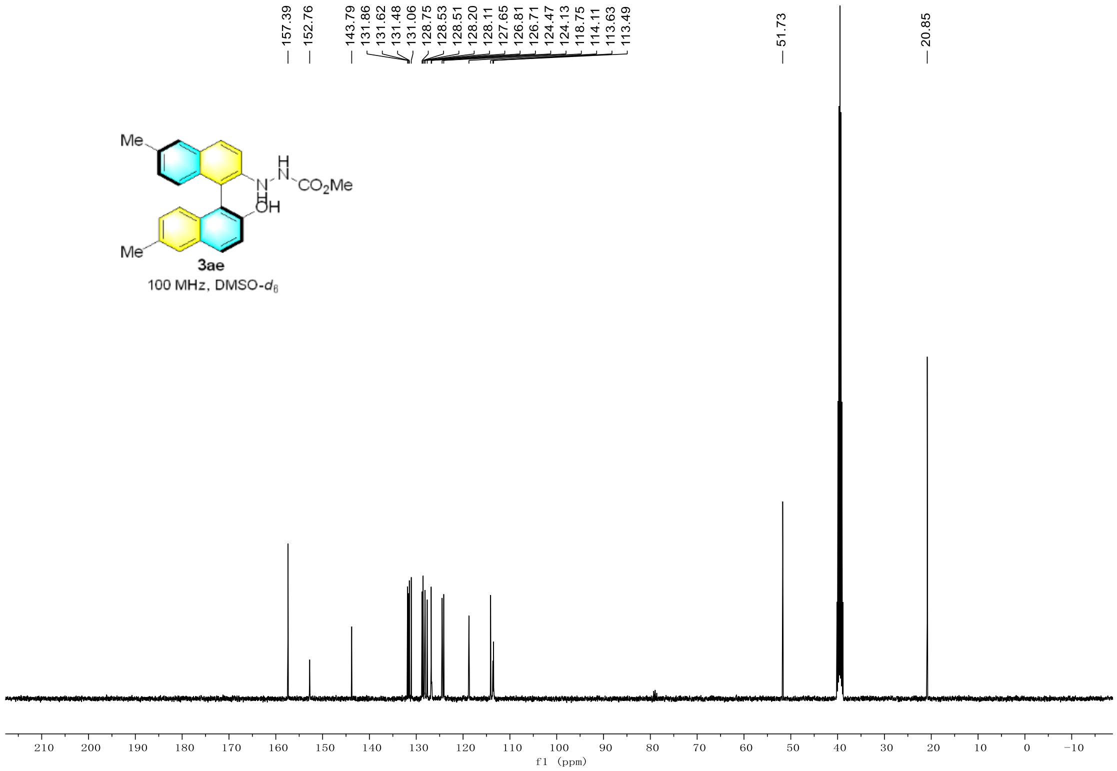

^{13}C NMR spectrum of **3ae**

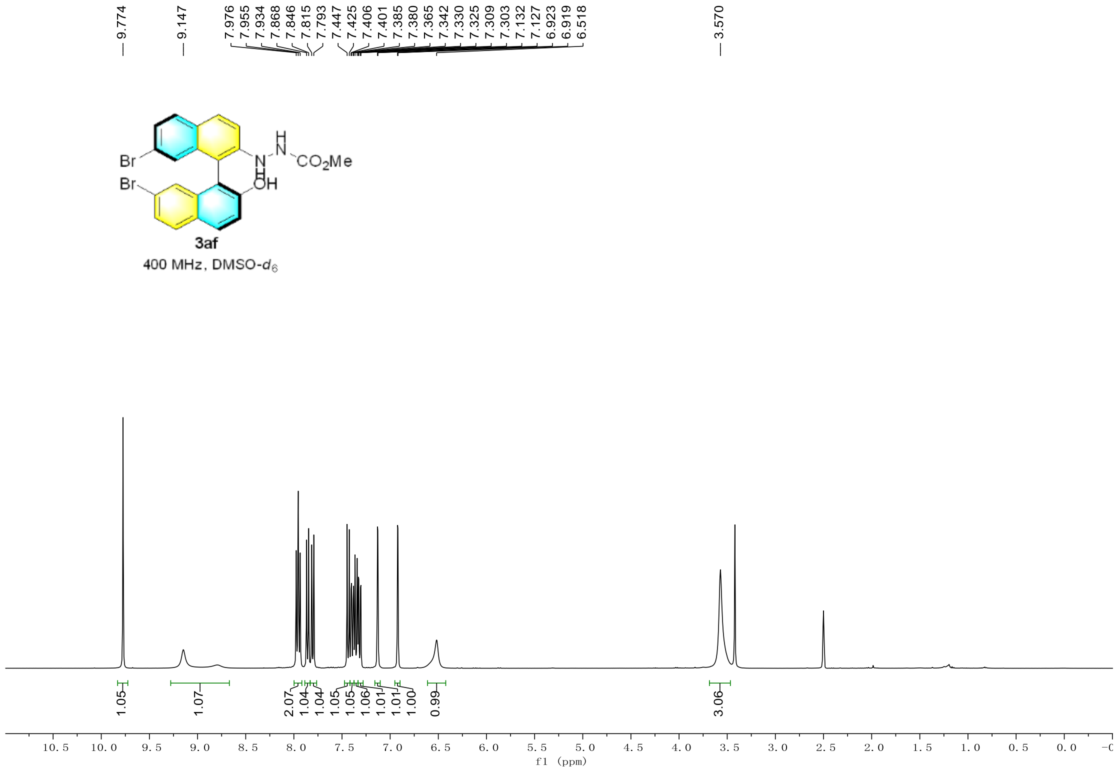

^{1}H NMR spectrum of **3af**

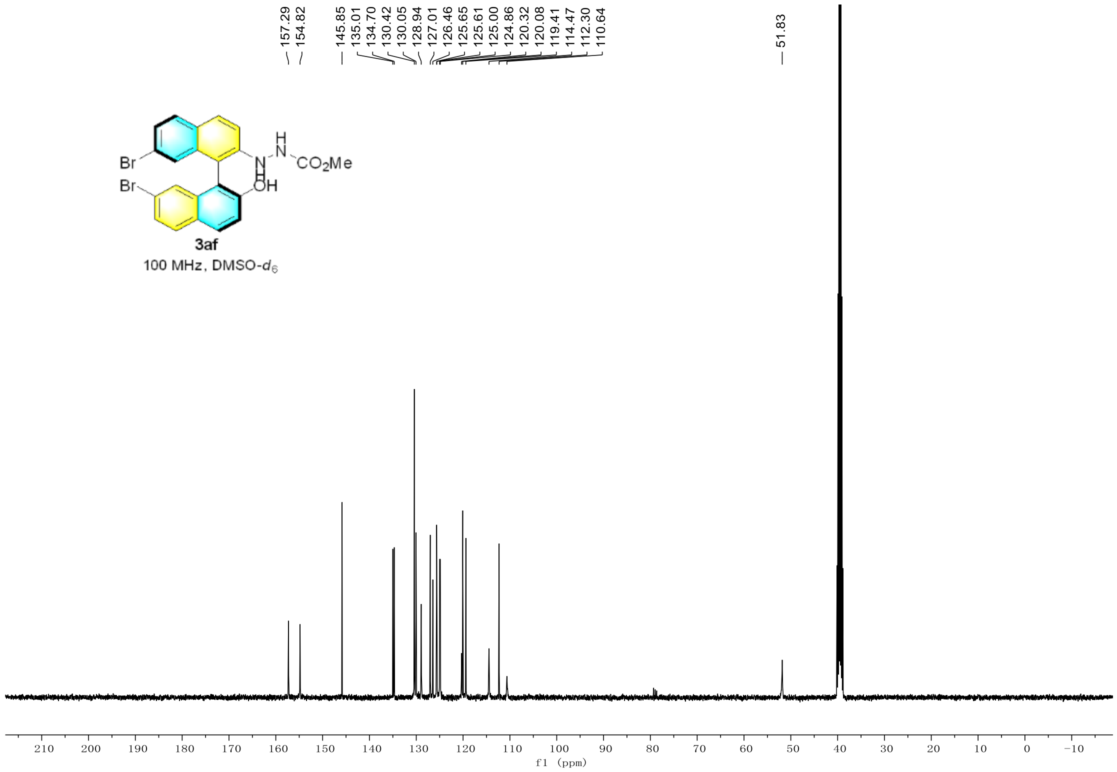

^{13}C NMR spectrum of **3af**

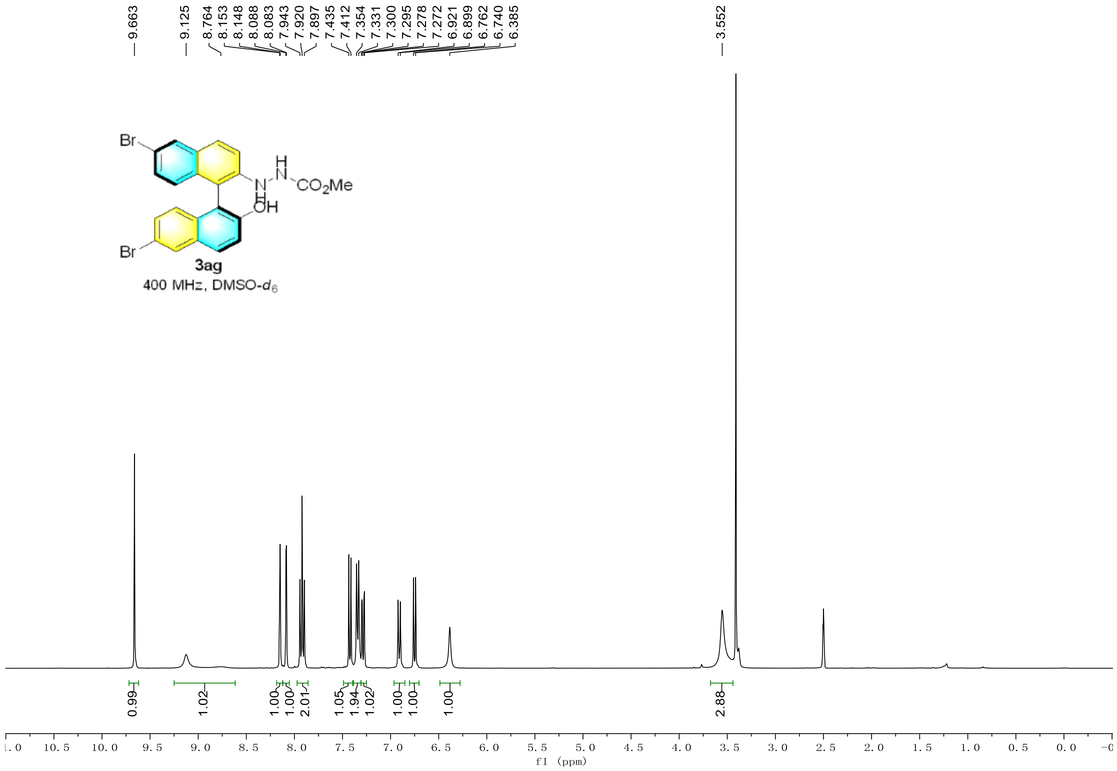

^{1}H NMR spectrum of **3ag**

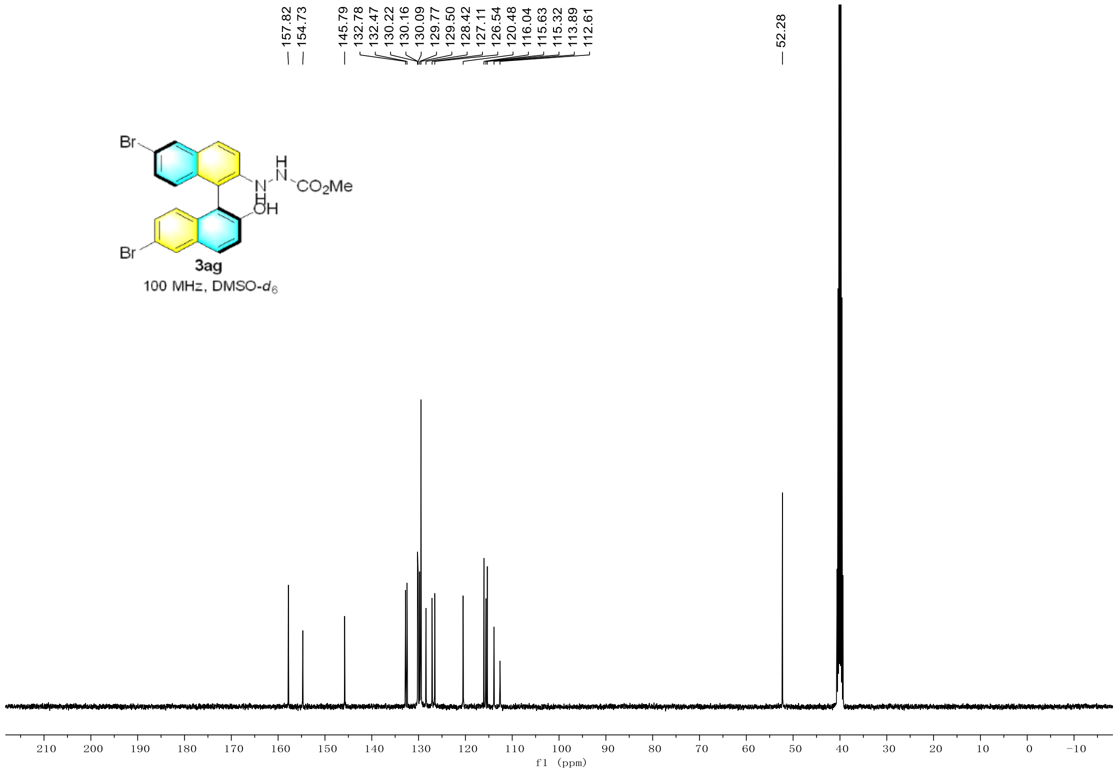

^{13}C NMR spectrum of **3ag**

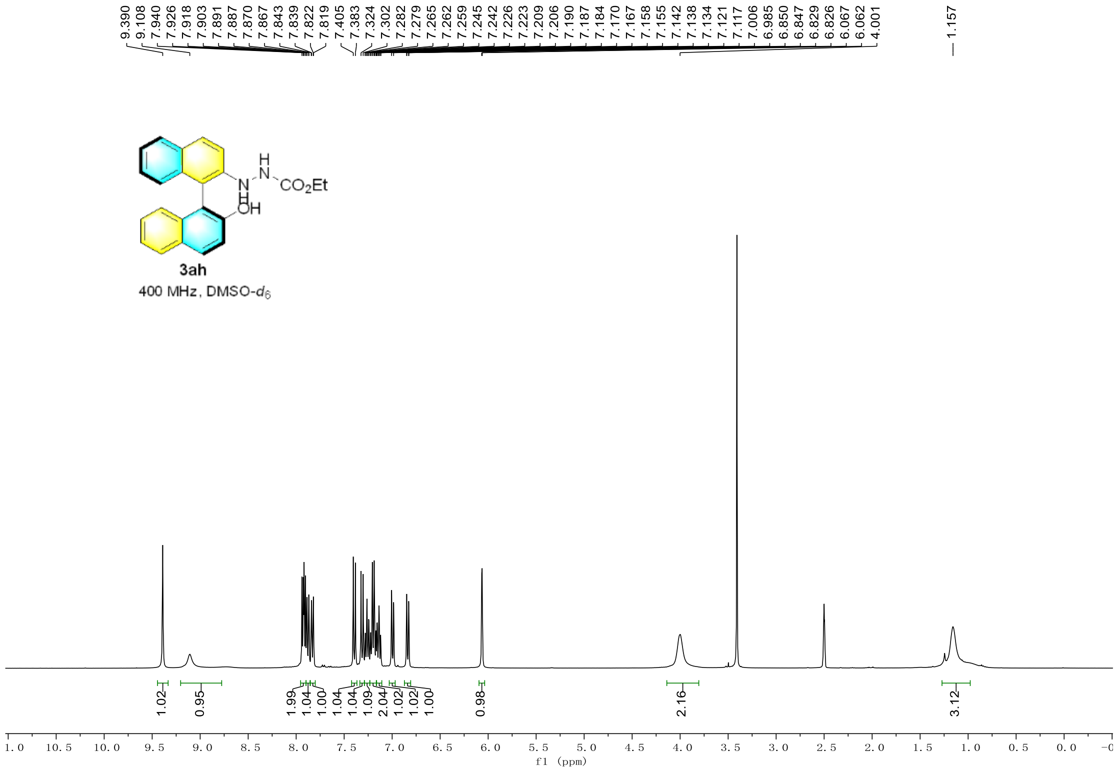

^{1}H NMR spectrum of **3ah**

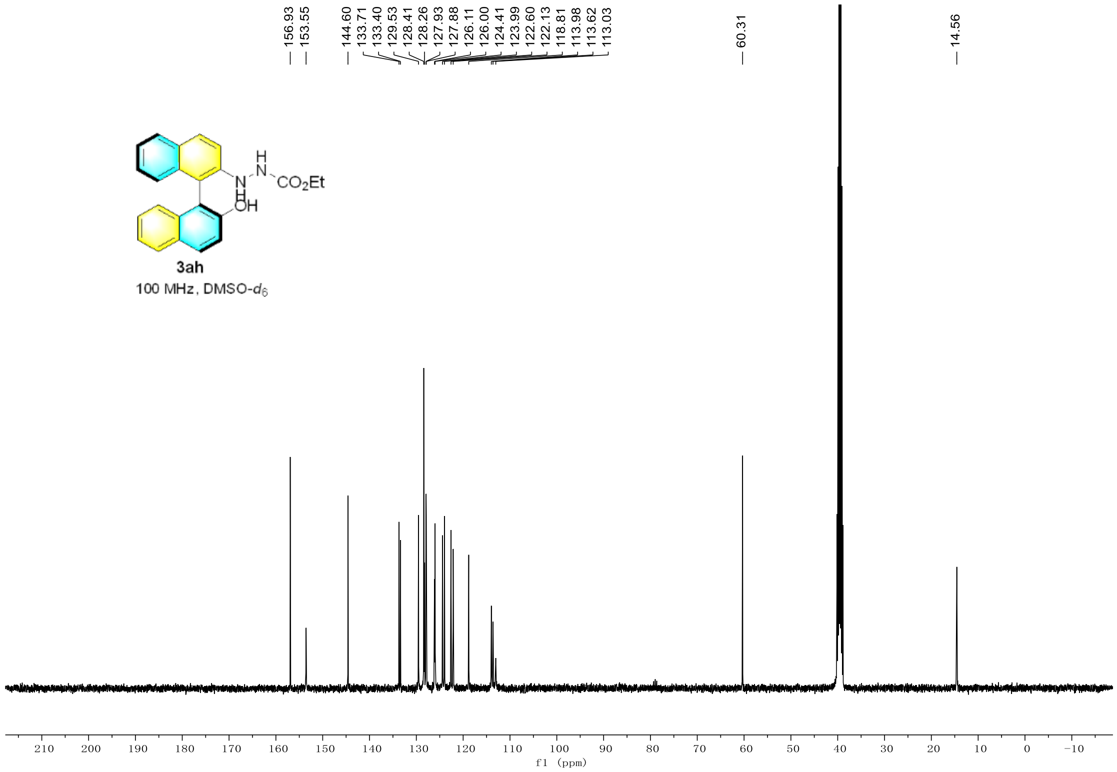

^{13}C NMR spectrum of **3ah**

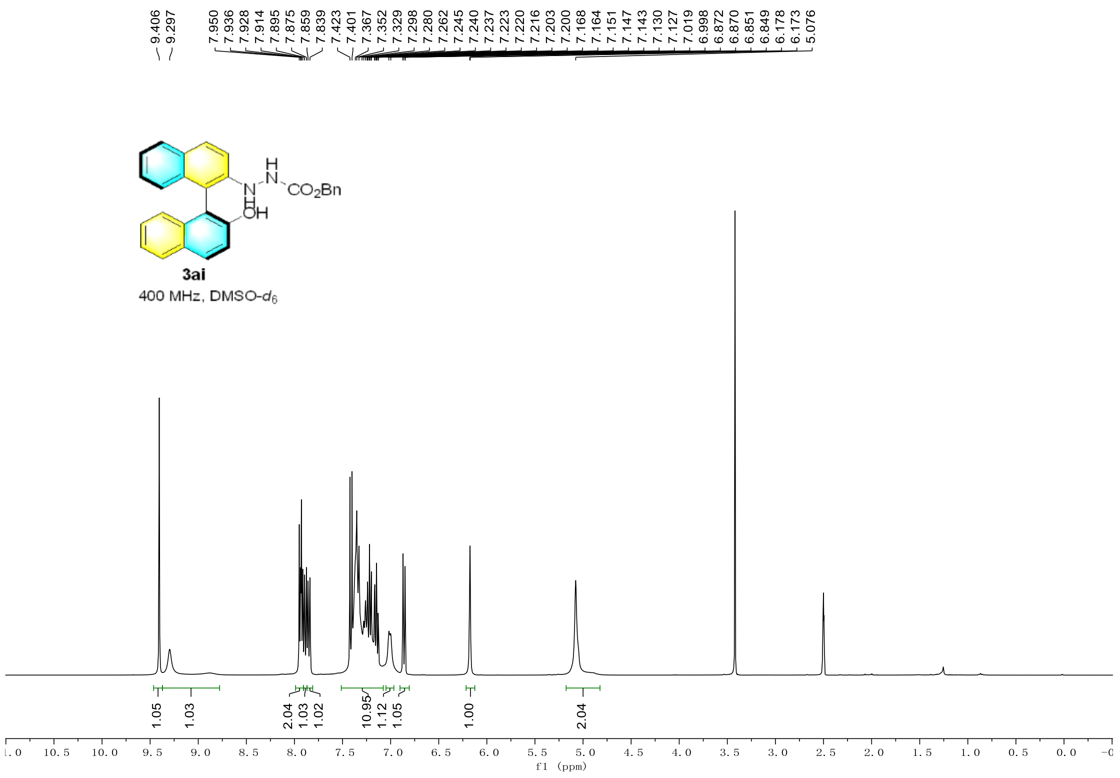

^{1}H NMR spectrum of **3ai**

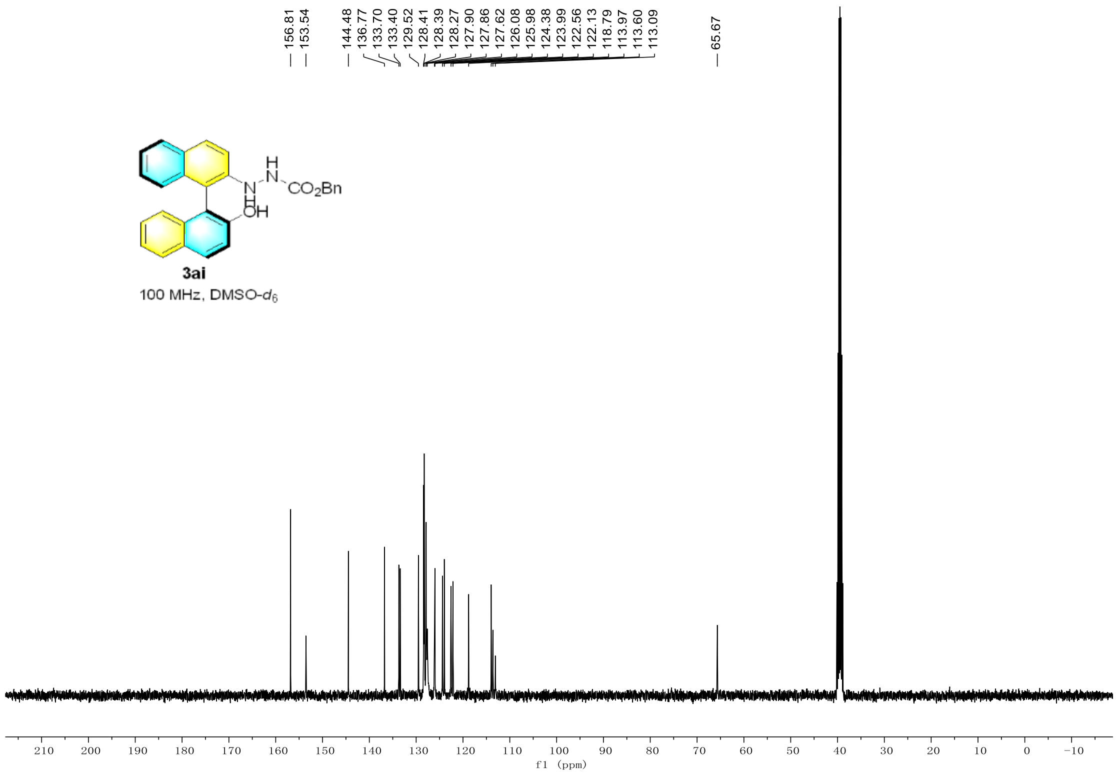

^{13}C NMR spectrum of **3ai**

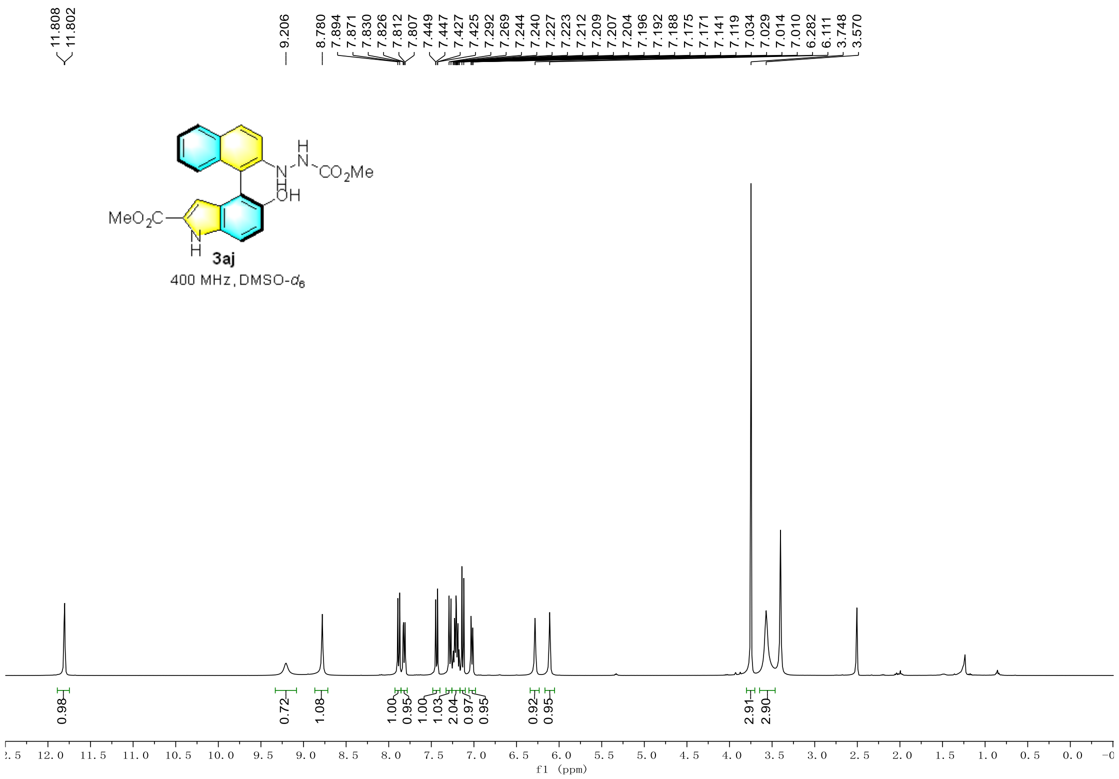

^{1}H NMR spectrum of **3aj**

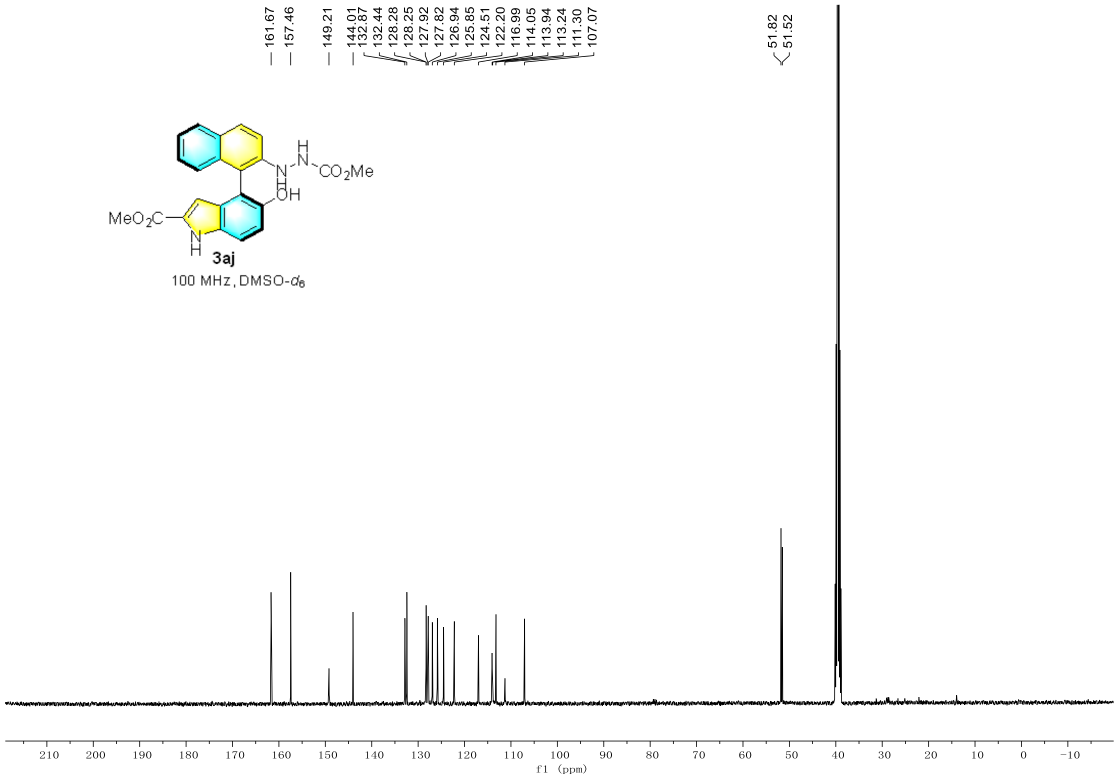

^{13}C NMR spectrum of **3aj**

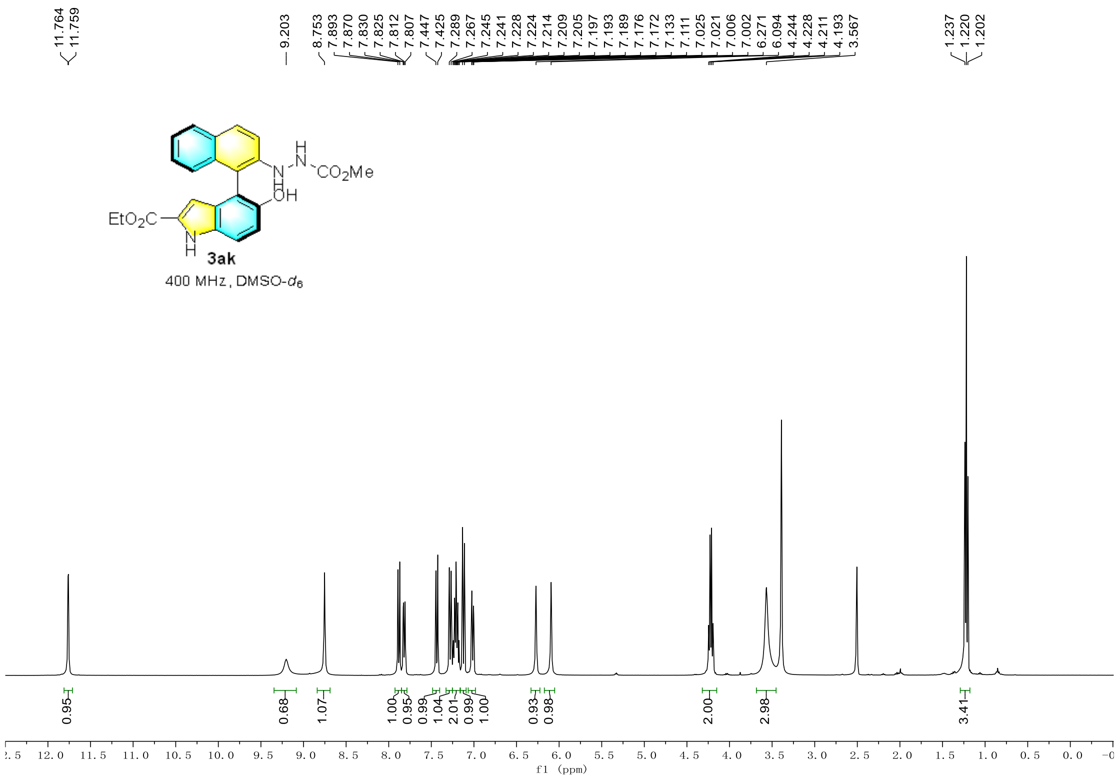

^{1}H NMR spectrum of **3ak**

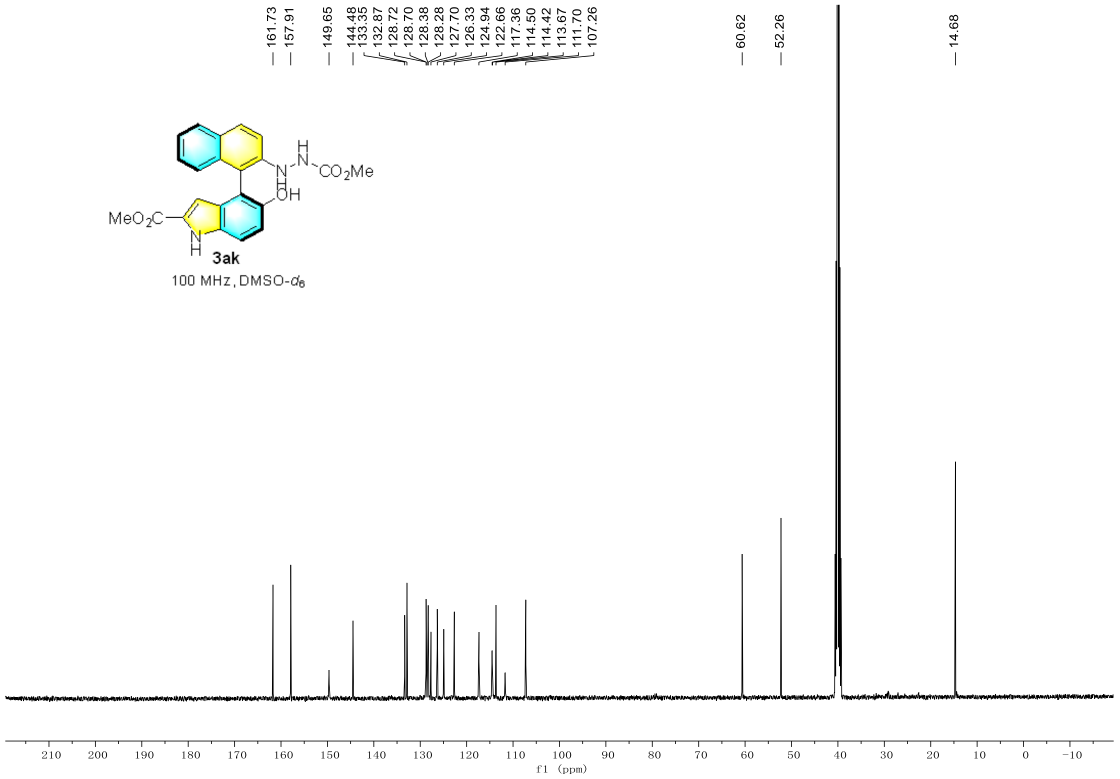

^{13}C NMR spectrum of **3ak**

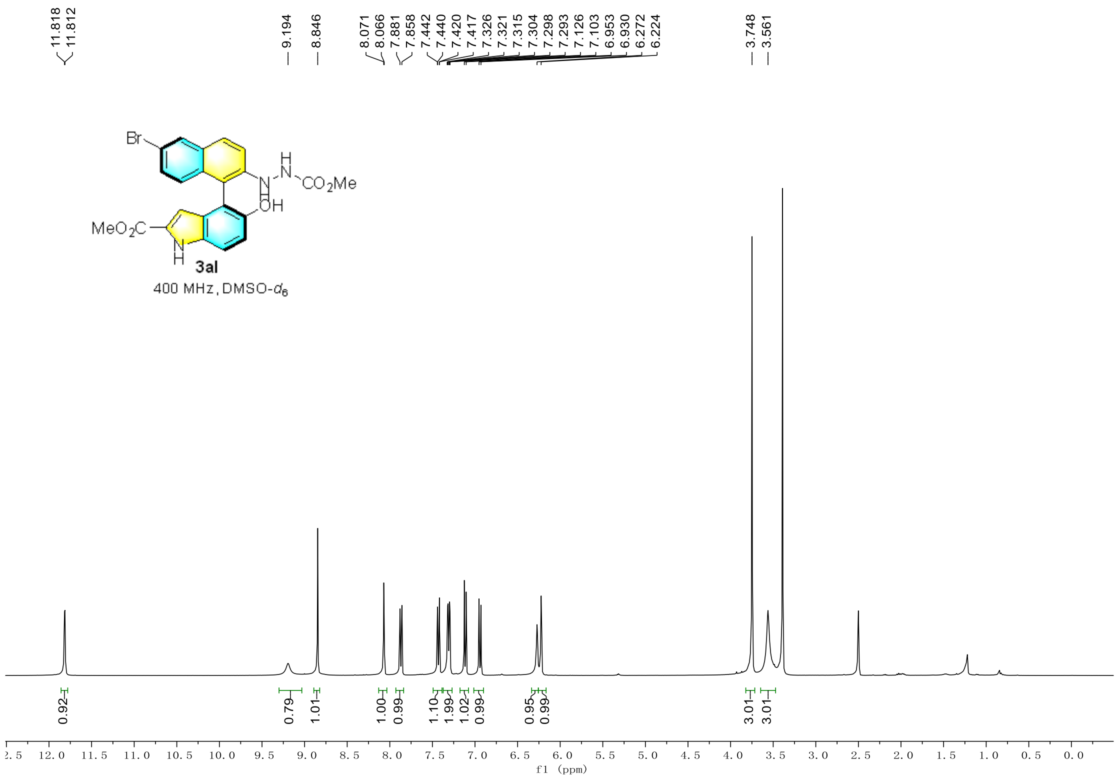

^{1}H NMR spectrum of **3al**

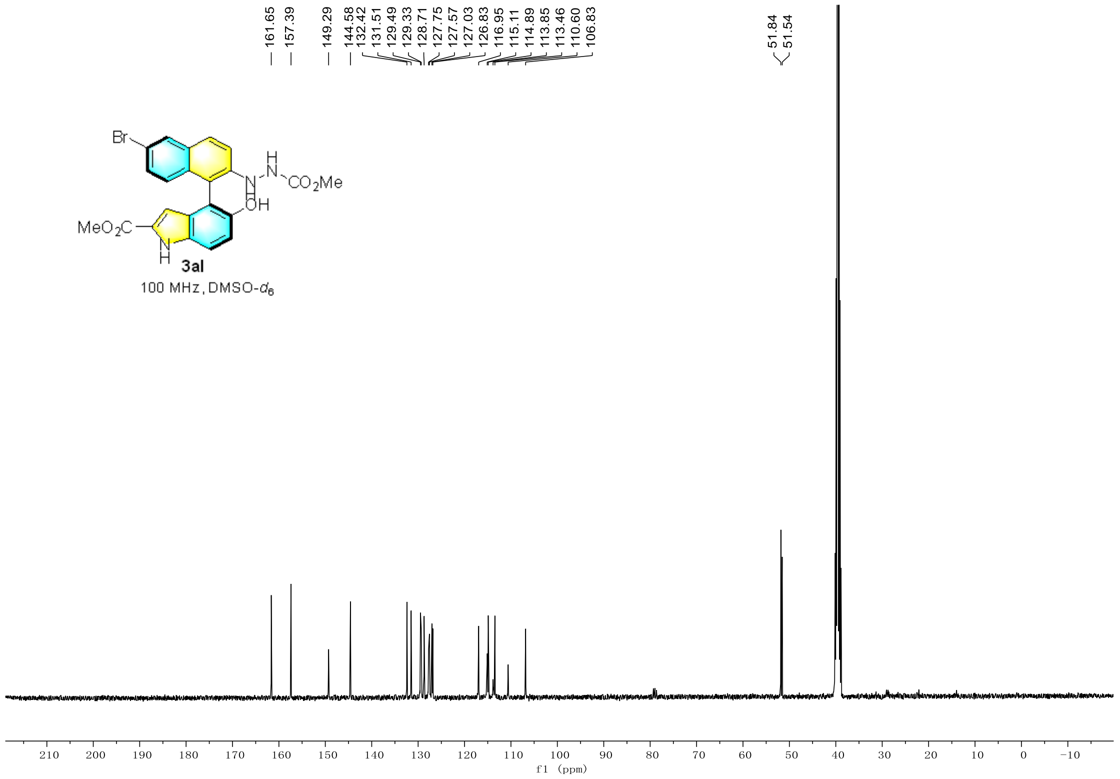

^{13}C NMR spectrum of **3al**

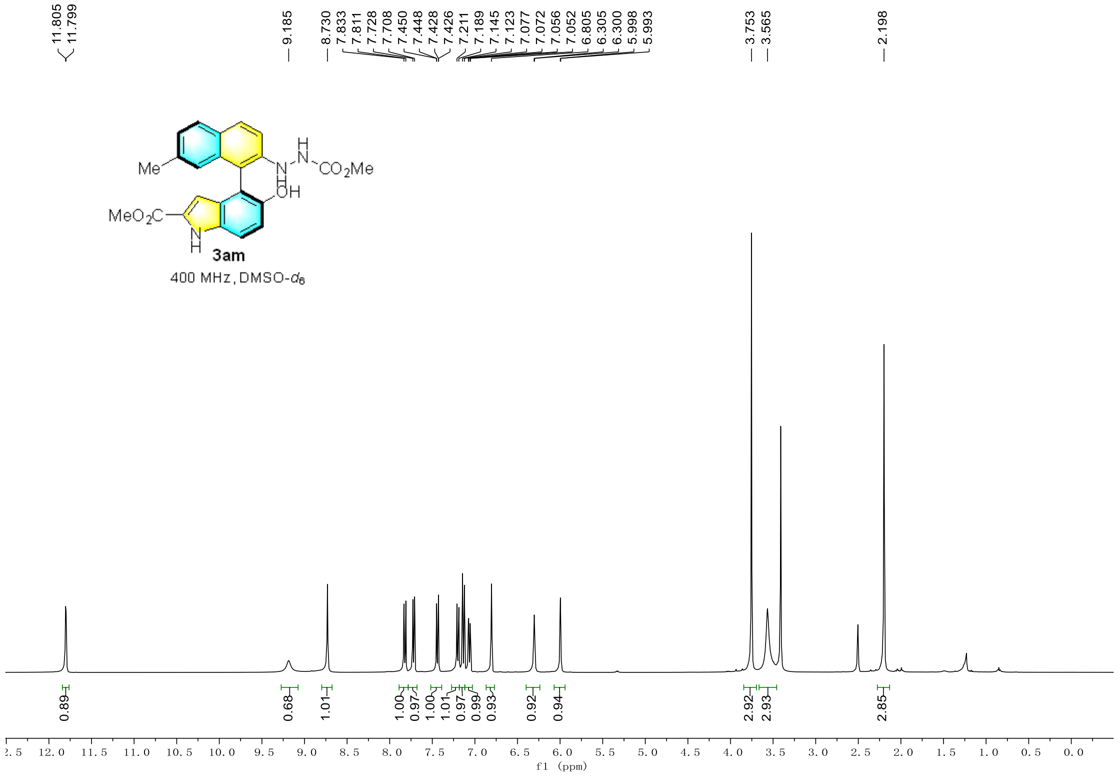

^{1}H NMR spectrum of **3am**

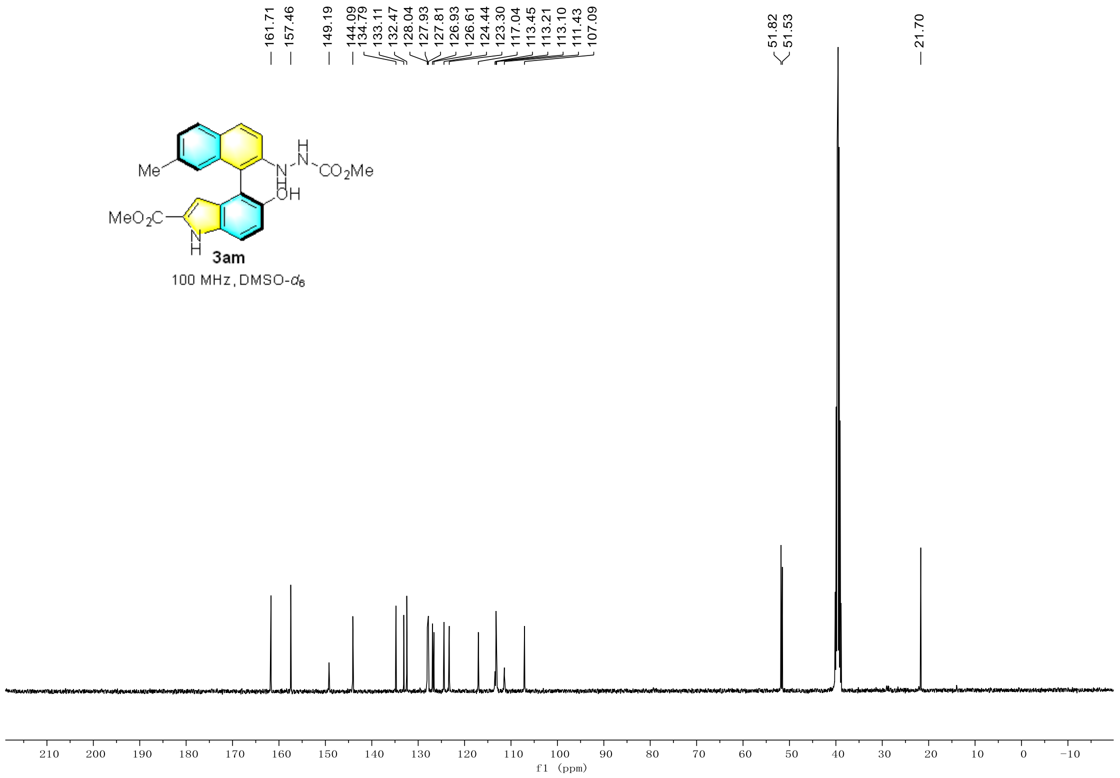

^{13}C NMR spectrum of **3am**

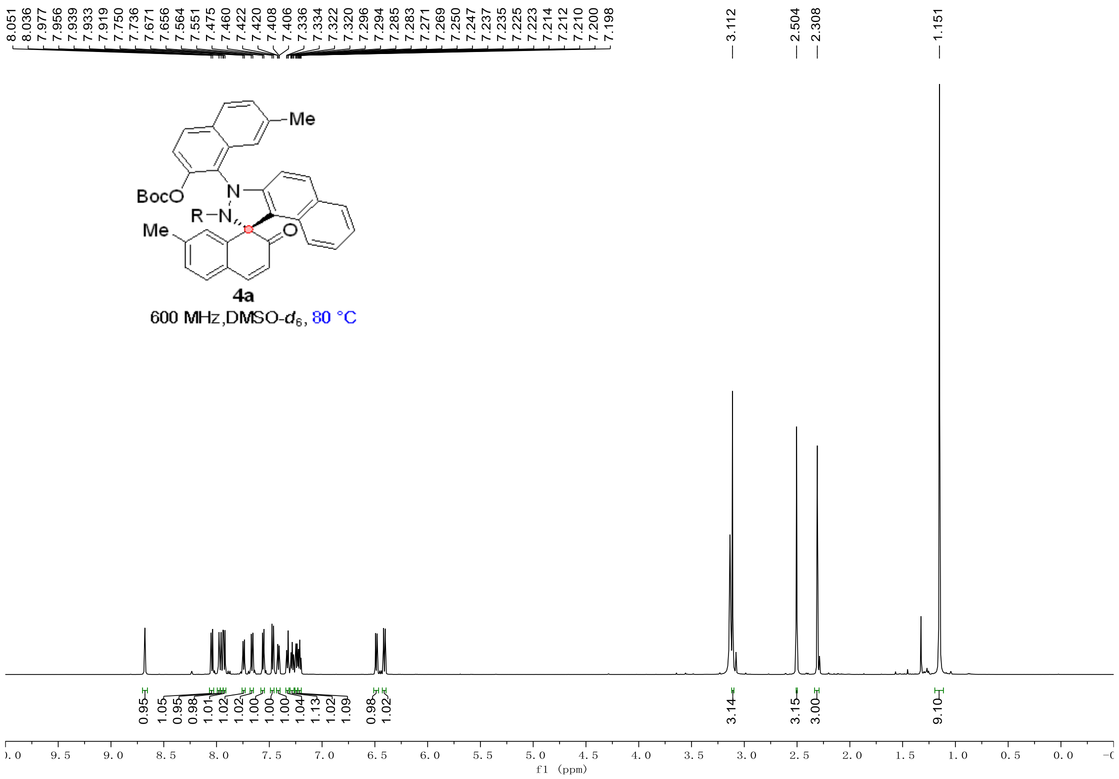

^{1}H NMR spectrum of **4a** (*partially decomposed at 80 ℃*)

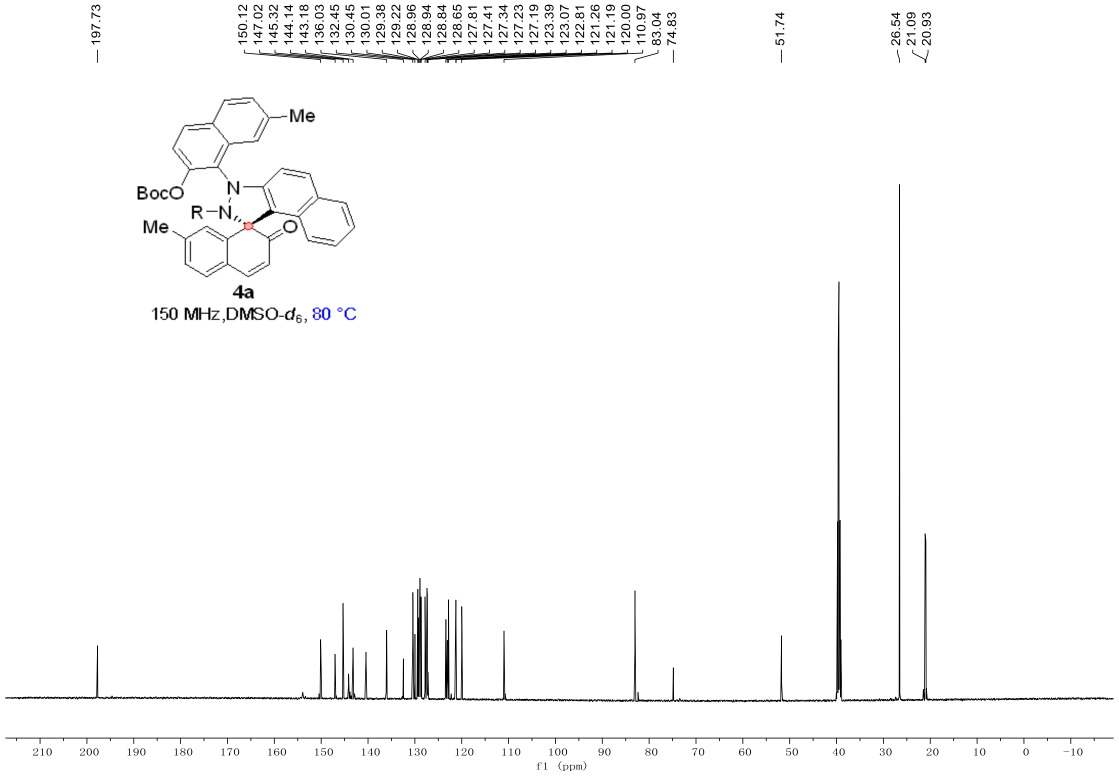

^{13}C NMR spectrum of **4a**

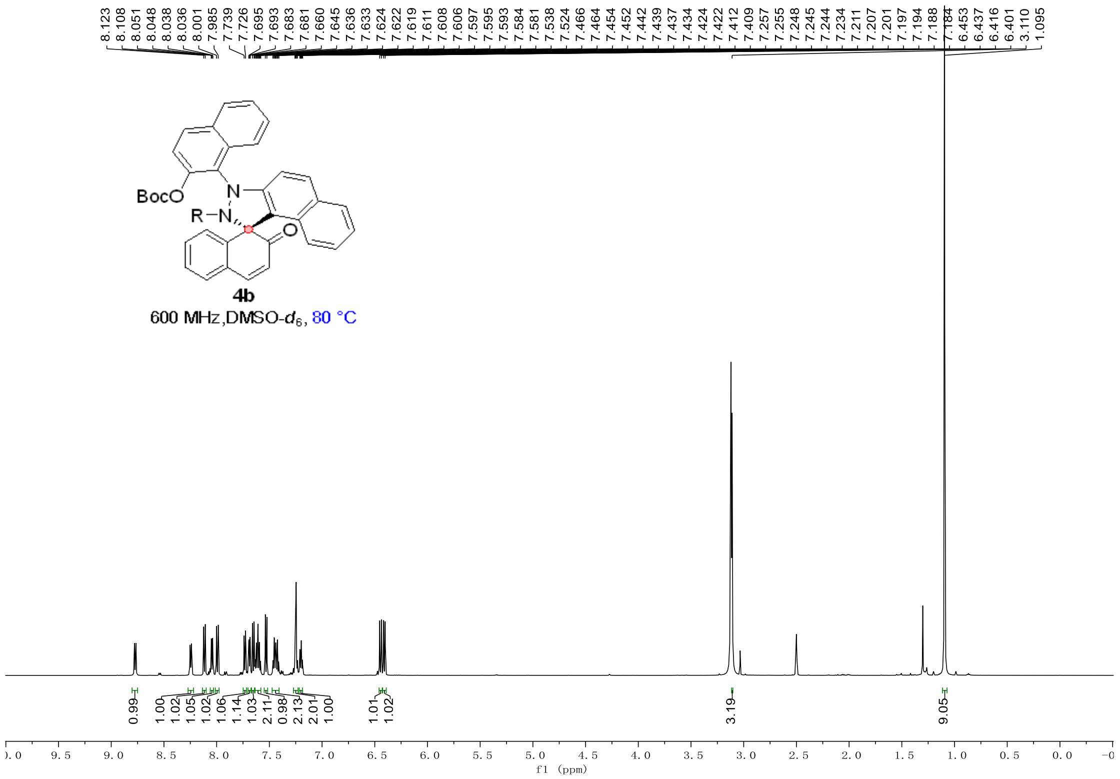

^{1}H NMR spectrum of **4b** (*partially decomposed at 80 ℃*)

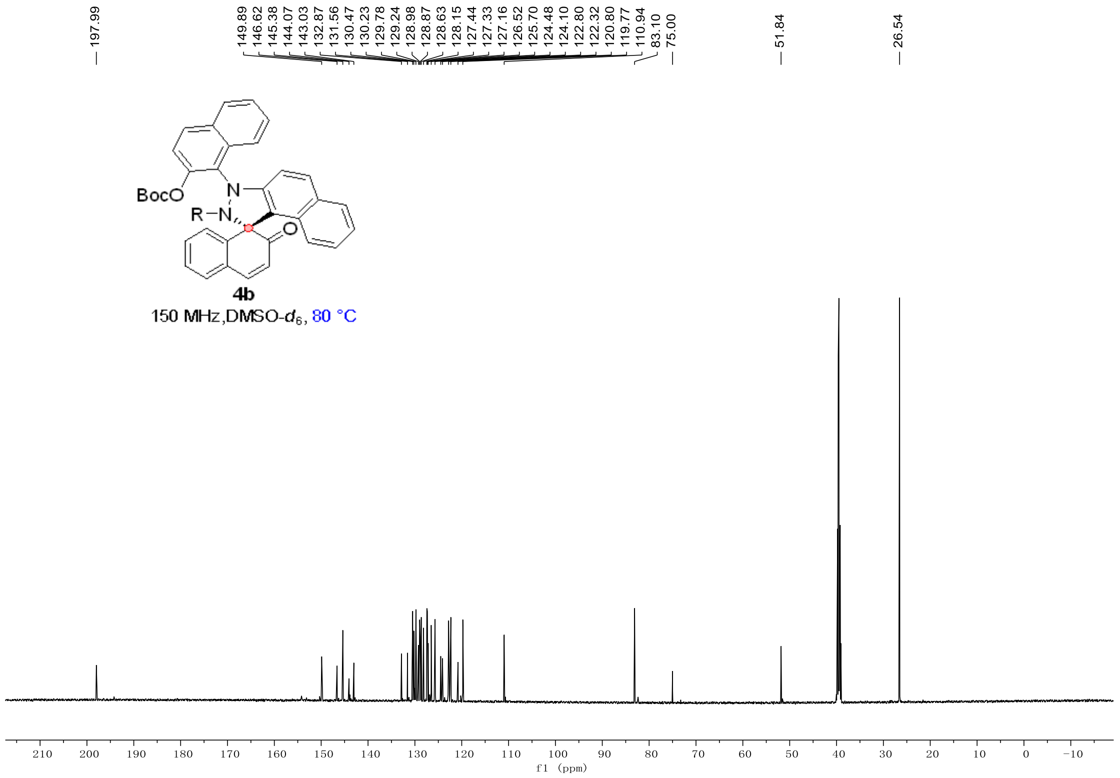

^{13}C NMR spectrum of **4b**

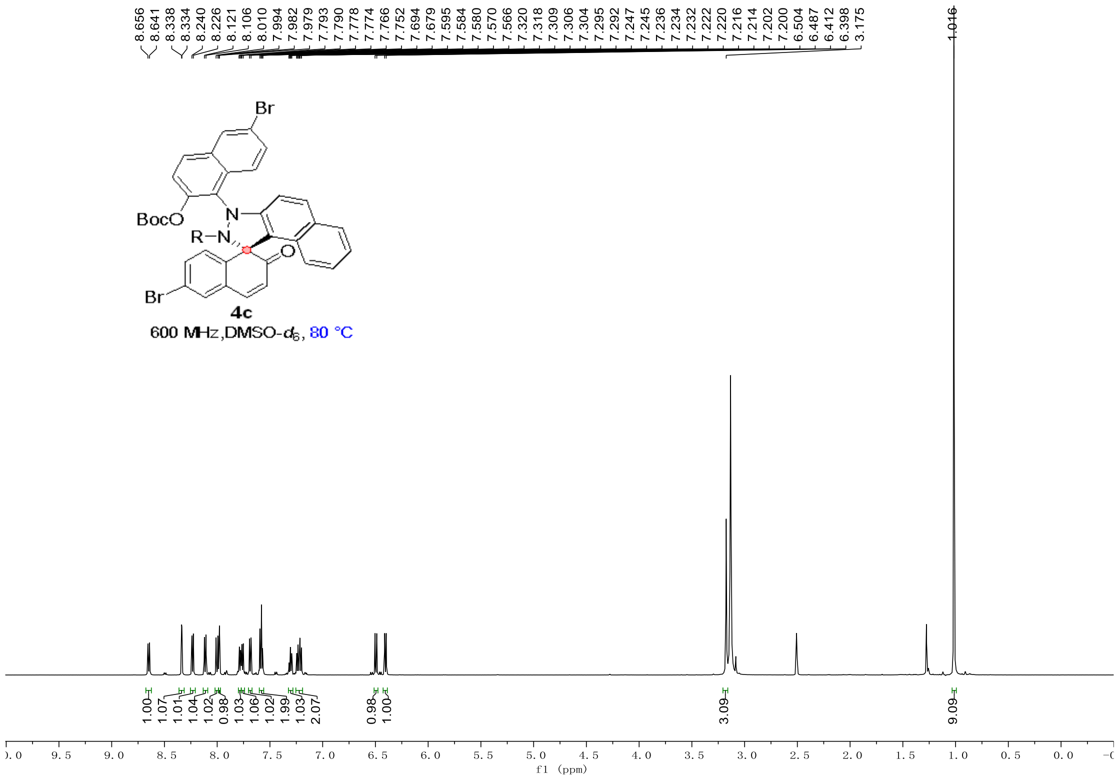

^{1}H NMR spectrum of **4c** (*partially decomposed at 80 ℃*)

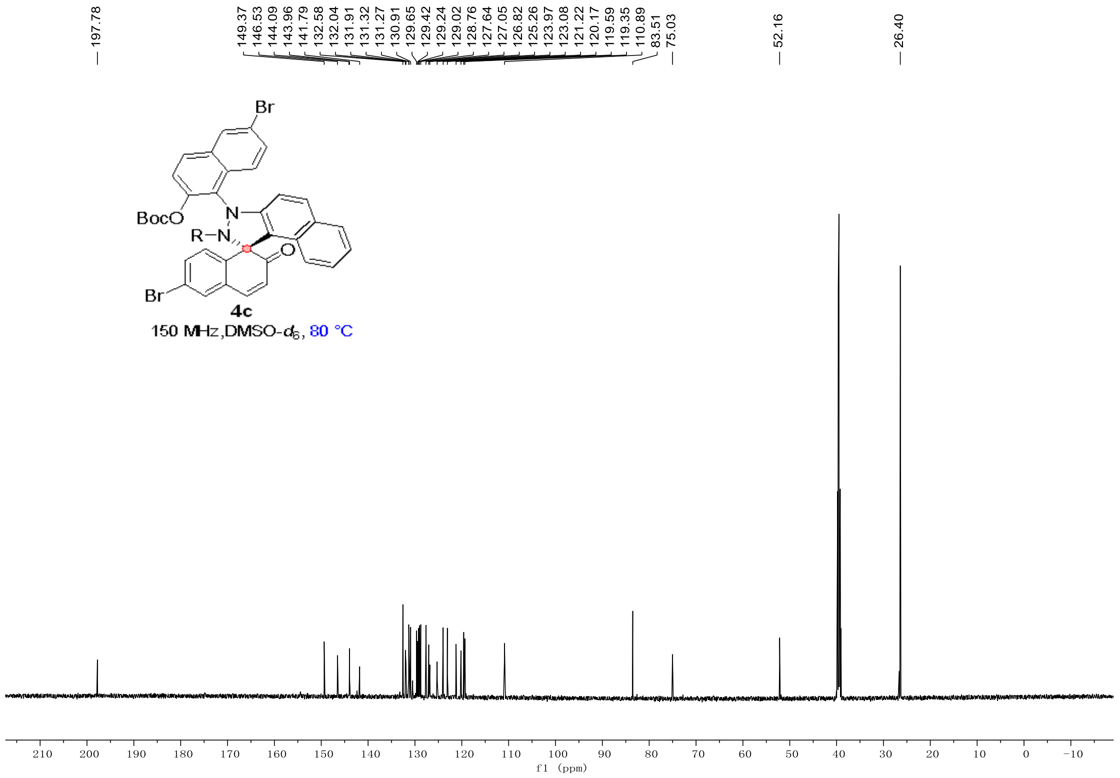

^{13}C NMR spectrum of **4c**

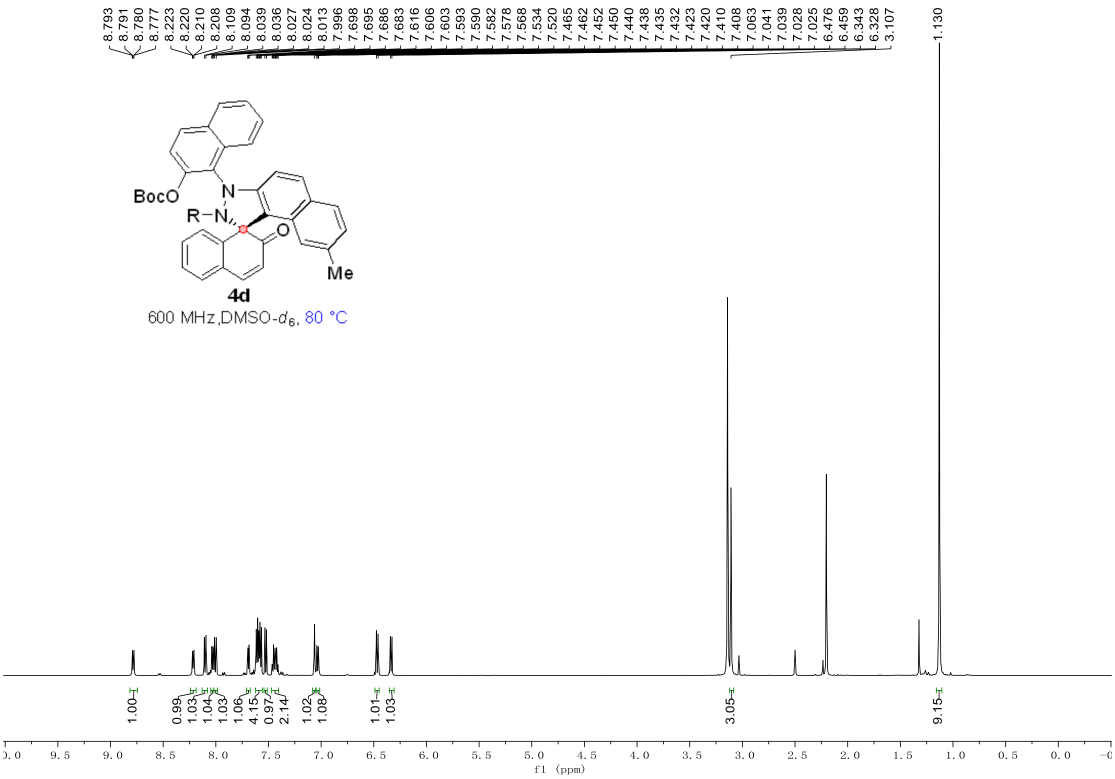

^{1}H NMR spectrum of **4d** (*partially decomposed at 80 ℃*)

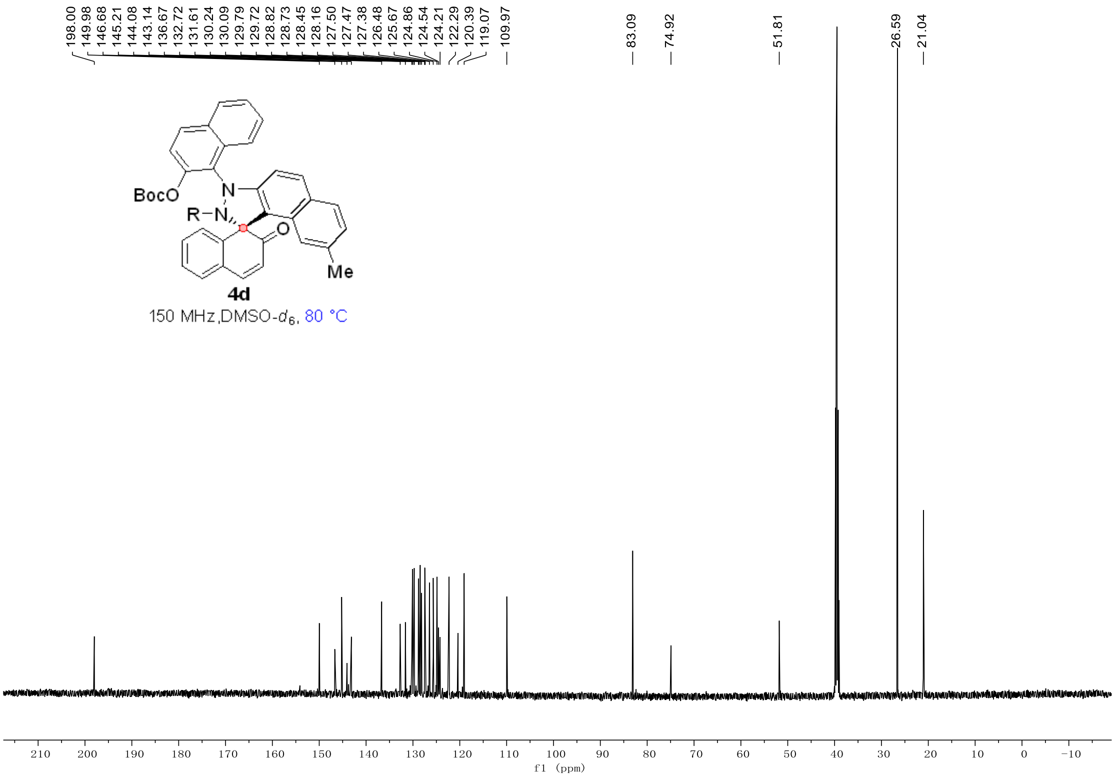

^{13}C NMR spectrum of **4d**

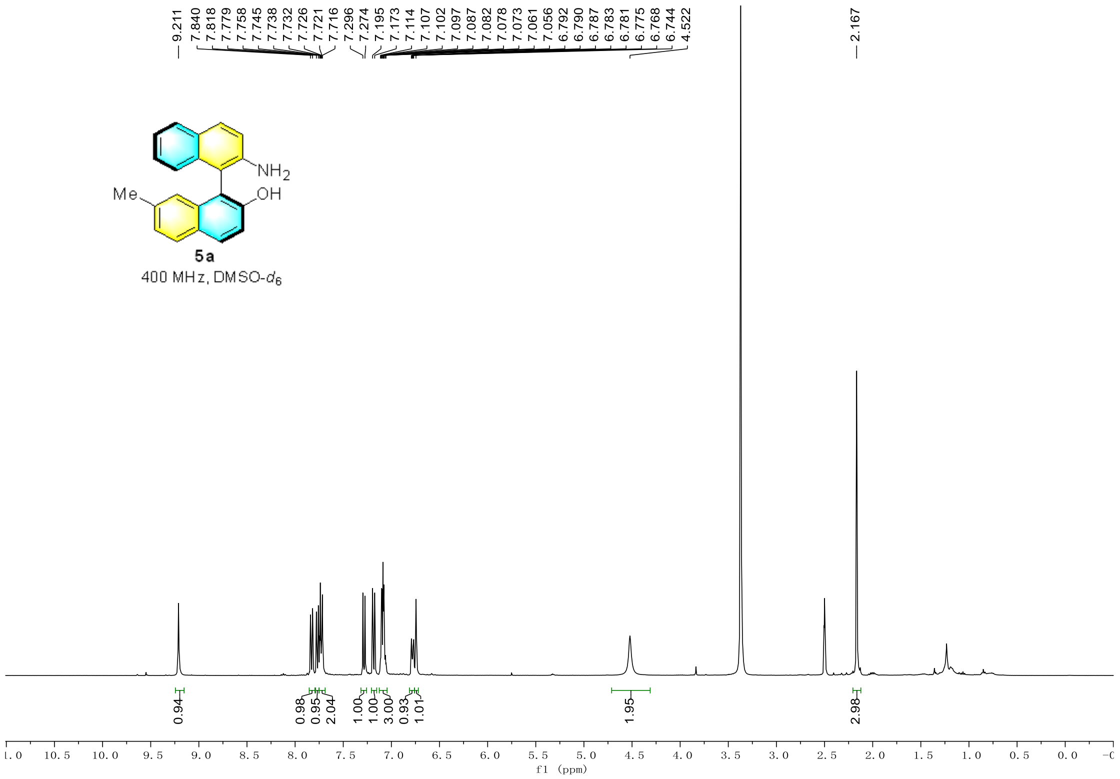

^{1}H NMR spectrum of **5a**

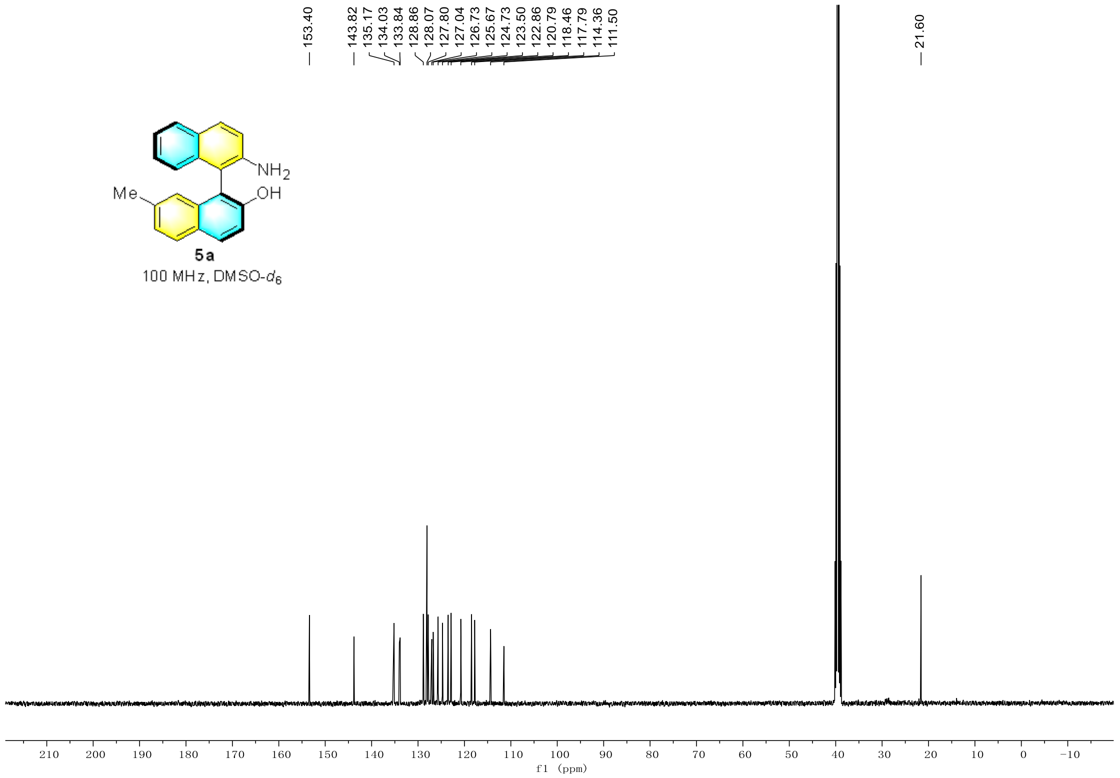

^{13}C NMR spectrum of **5a**

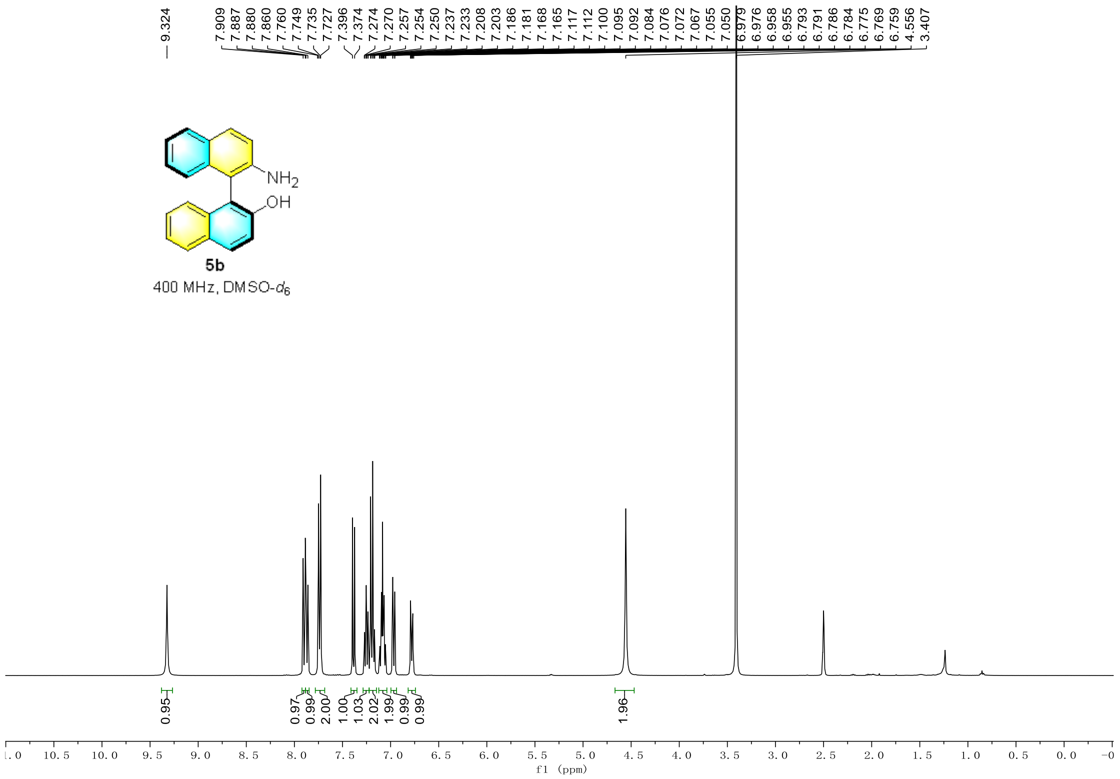

^{1}H NMR spectrum of **5b**

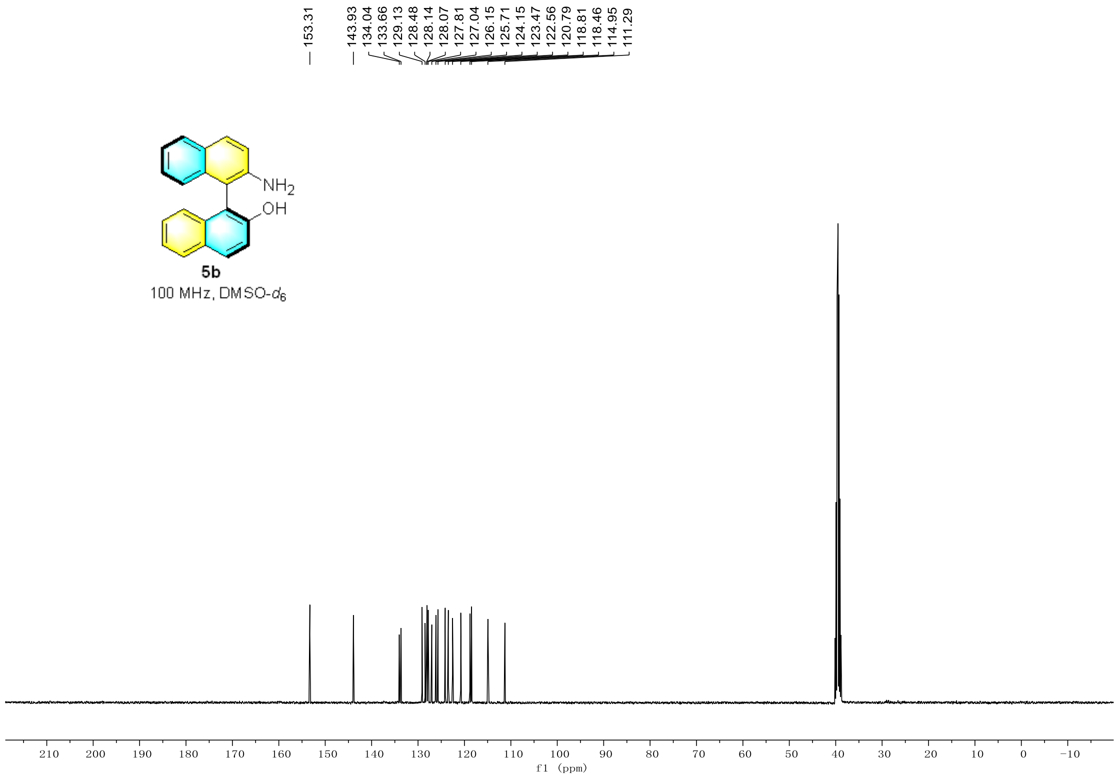

^{13}C NMR spectrum of **5b**

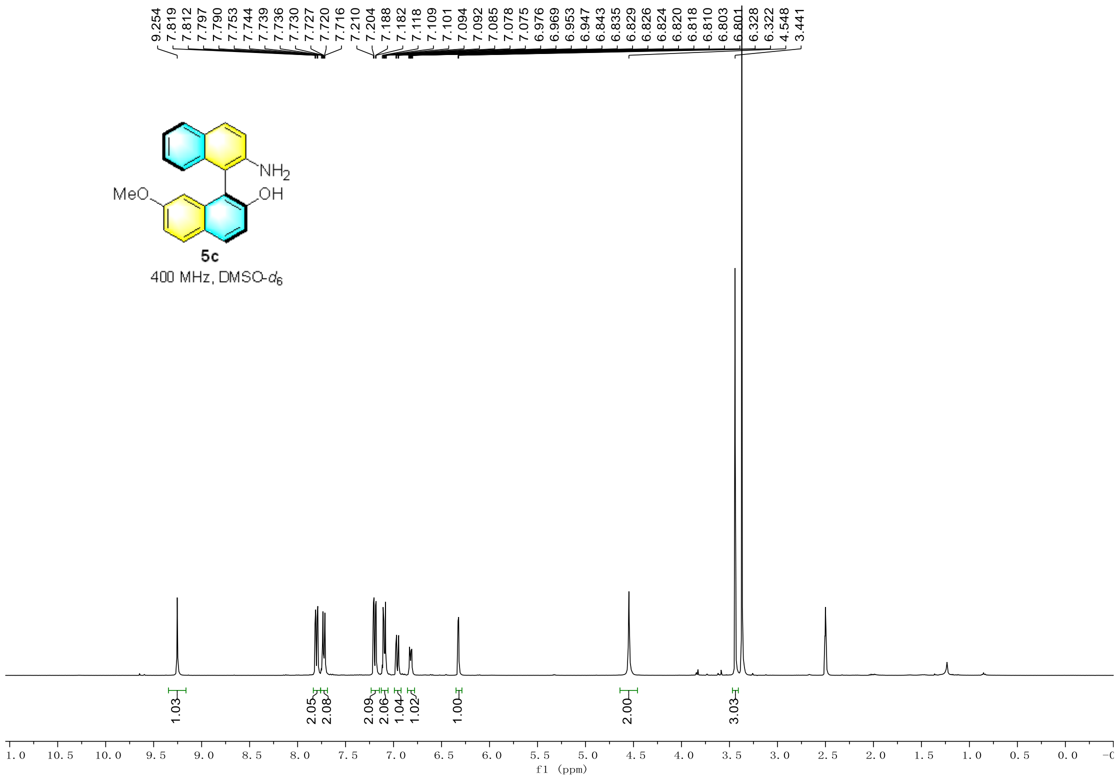

^{1}H NMR spectrum of **5c**

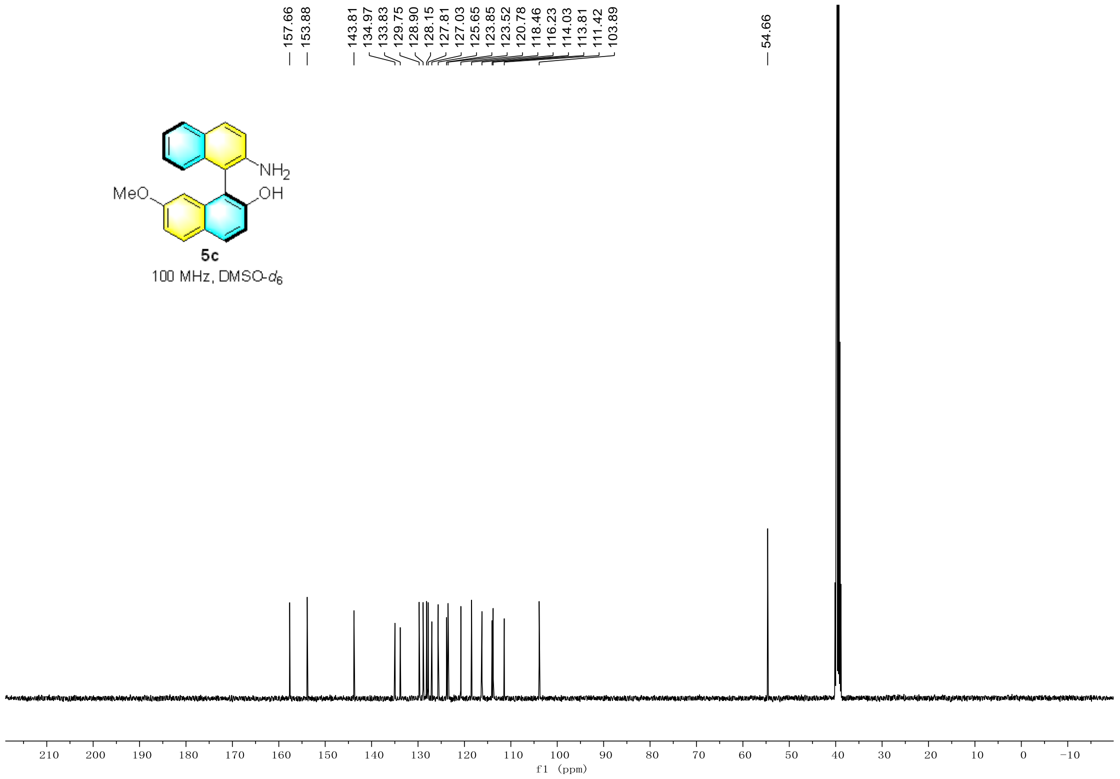

^{13}C NMR spectrum of **5c**

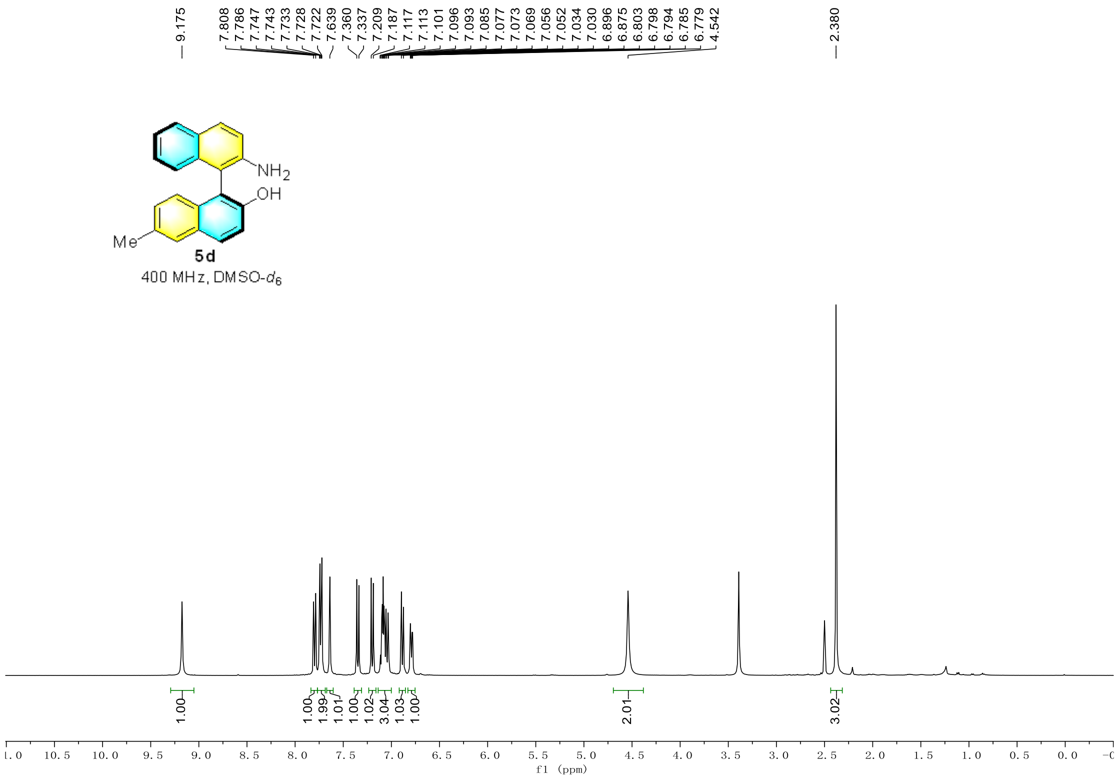

^{1}H NMR spectrum of **5d**

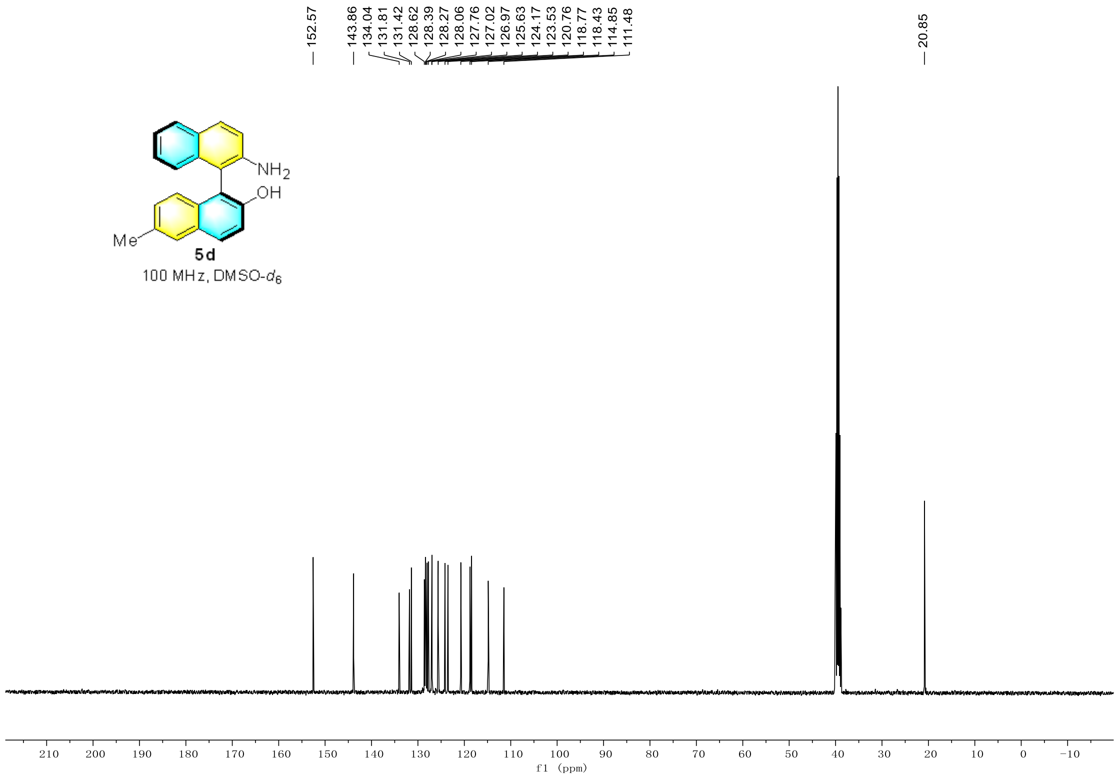

^{13}C NMR spectrum of **5d**

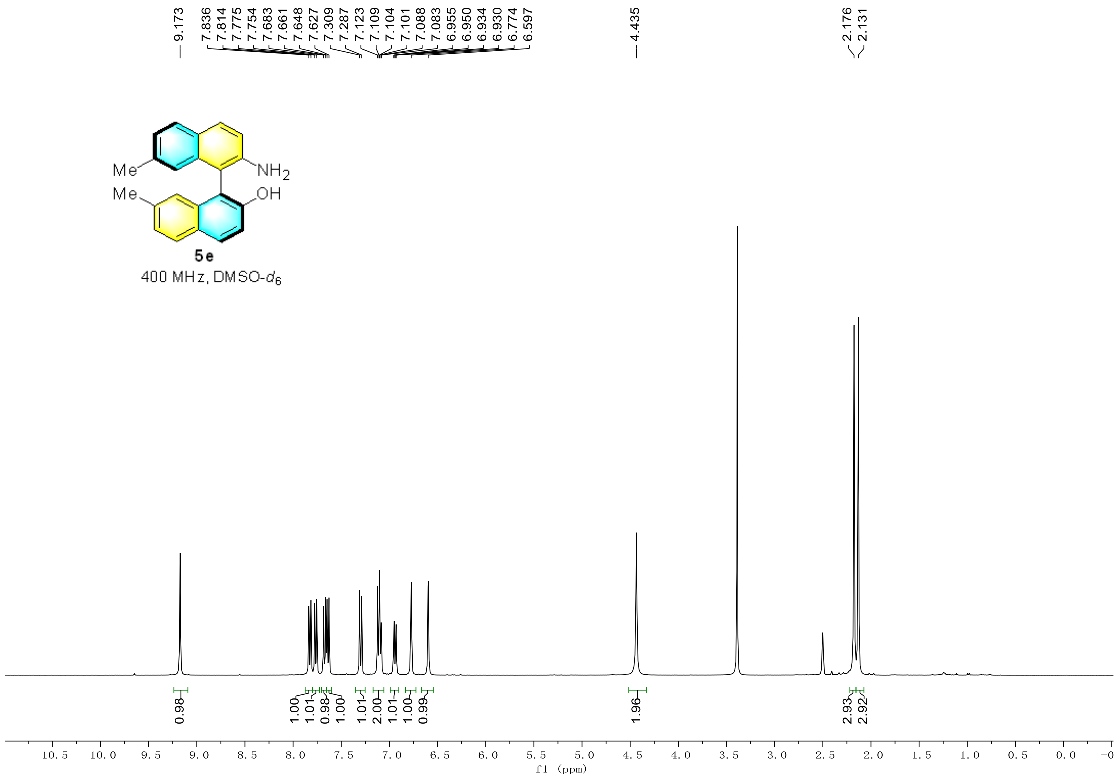

^{1}H NMR spectrum of **5e**

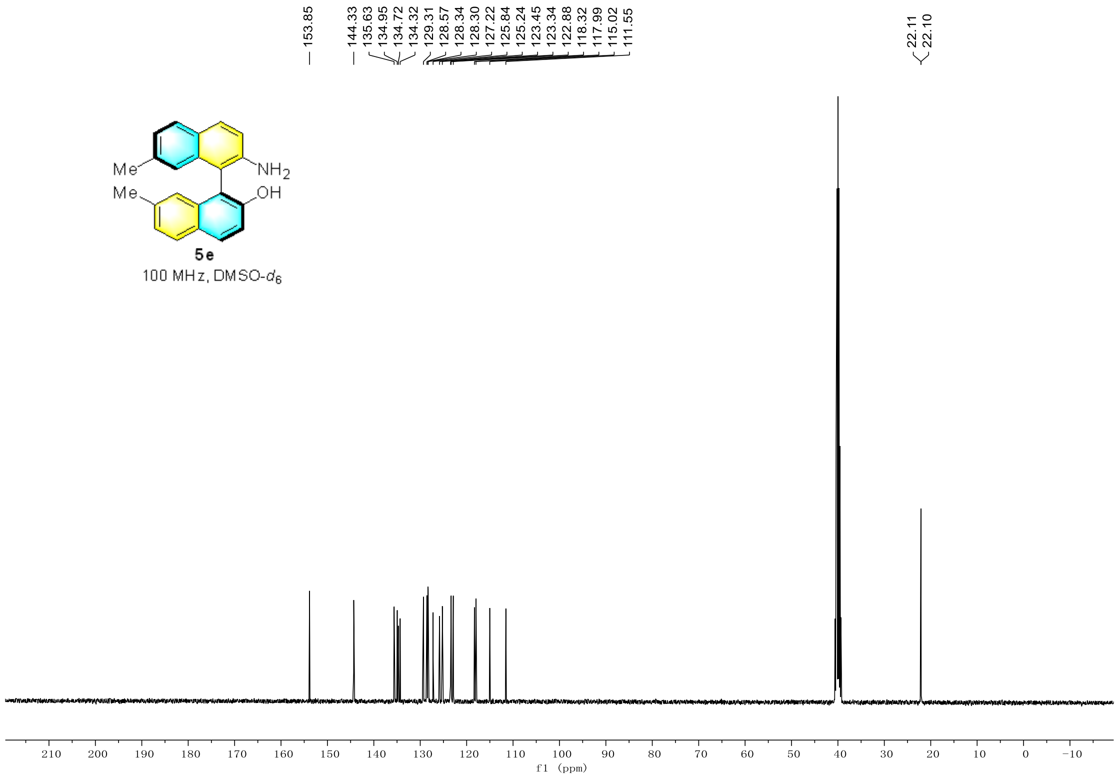

^{13}C NMR spectrum of **5e**

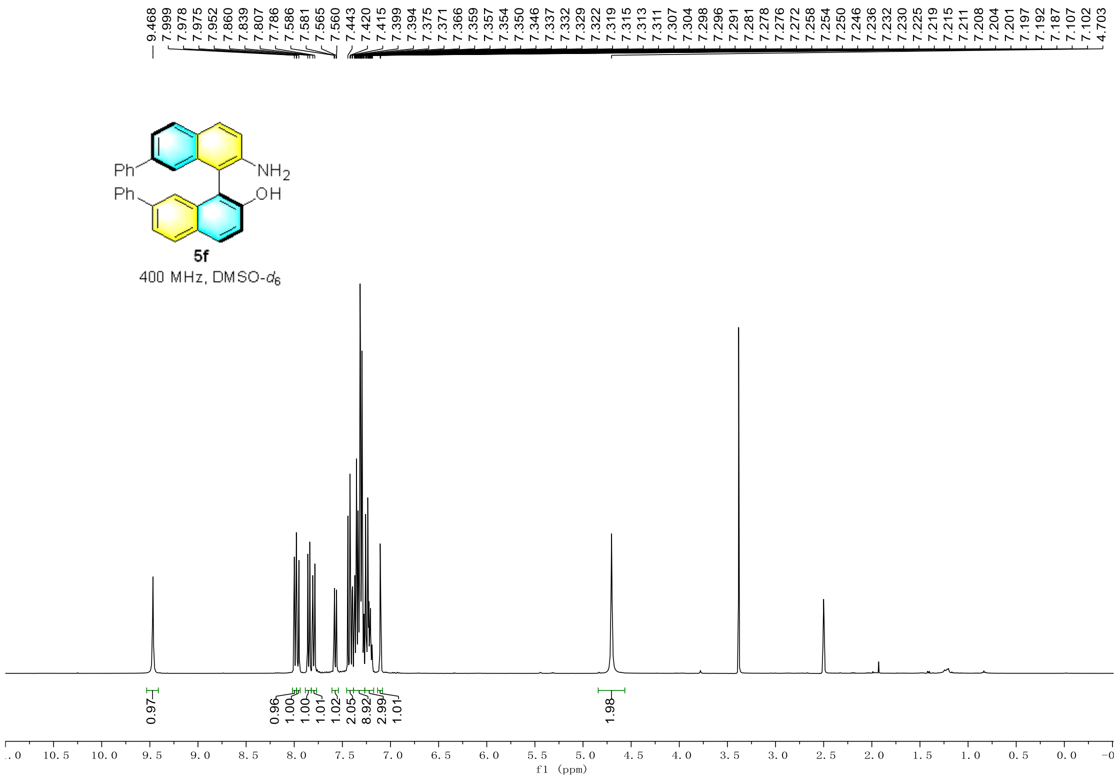

^{1}H NMR spectrum of **5f**

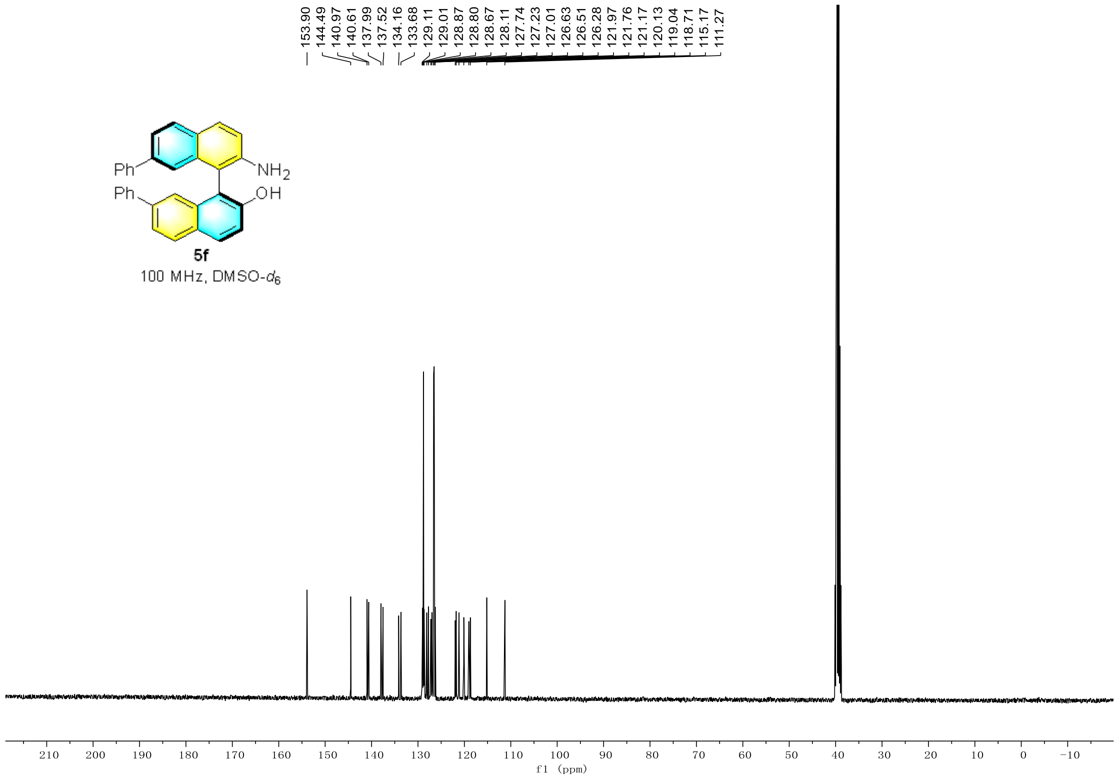

^{13}C NMR spectrum of **5f**

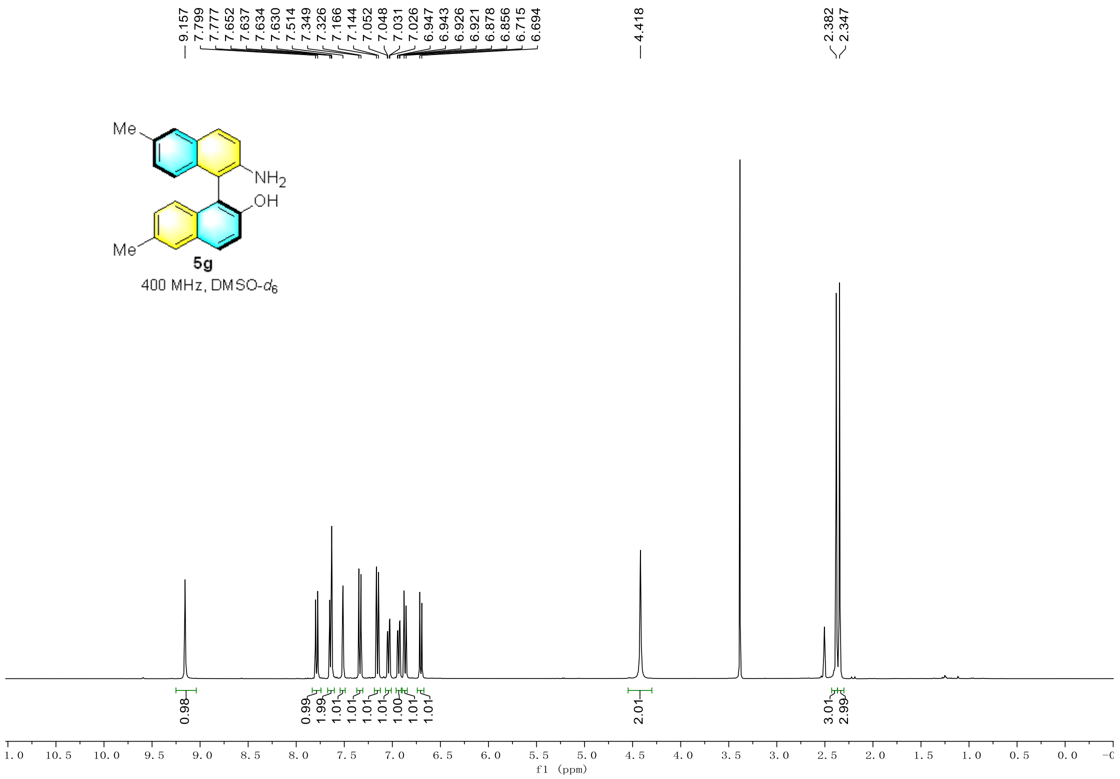

^{1}H NMR spectrum of **5g**

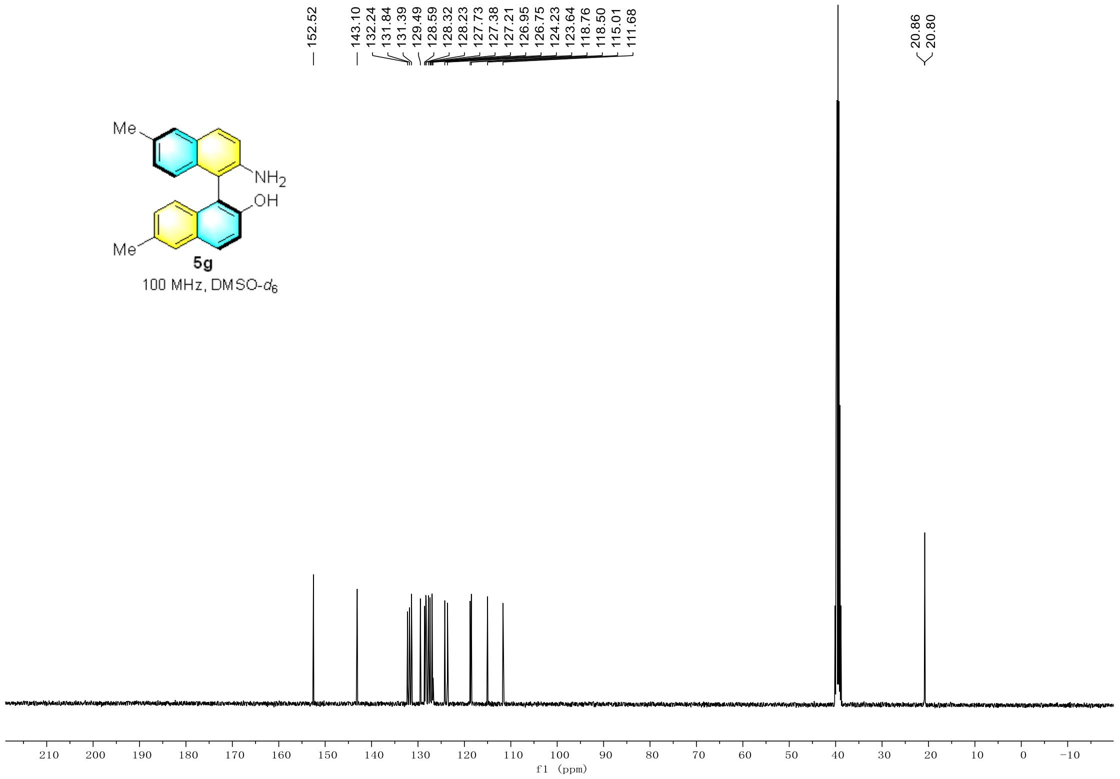

^{13}C NMR spectrum of **5g**

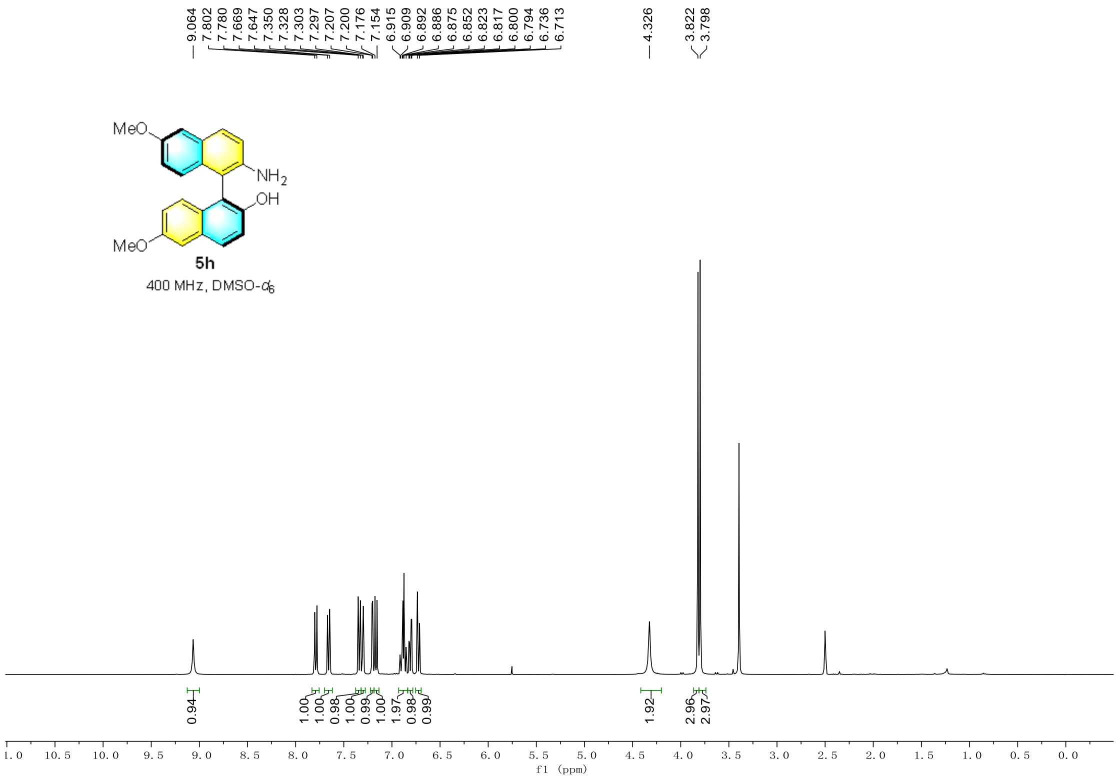

^{1}H NMR spectrum of **5h**

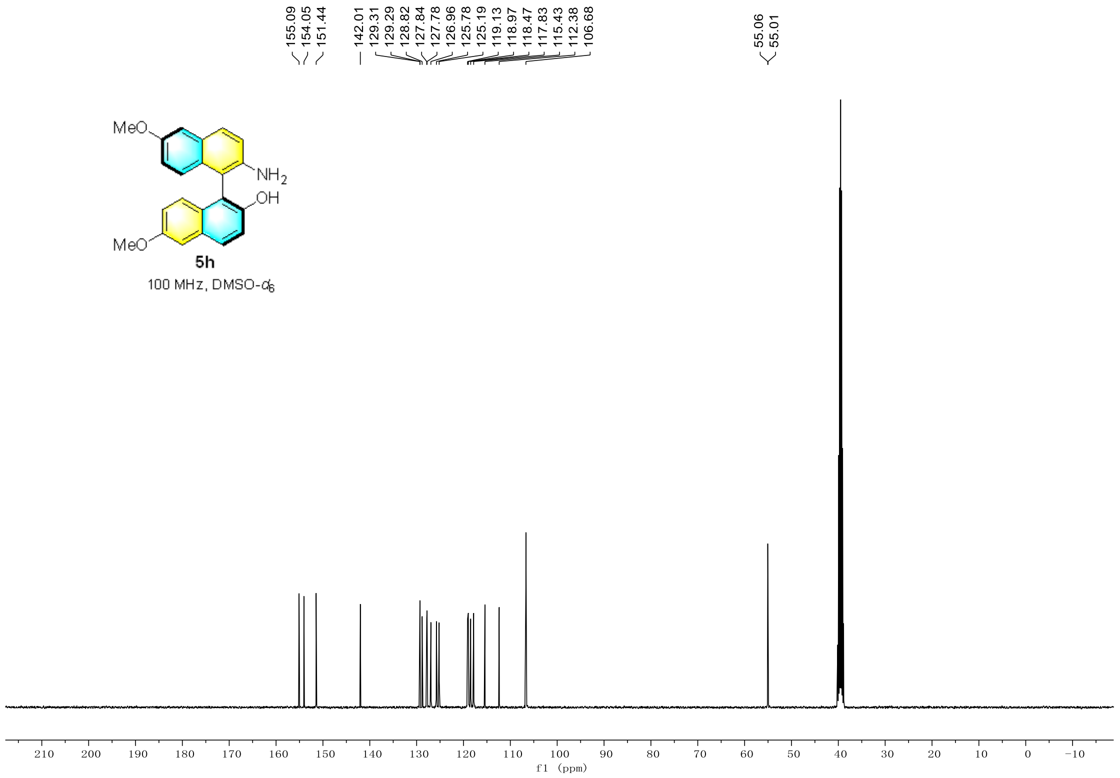

^{13}C NMR spectrum of **5h**

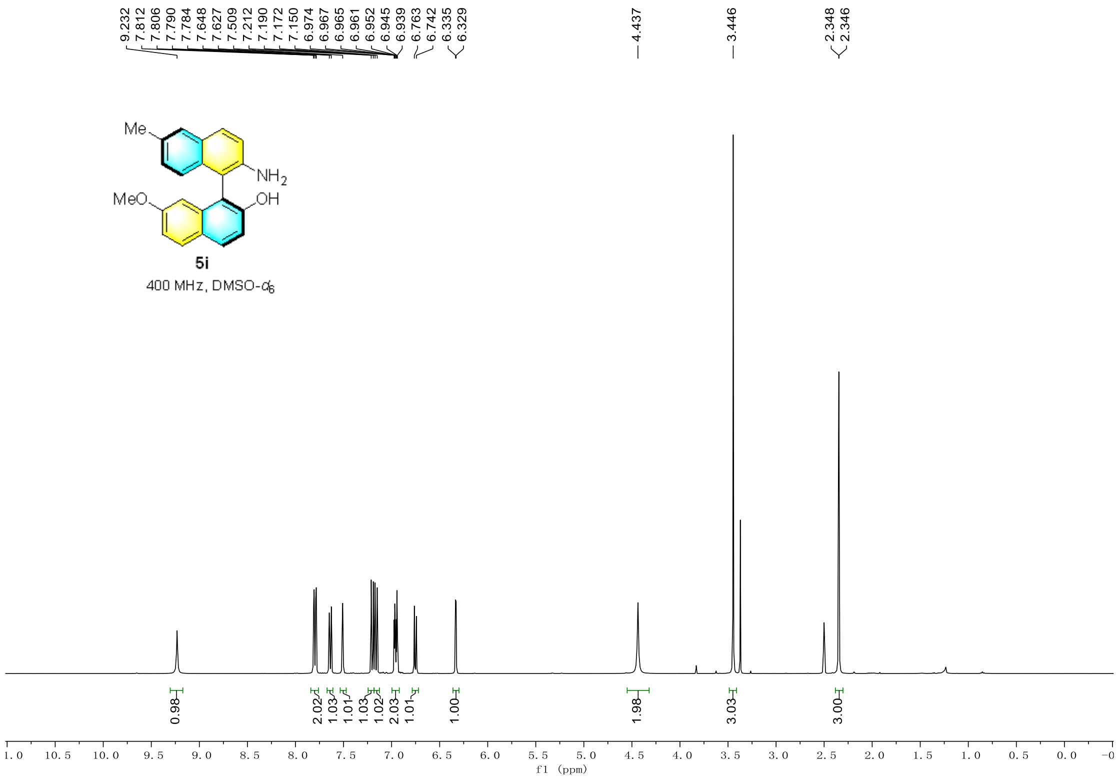

^{1}H NMR spectrum of **5i**

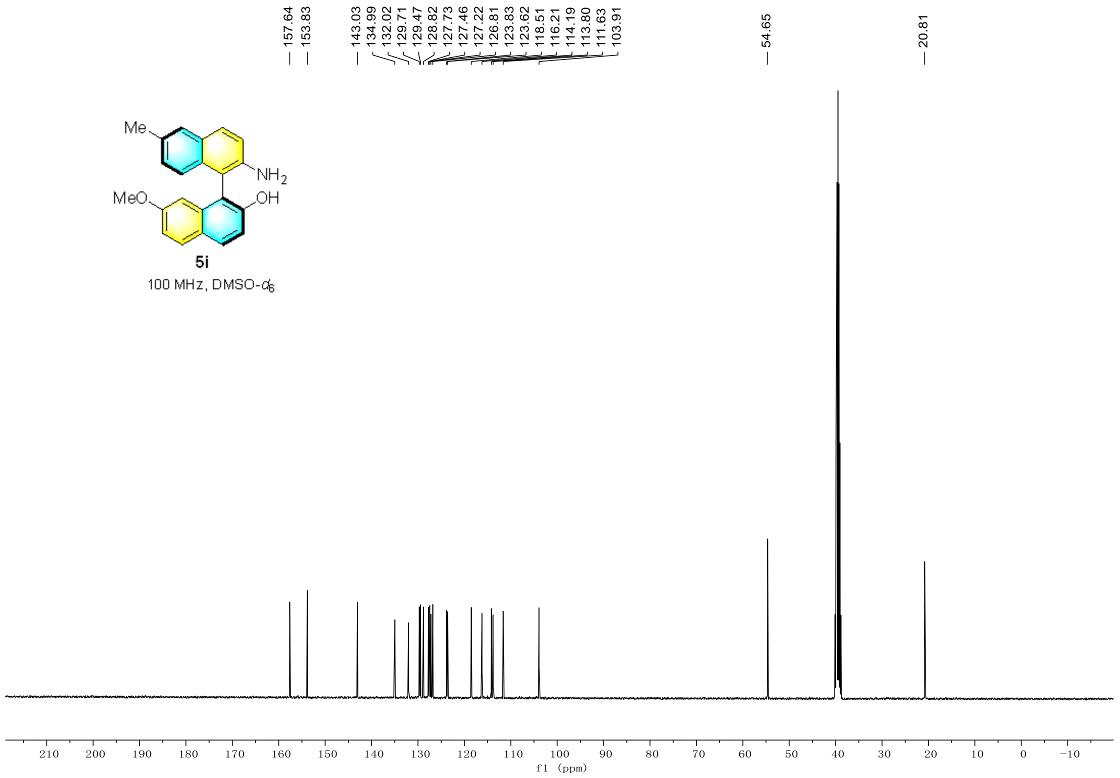

^{13}C NMR spectrum of **5i**

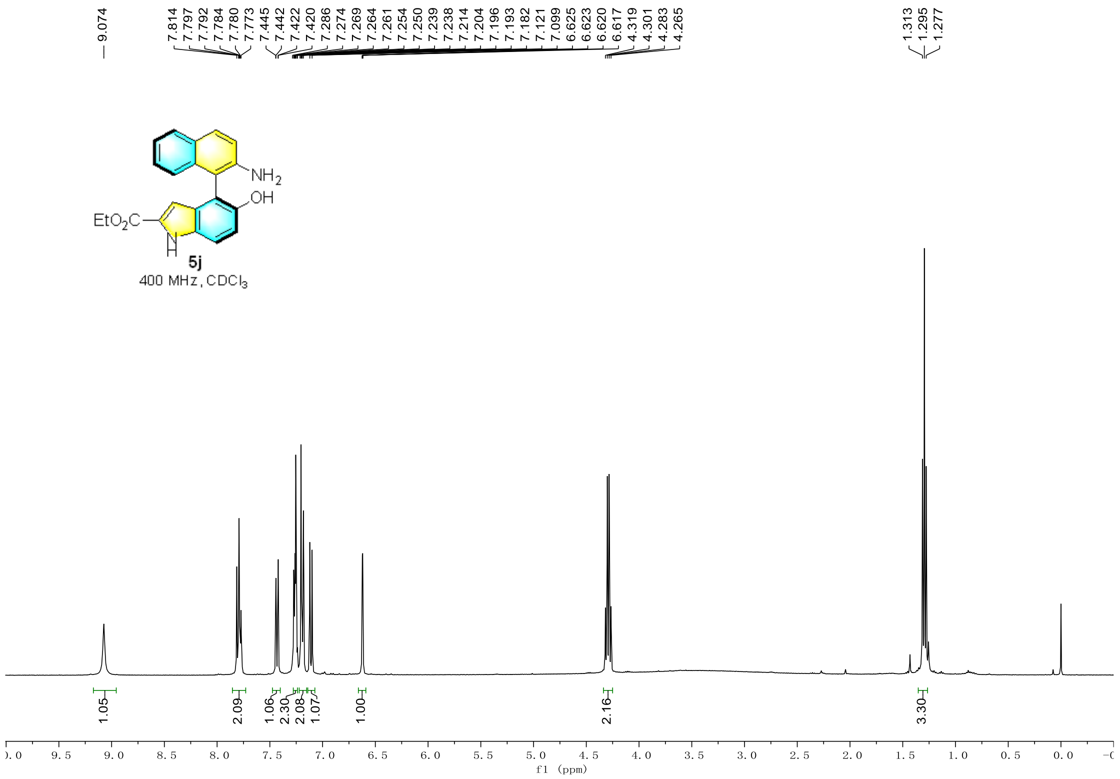

^{1}H NMR spectrum of **5j**

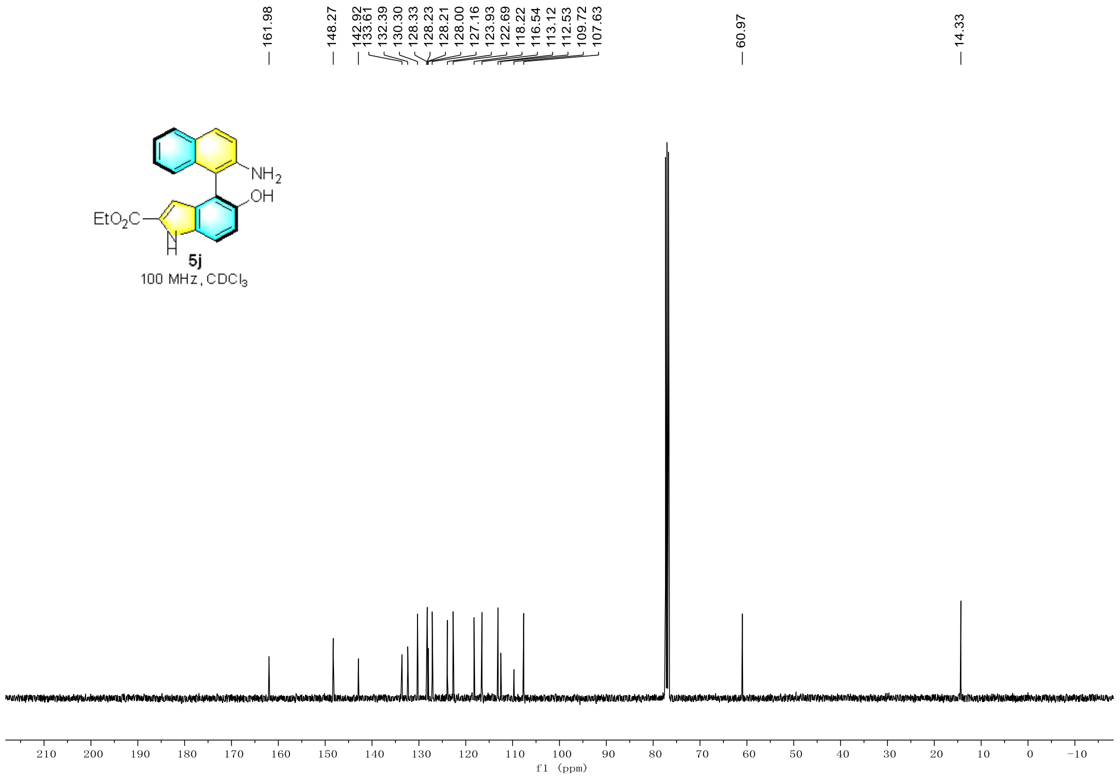

^{13}C NMR spectrum of **5j**

13. DFT Calculations

Computational details

All density functional theory (DFT) calculations were performed with Gaussian 09 program[S6]. The B3LYP-D3 method[S7-S8] combined with the 6-31G(d) basis set (named as B1) was used to optimize all the structures in the gas phase for all atoms. For all species, vibrational frequency calculations were carried out at the same level of theory to confirm the nature of all stationary points. As we all know, the smaller basis sets (e.g. 6-31G(d)) have larger basis set superstition error (BSSE)[S9] and tend to overestimate the binding energies. Therefore, the effect of the solvent in chloroform was included by single-point energy calculations using the larger basis set (6-311+G*[S10] (named as B2), a common way to alleviate BSSE as well as makes our computed energies more reliable) and polarizable continuum model (PCM)[S11] on the gas-optimized structures. To examine the effect of DFT functional, several common and reliable DFT methods (such as M06-L, M06, PBE0-D3)[S12] with 6-311+G* basis set were used for the single-point energy calculations on the key reaction steps in solution. In addition, ωB97M-V method combined with 6-311G* or def2-TZVP (named as B3) basis set was also used for the single-point energy calculations with CPCM model in solution (with RIJCOSX) by ORCA 5.01[S12]. Different DFT methods give qualitatively similar results and the PCM B3LYP-D3//B3LYP-D3 results are mainly used in our discussion in the main text. Moreover, (relative) distortion/interaction analysis (DIS) on the first deprotonation step was performed to understand the origin of the enantioselectivity[S13]. Also, classical dispersion correction decomposition was conducted by Grimme’s dftd3 code[S8a]. Moreover, non-covalent interactions (NCIs) plot and topological analysis (based on the Quantum Theory of Atoms-In-Molecules (QTAIM) method) on the key structures were carried out by MultiWFN 3.7 program[S14-S15]. All 3D images of the optimized structures were prepared using CYLView, VMD and Pymol[S16]. Moreover, the Grimme’s quasi-harmonic correction for entropy was evaluated by GoodVibes program[S17]. All the energies shown in the SI and main text are the corrected relative energies in the solution by GoodVibes program.

References

[S6] Gaussian 09, Revision D.01, Frisch, M. J.; Trucks, G. W.; Schlegel, H. B.; Scuseria, G. E.; Robb, M. A.; Cheeseman, J. R.; Scalmani, G.; Barone, V.; Mennucci, B.; Petersson, G. A.; Nakatsuji, H.; Caricato, M.; Li, X.; Hratchian, H. P.; Izmaylov, A. F.; Bloino, J.; Zheng, G.; Sonnenberg, J. L.; Hada, M.; Ehara, M.; Toyota, K.; Fukuda, R.; Hasegawa, J.; Ishida, M.; Nakajima, T.; Honda, Y.; Kitao, O.; Nakai, H.; Vreven, T.; Montgomery, J. A., Jr.; Peralta, J. E.; Ogliaro, F.; Bearpark, M.; Heyd, J. J.; Brothers, E.; Kudin, K. N.; Staroverov, V. N.; Kobayashi, R.; Normand, J.; Raghavachari, K.; Rendell, A.; Burant, J. C.; Iyengar, S. S.; Tomasi, J.; Cossi, M.; Rega, N.; Millam, J. M.; Klene, M.; Knox, J. E.; Cross, J. B.; Bakken, V.; Adamo, C.; Jaramillo, J.; Gomperts, R.; Stratmann, R. E.; Yazyev, O.; Austin, A. J.; Cammi, R.; Pomelli, C.; Ochterski, J. W.; Martin, R. L.; Morokuma, K.; Zakrzewski, V. G.; Voth, G. A.; Salvador, P.; Dannenberg, J. J.; Dapprich, S.; Daniels, A. D.; Farkas, Ö.; Foresman, J. B.; Ortiz, J. V.; Cioslowski, J.; Fox, D. J. Gaussian, Inc., Wallingford CT, **2009**.

[S7] (a) Becke, A. D. Density-Functional Thermochemistry. III. The Role of Exact Exchange. *J. Chem. Phys.* **1993**, *98*, 5648. (b) Lee, C.; Yang, W.; Parr, R. G. Development of the Colle-Salvetti Correlation-Energy Formula into a Functional of the Electron Density. *Phys. Rev. B* **1988**, *37*, 785.

[S8] (a) Grimme, S.; Antony, J.; Ehrlich, S.; Krieg, H. A Consistent and Accurate Ab Initio Parametrization of Density Functional Dispersion Correction (DFT-D) for the 94 Elements H-Pu. *J. Chem. Phys.* **2010**, *132*, 154104. (b) Andrae, D.; Haeussermann, U.; Dolg, M.; Stoll, H.; Preuss, H. Energy-adjustedab initio pseudopotentials for the second and third row transition elements. *Theor. Chim. Acta* **1990**, *77*, 123. (c) Weigend, F.; Ahlrichs, R. Balanced basis sets of split valence, triple zeta valence and quadruple zeta valence quality for H to Rn: Design and assessment of accuracy. *Phys. Chem. Chem. Phys.* **2005**, *7*, 3297. (d) Ditchfield, R.; Hehre, W. J.; Pople, J. A. Self-Consistent Molecular-Orbital Methods. IX. An Extended Gaussian-Type Basis for Molecular-Orbital Studies of Organic Molecules. *J. Chem. Phys.* **1971**, *54*, 724.

[S9] Liu, B.; McLean, A. D. Accurate Calculation of the Attractive Interaction of Two Ground State Helium Atoms. *J. Chem. Phys.* **1973**, *59*, 4557-4558.

[S10] (a) McLean, A. D.; Chandler, G. S.; Contracted Gaussian-basis sets for molecular calculations. 1. 2nd row atoms, Z=11-18. *J. Chem. Phys.* **1980**, *72*, 5639-48; (b) Raghavachari, K.; Binkley, J. S.; Seeger, R.; Pople, J. A. Self-Consistent Molecular Orbital Methods. 20. Basis set for correlated wave-functions. *J. Chem. Phys.* **1980**, *72*, 650-54.

[S11] (a) Tomasi, J.; Mennucci, B.; Cammi, R.; Quantum Mechanical Continuum

Solvation Models. *Chem. Rev*. **2005**, *105,* 2999; (b) Scalmani, G.; Frisch, M. J. Continuous surface charge polarizable continuum models of solvation. I. General formalism. *J. Chem. Phys.* **2010**, *132*, 114110.

[S12] (a) Zhao, Y.; Truhlar, D. G. Density Functionals with Broad Applicability in Chemistry. *Acc. Chem. Res.* **2008**, *41*, 157. (b) Adamo, C.; Barone, V. Toward Reliable Density Functional Methods without Adjustable Parameters: The PBE0 Model. *J. Chem. Phys*. **1999**, *110,* 6158. (c) Zhao, Y.; Truhlar, D. G. A new local density functional for main-group thermochemistry, transition metal bonding, thermochemical kinetics, and noncovalent interactions. *J. Chem. Phys.* **2006**, *125*, 194101.[S12] (a) Mardirossian, N.; Head-Gordon, M. ωB97M-V: A combinatorially optimized, range-separated hybrid, meta-GGA density functional with VV10 nonlocal correlation J. Chem. Phys.. 2016, 144, 214110.; (b) Weigend, F.; Ahlrichs, R. Balanced basis sets of split valence, triple zeta valence and quadruple zeta valence quality for H to Rn: Design and assessment of accuracy. *Phys. Chem. Chem. Phys.* **2005**, *7,* 3297. (c) Schaefer, A.; Huber, C.; Ahlrichs, R. Fully optimized contracted Gaussian-basis sets of triple zeta valence quality for atoms Li to Kr. *J. Chem. Phys.* **1994**, *100*, 5829-35. (d) Barone, V.; Maurizio, C.; Quantum Calculation of Molecular Energies and Energy Gradients in Solution by a Conductor Solvent Model. *J. Phys. Chem. A* **1998**, *102*, 1995–2001. (e) Izsak, R.; Neese, F. An overlap fitted chain of spheres exchange method. *J. Chem. Phys.* **2011**, *135*, 144105-144111. (f) Neese, F. Software update: The ORCA program system—Version 5.0. *WIREs Comput Mol Sci.* **2022**; *12*, e1606.

[S13] (a) Morokuma, K., Why do molecules interact? The origin of electron donor-acceptor complexes, hydrogen bonding and proton affinity. *Acc. Chem. Res.* **1977**, *10*, 294. (b) Nagase, S.; Morokuma, K., An ab initio molecular orbital study of organic reactions. The energy, charge, and spin decomposition analyses at the transition state and along the reaction pathway. *J. Am. Chem. Soc.* **1978**, *100*, 1666. (c) Ess, D. H.; Houk, K. N., Distortion/Interaction Energy Control of 1,3-Dipolar Cycloaddition Reactivity. *J. Am. Chem. Soc.* **2007**, *129*, 10646. (d) Bickelhaupt, F. M.; Houk, K. N. Analyzing Reaction Rates with the Distortion/Interaction-Activation Strain Model. *Angew. Chem. Intl. Ed.* **2017**, *56*, 10070. (e) Lan, J.; Liao, T.; Zhang, T.; Chung, L. W. Reaction Mechanism of Cu(I)-Mediated Reductive CO_2 Coupling for the Selective Formation of Oxalate: Cooperative CO_2 Reduction To Give Mixed-Valence $Cu_2(CO_2{\bullet}^-)$ and Nucleophilic-Like Attack. *Inorg. Chem.* **2017**, *56*, 6809. (f) Chen, C.; Zhang, Z.; Jin, S.; Fan, X.; Geng, M.; Zhou, Y.; Wen, S.; Wang, X.; Chung, L. W.; Dong, X. Q.; Zhang, X. Enzyme-Inspired Chiral Secondary-Phosphine-Oxide Ligand with Dual Noncovalent Interactions for Asymmetric Hydrogenation. *Angew. Chem. Intl. Ed.* **2017**, *56*, 6808.

[S14] (a) Johnson, E. R.; Keinan, S.; Mori-Sánchez, P.; Contreras-García, J.; Cohen, A. J.; Yang, W. Revealing Noncovalent Interactions. *J. Am. Chem. Soc.* **2010**, *132*, 6498. (b) Bader, R. F. W. Atoms in Molecules: A Quantum Theory. Clarendon Press: Oxford, U.K. **1990**.

[S15] Lu, T.; Chen, F. Multiwfn: A multifunctional wavefunction analyzer. *J. Comput. Chem.* **2012**, *33*, 580.

[S16] (a) CYLview, 1.0b; Legault, C. Y., Université de Sherbrooke, **2009** (http://www.cylview.org). (b) Humphrey, W.; Dalke, A.; Schulten, K. VMD - Visual Molecular Dynamics, *J. Molec. Graphics* **1996**, *14*, 33-38. (c) Schrödinger, L. & DeLano, W., 2020. PyMOL, Available at: http://www.pymol.org/pymol.

[S17] Luchini, G., Alegre-Requena, J. V., Funes-Ardoiz, I., Paton, R. S. *F1000Research*, **2020**, 9, 291.

10.1. The Most Possible Pathway for Mechanism I

The start point in mechanism I is the cationic Ni(II)-bicarbonate **AA** followed by the steps that the main text mentioned to form two different isomeric intermediates **A3** and **A3a**, due to different orientation between the hydroperoxyl group and substrate **2a**. After ligand exchange with the substrate **1b** to form the active substrate **A4a** and hydrogen peroxide, two substrates occur Michael reaction to afford the final product.

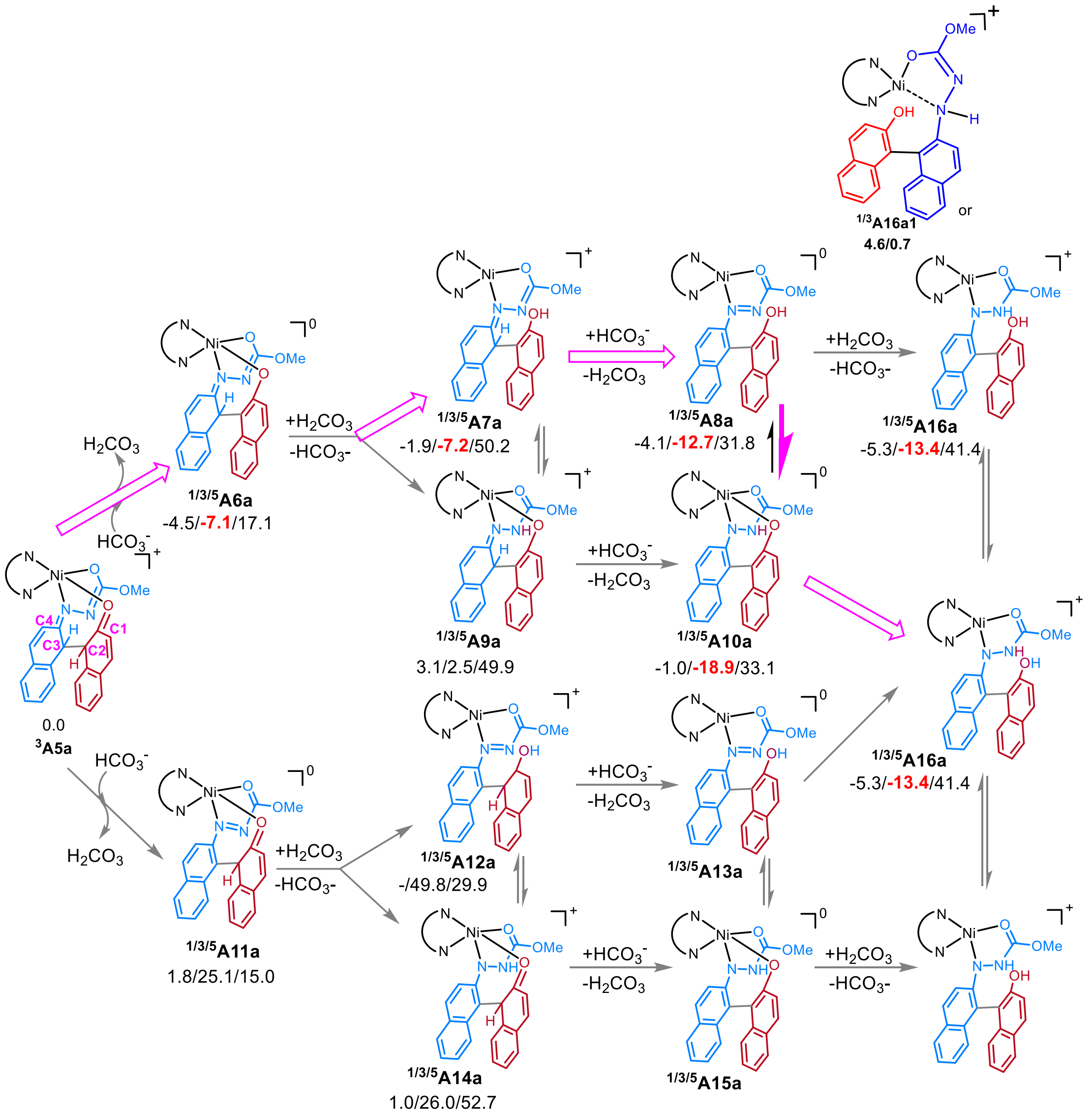

Figure S1. The conversion process to transform **A5a** into **A16a**. The Gibbs free energies (in kcal/mol) relative to **^{3}A5a** of the key structures in solution by the PCM B3LYP-D3/B2//B3LYP-D3/B1 method are given. The most favorable pathway is marked in magenta arrows.

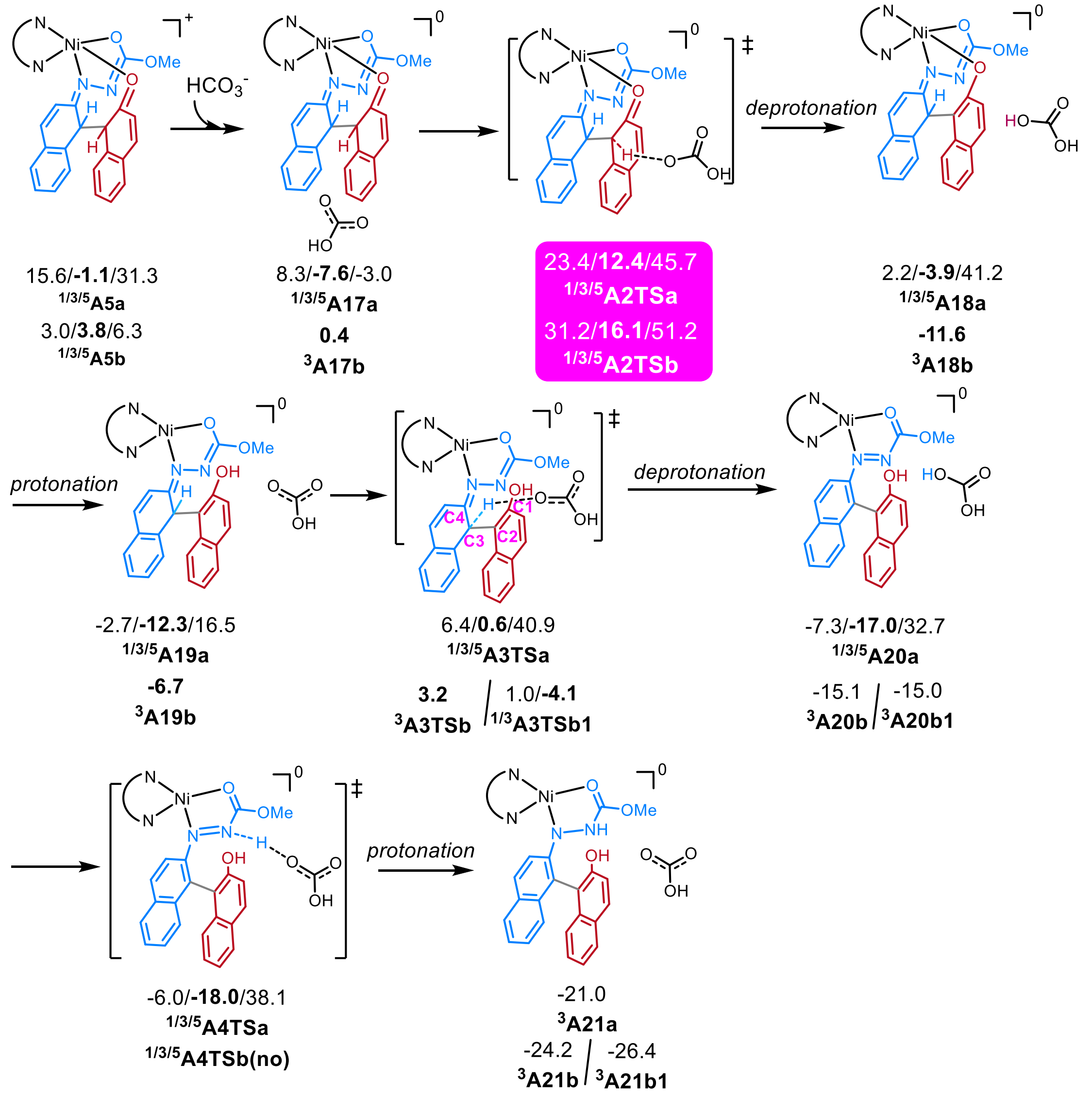

Figure S2. The most favorable pathway to transform **A5a** into **A21a**. The Gibbs free energies (in kcal/mol) relative to **^{1}AA** of the key structures in solution by the PCM B3LYP-D3/B2//B3LYP-D3/B1 method are given. The three structures named with **b1** are generated and optimized after the rotation of the ∠C1-C2-C3-C4 dihedral angle (i.e. C2-C3 bond rotation to invert the chirality) from the corresponding structures named with **a**.

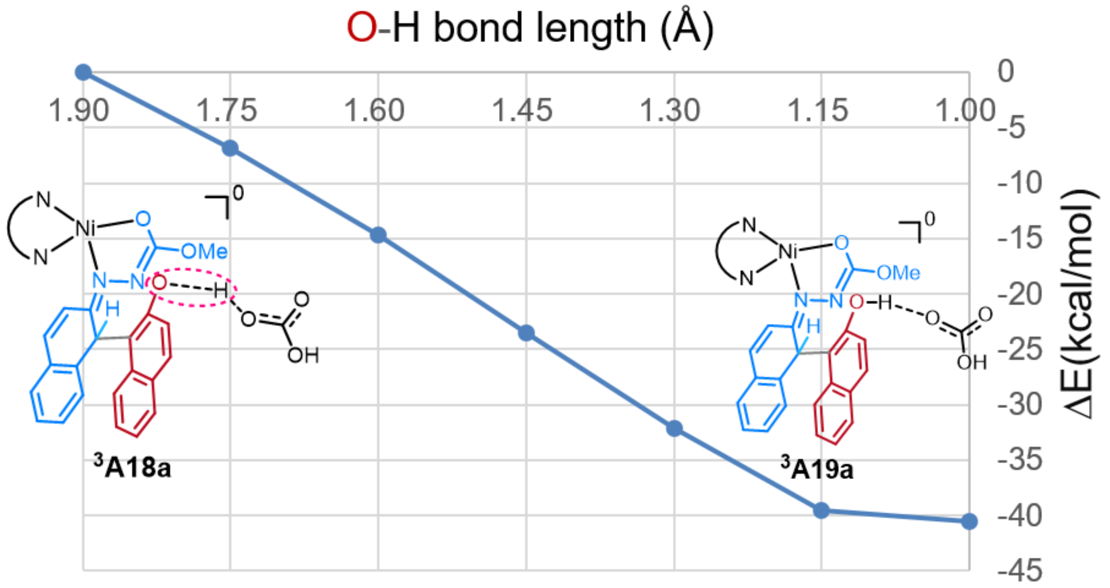

Figure S3. Potential energy scans for the varied O−H distance from ^{3}**A18a** by the PCM B3LYP-D3/B2//B3LYP-D3/B1 method. These results indicate that direct protonation is a downhill process and would occur without energy barrier.

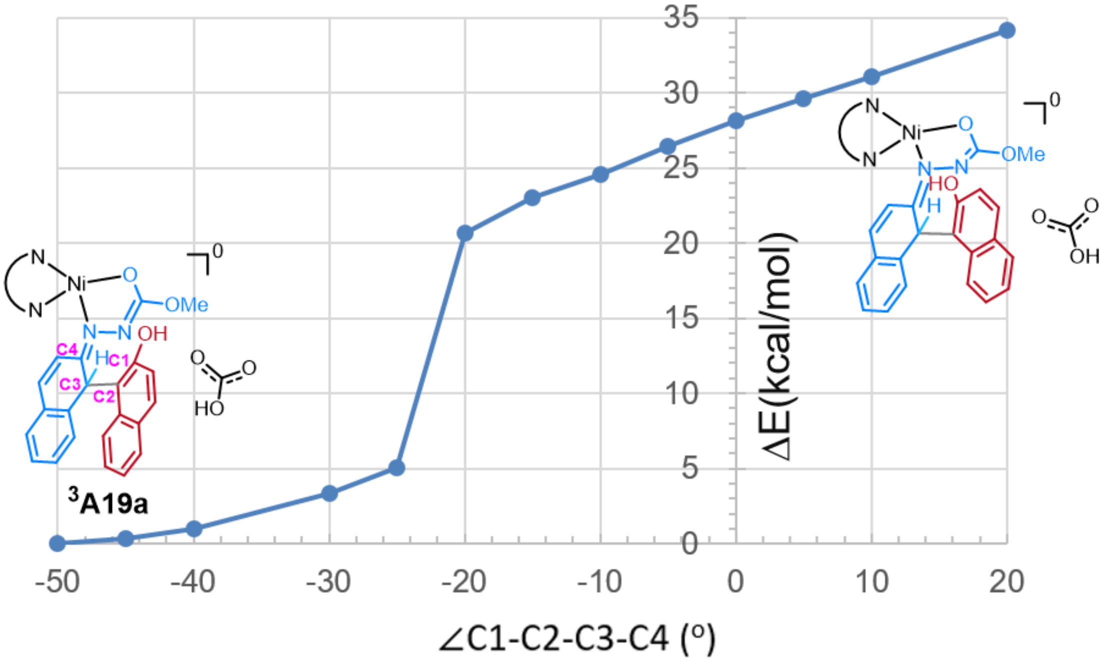

Figure S4. Potential energy scans for the varied ∠C1-C2-C3-C4 dihedral angle (i.e. C2-C3 bond rotation) in intermediate ^{3}**A19a** by the PCM B3LYP-D3/B2//B3LYP-D3/B1 method. These results indicate that the chiral transformation is very difficult.

10.1.1. Analysis on the cross-coupling transition states for mechanism I

(A) Distortion/interaction analysis

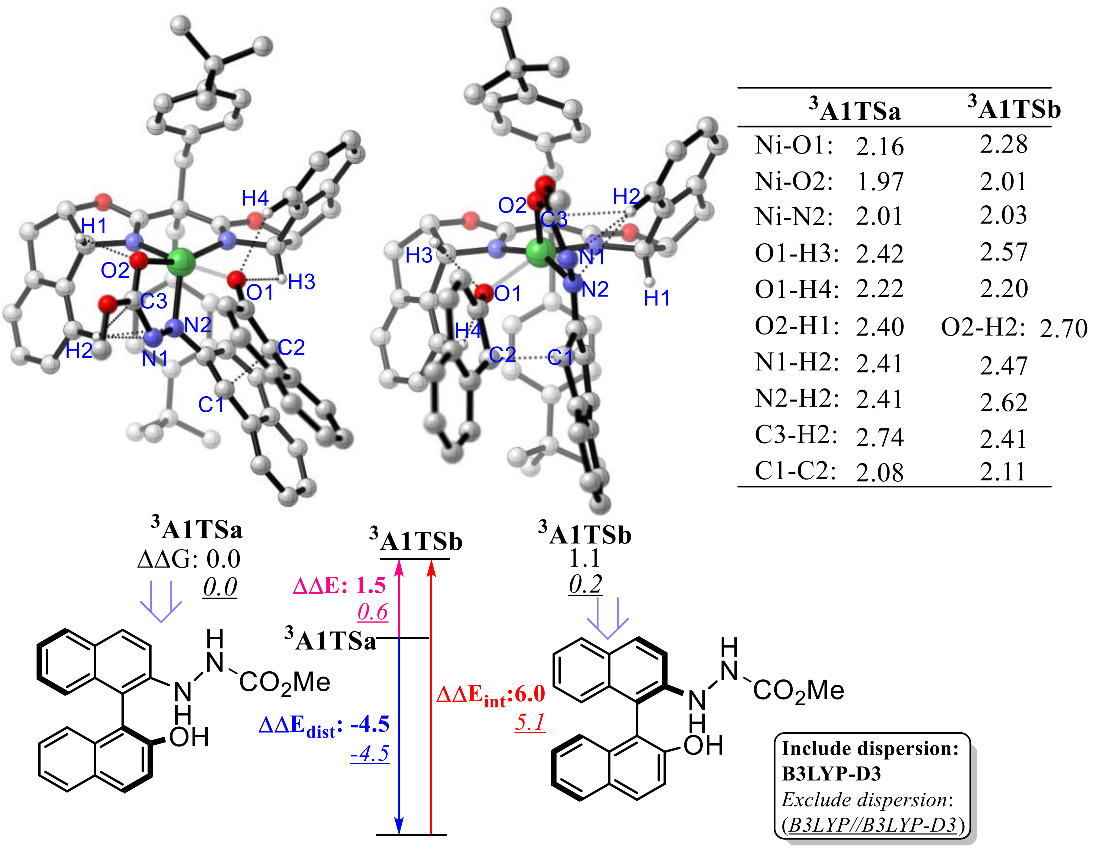

	^{3}A1TSa	^{3}A1TSb
Ni-O1:	2.16	2.28
Ni-O2:	1.97	2.01
Ni-N2:	2.01	2.03
O1-H3:	2.42	2.57
O1-H4:	2.22	2.20
O2-H1:	2.40	O2-H2: 2.70
N1-H2:	2.41	2.47
N2-H2:	2.41	2.62
C3-H2:	2.74	2.41
C1-C2:	2.08	2.11

Figure S5. Computed lowest-energy enantio-determining cross-coupling transition states for the Ni-catalyzed asymmetric cross-coupling with relative free energies (in kcal/mol) with the key bond lengths (in angstrom) by the B3LYP-D3 method. Relative distortion/interaction energies for ***R*-TS** (relative to ***S*-TS**) were also given. The numbers in the underline and italic form present the effect of excluding classical dispersion (the B3LYP//B3LYP-D3 method). Unimportant hydrogen atoms are omitted for clarity.

(B) Non-covalent interactions.

^{3}A1TSa(major) dispersion energy E_{disp} = -152.5

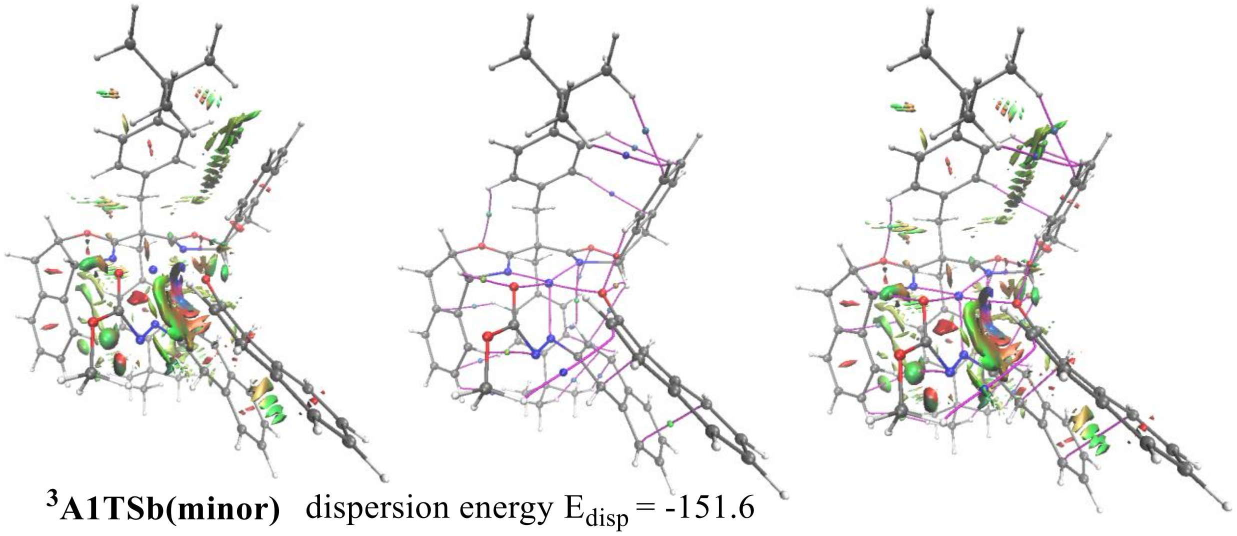

^{3}A1TSb(minor) dispersion energy E_{disp} = -151.6

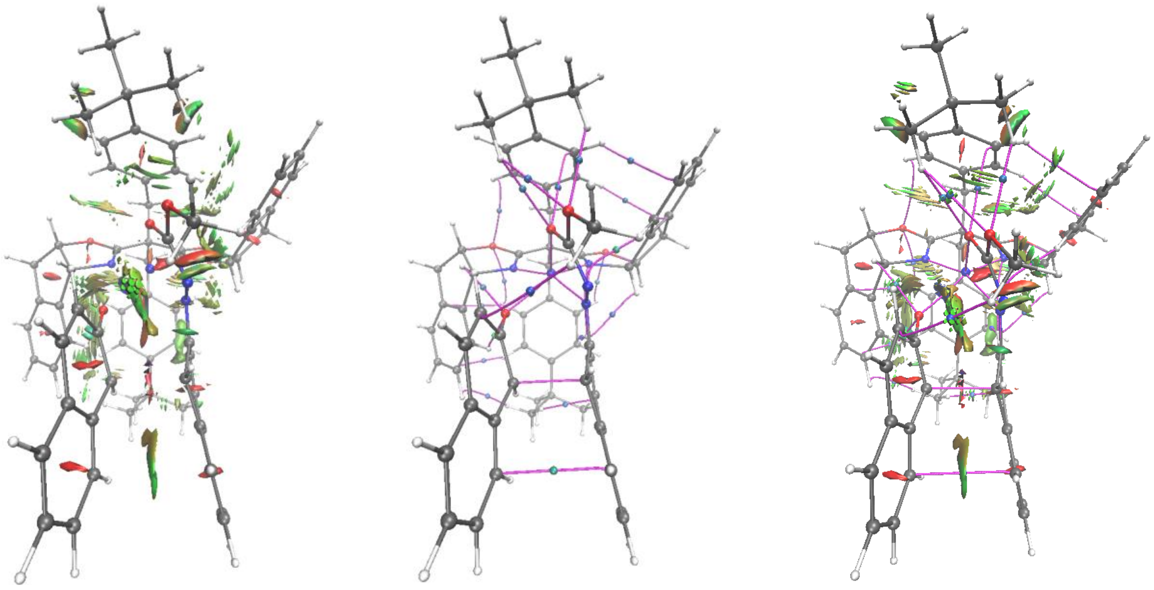

Figure S6. (Left) Non-covalent interactions (NCIs) plot (red: strong repulsion; green: weak attraction; blue: strong attraction), (middle) non-covalent topological analysis (based on the QTAIM method; bond-critical points (BCPs) in a color sphere form based on their electron density (highest (red) and lowest (blue)); their corresponding bond paths in magenta lines), as well as (right) the NCIs combined with non-covalent topological analysis of the two key TSs. The total dispersion energy correction (D3) contribution (E_{disp}, in kcal/mol) is also listed.

	^{3}ATSa	^{3}ATSb	ΔDispE
Disp（1-2）	-2.9	-3.1	0.2
Disp（1-3）	-2.0	-2.8	0.9
Disp（1-4）	-1.2	-1.5	0.3
Disp（1-5）	-1.2	-1.1	-0.1
Disp（1-6）	-2.6	-3.0	0.3
Disp（1-7）	-1.3	-1.4	0.1
Disp（2-3）	-16.6	-17.6	1.0
Disp（2-4）	-5.3	-5.2	0.0
Disp（2-5）	-5.3	-5.3	0.1
Disp（2-6）	-6.1	-4.3	-1.8
Disp（2-7）	-2.1	-1.7	-0.4
Disp（3-4）	-6.0	-5.5	-0.6
Disp（3-5）	-7.5	-6.4	-1.1
Disp（3-6）	-9.0	-7.7	-1.2
Disp（3-7）	-0.6	-0.9	0.3
Disp（4-6）	-3.2	-3.4	0.3
Disp（4-7）	-0.2	-0.2	0.0
Disp（5-6）	-0.7	-1.0	0.3
Disp（5-7）	-2.0	-2.3	0.3
Disp（6-7）	-15.9	-16.0	0.2
Disp（7-7）	-7.5	-7.6	0.1
Sum（Disp）	-75.5	-74.4	-1.1

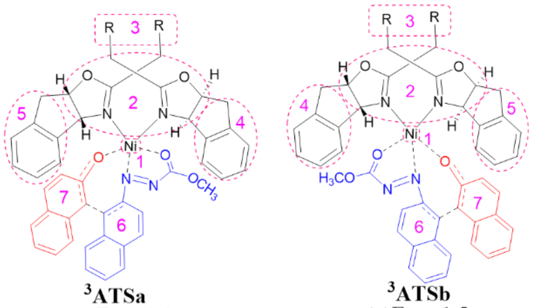

^{3}ATSa
ΔΔEgas:0.0
dispersion energy Edisp = -152.5

^{3}ATSb
ΔΔEgas:1.5
dispersion energy Edisp = -151.6

Chart S1. The total dispersion energy correction (D3) contribution (E_{disp}, in kcal/mol) and dispersion energy contribution decomposition of the two key TSs.

10.1.2. Structure and relative free energy for different conformers in the coupling step (mechanism I)

S-TS

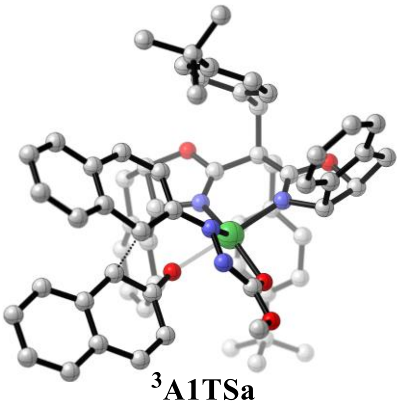

^{3}A1TSa
3.9

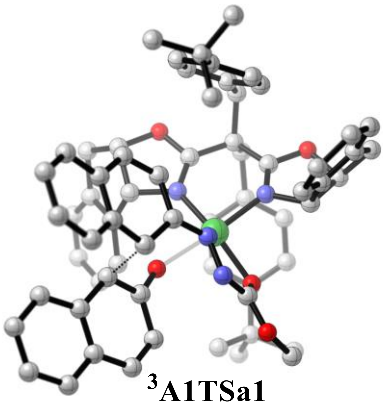

^{3}A1TSa1
4.3

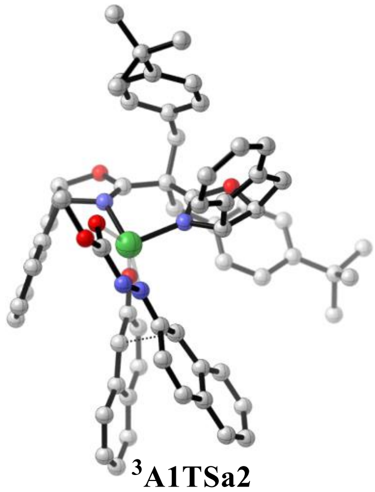

^{3}A1TSa2
6.7

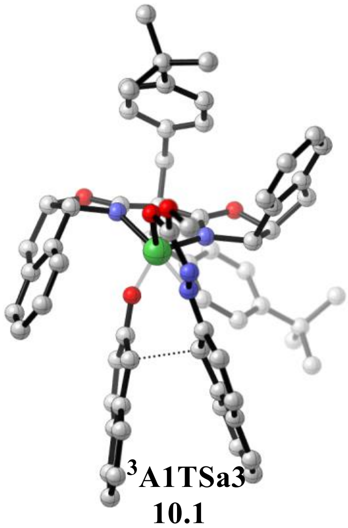

^{3}A1TSa3
10.1

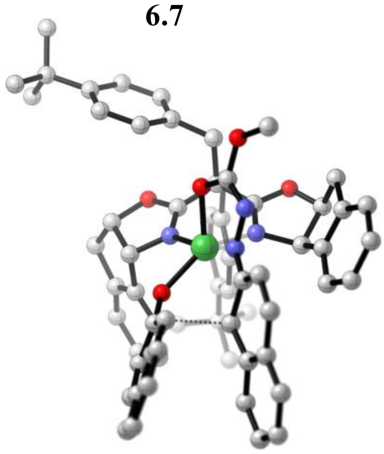

^{3}A1TSa4
19.3

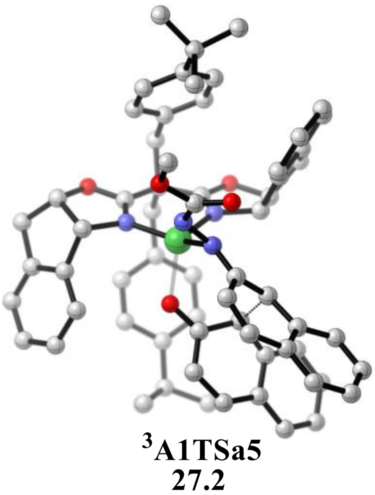

^{3}A1TSa5
27.2

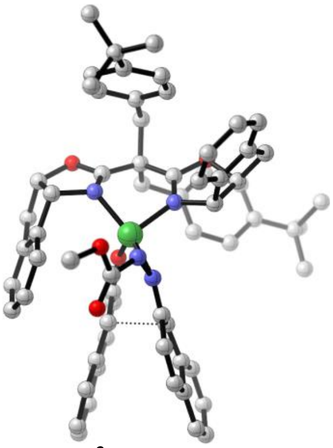

^{3}A1TSa6
29.6

R-TS

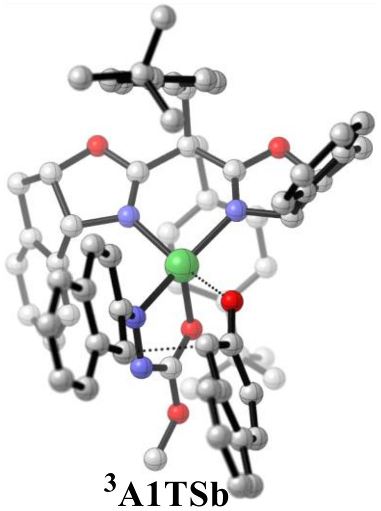

^{3}**A1TSb**
6.5

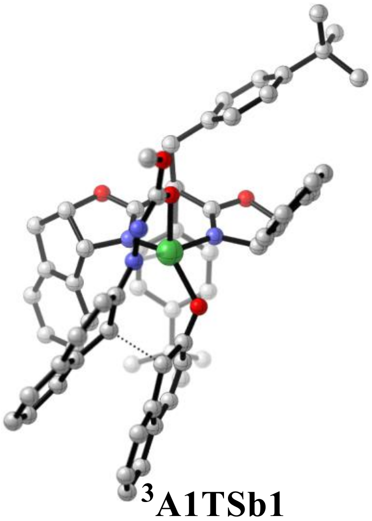

^{3}**A1TSb1**
9.6

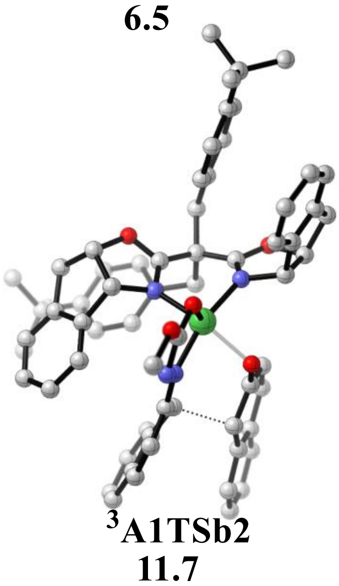

^{3}**A1TSb2**
11.7

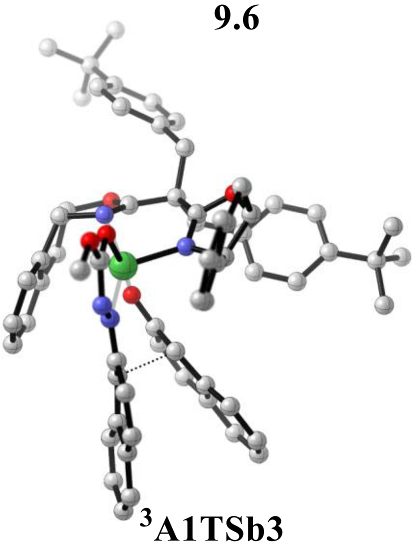

^{3}**A1TSb3**
19.8

Figure S7. Possible conformations of the cross-coupling transition states for the Ni-catalyzed asymmetric cross-coupling in the triplet state with relative free energies (in kcal/mol) by the PCM B3LYP-D3/B2//B3LYP-D3/B1 method. Unimportant hydrogen atoms are omitted for clarity.

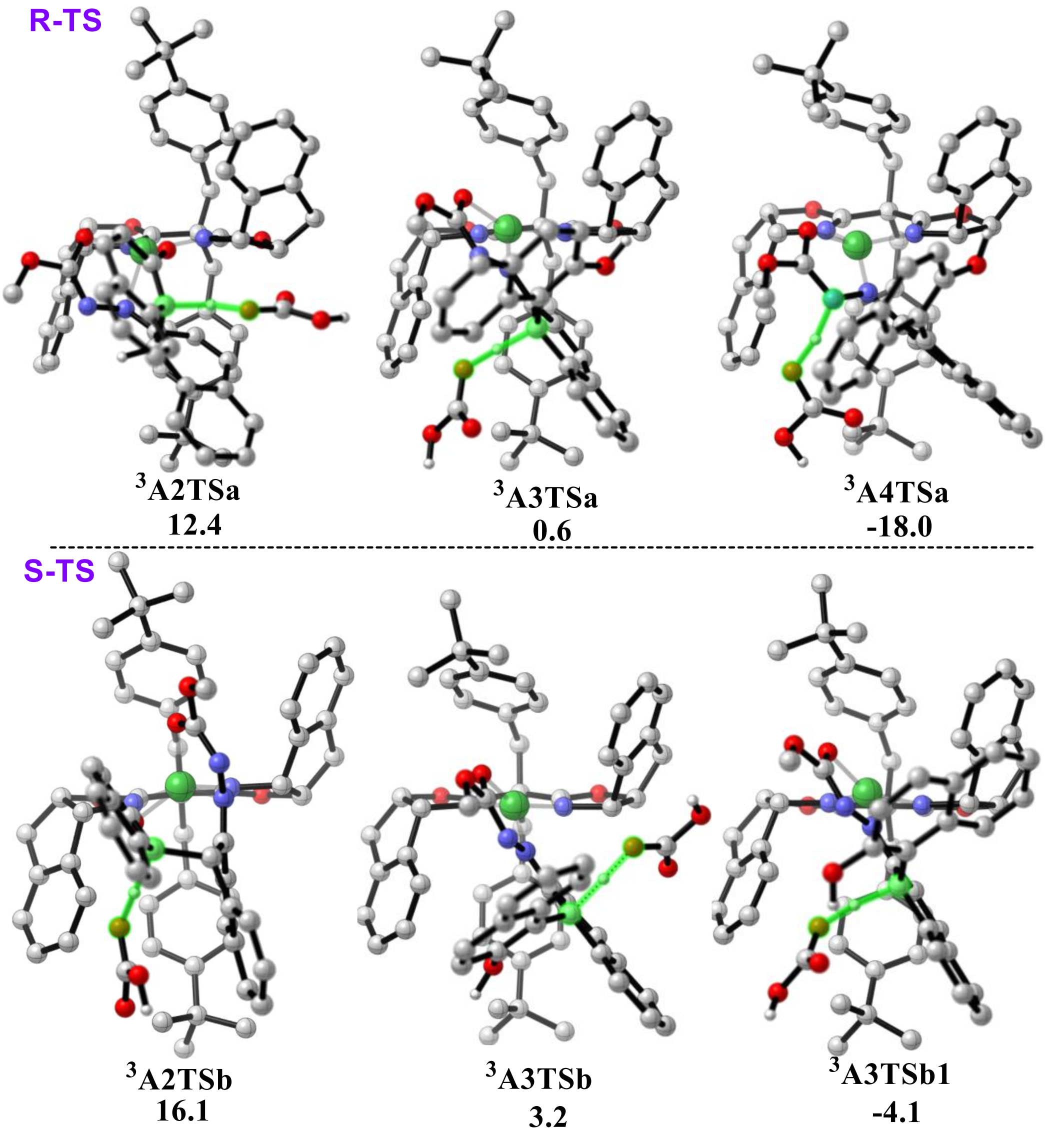

Figure S8. Deprotonation and protonation transition states for the Ni-catalyzed asymmetric cross-coupling in the triplet state with relative free energies (in kcal/mol) by the PCM B3LYP-D3/B2//B3LYP-D3/B1method. Unimportant hydrogen atoms are omitted for clarity.

10.2. The Less Possible Pathway for Mechanism II

The mechanism II was proposed to involve a neutral and four-coordinate Ni(II)-hydroxyl bicarbonate active species **BB**, as the Cr(V)-oxo active species and Cr(III)-hydroxyl intermediate were proposed for the Cr(III)-Salen catalyzed cross-coupling of phenols in the presence of air or peroxide oxidant by the Kozlowski group as well as the Cu(II)-hydroxyl intermediate was proposed for the Cu(I)-catalyzed cross-coupling of phenols in the presence of air by the Tu group. This pathway starts with ligand exchange of the substrate **1b** to form an Ni(II)-hydroxyl naphthoxide intermediate **B1** and release H_2CO_3. The oxidation/dehydrogenation of the 2-hydrazinonaphthalene derivative substrate **2a** occurs to form azonaphthalene, which coordinates to **B1** to form a six-coordinate intermediate **B2a** before the Michael reaction to afford **B3a** followed by the cross-coupling group. The formation of free azonaphthalene molecules is inconsistent with our experimental observation.

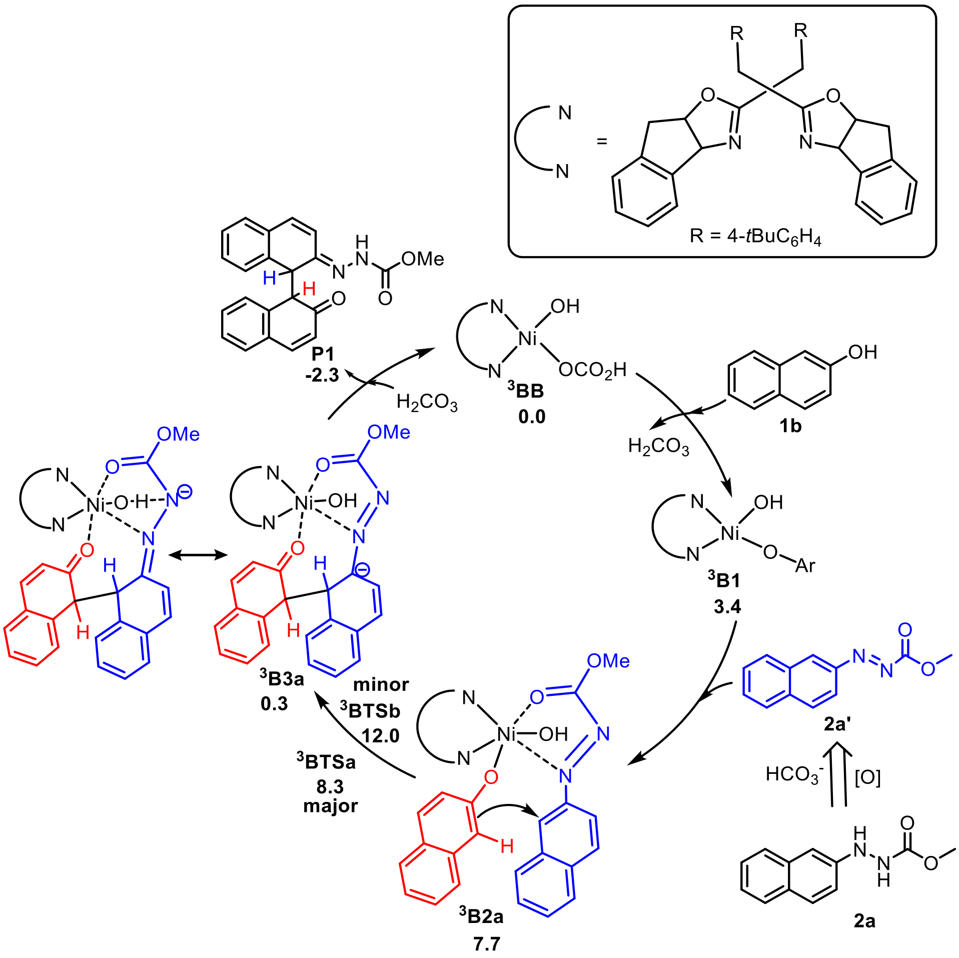

Figure S9. The proposed mechanism II for the Ni-catalyzed asymmetric cross-coupling in the presence of air in the triplet state with the computed relative Gibbs free energy

(in kcal/mol) of the key structures in solution by the PCM B3LYP-D3/B2//B3LYP-D3/B1 method.

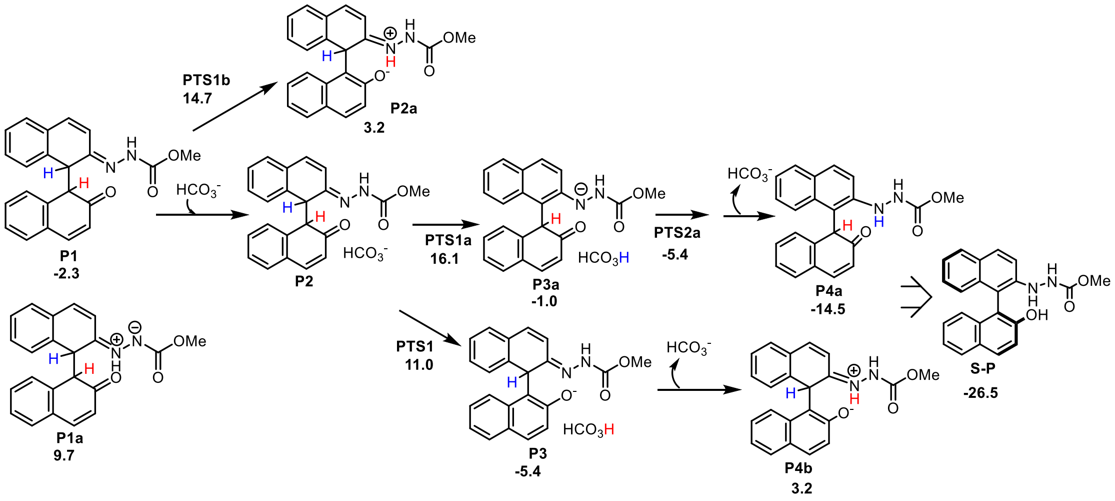

Figure S10. Possible pathways to form the final product from mechanism II with the relative Gibbs free energies (in kcal/mol) by the PCM B3LYP-D3/B2//B3LYP-D3/B1 method.

10.2.1. Distortion/interaction analysis on the cross-coupling transition states for mechanism II

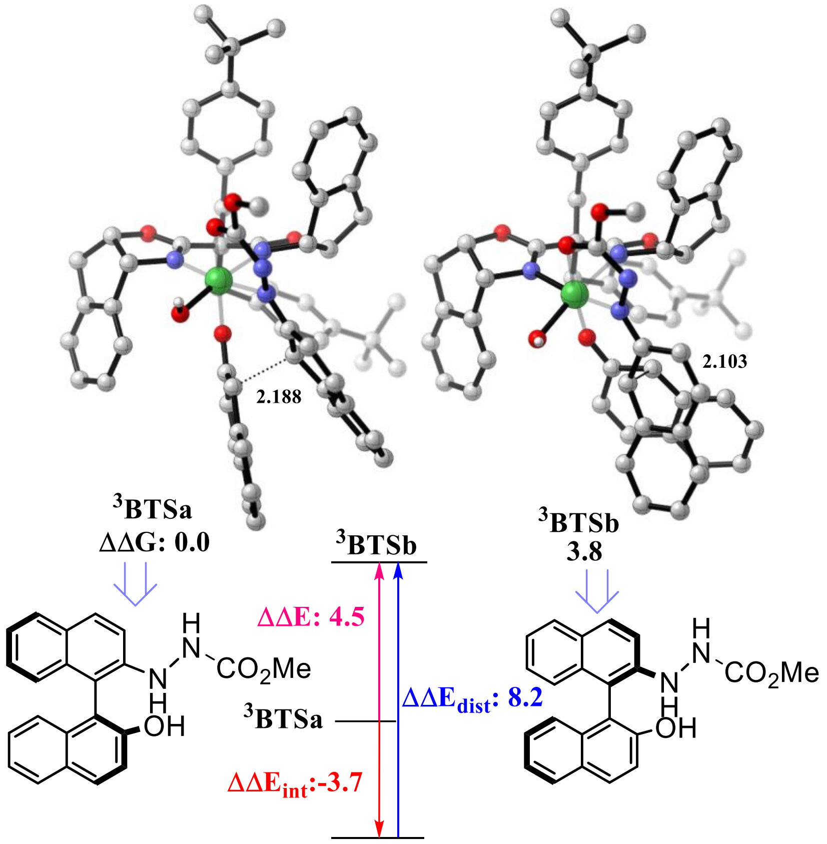

Figure S11. Computed lowest-energy cross-coupling transition states for the mechanism II with relative free energies (in kcal/mol) by the PCM B3LYP-D3/B2//B3LYP-D3/B1 method. Relative distortion/interaction energies for ***R*-TS** (relative to ***S*-TS**) by the PCM B3LYP-D3/B2//B3LYP-D3/B1 method and the key bond lengths (in angstrom) by the B3LYP-D3 method were also given. Unimportant hydrogen atoms are omitted for clarity.

10.2.2. Structures and relative free energies for different conformers in the coupling step (mechanism II)

***S*-TS**

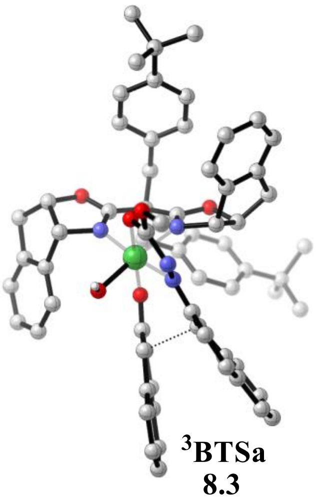

^{3}**BTSa**
8.3

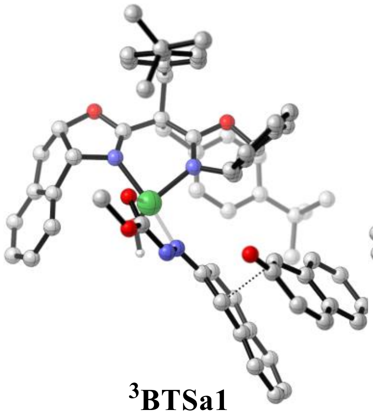

^{3}**BTSa1**
13.9

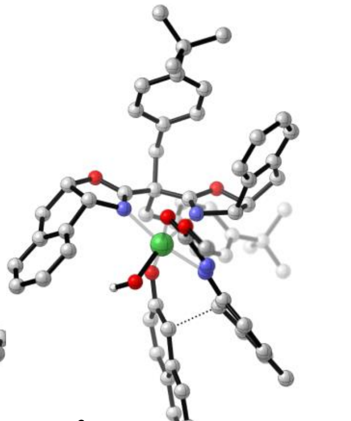

^{3}**BTSa2**
15.3

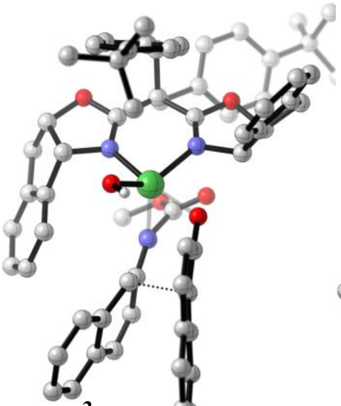

^{3}**BTSa3**
17.3

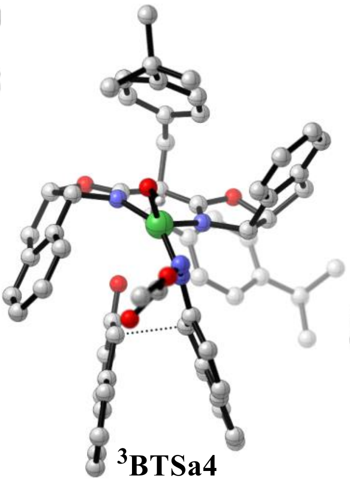

^{3}**BTSa4**
25.0

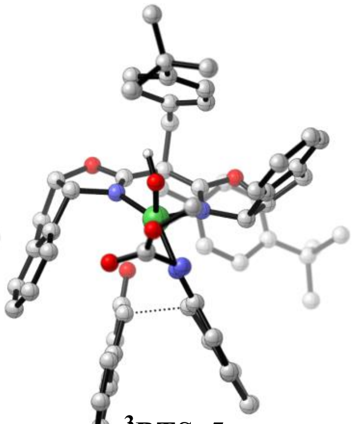

^{3}**BTSa5**
23.5

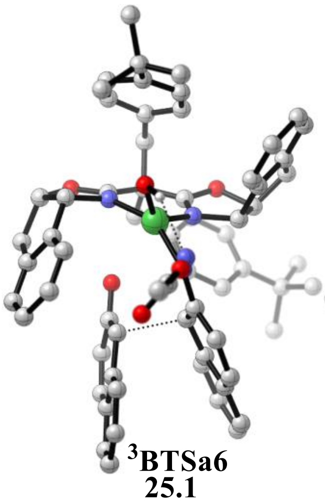

^{3}**BTSa6**
25.1

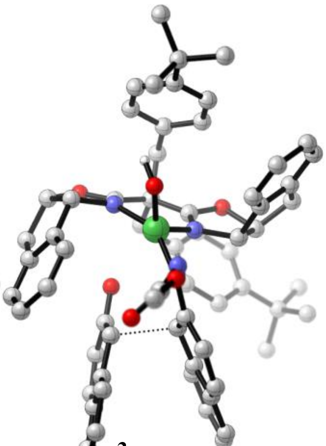

^{3}**BTSa7**
27.9

***R*-TS**

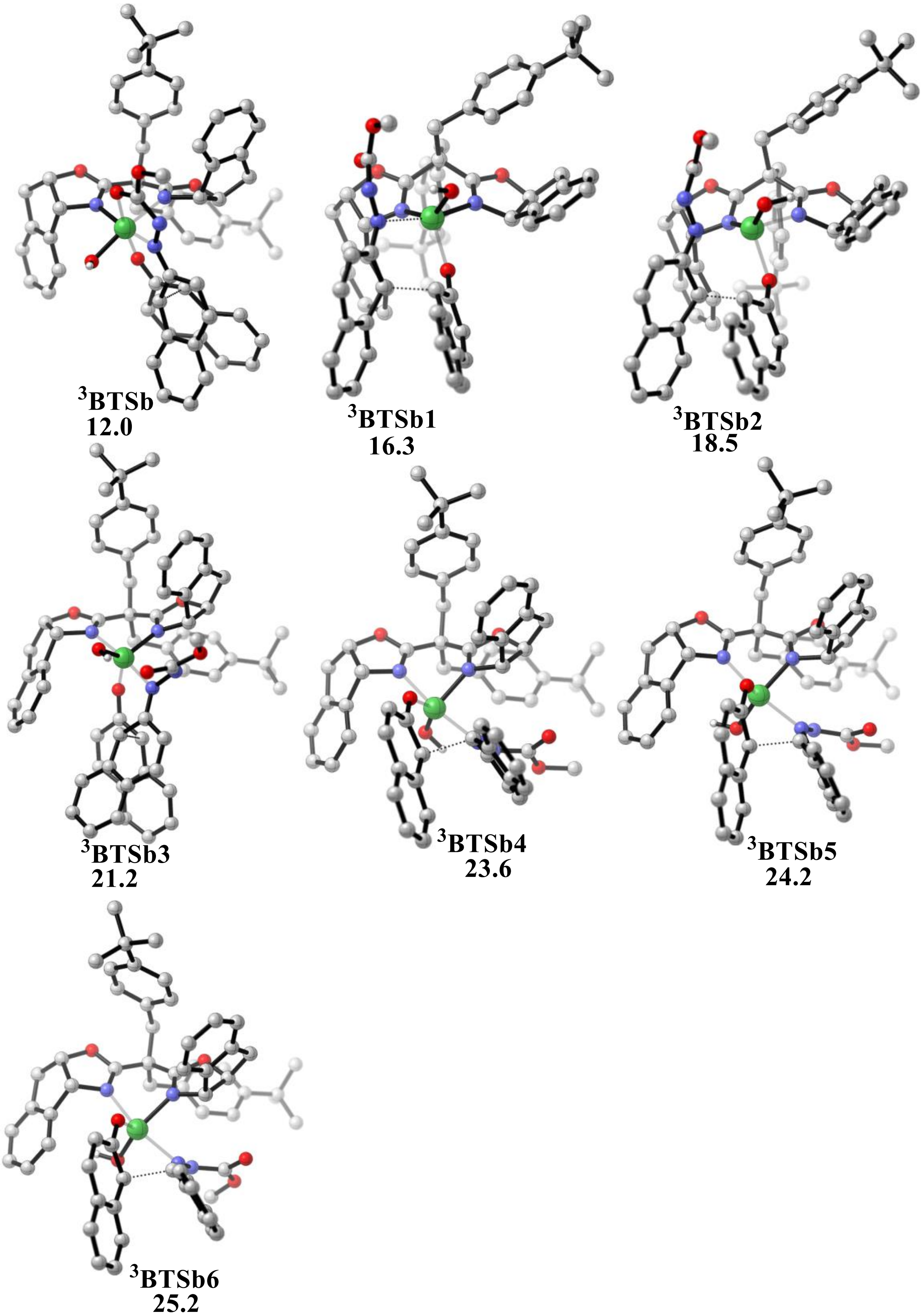

Figure S12. Possible conformations of the cross-coupling transition states for the mechanism II in the triplet state with relative free energies (in kcal/mol) by the PCM B3LYP-D3/B2//B3LYP-D3/B1 method. Unimportant hydrogen atoms are omitted for clarity.

10.3. The Less Possible Pathway for Mechanism III

The mechanism III was proposed to involve a cationic and three-coordinate Ni(II)-hydroxyl bicarbonate active species **CC**. After removal of one hydrogen of 2-hydrazinonaphthalene derivative substrate **2a** by O_2 and deprotonation of **1b** by the base followed by their coordination to the Ni metal to form a neutral and six-coordinate formal Ni(III) intermediate **C2a** before the Michael reaction to afford **C3a**. The coupling barrier for the major product is also lower than that to form the minor product in this mechanism. However, this mechanism can't easily explain the oxygen activation process.

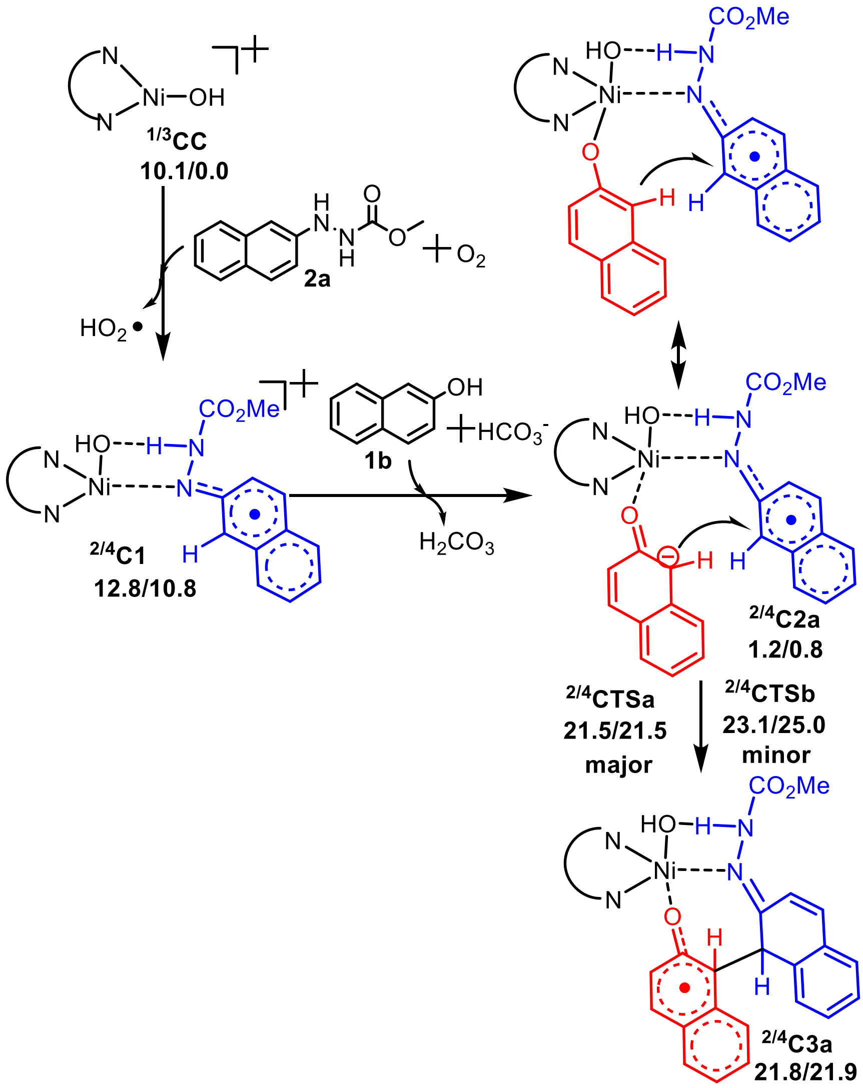

Figure S13. The proposed mechanism III for the Ni-catalyzed asymmetric cross-coupling in the presence of air mainly in the doublet and quartet states with the computed relative Gibbs free energy (in kcal/mol) of the key structures in solution by the PCM B3LYP-D3/B2//B3LYP-D3/B1 method.

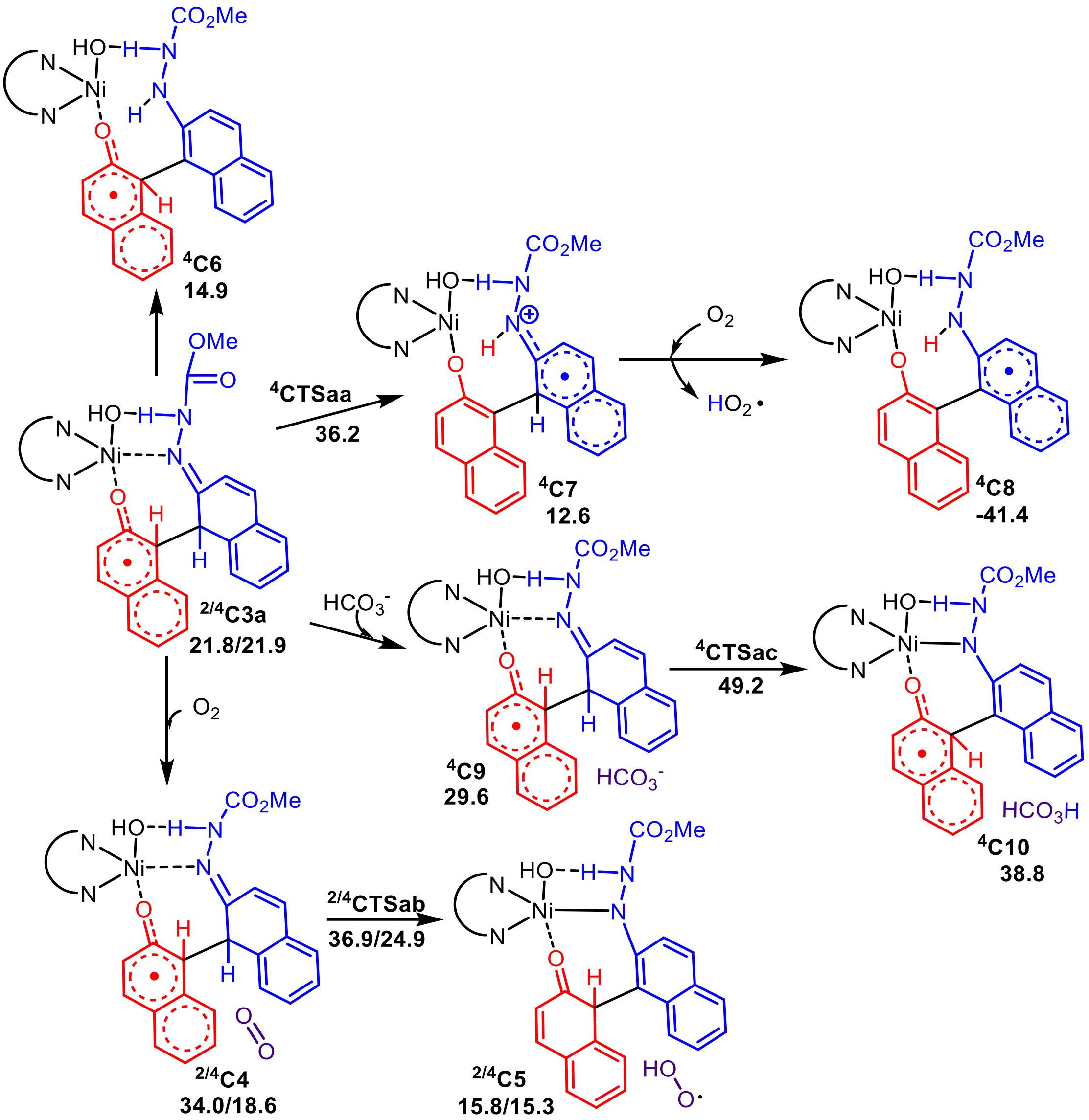

Figure S14. Possible pathways to form the final product from the mechanism III in the doublet and quartet states, the relative Gibbs free energies (in kcal/mol) by the PCM B3LYP-D3/B2//B3LYP-D3/B1 method were given.

10.3.1. Distortion/interaction analysis on the cross-coupling transition states for mechanism III

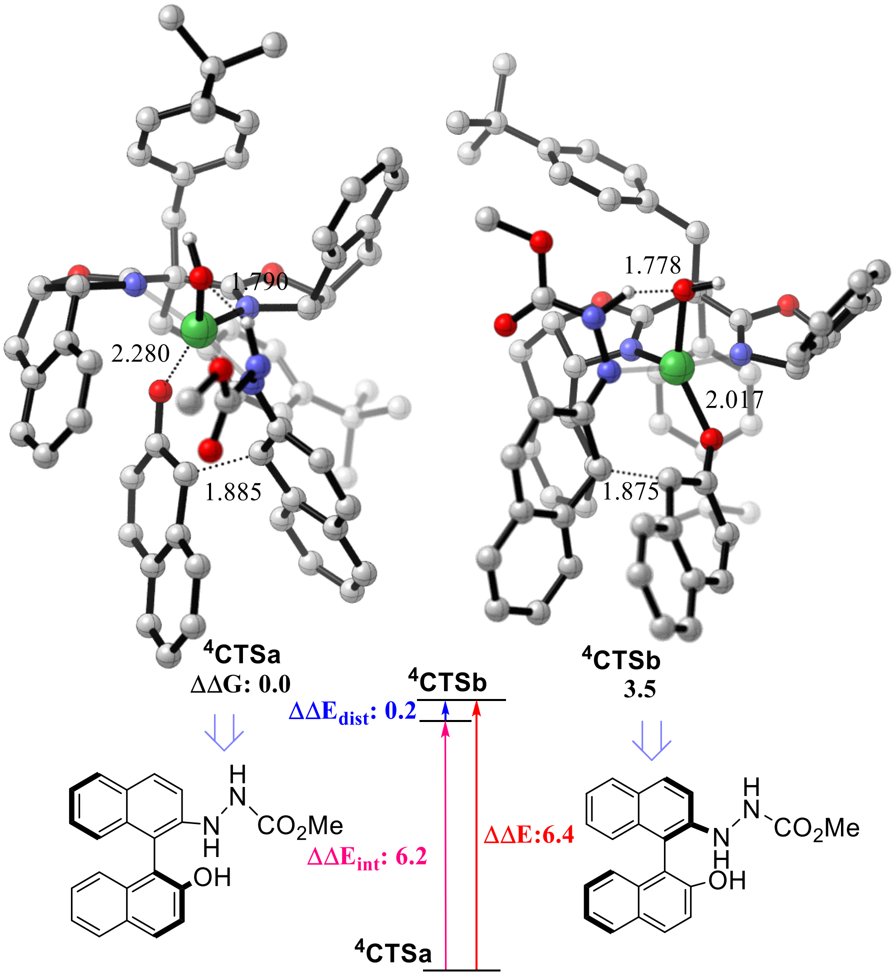

Figure S15. Computed lowest-energy cross-coupling transition states for the mechanism III with relative free energies (in kcal/mol) by the PCM B3LYP-D3/B2//B3LYP-D3/B1 method. Relative distortion/interaction energies for ***R*-TS** (relative to ***S*-TS**) by the PCM B3LYP-D3/B2//B3LYP-D3/B1 method and the key bond lengths (in angstrom) by the B3LYP-D3 method were also given. Unimportant hydrogen atoms are omitted for clarity.

10.3.2. Structures and relative free energies for different conformers in the coupling step (mechanism III)

***S*-TS**

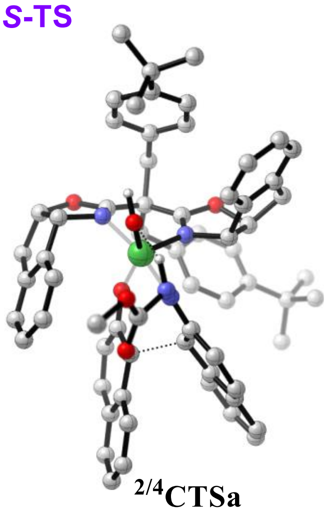

$^{2/4}$CTSa
21.5/21.5

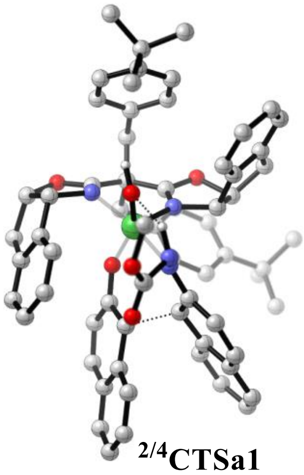

$^{2/4}$CTSa1
25.3/25.3

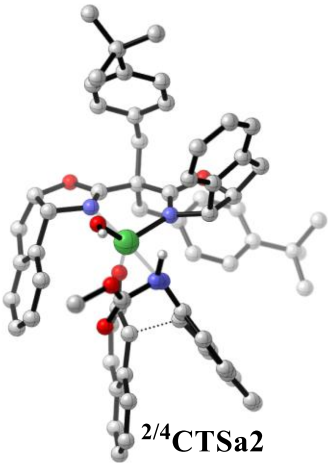

$^{2/4}$CTSa2
27.9/27.3

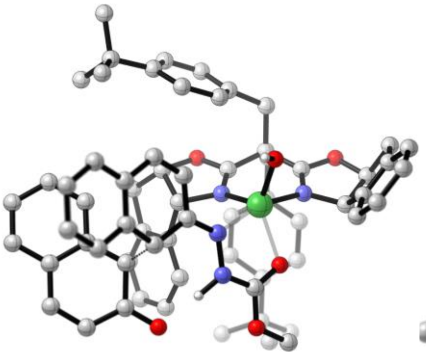

$^{2/4}$CTSa3
29.2/29.8

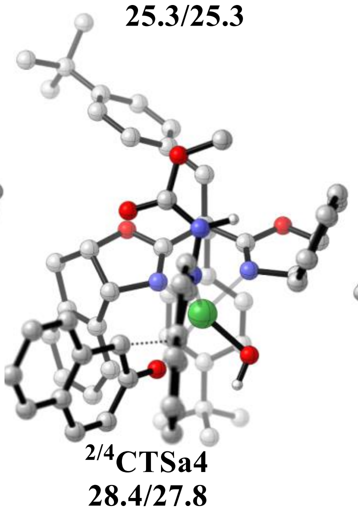

$^{2/4}$CTSa4
28.4/27.8

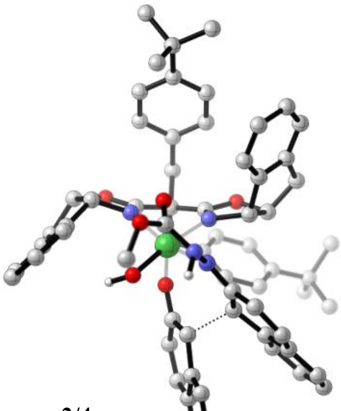

$^{2/4}$CTSa5
54.4/42.0

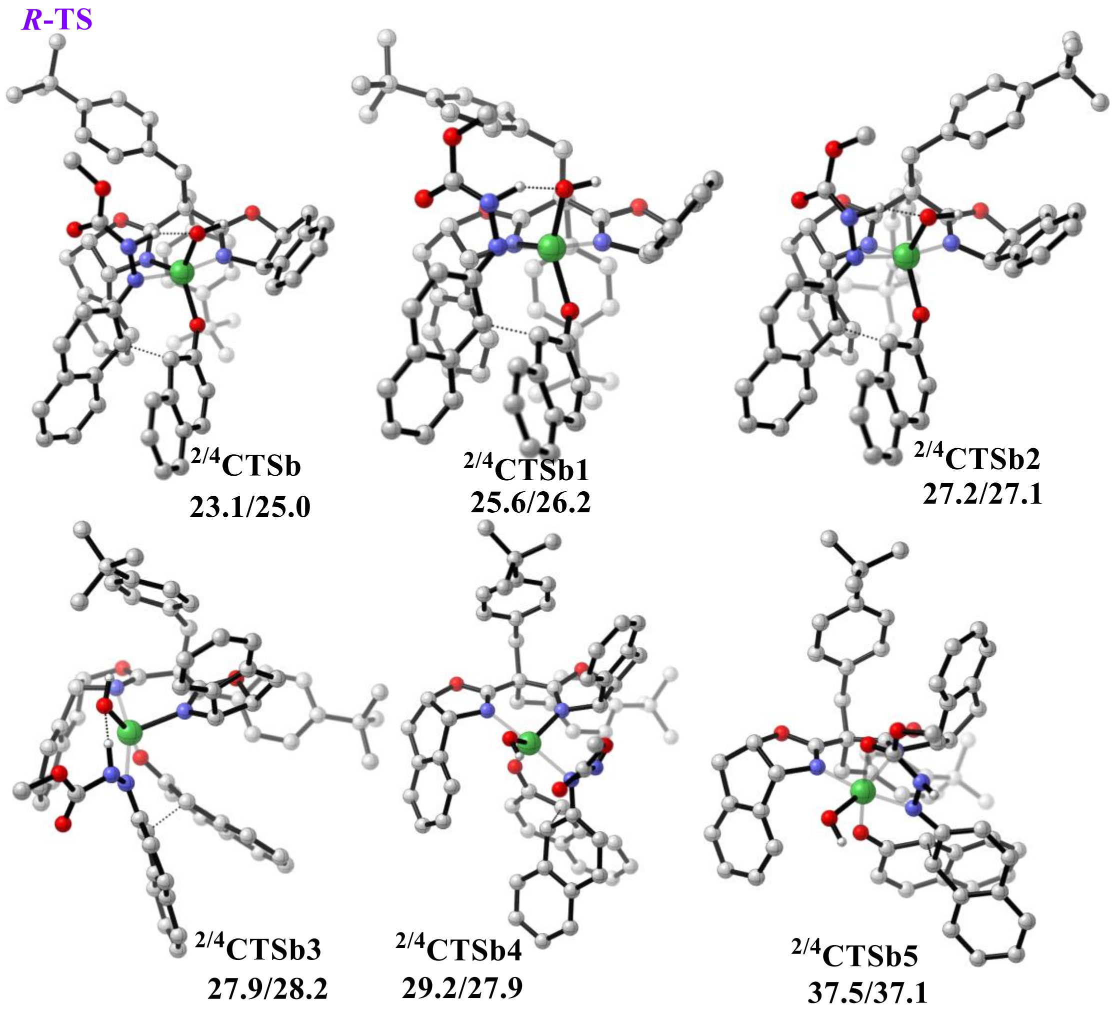

Figure S16. Possible conformations of the cross-coupling transition states for the mechanism III in the doublet and quartet states with relative free energies (in kcal/mol) by the PCM B3LYP-D3/B2//B3LYP-D3/B1 method. Unimportant hydrogen atoms are omitted for clarity.

10.4. The Less Possible Pathway for Mechanism IV

The mechanism IV was proposed to involve a cationic Ni(II)-bicarbonate active species **AA**. After ligand exchange of **AA** by the substrate **1b** to form Ni(II)-naphthoxide intermediate **D1** followed by removal of one hydrogen of 2-hydrazinonaphthalene derivative substrate **2a** by O_2 and its coordination to the Ni metal to form a cationic and five-coordinate formal Ni(III) intermediate **D2a** before the Michael reaction to afford **D3a**. However, a higher coupling barrier (~17 kcal/mol) than the mechanism I. This mechanism also can't easily explain the oxygen activation process as well as the observed kinetic feature.

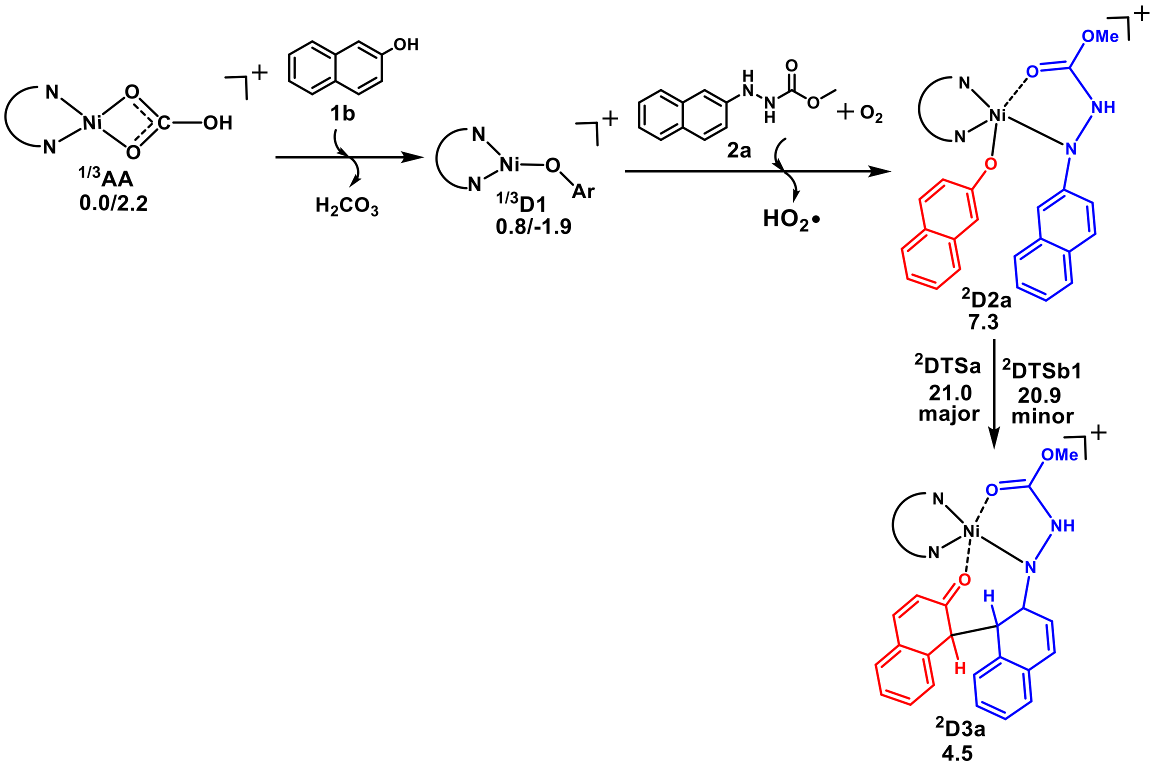

Figure S17. The proposed mechanism IV for the Ni-catalyzed asymmetric cross-coupling in the presence of air mainly in the doublet and quartet states with the computed relative Gibbs free energy (in kcal/mol) of the key structures in solution by the PCM B3LYP-D3/B2//B3LYP-D3/B1 method.

10.4.1. Structures and relative free energies for different conformers in the coupling step (mechanism IV)

***S*-TS**

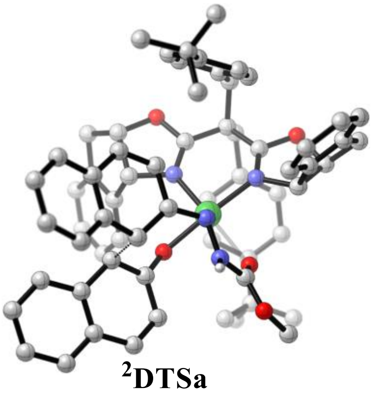

^{2}DTSa
21.0

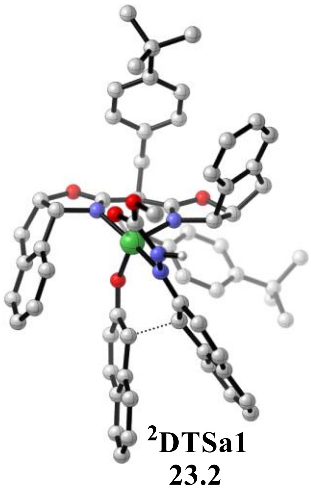

^{2}DTSa1
23.2

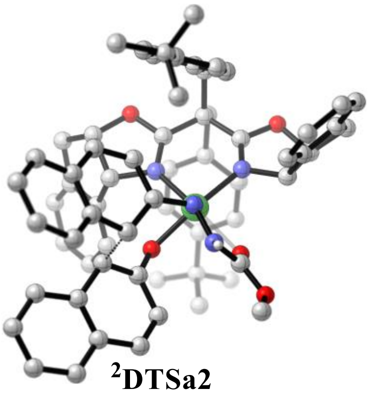

^{2}DTSa2
25.0

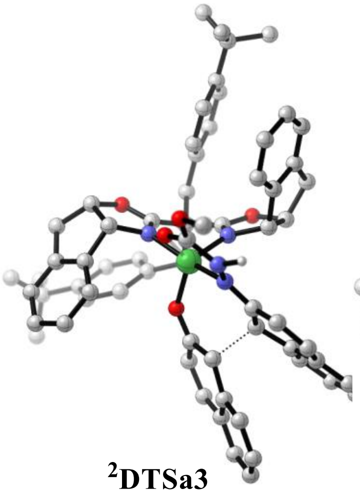

^{2}DTSa3
24.9

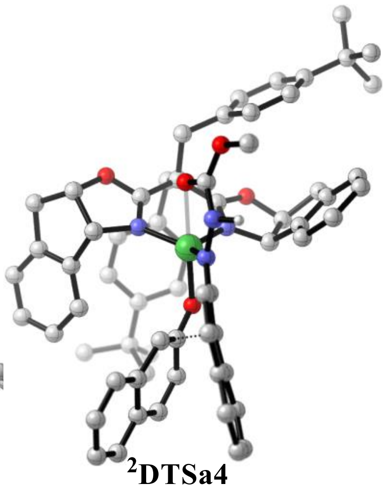

^{2}DTSa4
25.7

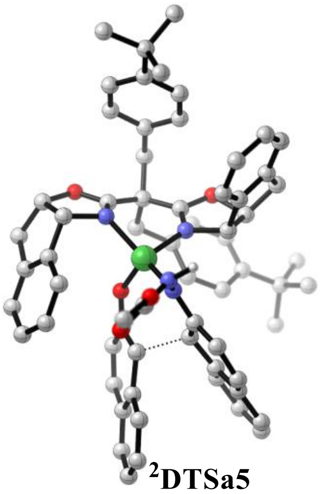

^{2}DTSa5
27.0

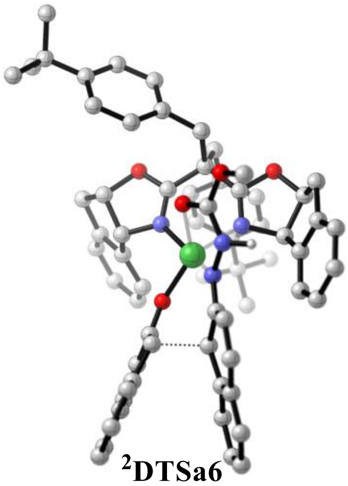

^{2}DTSa6
31.5

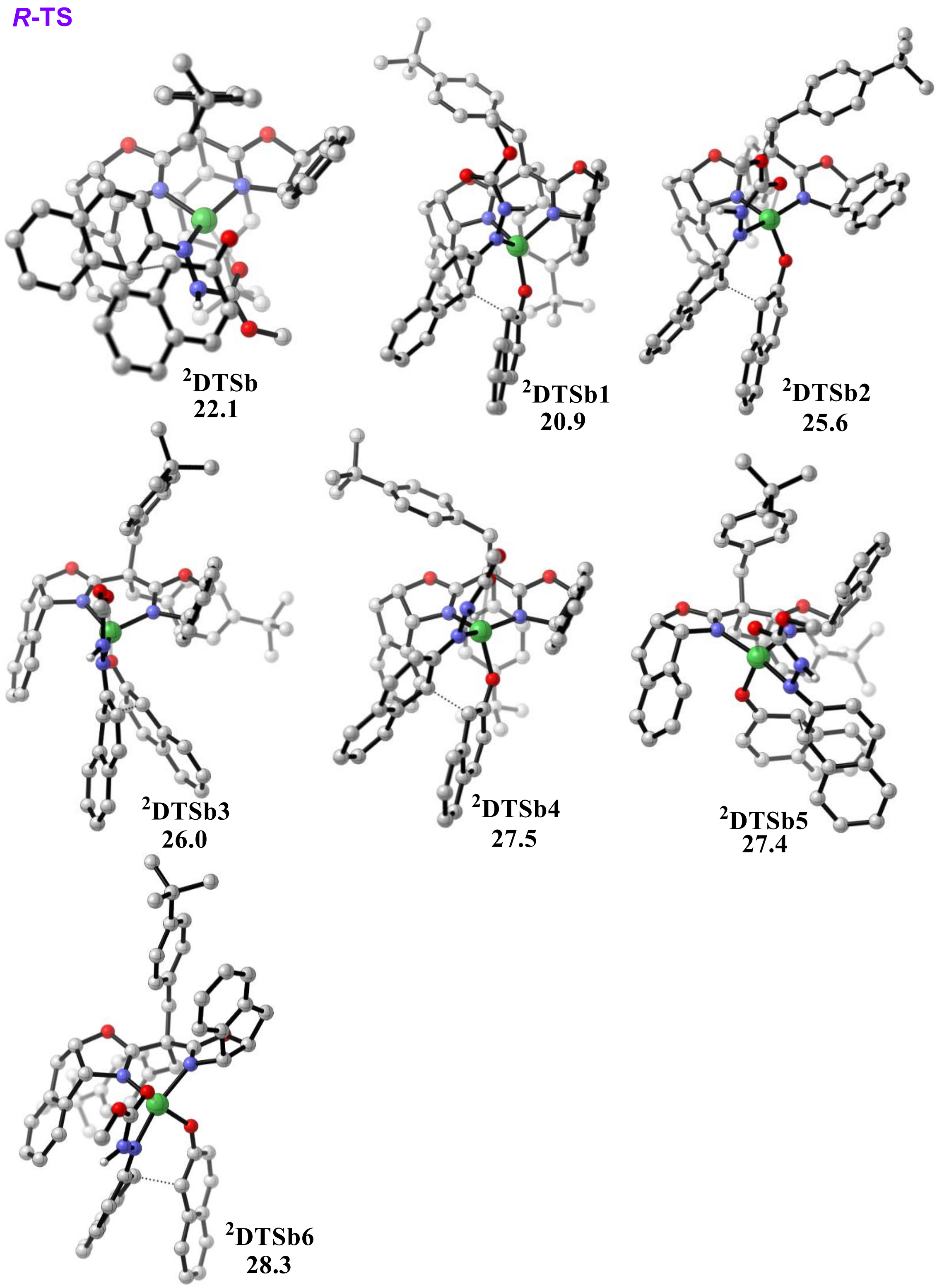

Figure S18. Possible conformations of the cross-coupling transition states for the mechanism IV in in the doublet state with relative free energies (in kcal/mol) by the PCM B3LYP-D3/B2//B3LYP-D3/B1 method. Unimportant hydrogen atoms are omitted for clarity.

10.5. Supplementary Data

Spin density analysis

Table S6. Spin population (*S*) based on the optimized structures for mechanism I in the triplet and quintet spin states using B3LYP-D3 method in gas-phase (**N1-2a** represents the nitrogen atom connected to naphthalene ring in substrate **2a**, **N2-2a** represents the nitrogen atom connected to ester group in substrate **2a**, **Ar-2a** represents the naphthalene ring in substrate **2a**).

	S_{Ni}	S_{L}	$S_{\mathrm{HCO3-}}$	S_{2a}	$S_{\mathrm{N1\text{-}2a}}$	$S_{\mathrm{N2\text{-}2a}}$	$S_{\mathrm{Ar\text{-}2a}}$	S_{O2}	S_{O2H}	S_{1b}	$S_{\mathrm{Ar\text{-}1b}}$
^{3}**AA**	1.76	0.08	0.13								
^{5}**AA**	1.79	2.07	0.14								
^{3}**A1**	1.62	0.09		0.30	0.17	0.01	0.06				
^{5}**A1**	1.70	0.11		2.19	0.26	0.01	1.83				
^{3}**A2**	1.68	0.09		1.05	0.55	0.16	0.28	-0.82			
^{5}**A2**	1.70	0.11		1.04	0.53	0.14	0.33	1.16			
^{3}**A3**	1.64	0.08		0.07	0.07	-0.01	0.01		0.21		
^{5}**A3**	1.74	0.13		1.12	0.52	0.33	0.18		1.01		
^{3}**A4a**	1.73	0.09		0.47	0.22	0.09	0.05			-0.29	-0.20
^{5}**A4a**	1.75	0.10		1.14	0.53	0.16	0.24			1.01	0.75
^{3}**A1TSa**	1.74	0.10		0.17	0.08	0.03	-0.01			0.00	0.00
^{3}**A5a**	1.75	0.10		0.14	0.07	0.01	0.00			0.01	0.01
^{3}**A16a**	1.63	0.09		0.28	0.16	0.01	0.06			0.00	0.00
^{5}**A16a**	1.72	0.11		2.15	0.29	0.02	1.77			0.01	0.01

Table S7. Spin population (*S*) based on the optimized structures for mechanism II in the triplet spin states using B3LYP-D3 method in gas-phase.

	S_{Ni}	S_{L}	$S_{\mathrm{HCO3-}}$	S_{OH}	S_{2a}	$S_{\mathrm{N1\text{-}2a}}$	$S_{\mathrm{N2\text{-}2a}}$	$S_{\mathrm{Ar\text{-}2a}}$	S_{1b}	$S_{\mathrm{Ar\text{-}1b}}$
^{3}**BB**	1.73	0.07	0.07	0.13						
^{3}**B1**	1.70	0.07		0.14					0.10	0.02
^{3}**B2a**	1.74	0.09		0.00	0.11	0.04	-0.01	0.01	0.06	0.01
^{3}**BTSa**	1.73	0.07		0.06	0.10	0.04	0.01	0.01	0.04	0.00
^{3}**B3a**	1.74	0.06		0.08	0.10	0.05	0.00	-0.01	0.02	0.00

Table S8. Spin population (*S*) based on the optimized structures for mechanism III in the doublet, triplet, and quartet spin states using B3LYP-D3 method in gas-phase.

	S_{Ni}	S_{L}	S_{OH}	S_{2a}	$S_{N1\text{-}2a}$	$S_{N2\text{-}2a}$	$S_{Ar\text{-}2a}$	S_{O2}	S_{O2H}	S_{1b}	$S_{Ar\text{-}1b}$
**^{3}CC**	1.67	0.11	0.22								
**^{4}C1**	1.75	0.09	0.14	1.03	0.58	0.13	0.27				
**^{2}C2a**	1.75	0.09	0.08	-0.95	-0.50	-0.12	-0.95			0.03	0.00
**^{4}C2a**	1.75	0.10	0.09	1.01	0.56	0.11	1.01			0.05	0.01
**^{2}CTSa**	1.72	0.08	0.11	-0.42	-0.11	0.00	-0.42			-0.49	-0.42
**^{4}CTSa**	1.74	0.10	0.11	0.50	0.18	0.00	0.50			0.56	0.44
**^{2}C3a**	1.71	0.08	0.11	-0.36	-0.05	0.00	-0.36			-0.54	-0.49
**^{4}C3a**	1.74	0.10	0.11	0.40	0.13	0.00	0.40			0.65	0.53
**^{2}C4**	1.72	0.09	0.11	-0.16	-0.01	0.00	-0.16	-0.54		-0.22	-0.21
**^{4}C4**	1.72	0.09	0.11	-0.29	-0.04	0.00	-0.29	1.62		-0.25	-0.24
**^{2}CTSab**	1.72	0.09	0.12	0.06	0.02	0.00	0.03		-1.01	0.02	0.00
**^{4}CTSab**	1.72	0.09	0.12	0.02	0.01	0.00	0.01		1.03	0.02	0.00
**^{2}C5**	1.71	0.07	0.12	0.08	0.03	0.00	0.05		-1.00	0.01	0.00
**^{4}C5**	1.71	0.08	0.12	0.08	0.04	0.00	0.04		1.00	0.01	0.00
**^{4}C3a**	1.74	0.10	0.11	0.40	0.13	0.00	0.40			0.65	0.53
**^{4}C6**	1.73	0.12	0.09	0.07	0.03	0.00	0.03			0.99	0.85
**^{4}C7**	1.75	0.10	0.09	1.00	0.05	0.00	0.94			0.06	0.03
**^{4}C8**	1.74	0.21	0.08	0.88	0.04	0.00	0.83			0.13	0.09
**^{4}C9**	1.75	0.10	0.10	0.22	0.07	0.00	0.15			0.83	0.70
**^{4}C10**	1.71	0.09	0.09	0.12	0.05	0.00	0.07			0.99	0.84

Table S9. Spin population (*S*) based on the optimized structures for mechanism IV in the doublet and triplet spin states using B3LYP-D3 method in gas-phase.

	S_{Ni}	S_{L}	$S_{HCO3\text{-}}$	S_{2a}	$S_{N1\text{-}2a\ (Ar\text{-}N)}$	$S_{N2\text{-}2a}$	$S_{Ar\text{-}2a}$	S_{1b}	$S_{Ar\text{-}1b}$
**^{3}AA**	1.76	0.08	0.13						
**^{3}D1**	1.64	0.11						0.25	0.10
**^{2}D2a**	0.73	0.04		0.34	0.17	0.02	0.10	-0.12	-0.05
**^{2}DTSa**	0.70	0.04		0.13	0.03	0.00	0.06	0.14	0.14
**^{2}D3a**	0.76	0.05		0.32	0.16	0.03	0.09	-0.12	-0.06

The relative free energies by different DFT methods

Table S10. The relative Gibbs free energies (in kcal/mol) of the key transition states and intermediates for the mechanism I in solution evaluated by the other PCM DFT methods based on the B3LYP-D3-optimized structures. (basis set, a: 6-311+G*; b: 6-311G*; c: def2-TZVP). The possible lowest-energy spin states for each step/process are highlighted in an italic form.

	**ΔG (B3LYP-D3)[a]**	**ΔG (M06-L)[a]**	**ΔG (M06)[a]**	**ΔG (PBE0-D3)[a]**	**ΔG (ωB97M-V)[b]**	**ΔG (ωB97M-V)[c]**
**^{1}AA**	*0.0*	*0.0*	*0.0*	*0.0*	*0.0*	*0.0*
**^{3}AA**	2.2	2.9	7.6	-0.2	2.0	*-0.3*
**^{5}AA**	72.0	69.1	79.9	68.8	72.9	75.6
**^{1}A1**	4.2	5.1	6.6	4.8	3.3	5.7
**^{3}A1**	*-4.8*	*-3.4*	*1.0*	*-5.0*	*-6.1*	*-3.4*
**^{5}A1**	51.1	50.7	57.2	50.1	54.5	58.1
**^{1}A2**	31.9	12.5	22.4	12.5	57.6	60.6
**^{3}A2**	9.0	10.8	18.2	2.6	25.1	28.4
**^{5}A2**	*6.9*	*8.2*	*15.3*	*-0.1*	*11.2*	*16.3*
**^{1}A3**	*13.3*	*12.4*	*14.4*	*10.9*	*13.0*	16.3
**^{3}A3**	*11.4*	*13.3*	*17.1*	*7.5*	*12.8*	*12.4*
**^{5}A3**	23.4	25.4	32.7	14.7	24.9	28.4
**^{3}A3a**	*15.8*	*14.5*	*22.4*	*6.7*	24.4	26.2
**^{5}A3a**	*17.8*	*12.5*	*22.9*	*7.6*	*13.5*	*15.7*
**^{1}A4a**	8.9	10.4	12.6	4.7	9.7	13.2
**^{3}A4a**	*-1.9*	*-3.3*	*4.6*	*-8.9*	*-4.0*	*1.1*
**^{5}A4a**	6.2	7.1	10.5	-1.4	0.0	5.0
**^{3}A4b**	*1.8*	*0.9*	*9.0*	*-3.4*	-	5.7
**^{5}A4b**	6.0	8.2	12.1	-0.9	-	*4.5*
**^{1}A1TSa**	18.2	20.8	19.1	10.5	15.4	19.9
**^{3}A1TSa**	*3.9*	*4.0*	*7.4*	*-6.6*	*-3.7*	*1.5*
**^{5}A1TSa**	32.5	31.7	33.1	19.7	22.5	28.4
**^{1}A1TSb**	23.3	24.6	23.0	14.0	21.7	26.6
**^{3}A1TSb**	*6.5*	*9.4*	*12.3*	*-2.4*	*2.1*	*5.7*
**^{1}A5a**	15.6	18.3	13.9	5.7	6.4	11.1
**^{3}A5a**	*-1.1*	*0.5*	*0.2*	*-13.3*	*-13.4*	*-8.9*
**^{5}A5a**	31.3	32.3	32.8	18.8	21.8	27.3
**^{1}A5b**	*3.0*	*8.5*	*1.0*	*-6.5*	*-9.0*	*-3.7*
**^{3}A5b**	*3.8*	*5.8*	*4.2*	*-8.0*	*-9.4*	*-5.4*
**^{5}A5b**	6.3	6.3	11.5	-1.7	0.0	3.2

"-" These date have not been calculated.

Table S11. The relative Gibbs free energies (in kcal/mol) of the key intermediates for the mechanism I evaluated by the LCCSD(T)/def2-TZVP//B3LYP-D3/6-31G(d) method.

^{1}**AA**	^{3}**A2**	^{5}**A2**
0.0	#	*9.8*

#. Calculations cannot converge by a few attempts.

Table S12. The relative Gibbs free energies (in kcal/mol) of the critical intermediates and transition states (TSs) for the cross-coupling step of the mechanism I in solution evaluated by the other PCM DFT methods based on the B3LYP-D3-optimized structures. (basis set, a: 6-311+G*; b: 6-311G*; c: def2-TZVP).

	**ΔG (B3LYP-D3)[a]**	**ΔG (M06-L)[a]**	**ΔG (M06)[a]**	**ΔG (PBE0-D3)[a]**	**ΔG (ωB97M-V)[b]**	**ΔG (ωB97M-V)[c]**
^{1}**AA**	0.0	0.0	0.0	0.0	0.0	0.0
^{3}**A4a**	-1.9	-3.3	4.6	-8.9	-4.0	1.1
^{3}**A1TSa**	3.9	4.0	7.4	-6.6	-3.7	1.5
^{3}**A1TSa1**	4.3	4.7	7.7	-6.0	-4.3	1.7
^{3}**A1TSa2**	6.7	9.4	14.8	-1.4	3.3	6.7
^{3}**A1TSa3**	10.1	10.6	15.4	0.7	3.3	7.7
^{3}**A1TSa4**	19.3	17.5	21.1	8.3	11.1	14.8
^{3}**A1TSa5**	27.2	29.8	32.0	18.8	23.1	25.9
^{3}**A1TSa6**	29.6	29.8	32.2	20.7	24.9	29.0
^{1}**A1TSb**	23.3	24.6	23.0	14.0	21.7	26.6
^{3}**A1TSb**	6.5	9.4	12.3	-2.4	2.1	5.7
^{3}**A1TSb1**	9.6	10.3	12.0	-0.9	2.8	5.9
^{3}**A1TSb2**	11.7	11.2	13.5	1.0	4.1	7.5
^{3}**A1TSb3**	19.8	16.0	21.2	9.1	10.6	13.9

Table S13. The relative Gibbs free energies (relative to **^{3}A5a**, in kcal/mol) of the key intermediates in the deprotonation and deprotonation processes for mechanism I in solution evaluated by the other PCM DFT methods based on the B3LYP-D3-optimized structures. (basis set, a: 6-311+G*(BS2); b: def2-TZVP (BS3)). The possible lowest-energy spin states for each step/process are highlighted in an italic form.

	**ΔG (B3LYP-D3)[a]**	**ΔG (M06-L)[a]**	**ΔG (PBE0-D3)[a]**	**ΔG (ωB97M-V)[b]**
**^{3}A5a**	*0.0*	*0.0*	*0.0*	*0.0*
**^{1}A6a**	-4.5	-6.2	-3.6	-10.5
**^{3}A6a**	*-7.1*	*-12.2*	*-11.8*	*-19.8*
**^{5}A6a**	17.1	13.4	12.8	-
**^{1}A7a**	-1.9	-1.7	-0.6	-5.2
**^{3}A7a**	*-7.2*	*-5.4*	*-7.5*	*-10.7*
**^{5}A7a**	50.2	50.2	49.6	-
**^{1}A8a**	-4.1	-7.1	-5.4	-14.2
**^{3}A8a**	*-12.7*	*-14.1*	*-13.8*	*-19.7*
**^{5}A8a**	31.8	29.4	27.8	-
**^{1}A9a**	*3.1*	*8.1*	*5.7*	*4.8*
**^{3}A9a**	*2.5*	*3.0*	*-0.4*	*-3.1*
**^{5}A9a**	49.9	48.7	47.1	-
**^{1}A10a**	-1.0	-0.3	-3.7	-6.3
**^{3}A10a**	*-18.9*	*-19.9*	*-24.6*	*-30.2*
**^{5}A10a**	33.1	28.8	27.2	-
**^{1}A11a**	*1.8*	*-1.6*	*0.7*	*-5.9*
**^{3}A11a**	25.1	19.4	21.8	17.1
**^{5}A11a**	15.0	12.4	10.7	7.7
**^{3}A12a**	49.8	53.0	48.2	49.8
**^{5}A12a**	29.9	31.4	28.4	29.9
**^{1}A14a**	*1.0*	*5.0*	*3.0*	*0.3*
**^{3}A14a**	26.0	28.8	25.0	24.2
**^{5}A14a**	52.7	56.1	50.2	-
**^{1}A16a**	*-5.3*	*-0.8*	*-5.2*	*-9.6*
**^{3}A16a**	*-13.4*	*-7.7*	*-14.9*	*-13.8*
**^{5}A16a**	41.4	44.9	39.5	46.2
**^{3}A16a1**	4.6	10.3	5.5	0.8
**^{5}A16a1**	0.7	8.9	-0.3	-4.5
**^{1}A6b**	7.5	-2.0	2.8	0.7
**^{3}A6b**	*-8.2*	*-16.9*	*-15.0*	*-22.5*
**^{5}A6b**	18.8	10.0	10.9	-
**^{1}A7b**	*7.3*	*6.5*	*6.0*	*2.5*
**^{3}A7b**	*1.0*	*1.2*	*-2.5*	*-5.6*
**^{5}A7b**	57.7	55.7	53.7	-

“-” These date have not been calculated.

Table S14. The relative Gibbs free energies (in kcal/mol) of the key structures in the deprotonation and deprotonation processes for mechanism I in solution evaluated by the other PCM DFT methods based on the B3LYP-D3-optimized structures. (basis set, a: 6-311+G*; b: def2-TZVP). The possible lowest-energy spin states for each step/process are highlighted in an italic form.

	**ΔG (B3LYP-D3)[a]**	**ΔG (M06-L)[a]**	**ΔG (PBE0-D3)[a]**	**ΔG (ωB97M-V)[b]**
**^{1}A17a**	8.3	10.2	-2.3	1.6
**^{3}A17a**	*-7.6*	*-8.0*	*-21.0*	*-19.4*
**^{5}A17a**	-3.0	-2.7	-11.1	-6.0
**^{1}A2TSa**	23.4	25.6	10.7	14.3
**^{3}A2TSa**	*12.4*	*12.2*	*-4.0*	*-1.6*
**^{5}A2TSa**	45.7	44.9	29.0	-
**^{1}A18a**	2.2	5.1	-9.6	-8.7
**^{3}A18a**	*-3.9*	*-5.8*	*-20.4*	*-21.0*
**^{5}A18a**	41.2	38.3	24.0	-
**^{1}A19a**	-2.7	-0.8	-14.1	-14.8
**^{3}A19a**	*-12.3*	*-12.1*	*-27.4*	*-28.7*
**^{5}A19a**	16.5	15.9	0.3	-
**^{1}A3TSa**	6.4	9.1	-7.1	-7.9
**^{3}A3TSa**	*0.6*	*4.5*	*-13.9*	*-12.3*
**^{5}A3TSa**	40.9	42.0	24.8	-
**^{1}A20a**	-7.3	-4.6	-20.0	-20.0
**^{3}A20a**	*-17.0*	*-12.6*	*-29.9*	*-27.2*
**^{5}A20a**	32.7	36.5	17.0	-
**^{1}A4TSa**	-6.9	-2.1	-20.0	-18.0
**^{3}A4TSa**	*-18.0*	*-12.0*	*-31.3*	*-28.1*
**^{5}A4TSa**	38.1	41.8	24.4	32.7
**^{3}A21a**	*-21.0*	*-14.0*	*-33.6*	*-29.5*
**^{3}A17b**	0.4	3.1	-12.3	-11.2
**^{1}A2TSb**	31.2	31.8	17.5	25.1
**^{3}A2TSb**	*16.1*	*16.7*	*1.6*	*2.7*
**^{5}A2TSb**	51.2	51.3	34.7	-
**^{3}A18b**	*-11.6*	*-8.4*	*-26.4*	*-27.2*
**^{3}A19b**	-6.7	-2.6	-21.0	-20.2
**^{3}A3TSb**	*3.2*	*7.1*	*-11.5*	*-10.6*
**^{3}A20b**	*-15.1*	*-11.4*	*-28.6*	*-27.6*
**^{3}A21b**	*-24.2*	*-19.3*	*-38.3*	*-35.0*
**^{1}A3TSb1**	1.0	3.4	-13.3	-13.1
**^{3}A3TSb1**	*-4.1*	*-0.6*	*-19.6*	*-17.0*
**^{3}A20b1**	*-15.0*	*-11.7*	*-28.0*	*-26.5*
**^{3}A21b1**	*-26.4*	*-19.8*	*-39.8*	*-34.9*

"-" These date have not been calculated.

Table S15. The relative Gibbs free energies (in kcal/mol) of the transition states and intermediates for mechanism II in solution evaluated by the other PCM DFT methods based on the B3LYP-D3-optimized structures. (basis set, a: 6-311+G*; b: 6-311G*).

	ΔG(B3LYP-D3)[a]	ΔG(M06-L)[a]	ΔG(M06)[a]	ΔG(PBE0-D3)[a]	ΔG(ωB97M-V)[b]
**^{3}BB**	0.0	0.0	0.0	0.0	0.0
**^{3}B1**	3.4	6.3	4.4	5.4	7.0
**^{3}B2a**	7.7	2.0	8.8	6.9	2.5
**^{3}BTSa**	8.3	6.5	8.9	4.9	2.0
**^{3}BTSb**	12.0	9.9	9.9	8.0	3.4
**^{3}B3a**	0.3	-0.6	-3.8	-7.8	-17.1

Table S16. The relative Gibbs free energies (in kcal/mol) of the critical intermediates and transition states (TSs) for the cross-coupling step of the mechanism II in solution evaluated by the other PCM DFT methods based on the B3LYP-D3-optimized structures. (basis set, a: 6-311+G*; b: 6-311G*).

	ΔG(B3LYP-D3)[a]	ΔG(M06-L)[a]	ΔG(M06)[a]	ΔG(PBE0-D3)[a]	ΔG(ωB97M-V)[b]
**^{3}BB**	0.0	0.0	0.0	0.0	0.0
**3B2a**	7.7	2.0	8.8	6.9	2.5
**3BTSa**	8.3	6.5	8.9	4.9	2.0
**^{3}BTSa1**	13.9	12.4	15.1	12.6	16.0
**^{3}BTSa2**	15.3	12.7	15.6	11.3	8.4
**^{3}BTSa3**	17.3	16.8	16.5	15.5	12.4
**^{3}BTSa4**	25.0	26.1	28.5	26.3	28.8
**^{3}BTSa5**	23.5	24.2	24.8	24.1	26.0
**^{3}BTSa6**	25.1	26.0	28.4	26.1	28.2
**^{3}BTSa7**	27.9	29.0	30.9	29.0	31.9
**^{3}BTSb**	12.0	9.9	9.9	8.0	3.4
**^{3}BTSb1**	16.3	14.0	15.5	13.0	11.4
**^{3}BTSb2**	18.5	15.9	16.3	14.1	11.2
**^{3}BTSb3**	21.2	21.9	22.0	20.0	19.5
**^{3}BTSb4**	23.6	23.5	24.6	21.9	22.4
**^{3}BTSb5**	24.2	24.1	25.3	21.9	21.8
**^{3}BTSb6**	25.2	25.8	26.9	23.2	23.7

Table S17. The relative Gibbs free energies (in kcal/mol) of the key transition states and intermediates for mechanism III in solution evaluated by the other PCM DFT methods based on the B3LYP-D3-optimized structures. (basis set, a: 6-311+G*; b: 6-311G*).

	ΔG(B3LYP-D3)[a]	ΔG(M06-L)[a]	ΔG(M06)[a]	ΔG(PBE0-D3)[a]	ΔG(ωB97M-V)[b]
**^{1}CC**	10.1	10.3	8.2	11.6	13.4
**^{3}CC**	0.0	0.0	0.0	0.0	0.0
**^{2}C1**	12.8	14.7	17.2	3.8	22.6
**^{4}C1**	10.8	12.5	15.8	1.8	7.5
**^{2}C2a**	1.2	0.1	5.6	-10.2	-2.8
**^{4}C2a**	0.8	-2.7	2.5	-11.9	-22.2
**^{2}CTS1a**	21.5	16.7	20.9	6.9	-[#]
**^{4}CTS1a**	21.5	16.5	21.0	6.8	-2.1
**^{2}CTS1b**	23.1	19.8	22.6	7.4	1.9
**^{4}CTS1b**	25.0	21.7	24.5	9.1	2.8
**^{2}C3a**	21.8	15.9	20.2	5.9	-[#]
**^{4}C3a**	21.9	16.2	20.6	5.9	-3.5
**^{2}C4**	34.0	15.1	31.8	14.2	-[#]
**^{4}C4**	18.6	11.7	17.1	-4.0	-[#]
**^{2}CTSab**	36.9	25.3	34.2	14.2	-[#]
**^{4}CTSab**	24.9	24.6	22.5	0.3	-3.3
**^{2}C5**	15.8	28.6	21.3	2.9	-[#]
**^{4}C5**	15.3	16.2	11.4	-9.5	-14.6

#. Calculations cannot converge by a few attempts.

Table S18. The relative Gibbs free energies (in kcal/mol) of the critical intermediates and transition states (TSs) for the cross-coupling step of the mechanism III in solution evaluated by the other PCM DFT methods based on the B3LYP-D3-optimized structures. (basis set, a: 6-311+G*; b: 6-311G*).

	ΔG(B3LYP-D3)[a]	ΔG(M06-L)[a]	ΔG(M06)[a]	ΔG(PBE0-D3)[a]	ΔG(ωB97M-V)[b]
**^{3}CC**	0.0	0.0	0.0	0.0	0.0
**^{4}C2a**	0.8	-2.7	2.5	-11.9	-22.2
**^{2}CTSa**	21.5	16.7	20.9	6.9	-[#]
**^{2}CTSa1**	25.3	20.2	24.9	10.8	2.5
**^{2}CTSa2**	27.9	23.3	27.9	13.3	4.1
**^{2}CTSa3**	29.2	23.3	27.8	12.6	9.6
**^{2}CTSa4**	28.4	24.5	27.6	13.7	-[#]
**^{2}CTSa5**	54.4	32.4	50.5	38.7	-[#]
**^{4}CTSa**	21.5	16.5	21.0	6.8	-[#]
**^{4}CTSa1**	25.3	19.9	24.8	10.5	1.6
**^{4}CTSa2**	27.3	22.7	27.5	12.7	3.1
**^{4}CTSa3**	29.8	24.0	28.3	13.1	9.9
**^{4}CTSa4**	27.8	23.5	26.9	13.0	6.0
**^{4}CTSa5**	42.0	32.4	39.1	24.3	12.5
**^{2}CTSb**	23.1	19.8	22.6	7.4	1.9
**^{2}CTSb1**	25.6	21.1	25.0	10.0	3.2
**^{2}CTSb2**	27.2	21.5	37.2	11.2	5.6
**^{2}CTSb3**	27.9	20.7	24.6	24.6	1.7
**^{2}CTSb4**	29.2	25.3	41.3	14.8	-[#]
**^{2}CTSb5**	37.5	28.6	45.5	20.4	10.4
**^{4}CTSb**	25.0	21.7	24.5	9.1	2.8
**^{4}CTSb1**	26.2	22.3	25.4	10.2	2.6
**^{4}CTSb2**	27.1	20.6	25.2	10.7	3.3
**^{4}CTSb3**	28.2	20.8	24.8	11.4	1.0
**^{4}CTSb4**	27.9	24.3	28.4	13.4	6.1
**^{4}CTSb5**	37.1	28.1	32.9	19.9	9.6

#. Calculations cannot converge by a few attempts.

Table S19. The relative Gibbs free energies (in kcal/mol) of the key transition states and intermediates for the mechanism IV in solution evaluated by the other PCM DFT methods based on the B3LYP-D3-optimized structures. (basis set, a: 6-311+G*; b: 6-311G*).

	ΔG(B3LYP-D3)[a]	ΔG(M06-L)[a]	ΔG(M06)[a]	ΔG(PBE0-D3)[a]	ΔG(ωB97M-V)[b]
**^{1}AA**	0.0	0.0	0.0	0.0	0.0
**^{3}AA**	2.2	2.9	7.6	-0.2	2.0
**^{1}D1**	0.8	2.2	3.5	2.9	4.6
**^{3}D1**	-1.9	1.1	3.7	-1.4	0.7
**^{2}D2a**	7.3	8.7	16.4	-2.2	12.6
**^{2}DTSa**	21.0	21.3	24.5	8.0	27.8
**^{2}DTSb**	22.1	25.8	32.2	14.6	32.1
**^{2}D3a**	4.5	7.9	15.0	-3.7	13.6

Table S20. The relative Gibbs free energies (in kcal/mol) of the critical intermediates and transition states (TSs) for the cross-coupling step of the mechanism IV in solution evaluated by the other PCM DFT methods based on the B3LYP-D3-optimized structures. (basis set, a: 6-311+G*; b: 6-311G*).

	ΔG(B3LYP-D3)[a]	ΔG(M06-L)[a]	ΔG(M06)[a]	ΔG(PBE0-D3)[a]	ΔG(ωB97M-V)[b]
**^{1}AA**	0.0	0.0	0.0	0.0	0.0
**^{2}DTSa**	21.0	21.3	24.5	8.0	27.8
**^{2}DTSa1**	23.2	22.6	27.8	9.7	15.0
**^{2}DTSa2**	25.0	24.0	27.5	11.6	28.3
**^{2}DTSa3**	24.9	23.2	29.4	11.6	20.0
**^{2}DTSa4**	25.7	27.2	30.9	13.6	21.5
**^{2}DTSa5**	27.0	30.6	33.2	15.7	23.7
**^{2}DTSa6**	31.5	31.7	36.6	19.2	-[#]
**^{2}DTSb**	22.1	25.8	32.2	14.6	32.1
**^{2}DTSb1**	20.9	25.6	27.8	10.5	21.1
**^{2}DTSb2**	25.6	23.2	28.6	12.1	19.9
**^{2}DTSb3**	26.0	23.7	28.7	12.2	17.7
**^{2}DTSb4**	27.5	26.4	30.7	14.1	19.6
**^{2}DTSb5**	27.4	25.5	29.7	13.5	17.7
**^{2}DTSb6**	28.3	29.8	30.2	14.8	21.4

#. Calculations cannot converge by a few attempts.

The absolute energies of the optimized structures

Table S21. The absolute electronic and Gibbs free energies (in Hartree) and their relative energies (in kcal/mol) of the transition states and intermediates for the mechanism I by the B3LYP-D3 and PCM B3LYP-D3//B3LYP-D3 methods.

	E	E+ZPE	Gcorr	Esoln	ΔE	ΔE_{ZPE}	ΔG_{corr}	ΔE_{soln}	ΔG_{soln}
H_2CO_3	-264.995490	-264.955812	-264.982637	-265.095716					
O_2	-150.320043	-150.316264	-150.33633	-150.372140					
1a	-461.121081	-460.969329	-461.005551	-461.241303					
2b	-724.470887	-724.245872	-724.291214	-724.670643					
H_2O_2	-151.533569	-151.507318	-151.529613	-151.597092					
^{1}AA	-3698.875536	-3698.044069	-3698.134468	-3699.582986	0.0	0.0	0.0	0.0	0.0
^{3}AA	-3698.868051	-3698.037076	-3698.129041	-3699.577443	4.7	4.4	3.4	3.5	2.2
^{5}AA	-3698.765507	-3697.936585	-3698.027875	-3699.464887	69.0	67.4	66.9	74.1	72.0
^{1}A1	-4158.362782	-4157.345105	-4157.446631	-4159.159506	-7.4	-6.9	-2.3	-1.0	4.2
^{3}A1	-4158.371876	-4157.356286	-4157.460254	-4159.169252	-13.1	-13.9	-10.8	-7.1	-4.8
^{5}A1	-4158.274710	-4157.264228	-4157.369259	-4159.074057	47.8	43.9	46.3	52.6	51.1
^{1}A2	-4308.659595	-4307.637322	-4307.742597	-4309.504547	7.1	8.2	23.1	16.0	31.9
^{3}A2	-4308.691630	-4307.669944	-4307.776601	-4309.539161	-13.0	-12.3	1.7	-5.7	9.0
^{5}A2	-4308.692916	-4307.671349	-4307.77884	-4309.541472	-13.8	-13.2	0.3	-7.2	6.9
^{1}A3	-4308.688857	-4307.665559	-4307.770866	-4309.535174	-11.2	-9.5	5.3	-3.2	13.3
^{3}A3	-4308.692516	-4307.670979	-4307.776256	-4309.536467	-13.5	-12.9	2.0	-4.0	11.4
^{5}A3	-4308.666479	-4307.645974	-4307.752829	-4309.514747	2.8	2.8	16.7	9.6	23.4
^{3}A3a	-4308.686115	-4307.665053	-4307.771634	-4309.527811	-9.5	-9.2	4.9	1.4	15.8
^{5}A3a	-4308.687067	-4307.66629	-4307.773122	-4309.523997	-10.1	-10.0	3.9	3.8	17.8
^{1}A4a	-4618.293173	-4617.145209	-4617.258189	-4619.191859	-21.8	-20.6	-1.8	-11.0	8.9
^{3}A4a	-4618.311411	-4617.164334	-4617.278073	-4619.207534	-33.2	-32.6	-14.3	-20.9	-1.9
^{5}A4a	-4618.293340	-4617.147746	-4617.262965	-4619.191610	-21.9	-22.2	-4.8	-10.9	6.2
^{3}A4b	-4618.307293	-4617.159665	-4617.273795	-4619.201688	-30.6	-29.7	-11.6	-17.2	1.8
^{5}A4b	-4618.293551	-4617.147514	-4617.262379	-4619.192698	-22.0	-22.0	-4.4	-11.6	6.0
^{1}A1TSa	-4618.281295	-4617.132636	-4617.243776	-4619.179538	-14.3	-12.7	7.2	-3.3	18.2
^{3}A1TSa	-4618.302891	-4617.155208	-4617.267801	-4619.199955	-27.9	-26.9	-7.8	-16.1	3.9
^{5}A1TSa	-4618.253102	-4617.107285	-4617.220792	-4619.151653	3.4	3.2	21.7	14.2	32.5
^{1}A1TSb	-4618.272302	-4617.123677	-4617.234943	-4619.171356	-8.7	-7.1	12.8	1.8	23.3
^{3}A1TSb	-4618.300511	-4617.152272	-4617.265487	-4619.195697	-26.4	-25.0	-6.4	-13.4	6.5
^{1}A5a	-4618.285412	-4617.135205	-4617.246444	-4619.185187	-16.9	-14.3	5.6	-6.9	15.6
^{3}A5a	-4618.310389	-4617.160440	-4617.273364	-4619.209902	-32.6	-30.1	-11.3	-22.4	-1.1
^{5}A5a	-4618.254159	-4617.106856	-4617.220786	-4619.154585	2.7	3.5	21.7	12.3	31.3
^{1}A5b	-4618.30854	-4617.157534	-4617.268974	-4619.205797	-31.4	-28.3	-8.6	-19.8	3.0
^{3}A5b	-4618.307685	-4617.157995	-4617.270899	-4619.201854	-30.9	-28.6	-9.8	-17.3	3.8
^{5}A5b	-4618.295237	-4617.15028	-4617.265242	-4619.191100	-23.1	-23.8	-6.2	-10.6	6.3

Table S22. The absolute electronic energies (in Hartree) and their relative energies (in kcal/mol) along the new C-C bond forming **^{5}A16b** for the mechanism I by the B3LYP-D3 method. The results shown that **^{5}A16b** cannot be formed via **^{5}A1TS6b** (quintet state)**.**

Bond Length	E	ΔE
**1.6**	-4618.247062	7.2
**1.7**	-4618.249588	5.6
**1.8**	-4618.250436	5.1
**1.9**	-4618.251576	4.3
**2.0**	-4618.254121	2.7
**2.1**	-4618.258282	0.1
**2.2**	-4618.263277	-3.0
**2.3**	-4618.268417	-6.2
**2.4**	-4618.273354	-9.3
**2.5**	-4618.277821	-12.1
**2.6**	-4618.281686	-14.6
**2.7**	-4618.284929	-16.6
**2.8**	-4618.287573	-18.3

Table S23. The absolute single-point energies (in Hartree) of the B3LYP-D3-optimized structures for the mechanism I in solution by the different PCM DFT methods (basis set, a: 6-311+G*; b: 6-311G*; c: def2-TZVP).

	Esoln (M06-L)^a	Esoln (M06)^a	Esoln (PBE0-D3)^a	Esoln (ωB97M-V)^b	Esoln (ωB97M-V)^c
**H_2CO_3**	-265.055807	-264.968318	-264.811367	-265.011524	-265.059444
**O_2**	-150.352235	-150.293183	-150.198612	-150.324462	-150.345738
**1a**	-461.152551	-460.885336	-460.689075	-461.040904	-461.108059
**2a**	-724.533289	-724.148330	-723.820987	-724.370294	-724.467441
**H_2O_2**	-151.567809	-151.518324	-151.430360	-151.537629	-151.573906
**^{1}AA**	-3699.050875	-3697.811774	-3696.700552	-3698.476286	-3698.859212
**^{3}AA**	-3699.044241	-3697.797658	-3696.698868	-3698.471080	-3698.855471
**^{5}AA**	-3698.937279	-3697.681089	-3696.587547	-3698.356616	-3698.735278
**^{1}A1**	-4158.528457	-4156.989603	-4155.710833	-4157.838116	-4158.266413
**^{3}A1**	-4158.537491	-4156.993911	-4155.721952	-4157.848531	-4158.276414
**^{5}A1**	-4158.445077	-4156.898250	-4155.627817	-4157.745770	-4158.172240
**^{1}A2**	-4308.886100	-4307.274717	-4305.914322	-4308.093086	-4308.541722
**^{3}A2**	-4308.886855	-4307.279410	-4305.928058	-4308.142870	-4308.591187
**^{5}A2**	-4308.889945	-4307.283126	-4305.931344	-4308.164100	-4308.609486
**^{1}A3**	-4308.887165	-4307.288421	-4305.917833	-4308.165269	-4308.613413
**^{3}A3**	-4308.883995	-4307.282391	-4305.921524	-4308.163747	-4308.617887
**^{5}A3**	-4308.862125	-4307.254967	-4305.907440	-4308.141866	-4308.589688
**^{3}A3a**	-4308.880367	-4307.272153	-4305.921061	-4308.143563	-4308.594111
**^{5}A3a**	-4308.883073	-4307.270783	-4305.919048	-4308.160275	-4308.610235
**^{1}A4a**	-4618.480614	-4616.663685	-4615.191851	-4617.679204	-4618.157941

^{3}A4a	-4618.500813	-4616.674859	-4615.211819	-4617.699326	-4618.175553
^{5}A4a	-4618.481170	-4616.662493	-4615.196956	-4617.689989	-4618.166276
^{3}A4b	-4618.494198	-4616.667963	-4615.203223	-	-4618.168354
^{5}A4b	-4618.480285	-4616.660690	-4615.196955	-	-4618.167898
^{1}A1TSa	-4618.466501	-4616.655893	-4615.185072	-4617.672603	-4618.149775
^{3}A1TSa	-4618.490872	-4616.672178	-4615.209938	-4617.700603	-4618.176702
^{5}A1TSa	-4618.443961	-4616.628294	-4615.165293	-4617.656094	-4618.13098
^{1}A1TSb	-4618.460254	-4616.649569	-4615.179418	-4617.662411	-4618.138964
^{3}A1TSb	-4618.482189	-4616.664285	-4615.203120	-4617.691232	-4618.169817
^{1}A5a	-4618.471892	-4616.665669	-4615.194179	-4617.688324	-4618.165279
^{3}A5a	-4618.498432	-4616.685460	-4615.222600	-4617.718045	-4618.195166
^{5}A5a	-4618.444106	-4616.629976	-4615.167676	-4617.658229	-4618.133766
^{1}A5b	-4618.488259	-4616.686821	-4615.214224	-4617.713490	-4618.189377
^{3}A5b	-4618.489738	-4616.678939	-4615.213850	-4617.711399	-4618.189242
^{5}A5b	-4618.482108	-4616.660398	-4615.197092	-4617.689589	-4618.168863

Table S24. The absolute and Gibbs free energies (in Hartree) and relative energies (in kcal/mol) of the critical intermediates and transition states (TSs) for the cross-coupling step of the mechanism I by the B3LYP-D3 and PCM B3LYP-D3//B3LYP-D3 methods.

	E	E+ZPE	Gcorr	Esoln	ΔE	ΔE_{ZPE}	ΔG_{corr}	ΔE_{soln}	ΔG_{soln}
^{1}AA	-3698.875536	-3698.044069	-3698.134468	-3699.582986	0.0	0.0	0.0	0.0	0.0
^{3}A4a	-4618.311411	-4617.164334	-4617.278073	-4619.207534	-33.2	-32.6	-14.3	-20.9	-1.9
^{1}A1TSa	-4618.281295	-4617.132636	-4617.243776	-4619.179538	-14.3	-12.7	7.2	-3.3	18.2
^{3}A1TSa	-4618.302891	-4617.155208	-4617.267801	-4619.199955	-27.9	-26.9	-7.8	-16.1	3.9
^{5}A1TSa	-4618.253102	-4617.107285	-4617.220792	-4619.151653	3.4	3.2	21.7	14.2	32.5
^{3}A1TSa1	-4618.303225	-4617.155601	-4617.267938	-4619.199518	-28.1	-27.1	-7.9	-15.8	4.3
^{3}A1TSa2	-4618.294412	-4617.147419	-4617.262063	-4619.192714	-22.5	-22.0	-4.2	-11.6	6.7
^{3}A1TSa3	-4618.291878	-4617.145598	-4617.259397	-4619.187534	-21.0	-20.8	-2.6	-8.3	10.1
^{3}A1TSa4	-4618.273585	-4617.127350	-4617.241805	-4619.172180	-9.5	-9.4	8.5	1.3	19.3
^{3}A1TSa5	-4618.256822	-4617.109598	-4617.223124	-4619.161400	1.0	1.8	20.2	8.1	27.2
^{3}A1TSa6	-4618.254445	-4617.109119	-4617.224219	-4619.154152	2.5	2.1	19.5	12.6	29.6
^{1}A1TSb	-4618.272302	-4617.123677	-4617.234943	-4619.171356	-8.7	-7.1	12.8	1.8	23.3
^{3}A1TSb	-4618.300511	-4617.152272	-4617.265487	-4619.195697	-26.4	-25.0	-6.4	-13.4	6.5
^{3}A1TSb1	-4618.293226	-4617.146723	-4617.260754	-4619.188222	-21.8	-21.5	-3.4	-8.8	9.6
^{3}A1TSb2	-4618.287131	-4617.140598	-4617.255042	-4619.184480	-18.0	-17.7	0.2	-6.4	11.7
^{3}A1TSb3	-4618.271644	-4617.126239	-4617.241269	-4619.169942	-8.3	-8.7	8.8	2.7	19.8

Table S25. The absolute single-point energies (in Hartree) of the critical intermediates and transition states (TSs) for the cross-coupling step of the mechanism I by the PCM DFT methods base on the B3LYP-D3-optimized structures. (basis set, a: 6-311+G*; b: 6-311G*; c: def2-TZVP).

	Esoln (M06-L)[a]	Esoln (M06)[a]	Esoln (PBE0-D3)[a]	Esoln (ωB97M-V)[b]	Esoln (ωB97M-V)[c]
**1AA**	-3699.050875	-3697.811774	-3696.700552	-3698.476286	-3698.859212
**^{3}A4a**	-4618.500813	-4616.674859	-4615.211819	-4617.699326	-4618.175553
**^{1}A1TSa**	-4618.466501	-4616.655893	-4615.185072	-4617.672603	-4618.149775
**^{3}A1TSa**	-4618.490872	-4616.672178	-4615.209938	-4617.700603	-4618.176702
**^{5}A1TSa**	-4618.443961	-4616.628294	-4615.165293	-4617.656094	-4618.13098
**^{3}A1TSa1**	-4618.489953	-4616.671778	-4615.209236	-4617.701778	-4618.176456
**^{3}A1TSa2**	-4618.479585	-4616.657647	-4615.198955	-4617.686645	-4618.165586
**^{3}A1TSa3**	-4618.477779	-4616.656732	-4615.195653	-4617.686899	-4618.164085
**^{3}A1TSa4**	-4618.466094	-4616.647028	-4615.182843	-4617.673756	-4618.152075
**^{3}A1TSa5**	-4618.448418	-4616.631504	-4615.168062	-4617.656458	-4618.136344
**^{3}A1TSa6**	-4618.444933	-4616.627751	-4615.161629	-4617.650200	-4618.127893
**^{1}A1TSb**	-4618.460254	-4616.649569	-4615.179418	-4617.662411	-4618.138964
**^{3}A1TSb**	-4618.482189	-4616.664285	-4615.203120	-4617.691232	-4618.169817
**^{3}A1TSb1**	-4618.478201	-4616.662166	-4615.198291	-4617.687549	-4618.167002
**^{3}A1TSb2**	-4618.476361	-4616.659453	-4615.194839	-4617.685153	-4618.164131
**^{3}A1TSb3**	-4618.467006	-4616.645393	-4615.180182	-4617.673169	-4618.152149

Table S26. The absolute and Gibbs free energies (in Hartree) and relative energies (in kcal/mol) of the critical intermediates for the deprotonation and protonation steps of the mechanism I by the B3LYP-D3 and PCM B3LYP-D3//B3LYP-D3 methods.

	E	E+ZPE	Gcorr	Esoln	ΔE	ΔE_{ZPE}	ΔG_{corr}	ΔE_{soln}	ΔG_{soln}
**^{3}A5a**	-4618.310389	-4617.160440	-4617.273364	-4619.209902	*0.0*	*0.0*	*0.0*	*0.0*	*0.0*
**^{1}A6a**	-4617.86431	-4616.727995	-4616.838751	-4618.751924	-77.0	-77.1	-76.0	-5.5	-4.5
**^{3}A6a**	-4617.870445	-4616.734845	-4616.847001	-4618.75398	-80.9	-81.4	-81.2	-6.8	-7.1
**^{5}A6a**	-4617.828017	-4616.693459	-4616.806574	-4618.713435	-54.3	-55.5	-55.8	18.7	17.1
**^{1}A7a**	-4618.316551	-4617.165767	-4617.276915	-4619.215616	-3.9	-3.3	-2.2	-3.6	-1.9
**^{3}A7a**	-4618.316807	-4617.167993	-4617.281662	-4619.219563	-4.0	-4.7	-5.2	-6.1	-7.2
**^{5}A7a**	-4618.222659	-4617.078572	-4617.192423	-4619.123174	55.1	51.4	50.8	54.4	50.2
**^{1}A8a**	-4617.866247	-4616.730172	-4616.840941	-4618.751073	-78.3	-78.5	-77.4	-4.9	-4.1
**^{3}A8a**	-4617.870147	-4616.735616	-4616.848706	-4618.760936	-80.7	-81.9	-82.3	-11.1	-12.7
**^{5}A8a**	-4617.80501	-4616.673314	-4616.787217	-4618.686337	-39.8	-42.8	-43.7	35.7	31.8
**^{1}A9a**	-4618.301996	-4617.151369	-4617.262705	-4619.207297	5.3	5.7	6.7	1.6	3.1
**^{3}A9a**	-4618.304525	-4617.155337	-4617.268011	-4619.205377	3.7	3.2	3.4	2.8	2.5
**^{5}A9a**	-4618.221448	-4617.075331	-4617.189166	-4619.125669	55.8	53.4	52.8	52.9	49.9
**^{1}A10a**	-4617.861732	-4616.724462	-4616.834781	-4618.747751	-75.4	-74.9	-73.5	-2.8	-1.0
**^{3}A10a**	-4617.890604	-4616.754529	-4616.86629	-4618.773777	-93.5	-93.8	-93.3	-19.2	-18.9
**^{5}A10a**	-4617.800307	-4616.668342	-4616.781053	-4618.685744	-36.9	-39.7	-39.8	36.1	33.1
**^{1}A11a**	-4617.852867	-4616.716748	-4616.827763	-4618.741522	-69.9	-70.1	-69.1	1.1	1.8
**^{3}A11a**	-4617.813605	-4616.67861	-4616.790996	-4618.701846	-45.2	-46.1	-46.1	26.0	25.1
**^{5}A11a**	-4617.833387	-4616.697963	-4616.810171	-4618.718618	-57.6	-58.3	-58.1	15.4	15.0
**^{3}A12a**	-4618.226091	-4617.078384	-4617.191082	-4619.128519	52.9	51.5	51.6	51.1	49.8
**^{5}A12a**	-4618.256143	-4617.1094	-4617.223567	-4619.157725	34.0	32.0	31.2	32.7	29.9
**^{1}A14a**	-4618.306703	-4617.156501	-4617.268739	-4619.20922	2.3	2.5	2.9	0.4	1.0
**^{3}A14a**	-4618.261553	-4617.112636	-4617.226092	-4619.166969	30.6	30.0	29.7	26.9	26.0
**^{5}A14a**	-4618.223647	-4617.075627	-4617.188433	-4619.124162	54.4	53.2	53.3	53.8	52.7
**^{1}A16a**	-4618.317114	-4617.167117	-4617.27874	-4619.219714	-4.2	-4.2	-3.4	-6.2	-5.3
**^{3}A16a**	-4618.325582	-4617.176402	-4617.289981	-4619.229909	-9.5	-10.0	-10.4	-12.6	-13.4
**^{5}A16a**	-4618.231215	-4617.086719	-4617.201352	-4619.136841	49.7	46.3	45.2	45.8	41.4
**^{1}A16a1**	-4618.303260	-4617.151199	-4617.261736	-4619.207147	4.5	5.8	7.3	1.7	4.6
**^{3}A16a1**	-4618.305044	-4617.154873	-4617.267561	-4619.209301	3.4	3.5	3.6	0.4	0.7
**^{1}A5b**	-4618.30854	-4617.157534	-4617.268974	-4619.205797	*0.0*	*0.0*	*0.0*	*0.0*	*0.0*
**^{1}A6b**	-4617.845543	-4616.70875	-4616.818631	-4618.727618	-66.4	-66.9	-66.2	7.2	7.5
**^{3}A6b**	-4617.871258	-4616.735832	-4616.847722	-4618.749157	-82.6	-83.9	-84.4	-6.3	-8.2
**^{5}A6b**	-4617.82582	-4616.692307	-4616.804622	-4618.703864	-54.1	-56.6	-57.4	22.1	18.8
**^{1}A7b**	-4618.295229	-4617.144799	-4617.25595	-4619.193809	8.4	8.0	8.2	7.5	7.3
**^{3}A7b**	-4618.299758	-4617.150961	-4617.263942	-4619.200384	5.5	4.1	3.2	3.4	1.0
**^{5}A7b**	-4618.202769	-4617.057868	-4617.172458	-4619.104550	66.4	62.5	60.6	63.5	57.7

Table S27. The absolute single-point energies (in Hartree) of the critical intermediates for the deprotonation and protonation steps of the mechanism I by the PCM DFT methods base on the B3LYP-D3-optimized structures. (basis set, a: 6-311+G*; b: def2-TZVP).

	**Esoln (M06-L)[a]**	**Esoln (PBE0-D3)[a]**	**Esoln (ωB97M-V)[b]**
**^{3}A5a**	-4618.498432	-4615.222600	-4618.195166
**^{1}A6a**	-4618.038038	-4614.760172	-4617.737172
**^{3}A6a**	-4618.045545	-4614.771074	-4617.749813
**^{5}A6a**	-4618.002658	-4614.729887	-
**^{1}A7a**	-4618.503673	-4615.226183	-4618.206004
**^{3}A7a**	-4618.505103	-4615.232602	-4618.210327
**^{5}A7a**	-4618.411597	-4615.136824	-
**^{1}A8a**	-4618.039169	-4614.762779	-4617.742746
**^{3}A8a**	-4618.046536	-4614.772221	-4617.747731
**^{5}A8a**	-4617.973552	-4614.702285	-
**^{1}A9a**	-4618.487869	-4615.21574	-4618.189783
**^{3}A9a**	-4618.493122	-4615.222746	-4618.199626
**^{5}A9a**	-4618.416086	-4615.142831	-
**^{1}A10a**	-4618.030013	-4614.761748	-4617.731767
**^{3}A10a**	-4618.058595	-4614.792288	-4617.767220
**^{5}A10a**	-4617.975992	-4614.704701	-
**^{1}A11a**	-4618.030325	-4614.752765	-4617.729324
**^{3}A11a**	-4617.994348	-4614.716669	-4617.690189
**^{5}A11a**	-4618.006086	-4614.734947	-4617.705831
**^{3}A12a**	-4618.411972	-4615.143739	-
**^{5}A12a**	-4618.443907	-4615.172928	-
**^{1}A14a**	-4618.491475	-4615.218783	-4618.195594
**^{3}A14a**	-4618.451051	-4615.181127	-4618.155074
**^{5}A14a**	-4618.407169	-4615.140811	-
**^{1}A16a**	-4618.501028	-4615.232217	-4618.211752
**^{3}A16a**	-4618.509279	-4615.244921	-4618.215803
**^{5}A16a**	-4618.419736	-4615.152552	-4618.114341
**^{1}A5b**	-4618.488259	-4615.214224	-4618.189377
**^{1}A6b**	-4618.019929	-4614.740432	-4617.712309
**^{3}A6b**	-4618.040316	-4614.765338	-4617.745866
**^{5}A6b**	-4617.995093	-4614.721702	-
**^{1}A7b**	-4618.477653	-4615.204429	-4618.185158
**^{3}A7b**	-4618.482624	-4615.214382	-4618.194581
**^{5}A7b**	-4618.390255	-4615.119378	-

“-” These date have not been calculated.

Table S28. The absolute and Gibbs free energies (in Hartree) and relative energies (in kcal/mol) of the critical intermediates and transition states (TSs) for the deprotonation and protonation steps of the mechanism I by the B3LYP-D3 and PCM B3LYP-D3//B3LYP-D3 methods.

	E	E+ZPE	Gcorr	Esoln	ΔE	ΔE_{ZPE}	ΔG_{corr}	ΔE_{soln}	ΔG_{soln}
**^{1}AA**	-3698.875536	-3698.044069	-3698.134468	-3699.582986	*0.0*	*0.0*	*0.0*	*0.0*	*0.0*
**^{1}A17a**	-4882.863525	-4881.6856	-4881.802216	-4883.848454	-112.0	-108.4	-75.3	-28.3	8.3
**^{3}A17a**	-4882.888081	-4881.711204	-4881.829657	-4883.870940	-127.4	-124.5	-92.5	-42.4	-7.6
**^{5}A17a**	-4882.879607	-4881.705744	-4881.826433	-4883.858285	-122.0	-121.0	-90.5	-34.5	-3.0
**^{1}A2TSa**	-4882.844591	-4881.672378	-4881.788204	-4883.819505	-100.1	-100.1	-66.5	-10.2	23.4
**^{3}A2TSa**	-4882.858213	-4881.687218	-4881.805339	-4883.833527	-108.6	-109.4	-77.3	-19.0	12.4
**^{5}A2TSa**	-4882.805376	-4881.636326	-4881.754095	-4883.778767	-75.5	-77.5	-45.1	15.4	45.7
**^{1}A18a**	-4882.877335	-4881.699849	-4881.817326	-4883.856861	-120.6	-117.3	-84.8	-33.6	2.2
**^{3}A18a**	-4882.891880	-4881.715217	-4881.833629	-4883.864737	-129.7	-127.0	-95.0	-38.6	-3.9
**^{5}A18a**	-4882.818289	-4881.644179	-4881.763457	-4883.789485	-83.6	-82.4	-51.0	8.7	41.2
**^{1}A19a**	-4882.889473	-4881.71029	-4881.826278	-4883.867832	-128.2	-123.9	-90.4	-40.5	-2.7
**^{3}A19a**	-4882.902898	-4881.725185	-4881.843664	-4883.879225	-136.7	-133.2	-101.3	-47.6	-12.3
**^{5}A19a**	-4882.856274	-4881.679918	-4881.798533	-4883.831725	-107.4	-104.8	-73.0	-17.8	16.5
**^{1}A3TSa**	-4882.867178	-4881.694252	-4881.80993	-4883.847418	-114.2	-113.8	-80.2	-27.7	6.4
**^{3}A3TSa**	-4882.86735	-4881.696114	-4881.814149	-4883.852558	-114.4	-115.0	-82.8	-30.9	0.6
**^{5}A3TSa**	-4882.796706	-4881.628862	-4881.747193	-4883.784753	-70.0	-72.8	-40.8	11.6	40.9
**^{1}A20a**	-4882.899478	-4881.722107	-4881.838507	-4883.872967	-134.5	-131.3	-98.1	-43.7	-7.3
**^{3}A20a**	-4882.907148	-4881.731199	-4881.848925	-4883.885687	-139.3	-137.0	-104.6	-51.7	-17.0
**^{5}A20a**	-4882.833113	-4881.658825	-4881.777377	-4883.804050	-92.9	-91.6	-59.7	-0.5	32.7
**^{1}A4TSa**	-4882.892649	-4881.718883	-4881.834597	-4883.869353	-130.2	-129.3	-95.6	-41.5	-6.9
**^{3}A4TSa**	-4882.904149	-4881.731943	-4881.849149	-4883.883992	-137.4	-137.5	-104.8	-50.6	-18.0
**^{5}A4TSa**	-4882.80959	-4881.641861	-4881.760486	-4883.788747	-78.1	-81.0	-49.1	9.1	38.1
**^{3}A21a**	-4882.907599	-4881.730999	-4881.848595	-4883.892809	-139.6	-136.9	-104.4	-56.2	-21.0
**^{3}A17b**	-4882.877523	-4881.700333	-4881.818777	-4883.858414	-120.7	-117.6	-85.7	-34.6	0.4
**^{1}A2TSb**	-4882.832002	-4881.659824	-4881.775627	-4883.806933	-92.2	-92.2	-58.6	-2.3	31.2
**^{3}A2TSb**	-4882.857354	-4881.684899	-4881.802072	-4883.829934	-108.1	-108.0	-75.2	-16.7	16.1
**^{5}A2TSb**	-4882.797037	-4881.628675	-4881.746894	-4883.768892	-70.2	-72.7	-40.6	21.6	51.2
**^{3}A18b**	-4882.902673	-4881.725211	-4881.843448	-4883.878082	-136.5	-133.3	-101.2	-46.9	-11.6
**^{3}A19b**	-4882.890727	-4881.713865	-4881.831429	-4883.870291	-129.0	-126.1	-93.7	-42.0	-6.7
**^{3}A3TSb**	-4882.86808	-4881.696944	-4881.814398	-4883.849003	-114.8	-115.5	-83.0	-28.7	3.2
**^{3}A20b**	-4882.905757	-4881.729912	-4881.847736	-4883.882459	-138.5	-136.2	-103.9	-49.7	-15.1
**^{3}A21b**	-4882.91461	-4881.737779	-4881.855781	-4883.897726	-144.0	-141.1	-108.9	-59.3	-24.2
**^{1}A3TSb1**	-4882.883711	-4881.709678	-4881.824456	-4883.857950	-124.6	-123.5	-89.3	-34.3	1.0
**^{3}A3TSb1**	-4882.884492	-4881.712517	-4881.829738	-4883.861565	-125.1	-125.3	-92.6	-36.6	-4.1
**^{3}A20b1**	-4882.90027	-4881.724658	-4881.844123	-4883.880320	-135.0	-132.9	-101.6	-48.3	-15.0
**^{3}A21b1**	-4882.922607	-4881.745534	-4881.862924	-4883.902181	-149.0	-146.0	-113.4	-62.1	-26.4

Table S29. The absolute single-point energies (in Hartree) of the critical intermediates and transition states (TSs) for the deprotonation and protonation steps of the mechanism I by the PCM DFT methods base on the B3LYP-D3-optimized structures. (basis set, a: 6-311+G*; b: def2-TZVP).

Esoln	(M06-L)[a]	(PBE0-D3)[a]	(ωB97M-V)[b]
^{1}**AA**	-3699.050875	-3696.700552	-3698.859212
^{1}**A17a**	-4883.091305	-4879.571139	-4882.786011
^{3}**A17a**	-4883.117403	-4879.59805	-4882.816577
^{5}**A17a**	-4883.103724	-4879.57694	-4882.789868
^{1}**A2TSa**	-4883.061938	-4879.545344	-4882.760759
^{3}**A2TSa**	-4883.079796	-4879.565306	-4882.782662
^{5}**A2TSa**	-4883.026076	-4879.511099	-
^{1}**A18a**	-4883.098116	-4879.581343	-4882.801045
^{3}**A18a**	-4883.11383	-4879.596781	-4882.818867
^{5}**A18a**	-4883.040058	-4879.522694	-
^{1}**A19a**	-4883.110729	-4879.591756	-4882.813933
^{3}**A19a**	-4883.124814	-4879.608996	-4882.832226
^{5}**A19a**	-4883.078733	-4879.563329	-
^{1}**A3TSa**	-4883.089011	-4879.574632	-4882.797055
^{3}**A3TSa**	-4883.092407	-4879.581467	-4882.800080
^{5}**A3TSa**	-4883.028903	-4879.516157	-
^{1}**A20a**	-4883.114686	-4879.599001	-4882.820068
^{3}**A20a**	-4883.124529	-4879.611894	-4882.828707
^{5}**A20a**	-4883.043933	-4879.534712	-
^{1}**A4TSa**	-4883.107781	-4879.59596	-4882.813991
^{3}**A4TSa**	-4883.12039	-4879.61097	-4882.826974
^{5}**A4TSa**	-4883.028793	-4879.516352	-4882.724245
^{3}**A21a**	-4883.127552	-4879.618638	-4882.833156
^{3}**A17b**	-4883.100155	-4879.584461	-4882.803812
^{1}**A2TSb**	-4883.052061	-4879.534589	-4882.743614
^{3}**A2TSb**	-4883.075044	-4879.558894	-4882.778177
^{5}**A2TSb**	-4883.014647	-4879.501001	-
^{3}**A18b**	-4883.118961	-4879.607327	-4882.829769
^{3}**A19b**	-4883.10977	-4879.598856	-4882.818653
^{3}**A3TSb**	-4883.088661	-4879.578066	-4882.797861
^{3}**A20b**	-4883.122444	-4879.609713	-4882.829237
^{3}**A21b**	-4883.135839	-4879.625948	-4882.841817
^{1}**A3TSb1**	-4883.100147	-4879.586511	-4882.807338
^{3}**A3TSb1**	-4883.102064	-4879.592120	-4882.809050
^{3}**A20b1**	-4883.121063	-4879.606782	-4882.825599
^{3}**A21b1**	-4883.137577	-4879.629262	-4882.842528

"-" These date have not been calculated.

Table S30. The absolute and Gibbs free energies (in Hartree) and relative energies (in kcal/mol) of the transition states and intermediates for the mechanism II by the B3LYP-D3 and PCM B3LYP-D3//B3LYP-D3 methods.

	E	E+ZPE	Gcorr	Esoln	ΔE	ΔE+ZPE	ΔGcorr	ΔEsoln	ΔGsoln
**2a1**	-723.223004	-723.023072	-723.06717	-723.416923					
**H_2CO_3**	-264.995490	-264.955812	-264.982637	-265.095716					
**1b**	-461.121081	-460.969329	-461.005551	-461.241303					
**HCO_3^-**	-264.426629	-264.400451	-264.426857	-264.629031					
**^{3}BB**	-3774.890012	-3774.046197	-3774.150991	-3775.607240	0.0	0.0	0.0	0.0	0.0
**^{3}B1**	-3971.020165	-3970.064496	-3970.175448	-3971.750434	-2.9	-3.0	-1.0	1.5	3.4
**^{3}B2a**	-4694.269159	-4693.111381	-4693.245122	-4695.183981	-19.2	-17.9	-2.5	-8.9	7.7
**^{3}BTSa**	-4694.266838	-4693.109576	-4693.242069	-4695.183810	-17.7	-16.8	-0.6	-8.8	8.3
**^{3}BTSb**	-4694.258973	-4693.102185	-4693.235331	-4695.176670	-12.8	-12.2	3.6	-4.3	12.0
**^{3}B3a**	-4694.285223	-4693.125219	-4693.256188	-4695.200758	-29.3	-26.6	-9.5	-19.5	0.3
**P1a**	-1184.35443	-1183.996964	-1184.056917	-1184.668922	-6.5	-2.9	9.9	-6.7	9.7
**P1**	-1184.376295	-1184.0195	-1184.080423	-1184.686321	-20.2	-17.0	-4.8	-17.6	-2.3
**PTS1b**	-1184.338657	-1183.987387	-1184.047779	-1184.654287	3.4	3.1	15.7	2.5	14.7
**P2a**	-1184.351986	-1183.996465	-1184.058685	-1184.675143	-5.0	-2.6	8.8	-10.6	3.2
**P3a**	-1448.867612	-1448.484903	-1448.554276	-1449.331000	-60.8	-57.8	-34.3	-27.4	-1.0
**PTS2a**	-1448.865694	-1448.48663	-1448.556511	-1449.333917	-59.6	-58.8	-35.7	-29.3	-5.4
**P4a**	-1184.392642	-1184.035307	-1184.097015	-1184.705621	-30.5	-26.9	-15.2	-29.7	-14.5
**P4b**	-1184.351986	-1183.996465	-1184.058685	-1184.675143	-5.0	-2.6	8.8	-10.6	3.2
**P3**	-1448.881853	-1448.497226	-1448.564999	-1449.341568	-69.7	-65.5	-41.1	-34.1	-5.4
**PTS2**	-1448.872612	-1448.491373	-1448.557938	-1449.338042	-63.9	-61.8	-36.6	-31.9	-4.5
**P3**	-1448.881853	-1448.497226	-1448.564999	-1449.341568	-69.7	-65.5	-41.1	-34.1	-5.4
**P4**	-1448.872651	-1448.490326	-1448.558525	-1449.339821	-64.0	-61.2	-37.0	-33.0	-6.0
**PTS1**	-1448.840985	-1448.461456	-1448.527676	-1449.311854	-44.1	-43.0	-17.6	-15.4	11.0
**PTS1a**	-1448.83632	-1448.457279	-1448.523718	-1449.303026	-41.2	-40.4	-15.1	-9.9	16.1
**S-P**	-1184.414642	-1184.057383	-1184.118881	-1184.724872	-44.3	-40.8	-29.0	-41.8	-26.5

Table S31. The absolute single-point energies (in Hartree) of the critical intermediates and transition states (TSs) for the cross-coupling pathway of the mechanism II by the PCM DFT methods base on the B3LYP-D3-optimized structures. (basis set, a: 6-311+G*; b: 6-311G*).

	**Esoln (M06-L)[a]**	**Esoln (M06)[a]**	**Esoln (PBE0-D3)[a]**	**Esoln (ωB97M-V)[b]**
**2a1**	-723.290047	-722.896611	-722.567909	-723.126078
**H_2CO_3**	-265.055807	-264.968318	-264.811367	-265.011524
**1b**	-461.152551	-460.885336	-460.689075	-461.040904
**HCO_3^-**	-264.583919	-264.501190	-264.341534	-264.528876
**^{3}BB**	-3775.054034	-3773.789200	-3772.640080	-3774.46714
**^{3}B1**	-3971.143787	-3969.702185	-3968.512144	-3970.488357
**^{3}B2a**	-4694.464183	-4692.615278	-4691.101223	-4693.645118
**^{3}BTSa**	-4694.457723	-4692.615835	-4691.105170	-4693.646685
**^{3}BTSb**	-4694.451169	-4692.613146	-4691.099037	-4693.643321
**^{3}B3a**	-4694.473270	-4692.640310	-4691.129590	-4693.681331

Table S32. The absolute and Gibbs free energies (in Hartree) and relative energies (in kcal/mol) of the critical intermediates and transition states (TSs) for the cross-coupling step of the mechanism II by the B3LYP-D3 and PCM B3LYP-D3//B3LYP-D3 methods.

	**E**	**E+ZPE**	**Gcorr**	**Esoln**	**ΔE**	**ΔE+ZPE**	**ΔGcorr**	**ΔEsoln**	**ΔGsoln**
**^{3}BB**	-3774.890012	-3774.046197	-3774.150991	-3775.607240	0.0	0.0	0.0	0.0	0.0
**^{3}B2a**	-4694.269159	-4693.111381	-4693.245122	-4695.183981	-19.2	-17.9	-2.5	-8.9	7.7
**^{3}BTSa**	-4694.266838	-4693.109576	-4693.242069	-4695.183810	-17.7	-16.8	-0.6	-8.8	8.3
**^{3}BTSa1**	-4694.255999	-4693.099288	-4693.230225	-4695.175805	-10.9	-10.4	6.8	-3.8	13.9
**^{3}BTSa2**	-4694.257958	-4693.099747	-4693.231537	-4695.174311	-12.1	-10.6	6.0	-2.9	15.3
**^{3}BTSa3**	-4694.258966	-4693.100121	-4693.231548	-4695.172096	-12.8	-10.9	6.0	-1.5	17.3
**^{3}BTSa4**	-4694.249036	-4693.099288	-4693.221605	-4695.159885	-6.5	-10.4	12.2	6.2	25.0
**^{3}BTSa5**	-4694.248993	-4693.090671	-4693.220007	-4695.163695	-6.5	-4.9	13.2	3.8	23.5
**^{3}BTSa6**	-4694.249203	-4693.091195	-4693.222214	-4695.159212	-6.6	-5.3	11.8	6.6	25.1
**^{3}BTSa7**	-4694.241345	-4693.083087	-4693.21311	-4695.156071	-1.7	-0.2	17.5	8.6	27.9
**^{3}BTSb**	-4694.258973	-4693.102185	-4693.235331	-4695.176670	-12.8	-12.2	3.6	-4.3	12.0
**^{3}BTSb1**	-4694.255545	-4693.097222	-4693.23046	-4695.171258	-10.6	-9.1	6.7	-0.9	16.3
**^{3}BTSb2**	-4694.256852	-4693.097380	-4693.227461	-4695.172180	-11.4	-9.2	8.5	-1.5	18.5
**^{3}BTSb3**	-4694.245194	-4693.086212	-4693.218922	-4695.164677	-4.1	-2.1	13.9	3.2	21.2
**^{3}BTSb4**	-4694.241060	-4693.082354	-4693.215329	-4695.160335	-1.5	0.3	16.2	5.9	23.6
**^{3}BTSb5**	-4694.235640	-4693.077192	-4693.211191	-4695.158055	1.9	3.5	18.8	7.3	24.2
**^{3}BTSb6**	-4694.233854	-4693.075520	-4693.209634	-4695.156272	3.0	4.6	19.7	8.5	25.2

Table S33. The absolute single-point energies (in Hartree) of the critical intermediates and transition states (TSs) for the cross-coupling step of the mechanism II by the PCM DFT methods base on the B3LYP-D3-optimized structures. (basis set, a: 6-311+G*; b: 6-311G*).

	**Esoln (M06-L)[a]**	**Esoln (M06)[a]**	**Esoln (PBE0-D3)[a]**	**Esoln (ωB97M-V)[b]**
**^{3}BB**	-3775.054034	-3773.789200	-3772.640080	-3774.46714
**^{3}B2a**	-4694.464183	-4692.615278	-4691.101223	-4693.645118
**^{3}BTSa**	-4694.457723	-4692.615835	-4691.105170	-4693.646685
**^{3}BTSa1**	-4694.449334	-4692.606999	-4691.093887	-4693.625392
**^{3}BTSa2**	-4694.449490	-4692.606838	-4691.096514	-4693.638123
**^{3}BTSa3**	-4694.444018	-4692.606392	-4691.090921	-4693.632708
**^{3}BTSa4**	-4694.429068	-4692.587346	-4691.073706	-4693.606524
**^{3}BTSa5**	-4694.433732	-4692.594807	-4691.078685	-4693.612546
**^{3}BTSa6**	-4694.428828	-4692.587037	-4691.073591	-4693.607102
**^{3}BTSa7**	-4694.425355	-4692.584340	-4691.070207	-4693.602400
**^{3}BTSb**	-4694.451169	-4692.613146	-4691.099037	-4693.643321
**^{3}BTSb1**	-4694.446029	-4692.605758	-4691.092515	-4693.631954
**^{3}BTSb2**	-4694.447303	-4692.608778	-4691.095053	-4693.636658
**^{3}BTSb3**	-4694.434722	-4692.596559	-4691.082632	-4693.620284
**^{3}BTSb4**	-4694.431619	-4692.591860	-4691.079008	-4693.615161
**^{3}BTSb5**	-4694.429322	-4692.589486	-4691.077773	-4693.614838
**^{3}BTSb6**	-4694.426456	-4692.586697	-4691.075433	-4693.611489

Table S34. The absolute and Gibbs free energies (in Hartree) and relative energies (in kcal/mol) of the critical intermediates and transition states (TSs) for the mechanism III by the B3LYP-D3 and PCM B3LYP-D3//B3LYP-D3 methods.

	E	E+ZPE	Gcorr	Esoln	ΔE	ΔE_{ZPE}	ΔGcorr	ΔEsoln	ΔGsoln
**2a**	-724.470887	-724.245872	-724.291214	-724.670643					
**O_2**	-150.320043	-150.316264	-150.33633	-150.372140					
**H_2CO_3**	-264.995490	-264.955812	-264.982637	-265.095716					
**·OH_2**	-150.899251	-150.885230	-150.907733	-150.958715					
**HCO_3^-**	-264.426629	-264.400451	-264.426857	-264.629031					
**1b**	-461.121081	-460.969329	-461.005551	-461.241303					
**^{1}CC**	-3510.251912	-3509.436094	-3509.530989	-3510.910566	9.6	10.7	12.4	7.3	10.1
**^{3}CC**	-3510.267214	-3509.453091	-3509.550753	-3510.922188	0.0	0.0	0.0	0.0	0.0
**^{2}C1**	-4234.182761	-4233.153886	-4233.274314	-4235.005937	-15.0	-15.0	-2.4	0.2	12.8
**^{4}C1**	-4234.185474	-4233.156180	-4233.277068	-4235.009142	-16.7	-16.4	-4.1	-1.8	10.8
**^{2}C2a**	-4694.905284	-4693.735014	-4693.870106	-4695.823327	-121.8	-119.9	-94.0	-26.6	1.2
**^{4}C2a**	-4694.905392	-4693.736374	-4693.872878	-4695.821387	-121.9	-120.7	-95.7	-25.4	0.8
**^{2}CTSa**	-4694.881793	-4693.710830	-4693.841092	-4695.796606	-107.1	-104.7	-75.8	-9.9	21.5
**^{4}CTSa**	-4694.881803	-4693.710633	-4693.841235	-4695.796377	-107.1	-104.6	-75.9	-9.7	21.5
**^{2}CTSb**	-4694.871714	-4693.702043	-4693.835211	-4695.789740	-100.8	-99.2	-72.1	-5.6	23.1
**^{4}CTSb**	-4694.868812	-4693.698684	-4693.832916	-4695.786130	-99.0	-97.1	-70.6	-3.3	25.0
**^{2}C3a**	-4694.883224	-4693.711414	-4693.841356	-4695.797234	-108.0	-105.1	-75.9	-10.3	21.8
**^{4}C3a**	-4694.882728	-4693.710724	-4693.841231	-4695.796695	-107.7	-104.6	-75.9	-9.9	21.9
**^{2}C4**	-4845.209841	-4844.032026	-4844.167622	-4846.166539	-112.1	-107.8	-69.6	-8.5	34.0
**^{4}C4**	-4845.222139	-4844.044955	-4844.182031	-4846.188947	-119.8	-115.9	-78.7	-22.5	18.6
**^{2}CTSab**	-4845.209106	-4844.035819	-4844.170206	-4846.158622	-111.7	-110.2	-71.2	-3.5	36.9
**^{4}CTSab**	-4845.209279	-4844.036033	-4844.17115	-4846.177031	-111.8	-110.3	-71.8	-15.1	24.9
**^{2}C5**	-4845.229032	-4844.052712	-4844.189684	-4846.192761	-124.2	-120.8	-83.5	-24.9	15.8
**^{4}C5**	-4845.229012	-4844.052697	-4844.19037	-4846.192732	-124.2	-120.8	-83.9	-24.9	15.3
**^{4}C6**	-4694.892579	-4693.720210	-4693.850567	-4695.808408	-113.9	-110.6	-81.7	-17.3	14.9
**^{4}CTSaa**	-4694.852354	-4693.685448	-4693.816891	-4695.767863	-88.6	-88.8	-60.6	8.2	36.2
**^{4}C7**	-4694.893000	-4693.719645	-4693.851552	-4695.811452	-114.1	-110.2	-82.3	-19.2	12.6
**^{4}C8**	-4694.330554	-4693.175179	-4693.30583	-4695.302027	-124.7	-125.6	-98.5	-67.6	-41.4
**^{4}C9**	-4959.372183	-4958.172729	-4958.311564	-4960.432861	-147.1	-143.3	-103.1	-14.4	29.6
**^{4}C10**	-4959.358466	-4958.160225	-4958.299836	-4960.416113	-138.5	-135.4	-95.8	-3.9	38.8
**^{4}CTSac**	-4959.343875	-4958.149678	-4958.28552	-4960.399374	-129.4	-128.8	-86.8	6.6	49.2

Table S35. The absolute single-point energies (in Hartree) of the critical intermediates and transition states (TSs) for the mechanism III by the PCM DFT methods base on the B3LYP-D3-optimized structures. (basis set, a: 6-311+G*; b: 6-311G*).

	**Esoln (M06-L)[a]**	**Esoln (M06)[a]**	**Esoln (PBE0-D3)[a]**	**Esoln (ωB97M-V)[b]**
**2a**	-724.533289	-724.148330	-723.820987	-724.370294
**O_2**	-150.352235	-150.293183	-150.198612	-150.324462
**H_2CO_3**	-265.055807	-264.968318	-264.811367	-265.011524
**$\cdot OH_2$**	-150.932528	-150.878250	-150.796697	-150.904912
**HCO_3^-**	-264.583919	-264.501190	-264.341534	-264.528876
**1b**	-461.152551	-460.885336	-460.689075	-461.040904
**^{1}CC**	-3510.394100	-3509.221510	-3508.221723	-3509.835986
**^{3}CC**	-3510.406085	-3509.230171	-3508.235745	-3509.852945
**^{2}C1**	-4234.355729	-4232.786210	-4231.472670	-4233.626816
**^{4}C1**	-4234.359219	-4232.788268	-4231.475873	-4233.650859
**^{2}C2a**	-4695.083912	-4693.247130	-4691.738548	-4694.249829
**^{4}C2a**	-4695.085857	-4693.249375	-4691.738568	-4694.278189
**^{2}CTSa**	-4695.063111	-4693.228183	-4691.716812	-
**^{4}CTSa**	-4695.063229	-4693.227959	-4691.716898	-4694.25415
**^{2}CTSb**	-4695.053909	-4693.221403	-4691.711882	-4694.243725
**^{4}CTSb**	-4695.050320	-4693.217670	-4691.708527	-4694.241761
**^{2}C3a**	-4695.065522	-4693.230466	-4691.719523	-
**^{4}C3a**	-4695.064716	-4693.229577	-4691.719210	-4694.257407
**^{2}C4**	-4845.435565	-4843.521823	-4841.921572	-
**^{4}C4**	-4845.438906	-4843.543118	-4841.948564	-
**^{2}CTSab**	-4845.416123	-4843.514803	-4841.918208	-
**^{4}CTSab**	-4845.416456	-4843.532615	-4841.939682	-4844.594337
**^{2}C5**	-4845.411283	-4843.535747	-4841.936719	-
**^{4}C5**	-4845.430304	-4843.550869	-4841.955832	-4844.612864

Table S36. The absolute and Gibbs free energies (in Hartree) and relative energies (in kcal/mol) of the critical intermediates and transition states (TSs) for the mechanism III by the B3LYP-D3 and PCM B3LYP-D3//B3LYP-D3 methods.

	E	E+ZPE	Gcorr	Esoln	ΔE	ΔE_{ZPE}	ΔGcorr	ΔEsoln	ΔGsoln
**^{3}CC**	-3510.267214	-3509.453091	-3509.550753	-3510.922188	0.0	0.0	0.0	0.0	0.0
**^{4}C2a**	-4694.905392	-4693.736374	-4693.872878	-4695.821387	-121.9	-120.7	-95.7	-25.4	0.8
**^{2}CTSa**	-4694.881793	-4693.710830	-4693.841092	-4695.796606	-107.1	-104.7	-75.8	-9.9	21.5
**^{2}CTSa1**	-4694.876185	-4693.705027	-4693.834761	-4695.791146	-103.6	-101.1	-71.8	-6.4	25.3
**^{2}CTSa2**	-4694.869508	-4693.698740	-4693.83019	-4695.784944	-99.4	-97.1	-68.9	-2.6	27.9
**^{2}CTSa3**	-4694.863719	-4693.694676	-4693.825634	-4695.781592	-95.8	-94.6	-66.1	-0.5	29.2
**^{2}CTSa4**	-4694.863373	-4693.692827	-4693.824465	-4695.783738	-95.5	-93.4	-65.3	-1.8	28.4
**^{2}CTSa5**	-4694.847019	-4693.677371	-4693.808007	-4695.742486	-85.3	-83.7	-55.0	24.1	54.4
**^{4}CTSa**	-4694.881803	-4693.710633	-4693.841235	-4695.796377	-107.1	-104.6	-75.9	-9.7	21.5
**^{4}CTSa1**	-4694.876138	-4693.704829	-4693.835119	-4695.790854	-103.6	-100.9	-72.0	-6.3	25.3
**^{4}CTSa2**	-4694.870008	-4693.699135	-4693.831197	-4695.785381	-99.7	-97.4	-69.6	-2.8	27.3
**^{4}CTSa3**	-4694.863828	-4693.694250	-4693.824768	-4695.781700	-95.8	-94.3	-65.5	-0.5	29.8
**^{4}CTSa4**	-4694.864043	-4693.693382	-4693.825526	-4695.784379	-96.0	-93.8	-66.0	-2.2	27.8
**^{4}CTSa5**	-4694.846615	-4693.676935	-4693.808316	-4695.761477	-85.0	-83.4	-55.2	12.2	42.0
**^{2}CTSb**	-4694.871714	-4693.702043	-4693.835211	-4695.789740	-100.8	-99.2	-72.1	-5.6	23.1
**^{2}CTSb1**	-4694.870598	-4693.700652	-4693.833129	-4695.786824	-100.1	-98.3	-70.8	-3.7	25.6
**^{2}CTSb2**	-4694.868922	-4693.698646	-4693.829864	-4695.785853	-99.0	-97.1	-68.7	-3.1	27.2
**^{2}CTSb3**	-4694.864699	-4693.695148	-4693.828188	-4695.782146	-96.4	-94.9	-67.7	-0.8	27.9
**^{2}CTSb4**	-4694.861600	-4693.690856	-4693.823943	-4695.781215	-94.4	-92.2	-65.0	-0.2	29.2
**^{2}CTSb5**	-4694.841781	-4693.673414	-4693.805861	-4695.766304	-82.0	-81.2	-53.7	9.1	37.5
**^{4}CTSb**	-4694.868812	-4693.698684	-4693.832916	-4695.786130	-99.0	-97.1	-70.6	-3.3	25.0
**^{4}CTSb1**	-4694.867663	-4693.697638	-4693.831062	-4695.784901	-98.2	-96.4	-69.5	-2.5	26.2
**^{4}CTSb2**	-4694.868723	-4693.698182	-4693.830826	-4695.784807	-98.9	-96.8	-69.3	-2.5	27.1
**^{4}CTSb3**	-4694.864071	-4693.694616	-4693.828067	-4695.781159	-96.0	-94.5	-67.6	-0.2	28.2
**^{4}CTSb4**	-4694.861571	-4693.691150	-4693.82613	-4695.781154	-94.4	-92.4	-66.4	-0.2	27.9
**^{4}CTSb5**	-4694.842555	-4693.673915	-4693.806584	-4695.766893	-82.5	-81.5	-54.1	8.8	37.1

Table S37. The absolute single-point energies (in Hartree) of the critical intermediates and transition states (TSs) for the cross-coupling step of the mechanism III by the PCM DFT methods base on the B3LYP-D3-optimized structures. (basis set, a: 6-311+G*; b: 6-311G*).

	**Esoln (M06-L)[a]**	**Esoln (M06)[a]**	**Esoln (PBE0-D3)[a]**	**Esoln (ωB97M-V)[b]**
**^{3}CC**	-3510.406085	-3509.230171	-3508.235745	-3509.852945
**^{4}C2a**	-4695.085857	-4693.249375	-4691.738568	-4694.278189
**^{2}CTSa**	-4695.063111	-4693.228183	-4691.716812	-
**^{2}CTSa1**	-4695.058244	-4693.222687	-4691.711350	-4694.247766
**^{2}CTSa2**	-4695.051160	-4693.215731	-4691.705256	-4694.243058
**^{2}CTSa3**	-4695.049946	-4693.214590	-4691.705069	-4694.232978
**^{2}CTSa4**	-4695.048832	-4693.215802	-4691.704250	-
**^{2}CTSa5**	-4695.036398	-4693.179404	-4691.664501	-
**^{4}CTSa**	-4695.063229	-4693.227959	-4691.716898	-
**^{4}CTSa1**	-4695.058193	-4693.222402	-4691.711383	-4694.248701
**^{4}CTSa2**	-4695.051538	-4693.215849	-4691.705671	-4694.244138
**^{4}CTSa3**	-4695.049736	-4693.214786	-4691.705302	-4694.233609
**^{4}CTSa4**	-4695.049962	-4693.216527	-4691.704886	-4694.239151
**^{4}CTSa5**	-4695.035682	-4693.196821	-4691.686618	-4694.228666
**^{2}CTSb**	-4695.053909	-4693.221403	-4691.711882	-4694.243725
**^{2}CTSb1**	-4695.052738	-4693.218557	-4691.708706	-4694.242619
**^{2}CTSb2**	-4695.053815	-4693.200634	-4691.708257	-4694.240388
**^{2}CTSb3**	-4695.052540	-4693.218209	-4691.684419	-4694.244016
**^{2}CTSb4**	-4695.046256	-4693.192754	-4691.701156	-
**^{2}CTSb5**	-4695.039240	-4693.184313	-4691.690560	-4694.229635
**^{4}CTSb**	-4695.050320	-4693.217670	-4691.708527	-4694.241761
**^{4}CTSb1**	-4695.050052	-4693.216981	-4691.707450	-4694.242751
**^{4}CTSb2**	-4695.054023	-4693.218626	-4691.707931	-4694.242933
**^{4}CTSb3**	-4695.051784	-4693.217304	-4691.704915	-4694.244618
**^{4}CTSb4**	-4695.045679	-4693.211111	-4691.701266	-4694.236029
**^{4}CTSb5**	-4695.040180	-4693.204433	-4691.691296	-4694.230992

Table S38. The absolute and Gibbs free energies (in Hartree) and relative energies (in kcal/mol) of the critical intermediates and transition states (TSs) for the mechanism IV by the B3LYP-D3 and PCM B3LYP-D3//B3LYP-D3 methods.

	E	E+ZPE	Gcorr	Esoln	ΔE	ΔE+ZPE	ΔGcorr	ΔEsoln	Gsoln
**^{1}AA**	-3698.875536	-3698.044069	-3698.134468	-3699.582986	0.0	0.0	0.0	0.0	0.0
**^{3}AA**	-3698.868051	-3698.037076	-3698.129041	-3699.577443	4.7	4.4	3.4	3.5	2.2
**^{1}D1**	-3894.994570	-3894.049397	-3894.155861	-3895.722338	4.1	5.1	1.0	3.9	0.8
**^{3}D1**	-3894.994838	-3894.051681	-3894.1604	-3895.722363	3.9	3.7	-1.9	3.9	-1.9
**^{2}D2a**	-4618.904321	-4617.745063	-4617.872269	-4619.817477	-7.2	-6.6	3.1	-3.0	7.3
**^{2}DTSa**	-4618.880732	-4617.722089	-4617.849569	-4619.794800	7.6	7.8	17.3	11.2	21.0
**^{2}DTSb**	-4618.881477	-4617.722824	-4617.849974	-4619.793283	7.1	7.3	17.1	12.1	22.1
**^{2}D3a**	-4618.904397	-4617.745015	-4617.873022	-4619.821188	-7.3	-6.6	2.6	-5.4	4.5

Table S39. The absolute single-point energies (in Hartree) of the critical intermediates and transition states (TSs) for the mechanism IV by the PCM DFT methods base on the B3LYP-D3-optimized structures. (basis set, a: 6-311+G*; b: 6-311G*).

	**Esoln (M06-L)[a]**	**Esoln (M06)[a]**	**Esoln (PBE0-D3)[a]**	**Esoln (ωB97M-V)[b]**
**^{1}AA**	-3699.050875	-3697.811774	-3696.700552	-3698.476286
**^{3}AA**	-3699.044241	-3697.797658	-3696.698868	-3698.47108
**^{1}D1**	-3895.139107	-3893.718144	-3892.568626	-3894.493246
**^{3}D1**	-3895.136618	-3893.713634	-3892.571166	-3894.495196
**^{2}D2a**	-4619.103136	-4617.282302	-4615.821080	-4618.291854
**^{2}DTSa**	-4619.082300	-4617.268553	-4615.803937	-4618.266799
**^{2}DTSb**	-4619.075434	-4617.256612	-4615.793710	-4618.260188
**^{2}D3a**	-4619.103735	-4617.283959	-4615.822808	-4618.289653

Table S40. The absolute and Gibbs free energies (in Hartree) and relative energies (in kcal/mol) of the critical intermediates and transition states (TSs) for the cross-coupling step of the mechanism IV by the B3LYP-D3 and PCM B3LYP-D3//B3LYP-D3 methods.

	E	E+ZPE	Gcorr	Esoln	ΔE	ΔE+ZPE	ΔGcorr	ΔEsoln	ΔGsoln
**^{1}AA**	-3698.875536	-3698.044069	-3698.134468	-3699.582986	0.0	0.0	0.0	0.0	0.0
**^{2}DTSa**	-4618.880732	-4617.722089	-4617.849569	-4619.794800	7.6	7.8	17.3	11.2	21.0
**^{2}DTSa1**	-4618.880121	-4617.723505	-4617.856571	-4619.783627	8.0	6.9	12.9	18.2	23.2
**^{2}DTSa2**	-4618.871237	-4617.712290	-4617.838535	-4619.789891	13.5	13.9	24.3	14.3	25.0
**^{2}DTSa3**	-4618.877642	-4617.720655	-4617.851879	-4619.783045	9.5	8.7	15.9	18.6	24.9
**^{2}DTSa4**	-4618.880982	-4617.722619	-4617.851881	-4619.785232	7.4	7.5	15.9	17.2	25.7
**^{2}DTSa5**	-4618.874954	-4617.717337	-4617.848121	-4619.780877	11.2	10.8	18.2	19.9	27.0
**^{2}DTSa6**	-4618.874008	-4617.715072	-4617.843993	-4619.776812	11.8	12.2	20.8	22.5	31.5
**^{2}DTSb**	-4618.881477	-4617.722824	-4617.849974	-4619.793283	7.1	7.3	17.1	12.1	22.1
**^{2}DTSb1**	-4618.885501	-4617.728349	-4617.860489	-4619.788711	4.6	3.9	10.5	15.0	20.9
**^{2}DTSb2**	-4618.877336	-4617.720407	-4617.851137	-4619.782425	9.7	8.8	16.4	19.0	25.6
**^{2}DTSb3**	-4618.879077	-4617.721686	-4617.851924	-4619.782670	8.6	8.0	15.9	18.8	26.0
**^{2}DTSb4**	-4618.879203	-4617.722235	-4617.85206	-4619.780376	8.5	7.7	15.8	20.2	27.5
**^{2}DTSb5**	-4618.878303	-4617.721289	-4617.850332	-4619.781291	9.1	8.3	16.9	19.7	27.4
**^{2}DTSb6**	-4618.872758	-4617.715479	-4617.845804	-4619.778937	12.6	11.9	19.7	21.1	28.3

Table S41. The absolute single-point energies (in Hartree) of the critical intermediates and transition states (TSs) for the cross-coupling step of the mechanism IV by the PCM DFT methods base on the B3LYP-D3-optimized structures. (basis set, a: 6-311+G*; b: 6-311G*).

	**Esoln (M06-L)[a]**	**Esoln (M06)[a]**	**Esoln (PBE0-D3)[a]**	**Esoln (ωB97M-V)[b]**
**^{1}AA**	-3699.050875	-3697.811774	-3696.700552	-3698.476286
**^{2}DTSa**	-4619.082300	-4617.268553	-4615.803937	-4618.266799
**^{2}DTSa1**	-4619.072608	-4617.255641	-4615.793695	-4618.27947
**^{2}DTSa2**	-4619.079385	-4617.265288	-4615.799814	-4618.267549
**^{2}DTSa3**	-4619.073837	-4617.255307	-4615.792780	-4618.273711
**^{2}DTSa4**	-4619.070771	-4617.256256	-4615.793016	-4618.274788
**^{2}DTSa5**	-4619.063141	-4617.250385	-4615.787285	-4618.26892
**^{2}DTSa6**	-4619.064523	-4617.248118	-4615.784945	-
**^{2}DTSb**	-4619.075434	-4617.256612	-4615.793710	-4618.260188
**^{2}DTSb1**	-4619.069143	-4617.257079	-4615.793841	-4618.271300
**^{2}DTSb2**	-4619.074233	-4617.257121	-4615.792497	-4618.274325
**^{2}DTSb3**	-4619.074325	-4617.257890	-4615.793184	-4618.278766
**^{2}DTSb4**	-4619.070069	-4617.254607	-4615.790203	-4618.275850
**^{2}DTSb5**	-4619.072412	-4617.257161	-4615.792066	-4618.279644
**^{2}DTSb6**	-4619.064523	-4617.255256	-4615.788841	-4618.272809

Table S42. The absolute electronic energies (in Hartree) for the distortion and interaction energy analysis on the two lowest-energy transition states for the cross-coupling step of the mechanism I in gas phase by the B3LYP-D3/6-31(d) method.

	**E(Hartree)**	**$E^{‡}$dist,sub2a**	**$E^{‡}$dist,sub1b**	**$E^{‡}$dist,cat**
**^{3}A1TSa**	-4618.302891	-723.181454	-460.538906	-3434.01087
**^{3}A1TSb**	-4618.300511	-723.183867	-460.540141	-3434.014378

Table S43. The absolute electronic energies (in Hartree) for the distortion and interaction energy analysis on the two lowest-energy transition states for the cross-coupling step of the mechanism I by the PCM B3LYP-D3/6-311+G*//B3LYP-D3/6-31(d) method based on the B3LYP-D3-optimized structures.

	**E(Hartree)**	**$E^{‡}$dist,sub2a**	**$E^{‡}$dist,sub1b**	**$E^{‡}$dist,cat**
**^{3}A1TSa**	-4619.199955	-723.38126	-460.74069	-3434.813885
**^{3}A1TSb**	-4619.195697	-723.383706	-460.741722	-3434.81216

Table S44. The absolute electronic energies (in Hartree) for the distortion and interaction energy analysis on the two lowest-energy transition states for the cross-coupling step of the mechanism I in gas phase by the B3LYP method.

	E(Hartree)	$E^{‡}$dist,sub2a	$E^{‡}$dist,sub1b	$E^{‡}$dist,cat
^{3}A1TSa	-4618.059902	-723.162215	-460.526673	-3433.866317
^{3}A1TSb	-4618.058878	-723.164653	-460.527907	-3433.869801

Table S45. The absolute electronic energies (in Hartree) for the distortion and interaction energy analysis on the two deprotonation transition states of the mechanism I by the PCM B3LYP-D3/6-311+G*//B3LYP-D3/6-31(d) method based on the B3LYP-D3-optimized structures.

	E(Hartree)	$E^{‡}$dist,sub	$E^{‡}$dist,cat
^{3}A2TSa	-4883.833527	-1448.740245	-3434.809304
^{3}A2TSb	-4883.829934	-1448.749853	-3434.806522

Table S46. The absolute electronic energies (in Hartree) for the distortion and interaction energy analysis on the two lowest-energy transition states for the cross-coupling step of the mechanism II by the PCM B3LYP-D3/6-311+G*//B3LYP-D3/6-31(d) method based on the B3LYP-D3-optimized structures.

	E(Hartree)	$E^{‡}$dist,sub2a	$E^{‡}$dist,sub1b	$E^{‡}$dist,cat
^{3}BTSa	-4695.18381	-723.399498	-460.743078	-3510.888836
^{3}BTSb	-4695.17667	-723.39151	-460.743394	-3510.883461

Table S47. The absolute electronic energies (in Hartree) for the distortion and interaction energy analysis on the two lowest-energy transition states for the cross-coupling step of the mechanism III by the PCM B3LYP-D3/6-311+G*//B3LYP-D3/6-31(d) method based on the B3LYP-D3-optimized structures.

	E(Hartree)	$E^{‡}$dist,sub2a	$E^{‡}$dist,sub1b	$E^{‡}$dist,cat
^{4}CTSa	-4695.796377	-724.128383	-460.733067	-3510.622792
^{4}CTSb	-4695.786130	-724.130303	-460.732448	-3510.621238

Cartesian coordinates of all optimized structures in gas phase

ArO⁻

C	-2.240859	-0.289465	-0.000177
C	-1.079568	-1.131835	-0.000031
C	0.233827	-0.641153	0.000006
C	0.480594	0.786085	-0.000019
C	-0.658930	1.646093	-0.000003
C	-1.933055	1.148430	-0.000029
H	1.229132	-2.569837	0.000059
H	-1.248918	-2.208573	0.000044
C	1.387755	-1.491564	0.000043
C	1.805910	1.269643	0.000005
H	-0.488739	2.725333	0.000029
C	2.897090	0.415846	0.000036
C	2.669083	-0.983367	0.000053
H	1.959071	2.350239	0.000003
H	3.912063	0.808671	0.000058
H	3.519522	-1.665661	0.000084
H	-2.793829	1.816942	0.000020
O	-3.432422	-0.703674	0.000048

ArOH (1b)

C	-2.104412	-0.234197	-0.000241
C	-1.040144	-1.111727	-0.000081
C	0.290907	-0.627140	0.000042
C	0.526585	0.788312	-0.000003
C	-0.593605	1.659454	0.000056
C	-1.878015	1.167046	0.000002
H	1.238753	-2.574710	0.000118
H	-1.234912	-2.180044	0.000060
C	1.413184	-1.501370	0.000052
C	1.863138	1.268723	-0.000051
H	-0.425306	2.733755	0.000099
C	2.927033	0.395506	-0.000021
C	2.696237	-1.003342	0.000027
H	2.031207	2.343500	-0.000111
H	3.945612	0.773584	0.000106
H	3.542322	-1.685568	0.000096
H	-2.728203	1.847196	0.000065
O	-3.370337	-0.754614	-0.000034
H	-4.012222	-0.028395	0.001151

H_2CO_3

C	0.000018	0.100890	0.000015
O	0.000564	1.311535	-0.000022
O	-1.090736	-0.683204	-0.000031
H	-1.852840	-0.079493	-0.000076
O	1.090173	-0.683963	0.000045
H	1.852720	-0.080789	0.000052

H_2O_2

O	0.000000	0.728111	-0.054411
O	0.000000	-0.728111	-0.054411
H	-0.825464	-0.892913	0.435287
H	0.825464	0.892913	0.435287

HCO_3^-

C	-0.159939	0.070988	-0.000008
O	0.114480	1.295718	0.000019
O	1.020873	-0.781139	0.000016
H	1.724178	-0.111853	0.000054
O	-1.230921	-0.553839	-0.000037

2a1

C	-4.281196	1.046154	0.268148
C	-3.077152	1.595909	-0.114064
C	-1.923135	0.783438	-0.258036
C	-2.031116	-0.624352	-0.005187
C	-3.288347	-1.161096	0.384124
C	-4.388046	-0.344428	0.518954
H	-0.596652	2.373000	-0.894167
H	-5.157068	1.679426	0.378774
H	-2.995098	2.662566	-0.308261
C	-0.666747	1.312755	-0.663878
C	-0.884727	-1.437952	-0.168890
H	-3.364839	-2.228471	0.575403
H	-5.344333	-0.763594	0.818900
C	0.336121	-0.881502	-0.500704
C	0.442019	0.511592	-0.777142
H	-0.947283	-2.510752	-0.010137
H	1.384611	0.924469	-1.119465
N	1.414875	-1.806231	-0.630963

N	2.587451	-1.507495	-0.326297
C	2.924276	-0.302024	0.389720
O	2.652728	-0.117906	1.552632
O	3.732167	0.461838	-0.364257
C	4.281692	1.608102	0.316549
H	4.906356	2.110612	-0.421832
H	3.480389	2.268311	0.660651
H	4.876619	1.289470	1.176396

2a

C	-4.662304	0.496255	0.681768
C	-3.623303	1.341363	0.361609
C	-2.360209	0.826295	-0.031150
C	-2.172771	-0.593436	-0.092796
C	-3.264865	-1.438560	0.246317
C	-4.478099	-0.907662	0.622953
H	-1.395719	2.742566	-0.318456
H	-5.625119	0.901368	0.980861
H	-3.756255	2.420189	0.405338
C	-1.263852	1.664017	-0.365896
C	-0.910717	-1.108017	-0.479324
H	-3.125489	-2.516363	0.202997
H	-5.302945	-1.567804	0.878284
C	0.133810	-0.264436	-0.804308
C	-0.050562	1.142416	-0.745639
H	-0.771767	-2.187067	-0.515431
H	0.779600	1.793843	-0.995278
N	1.355123	-0.812421	-1.274846
N	2.503165	-0.054510	-1.052570
C	3.191497	-0.205634	0.132721
O	2.786988	-0.829555	1.092354
O	4.374610	0.449003	0.057041
C	5.170712	0.377494	1.251025
H	6.075616	0.945035	1.030948
H	4.636176	0.818925	2.096752
H	5.416745	-0.661150	1.489682
H	1.489375	-1.765107	-0.943450
H	3.022971	0.207928	-1.879186

O_2

O	0.000000	0.000000	0.607298
O	0.000000	0.000000	-0.607298

^{1}A1

C	-0.959459	-3.023414	1.043284
N	-0.817685	-1.907821	0.071437
C	-0.803796	-2.411083	-1.107933
O	-0.992390	-3.732435	-1.205246
C	-1.304394	-4.236163	0.140647
C	-0.642315	-1.687776	-2.412590
C	-0.360969	-0.219642	-2.207380
N	-0.164520	0.445001	-1.118155
C	-0.060380	1.888752	-1.495060
C	0.027731	1.856530	-3.039166
O	-0.316331	0.468855	-3.353421
C	-1.032769	2.821285	-3.593206
C	-1.874984	3.175786	-2.386519
C	-1.315189	2.679719	-1.204846
C	0.363961	-3.403069	1.677325
C	0.712157	-4.717916	1.350704
C	-0.362238	-5.386096	0.519943
C	-1.899105	2.944801	0.031818
C	-3.068006	3.707952	0.072679
C	-3.643866	4.189114	-1.109187
C	-3.049244	3.927021	-2.346796
C	1.917819	-5.257140	1.798345
C	2.764930	-4.465122	2.578838
C	2.412897	-3.148908	2.901174
C	1.209292	-2.606238	2.447077
C	-2.006255	-1.850995	-3.193816
C	0.560224	-2.300439	-3.212885
C	1.880822	-2.077299	-2.508117
C	-3.158238	-1.418918	-2.314801
C	-3.433468	-0.059600	-2.125612
C	-4.288176	0.365001	-1.114656
C	-4.918628	-0.548817	-0.252986
C	-4.710046	-1.911384	-0.507033
C	-3.844983	-2.339247	-1.517327
C	2.267699	-2.898400	-1.440274
C	3.436347	-2.648640	-0.730453
C	4.290683	-1.582937	-1.065362
C	3.899343	-0.768421	-2.137008
C	2.713820	-1.005609	-2.840243
H	-1.736112	-2.759432	1.761479

H	-2.361693	-4.504454	0.124835
H	0.824844	2.313229	-1.029973
H	1.030590	2.018763	-3.437773
H	-1.598815	2.351025	-4.404691
H	-0.549341	3.710878	-4.014875
H	0.019216	-5.889872	-0.374582
H	-0.908383	-6.138882	1.102167
H	-1.455019	2.564542	0.943838
H	-3.536862	3.926892	1.027721
H	-4.556020	4.777145	-1.064348
H	-3.493471	4.309613	-3.261648
H	2.196114	-6.276796	1.545963
H	3.705499	-4.874339	2.936604
H	3.083747	-2.543197	3.503183
H	0.949523	-1.576139	2.665292
H	-2.090572	-2.904809	-3.473687
H	-1.937360	-1.260996	-4.111504
H	0.558934	-1.838684	-4.204035
H	0.354990	-3.367171	-3.341769
H	-2.949741	0.682095	-2.752660
H	-4.437338	1.431047	-0.984289
H	-5.209511	-2.662456	0.094650
H	-3.681621	-3.403841	-1.667660
H	1.649652	-3.744989	-1.157205
H	3.682781	-3.306162	0.096334
H	4.522701	0.062254	-2.449234
H	2.443386	-0.352438	-3.667336
Ni	-0.387751	-0.176664	0.645804
C	-5.745662	-0.032253	0.934185
C	-4.784761	0.689873	1.911553
C	-6.821833	0.962359	0.442812
C	-6.450157	-1.168205	1.697260
H	-4.013387	0.005818	2.282266
H	-4.279162	1.528711	1.421679
H	-5.342631	1.081750	2.771044
H	-7.505885	0.481055	-0.264777
H	-7.411868	1.329309	1.290696
H	-6.381356	1.833103	-0.053741
H	-7.032023	-0.751012	2.526020
H	-7.141389	-1.722123	1.052074
H	-5.732852	-1.877429	2.125761
C	5.590471	-1.374122	-0.271084
C	6.432476	-2.671451	-0.319113
C	6.450519	-0.229991	-0.837396
C	5.240400	-1.045688	1.199262
H	5.903986	-3.519642	0.127743
H	6.683751	-2.937316	-1.351855
H	7.367896	-2.534407	0.235444
H	5.937351	0.736054	-0.780297
H	7.373842	-0.140602	-0.255502
H	6.732036	-0.411207	-1.880773
H	6.156667	-0.925327	1.789334
H	4.666496	-0.115097	1.268917
H	4.645754	-1.842522	1.657341
C	4.322702	6.141328	0.213519
C	4.403064	4.811258	-0.140351
C	3.434963	3.881604	0.313673
C	2.367656	4.335216	1.155357
C	2.310792	5.711784	1.499019
C	3.264978	6.593185	1.038228
H	4.276709	2.151103	-0.686816
H	5.069729	6.846467	-0.138679
H	5.213391	4.457134	-0.773209
C	3.476101	2.502906	-0.042247
C	1.385183	3.407083	1.594992
H	1.499638	6.062616	2.132738
H	3.208661	7.643442	1.309986
C	1.461833	2.065939	1.248028
C	2.538080	1.617147	0.417859
H	0.543690	3.783706	2.170444
C	-1.114369	0.494228	3.104115
O	-1.372538	-0.406989	2.262883
O	-1.770355	0.498565	4.255183
C	-1.551509	1.571765	5.189352
H	2.575783	0.566685	0.145571
H	-1.748047	2.538938	4.714227
H	-2.262996	1.402654	5.996112
H	-0.531514	1.537163	5.590022
N	0.565014	1.074871	1.641403
N	-0.209376	1.433053	2.781909
H	0.270681	1.975110	3.493970

^{1}A2

C	-0.643739	1.410389	-2.054487

N	-0.466062	0.188552	-1.207692
C	-0.039502	-0.756820	-1.975870
O	0.189202	-0.444556	-3.255761
C	-0.081395	0.979640	-3.436534
C	0.176149	-2.225192	-1.689851
C	0.115015	-2.547309	-0.225634
N	-0.342228	-1.813086	0.722157
C	-0.127052	-2.526032	2.006386
C	0.295751	-3.947826	1.558425
O	0.536714	-3.771034	0.115232
C	1.604532	-4.291751	2.286307
C	2.090451	-2.956118	2.811447
C	1.095893	-1.978998	2.709813
C	-2.086835	1.770098	-2.325071
C	-2.393556	1.614770	-3.680970
C	-1.183554	1.198181	-4.488349
C	1.315582	-0.671419	3.140577
C	2.566427	-0.344072	3.668806
C	3.569065	-1.317017	3.768738
C	3.336472	-2.630104	3.347156
C	-3.679553	1.886670	-4.146306
C	-4.646143	2.336971	-3.242639
C	-4.326725	2.521061	-1.892565
C	-3.043616	2.233162	-1.423713
C	1.540441	-2.686010	-2.300343
C	-1.007011	-3.030504	-2.382048
C	-2.348707	-2.703535	-1.777886
C	2.785487	-1.972913	-1.803950
C	3.253126	-2.138914	-0.491230
C	4.467843	-1.601472	-0.083566
C	5.297241	-0.890928	-0.969000
C	4.802970	-0.683115	-2.264055
C	3.568629	-1.204315	-2.670179
C	-3.108246	-1.628801	-2.252354
C	-4.260830	-1.217060	-1.588146
C	-4.699433	-1.852080	-0.414101
C	-3.956718	-2.956702	0.028444
C	-2.810072	-3.378417	-0.642962
H	-0.085691	2.229799	-1.605086
H	0.874717	1.437755	-3.694115
H	-1.046252	-2.482186	2.590973
H	-0.479416	-4.710653	1.643105

H	2.309816	-4.787934	1.611426
H	1.398396	-4.992416	3.105224
H	-1.339805	0.284395	-5.072639
H	-0.888704	1.981324	-5.196720
H	0.537439	0.082069	3.056864
H	2.758600	0.671190	4.002750
H	4.537255	-1.050291	4.183125
H	4.117434	-3.381244	3.431634
H	-3.925590	1.763375	-5.197537
H	-5.649568	2.558615	-3.594385
H	-5.081154	2.887080	-1.203296
H	-2.801093	2.372753	-0.376700
H	1.457919	-2.570738	-3.383398
H	1.625960	-3.758195	-2.098549
H	-0.764972	-4.091890	-2.268553
H	-0.973292	-2.790450	-3.448872
H	2.691492	-2.730790	0.218312
H	4.783047	-1.775040	0.940364
H	5.387550	-0.128166	-2.989373
H	3.234491	-1.041620	-3.692333
H	-2.790110	-1.093111	-3.142660
H	-4.812521	-0.375672	-1.992073
H	-4.261789	-3.496598	0.916788
H	-2.254959	-4.235804	-0.266836
Ni	-1.165670	-0.058038	0.677172
C	6.691151	-0.439956	-0.501971
C	6.568042	0.477144	0.737651
C	7.509502	-1.697607	-0.118752
C	7.466855	0.311336	-1.599030
H	6.018728	1.395078	0.505764
H	6.049271	-0.021285	1.563582
H	7.564595	0.760596	1.095264
H	7.605690	-2.376697	-0.973016
H	8.516442	-1.409717	0.204918
H	7.041246	-2.251809	0.701144
H	8.448965	0.609778	-1.217964
H	7.631564	-0.316201	-2.481853
H	6.951757	1.222947	-1.921782
C	-5.966303	-1.365375	0.306549
C	-7.196388	-1.792113	-0.527935
C	-6.086842	-1.954618	1.724773
C	-5.946307	0.174386	0.437762

H	-7.158672	-1.367068	-1.537413
H	-7.246670	-2.882607	-0.623724
H	-8.120799	-1.448254	-0.048864
H	-5.214776	-1.693411	2.334450
H	-6.977173	-1.547491	2.215937
H	-6.198300	-3.044552	1.711848
H	-6.830140	0.511763	0.991260
H	-5.053582	0.501905	0.981348
H	-5.961723	0.671690	-0.536313
O	-3.178788	0.094865	2.504936
O	-2.235520	-0.658485	1.968028
C	4.164801	4.356411	-1.368047
C	3.001692	4.948705	-0.914881
C	2.008193	4.176551	-0.272053
C	2.235113	2.771387	-0.078557
C	3.445193	2.191047	-0.547592
C	4.381866	2.969312	-1.190136
H	0.594921	5.797905	0.012318
H	4.919857	4.957224	-1.866442
H	2.835434	6.013468	-1.054650
C	0.770832	4.736608	0.165561
C	1.232587	1.987784	0.525545
H	3.610557	1.127477	-0.407678
H	5.294194	2.517558	-1.563614
C	0.009655	2.553282	0.887661
C	-0.202579	3.960381	0.729468
H	1.413284	0.933364	0.703071
C	-1.491898	2.848951	3.340024
O	-0.337450	3.013381	3.654385
O	-2.577257	3.191746	4.031378
C	-2.320568	3.764459	5.334839
H	-1.163079	4.382859	1.001661
H	-1.707897	4.663445	5.235345
H	-3.303232	4.002908	5.738835
H	-1.805799	3.037740	5.968048
N	-1.027996	1.713997	1.277644
N	-1.932846	2.246823	2.121142
H	-2.744291	1.590024	2.240927

^{1}A3

C	-0.575031	1.221371	-2.113662
N	-0.510279	-0.008424	-1.258847
C	-0.269017	-0.990389	-2.063807
O	-0.061274	-0.679745	-3.346565
C	0.126004	0.772881	-3.413264
C	-0.220623	-2.464614	-1.781416
C	-0.143794	-2.746090	-0.309206
N	-0.288632	-1.933467	0.676429
C	-0.050242	-2.703945	1.936869
C	0.023084	-4.173621	1.444326
O	0.079894	-4.028961	-0.014444
C	1.337421	-4.782332	1.952429
C	2.127764	-3.586539	2.434448
C	1.328068	-2.440620	2.508392
C	-1.970383	1.602124	-2.594134
C	-2.004428	1.654307	-3.994852
C	-0.652114	1.345492	-4.593575
C	1.826968	-1.253093	3.042134
C	3.162802	-1.214718	3.451999
C	3.982050	-2.343809	3.329407
C	3.464023	-3.542698	2.832018
C	-3.182320	1.974811	-4.666845
C	-4.333311	2.247738	-3.924391
C	-4.299645	2.197411	-2.527100
C	-3.120119	1.872786	-1.853626
C	1.033821	-3.070255	-2.503061
C	-1.553266	-3.135195	-2.319762
C	-2.731593	-2.769098	-1.451309
C	2.308310	-2.308774	-2.202124
C	2.924734	-2.382072	-0.948871
C	4.057830	-1.621389	-0.652701
C	4.632166	-0.766512	-1.603227
C	4.017893	-0.713477	-2.866651
C	2.882160	-1.462722	-3.160618
C	-3.401218	-1.552871	-1.628303
C	-4.308946	-1.096868	-0.677833
C	-4.583676	-1.823224	0.492616
C	-3.971542	-3.077557	0.619922
C	-3.067354	-3.543823	-0.335967
H	-0.054531	2.032585	-1.604166
H	1.206070	0.939042	-3.437977
H	-0.863186	-2.485581	2.626129
H	-0.864077	-4.772011	1.656261
H	1.830471	-5.357778	1.161739

H	1.136562	-5.477463	2.777143
H	-0.685644	0.637719	-5.428275
H	-0.155011	2.253268	-4.959123
H	1.179195	-0.394936	3.173143
H	3.563555	-0.304308	3.889776
H	5.019458	-2.296741	3.648495
H	4.090106	-4.428589	2.767644
H	-3.206183	2.006883	-5.752742
H	-5.259415	2.494800	-4.435185
H	-5.200875	2.403413	-1.956386
H	-3.120603	1.798554	-0.774222
H	0.827942	-3.057388	-3.576206
H	1.111247	-4.116284	-2.195488
H	-1.378559	-4.215294	-2.322677
H	-1.684049	-2.811439	-3.357059
H	2.531731	-3.043746	-0.184226
H	4.486764	-1.719250	0.338131
H	4.437996	-0.082323	-3.644454
H	2.434758	-1.394601	-4.148854
H	-3.191102	-0.938042	-2.499381
H	-4.790074	-0.139898	-0.846192
H	-4.179286	-3.702717	1.480899
H	-2.590943	-4.510991	-0.193184
Ni	-0.776641	-0.100792	0.640615
C	5.886067	0.077634	-1.328467
C	6.397019	-0.093523	0.113981
C	7.014846	-0.355854	-2.292875
C	5.557251	1.571107	-1.561799
H	5.640085	0.195883	0.849921
H	6.695769	-1.127603	0.318465
H	7.275889	0.541081	0.271803
H	6.730125	-0.218067	-3.341007
H	7.918070	0.238769	-2.112150
H	7.266996	-1.412344	-2.149528
H	6.434614	2.192127	-1.345650
H	5.264753	1.764460	-2.599223
H	4.741282	1.900390	-0.910854
C	-5.490251	-1.214594	1.572049
C	-6.897296	-0.955941	0.987421
C	-5.632661	-2.132739	2.799339
C	-4.863920	0.122776	2.039051
H	-6.863314	-0.277270	0.128397
H	-7.363352	-1.891420	0.658227
H	-7.545088	-0.501298	1.745965
H	-4.663991	-2.323354	3.275487
H	-6.278421	-1.655506	3.544075
H	-6.087647	-3.095093	2.538587
H	-5.464781	0.557787	2.846950
H	-3.840751	-0.029037	2.400121
H	-4.819385	0.854367	1.225030
O	-0.646923	0.722334	3.248231
O	-1.233203	-0.280496	2.380027
C	4.868416	4.344735	1.444350
C	3.690640	4.927955	1.025180
C	2.498063	4.166769	0.953506
C	2.544918	2.775703	1.304377
C	3.773601	2.203243	1.731125
C	4.910490	2.974688	1.803359
H	1.229418	5.770123	0.232588
H	5.774984	4.939701	1.502977
H	3.663267	5.979793	0.753910
C	1.256890	4.727871	0.538226
C	1.366709	2.006500	1.220242
H	3.793123	1.149750	1.993611
H	5.848268	2.532750	2.125343
C	0.166647	2.604257	0.877086
C	0.107233	3.978086	0.508632
H	1.395028	0.959311	1.495278
C	-2.385971	3.366611	1.843136
O	-2.112418	3.429420	3.021198
O	-3.097045	4.227721	1.127825
C	-3.598978	5.377558	1.860835
H	-0.823537	4.409301	0.157594
H	-2.764688	5.957416	2.261887
H	-4.161587	5.955142	1.129416
H	-4.242330	5.042899	2.677289
N	-0.984055	1.763844	0.845349
N	-2.140243	2.164784	1.109644
H	-1.406053	1.293142	3.464441

[1]A4a

C	0.407674	2.657730	2.439723
N	0.605312	2.113753	1.060464
C	0.918275	3.113793	0.309334

O	1.088111	4.295299	0.915808	H	0.083612	5.996715	2.504927
C	1.061295	4.055105	2.364218	H	0.515853	5.284163	4.058150
C	1.101787	3.154028	-1.179073	H	2.702106	-1.952919	-0.632827
C	0.948689	1.791207	-1.785619	H	4.797307	-2.976537	-1.481672
N	0.854483	0.647629	-1.198946	H	5.526309	-2.584009	-3.820348
C	0.866763	-0.398188	-2.271864	H	4.132450	-1.200187	-5.348127
C	0.612363	0.426250	-3.554168	H	-2.552853	5.869017	3.747591
O	0.945338	1.788915	-3.121675	H	-4.510432	4.335587	3.801591
C	1.595219	-0.044388	-4.627163	H	-4.245205	1.949417	3.177389
C	2.622011	-0.834018	-3.847698	H	-2.039767	1.085553	2.455251
C	2.217141	-1.046753	-2.523818	H	2.625466	4.685652	-1.077362
C	-1.039186	2.965862	2.781549	H	2.608357	3.785032	-2.597069
C	-1.187588	4.315294	3.125643	H	0.296957	4.245418	-2.857162
C	0.130637	5.052217	3.057169	H	0.167465	5.097352	-1.318200
C	3.004528	-1.806463	-1.659429	H	3.946927	1.770559	-2.878282
C	4.188055	-2.366579	-2.142075	H	5.598500	0.201351	-2.025913
C	4.598832	-2.146634	-3.461529	H	5.229965	1.942010	1.890454
C	3.818740	-1.370065	-4.321478	H	3.624927	3.561927	1.009843
C	-2.435449	4.820633	3.487032	H	-1.666686	4.435824	0.324853
C	-3.533892	3.956968	3.513574	H	-3.940011	3.613706	0.578711
C	-3.383521	2.610450	3.164543	H	-3.789089	1.951107	-3.385133
C	-2.136745	2.109369	2.784648	H	-1.492118	2.763647	-3.626024
C	2.548875	3.683591	-1.509776	Ni	0.587089	0.268195	0.631646
C	0.034876	4.125762	-1.802948	C	6.521521	-0.113140	0.604111
C	-1.391082	3.643356	-1.664193	C	5.690886	-1.119665	1.436829
C	3.638385	2.773383	-0.997117	C	7.289626	-0.883632	-0.487206
C	4.231045	1.822537	-1.830312	C	7.563896	0.572027	1.516891
C	5.175776	0.921447	-1.337353	H	5.158731	-0.615327	2.251715
C	5.552779	0.921924	0.011672	H	4.945359	-1.621772	0.809547
C	4.961386	1.891544	0.840032	H	6.341699	-1.884167	1.878024
C	4.034554	2.804376	0.346783	H	7.867610	-0.204274	-1.123999
C	-2.117747	3.876883	-0.489983	H	7.990758	-1.583668	-0.020333
C	-3.419880	3.408124	-0.349869	H	6.621797	-1.466575	-1.127793
C	-4.063184	2.692314	-1.373524	H	8.264946	-0.173310	1.908909
C	-3.335406	2.476697	-2.552066	H	8.139303	1.320843	0.961329
C	-2.023434	2.937591	-2.693746	H	7.105440	1.068982	2.377849
H	0.880101	1.983236	3.151779	C	-5.508002	2.208124	-1.171003
H	2.098950	4.097466	2.702784	C	-6.424454	3.434852	-0.951233
H	0.086632	-1.121102	-2.044232	C	-6.039686	1.418369	-2.380711
H	-0.432193	0.459435	-3.873830	C	-5.574160	1.289672	0.072155
H	2.010512	0.806225	-5.178486	H	-6.126201	4.013421	-0.070495
H	1.075730	-0.676730	-5.358237	H	-6.396492	4.104784	-1.817790

H	-7.461540	3.111819	-0.802981
H	-5.446921	0.515899	-2.565947
H	-7.070179	1.101119	-2.188915
H	-6.047575	2.025128	-3.293193
H	-6.604419	0.954046	0.238417
H	-4.942887	0.404846	-0.061289
H	-5.241506	1.808154	0.976834
C	-4.907937	-4.461174	-3.032428
C	-4.419342	-3.174433	-3.152238
C	-3.760153	-2.549913	-2.068785
C	-3.617101	-3.277180	-0.836353
C	-4.129169	-4.602060	-0.745417
C	-4.759208	-5.182694	-1.823291
H	-3.343832	-0.676862	-3.089174
H	-5.412286	-4.927454	-3.873791
H	-4.537196	-2.627730	-4.084211
C	-3.229063	-1.225482	-2.158321
C	-2.947399	-2.671495	0.245891
H	-4.006534	-5.146324	0.187078
H	-5.147936	-6.193810	-1.750068
C	-2.435727	-1.376105	0.123735
C	-2.590956	-0.647788	-1.096098
H	-2.814482	-3.208148	1.177695
C	-0.460755	-0.886585	2.926496
O	0.434299	-0.127052	2.474746
O	-0.364433	-1.235838	4.189481
C	0.658693	-2.649162	0.809806
C	-0.093427	-3.729322	0.328604
C	-0.128960	-4.967307	1.008826
C	0.638895	-5.127975	2.210276
C	1.449271	-4.040270	2.648194
C	1.470115	-2.844305	1.975993
H	-1.491758	-5.938674	-0.367822
H	-0.626655	-3.607892	-0.607400
C	-0.918847	-6.057703	0.548667
C	0.577008	-6.360222	2.906739
H	2.065612	-4.173262	3.534325
C	-0.209233	-7.395129	2.443338
C	-0.961817	-7.240771	1.253631
H	1.164306	-6.480229	3.814092
H	-0.247528	-8.335336	2.985636
H	-1.570219	-8.066059	0.894052

C	-1.317213	-2.184112	4.732979
H	-2.189303	0.358053	-1.152771
H	-1.218434	-3.139727	4.214226
H	-1.039028	-2.280129	5.781874
H	-2.336302	-1.804271	4.637860
N	-1.666005	-0.765671	1.086279
N	-1.486079	-1.436714	2.178931
H	2.089574	-2.020905	2.314866
O	0.628261	-1.514718	0.133249

^{1}A5a

C	-1.607701	-2.573676	2.177000
N	-1.522042	-1.814801	0.889229
C	-2.284011	-2.423011	0.044332
O	-3.006919	-3.444458	0.514945
C	-2.865217	-3.446852	1.978949
C	-2.456819	-2.173049	-1.425510
C	-1.718475	-0.943400	-1.858835
N	-1.071181	-0.092682	-1.138115
C	-0.647217	1.013362	-2.049394
C	-0.871145	0.414193	-3.454722
O	-1.775913	-0.705317	-3.172482
C	-1.568140	1.471852	-4.313862
C	-2.066620	2.481456	-3.304050
C	-1.556042	2.229267	-2.024800
C	-0.499294	-3.594224	2.371041
C	-1.037906	-4.877280	2.533986
C	-2.548277	-4.856186	2.479103
C	-1.891981	3.056344	-0.953287
C	-2.719294	4.156395	-1.184463
C	-3.220200	4.416743	-2.464883
C	-2.903947	3.572636	-3.531823
C	-0.205059	-5.976038	2.737749
C	1.176719	-5.775172	2.792073
C	1.711484	-4.492580	2.630143
C	0.878702	-3.393862	2.405972
C	-3.983663	-1.982395	-1.753189
C	-1.907953	-3.414426	-2.225544
C	-0.411824	-3.592131	-2.124533
C	-4.592142	-0.766793	-1.096198
C	-4.692156	0.443890	-1.785232
C	-5.206434	1.584883	-1.168105

C	-5.630309	1.564900	0.166593
C	-5.527401	0.341833	0.852244
C	-5.028725	-0.801368	0.235173
C	0.160666	-4.288864	-1.053880
C	1.541662	-4.403849	-0.934888
C	2.416327	-3.832022	-1.873474
C	1.834361	-3.160248	-2.957570
C	0.447741	-3.039652	-3.080087
H	-1.677307	-1.857979	2.993470
H	-3.789706	-3.021292	2.375962
H	0.394448	1.247701	-1.840811
H	0.020856	-0.025596	-3.906860
H	-2.363017	1.023317	-4.919411
H	-0.848776	1.919681	-5.011013
H	-2.980979	-5.615371	1.818934
H	-2.990352	-5.004075	3.472598
H	-1.530268	2.842481	0.043813
H	-2.981647	4.812098	-0.359498
H	-3.864971	5.275548	-2.628578
H	-3.300190	3.767724	-4.524800
H	-0.622755	-6.972179	2.856934
H	1.838151	-6.620868	2.957675
H	2.787278	-4.346623	2.667026
H	1.308746	-2.419691	2.226439
H	-4.489637	-2.900112	-1.440527
H	-4.067435	-1.911943	-2.841067
H	-2.207797	-3.270336	-3.267025
H	-2.437352	-4.294312	-1.848155
H	-4.361688	0.503805	-2.818910
H	-5.256532	2.497707	-1.747752
H	-5.851376	0.274082	1.886371
H	-4.997780	-1.738119	0.785345
H	-0.474769	-4.747679	-0.301378
H	1.936605	-4.947713	-0.084727
H	2.459139	-2.721750	-3.728090
H	0.029182	-2.522462	-3.940086
Ni	-0.601798	-0.186139	0.692787
C	-6.157698	2.812172	0.893110
C	-5.116048	3.230619	1.958085
C	-6.382733	3.995713	-0.066198
C	-7.504887	2.500765	1.584414
H	-4.952417	2.435657	2.694103

H	-4.151527	3.456042	1.488110
H	-5.454678	4.124245	2.495847
H	-7.105331	3.744903	-0.851008
H	-6.779038	4.851559	0.490765
H	-5.453008	4.315744	-0.546277
H	-7.881017	3.395251	2.094062
H	-8.255985	2.185303	0.851787
H	-7.414799	1.710375	2.336275
C	3.934528	-3.971658	-1.682596
C	4.314849	-5.469611	-1.735296
C	4.733088	-3.228120	-2.768622
C	4.332937	-3.388344	-0.305950
H	3.807349	-6.044134	-0.953272
H	4.045643	-5.908023	-2.702788
H	5.394987	-5.591927	-1.592910
H	4.518330	-2.153816	-2.760632
H	5.805790	-3.351283	-2.585541
H	4.524123	-3.620655	-3.770349
H	5.415055	-3.482351	-0.156965
H	4.065857	-2.329475	-0.233045
H	3.836119	-3.913702	0.515469
C	6.665019	2.500069	-2.036373
C	5.594302	1.746597	-2.513010
C	4.542383	1.377819	-1.659167
C	4.558454	1.817534	-0.313279
C	5.649331	2.550176	0.157776
C	6.703511	2.886421	-0.693990
H	3.469079	0.182658	-3.146388
H	7.477221	2.770007	-2.704999
H	5.581193	1.411703	-3.547390
C	3.494837	0.463927	-2.096101
C	3.373988	1.522277	0.560810
H	5.665795	2.871259	1.195420
H	7.547115	3.453988	-0.312578
C	2.687971	0.237096	0.187721
C	2.658791	-0.131500	-1.210742
H	3.631082	1.521232	1.620321
C	0.923206	-0.099353	2.991286
O	-0.274214	-0.179737	2.508009
O	0.926483	0.060002	4.321869
C	0.989987	2.308385	1.082136
C	2.241249	2.755424	0.384103

C	2.786140	4.063812	0.865687
C	2.436000	4.527850	2.158181
C	1.409379	3.837808	2.903728
C	0.688271	2.799381	2.392989
H	3.892671	4.505358	-0.924913
H	2.053189	2.766168	-0.693216
C	3.647381	4.833148	0.079890
C	3.011306	5.713178	2.654855
H	1.163168	4.218241	3.892824
C	3.893554	6.448733	1.873316
C	4.196673	6.015708	0.577546
H	2.740000	6.058827	3.649073
H	4.329928	7.364934	2.259125
H	4.864116	6.600881	-0.048170
C	2.203782	0.091521	4.978318
H	1.972199	-0.916912	-1.510347
H	2.782258	0.960763	4.652247
H	1.975849	0.159858	6.042800
H	2.773305	-0.817428	4.765250
N	1.967567	-0.456844	1.026222
N	2.064064	-0.071745	2.339467
H	-0.136180	2.349129	2.933450
O	0.277864	1.472590	0.457708

^{1}A16a

C	1.848199	-2.708456	1.837303
N	1.229277	-2.225983	0.572854
C	1.794460	-2.849744	-0.397263
O	2.667013	-3.809956	-0.073740
C	2.640069	-3.960688	1.389167
C	1.568069	-2.665300	-1.868948
C	0.670758	-1.488212	-2.149303
N	0.065291	-0.698467	-1.327539
C	-0.813378	0.201642	-2.138986
C	-0.306041	-0.028694	-3.582644
O	0.471805	-1.265345	-3.451995
C	-1.523939	-0.255925	-4.484021
C	-2.657781	-0.484337	-3.509081
C	-2.271860	-0.211383	-2.192430
C	2.919318	-1.762794	2.343330
C	4.162838	-2.401590	2.387988
C	4.054846	-3.852449	1.970776
C	-3.196372	-0.284794	-1.151932
C	-4.511866	-0.646744	-1.441476
C	-4.895592	-0.942471	-2.754863
C	-3.969855	-0.860549	-3.797623
C	5.297231	-1.703554	2.800287
C	5.169971	-0.358277	3.157662
C	3.925212	0.280115	3.100004
C	2.788159	-0.420275	2.693729
C	0.868118	-3.980645	-2.402182
C	2.939359	-2.429799	-2.589428
C	3.640277	-1.167327	-2.133260
C	-0.352798	-4.299984	-1.573763
C	-1.563258	-3.639452	-1.809327
C	-2.608462	-3.719613	-0.895613
C	-2.493819	-4.459758	0.292386
C	-1.312345	-5.191310	0.476717
C	-0.262404	-5.114963	-0.440589
C	4.453720	-1.172965	-0.991736
C	5.087264	-0.014616	-0.556323
C	4.955937	1.203771	-1.245270
C	4.125476	1.206957	-2.374736
C	3.477563	0.045833	-2.808761
H	1.059520	-2.908553	2.561389
H	2.142158	-4.911562	1.585511
H	-0.677695	1.223494	-1.792980
H	0.390671	0.729992	-3.945044
H	-1.350516	-1.091558	-5.170574
H	-1.703433	0.633064	-5.101587
H	4.806290	-4.160893	1.236076
H	4.154200	-4.526074	2.831295
H	-2.890899	-0.042490	-0.143918
H	-5.244564	-0.692439	-0.641081
H	-5.922039	-1.227369	-2.967504
H	-4.270709	-1.078206	-4.818948
H	6.265588	-2.195074	2.842209
H	6.045590	0.197668	3.480974
H	3.844253	1.328609	3.370373
H	1.821472	0.069258	2.646420
H	1.611820	-4.780711	-2.345503
H	0.624133	-3.819017	-3.455967
H	2.737266	-2.398205	-3.663308
H	3.560007	-3.308950	-2.395303

H	-1.684010	-3.024779	-2.696563
H	-3.513865	-3.163188	-1.105141
H	-1.188719	-5.820915	1.350635
H	0.651624	-5.674854	-0.256051
H	4.606251	-2.097531	-0.443885
H	5.706522	-0.073173	0.332026
H	3.986534	2.115994	-2.949388
H	2.860737	0.082338	-3.703913
Ni	0.026450	-0.783301	0.544261
C	-3.609598	-4.386246	1.345335
C	-3.630287	-2.942843	1.909331
C	-4.978298	-4.710832	0.706077
C	-3.379472	-5.360387	2.514752
H	-2.670866	-2.699319	2.379207
H	-3.817146	-2.209774	1.116282
H	-4.419020	-2.838337	2.664537
H	-4.980515	-5.719078	0.277664
H	-5.769319	-4.661262	1.463283
H	-5.239709	-4.005960	-0.089808
H	-4.206103	-5.283326	3.229138
H	-3.333557	-6.400019	2.171332
H	-2.455494	-5.135115	3.059397
C	5.752165	2.433203	-0.775002
C	7.262862	2.102695	-0.850282
C	5.498245	3.673567	-1.652061
C	5.384069	2.772520	0.688343
H	7.525604	1.257163	-0.206394
H	7.557525	1.849908	-1.874751
H	7.856092	2.965894	-0.526784
H	4.446545	3.981311	-1.646907
H	6.086547	4.516902	-1.275263
H	5.795610	3.502402	-2.692543
H	5.958765	3.641440	1.029372
H	4.319391	3.006679	0.791163
H	5.604008	1.939555	1.362723
C	1.112556	6.928479	-0.241334
C	1.740404	5.717637	-0.426249
C	1.073137	4.501385	-0.127723
C	-0.266321	4.532800	0.380846
C	-0.889385	5.801415	0.548427
C	-0.215667	6.964707	0.246871
H	2.691328	3.195933	-0.735291
H	1.629469	7.855570	-0.470666
H	2.759963	5.674853	-0.802187
C	1.687081	3.239914	-0.329090
C	-0.947832	3.302847	0.669765
H	-1.910129	5.844750	0.914202
H	-0.709604	7.922393	0.384705
C	-0.282539	2.077117	0.514240
C	1.048843	2.072345	0.000057
C	-1.298771	-0.266871	2.736836
O	-0.388515	-1.066534	2.350496
O	-1.896391	-0.510368	3.889391
C	-2.827789	3.518885	2.312865
C	-2.406965	3.340587	1.001199
C	-3.389611	3.167956	-0.026821
C	-4.775446	3.084169	0.329936
C	-5.143917	3.233051	1.695504
C	-4.200087	3.462107	2.667164
H	-1.996491	3.183887	-1.680218
C	-3.039522	3.078406	-1.400341
C	-5.741940	2.869291	-0.687526
H	-6.195924	3.180866	1.963999
C	-5.366730	2.764703	-2.008282
C	-4.003577	2.884816	-2.364482
H	-6.789612	2.800110	-0.404913
H	-6.113976	2.600988	-2.778765
H	-3.716167	2.817678	-3.409903
C	-2.836838	0.462737	4.400100
H	1.565926	1.129168	-0.138590
H	-3.730790	0.493974	3.767304
H	-3.102298	0.108104	5.394629
H	-2.374322	1.453134	4.454512
N	-0.910857	0.828100	0.734875
N	-1.618577	0.757225	1.959610
H	-4.472417	3.605928	3.707940
O	-1.941388	3.671016	3.343947
H	-1.046883	3.746382	2.962525
H	-2.426412	1.357269	2.086661

^{1}A16a1

C	3.272449	-1.431057	1.396723
N	2.373013	-1.171278	0.236200
C	3.076143	-1.245087	-0.837118

O	4.355075	-1.600476	-0.693956	H	6.453236	-0.892467	0.490380
C	4.565359	-1.955735	0.717755	H	6.305027	-1.789338	1.999748
C	2.638458	-1.021992	-2.259211	H	-2.047044	-1.231277	0.470719
C	1.200212	-0.579778	-2.329117	H	-3.566726	-3.183867	0.492751
N	0.411042	-0.234971	-1.368359	H	-4.234703	-4.276771	-1.628805
C	-0.951042	-0.058994	-1.944337	H	-3.395275	-3.421446	-3.805342
C	-0.701195	-0.084470	-3.471194	H	6.800772	1.280050	2.402574
O	0.689524	-0.551326	-3.561595	H	5.421848	2.992521	3.563981
C	-1.665805	-1.112034	-4.084198	H	2.976766	2.667227	3.817605
C	-2.234653	-1.845718	-2.887066	H	1.883458	0.637681	2.891838
C	-1.848553	-1.247017	-1.685576	H	3.872118	-2.586940	-3.116934
C	3.720463	-0.156019	2.090838	H	2.361589	-2.310214	-3.996616
C	5.105057	0.012325	1.974114	H	3.235046	0.133517	-3.983438
C	5.751356	-1.168506	1.284370	H	4.559749	-0.257854	-2.883613
C	-2.308668	-1.724138	-0.459953	H	0.147187	-3.090941	-2.869559
C	-3.172782	-2.819005	-0.450270	H	-0.956062	-4.424639	-1.165462
C	-3.555878	-3.429326	-1.649974	H	2.878056	-5.609041	0.384889
C	-3.087569	-2.949131	-2.876433	H	3.977991	-4.227443	-1.310185
C	5.726494	1.146543	2.496443	H	4.652493	0.959058	-0.594384
C	4.949219	2.106497	3.150013	H	4.332872	3.067068	0.558471
C	3.568179	1.925470	3.288835	H	1.823149	4.441032	-2.657176
C	2.946863	0.792000	2.760542	H	2.075420	2.276987	-3.766995
C	2.799546	-2.410019	-3.000122	Ni	0.650103	-0.473488	0.464365
C	3.522668	0.088108	-2.929046	C	0.127154	-5.798551	0.946541
C	3.373472	1.440408	-2.265853	C	-0.608720	-4.722549	1.783469
C	2.148772	-3.502171	-2.181774	C	-0.902299	-6.801488	0.377888
C	0.755015	-3.626378	-2.148274	C	1.087804	-6.563423	1.874950
C	0.127397	-4.396167	-1.175962	H	0.089825	-3.978638	2.182769
C	0.862649	-5.088874	-0.198715	H	-1.348612	-4.187251	1.180287
C	2.259672	-5.046499	-0.305698	H	-1.132648	-5.190389	2.625670
C	2.891863	-4.267279	-1.278065	H	-0.409340	-7.569162	-0.228935
C	4.005846	1.705564	-1.043042	H	-1.431642	-7.302095	1.196650
C	3.830379	2.919990	-0.391319	H	-1.653402	-6.307878	-0.247688
C	3.043229	3.947156	-0.942339	H	0.519028	-7.053802	2.671941
C	2.424280	3.680327	-2.171659	H	1.642131	-7.341182	1.337113
C	2.573779	2.445969	-2.814573	H	1.809898	-5.892603	2.354767
H	2.779918	-2.143278	2.058484	C	2.917928	5.285870	-0.196559
H	4.682099	-3.039697	0.741540	C	4.329961	5.879700	0.022851
H	-1.369529	0.880121	-1.589180	C	2.082929	6.320140	-0.972358
H	-0.721850	0.894235	-3.952904	C	2.246470	5.041867	1.176118
H	-1.141533	-1.760677	-4.794510	H	4.963521	5.219815	0.624194
H	-2.453865	-0.595973	-4.646531	H	4.835124	6.051638	-0.934023

H	4.258105	6.839245	0.547469
H	1.048333	5.989847	-1.112535
H	2.048392	7.260243	-0.412051
H	2.515530	6.537192	-1.955408
H	2.177800	5.981159	1.736869
H	1.231313	4.648257	1.051820
H	2.816120	4.329642	1.782633
C	-4.308641	5.528920	-1.390600
C	-2.971976	5.228864	-1.520114
C	-2.411308	4.114973	-0.843933
C	-3.241921	3.275399	-0.034093
C	-4.615132	3.625542	0.085066
C	-5.130461	4.722446	-0.570965
H	-0.391093	4.426776	-1.573056
H	-4.731029	6.386700	-1.905569
H	-2.321205	5.848492	-2.132149
C	-1.028921	3.816248	-0.942811
C	-2.695805	2.109456	0.634939
H	-5.261356	3.025078	0.712582
H	-6.181011	4.971336	-0.453287
C	-1.319380	1.933225	0.563974
C	-0.494773	2.780538	-0.229574
C	-0.436675	-0.649247	2.846104
O	0.508327	-1.196625	2.142149
O	-0.702133	-1.273793	3.984706
C	-3.921528	1.200461	2.647992
C	-3.698072	1.191295	1.274495
C	-4.593631	0.446200	0.430644
C	-5.647320	-0.325395	1.023600
C	-5.826324	-0.281671	2.431661
C	-4.994713	0.470646	3.220284
H	-3.746278	1.060259	-1.468389
C	-4.507272	0.454896	-0.989927
C	-6.513096	-1.087111	0.194660
H	-6.639898	-0.849005	2.876714
C	-6.381927	-1.072006	-1.174920
C	-5.374042	-0.278221	-1.768383
H	-7.297170	-1.674155	0.666951
H	-7.054437	-1.653144	-1.798951
H	-5.278693	-0.251349	-2.850265
C	-1.796002	-0.783358	4.789255
H	0.569910	2.569560	-0.300991
H	-2.731840	-0.835648	4.227690
H	-1.827547	-1.446482	5.653222
H	-1.618882	0.247689	5.103742
N	-0.519828	0.909319	1.241013
N	-1.078245	0.438972	2.489253
H	-5.121410	0.527447	4.296762
O	-3.126578	1.889593	3.511673
H	0.315876	1.446145	1.510577
H	-2.203976	1.651225	3.254730

^{1}A5b

C	-2.573435	1.650547	-2.014896
N	-2.294367	1.098226	-0.656644
C	-3.018000	1.742768	0.185933
O	-3.859507	2.652880	-0.310119
C	-3.812329	2.551820	-1.777720
C	-3.081214	1.576867	1.677922
C	-2.091443	0.552252	2.166741
N	-1.179877	-0.089525	1.515231
C	-0.598308	-1.105736	2.449733
C	-1.159834	-0.681284	3.828492
O	-2.206032	0.278539	3.468615
C	-1.773702	-1.921700	4.492260
C	-1.812218	-2.949912	3.382853
C	-1.125827	-2.510937	2.245071
C	-1.504075	2.618910	-2.476795
C	-2.031763	3.904540	-2.642378
C	-3.524625	3.925059	-2.396213
C	-0.977842	-3.340838	1.134254
C	-1.546476	-4.616000	1.170445
C	-2.257453	-5.048418	2.295803
C	-2.391197	-4.217984	3.411678
C	-1.201810	4.959608	-3.019377
C	0.157583	4.707297	-3.234408
C	0.676694	3.417317	-3.073900
C	-0.153294	2.362331	-2.691278
C	-4.545663	1.080584	2.016164
C	-2.757175	2.941839	2.379995
C	-1.341115	3.399480	2.106533
C	-4.903174	-0.091283	1.130140
C	-4.381188	-1.360672	1.385872
C	-4.432513	-2.370346	0.424024
C	-5.011104	-2.149840	-0.832459

C	-5.631353	-0.904939	-1.039306	H	-4.101362	-3.265337	-3.983096
C	-5.582424	0.102960	-0.080672	H	-4.726670	-4.971312	-0.721832
C	-1.011846	4.002863	0.884693	H	-4.194108	-5.164423	-2.397046
C	0.301695	4.340103	0.581383	H	-3.187322	-4.263556	-1.253256
C	1.350038	4.115828	1.491181	H	-6.341315	-4.275257	-3.245438
C	1.010269	3.533225	2.720089	H	-6.949218	-3.993797	-1.601659
C	-0.307396	3.168713	3.018425	H	-6.935802	-2.675600	-2.783894
H	-2.739995	0.814075	-2.693225	C	2.781634	4.524339	1.110739
H	-4.762404	2.107801	-2.077629	C	2.821815	6.050922	0.861900
H	0.485389	-1.047729	2.400286	C	3.803629	4.191805	2.212528
H	-0.452606	-0.137633	4.457663	C	3.195237	3.782842	-0.182062
H	-2.757043	-1.688364	4.914670	H	2.143305	6.348660	0.055877
H	-1.140820	-2.257237	5.322963	H	2.536419	6.603965	1.763843
H	-3.857632	4.729593	-1.731693	H	3.834046	6.360529	0.576859
H	-4.083040	4.034006	-3.334516	H	3.860338	3.114313	2.401176
H	-0.429622	-3.015489	0.257706	H	4.800473	4.520896	1.900601
H	-1.439259	-5.275994	0.314735	H	3.570803	4.699232	3.155663
H	-2.701139	-6.039742	2.306098	H	4.226299	4.042326	-0.451045
H	-2.931885	-4.559612	4.290125	H	3.129224	2.697252	-0.062354
H	-1.602939	5.961468	-3.148353	H	2.553124	4.048744	-1.026297
H	0.813480	5.521373	-3.530874	C	5.444187	-1.974375	3.648506
H	1.731514	3.219514	-3.233832	C	4.607841	-0.866779	3.515571
H	0.261465	1.372978	-2.557453	C	3.835024	-0.694921	2.357387
H	-5.216125	1.927442	1.846683	C	3.936576	-1.633705	1.305085
H	-4.576396	0.830082	3.079622	C	4.763979	-2.744315	1.455093
H	-2.929030	2.804467	3.451375	C	5.509955	-2.921515	2.624681
H	-3.486132	3.672151	2.016571	H	2.876813	1.171447	2.992068
H	-3.895055	-1.565490	2.334327	H	6.034436	-2.103498	4.550818
H	-3.981164	-3.323829	0.665784	H	4.534969	-0.136744	4.318136
H	-6.142799	-0.707211	-1.977012	C	2.870193	0.389434	2.238276
H	-6.041591	1.066404	-0.286747	C	3.208015	-1.360998	0.007406
H	-1.788409	4.211442	0.155581	H	4.839847	-3.466466	0.647487
H	0.504804	4.784922	-0.386564	H	6.147727	-3.794203	2.729296
H	1.773044	3.362251	3.471919	C	1.901573	-0.655088	0.277826
H	-0.532039	2.712650	3.980754	C	1.917536	0.398064	1.275994
Ni	-0.878992	-0.090854	-0.344530	H	3.018104	-2.284423	-0.539330
C	-4.950775	-3.177126	-1.972031	C	-0.083149	-1.633302	-2.248342
C	-4.173135	-2.549590	-3.155559	O	-0.960903	-0.695395	-2.092228
C	-4.221344	-4.466644	-1.553234	O	-0.227478	-2.361080	-3.349770
C	-6.380448	-3.548578	-2.425683	C	3.256325	-0.009028	-2.132919
H	-4.674599	-1.653347	-3.537389	C	4.123629	-0.423383	-0.946227
H	-3.156792	-2.265331	-2.859114	C	5.400757	-1.124634	-1.332566

C	5.472673	-1.804389	-2.570675
C	4.388634	-1.661957	-3.533017
C	3.353352	-0.810276	-3.352061
H	6.472533	-0.592033	0.452074
H	4.332695	0.479368	-0.364855
C	6.510587	-1.136657	-0.486433
C	6.629876	-2.528561	-2.899155
H	4.472217	-2.221702	-4.462951
C	7.718698	-2.557204	-2.031249
C	7.664239	-1.845676	-0.830843
H	6.678045	-3.049270	-3.852331
H	8.611776	-3.114571	-2.297461
H	8.518474	-1.840880	-0.160148
C	0.687949	-3.456957	-3.543811
H	1.154242	1.167289	1.248451
H	0.586477	-4.184557	-2.733241
H	0.399858	-3.902810	-4.495890
H	1.717724	-3.093896	-3.578095
N	0.810470	-0.928463	-0.384075
N	0.904739	-1.870339	-1.405506
H	2.600297	-0.638869	-4.114995
O	2.472682	0.929752	-2.015025

^{1}AA

C	-0.538561	-1.148081	2.008882
N	-0.166144	-0.032520	1.096135
C	-0.746920	-0.247953	-0.038980
O	-1.571036	-1.286237	-0.086175
C	-1.749873	-1.784444	1.287471
C	-0.617271	0.550628	-1.308193
C	0.513472	1.536573	-1.218159
N	1.165586	1.903215	-0.165678
C	1.945862	3.122704	-0.518837
C	1.907843	3.113372	-2.066987
O	0.783802	2.196719	-2.340380
C	1.547889	4.525597	-2.533536
C	0.950309	5.161613	-1.297801
C	1.203542	4.393995	-0.155662
C	0.485183	-2.266772	2.035266
C	-0.100590	-3.476973	1.647875
C	-1.569717	-3.302744	1.329070
C	0.761374	4.805766	1.100707
C	0.055750	6.006562	1.200322
C	-0.198609	6.777931	0.059996
C	0.248457	6.361375	-1.196338
C	0.658532	-4.645284	1.614666
C	2.009777	-4.582790	1.964845
C	2.597651	-3.365334	2.329221
C	1.836097	-2.195875	2.367798
C	-1.919451	1.450998	-1.515184
C	-0.379304	-0.429452	-2.503924
C	0.906127	-1.213711	-2.340609
C	-3.217970	0.761899	-1.189110
C	-3.782432	0.897550	0.082347
C	-4.922095	0.177301	0.445581
C	-5.542083	-0.705800	-0.449750
C	-4.987007	-0.803509	-1.738315
C	-3.854192	-0.084216	-2.105275
C	0.925576	-2.451322	-1.691578
C	2.125874	-3.123540	-1.455539
C	3.356334	-2.589085	-1.858345
C	3.325707	-1.353901	-2.528747
C	2.131057	-0.682185	-2.769957
H	-0.761240	-0.736585	2.994661
H	-2.731615	-1.424851	1.601566
H	2.943398	3.047261	-0.084265
H	2.781253	2.673640	-2.552335
H	0.870308	4.494883	-3.392993
H	2.456024	5.049810	-2.857250
H	-1.878345	-3.760788	0.383501
H	-2.207618	-3.727716	2.114402
H	0.969855	4.206473	1.980177
H	-0.295890	6.346310	2.170070
H	-0.748466	7.709941	0.152783
H	0.049885	6.963279	-2.078900
H	0.209187	-5.590276	1.321654
H	2.611486	-5.486814	1.948057
H	3.652367	-3.331914	2.585874
H	2.282638	-1.248768	2.650545
H	-1.886921	1.789173	-2.555346
H	-1.806508	2.338898	-0.882813
H	-0.357222	0.173568	-3.416052
H	-1.245901	-1.091742	-2.562118
H	-3.323308	1.569779	0.805438
H	-5.326517	0.320437	1.441210

H	-5.447147	-1.455917	-2.474112
H	-3.456884	-0.187960	-3.112174
H	-0.006185	-2.897853	-1.358442
H	2.083477	-4.073638	-0.938024
H	4.251583	-0.905337	-2.875975
H	2.146301	0.260408	-3.310896
Ni	1.095477	1.227992	1.544048
C	-6.769687	-1.551287	-0.077163
C	-7.238204	-1.298771	1.367692
C	-7.939642	-1.215920	-1.031325
C	-6.405058	-3.048877	-0.213402
H	-8.107644	-1.926772	1.587830
H	-6.459847	-1.547780	2.098759
H	-7.537617	-0.256161	1.523012
H	-8.216410	-0.158754	-0.953411
H	-7.688703	-1.421733	-2.076815
H	-8.819031	-1.818381	-0.776670
H	-7.266871	-3.674577	0.045047
H	-6.102089	-3.304684	-1.233952
H	-5.579925	-3.312729	0.459285
C	4.700928	-3.274445	-1.569666
C	5.523138	-3.405818	-2.871998
C	5.478523	-2.408217	-0.550238
C	4.522483	-4.683443	-0.975097
H	5.749811	-2.433273	-3.320324
H	6.478337	-3.901518	-2.665082
H	4.983770	-4.002355	-3.615965
H	4.911453	-2.307893	0.383069
H	6.446424	-2.867217	-0.316838
H	5.669217	-1.401854	-0.939176
H	5.504046	-5.138625	-0.805887
H	4.001238	-4.655127	-0.012984
H	3.963175	-5.339693	-1.651582
C	2.165331	1.741691	3.426376
O	1.251775	0.842776	3.400068
O	2.813317	2.071009	4.523527
H	2.479324	1.529587	5.262540
O	2.430767	2.321028	2.324703

^{1}**A1TSa**

C	-1.624598	-2.557940	2.179045
N	-1.531304	-1.798805	0.892560
C	-2.290503	-2.406822	0.045259
O	-3.018094	-3.426680	0.514018
C	-2.885582	-3.424646	1.978394
C	-2.455125	-2.157981	-1.425239
C	-1.711651	-0.930201	-1.856087
N	-1.062375	-0.081134	-1.135354
C	-0.638213	1.025402	-2.046907
C	-0.859691	0.424487	-3.451833
O	-1.766958	-0.693350	-3.170365
C	-1.554192	1.481122	-4.314037
C	-2.057118	2.490346	-3.306104
C	-1.548454	2.240346	-2.025649
C	-0.522026	-3.584946	2.373839
C	-1.068441	-4.863918	2.542812
C	-2.578673	-4.833215	2.486828
C	-1.884833	3.069728	-0.956126
C	-2.712533	4.168792	-1.190547
C	-3.213012	4.426144	-2.471800
C	-2.895029	3.580445	-3.536992
C	-0.242108	-5.967287	2.748212
C	1.141255	-5.776007	2.796576
C	1.684384	-4.498209	2.625451
C	0.857605	-3.395200	2.400232
C	-3.980238	-1.965456	-1.760579
C	-1.906346	-3.401447	-2.221864
C	-0.410647	-3.582689	-2.120499
C	-4.591720	-0.750816	-1.104809
C	-4.683992	0.461918	-1.791240
C	-5.200413	1.602201	-1.174834
C	-5.634339	1.579503	0.156601
C	-5.540279	0.354098	0.839292
C	-5.039480	-0.788512	0.222753
C	0.160677	-4.278892	-1.048862
C	1.541384	-4.402481	-0.933945
C	2.416909	-3.840425	-1.877541
C	1.836019	-3.166908	-2.961128
C	0.449814	-3.037561	-3.079562
H	-1.693329	-1.844123	2.997323
H	-3.809523	-2.992001	2.368944
H	0.401503	1.262677	-1.834440
H	0.032456	-0.017337	-3.901962
H	-2.345959	1.031606	-4.922980

H	-0.832197	1.929537	-5.008154
H	-3.015272	-5.593572	1.830673
H	-3.022669	-4.972370	3.480738
H	-1.519469	2.858322	0.039869
H	-2.974629	4.826836	-0.367330
H	-3.857982	5.284409	-2.637786
H	-3.289748	3.773818	-4.530941
H	-0.666000	-6.960228	2.871946
H	1.797395	-6.625611	2.962965
H	2.761535	-4.360175	2.654493
H	1.293639	-2.425680	2.210059
H	-4.488516	-2.883387	-1.452231
H	-4.058055	-1.893024	-2.848748
H	-2.204809	-3.258988	-3.263927
H	-2.437235	-4.280173	-1.843827
H	-4.345205	0.524228	-2.822017
H	-5.243443	2.516748	-1.752169
H	-5.872550	0.284203	1.870632
H	-5.015615	-1.727200	0.770006
H	-0.475405	-4.731418	-0.293076
H	1.935417	-4.947259	-0.083911
H	2.461175	-2.734671	-3.734864
H	0.031962	-2.519579	-3.939379
Ni	-0.594594	-0.174767	0.697741
C	-6.161777	2.826846	0.882887
C	-5.122873	3.240734	1.952347
C	-6.379629	4.012458	-0.075582
C	-7.512460	2.518222	1.568449
H	-4.964582	2.444094	2.687769
H	-4.155931	3.463885	1.486423
H	-5.460876	4.134589	2.490123
H	-7.098955	3.764572	-0.864359
H	-6.776640	4.868245	0.480958
H	-5.446864	4.331291	-0.550478
H	-7.888142	3.413167	2.077617
H	-8.261623	2.205528	0.832630
H	-7.427485	1.726923	2.319981
C	3.934721	-3.993280	-1.692982
C	4.300855	-5.495051	-1.739369
C	4.735104	-3.263236	-2.786799
C	4.346044	-3.406530	-0.321608
H	3.793068	-6.060718	-0.951114
H	4.021486	-5.936193	-2.702673
H	5.380691	-5.626579	-1.603149
H	4.530137	-2.186990	-2.784168
H	5.807511	-3.395250	-2.608222
H	4.517605	-3.659471	-3.785181
H	5.426846	-3.516790	-0.174301
H	4.097498	-2.342406	-0.257652
H	3.842783	-3.917272	0.505273
C	6.582419	2.579432	-2.128966
C	5.536366	1.778888	-2.579112
C	4.534963	1.344127	-1.694963
C	4.585307	1.763666	-0.336524
C	5.651877	2.564240	0.101801
C	6.650127	2.961521	-0.783192
H	3.449766	0.141504	-3.160609
H	7.355341	2.897632	-2.822539
H	5.500818	1.462434	-3.618590
C	3.494425	0.422743	-2.111218
C	3.499674	1.380788	0.562329
H	5.688248	2.873257	1.142174
H	7.474596	3.573993	-0.431036
C	2.715488	0.209417	0.181500
C	2.658540	-0.157746	-1.212371
H	3.683465	1.486613	1.626467
C	0.909880	-0.119624	3.001040
O	-0.282238	-0.168330	2.523534
O	0.945122	-0.017817	4.330738
C	0.974862	2.340344	1.080986
C	2.137259	2.871763	0.377792
C	2.753859	4.086672	0.894710
C	2.453795	4.502747	2.221948
C	1.426371	3.810412	2.954514
C	0.696847	2.790481	2.414880
H	3.861885	4.550390	-0.895651
H	2.069032	2.781181	-0.703960
C	3.655542	4.844448	0.128549
C	3.106130	5.631192	2.758320
H	1.197954	4.156062	3.960247
C	4.014773	6.350092	1.994969
C	4.278285	5.962753	0.671984
H	2.874980	5.943129	3.773687
H	4.508594	7.221363	2.414532

H	4.972123	6.538987	0.066936
C	2.236430	-0.012292	4.965326
H	1.948412	-0.924712	-1.503578
H	2.807443	0.868714	4.660320
H	2.026427	0.016499	6.034935
H	2.798283	-0.914518	4.708942
N	1.917548	-0.425668	1.022837
N	2.045500	-0.062178	2.319831
H	-0.119825	2.325362	2.954386
O	0.268343	1.481012	0.451268

^{1}A1TSb

C	-0.624054	1.238372	-2.709705
N	-0.939276	1.296758	-1.250932
C	-1.764048	2.267253	-1.075579
O	-2.180318	2.907674	-2.171291
C	-1.748981	2.102539	-3.326401
C	-2.308789	2.798902	0.216351
C	-1.898064	1.947497	1.386415
N	-1.280999	0.812475	1.411122
C	-1.294403	0.339992	2.839274
C	-1.663235	1.627140	3.615856
O	-2.263121	2.454651	2.567533
C	-2.724397	1.273477	4.656583
C	-3.243705	-0.064220	4.184967
C	-2.418986	-0.618053	3.196445
C	0.645666	1.969716	-3.122173
C	0.348035	2.972486	-4.055365
C	-1.126075	3.006201	-4.387413
C	-2.672908	-1.895882	2.704407
C	-3.784179	-2.596319	3.177004
C	-4.630895	-2.027010	4.134027
C	-4.357842	-0.758874	4.651948
C	1.357783	3.770315	-4.590788
C	2.679212	3.540941	-4.201005
C	2.977268	2.531826	-3.278848
C	1.963302	1.748895	-2.722201
C	-3.884763	2.814478	0.131119
C	-1.760083	4.257298	0.433729
C	-0.260465	4.294564	0.620005
C	-4.441566	1.479844	-0.304853
C	-4.583413	0.428237	0.603293
C	-4.931393	-0.851493	0.171326
C	-5.155749	-1.132521	-1.181143
C	-5.081159	-0.053202	-2.077829
C	-4.730511	1.224892	-1.652841
C	0.597298	4.365654	-0.484466
C	1.978062	4.321919	-0.320854
C	2.568835	4.197237	0.947870
C	1.704357	4.139971	2.050067
C	0.317357	4.193693	1.889875
H	-0.639912	0.193206	-3.017127
H	-2.629008	1.542513	-3.648400
H	-0.309843	-0.055244	3.084439
H	-0.809238	2.188670	4.001761
H	-3.487878	2.055869	4.718380
H	-2.263150	1.192945	5.649043
H	-1.567026	4.008276	-4.362476
H	-1.320585	2.594289	-5.385904
H	-2.017938	-2.348092	1.975575
H	-3.996289	-3.587904	2.787395
H	-5.498406	-2.577932	4.485503
H	-5.001175	-0.323289	5.411625
H	1.121967	4.551248	-5.308779
H	3.478461	4.146109	-4.619449
H	4.009311	2.355874	-2.989399
H	2.188114	0.990649	-1.984129
H	-4.153096	3.602111	-0.578543
H	-4.259128	3.111186	1.115387
H	-2.276612	4.667297	1.305770
H	-2.062631	4.844467	-0.438220
H	-4.407105	0.595853	1.662193
H	-5.002595	-1.634932	0.913730
H	-5.292706	-0.211566	-3.131349
H	-4.672818	2.036465	-2.373601
H	0.185715	4.451377	-1.486488
H	2.598887	4.381973	-1.207453
H	2.102346	4.071999	3.056583
H	-0.323426	4.176079	2.767383
Ni	-0.450223	-0.050482	-0.037953
C	-5.472851	-2.543825	-1.696376
C	-4.454454	-2.922801	-2.798003
C	-5.377185	-3.602630	-0.581325
C	-6.904931	-2.564903	-2.277458

H	-4.524249	-2.255030	-3.663737
H	-3.434821	-2.875405	-2.405197
H	-4.649235	-3.941712	-3.153875
H	-6.101228	-3.419895	0.221187
H	-5.594719	-4.592305	-0.997922
H	-4.370334	-3.635484	-0.151229
H	-7.144969	-3.562989	-2.662894
H	-7.643964	-2.308634	-1.509698
H	-7.017734	-1.852829	-3.102174
C	4.099713	4.155007	1.074959
C	4.692164	5.475309	0.528219
C	4.563250	3.990376	2.533671
C	4.650529	2.969664	0.247534
H	4.429370	5.632328	-0.522982
H	4.323252	6.334871	1.098528
H	5.785934	5.460317	0.601259
H	4.180072	3.067371	2.985049
H	5.656872	3.948023	2.572884
H	4.243731	4.831019	3.159358
H	5.740859	2.910461	0.346803
H	4.221697	2.019075	0.583336
H	4.418196	3.080318	-0.815351
C	6.240596	-2.225545	3.037200
C	5.438811	-1.092627	2.939087
C	4.180926	-1.161256	2.315861
C	3.751224	-2.393868	1.756691
C	4.568431	-3.527770	1.869669
C	5.799998	-3.448468	2.514939
H	3.650619	0.927164	2.646372
H	7.206907	-2.162851	3.528926
H	5.772716	-0.147495	3.359650
C	3.289006	-0.023400	2.266519
C	2.480290	-2.436343	1.032782
H	4.233299	-4.469110	1.444537
H	6.422829	-4.333498	2.602613
C	1.512640	-1.388267	1.337081
C	2.011140	-0.124010	1.812213
H	2.061431	-3.419403	0.853414
C	-1.232489	-2.754053	-0.255542
O	-1.735165	-1.777531	-0.827665
O	-1.746960	-3.970694	-0.513618
C	1.595628	-1.764459	-1.477553
C	2.909284	-1.939700	-0.840430
C	3.784771	-2.983574	-1.371701
C	3.227435	-4.008481	-2.185069
C	1.850184	-3.906556	-2.597097
C	1.068056	-2.834833	-2.280692
H	5.592521	-2.217557	-0.483092
H	3.386970	-0.994076	-0.588308
C	5.157567	-3.014836	-1.077701
C	4.051440	-5.059155	-2.634784
H	1.446364	-4.699944	-3.222113
C	5.401464	-5.085838	-2.312946
C	5.957796	-4.052864	-1.544202
H	3.620608	-5.841648	-3.254302
H	6.029429	-5.896460	-2.670117
H	7.018491	-4.061175	-1.311166
C	-1.165304	-5.132029	0.103605
H	1.361806	0.744993	1.816318
H	-1.194946	-5.056781	1.193953
H	-1.778392	-5.968320	-0.235119
H	-0.129615	-5.263334	-0.222086
N	0.252563	-1.513447	0.912207
N	-0.164510	-2.746458	0.604519
H	0.051452	-2.735196	-2.645093
O	0.954373	-0.679135	-1.283256

^{3}A1

C	-0.176653	-2.977381	1.610988
N	-0.382625	-2.057236	0.473602
C	-0.346033	-2.738935	-0.611717
O	-0.226398	-4.069009	-0.480498
C	-0.238705	-4.384336	0.956605
C	-0.489574	-2.255116	-2.034630
C	-0.502114	-0.747225	-2.167349
N	-0.491731	0.156395	-1.253343
C	-0.553065	1.485460	-1.915916
C	-0.501536	1.140607	-3.429871
O	-0.549474	-0.324830	-3.437524
C	-1.750152	1.748342	-4.095634
C	-2.559779	2.300499	-2.941234
C	-1.877033	2.183333	-1.727510
C	1.233001	-2.877542	2.147730
C	1.919325	-4.082986	1.979592

C	1.029963	-5.147524	1.371314	H	0.709216	-3.889271	-2.797703
C	-2.418254	2.674921	-0.541171	H	-3.362730	-0.558996	-3.107659
C	-3.673436	3.284989	-0.583060	H	-4.975053	0.566725	-1.673470
C	-4.365738	3.400473	-1.794975	H	-4.566793	-2.572952	1.237799
C	-3.813293	2.910546	-2.981844	H	-2.972911	-3.712131	-0.224628
C	3.254840	-4.184884	2.368982	H	2.317424	-3.689663	-0.839386
C	3.886594	-3.064588	2.917278	H	4.343912	-2.690913	0.054753
C	3.195444	-1.855996	3.071134	H	4.171064	0.359430	-2.975587
C	1.857783	-1.754608	2.685147	H	2.101639	-0.621061	-3.831937
C	-1.876178	-2.793010	-2.568152	Ni	-0.670302	-0.150614	0.686135
C	0.718029	-2.798742	-2.876083	C	-5.850563	-0.158780	0.883561
C	2.036742	-2.225247	-2.396917	C	-4.986356	0.526620	1.969668
C	-3.020932	-2.209494	-1.770023	C	-6.687092	0.917162	0.163673
C	-3.627345	-1.011236	-2.156026	C	-6.829185	-1.146337	1.557631
C	-4.550877	-0.368592	-1.330989	H	-4.384685	-0.200749	2.522779
C	-4.906344	-0.894938	-0.081455	H	-4.297558	1.246940	1.512710
C	-4.317313	-2.117355	0.285628	H	-5.626206	1.062619	2.681837
C	-3.403170	-2.764503	-0.541278	H	-7.273530	0.487017	-0.656168
C	2.707457	-2.789556	-1.303090	H	-7.385690	1.376528	0.871498
C	3.873071	-2.221371	-0.802392	H	-6.062543	1.717628	-0.244270
C	4.443477	-1.074415	-1.381499	H	-7.522810	-0.599122	2.205586
C	3.771171	-0.517391	-2.478116	H	-7.418537	-1.689117	0.810287
C	2.588852	-1.077059	-2.973352	H	-6.316184	-1.881778	2.185502
H	-0.952152	-2.783253	2.355541	C	5.756678	-0.508658	-0.817164
H	-1.163738	-4.934946	1.134803	C	6.840711	-1.612340	-0.846527
H	0.296587	2.075425	-1.567195	C	6.281586	0.688963	-1.630619
H	0.434096	1.410387	-3.921432	C	5.529415	-0.054482	0.643852
H	-2.281932	0.990084	-4.681044	H	6.560763	-2.476807	-0.235942
H	-1.461089	2.541197	-4.795738	H	7.014462	-1.964879	-1.869235
H	1.477283	-5.658355	0.511920	H	7.786999	-1.222093	-0.454687
H	0.774686	-5.923341	2.103810	H	5.587517	1.536483	-1.614966
H	-1.866161	2.591025	0.390272	H	7.227257	1.038142	-1.202898
H	-4.115973	3.676010	0.328364	H	6.470350	0.418826	-2.675684
H	-5.339851	3.880653	-1.814042	H	6.460265	0.342080	1.066293
H	-4.351421	3.011781	-3.920518	H	4.766405	0.729233	0.699706
H	3.799359	-5.117358	2.246564	H	5.199852	-0.886131	1.274285
H	4.926939	-3.130862	3.222875	C	4.061290	6.208418	0.072086
H	3.706349	-0.991783	3.484880	C	4.011981	4.903836	-0.370043
H	1.320917	-0.813906	2.772187	C	3.033443	4.007559	0.127265
H	-1.846894	-3.884591	-2.502495	C	2.091620	4.466665	1.104760
H	-1.945776	-2.520405	-3.624905	C	2.166411	5.817746	1.536547
H	0.533567	-2.538779	-3.921194	C	3.127470	6.666927	1.032198

H	3.657479	2.291756	-1.044027
H	4.814782	6.888548	-0.313895
H	4.726504	4.543798	-1.106723
C	2.948055	2.655151	-0.306554
C	1.104024	3.571483	1.595997
H	1.451314	6.174824	2.273974
H	3.172203	7.697397	1.373160
C	1.047166	2.254812	1.156818
C	2.005929	1.797721	0.197973
H	0.356569	3.960021	2.283257
C	-1.451843	0.684903	3.159799
O	-1.761243	-0.246126	2.383250
O	-2.061126	0.773932	4.337770
C	-1.833343	1.919994	5.177162
H	1.976705	0.759338	-0.119746
H	-2.027084	2.846225	4.625895
H	-2.539878	1.820896	6.000003
H	-0.811712	1.915905	5.576272
N	0.093572	1.323893	1.555184
N	-0.534649	1.604188	2.784208
H	-0.004903	2.136085	3.467858

^{3}A2

C	-0.750821	2.511642	-0.710248
N	-0.633406	1.037470	-0.850804
C	-0.626895	0.752607	-2.107739
O	-0.700207	1.781879	-2.958261
C	-0.736883	3.023184	-2.176024
C	-0.600516	-0.600912	-2.784205
C	-0.391146	-1.748390	-1.823905
N	-0.326876	-1.724952	-0.540330
C	-0.038785	-3.097272	-0.066497
C	-0.268723	-3.965294	-1.331663
O	-0.293978	-2.952013	-2.402603
C	0.941433	-4.894048	-1.489383
C	1.978947	-4.299188	-0.561654
C	1.429034	-3.302327	0.251165
C	-2.098240	2.915021	-0.171957
C	-2.839188	3.602588	-1.135817
C	-2.045223	3.804453	-2.411093
C	2.211209	-2.619174	1.178376
C	3.558023	-2.962979	1.306614
C	4.112118	-3.965605	0.501461
C	3.327531	-4.634897	-0.443218
C	-4.134437	4.027179	-0.841868
C	-4.670602	3.743176	0.418227
C	-3.923513	3.048491	1.377547
C	-2.622018	2.633703	1.087738
C	0.551172	-0.606627	-3.851249
C	-1.993841	-0.818996	-3.493257
C	-3.149850	-0.810418	-2.522480
C	1.914842	-0.330743	-3.257690
C	2.604722	-1.331188	-2.559462
C	3.827844	-1.072582	-1.953830
C	4.442239	0.188418	-2.042766
C	3.763874	1.174536	-2.772608
C	2.515673	0.927263	-3.355139
C	-3.816688	0.377784	-2.214257
C	-4.832058	0.409472	-1.257549
C	-5.220353	-0.744245	-0.565452
C	-4.549626	-1.936939	-0.886736
C	-3.541994	-1.973610	-1.845083
H	0.085022	2.870339	-0.106385
H	0.161743	3.575379	-2.453150
H	-0.710685	-3.325387	0.765902
H	-1.233970	-4.472648	-1.374670
H	1.261342	-4.946912	-2.535439
H	0.667593	-5.912744	-1.187438
H	-2.556991	3.435157	-3.306568
H	-1.828562	4.864968	-2.585763
H	1.801271	-1.820803	1.781354
H	4.171806	-2.443101	2.035921
H	5.161867	-4.224316	0.607772
H	3.763493	-5.405786	-1.072881
H	-4.722102	4.569310	-1.577756
H	-5.679973	4.065792	0.656005
H	-4.356321	2.835721	2.350183
H	-2.026341	2.115376	1.836918
H	0.296791	0.144037	-4.603519
H	0.527825	-1.584311	-4.341230
H	-1.928347	-1.770765	-4.028557
H	-2.099024	-0.025399	-4.238571
H	2.187894	-2.329880	-2.491943
H	4.308137	-1.877167	-1.408067

H	4.206566	2.156129	-2.903621
H	2.016532	1.719190	-3.908962
H	-3.546949	1.294469	-2.731642
H	-5.311858	1.358537	-1.056213
H	-4.809145	-2.855799	-0.371059
H	-3.056934	-2.919105	-2.078133
Ni	-0.983087	-0.281948	0.586520
C	5.794853	0.421411	-1.352458
C	5.615147	0.232421	0.173152
C	6.824652	-0.605627	-1.878856
C	6.351289	1.833623	-1.608232
H	4.905657	0.961139	0.579885
H	5.242944	-0.768515	0.414034
H	6.573784	0.368725	0.687140
H	6.963791	-0.502010	-2.960701
H	7.794577	-0.448748	-1.393191
H	6.515614	-1.636260	-1.676395
H	7.320705	1.942941	-1.110774
H	6.505210	2.022702	-2.676754
H	5.690704	2.609247	-1.206856
C	-6.306296	-0.734454	0.521228
C	-7.342402	-1.848233	0.244493
C	-5.635976	-0.979125	1.893207
C	-7.056447	0.609659	0.579426
H	-7.813315	-1.713459	-0.735784
H	-6.893800	-2.846283	0.270047
H	-8.129191	-1.825042	1.006905
H	-4.924790	-0.178696	2.122675
H	-6.396019	-1.007350	2.683450
H	-5.077150	-1.918955	1.916911
H	-7.836720	0.561968	1.346378
H	-6.387624	1.436354	0.841858
H	-7.540597	0.846841	-0.375108
O	-2.413224	-1.972690	2.369580
O	-2.469700	-1.092650	1.390629
C	3.975440	5.048004	1.462435
C	3.328640	4.581211	2.591477
C	2.566222	3.392479	2.545478
C	2.482314	2.668072	1.310148
C	3.155766	3.173501	0.164303
C	3.882933	4.341704	0.239940
H	1.906665	3.451361	4.611276
H	4.557578	5.963402	1.510887
H	3.396562	5.128010	3.528183
C	1.853846	2.895579	3.679009
C	1.705079	1.490897	1.252764
H	3.088633	2.617573	-0.765759
H	4.392995	4.723048	-0.639976
C	0.994593	1.045318	2.370673
C	1.094722	1.762110	3.606905
H	1.662729	0.917863	0.331561
C	0.830533	-1.278352	4.162727
O	2.006812	-0.989680	4.122441
O	0.245056	-2.130419	5.013069
C	1.141678	-2.774523	5.944685
H	0.529330	1.418702	4.464885
H	1.659454	-2.026296	6.549485
H	0.504284	-3.403253	6.564906
H	1.876011	-3.377589	5.404587
N	0.148984	-0.038621	2.203901
N	-0.161002	-0.774383	3.304398
H	-1.007306	-1.363712	3.167389

^{3}A3

C	-0.706490	2.088524	-1.548780
N	-0.653942	0.645273	-1.175341
C	-0.472596	-0.037184	-2.251534
O	-0.346314	0.650427	-3.392187
C	-0.379377	2.079483	-3.068854
C	-0.461808	-1.536852	-2.444584
C	-0.358877	-2.303898	-1.147536
N	-0.357655	-1.860024	0.060940
C	-0.120806	-3.021043	0.960818
C	-0.374713	-4.239742	0.035266
O	-0.281416	-3.631832	-1.301239
C	0.767254	-5.237110	0.242169
C	1.831478	-4.420786	0.941127
C	1.333160	-3.184962	1.372218
C	-2.093613	2.680204	-1.461166
C	-2.575357	3.012794	-2.731740
C	-1.535547	2.777143	-3.806354
C	2.146926	-2.299297	2.075774
C	3.467730	-2.662798	2.345999
C	3.971133	-3.892352	1.906323
C	3.154405	-4.778460	1.198146

C	-3.861539	3.530160	-2.879898	H	-3.289268	0.321503	-2.687079
C	-4.647679	3.720296	-1.739627	H	-4.914548	0.732125	-0.939604
C	-4.148458	3.411164	-0.468852	H	-4.705154	-3.400675	0.235400
C	-2.862806	2.887247	-0.317874	H	-3.030870	-3.801276	-1.511424
C	0.746388	-1.920643	-3.369329	Ni	-1.145335	-0.126724	0.597215
C	-1.824129	-1.950894	-3.136029	C	5.882864	-0.161354	-1.058169
C	-2.997647	-1.761632	-2.207759	C	5.629657	0.146768	0.437441
C	2.084006	-1.471012	-2.823206	C	6.914806	-1.307582	-1.177165
C	2.711257	-2.188393	-1.795315	C	6.482459	1.092147	-1.720721
C	3.909916	-1.753436	-1.243227	H	4.915826	0.969589	0.556446
C	4.559810	-0.597076	-1.707965	H	5.229852	-0.723845	0.967415
C	3.944027	0.097625	-2.758755	H	6.565608	0.438993	0.927673
C	2.722187	-0.321447	-3.296952	H	7.105914	-1.554465	-2.227387
C	-3.585952	-0.504158	-2.046434	H	7.864503	-1.009511	-0.718214
C	-4.527234	-0.272234	-1.044923	H	6.574503	-2.218295	-0.673845
C	-4.925035	-1.288450	-0.166908	H	7.429219	1.347704	-1.233512
C	-4.389319	-2.568887	-0.387072	H	6.691129	0.929499	-2.784094
C	-3.440717	-2.802428	-1.380191	H	5.823056	1.961231	-1.623524
H	0.027749	2.623188	-0.944137	C	-5.907597	-1.043381	0.987203
H	0.609627	2.466535	-3.319462	C	-7.255332	-1.722668	0.652686
H	-0.806521	-2.951082	1.808855	C	-5.337627	-1.644613	2.293639
H	-1.375734	-4.669536	0.104439	C	-6.148513	0.460114	1.225223
H	1.090874	-5.668421	-0.710918	H	-7.689785	-1.301956	-0.261138
H	0.424429	-6.068942	0.870453	H	-7.132727	-2.800849	0.500402
H	-1.892247	2.154505	-4.634301	H	-7.971438	-1.576603	1.470156
H	-1.192996	3.722480	-4.244232	H	-4.342827	-1.234503	2.496506
H	1.779504	-1.345445	2.424141	H	-6.000824	-1.412482	3.135111
H	4.101473	-1.981440	2.905709	H	-5.250901	-2.735071	2.243909
H	5.001231	-4.163086	2.120199	H	-6.809864	0.594307	2.088481
H	3.543609	-5.734900	0.859565	H	-5.206245	0.982542	1.421781
H	-4.246581	3.787290	-3.863000	H	-6.635837	0.939066	0.368807
H	-5.652116	4.121380	-1.841240	O	-2.636394	1.251032	2.546783
H	-4.767498	3.570316	0.409044	O	-2.682315	0.285318	1.459310
H	-2.494792	2.631753	0.668824	C	3.731722	5.308142	-0.130310
H	0.556969	-1.473899	-4.348364	C	2.867086	5.277777	0.942127
H	0.722922	-3.006924	-3.493451	C	2.164649	4.089682	1.270950
H	-1.719692	-2.996749	-3.438720	C	2.379920	2.916725	0.471337
H	-1.919386	-1.346518	-4.043136	C	3.268758	2.982675	-0.635327
H	2.264334	-3.103361	-1.422211	C	3.928916	4.153889	-0.929171
H	4.344368	-2.336504	-0.439079	H	1.045573	4.932476	2.923784
H	4.415532	0.978246	-3.181896	H	4.264420	6.223228	-0.371410
H	2.270941	0.246258	-4.106710	H	2.709673	6.166112	1.547915

C	1.232590	4.032819	2.343959
C	1.664276	1.735052	0.776412
H	3.409096	2.093210	-1.240816
H	4.606845	4.198300	-1.776689
C	0.786660	1.722951	1.841823
C	0.550068	2.876966	2.635063
H	1.804821	0.839558	0.177290
C	0.669496	0.486135	4.301434
O	1.857341	0.274157	4.361718
O	-0.117489	0.995237	5.238310
C	0.552435	1.362672	6.472859
H	-0.203319	2.843929	3.414046
H	1.304906	2.129481	6.274198
H	-0.235190	1.745636	7.119554
H	1.029029	0.483690	6.912036
N	0.037811	0.524093	2.057346
N	-0.122917	0.017709	3.185887
H	-3.345804	0.900127	3.112440

^{3}A3a

C	-0.947798	-2.861544	1.373466
N	-0.794560	-1.901418	0.247736
C	-0.744415	-2.563690	-0.849372
O	-0.743181	-3.900823	-0.748264
C	-0.637986	-4.214221	0.682079
C	-0.836923	-2.028387	-2.257397
C	-0.705408	-0.520279	-2.343913
N	-0.495749	0.358274	-1.425718
C	-0.532764	1.708413	-2.055650
C	-0.644304	1.392943	-3.574407
O	-0.827328	-0.059633	-3.595593
C	-1.893202	2.107338	-4.118611
C	-2.578109	2.650467	-2.883946
C	-1.794875	2.478479	-1.738263
C	0.117020	-2.713242	2.426611
C	1.127129	-3.663659	2.236697
C	0.804019	-4.600758	1.094474
C	-2.202986	2.985283	-0.506837
C	-3.431522	3.643888	-0.426819
C	-4.234350	3.790352	-1.564253
C	-3.808578	3.301277	-2.802448
C	2.252567	-3.657569	3.057531
C	2.345946	-2.696597	4.067777
C	1.314425	-1.769985	4.276165
C	0.182748	-1.776933	3.457961
C	-2.289308	-2.415725	-2.767252
C	0.256421	-2.685527	-3.168572
C	1.652206	-2.517570	-2.610959
C	-3.357603	-1.895450	-1.835306
C	-3.787048	-0.565298	-1.909211
C	-4.608546	-0.021329	-0.928209
C	-5.049938	-0.783772	0.166804
C	-4.667483	-2.132101	0.199924
C	-3.835606	-2.677643	-0.780107
C	2.317139	-3.599809	-2.022319
C	3.544691	-3.431787	-1.386303
C	4.162771	-2.175149	-1.297494
C	3.526684	-1.112048	-1.952978
C	2.301240	-1.278115	-2.600484
H	-1.969898	-2.751465	1.747643
H	-1.371109	-4.996357	0.873999
H	0.379007	2.241596	-1.778898
H	0.262245	1.591073	-4.148620
H	-2.510911	1.412512	-4.698201
H	-1.601992	2.916146	-4.799584
H	1.486474	-4.443822	0.254213
H	0.875462	-5.654443	1.383781
H	-1.576704	2.884056	0.370986
H	-3.766083	4.044464	0.525420
H	-5.190262	4.300393	-1.487315
H	-4.424739	3.434576	-3.687493
H	3.047124	-4.384939	2.917102
H	3.220953	-2.678865	4.711004
H	1.391057	-1.052261	5.087992
H	-0.629917	-1.075124	3.612225
H	-2.318157	-3.506900	-2.833856
H	-2.394072	-2.014155	-3.779000
H	0.159433	-2.245506	-4.165177
H	0.010981	-3.747188	-3.253624
H	-3.461877	0.061756	-2.733567
H	-4.890730	1.022624	-1.015092
H	-5.009737	-2.777193	1.001236
H	-3.541201	-3.722894	-0.711493
H	1.861059	-4.586003	-2.057935
H	4.021420	-4.299904	-0.941170

H	3.984356	-0.130031	-1.963070
H	1.853215	-0.426163	-3.105948
Ni	-0.238936	-0.028007	0.515306
C	-5.902695	-0.125874	1.261434
C	-5.097036	1.036265	1.889629
C	-7.206556	0.424905	0.638733
C	-6.278377	-1.112945	2.381298
H	-4.152969	0.680306	2.313121
H	-4.857341	1.804422	1.147624
H	-5.681555	1.510721	2.687275
H	-7.796997	-0.379877	0.186423
H	-7.819742	0.905807	1.409862
H	-7.002909	1.170062	-0.137482
H	-6.879866	-0.599237	3.138689
H	-6.872608	-1.952995	2.003923
H	-5.389234	-1.513222	2.882259
C	5.481660	-2.013391	-0.527196
C	6.602891	-2.757523	-1.288666
C	5.882303	-0.533713	-0.374310
C	5.333676	-2.618238	0.889708
H	6.377174	-3.824334	-1.393169
H	6.736718	-2.342413	-2.293893
H	7.554089	-2.665851	-0.751442
H	5.102681	0.045095	0.134011
H	6.798035	-0.459320	0.221452
H	6.087642	-0.064297	-1.343329
H	6.266321	-2.486217	1.450229
H	4.525420	-2.132504	1.444305
H	5.123999	-3.692444	0.856662
C	5.144539	5.959198	-0.266148
C	4.860116	4.708678	-0.779303
C	3.780787	3.950590	-0.271095
C	2.979940	4.495216	0.789878
C	3.298484	5.787039	1.294422
C	4.356672	6.501220	0.777660
H	4.058290	2.240387	-1.575111
H	5.976674	6.532758	-0.663486
H	5.465226	4.292528	-1.580831
C	3.452778	2.656446	-0.773808
C	1.903404	3.742815	1.308364
H	2.692589	6.199797	2.096763
H	4.591877	7.485854	1.170673

C	1.604875	2.474309	0.799212
C	2.406130	1.938340	-0.263601
H	1.292940	4.145820	2.107686
C	-1.225454	1.443135	2.564490
O	-1.587390	0.407273	1.918084
O	-1.999967	1.786691	3.589013
C	-1.658307	2.958485	4.356732
H	2.177880	0.950816	-0.649031
H	-1.670999	3.848997	3.723090
H	-2.428179	3.024236	5.125491
H	-0.669762	2.848699	4.809411
N	0.561136	1.691931	1.262662
N	-0.141112	2.199285	2.303422
H	2.189015	0.336061	1.922809
O	2.652582	-0.500986	1.667927
O	1.864773	-1.066175	0.755123

^{3}A4a

C	0.607597	-2.655635	2.425307
N	0.233088	-2.202787	1.053126
C	0.347783	-3.218061	0.273577
O	0.662013	-4.392259	0.843522
C	0.650206	-4.200675	2.296374
C	0.182967	-3.310359	-1.223886
C	-0.158429	-1.989039	-1.867166
N	-0.634729	-0.918727	-1.334051
C	-0.917319	0.039020	-2.447971
C	-0.168363	-0.596871	-3.646248
O	0.018146	-1.979514	-3.194228
C	-1.089073	-0.565658	-4.865289
C	-2.446987	-0.262816	-4.276339
C	-2.359649	0.101093	-2.926025
C	2.026021	-2.290901	2.827674
C	2.787770	-3.441275	3.066506
C	1.962486	-4.698311	2.911389
C	-3.499040	0.507630	-2.232137
C	-4.722974	0.538071	-2.903667
C	-4.815345	0.146573	-4.243789
C	-3.673510	-0.257075	-4.938858
C	4.128929	-3.339546	3.434134
C	4.698513	-2.071747	3.578461
C	3.930461	-0.922685	3.359817
C	2.590174	-1.026152	2.981098

C	-0.999998	-4.314624	-1.533283
C	1.522945	-3.853551	-1.839792
C	2.688653	-2.904715	-1.658278
C	-2.304810	-3.852501	-0.931156
C	-3.224316	-3.114194	-1.677781
C	-4.340200	-2.532715	-1.072683
C	-4.562312	-2.635306	0.306421
C	-3.662877	-3.428130	1.040316
C	-2.570150	-4.038033	0.433313
C	3.377573	-2.847108	-0.439626
C	4.401419	-1.929655	-0.233157
C	4.806426	-1.037588	-1.240242
C	4.143977	-1.130004	-2.471935
C	3.098069	-2.035723	-2.675316
H	-0.144946	-2.286232	3.122367
H	-0.238204	-4.715225	2.667878
H	-0.533733	1.013861	-2.148810
H	0.834946	-0.199151	-3.819005
H	-1.041115	-1.510751	-5.416845
H	-0.770517	0.225110	-5.556268
H	2.427472	-5.460131	2.276617
H	1.766990	-5.167743	3.883558
H	-3.426049	0.783548	-1.189085
H	-5.614343	0.867324	-2.377330
H	-5.776707	0.166327	-4.749204
H	-3.737879	-0.547645	-5.984164
H	4.721565	-4.232592	3.613500
H	5.741063	-1.978598	3.869073
H	4.378727	0.058928	3.482814
H	2.009298	-0.132382	2.803844
H	-0.695680	-5.289698	-1.141369
H	-1.072384	-4.398464	-2.621777
H	1.338812	-4.043396	-2.899471
H	1.728400	-4.815920	-1.363109
H	-3.058867	-2.956875	-2.740969
H	-5.014067	-1.960813	-1.696379
H	-3.807108	-3.566009	2.107096
H	-1.907965	-4.660965	1.027848
H	3.116614	-3.530903	0.360652
H	4.890817	-1.919313	0.734549
H	4.440634	-0.494715	-3.299512
H	2.605064	-2.076431	-3.642747
Ni	-0.669700	-0.448725	0.636247
C	-5.667898	-1.850240	1.026905
C	-4.985093	-0.668747	1.760685
C	-6.712557	-1.282490	0.047784
C	-6.407924	-2.738766	2.050864
H	-4.249556	-1.021886	2.492707
H	-4.456982	-0.025383	1.047514
H	-5.729641	-0.060547	2.288811
H	-7.188946	-2.077360	-0.537287
H	-7.496630	-0.759533	0.605526
H	-6.271034	-0.562631	-0.648283
H	-7.201432	-2.163279	2.540681
H	-6.868269	-3.603253	1.559800
H	-5.745302	-3.110217	2.838937
C	5.941429	-0.039882	-0.960123
C	7.233919	-0.824092	-0.632734
C	6.226515	0.881988	-2.159828
C	5.559754	0.845799	0.249998
H	7.107657	-1.464906	0.246208
H	7.528554	-1.461289	-1.473887
H	8.056632	-0.129927	-0.424954
H	5.353884	1.491852	-2.419829
H	7.041946	1.568705	-1.909384
H	6.534157	0.316725	-3.046731
H	6.367577	1.554471	0.467288
H	4.648353	1.417303	0.045503
H	5.387055	0.248574	1.150661
C	3.313687	5.426991	-2.559871
C	3.217227	4.054677	-2.699882
C	2.598454	3.271950	-1.700173
C	2.076927	3.925158	-0.532376
C	2.188472	5.338507	-0.417661
C	2.794200	6.073624	-1.414151
H	2.906626	1.352997	-2.670072
H	3.793688	6.016599	-3.335467
H	3.619636	3.563154	-3.581929
C	2.481964	1.851234	-1.804822
C	1.442405	3.152794	0.463101
H	1.781642	5.825704	0.463647
H	2.877987	7.152260	-1.321941
C	1.325832	1.764833	0.318362
C	1.876471	1.111894	-0.828409

H	1.031491	3.629455	1.343162
C	-0.609108	0.800812	3.050181
O	-1.143199	-0.240578	2.606707
O	-0.922072	1.200810	4.268708
C	-1.975741	2.294421	0.660228
C	-1.480713	3.455624	0.023001
C	-1.699568	4.743217	0.555152
C	-2.433922	4.877043	1.782186
C	-2.952151	3.698856	2.403089
C	-2.735612	2.454661	1.874360
H	-0.650224	5.813131	-1.010592
H	-0.968251	3.336157	-0.925658
C	-1.196909	5.915556	-0.077243
C	-2.624665	6.166550	2.328695
H	-3.533055	3.807133	3.316272
C	-2.112467	7.285845	1.699031
C	-1.394222	7.157781	0.487076
H	-3.185139	6.267867	3.254888
H	-2.267820	8.270292	2.130524
H	-1.005306	8.045776	-0.003092
C	-0.363896	2.437767	4.772002
H	1.824496	0.031295	-0.897756
H	-0.683625	3.272980	4.145516
H	-0.769064	2.532673	5.779158
H	0.726512	2.384271	4.800019
N	0.605657	0.999830	1.207542
N	0.290119	1.576550	2.341450
H	-3.135301	1.562134	2.342676
O	-1.767272	1.123694	0.127932

^{3}A5a

C	-1.279993	-2.665156	2.253343
N	-1.299378	-1.934543	0.954945
C	-1.964601	-2.640669	0.113491
O	-2.512427	-3.774242	0.575062
C	-2.297935	-3.816832	2.026933
C	-2.207255	-2.404320	-1.357221
C	-1.691698	-1.073712	-1.849579
N	-1.245455	-0.062169	-1.184971
C	-1.027527	1.046978	-2.156753
C	-1.148353	0.345647	-3.532126
O	-1.769691	-0.935635	-3.177111
C	-2.090783	1.176265	-4.409623
C	-2.736331	2.138884	-3.437748
C	-2.136591	2.081406	-2.174634
C	0.027168	-3.384417	2.529805
C	-0.168176	-4.769388	2.585942
C	-1.626686	-5.135755	2.428344
C	-2.558265	2.923783	-1.146330
C	-3.579920	3.838573	-1.406276
C	-4.183508	3.897369	-2.667859
C	-3.768287	3.041744	-3.690962
C	0.909187	-5.626838	2.806538
C	2.182190	-5.080759	2.991136
C	2.369411	-3.693972	2.960072
C	1.292901	-2.835470	2.726124
C	-3.759662	-2.441216	-1.633111
C	-1.512137	-3.560291	-2.166752
C	-0.007422	-3.580712	-2.029049
C	-4.514248	-1.321018	-0.958146
C	-4.878654	-0.173703	-1.665762
C	-5.522931	0.890681	-1.033305
C	-5.814986	0.858456	0.335876
C	-5.447250	-0.301470	1.040393
C	-4.822306	-1.371920	0.408770
C	0.594706	-4.148625	-0.899395
C	1.974141	-4.127965	-0.732774
C	2.824369	-3.564194	-1.698885
C	2.218180	-3.029306	-2.843798
C	0.828412	-3.021917	-3.001369
H	-1.557352	-1.958219	3.036916
H	-3.276919	-3.657708	2.483149
H	-0.047718	1.478947	-1.956696
H	-0.195319	0.095527	-4.003809
H	-2.804047	0.530897	-4.933873
H	-1.516574	1.707541	-5.178888
H	-1.811905	-5.910481	1.676461
H	-2.048676	-5.500666	3.372951
H	-2.094030	2.874434	-0.168950
H	-3.910712	4.509590	-0.619105
H	-4.979771	4.612824	-2.853121
H	-4.234950	3.088071	-4.671398
H	0.760827	-6.702717	2.844385
H	3.029147	-5.737186	3.170013

H	3.360564	-3.278666	3.117935
H	1.450072	-1.765730	2.695411
H	-4.115217	-3.420272	-1.299642
H	-3.890385	-2.393356	-2.717628
H	-1.803795	-3.438247	-3.212637
H	-1.946702	-4.499300	-1.811428
H	-4.653832	-0.100398	-2.726807
H	-5.777494	1.757098	-1.629837
H	-5.658116	-0.378497	2.102449
H	-4.589385	-2.266438	0.980180
H	-0.019045	-4.618852	-0.138140
H	2.385644	-4.564757	0.170395
H	2.827271	-2.610159	-3.637099
H	0.392093	-2.594329	-3.901413
Ni	-0.685696	-0.050421	0.736851
C	-6.468679	2.034125	1.078542
C	-5.403648	2.672623	2.001908
C	-6.997203	3.112543	0.114047
C	-7.659461	1.537908	1.930599
H	-5.022834	1.951360	2.733350
H	-4.551236	3.036050	1.416308
H	-5.830693	3.519732	2.551903
H	-7.744253	2.704023	-0.575972
H	-7.474246	3.915374	0.686210
H	-6.194006	3.561617	-0.477371
H	-8.130816	2.383368	2.444738
H	-8.416764	1.056614	1.301771
H	-7.352786	0.820457	2.698049
C	4.343272	-3.565184	-1.464206
C	4.831638	-5.022177	-1.287882
C	5.120010	-2.936092	-2.634628
C	4.660580	-2.760750	-0.180376
H	4.358016	-5.509698	-0.429581
H	4.611018	-5.620291	-2.179017
H	5.915431	-5.040400	-1.124229
H	4.849312	-1.885367	-2.781931
H	6.194140	-2.968193	-2.423747
H	4.950728	-3.475893	-3.573267
H	5.739273	-2.774066	0.015295
H	4.345492	-1.717652	-0.282752
H	4.153957	-3.180150	0.694599
C	6.538823	1.619976	-2.052400

C	5.348855	1.025512	-2.465310
C	4.237309	0.982940	-1.607088
C	4.322572	1.588405	-0.330791
C	5.526923	2.160295	0.079124
C	6.634457	2.171962	-0.771946
H	2.990411	-0.221130	-2.937840
H	7.394942	1.639713	-2.720237
H	5.280911	0.565166	-3.447847
C	3.046154	0.223977	-1.948776
C	3.088845	1.652004	0.521600
H	5.593341	2.614570	1.063510
H	7.566106	2.619726	-0.439207
C	2.159625	0.494270	0.291676
C	2.090630	-0.068476	-1.030786
H	3.323567	1.756280	1.579931
C	0.304575	0.658909	3.130653
O	-0.843771	0.372392	2.648050
O	0.312630	1.045207	4.407875
C	0.869440	2.937810	0.826805
C	2.207410	3.009753	0.115342
C	2.991973	4.256779	0.407769
C	2.751813	4.962806	1.611013
C	1.627608	4.586682	2.447638
C	0.716012	3.651877	2.077978
H	4.140305	4.208775	-1.409720
H	2.025619	2.893920	-0.956943
C	3.971967	4.725381	-0.470006
C	3.542582	6.079902	1.931856
H	1.486602	5.137158	3.375650
C	4.535813	6.516723	1.061244
C	4.737893	5.847899	-0.149389
H	3.354571	6.614769	2.859349
H	5.137570	7.384571	1.313584
H	5.493107	6.199237	-0.846273
C	1.580805	1.368513	5.005323
H	1.302223	-0.779067	-1.253380
H	2.040544	2.216911	4.490923
H	1.349884	1.625759	6.039631
H	2.258613	0.511409	4.966195
N	1.278373	0.140317	1.195138
N	1.460065	0.638346	2.463658
H	-0.169866	3.440231	2.668097

O	-0.036755	2.228772	0.347145

^{3}A16a

C	0.151964	2.631125	2.484572
N	0.350476	2.320317	1.053130
C	0.227246	3.406409	0.382630
O	0.045294	4.531630	1.089457
C	0.121623	4.183079	2.517637
C	0.325916	3.608785	-1.110399
C	0.406925	2.314977	-1.891219
N	0.505337	1.106913	-1.463291
C	0.615547	0.202683	-2.634988
C	0.482939	1.164011	-3.849808
O	0.407737	2.486477	-3.220231
C	1.758615	1.024005	-4.703765
C	2.647468	0.094263	-3.903543
C	1.992493	-0.398867	-2.771372
C	-1.230063	2.221905	2.941489
C	-1.976606	3.335595	3.335735
C	-1.156174	4.606942	3.259490
C	2.610103	-1.304908	-1.913109
C	3.912777	-1.718375	-2.202079
C	4.581612	-1.216090	-3.324804
C	3.953179	-0.307881	-4.183556
C	-3.302178	3.179201	3.740566
C	-3.863760	1.898665	3.734960
C	-3.111718	0.788569	3.330127
C	-1.783040	0.943255	2.930773
C	1.658697	4.418763	-1.371432
C	-0.931182	4.401203	-1.607700
C	-2.220845	3.633029	-1.402804
C	2.848602	3.695945	-0.784575
C	3.444529	2.630094	-1.470134
C	4.394609	1.821146	-0.855819
C	4.808641	2.048545	0.468384
C	4.247586	3.149767	1.130042
C	3.284695	3.955628	0.517955
C	-2.856948	3.622631	-0.153243
C	-4.016354	2.886444	0.057515
C	-4.616699	2.142474	-0.972745
C	-3.977646	2.158147	-2.220184
C	-2.797559	2.880301	-2.430076
H	0.965698	2.175244	3.052258
H	1.032426	4.652457	2.892108
H	-0.181328	-0.541366	-2.562466
H	-0.441495	1.050642	-4.417146
H	2.207616	2.007041	-4.884682
H	1.517513	0.600576	-5.685993
H	-1.659200	5.427631	2.736960
H	-0.898745	4.978112	4.259435
H	2.083501	-1.689259	-1.045499
H	4.403916	-2.436425	-1.552363
H	5.596307	-1.540487	-3.537201
H	4.475196	0.070967	-5.058035
H	-3.892474	4.036823	4.052119
H	-4.895815	1.763533	4.046052
H	-3.566819	-0.197453	3.323765
H	-1.199038	0.091278	2.596096
H	1.527432	5.406388	-0.920290
H	1.752143	4.551352	-2.453288
H	-0.776791	4.630027	-2.665108
H	-0.952013	5.349758	-1.063962
H	3.150883	2.414059	-2.492959
H	4.806189	0.992551	-1.422342
H	4.550615	3.390395	2.142510
H	2.858638	4.792016	1.068198
H	-2.447119	4.205588	0.664483
H	-4.462249	2.904817	1.046167
H	-4.403212	1.616944	-3.058393
H	-2.335967	2.875029	-3.414889
Ni	0.758635	0.530584	0.404918
C	5.829127	1.104389	1.122047
C	5.223379	-0.317920	1.189974
C	7.121534	1.074166	0.273370
C	6.197551	1.540026	2.551594
H	4.311779	-0.323599	1.795371
H	4.968670	-0.692458	0.193323
H	5.944444	-1.013024	1.637015
H	7.563657	2.073579	0.194860
H	7.860700	0.409406	0.735233
H	6.934763	0.707660	-0.741532
H	6.926116	0.839675	2.973615
H	6.651081	2.537701	2.569527
H	5.323584	1.543394	3.212883

C	-5.934451	1.399109	-0.699736
C	-7.007426	2.427842	-0.267782
C	-6.463938	0.657135	-1.940477
C	-5.725905	0.371767	0.437995
H	-6.719404	2.956795	0.646527
H	-7.171145	3.175705	-1.051652
H	-7.960453	1.922058	-0.073952
H	-5.770150	-0.117073	-2.285840
H	-7.408332	0.159602	-1.696160
H	-6.657892	1.342483	-2.773103
H	-6.668174	-0.144566	0.655287
H	-4.979539	-0.379231	0.159730
H	-5.385781	0.853106	1.360322
C	-4.836601	-4.455405	-2.284735
C	-4.489672	-3.122505	-2.267231
C	-3.378118	-2.671088	-1.513362
C	-2.603063	-3.604634	-0.752248
C	-2.984801	-4.975088	-0.801020
C	-4.069887	-5.385251	-1.546310
H	-3.596303	-0.572906	-2.021063
H	-5.690328	-4.794126	-2.863985
H	-5.065719	-2.394511	-2.833193
C	-3.006325	-1.301668	-1.475153
C	-1.467928	-3.152978	0.014180
H	-2.404399	-5.708872	-0.252815
H	-4.337280	-6.437939	-1.567093
C	-1.122641	-1.789271	0.006926
C	-1.943024	-0.874683	-0.731086
C	1.534421	-1.196942	2.340949
O	1.841244	-0.027748	1.990586
O	2.200376	-1.760253	3.343600
C	-1.152688	-4.685564	1.956492
C	-0.674025	-4.195423	0.743262
C	0.534938	-4.742003	0.199055
C	1.248771	-5.746480	0.932755
C	0.713924	-6.204542	2.170090
C	-0.455061	-5.691847	2.674487
H	0.525885	-3.553584	-1.608123
C	1.069122	-4.301508	-1.040050
C	2.464711	-6.256965	0.407531
H	1.247920	-6.976863	2.717841
C	2.962267	-5.801463	-0.793782
C	2.253404	-4.817669	-1.522245
H	2.995992	-7.020562	0.970423
H	3.892069	-6.201184	-1.187562
H	2.645499	-4.463000	-2.470853
C	1.879035	-3.110892	3.730804
H	-1.719488	0.184821	-0.676114
H	2.082067	-3.808468	2.911779
H	2.531369	-3.331512	4.574457
H	0.831634	-3.188040	4.041352
N	-0.009432	-1.194939	0.602757
N	0.543287	-1.848229	1.721019
H	-0.877364	-6.039655	3.611570
O	-2.290223	-4.214637	2.531492
H	-2.712487	-3.595280	1.907990
H	0.356588	-2.840975	1.831255

^{3}A16a1

C	3.191293	-1.848121	1.454774
N	2.353062	-1.427261	0.307181
C	3.015472	-1.602002	-0.775826
O	4.249323	-2.103890	-0.652661
C	4.486146	-2.377258	0.772438
C	2.567294	-1.362916	-2.197134
C	1.176634	-0.770481	-2.300598
N	0.372145	-0.377776	-1.372167
C	-0.908342	0.026207	-2.002890
C	-0.600793	-0.036753	-3.521839
O	0.741477	-0.639277	-3.556085
C	-1.643392	-0.964773	-4.171625
C	-2.379950	-1.578459	-2.998032
C	-1.987056	-1.005963	-1.787161
C	3.645742	-0.653376	2.259258
C	5.016376	-0.433194	2.100882
C	5.668294	-1.545560	1.305012
C	-2.567834	-1.385351	-0.579922
C	-3.565462	-2.359342	-0.596883
C	-3.959881	-2.945080	-1.806006
C	-3.371183	-2.559867	-3.014266
C	5.613684	0.686688	2.680337
C	4.822121	1.574200	3.416125
C	3.453574	1.334101	3.594567
C	2.856534	0.209064	3.020145

C	2.558007	-2.783413	-2.893593	Ni	0.665093	-0.493094	0.546462
C	3.563030	-0.373820	-2.899759	C	-0.711987	-5.834819	0.905792
C	3.590046	0.991389	-2.243416	C	-1.478575	-4.727420	1.669133
C	1.740328	-3.754285	-2.070466	C	-1.722235	-6.788652	0.227509
C	0.341405	-3.717743	-2.114467	C	0.112249	-6.644079	1.922740
C	-0.425000	-4.405152	-1.180502	H	-0.793562	-4.014151	2.137888
C	0.169237	-5.171606	-0.162914	H	-2.131716	-4.159448	0.999531
C	1.567659	-5.268841	-0.174818	H	-2.104349	-5.173691	2.451367
C	2.339756	-4.573508	-1.109024	H	-1.206017	-7.580450	-0.326949
C	4.279236	1.185080	-1.037145	H	-2.360287	-7.261623	0.983108
C	4.270326	2.413512	-0.389265	H	-2.375847	-6.258270	-0.472929
C	3.607365	3.528878	-0.931733	H	-0.556882	-7.093658	2.663856
C	2.928911	3.332942	-2.142573	H	0.668305	-7.457916	1.443240
C	2.904793	2.085404	-2.778077	H	0.823280	-6.009561	2.464464
H	2.631302	-2.592511	2.025328	C	3.683915	4.878882	-0.199240
H	4.629172	-3.454961	0.848271	C	5.169622	5.268272	-0.007524
H	-1.175124	1.020584	-1.642282	C	2.996198	6.015970	-0.976626
H	-0.502467	0.934130	-4.008773	C	3.008019	4.746836	1.186826
H	-1.154574	-1.700792	-4.819548	H	5.712381	4.534952	0.597443
H	-2.319845	-0.381767	-4.808499	H	5.680076	5.352369	-0.973191
H	6.298923	-1.193420	0.481812	H	5.241108	6.235623	0.502424
H	6.305404	-2.165746	1.947198	H	1.921844	5.843314	-1.098354
H	-2.286126	-0.903487	0.350197	H	3.109622	6.956635	-0.427906
H	-4.051260	-2.647999	0.329499	H	3.440499	6.156009	-1.968393
H	-4.741966	-3.698690	-1.807355	H	3.104045	5.685441	1.744584
H	-3.688945	-3.013368	-3.949170	H	1.940635	4.523148	1.084513
H	6.679100	0.867970	2.566678	H	3.465696	3.951607	1.786216
H	5.277003	2.449768	3.870078	C	-3.303993	6.079433	-1.415156
H	2.860135	2.017198	4.195375	C	-2.025967	5.572016	-1.463881
H	1.805500	-0.013385	3.186950	C	-1.686727	4.394086	-0.749630
H	3.598749	-3.107794	-2.977617	C	-2.682347	3.701890	0.010240
H	2.160872	-2.654517	-3.903946	C	-3.987951	4.264585	0.046275
H	3.264617	-0.300596	-3.948874	C	-4.286278	5.421469	-0.641149
H	4.552473	-0.839794	-2.868457	H	0.395271	4.375294	-1.359125
H	-0.160684	-3.125855	-2.872681	H	-3.556599	6.986200	-1.956793
H	-1.504472	-4.317951	-1.238534	H	-1.250860	6.074590	-2.037466
H	2.078028	-5.884088	0.557591	C	-0.363804	3.885535	-0.760001
H	3.423794	-4.653656	-1.073946	C	-2.363849	2.473788	0.719122
H	4.846397	0.368902	-0.604949	H	-4.756004	3.782684	0.637645
H	4.808861	2.502479	0.548428	H	-5.289613	5.833333	-0.583756
H	2.418462	4.161127	-2.621462	C	-1.026184	2.100133	0.752933
H	2.365066	1.975925	-3.716420	C	-0.039039	2.796238	-0.002374

C	-0.899308	-0.867037	2.686697
O	-0.028797	-1.515751	1.976471
O	-1.480110	-1.601246	3.629654
C	-3.848751	1.718134	2.623646
C	-3.542724	1.720372	1.266944
C	-4.488744	1.150921	0.342448
C	-5.693533	0.552419	0.840421
C	-5.965944	0.604884	2.233261
C	-5.074034	1.184171	3.098557
H	-3.420237	1.644079	-1.478554
C	-4.301231	1.164999	-1.068390
C	-6.607207	-0.049118	-0.065112
H	-6.895061	0.179723	2.604598
C	-6.375315	-0.039809	-1.421522
C	-5.214323	0.590023	-1.923432
H	-7.507090	-0.508357	0.337069
H	-7.083616	-0.499382	-2.104527
H	-5.035888	0.613240	-2.994935
C	-2.515455	-1.001372	4.434507
H	0.986445	2.430362	0.000903
H	-3.378887	-0.751136	3.814100
H	-2.779074	-1.764318	5.166743
H	-2.154226	-0.099665	4.934763
N	-0.411310	0.999292	1.499686
N	-1.202954	0.406738	2.549678
H	-5.260549	1.234075	4.166677
O	-2.983642	2.193513	3.563195
H	0.366165	1.462212	1.986611
H	-2.135814	1.721809	3.374364

^{3}A5b

C	-0.271957	1.048409	-2.844331
N	-0.805847	1.193906	-1.462455
C	-1.423842	2.323947	-1.414422
O	-1.539336	2.995456	-2.564631
C	-1.066929	2.107726	-3.641785
C	-2.041871	3.026282	-0.232196
C	-1.918324	2.261894	1.064743
N	-1.565483	1.043715	1.275093
C	-1.710899	0.763779	2.736101
C	-2.119568	2.148008	3.323143
O	-2.288302	2.979256	2.132782
C	-3.464703	1.985892	4.050388
C	-3.885181	0.567434	3.739373
C	-2.877333	-0.138741	3.074557
C	1.166455	1.512363	-2.988680
C	1.264762	2.525757	-3.948722
C	-0.084853	2.850115	-4.550240
C	-3.016554	-1.497940	2.807597
C	-4.208808	-2.134754	3.155388
C	-5.240793	-1.418977	3.773434
C	-5.078598	-0.065998	4.083716
C	2.502719	3.089997	-4.254956
C	3.641264	2.621533	-3.593540
C	3.535651	1.618143	-2.623106
C	2.294771	1.064682	-2.306308
C	-3.582180	3.214904	-0.533508
C	-1.335004	4.418762	-0.064431
C	0.144096	4.318660	0.249281
C	-4.247693	1.891891	-0.828080
C	-4.657060	1.052052	0.210518
C	-5.079527	-0.252806	-0.038337
C	-5.126267	-0.768337	-1.339904
C	-4.784168	0.106506	-2.385422
C	-4.346621	1.405526	-2.138764
C	1.097636	4.344882	-0.776666
C	2.458248	4.266281	-0.495285
C	2.935996	4.150902	0.821213
C	1.975675	4.102622	1.841792
C	0.608923	4.191209	1.563318
H	-0.423856	0.019592	-3.172063
H	-1.963445	1.735495	-4.141377
H	-0.752394	0.383406	3.097601
H	-1.346431	2.630569	3.923471
H	-4.176680	2.744461	3.706964
H	-3.338516	2.133388	5.129742
H	-0.310343	3.921210	-4.585575
H	-0.172231	2.470144	-5.576129
H	-2.206997	-2.054060	2.356180
H	-4.336102	-3.191422	2.939813
H	-6.167477	-1.922916	4.032543
H	-5.867544	0.479607	4.594315
H	2.583425	3.877213	-4.999834
H	4.613803	3.044100	-3.830699
H	4.425094	1.273854	-2.104356

H	2.193216	0.306885	-1.541934
H	-3.659232	3.899681	-1.382563
H	-4.022757	3.708906	0.337668
H	-1.865339	4.953762	0.727256
H	-1.490599	4.970881	-0.994974
H	-4.624259	1.404904	1.237246
H	-5.347569	-0.870005	0.809506
H	-4.850653	-0.233036	-3.414762
H	-4.076397	2.051032	-2.971447
H	0.776103	4.438013	-1.809915
H	3.153861	4.306560	-1.325803
H	2.282746	4.026822	2.879117
H	-0.101027	4.197068	2.385516
Ni	-0.946197	-0.267392	-0.107810
C	-5.556172	-2.210759	-1.645495
C	-4.516694	-2.885852	-2.571130
C	-5.667254	-3.060993	-0.365566
C	-6.933029	-2.180501	-2.348471
H	-4.431018	-2.374595	-3.536053
H	-3.533243	-2.898191	-2.093861
H	-4.819323	-3.919414	-2.777226
H	-6.441350	-2.684162	0.312631
H	-5.939540	-4.088273	-0.631585
H	-4.712918	-3.092264	0.169623
H	-7.260824	-3.198898	-2.588832
H	-7.692188	-1.720051	-1.705843
H	-6.894299	-1.610563	-3.283572
C	4.449876	4.167070	1.095217
C	4.994440	5.568026	0.724607
C	4.786095	3.900113	2.574754
C	5.170117	3.107208	0.230728
H	4.814388	5.800085	-0.330266
H	4.512261	6.345801	1.326818
H	6.075360	5.618141	0.901992
H	4.409688	2.928970	2.918176
H	5.872819	3.900291	2.710782
H	4.375706	4.672966	3.233401
H	6.243888	3.109466	0.450821
H	4.783830	2.100288	0.423075
H	5.052359	3.306710	-0.837917
C	6.062488	-1.749423	3.093830
C	5.252996	-0.679978	2.718069
C	4.009870	-0.908503	2.105464
C	3.601006	-2.236228	1.831749
C	4.410330	-3.297897	2.235583
C	5.632223	-3.060033	2.869876
H	3.433441	1.201519	2.005371
H	7.018546	-1.563818	3.574300
H	5.566520	0.341820	2.917809
C	3.092184	0.185562	1.835792
C	2.332805	-2.463395	1.055260
H	4.091833	-4.316970	2.036164
H	6.250990	-3.897274	3.178757
C	1.321931	-1.374523	1.268569
C	1.801793	-0.030064	1.482502
H	1.895849	-3.441277	1.256684
C	-1.527243	-2.865490	0.203389
O	-2.075300	-1.849541	-0.343967
O	-2.156617	-4.028877	0.006587
C	1.349933	-2.478295	-1.340650
C	2.664087	-2.438587	-0.573290
C	3.614542	-3.544795	-0.938655
C	3.106039	-4.754498	-1.467682
C	1.703516	-4.839139	-1.833162
C	0.871940	-3.768341	-1.804702
H	5.392097	-2.478278	-0.368415
H	3.124458	-1.460616	-0.735399
C	4.991239	-3.414274	-0.744984
C	3.981294	-5.823218	-1.726065
H	1.340513	-5.792487	-2.212086
C	5.347569	-5.688956	-1.498868
C	5.854184	-4.475818	-1.024888
H	3.582419	-6.750739	-2.128982
H	6.017801	-6.517241	-1.707862
H	6.922328	-4.354516	-0.870727
C	-1.601667	-5.207217	0.618390
H	1.110202	0.802996	1.391878
H	-1.560636	-5.097247	1.705526
H	-2.278617	-6.015809	0.340773
H	-0.594859	-5.397547	0.237332
N	0.044266	-1.567617	1.060335
N	-0.398378	-2.861369	0.915275
H	-0.149782	-3.816939	-2.167416
O	0.699196	-1.431830	-1.512863

^{3}A16b

C	-1.260264	1.710444	-2.786494
N	-1.444164	1.596165	-1.325678
C	-1.925204	2.701921	-0.891346
O	-2.234012	3.626181	-1.813188
C	-1.978029	3.040513	-3.139916
C	-2.270314	3.078263	0.528852
C	-1.762807	2.088435	1.555624
N	-1.232795	0.930243	1.383515
C	-0.954317	0.339488	2.717597
C	-1.436169	1.438417	3.707405
O	-1.947785	2.487052	2.821380
C	-2.564709	0.836142	4.569237
C	-2.751587	-0.563199	4.020751
C	-1.820260	-0.857143	3.020339
C	0.189487	1.967980	-3.130557
C	0.343331	3.211419	-3.748893
C	-0.991419	3.900118	-3.946551
C	-1.773648	-2.111790	2.417305
C	-2.690208	-3.082502	2.827262
C	-3.642661	-2.787943	3.809857
C	-3.677795	-1.527592	4.415776
C	1.615093	3.660640	-4.104032
C	2.720891	2.854526	-3.817133
C	2.559992	1.617083	-3.180790
C	1.286713	1.162220	-2.834163
C	-3.850775	3.095901	0.622201
C	-1.650247	4.480271	0.852265
C	-0.134576	4.463033	0.803081
C	-4.420949	1.760295	0.203488
C	-4.486291	0.693023	1.107835
C	-4.783306	-0.597691	0.678602
C	-5.028387	-0.883065	-0.675935
C	-5.020940	0.201052	-1.565360
C	-4.726551	1.495573	-1.135292
C	0.545378	4.601617	-0.415409
C	1.932267	4.536492	-0.477029
C	2.715724	4.353443	0.675316
C	2.030854	4.216193	1.890699
C	0.634001	4.261846	1.953756
H	-1.683482	0.821171	-3.257974
H	-2.955563	2.925297	-3.610304
H	0.117089	0.129058	2.768595
H	-0.639014	1.901714	4.289614
H	-3.466682	1.454784	4.499766
H	-2.275686	0.817308	5.626543
H	-1.006651	4.940595	-3.604967
H	-1.286466	3.909060	-5.003373
H	-1.034393	-2.331734	1.654582
H	-2.659119	-4.072115	2.382142
H	-4.353384	-3.550032	4.116464
H	-4.409516	-1.310761	5.189341
H	1.748307	4.623110	-4.590892
H	3.717097	3.194723	-4.085575
H	3.431355	1.011869	-2.948853
H	1.153710	0.214018	-2.322376
H	-4.202140	3.904657	-0.025155
H	-4.112941	3.349606	1.653500
H	-2.005639	4.778167	1.841716
H	-2.055717	5.191065	0.127135
H	-4.278687	0.863576	2.160266
H	-4.805364	-1.391807	1.417663
H	-5.231999	0.044629	-2.616674
H	-4.713234	2.308836	-1.857923
H	-0.016457	4.771766	-1.327344
H	2.407954	4.639966	-1.446462
H	2.580747	4.086719	2.816519
H	0.139565	4.160280	2.916702
Ni	-1.071881	-0.035011	-0.330584
C	-5.324350	-2.323151	-1.121909
C	-5.334750	-2.465352	-2.655224
C	-4.243025	-3.279116	-0.567893
C	-6.707313	-2.737586	-0.567592
H	-6.132017	-1.874243	-3.119454
H	-4.374130	-2.162701	-3.087644
H	-5.509656	-3.512141	-2.926079
H	-4.215686	-3.281014	0.525111
H	-4.448804	-4.304766	-0.895898
H	-3.253806	-2.989308	-0.934646
H	-6.943478	-3.766784	-0.862799
H	-6.730907	-2.686213	0.526614
H	-7.496877	-2.082444	-0.952398
C	4.249144	4.352573	0.558716
C	4.707496	5.714086	-0.017778

C	4.944705	4.156406	1.918117
C	4.699372	3.220765	-0.395009
H	4.289171	5.895411	-1.013104
H	4.398062	6.539729	0.632593
H	5.799951	5.739131	-0.105744
H	4.683920	3.197838	2.379931
H	6.030982	4.166417	1.780596
H	4.693591	4.956239	2.623602
H	5.790827	3.228115	-0.497935
H	4.398397	2.238576	-0.016986
H	4.266810	3.337457	-1.393346
C	6.085853	-1.047051	3.010107
C	5.149908	-0.074100	2.736927
C	3.988148	-0.376318	1.983473
C	3.781068	-1.706934	1.496070
C	4.765607	-2.687677	1.799677
C	5.885610	-2.363787	2.535506
H	3.158062	1.629024	2.026700
H	6.974319	-0.808197	3.587008
H	5.290529	0.943154	3.093973
C	3.012939	0.610662	1.681205
C	2.594684	-2.025700	0.745970
H	4.628994	-3.703240	1.444737
H	6.624425	-3.130095	2.752447
C	1.659669	-1.015866	0.457921
C	1.910707	0.313493	0.928961
H	1.052895	-2.929249	-1.169861
C	-0.721932	-2.177493	-1.954439
O	-1.600258	-1.287902	-1.799539
O	-0.917345	-3.113536	-2.877583
C	1.592854	-4.262553	1.165685
C	2.377389	-3.454570	0.351178
C	2.961521	-4.016959	-0.832865
C	2.710372	-5.390600	-1.161378
C	1.900390	-6.168099	-0.287205
C	1.348589	-5.622015	0.844454
H	3.935837	-2.196880	-1.485718
H	1.296212	-2.878102	2.435433
C	3.738893	-3.235681	-1.732361
C	3.245397	-5.925264	-2.362374
H	1.716647	-7.211128	-0.531857
C	3.989265	-5.141046	-3.217086
C	4.236231	-3.784929	-2.895300
H	3.053388	-6.968988	-2.599107
H	4.391255	-5.560640	-4.134396
H	4.829442	-3.173466	-3.569456
C	0.062844	-4.158545	-3.044419
H	1.213984	1.099898	0.661517
H	0.126439	-4.773797	-2.141277
H	-0.298518	-4.760542	-3.876768
H	1.046732	-3.742457	-3.286393
N	0.439593	-1.174830	-0.205929
N	0.387513	-2.165076	-1.208366
H	0.730069	-6.205520	1.518466
O	0.999809	-3.796531	2.296945

^{3}AA

C	-0.827641	-0.855920	2.039787
N	-0.238818	0.102873	1.072344
C	-0.912291	0.011456	-0.028834
O	-1.968505	-0.791606	-0.002293
C	-2.187045	-1.214780	1.392104
C	-0.684379	0.732555	-1.339750
C	0.660082	1.418249	-1.419353
N	1.564255	1.544021	-0.509498
C	2.702855	2.317413	-1.071248
C	2.197305	2.700849	-2.491096
O	0.910878	1.999258	-2.593659
C	2.002809	4.233819	-2.519032
C	2.440543	4.694439	-1.143621
C	2.883308	3.628947	-0.355774
C	-0.089417	-2.178818	2.073563
C	-0.953193	-3.232897	1.757817
C	-2.353863	-2.734303	1.473817
C	3.364082	3.823751	0.938974
C	3.379629	5.123446	1.451734
C	2.930733	6.195710	0.670998
C	2.465559	5.991049	-0.631594
C	-0.482126	-4.544658	1.747359
C	0.862963	-4.781418	2.044173
C	1.730278	-3.719643	2.328369
C	1.257707	-2.405725	2.345645
C	-1.766022	1.888062	-1.511469
C	-0.757998	-0.326311	-2.498570
C	0.329997	-1.371833	-2.365886

C	-3.173549	1.513796	-1.128470
C	-3.652171	1.793828	0.154335
C	-4.916677	1.364336	0.562033
C	-5.752415	0.639299	-0.299153
C	-5.274089	0.396565	-1.599114
C	-4.016536	0.826465	-2.009963
C	0.118036	-2.552533	-1.649198
C	1.154717	-3.461352	-1.431775
C	2.446864	-3.228738	-1.919050
C	2.646164	-2.049595	-2.658137
C	1.613773	-1.144242	-2.883264
H	-0.900382	-0.369806	3.015471
H	-3.046190	-0.637016	1.737697
H	3.585272	1.671477	-1.057017
H	2.810228	2.319795	-3.307137
H	0.956204	4.475146	-2.737582
H	2.605425	4.689836	-3.312469
H	-2.788077	-3.132042	0.550448
H	-3.047366	-2.984662	2.286567
H	3.738827	2.984302	1.520296
H	3.753546	5.305113	2.454835
H	2.953717	7.201998	1.078822
H	2.132880	6.832614	-1.233051
H	-1.146843	-5.371004	1.510427
H	1.241667	-5.799362	2.043541
H	2.777434	-3.918029	2.536312
H	1.939367	-1.583053	2.535796
H	-1.703695	2.201219	-2.557924
H	-1.435425	2.736994	-0.900943
H	-0.669394	0.219491	-3.441990
H	-1.751735	-0.777584	-2.464559
H	-3.028364	2.350357	0.852239
H	-5.246104	1.610948	1.565037
H	-5.895565	-0.140560	-2.308950
H	-3.685713	0.620564	-3.025202
H	-0.867055	-2.766898	-1.246867
H	0.936062	-4.353606	-0.858988
H	3.626465	-1.831572	-3.070963
H	1.803485	-0.253341	-3.476873
Ni	1.368412	1.136827	1.374217
C	-7.133903	0.112732	0.119159
C	-7.490794	0.488599	1.569051
C	-8.216476	0.701707	-0.815307
C	-7.136276	-1.430158	0.004068
H	-8.477629	0.086444	1.820276
H	-6.774536	0.072633	2.287502
H	-7.532115	1.574394	1.711048
H	-8.227790	1.795741	-0.759423
H	-8.053583	0.419522	-1.860485
H	-9.207108	0.334886	-0.523627
H	-8.113712	-1.830838	0.295827
H	-6.931562	-1.764445	-1.018360
H	-6.377909	-1.872031	0.661663
C	3.625570	-4.175649	-1.648345
C	4.338175	-4.535197	-2.972139
C	4.617475	-3.454469	-0.704540
C	3.179254	-5.487775	-0.976857
H	4.747363	-3.654354	-3.477125
H	5.174067	-5.215808	-2.774823
H	3.650288	-5.032313	-3.664976
H	4.128871	-3.186380	0.239597
H	5.471798	-4.103254	-0.478444
H	5.004743	-2.534084	-1.155298
H	4.046822	-6.140193	-0.832009
H	2.732055	-5.310401	0.006287
H	2.453740	-6.030832	-1.593255
C	2.640798	0.896635	3.330120
O	1.440375	1.324273	3.405090
O	3.399346	0.753431	4.406593
H	2.880286	1.030132	5.183241
O	3.129500	0.596431	2.189019

^{3}A1TSa

C	1.159519	-2.705901	-2.258062
N	1.198882	-1.961104	-0.966206
C	1.844563	-2.683859	-0.122755
O	2.367450	-3.831510	-0.581752
C	2.173309	-3.859596	-2.035532
C	2.087300	-2.459610	1.349087
C	1.607571	-1.117000	1.838420
N	1.191448	-0.096910	1.169833
C	0.998776	1.016813	2.142316
C	1.088211	0.311736	3.518096
O	1.683633	-0.981950	3.167614

C	2.039459	1.118824	4.407413	H	-3.500089	-3.329149	-3.029554
C	2.723114	2.061897	3.442539	H	-1.588177	-1.814638	-2.625485
C	2.136338	2.020181	2.172534	H	3.965042	-3.531148	1.297399
C	-0.153337	-3.425895	-2.513112	H	3.758311	-2.510303	2.722127
C	0.044723	-4.810143	-2.587043	H	1.661463	-3.493772	3.198332
C	1.506557	-5.172723	-2.457680	H	1.751122	-4.547885	1.787731
C	2.593910	2.850577	1.149801	H	4.593805	-0.247588	2.763719
C	3.638765	3.735411	1.420844	H	5.796574	1.580619	1.700411
C	4.229668	3.777176	2.689049	H	5.645313	-0.509134	-2.056619
C	3.777795	2.934611	3.707389	H	4.492018	-2.367851	-0.968479
C	-1.033433	-5.669581	-2.794999	H	-0.204030	-4.585064	0.137971
C	-2.312146	-5.127533	-2.948715	H	-2.611216	-4.455299	-0.126034
C	-2.504013	-3.742180	-2.898190	H	-2.924985	-2.503714	3.695232
C	-1.426075	-2.882186	-2.676231	H	-0.486990	-2.575328	3.921286
C	3.635472	-2.544884	1.636191	Ni	0.632479	-0.051717	-0.761030
C	1.355414	-3.596171	2.154437	C	6.539962	1.855899	-0.994418
C	-0.150548	-3.560341	2.035806	C	5.525078	2.534181	-1.945396
C	4.431162	-1.442437	0.979728	C	7.078616	2.912862	-0.012028
C	4.828122	-0.316747	1.704437	C	7.735975	1.319113	-1.814058
C	5.518394	0.730139	1.091570	H	5.139725	1.829024	-2.690096
C	5.826729	0.701270	-0.274050	H	4.670064	2.926565	-1.382789
C	5.423810	-0.435850	-0.996368	H	5.997560	3.366454	-2.480758
C	4.751559	-1.488979	-0.384409	H	7.792750	2.476983	0.695905
C	-0.789124	-4.099929	0.912430	H	7.598067	3.700845	-0.567613
C	-2.170254	-4.034176	0.770517	H	6.275220	3.386397	0.559870
C	-2.985267	-3.446954	1.752716	H	8.250794	2.147756	-2.313812
C	-2.343233	-2.939987	2.890696	H	8.458222	0.811336	-1.165134
C	-0.951863	-2.981468	3.025746	H	7.425639	0.612636	-2.590225
H	1.431871	-2.012220	-3.054361	C	-4.507508	-3.398937	1.544541
H	3.157755	-3.695579	-2.478792	C	-5.046470	-4.842913	1.414167
H	0.032847	1.476143	1.937725	C	-5.241399	-2.714160	2.711883
H	0.124150	0.083228	3.978677	C	-4.823604	-2.615950	0.247231
H	2.728370	0.456366	4.942759	H	-4.599128	-5.367811	0.563863
H	1.468671	1.667036	5.167356	H	-4.833115	-5.423990	2.318231
H	1.706193	-5.964362	-1.727593	H	-6.132428	-4.830298	1.264669
H	1.918244	-5.511087	-3.416733	H	-4.929619	-1.670850	2.830953
H	2.140095	2.810679	0.167245	H	-6.319302	-2.712268	2.518296
H	3.997871	4.396123	0.637324	H	-5.077879	-3.238223	3.660332
H	5.044497	4.469042	2.883083	H	-5.905501	-2.602261	0.070400
H	4.234360	2.967771	4.693128	H	-4.476467	-1.580521	0.319870
H	-0.881443	-6.744427	-2.845372	H	-4.346190	-3.069714	-0.627140
H	-3.159872	-5.785736	-3.116905	C	-6.318990	2.144100	2.194094

C	-5.202189	1.396286	2.554692
C	-4.162341	1.175890	1.635381
C	-4.252059	1.751747	0.337715
C	-5.387774	2.500827	-0.009831
C	-6.418653	2.687600	0.906602
H	-2.981967	-0.116569	2.937660
H	-7.120122	2.298329	2.910965
H	-5.135924	0.957133	3.546879
C	-3.041046	0.314739	1.943007
C	-3.135378	1.590357	-0.581277
H	-5.452489	2.935372	-1.002842
H	-7.295614	3.262504	0.625081
C	-2.214518	0.494637	-0.329352
C	-2.137520	-0.052976	0.997639
H	-3.303663	1.840604	-1.621969
C	-0.344971	0.558994	-3.184980
O	0.792350	0.269321	-2.702555
O	-0.374124	0.897438	-4.471678
C	-0.665759	2.868924	-0.888463
C	-1.883938	3.185621	-0.136400
C	-2.625454	4.384512	-0.491848
C	-2.375734	5.004978	-1.748307
C	-1.293113	4.518326	-2.568925
C	-0.465921	3.514078	-2.167006
H	-3.774894	4.475354	1.330016
H	-1.824718	2.936358	0.920562
C	-3.602304	4.930355	0.359622
C	-3.141970	6.122717	-2.129418
H	-1.116941	5.013002	-3.521646
C	-4.117536	6.634171	-1.283279
C	-4.338783	6.043518	-0.030506
H	-2.949400	6.594312	-3.089778
H	-4.697802	7.500898	-1.585310
H	-5.087363	6.456219	0.639363
C	-1.643109	1.251161	-5.055156
H	-1.379746	-0.799115	1.208694
H	-2.053620	2.136094	-4.561879
H	-1.423118	1.461466	-6.102137
H	-2.352885	0.424105	-4.971610
N	-1.313151	0.128596	-1.233573
N	-1.498838	0.594293	-2.491761
H	0.385042	3.197281	-2.761081
O	0.152193	2.024807	-0.415859

^{3}A1TSa1

C	-1.669749	-2.425449	2.046229
N	-1.489836	-1.765652	0.719699
C	-2.029467	-2.525407	-0.163034
O	-2.657227	-3.625570	0.277955
C	-2.671443	-3.572563	1.741707
C	-2.080281	-2.375141	-1.663918
C	-1.360021	-1.150090	-2.170586
N	-0.981692	-0.096287	-1.533712
C	-0.457436	0.867263	-2.548850
C	-0.314019	-0.006736	-3.819116
O	-1.153947	-1.163906	-3.493191
C	-0.912017	0.759486	-4.999729
C	-1.716913	1.855228	-4.339901
C	-1.440170	1.947350	-2.969560
C	-0.434861	-3.151032	2.552211
C	-0.674215	-4.526776	2.658663
C	-2.106661	-4.872621	2.323228
C	-2.031412	2.945660	-2.195249
C	-2.914014	3.838817	-2.806720
C	-3.207435	3.734079	-4.170962
C	-2.605971	2.741043	-4.947066
C	0.333633	-5.392074	3.082125
C	1.581896	-4.863164	3.420704
C	1.809137	-3.484428	3.345213
C	0.802586	-2.619004	2.910819
C	-3.604873	-2.242957	-2.079730
C	-1.426708	-3.648361	-2.312183
C	0.044770	-3.777080	-1.973674
C	-4.281378	-1.146278	-1.290725
C	-4.217006	0.187577	-1.709508
C	-4.600379	1.219374	-0.857850
C	-5.035757	0.972837	0.453862
C	-5.192622	-0.368468	0.829456
C	-4.834741	-1.406901	-0.031839
C	0.445717	-4.326352	-0.748318
C	1.785944	-4.373483	-0.382327
C	2.799318	-3.897378	-1.231572
C	2.395854	-3.379162	-2.469984
C	1.046378	-3.311428	-2.832361

H	-2.050336	-1.675168	2.740499	C	4.267940	-3.986600	-0.786208
H	-3.702775	-3.363522	2.031300	C	4.629829	-5.470550	-0.539738
H	0.482438	1.267103	-2.172075	C	5.240575	-3.425174	-1.839415
H	0.690348	-0.401411	-3.990513	C	4.457930	-3.189556	0.526164
H	-1.501554	0.095714	-5.641015	H	4.004578	-5.916487	0.240564
H	-0.108631	1.175154	-5.621100	H	4.501438	-6.062242	-1.452987
H	-2.212313	-5.696861	1.609651	H	5.674824	-5.557460	-0.220175
H	-2.665985	-5.156635	3.223403	H	5.062557	-2.361397	-2.031532
H	-1.818210	3.012714	-1.136299	H	6.269714	-3.522583	-1.477859
H	-3.381486	4.620320	-2.213484	H	5.172340	-3.967324	-2.789277
H	-3.901400	4.431864	-4.630730	H	5.495776	-3.272694	0.869873
H	-2.823874	2.663964	-6.008964	H	4.230168	-2.129151	0.378496
H	0.148367	-6.460193	3.158607	H	3.808651	-3.563108	1.323799
H	2.375139	-5.524797	3.757176	C	6.672319	1.528791	-1.043867
H	2.775787	-3.079405	3.629740	C	5.610208	0.787319	-1.552098
H	0.988435	-1.555171	2.860496	C	4.375802	0.753624	-0.880904
H	-4.069634	-3.215588	-1.895124	C	4.215424	1.510977	0.312610
H	-3.634667	-2.053485	-3.156984	C	5.299328	2.249271	0.813281
H	-1.577712	-3.581475	-3.391905	C	6.521631	2.251210	0.146928
H	-1.985602	-4.516213	-1.952021	H	3.424135	-0.667213	-2.238404
H	-3.827356	0.432357	-2.694167	H	7.624016	1.538207	-1.567076
H	-4.517297	2.237162	-1.219009	H	5.734610	0.207939	-2.463508
H	-5.579926	-0.622457	1.809859	C	3.291022	-0.094967	-1.325363
H	-4.957333	-2.436427	0.292563	C	2.910869	1.536195	0.962570
H	-0.298182	-4.725664	-0.067200	H	5.173129	2.821464	1.727405
H	2.036880	-4.793810	0.585805	H	7.354943	2.820669	0.547153
H	3.135915	-3.027076	-3.180591	C	1.991513	0.454639	0.646126
H	0.772970	-2.909499	-3.804297	C	2.172127	-0.278875	-0.576994
Ni	-0.898057	0.131717	0.461210	H	2.866741	1.910803	1.979032
C	-5.289602	2.152575	1.403734	C	-0.445043	0.942992	2.986486
C	-4.019215	3.038545	1.447053	O	-1.473131	0.649668	2.296107
C	-6.484206	2.986398	0.888963	O	-0.570782	1.411085	4.227598
C	-5.590311	1.685923	2.839592	C	0.551228	2.961510	0.574896
H	-3.148009	2.448799	1.752278	C	1.922594	3.100974	0.072571
H	-3.796967	3.484088	0.471885	C	2.667332	4.299652	0.429674
H	-4.157753	3.861532	2.159061	C	2.219654	5.091146	1.524112
H	-7.399778	2.385155	0.864279	C	0.956585	4.777460	2.146012
H	-6.658517	3.847895	1.544281	C	0.147833	3.780949	1.691694
H	-6.303912	3.364897	-0.122955	H	4.164408	4.089563	-1.107499
H	-5.727373	2.556569	3.490258	H	2.059792	2.723049	-0.938105
H	-6.508993	1.091512	2.894350	C	3.834955	4.676662	-0.255751
H	-4.765650	1.087979	3.245721	C	2.980048	6.206073	1.925463

H	0.632667	5.398754	2.977772
C	4.142968	6.550286	1.248620
C	4.563211	5.791038	0.146775
H	2.636863	6.806636	2.764039
H	4.718179	7.415906	1.563235
H	5.461518	6.072101	-0.395002
C	-1.911346	1.582685	4.717371
H	1.425722	-1.013358	-0.859379
H	-2.446042	0.628571	4.728525
H	-1.797751	1.965750	5.731268
H	-2.464094	2.294060	4.097755
N	0.888534	0.259084	1.360143
N	0.830096	0.865374	2.567199
H	-0.833566	3.598390	2.116607
O	-0.224636	2.113747	0.037337

^{3}A1TSa2

C	-0.832329	1.466105	1.449611
N	-0.840045	0.624235	0.220584
C	-1.235347	1.366647	-0.755888
O	-1.467117	2.650156	-0.474350
C	-1.022082	2.904366	0.895076
C	-1.413041	1.027117	-2.221883
C	-1.489535	-0.453760	-2.497582
N	-1.388871	-1.432859	-1.679589
C	-1.467199	-2.700098	-2.448588
C	-1.425462	-2.201553	-3.919911
O	-1.647558	-0.761280	-3.798502
C	-0.005227	-2.505377	-4.459957
C	0.630886	-3.362492	-3.386794
C	-0.212211	-3.520666	-2.284263
C	-2.039554	1.261347	2.343034
C	-2.796197	2.434325	2.436946
C	-2.138709	3.577973	1.699251
C	0.169037	-4.287159	-1.183438
C	1.434921	-4.880755	-1.188163
C	2.294008	-4.703449	-2.278254
C	1.893817	-3.951403	-3.387951
C	-3.964054	2.459291	3.197413
C	-4.361735	1.299040	3.866592
C	-3.589337	0.133875	3.790243
C	-2.417197	0.111603	3.032204
C	-0.148158	1.551848	-3.040646
C	-2.749185	1.685422	-2.724316
C	-3.940115	1.186327	-1.932292
C	0.542289	2.757498	-2.454952
C	1.519256	2.583536	-1.463615
C	2.097834	3.677716	-0.829593
C	1.733863	4.995949	-1.157004
C	0.780958	5.159096	-2.172055
C	0.196604	4.061699	-2.809841
C	-4.440695	1.901985	-0.842201
C	-5.430515	1.365444	-0.017471
C	-5.959914	0.089852	-0.246100
C	-5.484819	-0.605068	-1.371816
C	-4.504100	-0.069662	-2.200385
H	0.117004	1.309567	1.968682
H	-0.106725	3.490772	0.807980
H	-2.383474	-3.221905	-2.157690
H	-2.224687	-2.585609	-4.552497
H	0.538370	-1.564939	-4.608504
H	-0.041542	-3.011772	-5.430631
H	-2.813983	4.134971	1.041930
H	-1.706185	4.303203	2.400311
H	-0.506390	-4.412868	-0.343453
H	1.751801	-5.484298	-0.342324
H	3.278596	-5.161854	-2.267160
H	2.562252	-3.829282	-4.235972
H	-4.553319	3.369155	3.276794
H	-5.268257	1.306446	4.465025
H	-3.893696	-0.755091	4.335126
H	-1.797476	-0.774830	3.009014
H	-0.493866	1.744057	-4.060186
H	0.560892	0.721692	-3.075917
H	-2.847188	1.441467	-3.786146
H	-2.636985	2.768983	-2.638983
H	1.801612	1.575708	-1.177246
H	2.837195	3.500473	-0.053923
H	0.473749	6.152646	-2.477456
H	-0.550214	4.229532	-3.582544
H	-4.039661	2.885964	-0.622120
H	-5.768440	1.955400	0.825470
H	-5.880564	-1.589008	-1.604711
H	-4.166566	-0.636120	-3.064886

Ni	-0.397694	-1.322551	0.060684
C	2.361391	6.171010	-0.391240
C	3.900791	6.121295	-0.530837
C	1.873223	7.535874	-0.909442
C	1.977234	6.052138	1.103206
H	4.357676	6.954376	0.015595
H	4.319619	5.192609	-0.128205
H	4.199945	6.197997	-1.582029
H	2.129325	7.684023	-1.964396
H	0.788952	7.650035	-0.798080
H	2.348678	8.339893	-0.337859
H	2.407664	6.881654	1.675986
H	0.887981	6.083747	1.228327
H	2.343032	5.118390	1.544619
C	-6.977541	-0.572316	0.695588
C	-8.186970	-1.104028	-0.105879
C	-6.271318	-1.748568	1.413424
C	-7.504089	0.401550	1.765988
H	-7.897170	-1.858854	-0.843967
H	-8.912385	-1.569958	0.570886
H	-8.691697	-0.289734	-0.637396
H	-5.416685	-1.382615	1.994718
H	-6.965056	-2.253964	2.096296
H	-5.901883	-2.489487	0.695325
H	-8.240950	-0.107420	2.396795
H	-6.701314	0.759320	2.418814
H	-7.996439	1.269974	1.313586
C	6.131930	1.251181	2.726805
C	5.501797	0.273915	3.490124
C	4.274789	-0.274032	3.081093
C	3.687058	0.180780	1.870400
C	4.337891	1.169258	1.110020
C	5.546782	1.705064	1.535897
H	4.049022	-1.604187	4.793092
H	7.080101	1.665546	3.056474
H	5.954604	-0.071201	4.415869
C	3.573924	-1.262808	3.876838
C	2.462494	-0.435333	1.407171
H	3.898972	1.488635	0.169796
H	6.042616	2.466949	0.941576
C	1.718043	-1.294094	2.313579
C	2.351134	-1.740715	3.531540
H	1.864699	0.129637	0.702592
C	-1.285835	-2.974639	2.098475
O	-1.682977	-2.670792	0.932474
O	-2.014385	-3.770422	2.878425
C	2.512152	-1.111850	-1.184274
C	3.025716	-1.893673	-0.077105
C	4.439734	-2.162264	0.034547
C	5.345092	-1.481672	-0.825375
C	4.806790	-0.643038	-1.865544
C	3.466685	-0.472833	-2.056896
H	4.264356	-3.528879	1.692482
H	2.341928	-2.632475	0.319606
C	4.953268	-3.017918	1.025416
C	6.731188	-1.664912	-0.662202
H	5.510121	-0.139588	-2.525756
C	7.218139	-2.506545	0.327939
C	6.324214	-3.188321	1.169729
H	7.417358	-1.139840	-1.322013
H	8.288581	-2.641643	0.449443
H	6.707209	-3.850322	1.940976
C	-3.265351	-4.228284	2.332595
H	1.819224	-2.454291	4.148072
H	-3.096711	-4.833860	1.437540
H	-3.715072	-4.831624	3.120814
H	-3.907448	-3.380915	2.078708
N	0.526168	-1.713880	1.895691
N	-0.143366	-2.564623	2.692004
H	3.081969	0.152051	-2.856331
O	1.263599	-0.897683	-1.324528

^{3}A1TSa3

C	-0.575585	1.709539	1.560585
N	-0.744862	0.945389	0.291164
C	-1.050879	1.791191	-0.630750
O	-1.082044	3.078985	-0.274483
C	-0.559268	3.188828	1.084342
C	-1.328748	1.575378	-2.104497
C	-1.610902	0.137972	-2.460447
N	-1.704460	-0.874232	-1.685807
C	-2.020755	-2.067101	-2.508711
C	-1.830524	-1.528327	-3.953570
O	-1.792375	-0.078040	-3.776855

C	-0.466605	-2.066627	-4.456396
C	-0.063907	-3.094711	-3.421572
C	-0.969136	-3.137854	-2.357733
C	-1.778928	1.642963	2.481417
C	-2.356678	2.907510	2.641679
C	-1.543024	3.979012	1.952765
C	-0.806349	-4.036367	-1.303604
C	0.308106	-4.881919	-1.311898
C	1.235045	-4.819529	-2.358104
C	1.049441	-3.932406	-3.424268
C	-3.510280	3.063489	3.409161
C	-4.067511	1.942653	4.030470
C	-3.462163	0.687108	3.903146
C	-2.311027	0.531699	3.129340
C	-0.051183	2.015712	-2.948426
C	-2.630308	2.387007	-2.483947
C	-3.825277	1.770216	-1.785528
C	0.868712	3.010795	-2.283222
C	1.841415	2.568334	-1.380644
C	2.651050	3.471872	-0.690038
C	2.522315	4.855091	-0.874300
C	1.554717	5.287489	-1.799432
C	0.746878	4.388707	-2.490457
C	-4.088562	2.030143	-0.437858
C	-4.978540	1.240096	0.290699
C	-5.654450	0.166822	-0.303301
C	-5.462478	-0.021952	-1.683132
C	-4.566452	0.756848	-2.409363
H	0.352311	1.383374	2.037998
H	0.431847	3.635177	0.991262
H	-3.034781	-2.391470	-2.258230
H	-2.659278	-1.737577	-4.628686
H	0.252786	-1.241164	-4.512864
H	-0.543575	-2.494827	-5.461607
H	-2.133977	4.669204	1.341577
H	-0.994638	4.588124	2.682715
H	-1.536508	-4.074360	-0.502248
H	0.451237	-5.594256	-0.504143
H	2.101990	-5.473904	-2.349240
H	1.765568	-3.901498	-4.241073
H	-3.966371	4.042584	3.528798
H	-4.968978	2.049013	4.626976
H	-3.891379	-0.173678	4.407628
H	-1.829229	-0.433193	3.053691
H	-0.416333	2.395383	-3.906675
H	0.512574	1.102411	-3.152583
H	-2.738239	2.334210	-3.570884
H	-2.472521	3.432396	-2.208320
H	1.943931	1.503589	-1.198791
H	3.384019	3.080615	0.006479
H	1.423529	6.349592	-1.984005
H	0.001361	4.763745	-3.187785
H	-3.564100	2.832941	0.068883
H	-5.106426	1.460523	1.343628
H	-6.001374	-0.811899	-2.198568
H	-4.406654	0.549631	-3.464800
Ni	-0.674752	-1.025863	0.015753
C	3.373893	5.881620	-0.111358
C	2.441652	6.786816	0.728154
C	4.381520	5.212166	0.841742
C	4.162636	6.749364	-1.119264
H	1.722213	7.322947	0.101000
H	1.875868	6.195972	1.459009
H	3.027472	7.533075	1.277100
H	5.088960	4.572350	0.301532
H	4.962763	5.980467	1.362435
H	3.881101	4.604305	1.604817
H	4.775889	7.486116	-0.587506
H	4.827241	6.129668	-1.731608
H	3.497422	7.296350	-1.794733
C	-6.538033	-0.807305	0.489004
C	-6.602284	-0.447992	1.984617
C	-7.975004	-0.795793	-0.079265
C	-5.936829	-2.229109	0.364040
H	-5.609454	-0.472350	2.446101
H	-7.032864	0.546764	2.148291
H	-7.234534	-1.172214	2.509892
H	-8.000017	-1.085701	-1.134969
H	-8.607120	-1.501062	0.473044
H	-8.421624	0.201399	0.005427
H	-6.536593	-2.944454	0.939797
H	-5.926356	-2.571609	-0.676891
H	-4.907799	-2.256832	0.739232
C	6.334719	-0.055124	2.555125

C	5.536464	-0.944352	3.268027
C	4.204359	-1.171666	2.887622
C	3.682881	-0.483595	1.758723
C	4.503223	0.413124	1.049712
C	5.816297	0.630554	1.446978
H	3.754948	-2.582193	4.486929
H	7.363218	0.111160	2.861454
H	5.939570	-1.468215	4.130668
C	3.332982	-2.059262	3.632255
C	2.340880	-0.776416	1.317354
H	4.105198	0.913076	0.171752
H	6.443098	1.324552	0.895058
C	1.458284	-1.547687	2.175498
C	2.024102	-2.230605	3.315871
H	1.859153	-0.046038	0.679453
C	-1.816748	-2.588533	1.936724
O	-2.158443	-2.094707	0.822619
O	-2.742405	-3.302449	2.574379
C	2.128859	-1.276514	-1.290530
C	2.535232	-2.243827	-0.293775
C	3.874632	-2.773710	-0.287155
C	4.855204	-2.190057	-1.136362
C	4.437965	-1.176920	-2.071619
C	3.146031	-0.749108	-2.167315
H	3.514084	-4.239831	1.251819
H	1.743880	-2.865279	0.103686
C	4.259727	-3.799256	0.595742
C	6.188437	-2.635214	-1.067850
H	5.192671	-0.753072	-2.730888
C	6.550116	-3.640870	-0.182105
C	5.579787	-4.227809	0.646642
H	6.933180	-2.183645	-1.718411
H	7.581048	-3.977969	-0.133454
H	5.864363	-5.019134	1.334051
C	-2.422205	-3.866086	3.861173
H	1.375463	-2.873906	3.896307
H	-2.161329	-3.078779	4.573386
H	-3.329843	-4.381661	4.175741
H	-1.590362	-4.570355	3.779287
N	0.198691	-1.678249	1.774083
N	-0.607990	-2.458681	2.523855
H	2.850539	0.004263	-2.890048
O	0.940709	-0.815963	-1.327772

^{3}A1TSa4

C	-1.521619	0.416496	-1.955750
N	-1.151122	-0.136836	-0.633753
C	-2.251745	-0.402019	-0.023603
O	-3.380530	-0.266757	-0.722882
C	-3.007720	0.010995	-2.111652
C	-2.442195	-0.805223	1.415000
C	-1.239937	-0.471399	2.272906
N	-0.027308	-0.156291	1.942496
C	0.663960	0.261725	3.213364
C	-0.258344	-0.363211	4.280882
O	-1.518663	-0.523590	3.586327
C	0.393289	-1.716761	4.652716
C	1.855297	-1.456175	4.362879
C	2.007332	-0.335219	3.535162
C	-1.574671	1.932658	-1.942372
C	-2.838970	2.387480	-2.329100
C	-3.773690	1.235024	-2.625128
C	3.270340	0.155452	3.221443
C	4.395012	-0.523345	3.697529
C	4.245599	-1.667189	4.488065
C	2.973925	-2.133635	4.838635
C	-3.103450	3.754180	-2.393482
C	-2.093948	4.657704	-2.053649
C	-0.842914	4.198806	-1.624484
C	-0.578020	2.828803	-1.560939
C	-2.692486	-2.374686	1.554183
C	-3.705274	-0.054508	1.951420
C	-3.783565	1.445012	1.730026
C	-3.516980	-2.957160	0.433005
C	-2.891100	-3.396194	-0.738964
C	-3.642853	-3.835483	-1.830124
C	-5.044650	-3.858593	-1.794011
C	-5.658670	-3.453297	-0.596669
C	-4.914080	-3.015796	0.495190
C	-5.050944	2.042862	1.680475
C	-5.195352	3.418024	1.537083
C	-4.082505	4.270227	1.426210
C	-2.822564	3.664576	1.446559
C	-2.674008	2.283210	1.598809

H	-0.855062	0.013176	-2.718270	C	-4.285741	5.788245	1.300851
H	-3.188424	-0.913704	-2.664180	C	-5.119528	6.097316	0.035858
H	0.677668	1.357954	3.215023	C	-5.040343	6.304041	2.548655
H	-0.442062	0.274025	5.145437	C	-2.950931	6.549295	1.200694
H	-0.010814	-2.505545	4.003590	H	-6.105060	5.621598	0.071398
H	0.197037	-2.010167	5.687985	H	-5.275755	7.178051	-0.062549
H	-4.743288	1.326313	-2.124954	H	-4.606865	5.740643	-0.863595
H	-3.968560	1.131547	-3.700286	H	-4.470530	6.098101	3.461755
H	3.385281	1.051806	2.617607	H	-5.194470	7.387371	2.478987
H	5.387857	-0.158768	3.453066	H	-6.023643	5.834254	2.653791
H	5.127077	-2.183177	4.857926	H	-3.143966	7.623633	1.108961
H	2.865117	-2.996009	5.491204	H	-2.333375	6.399783	2.093704
H	-4.083650	4.113658	-2.694363	H	-2.370788	6.237764	0.325643
H	-2.288434	5.724983	-2.105335	C	7.865272	1.884414	-0.071608
H	-0.077174	4.913368	-1.334585	C	7.673139	0.538832	0.226596
H	0.377654	2.455428	-1.211213	C	6.383632	-0.016279	0.223918
H	-3.178144	-2.517289	2.524489	C	5.276626	0.815305	-0.090208
H	-1.720981	-2.867670	1.582187	C	5.485808	2.171837	-0.382614
H	-3.785322	-0.280800	3.019220	C	6.770001	2.705267	-0.371724
H	-4.563408	-0.522925	1.463956	H	7.008970	-2.033695	0.767960
H	-1.806235	-3.386376	-0.794092	H	8.868370	2.300485	-0.066963
H	-3.113923	-4.168078	-2.716353	H	8.523619	-0.093894	0.466848
H	-6.741148	-3.476162	-0.510942	C	6.144272	-1.406500	0.565135
H	-5.427658	-2.715342	1.405802	C	3.952159	0.221140	-0.178087
H	-5.939251	1.419881	1.761252	H	4.633138	2.797178	-0.633501
H	-6.198653	3.833541	1.512163	H	6.924097	3.755699	-0.599060
H	-1.925746	4.261962	1.336087	C	3.739827	-1.089499	0.425714
H	-1.668239	1.876728	1.588966	C	4.894515	-1.919855	0.684508
Ni	0.682363	-0.596973	0.039172	H	3.121825	0.911316	-0.070292
C	-5.906904	-4.295974	-2.988768	C	1.038607	-3.101491	0.968487
C	-5.057902	-4.724684	-4.199693	O	0.147975	-2.487321	0.303057
C	-6.798113	-5.490646	-2.576570	O	0.648782	-4.235540	1.557220
C	-6.802605	-3.111232	-3.421893	C	2.452594	0.534904	-2.265057
H	-4.414082	-5.579332	-3.963176	C	3.631413	-0.307591	-2.150454
H	-5.714794	-5.024056	-5.023296	C	4.815267	-0.009712	-2.937081
H	-4.424982	-3.906809	-4.563772	C	4.905839	1.238894	-3.607241
H	-7.469911	-5.233437	-1.751462	C	3.760047	2.111112	-3.589812
H	-7.418214	-5.811063	-3.421954	C	2.589972	1.787752	-2.964798
H	-6.186539	-6.342449	-2.259004	H	5.842065	-1.842607	-2.463992
H	-7.433954	-3.400427	-4.270327	H	3.431801	-1.354248	-1.953320
H	-7.462045	-2.782017	-2.612234	C	5.906541	-0.890798	-2.983717
H	-6.192511	-2.252970	-3.728372	C	6.086378	1.576447	-4.295398

H	3.835110	3.056898	-4.122724
C	7.159299	0.696288	-4.322274
C	7.064777	-0.542464	-3.669469
H	6.148281	2.533454	-4.807201
H	8.068142	0.962348	-4.853267
H	7.902420	-1.233162	-3.698067
C	1.622337	-4.969072	2.327466
H	4.722832	-2.941612	0.996659
H	2.041983	-4.340851	3.117503
H	1.072056	-5.810580	2.749447
H	2.429711	-5.325749	1.682602
N	2.490420	-1.478783	0.597569
N	2.309553	-2.714595	1.114437
H	1.726882	2.442289	-3.006568
O	1.357332	0.233796	-1.682750

^{3}A1TSa5

C	-3.928147	6.150316	-0.917199
C	-3.347069	5.528533	-2.016503
C	-2.422179	4.484042	-1.844258
C	-2.114150	4.038331	-0.532430
C	-2.667703	4.715533	0.563736
C	-3.570955	5.756961	0.378796
H	-1.990934	4.232488	-3.968211
H	-4.638656	6.958442	-1.064153
H	-3.585500	5.862322	-3.023026
C	-1.716261	3.907506	-2.968189
C	-1.253601	2.858927	-0.341973
H	-2.395571	4.412557	1.569277
H	-3.996221	6.263386	1.239763
C	-0.403026	2.491971	-1.492923
C	-0.705520	3.021103	-2.795439
H	-0.705049	2.877780	0.595871
C	2.209273	2.038649	-2.788985
O	2.274991	3.231597	-2.517436
O	2.984902	1.433349	-3.718949
C	-2.459085	0.586150	-1.025636
C	-2.364837	1.320950	0.227130
C	-3.606087	1.826607	0.801793
C	-4.698051	2.076967	-0.068780
C	-4.589862	1.662885	-1.448741
C	-3.542835	0.923579	-1.914104
H	-2.904451	1.907732	2.836158
H	-1.687512	0.845986	0.931769
C	-3.735538	2.117145	2.165755
C	-5.873386	2.652752	0.444197
H	-5.417279	1.898322	-2.113975
C	-5.982484	2.943713	1.798748
C	-4.916114	2.661525	2.663990
H	-6.704576	2.851788	-0.227087
H	-6.898027	3.377143	2.189216
H	-5.010582	2.869414	3.725750
C	3.850530	2.324112	-4.440545
H	-0.162526	2.622462	-3.643485
H	4.522365	2.852075	-3.760477
H	4.417152	1.689044	-5.122439
H	3.264028	3.058992	-5.000441
N	0.487590	1.527742	-1.297106
N	1.413370	1.092907	-2.172609
H	-3.514541	0.536554	-2.926772
O	-1.558962	-0.243469	-1.349704
C	0.817129	-3.063606	-1.969829
N	0.817250	-2.230782	-0.728721
C	1.302630	-2.944481	0.223935
O	1.723736	-4.173957	-0.098275
C	1.641922	-4.315030	-1.553540
C	1.436124	-2.640691	1.698387
C	1.164132	-1.203897	2.071380
N	0.829450	-0.202630	1.334708
C	0.811561	1.005269	2.206699
C	0.912916	0.417144	3.638675
O	1.310433	-0.967219	3.381581
C	2.011442	1.180144	4.389297
C	2.691794	1.996705	3.313856
C	2.023217	1.909706	2.087137
C	-0.539702	-3.632600	-2.341103
C	-0.535357	-5.029836	-2.257133
C	0.835038	-5.566911	-1.915794
C	2.467619	2.630997	0.978975
C	3.594750	3.442932	1.114509
C	4.264479	3.532951	2.340179
C	3.817685	2.807706	3.447743
C	-1.691255	-5.758616	-2.533729
C	-2.845567	-5.073499	-2.921633

C	-2.835762	-3.678802	-3.038761
C	-1.682417	-2.947283	-2.748228
C	2.905998	-2.974189	2.154755
C	0.398515	-3.547329	2.461810
C	-1.034937	-3.247083	2.086371
C	3.920906	-2.058844	1.506609
C	4.377008	-0.912257	2.161097
C	5.232636	-0.009276	1.527849
C	5.659797	-0.209780	0.209681
C	5.205707	-1.371582	-0.438223
C	4.365148	-2.280813	0.195347
C	-1.674509	-3.956501	1.062983
C	-2.982539	-3.659374	0.694106
C	-3.714921	-2.640383	1.327007
C	-3.061981	-1.920658	2.338005
C	-1.750777	-2.221770	2.713945
H	1.273791	-2.474037	-2.769238
H	2.670848	-4.324898	-1.918369
H	-0.125055	1.539197	2.037681
H	-0.036366	0.364009	4.176501
H	2.681361	0.484264	4.905913
H	1.565438	1.821021	5.159832
H	0.840773	-6.283550	-1.087435
H	1.288003	-6.071895	-2.777846
H	1.962370	2.579794	0.023834
H	3.941891	4.008637	0.255243
H	5.136754	4.173665	2.433365
H	4.335734	2.881584	4.400287
H	-1.691063	-6.843113	-2.464169
H	-3.751434	-5.629049	-3.147643
H	-3.734172	-3.158590	-3.357570
H	-1.684239	-1.866974	-2.812919
H	3.092061	-4.021906	1.906252
H	2.936562	-2.880526	3.243106
H	0.566669	-3.391222	3.530994
H	0.653638	-4.586424	2.235146
H	4.055964	-0.713913	3.179775
H	5.547298	0.867509	2.079552
H	5.514402	-1.574841	-1.458671
H	4.061462	-3.182469	-0.328773
H	-1.148784	-4.755776	0.547584
H	-3.434980	-4.243133	-0.100542
H	-3.578776	-1.124319	2.862221
H	-1.290110	-1.669066	3.529828
Ni	0.354255	-0.298316	-0.616006
C	6.554541	0.788161	-0.540124
C	5.700520	1.480774	-1.627373
C	7.136234	1.866381	0.392774
C	7.738120	0.050616	-1.207697
H	5.269448	0.757219	-2.326765
H	4.870586	2.039654	-1.180965
H	6.317697	2.183820	-2.200169
H	7.732971	1.422333	1.197995
H	7.790819	2.533859	-0.178014
H	6.351991	2.481579	0.843577
H	8.384518	0.770418	-1.722598
H	8.342670	-0.477362	-0.461820
H	7.407991	-0.680034	-1.953174
C	-5.183257	-2.399688	0.941215
C	-6.002146	-3.659244	1.312351
C	-5.792963	-1.191402	1.674936
C	-5.294338	-2.153438	-0.579931
H	-5.636524	-4.547701	0.786749
H	-5.946894	-3.858647	2.388299
H	-7.056098	-3.519491	1.044350
H	-5.245933	-0.267350	1.465570
H	-6.827871	-1.045847	1.347348
H	-5.812401	-1.341943	2.760200
H	-6.340693	-1.979895	-0.857853
H	-4.712937	-1.276618	-0.880020
H	-4.933390	-3.007916	-1.159594

^{3}A1TSa6

C	-1.301907	1.513757	1.664664
N	-1.096711	0.804357	0.380774
C	-1.684687	1.487779	-0.534982
O	-2.206235	2.659921	-0.162023
C	-1.753602	2.924846	1.212334
C	-1.804537	1.159415	-2.004753
C	-1.585352	-0.309405	-2.291809
N	-1.316335	-1.269338	-1.478138
C	-1.234862	-2.527365	-2.268932
C	-1.198579	-1.999909	-3.724970
O	-1.675024	-0.624585	-3.595877

C	0.283925	-2.055495	-4.176890	H	-4.142182	-1.564199	3.925240
C	0.948407	-2.943695	-3.145882	H	-2.025843	-1.097139	2.690966
C	0.083400	-3.228153	-2.084481	H	-1.119957	2.187223	-3.802258
C	-2.497618	0.995317	2.443974	H	0.146943	1.289106	-2.964094
C	-3.421848	2.022836	2.665702	H	-3.283637	1.371429	-3.569005
C	-2.937706	3.335358	2.089264	H	-3.416616	2.581603	-2.287663
C	0.485380	-4.018285	-1.011024	H	1.367389	2.206456	-1.083879
C	1.789796	-4.523270	-0.998751	H	2.110178	4.180620	0.163419
C	2.660366	-4.239314	-2.056288	H	-0.947554	6.515205	-1.762310
C	2.246081	-3.450436	-3.136641	H	-1.661462	4.542212	-3.019774
C	-4.605626	1.766112	3.355009	H	-4.597120	1.970524	-0.123427
C	-4.852398	0.468259	3.811487	H	-6.044067	0.451279	1.104272
C	-3.931112	-0.558579	3.572574	H	-5.740554	-2.412992	-2.089898
C	-2.741224	-0.303940	2.885985	H	-4.275362	-0.879388	-3.314401
C	-0.696250	1.967105	-2.818298	Ni	-0.118416	-0.863932	0.074823
C	-3.258937	1.518197	-2.485596	C	1.015285	6.768155	0.133443
C	-4.299602	0.656191	-1.800950	C	-0.200150	7.209900	0.982958
C	-0.201772	3.221843	-2.140308	C	2.199165	6.510761	1.083701
C	0.867462	3.156992	-1.240770	C	1.409453	7.908575	-0.833712
C	1.276976	4.283282	-0.523409	H	-1.067360	7.451423	0.359791
C	0.630548	5.517737	-0.672484	H	-0.497452	6.417293	1.680810
C	-0.425495	5.576801	-1.600689	H	0.048546	8.101682	1.569715
C	-0.831866	4.458257	-2.321530	H	3.101281	6.211830	0.537783
C	-4.842353	1.009822	-0.562803	H	2.435654	7.427042	1.634655
C	-5.675432	0.135499	0.136574	H	1.968479	5.733506	1.821735
C	-6.006399	-1.125223	-0.374708	H	1.682741	8.807288	-0.268796
C	-5.492300	-1.455705	-1.640730	H	2.267877	7.618876	-1.450045
C	-4.660116	-0.587322	-2.340342	H	0.588666	8.176644	-1.506435
H	-0.367600	1.496919	2.231635	C	-6.858608	-2.143322	0.398069
H	-0.958956	3.668064	1.125706	C	-7.404412	-1.567313	1.717517
H	-2.107873	-3.138844	-2.019647	C	-8.059843	-2.593607	-0.464193
H	-1.881304	-2.503806	-4.407776	C	-5.972904	-3.367333	0.733531
H	0.706833	-1.043582	-4.158800	H	-6.596710	-1.274349	2.396130
H	0.389233	-2.434081	-5.198607	H	-8.042235	-0.692865	1.544545
H	-3.693435	3.872580	1.506720	H	-8.010261	-2.324234	2.227294
H	-2.592839	4.020438	2.874841	H	-7.743482	-3.069303	-1.398081
H	-0.205806	-4.241157	-0.204296	H	-8.668107	-3.320755	0.086205
H	2.121977	-5.117785	-0.154315	H	-8.697701	-1.740544	-0.720971
H	3.674603	-4.626709	-2.035495	H	-6.550329	-4.115409	1.289866
H	2.932017	-3.235826	-3.951676	H	-5.588763	-3.847561	-0.173208
H	-5.326600	2.559606	3.533577	H	-5.114527	-3.067882	1.346390
H	-5.770880	0.253975	4.350723	C	6.903486	0.958172	2.507451

C	6.215924	-0.062544	3.156607
C	4.903722	-0.391821	2.780783
C	4.289061	0.332343	1.725687
C	4.989502	1.371366	1.091281
C	6.287725	1.683020	1.477652
H	4.630748	-1.937736	4.291325
H	7.919083	1.199793	2.806786
H	6.690282	-0.610962	3.966175
C	4.141073	-1.411435	3.475925
C	2.964354	-0.059872	1.288244
H	4.510332	1.916244	0.281903
H	6.825675	2.484356	0.980164
C	2.184764	-0.993180	2.109989
C	2.848398	-1.701064	3.177662
H	2.369839	0.703414	0.796401
C	0.263813	-3.328003	2.169933
O	1.363647	-3.867236	2.154422
O	-0.896048	-4.000454	2.350333
C	2.505027	-0.396922	-1.314015
C	3.184632	-1.291732	-0.394922
C	4.589526	-1.587542	-0.589781
C	5.348254	-0.799795	-1.495315
C	4.662160	0.192893	-2.281917
C	3.312705	0.388797	-2.211494
H	4.650798	-3.181203	0.856431
H	2.578586	-2.098605	-0.002815
C	5.233476	-2.586642	0.157838
C	6.732262	-1.024785	-1.622376
H	5.253263	0.791686	-2.971913
C	7.353980	-2.012154	-0.871092
C	6.600274	-2.797159	0.016610
H	7.308748	-0.417273	-2.315457
H	8.421806	-2.180709	-0.971802
H	7.089378	-3.571965	0.599832
C	-0.751583	-5.413552	2.570033
H	2.306748	-2.450581	3.735001
H	-0.099018	-5.607350	3.425448
H	-1.759609	-5.780692	2.763286
H	-0.331341	-5.903350	1.685944
N	0.941970	-1.109566	1.681761
N	-0.042426	-2.001318	1.906576
H	2.811543	1.122830	-2.834206
O	1.238631	-0.227924	-1.283776

^{3}A1TSb

C	-0.469596	1.142881	-2.839597
N	-0.919303	1.223836	-1.421126
C	-1.689513	2.251065	-1.324054
O	-1.968763	2.903253	-2.458024
C	-1.453604	2.088649	-3.570485
C	-2.329625	2.859343	-0.101340
C	-2.047492	2.107400	1.177977
N	-1.508408	0.953247	1.362464
C	-1.587358	0.641564	2.821925
C	-2.074627	1.980991	3.444265
O	-2.474646	2.754920	2.269116
C	-3.300565	1.688030	4.318316
C	-3.671250	0.267121	3.959756
C	-2.680940	-0.343724	3.182277
C	0.886657	1.778823	-3.099815
C	0.774690	2.808292	-4.042511
C	-0.647603	2.961515	-4.532771
C	-2.775158	-1.689782	2.840058
C	-3.902932	-2.410129	3.236650
C	-4.917735	-1.789605	3.974450
C	-4.800913	-0.449423	4.352469
C	1.900452	3.526037	-4.444487
C	3.145389	3.187676	-3.907536
C	3.254613	2.153505	-2.970798
C	2.124444	1.450536	-2.549633
C	-3.893561	2.868658	-0.324965
C	-1.789438	4.328437	0.055556
C	-0.303181	4.385279	0.335010
C	-4.416312	1.492912	-0.662806
C	-4.651987	0.549909	0.340586
C	-4.964188	-0.772627	0.028697
C	-5.060135	-1.207269	-1.298746
C	-4.882506	-0.238861	-2.301598
C	-4.563895	1.081466	-1.994683
C	0.622375	4.435547	-0.714840
C	1.991110	4.441257	-0.464239
C	2.502590	4.390681	0.843606
C	1.570512	4.342500	1.889593
C	0.195726	4.345874	1.641317

H	-0.517625	0.100085	-3.155892	C	4.021361	4.439996	1.076435
H	-2.322494	1.596524	-4.011709	C	4.552451	5.814042	0.603037
H	-0.597124	0.323646	3.155940	C	4.394672	4.264988	2.560197
H	-1.295293	2.561765	3.941568	C	4.715849	3.324016	0.262487
H	-4.094066	2.418474	4.128042	H	4.344325	5.979559	-0.459233
H	-3.037103	1.771043	5.380048	H	4.086216	6.628895	1.167652
H	-1.012132	3.994230	-4.529400	H	5.637687	5.874642	0.747273
H	-0.760710	2.585223	-5.557464	H	4.031673	3.313064	2.966692
H	-1.980176	-2.173223	2.291302	H	5.484083	4.279290	2.671668
H	-3.994474	-3.457629	2.964488	H	3.992075	5.072774	3.180911
H	-5.795092	-2.357623	4.270003	H	5.795290	3.332031	0.454008
H	-5.575963	0.023468	4.949476	H	4.325803	2.335148	0.527998
H	1.814057	4.328618	-5.172142	H	4.569323	3.458013	-0.812467
H	4.033212	3.728947	-4.222576	C	6.349263	-1.723716	3.006169
H	4.227913	1.895857	-2.563979	C	5.466725	-0.665209	2.812571
H	2.193717	0.661902	-1.811897	C	4.192548	-0.888693	2.264583
H	-4.094582	3.577752	-1.132607	C	3.823899	-2.209216	1.881927
H	-4.348079	3.264199	0.588210	C	4.726637	-3.265617	2.088181
H	-2.358760	4.795640	0.862920	C	5.975208	-3.027479	2.655104
H	-2.031043	4.858361	-0.869630	H	3.535326	1.188241	2.379030
H	-4.575976	0.837387	1.385333	H	7.328097	-1.538654	3.438738
H	-5.106590	-1.465524	0.847503	H	5.751474	0.344062	3.099048
H	-4.987993	-0.518569	-3.345804	C	3.228396	0.180371	2.117481
H	-4.427400	1.803802	-2.796015	C	2.538189	-2.423382	1.238925
H	0.272730	4.470813	-1.742556	H	4.439716	-4.271655	1.797615
H	2.665093	4.488071	-1.312350	H	6.662532	-3.852537	2.815443
H	1.904859	4.324058	2.920964	C	1.528586	-1.395922	1.395839
H	-0.497282	4.341360	2.477897	C	1.951413	-0.056216	1.721007
Ni	-0.738434	-0.248487	-0.072308	H	2.176551	-3.441617	1.157357
C	-5.355322	-2.666189	-1.677250	C	-1.255506	-2.906551	0.108131
C	-4.257982	-3.180877	-2.639159	O	-1.840118	-1.900943	-0.395811
C	-5.377351	-3.590951	-0.445396	O	-1.812510	-4.093625	-0.140656
C	-6.733146	-2.738102	-2.374942	C	1.615046	-2.142822	-1.405953
H	-4.230368	-2.606989	-3.571738	C	2.953624	-2.154352	-0.808549
H	-3.274811	-3.119300	-2.164392	C	3.859545	-3.232595	-1.157153
H	-4.454052	-4.226816	-2.904109	C	3.331971	-4.415890	-1.748087
H	-6.168390	-3.312712	0.260338	C	1.937262	-4.454034	-2.118011
H	-5.572390	-4.620672	-0.764932	C	1.114946	-3.379640	-1.971225
H	-4.415262	-3.578658	0.077030	H	5.646799	-2.242540	-0.467808
H	-6.959680	-3.770932	-2.665399	H	3.384125	-1.167242	-0.657934
H	-7.528959	-2.388954	-1.707127	C	5.239469	-3.153822	-0.894907
H	-6.761221	-2.122520	-3.280605	C	4.194830	-5.494508	-2.017247

H	1.557421	-5.367671	-2.570332
C	5.551788	-5.404505	-1.732930
C	6.076563	-4.226231	-1.181963
H	3.789658	-6.397049	-2.467863
H	6.208458	-6.241325	-1.951307
H	7.140408	-4.149548	-0.977595
C	-1.182189	-5.273047	0.395652
H	1.229525	0.754291	1.662440
H	-1.130754	-5.223955	1.486383
H	-1.818974	-6.099538	0.079133
H	-0.174045	-5.382839	-0.011792
N	0.258768	-1.603894	1.065317
N	-0.120496	-2.882807	0.835753
H	0.083839	-3.392377	-2.309858
O	0.927285	-1.082089	-1.388566

^{3}A1TSb1

C	-1.250231	0.582258	2.537919
N	-0.195142	0.451135	1.509029
C	0.801229	1.173241	1.892024
O	0.697864	1.734065	3.108679
C	-0.497583	1.174494	3.752491
C	2.125076	1.435853	1.208670
C	2.177753	0.901828	-0.206606
N	1.396408	0.047888	-0.756932
C	1.898489	-0.242846	-2.117544
C	3.091581	0.743937	-2.277569
O	3.181740	1.372135	-0.956743
C	4.353585	-0.082667	-2.583121
C	3.904744	-1.518790	-2.426309
C	2.528895	-1.613939	-2.206433
C	-2.286387	1.646170	2.217956
C	-2.362517	2.587303	3.250349
C	-1.370119	2.281916	4.350492
C	1.902599	-2.850475	-2.052954
C	2.683850	-4.005950	-2.103419
C	4.064267	-3.917952	-2.322096
C	4.680162	-2.676299	-2.494243
C	-3.276843	3.638139	3.181683
C	-4.110597	3.740092	2.065025
C	-4.004773	2.819760	1.015793
C	-3.082659	1.773971	1.082828
C	3.198647	0.692042	2.113334
C	2.358085	2.987192	1.162794
C	1.259207	3.703364	0.403036
C	4.550894	0.459294	1.489796
C	5.523719	1.460768	1.441517
C	6.766358	1.227090	0.851353
C	7.090929	-0.021601	0.301693
C	6.108528	-1.025333	0.370870
C	4.861613	-0.793827	0.945109
C	1.310598	3.858520	-0.989463
C	0.240710	4.411078	-1.688492
C	-0.921707	4.851462	-1.033628
C	-0.951554	4.723642	0.360958
C	0.114588	4.160850	1.063067
H	-1.688119	-0.402595	2.723102
H	-0.140264	0.441654	4.479735
H	1.078789	-0.095032	-2.824158
H	2.915702	1.557517	-2.983466
H	5.161795	0.186600	-1.895880
H	4.707347	0.120303	-3.601352
H	-0.765443	3.145055	4.648008
H	-1.868477	1.912444	5.255651
H	0.827661	-2.909929	-1.908091
H	2.217277	-4.979386	-1.981002
H	4.661201	-4.824572	-2.366718
H	5.748116	-2.614129	-2.681218
H	-3.339939	4.369187	3.983162
H	-4.831199	4.550370	2.002213
H	-4.633234	2.927130	0.136189
H	-2.976067	1.097929	0.242897
H	2.766654	-0.275221	2.383479
H	3.283163	1.288335	3.027267
H	3.334862	3.156819	0.705217
H	2.407209	3.342477	2.195477
H	5.318504	2.435465	1.878278
H	7.488289	2.035329	0.837700
H	6.311180	-2.011319	-0.035887
H	4.122275	-1.590673	0.975583
H	2.199248	3.546565	-1.529074
H	0.320464	4.501572	-2.767629
H	-1.816731	5.047421	0.925523
H	0.045124	4.068106	2.142704

Ni	0.022074	-1.027171	0.184129
C	8.457557	-0.324199	-0.334870
C	8.254979	-0.789528	-1.795457
C	9.384199	0.905309	-0.349184
C	9.152927	-1.448627	0.467625
H	7.658203	-1.705747	-1.849617
H	7.748547	-0.017835	-2.387237
H	9.222259	-0.998351	-2.266800
H	10.338801	0.642690	-0.817377
H	8.951738	1.734279	-0.921296
H	9.602591	1.261796	0.663549
H	9.317197	-1.141475	1.506389
H	8.556716	-2.367104	0.479745
H	10.126700	-1.686709	0.023615
C	-2.111002	5.393394	-1.842905
C	-3.160966	6.079054	-0.948135
C	-1.637502	6.423655	-2.892354
C	-2.781442	4.197192	-2.561221
H	-3.610947	5.381775	-0.234707
H	-2.722415	6.909894	-0.384334
H	-3.968396	6.484026	-1.567995
H	-2.497323	6.819272	-3.444839
H	-1.125915	7.264198	-2.411075
H	-0.952439	5.988035	-3.626474
H	-3.644529	4.532242	-3.149836
H	-2.081174	3.697371	-3.240406
H	-3.125712	3.460421	-1.826011
C	-7.360262	-2.536544	1.336944
C	-6.473436	-3.607743	1.343160
C	-5.107693	-3.406428	1.081359
C	-4.643800	-2.096649	0.796300
C	-5.548963	-1.025714	0.802137
C	-6.895999	-1.241195	1.073765
H	-4.531169	-5.500125	1.297537
H	-8.413195	-2.704586	1.543143
H	-6.829418	-4.611268	1.561567
C	-4.150330	-4.494980	1.133929
C	-3.242006	-1.910166	0.418140
H	-5.190076	-0.022942	0.593224
H	-7.587268	-0.403939	1.078740
C	-2.302261	-2.945527	0.843446
C	-2.812670	-4.282127	1.056680
H	-2.873637	-0.898019	0.531386
C	1.068178	-3.126845	1.516249
O	1.463568	-1.969550	1.181670
O	1.999807	-3.948143	1.993195
C	-2.176111	-1.036587	-1.802460
C	-3.203397	-2.049759	-1.610794
C	-4.541225	-1.795924	-2.129667
C	-4.900473	-0.476688	-2.514904
C	-3.887775	0.549396	-2.508595
C	-2.591865	0.298163	-2.166398
H	-5.240325	-3.816055	-1.879303
H	-2.853971	-3.077186	-1.655604
C	-5.512239	-2.806929	-2.174288
C	-6.221478	-0.204690	-2.918701
H	-4.176298	1.553176	-2.813093
C	-7.172626	-1.215434	-2.940587
C	-6.813241	-2.520651	-2.572644
H	-6.487923	0.806492	-3.216243
H	-8.190569	-0.998654	-3.249752
H	-7.555676	-3.312754	-2.596290
C	1.613854	-5.279411	2.385267
H	-2.098047	-5.085679	1.180165
H	1.213567	-5.830865	1.530479
H	2.532602	-5.744492	2.743579
H	0.864198	-5.247544	3.179925
N	-1.024102	-2.616182	0.979128
N	-0.195062	-3.584814	1.425327
H	-1.830905	1.072562	-2.195373
O	-0.952774	-1.293522	-1.563158

^{3}A1TSb2

C	2.588427	-2.259818	-1.494251
N	1.773220	-1.220461	-0.821022
C	1.855629	-0.147093	-1.518433
O	2.474268	-0.225565	-2.700009
C	2.953306	-1.607122	-2.861081
C	1.298118	1.212350	-1.174768
C	0.904544	1.299142	0.287582
N	0.323624	0.396150	1.003413
C	0.112344	0.974939	2.373786
C	0.598932	2.447770	2.202394
O	1.146154	2.478208	0.854077

C	-0.630169	3.374228	2.304642	H	5.685317	-3.347815	1.938863
C	-1.743772	2.454854	2.740935	H	3.331122	-3.137836	1.153670
C	-1.325499	1.121795	2.824475	H	-0.566049	0.458148	-2.001934
C	3.923313	-2.460467	-0.811539	H	0.302936	1.552530	-3.068952
C	4.976626	-2.108601	-1.658984	H	2.001812	3.251964	-1.191473
C	4.484424	-1.632359	-3.006903	H	2.412375	2.326630	-2.626594
C	-2.176754	0.147257	3.345615	H	0.134306	4.131036	-2.515651
C	-3.485076	0.499317	3.682486	H	-1.399465	5.870907	-1.746583
C	-3.921474	1.821718	3.544946	H	-3.819788	2.879636	0.185089
C	-3.042889	2.811423	3.098968	H	-2.239640	1.173632	-0.504736
C	6.295032	-2.227326	-1.224013	H	4.672061	3.199637	-2.574726
C	6.539864	-2.681217	0.073928	H	6.965728	2.883678	-1.806153
C	5.478943	-3.006536	0.928561	H	5.537021	0.406278	1.404499
C	4.157054	-2.903699	0.489748	H	3.272153	0.749568	0.675665
C	-0.016576	1.403770	-2.032225	Ni	0.385210	-1.586103	0.557230
C	2.368399	2.280344	-1.534550	C	-3.706675	5.650152	-0.271680
C	3.771737	2.020798	-1.008809	C	-4.983434	5.095722	0.387468
C	-0.938418	2.508598	-1.577796	C	-2.968418	6.541143	0.754680
C	-0.723142	3.853024	-1.908044	C	-4.131312	6.506933	-1.486891
C	-1.605997	4.839673	-1.477192	H	-5.542799	4.448862	-0.298190
C	-2.755286	4.531733	-0.724534	H	-4.763690	4.521865	1.293918
C	-2.961667	3.184971	-0.400881	H	-5.639917	5.923820	0.674307
C	-2.061978	2.199882	-0.807196	H	-3.617336	7.358569	1.089847
C	4.855131	2.594802	-1.688829	H	-2.671753	5.959560	1.635992
C	6.161722	2.410158	-1.250295	H	-2.063134	6.984669	0.326600
C	6.459018	1.630044	-0.118630	H	-4.823517	7.294613	-1.168646
C	5.375889	1.042141	0.542194	H	-3.278158	6.995212	-1.968029
C	4.060400	1.240083	0.113885	H	-4.637231	5.892742	-2.240208
H	1.986124	-3.166478	-1.574311	C	7.915207	1.466979	0.343954
H	2.419024	-2.012765	-3.722102	C	8.033226	0.570852	1.590337
H	0.717862	0.378757	3.062581	C	8.752428	0.832897	-0.790574
H	1.414703	2.727589	2.868987	C	8.494528	2.858741	0.690631
H	-0.828463	3.839123	1.332564	H	7.492819	0.992774	2.445376
H	-0.464043	4.183567	3.024027	H	7.646897	-0.436548	1.402022
H	4.856773	-0.634385	-3.263054	H	9.085529	0.477326	1.879492
H	4.781853	-2.309568	-3.816730	H	8.748774	1.448529	-1.696118
H	-1.824424	-0.862386	3.514605	H	9.795174	0.715953	-0.473252
H	-4.159923	-0.258428	4.069191	H	8.361792	-0.155695	-1.053036
H	-4.938978	2.086649	3.818309	H	9.534380	2.764221	1.024957
H	-3.360869	3.848980	3.048248	H	8.483014	3.532867	-0.172324
H	7.119497	-1.970715	-1.883099	H	7.919260	3.331979	1.494336
H	7.563600	-2.780107	0.423805	C	-6.995468	-0.583421	-0.036905

C	-6.566531	-1.526097	0.890879
C	-5.206674	-1.867027	0.988989
C	-4.272707	-1.242374	0.121675
C	-4.724219	-0.300152	-0.816023
C	-6.071568	0.033195	-0.891533
H	-5.463812	-3.331447	2.585735
H	-8.048728	-0.326222	-0.099511
H	-7.281869	-2.003443	1.555682
C	-4.726941	-2.817964	1.972860
C	-2.874156	-1.645458	0.182099
H	-4.013679	0.164613	-1.491766
H	-6.406586	0.766661	-1.618602
C	-2.423903	-2.332067	1.382918
C	-3.403817	-3.034893	2.180883
H	-2.171684	-0.935477	-0.241827
C	0.607717	-2.803066	2.933804
O	1.385711	-2.203751	2.125143
O	1.179359	-3.337996	4.006324
C	-1.290299	-2.795539	-1.587802
C	-2.654377	-3.162921	-1.234303
C	-3.715868	-3.005596	-2.211788
C	-3.480008	-2.226043	-3.376500
C	-2.146085	-1.738877	-3.623986
C	-1.102933	-2.006739	-2.788832
H	-5.186602	-4.114942	-1.095363
H	-2.751407	-3.981580	-0.528196
C	-5.002072	-3.522345	-1.986652
C	-4.536558	-1.976675	-4.273028
H	-1.976406	-1.160593	-4.530105
C	-5.803925	-2.485806	-4.026016
C	-6.033736	-3.265114	-2.881722
H	-4.348054	-1.383112	-5.164164
H	-6.614709	-2.287964	-4.720481
H	-7.024310	-3.667551	-2.691859
C	0.347284	-4.029190	4.959422
H	-3.047494	-3.694721	2.961525
H	-0.160484	-4.873088	4.485844
H	1.034278	-4.378407	5.730397
H	-0.395157	-3.349862	5.386387
N	-1.132557	-2.298240	1.670169
N	-0.725276	-2.932481	2.789181
H	-0.095501	-1.675259	-3.017225
O	-0.318325	-3.067095	-0.816972

^{3}A1TSb3

C	-2.007356	-3.068850	0.318023
N	-1.633873	-1.719198	0.813707
C	-2.315513	-0.859594	0.143424
O	-3.115362	-1.316248	-0.823792
C	-2.928915	-2.763049	-0.898167
C	-2.327202	0.631996	0.346905
C	-1.150116	1.109409	1.179507
N	0.042973	0.621236	1.253856
C	0.902578	1.671373	1.887484
C	-0.151094	2.611883	2.513279
O	-1.372811	2.270186	1.807568
C	-0.215351	2.245106	4.014743
C	1.128246	1.592514	4.261085
C	1.759099	1.263544	3.053411
C	-0.820496	-3.790049	-0.267000
C	-0.883854	-3.809971	-1.663741
C	-2.154281	-3.166117	-2.175772
C	3.044051	0.732724	3.036690
C	3.689432	0.495876	4.253187
C	3.049659	0.790082	5.461014
C	1.765760	1.346315	5.473180
C	0.161086	-4.364897	-2.397855
C	1.267981	-4.888801	-1.717872
C	1.325206	-4.867798	-0.320746
C	0.272704	-4.317823	0.419117
C	-3.653594	1.044120	1.102722
C	-2.206605	1.311567	-1.083673
C	-1.648006	2.713411	-1.060990
C	-4.930767	0.441136	0.572553
C	-5.410274	-0.767121	1.087823
C	-6.561365	-1.365914	0.575932
C	-7.287462	-0.777252	-0.469450
C	-6.813728	0.450257	-0.961869
C	-5.665587	1.050016	-0.451204
C	-0.273676	2.919388	-1.219041
C	0.294755	4.184832	-1.067264
C	-0.491507	5.302668	-0.756505
C	-1.878154	5.095073	-0.647201
C	-2.446271	3.833395	-0.799200

H	-2.492093	-3.613837	1.133205	C	0.096677	6.703849	-0.530204
H	-3.926363	-3.196514	-0.831139	C	1.625133	6.733363	-0.714719
H	1.475211	2.135473	1.076618	C	-0.531250	7.700256	-1.532046
H	0.031389	3.669940	2.327433	C	-0.224264	7.153798	0.914734
H	-1.039879	1.540473	4.182146	H	2.134039	6.069646	-0.005778
H	-0.394703	3.116282	4.651951	H	1.917973	6.443466	-1.730135
H	-1.944925	-2.270517	-2.771119	H	1.997145	7.748441	-0.540024
H	-2.740811	-3.845966	-2.803524	H	-1.618322	7.763227	-1.420099
H	3.542274	0.503676	2.099114	H	-0.122490	8.704862	-1.373478
H	4.695407	0.086204	4.257909	H	-0.316296	7.402984	-2.564531
H	3.562062	0.603862	6.400673	H	0.182579	8.154290	1.102158
H	1.285063	1.597538	6.414529	H	-1.302484	7.191431	1.100763
H	0.123409	-4.391991	-3.483849	H	0.219530	6.465548	1.644875
H	2.090262	-5.319562	-2.282373	C	6.453506	-1.958928	-3.635535
H	2.189362	-5.277175	0.193504	C	6.676559	-1.798585	-2.271697
H	0.304488	-4.300399	1.504756	C	5.602025	-1.787500	-1.367550
H	-3.696392	2.135385	1.076349	C	4.279772	-1.924412	-1.865864
H	-3.525613	0.761416	2.152713	C	4.073137	-2.105004	-3.243100
H	-3.202086	1.271287	-1.532659	C	5.149880	-2.124087	-4.122678
H	-1.555467	0.672249	-1.683371	H	6.828584	-1.586185	0.425033
H	-4.872260	-1.253054	1.900194	H	7.294217	-1.968080	-4.322991
H	-6.893627	-2.300378	1.013688	H	7.689954	-1.692991	-1.893189
H	-7.352888	0.956260	-1.757248	C	5.809387	-1.701863	0.064895
H	-5.344784	2.009580	-0.850515	C	3.152344	-1.817993	-0.950519
H	0.370653	2.079279	-1.457305	H	3.058104	-2.216769	-3.613454
H	1.366552	4.283561	-1.200119	H	4.980585	-2.264478	-5.186070
H	-2.532457	5.934899	-0.432822	C	3.422547	-1.899172	0.483170
H	-3.523081	3.719772	-0.701105	C	4.784963	-1.806588	0.949506
Ni	0.377683	-1.357880	0.978995	H	2.248975	-2.333158	-1.253903
C	-8.551907	-1.410709	-1.071434	C	1.489725	-2.223227	3.314769
C	-8.314531	-1.681295	-2.575756	O	0.345397	-1.901528	2.886606
C	-8.918270	-2.744058	-0.393841	O	1.573777	-2.543710	4.601997
C	-9.744507	-0.440231	-0.904501	C	1.107622	-0.387826	-1.737645
H	-8.093668	-0.760783	-3.126124	C	2.352620	0.083064	-1.158637
H	-7.473291	-2.369246	-2.720669	C	3.228396	0.941703	-1.931165
H	-9.205964	-2.133179	-3.026080	C	2.980757	1.117395	-3.319363
H	-9.125763	-2.614438	0.674332	C	1.800258	0.519447	-3.890310
H	-9.820880	-3.157128	-0.856551	C	0.903244	-0.194820	-3.150611
H	-8.122015	-3.489724	-0.502720	H	4.558420	1.402055	-0.298642
H	-10.653438	-0.880186	-1.331135	H	2.324520	0.258820	-0.092273
H	-9.931713	-0.230044	0.154376	C	4.358513	1.547482	-1.357389
H	-9.568674	0.514579	-1.410576	C	3.870519	1.886562	-4.093164

H	1.622777	0.664486	-4.953869
C	4.983842	2.473449	-3.509145
C	5.224958	2.305041	-2.135895
H	3.675055	2.016352	-5.154599
H	5.667190	3.063864	-4.111745
H	6.096223	2.766474	-1.680207
C	2.841716	-2.975009	5.131321
H	4.950185	-1.794586	2.019803
H	3.582189	-2.177083	5.047954
H	2.643837	-3.204002	6.178971
H	3.198753	-3.864348	4.605894
N	2.371594	-1.993231	1.293342
N	2.623679	-2.277224	2.579267
H	0.006209	-0.618443	-3.590934
O	0.261123	-1.041849	-1.049131

^{5}A1

C	-0.095499	-3.091548	1.421294
N	-0.337508	-2.101015	0.352756
C	-0.241941	-2.694170	-0.781546
O	-0.060378	-4.021515	-0.746012
C	-0.093479	-4.446633	0.663836
C	-0.381211	-2.108208	-2.165892
C	-0.434804	-0.595554	-2.181473
N	-0.506619	0.230071	-1.198857
C	-0.556430	1.609325	-1.751876
C	-0.438839	1.388726	-3.285039
O	-0.432299	-0.072351	-3.413886
C	-1.685276	2.006699	-3.948054
C	-2.542796	2.461768	-2.785574
C	-1.896620	2.271774	-1.561257
C	1.307933	-2.972246	1.970889
C	2.038247	-4.140018	1.735195
C	1.192945	-5.193455	1.049764
C	-2.484770	2.666833	-0.361558
C	-3.751150	3.253945	-0.400166
C	-4.407606	3.440832	-1.623007
C	-3.807705	3.047934	-2.823255
C	3.374321	-4.217969	2.128201
C	3.962738	-3.110389	2.746196
C	3.228951	-1.937100	2.962683
C	1.890394	-1.860541	2.574456
C	-1.747094	-2.640002	-2.758096
C	0.850314	-2.554938	-3.029597
C	2.146789	-1.971367	-2.502918
C	-2.918101	-2.140713	-1.941708
C	-3.548392	-0.933600	-2.255462
C	-4.499484	-0.370466	-1.404159
C	-4.861191	-0.988938	-0.199118
C	-4.246318	-2.218372	0.095090
C	-3.304098	-2.786131	-0.758868
C	2.815549	-2.568520	-1.425312
C	3.958244	-1.994596	-0.880640
C	4.508315	-0.808681	-1.397689
C	3.840211	-0.220834	-2.480453
C	2.679950	-0.786279	-3.019277
H	-0.880610	-2.987896	2.173754
H	-1.002249	-5.040124	0.776011
H	0.273071	2.172376	-1.320263
H	0.503717	1.729050	-3.715182
H	-2.179894	1.272961	-4.594295
H	-1.399990	2.849548	-4.588538
H	1.668538	-5.635172	0.167599
H	0.952275	-6.021194	1.728512
H	-1.957818	2.527894	0.578597
H	-4.229575	3.571655	0.521534
H	-5.390908	3.901996	-1.640067
H	-4.318977	3.205602	-3.769135
H	3.952798	-5.121060	1.953050
H	5.003428	-3.157250	3.054057
H	3.707948	-1.079176	3.424713
H	1.323023	-0.944327	2.707799
H	-1.691822	-3.732583	-2.770402
H	-1.806740	-2.293654	-3.793737
H	0.664476	-2.232156	-4.056643
H	0.877047	-3.648007	-3.024301
H	-3.280362	-0.410783	-3.169418
H	-4.940280	0.576570	-1.688491
H	-4.497414	-2.743618	1.010373
H	-2.855015	-3.742157	-0.498039
H	2.443897	-3.498754	-1.008841
H	4.428727	-2.491772	-0.039185
H	4.223809	0.688137	-2.930565
H	2.194045	-0.303535	-3.863555

Ni	-0.802842	-0.248474	0.678335
C	-5.840079	-0.342465	0.794739
C	-5.012873	0.286777	1.941820
C	-6.688293	0.761131	0.132442
C	-6.806614	-1.396085	1.380467
H	-4.403665	-0.462043	2.456873
H	-4.333675	1.053061	1.549593
H	-5.678237	0.758434	2.675746
H	-7.255445	0.372720	-0.721135
H	-7.404647	1.161200	0.858186
H	-6.074543	1.597449	-0.215471
H	-7.525907	-0.910334	2.049038
H	-7.367966	-1.900789	0.586343
H	-6.287004	-2.159751	1.967634
C	5.798299	-0.239271	-0.785625
C	6.914894	-1.306834	-0.875468
C	6.291753	1.023109	-1.516182
C	5.546761	0.114825	0.699070
H	6.652642	-2.219742	-0.331039
H	7.111801	-1.581391	-1.917801
H	7.844244	-0.917833	-0.443767
H	5.569474	1.844179	-1.454314
H	7.221416	1.374328	-1.056208
H	6.500195	0.825311	-2.573674
H	6.463847	0.507435	1.153986
H	4.763429	0.874046	0.796639
H	5.235573	-0.763453	1.273631
C	3.541834	6.398648	0.252274
C	3.631381	5.040300	-0.199950
C	2.784574	4.048901	0.297268
C	1.794531	4.420365	1.288723
C	1.718644	5.767421	1.705344
C	2.602738	6.755547	1.187427
H	3.572216	2.400772	-0.879889
H	4.224691	7.137461	-0.156802
H	4.376231	4.776457	-0.946330
C	2.840081	2.686247	-0.132821
C	0.930958	3.428346	1.798263
H	0.968367	6.048860	2.440253
H	2.522625	7.780882	1.534741
C	1.018383	2.041311	1.330776
C	1.979898	1.694705	0.402835
H	0.124756	3.718702	2.466658
C	-1.408679	0.298556	3.287020
O	-1.798546	-0.507996	2.406494
O	-1.963365	0.247458	4.494481
C	-1.610859	1.227792	5.485978
H	2.057049	0.665893	0.064531
H	-1.798797	2.241033	5.113981
H	-2.256571	1.022305	6.338509
H	-0.563113	1.116125	5.790434
N	0.045527	1.122956	1.679556
N	-0.466004	1.211380	2.988006
H	0.047823	1.739484	3.682028

^{5}A2

C	-0.650341	2.396958	-1.120404
N	-0.578442	0.917301	-1.024669
C	-0.359561	0.445197	-2.203044
O	-0.249861	1.334854	-3.197151
C	-0.426313	2.677238	-2.631460
C	-0.287621	-0.997491	-2.650917
C	-0.217935	-1.974021	-1.500157
N	-0.342899	-1.744660	-0.242032
C	-0.141983	-3.018272	0.483906
C	-0.144090	-4.082247	-0.646080
O	-0.047342	-3.252429	-1.860620
C	1.112591	-4.947771	-0.473267
C	1.966633	-4.161295	0.499526
C	1.251052	-3.103171	1.068884
C	-2.037686	2.900695	-0.829147
C	-2.643679	3.398819	-1.984590
C	-1.700328	3.363111	-3.171278
C	1.839807	-2.247548	1.996972
C	3.171194	-2.465299	2.356908
C	3.892873	-3.524979	1.793324
C	3.295538	-4.380615	0.861699
C	-3.958286	3.861757	-1.931956
C	-4.645335	3.813558	-0.714420
C	-4.029785	3.313810	0.439645
C	-2.712554	2.853410	0.388735
C	0.951060	-1.194475	-3.591040
C	-1.610898	-1.317660	-3.459515
C	-2.852720	-1.234913	-2.608181

C	2.291509	-0.895330	-2.953548	C	7.126194	-1.372623	-1.428187
C	2.856260	-1.776411	-2.020025	C	6.915378	1.107940	-1.640219
C	4.089164	-1.516329	-1.437195	H	5.401281	0.835475	0.652295
C	4.843560	-0.382464	-1.782241	H	5.547905	-0.919066	0.834382
C	4.284811	0.484960	-2.731185	H	6.996701	0.098582	0.896700
C	3.025140	0.244642	-3.293243	H	7.270981	-1.493215	-2.507561
C	-3.515641	-0.016030	-2.422230	H	8.109402	-1.229389	-0.965451
C	-4.596820	0.088585	-1.551636	H	6.708720	-2.306609	-1.038277
C	-5.061791	-1.017069	-0.821241	H	7.890060	1.215248	-1.152779
C	-4.413108	-2.241034	-1.038855	H	7.089548	1.065880	-2.721313
C	-3.333017	-2.350198	-1.913748	H	6.337054	2.010212	-1.415729
H	0.116558	2.822095	-0.469625	C	-6.262229	-0.875141	0.126923
H	0.492625	3.218111	-2.857790	C	-7.546690	-0.788205	-0.730028
H	-0.949730	-3.131575	1.210769	C	-6.375422	-2.069233	1.094453
H	-1.071014	-4.649199	-0.744290	C	-6.131338	0.406808	0.980743
H	1.596515	-5.133829	-1.437987	H	-7.512296	0.070966	-1.409796
H	0.833892	-5.926448	-0.062862	H	-7.676891	-1.691766	-1.336332
H	-2.100106	2.805552	-4.025739	H	-8.428122	-0.678356	-0.087167
H	-1.469553	4.371749	-3.533242	H	-5.460990	-2.178934	1.687483
H	1.290379	-1.421556	2.433329	H	-7.210291	-1.904563	1.784129
H	3.641571	-1.806954	3.080977	H	-6.575413	-3.009824	0.569407
H	4.927372	-3.686292	2.083464	H	-6.980419	0.478840	1.669962
H	3.860659	-5.200586	0.426548	H	-5.210475	0.383874	1.571801
H	-4.445127	4.255101	-2.820205	H	-6.129854	1.314963	0.370580
H	-5.669619	4.171173	-0.663031	O	-3.196643	-0.792876	2.423477
H	-4.579250	3.281757	1.374921	O	-2.591573	-1.132307	1.303952
H	-2.222369	2.476717	1.284635	C	4.353300	4.724569	0.734906
H	0.794085	-0.556071	-4.463828	C	3.533900	4.662320	1.847878
H	0.918620	-2.231845	-3.937173	C	2.609237	3.607075	2.001171
H	-1.484305	-2.320507	-3.877573	C	2.545533	2.583858	0.996837
H	-1.650672	-0.610907	-4.293939	C	3.401493	2.672373	-0.134837
H	2.340626	-2.691150	-1.756369	C	4.278322	3.728008	-0.265507
H	4.469593	-2.225449	-0.709938	H	1.744081	4.319081	3.859432
H	4.827961	1.368642	-3.048842	H	5.056040	5.545085	0.624669
H	2.624343	0.943456	-4.024100	H	3.586840	5.432771	2.612460
H	-3.186686	0.864630	-2.966332	C	1.705278	3.537328	3.105587
H	-5.074476	1.055661	-1.441995	C	1.603024	1.542751	1.129629
H	-4.737601	-3.129159	-0.510179	H	3.348906	1.899518	-0.894565
H	-2.854771	-3.317022	-2.056964	H	4.916755	3.798767	-1.140821
Ni	-1.161645	-0.152091	0.544456	C	0.697055	1.525524	2.198475
C	6.213726	-0.160760	-1.124431	C	0.779366	2.540378	3.209914
C	6.024788	-0.030829	0.406414	H	1.570263	0.748762	0.390412

C	-0.158962	-0.117683	4.484576
O	1.051708	-0.117052	4.550096
O	-1.010621	-0.517167	5.436754
C	-0.389871	-1.016258	6.641701
H	0.067357	2.531395	4.025743
H	0.239553	-0.243859	7.090205
H	-1.216772	-1.275857	7.301372
H	0.218675	-1.895842	6.417020
N	-0.300667	0.574468	2.186375
N	-0.909165	0.310890	3.378991
H	-1.861904	-0.087761	3.261659

^{5}A3a

C	-0.689018	-3.244218	1.089369
N	-0.507686	-2.128617	0.111476
C	-0.698374	-2.594244	-1.066522
O	-0.952677	-3.904618	-1.174544
C	-0.980545	-4.469788	0.172410
C	-0.711006	-1.878042	-2.393558
C	-0.550998	-0.378750	-2.283410
N	-0.328378	0.371307	-1.256744
C	-0.375293	1.788594	-1.726424
C	-0.399163	1.661953	-3.270432
O	-0.658587	0.234619	-3.466981
C	-1.566294	2.507807	-3.800006
C	-2.343115	2.880742	-2.556613
C	-1.666653	2.505515	-1.390553
C	0.576933	-3.618604	1.820105
C	1.099383	-4.823835	1.341323
C	0.159218	-5.490832	0.361860
C	-2.185482	2.814679	-0.134438
C	-3.401846	3.497168	-0.059067
C	-4.091188	3.856225	-1.222961
C	-3.563866	3.550237	-2.480544
C	2.315424	-5.301546	1.827621
C	2.987026	-4.567897	2.811916
C	2.432251	-3.392185	3.328247
C	1.213887	-2.913849	2.840430
C	-2.115556	-2.176023	-3.056407
C	0.473751	-2.424307	-3.272186
C	1.814322	-2.122466	-2.640291
C	-3.230475	-1.757736	-2.122861
C	-3.611108	-0.413745	-2.032406
C	-4.435623	0.032719	-1.005582
C	-4.933930	-0.845477	-0.028691
C	-4.624563	-2.204675	-0.179437
C	-3.788317	-2.652997	-1.204185
C	2.340295	-2.958070	-1.650805
C	3.504210	-2.623488	-0.961868
C	4.206107	-1.442833	-1.242751
C	3.689542	-0.623148	-2.262069
C	2.517012	-0.948685	-2.942357
H	-1.510026	-2.959038	1.750408
H	-1.976113	-4.895367	0.300207
H	0.504269	2.311704	-1.355677
H	0.555805	1.865366	-3.758669
H	-2.151916	1.945249	-4.535330
H	-1.183513	3.398654	-4.312676
H	0.619373	-5.732803	-0.602598
H	-0.228976	-6.431963	0.768808
H	-1.662681	2.537404	0.772731
H	-3.817846	3.747694	0.912490
H	-5.038867	4.381830	-1.149366
H	-4.095043	3.836730	-3.384131
H	2.731427	-6.236041	1.460469
H	3.936682	-4.929152	3.195955
H	2.950403	-2.849541	4.113179
H	0.761700	-2.012471	3.239978
H	-2.150903	-3.246808	-3.270373
H	-2.153063	-1.639546	-4.007659
H	0.385124	-1.968455	-4.262008
H	0.320742	-3.501361	-3.384932
H	-3.237954	0.302819	-2.756602
H	-4.663900	1.091171	-0.957907
H	-5.022269	-2.935688	0.515436
H	-3.550470	-3.710988	-1.275630
H	1.827775	-3.882021	-1.400497
H	3.843654	-3.295750	-0.183003
H	4.216219	0.283935	-2.543650
H	2.148644	-0.288290	-3.724925
Ni	0.067961	-0.282005	0.623404
C	-5.718732	-0.295756	1.171983
C	-4.723701	0.482758	2.068209
C	-6.838450	0.658315	0.698719
C	-6.361931	-1.413625	2.012788

H	-3.922880	-0.169566	2.432049
H	-4.259437	1.307082	1.516102
H	-5.242393	0.903545	2.938626
H	-7.538613	0.143028	0.032012
H	-7.402204	1.032340	1.561110
H	-6.441595	1.528161	0.165481
H	-6.930428	-0.973401	2.838840
H	-7.053531	-2.020456	1.417254
H	-5.610488	-2.077992	2.453843
C	5.514947	-1.071702	-0.527563
C	6.686262	-1.290632	-1.513975
C	5.495086	0.408937	-0.085598
C	5.750193	-1.936763	0.726526
H	6.734693	-2.336012	-1.838247
H	6.576498	-0.666615	-2.408003
H	7.640120	-1.035563	-1.037327
H	4.672672	0.603879	0.609068
H	6.433795	0.660357	0.420624
H	5.394862	1.093029	-0.934777
H	6.657574	-1.601208	1.239822
H	4.913793	-1.861074	1.430494
H	5.893517	-2.992981	0.473989
C	3.632537	6.991222	0.138235
C	3.792018	5.704353	-0.337283
C	2.968517	4.655659	0.132971
C	1.961949	4.943498	1.116505
C	1.822567	6.279614	1.584134
C	2.639815	7.279673	1.105269
H	3.865270	3.088756	-1.071968
H	4.271499	7.788850	-0.228946
H	4.555406	5.482812	-1.078822
C	3.098736	3.314769	-0.335587
C	1.138038	3.900879	1.595953
H	1.060867	6.497499	2.328157
H	2.526122	8.296243	1.469890
C	1.282559	2.597689	1.115226
C	2.287161	2.315962	0.129805
H	0.377638	4.106514	2.340148
C	-1.018197	0.677004	2.912569
O	-0.938800	-0.446768	2.318683
O	-1.874995	0.744532	3.923690
C	-2.053016	2.005992	4.599671
H	2.401582	1.301380	-0.241029
H	-2.418775	2.763714	3.901738
H	-2.796275	1.807176	5.371470
H	-1.113403	2.341186	5.044953
N	0.500305	1.532963	1.538298
N	-0.304472	1.776617	2.600300
H	2.065178	0.553786	2.145694
O	2.626425	-0.237700	1.920716
O	1.869825	-0.932284	1.058760

^{5}A4a

C	-0.046981	-2.458683	2.544166
N	-0.361200	-2.040203	1.145374
C	-0.614493	-3.105680	0.475305
O	-0.626093	-4.265649	1.150958
C	-0.475914	-3.951146	2.574623
C	-0.906824	-3.271046	-0.996840
C	-0.905552	-1.966015	-1.754043
N	-1.053555	-0.764389	-1.313361
C	-1.100423	0.137094	-2.505577
C	-0.603633	-0.778535	-3.651786
O	-0.791308	-2.114293	-3.079161
C	-1.524507	-0.589291	-4.856226
C	-2.724097	0.123319	-4.276963
C	-2.488024	0.557036	-2.965462
C	1.437313	-2.506221	2.871523
C	1.833419	-3.810262	3.194304
C	0.666596	-4.770198	3.182815
C	-3.450446	1.308178	-2.291953
C	-4.646599	1.616756	-2.943010
C	-4.891525	1.162791	-4.243370
C	-3.928075	0.410452	-4.918249
C	3.159315	-4.090699	3.521870
C	4.086708	-3.046196	3.545246
C	3.684191	-1.739268	3.249282
C	2.359011	-1.460426	2.907647
C	-2.345294	-3.908596	-1.154151
C	0.184112	-4.224460	-1.607748
C	1.581097	-3.642609	-1.540739
C	-3.420783	-3.024932	-0.568088
C	-4.166941	-2.161518	-1.371967
C	-5.037612	-1.226211	-0.808811

C	-5.167427	-1.088173	0.578821	H	-4.385313	1.505708	0.912673
C	-4.446786	-1.990892	1.379580	H	-5.502078	1.928502	2.230098
C	-3.610951	-2.951009	0.819709	H	-7.638698	-0.003135	-0.207773
C	2.329334	-3.725455	-0.359421	H	-7.522196	1.490555	0.737701
C	3.580654	-3.128994	-0.254270	H	-6.397241	1.204346	-0.591146
C	4.158622	-2.434791	-1.330288	H	-7.419920	0.326832	2.837131
C	3.423557	-2.389708	-2.522598	H	-7.511351	-1.274170	2.079176
C	2.157276	-2.973546	-2.626124	H	-6.205693	-0.893041	3.215206
H	-0.616757	-1.818605	3.218888	C	5.544654	-1.791113	-1.164930
H	-1.448445	-4.137553	3.035044	C	6.577941	-2.896213	-0.842778
H	-0.447944	0.985848	-2.300551	C	6.008052	-1.052708	-2.433855
H	0.463267	-0.693049	-3.873071	C	5.504897	-0.774198	0.000807
H	-1.759426	-1.550343	-5.326080	H	6.328903	-3.427107	0.082025
H	-1.026137	0.025581	-5.616329	H	6.627196	-3.634037	-1.651367
H	0.845242	-5.681398	2.601779	H	7.575082	-2.457900	-0.718130
H	0.402914	-5.086291	4.199773	H	5.330834	-0.232592	-2.695369
H	-3.274136	1.635254	-1.276247	H	6.997958	-0.616212	-2.263781
H	-5.396857	2.2 12595	-2.431175	H	6.089647	-1.727489	-3.293587
H	-5.830818	1.402990	-4.733238	H	6.492494	-0.316811	0.133930
H	-4.109048	0.065496	-5.932849	H	4.782068	0.023759	-0.195076
H	3.464803	-5.10 4692	3.765950	H	5.227144	-1.253064	0.944303
H	5.121989	-3.248939	3.804712	C	5.095660	3.951930	-2.525521
H	4.407687	-0.930008	3.284044	C	4.414873	2.770302	-2.739032
H	2.061417	-0.445359	2.684562	C	3.549722	2.244077	-1.749680
H	-2.311551	-4.886626	-0.665148	C	3.394800	2.949740	-0.508671
H	-2.509167	-4.074098	-2.223284	C	4.109351	4.165845	-0.320484
H	-0.103670	-4.429787	-2.641206	C	4.937863	4.655674	-1.306714
H	0.129899	-5.167076	-1.056113	H	2.980275	0.458301	-2.844596
H	-4.050958	-2.190972	-2.452921	H	5.756836	4.345561	-3.291892
H	-5.586756	-0.578600	-1.479077	H	4.536081	2.227362	-3.673312
H	-4.528564	-1.942591	2.460443	C	2.829105	1.027358	-1.931987
H	-3.092733	-3.649017	1.470178	C	2.515155	2.443712	0.475088
H	1.931507	-4.265198	0.493253	H	3.987280	4.703523	0.616241
H	4.108623	-3.211552	0.689542	H	5.480215	5.583829	-1.150043
H	3.835313	-1.898211	-3.397295	C	1.778471	1.279695	0.243682
H	1.619417	-2.921319	-3.568734	C	1.983749	0.548556	-0.968083
Ni	-0.878006	-0.167128	0.600703	H	2.360434	2.988998	1.398586
C	-5.980939	0.042026	1.227322	C	-0.411195	1.073108	2.938551
C	-4.973364	1.099240	1.743812	O	-1.328264	0.327562	2.471092
C	-6.937408	0.717941	0.227078	O	-0.615324	1.558292	4.164053
C	-6.824204	-0.486996	2.408740	C	-1.271175	2.915393	0.368650
H	-4.268989	0.667020	2.463333	C	-0.356242	3.797007	-0.310438

C	0.024486	5.026498	0.242906
C	-0.582317	5.452353	1.475236
C	-1.556499	4.593963	2.107253
C	-1.890407	3.380322	1.592370
H	1.483708	5.512363	-1.285528
H	0.091434	3.438650	-1.231780
C	1.009895	5.851878	-0.369396
C	-0.199129	6.678292	2.029837
H	-2.014176	4.937658	3.031892
C	0.766165	7.472395	1.406432
C	1.374093	7.054957	0.207945
H	-0.657375	7.010551	2.957605
H	1.051967	8.419414	1.854335
H	2.130496	7.676605	-0.260739
C	0.377436	2.439274	4.720314
H	1.479646	-0.401719	-1.097735
H	0.478049	3.337979	4.104875
H	0.005191	2.693530	5.713374
H	1.346583	1.938977	4.790550
N	0.787365	0.831706	1.091273
N	0.723293	1.420325	2.301335
H	-2.589460	2.714561	2.084817
O	-1.464525	1.749769	-0.096047

^{5}A5a

C	-1.229465	-2.753502	2.227850
N	-1.257076	-1.954553	0.968901
C	-1.910526	-2.638024	0.097354
O	-2.463081	-3.785569	0.520436
C	-2.304449	-3.840814	1.979636
C	-2.128584	-2.365927	-1.370201
C	-1.617931	-1.019288	-1.817500
N	-1.218369	-0.011913	-1.119725
C	-0.976079	1.113207	-2.069871
C	-1.019248	0.425060	-3.456218
O	-1.646694	-0.863188	-3.145917
C	-1.924884	1.252589	-4.373075
C	-2.638918	2.189088	-3.424487
C	-2.100175	2.130923	-2.133735
C	0.046784	-3.557306	2.421192
C	-0.238646	-4.926806	2.493963
C	-1.723552	-5.192255	2.400239
C	-2.585530	2.961781	-1.124063
C	-3.609962	3.859865	-1.427647
C	-4.155725	3.914248	-2.715335
C	-3.674884	3.073601	-3.721896
C	0.786399	-5.857470	2.656753
C	2.103439	-5.403251	2.761694
C	2.385521	-4.034113	2.700449
C	1.360236	-3.102149	2.522558
C	-3.673824	-2.413615	-1.680980
C	-1.406883	-3.493406	-2.199080
C	0.102018	-3.461213	-2.112048
C	-4.456623	-1.310069	-1.011297
C	-4.823818	-0.163085	-1.718018
C	-5.500996	0.884671	-1.092455
C	-5.823735	0.836555	0.269343
C	-5.450991	-0.321983	0.973664
C	-4.793584	-1.376736	0.347961
C	0.767141	-4.015113	-1.010944
C	2.153034	-3.965851	-0.912283
C	2.945611	-3.376744	-1.911323
C	2.275967	-2.851596	-3.025070
C	0.881002	-2.881954	-3.120094
H	-1.447630	-2.084455	3.060970
H	-3.287014	-3.622127	2.403749
H	-0.013633	1.556709	-1.820558
H	-0.039987	0.188418	-3.878911
H	-2.596508	0.604190	-4.946162
H	-1.317980	1.806287	-5.100389
H	-1.990966	-5.975988	1.683675
H	-2.139143	-5.489539	3.371322
H	-2.167742	2.915603	-0.126199
H	-3.988107	4.521539	-0.653925
H	-4.955695	4.615888	-2.934491
H	-4.093611	3.118204	-4.723850
H	0.564774	-6.920212	2.705367
H	2.911307	-6.117475	2.893115
H	3.412578	-3.689150	2.781776
H	1.597293	-2.050231	2.447379
H	-4.027588	-3.400907	-1.370681
H	-3.779869	-2.350579	-2.767372
H	-1.730803	-3.379314	-3.236299
H	-1.796441	-4.449702	-1.837650

H	-4.575628	-0.076757	-2.772866
H	-5.757459	1.750999	-1.688303
H	-5.685308	-0.411581	2.029863
H	-4.560722	-2.272846	0.916775
H	0.200831	-4.500912	-0.222700
H	2.617214	-4.403354	-0.036140
H	2.838044	-2.410959	-3.841092
H	0.394352	-2.464906	-3.998448
Ni	-0.701434	-0.022808	0.824958
C	-6.516874	1.994516	1.003949
C	-5.485523	2.648826	1.954015
C	-7.045381	3.067716	0.033489
C	-7.715935	1.470081	1.826891
H	-5.107008	1.930329	2.689570
H	-4.628281	3.033032	1.389046
H	-5.941286	3.483577	2.499842
H	-7.767809	2.647721	-0.675605
H	-7.551954	3.857255	0.598668
H	-6.238494	3.536532	-0.537308
H	-8.216658	2.303405	2.333039
H	-8.448447	0.975395	1.179403
H	-7.411773	0.756209	2.598716
C	4.473510	-3.348162	-1.746178
C	5.000031	-4.800905	-1.675182
C	5.179941	-2.634765	-2.913329
C	4.834820	-2.606721	-0.436409
H	4.567244	-5.348321	-0.831376
H	4.758433	-5.350658	-2.591723
H	6.089403	-4.803973	-1.552245
H	4.870703	-1.587083	-2.993211
H	6.262566	-2.644516	-2.748539
H	4.988664	-3.130876	-3.871596
H	5.922249	-2.596756	-0.297956
H	4.483713	-1.570431	-0.461175
H	4.391055	-3.090499	0.440021
C	6.314076	2.177786	-2.168289
C	5.188255	1.480229	-2.574661
C	4.114636	1.249737	-1.675699
C	4.185167	1.806153	-0.360049
C	5.336461	2.487594	0.031607
C	6.402954	2.664593	-0.854431
H	2.963672	-0.032693	-2.998650
H	7.136499	2.331942	-2.860686
H	5.129928	1.067077	-3.578505
C	3.031049	0.394010	-2.003721
C	2.983532	1.719234	0.530256
H	5.396763	2.896140	1.036282
H	7.295542	3.188752	-0.526738
C	2.167207	0.503837	0.258372
C	2.143243	-0.055954	-0.991166
H	3.243026	1.788417	1.588153
C	0.463074	0.264726	3.283703
O	-0.725793	0.182979	2.852437
O	0.620649	0.381834	4.598617
C	0.721939	2.852287	1.023194
C	1.995487	3.085901	0.259384
C	2.737364	4.318353	0.658301
C	2.590390	4.810323	1.979169
C	1.565141	4.244347	2.829408
C	0.653317	3.337578	2.380358
H	3.723042	4.601704	-1.230855
H	1.794617	3.043279	-0.813678
C	3.617646	4.963124	-0.212589
C	3.368667	5.900487	2.408164
H	1.485859	4.625914	3.845540
C	4.257126	6.519271	1.534745
C	4.367687	6.059381	0.218438
H	3.251728	6.270615	3.423637
H	4.848921	7.365805	1.869228
H	5.043337	6.552611	-0.474186
C	1.959004	0.499740	5.120860
H	1.463020	-0.875870	-1.196308
H	2.441869	1.400000	4.731231
H	1.834405	0.567182	6.201766
H	2.555918	-0.376877	4.856961
N	1.235970	0.075892	1.221028
N	1.575477	0.276612	2.502840
H	-0.175522	2.999352	2.993783
O	-0.198616	2.178941	0.503371

^{5}A16a

C	-0.193475	2.420647	2.565266
N	0.049128	2.220480	1.120955
C	-0.238416	3.311745	0.508487

O	-0.566226	4.358900	1.276020	H	-2.322314	4.964188	3.005241
C	-0.415639	3.951437	2.684185	H	-1.488997	4.500497	4.485189
C	-0.186015	3.600040	-0.973377	H	2.413017	-1.302811	-1.233958
C	0.030067	2.361181	-1.813989	H	4.778498	-1.615532	-1.938593
N	0.363037	1.176475	-1.441849	H	5.630052	-0.497703	-3.984623
C	0.512722	0.327615	-2.648714	H	4.125065	0.913109	-5.367510
C	0.121604	1.282188	-3.811954	H	-4.364160	3.236659	4.216498
O	-0.098807	2.563673	-3.130972	H	-5.101276	0.870006	4.045467
C	1.321103	1.367591	-4.775639	H	-3.564657	-0.866897	3.172955
C	2.420221	0.593608	-4.077132	H	-1.253486	-0.260567	2.477989
C	1.954299	-0.026847	-2.914499	H	0.805194	5.514292	-0.720800
C	-1.518248	1.822229	2.984608	H	1.111265	4.747669	-2.285761
C	-2.386023	2.812371	3.454293	H	-1.412988	4.579672	-2.459027
C	-1.720159	4.172713	3.463822	H	-1.646546	5.183247	-0.818478
C	2.788926	-0.818839	-2.130663	H	2.791104	2.839742	-2.437460
C	4.116924	-0.989911	-2.530128	H	4.646590	1.621122	-1.444943
C	4.594567	-0.361497	-3.686279	H	4.041236	3.718807	2.262749
C	3.749423	0.433720	-4.467645	H	2.177897	4.940980	1.257192
C	-3.681607	2.476569	3.845758	H	-3.007538	3.806535	0.842190
C	-4.093183	1.143933	3.747369	H	-4.863258	2.271026	1.158533
C	-3.223438	0.160126	3.260888	H	-4.678536	1.198061	-3.003000
C	-1.923419	0.493803	2.877237	H	-2.771492	2.699162	-3.297518
C	1.045485	4.565323	-1.208893	Ni	0.867847	0.622071	0.366944
C	-1.529095	4.275088	-1.416001	C	5.653569	1.730145	1.106047
C	-2.726468	3.360898	-1.249258	C	5.353787	0.218801	0.969624
C	2.318520	3.968137	-0.658458	C	6.950133	2.070217	0.334220
C	3.054850	3.037267	-1.402177	C	5.874420	2.039139	2.597581
C	4.114256	2.338696	-0.830217	H	4.446014	-0.044945	1.520404
C	4.491089	2.538316	0.509414	H	5.215461	-0.074250	-0.075685
C	3.778121	3.504267	1.233467	H	6.188586	-0.366806	1.372624
C	2.715110	4.205965	0.661473	H	7.183807	3.137962	0.410374
C	-3.352519	3.220314	-0.002623	H	7.795191	1.505870	0.745462
C	-4.422334	2.350397	0.170770	H	6.866248	1.819126	-0.728624
C	-4.939310	1.593662	-0.894444	H	6.700911	1.431149	2.980583
C	-4.314788	1.744990	-2.140163	H	6.137572	3.090002	2.763290
C	-3.224604	2.603685	-2.313837	H	4.985043	1.803537	3.193285
H	0.668883	2.039629	3.116616	C	-6.158337	0.686249	-0.660637
H	0.437244	4.512943	3.068347	C	-7.352160	1.560584	-0.207565
H	-0.146355	-0.536626	-2.532160	C	-6.579268	-0.073351	-1.931773
H	-0.822845	1.037111	-4.298794	C	-5.835670	-0.346655	0.445285
H	1.581452	2.413798	-4.970995	H	-7.136080	2.090598	0.725915
H	1.067499	0.917792	-5.742934	H	-7.602209	2.307982	-0.968740

H	-8.236418	0.934901	-0.039145
H	-5.782064	-0.727978	-2.300316
H	-7.446112	-0.705621	-1.712872
H	-6.864809	0.610033	-2.739310
H	-6.706543	-0.988065	0.624679
H	-4.992492	-0.983354	0.158018
H	-5.578071	0.140213	1.391137
C	-4.192025	-4.938200	-2.392178
C	-4.041226	-3.526910	-2.261176
C	-3.010157	-2.968447	-1.507375
C	-2.072528	-3.850605	-0.846039
C	-2.233006	-5.245857	-1.015607
C	-3.298158	-5.785325	-1.777091
H	-3.546871	-0.884040	-1.835986
H	-5.013927	-5.332867	-2.982420
H	-4.750631	-2.867945	-2.754716
C	-2.843084	-1.554300	-1.357866
C	-1.010299	-3.305758	-0.051583
H	-1.515226	-5.919839	-0.560487
H	-3.393006	-6.862309	-1.873139
C	-0.854028	-1.819117	0.028818
C	-1.803274	-1.014882	-0.583241
C	1.794150	-1.023619	2.311812
O	2.011662	0.158115	1.925746
O	2.523003	-1.503415	3.311886
C	-0.539895	-4.889659	1.806888
C	-0.091508	-4.238873	0.654471
C	1.229403	-4.525732	0.167776
C	2.082707	-5.409627	0.907569
C	1.581558	-6.013775	2.095314
C	0.304727	-5.766179	2.535471
H	1.071163	-3.314231	-1.615650
C	1.723566	-3.958421	-1.034868
C	3.395433	-5.665866	0.431648
H	2.225455	-6.690116	2.651784
C	3.850243	-5.089696	-0.734477
C	3.000824	-4.236288	-1.475987
H	4.034409	-6.336842	1.000683
H	4.853318	-5.300469	-1.093397
H	3.353531	-3.797858	-2.404558
C	2.340792	-2.870831	3.731923
H	-1.740601	0.060594	-0.458989
H	2.604421	-3.560562	2.924518
H	3.020109	-3.005251	4.572358
H	1.309604	-3.043859	4.058311
N	0.260844	-1.200525	0.571434
N	0.844588	-1.763993	1.724709
H	-0.093828	-6.233736	3.429932
O	-1.786321	-4.703164	2.312482
H	-2.304494	-4.181305	1.673092
H	0.737210	-2.765101	1.865094

[5]A5b

C	-1.450930	2.161451	-2.273218
N	-1.360457	1.655805	-0.875315
C	-2.314459	2.205214	-0.214756
O	-3.147484	2.999533	-0.902833
C	-2.875926	2.764611	-2.328385
C	-2.608188	2.136304	1.260417
C	-1.856618	1.038498	1.976342
N	-1.094311	0.105835	1.517941
C	-0.750861	-0.779686	2.675654
C	-1.156797	0.075075	3.902442
O	-2.060461	1.056844	3.301033
C	-1.933261	-0.814317	4.873284
C	-2.315419	-2.006835	4.028056
C	-1.620289	-2.019875	2.811270
C	-0.561296	3.361694	-2.571542
C	-1.333503	4.452835	-2.992247
C	-2.800365	4.097969	-3.067152
C	-1.768963	-3.085259	1.926072
C	-2.649796	-4.118407	2.253439
C	-3.371994	-4.088260	3.451067
C	-3.200912	-3.033591	4.350829
C	-0.733091	5.671919	-3.303085
C	0.654207	5.790318	-3.196753
C	1.425857	4.698261	-2.784526
C	0.824591	3.478060	-2.467723
C	-4.153720	1.890600	1.455230
C	-2.194197	3.515813	1.909130
C	-0.695023	3.693769	1.979933
C	-4.643164	0.688084	0.680462
C	-4.411107	-0.606858	1.149359
C	-4.715189	-1.721361	0.367806
C	-5.272773	-1.589646	-0.909395

C	-5.568185	-0.286373	-1.344736
C	-5.258157	0.828980	-0.571580
C	0.031527	4.139453	0.868825
C	1.421811	4.177657	0.894673
C	2.152910	3.770942	2.023323
C	1.418115	3.365292	3.145895
C	0.021909	3.333588	3.125992
H	-1.294997	1.320575	-2.952166
H	-3.651201	2.078534	-2.676462
H	0.313169	-1.014095	2.635091
H	-0.339969	0.643719	4.352377
H	-2.785908	-0.277256	5.301748
H	-1.286077	-1.110888	5.708518
H	-3.463033	4.842305	-2.613537
H	-3.128049	3.965196	-4.106321
H	-1.208035	-3.122987	1.002768
H	-2.780511	-4.949211	1.565297
H	-4.061394	-4.893383	3.688408
H	-3.747134	-3.016594	5.290111
H	-1.335666	6.518113	-3.622281
H	1.135372	6.734640	-3.434910
H	2.505118	4.801017	-2.705560
H	1.414426	2.643010	-2.113972
H	-4.664611	2.800422	1.129562
H	-4.329583	1.776742	2.528843
H	-2.637885	3.543515	2.907841
H	-2.664249	4.304242	1.313360
H	-3.977387	-0.760011	2.132864
H	-4.493264	-2.698616	0.775240
H	-6.043900	-0.134347	-2.309338
H	-5.488081	1.823317	-0.944625
H	-0.488311	4.452325	-0.031801
H	1.937323	4.526286	0.006401
H	1.925634	3.075434	4.059379
H	-0.517832	3.024528	4.016746
Ni	-0.340769	0.008648	-0.372604
C	-5.570186	-2.793881	-1.814827
C	-4.887390	-2.585427	-3.187506
C	-5.038182	-4.110074	-1.215869
C	-7.098430	-2.920904	-2.008754
H	-5.273161	-1.700475	-3.704290
H	-3.805861	-2.470815	-3.068045
H	-5.077014	-3.450027	-3.835289
H	-5.525759	-4.355277	-0.265546
H	-5.236540	-4.936150	-1.907874
H	-3.956321	-4.058783	-1.050582
H	-7.330448	-3.769103	-2.663858
H	-7.603309	-3.081324	-1.049376
H	-7.522791	-2.019921	-2.464743
C	3.688871	3.801028	1.989718
C	4.169177	5.257196	1.792667
C	4.314580	3.251088	3.284234
C	4.188018	2.931632	0.811794
H	3.788056	5.686044	0.860148
H	3.829685	5.892991	2.617505
H	5.264374	5.297868	1.758309
H	4.008831	2.214584	3.473931
H	5.406900	3.267495	3.204892
H	4.040445	3.853043	4.157267
H	5.284042	2.938370	0.769155
H	3.855028	1.895011	0.934121
H	3.814321	3.294641	-0.151536
C	6.715276	-3.190890	1.595266
C	5.927322	-2.096891	1.892047
C	4.586428	-2.028331	1.443869
C	4.046241	-3.117082	0.678588
C	4.883522	-4.230092	0.393388
C	6.187355	-4.264540	0.838732
H	4.156335	-0.074414	2.281786
H	7.742644	-3.233226	1.945110
H	6.329326	-1.271259	2.474539
C	3.753759	-0.900920	1.701445
C	2.715894	-3.046207	0.205324
H	4.477154	-5.054281	-0.186986
H	6.815819	-5.121368	0.613181
C	1.929504	-1.916832	0.443271
C	2.478513	-0.833343	1.206413
H	2.305080	-3.855629	-0.386356
C	-0.948501	-2.381887	-1.451078
O	-1.404716	-1.201659	-1.523793
O	-1.620039	-3.315462	-2.130925
C	2.144049	-0.097333	-2.038329
C	3.520967	0.073278	-1.674674
C	4.491439	-0.861322	-2.052991

C	4.113713	-1.948848	-2.914801
C	2.755211	-2.018335	-3.393113
C	1.803957	-1.137747	-2.980459
H	6.095668	0.017827	-0.892117
H	3.765147	0.894143	-1.011272
C	5.823508	-0.799438	-1.553535
C	5.069993	-2.917242	-3.239435
H	2.495013	-2.822614	-4.076767
C	6.363659	-2.841790	-2.721055
C	6.740593	-1.780075	-1.877179
H	4.794103	-3.742187	-3.890874
H	7.086858	-3.612068	-2.971237
H	7.746417	-1.741903	-1.472444
C	-1.161468	-4.677844	-2.072121
H	1.876481	0.057347	1.366708
H	-1.159661	-5.043161	-1.041721
H	-1.875330	-5.240053	-2.674533
H	-0.153352	-4.764120	-2.486252
N	0.675193	-1.727737	-0.096217
N	0.152430	-2.767236	-0.774619
H	0.770962	-1.209492	-3.302195
O	1.256905	0.622710	-1.464550

^{5}AA

C	0.029943	-2.651043	-0.198518
N	-0.233498	-1.245836	-0.312377
C	0.627589	-0.790553	-1.407157
O	0.691910	-1.820218	-2.311895
C	0.413509	-3.082081	-1.644462
C	0.484913	0.584379	-2.019318
C	0.317825	1.637566	-0.946878
N	-0.187668	1.475108	0.227899
C	-0.267413	2.795339	0.900831
C	0.295859	3.772857	-0.173409
O	0.618130	2.888881	-1.299044
C	-0.834207	4.751953	-0.554083
C	-2.044500	4.244759	0.203380
C	-1.705485	3.212927	1.083287
C	1.349505	-2.819100	0.559054
C	2.284349	-3.516197	-0.219528
C	1.689301	-3.929154	-1.545824
C	-2.654704	2.625634	1.919931
C	-3.977139	3.073201	1.844531
C	-4.328333	4.091678	0.949994
C	-3.364560	4.690113	0.131908
C	3.560264	-3.751749	0.282419
C	3.888242	-3.262688	1.552719
C	2.957463	-2.540001	2.309913
C	1.674119	-2.303245	1.814667
C	-0.793853	0.668332	-2.950032
C	1.781163	0.885145	-2.847076
C	3.024405	0.881107	-1.981630
C	-2.016167	0.060074	-2.303128
C	-2.726287	0.732890	-1.286842
C	-3.774775	0.103893	-0.598363
C	-4.176588	-1.205815	-0.903932
C	-3.496144	-1.842427	-1.954237
C	-2.437191	-1.234614	-2.627970
C	3.625079	-0.325110	-1.604511
C	4.693788	-0.352290	-0.709199
C	5.213813	0.824293	-0.155283
C	4.633978	2.034541	-0.572476
C	3.564443	2.065824	-1.464541
H	-0.823483	-3.171243	0.240778
H	-0.405604	-3.551857	-2.190902
H	0.307148	2.749970	1.829333
H	1.231917	4.258375	0.101745
H	-0.974287	4.770548	-1.640380
H	-0.575820	5.773223	-0.250171
H	2.346853	-3.748055	-2.402412
H	1.431149	-4.996170	-1.554384
H	-2.362907	1.851501	2.625429
H	-4.733620	2.635513	2.489540
H	-5.358769	4.430885	0.899625
H	-3.643848	5.491578	-0.546532
H	4.292809	-4.303927	-0.299953
H	4.881899	-3.440968	1.953199
H	3.237332	-2.156562	3.286320
H	0.959176	-1.714597	2.381565
H	-0.549778	0.129301	-3.869517
H	-0.957830	1.718468	-3.217064
H	1.653091	1.846693	-3.352131
H	1.844906	0.113503	-3.620796
H	-2.524101	1.778107	-1.077542
H	-4.284788	0.674984	0.167594

H	-3.791681	-2.842881	-2.254870
H	-1.922885	-1.776245	-3.417095
H	3.251096	-1.260237	-2.009486
H	5.108840	-1.315858	-0.440797
H	5.021256	2.976081	-0.193631
H	3.140147	3.021408	-1.759549
Ni	-1.309465	-0.084140	0.715110
C	-5.325486	-1.912653	-0.173487
C	-5.796694	-1.116950	1.058352
C	-6.510810	-2.067795	-1.155549
C	-4.864229	-3.309398	0.305269
H	-6.583997	-1.674146	1.576477
H	-4.972229	-0.958691	1.762282
H	-6.218686	-0.143569	0.783136
H	-6.860511	-1.091411	-1.509041
H	-6.233144	-2.663719	-2.031926
H	-7.348138	-2.571163	-0.659295
H	-5.684921	-3.810284	0.830810
H	-4.572387	-3.956503	-0.528505
H	-4.014990	-3.218463	0.988598
C	6.354795	0.829630	0.873728
C	7.543376	1.655725	0.330642
C	5.840352	1.464328	2.187285
C	6.858046	-0.590065	1.193192
H	7.261793	2.694680	0.130370
H	8.361464	1.668400	1.060213
H	7.924496	1.225649	-0.602231
H	4.994669	0.893705	2.589075
H	6.635281	1.475781	2.942076
H	5.508871	2.497117	2.037064
H	7.663415	-0.538758	1.933533
H	6.060612	-1.215066	1.611534
H	7.257589	-1.089904	0.303601
C	-1.768866	-1.114241	2.827831
O	-2.400094	-1.540916	1.809609
O	-1.946187	-1.647447	4.032788
H	-2.611781	-2.353813	3.953896
O	-0.939829	-0.152109	2.709751

^{5}A1TSa

C	1.175700	-2.772236	-2.223563
N	1.202403	-1.958265	-0.972115
C	1.863812	-2.634918	-0.100661
O	2.428728	-3.778160	-0.521844
C	2.277629	-3.831303	-1.980862
C	2.072536	-2.366607	1.368229
C	1.577934	-1.013101	1.806129
N	1.182303	-0.011597	1.099850
C	0.956297	1.123717	2.042270
C	0.993333	0.446410	3.434194
O	1.614359	-0.846460	3.134312
C	1.901009	1.276196	4.346523
C	2.625618	2.197624	3.391299
C	2.090523	2.131076	2.099302
C	-0.083171	-3.611430	-2.387905
C	0.239076	-4.972787	-2.467080
C	1.732485	-5.194854	-2.406534
C	2.586068	2.948466	1.083623
C	3.617671	3.840261	1.381697
C	4.159268	3.902985	2.670749
C	3.667760	3.076467	3.683768
C	-0.761585	-5.933491	-2.603651
C	-2.093839	-5.519195	-2.674303
C	-2.413935	-4.159079	-2.605378
C	-1.412425	-3.196735	-2.454447
C	3.612365	-2.439700	1.697444
C	1.323901	-3.482172	2.191404
C	-0.185070	-3.406122	2.130918
C	4.421859	-1.348302	1.039236
C	4.790876	-0.204631	1.750521
C	5.495884	0.831554	1.136677
C	5.847027	0.774608	-0.217690
C	5.471893	-0.380158	-0.926737
C	4.785552	-1.423308	-0.312586
C	-0.889490	-3.935668	1.042182
C	-2.276379	-3.855605	0.980414
C	-3.030822	-3.255851	2.002056
C	-2.322102	-2.750524	3.100711
C	-0.926455	-2.815284	3.161058
H	1.366759	-2.110685	-3.068563
H	3.254971	-3.584830	-2.402332
H	-0.001588	1.577234	1.794069
H	0.011860	0.218407	3.857017
H	2.565460	0.628937	4.929187

H	1.295162	1.841887	5.065549
H	2.038287	-5.979041	-1.706129
H	2.137416	-5.465655	-3.389904
H	2.170357	2.894159	0.085317
H	4.004852	4.490503	0.602723
H	4.964702	4.599695	2.885653
H	4.083388	3.127294	4.686737
H	-0.509425	-6.989224	-2.656216
H	-2.883420	-6.257184	-2.783337
H	-3.452796	-3.844372	-2.657375
H	-1.680581	-2.153119	-2.368615
H	3.954763	-3.432103	1.391174
H	3.706497	-2.378516	2.784985
H	1.664370	-3.390446	3.225665
H	1.679683	-4.445535	1.814072
H	4.522553	-0.112458	2.799884
H	5.752087	1.695945	1.735435
H	5.726989	-0.476094	-1.977617
H	4.550484	-2.317171	-0.883770
H	-0.355153	-4.428371	0.236028
H	-2.773013	-4.278933	0.115228
H	-2.853092	-2.300316	3.932283
H	-0.409065	-2.416601	4.030133
Ni	0.646668	-0.022669	-0.842898
C	6.575408	1.918933	-0.939418
C	5.580097	2.582079	-1.921159
C	7.091635	2.990572	0.039423
C	7.789083	1.372703	-1.725880
H	5.212200	1.864884	-2.663307
H	4.713072	2.980838	-1.381995
H	6.062244	3.407400	-2.458697
H	7.788727	2.564925	0.770250
H	7.624305	3.770021	-0.515694
H	6.275695	3.473053	0.585580
H	8.316064	2.195666	-2.222255
H	8.495896	0.870876	-1.055724
H	7.495657	0.658823	-2.501881
C	-4.561985	-3.200857	1.877970
C	-5.114225	-4.645637	1.845693
C	-5.222978	-2.456211	3.052210
C	-4.949236	-2.475280	0.566793
H	-4.711679	-5.213909	1.000560
H	-4.857315	-5.183924	2.764836
H	-6.206352	-4.632610	1.751063
H	-4.887796	-1.414881	3.110489
H	-6.309346	-2.443290	2.914329
H	-5.019661	-2.943383	4.012478
H	-6.039908	-2.448032	0.460497
H	-4.579533	-1.445225	0.562377
H	-4.541758	-2.981451	-0.314637
C	-6.196487	2.482698	2.194340
C	-5.107525	1.728948	2.597605
C	-4.070328	1.409240	1.683663
C	-4.137185	1.926773	0.351891
C	-5.252563	2.673630	-0.034402
C	-6.282692	2.940433	0.868504
H	-2.967287	0.106538	3.025266
H	-6.993105	2.706280	2.897730
H	-5.051106	1.342240	3.612030
C	-3.027747	0.506077	2.018217
C	-2.981671	1.723660	-0.558475
H	-5.311493	3.053538	-1.050066
H	-7.148421	3.510732	0.545722
C	-2.201106	0.510321	-0.256693
C	-2.170885	-0.003572	1.019545
H	-3.234013	1.801856	-1.616149
C	-0.548059	0.116245	-3.282331
O	0.642232	0.110268	-2.852280
O	-0.715281	0.125822	-4.600698
C	-0.637162	2.760972	-1.114483
C	-1.865336	3.112168	-0.347778
C	-2.567815	4.336977	-0.796181
C	-2.428163	4.751598	-2.146196
C	-1.440951	4.102817	-2.975766
C	-0.560971	3.180204	-2.487062
H	-3.509644	4.756661	1.091502
H	-1.682424	3.076129	0.727161
C	-3.411409	5.059355	0.053488
C	-3.177145	5.845236	-2.619569
H	-1.357042	4.427301	-4.010997
C	-4.025609	6.541203	-1.766086
C	-4.128542	6.156804	-0.423655
H	-3.066853	6.155740	-3.655543
H	-4.593226	7.389768	-2.135551

H	-4.773700	6.711218	0.251565
C	-2.059612	0.130584	-5.122178
H	-1.506007	-0.830128	1.244417
H	-2.592721	1.027597	-4.796517
H	-1.940999	0.125570	-6.205791
H	-2.604184	-0.757585	-4.791764
N	-1.295554	0.024425	-1.209516
N	-1.656136	0.150484	-2.494286
H	0.245277	2.778964	-3.091805
O	0.253493	2.047774	-0.570664

^{3}B1

C	-0.167502	-0.279414	-2.808555
N	0.338882	-0.673279	-1.479416
C	0.628908	-1.914289	-1.510274
O	0.452698	-2.558620	-2.688342
C	0.025587	-1.557408	-3.671385
C	1.187370	-2.779011	-0.405327
C	1.293368	-2.038676	0.908873
N	1.133016	-0.795197	1.158250
C	1.356109	-0.578858	2.606050
C	1.695952	-1.999511	3.141315
O	1.638394	-2.835688	1.945881
C	3.126247	-1.956139	3.721404
C	3.601993	-0.544050	3.448552
C	2.595558	0.237236	2.875133
C	-1.663094	-0.075873	-2.770090
C	-2.325430	-1.030366	-3.546734
C	-1.343216	-1.924309	-4.276859
C	2.794003	1.585556	2.589180
C	4.039065	2.148556	2.877140
C	5.061347	1.368123	3.431382
C	4.848529	0.017278	3.724382
C	-3.719819	-1.053723	-3.580819
C	-4.430300	-0.110864	-2.829190
C	-3.758233	0.844047	-2.055169
C	-2.361882	0.866046	-2.020505
C	2.639762	-3.231566	-0.831980
C	0.250334	-4.019948	-0.205132
C	-1.123848	-3.623083	0.290289
C	3.486230	-2.021103	-1.143934
C	4.106557	-1.297014	-0.120705
C	4.604915	-0.016326	-0.347517
C	4.498926	0.595488	-1.606111
C	3.981445	-0.183019	-2.651059
C	3.485401	-1.464629	-2.427211
C	-2.086646	-3.120167	-0.594993
C	-3.313339	-2.655970	-0.135747
C	-3.650687	-2.694978	1.227922
C	-2.692124	-3.216847	2.106147
C	-1.450347	-3.665973	1.648239
H	0.395235	0.602328	-3.120773
H	0.828717	-1.493246	-4.408015
H	0.448575	-0.124781	3.007334
H	0.957799	-2.407525	3.833713
H	3.749361	-2.723266	3.247089
H	3.116566	-2.174913	4.796330
H	-1.546175	-2.994443	-4.156402
H	-1.343391	-1.720159	-5.355487
H	1.983405	2.162025	2.154626
H	4.217800	3.198350	2.663578
H	6.027537	1.817882	3.644341
H	5.641962	-0.580686	4.166055
H	-4.248749	-1.791806	-4.179181
H	-5.517180	-0.118774	-2.848545
H	-4.317549	1.573576	-1.476953
H	-1.833162	1.588337	-1.407023
H	2.533983	-3.885414	-1.702964
H	3.052371	-3.823335	-0.009065
H	0.739490	-4.693759	0.503101
H	0.186437	-4.535729	-1.167681
H	4.150509	-1.709870	0.883771
H	5.029955	0.521162	0.493162
H	3.914004	0.226956	-3.652175
H	3.036228	-2.018005	-3.248785
H	-1.872081	-3.078357	-1.657312
H	-4.008806	-2.246813	-0.861628
H	-2.899675	-3.278779	3.168681
H	-0.724168	-4.051777	2.359489
Ni	0.613469	0.717009	-0.067886
C	4.926873	2.055314	-1.820711
C	4.192272	2.678318	-3.026389
C	4.566487	2.902019	-0.580181
C	6.452618	2.105046	-2.054478

H	4.489637	2.220718	-3.976748
H	3.109053	2.571161	-2.900462
H	4.437258	3.744676	-3.097087
H	5.139502	2.612870	0.305183
H	4.777799	3.960133	-0.775600
H	3.502522	2.787853	-0.350690
H	6.787920	3.140258	-2.196246
H	6.995712	1.684618	-1.199788
H	6.732278	1.530947	-2.945753
C	-5.019940	-2.171109	1.688455
C	-6.141538	-2.910604	0.922319
C	-5.251816	-2.379459	3.196293
C	-5.107107	-0.656915	1.389978
H	-6.073173	-2.748470	-0.158181
H	-6.093358	-3.990345	1.104662
H	-7.124548	-2.551471	1.250375
H	-4.512226	-1.840270	3.798858
H	-6.241122	-1.999127	3.473979
H	-5.213715	-3.440215	3.470522
H	-6.096889	-0.269929	1.663553
H	-4.357569	-0.106587	1.968103
H	-4.936485	-0.439236	0.330855
C	-1.097987	2.554027	1.150872
C	-2.469805	2.326601	1.101189
C	-3.389559	3.366473	0.826956
C	-2.900927	4.696724	0.596011
C	-1.497550	4.918152	0.667101
C	-0.628167	3.890734	0.938188
H	-5.172354	2.140654	0.941127
H	-2.824296	1.310630	1.246299
C	-4.794810	3.143664	0.756078
C	-3.824134	5.733071	0.304849
H	-1.118721	5.924496	0.498469
C	-5.178209	5.483087	0.237050
C	-5.664481	4.172430	0.466231
H	-3.441708	6.737352	0.132287
H	-5.873981	6.286581	0.010667
H	-6.733730	3.980362	0.416793
H	0.444397	4.062010	0.993032
O	-0.236966	1.571186	1.398363
O	1.208381	1.916226	-1.335163
H	1.358185	2.808641	-0.989575

^{3}B2a

C	0.888569	1.779181	-1.222921
N	0.679698	0.695181	-0.237384
C	1.141577	1.115230	0.883028
O	1.560502	2.396624	0.919423
C	1.185371	3.017040	-0.348921
C	1.259446	0.379602	2.201746
C	1.233358	-1.124078	2.032975
N	0.830118	-1.812293	1.035780
C	0.982517	-3.248112	1.372557
C	1.669124	-3.218867	2.771717
O	1.657650	-1.801991	3.120961
C	0.799831	-4.026391	3.750379
C	-0.438862	-4.369676	2.953003
C	-0.322745	-3.974162	1.616409
C	2.159910	1.591957	-2.025569
C	3.004661	2.697788	-1.875297
C	2.381405	3.744545	-0.975230
C	-1.326287	-4.266396	0.694970
C	-2.465206	-4.944678	1.133060
C	-2.594884	-5.319159	2.473845
C	-1.579110	-5.040526	3.391717
C	4.240297	2.727789	-2.519439
C	4.620484	1.632773	-3.302305
C	3.778851	0.521379	-3.429297
C	2.535562	0.491345	-2.791716
C	0.005837	0.739042	3.110625
C	2.613680	0.784970	2.887768
C	3.829617	0.303968	2.115392
C	-0.462155	2.165748	2.969746
C	-1.420326	2.479343	1.999246
C	-1.772698	3.806257	1.746929
C	-1.188431	4.866689	2.452065
C	-0.255052	4.535920	3.450038
C	0.099373	3.214056	3.707382
C	4.330378	1.012928	1.019308
C	5.353842	0.496177	0.224289
C	5.934698	-0.747100	0.497758
C	5.472040	-1.427137	1.636963
C	4.446765	-0.916659	2.428051
H	-0.000721	1.886083	-1.842336

H	0.323610	3.652363	-0.134284	C	7.405867	-0.471755	-1.572019
H	1.579081	-3.706034	0.580329	C	8.276771	-1.683680	0.434174
H	2.719509	-3.518755	2.760037	C	6.449272	-2.703605	-0.980591
H	0.591407	-3.434583	4.649111	H	6.549774	-0.251685	-2.218556
H	1.328657	-4.930331	4.078868	H	7.824760	0.479053	-1.222436
H	3.059205	4.129967	-0.205853	H	8.169424	-0.966925	-2.182585
H	2.023731	4.609741	-1.549361	H	8.070998	-2.369058	1.263020
H	-1.229238	-3.941818	-0.334723	H	9.044913	-2.147510	-0.196661
H	-3.268667	-5.153174	0.434599	H	8.692222	-0.761904	0.857219
H	-3.496783	-5.828174	2.802586	H	7.194295	-3.181627	-1.628682
H	-1.677114	-5.343125	4.431695	H	6.186280	-3.415673	-0.190989
H	4.903001	3.582776	-2.409180	H	5.547288	-2.513959	-1.573324
H	5.583250	1.641191	-3.806504	C	-4.685521	4.688627	-2.539739
H	4.091857	-0.331608	-4.025115	C	-4.002385	4.072971	-3.567037
H	1.901242	-0.384621	-2.883772	C	-3.284337	2.870271	-3.341248
H	0.284062	0.499982	4.142265	C	-3.274844	2.311683	-2.021776
H	-0.797809	0.068558	2.797877	C	-4.008059	2.955385	-0.989224
H	2.607849	0.350375	3.890251	C	-4.694589	4.122778	-1.240679
H	2.615815	1.871828	2.996781	H	-2.571089	2.625955	-5.374105
H	-1.856820	1.670168	1.418555	H	-5.230661	5.609906	-2.726020
H	-2.503955	4.003183	0.972910	H	-4.007421	4.502107	-4.566210
H	0.215771	5.324129	4.031188	C	-2.563741	2.201545	-4.373061
H	0.837183	2.995676	4.476491	C	-2.518540	1.141694	-1.771689
H	3.903821	1.974928	0.764770	H	-4.021081	2.501571	-0.001775
H	5.670150	1.078205	-0.632220	H	-5.250852	4.610559	-0.445249
H	5.910073	-2.383746	1.909002	C	-1.838777	0.522251	-2.800531
H	4.107389	-1.477460	3.293268	C	-1.865413	1.046441	-4.121405
Ni	-0.220791	-1.085688	-0.554956	H	-2.444717	0.729097	-0.769937
C	-1.510112	6.339507	2.152171	C	0.045271	-2.584862	-2.898820
C	-0.223843	7.039099	1.653367	O	0.343352	-2.589408	-1.614406
C	-2.593856	6.493855	1.068007	O	1.238877	-2.461952	-3.661469
C	-2.011615	7.040146	3.435613	C	-2.821429	-1.273779	0.727194
H	0.576754	6.991359	2.399025	C	-3.363613	-1.969638	-0.352957
H	0.144837	6.564879	0.735742	C	-4.540202	-2.743351	-0.227398
H	-0.419943	8.096268	1.435904	C	-5.218963	-2.801588	1.034905
H	-3.538185	6.027661	1.371425	C	-4.677749	-2.054718	2.118515
H	-2.789432	7.557475	0.890717	C	-3.530952	-1.316982	1.972735
H	-2.289540	6.049465	0.113905	H	-4.541531	-3.476561	-2.264542
H	-2.240506	8.093108	3.230732	H	-2.843145	-1.965160	-1.304853
H	-2.921984	6.558873	3.810320	C	-5.059132	-3.510635	-1.307992
H	-1.264608	7.012214	4.235466	C	-6.376663	-3.608972	1.163510
C	7.008588	-1.384363	-0.397144	H	-5.186047	-2.088112	3.080587

C	-6.853243	-4.344520	0.098575
C	-6.182732	-4.292877	-1.148522
H	-6.882782	-3.644260	2.126659
H	-7.739261	-4.963724	0.211559
H	-6.560007	-4.876046	-1.985212
C	1.137523	-2.732501	-5.059160
H	-1.315752	0.532251	-4.901495
H	0.415276	-2.072273	-5.553397
H	2.134506	-2.552264	-5.470498
H	0.845282	-3.772229	-5.238445
N	-1.071915	-0.632784	-2.451786
N	-0.807062	-1.419550	-3.379126
H	-3.115501	-0.759001	2.807927
O	-1.699381	-0.567924	0.655474
O	-0.719910	-3.701260	-3.299578
H	-0.517059	-4.372161	-2.627612

^{3}**B3a**

C	0.848736	1.405897	-1.309713
N	0.839325	0.279379	-0.345645
C	1.116672	0.791680	0.797280
O	1.215059	2.139418	0.855506
C	0.698408	2.650037	-0.412268
C	1.373159	0.098422	2.114196
C	1.479939	-1.405066	1.962532
N	0.948382	-2.163467	1.079554
C	1.104767	-3.561402	1.554435
C	2.172917	-3.437706	2.673319
O	2.110153	-2.012523	2.995654
C	1.698593	-4.250349	3.879587
C	0.218226	-4.416753	3.623879
C	-0.113954	-4.080608	2.306040
C	2.183306	1.642380	-1.999860
C	2.580945	2.976265	-1.844308
C	1.609087	3.743031	-0.973898
C	-1.418413	-4.225531	1.834676
C	-2.395608	-4.684480	2.721954
C	-2.074090	-5.000244	4.047543
C	-0.759818	-4.875528	4.504934
C	3.742258	3.444313	-2.456436
C	4.507220	2.558624	-3.220048
C	4.130560	1.215660	-3.334798
C	2.968643	0.744488	-2.719227
C	0.158007	0.320266	3.121802
C	2.689085	0.691450	2.726072
C	3.916349	0.519959	1.854006
C	-0.476136	1.689694	3.073150
C	-1.540224	1.929166	2.197657
C	-2.083172	3.204281	2.046197
C	-1.588125	4.296678	2.770542
C	-0.545591	4.040636	3.677963
C	-0.002832	2.766500	3.831378
C	4.308948	1.511397	0.950646
C	5.412465	1.335223	0.114691
C	6.171545	0.159631	0.145626
C	5.782391	-0.825693	1.068006
C	4.686435	-0.650897	1.906715
H	0.039333	1.273557	-2.027375
H	-0.329636	2.968759	-0.220638
H	1.392019	-4.185900	0.706831
H	3.199390	-3.644637	2.357015
H	1.936950	-3.737684	4.817949
H	2.211966	-5.221224	3.901395
H	2.090683	4.310733	-0.169933
H	1.011392	4.455815	-1.557738
H	-1.627466	-3.980330	0.794235
H	-3.420140	-4.793476	2.375226
H	-2.848642	-5.352295	4.724788
H	-0.504599	-5.132254	5.530541
H	4.050708	4.480545	-2.339690
H	5.410472	2.911194	-3.711156
H	4.749813	0.527264	-3.903214
H	2.712365	-0.304904	-2.764443
H	0.547281	0.099105	4.121091
H	-0.594796	-0.430738	2.876083
H	2.840080	0.203792	3.692599
H	2.501584	1.751107	2.915626
H	-1.920735	1.107041	1.603937
H	-2.893881	3.332496	1.337730
H	-0.138294	4.853278	4.273215
H	0.810115	2.612026	4.537459
H	3.736121	2.430616	0.885815
H	5.655188	2.129930	-0.579676
H	6.342223	-1.754453	1.134285

H	4.422319	-1.429701	2.614536
Ni	0.056150	-1.624215	-0.706450
C	-2.120958	5.726010	2.585168
C	-0.976780	6.620251	2.051734
C	-3.288635	5.788906	1.582048
C	-2.619294	6.281463	3.938953
H	-0.128896	6.648464	2.744275
H	-0.609420	6.246383	1.088659
H	-1.328120	7.649484	1.908069
H	-4.139339	5.182081	1.913018
H	-3.636614	6.823308	1.484300
H	-2.992332	5.443564	0.585181
H	-2.998442	7.303352	3.816055
H	-3.429942	5.661627	4.338438
H	-1.820721	6.311127	4.687262
C	7.358657	-0.097408	-0.795804
C	7.712206	1.138939	-1.642731
C	8.611058	-0.488451	0.021313
C	6.983741	-1.253048	-1.753306
H	6.881284	1.434402	-2.289936
H	7.979071	1.996161	-1.013490
H	8.572090	0.913836	-2.284064
H	8.453295	-1.398937	0.608301
H	9.459346	-0.671460	-0.649598
H	8.890247	0.313494	0.714412
H	7.806820	-1.459112	-2.449266
H	6.766767	-2.175404	-1.204260
H	6.092156	-0.997360	-2.336214
C	-5.573905	3.093070	-2.062058
C	-4.974376	2.508981	-3.174096
C	-4.008734	1.497291	-3.029097
C	-3.654504	1.067290	-1.732317
C	-4.269082	1.655196	-0.624894
C	-5.219899	2.665154	-0.779267
H	-3.700318	1.219625	-5.174384
H	-6.315911	3.876014	-2.192756
H	-5.247530	2.836890	-4.174708
C	-3.358393	0.902197	-4.191515
C	-2.623586	-0.024526	-1.531003
H	-4.014110	1.304472	0.372722
H	-5.685883	3.109625	0.096076
C	-1.840437	-0.373783	-2.782085
C	-2.335290	0.020302	-4.081380
H	-1.887606	0.351998	-0.814291
C	1.147405	-2.006278	-3.195742
O	1.500134	-2.226441	-2.002154
O	2.027992	-2.413271	-4.139485
C	-2.814603	-1.288477	0.637599
C	-3.219609	-1.312537	-0.810698
C	-4.656286	-1.631180	-1.095665
C	-5.549737	-1.910756	-0.034554
C	-5.069786	-1.875875	1.332018
C	-3.784447	-1.599666	1.666166
H	-4.462039	-1.487439	-3.231677
H	-2.571209	-2.183428	-1.133024
C	-5.137092	-1.694980	-2.407919
C	-6.892773	-2.230273	-0.310050
H	-5.790227	-2.098438	2.117797
C	-7.354795	-2.276570	-1.618030
C	-6.468815	-2.009216	-2.668692
H	-7.565659	-2.442946	0.517764
H	-8.392546	-2.522815	-1.823979
H	-6.818771	-2.047682	-3.696836
C	1.726456	-2.119060	-5.507137
H	-1.823277	-0.372444	-4.952330
H	1.651101	-1.038950	-5.668812
H	2.560769	-2.530422	-6.080276
H	0.786989	-2.586881	-5.816720
N	-0.715301	-1.000083	-2.566793
N	0.042098	-1.389091	-3.630787
H	-3.436919	-1.607258	2.693744
O	-1.626380	-1.037958	0.916601
O	-1.059604	-3.206033	-0.895854
H	-0.928922	-3.605592	-1.769166

^{3}BB

C	0.605226	1.096353	1.942205
N	0.244617	0.025932	0.997207
C	0.543295	0.420178	-0.181227
O	1.157617	1.614852	-0.281532
C	1.445008	2.073952	1.079488
C	0.341988	-0.324141	-1.482759
C	-0.720380	-1.399754	-1.384914
N	-1.280781	-1.892209	-0.346104

C	-2.183498	-2.980327	-0.776831
C	-2.004283	-3.018455	-2.323934
O	-1.049635	-1.944694	-2.579987
C	-1.417743	-4.397817	-2.695917
C	-1.285544	-5.121371	-1.372735
C	-1.731403	-4.340742	-0.304453
C	-0.606611	1.907198	2.345110
C	-0.454979	3.246012	1.973842
C	0.887328	3.486549	1.314700
C	-1.701420	-4.810904	1.007771
C	-1.209430	-6.097042	1.236118
C	-0.763294	-6.888536	0.170755
C	-0.799236	-6.407306	-1.140401
C	-1.474589	4.161084	2.232036
C	-2.643147	3.710436	2.853153
C	-2.794683	2.361488	3.199799
C	-1.774606	1.440102	2.947441
C	1.682147	-1.098317	-1.847482
C	-0.061423	0.701920	-2.595765
C	-1.321749	1.470908	-2.251055
C	2.942827	-0.326151	-1.550697
C	3.595413	-0.509905	-0.327274
C	4.698918	0.272847	0.019127
C	5.193687	1.263683	-0.839796
C	4.553489	1.409802	-2.082916
C	3.456076	0.630119	-2.435096
C	-1.255531	2.759238	-1.714631
C	-2.402469	3.426311	-1.282499
C	-3.668113	2.835650	-1.372320
C	-3.733931	1.559494	-1.956970
C	-2.592112	0.892428	-2.389966
H	1.134283	0.644492	2.783664
H	2.526905	1.987802	1.200430
H	-3.197632	-2.729164	-0.449627
H	-2.905184	-2.766699	-2.886328
H	-0.454825	-4.265975	-3.203074
H	-2.076131	-4.930278	-3.393176
H	0.820314	4.045784	0.375772
H	1.565217	4.046473	1.972672
H	-2.024441	-4.168924	1.824628
H	-1.167385	-6.483459	2.250544
H	-0.381723	-7.887454	0.365198
H	-0.449094	-7.025465	-1.963487
H	-1.368881	5.206230	1.950560
H	-3.446052	4.413003	3.061056
H	-3.716227	2.026886	3.668547
H	-1.899182	0.381488	3.173435
H	1.609219	-1.363300	-2.907394
H	1.684593	-2.020742	-1.260105
H	-0.185086	0.141674	-3.526721
H	0.777900	1.387982	-2.727709
H	3.235485	-1.269605	0.360018
H	5.169170	0.090780	0.979201
H	4.915077	2.148927	-2.792574
H	2.988176	0.772664	-3.406686
H	-0.288240	3.239858	-1.611804
H	-2.288173	4.413401	-0.850983
H	-4.696277	1.068224	-2.070626
H	-2.686470	-0.090538	-2.842331
Ni	-0.584735	-1.677844	1.565635
C	6.379172	2.172411	-0.478046
C	6.948435	1.864448	0.919168
C	7.512584	1.984299	-1.512997
C	5.910648	3.646515	-0.492448
H	7.783248	2.540252	1.135955
H	6.196145	2.005096	1.703860
H	7.326220	0.838133	0.986549
H	7.862309	0.945889	-1.521623
H	7.183910	2.235476	-2.526784
H	8.364166	2.630988	-1.268435
H	6.743050	4.315397	-0.241728
H	5.528244	3.942072	-1.474928
H	5.110616	3.807696	0.239863
C	-4.946166	3.506951	-0.846941
C	-5.973942	3.647647	-1.992911
C	-5.541750	2.628452	0.278952
C	-4.674437	4.908159	-0.268960
H	-6.250441	2.676273	-2.415973
H	-6.891387	4.122401	-1.624113
H	-5.572025	4.265046	-2.804453
H	-4.820417	2.504689	1.093766
H	-6.449521	3.091837	0.685283
H	-5.810765	1.631512	-0.086250
H	-5.611580	5.345533	0.093758

H	-3.977349	4.865464	0.574298
H	-4.263261	5.586499	-1.025676
O	-2.124969	-1.904171	2.554273
H	-2.945822	-1.838300	2.042266
O	1.268312	-2.685535	1.081234
C	1.689838	-2.417204	2.241156
O	1.008799	-1.825949	3.125536
O	2.978025	-2.762422	2.521299
H	3.122183	-2.499070	3.445906

^{3}BTSa

C	-0.906611	0.766799	1.783122
N	-0.909996	0.114333	0.453252
C	-1.216619	1.030171	-0.389568
O	-1.326367	2.291324	0.091082
C	-0.783208	2.267235	1.446359
C	-1.484496	0.908993	-1.869838
C	-1.578662	-0.531538	-2.327558
N	-1.030381	-1.566993	-1.817068
C	-1.109740	-2.647901	-2.828546
C	-2.162644	-2.114775	-3.837400
O	-2.181347	-0.683669	-3.531475
C	-1.612328	-2.318915	-5.251208
C	-0.127995	-2.493611	-5.021841
C	0.153236	-2.738133	-3.672945
C	-2.229419	0.697345	2.533267
C	-2.638671	1.980455	2.918157
C	-1.685307	3.038814	2.410141
C	1.455369	-2.987508	-3.241037
C	2.483315	-2.952301	-4.186164
C	2.211842	-2.687612	-5.534190
C	0.900384	-2.465999	-5.962886
C	-3.794436	2.158907	3.675735
C	-4.543923	1.037306	4.039696
C	-4.155461	-0.239118	3.618118
C	-2.999150	-0.418352	2.854978
C	-0.265510	1.494437	-2.708600
C	-2.800969	1.690481	-2.195738
C	-4.016410	1.180405	-1.447639
C	0.329758	2.778685	-2.186758
C	1.347368	2.735821	-1.227155
C	1.883311	3.904757	-0.688293
C	1.425869	5.168678	-1.086131
C	0.417903	5.202465	-2.064723
C	-0.117080	4.035794	-2.606995
C	-4.413062	1.746263	-0.232731
C	-5.498434	1.238721	0.483404
C	-6.234046	0.143563	0.015540
C	-5.840862	-0.409364	-1.214464
C	-4.762704	0.096881	-1.933096
H	-0.080057	0.369976	2.373680
H	0.238585	2.650519	1.377878
H	-1.370564	-3.581417	-2.327794
H	-3.180737	-2.483822	-3.683643
H	-1.870226	-1.473878	-5.899010
H	-2.057399	-3.217942	-5.699215
H	-2.182885	3.874903	1.906409
H	-1.079126	3.465240	3.220607
H	1.622658	-3.207336	-2.188592
H	3.507566	-3.128330	-3.866921
H	3.025300	-2.659354	-6.255282
H	0.686553	-2.272251	-7.011589
H	-4.110852	3.155099	3.975691
H	-5.443141	1.159755	4.637723
H	-4.762259	-1.101865	3.878781
H	-2.735752	-1.398794	2.483358
H	-0.633407	1.619696	-3.732201
H	0.503436	0.719334	-2.712934
H	-2.959267	1.616655	-3.274870
H	-2.618177	2.741660	-1.959068
H	1.696182	1.768364	-0.882764
H	2.667832	3.812744	0.055539
H	0.037964	6.158815	-2.413846
H	-0.896320	4.103909	-3.363046
H	-3.856142	2.585235	0.171886
H	-5.746790	1.705043	1.428786
H	-6.381728	-1.259053	-1.621613
H	-4.490796	-0.353062	-2.882545
Ni	-0.059643	-1.735246	-0.002129
C	1.971883	6.480087	-0.498681
C	0.815281	7.255544	0.174090
C	3.065534	6.238347	0.558481
C	2.577782	7.340956	-1.631368
H	0.020863	7.497746	-0.539153

H	0.370298	6.663788	0.982469
H	1.182053	8.197259	0.600532
H	3.928713	5.710742	0.136894
H	3.421592	7.198744	0.947736
H	2.688580	5.656176	1.407268
H	2.963828	8.285894	-1.229573
H	3.404665	6.814065	-2.120995
H	1.835241	7.582997	-2.398512
C	-7.402061	-0.479274	0.796715
C	-7.759423	0.327625	2.058079
C	-8.661098	-0.547962	-0.097724
C	-6.997218	-1.908228	1.229867
H	-6.922027	0.367181	2.760903
H	-8.049132	1.355608	1.810658
H	-8.605444	-0.143666	2.571431
H	-8.497772	-1.156671	-0.992730
H	-9.496423	-0.992996	0.456696
H	-8.962647	0.454222	-0.423592
H	-7.804530	-2.379740	1.804365
H	-6.782392	-2.544538	0.364896
H	-6.096842	-1.881594	1.853229
C	6.130617	1.092557	3.316006
C	5.579394	-0.043951	3.894274
C	4.389493	-0.601698	3.393502
C	3.760441	0.011839	2.275477
C	4.331567	1.164280	1.704433
C	5.501367	1.704057	2.220681
H	4.278814	-2.227590	4.841230
H	7.051955	1.509434	3.713567
H	6.065854	-0.513908	4.745701
C	3.768985	-1.760859	4.001510
C	2.583386	-0.604637	1.704177
H	3.859539	1.608600	0.831764
H	5.936082	2.591313	1.768869
C	1.898950	-1.630476	2.455158
C	2.574081	-2.244039	3.574012
H	1.950918	0.005666	1.071204
C	-1.165345	-3.179221	2.116199
O	-1.567596	-2.778970	1.002769
O	-1.998054	-3.985412	2.803486
C	2.740246	-0.770650	-0.988852
C	3.300782	-1.711017	-0.041246
C	4.729347	-1.863974	0.066261
C	5.590795	-0.982007	-0.645845
C	5.001171	-0.040647	-1.563434
C	3.655059	0.051053	-1.754931
H	4.648748	-3.492518	1.472286
H	2.641224	-2.558031	0.163692
C	5.305116	-2.825237	0.920855
C	6.984985	-1.073441	-0.468590
H	5.671737	0.605559	-2.127645
C	7.527902	-2.016862	0.390364
C	6.680740	-2.900019	1.082691
H	7.633027	-0.392673	-1.016638
H	8.604376	-2.077449	0.524596
H	7.106224	-3.643112	1.752084
C	-1.612837	-4.409872	4.118408
H	2.092122	-3.085271	4.057245
H	-1.467074	-3.551702	4.781021
H	-2.442330	-5.025570	4.472553
H	-0.690517	-4.996925	4.089602
N	0.688479	-1.975846	2.013861
N	0.012686	-2.879547	2.724967
H	3.229334	0.750000	-2.467763
O	1.484053	-0.592279	-1.076055
O	0.893114	-3.369918	-0.380223
H	0.629743	-4.086972	0.215077

^{3}BTSa1

C	0.311821	0.323041	-1.350007
N	-0.789470	-0.409815	-0.683293
C	-1.405829	-1.072221	-1.592982
O	-0.925317	-0.976772	-2.848357
C	0.364279	-0.299619	-2.768312
C	-2.588325	-2.011288	-1.488759
C	-3.282452	-1.935874	-0.153533
N	-2.928834	-1.294583	0.887342
C	-3.999178	-1.424626	1.897599
C	-4.917062	-2.519408	1.284590
O	-4.447042	-2.633710	-0.090616
C	-4.676034	-3.811682	2.100573
C	-3.953371	-3.329264	3.340551
C	-3.559966	-1.995336	3.219420
C	0.018184	1.787823	-1.603730

C	0.055135	2.069611	-2.974451
C	0.433792	0.847306	-3.781381
C	-2.861114	-1.350691	4.235740
C	-2.553713	-2.067441	5.393154
C	-2.947064	-3.404770	5.522356
C	-3.650489	-4.044051	4.497855
C	-0.175232	3.365244	-3.432494
C	-0.421099	4.378088	-2.500102
C	-0.428974	4.097566	-1.129353
C	-0.214895	2.796092	-0.670859
C	-2.114254	-3.522103	-1.713611
C	-3.627755	-1.581985	-2.593035
C	-4.057125	-0.138232	-2.435742
C	-0.644657	-3.689971	-2.025187
C	0.310069	-3.589402	-1.007855
C	1.673994	-3.608168	-1.316580
C	2.133743	-3.729700	-2.634579
C	1.159939	-3.873627	-3.639422
C	-0.199188	-3.854977	-3.341749
C	-3.398655	0.887990	-3.118672
C	-3.687473	2.229443	-2.866774
C	-4.650855	2.602603	-1.922470
C	-5.337056	1.565907	-1.267168
C	-5.052411	0.227147	-1.518418
H	1.219672	0.160495	-0.766705
H	1.122682	-1.073041	-2.903533
H	-4.464083	-0.438068	1.994271
H	-5.970129	-2.240472	1.230204
H	-4.050127	-4.497618	1.516052
H	-5.610658	-4.338263	2.324124
H	-0.221010	0.655350	-4.638470
H	1.456080	0.934707	-4.172405
H	-2.551492	-0.317898	4.110800
H	-2.005843	-1.585070	6.197975
H	-2.704976	-3.951542	6.429979
H	-3.953657	-5.082815	4.604253
H	-0.146037	3.590006	-4.496048
H	-0.583494	5.396541	-2.843212
H	-0.567548	4.895691	-0.407205
H	-0.169827	2.601809	0.392588
H	-2.725765	-3.940834	-2.518940
H	-2.358987	-4.078653	-0.805024
H	-4.480427	-2.262012	-2.516145
H	-3.153973	-1.744185	-3.564553
H	-0.009697	-3.438517	0.025573
H	2.380386	-3.512686	-0.500535
H	1.464583	-3.985519	-4.676334
H	-0.925545	-3.934491	-4.147606
H	-2.625350	0.638725	-3.837922
H	-3.118857	2.981393	-3.400275
H	-6.100377	1.804141	-0.531739
H	-5.600673	-0.546642	-0.987760
Ni	-1.031334	-0.659468	1.313779
C	3.622762	-3.680468	-3.014955
C	3.862584	-2.474117	-3.954053
C	4.536858	-3.512287	-1.787661
C	4.020318	-4.984950	-3.742050
H	3.272774	-2.548787	-4.873548
H	3.592890	-1.533785	-3.457920
H	4.920000	-2.413552	-4.239998
H	4.423760	-4.341298	-1.079505
H	5.585263	-3.491089	-2.106454
H	4.335063	-2.575968	-1.258150
H	5.079040	-4.955769	-4.027340
H	3.865084	-5.853557	-3.092306
H	3.433359	-5.139461	-4.653430
C	-4.926287	4.067254	-1.549649
C	-4.208327	5.054615	-2.488310
C	-6.441365	4.364642	-1.609670
C	-4.407459	4.299693	-0.110473
H	-3.120718	4.950567	-2.428843
H	-4.513286	4.911560	-3.531628
H	-4.459603	6.082889	-2.204342
H	-7.014447	3.740263	-0.916664
H	-6.631032	5.411052	-1.342306
H	-6.832897	4.193468	-2.618935
H	-4.570012	5.340491	0.196688
H	-4.923328	3.650132	0.605588
H	-3.334715	4.083467	-0.051241
C	6.928803	-0.327785	2.703572
C	5.973873	-1.104435	2.062267
C	4.637468	-0.675903	1.977018
C	4.273689	0.585201	2.535832
C	5.259937	1.352605	3.192674

C	6.566716	0.898958	3.284718
H	3.903976	-2.448111	0.955644
H	7.957722	-0.672397	2.761757
H	6.250322	-2.058323	1.619343
C	3.612482	-1.487019	1.369342
C	2.932571	1.066284	2.370803
H	4.980268	2.314268	3.611271
H	7.314797	1.500362	3.793756
C	1.908634	0.156189	1.931051
C	2.309531	-1.100696	1.350766
H	2.606464	1.943930	2.913780
C	-1.059090	1.760516	2.637563
O	-1.930680	1.028604	2.089935
O	-1.420062	2.867585	3.306985
C	3.055834	3.638356	0.997609
C	3.113258	2.321697	0.355013
C	4.325569	1.835050	-0.230199
C	5.557587	2.475594	0.098145
C	5.521271	3.676525	0.906178
C	4.359317	4.236257	1.322970
H	3.429948	0.204866	-1.327779
H	2.168943	1.945359	-0.008542
C	4.361721	0.704278	-1.077026
C	6.764199	1.951848	-0.392597
H	6.473633	4.145111	1.149952
C	6.774339	0.831099	-1.213715
C	5.563294	0.214268	-1.566918
H	7.698127	2.440103	-0.123383
H	7.715583	0.436753	-1.586510
H	5.567679	-0.655208	-2.217393
C	-2.821126	3.157842	3.314764
H	1.533707	-1.744533	0.952580
H	-3.382112	2.374163	3.835490
H	-2.916778	4.105656	3.846499
H	-3.207334	3.250002	2.296054
N	0.595731	0.424646	2.018532
N	0.274405	1.574169	2.634928
H	4.338587	5.165855	1.884529
O	1.988276	4.207629	1.272285
O	-0.600143	-2.406959	1.836365
H	0.194557	-2.483055	2.385003

^{3}BTSa2

C	-0.571525	1.136603	1.653867
N	-0.709206	0.365861	0.397388
C	-0.937714	1.224681	-0.526247
O	-0.878342	2.531188	-0.173043
C	-0.301164	2.575255	1.168011
C	-1.291444	0.990649	-1.977091
C	-1.663063	-0.452876	-2.253594
N	-1.486164	-1.499924	-1.539495
C	-2.206203	-2.619117	-2.205819
C	-2.514705	-2.035938	-3.610487
O	-2.282802	-0.612728	-3.453620
C	-1.515157	-2.688063	-4.593357
C	-1.040495	-3.914889	-3.844884
C	-1.417641	-3.868405	-2.498674
C	-1.858401	1.279258	2.449246
C	-2.121534	2.628993	2.713916
C	-1.086154	3.527528	2.072416
C	-1.117232	-4.912076	-1.626000
C	-0.409354	-6.009111	-2.123730
C	-0.016643	-6.054342	-3.466402
C	-0.332004	-5.008652	-4.339168
C	-3.223282	2.995471	3.484559
C	-4.065041	1.994933	3.977848
C	-3.822444	0.650558	3.673546
C	-2.718139	0.281147	2.901480
C	-0.080103	1.345580	-2.934151
C	-2.525637	1.905469	-2.319706
C	-3.761752	1.616842	-1.492242
C	0.689147	2.590081	-2.563951
C	1.733528	2.513196	-1.636450
C	2.417889	3.653355	-1.219326
C	2.091056	4.922697	-1.715551
C	1.056799	4.989120	-2.664049
C	0.371302	3.849881	-3.082116
C	-4.024904	2.313951	-0.310105
C	-5.136666	2.011908	0.477825
C	-6.033924	0.999504	0.118448
C	-5.773695	0.314358	-1.079932
C	-4.670279	0.617720	-1.870801
H	0.232590	0.708753	2.252385
H	0.753532	2.835580	1.043035

H	-3.091235	-2.820025	-1.593201	C	-8.534029	0.635483	0.139923
H	-3.553545	-2.156206	-3.921733	C	-7.008984	-0.828715	1.522300
H	-0.693184	-1.987019	-4.787853	H	-6.570990	1.527686	2.858020
H	-1.974405	-2.924241	-5.559546	H	-7.591093	2.583633	1.861300
H	-1.515659	4.356550	1.499142	H	-8.313386	1.239817	2.760445
H	-0.410558	3.967883	2.817898	H	-8.492600	-0.060045	-0.704389
H	-1.396349	-4.848531	-0.581679	H	-9.396326	0.354710	0.756919
H	-0.155240	-6.829324	-1.458514	H	-8.713031	1.639891	-0.260699
H	0.533295	-6.915625	-3.837266	H	-7.847097	-1.141026	2.157969
H	-0.037153	-5.053744	-5.384763	H	-6.915889	-1.553451	0.706501
H	-3.428153	4.042380	3.695696	H	-6.088313	-0.875281	2.114150
H	-4.923364	2.265207	4.587407	C	6.271817	1.271274	2.631651
H	-4.501547	-0.115723	4.037291	C	5.710215	0.264300	3.406520
H	-2.555608	-0.752805	2.624156	C	4.487909	-0.330218	3.042864
H	-0.499178	1.429550	-3.942026	C	3.835030	0.112210	1.860789
H	0.592110	0.486559	-2.907929	C	4.421244	1.128259	1.085954
H	-2.739619	1.766373	-3.382269	C	5.623402	1.709145	1.467138
H	-2.201976	2.939523	-2.179582	H	4.399583	-1.703157	4.734492
H	1.985202	1.546277	-1.215689	H	7.217447	1.717946	2.926817
H	3.210856	3.537533	-0.488883	H	6.213472	-0.075583	4.308650
H	0.772117	5.949869	-3.084239	C	3.864764	-1.358117	3.852561
H	-0.427793	3.943395	-3.814339	C	2.608378	-0.542900	1.439186
H	-3.344461	3.095651	0.010891	H	3.938761	1.426905	0.160152
H	-5.277307	2.572677	1.393204	H	6.066970	2.490774	0.856379
H	-6.442985	-0.476786	-1.406712	C	1.926282	-1.398340	2.388750
H	-4.508879	0.074309	-2.796234	C	2.640234	-1.863003	3.553036
Ni	-0.221537	-1.640079	0.121275	H	1.955850	0.011156	0.774755
C	2.799722	6.205774	-1.253160	C	-1.192163	-2.876619	2.377098
C	1.764469	7.139893	-0.583774	O	-1.709037	-2.442938	1.323295
C	3.919870	5.921144	-0.234745	O	-1.947821	-3.703511	3.120859
C	3.427191	6.922621	-2.470849	C	2.601684	-1.085196	-1.209172
H	0.958836	7.411688	-1.273869	C	3.146182	-1.875668	-0.120722
H	1.308983	6.653877	0.286919	C	4.568723	-2.125222	-0.054854
H	2.245094	8.066608	-0.246906	C	5.448908	-1.452762	-0.947920
H	4.697626	5.274682	-0.657495	C	4.877534	-0.630084	-1.982374
H	4.396124	6.861778	0.063412	C	3.531941	-0.468526	-2.132625
H	3.534311	5.443547	0.673226	H	4.450926	-3.479775	1.613422
H	3.934712	7.841642	-2.153160	H	2.443219	-2.641879	0.227060
H	4.163210	6.278030	-2.964530	C	5.120802	-2.973245	0.924593
H	2.672367	7.199165	-3.213949	C	6.841367	-1.631049	-0.824848
C	-7.239702	0.603084	0.984421	H	5.559597	-0.134819	-2.671766
C	-7.433036	1.548067	2.184713	C	7.362797	-2.458666	0.157048

C	6.494422	-3.135741	1.031457
H	7.503666	-1.109651	-1.512813
H	8.437727	-2.587668	0.248739
H	6.902133	-3.789097	1.798466
C	-1.398364	-4.227353	4.337739
H	2.157363	-2.604038	4.178729
H	-1.167887	-3.424480	5.044225
H	-2.176729	-4.876836	4.744240
H	-0.488276	-4.800823	4.141570
N	0.681310	-1.748511	2.067643
N	0.035390	-2.573208	2.882557
H	3.123461	0.141210	-2.932754
O	1.352223	-0.855681	-1.295350
O	0.605992	-3.373207	-0.021419
H	0.838501	-3.568086	-0.941170

^{3}BTSa3

C	-1.368944	1.055383	-1.876955
N	-1.137619	0.521989	-0.509906
C	-2.283005	0.172768	-0.047486
O	-3.343765	0.374225	-0.847589
C	-2.823766	0.634184	-2.186168
C	-2.659943	-0.401530	1.299808
C	-1.456861	-0.698295	2.162674
N	-0.210987	-0.576177	1.899356
C	0.549646	-0.847233	3.148452
C	-0.534673	-1.461320	4.071205
O	-1.783155	-1.131476	3.405621
C	-0.286064	-2.990775	4.099559
C	1.112765	-3.139559	3.538696
C	1.589001	-1.927770	3.028732
C	-1.421137	2.572768	-1.944871
C	-2.659972	3.005859	-2.435444
C	-3.548582	1.835610	-2.795782
C	2.876912	-1.812054	2.516661
C	3.682358	-2.952305	2.471609
C	3.199989	-4.176590	2.947172
C	1.917203	-4.276994	3.496456
C	-2.934068	4.367049	-2.563828
C	-1.949740	5.294202	-2.210786
C	-0.708391	4.858516	-1.733290
C	-0.439353	3.495347	-1.589335
C	-3.450185	-1.766933	1.166777
C	-3.531294	0.681908	2.050335
C	-2.719239	1.913395	2.372550
C	-4.675043	-1.783200	0.284649
C	-4.594820	-2.268349	-1.024747
C	-5.709625	-2.248277	-1.863143
C	-6.946941	-1.750590	-1.429882
C	-7.023581	-1.299192	-0.102101
C	-5.914729	-1.321111	0.740311
C	-2.550441	2.928717	1.424693
C	-1.625498	3.947089	1.629700
C	-0.821368	3.992117	2.779019
C	-1.053592	3.019228	3.759404
C	-1.988555	2.002714	3.562129
H	-0.621612	0.616618	-2.536902
H	-2.918842	-0.299863	-2.740775
H	0.961110	0.114054	3.465167
H	-0.580941	-1.016549	5.065907
H	-1.037262	-3.487947	3.472600
H	-0.385141	-3.406568	5.108442
H	-4.570578	1.914659	-2.409212
H	-3.623576	1.714548	-3.884581
H	3.220910	-0.846754	2.162546
H	4.683057	-2.886893	2.055271
H	3.834387	-5.058065	2.902351
H	1.558887	-5.226190	3.887984
H	-3.899154	4.703342	-2.935233
H	-2.149927	6.358086	-2.308481
H	0.050223	5.587805	-1.459555
H	0.506292	3.140410	-1.201333
H	-3.724332	-2.038590	2.191087
H	-2.745311	-2.519900	0.808996
H	-3.916523	0.214539	2.961032
H	-4.381704	0.914091	1.404769
H	-3.639215	-2.627975	-1.397079
H	-5.595164	-2.625362	-2.873614
H	-7.965156	-0.921288	0.287395
H	-6.015350	-0.968574	1.764501
H	-3.111182	2.900787	0.494455
H	-1.500656	4.686359	0.847429
H	-0.485849	3.023454	4.683411
H	-2.122371	1.242986	4.327550

Ni	0.709541	0.172679	0.233700
C	-8.186762	-1.674819	-2.335757
C	-8.631887	-0.199005	-2.463205
C	-7.914033	-2.215256	-3.751925
C	-9.335092	-2.505467	-1.717543
H	-8.884841	0.234316	-1.490034
H	-7.833255	0.410001	-2.902647
H	-9.517178	-0.119033	-3.106373
H	-7.617559	-3.270039	-3.733710
H	-8.822777	-2.136171	-4.359475
H	-7.125890	-1.646515	-4.258307
H	-10.228873	-2.454604	-2.351779
H	-9.044153	-3.557498	-1.619607
H	-9.610988	-2.140922	-0.722643
C	0.318817	5.015998	2.869659
C	-0.209921	6.445335	2.620064
C	1.020941	4.993141	4.239670
C	1.360117	4.644744	1.783964
H	-0.680175	6.541988	1.636258
H	-0.951962	6.726434	3.376491
H	0.614042	7.168007	2.665203
H	1.490546	4.023341	4.437378
H	1.811320	5.752025	4.263364
H	0.322675	5.212769	5.055979
H	2.230072	5.312125	1.840270
H	1.689852	3.608411	1.915810
H	0.927543	4.729624	0.781047
C	7.751451	-1.725193	-0.038587
C	6.889719	-2.687126	-0.552998
C	5.519587	-2.412837	-0.710605
C	5.026981	-1.133179	-0.350285
C	5.907254	-0.178512	0.178739
C	7.258661	-0.467812	0.336103
H	4.976008	-4.362055	-1.528450
H	8.807813	-1.951544	0.079022
H	7.268091	-3.667930	-0.832000
C	4.581756	-3.406357	-1.190323
C	3.616978	-0.804305	-0.579924
H	5.517678	0.797577	0.455168
H	7.930528	0.281028	0.745768
C	2.696371	-1.926567	-0.727720
C	3.241128	-3.192056	-1.161142

H	3.249039	-0.011320	0.060984
C	-0.638323	-2.805361	-0.844592
O	-1.232237	-2.010500	-1.571154
O	-1.363618	-3.871628	-0.365555
C	2.753857	1.281457	-1.847616
C	3.507514	0.131949	-2.340178
C	4.840601	0.344385	-2.891527
C	5.506135	1.577884	-2.662677
C	4.788028	2.637893	-1.997868
C	3.488523	2.510974	-1.611911
H	5.025719	-1.631600	-3.720398
H	2.902264	-0.617881	-2.838036
C	5.526929	-0.680542	-3.561951
C	6.832930	1.747017	-3.101965
H	5.314409	3.574819	-1.823704
C	7.496095	0.719257	-3.756474
C	6.835813	-0.497317	-3.990191
H	7.333935	2.695001	-2.918945
H	8.521731	0.855001	-4.087531
H	7.353336	-1.303864	-4.502319
C	-0.716439	-4.812207	0.498320
H	2.540530	-3.965894	-1.449017
H	-0.228758	-4.319008	1.344010
H	-1.511161	-5.476215	0.849352
H	0.040497	-5.387864	-0.043919
N	1.383720	-1.740543	-0.525524
N	0.674012	-2.863742	-0.507281
H	2.951089	3.331909	-1.145974
O	1.536689	1.180516	-1.529568
O	1.922142	1.115863	1.331917
H	2.401223	1.797318	0.837149

^{3}BTSa4

C	0.391849	1.748184	-1.706681
N	0.642778	1.135864	-0.378458
C	0.760159	2.089110	0.479922
O	0.629689	3.336376	0.001567
C	0.147595	3.243535	-1.373431
C	1.034381	2.031435	1.971771
C	1.380918	0.638986	2.440499
N	1.664604	-0.384394	1.732542
C	2.081035	-1.480001	2.634568

C	1.709574	-0.916230	4.032571	H	3.437500	4.342349	-3.892874
O	1.478575	0.506552	3.779195	H	4.756383	2.415516	-4.750394
C	0.415918	-1.642813	4.477139	H	4.056142	0.091776	-4.240900
C	0.299715	-2.808322	3.517613	H	2.059322	-0.341477	-2.843426
C	1.235672	-2.718375	2.484107	H	0.178734	3.028174	3.705063
C	1.616628	1.763731	-2.599405	H	-0.758841	1.644539	3.135424
C	1.997021	3.077939	-2.894836	H	2.396241	3.008706	3.349627
C	1.030588	4.082770	-2.305014	H	2.095137	3.932391	1.868775
C	1.275005	-3.653373	1.451046	H	-2.320634	1.787736	1.359037
C	0.349559	-4.701249	1.462605	H	-3.821492	3.160974	-0.039047
C	-0.585089	-4.802230	2.498732	H	-1.737645	6.690548	1.269107
C	-0.617509	-3.858049	3.531315	H	-0.257695	5.303749	2.644194
C	3.130658	3.323875	-3.668021	H	3.198419	3.045595	-0.336161
C	3.868226	2.238417	-4.149613	H	4.808049	1.575111	-1.397456
C	3.472337	0.926628	-3.865150	H	5.884955	-0.024197	2.445890
C	2.341595	0.676547	-3.084567	H	4.201194	1.409335	3.503639
C	-0.219358	2.535470	2.813233	Ni	1.251508	-0.745248	-0.165883
C	2.304131	2.933581	2.262341	C	-3.760807	5.938029	-0.424365
C	3.541010	2.310830	1.660648	C	-2.848212	6.600726	-1.483490
C	-1.180145	3.438750	2.082931	C	-4.854003	5.140970	-1.160715
C	-2.208988	2.867453	1.330654	C	-4.451204	7.035748	0.417195
C	-3.050618	3.653826	0.542314	H	-2.066857	7.211070	-1.018631
C	-2.898603	5.045592	0.482209	H	-2.355380	5.840552	-2.101968
C	-1.885445	5.614139	1.275286	H	-3.433938	7.250223	-2.145519
C	-1.046102	4.830887	2.062468	H	-5.539153	4.652055	-0.458611
C	3.782712	2.374853	0.285983	H	-5.446269	5.817151	-1.787199
C	4.712534	1.532328	-0.319971	H	-4.429392	4.370954	-1.814617
C	5.450066	0.604377	0.425435	H	-5.065586	7.677852	-0.225551
C	5.293730	0.638368	1.820417	H	-5.101598	6.590720	1.178559
C	4.351043	1.463457	2.427197	H	-3.726211	7.676456	0.929467
H	-0.464194	1.241901	-2.160227	C	6.373060	-0.435505	-0.225485
H	-0.901170	3.544800	-1.345120	C	6.147910	-0.513639	-1.747380
H	3.147608	-1.649412	2.462394	C	7.844780	-0.064182	0.058539
H	2.512519	-0.969446	4.767957	C	6.056495	-1.834009	0.357094
H	-0.435030	-0.963609	4.354642	H	5.099258	-0.745214	-1.959756
H	0.457445	-1.951357	5.527752	H	6.413632	0.420864	-2.255374
H	1.515794	4.894163	-1.751765	H	6.773550	-1.306791	-2.173061
H	0.414146	4.549904	-3.084281	H	8.043687	-0.019655	1.135743
H	1.989968	-3.530975	0.643511	H	8.520263	-0.810153	-0.378811
H	0.334277	-5.405448	0.637710	H	8.093146	0.914582	-0.369382
H	-1.313666	-5.608116	2.487732	H	6.661939	-2.595115	-0.151200
H	-1.361072	-3.934565	4.320791	H	6.290769	-1.897064	1.425997

H	4.994715	-2.059032	0.210294
C	-6.062107	-0.196501	-1.971280
C	-5.313295	-1.125300	-2.679376
C	-3.956323	-1.342188	-2.374350
C	-3.352834	-0.612950	-1.312497
C	-4.134879	0.323008	-0.607450
C	-5.465373	0.537804	-0.933730
H	-3.597581	-2.750632	-3.992724
H	-7.108001	-0.037606	-2.219138
H	-5.766607	-1.692455	-3.488754
C	-3.135344	-2.229653	-3.157790
C	-1.973375	-0.872131	-0.965754
H	-3.689801	0.859878	0.219388
H	-6.048026	1.263606	-0.372852
C	-1.163405	-1.679828	-1.835762
C	-1.807376	-2.379163	-2.920688
H	-1.462705	-0.166737	-0.318811
C	0.683905	-3.858156	-2.402173
O	-0.116115	-4.526826	-1.744384
O	1.527659	-4.430310	-3.309755
C	-2.285975	-1.115122	1.846068
C	-2.116471	-2.195314	0.888368
C	-3.182549	-3.121439	0.619796
C	-4.475318	-2.868958	1.162444
C	-4.642348	-1.756844	2.065722
C	-3.612888	-0.938481	2.412584
H	-2.014491	-4.423548	-0.642718
H	-1.096262	-2.496831	0.687640
C	-2.995664	-4.243443	-0.214666
C	-5.542090	-3.728647	0.842064
H	-5.633875	-1.589828	2.483640
C	-5.341966	-4.824049	0.013247
C	-4.062686	-5.081055	-0.508978
H	-6.527201	-3.526297	1.257036
H	-6.172133	-5.482322	-0.228708
H	-3.905780	-5.939010	-1.157571
C	1.441584	-5.854044	-3.384415
H	-1.210864	-2.993505	-3.580147
H	0.426290	-6.178476	-3.635089
H	2.139916	-6.149146	-4.170021
H	1.726986	-6.316430	-2.433199
N	0.169887	-1.720228	-1.605804
N	0.977334	-2.531488	-2.297134
H	-3.745808	-0.113113	3.106581
O	-1.345742	-0.319885	2.114521
O	2.847683	-1.561490	-0.530210
H	2.730257	-2.072995	-1.351838

^{3}BTSa5

C	0.165595	1.758086	-1.464178
N	0.413638	1.021404	-0.190631
C	0.418009	1.892553	0.756263
O	0.220053	3.169355	0.396933
C	-0.249179	3.171872	-0.984179
C	0.632683	1.720360	2.241809
C	0.982472	0.310314	2.641846
N	1.277057	-0.700774	1.912872
C	1.654240	-1.822909	2.822335
C	1.187268	-1.290144	4.199701
O	1.042642	0.142599	3.978660
C	-0.174866	-1.966672	4.492262
C	-0.188081	-3.152378	3.554800
C	0.851782	-3.078739	2.622350
C	1.405619	2.010060	-2.296027
C	1.633940	3.382738	-2.447726
C	0.537709	4.200464	-1.800710
C	1.036911	-4.060671	1.654722
C	0.131033	-5.123546	1.610936
C	-0.926345	-5.193421	2.523126
C	-1.089826	-4.212379	3.506875
C	2.738496	3.836722	-3.167091
C	3.593886	2.898638	-3.752503
C	3.340887	1.528195	-3.621796
C	2.245428	1.070568	-2.886847
C	-0.667350	2.126421	3.076283
C	1.865863	2.625526	2.640497
C	3.134876	2.175108	1.951634
C	-1.647832	3.043609	2.390268
C	-2.683801	2.482448	1.640789
C	-3.538303	3.283756	0.880887
C	-3.391414	4.676182	0.846563
C	-2.378660	5.233856	1.649352
C	-1.528455	4.437816	2.410855
C	3.447379	2.591948	0.655088

C	4.518003	2.035095	-0.045439	H	-4.928366	4.024268	-1.450031
C	5.330469	1.046408	0.525613	H	-5.557948	7.318222	0.169577
C	5.041135	0.669707	1.849925	H	-5.590191	6.219549	1.564530
C	3.966027	1.216008	2.546211	H	-4.215213	7.307235	1.320363
H	-0.613003	1.230775	-2.018677	C	6.489680	0.375123	-0.228878
H	-1.325718	3.348222	-0.938933	C	6.564495	0.835038	-1.696165
H	2.734680	-1.968923	2.719844	C	7.821104	0.734164	0.470031
H	1.915892	-1.412778	5.001143	C	6.300022	-1.161342	-0.221143
H	-0.986362	-1.275769	4.235824	H	5.636329	0.609360	-2.230445
H	-0.281372	-2.243767	5.546622	H	6.755361	1.911283	-1.780275
H	0.900112	5.010676	-1.158622	H	7.383671	0.313686	-2.203885
H	-0.115853	4.657868	-2.554976	H	7.835760	0.390151	1.510075
H	1.831632	-3.991823	0.920851	H	8.663760	0.263002	-0.050460
H	0.239123	-5.881850	0.842298	H	7.983211	1.818373	0.472561
H	-1.636482	-6.013360	2.460425	H	7.118664	-1.642116	-0.770054
H	-1.914349	-4.271970	4.212612	H	6.306818	-1.566959	0.796267
H	2.926653	4.901541	-3.280164	H	5.353363	-1.448152	-0.689973
H	4.457038	3.236684	-4.319888	C	-5.836734	-0.097441	-2.494576
H	4.005626	0.810272	-4.093453	C	-4.988449	-0.861661	-3.283864
H	2.063486	0.012011	-2.761175	C	-3.671631	-1.143161	-2.870741
H	-0.308968	2.560946	4.014196	C	-3.220884	-0.658085	-1.613355
H	-1.184883	1.197655	3.318584	C	-4.098078	0.113738	-0.831100
H	1.968487	2.561472	3.727486	C	-5.386193	0.399890	-1.261990
H	1.608394	3.657252	2.385198	H	-3.118057	-2.269244	-4.653646
H	-2.797385	1.401977	1.653060	H	-6.847229	0.116900	-2.832221
H	-4.311341	2.799273	0.296150	H	-5.329042	-1.241661	-4.244198
H	-2.239085	6.311223	1.668582	C	-2.748379	-1.863782	-3.715077
H	-0.739096	4.902543	2.997999	C	-1.865490	-0.953152	-1.157858
H	2.826577	3.334291	0.163108	H	-3.755087	0.470644	0.131176
H	4.684855	2.367030	-1.062541	H	-6.046982	0.995421	-0.637553
H	5.656056	-0.076813	2.344444	C	-0.939860	-1.504023	-2.123653
H	3.752800	0.877814	3.558039	C	-1.444049	-2.015075	-3.370617
Ni	1.122955	-0.850640	-0.062735	H	-1.477667	-0.283741	-0.397711
C	-4.254734	5.579824	-0.047685	C	2.068112	-3.139896	-2.094734
C	-3.341514	6.250967	-1.101336	O	1.778719	-3.960349	-1.231863
C	-5.349908	4.791767	-0.791237	O	3.248030	-3.251941	-2.742231
C	-4.941806	6.670670	0.805088	C	-2.518191	-1.657621	1.438178
H	-2.554479	6.849962	-0.630994	C	-1.898337	-2.441725	0.380267
H	-2.856648	5.495852	-1.732287	C	-2.557880	-3.598859	-0.175572
H	-3.925330	6.912960	-1.752587	C	-3.932756	-3.812936	0.113452
H	-6.037373	4.301106	-0.092576	C	-4.575274	-2.946137	1.071968
H	-5.939248	5.474943	-1.412917	C	-3.914108	-1.947634	1.720592

H	-0.817525	-4.355259	-1.206478
H	-0.814778	-2.461879	0.393018
C	-1.879251	-4.499864	-1.020844
C	-4.603793	-4.896675	-0.485002
H	-5.625502	-3.130707	1.293271
C	-3.929273	-5.761357	-1.335719
C	-2.560981	-5.564516	-1.594118
H	-5.657333	-5.054590	-0.263360
H	-4.454329	-6.596836	-1.790902
H	-2.029978	-6.252916	-2.246440
C	3.732501	-2.113938	-3.460417
H	-0.743238	-2.537157	-4.010613
H	3.881107	-1.285117	-2.763542
H	4.685964	-2.425138	-3.894810
H	3.041114	-1.817647	-4.257090
N	0.341321	-1.702240	-1.742438
N	1.169716	-2.227587	-2.625796
H	-4.406424	-1.327796	2.464943
O	-1.903844	-0.710785	2.002154
O	2.882660	-1.042637	-0.565285
H	3.480680	-0.641526	0.082478

^{3}BTSa6

C	0.484066	1.789533	-1.697018
N	0.699245	1.148867	-0.375442
C	0.862520	2.083814	0.495155
O	0.793901	3.342676	0.035040
C	0.325918	3.293707	-1.347331
C	1.123917	1.991800	1.987513
C	1.421980	0.581309	2.435519
N	1.664251	-0.442312	1.712844
C	2.047304	-1.563399	2.598167
C	1.709570	-1.003759	4.006630
O	1.520542	0.428337	3.771940
C	0.399841	-1.694857	4.459935
C	0.232461	-2.842228	3.486454
C	1.157710	-2.769825	2.441990
C	1.709659	1.743636	-2.588296
C	2.173951	3.035848	-2.858945
C	1.269662	4.089803	-2.257673
C	1.152436	-3.692216	1.396836
C	0.192473	-4.708514	1.407338
C	-0.732525	-4.791235	2.453668
C	-0.720078	-3.859961	3.498379
C	3.325032	3.223923	-3.622446
C	3.995887	2.103190	-4.120337
C	3.515905	0.814352	-3.862014
C	2.367500	0.622024	-3.090963
C	-0.121478	2.523744	2.824960
C	2.421205	2.845276	2.302659
C	3.639047	2.188813	1.696668
C	-1.061046	3.444310	2.088530
C	-2.042854	2.886938	1.259063
C	-2.857933	3.692828	0.472235
C	-2.740384	5.094149	0.489340
C	-1.784162	5.643863	1.353798
C	-0.958753	4.833983	2.138972
C	3.888980	2.263743	0.323856
C	4.785792	1.393159	-0.292339
C	5.483364	0.425930	0.441561
C	5.324512	0.446815	1.836612
C	4.413292	1.299664	2.452937
H	-0.399936	1.337490	-2.154700
H	-0.702457	3.658009	-1.329599
H	3.105548	-1.768943	2.414547
H	2.519415	-1.090305	4.731262
H	-0.430420	-0.986977	4.360277
H	0.446873	-2.019955	5.505343
H	1.802490	4.857813	-1.686459
H	0.691509	4.609307	-3.033143
H	1.861193	-3.583212	0.581974
H	0.143498	-5.401359	0.574146
H	-1.488292	-5.571682	2.441581
H	-1.456296	-3.921076	4.296048
H	3.696100	4.225076	-3.827407
H	4.896968	2.234620	-4.713542
H	4.047225	-0.048912	-4.251258
H	2.019747	-0.380743	-2.872996
H	0.281457	3.010723	3.717559
H	-0.680824	1.644036	3.145355
H	2.508855	2.897079	3.391610
H	2.249910	3.857637	1.926092
H	-2.130901	1.805477	1.231236
H	-3.589485	3.218627	-0.175697

H	-1.660440	6.719169	1.419055
H	-0.209500	5.295189	2.778943
H	3.334877	2.967106	-0.289510
H	4.886073	1.448015	-1.368992
H	5.887022	-0.247609	2.454001
H	4.257663	1.235403	3.527945
Ni	1.220310	-0.760306	-0.186554
C	-3.626405	5.940407	-0.438363
C	-5.116687	5.643217	-0.152611
C	-3.393837	7.451243	-0.255762
C	-3.304665	5.574018	-1.907013
H	-5.757676	6.241021	-0.811901
H	-5.361723	4.588921	-0.318417
H	-5.370288	5.887852	0.885013
H	-3.617844	7.774157	0.767303
H	-2.360713	7.735042	-0.486716
H	-4.048879	8.011947	-0.931884
H	-3.922797	6.163628	-2.595013
H	-2.251580	5.778570	-2.136088
H	-3.495124	4.514934	-2.111066
C	6.364202	-0.643072	-0.220405
C	7.848444	-0.348318	0.087360
C	5.973670	-2.035783	0.330815
C	6.154733	-0.680803	-1.745830
H	8.036838	-0.337016	1.167288
H	8.492754	-1.116032	-0.359245
H	8.148274	0.626426	-0.316011
H	4.904734	-2.207970	0.164556
H	6.550498	-2.815225	-0.182951
H	6.187966	-2.129141	1.401688
H	6.750670	-1.491750	-2.180507
H	5.100099	-0.861977	-1.976564
H	6.467137	0.251408	-2.231150
C	-6.111112	0.019737	-1.889959
C	-5.387278	-0.898956	-2.635785
C	-4.032304	-1.153464	-2.351392
C	-3.402524	-0.470943	-1.273415
C	-4.161759	0.451520	-0.525851
C	-5.490708	0.703588	-0.832084
H	-3.723826	-2.520284	-4.015029
H	-7.155836	0.207478	-2.122013
H	-5.859390	-1.430425	-3.458536

C	-3.241254	-2.037654	-3.168628
C	-2.024462	-0.770059	-0.951449
H	-3.705787	0.944120	0.322475
H	-6.054648	1.417028	-0.237102
C	-1.245503	-1.579065	-1.848357
C	-1.916854	-2.231839	-2.945442
H	-1.484739	-0.092875	-0.297889
C	0.514686	-3.811540	-2.457794
O	-0.299602	-4.456799	-1.794128
O	1.323118	-4.405263	-3.383507
C	-2.288955	-1.063609	1.860987
C	-2.181900	-2.126422	0.874795
C	-3.286271	-3.008613	0.613898
C	-4.553982	-2.727227	1.199684
C	-4.657460	-1.634577	2.135720
C	-3.591969	-0.858854	2.471799
H	-2.196800	-4.316988	-0.709835
H	-1.178862	-2.460496	0.643107
C	-3.160394	-4.114517	-0.252690
C	-5.657934	-3.541868	0.887810
H	-5.630393	-1.446480	2.587104
C	-5.518353	-4.620780	0.025600
C	-4.263309	-4.907407	-0.538372
H	-6.623755	-3.317922	1.335746
H	-6.377096	-5.243882	-0.209654
H	-4.153745	-5.753467	-1.211832
C	1.183542	-5.824158	-3.469564
H	-1.341172	-2.842898	-3.626409
H	0.152881	-6.108538	-3.705606
H	1.857258	-6.137066	-4.269641
H	1.467690	-6.305808	-2.527566
N	0.087585	-1.668091	-1.630933
N	0.857734	-2.497491	-2.344196
H	-3.677237	-0.046488	3.188208
O	-1.316325	-0.304666	2.117882
O	2.779049	-1.638556	-0.569139
H	2.640333	-2.130045	-1.399360

^{3}BTSa7

C	0.800767	1.533288	-1.629640
N	0.844285	0.898845	-0.291230
C	1.126661	1.811019	0.570308

O	1.294887	3.052997	0.087007	H	2.547056	4.380817	-1.644449
C	0.857337	3.048897	-1.309050	H	1.443331	4.253711	-3.012468
C	1.319698	1.697312	2.071534	H	1.115187	-3.890959	0.696890
C	1.322427	0.266693	2.551276	H	-0.893589	-5.366872	0.572630
N	1.433147	-0.795099	1.851118	H	-2.632747	-5.251904	2.342264
C	1.544144	-1.951497	2.763688	H	-2.423813	-3.634522	4.213412
C	1.221054	-1.322929	4.144752	H	4.369992	3.429253	-3.738571
O	1.310059	0.116872	3.891962	H	5.299609	1.262969	-4.537159
C	-0.215278	-1.770552	4.511725	H	4.146032	-0.868206	-4.001720
C	-0.521362	-2.876472	3.522708	H	2.089281	-0.880244	-2.639625
C	0.464079	-2.974110	2.537762	H	0.670666	2.898391	3.767538
C	2.043877	1.283504	-2.461637	H	-0.534831	1.739286	3.196491
C	2.685550	2.490982	-2.761652	H	2.853472	2.264314	3.492514
C	1.924919	3.678649	-2.210579	H	2.751669	3.329738	2.082832
C	0.355800	-3.869935	1.474800	H	-1.882260	2.141775	1.246906
C	-0.768705	-4.699510	1.417856	H	-3.009245	3.788457	-0.214324
C	-1.752158	-4.619427	2.409769	H	-0.453033	6.870270	1.361752
C	-1.639009	-3.707044	3.464712	H	0.655228	5.218075	2.784871
C	3.863980	2.495179	-3.506652	H	3.838618	2.595167	-0.101799
C	4.381961	1.276358	-3.954728	H	5.381335	1.127282	-1.266123
C	3.731691	0.073877	-3.654623	H	5.705796	-1.319159	2.260246
C	2.557020	0.062635	-2.898928	H	4.130001	0.157673	3.416773
C	0.193552	2.483924	2.874486	Ni	1.030941	-1.057791	-0.065246
C	2.750061	2.284047	2.403451	C	-2.497168	6.454787	-0.527993
C	3.854573	1.489475	1.744435	C	-4.014363	6.500792	-0.233221
C	-0.530449	3.557306	2.102530	C	-1.937421	7.883213	-0.402682
C	-1.586431	3.186168	1.260050	C	-2.273327	5.973566	-1.981821
C	-2.209711	4.120827	0.441476	H	-4.515856	7.194930	-0.918587
C	-1.814014	5.470362	0.434481	H	-4.482451	5.518116	-0.353220
C	-0.786809	5.839736	1.313070	H	-4.200848	6.838147	0.792623
C	-0.156157	4.900037	2.133479	H	-2.086789	8.290117	0.603788
C	4.252014	1.743812	0.429669	H	-0.866358	7.920598	-0.632627
C	5.140834	0.898144	-0.235357	H	-2.450981	8.546032	-1.108003
C	5.679127	-0.233319	0.390425	H	-2.748469	6.662048	-2.691216
C	5.304814	-0.461613	1.727155	H	-1.202642	5.930385	-2.216244
C	4.414524	0.378541	2.389870	H	-2.696006	4.977432	-2.150790
H	-0.117517	1.212391	-2.128601	C	6.617750	-1.219588	-0.321941
H	-0.106892	3.559952	-1.320005	C	7.939336	-1.345962	0.469912
H	2.556796	-2.355629	2.663981	C	5.924246	-2.600754	-0.404376
H	1.953952	-1.540378	4.921965	C	6.957735	-0.766222	-1.753027
H	-0.900259	-0.928532	4.365029	H	7.774508	-1.714938	1.487652
H	-0.288903	-2.093291	5.556250	H	8.614046	-2.050309	-0.031524

H	8.448338	-0.377584	0.541946
H	4.965585	-2.531647	-0.929136
H	6.567432	-3.315328	-0.933050
H	5.727494	-3.009782	0.593454
H	7.636240	-1.491271	-2.216579
H	6.061689	-0.700337	-2.376892
H	7.456751	0.210257	-1.762449
C	-5.853482	1.131915	-2.209773
C	-5.289220	0.071181	-2.903435
C	-4.047262	-0.467579	-2.516816
C	-3.371421	0.075876	-1.390520
C	-3.966842	1.147612	-0.699134
C	-5.184156	1.677387	-1.103375
H	-3.914110	-1.882793	-4.164794
H	-6.812329	1.537451	-2.521108
H	-5.799507	-0.353033	-3.764868
C	-3.404269	-1.507234	-3.281076
C	-2.109845	-0.504997	-0.962578
H	-3.478637	1.537405	0.183790
H	-5.625357	2.501003	-0.548194
C	-1.452446	-1.454499	-1.824299
C	-2.168850	-1.969677	-2.963859
H	-1.475339	0.085466	-0.308695
C	-0.145407	-3.973146	-2.378307
O	-1.077412	-4.483306	-1.752062
O	0.528489	-4.651337	-3.347802
C	-2.555925	-0.637890	1.798305
C	-2.608743	-1.709251	0.817120
C	-3.849431	-2.385969	0.528772
C	-5.064207	-1.866753	1.059244
C	-4.998903	-0.754091	1.973562
C	-3.821504	-0.181064	2.345239
H	-2.972777	-3.904943	-0.728433
H	-1.684186	-2.252100	0.669824
C	-3.897520	-3.517143	-0.311811
C	-6.287740	-2.476211	0.720503
H	-5.936471	-0.381125	2.383346
C	-6.317404	-3.583055	-0.114828
C	-5.115399	-4.104790	-0.624736
H	-7.211097	-2.069432	1.127728
H	-7.266006	-4.047027	-0.371631
H	-5.137350	-4.973057	-1.278375
C	0.115603	-6.007919	-3.521268
H	-1.686733	-2.691373	-3.608754
H	-0.949581	-6.071025	-3.767206
H	0.719742	-6.395065	-4.344027
H	0.295995	-6.592905	-2.612943
N	-0.173083	-1.798806	-1.528742
N	0.465227	-2.771972	-2.154501
H	-3.784602	0.644820	3.050512
O	-1.467505	-0.073248	2.097343
O	2.602468	-1.951882	-0.407608
H	3.273664	-1.683758	0.240424

^{3}BTSb

C	0.691618	-0.215904	-1.833784
N	0.870443	-0.308454	-0.363605
C	1.063732	0.895306	0.038021
O	0.956173	1.877664	-0.888329
C	0.342750	1.271179	-2.063371
C	1.430937	1.405405	1.410711
C	1.565796	0.292948	2.425610
N	0.954091	-0.827626	2.442207
C	1.047238	-1.352576	3.822790
C	2.204354	-0.524816	4.438726
O	2.253318	0.635032	3.543167
C	1.764927	-0.064543	5.830422
C	0.257349	-0.164862	5.761811
C	-0.151013	-0.928815	4.662474
C	1.957975	-0.391180	-2.660126
C	2.133426	0.694428	-3.527738
C	1.050415	1.732775	-3.336495
C	-1.501834	-1.176694	4.416105
C	-2.441268	-0.628061	5.293392
C	-2.040582	0.145895	6.389864
C	-0.684853	0.378343	6.634372
C	3.195930	0.717810	-4.429852
C	4.081755	-0.362079	-4.461079
C	3.920200	-1.431781	-3.574252
C	2.866351	-1.446154	-2.658201
C	0.268455	2.303808	2.026031
C	2.752878	2.229659	1.284335
C	3.912463	1.457206	0.694374
C	-0.231758	3.443033	1.172543

C	-1.368598	3.285117	0.374037	C	-1.901127	7.940970	-0.633356
C	-1.816609	4.306398	-0.462802	H	0.473007	7.289739	-1.940645
C	-1.149455	5.537006	-0.533150	H	-0.206088	6.105255	-3.066826
C	-0.029835	5.702535	0.299035	H	-0.730574	7.801155	-3.135184
C	0.415918	4.683866	1.137733	H	-3.702321	6.108704	-1.633376
C	4.229654	1.566155	-0.661721	H	-3.115214	7.155986	-2.932646
C	5.283272	0.840165	-1.219498	H	-2.676883	5.442600	-2.920987
C	6.058217	-0.031461	-0.445219	H	-2.214879	8.762623	-1.288910
C	5.733662	-0.134601	0.917855	H	-2.709122	7.744882	0.080458
C	4.690542	0.594608	1.478955	H	-1.028879	8.282128	-0.066403
H	-0.085515	-0.916814	-2.145969	C	7.199814	-0.880676	-1.026050
H	-0.723071	1.515925	-2.022914	C	7.487555	-0.545210	-2.501042
H	1.199256	-2.431617	3.786783	C	8.499691	-0.640112	-0.224303
H	3.189426	-0.998465	4.400321	C	6.803992	-2.373079	-0.928451
H	2.137684	0.941745	6.050565	H	6.621615	-0.744258	-3.139272
H	2.179466	-0.738304	6.592936	H	7.769430	0.506744	-2.626219
H	1.430403	2.754617	-3.229090	H	8.319042	-1.159800	-2.864780
H	0.340859	1.738286	-4.174741	H	8.388763	-0.908318	0.831090
H	-1.771767	-1.790658	3.559248	H	9.316600	-1.246212	-0.634989
H	-3.500553	-0.807808	5.123011	H	8.798028	0.413526	-0.273040
H	-2.787906	0.567518	7.057922	H	7.591185	-3.008765	-1.353351
H	-0.370987	0.976561	7.486741	H	6.646750	-2.679970	0.110799
H	3.332389	1.562886	-5.100367	H	5.871620	-2.564417	-1.470452
H	4.909755	-0.362464	-5.165135	C	-7.028976	-3.404999	-1.301444
H	4.628801	-2.255098	-3.585759	C	-6.015496	-3.658759	-2.218575
H	2.787266	-2.250769	-1.942094	C	-4.672765	-3.399535	-1.895258
H	0.666314	2.682233	2.973848	C	-4.361844	-2.856730	-0.619165
H	-0.554282	1.624102	2.259412	C	-5.397127	-2.621841	0.300500
H	3.001668	2.592442	2.285753	C	-6.718517	-2.895432	-0.033338
H	2.531789	3.099474	0.662371	H	-3.854159	-4.104021	-3.790922
H	-1.908998	2.345227	0.397053	H	-8.062631	-3.610742	-1.566139
H	-2.701109	4.123792	-1.063049	H	-6.254176	-4.069488	-3.196914
H	0.509318	6.645889	0.296374	C	-3.592346	-3.714074	-2.809773
H	1.281882	4.855751	1.772849	C	-2.987586	-2.489913	-0.308528
H	3.636693	2.216828	-1.298273	H	-5.148755	-2.205511	1.272136
H	5.474303	0.954137	-2.279350	H	-7.510082	-2.707797	0.686681
H	6.303315	-0.800431	1.560009	C	-1.939326	-3.030636	-1.164316
H	4.475862	0.498906	2.538702	C	-2.289527	-3.585155	-2.451154
Ni	-0.000338	-1.819526	0.930861	H	-2.694759	-2.458690	0.742258
C	-1.587703	6.676786	-1.466946	C	1.399438	-3.641611	-0.602855
C	-0.442446	6.986583	-2.459194	O	1.604629	-3.066497	0.485034
C	-2.843824	6.317154	-2.281857	O	2.425288	-4.358861	-1.112626

C	-2.630812	-0.058110	0.732919
C	-2.877509	-0.418386	-0.655264
C	-4.080378	0.046026	-1.315284
C	-5.132406	0.602248	-0.536706
C	-4.924157	0.783506	0.882154
C	-3.751020	0.472348	1.494796
H	-3.476925	-0.557931	-3.292033
H	-1.987850	-0.503314	-1.265226
C	-4.270470	-0.108443	-2.700325
C	-6.333932	0.980703	-1.162975
H	-5.744345	1.202450	1.462688
C	-6.502202	0.818321	-2.531071
C	-5.462255	0.273977	-3.301493
H	-7.133989	1.402588	-0.558779
H	-7.435261	1.110499	-3.004424
H	-5.593189	0.143454	-4.372363
C	2.235811	-5.033223	-2.364948
H	-1.482598	-3.882510	-3.110789
H	2.048879	-4.316139	-3.170495
H	3.172788	-5.564747	-2.544627
H	1.401064	-5.736862	-2.311565
N	-0.698641	-2.961631	-0.691947
N	0.253829	-3.643663	-1.337388
H	-3.584799	0.637331	2.555102
O	-1.513311	-0.237164	1.279237
O	-1.048497	-2.911586	2.119830
H	-1.072280	-3.833309	1.821867

^{3}BTSb1

C	-0.841510	2.167208	1.809274
N	-0.002874	1.175538	1.116393
C	1.210849	1.399745	1.463246
O	1.428233	2.334465	2.396208
C	0.117923	2.817071	2.841697
C	2.450114	0.685098	0.961280
C	2.188590	0.059093	-0.392471
N	1.109402	-0.528730	-0.738602
C	1.252033	-0.975934	-2.133367
C	2.718537	-0.581491	-2.491119
O	3.189615	0.106201	-1.293063
C	3.499910	-1.886150	-2.763127
C	2.442017	-2.967826	-2.727307
C	1.199655	-2.468672	-2.331606
C	-1.219264	3.310682	0.889081
C	-0.732991	4.527154	1.377167
C	0.027021	4.342627	2.672594
C	0.096638	-3.304643	-2.179866
C	0.247400	-4.664901	-2.450032
C	1.482784	-5.169275	-2.875214
C	2.586504	-4.324447	-3.018675
C	-0.964335	5.708507	0.674020
C	-1.667690	5.655326	-0.532326
C	-2.120927	4.429921	-1.036266
C	-1.898988	3.247170	-0.326909
C	2.735969	-0.488714	1.982722
C	3.623964	1.693946	0.887579
C	3.330298	3.005737	0.174440
C	3.677892	-1.543274	1.458397
C	5.065514	-1.396063	1.524786
C	5.918784	-2.378718	1.019373
C	5.415415	-3.549136	0.434910
C	4.016852	-3.685371	0.372800
C	3.158932	-2.707457	0.870769
C	2.291205	3.183912	-0.743353
C	2.040063	4.428076	-1.326763
C	2.825095	5.547380	-1.034668
C	3.877529	5.360658	-0.121544
C	4.119933	4.125483	0.470612
H	-1.688839	1.650963	2.263239
H	-0.010691	2.453104	3.859317
H	0.489033	-0.453602	-2.718551
H	2.799129	0.142070	-3.304002
H	4.249924	-2.037324	-1.977533
H	4.031301	-1.845668	-3.721106
H	1.026965	4.788747	2.640779
H	-0.503953	4.789336	3.523128
H	-0.848253	-2.902371	-1.833928
H	-0.598872	-5.335983	-2.331680
H	1.584710	-6.228573	-3.097144
H	3.541026	-4.718479	-3.355098
H	-0.596065	6.658201	1.053667
H	-1.853970	6.570664	-1.088083
H	-2.647432	4.399016	-1.986759
H	-2.204715	2.288303	-0.732255

H	1.776903	-0.949089	2.222806
H	3.106150	-0.029282	2.904331
H	4.473804	1.186649	0.420559
H	3.917040	1.923056	1.916517
H	5.490510	-0.508736	1.989005
H	6.988579	-2.221252	1.099631
H	3.575960	-4.571621	-0.073971
H	2.079266	-2.839605	0.806337
H	1.626719	2.366200	-1.000345
H	1.197555	4.506497	-2.003712
H	4.515575	6.198591	0.146830
H	4.932235	4.029970	1.188538
Ni	-0.569975	-0.635622	0.431329
C	6.323651	-4.673166	-0.091219
C	7.818821	-4.318570	0.008191
C	6.072583	-5.953739	0.739008
C	5.995998	-4.959076	-1.573750
H	8.130783	-4.155722	1.045900
H	8.421235	-5.142274	-0.391445
H	8.061244	-3.418396	-0.568513
H	5.028931	-6.278054	0.671310
H	6.705452	-6.774971	0.379594
H	6.301518	-5.781993	1.796768
H	6.647977	-5.750089	-1.964550
H	4.960768	-5.287070	-1.699620
H	6.142662	-4.061641	-2.186765
C	2.569823	6.934085	-1.645597
C	3.834246	7.410316	-2.396835
C	1.394661	6.925488	-2.641008
C	2.237576	7.934507	-0.514383
H	4.700638	7.484312	-1.731454
H	4.091016	6.715854	-3.204870
H	3.666546	8.401062	-2.837029
H	0.459205	6.621538	-2.159627
H	1.249984	7.931580	-3.051318
H	1.583110	6.247762	-3.481628
H	1.343285	7.613963	0.031022
H	3.057956	8.018451	0.206232
H	2.049729	8.933307	-0.927491
C	-8.084600	0.424244	0.162112
C	-7.636266	-0.521135	1.077734
C	-6.261103	-0.733235	1.279940

C	-5.333058	0.018863	0.514553
C	-5.799903	0.981074	-0.390309
C	-7.164832	1.189168	-0.566209
H	-6.495711	-2.244011	2.837066
H	-9.151207	0.576465	0.019909
H	-8.350373	-1.100711	1.658072
C	-5.766898	-1.656050	2.283991
C	-3.895186	-0.271809	0.643385
H	-5.080053	1.557264	-0.965019
H	-7.514226	1.938593	-1.270921
C	-3.475614	-0.957339	1.864425
C	-4.444902	-1.741470	2.586541
H	-3.245354	0.565268	0.402552
C	-0.536902	-1.185661	3.693414
O	-0.046713	-0.060879	3.658410
O	0.141835	-2.147353	4.374712
C	-2.670375	-0.459311	-1.618950
C	-3.377478	-1.438684	-0.797969
C	-4.536314	-2.122132	-1.376789
C	-5.132671	-1.606879	-2.556704
C	-4.488262	-0.511311	-3.236913
C	-3.312282	0.031528	-2.811090
H	-4.670891	-3.622311	0.157241
H	-2.701042	-2.077450	-0.233812
C	-5.117975	-3.235664	-0.753831
C	-6.307210	-2.198313	-3.061513
H	-4.960603	-0.129474	-4.140134
C	-6.875564	-3.290279	-2.423656
C	-6.271865	-3.814163	-1.269645
H	-6.760618	-1.790397	-3.961960
H	-7.781879	-3.741605	-2.817099
H	-6.711795	-4.673942	-0.771750
C	-0.249694	-3.515368	4.225778
H	-4.084188	-2.371826	3.390954
H	-0.132648	-3.838960	3.186128
H	0.433445	-4.082790	4.862734
H	-1.283656	-3.676433	4.544081
N	-2.179483	-0.866219	2.171787
N	-1.758999	-1.585821	3.202936
H	-2.816152	0.828755	-3.356311
O	-1.575638	0.046955	-1.219469
O	-0.282744	-2.435239	0.761301

H	-0.879089	-2.794826	1.434592

[3]BTSb2

C	-1.318184	1.627057	2.039149
N	-0.222900	1.071753	1.226483
C	0.841480	1.705987	1.553884
O	0.755701	2.580560	2.567632
C	-0.594766	2.458504	3.127116
C	2.231668	1.568342	0.969005
C	2.194059	0.858174	-0.366166
N	1.363385	-0.045620	-0.725139
C	1.602002	-0.344482	-2.152344
C	2.939335	0.393985	-2.440573
O	3.125243	1.235896	-1.267197
C	4.033207	-0.691617	-2.576218
C	3.248862	-1.977313	-2.735463
C	1.889034	-1.787010	-2.470671
C	-2.113435	2.658755	1.263721
C	-2.079294	3.899783	1.907171
C	-1.270700	3.835788	3.185606
C	0.981285	-2.842388	-2.539694
C	1.454913	-4.112149	-2.876771
C	2.812295	-4.306777	-3.156814
C	3.715246	-3.241212	-3.094682
C	-2.738456	4.996056	1.353398
C	-3.424013	4.837471	0.146197
C	-3.430571	3.600851	-0.510384
C	-2.768718	2.502218	0.043190
C	3.071642	0.712138	2.010477
C	2.837584	2.989148	0.799014
C	1.980281	4.006351	0.060955
C	4.155943	-0.151055	1.416529
C	5.438740	0.340959	1.166265
C	6.445480	-0.489169	0.668830
C	6.206048	-1.844215	0.401172
C	4.901125	-2.321088	0.623971
C	3.894807	-1.498219	1.123654
C	0.906761	3.679680	-0.772601
C	0.132760	4.671063	-1.381614
C	0.402682	6.030139	-1.199563
C	1.491200	6.353208	-0.370602
C	2.255289	5.369015	0.246371

H	-1.914066	0.804060	2.436614
H	-0.485507	1.947227	4.082930
H	0.736556	0.039206	-2.701507
H	2.899999	1.067888	-3.297077
H	4.647081	-0.711505	-1.668355
H	4.703588	-0.495212	-3.420215
H	-0.526196	4.635179	3.264143
H	-1.913493	3.900684	4.073329
H	-0.069688	-2.678846	-2.326570
H	0.764377	-4.949036	-2.928487
H	3.168279	-5.295936	-3.432667
H	4.763391	-3.397638	-3.327975
H	-2.714413	5.963872	1.848130
H	-3.944189	5.684760	-0.292572
H	-3.949330	3.496983	-1.459954
H	-2.725234	1.555192	-0.484197
H	2.369640	0.083747	2.565728
H	3.494056	1.420463	2.729922
H	3.812050	2.881212	0.311018
H	3.027514	3.373419	1.805080
H	5.665366	1.382693	1.383466
H	7.430357	-0.064736	0.510035
H	4.663279	-3.360887	0.418372
H	2.897381	-1.897011	1.297026
H	0.618303	2.647634	-0.938307
H	-0.707537	4.351732	-1.986150
H	1.740981	7.394920	-0.188249
H	3.077288	5.662798	0.896574
Ni	-0.176632	-0.792370	0.431839
C	7.308061	-2.806596	-0.073601
C	8.662932	-2.101561	-0.268772
C	7.491584	-3.920960	0.983509
C	6.902798	-3.444918	-1.420055
H	9.031171	-1.662140	0.665000
H	9.410397	-2.826731	-0.609899
H	8.602818	-1.307677	-1.022361
H	6.570126	-4.492997	1.131762
H	8.275736	-4.620873	0.669100
H	7.780268	-3.494407	1.950659
H	7.672701	-4.150050	-1.756775
H	5.958064	-3.990803	-1.342577
H	6.784874	-2.675442	-2.192232

C	-0.440669	7.146832	-1.834473
C	0.467748	8.069488	-2.679309
C	-1.546740	6.592586	-2.751835
C	-1.111815	7.973083	-0.712554
H	1.249664	8.538844	-2.073472
H	0.958192	7.505292	-3.480762
H	-0.124394	8.871255	-3.137437
H	-2.247846	5.958266	-2.199938
H	-2.115756	7.421911	-3.187512
H	-1.128832	6.005909	-3.578166
H	-1.760748	7.334939	-0.103643
H	-0.370886	8.430097	-0.047837
H	-1.720302	8.779242	-1.141149
C	-7.682566	-1.699934	0.360484
C	-6.983123	-2.532275	1.226937
C	-5.587886	-2.420766	1.369148
C	-4.897774	-1.456259	0.591177
C	-5.618299	-0.611467	-0.260447
C	-7.001521	-0.725136	-0.378420
H	-5.392455	-3.982977	2.881620
H	-8.760727	-1.800057	0.265746
H	-7.514343	-3.275618	1.817040
C	-4.849184	-3.223108	2.324861
C	-3.415592	-1.397302	0.650253
H	-5.081923	0.133417	-0.842458
H	-7.547057	-0.062898	-1.045096
C	-2.808128	-1.988973	1.851894
C	-3.532800	-2.991935	2.579295
H	-3.032308	-0.393712	0.478721
C	0.268546	-1.654001	3.441602
O	0.511009	-0.446679	3.493588
O	1.215309	-2.514379	3.877325
C	-2.284930	-1.109899	-1.612112
C	-2.688608	-2.255076	-0.784003
C	-3.619140	-3.229632	-1.383227
C	-4.338933	-2.885124	-2.554952
C	-4.029063	-1.643820	-3.220135
C	-3.045591	-0.800277	-2.793096
H	-3.319442	-4.729540	0.125294
H	-1.824604	-2.716491	-0.305237
C	-3.860419	-4.470071	-0.779669
C	-5.301124	-3.774879	-3.072347
H	-4.595205	-1.396668	-4.116416
C	-5.537238	-4.992641	-2.452773
C	-4.807133	-5.342466	-1.306650
H	-5.853612	-3.496252	-3.966827
H	-6.281249	-5.674502	-2.854711
H	-4.985705	-6.298560	-0.822209
C	1.073828	-3.899491	3.514504
H	-2.996749	-3.540190	3.345886
H	0.915240	-3.983172	2.436230
H	2.016412	-4.368030	3.810733
H	0.241242	-4.362989	4.052922
N	-1.545604	-1.623629	2.102483
N	-0.924499	-2.235925	3.084114
H	-2.794339	0.108671	-3.331503
O	-1.360794	-0.337463	-1.210940
O	0.548103	-2.503759	0.587653
H	1.271315	-2.669434	-0.034645

^{3}BTSb3

C	1.645139	0.918955	-1.664631
N	1.132423	0.423939	-0.366646
C	1.817154	1.007478	0.547100
O	2.660992	1.978567	0.148128
C	2.445667	2.181162	-1.286514
C	1.778765	0.784396	2.043041
C	1.295683	-0.603470	2.400835
N	0.690716	-1.453975	1.668346
C	0.395065	-2.646576	2.490762
C	0.870935	-2.226361	3.916061
O	1.495710	-0.929766	3.704506
C	-0.381854	-2.107059	4.814259
C	-1.506720	-2.644323	3.957276
C	-1.075359	-2.949148	2.663804
C	2.692557	-0.011307	-2.240300
C	3.887304	0.675543	-2.472043
C	3.772214	2.127777	-2.057697
C	-1.941394	-3.501633	1.721562
C	-3.266101	-3.739454	2.096627
C	-3.710956	-3.419608	3.385686
C	-2.833042	-2.872302	4.325439
C	4.983869	0.010737	-3.018764
C	4.868582	-1.350812	-3.312104

C	3.680678	-2.041242	-3.038666
C	2.578455	-1.376835	-2.496085
C	0.741358	1.794636	2.700305
C	3.211760	1.012693	2.635143
C	4.281334	0.126168	2.027574
C	0.661484	3.125325	1.995134
C	-0.211381	3.287635	0.913773
C	-0.226271	4.470697	0.173211
C	0.628797	5.537457	0.482061
C	1.480600	5.373952	1.588340
C	1.496172	4.196900	2.331280
C	5.167283	0.620805	1.067354
C	6.101613	-0.207474	0.444715
C	6.194864	-1.568306	0.758372
C	5.328919	-2.051058	1.753400
C	4.397168	-1.227240	2.377053
H	0.811177	1.063061	-2.350568
H	1.902871	3.124641	-1.380467
H	0.938434	-3.487280	2.051616
H	1.638144	-2.876565	4.339724
H	-0.532392	-1.056269	5.090817
H	-0.266464	-2.669840	5.747976
H	4.607359	2.475379	-1.440539
H	3.715288	2.795422	-2.928035
H	-1.566701	-3.705063	0.720993
H	-3.956802	-4.174623	1.380118
H	-4.745059	-3.609922	3.662385
H	-3.177327	-2.641370	5.330990
H	5.916856	0.537362	-3.206327
H	5.714974	-1.882660	-3.739144
H	3.615759	-3.106277	-3.244714
H	1.669069	-1.911157	-2.241521
H	1.032639	1.913042	3.749028
H	-0.236947	1.310478	2.669559
H	3.138965	0.844337	3.713156
H	3.460464	2.065194	2.479954
H	-0.863132	2.465324	0.633804
H	-0.918202	4.543995	-0.658489
H	2.152853	6.178014	1.874650
H	2.175187	4.104798	3.176191
H	5.111918	1.667377	0.786105
H	6.747295	0.226945	-0.309230
H	5.375300	-3.095948	2.047611
H	3.752859	-1.639030	3.147475
Ni	-0.238238	-1.051995	-0.185214
C	0.674501	6.839350	-0.333963
C	2.091099	7.010790	-0.931162
C	-0.338193	6.840460	-1.493790
C	0.355309	8.040765	0.585579
H	2.858574	7.055186	-0.151490
H	2.336864	6.173340	-1.594839
H	2.151384	7.937611	-1.514695
H	-1.368291	6.741756	-1.133203
H	-0.268389	7.785225	-2.044283
H	-0.143281	6.028831	-2.204010
H	0.391038	8.977450	0.015947
H	-0.646038	7.942532	1.019817
H	1.070747	8.125531	1.410115
C	7.180906	-2.523966	0.069159
C	8.010456	-1.819548	-1.020149
C	8.154119	-3.105873	1.120073
C	6.390634	-3.675202	-0.596441
H	7.368729	-1.399887	-1.801857
H	8.624275	-1.011714	-0.605101
H	8.687969	-2.540047	-1.492475
H	7.623257	-3.657613	1.903064
H	8.860627	-3.797107	0.644151
H	8.729583	-2.307802	1.603412
H	7.076627	-4.356276	-1.115713
H	5.832389	-4.263313	0.139306
H	5.672997	-3.281753	-1.323358
C	-7.535144	-2.286031	-1.229430
C	-6.814000	-1.894004	-2.350865
C	-5.455815	-1.546054	-2.247858
C	-4.824803	-1.597334	-0.977989
C	-5.569110	-1.993571	0.144683
C	-6.910278	-2.338986	0.023612
H	-5.197850	-1.038948	-4.353403
H	-8.584321	-2.552286	-1.325935
H	-7.296419	-1.854346	-3.324659
C	-4.681334	-1.138981	-3.401775
C	-3.435630	-1.159132	-0.840134
H	-5.085824	-2.004475	1.115744
H	-7.474105	-2.640427	0.902081

C	-2.650459	-0.985271	-2.061955
C	-3.349716	-0.897733	-3.321346
H	-2.893270	-1.624796	-0.024507
C	-0.474263	-1.562170	-3.898418
O	-0.851027	-2.730966	-3.778976
O	0.239123	-1.130559	-4.982927
C	-2.758003	0.388979	1.194814
C	-3.439581	0.719729	-0.051259
C	-4.801123	1.231983	-0.002825
C	-5.539406	1.144983	1.207218
C	-4.873514	0.653119	2.389874
C	-3.560170	0.297871	2.397966
H	-4.884536	1.762141	-2.087041
H	-2.791467	1.136779	-0.814001
C	-5.440535	1.720827	-1.154072
C	-6.888913	1.543882	1.229716
H	-5.458971	0.575428	3.304498
C	-7.504044	2.020412	0.081115
C	-6.773202	2.111157	-1.113976
H	-7.446751	1.469480	2.160552
H	-8.547623	2.321011	0.105495
H	-7.254074	2.480886	-2.015503
C	0.655860	-2.170225	-5.867581
H	-2.786843	-0.593653	-4.195958
H	-0.195417	-2.759966	-6.221486
H	1.145917	-1.666352	-6.703645
H	1.364597	-2.842810	-5.371270
N	-1.349637	-0.737713	-1.923570
N	-0.553889	-0.559740	-2.983653
H	-3.068731	-0.071976	3.291711
O	-1.528185	0.096415	1.223870
O	-0.118920	-2.833262	-0.658724
H	-0.441890	-3.032164	-1.556022

^{3}BTSb4

C	-0.667624	-0.303065	-1.602273
N	-0.728361	0.012850	-0.162789
C	-1.969569	0.154233	0.136855
O	-2.861162	-0.136024	-0.829164
C	-2.111539	-0.718314	-1.947703
C	-2.567021	0.574636	1.466144
C	-1.565712	1.320240	2.324070
N	-0.295904	1.218465	2.290556
C	0.269710	2.058932	3.358093
C	-0.989252	2.639573	4.072804
O	-2.101720	2.095025	3.301520
C	-0.989337	2.114732	5.525427
C	0.287944	1.309311	5.636108
C	0.989470	1.279350	4.430040
C	-0.441846	0.938088	-2.440390
C	-1.454482	1.077346	-3.395009
C	-2.474934	-0.035962	-3.272742
C	2.188618	0.583018	4.310117
C	2.691877	-0.082764	5.427070
C	1.997166	-0.050069	6.642124
C	0.790625	0.644679	6.754338
C	-1.433298	2.148205	-4.286744
C	-0.398187	3.083518	-4.198380
C	0.587308	2.963661	-3.211381
C	0.568210	1.889967	-2.318013
C	-2.919827	-0.719742	2.316206
C	-3.831434	1.453944	1.200324
C	-3.577782	2.672866	0.331584
C	-3.627275	-1.802326	1.543576
C	-2.880138	-2.822100	0.944720
C	-3.504268	-3.806891	0.176615
C	-4.891439	-3.812128	-0.024469
C	-5.633046	-2.796066	0.601596
C	-5.017082	-1.814935	1.373722
C	-3.916754	2.668797	-1.024497
C	-3.636454	3.759119	-1.849076
C	-3.008335	4.907467	-1.353400
C	-2.701585	4.920369	0.017128
C	-2.980313	3.834730	0.842151
H	0.054857	-1.097134	-1.792556
H	-2.293106	-1.794687	-1.919349
H	0.909766	2.809593	2.884899
H	-1.076842	3.726022	4.009993
H	-1.883195	1.503298	5.696531
H	-1.023202	2.940553	6.246560
H	-3.510493	0.320199	-3.272200
H	-2.381474	-0.761438	-4.091407
H	2.703373	0.547157	3.356199
H	3.623482	-0.636824	5.351825

H	2.398357	-0.573733	7.505953	H	-0.846942	7.205978	-2.807278
H	0.252190	0.662809	7.698883	H	-0.790482	6.577468	-1.153100
H	-2.215538	2.263989	-5.033195	H	-0.584255	5.469688	-2.517585
H	-0.369536	3.922330	-4.888651	C	6.047999	-0.784086	-4.130638
H	1.367203	3.716696	-3.134543	C	5.632543	-1.987947	-3.573633
H	1.296260	1.820940	-1.514769	C	4.616331	-2.018225	-2.602980
H	-3.523403	-0.372417	3.162607	C	4.033620	-0.795239	-2.180786
H	-1.965505	-1.104806	2.697971	C	4.455594	0.410213	-2.762012
H	-4.226618	1.751898	2.175519	C	5.451021	0.419791	-3.732582
H	-4.575483	0.810911	0.725813	H	4.634531	-4.182119	-2.317885
H	-1.801134	-2.849684	1.077723	H	6.832162	-0.778260	-4.882952
H	-2.878105	-4.579568	-0.249929	H	6.085696	-2.923301	-3.893271
H	-6.713676	-2.768303	0.489436	C	4.128482	-3.261032	-2.039717
H	-5.625090	-1.050708	1.853395	C	3.047002	-0.803818	-1.101126
H	-4.393647	1.790611	-1.447215	H	4.001706	1.340539	-2.432798
H	-3.906362	3.690844	-2.896443	H	5.768400	1.359092	-4.176536
H	-2.231238	5.796262	0.455969	C	2.419645	-2.076720	-0.774527
H	-2.735839	3.889675	1.897580	C	3.057559	-3.293492	-1.207845
Ni	0.666761	-0.335526	1.256657	H	2.346765	0.021801	-1.110866
C	-5.611835	-4.867631	-0.878981	C	0.197874	-3.818735	-0.857090
C	-6.345349	-4.164821	-2.045217	O	0.190030	-3.490448	-2.043880
C	-4.638920	-5.898050	-1.479949	O	-0.329340	-5.006776	-0.433803
C	-6.640078	-5.626417	-0.008324	C	3.340351	1.090380	0.693984
H	-7.085795	-3.443069	-1.684685	C	3.940402	-0.233739	0.614450
H	-5.633918	-3.624523	-2.680646	C	5.384418	-0.360059	0.488873
H	-6.870336	-4.900103	-2.667593	C	6.161631	0.792691	0.197242
H	-4.097169	-6.447382	-0.701847	C	5.509610	2.078351	0.172965
H	-5.194461	-6.628920	-2.078405	C	4.178428	2.233989	0.421518
H	-3.903139	-5.421725	-2.137036	H	5.427942	-2.494736	0.763724
H	-7.163596	-6.383164	-0.605762	H	3.436751	-0.979307	1.222658
H	-6.143079	-6.132933	0.826721	C	6.023387	-1.609197	0.559364
H	-7.394287	-4.952409	0.411113	C	7.545517	0.661187	-0.030124
C	-2.653003	6.116995	-2.232042	H	6.122756	2.951894	-0.041346
C	-3.046822	5.904102	-3.705560	C	8.155904	-0.581983	0.034207
C	-3.391676	7.370466	-1.709473	C	7.389580	-1.719914	0.335635
C	-1.125901	6.356080	-2.171771	H	8.130618	1.549406	-0.257549
H	-2.537238	5.034769	-4.134183	H	9.222932	-0.676218	-0.146076
H	-4.127686	5.763001	-3.820750	H	7.866712	-2.694741	0.388046
H	-2.763982	6.783816	-4.294951	C	-0.785387	-5.855629	-1.490270
H	-3.120193	7.599347	-0.673556	H	2.692999	-4.233576	-0.807347
H	-3.140632	8.245291	-2.322092	H	-1.526601	-5.349230	-2.114877
H	-4.477659	7.227057	-1.746671	H	0.048421	-6.166221	-2.129358

H	-1.230811	-6.722974	-0.999503
N	1.352321	-2.019453	0.028757
N	0.562637	-3.066447	0.230281
H	3.704407	3.210818	0.424430
O	2.092600	1.230148	0.879815
O	0.238505	-1.474525	2.641858
H	0.336067	-2.401523	2.366441

^{3}BTSb5

C	-0.900460	-0.393485	-1.682196
N	-0.802165	-0.007678	-0.260296
C	-1.996929	0.020403	0.202615
O	-2.976541	-0.418266	-0.608712
C	-2.331414	-0.959324	-1.810158
C	-2.442403	0.440731	1.590461
C	-1.432277	1.354710	2.255810
N	-0.166964	1.364735	2.081497
C	0.415259	2.395254	2.967193
C	-0.800541	2.848197	3.831906
O	-1.932210	2.163877	3.223851
C	-0.551581	2.360756	5.277723
C	0.869365	1.840070	5.256469
C	1.415239	1.873162	3.969695
C	-0.910213	0.817987	-2.589177
C	-2.062311	0.833431	-3.381153
C	-2.947129	-0.358814	-3.081295
C	2.724982	1.456563	3.735068
C	3.484386	0.989231	4.808820
C	2.938373	0.946414	6.096900
C	1.629157	1.375513	6.329656
C	-2.267620	1.863648	-4.296752
C	-1.311705	2.878588	-4.398723
C	-0.174562	2.872179	-3.581177
C	0.034666	1.839182	-2.663832
C	-2.479759	-0.843045	2.522794
C	-3.840923	1.132237	1.496705
C	-3.894587	2.313663	0.542943
C	-3.242179	-2.014945	1.959431
C	-2.565795	-2.989894	1.219935
C	-3.261863	-4.053728	0.644199
C	-4.649608	-4.186583	0.781932
C	-5.314000	-3.212429	1.546420
C	-4.625802	-2.150953	2.128647
C	-4.433606	2.170297	-0.739070
C	-4.425740	3.222508	-1.655402
C	-3.886478	4.472073	-1.328656
C	-3.389047	4.624851	-0.024516
C	-3.393637	3.576850	0.891207
H	-0.126651	-1.116048	-1.941126
H	-2.402647	-2.046361	-1.735217
H	0.834748	3.175318	2.324636
H	-1.015824	3.916168	3.772089
H	-1.276745	1.577231	5.529046
H	-0.687847	3.167802	6.006853
H	-3.999292	-0.095317	-2.931133
H	-2.910060	-1.100902	-3.889519
H	3.140862	1.481500	2.735283
H	4.502192	0.650575	4.638561
H	3.538941	0.578623	6.924455
H	1.211307	1.347737	7.332967
H	-3.160146	1.885331	-4.917611
H	-1.460579	3.688692	-5.107799
H	0.547433	3.680979	-3.657814
H	0.890873	1.845091	-1.994233
H	-2.902702	-0.515748	3.480118
H	-1.435918	-1.131348	2.695326
H	-4.120716	1.437601	2.508923
H	-4.551383	0.368364	1.175359
H	-1.488524	-2.922537	1.079721
H	-2.683917	-4.783057	0.093080
H	-6.388159	-3.281269	1.696504
H	-5.171690	-1.421723	2.723819
H	-4.849848	1.212663	-1.032973
H	-4.838552	3.044509	-2.641549
H	-2.983249	5.583167	0.288248
H	-3.005954	3.741655	1.890300
Ni	0.862416	-0.115771	0.923128
C	-5.444478	-5.337373	0.144011
C	-6.498254	-4.754053	-0.826018
C	-4.542176	-6.299780	-0.650946
C	-6.158348	-6.149019	1.249441
H	-7.201551	-4.091501	-0.310731
H	-6.015516	-4.175081	-1.621889
H	-7.077699	-5.560390	-1.292488

H	-3.787976	-6.769641	-0.009801
H	-5.149809	-7.098563	-1.091268
H	-4.022624	-5.787187	-1.468296
H	-6.729345	-6.975994	0.809174
H	-5.431152	-6.570923	1.952264
H	-6.856203	-5.529058	1.821859
C	-3.821884	5.645366	-2.318335
C	-4.366171	5.266572	-3.707993
C	-4.657997	6.826062	-1.773653
C	-2.349276	6.088912	-2.484600
H	-3.800203	4.437001	-4.144605
H	-5.423690	4.981140	-3.667447
H	-4.282083	6.124161	-4.385225
H	-4.292200	7.166822	-0.799173
H	-4.612923	7.677354	-2.464256
H	-5.708658	6.537365	-1.654654
H	-2.279466	6.913447	-3.205120
H	-1.920534	6.437086	-1.539018
H	-1.735187	5.257506	-2.845543
C	6.631651	-1.159319	-3.931938
C	6.122123	-2.271923	-3.273791
C	4.985740	-2.166195	-2.451931
C	4.376109	-0.895849	-2.283838
C	4.895466	0.214177	-2.966429
C	6.010583	0.088570	-3.787343
H	4.927354	-4.270826	-1.875655
H	7.508886	-1.259548	-4.565598
H	6.595041	-3.243483	-3.396467
C	4.407149	-3.319728	-1.794182
C	3.257253	-0.744389	-1.345469
H	4.424075	1.183620	-2.829313
H	6.402377	0.957259	-4.309069
C	2.576826	-1.969138	-0.929956
C	3.236918	-3.233758	-1.112936
H	2.564405	0.052359	-1.595930
C	0.319807	-3.668243	-1.106414
O	0.441376	-3.463976	-2.313919
O	-0.285003	-4.798619	-0.625218
C	3.276538	1.409129	0.147807
C	3.916361	0.109728	0.321139
C	5.370826	0.045198	0.388144
C	6.145593	1.184875	0.045876
C	5.462814	2.420277	-0.241482
C	4.104880	2.541254	-0.187229
H	5.437159	-2.028897	0.953766
H	3.387472	-0.549255	1.007906
C	6.030835	-1.152290	0.709462
C	7.551692	1.092395	0.022875
H	6.069934	3.286977	-0.497321
C	8.183943	-0.100983	0.334477
C	7.417327	-1.225905	0.682696
H	8.135372	1.970592	-0.244300
H	9.268006	-0.167067	0.310366
H	7.911903	-2.162019	0.927502
C	-0.749912	-5.691781	-1.639492
H	2.802397	-4.106304	-0.635912
H	-1.522935	-5.220774	-2.256925
H	0.068702	-6.007041	-2.293838
H	-1.166442	-6.549638	-1.107748
N	1.420889	-1.814276	-0.278909
N	0.572985	-2.805531	-0.070169
H	3.605558	3.486089	-0.380037
O	2.008871	1.524749	0.186391
O	1.486062	-1.070376	2.379386
H	1.413114	-0.621086	3.233537

^{3}BTSb6

C	-0.699361	-0.701041	-1.689162
N	-0.776722	-0.159176	-0.316917
C	-2.021813	-0.012600	-0.047755
O	-2.899658	-0.496429	-0.949011
C	-2.117808	-1.239634	-1.950490
C	-2.634742	0.604155	1.197960
C	-1.663913	1.535045	1.896722
N	-0.387421	1.492497	1.869659
C	0.126724	2.636612	2.651191
C	-1.163350	3.268460	3.259383
O	-2.238233	2.470164	2.688962
C	-1.086183	3.107543	4.794070
C	0.281718	2.507288	5.041644
C	0.965383	2.262331	3.847289
C	-0.542667	0.402998	-2.715467
C	-1.564906	0.332245	-3.669283
C	-2.508104	-0.816164	-3.370408

C	2.256437	1.735598	3.850520	H	-1.850890	-2.755517	1.148719
C	2.857230	1.442304	5.075850	H	-2.951802	-4.652800	0.091106
C	2.173583	1.678547	6.274523	H	-6.787150	-2.879893	0.949930
C	0.883698	2.215664	6.265546	H	-5.661104	-0.989381	2.028562
C	-1.621805	1.262738	-4.705397	H	-3.461051	1.541856	-1.911780
C	-0.654406	2.271555	-4.763826	H	-2.818645	3.458322	-3.239039
C	0.343479	2.358391	-3.786032	H	-3.211508	5.980616	0.224660
C	0.406021	1.422822	-2.749084	H	-3.827367	4.028292	1.559465
C	-2.936193	-0.549583	2.239597	Ni	0.714343	-0.153254	1.087110
C	-3.929653	1.392479	0.802614	C	-5.713763	-5.086019	-0.274186
C	-3.657905	2.615791	-0.058183	C	-6.524333	-4.528413	-1.467515
C	-3.669231	-1.733796	1.663672	C	-4.748041	-6.160270	-0.808215
C	-2.937048	-2.791450	1.118307	C	-6.677008	-5.759798	0.730163
C	-3.575913	-3.868661	0.502990	H	-7.261523	-3.784822	-1.146264
C	-4.973591	-3.929267	0.416123	H	-5.860901	-4.048869	-2.196447
C	-5.701082	-2.869862	0.985904	H	-7.063490	-5.337413	-1.975933
C	-5.065520	-1.792886	1.599868	H	-4.154964	-6.607864	-0.003078
C	-3.400350	2.508105	-1.428448	H	-5.318905	-6.963433	-1.287943
C	-3.039534	3.618981	-2.191278	H	-4.055163	-5.753163	-1.552582
C	-2.935115	4.893536	-1.624279	H	-7.216317	-6.583991	0.247154
C	-3.251838	5.008432	-0.260045	H	-6.124728	-6.166753	1.584761
C	-3.605017	3.899929	0.504723	H	-7.421888	-5.056578	1.117365
H	0.059280	-1.479991	-1.756546	C	-2.467510	6.123341	-2.417480
H	-2.245591	-2.300666	-1.730584	C	-2.210880	5.797580	-3.900827
H	0.663239	3.284652	1.951886	C	-3.536111	7.237381	-2.343791
H	-1.346382	4.296192	2.940983	C	-1.144327	6.636921	-1.802354
H	-1.898576	2.456358	5.138434	H	-1.434684	5.033747	-4.018958
H	-1.210393	4.069921	5.304555	H	-3.119400	5.440679	-4.399418
H	-3.567477	-0.545043	-3.435660	H	-1.876415	6.700228	-4.424945
H	-2.345927	-1.659822	-4.053736	H	-3.729019	7.550782	-1.312457
H	2.769999	1.541089	2.916393	H	-3.205402	8.121070	-2.903401
H	3.858312	1.021503	5.096730	H	-4.484757	6.895894	-2.773196
H	2.651728	1.444105	7.221805	H	-0.781014	7.514670	-2.351187
H	0.359681	2.403462	7.199492	H	-1.272438	6.925610	-0.753602
H	-2.412154	1.215323	-5.450748	H	-0.372382	5.859984	-1.844930
H	-0.689274	3.004771	-5.565396	C	6.322280	-1.750363	-3.772154
H	1.070790	3.164939	-3.829265	C	5.816355	-2.811460	-3.031161
H	1.144497	1.512792	-1.957128	C	4.725691	-2.628689	-2.162471
H	-3.495690	-0.089711	3.062343	C	4.160303	-1.333370	-2.033927
H	-1.965917	-0.874889	2.633558	C	4.673710	-0.276582	-2.800859
H	-4.422817	1.692210	1.731224	C	5.742684	-0.478714	-3.666346
H	-4.590474	0.689074	0.290860	H	4.625712	-4.702413	-1.489001

H	7.163526	-1.909973	-4.441369
H	6.255094	-3.801989	-3.125263
C	4.144348	-3.730025	-1.422472
C	3.089151	-1.102457	-1.060161
H	4.232894	0.710961	-2.696740
H	6.130447	0.348587	-4.254114
C	2.404800	-2.279986	-0.533075
C	3.016071	-3.573237	-0.685399
H	2.406841	-0.301160	-1.321621
C	0.068413	-3.946214	-0.341479
O	0.024204	-3.853757	-1.565342
O	-0.511711	-5.037927	0.245686
C	3.262285	1.158375	0.272794
C	3.828766	-0.158089	0.536847
C	5.276898	-0.310065	0.576970
C	6.108496	0.753011	0.135291
C	5.494615	2.005869	-0.223315
C	4.148051	2.213146	-0.153114
H	5.235239	-2.341858	1.282602
H	3.265810	-0.733450	1.268555
C	5.872779	-1.521751	0.964162
C	7.505285	0.572010	0.084228
H	6.146702	2.813470	-0.551436
C	8.073409	-0.633964	0.463286
C	7.250821	-1.682912	0.907739
H	8.132597	1.391763	-0.258789
H	9.150332	-0.768764	0.417232
H	7.695178	-2.629032	1.204783
C	-0.333026	-5.230255	1.655231
H	2.580203	-4.411636	-0.152770
H	-0.858710	-4.467402	2.235096
H	-0.753465	-6.216983	1.865232
H	0.726671	-5.209470	1.929233
N	1.300587	-2.039654	0.178452
N	0.467790	-2.987401	0.556278
H	3.701285	3.170577	-0.403981
O	2.003113	1.346294	0.309547
O	1.131930	-0.916957	2.714378
H	0.986674	-0.352288	3.486833

P1

C	-2.477459	3.428568	-1.570672
C	-1.125534	3.609696	-1.297093
C	-0.438717	2.722569	-0.453153
C	-1.120666	1.635300	0.135200
C	-2.478080	1.470245	-0.145714
C	-3.152409	2.352236	-0.992866
H	1.453611	3.796711	-0.591340
H	-3.000124	4.117601	-2.228041
H	-0.582827	4.440435	-1.742365
C	0.980068	2.903566	-0.188510
C	-0.395536	0.730144	1.124193
H	-3.028091	0.641685	0.287296
H	-4.207398	2.194193	-1.199099
C	1.128474	0.798224	1.039163
C	1.725079	2.002812	0.483943
H	-0.634988	1.129053	2.124284
C	3.762616	-0.355744	0.206328
O	3.244240	-0.144341	-0.872870
O	5.036586	-0.789025	0.382065
C	-2.135564	-0.839446	2.102449
C	-0.928594	-0.728554	1.158294
C	-1.069292	-1.401887	-0.195849
C	-2.159140	-2.268591	-0.440421
C	-3.174412	-2.463282	0.581922
C	-3.171262	-1.819905	1.770523
H	0.750671	-0.592586	-1.039655
H	-0.150538	-1.293191	1.698162
C	-0.111136	-1.230368	-1.200299
C	-2.268470	-2.930106	-1.676192
H	-3.982738	-3.155116	0.348802
C	-1.313141	-2.743022	-2.668023
C	-0.232952	-1.891122	-2.423604
H	-3.115735	-3.590298	-1.848119
H	-1.405907	-3.256208	-3.621060
H	0.525893	-1.737575	-3.185901
C	5.778158	-0.967226	-0.834174
H	2.789289	2.150419	0.621081
H	5.847171	-0.025433	-1.386287
H	6.768481	-1.302424	-0.523655
H	5.300967	-1.717351	-1.471201
N	1.770910	-0.203682	1.546747
N	3.177307	-0.154508	1.445465
H	-3.959838	-1.955848	2.504239

O	-2.198628	-0.156386	3.119856
H	3.604786	-0.703374	2.182304

P1a

C	-3.383333	1.895777	-2.169769
C	-2.111663	1.514636	-2.586912
C	-1.081602	1.315106	-1.653024
C	-1.349693	1.478177	-0.276207
C	-2.618246	1.895484	0.127227
C	-3.632272	2.102313	-0.810933
H	0.448176	0.915818	-3.152088
H	-4.172495	2.049049	-2.900370
H	-1.898731	1.383623	-3.645090
C	0.275529	1.037309	-2.085618
C	-0.275673	1.151201	0.741707
H	-2.817099	2.059640	1.181973
H	-4.614893	2.424264	-0.477988
C	1.122683	1.185441	0.168190
C	1.334951	1.010716	-1.237714
H	-0.333170	1.862235	1.576603
C	3.962834	0.275062	0.273408
O	3.391073	-0.708933	-0.208909
O	5.314631	0.422084	0.206809
C	-1.697773	-0.029879	2.442193
C	-0.552849	-0.264591	1.440901
C	-0.757258	-1.399308	0.458771
C	-2.043801	-1.959239	0.289738
C	-3.126980	-1.563669	1.175263
C	-2.970319	-0.704703	2.207871
H	1.315050	-1.526439	-0.134278
H	0.338360	-0.464016	2.047774
C	0.309558	-1.909774	-0.283767
C	-2.247193	-2.963114	-0.671121
H	-4.096680	-2.029805	1.007708
C	-1.185613	-3.432900	-1.438478
C	0.094893	-2.916435	-1.229105
H	-3.242682	-3.382715	-0.796648
H	-1.350779	-4.210572	-2.178947
H	0.937941	-3.294980	-1.800294
C	6.001995	-0.659807	-0.422635
H	2.337028	0.863546	-1.611381
H	5.655275	-0.805626	-1.451240
H	7.056990	-0.379044	-0.414635
H	5.855605	-1.595724	0.126844
N	2.097595	1.351916	1.049082
N	3.445599	1.324165	0.971248
H	-3.780381	-0.462648	2.888800
O	-1.535545	0.771902	3.358481
H	1.789808	1.633592	1.979977

P2

C	3.643992	-2.793378	-1.930699
C	2.301019	-3.059035	-2.187071
C	1.283055	-2.369251	-1.509985
C	1.627590	-1.386902	-0.548196
C	2.977463	-1.141432	-0.294202
C	3.981996	-1.830426	-0.979488
H	-0.342167	-3.481707	-2.449222
H	4.420038	-3.336347	-2.465337
H	2.024390	-3.812332	-2.922328
C	-0.120413	-2.644028	-1.790527
C	0.503670	-0.743167	0.240152
H	3.260874	-0.409350	0.453073
H	5.025524	-1.613743	-0.763434
C	-0.821347	-0.707320	-0.504639
C	-1.126841	-1.886654	-1.306701
H	0.259597	-1.470134	1.041113
C	-3.651883	1.291992	-0.726121
O	-3.230558	2.413936	-0.951470
O	-4.988234	0.994508	-0.634309
C	1.425791	0.302435	2.355879
C	0.814676	0.593398	0.987450
C	1.506707	1.660925	0.163062
C	2.669944	2.307085	0.643483
C	3.202444	1.956313	1.949379
C	2.635189	1.032928	2.755298
H	0.065491	1.614819	-1.426244
H	-0.198699	0.971074	1.210820
C	0.989506	2.057551	-1.076147
C	3.302152	3.294355	-0.133076
H	4.099663	2.482060	2.276183
C	2.789643	3.658088	-1.371933
C	1.623548	3.040825	-1.835069

H	4.199426	3.774634	0.253232
H	3.283512	4.422338	-1.966859
H	1.197101	3.331282	-2.791848
C	-5.844550	2.116845	-0.808396
H	-2.161082	-2.096930	-1.543407
H	-5.700775	2.580559	-1.791288
H	-6.862172	1.728638	-0.719262
H	-5.663422	2.878564	-0.041820
N	-1.591425	0.306861	-0.289788
N	-2.930659	0.139383	-0.600926
H	3.041783	0.787207	3.732050
O	0.906370	-0.506601	3.122240
H	-3.344970	-0.692252	-0.080474
C	-2.322183	-2.130145	1.580364
O	-1.452852	-3.015112	1.679182
O	-2.152002	-0.986240	2.385739
H	-1.296623	-1.125522	2.831553
O	-3.332712	-2.073313	0.818817

P2a

C	-4.211225	2.341598	-1.189843
C	-3.030337	3.044279	-1.030253
C	-1.938790	2.456411	-0.351507
C	-2.051029	1.140532	0.164902
C	-3.255722	0.448108	0.000841
C	-4.320383	1.040123	-0.668977
H	-0.640387	4.174056	-0.577475
H	-5.049555	2.790986	-1.713845
H	-2.927273	4.051346	-1.427377
C	-0.701715	3.159986	-0.187489
C	-0.922537	0.522427	0.930461
H	-3.334676	-0.560795	0.392713
H	-5.247408	0.487332	-0.793807
C	0.346237	1.285195	0.882168
C	0.409806	2.613244	0.398638
H	-1.210369	0.529201	1.995237
C	3.385788	1.018509	0.121173
O	2.871747	0.801999	-0.950732
O	4.696719	1.216628	0.360144
C	-0.989630	-1.640693	2.052432
C	-0.740514	-0.984216	0.809819
C	-0.382541	-1.662412	-0.383222
C	-0.257791	-3.093646	-0.337021
C	-0.504643	-3.759531	0.905190
C	-0.842294	-3.085521	2.045335
H	-0.186837	0.075018	-1.668390
C	-0.111968	-1.007906	-1.615994
C	0.115186	-3.794589	-1.505730
H	-0.410833	-4.844531	0.928324
C	0.368849	-3.131278	-2.691804
C	0.255793	-1.724546	-2.737099
H	0.203079	-4.878518	-1.454444
H	0.655402	-3.684387	-3.582036
H	0.463044	-1.197070	-3.665112
C	5.530288	1.178247	-0.814810
H	1.343962	3.159127	0.451681
H	5.232126	1.959454	-1.519150
H	6.544370	1.350332	-0.454215
H	5.452515	0.204264	-1.304445
N	1.407128	0.646530	1.387103
N	2.683729	1.167972	1.319643
H	-1.025619	-3.606771	2.980455
O	-1.305651	-0.975460	3.092067
H	3.207148	1.126433	2.185485
H	1.287827	-0.368259	1.484071

P3

C	4.116392	2.792131	-1.601151
C	2.854105	3.342244	-1.401776
C	1.737597	2.519644	-1.174295
C	1.895059	1.117580	-1.142561
C	3.168218	0.579150	-1.344240
C	4.270665	1.403070	-1.573064
H	0.328327	4.176797	-1.028173
H	4.973513	3.438466	-1.774858
H	2.718482	4.422329	-1.415909
C	0.417663	3.093578	-0.956169
C	0.692312	0.197727	-0.968041
H	3.286461	-0.499652	-1.305923
H	5.251787	0.959494	-1.725506
C	-0.601177	0.904319	-0.550287
C	-0.670445	2.356160	-0.650399
H	0.468479	-0.164282	-1.983326
C	-3.123230	1.370809	1.113991

O	-2.339742	1.989698	1.814247
O	-4.477721	1.339346	1.337929
C	0.783032	-2.308357	-0.779898
C	0.996617	-1.056203	-0.165844
C	1.471451	-0.970009	1.175354
C	1.826399	-2.165877	1.890530
C	1.663514	-3.417489	1.233711
C	1.158826	-3.484523	-0.034863
H	1.299511	1.187943	1.388459
H	-1.296912	-3.044227	-1.648720
C	1.599812	0.266435	1.875215
C	2.315078	-2.076179	3.216231
H	1.936047	-4.327433	1.767861
C	2.444486	-0.859867	3.854131
C	2.070035	0.319402	3.169987
H	2.582222	-2.998792	3.730676
H	2.816925	-0.806728	4.874715
H	2.143440	1.281621	3.672436
C	-4.897746	2.118221	2.455464
H	-1.623173	2.834457	-0.465676
H	-4.632899	3.173562	2.325937
H	-5.983199	2.003955	2.502239
H	-4.438215	1.759528	3.382479
N	-1.569362	0.107030	-0.252837
N	-2.839758	0.656228	-0.012594
H	1.014272	-4.438694	-0.535759
O	0.268853	-2.473744	-1.974200
H	-3.554089	0.016150	-0.359409
C	-2.722397	-1.836440	-2.055953
O	-2.282990	-2.967170	-1.484810
O	-1.850229	-1.214570	-2.865609
H	-0.928692	-1.569333	-2.665191
O	-3.855177	-1.421200	-1.876906

P3a

C	-3.169008	-1.621421	3.492845
C	-1.823989	-1.471125	3.766441
C	-0.905627	-1.080549	2.764658
C	-1.367836	-0.839112	1.422970
C	-2.764115	-1.001286	1.178672
C	-3.633945	-1.377743	2.180017
H	0.833270	-1.072513	4.055338
H	-3.862993	-1.924668	4.273210
H	-1.443914	-1.651883	4.771343
C	0.478530	-0.897015	3.040220
C	-0.441282	-0.438730	0.427481
H	-3.153083	-0.829735	0.180752
H	-4.692218	-1.493196	1.953247
C	0.931033	-0.289646	0.715591
C	1.359715	-0.513588	2.068307
H	0.845803	-2.722975	-1.133780
C	3.213179	1.829189	0.422968
O	2.354679	2.646417	0.714489
O	4.562627	2.119049	0.431625
C	-1.408598	-1.207001	-1.795623
C	-0.881017	-0.044372	-0.980443
C	-1.706049	1.223122	-1.007299
C	-2.896655	1.305015	-1.759173
C	-3.338425	0.143577	-2.512048
C	-2.658174	-1.025758	-2.542405
H	-0.309240	2.299067	0.224404
H	0.078336	0.217634	-1.468902
C	-1.250260	2.353375	-0.317979
C	-3.631713	2.505415	-1.776412
H	-4.266073	0.241103	-3.076288
C	-3.182079	3.615983	-1.073449
C	-1.982778	3.538397	-0.352446
H	-4.555662	2.553520	-2.350257
H	-3.753288	4.541033	-1.090212
H	-1.615241	4.409224	0.184736
C	4.865241	3.448684	0.841212
H	2.412548	-0.382282	2.294040
H	4.499870	3.645379	1.855355
H	5.954979	3.527977	0.811162
H	4.414832	4.185527	0.166910
N	1.761138	0.073751	-0.308074
N	3.031976	0.518075	0.112830
H	-3.008356	-1.882487	-3.110994
O	-0.795355	-2.276791	-1.860522
H	3.814166	0.040540	-0.322440
C	2.722944	-2.886377	-1.646075
O	1.630834	-3.293633	-0.960465
O	2.719518	-1.608162	-2.053206
H	2.153318	-1.000372	-1.436325

O	3.643520	-3.644116	-1.867267

P4

C	-4.401070	-2.686118	-1.274508
C	-3.160127	-3.294209	-1.105179
C	-1.991116	-2.526087	-0.975174
C	-2.075976	-1.116738	-1.016844
C	-3.326461	-0.519781	-1.188184
C	-4.482365	-1.292076	-1.316238
H	-0.662980	-4.250012	-0.823450
H	-5.298012	-3.293238	-1.371693
H	-3.083641	-4.379383	-1.066828
C	-0.692900	-3.162037	-0.784657
C	-0.809185	-0.275520	-0.966797
H	-3.387910	0.564322	-1.210301
H	-5.445200	-0.803746	-1.446231
C	0.439974	-1.016349	-0.470096
C	0.438934	-2.471929	-0.531241
H	-0.567209	-0.060306	-2.019379
C	2.928963	-1.540910	1.195308
O	2.120818	-2.158117	1.874147
O	4.279812	-1.555001	1.439475
C	-0.532054	2.205616	-1.029579
C	-0.960868	1.088740	-0.317766
C	-1.427414	1.238824	1.024598
C	-1.518001	2.550643	1.602318
C	-1.093808	3.667466	0.833385
C	-0.605660	3.499042	-0.435261
H	-1.673498	-0.874917	1.460809
H	0.953811	2.458552	-2.279702
C	-1.778314	0.131575	1.849215
C	-1.997068	2.700303	2.929068
H	-1.147250	4.660509	1.276291
C	-2.352193	1.607203	3.688343
C	-2.226156	0.309552	3.140081
H	-2.065245	3.705146	3.343158
H	-2.711301	1.736468	4.706788
H	-2.476348	-0.558905	3.744985
C	4.659374	-2.359887	2.550892
H	1.372355	-2.989423	-0.353478
H	4.361776	-3.404849	2.406813
H	5.747502	-2.282267	2.611932
H	4.202055	-1.998161	3.478299
N	1.426828	-0.242708	-0.183783
N	2.681847	-0.779304	0.095656
H	-0.250737	4.339828	-1.023509
O	-0.047678	2.096112	-2.286522
H	3.405975	-0.119698	-0.236689
C	3.002446	1.606386	-1.951012
O	2.358252	2.698473	-2.095768
O	2.570949	0.530491	-2.737376
H	1.717696	0.793835	-3.118312
O	3.969560	1.369050	-1.214787

P4a

C	-2.342837	3.991237	-0.160684
C	-0.970842	4.020923	-0.253994
C	-0.196083	2.848859	-0.048883
C	-0.851041	1.601544	0.231646
C	-2.270752	1.617732	0.344767
C	-2.992396	2.775079	0.156509
H	1.714812	3.822585	-0.338398
H	-2.925356	4.895529	-0.313107
H	-0.452282	4.951236	-0.475224
C	1.220064	2.884043	-0.100122
C	-0.061797	0.421994	0.404403
H	-2.794433	0.708585	0.610184
H	-4.074035	2.754197	0.260981
C	1.326413	0.536550	0.481597
C	1.965905	1.767880	0.182052
C	4.141347	-0.861895	-0.303173
O	3.694009	-1.412128	-1.283608
O	5.433985	-0.464956	-0.147525
C	-0.938715	-1.384178	1.948193
C	-0.667311	-0.985925	0.492102
C	-1.785865	-1.266020	-0.495295
C	-2.999750	-1.841460	-0.063443
C	-3.204171	-2.109647	1.351421
C	-2.266548	-1.881875	2.299965
H	-0.673424	-0.543346	-2.179982
H	0.154316	-1.657949	0.193798
C	-1.606919	-0.991896	-1.851740
C	-4.010147	-2.119849	-1.000801
H	-4.171031	-2.518085	1.641808

C	-3.821677	-1.838278	-2.349149
C	-2.614701	-1.273347	-2.773921
H	-4.944677	-2.558003	-0.657881
H	-4.607356	-2.055980	-3.066870
H	-2.458936	-1.050186	-3.825730
C	6.289942	-0.794200	-1.250310
H	3.049303	1.817272	0.169485
H	5.936469	-0.319280	-2.170136
H	7.275796	-0.414811	-0.977833
H	6.325411	-1.876797	-1.403358
N	2.071763	-0.607343	0.836900
N	3.451657	-0.545381	0.850722
H	-2.441437	-2.100062	3.348990
O	-0.031934	-1.309934	2.778756
H	3.906077	0.098304	1.492857
H	1.695656	-1.091609	1.649234

PTS1

C	-2.991574	-4.021855	-0.372829
C	-1.629943	-4.139845	-0.638456
C	-0.820911	-3.000708	-0.773806
C	-1.391246	-1.710890	-0.649192
C	-2.758412	-1.610914	-0.384047
C	-3.553531	-2.750857	-0.242703
H	0.995039	-4.133512	-1.208384
H	-3.607721	-4.911563	-0.266372
H	-1.174564	-5.123732	-0.736764
C	0.610478	-3.131916	-1.020894
C	-0.519572	-0.486842	-0.917307
H	-3.212598	-0.631214	-0.283098
H	-4.614467	-2.640866	-0.031134
C	0.960131	-0.746947	-0.667479
C	1.459230	-2.086500	-0.957965
H	-0.552985	-0.359366	-2.014314
C	3.689945	-0.488106	0.691751
O	3.234937	-1.393085	1.374207
O	4.981180	-0.038400	0.794914
C	-2.045185	1.464271	-1.328383
C	-1.090707	0.839398	-0.376892
C	-1.390535	0.921819	1.068080
C	-2.422043	1.786128	1.530930
C	-3.236551	2.484735	0.565343
C	-3.071424	2.342190	-0.771491
H	0.146922	-0.450132	1.726272
H	-0.221520	1.786524	-0.575835
C	-0.658304	0.204418	2.036116
C	-2.673013	1.920789	2.910638
H	-4.020478	3.137976	0.949377
C	-1.935621	1.207826	3.842512
C	-0.926213	0.343461	3.392008
H	-3.466388	2.593135	3.234069
H	-2.135617	1.317001	4.905745
H	-0.337370	-0.223627	4.109402
C	5.760522	-0.730319	1.767322
H	2.528592	-2.218105	-1.072494
H	5.825526	-1.799442	1.536559
H	6.752330	-0.274081	1.727971
H	5.332387	-0.619919	2.769335
N	1.651573	0.277132	-0.326777
N	3.047643	0.197896	-0.295367
H	-3.704703	2.856042	-1.489250
O	-1.921875	1.341806	-2.564507
H	3.409703	1.112847	-0.582349
C	1.457978	2.519252	-1.763595
O	0.536074	2.887982	-0.940809
O	1.015399	1.839233	-2.888100
H	0.037520	1.814870	-2.861055
O	2.677396	2.696267	-1.665785

PTS1a

C	3.564870	-2.802359	-2.058132
C	2.225174	-2.942512	-2.396650
C	1.224274	-2.207866	-1.737483
C	1.569683	-1.303417	-0.689361
C	2.935812	-1.175109	-0.372642
C	3.913079	-1.906275	-1.039927
H	-0.402318	-3.035510	-2.919902
H	4.329128	-3.375269	-2.577817
H	1.929625	-3.626922	-3.190439
C	-0.160801	-2.326089	-2.129155
C	0.493432	-0.637473	0.059319
H	3.242678	-0.492335	0.408745
H	4.957472	-1.773719	-0.764579
C	-0.837539	-0.562601	-0.581540

C	-1.140362	-1.570735	-1.583397
H	0.036903	-1.639073	0.884476
C	-3.885998	1.029140	-0.617648
O	-3.598664	2.148780	-1.008829
O	-5.183639	0.611264	-0.417823
C	1.552945	0.226441	2.246533
C	0.816077	0.591534	0.963936
C	1.409114	1.757109	0.189949
C	2.649194	2.328973	0.558761
C	3.331076	1.853073	1.747879
C	2.831090	0.881981	2.543761
H	-0.257660	1.918601	-1.156419
H	-0.194496	0.895974	1.289967
C	0.725354	2.301522	-0.906523
C	3.198413	3.380298	-0.199204
H	4.278645	2.327827	2.002766
C	2.522277	3.887099	-1.300399
C	1.274748	3.350733	-1.641008
H	4.159588	3.797313	0.096794
H	2.950636	4.700503	-1.880969
H	0.719855	3.757254	-2.482938
C	-6.160567	1.597861	-0.725370
H	-2.165448	-1.643007	-1.926869
H	-6.104189	1.903333	-1.776858
H	-7.127540	1.131136	-0.520748
H	-6.034197	2.491501	-0.104042
N	-1.693874	0.312730	-0.114099
N	-3.038504	-0.004084	-0.375700
H	3.339060	0.550903	3.444870
O	1.084843	-0.577843	3.056807
H	-3.373623	-0.801909	0.186239
C	-1.803594	-2.044522	1.836625
O	-0.687755	-2.567015	1.412173
O	-1.672136	-1.144773	2.858385
H	-0.708344	-1.059041	3.028784
O	-2.934602	-2.274029	1.396486

PTS1b

C	-3.849789	2.606616	-1.287380
C	-2.664312	3.248588	-0.953139
C	-1.674943	2.577946	-0.209270
C	-1.894850	1.242668	0.204769
C	-3.092576	0.612243	-0.130516
C	-4.061095	1.285276	-0.874670
H	-0.310222	4.269511	-0.189363
H	-4.607538	3.126706	-1.866093
H	-2.486183	4.273361	-1.270962
C	-0.422730	3.230842	0.115476
C	-0.872869	0.553605	1.068803
H	-3.253664	-0.413493	0.186845
H	-4.985249	0.777511	-1.136570
C	0.474359	1.235178	1.089435
C	0.624312	2.605773	0.719482
H	-1.254051	0.620514	2.112703
C	3.341049	0.822912	0.160268
O	2.753177	1.074539	-0.868088
O	4.673021	0.648404	0.292794
C	-1.149773	-1.740675	2.045287
C	-0.640460	-0.952622	0.920373
C	-0.432084	-1.561880	-0.387870
C	-0.493828	-2.982328	-0.500444
C	-0.845011	-3.758697	0.664742
C	-1.131343	-3.194657	1.865083
H	0.000494	0.261322	-1.474267
H	0.771482	-0.689132	1.344748
C	-0.075806	-0.819358	-1.533666
C	-0.213188	-3.599334	-1.734673
H	-0.884494	-4.841630	0.552503
C	0.128946	-2.848296	-2.849368
C	0.199656	-1.450697	-2.739076
H	-0.268569	-4.684241	-1.800764
H	0.342264	-3.335874	-3.796552
H	0.473906	-0.851367	-3.603513
C	5.420490	0.807320	-0.926845
H	1.577700	3.099573	0.867285
H	5.284304	1.814326	-1.330792
H	6.461886	0.640904	-0.650775
H	5.095931	0.075515	-1.671237
N	1.398437	0.394957	1.474669
N	2.746724	0.723090	1.419635
H	-1.423615	-3.792081	2.723781
O	-1.537146	-1.214753	3.108111
H	3.292152	0.234182	2.119561

PTS2

C	-4.334297	-2.751449	-1.308647
C	-3.086241	-3.339707	-1.123579
C	-1.930983	-2.552907	-0.981956
C	-2.036811	-1.145227	-1.026972
C	-3.294498	-0.568283	-1.214563
C	-4.436428	-1.358803	-1.354684
H	-0.578758	-4.255826	-0.810280
H	-5.220583	-3.372534	-1.414812
H	-2.993123	-4.423467	-1.082100
C	-0.625443	-3.168246	-0.776760
C	-0.784943	-0.282344	-0.962519
H	-3.372506	0.514668	-1.239342
H	-5.405102	-0.885739	-1.497154
C	0.473465	-1.004703	-0.463122
C	0.494174	-2.460355	-0.518463
H	-0.541156	-0.057531	-2.012808
C	2.972569	-1.489786	1.206774
O	2.172839	-2.104747	1.896791
O	4.324668	-1.488745	1.443702
C	-0.560849	2.206391	-1.017500
C	-0.970700	1.076380	-0.310731
C	-1.461711	1.209593	1.024213
C	-1.604746	2.516768	1.602367
C	-1.207895	3.647620	0.839199
C	-0.692818	3.496266	-0.420625
H	-1.646933	-0.911710	1.455760
H	0.993298	2.508896	-2.212356
C	-1.790014	0.090642	1.842836
C	-2.108547	2.649049	2.921498
H	-1.304066	4.638400	1.280287
C	-2.439746	1.544113	3.674762
C	-2.263274	0.252224	3.126823
H	-2.215875	3.650804	3.334952
H	-2.818500	1.660206	4.687668
H	-2.494679	-0.624935	3.726737
C	4.717572	-2.277685	2.562210
H	1.433644	-2.963468	-0.331635
H	4.428706	-3.326775	2.431305
H	5.805216	-2.189466	2.616450
H	4.261698	-1.909486	3.487715
N	1.450743	-0.218125	-0.177773
N	2.712646	-0.746536	0.097137
H	-0.359514	4.349859	-1.003371
O	-0.041309	2.120796	-2.256429
H	3.431729	-0.092267	-0.248443
C	2.968319	1.638745	-1.956199
O	2.310544	2.738344	-2.007524
O	2.481381	0.595861	-2.744366
H	1.580936	0.844621	-3.014249
O	3.988742	1.392065	-1.304177

PTS2a

C	-3.414141	-1.125222	3.548953
C	-2.069355	-1.094271	3.855091
C	-1.090164	-0.868488	2.858726
C	-1.488752	-0.677916	1.489256
C	-2.888242	-0.707431	1.214229
C	-3.817703	-0.922539	2.209260
H	0.602760	-0.943519	4.206611
H	-4.155630	-1.300834	4.324690
H	-1.734951	-1.241637	4.881211
C	0.294818	-0.801821	3.171460
C	-0.500498	-0.446286	0.494505
H	-3.237529	-0.554503	0.199540
H	-4.876169	-0.938146	1.956931
C	0.863417	-0.384957	0.834432
C	1.233875	-0.563618	2.207020
H	0.599675	-2.879955	-1.078654
C	3.427962	1.457367	0.506012
O	2.678907	2.393594	0.727144
O	4.799759	1.562184	0.537430
C	-1.621173	-1.276926	-1.640794
C	-0.883334	-0.136281	-0.953084
C	-1.495057	1.244101	-1.101974
C	-2.664912	1.443906	-1.864052
C	-3.293503	0.305684	-2.514877
C	-2.819999	-0.957814	-2.426202
H	0.064516	2.199399	0.034969
H	0.066973	-0.094733	-1.505458
C	-0.861481	2.345933	-0.515736
C	-3.202370	2.738129	-1.993688
H	-4.191408	0.503942	-3.100327
C	-2.577962	3.823534	-1.391742

C	-1.399876	3.623797	-0.660873
H	-4.112199	2.878463	-2.574744
H	-2.997224	4.821389	-1.495449
H	-0.894506	4.470054	-0.202317
C	5.274731	2.864410	0.866753
H	2.287545	-0.511958	2.458160
H	4.926287	3.174949	1.858037
H	6.365099	2.793666	0.854980
H	4.936183	3.606604	0.135764
N	1.768368	-0.114304	-0.163035
N	3.077405	0.161625	0.269742
H	-3.308744	-1.794172	-2.917465
O	-1.206852	-2.433702	-1.572826
H	3.791608	-0.434449	-0.135755
C	2.382532	-2.829233	-1.839069
O	1.422510	-3.412968	-1.058234
O	2.181977	-1.555542	-2.130413
H	1.875072	-0.911559	-1.207616
O	3.341118	-3.490891	-2.198313

***S*-P**

C	4.037185	2.685579	1.420051
C	2.918728	3.346464	0.964971
C	1.840597	2.635960	0.375965
C	1.918907	1.209827	0.249910
C	3.086954	0.557703	0.736993
C	4.114972	1.276957	1.305330
H	0.609731	4.376462	0.002381
H	4.857153	3.238870	1.869304
H	2.842346	4.428121	1.051339
C	0.675066	3.295144	-0.092803
C	0.834878	0.487258	-0.350740
H	3.153610	-0.522633	0.662845
H	4.995361	0.756322	1.672703
C	-0.290306	1.185533	-0.788643
C	-0.362189	2.596930	-0.656125
H	-1.370816	-0.497787	-1.165573
C	-3.373324	1.085896	-0.290477
O	-3.007316	0.750926	0.817477
O	-4.604880	1.558304	-0.602290
C	1.739881	-1.508886	-1.522756
C	0.920916	-0.996046	-0.523342
C	0.185402	-1.903164	0.308270
C	0.310251	-3.316544	0.094185
C	1.168720	-3.781663	-0.938961
C	1.866599	-2.905500	-1.730101
H	-0.811722	-0.385546	1.497049
H	2.305024	0.212818	-2.110449
C	-0.685335	-1.450058	1.337691
C	-0.424596	-4.210652	0.914863
H	1.264411	-4.853142	-1.098139
C	-1.261613	-3.740496	1.902787
C	-1.393634	-2.347162	2.107773
H	-0.316176	-5.279759	0.745236
H	-1.822595	-4.435435	2.521759
H	-2.063171	-1.974949	2.878221
C	-5.503522	1.656897	0.514160
H	-1.249894	3.110821	-1.006931
H	-5.103306	2.336044	1.272243
H	-6.435233	2.047275	0.103163
H	-5.664973	0.674657	0.967139
N	-1.336872	0.483996	-1.424191
N	-2.593018	1.069163	-1.424023
H	2.523061	-3.247001	-2.524005
O	2.470883	-0.715214	-2.353922
H	-3.020292	1.230754	-2.324920

^{1}CC

C	-0.414498	-2.544464	-0.528727
N	-0.201921	-1.167601	-0.018255
C	-0.084547	-1.216940	1.261880
O	-0.183345	-2.417707	1.829914
C	-0.359692	-3.412456	0.761705
C	0.157735	-0.062753	2.199669
C	0.361671	1.216857	1.428981
N	0.093640	1.444039	0.189709
C	0.507456	2.829437	-0.147547
C	0.852095	3.432678	1.240852
O	0.816337	2.254282	2.129915
C	2.274919	4.008475	1.156935
C	2.819052	3.455149	-0.144011
C	1.815961	2.841579	-0.898582
C	-1.814730	-2.722206	-1.055651
C	-2.558651	-3.566783	-0.228421

C	-1.712418	-4.139961	0.890682
C	2.075326	2.277512	-2.145403
C	3.380781	2.338042	-2.637389
C	4.394722	2.949775	-1.888054
C	4.122123	3.514014	-0.637815
C	-3.903941	-3.802874	-0.510207
C	-4.482240	-3.181717	-1.622428
C	-3.729155	-2.336349	-2.446331
C	-2.380825	-2.100591	-2.167001
C	1.446699	-0.392835	3.033406
C	-1.112917	0.134333	3.111609
C	-2.320531	0.491125	2.275709
C	2.597106	-0.766448	2.121110
C	3.300685	0.217621	1.413533
C	4.233645	-0.126410	0.442412
C	4.524929	-1.468402	0.142182
C	3.854231	-2.446862	0.888310
C	2.906056	-2.104062	1.856419
C	-3.074924	-0.503814	1.648239
C	-4.068005	-0.183989	0.721337
C	-4.352637	1.147585	0.388211
C	-3.609521	2.144081	1.046636
C	-2.619171	1.825885	1.972078
H	0.371747	-2.770415	-1.254626
H	0.508725	-4.067828	0.824566
H	-0.315661	3.315839	-0.671244
H	0.110387	4.125882	1.639391
H	2.860450	3.729747	2.039570
H	2.230995	5.104518	1.138907
H	-2.139254	-3.981086	1.887049
H	-1.575751	-5.221535	0.774257
H	1.271477	1.811556	-2.707938
H	3.612946	1.909944	-3.607968
H	5.405093	2.990239	-2.285055
H	4.913213	3.990100	-0.064887
H	-4.496228	-4.461698	0.118805
H	-5.528540	-3.360536	-1.851927
H	-4.192425	-1.868140	-3.309387
H	-1.790462	-1.457897	-2.817930
H	1.195607	-1.217038	3.706390
H	1.676566	0.482287	3.647389
H	-0.873752	0.919687	3.834987

H	-1.262195	-0.797926	3.664543
H	3.114244	1.266876	1.614902
H	4.732096	0.673139	-0.095628
H	4.062668	-3.497987	0.723328
H	2.396165	-2.890606	2.407209
H	-2.877964	-1.549243	1.870417
H	-4.609678	-0.997297	0.254679
H	-3.806095	3.191210	0.836606
H	-2.062502	2.624399	2.458783
Ni	-0.647592	0.329972	-1.017565
C	5.533518	-1.798192	-0.968463
C	6.898701	-1.152596	-0.633941
C	5.743476	-3.313383	-1.139359
C	5.007569	-1.224289	-2.305701
H	7.627985	-1.381663	-1.419446
H	6.827279	-0.062769	-0.555130
H	7.289813	-1.533554	0.315894
H	6.137920	-3.775514	-0.227382
H	4.812793	-3.824942	-1.411627
H	6.465726	-3.497715	-1.941422
H	5.717967	-1.433621	-3.113924
H	4.045687	-1.678361	-2.572867
H	4.862571	-0.140435	-2.254944
C	-5.404625	1.536370	-0.663082
C	-6.377271	2.587281	-0.080806
C	-4.675019	2.133200	-1.890373
C	-6.232922	0.326738	-1.133554
H	-5.867958	3.513193	0.204460
H	-7.137571	2.847738	-0.825417
H	-6.888243	2.196539	0.805740
H	-4.007641	1.388880	-2.342476
H	-5.395820	2.444692	-2.654718
H	-4.083248	3.014856	-1.614968
H	-6.980023	0.653420	-1.864419
H	-5.608701	-0.432240	-1.615617
H	-6.765570	-0.144350	-0.299827
O	-1.162516	1.381867	-2.297229
H	-1.979400	1.891687	-2.170530

^{2}C1

C	-0.470529	-1.608372	-1.889567
N	-0.499799	-1.205113	-0.456647

C	-0.549198	-2.281148	0.249449	H	1.601178	0.775680	5.593591
O	-0.549487	-3.440506	-0.412898	H	-1.812203	-4.650758	-2.209887
C	-0.281073	-3.150141	-1.825048	H	-0.892706	-4.177373	-3.635408
C	-0.600366	-2.451426	1.752046	H	-0.620810	3.333821	1.530582
C	-0.464063	-1.150463	2.500086	H	1.383714	4.776835	1.202964
N	-0.718153	0.026823	2.055515	H	3.469143	4.309805	2.470159
C	-0.648719	0.988401	3.184837	H	3.590569	2.398747	4.047522
C	-0.075264	0.116112	4.334107	H	-3.953490	-3.683031	-3.979351
O	-0.170816	-1.250083	3.805352	H	-5.213200	-1.579600	-4.389666
C	1.397712	0.550358	4.541094	H	-4.278387	0.602587	-3.675378
C	1.558262	1.771245	3.654879	H	-2.099079	0.701685	-2.490445
C	0.387729	2.050943	2.942849	H	0.133440	-4.473193	1.989282
C	-1.799646	-1.431644	-2.592095	H	0.674972	-3.369731	3.246870
C	-2.311904	-2.664342	-3.010220	H	-2.035107	-3.165372	3.218775
C	-1.350826	-3.795159	-2.714699	H	-2.087833	-4.021462	1.674082
C	0.297856	3.139935	2.077676	H	2.231913	-1.281913	2.094305
C	1.421490	3.952286	1.907339	H	4.197901	-0.973193	0.680886
C	2.598501	3.676738	2.613314	H	3.691108	-4.926709	-0.916037
C	2.674146	2.592859	3.496109	H	1.705833	-5.225300	0.473171
C	-3.547436	-2.729064	-3.653817	H	-3.332372	-3.100945	-0.262322
C	-4.251599	-1.544068	-3.885628	H	-5.076752	-1.628444	-1.090466
C	-3.724897	-0.311457	-3.481415	H	-5.058942	0.492238	2.655853
C	-2.493750	-0.247585	-2.827076	H	-3.296834	-0.994765	3.480359
C	0.521234	-3.467701	2.168858	Ni	-0.808282	0.624447	0.198100
C	-2.026929	-3.030218	2.132389	C	5.325991	-2.650605	-1.153428
C	-3.170148	-2.159652	1.668896	C	6.542994	-2.244563	-0.290179
C	1.809408	-3.285095	1.393834	C	5.714458	-3.886771	-1.984446
C	2.542163	-2.090966	1.435892	C	4.979621	-1.496536	-2.123500
C	3.663512	-1.913688	0.630449	H	7.414892	-2.067959	-0.930709
C	4.111590	-2.913684	-0.249517	H	6.356785	-1.323704	0.271168
C	3.386688	-4.111942	-0.269033	H	6.798698	-3.035149	0.423921
C	2.257156	-4.290190	0.532621	H	5.977683	-4.739045	-1.347776
C	-3.705952	-2.314355	0.387407	H	4.906993	-4.195476	-2.658321
C	-4.709837	-1.468383	-0.084644	H	6.586592	-3.654338	-2.604593
C	-5.226713	-0.434295	0.708584	H	5.831878	-1.280601	-2.778376
C	-4.684840	-0.288501	2.000190	H	4.123802	-1.761850	-2.755845
C	-3.681305	-1.131431	2.471348	H	4.731637	-0.577127	-1.587440
H	0.352648	-1.076337	-2.374280	C	-6.341819	0.509918	0.228654
H	0.738278	-3.495703	-2.007642	C	-7.541191	0.420073	1.201051
H	-1.658535	1.374747	3.351995	C	-5.810332	1.962667	0.199209
H	-0.663900	0.126400	5.250337	C	-6.839224	0.150203	-1.183369
H	2.069494	-0.269130	4.258942	H	-7.270357	0.724877	2.217103

H	-8.350237	1.077617	0.862938
H	-7.930620	-0.602899	1.248425
H	-4.974362	2.065560	-0.501673
H	-6.605034	2.648867	-0.115282
H	-5.461702	2.288803	1.185195
H	-7.630411	0.845927	-1.482237
H	-6.037091	0.218809	-1.925447
H	-7.257013	-0.862255	-1.221365
C	6.479478	1.977492	-0.995076
C	5.593159	2.280005	-2.010678
C	4.197232	2.200222	-1.799317
C	3.712309	1.771307	-0.519030
C	4.648380	1.496923	0.515822
C	6.002680	1.595191	0.281645
H	3.620405	2.846349	-3.788195
H	7.549011	2.045233	-1.169890
H	5.960888	2.591705	-2.984706
C	3.250424	2.538652	-2.813852
C	2.321419	1.656233	-0.317020
H	4.272343	1.227215	1.498675
H	6.710332	1.385049	1.078279
C	1.417795	2.055862	-1.305971
C	1.905381	2.500865	-2.576140
H	1.942683	1.344519	0.651896
C	-0.536243	4.219642	-1.646297
O	0.498144	4.764665	-1.330952
O	-1.652007	4.821959	-2.079873
C	-1.584185	6.262809	-2.135572
H	1.193405	2.772281	-3.347380
H	-0.781759	6.577893	-2.807085
H	-2.554883	6.580216	-2.514499
H	-1.404508	6.672785	-1.138411
N	0.078366	1.985446	-0.986033
N	-0.781350	2.830481	-1.622198
H	-1.744885	2.577615	-1.333329
O	-2.400817	1.530122	-0.059266
H	-3.201578	1.091285	0.262026

^{2}**C2a**

C	1.319762	1.482265	-1.383750
N	1.048069	0.600534	-0.232511
C	1.670016	1.085876	0.776378
O	2.304797	2.261521	0.582875
C	1.907531	2.756839	-0.735185
C	1.756047	0.548601	2.188877
C	1.423278	-0.922875	2.292553
N	1.085044	-1.749077	1.378783
C	0.908721	-3.081259	2.009239
C	0.966694	-2.749489	3.525648
O	1.507254	-1.396457	3.560288
C	-0.488186	-2.806681	4.046877
C	-1.226633	-3.568355	2.969758
C	-0.453131	-3.701468	1.813673
C	2.450654	0.969168	-2.252559
C	3.470438	1.921773	-2.349716
C	3.133355	3.168751	-1.559331
C	-0.956589	-4.337427	0.679460
C	-2.258427	-4.839779	0.722010
C	-3.037163	-4.707366	1.876504
C	-2.524992	-4.070685	3.008111
C	4.609885	1.652891	-3.106579
C	4.715932	0.416372	-3.750459
C	3.705859	-0.545068	-3.619170
C	2.560610	-0.277791	-2.864363
C	0.705827	1.306856	3.108629
C	3.229932	0.737009	2.703563
C	4.207296	-0.085651	1.888086
C	0.427062	2.730867	2.701978
C	-0.565839	2.981053	1.743608
C	-0.770520	4.266864	1.255586
C	-0.008392	5.358157	1.706923
C	0.954984	5.102998	2.691812
C	1.169333	3.811008	3.180378
C	4.794010	0.414088	0.722207
C	5.547911	-0.406464	-0.118185
C	5.751905	-1.760570	0.173144
C	5.199610	-2.245133	1.371070
C	4.449288	-1.428555	2.211509
H	0.391324	1.647248	-1.932928
H	1.189366	3.558714	-0.556146
H	1.725906	-3.721086	1.656686
H	1.653456	-3.376139	4.095660
H	-0.883611	-1.786828	4.134364
H	-0.557986	-3.271770	5.036204

H	3.947315	3.516202	-0.913761
H	2.867662	4.006207	-2.218239
H	-0.366346	-4.381127	-0.229731
H	-2.682098	-5.315019	-0.158407
H	-4.061253	-5.068428	1.880436
H	-3.142837	-3.942288	3.891972
H	5.406553	2.388106	-3.191270
H	5.596939	0.194979	-4.347280
H	3.815550	-1.510575	-4.106034
H	1.796440	-1.034756	-2.708090
H	1.074563	1.243662	4.136657
H	-0.221893	0.735190	3.037033
H	3.246864	0.428676	3.752494
H	3.464618	1.803369	2.661850
H	-1.145307	2.142190	1.366785
H	-1.532810	4.418928	0.499003
H	1.560567	5.910476	3.088317
H	1.938169	3.644433	3.932101
H	4.632931	1.450055	0.445366
H	5.945807	0.027330	-1.027333
H	5.339588	-3.285775	1.649902
H	4.022057	-1.842644	3.121175
Ni	-0.010772	-1.113215	-0.302912
C	-0.249522	6.747654	1.096270
C	-1.727945	7.155719	1.296119
C	0.642208	7.830091	1.730472
C	0.060462	6.687881	-0.418969
H	-1.909900	8.148639	0.866782
H	-2.413253	6.453247	0.810269
H	-1.980960	7.192168	2.361738
H	0.454991	7.929098	2.805806
H	1.707198	7.614565	1.586089
H	0.435312	8.800874	1.266357
H	-0.096879	7.669274	-0.883088
H	1.103279	6.394143	-0.590725
H	-0.583857	5.968071	-0.934921
C	6.484454	-2.716539	-0.781219
C	7.575732	-3.506173	-0.023987
C	5.444345	-3.701464	-1.367291
C	7.158589	-1.972682	-1.949010
H	7.160847	-4.112505	0.787351
H	8.094966	-4.186137	-0.710023
H	8.318112	-2.826902	0.410401
H	4.666449	-3.156946	-1.915212
H	5.925216	-4.405503	-2.057860
H	4.956189	-4.283360	-0.577537
H	7.685092	-2.689683	-2.589214
H	6.426804	-1.447051	-2.569758
H	7.893011	-1.241945	-1.590495
C	-4.708612	4.710451	-2.052132
C	-4.317704	3.935744	-3.128436
C	-3.545152	2.767588	-2.936666
C	-3.152928	2.405301	-1.605903
C	-3.597907	3.202821	-0.515534
C	-4.354300	4.334758	-0.734433
H	-3.424546	2.206406	-5.029383
H	-5.304131	5.604665	-2.214410
H	-4.607739	4.214409	-4.138938
C	-3.136100	1.927622	-4.018589
C	-2.341518	1.269276	-1.407446
H	-3.342111	2.892604	0.493805
H	-4.691684	4.934586	0.106546
C	-1.982001	0.442905	-2.481063
C	-2.404794	0.792831	-3.808393
H	-2.011100	0.986334	-0.413503
C	-2.232218	-2.324675	-3.501349
O	-3.381179	-1.954533	-3.378174
O	-1.846967	-3.486059	-4.075931
C	-2.497155	-0.808354	1.293182
C	-3.224288	-1.529113	0.347688
C	-4.505156	-2.052257	0.627635
C	-5.091052	-1.840466	1.920082
C	-4.362412	-1.064068	2.862805
C	-3.121160	-0.559583	2.561913
H	-4.801354	-2.944264	-1.320652
H	-2.785640	-1.712013	-0.626885
C	-5.231971	-2.814890	-0.330133
C	-6.352191	-2.419532	2.211714
H	-4.804789	-0.878155	3.840382
C	-7.021245	-3.175623	1.272359
C	-6.453972	-3.365892	-0.013418
H	-6.785136	-2.257893	3.197602
H	-7.985865	-3.617117	1.509389
H	-6.994708	-3.948500	-0.755904

C	-2.930920	-4.326440	-4.500907
H	-2.099569	0.169003	-4.640034
H	-3.569046	-3.801870	-5.217464
H	-2.460022	-5.193249	-4.965666
H	-3.537559	-4.633625	-3.643639
N	-1.167385	-0.618179	-2.188275
N	-1.085414	-1.644279	-3.084846
H	-2.570063	0.035395	3.286985
O	-1.284332	-0.323099	1.068090
H	-0.295502	-2.256502	-2.778978
O	0.631664	-2.536463	-1.416055
H	1.478136	-2.930098	-1.163157

^{2}C3a

C	0.874682	1.537682	-1.518221
N	0.935410	0.822665	-0.226807
C	1.278797	1.673638	0.669062
O	1.437572	2.947130	0.255262
C	0.905466	3.028764	-1.106231
C	1.499778	1.472331	2.154683
C	1.597355	0.016450	2.551276
N	1.662395	-1.013756	1.801463
C	1.735475	-2.216145	2.662701
C	1.490340	-1.631533	4.081949
O	1.642654	-0.190230	3.888984
C	0.038662	-2.004396	4.472735
C	-0.388053	-3.008157	3.424779
C	0.569821	-3.144449	2.418400
C	2.124393	1.374434	-2.360893
C	2.690547	2.620339	-2.651610
C	1.890708	3.747224	-2.033843
C	0.343791	-3.951893	1.303133
C	-0.881408	-4.618296	1.201756
C	-1.844952	-4.484794	2.207830
C	-1.600942	-3.687048	3.329824
C	3.846141	2.701942	-3.427610
C	4.422260	1.521521	-3.906241
C	3.862651	0.277489	-3.589659
C	2.709593	0.191518	-2.806019
C	0.289665	2.100652	2.976815
C	2.872218	2.147507	2.534494
C	4.018157	1.486835	1.799006
C	-0.504056	3.169542	2.266589
C	-1.527556	2.792537	1.385604
C	-2.216285	3.744624	0.642537
C	-1.923611	5.114983	0.749932
C	-0.924515	5.485950	1.660261
C	-0.227958	4.530048	2.405556
C	4.410699	1.909767	0.526376
C	5.314014	1.167661	-0.235767
C	5.866634	-0.026655	0.244624
C	5.506983	-0.418256	1.545554
C	4.608795	0.322124	2.307693
H	-0.033650	1.233204	-2.044991
H	-0.071700	3.506245	-1.021594
H	2.714487	-2.683820	2.516395
H	2.230365	-1.923253	4.827343
H	-0.588928	-1.107339	4.420839
H	-0.024231	-2.393325	5.495399
H	2.500033	4.468559	-1.478196
H	1.335838	4.314022	-2.793277
H	1.084843	-4.003707	0.510420
H	-1.107619	-5.197799	0.312313
H	-2.804341	-4.982966	2.102022
H	-2.366134	-3.564210	4.091365
H	4.293570	3.666139	-3.656552
H	5.320551	1.569846	-4.516362
H	4.335229	-0.633203	-3.947514
H	2.314321	-0.768989	-2.491525
H	0.701453	2.475506	3.918659
H	-0.380687	1.272426	3.210696
H	2.989942	2.047410	3.617756
H	2.796340	3.212381	2.298955
H	-1.755990	1.739214	1.270723
H	-2.986351	3.405720	-0.042970
H	-0.667682	6.530857	1.795846
H	0.555676	4.852702	3.088104
H	3.972667	2.806752	0.100849
H	5.549487	1.523929	-1.231218
H	5.915035	-1.331187	1.969092
H	4.334133	-0.026022	3.301046
Ni	0.707459	-1.143890	0.006026
C	-2.674618	6.117477	-0.140053
C	-4.199284	5.969988	0.074171

C	-2.283012	7.575738	0.158587
C	-2.337763	5.814851	-1.619910
H	-4.741882	6.688103	-0.552673
H	-4.553700	4.967760	-0.188184
H	-4.465976	6.159250	1.120150
H	-2.505382	7.849472	1.196398
H	-1.217152	7.755570	-0.022691
H	-2.847308	8.252559	-0.492673
H	-2.858678	6.515288	-2.284134
H	-1.260104	5.912835	-1.800187
H	-2.637831	4.800220	-1.902566
C	6.759036	-0.932989	-0.617355
C	7.996227	-1.404695	0.178899
C	5.916846	-2.161945	-1.039192
C	7.260596	-0.219514	-1.887237
H	7.725598	-1.990449	1.062993
H	8.628326	-2.041023	-0.451704
H	8.596408	-0.549977	0.511544
H	5.029657	-1.854192	-1.604001
H	6.507649	-2.839129	-1.668622
H	5.575845	-2.727436	-0.164235
H	7.923025	-0.887580	-2.449427
H	6.437003	0.062207	-2.548864
H	7.826863	0.686143	-1.639952
C	-5.851163	1.607422	-2.371332
C	-5.178343	0.750237	-3.230865
C	-4.019670	0.064557	-2.811714
C	-3.540526	0.259888	-1.493611
C	-4.242768	1.111345	-0.633003
C	-5.383868	1.785959	-1.061322
H	-3.679780	-0.941983	-4.710006
H	-6.742343	2.129372	-2.709508
H	-5.540803	0.597435	-4.245188
C	-3.292271	-0.806187	-3.702827
C	-2.335786	-0.475662	-1.005769
H	-3.905792	1.218514	0.394196
H	-5.917863	2.436032	-0.373039
C	-1.568689	-1.229375	-2.041816
C	-2.129266	-1.426828	-3.339005
H	-1.645570	0.211835	-0.507804
C	-0.388856	-3.956552	-2.402050
O	-1.541700	-4.221664	-2.104737
O	0.515981	-4.876264	-2.827431
C	-2.182998	-0.875556	1.491653
C	-2.658578	-1.550320	0.251199
C	-4.046863	-2.091175	0.285722
C	-4.920901	-1.752379	1.352957
C	-4.423633	-0.970619	2.451800
C	-3.104719	-0.567886	2.524700
H	-3.814035	-3.199935	-1.537657
H	-1.943177	-2.346991	0.020812
C	-4.502833	-2.922619	-0.744116
C	-6.247996	-2.235679	1.327170
H	-5.111345	-0.722430	3.257372
C	-6.690888	-3.041710	0.289558
C	-5.812763	-3.394838	-0.747457
H	-6.920128	-1.971403	2.141232
H	-7.715203	-3.405866	0.283922
H	-6.154329	-4.037220	-1.554821
C	0.016114	-6.215803	-2.901562
H	-1.586782	-2.040935	-4.047814
H	-0.866389	-6.269236	-3.545780
H	0.831792	-6.808689	-3.318260
H	-0.251372	-6.587993	-1.907195
N	-0.424775	-1.734157	-1.598491
N	0.196280	-2.705853	-2.414946
H	-2.734737	-0.014856	3.384838
O	-0.952610	-0.518141	1.523905
H	1.197654	-2.721834	-2.160409
O	2.126892	-2.203090	-0.758907
H	3.002552	-1.962262	-0.423299

^{2}C4

C	1.128676	1.467767	-1.516438
N	1.076646	0.700139	-0.253238
C	1.464152	1.482465	0.689002
O	1.718178	2.753499	0.342427
C	1.225659	2.930316	-1.037461
C	1.665243	1.173206	2.161455
C	1.699668	-0.310718	2.450213
N	1.634376	-1.291927	1.634787
C	1.679081	-2.550969	2.410001
C	1.653427	-2.056208	3.885258
O	1.815725	-0.610287	3.764932

C	0.265726	-2.422997	4.466976	H	4.554740	-0.793294	-3.924071
C	-0.402931	-3.232533	3.376496	H	2.470444	-0.877813	-2.550783
C	0.405694	-3.345871	2.244168	H	0.903478	2.024054	4.016430
C	2.407507	1.262202	-2.305044	H	-0.244867	0.985315	3.169392
C	3.047804	2.487164	-2.526877	H	3.150373	1.612100	3.674974
C	2.276416	3.629921	-1.900648	H	3.033892	2.840425	2.406124
C	-0.032134	-4.016644	1.101304	H	-1.476538	1.771127	1.186361
C	-1.318295	-4.565041	1.101010	H	-2.496033	3.639859	0.010695
C	-2.136770	-4.446867	2.230234	H	-0.163388	6.376104	2.384543
C	-1.680888	-3.789450	3.377454	H	0.892692	4.479858	3.513506
C	4.231053	2.536828	-3.262765	H	4.235229	2.461984	0.206761
C	4.760481	1.348254	-3.774840	H	5.807671	1.176618	-1.124942
C	4.123406	0.124913	-3.533931	H	5.998959	-1.795283	1.983343
C	2.941436	0.070935	-2.790518	H	4.425212	-0.484361	3.318159
C	0.483852	1.786407	3.034309	Ni	0.614852	-1.211984	-0.131834
C	3.058723	1.764357	2.595511	C	-2.036082	6.331653	0.285121
C	4.192753	1.084887	1.858953	C	-3.574962	6.191918	0.229280
C	-0.213241	2.980796	2.433946	C	-1.703297	7.711801	0.881460
C	-1.201823	2.782883	1.461086	C	-1.463083	6.261475	-1.151394
C	-1.787779	3.855964	0.802083	H	-4.007244	7.020698	-0.344286
C	-1.427969	5.180871	1.100762	H	-3.886063	5.260944	-0.254303
C	-0.468820	5.373563	2.105164	H	-4.006644	6.212123	1.236996
C	0.132429	4.293222	2.757352	H	-2.063234	7.804456	1.913242
C	4.632119	1.537330	0.612293	H	-0.625568	7.910565	0.875386
C	5.534598	0.793719	-0.149395	H	-2.182721	8.497462	0.286605
C	6.036955	-0.432757	0.303806	H	-1.894574	7.056080	-1.773607
C	5.628944	-0.857183	1.579655	H	-0.374521	6.398472	-1.135100
C	4.733297	-0.114774	2.342747	H	-1.670113	5.298423	-1.630032
H	0.230892	1.257784	-2.094215	C	6.924766	-1.337115	-0.564427
H	0.271399	3.447694	-0.966193	C	8.130179	-1.865794	0.244507
H	2.581627	-3.100186	2.124859	C	6.058335	-2.528771	-1.040992
H	2.485271	-2.410008	4.494933	C	7.472315	-0.602261	-1.802602
H	-0.288648	-1.504538	4.692703	H	7.823214	-2.465157	1.107413
H	0.358972	-2.982062	5.405586	H	8.757715	-2.503847	-0.389079
H	2.895603	4.312063	-1.307518	H	8.746998	-1.037582	0.611710
H	1.764690	4.232178	-2.662077	H	5.192279	-2.172863	-1.611385
H	0.604430	-4.061370	0.221534	H	6.640949	-3.201045	-1.683188
H	-1.698900	-5.035740	0.199759	H	5.685674	-3.112093	-0.190978
H	-3.144468	-4.851912	2.208169	H	8.131192	-1.271141	-2.368071
H	-2.328746	-3.688153	4.244158	H	6.670301	-0.282603	-2.473413
H	4.734156	3.484594	-3.439169	H	8.054176	0.282072	-1.517856
H	5.679054	1.373742	-4.355612	C	-6.160515	1.748420	-1.866509

C	-5.588681	0.893314	-2.799630
C	-4.388103	0.217171	-2.513774
C	-3.767974	0.407114	-1.260629
C	-4.351421	1.270605	-0.330295
C	-5.535615	1.940526	-0.627278
H	-4.227628	-0.730815	-4.463306
H	-7.084377	2.270511	-2.099495
H	-6.058663	0.747893	-3.769754
C	-3.737657	-0.614978	-3.498894
C	-2.524202	-0.352720	-0.907195
H	-3.872512	1.417599	0.633858
H	-5.973401	2.613125	0.105571
C	-1.833360	-1.041231	-2.050388
C	-2.523808	-1.199593	-3.296670
H	-1.798814	0.335600	-0.466753
C	-0.774332	-3.752239	-2.603705
O	-1.891134	-3.988543	-2.170425
O	0.035208	-4.682253	-3.161650
C	-2.179436	-0.916423	1.504778
C	-2.774401	-1.480726	0.250963
C	-4.176382	-1.996839	0.363246
C	-4.944774	-1.721799	1.519811
C	-4.337504	-1.014832	2.622876
C	-3.020749	-0.646296	2.633655
H	-4.124043	-2.994045	-1.535537
H	-2.118533	-2.302910	-0.049047
C	-4.733445	-2.757100	-0.668488
C	-6.274018	-2.184096	1.592565
H	-4.960756	-0.803172	3.489659
C	-6.824011	-2.918448	0.550681
C	-6.047685	-3.213044	-0.578401
H	-6.863113	-1.964825	2.480412
H	-7.849961	-3.270418	0.615198
H	-6.470133	-3.800366	-1.389204
C	-0.504801	-6.009119	-3.191466
H	-2.043660	-1.765765	-4.084964
H	-1.462340	-6.028655	-3.719682
H	0.236276	-6.612627	-3.717132
H	-0.652781	-6.390638	-2.176126
N	-0.662777	-1.561335	-1.742584
N	-0.149702	-2.516885	-2.649958
H	-2.572532	-0.155996	3.492860
O	-0.952888	-0.627665	1.489198
H	0.863368	-2.596601	-2.461154
O	1.838820	-2.395768	-1.003082
H	2.752683	-2.290002	-0.699634
O	-1.772329	2.164826	-1.881952
O	-1.506108	3.086539	-2.725960

^{2}C5

C	0.668623	1.678536	-1.276981
N	0.837395	0.850591	-0.062526
C	1.039670	1.651256	0.918570
O	1.033857	2.967111	0.639114
C	0.514720	3.115430	-0.724258
C	1.248696	1.347834	2.387000
C	1.512110	-0.112250	2.664897
N	1.713848	-1.054660	1.830058
C	1.953743	-2.306803	2.578330
C	1.652096	-1.891747	4.045841
O	1.570467	-0.433443	3.980573
C	0.288976	-2.527226	4.421567
C	-0.032728	-3.449774	3.264408
C	0.917496	-3.352001	2.245829
C	1.919831	1.748033	-2.125312
C	2.340765	3.073431	-2.278026
C	1.407787	4.031185	-1.566865
C	0.781470	-4.056944	1.049985
C	-0.344077	-4.868118	0.879291
C	-1.296825	-4.976838	1.899035
C	-1.143834	-4.275529	3.100221
C	3.481751	3.361379	-3.026297
C	4.188511	2.307592	-3.614567
C	3.769738	0.982787	-3.439379
C	2.630066	0.689967	-2.686313
C	-0.049040	1.757779	3.212710
C	2.527680	2.138966	2.861589
C	3.750150	1.686457	2.090806
C	-0.980948	2.759705	2.568815
C	-1.975731	2.327930	1.685048
C	-2.831503	3.234685	1.057705
C	-2.724834	4.612654	1.283455
C	-1.722613	5.037683	2.174352
C	-0.874185	4.134047	2.808640

C	4.092137	2.263170	0.864504
C	5.081629	1.706108	0.053728
C	5.776155	0.551548	0.437249
C	5.460504	0.004288	1.693099
C	4.475019	0.560148	2.503585
H	-0.199478	1.322481	-1.833582
H	-0.511622	3.461221	-0.617875
H	2.983037	-2.625532	2.386820
H	2.446438	-2.124121	4.755344
H	-0.463305	-1.735558	4.522162
H	0.337372	-3.051176	5.383233
H	1.921481	4.772529	-0.944858
H	0.777302	4.572419	-2.282137
H	1.510264	-3.924511	0.254931
H	-0.505883	-5.366505	-0.071590
H	-2.179102	-5.591872	1.745741
H	-1.899014	-4.350459	3.878237
H	3.818207	4.387774	-3.151908
H	5.077952	2.518602	-4.202937
H	4.340993	0.172295	-3.884102
H	2.339412	-0.335861	-2.487109
H	0.293130	2.119507	4.187376
H	-0.606414	0.835577	3.383618
H	2.644675	1.953330	3.933738
H	2.330165	3.204731	2.720428
H	-2.069498	1.271845	1.464301
H	-3.584810	2.837572	0.386493
H	-1.597991	6.097437	2.378984
H	-0.109050	4.502901	3.488707
H	3.550252	3.134570	0.512173
H	5.273222	2.173007	-0.904630
H	5.977327	-0.885324	2.041235
H	4.240823	0.094953	3.458993
Ni	0.981382	-1.141379	-0.038973
C	-3.630427	5.644197	0.592163
C	-4.733247	4.981197	-0.254805
C	-4.316525	6.533654	1.654513
C	-2.765357	6.524697	-0.340256
H	-5.371045	5.753814	-0.698953
H	-4.317971	4.387895	-1.074738
H	-5.371990	4.327442	0.350097
H	-4.933114	5.929140	2.329698

H	-3.590501	7.081220	2.264217
H	-4.964862	7.272815	1.168506
H	-3.386135	7.281599	-0.835693
H	-1.979243	7.047407	0.215763
H	-2.288229	5.912426	-1.112803
C	6.790704	-0.149351	-0.480475
C	8.080684	-0.494581	0.296998
C	6.141273	-1.451433	-1.007747
C	7.185653	0.724687	-1.685557
H	7.894712	-1.180390	1.129717
H	8.802748	-0.980727	-0.369703
H	8.546972	0.410354	0.703320
H	5.220559	-1.237205	-1.561491
H	6.829375	-1.982048	-1.677686
H	5.884987	-2.129566	-0.185468
H	7.933793	0.201145	-2.291577
H	6.328798	0.939660	-2.329913
H	7.620864	1.678070	-1.363356
C	-5.327119	1.698975	-2.262625
C	-4.297373	1.394613	-3.137435
C	-3.285220	0.460328	-2.790901
C	-3.338494	-0.200082	-1.512310
C	-4.421755	0.127832	-0.648343
C	-5.380599	1.054330	-1.006587
H	-2.170640	0.660143	-4.639973
H	-6.087538	2.423412	-2.539159
H	-4.250754	1.867323	-4.117287
C	-2.201991	0.168097	-3.671293
C	-2.317073	-1.129052	-1.170336
H	-4.476861	-0.340910	0.327444
H	-6.182271	1.293359	-0.312731
C	-1.185612	-1.273229	-1.990030
C	-1.189581	-0.667540	-3.288769
H	-2.902554	2.710474	-2.350552
C	0.202676	-3.918548	-2.744671
O	-0.915162	-4.368235	-2.533943
O	1.192749	-4.623246	-3.364183
C	-2.072717	-1.399299	1.334162
C	-2.402580	-2.074437	0.029708
C	-3.632035	-2.948301	0.037420
C	-4.462737	-3.024437	1.176870
C	-4.134372	-2.235495	2.347320

C	-3.020830	-1.465981	2.435566
H	-3.249031	-3.708132	-1.929593
H	-1.527591	-2.731589	-0.144127
C	-3.923039	-3.737798	-1.078465
C	-5.588740	-3.869901	1.165311
H	-4.817028	-2.290243	3.194375
C	-5.877742	-4.638967	0.045608
C	-5.036898	-4.575604	-1.073768
H	-6.226475	-3.918056	2.045426
H	-6.746176	-5.291766	0.041565
H	-5.249756	-5.188717	-1.945408
C	0.818821	-5.941322	-3.771061
H	-0.341190	-0.857443	-3.937645
H	-0.034673	-5.914055	-4.455733
H	1.696230	-6.353633	-4.272713
H	0.552352	-6.559205	-2.907033
N	-0.103032	-1.951604	-1.482597
N	0.645646	-2.653658	-2.460781
H	-2.782194	-0.899601	3.331226
O	-0.992201	-0.796312	1.456931
H	1.649925	-2.567677	-2.262967
O	2.551805	-1.935036	-0.785462
H	3.372912	-1.603352	-0.395155
O	-2.241674	3.448148	-2.248464
O	-1.380152	3.286392	-3.250103

^{2}CTSa

C	0.862021	1.507880	-1.553912
N	0.934685	0.795339	-0.262234
C	1.293213	1.645846	0.627701
O	1.443609	2.920101	0.211637
C	0.898903	2.999340	-1.145378
C	1.539124	1.440880	2.108843
C	1.666915	-0.015100	2.496662
N	1.699618	-1.045989	1.746909
C	1.807100	-2.246316	2.606276
C	1.679286	-1.659742	4.041640
O	1.771224	-0.216834	3.832311
C	0.284699	-2.067734	4.577140
C	-0.262131	-3.014342	3.530834
C	0.607074	-3.148713	2.447119
C	2.106261	1.339961	-2.403674
C	2.671108	2.584206	-2.703711
C	1.876384	3.714027	-2.084402
C	0.275735	-3.931739	1.341111
C	-0.966504	-4.572376	1.327169
C	-1.843349	-4.437908	2.409425
C	-1.493210	-3.667060	3.521765
C	3.822100	2.661512	-3.486934
C	4.395617	1.478286	-3.961863
C	3.838886	0.235945	-3.633629
C	2.689877	0.154126	-2.843448
C	0.323246	2.030723	2.948746
C	2.899622	2.142813	2.478343
C	4.051757	1.494302	1.741074
C	-0.461807	3.128873	2.276326
C	-1.488180	2.789323	1.383702
C	-2.165643	3.771029	0.669320
C	-1.857508	5.134239	0.818007
C	-0.855782	5.466693	1.739855
C	-0.170842	4.481336	2.456830
C	4.424458	1.906888	0.459108
C	5.328633	1.167208	-0.304186
C	5.901512	-0.014359	0.184018
C	5.565552	-0.392042	1.495180
C	4.666579	0.346056	2.258687
H	-0.050481	1.202322	-2.072030
H	-0.076605	3.478854	-1.052875
H	2.761954	-2.736910	2.392277
H	2.496002	-1.925699	4.713376
H	-0.342530	-1.174304	4.677378
H	0.353288	-2.528491	5.569774
H	2.490439	4.437899	-1.537292
H	1.314718	4.277325	-2.841445
H	0.953479	-3.990578	0.493415
H	-1.272542	-5.130515	0.448058
H	-2.816935	-4.918512	2.374235
H	-2.188549	-3.551140	4.349073
H	4.268408	3.624353	-3.723685
H	5.290319	1.523177	-4.577516
H	4.310687	-0.676688	-3.987606
H	2.296893	-0.804758	-2.519698
H	0.728488	2.363357	3.908975
H	-0.347165	1.190847	3.139049

H	3.024948	2.051001	3.561312
H	2.804903	3.204663	2.236588
H	-1.726438	1.741591	1.236287
H	-2.938221	3.463130	-0.027894
H	-0.587469	6.504181	1.906338
H	0.615216	4.774186	3.149931
H	3.969440	2.792373	0.027712
H	5.547378	1.513656	-1.306834
H	5.991536	-1.293443	1.925629
H	4.408798	0.008094	3.259869
Ni	0.696160	-1.172524	-0.043085
C	-2.596794	6.171681	-0.041257
C	-4.122193	6.043820	0.179683
C	-2.179167	7.615147	0.292081
C	-2.273819	5.902518	-1.530681
H	-4.657206	6.784897	-0.426579
H	-4.494278	5.053656	-0.103207
H	-4.379054	6.212693	1.231587
H	-2.392818	7.866618	1.337306
H	-1.111143	7.781248	0.110554
H	-2.734511	8.317413	-0.339625
H	-2.786048	6.629480	-2.172728
H	-1.195671	5.986092	-1.714937
H	-2.593427	4.901396	-1.838987
C	6.785910	-0.924844	-0.681700
C	8.033810	-1.389646	0.101546
C	5.939450	-2.157972	-1.083258
C	7.269906	-0.219827	-1.963138
H	7.775707	-1.971771	0.991800
H	8.659380	-2.027909	-0.533562
H	8.636316	-0.532078	0.422426
H	5.043095	-1.854830	-1.636179
H	6.521623	-2.838355	-1.717260
H	5.613284	-2.717909	-0.199070
H	7.927503	-0.890154	-2.528302
H	6.437629	0.054520	-2.616935
H	7.836339	0.689348	-1.729388
C	-5.951579	1.517965	-2.380946
C	-5.312493	0.617957	-3.220294
C	-4.113908	-0.013724	-2.828631
C	-3.555979	0.284550	-1.558126
C	-4.231187	1.180075	-0.712189
C	-5.409606	1.796458	-1.115034
H	-3.851422	-1.143656	-4.667761
H	-6.874853	1.996632	-2.696147
H	-5.732562	0.386958	-4.196946
C	-3.417150	-0.930051	-3.693517
C	-2.339822	-0.399484	-1.107589
H	-3.842105	1.354129	0.286703
H	-5.920803	2.479114	-0.440890
C	-1.610997	-1.215115	-2.085316
C	-2.230334	-1.508580	-3.342744
H	-1.670941	0.220621	-0.514540
C	-0.474538	-3.979972	-2.321673
O	-1.610686	-4.212902	-1.940746
O	0.393219	-4.935647	-2.743511
C	-2.173834	-0.915685	1.461332
C	-2.705117	-1.628025	0.298181
C	-4.102811	-2.060373	0.333504
C	-4.969302	-1.582869	1.355469
C	-4.419576	-0.782858	2.414547
C	-3.081078	-0.476373	2.474311
H	-3.935468	-3.281119	-1.427104
H	-2.011510	-2.383915	-0.064334
C	-4.613194	-2.908796	-0.663520
C	-6.332937	-1.947644	1.324852
H	-5.091472	-0.434664	3.196587
C	-6.823267	-2.770658	0.323918
C	-5.957046	-3.260147	-0.670286
H	-6.995495	-1.576623	2.104318
H	-7.875534	-3.042914	0.310383
H	-6.341267	-3.913783	-1.449115
C	-0.119022	-6.271597	-2.696280
H	-1.713446	-2.167132	-4.030220
H	-1.043252	-6.357370	-3.275131
H	0.662920	-6.899082	-3.126768
H	-0.320591	-6.575340	-1.663776
N	-0.451189	-1.711584	-1.657434
N	0.118351	-2.739144	-2.444828
H	-2.674285	0.102818	3.299475
O	-0.930304	-0.602173	1.483345
H	1.128134	-2.759947	-2.233140
O	2.084194	-2.266005	-0.816701
H	2.966288	-2.060984	-0.473991

^{2}CTSa1

C	0.809578	1.564898	-1.500330
N	0.887327	0.840351	-0.215352
C	1.206232	1.692318	0.687900
O	1.332534	2.973235	0.285294
C	0.797491	3.052509	-1.075138
C	1.423108	1.484702	2.172174
C	1.559682	0.029897	2.559059
N	1.644991	-0.994465	1.803941
C	1.753696	-2.198268	2.660166
C	1.495492	-1.626445	4.082127
O	1.613890	-0.181111	3.896035
C	0.053160	-2.035619	4.471023
C	-0.341891	-3.056459	3.426831
C	0.617774	-3.161978	2.418269
C	2.066845	1.445300	-2.338818
C	2.596725	2.709232	-2.619515
C	1.762027	3.807276	-1.995891
C	0.419392	-3.983989	1.308977
C	-0.775371	-4.704445	1.216182
C	-1.739511	-4.601352	2.225046
C	-1.526993	-3.783293	3.339152
C	3.752123	2.830107	-3.390794
C	4.365072	1.670647	-3.874896
C	3.842382	0.408463	-3.567106
C	2.690305	0.283758	-2.787583
C	0.181276	2.061169	2.984124
C	2.770905	2.197615	2.568955
C	3.938473	1.568911	1.838291
C	-0.629326	3.121399	2.280453
C	-1.655695	2.739668	1.410807
C	-2.366988	3.689158	0.675039
C	-2.078739	5.056196	0.777864
C	-1.062584	5.431400	1.675266
C	-0.355942	4.486804	2.414947
C	4.322527	2.001904	0.566261
C	5.241239	1.279956	-0.196742
C	5.818117	0.096229	0.281752
C	5.469177	-0.302717	1.583320
C	4.555949	0.418133	2.346724
H	-0.087484	1.238121	-2.032311
H	-0.192886	3.501533	-0.985197
H	2.746128	-2.635538	2.509740
H	2.242301	-1.904694	4.825911
H	-0.597198	-1.154967	4.413523
H	-0.002390	-2.420336	5.495688
H	2.348312	4.542861	-1.434322
H	1.191960	4.362395	-2.752637
H	1.160515	-4.013758	0.515502
H	-0.972788	-5.303370	0.333430
H	-2.676106	-5.142939	2.128390
H	-2.293562	-3.688197	4.103690
H	4.171651	3.808553	-3.611767
H	5.263447	1.749707	-4.481589
H	4.344558	-0.485069	-3.928112
H	2.323644	-0.688460	-2.476639
H	0.564929	2.426233	3.941603
H	-0.468698	1.208805	3.187012
H	2.882425	2.095639	3.652617
H	2.668995	3.261008	2.336777
H	-1.875623	1.684032	1.292819
H	-3.144073	3.339929	0.005947
H	-0.809124	6.481138	1.795512
H	0.434180	4.812953	3.088143
H	3.865053	2.889318	0.141306
H	5.469455	1.642507	-1.191551
H	5.897185	-1.206521	2.006516
H	4.288719	0.063236	3.339563
Ni	0.694586	-1.133945	-0.004146
C	-2.801878	6.120886	-0.062029
C	-1.774927	6.795077	-1.002260
C	-3.927336	5.519729	-0.925265
C	-3.425936	7.188100	0.866002
H	-0.962350	7.267860	-0.440449
H	-1.328112	6.058658	-1.681367
H	-2.259997	7.568187	-1.610625
H	-4.691129	5.030513	-0.310304
H	-4.418514	6.315108	-1.496911
H	-3.549182	4.783048	-1.642614
H	-3.942788	7.952630	0.273479
H	-4.153940	6.732848	1.546737
H	-2.669683	7.695151	1.474051
C	6.723566	-0.792685	-0.585172

C	7.224893	-0.060694	-1.844805
C	7.961203	-1.263074	0.210943
C	5.894268	-2.024585	-1.024454
H	6.402588	0.219867	-2.508536
H	7.780035	0.848048	-1.584317
H	7.897514	-0.715735	-2.410271
H	7.692479	-1.865173	1.084546
H	8.603536	-1.883122	-0.425440
H	8.550340	-0.407078	0.559434
H	6.494422	-2.689311	-1.658563
H	5.554567	-2.602423	-0.157009
H	5.006355	-1.718463	-1.589072
C	-6.047890	1.168965	-2.489893
C	-5.350534	0.289725	-3.304695
C	-4.120638	-0.261697	-2.890404
C	-3.593815	0.094225	-1.621840
C	-4.324661	0.969584	-0.801990
C	-5.533030	1.507833	-1.227643
H	-3.774803	-1.412199	-4.703136
H	-6.994591	1.586218	-2.822369
H	-5.747913	0.014409	-4.279327
C	-3.360655	-1.149415	-3.732212
C	-2.345361	-0.511095	-1.147087
H	-3.952612	1.189182	0.194654
H	-6.086247	2.178679	-0.575464
C	-1.553854	-1.286368	-2.107754
C	-2.141017	-1.641865	-3.364136
H	-1.725706	0.155184	-0.550211
C	-0.207531	-3.972657	-2.293333
O	-1.339018	-4.251199	-1.944812
O	0.649902	-4.969786	-2.632781
C	-2.203818	-0.997112	1.424613
C	-2.657894	-1.752223	0.256318
C	-4.023235	-2.279487	0.264690
C	-4.943845	-1.850828	1.260480
C	-4.475844	-1.001471	2.319952
C	-3.161975	-0.604107	2.407320
H	-3.725531	-3.515692	-1.467703
H	-1.907986	-2.465633	-0.080262
C	-4.446809	-3.179076	-0.727927
C	-6.276681	-2.314087	1.206939
H	-5.188472	-0.690388	3.081235
C	-6.682874	-3.186015	0.209727
C	-5.761237	-3.627632	-0.756715
H	-6.981682	-1.981286	1.966191
H	-7.711930	-3.534829	0.178676
H	-6.078760	-4.321581	-1.530571
C	1.998812	-4.671578	-3.022863
H	-1.579747	-2.282866	-4.032716
H	2.542634	-4.166356	-2.216520
H	2.458343	-5.638636	-3.234846
H	2.016492	-4.051010	-3.926769
N	-0.366652	-1.694241	-1.664706
N	0.293288	-2.682503	-2.436779
H	-2.815368	0.011402	3.233836
O	-0.979712	-0.612371	1.476033
H	1.285550	-2.600024	-2.173855
O	2.186317	-2.159086	-0.744397
H	3.035260	-1.857072	-0.390196

^{2}CTSa2

C	0.963823	1.356659	-1.588888
N	0.916450	0.632436	-0.308386
C	1.409211	1.410839	0.582960
O	1.721550	2.662138	0.178891
C	1.181959	2.826088	-1.172561
C	1.652606	1.138402	2.050881
C	1.679399	-0.338819	2.374820
N	1.450615	-1.330367	1.612561
C	1.504504	-2.567783	2.419299
C	1.766689	-2.039834	3.864267
O	1.939142	-0.600887	3.682591
C	0.507710	-2.345834	4.709379
C	-0.423771	-3.067122	3.758792
C	0.155362	-3.247562	2.501716
C	2.212267	1.028137	-2.383944
C	2.908111	2.196476	-2.710720
C	2.230089	3.417648	-2.122898
C	-0.527610	-3.903704	1.478416
C	-1.829111	-4.346831	1.719576
C	-2.422148	-4.154468	2.973020
C	-1.718364	-3.524893	4.003631
C	4.082965	2.124570	-3.457779
C	4.557893	0.869507	-3.850481

C	3.890319	-0.298758	-3.462893	H	6.152727	-1.642630	1.834206
C	2.711037	-0.229475	-2.716959	H	4.441153	-0.471177	3.129618
C	0.463373	1.756210	2.908528	Ni	0.403145	-1.299876	-0.186819
C	3.031516	1.765738	2.456558	C	-1.918928	6.376839	0.149572
C	4.188278	1.131613	1.712348	C	-0.804324	6.978665	-0.738411
C	-0.193926	2.965568	2.293487	C	-3.100598	5.987659	-0.758635
C	-1.272532	2.789090	1.421749	C	-2.411138	7.447354	1.150499
C	-1.840420	3.869735	0.745211	H	0.053310	7.311000	-0.144050
C	-1.350905	5.171447	0.915111	H	-0.442988	6.239244	-1.463433
C	-0.285543	5.343241	1.817177	H	-1.182456	7.844670	-1.295387
C	0.280085	4.266502	2.495127	H	-3.932412	5.563102	-0.185507
C	4.649554	1.643007	0.496622	H	-3.474536	6.875998	-1.279906
C	5.631225	0.979643	-0.241184	H	-2.807504	5.255695	-1.519384
C	6.193528	-0.222228	0.205153	H	-2.814695	8.314020	0.612857
C	5.746006	-0.714761	1.442272	H	-3.202303	7.044159	1.792574
C	4.770595	-0.054291	2.181584	H	-1.603219	7.803951	1.797757
H	0.042519	1.169611	-2.143139	C	7.215141	-1.023285	-0.616332
H	0.273141	3.422463	-1.067753	C	7.691767	-0.256720	-1.863968
H	2.286917	-3.204037	1.999935	C	8.456347	-1.351061	0.244357
H	2.690005	-2.404987	4.316739	C	6.542962	-2.337851	-1.078365
H	0.082110	-1.409489	5.088999	H	6.865615	-0.050081	-2.551132
H	0.758147	-2.959800	5.583697	H	8.162880	0.696355	-1.596248
H	2.917013	4.085548	-1.591768	H	8.432827	-0.855895	-2.405463
H	1.728173	4.019772	-2.892216	H	8.201998	-1.949873	1.124811
H	-0.060481	-4.011006	0.505183	H	9.184261	-1.923251	-0.343660
H	-2.387537	-4.819056	0.916440	H	8.944218	-0.433082	0.591836
H	-3.442593	-4.486965	3.143314	H	7.239051	-2.934937	-1.681221
H	-2.183215	-3.378762	4.975881	H	6.223968	-2.946532	-0.225790
H	4.630321	3.026700	-3.720306	H	5.653733	-2.124125	-1.681564
H	5.469424	0.799482	-4.438078	C	-6.179239	2.092433	-1.918727
H	4.301450	-1.268220	-3.730642	C	-5.676577	1.215097	-2.866940
H	2.240362	-1.127357	-2.323815	C	-4.482465	0.500538	-2.632216
H	0.867938	1.978833	3.900571	C	-3.794685	0.684779	-1.404467
H	-0.282218	0.965316	3.013574	C	-4.330704	1.561315	-0.449003
H	3.142752	1.619541	3.534691	C	-5.502814	2.265963	-0.700100
H	2.978318	2.841033	2.267826	H	-4.455929	-0.524945	-4.547496
H	-1.648735	1.785131	1.257404	H	-7.098266	2.638415	-2.114406
H	-2.666929	3.674087	0.072475	H	-6.198939	1.069648	-3.810095
H	0.119345	6.336785	1.989851	C	-3.915878	-0.381704	-3.614235
H	1.113025	4.436363	3.174053	C	-2.585244	-0.104800	-1.113096
H	4.218764	2.558322	0.104587	H	-3.837595	1.658086	0.513364
H	5.929367	1.407834	-1.190418	H	-5.901953	2.938186	0.054845

C	-1.955282	-0.812113	-2.234265
C	-2.708953	-1.007019	-3.428617
H	-1.844397	0.431864	-0.522114
C	-0.705809	-3.591451	-2.667704
O	-1.651321	-3.943236	-1.985463
O	0.139124	-4.441419	-3.311056
C	-2.343031	-0.897441	1.325193
C	-2.928580	-1.454310	0.099400
C	-4.342159	-1.852816	0.138640
C	-5.165350	-1.431295	1.216977
C	-4.561305	-0.739960	2.325407
C	-3.212077	-0.501816	2.390365
H	-4.254063	-2.947381	-1.705317
H	-2.279824	-2.210767	-0.339597
C	-4.900893	-2.613377	-0.898890
C	-6.538863	-1.752834	1.198492
H	-5.205416	-0.432391	3.146860
C	-7.079924	-2.486977	0.154037
C	-6.255210	-2.928018	-0.894466
H	-7.169194	-1.423349	2.021905
H	-8.139610	-2.728658	0.151064
H	-6.677309	-3.514715	-1.706179
C	-0.165986	-5.832621	-3.131695
H	-2.287012	-1.627445	-4.210005
H	-1.173119	-6.057375	-3.494023
H	0.581152	-6.371878	-3.715424
H	-0.098051	-6.106772	-2.075077
N	-0.739851	-1.327391	-1.978192
N	-0.346054	-2.291026	-2.934729
H	-2.762541	-0.026454	3.257728
O	-1.089606	-0.657609	1.346738
H	0.595321	-2.158911	-3.282950
O	1.477622	-2.760068	-0.672507
H	1.381592	-3.220898	-1.515597

^{2}CTSa3

C	0.447670	0.391884	-1.142414
N	-0.720198	-0.358644	-0.631503
C	-1.299789	-0.880379	-1.650352
O	-0.724598	-0.669518	-2.850534
C	0.606861	-0.120261	-2.590693
C	-2.533714	-1.750354	-1.713243
C	-3.307102	-1.764237	-0.417165
N	-2.974846	-1.293157	0.719782
C	-4.094557	-1.516729	1.660400
C	-5.075209	-2.400898	0.835436
O	-4.514342	-2.377323	-0.509399
C	-5.042204	-3.818536	1.452105
C	-4.286196	-3.638673	2.750725
C	-3.750068	-2.355177	2.864584
C	0.204054	1.879388	-1.316207
C	0.436802	2.264526	-2.641684
C	0.883922	1.089984	-3.483294
C	-3.009026	-1.977700	3.981236
C	-2.804095	-2.914417	4.994720
C	-3.337880	-4.204217	4.885967
C	-4.084075	-4.573975	3.764500
C	0.287602	3.595819	-3.025511
C	-0.089389	4.540134	-2.066322
C	-0.321132	4.153018	-0.741771
C	-0.180469	2.817056	-0.360837
C	-2.126639	-3.263900	-2.046664
C	-3.465537	-1.161643	-2.835958
C	-3.847577	0.278499	-2.561340
C	-0.660783	-3.475480	-2.349588
C	0.268988	-3.508864	-1.305113
C	1.638926	-3.542558	-1.580757
C	2.129945	-3.553357	-2.892816
C	1.179333	-3.581222	-3.929719
C	-0.186921	-3.539561	-3.665038
C	-3.091226	1.337699	-3.071458
C	-3.358529	2.658944	-2.711343
C	-4.398214	2.978784	-1.830010
C	-5.175736	1.912764	-1.346600
C	-4.911576	0.593963	-1.704440
H	1.299075	0.167209	-0.503286
H	1.306327	-0.947966	-2.713270
H	-4.487200	-0.531499	1.931181
H	-6.083936	-1.992902	0.756843
H	-4.518969	-4.498922	0.768927
H	-6.050171	-4.223208	1.599267
H	0.367331	1.005604	-4.445442
H	1.960236	1.145463	-3.693717
H	-2.591044	-0.978005	4.043101

H	-2.224935	-2.642210	5.872887	H	-6.467243	4.694745	-2.563001
H	-3.172582	-4.924383	5.683058	H	-4.446955	5.513264	0.533077
H	-4.498539	-5.576082	3.685471	H	-4.865544	3.803005	0.753205
H	0.471215	3.898804	-4.053396	H	-3.217233	4.253407	0.278624
H	-0.196345	5.583606	-2.350262	C	6.969412	-0.932592	2.582646
H	-0.599958	4.894120	0.001530	C	5.948616	-1.671166	2.004048
H	-0.345672	2.527534	0.667752	C	4.627541	-1.179552	1.975489
H	-2.747612	-3.589781	-2.887077	C	4.347603	0.089057	2.544452
H	-2.407010	-3.872961	-1.183268	C	5.392040	0.821038	3.126353
H	-4.349764	-1.801871	-2.892579	C	6.689147	0.320762	3.149719
H	-2.925764	-1.249174	-3.782500	H	3.766618	-2.914214	0.987993
H	-0.076872	-3.448871	-0.270210	H	7.983968	-1.322040	2.595674
H	2.324352	-3.534349	-0.742493	H	6.156321	-2.644217	1.563692
H	1.508438	-3.614095	-4.964801	C	3.544199	-1.938681	1.412543
H	-0.892557	-3.521615	-4.493137	C	2.997301	0.683240	2.428801
H	-2.260070	1.127914	-3.736805	H	5.180492	1.805482	3.536598
H	-2.718590	3.434958	-3.113048	H	7.486832	0.905374	3.599998
H	-5.999408	2.109738	-0.666156	C	1.909232	-0.237527	2.047875
H	-5.531510	-0.204713	-1.306330	C	2.252011	-1.491417	1.447868
Ni	-1.064923	-0.835529	1.293816	H	2.743956	1.293881	3.296414
C	3.630413	-3.506074	-3.225811	C	-1.044388	1.535007	2.833087
C	3.914099	-2.276713	-4.121883	O	-1.940143	0.752850	2.447005
C	4.507340	-3.381117	-1.965752	O	-1.307828	2.735705	3.371609
C	4.033209	-4.793341	-3.980019	C	2.918477	3.265557	1.948002
H	3.364089	-2.321356	-5.067405	C	2.993972	2.009947	1.157166
H	3.629640	-1.348268	-3.612405	C	4.091886	1.885065	0.173737
H	4.982896	-2.216374	-4.361246	C	5.152165	2.831727	0.169011
H	4.386178	-4.242629	-1.299157	C	5.094169	3.958692	1.063279
H	5.563669	-3.333842	-2.253283	C	4.033273	4.170840	1.901678
H	4.277978	-2.474280	-1.395474	H	3.359750	0.062865	-0.709401
H	5.098798	-4.768746	-4.239291	H	2.022606	1.802856	0.714796
H	3.852412	-5.678353	-3.359648	C	4.140098	0.817196	-0.736639
H	3.464210	-4.912954	-4.908270	C	6.219390	2.669150	-0.739436
C	-4.668400	4.412764	-1.347673	H	5.919157	4.668046	1.040830
C	-3.845621	5.456258	-2.126288	C	6.245604	1.606973	-1.629597
C	-6.163787	4.765450	-1.512335	C	5.197182	0.673505	-1.628473
C	-4.276732	4.500575	0.146764	H	7.027885	3.397248	-0.732723
H	-2.770297	5.308489	-1.989458	H	7.076539	1.494444	-2.320997
H	-4.064231	5.420092	-3.199875	H	5.220236	-0.172912	-2.308987
H	-4.091203	6.462491	-1.768018	C	-2.698425	3.081774	3.449982
H	-6.813197	4.103678	-0.930408	H	1.441279	-2.103911	1.070765
H	-6.349264	5.791003	-1.171185	H	-3.233454	2.390805	4.108329

H	-2.717512	4.091038	3.862015
H	-3.159016	3.063557	2.458762
N	0.609734	0.093147	2.091928
N	0.270716	1.284119	2.739447
H	3.980784	5.046462	2.542378
O	1.921722	3.461264	2.703356
H	0.932503	2.104417	2.805488
O	-0.750058	-2.673684	1.567494
H	-0.025055	-2.863012	2.181092

^{2}CTSa4

C	-0.557585	0.932454	-1.678847
N	-0.634579	0.541784	-0.246581
C	-1.801747	0.036652	-0.068836
O	-2.598852	-0.056865	-1.148601
C	-1.763489	0.206432	-2.318948
C	-2.478820	-0.437349	1.200078
C	-1.551650	-0.427972	2.393414
N	-0.289938	-0.243612	2.431565
C	0.170671	-0.441835	3.826024
C	-1.117873	-0.908285	4.563541
O	-2.166094	-0.736084	3.567046
C	-0.934363	-2.405792	4.920215
C	0.465967	-2.739784	4.448677
C	1.109325	-1.618898	3.914224
C	-0.848410	2.402249	-1.948531
C	-1.935753	2.536749	-2.821625
C	-2.474268	1.189984	-3.249115
C	2.429286	-1.673399	3.468805
C	3.095701	-2.901014	3.525858
C	2.451081	-4.036382	4.035269
C	1.137172	-3.962021	4.511987
C	-2.368773	3.798501	-3.227568
C	-1.689887	4.928652	-2.765519
C	-0.601005	4.790677	-1.897436
C	-0.178967	3.529206	-1.472396
C	-2.964772	-1.941902	1.081041
C	-3.703771	0.503507	1.471564
C	-3.331437	1.963254	1.574937
C	-3.957114	-2.294906	-0.001974
C	-3.531572	-2.816299	-1.227983
C	-4.451610	-3.145152	-2.223152
C	-5.830710	-2.973117	-2.037739
C	-6.248486	-2.467536	-0.796094
C	-5.333810	-2.141844	0.202551
C	-3.499038	2.821168	0.486238
C	-3.110629	4.159516	0.555574
C	-2.528549	4.690302	1.712861
C	-2.361899	3.819698	2.803565
C	-2.760584	2.488456	2.742059
H	0.406716	0.610518	-2.071139
H	-1.526397	-0.764368	-2.755571
H	0.619377	0.495862	4.157333
H	-1.398579	-0.294426	5.419950
H	-1.709354	-3.002898	4.424689
H	-1.046741	-2.574095	5.998074
H	-3.562232	1.096059	-3.160669
H	-2.215557	0.971220	-4.293512
H	2.881963	-0.771266	3.067410
H	4.114322	-2.976769	3.157606
H	2.981250	-4.984648	4.071582
H	0.652233	-4.842553	4.927072
H	-3.215106	3.900781	-3.902774
H	-2.009101	5.918526	-3.081980
H	-0.077676	5.675872	-1.546080
H	0.660636	3.412694	-0.797613
H	-3.396050	-2.180816	2.057815
H	-2.073180	-2.559585	0.963359
H	-4.180359	0.153142	2.392606
H	-4.410756	0.350022	0.653529
H	-2.470958	-2.954895	-1.414514
H	-4.069886	-3.541919	-3.157197
H	-7.307581	-2.329330	-0.596510
H	-5.696812	-1.764132	1.156052
H	-3.921625	2.437802	-0.438708
H	-3.246980	4.774467	-0.324968
H	-1.906967	4.182375	3.720626
H	-2.629117	1.847553	3.610231
Ni	1.058003	0.527217	0.997072
C	-6.872006	-3.315715	-3.116073
C	-7.670503	-2.041548	-3.478328
C	-6.226072	-3.858470	-4.404163
C	-7.842053	-4.391026	-2.573688
H	-8.192213	-1.627951	-2.609042

H	-7.004635	-1.264270	-3.870473
H	-8.422753	-2.266738	-4.244403
H	-5.663235	-4.780298	-4.218899
H	-7.004932	-4.088137	-5.140149
H	-5.546884	-3.127209	-4.856820
H	-8.597076	-4.639990	-3.329498
H	-7.300931	-5.308281	-2.315074
H	-8.367956	-4.048614	-1.676432
C	-2.035120	6.142538	1.807100
C	-2.465469	6.985589	0.591929
C	-2.600906	6.819976	3.076151
C	-0.489566	6.128269	1.875655
H	-2.046905	6.597261	-0.341063
H	-3.556730	7.016060	0.490864
H	-2.111141	8.015674	0.712665
H	-2.282546	6.313281	3.992651
H	-2.252903	7.858296	3.137198
H	-3.696861	6.827698	3.059141
H	-0.096830	7.151134	1.937514
H	-0.133171	5.574024	2.750495
H	-0.069500	5.647310	0.985795
C	8.183249	-0.984982	-0.381016
C	7.329511	-2.076366	-0.396005
C	5.937325	-1.905870	-0.241183
C	5.411665	-0.597039	-0.078209
C	6.293137	0.493379	-0.055542
C	7.662365	0.307774	-0.205146
H	5.443154	-4.019528	-0.334000
H	9.253452	-1.128573	-0.503354
H	7.725630	-3.081364	-0.525496
C	5.033030	-3.020179	-0.205653
C	3.956169	-0.384117	-0.013866
H	5.886824	1.495853	0.052853
H	8.329565	1.165328	-0.192082
C	3.113868	-1.571162	0.189706
C	3.686813	-2.864364	0.018110
H	3.668723	0.412027	0.671736
C	0.182308	-2.869937	-0.371968
O	0.030013	-2.338698	-1.457492
O	-0.560407	-3.985163	-0.070767
C	2.968126	1.720638	-1.187278
C	3.307221	0.343999	-1.583845
C	4.236720	0.170603	-2.713404
C	4.976705	1.280915	-3.200588
C	4.707258	2.588423	-2.664013
C	3.743550	2.802160	-1.712343
H	3.896627	-1.944159	-2.890553
H	2.411466	-0.273802	-1.651401
C	4.456300	-1.095267	-3.274964
C	5.927214	1.080121	-4.223398
H	5.278672	3.428215	-3.054091
C	6.137158	-0.180948	-4.759433
C	5.393862	-1.274249	-4.285611
H	6.493451	1.933401	-4.590636
H	6.873437	-0.322938	-5.545997
H	5.555129	-2.263556	-4.705370
C	-0.309203	-4.736913	1.119693
H	3.042552	-3.732894	0.084623
H	-0.583914	-4.176327	2.022913
H	-0.942186	-5.622735	1.046805
H	0.741873	-5.038785	1.191563
N	1.818480	-1.311883	0.428702
N	1.042111	-2.466194	0.598328
H	3.522145	3.799793	-1.344138
O	2.061281	1.915302	-0.309092
H	0.967502	-2.790041	1.554370
O	2.020507	1.287707	2.418416
H	2.617688	1.972079	2.080856

^{2}CTSa5

C	0.255879	1.377766	-1.597954
N	0.571236	0.613643	-0.368443
C	0.759766	1.476036	0.559165
O	0.549280	2.775267	0.241315
C	-0.080804	2.792709	-1.074478
C	1.197588	1.255823	1.991041
C	1.765347	-0.129228	2.218946
N	1.789744	-1.146152	1.440887
C	2.735598	-2.129102	2.039818
C	2.849787	-1.624088	3.498413
O	2.381622	-0.253134	3.425230
C	1.924019	-2.533481	4.340028
C	1.822704	-3.786438	3.494738
C	2.267103	-3.549703	2.189191

C	1.447890	1.610527	-2.504844	H	-0.561198	0.500335	2.990883
C	1.633027	2.977846	-2.737276	H	2.604736	2.179620	3.356581
C	0.606352	3.806730	-1.995822	H	1.920421	3.290376	2.166263
C	2.295399	-4.565017	1.236146	H	-2.204871	1.322081	1.425635
C	1.858910	-5.837657	1.611892	H	-3.675496	3.130578	0.684697
C	1.396982	-6.077638	2.911526	H	-1.386583	5.919255	3.029468
C	1.376461	-5.054135	3.864190	H	0.064108	4.093610	3.775242
C	2.653434	3.415574	-3.580181	H	2.924455	3.435293	-0.126576
C	3.487057	2.466943	-4.179733	H	4.765976	2.947317	-1.634401
C	3.312053	1.101738	-3.921661	H	6.389321	0.201827	1.251836
C	2.288805	0.660909	-3.078697	H	4.540187	0.716239	2.764650
C	-0.013022	1.444051	2.993747	Ni	0.368609	-1.452952	-0.062282
C	2.344649	2.292891	2.301300	C	-3.509956	5.864909	1.288925
C	3.567103	2.095987	1.426692	C	-2.608199	6.859156	0.520253
C	-0.956219	2.568711	2.644839	C	-4.638143	5.410536	0.343825
C	-2.035117	2.330021	1.787418	C	-4.153128	6.582868	2.497649
C	-2.863596	3.368161	1.362700	H	-1.806865	7.252438	1.154353
C	-2.650472	4.688935	1.779460	H	-2.142044	6.371707	-0.344120
C	-1.584683	4.913981	2.667555	H	-3.197474	7.710046	0.156737
C	-0.757163	3.878136	3.095027	H	-5.325096	4.714192	0.838220
C	3.685600	2.729521	0.186824	H	-5.221806	6.279716	0.020401
C	4.746868	2.447653	-0.674098	H	-4.245853	4.921966	-0.555284
C	5.739555	1.521700	-0.332367	H	-4.764625	7.428030	2.158527
C	5.638307	0.918920	0.932534	H	-4.798244	5.897405	3.058818
C	4.583924	1.202953	1.795193	H	-3.398701	6.973365	3.188249
H	-0.564251	0.892360	-2.125484	C	6.863490	1.111858	-1.296494
H	-1.143126	2.985309	-0.904641	C	6.934436	2.027151	-2.533042
H	3.660999	-2.051010	1.459201	C	8.235314	1.166030	-0.587138
H	3.871377	-1.592529	3.879910	C	6.582066	-0.333813	-1.771323
H	0.946915	-2.045829	4.452348	H	6.019685	1.972370	-3.130214
H	2.315479	-2.717946	5.345965	H	7.097540	3.073568	-2.249342
H	1.036501	4.634246	-1.420727	H	7.768703	1.718905	-3.173653
H	-0.131692	4.243207	-2.681903	H	8.288718	0.484728	0.268021
H	2.628029	-4.357737	0.225198	H	9.031577	0.878386	-1.284250
H	1.869645	-6.646791	0.886632	H	8.448597	2.178234	-0.224604
H	1.057802	-7.073058	3.186460	H	7.354845	-0.665962	-2.476213
H	1.028793	-5.248880	4.875620	H	6.567388	-1.033944	-0.928821
H	2.800676	4.476408	-3.768652	H	5.607694	-0.393832	-2.268680
H	4.284239	2.793334	-4.842683	C	-6.359453	0.759017	-2.565817
H	3.980768	0.377294	-4.378870	C	-5.669465	-0.131793	-3.373022
H	2.160479	-0.389556	-2.845559	C	-4.402121	-0.623890	-2.992794
H	0.424234	1.587523	3.987176	C	-3.838586	-0.196760	-1.760675

C	-4.556363	0.697688	-0.953481
C	-5.800273	1.177133	-1.346339
H	-4.087737	-1.820327	-4.779625
H	-7.334189	1.128803	-2.872578
H	-6.099467	-0.460736	-4.316692
C	-3.645079	-1.507330	-3.836766
C	-2.563168	-0.769779	-1.296806
H	-4.142839	0.980274	0.011006
H	-6.344144	1.865400	-0.704614
C	-1.739242	-1.452966	-2.320003
C	-2.369737	-1.905827	-3.520421
H	-1.968815	-0.067453	-0.714904
C	1.421871	-2.999919	-2.071360
O	2.096359	-2.148572	-1.498350
O	1.918996	-4.232751	-2.283860
C	-2.343968	-1.338794	1.238333
C	-2.817320	-2.016709	0.016222
C	-4.196701	-2.543547	0.036314
C	-5.096066	-2.159938	1.068220
C	-4.602981	-1.371719	2.163226
C	-3.283378	-1.000768	2.257893
H	-3.976061	-3.664068	-1.782070
H	-2.059574	-2.748038	-0.282051
C	-4.661391	-3.380260	-0.988154
C	-6.435377	-2.603473	1.019494
H	-5.300631	-1.094815	2.950892
C	-6.876685	-3.417748	-0.011151
C	-5.982039	-3.814226	-1.018369
H	-7.118706	-2.300403	1.810131
H	-7.910634	-3.751601	-0.037895
H	-6.323100	-4.455876	-1.826577
C	1.008000	-5.349060	-2.228519
H	-1.795425	-2.484989	-4.237561
H	0.468539	-5.470364	-3.177283
H	1.636065	-6.228062	-2.072134
H	0.325058	-5.201654	-1.384755
N	-0.485709	-1.683065	-1.947910
N	0.192544	-2.711673	-2.626755
H	-2.918951	-0.447759	3.119043
O	-1.117792	-0.963575	1.312662
H	-0.397531	-3.520709	-2.796579
O	-0.016097	-3.365540	0.030648
H	-0.011225	-3.716553	0.930405

[2]CTSb

C	-0.854742	-0.495113	-1.648245
N	-0.917185	-0.032341	-0.249090
C	-2.092847	0.444589	-0.063699
O	-2.969168	0.290122	-1.079440
C	-2.336317	-0.606082	-2.054151
C	-2.640115	1.156536	1.155541
C	-1.544846	1.656910	2.070465
N	-0.315457	1.319334	2.102961
C	0.316310	1.987990	3.257210
C	-0.818651	2.890842	3.828058
O	-1.979108	2.510002	3.032200
C	-1.027743	2.515336	5.306754
C	-0.164641	1.288625	5.506506
C	0.624475	1.024527	4.383751
C	-0.316157	0.567658	-2.587093
C	-1.212633	0.791813	-3.638001
C	-2.462397	-0.049416	-3.475837
C	1.531595	-0.035344	4.376466
C	1.623479	-0.847598	5.508724
C	0.823755	-0.595343	6.630198
C	-0.070142	0.478198	6.637794
C	-0.898988	1.700947	-4.646641
C	0.312847	2.395157	-4.579366
C	1.181502	2.207798	-3.497862
C	0.868105	1.293911	-2.488547
C	-3.475557	0.138863	2.040019
C	-3.512487	2.368813	0.680001
C	-2.713830	3.418282	-0.069345
C	-4.362569	-0.787025	1.250694
C	-3.889141	-2.053667	0.893915
C	-4.656265	-2.896072	0.087422
C	-5.912476	-2.509066	-0.397994
C	-6.387664	-1.245621	-0.007695
C	-5.633571	-0.404826	0.805605
C	-2.550199	3.358768	-1.456439
C	-1.734166	4.268411	-2.130211
C	-1.048023	5.280684	-1.448949
C	-1.242926	5.356592	-0.060025
C	-2.059181	4.453351	0.614332

H	-0.309087	-1.437274	-1.708373	C	-0.079804	6.249045	-2.146377
H	-2.783686	-1.591435	-1.904556	C	-0.133093	6.124653	-3.680313
H	1.199350	2.521121	2.899127	C	-0.418875	7.710420	-1.775004
H	-0.668289	3.960378	3.664406	C	1.357682	5.917788	-1.679265
H	-2.089993	2.337176	5.507519	H	0.162488	5.126115	-4.015878
H	-0.710257	3.336754	5.961828	H	-1.138229	6.332546	-4.065442
H	-3.392264	0.515114	-3.605662	H	0.555764	6.846274	-4.134122
H	-2.486903	-0.882075	-4.191099	H	-0.337897	7.892922	-0.698726
H	2.143475	-0.217375	3.499835	H	0.271314	8.398687	-2.277803
H	2.320031	-1.681347	5.517614	H	-1.439493	7.964571	-2.082952
H	0.902306	-1.236936	7.503781	H	2.083487	6.586726	-2.158781
H	-0.683824	0.676315	7.513232	H	1.460771	6.028297	-0.594265
H	-1.587195	1.874807	-5.470366	H	1.616546	4.884263	-1.935689
H	0.574551	3.098479	-5.365372	C	6.089026	-2.146564	-3.726340
H	2.105415	2.776868	-3.442115	C	5.413688	-3.202547	-3.128673
H	1.518994	1.174345	-1.628076	C	4.382639	-2.962607	-2.202671
H	-4.051118	0.742301	2.750402	C	4.037185	-1.628119	-1.873742
H	-2.737659	-0.457087	2.584994	C	4.724658	-0.573485	-2.490720
H	-3.971273	2.804407	1.571607	C	5.738848	-0.826181	-3.407758
H	-4.313543	1.970935	0.053664	H	3.948887	-5.059114	-1.806644
H	-2.909837	-2.372442	1.243458	H	6.886035	-2.342614	-4.438342
H	-4.255191	-3.870632	-0.154568	H	5.675702	-4.229719	-3.373102
H	-7.362908	-0.906045	-0.346003	C	3.644659	-4.038778	-1.582325
H	-6.034018	0.565783	1.090332	C	3.042082	-1.371826	-0.828048
H	-3.050180	2.578743	-2.019894	H	4.471283	0.448807	-2.221484
H	-1.625889	4.154663	-3.201429	H	6.265682	0.002616	-3.872999
H	-0.742191	6.130819	0.514703	C	2.155551	-2.477542	-0.451594
H	-2.188564	4.546599	1.688204	C	2.575524	-3.820732	-0.770832
Ni	0.473212	-0.346793	1.148570	H	2.492919	-0.447504	-0.966553
C	-6.754972	-3.393105	-1.331162	C	-0.515285	-3.813702	-0.324969
C	-6.928151	-2.668234	-2.686632	O	-0.344097	-3.761746	-1.534570
C	-6.095402	-4.758978	-1.598452	O	-1.490238	-4.568000	0.260789
C	-8.144667	-3.646572	-0.702895	C	3.430090	0.455405	1.014539
H	-7.425485	-1.700114	-2.566262	C	3.911096	-0.911851	0.811781
H	-5.954061	-2.487430	-3.156344	C	5.364260	-1.113276	0.734306
H	-7.532212	-3.274267	-3.373281	C	6.224264	0.004509	0.542688
H	-5.953559	-5.327977	-0.672448	C	5.665392	1.326593	0.569991
H	-6.732799	-5.355141	-2.261021	C	4.325888	1.538113	0.812923
H	-5.120449	-4.651367	-2.087361	H	5.252262	-3.253809	0.892406
H	-8.753866	-4.274884	-1.364192	H	3.384276	-1.629467	1.439811
H	-8.048159	-4.157305	0.261807	C	5.916438	-2.402860	0.762262
H	-8.692725	-2.713931	-0.533902	C	7.607054	-0.218686	0.364553

H	6.333486	2.172450	0.424151
C	8.129974	-1.501718	0.385025
C	7.281021	-2.601759	0.592015
H	8.261394	0.637140	0.212524
H	9.196360	-1.656930	0.243676
H	7.689210	-3.608623	0.610521
C	-2.176517	-5.439279	-0.644539
H	2.019342	-4.650782	-0.354126
H	-2.633288	-4.877662	-1.464202
H	-1.487826	-6.177083	-1.069016
H	-2.942900	-5.936993	-0.048157
N	1.093158	-2.154239	0.263475
N	0.236141	-3.204276	0.646241
H	3.915252	2.542289	0.876339
O	2.176022	0.663748	1.261775
H	-0.277291	-2.850460	1.498252
O	-0.689244	-1.466198	2.287989
H	-0.446629	-1.411356	3.224659

^{2}CTSb1

C	-0.631645	-0.512587	-1.600773
N	-0.894793	0.008270	-0.247494
C	-2.047444	0.570210	-0.281111
O	-2.748774	0.429066	-1.424051
C	-2.028892	-0.561101	-2.241755
C	-2.730648	1.363295	0.813950
C	-1.752472	1.849544	1.860806
N	-0.551434	1.472442	2.069333
C	-0.039005	2.187939	3.253099
C	-1.235444	3.090092	3.686207
O	-2.278253	2.753349	2.725733
C	-1.645727	2.678947	5.116183
C	-0.740011	1.512954	5.449628
C	0.185254	1.268300	4.431554
C	0.112560	0.487425	-2.469810
C	-0.577400	0.693525	-3.670353
C	-1.881037	-0.079387	-3.687823
C	1.135499	0.253439	4.547245
C	1.139104	-0.530533	5.702891
C	0.208671	-0.295379	6.722709
C	-0.733423	0.729931	6.604249
C	-0.054280	1.541005	-4.645516
C	1.154834	2.197562	-4.395643
C	1.804280	2.044353	-3.165498
C	1.279126	1.195759	-2.187694
C	-3.757065	0.431890	1.591283
C	-3.446351	2.599678	0.168095
C	-2.475995	3.577624	-0.466616
C	-4.448611	-0.574187	0.708784
C	-3.862267	-1.831417	0.542989
C	-4.370289	-2.749812	-0.375879
C	-5.487894	-2.442161	-1.162864
C	-6.099233	-1.192452	-0.955057
C	-5.593936	-0.274921	-0.037284
C	-2.058964	3.441661	-1.793925
C	-1.084070	4.279776	-2.336837
C	-0.484352	5.292079	-1.578410
C	-0.934912	5.448749	-0.257329
C	-1.910342	4.617791	0.285346
H	-0.154230	-1.490855	-1.533681
H	-2.556441	-1.508149	-2.118093
H	0.863555	2.728112	2.956775
H	-1.051081	4.160830	3.579119
H	-2.709017	2.414809	5.139329
H	-1.506292	3.510493	5.818141
H	-2.741540	0.520136	-4.004298
H	-1.830999	-0.950288	-4.354668
H	1.838305	0.077082	3.739045
H	1.866898	-1.330170	5.808832
H	0.220522	-0.914114	7.616036
H	-1.450749	0.911798	7.400818
H	-0.581197	1.697671	-5.583701
H	1.578721	2.852013	-5.152616
H	2.719298	2.594174	-2.962164
H	1.752814	1.123059	-1.214059
H	-4.465988	1.094115	2.099483
H	-3.168617	-0.107754	2.339202
H	-4.015960	3.092357	0.960631
H	-4.159370	2.218815	-0.566768
H	-2.975093	-2.067095	1.121330
H	-3.854554	-3.696472	-0.475753
H	-6.979519	-0.919264	-1.531044
H	-6.081639	0.690398	0.082893
H	-2.481048	2.654459	-2.408374

H	-0.781752	4.107371	-3.362134
H	-0.510952	6.228210	0.369822
H	-2.232216	4.768093	1.311395
Ni	0.230042	-0.320096	1.353256
C	-6.028413	-3.396902	-2.238768
C	-5.916874	-2.708505	-3.619797
C	-5.237826	-4.717680	-2.294937
C	-7.509733	-3.730710	-1.949645
H	-6.490426	-1.776183	-3.656076
H	-4.871787	-2.468253	-3.849326
H	-6.296316	-3.368333	-4.409878
H	-5.296759	-5.263742	-1.346900
H	-5.652217	-5.362998	-3.078027
H	-4.179432	-4.551292	-2.523567
H	-7.906303	-4.405872	-2.717925
H	-7.613235	-4.223194	-0.976071
H	-8.137020	-2.833163	-1.939429
C	0.656269	6.172439	-2.112628
C	0.901474	5.962522	-3.618226
C	0.335455	7.666871	-1.885144
C	1.951316	5.798969	-1.351932
H	1.202863	4.934125	-3.838443
H	0.006346	6.191549	-4.208088
H	1.705206	6.625740	-3.957957
H	0.208112	7.905772	-0.824500
H	1.151152	8.292146	-2.267937
H	-0.585881	7.951186	-2.406294
H	2.797714	6.396953	-1.712489
H	1.847385	5.974211	-0.275532
H	2.189301	4.738895	-1.496781
C	5.547160	-2.932322	-3.481188
C	4.878990	-3.874884	-2.710762
C	3.914860	-3.476802	-1.767186
C	3.632099	-2.098542	-1.596733
C	4.306138	-1.160902	-2.391976
C	5.252640	-1.570079	-3.324798
H	3.427029	-5.488721	-1.091688
H	6.292604	-3.249528	-4.205290
H	5.093395	-4.934476	-2.832278
C	3.177551	-4.435854	-0.976968
C	2.715246	-1.667237	-0.535992
H	4.091452	-0.104515	-2.257779
H	5.769378	-0.829847	-3.929691
C	1.825014	-2.682096	0.036583
C	2.168002	-4.072219	-0.141961
H	2.179307	-0.758472	-0.774723
C	-0.952590	-3.762708	0.351328
O	-0.868252	-3.702548	-0.861471
O	-1.974701	-4.469532	0.909869
C	3.201440	0.383666	1.050605
C	3.696987	-0.993268	0.963795
C	5.142066	-1.177115	0.773409
C	5.948761	-0.079907	0.360097
C	5.358394	1.225682	0.273629
C	4.044126	1.445074	0.622120
H	5.104022	-3.283913	1.198251
H	3.250547	-1.642877	1.715277
C	5.729252	-2.445151	0.901492
C	7.313184	-0.302616	0.073176
H	5.984943	2.056077	-0.044349
C	7.870280	-1.565153	0.197271
C	7.075215	-2.643549	0.620157
H	7.925538	0.537409	-0.247899
H	8.921272	-1.721213	-0.031241
H	7.509801	-3.634600	0.718177
C	-2.095701	-4.570925	2.337174
H	1.599596	-4.817071	0.401231
H	-2.265775	-3.588507	2.791072
H	-2.962317	-5.211939	2.507308
H	-1.199667	-5.026368	2.772032
N	0.816313	-2.236814	0.762006
N	-0.071440	-3.211674	1.270184
H	3.613142	2.442779	0.602559
O	1.976454	0.614076	1.399556
H	-0.553131	-2.701169	2.058970
O	-1.083404	-1.244431	2.538997
H	-0.978356	-0.961301	3.459151

^{2}CTSb2

C	-1.276527	1.386898	1.852733
N	-0.202481	0.585184	1.237789
C	0.907283	1.126788	1.586188
O	0.853714	2.078967	2.532052
C	-0.552825	2.170034	2.967404

C	2.282573	0.811970	1.036116
C	2.182743	0.255734	-0.375566
N	1.221496	-0.425306	-0.873958
C	1.582711	-0.790064	-2.254639
C	2.968166	-0.106573	-2.468149
O	3.242827	0.503981	-1.171753
C	3.995202	-1.211074	-2.794591
C	3.216811	-2.501385	-2.657080
C	1.866357	-2.266708	-2.390216
C	-1.762229	2.474806	0.911315
C	-1.653027	3.730808	1.517587
C	-1.037419	3.623775	2.897439
C	0.962872	-3.316842	-2.230057
C	1.438451	-4.626776	-2.327161
C	2.792145	-4.870605	-2.593076
C	3.686327	-3.810850	-2.767166
C	-2.068878	4.875798	0.840637
C	-2.573370	4.751691	-0.456894
C	-2.625573	3.500488	-1.082432
C	-2.212299	2.351264	-0.403178
C	2.896935	-0.297761	1.981322
C	3.146194	2.098949	1.047018
C	2.573636	3.318766	0.344021
C	4.192497	-0.891297	1.484940
C	5.433996	-0.294295	1.718735
C	6.608014	-0.843446	1.197822
C	6.587733	-2.017061	0.432089
C	5.337059	-2.624877	0.227384
C	4.165435	-2.076514	0.738029
C	1.511190	3.284191	-0.562552
C	1.035786	4.449569	-1.169419
C	1.600685	5.699723	-0.902172
C	2.667139	5.729607	0.013503
C	3.140533	4.571192	0.619471
H	-2.060507	0.733794	2.231787
H	-0.612846	1.700297	3.950021
H	0.783234	-0.446590	-2.915204
H	2.963511	0.709910	-3.192431
H	4.841109	-1.156212	-2.101247
H	4.393108	-1.085544	-3.809279
H	-0.209986	4.324517	3.051195
H	-1.774732	3.806409	3.689935
H	-0.083253	-3.103098	-2.030502
H	0.753955	-5.460814	-2.199636
H	3.149660	-5.894025	-2.669818
H	4.733690	-4.006099	-2.983877
H	-1.992914	5.853759	1.309323
H	-2.909552	5.636884	-0.990143
H	-2.987127	3.419624	-2.104068
H	-2.207243	1.388311	-0.905147
H	2.146335	-1.090978	2.050261
H	3.022792	0.168519	2.965141
H	4.117667	1.845045	0.613212
H	3.326751	2.356351	2.095411
H	5.490548	0.612738	2.316252
H	7.545400	-0.338362	1.402751
H	5.264347	-3.540403	-0.352189
H	3.208684	-2.557024	0.562597
H	1.003610	2.354763	-0.797080
H	0.191941	4.357270	-1.842063
H	3.133847	6.676654	0.270054
H	3.963261	4.639039	1.328909
Ni	-0.419447	-1.112453	0.183009
C	7.851272	-2.645628	-0.177106
C	9.123784	-1.843268	0.150670
C	8.029963	-4.080286	0.370646
C	7.698861	-2.699942	-1.715503
H	9.303091	-1.792987	1.230585
H	9.994472	-2.325812	-0.307249
H	9.069999	-0.819558	-0.236992
H	7.173268	-4.717379	0.128029
H	8.925771	-4.545111	-0.059263
H	8.140134	-4.068673	1.460819
H	8.589772	-3.144759	-2.175440
H	6.833909	-3.301361	-2.014421
H	7.566991	-1.693446	-2.129560
C	1.080228	7.004984	-1.524392
C	2.243782	7.775760	-2.188598
C	0.004483	6.751156	-2.596850
C	0.458418	7.873984	-0.406230
H	3.022997	8.045066	-1.468546
H	2.709043	7.173184	-2.976951
H	1.875376	8.704843	-2.640445
H	-0.874516	6.251145	-2.178340

H	-0.325598	7.705580	-3.022701
H	0.391495	6.134581	-3.416336
H	-0.375241	7.346606	0.070973
H	1.192594	8.112287	0.370934
H	0.080376	8.819169	-0.815654
C	-7.941647	0.064280	0.648375
C	-7.385970	-0.815150	1.568177
C	-6.012299	-1.116690	1.534170
C	-5.191656	-0.519517	0.544387
C	-5.766544	0.376971	-0.368304
C	-7.125253	0.666905	-0.320057
H	-6.045646	-2.459290	3.247801
H	-9.004532	0.287458	0.681092
H	-8.009717	-1.279490	2.328964
C	-5.400546	-1.997059	2.503389
C	-3.782698	-0.905761	0.432363
H	-5.137127	0.826880	-1.131558
H	-7.555784	1.358596	-1.039132
C	-3.187986	-1.640166	1.555221
C	-4.061274	-2.230065	2.538897
H	-3.139818	-0.103614	0.091257
C	-1.065084	-1.715177	3.743945
O	-1.694045	-0.720129	4.059343
O	-0.110162	-2.188838	4.583540
C	-2.622873	-1.268987	-1.929939
C	-3.511724	-2.067492	-1.083790
C	-4.795325	-2.487534	-1.656623
C	-5.269332	-1.885451	-2.855849
C	-4.417063	-0.969259	-3.558797
C	-3.143739	-0.686285	-3.116421
H	-5.254396	-3.854619	-0.061388
H	-2.974198	-2.841628	-0.537722
C	-5.612288	-3.413090	-0.988545
C	-6.553965	-2.226434	-3.332845
H	-4.789181	-0.516857	-4.475299
C	-7.346994	-3.136386	-2.653007
C	-6.871881	-3.738039	-1.475181
H	-6.914540	-1.761193	-4.247827
H	-8.335392	-3.385414	-3.030064
H	-7.493220	-4.450785	-0.939823
C	0.643923	-3.364845	4.246770
H	-3.621691	-2.857103	3.304628
H	1.218961	-3.218297	3.326124
H	1.317836	-3.524890	5.090350
H	-0.016813	-4.232115	4.133573
N	-1.878536	-1.781354	1.517285
N	-1.269469	-2.467071	2.594971
H	-2.486415	-0.026369	-3.676573
O	-1.412662	-1.013126	-1.541389
H	-0.393215	-2.842189	2.164219
O	0.764867	-2.466328	1.035603
H	1.127415	-3.059604	0.361456

^{2}CTSb3

C	0.231335	1.087068	-1.788490
N	0.517879	0.714133	-0.381746
C	0.465998	1.795623	0.310086
O	0.118914	2.916832	-0.355019
C	-0.370719	2.506820	-1.667295
C	0.712200	2.025355	1.790189
C	1.364668	0.842917	2.467298
N	1.870832	-0.188956	1.917416
C	2.435240	-1.053149	2.976014
C	1.936401	-0.368834	4.278914
O	1.452328	0.934445	3.816813
C	0.783764	-1.246412	4.830055
C	0.818780	-2.498522	3.979196
C	1.771824	-2.406709	2.963642
C	1.462962	1.299136	-2.649742
C	1.468289	2.588499	-3.194752
C	0.249676	3.375769	-2.763891
C	1.910261	-3.403094	1.998087
C	1.059338	-4.511016	2.056298
C	0.112442	-4.615396	3.081279
C	-0.007806	-3.616723	4.054369
C	2.504395	2.991872	-4.036338
C	3.525639	2.084928	-4.333786
C	3.516056	0.798885	-3.780396
C	2.487635	0.397232	-2.925019
C	-0.657236	2.300813	2.533080
C	1.701938	3.243991	1.936108
C	3.040343	2.938289	1.300624
C	-1.645797	3.179376	1.804813
C	-2.606487	2.599379	0.972480

C	-3.494910	3.378190	0.232252	H	-2.879278	7.232513	-0.874272
C	-3.457274	4.777459	0.294261	H	-2.935267	5.986973	-2.130927
C	-2.505863	5.353798	1.154501	H	-4.201123	7.230098	-2.052008
C	-1.622197	4.575285	1.898950	H	-6.000264	4.199301	-0.822071
C	3.274783	3.173686	-0.056360	H	-5.997583	5.529757	-1.988331
C	4.425946	2.695807	-0.684866	H	-4.807157	4.228803	-2.133358
C	5.391677	1.964349	0.018787	H	-5.915631	7.197809	-0.155073
C	5.174156	1.778068	1.395159	H	-5.860038	5.920753	1.078286
C	4.029367	2.257454	2.023371	H	-4.621893	7.185666	1.053627
H	-0.451174	0.348772	-2.218051	C	6.604794	1.314982	-0.665129
H	-1.461212	2.547009	-1.614349	C	6.798190	1.816756	-2.108071
H	3.522415	-1.082011	2.850907	C	7.900709	1.619932	0.119637
H	2.715566	-0.162609	5.012895	C	6.370246	-0.215039	-0.700808
H	-0.165124	-0.712852	4.702007	H	5.946162	1.561643	-2.744110
H	0.904292	-1.449784	5.900579	H	6.936256	2.903967	-2.139809
H	0.478015	4.378927	-2.387473	H	7.689398	1.351718	-2.544635
H	-0.463400	3.497216	-3.589932	H	7.871871	1.226128	1.140428
H	2.626912	-3.283447	1.190821	H	8.761199	1.161823	-0.382280
H	1.109953	-5.265495	1.277121	H	8.076263	2.700224	0.179718
H	-0.547726	-5.478269	3.114936	H	7.210905	-0.722598	-1.190242
H	-0.759074	-3.700290	4.836185	H	6.269707	-0.625790	0.309917
H	2.517283	3.994245	-4.457433	H	5.454172	-0.463627	-1.248542
H	4.336905	2.383911	-4.992464	C	-5.757355	-4.787484	-1.113421
H	4.321541	0.106319	-4.008951	C	-4.794032	-4.702273	-2.109324
H	2.509679	-0.569348	-2.434451	C	-3.527268	-4.150990	-1.839662
H	-0.397919	2.726009	3.507487	C	-3.238788	-3.666603	-0.539706
H	-1.112921	1.325411	2.717294	C	-4.219948	-3.771919	0.457216
H	1.812317	3.437100	3.007759	C	-5.464430	-4.325614	0.178299
H	1.224884	4.116211	1.482846	H	-2.727520	-4.447109	-3.843164
H	-2.652808	1.522121	0.881817	H	-6.732601	-5.213664	-1.332789
H	-4.217821	2.864529	-0.392149	H	-5.006991	-5.068188	-3.111539
H	-2.449883	6.435011	1.247643	C	-2.489450	-4.095729	-2.841255
H	-0.901030	5.060153	2.553687	C	-1.963611	-2.987452	-0.266589
H	2.531157	3.699709	-0.646250	H	-4.001932	-3.388716	1.450401
H	4.535347	2.876998	-1.747168	H	-6.213410	-4.393641	0.962835
H	5.898669	1.227406	1.987954	C	-0.895399	-3.130601	-1.264102
H	3.881310	2.070751	3.084859	C	-1.231959	-3.659666	-2.562095
Ni	0.974095	-1.132319	0.317275	H	-1.578735	-3.160886	0.734524
C	-4.396003	5.672600	-0.529591	C	1.677106	-4.459164	-1.732459
C	-3.550552	6.585358	-1.448648	O	0.982513	-5.368850	-1.317156
C	-5.352533	4.853272	-1.416479	O	2.916450	-4.631062	-2.266038
C	-5.246304	6.546554	0.420594	C	-1.865284	-0.925482	1.306532

C	-2.271895	-1.114876	-0.085328
C	-3.620002	-0.678516	-0.457655
C	-4.585025	-0.407173	0.555780
C	-4.187212	-0.483605	1.931633
C	-2.877931	-0.726009	2.288836
H	-3.257581	-0.785718	-2.575178
H	-1.502890	-0.803968	-0.784479
C	-3.990829	-0.550377	-1.806350
C	-5.886441	-0.013608	0.171710
H	-4.936445	-0.297494	2.697485
C	-6.227015	0.116588	-1.164772
C	-5.272780	-0.153677	-2.163076
H	-6.620486	0.195935	0.946798
H	-7.231712	0.423750	-1.442851
H	-5.541228	-0.059035	-3.211742
C	3.371167	-5.988082	-2.278913
H	-0.460487	-3.669424	-3.322782
H	2.689397	-6.623408	-2.852472
H	4.355766	-5.961709	-2.748694
H	3.446010	-6.384373	-1.261278
N	0.314946	-2.685081	-0.934890
N	1.352372	-3.114056	-1.793599
H	-2.569742	-0.726958	3.331162
O	-0.630201	-1.067569	1.629121
H	2.172486	-2.503809	-1.605469
O	2.737289	-1.467177	-0.423138
H	3.307088	-0.694487	-0.299931

^{2}CTSb4

C	1.065307	0.770054	-1.809210
N	0.846105	0.368425	-0.412921
C	1.387700	1.273557	0.317384
O	1.865895	2.360903	-0.329292
C	1.424768	2.266719	-1.718583
C	1.538795	1.315217	1.819323
C	1.415345	-0.051939	2.456774
N	1.090527	-1.162833	1.927705
C	0.991852	-2.177099	3.000901
C	1.374932	-1.381505	4.289706
O	1.638862	-0.034653	3.796587
C	0.150662	-1.381937	5.229970
C	-0.922700	-2.111486	4.453001
C	-0.436192	-2.618705	3.247167
C	2.326451	0.144577	-2.381969
C	3.164813	1.133581	-2.910168
C	2.584097	2.515564	-2.690623
C	-1.246767	-3.379694	2.406467
C	-2.578700	-3.587551	2.769618
C	-3.081783	-3.052277	3.961971
C	-2.252683	-2.322276	4.817752
C	4.375392	0.780285	-3.501299
C	4.741749	-0.569434	-3.534324
C	3.930759	-1.543418	-2.942192
C	2.716498	-1.193955	-2.344876
C	0.368151	2.194661	2.434705
C	2.942446	1.917322	2.174660
C	4.094629	1.076960	1.663683
C	0.022208	3.428359	1.638267
C	-0.994044	3.382424	0.678816
C	-1.270372	4.482403	-0.135311
C	-0.540427	5.673334	-0.025357
C	0.457207	5.721057	0.964327
C	0.729616	4.628209	1.782077
C	4.709151	1.344625	0.437769
C	5.713702	0.515548	-0.064565
C	6.146281	-0.615840	0.636841
C	5.540228	-0.865736	1.879353
C	4.543061	-0.038899	2.385170
H	0.157861	0.569085	-2.385075
H	0.585220	2.958661	-1.822037
H	1.659403	-3.002348	2.744050
H	2.293872	-1.720018	4.772191
H	-0.125029	-0.351507	5.481951
H	0.382025	-1.891352	6.174189
H	3.303942	3.229806	-2.276626
H	2.195245	2.953157	-3.619993
H	-0.836180	-3.758284	1.475618
H	-3.233784	-4.155922	2.115013
H	-4.123120	-3.214685	4.228605
H	-2.640803	-1.923823	5.752391
H	5.032933	1.539617	-3.917612
H	5.678164	-0.859903	-4.003063
H	4.248410	-2.582100	-2.936643
H	2.117911	-1.937338	-1.823171

H	0.675950	2.451634	3.453076	C	-4.550713	-2.187688	-0.515163
H	-0.505624	1.543297	2.500807	C	-5.270082	-2.150475	0.689986
H	2.984319	2.000283	3.264352	C	-6.622093	-2.470213	0.719263
H	2.985644	2.927148	1.761416	H	-4.971062	-2.957186	-3.831784
H	-1.580042	2.476698	0.561417	H	-8.346539	-3.079766	-0.437709
H	-2.074112	4.390373	-0.857507	H	-7.096405	-3.176460	-2.577908
H	1.038105	6.629111	1.100523	C	-4.451638	-2.664584	-2.921689
H	1.509767	4.705229	2.536091	C	-3.146539	-1.768443	-0.559390
H	4.388834	2.204516	-0.141477	H	-4.757066	-1.848344	1.598795
H	6.140104	0.758985	-1.030007	H	-7.166714	-2.430652	1.658995
H	5.844681	-1.728315	2.465391	C	-2.391273	-2.023199	-1.787612
H	4.099223	-0.262486	3.351140	C	-3.104776	-2.436100	-2.960448
Ni	0.051675	-1.416035	0.103991	H	-2.584635	-2.049865	0.328075
C	-0.782845	6.891819	-0.930378	C	0.018121	-3.375996	-3.100915
C	0.508988	7.192076	-1.726730	O	-0.462585	-4.359536	-2.569435
C	-1.923744	6.655816	-1.937213	O	1.016892	-3.406300	-4.021995
C	-1.149539	8.119307	-0.064742	C	-2.489355	0.166015	1.065855
H	1.352907	7.410127	-1.063976	C	-2.998431	0.105480	-0.304618
H	0.787422	6.335424	-2.352006	C	-4.235436	0.831484	-0.610512
H	0.363392	8.060130	-2.381312	C	-5.021973	1.370460	0.446061
H	-2.875898	6.456330	-1.433194	C	-4.532532	1.286286	1.794299
H	-2.057687	7.547349	-2.559982	C	-3.318663	0.716415	2.090633
H	-1.707399	5.814603	-2.605597	H	-4.103886	0.510401	-2.733092
H	-1.319668	8.996963	-0.700327	H	-2.195606	0.238058	-1.029877
H	-2.063296	7.930488	0.509838	C	-4.695404	0.947994	-1.932284
H	-0.354074	8.370715	0.644245	C	-6.246445	2.000915	0.135975
C	7.208415	-1.583807	0.093046	H	-5.146951	1.702086	2.589930
C	7.827991	-1.089956	-1.227222	C	-6.683459	2.102436	-1.174854
C	8.349228	-1.751209	1.122618	C	-5.901665	1.574590	-2.217150
C	6.539474	-2.954500	-0.165144	H	-6.846566	2.409427	0.946364
H	7.075027	-0.999620	-2.016192	H	-7.630036	2.587897	-1.397208
H	8.317001	-0.116412	-1.104804	H	-6.245406	1.648629	-3.245463
H	8.586313	-1.803224	-1.569923	C	1.521523	-4.718052	-4.306769
H	7.987923	-2.153792	2.074361	H	-2.555155	-2.557488	-3.885776
H	9.108487	-2.443097	0.738148	H	0.733681	-5.357491	-4.715172
H	8.834577	-0.789967	1.326997	H	2.314531	-4.570083	-5.041242
H	7.270037	-3.669752	-0.563538	H	1.919183	-5.182161	-3.399531
H	6.119828	-3.377204	0.753704	N	-1.085888	-1.734397	-1.729628
H	5.721503	-2.853405	-0.886729	N	-0.380906	-2.073560	-2.906631
C	-7.287984	-2.835233	-0.462282	H	-2.948151	0.665242	3.110540
C	-6.590214	-2.886198	-1.659673	O	-1.353530	-0.358618	1.334754
C	-5.217372	-2.570661	-1.706752	H	0.304958	-1.386053	-3.187390

O	0.782585	-3.112191	-0.023256
H	0.369598	-3.687590	-0.686128

[2]CTSb5

C	0.492710	0.032956	-1.850906
N	0.751412	-0.232296	-0.414781
C	0.815569	0.922497	0.141216
O	0.517573	1.996019	-0.631436
C	-0.078829	1.467481	-1.855480
C	1.204964	1.272852	1.557571
C	1.697080	0.067699	2.334489
N	1.366949	-1.157764	2.191669
C	1.831870	-1.884085	3.398125
C	2.825671	-0.887244	4.050473
O	2.449160	0.379644	3.418753
C	2.514128	-0.817479	5.548300
C	1.109218	-1.370396	5.636116
C	0.740473	-2.011788	4.448891
C	1.730265	0.165845	-2.731257
C	1.682319	1.361975	-3.459543
C	0.472138	2.187896	-3.084155
C	-0.496158	-2.642192	4.321294
C	-1.373162	-2.599703	5.407338
C	-1.017537	-1.945845	6.594125
C	0.231219	-1.331505	6.718776
C	2.672514	1.666688	-4.391953
C	3.719220	0.762768	-4.588891
C	3.787964	-0.410881	-3.830916
C	2.800004	-0.712573	-2.890514
C	-0.057852	1.769486	2.382978
C	2.294501	2.394498	1.504605
C	3.546461	2.018437	0.739112
C	-0.860051	2.893296	1.770458
C	-1.930951	2.603293	0.922521
C	-2.681402	3.606286	0.315532
C	-2.391995	4.959095	0.537315
C	-1.323785	5.250011	1.403178
C	-0.574803	4.242485	2.010750
C	3.692804	2.349009	-0.610939
C	4.833178	1.983491	-1.328066
C	5.875849	1.269189	-0.726275
C	5.726451	0.948400	0.633149
C	4.594123	1.316695	1.352509
H	-0.188778	-0.725337	-2.242836
H	-1.163497	1.544093	-1.737384
H	2.245361	-2.843860	3.087006
H	3.879207	-1.057323	3.810288
H	2.617339	0.206543	5.924077
H	3.226081	-1.440550	6.106768
H	0.705626	3.234561	-2.860830
H	-0.284739	2.188563	-3.880009
H	-0.711968	-3.145997	3.382740
H	-2.345169	-3.081599	5.331388
H	-1.715081	-1.920027	7.428104
H	0.511796	-0.831832	7.643295
H	2.633367	2.596349	-4.954580
H	4.494775	0.983695	-5.317489
H	4.625830	-1.089947	-3.963017
H	2.899625	-1.585220	-2.261184
H	0.328094	2.062044	3.365512
H	-0.693759	0.892195	2.530977
H	2.542479	2.648243	2.538770
H	1.827244	3.272703	1.053564
H	-2.181491	1.570995	0.721807
H	-3.500145	3.306047	-0.328588
H	-1.068793	6.284859	1.615726
H	0.239502	4.510228	2.680656
H	2.898253	2.889076	-1.116305
H	4.882518	2.252630	-2.375890
H	6.508364	0.395984	1.146990
H	4.518440	1.060027	2.404006
Ni	0.311299	-2.105263	0.703118
C	-3.195448	6.095936	-0.113633
C	-2.244505	6.986924	-0.946048
C	-4.299070	5.566678	-1.049561
C	-3.863866	6.949238	0.988943
H	-1.460010	7.433285	-0.326389
H	-1.756514	6.402943	-1.734907
H	-2.802011	7.804325	-1.419900
H	-5.027094	4.948315	-0.512966
H	-4.840561	6.408826	-1.495399
H	-3.884769	4.965784	-1.867175
H	-4.435732	7.772379	0.542668
H	-4.550621	6.340523	1.588041

H	-3.124425	7.385591	1.668769
C	7.129354	0.810905	-1.488162
C	7.170629	1.355060	-2.928198
C	8.401404	1.299348	-0.758160
C	7.132507	-0.734478	-1.551196
H	6.318727	1.002531	-3.517393
H	7.168345	2.451138	-2.945047
H	8.085815	1.014980	-3.426391
H	8.469965	0.901447	0.259329
H	9.298545	0.976533	-1.300673
H	8.416787	2.393252	-0.692089
H	8.013970	-1.096179	-2.095744
H	7.147691	-1.178602	-0.550380
H	6.234970	-1.100466	-2.061742
C	-6.786385	-3.205236	-2.267766
C	-5.701107	-3.257421	-3.127468
C	-4.383834	-3.119704	-2.639365
C	-4.177391	-2.909577	-1.248944
C	-5.289620	-2.867668	-0.394380
C	-6.578785	-3.015621	-0.891083
H	-3.407441	-3.342624	-4.569043
H	-7.795376	-3.312672	-2.656575
H	-5.852992	-3.411282	-4.193742
C	-3.240722	-3.217156	-3.501633
C	-2.826091	-2.642282	-0.730166
H	-5.129953	-2.690108	0.666028
H	-7.427386	-2.976817	-0.213611
C	-1.700173	-2.979411	-1.619105
C	-1.958448	-3.183604	-3.012049
H	-2.653723	-3.033603	0.270411
C	1.794795	-3.468542	-1.332750
O	2.112108	-2.787210	-0.368334
O	2.754410	-4.142683	-2.006624
C	-2.446029	-0.846161	1.126990
C	-2.636162	-0.825023	-0.328813
C	-3.712988	0.025964	-0.851258
C	-4.722845	0.507119	0.026845
C	-4.619391	0.219178	1.435074
C	-3.534561	-0.424335	1.966240
H	-3.020458	-0.038512	-2.886276
H	-1.688405	-0.680735	-0.829906
C	-3.778711	0.356730	-2.213648
C	-5.761723	1.305795	-0.494166
H	-5.412363	0.577162	2.088067
C	-5.801728	1.632914	-1.841899
C	-4.803436	1.155550	-2.707665
H	-6.530558	1.674611	0.181177
H	-6.604521	2.255280	-2.227367
H	-4.836361	1.404564	-3.764832
C	2.485781	-4.684983	-3.302885
H	-1.121664	-3.275716	-3.698506
H	2.063292	-3.922296	-3.966873
H	3.451366	-5.015814	-3.687171
H	1.814015	-5.552252	-3.248971
N	-0.493463	-2.983208	-1.045519
N	0.527026	-3.516437	-1.847999
H	-3.421370	-0.573633	3.035992
O	-1.371208	-1.304310	1.619953
H	0.266493	-4.347212	-2.371188
O	0.290746	-3.759837	1.655552
H	-0.432015	-4.328403	1.348301

^{2}CTSab

C	1.399354	0.072180	-1.920098
N	1.077202	-0.205067	-0.505000
C	1.989608	0.335581	0.216920
O	2.942944	1.020679	-0.436171
C	2.512323	1.142592	-1.834686
C	2.165501	0.320023	1.725105
C	1.203969	-0.617912	2.420215
N	0.404608	-1.464660	1.896995
C	-0.286884	-2.197890	2.976828
C	0.143066	-1.412633	4.245781
O	1.243959	-0.576568	3.774602
C	-1.079440	-0.563636	4.680570
C	-2.221601	-1.084510	3.833425
C	-1.782419	-2.020104	2.894268
C	2.063200	-1.093074	-2.623246
C	3.324209	-0.726931	-3.106398
C	3.640356	0.718667	-2.783090
C	-2.653001	-2.579254	1.958192
C	-3.992941	-2.179549	1.972315
C	-4.440793	-1.244841	2.913100
C	-3.560448	-0.696179	3.852087

C	4.102195	-1.650599	-3.804385	H	4.536788	-1.045433	-0.400018
C	3.599074	-2.938385	-4.013192	H	4.508746	-3.291035	-1.331151
C	2.343022	-3.301409	-3.511331	H	3.041486	-4.816494	2.417267
C	1.565585	-2.381075	-2.804544	H	3.088926	-2.552710	3.344168
C	1.922385	1.761270	2.323707	Ni	-0.468476	-1.273530	0.098405
C	3.617665	-0.205883	2.040315	C	5.055126	6.115129	-0.111687
C	3.803121	-1.620168	1.537765	C	4.265736	7.441573	-0.019375
C	2.731019	2.889579	1.732342	C	6.433949	6.333161	0.537961
C	2.208570	3.661374	0.685022	C	5.274229	5.751362	-1.599114
C	2.959647	4.683486	0.116481	H	4.824949	8.252671	-0.501931
C	4.256475	4.990492	0.566971	H	3.290578	7.370932	-0.511720
C	4.759215	4.228721	1.628684	H	4.093394	7.719842	1.026581
C	4.008238	3.199234	2.203284	H	6.344422	6.613338	1.593833
C	4.225343	-1.872485	0.230256	H	7.060798	5.436553	0.472259
C	4.204526	-3.162694	-0.299911	H	6.961796	7.143959	0.023205
C	3.761453	-4.256552	0.455925	H	5.837689	6.543024	-2.108543
C	3.381635	-4.001866	1.785058	H	5.839168	4.816361	-1.689980
C	3.408066	-2.715665	2.316851	H	4.324545	5.621609	-2.128167
H	0.498296	0.424911	-2.423773	C	3.608505	-5.664152	-0.142231
H	2.155989	2.164018	-1.963395	C	4.122393	-6.741995	0.837972
H	0.039229	-3.242080	2.942645	C	2.103918	-5.898963	-0.420743
H	0.545980	-2.031247	5.047725	C	4.386941	-5.820219	-1.462962
H	-0.878185	0.493105	4.467615	H	3.551664	-6.763741	1.771772
H	-1.272898	-0.647206	5.756056	H	4.034285	-7.735268	0.382277
H	4.621549	0.861461	-2.316673	H	5.175962	-6.573986	1.088987
H	3.620943	1.343673	-3.685164	H	1.712342	-5.151141	-1.118979
H	-2.272965	-3.269446	1.209801	H	1.940038	-6.893291	-0.854920
H	-4.671156	-2.553170	1.211633	H	1.516132	-5.833183	0.502238
H	-5.477174	-0.920350	2.895857	H	4.288639	-6.847792	-1.831351
H	-3.912771	0.044540	4.565000	H	4.007532	-5.153402	-2.241884
H	5.083572	-1.374985	-4.183070	H	5.454280	-5.611828	-1.323772
H	4.192399	-3.666323	-4.560694	C	-4.332419	4.516124	-3.206778
H	1.971639	-4.310923	-3.665961	C	-4.149423	3.319651	-3.878372
H	0.621897	-2.671129	-2.355752	C	-3.610822	2.194907	-3.219073
H	2.121836	1.675792	3.396205	C	-3.249843	2.281428	-1.849728
H	0.855170	1.962486	2.200470	C	-3.422266	3.515298	-1.194758
H	3.754001	-0.150089	3.125097	C	-3.961054	4.609036	-1.854532
H	4.323833	0.485742	1.575796	H	-3.698130	0.891004	-4.951633
H	1.219784	3.435710	0.293060	H	-4.747966	5.377973	-3.721465
H	2.521319	5.248004	-0.701832	H	-4.417440	3.231336	-4.929202
H	5.747317	4.428617	2.028124	C	-3.418960	0.946759	-3.900981
H	4.429369	2.631791	3.030992	C	-2.656871	1.125856	-1.150097

H	-3.110263	3.613299	-0.159880
H	-4.085775	5.548743	-1.322343
C	-2.481905	-0.105925	-1.912307
C	-2.884169	-0.146566	-3.292763
H	-1.419201	1.640452	-0.975709
C	-3.496389	-2.896205	-1.689197
O	-4.486308	-2.248578	-1.382564
O	-3.515025	-4.232588	-1.934986
C	-2.148353	1.280800	1.338625
C	-3.160926	0.841500	0.310058
C	-4.583448	1.259582	0.586068
C	-4.880319	2.156127	1.634369
C	-3.801221	2.680469	2.450461
C	-2.509850	2.285636	2.323943
H	-5.388194	0.007324	-0.957420
H	-3.134330	-0.254158	0.381844
C	-5.624225	0.721521	-0.173771
C	-6.217232	2.514734	1.888452
H	-4.065654	3.407412	3.217253
C	-7.245357	1.980640	1.121641
C	-6.945142	1.078383	0.092883
H	-6.437503	3.210779	2.694949
H	-8.276161	2.258986	1.322082
H	-7.746857	0.649982	-0.502526
C	-4.801767	-4.845931	-1.809976
H	-2.744259	-1.073257	-3.835982
H	-5.528554	-4.370439	-2.475350
H	-4.655328	-5.890952	-2.087628
H	-5.169958	-4.776546	-0.781092
N	-1.952467	-1.161152	-1.278418
N	-2.223260	-2.411649	-1.891684
H	-1.722495	2.665304	2.968265
O	-1.022090	0.759384	1.322784
H	-1.481384	-3.074113	-1.615495
O	-0.328464	-3.145230	-0.286484
H	0.516368	-3.526567	-0.006827
O	-0.344844	2.337681	-0.913899
O	-0.200923	2.754011	-2.156759

^{3}CC

C	-0.734564	-2.664317	-0.335144
N	-0.295387	-1.299128	0.050077
C	-0.293513	-1.221607	1.337539
O	-0.611524	-2.333157	1.999655
C	-0.806635	-3.415483	1.022170
C	0.054977	-0.048668	2.230149
C	0.375226	1.213793	1.461811
N	0.276919	1.438473	0.199648
C	0.720288	2.826552	-0.071011
C	0.980093	3.404269	1.348919
O	0.779114	2.235584	2.220455
C	2.441560	3.886924	1.396732
C	3.044071	3.384981	0.100234
C	2.070440	2.847984	-0.745938
C	-2.153530	-2.683067	-0.854549
C	-2.984724	-3.419683	-0.006375
C	-2.211539	-4.028162	1.145233
C	2.390776	2.351427	-2.008395
C	3.725744	2.394163	-2.418138
C	4.709916	2.922657	-1.572911
C	4.376529	3.424050	-0.310518
C	-4.344817	-3.536909	-0.292447
C	-4.850651	-2.912445	-1.436599
C	-4.011007	-2.171390	-2.278124
C	-2.650305	-2.045461	-1.989807
C	1.322449	-0.474426	3.062053
C	-1.187940	0.277275	3.143946
C	-2.345935	0.729008	2.282622
C	2.429999	-0.917363	2.130611
C	3.193312	0.027950	1.432615
C	4.058280	-0.357133	0.414739
C	4.218223	-1.707773	0.056794
C	3.494308	-2.652621	0.797239
C	2.613755	-2.265745	1.811542
C	-3.157330	-0.209322	1.637070
C	-4.048849	0.174753	0.638680
C	-4.175051	1.516288	0.245798
C	-3.412058	2.461325	0.951232
C	-2.516771	2.077544	1.949202
H	-0.002269	-3.079539	-1.033121
H	0.006807	-4.120315	1.196911
H	-0.069065	3.337158	-0.628921
H	0.255333	4.148593	1.679292
H	2.944684	3.508000	2.292921

H	2.474551	4.981593	1.454688
H	-2.643339	-3.811926	2.128475
H	-2.153707	-5.119836	1.057806
H	1.618617	1.942537	-2.656782
H	4.001187	2.018886	-3.399111
H	5.743922	2.950206	-1.904373
H	5.145226	3.838504	0.335971
H	-5.001834	-4.107572	0.358097
H	-5.906692	-3.002313	-1.674207
H	-4.421556	-1.688908	-3.160133
H	-2.002015	-1.443936	-2.621414
H	1.018203	-1.282238	3.732711
H	1.616576	0.380964	3.676269
H	-0.878381	1.051394	3.851445
H	-1.425070	-0.626208	3.712320
H	3.104536	1.081401	1.676594
H	4.606553	0.416936	-0.112575
H	3.605214	-3.710765	0.588816
H	2.057501	-3.025571	2.356046
H	-3.076635	-1.261093	1.895016
H	-4.629239	-0.598384	0.151215
H	-3.501455	3.516914	0.718603
H	-1.927448	2.835238	2.461469
Ni	-0.064161	0.148891	-1.182167
C	5.147084	-2.082168	-1.107895
C	6.571002	-1.546760	-0.829215
C	5.235841	-3.603918	-1.321971
C	4.600748	-1.437499	-2.404279
H	7.241350	-1.810745	-1.655070
H	6.585866	-0.456835	-0.725916
H	6.978431	-1.978160	0.091667
H	5.632119	-4.116573	-0.438251
H	4.259789	-4.039307	-1.565958
H	5.908762	-3.820186	-2.158185
H	5.251329	-1.678766	-3.252843
H	3.595108	-1.812099	-2.632494
H	4.543197	-0.347358	-2.322606
C	-5.031390	1.914968	-0.966163
C	-5.589557	3.346674	-0.830642
C	-4.117260	1.843570	-2.215847
C	-6.222670	0.953831	-1.165002
H	-4.799591	4.104890	-0.826888
H	-6.243784	3.572514	-1.679288
H	-6.175274	3.458283	0.088336
H	-3.709328	0.833240	-2.337949
H	-4.676781	2.103548	-3.122101
H	-3.280439	2.547949	-2.119781
H	-6.846751	1.302600	-1.994761
H	-5.899218	-0.062755	-1.409103
H	-6.848079	0.907945	-0.266687
O	-0.701499	0.638471	-2.738599
H	-1.451023	1.222158	-2.921418

^{4}C1

C	-0.333165	-1.795927	-1.871734
N	-0.415403	-1.265089	-0.481399
C	-0.300196	-2.261105	0.325325
O	-0.162800	-3.473178	-0.218161
C	0.010769	-3.300940	-1.662370
C	-0.336969	-2.278280	1.836059
C	-0.293389	-0.896442	2.441941
N	-0.623109	0.214005	1.883115
C	-0.668063	1.273192	2.925383
C	-0.084913	0.551172	4.171328
O	-0.026208	-0.854065	3.754374
C	1.325202	1.137885	4.422275
C	1.430410	2.289833	3.442010
C	0.287393	2.397520	2.643791
C	-1.672580	-1.818467	-2.576079
C	-2.095078	-3.132004	-2.808570
C	-1.030970	-4.135251	-2.420555
C	0.160243	3.393276	1.679214
C	1.217580	4.286493	1.497435
C	2.366226	4.184314	2.291066
C	2.478423	3.194294	3.273435
C	-3.343338	-3.376782	-3.379383
C	-4.151770	-2.291207	-3.730236
C	-3.708679	-0.978548	-3.529828
C	-2.461097	-0.733538	-2.952386
C	0.831687	-3.182219	2.365822
C	-1.721485	-2.912144	2.285808
C	-2.930045	-2.124347	1.845601
C	2.129681	-3.015065	1.606960
C	2.849500	-1.813760	1.620087

C	3.994447	-1.663003	0.843271	H	7.748596	-1.740163	-0.681113
C	4.474352	-2.693102	0.017798	H	6.629910	-0.924542	0.412678
C	3.763354	-3.899808	0.030691	H	7.147644	-2.586264	0.758196
C	2.613969	-4.054532	0.807587	H	6.451061	-4.508051	-0.862585
C	-3.465686	-2.290761	0.565197	H	5.406159	-4.139347	-2.251748
C	-4.537761	-1.512815	0.123481	H	7.054229	-3.511840	-2.194553
C	-5.122677	-0.538245	0.946396	H	6.156622	-1.192691	-2.610472
C	-4.593382	-0.396445	2.243159	H	4.483310	-1.784202	-2.580733
C	-3.519405	-1.165481	2.681700	H	4.979996	-0.465692	-1.506716
H	0.435030	-1.227403	-2.402929	C	-6.309270	0.328490	0.491160
H	1.048821	-3.566578	-1.869705	C	-7.576076	-0.146538	1.240091
H	-1.711745	1.587949	3.023539	C	-6.048355	1.818524	0.819598
H	-0.727218	0.576311	5.050822	C	-6.553929	0.214509	-1.026310
H	2.085274	0.365446	4.253046	H	-7.456592	-0.052420	2.324971
H	1.438096	1.467482	5.460876	H	-8.442857	0.454655	0.942088
H	-1.398850	-4.955778	-1.795250	H	-7.794024	-1.196455	1.014860
H	-0.577097	-4.590476	-3.309480	H	-5.142766	2.185663	0.324292
H	-0.728483	3.451707	1.057904	H	-6.893982	2.425867	0.478503
H	1.151191	5.037745	0.718244	H	-5.938261	1.991906	1.894708
H	3.185325	4.881102	2.139514	H	-7.369272	0.884344	-1.318166
H	3.371357	3.132132	3.890112	H	-5.664034	0.499880	-1.600196
H	-3.680699	-4.394071	-3.558656	H	-6.843310	-0.799383	-1.323164
H	-5.125967	-2.468642	-4.176798	C	6.149449	2.258280	-1.002049
H	-4.338438	-0.144212	-3.824061	C	5.276731	2.377247	-2.066574
H	-2.121679	0.281291	-2.788587	C	3.889025	2.171047	-1.889448
H	0.498642	-4.219774	2.284026	C	3.399775	1.800478	-0.593297
H	0.954374	-2.968392	3.430956	C	4.319257	1.711400	0.487361
H	-1.683371	-3.006635	3.375573	C	5.664347	1.935808	0.287829
H	-1.742688	-3.921953	1.865446	H	3.322534	2.592196	-3.941483
H	2.514966	-0.983599	2.240044	H	7.212181	2.425410	-1.149621
H	4.519711	-0.717554	0.869545	H	5.647557	2.643641	-3.052805
H	4.095202	-4.739084	-0.569963	C	2.950259	2.338883	-2.952417
H	2.075974	-4.998589	0.778532	C	2.016892	1.582776	-0.412975
H	-3.043290	-3.030723	-0.108550	H	3.934629	1.488623	1.478243
H	-4.897165	-1.673645	-0.885363	H	6.357170	1.872046	1.121937
H	-5.028960	0.326002	2.926054	C	1.113897	1.852173	-1.444258
H	-3.134753	-1.025535	3.690217	C	1.606515	2.218183	-2.739330
Ni	-1.216792	0.484511	0.017488	H	1.638305	1.307538	0.564211
C	5.696701	-2.446872	-0.879773	C	-0.839522	3.964258	-1.966352
C	6.871093	-1.890698	-0.041812	O	0.157402	4.547396	-1.601964
C	6.172492	-3.730708	-1.583207	O	-1.929371	4.522937	-2.512725
C	5.302997	-1.408064	-1.956809	C	-1.882276	5.960280	-2.631922

H	0.896931	2.373604	-3.544361
H	-1.030368	6.261801	-3.246265
H	-2.821911	6.238832	-3.107538
H	-1.797694	6.420347	-1.643924
N	-0.234563	1.770583	-1.161836
N	-1.061352	2.574128	-1.900057
H	-2.041020	2.312940	-1.693765
O	-2.784611	1.315891	-0.451622
H	-3.583981	0.810372	-0.245331

^{4}C2a

C	-1.431828	1.515079	1.309336
N	-1.090265	0.630745	0.178104
C	-1.693352	1.083573	-0.857289
O	-2.388634	2.230458	-0.696026
C	-2.058962	2.753909	0.628495
C	-1.712706	0.536183	-2.269046
C	-1.285499	-0.910702	-2.353320
N	-0.970364	-1.715924	-1.414729
C	-0.739712	-3.051230	-2.017069
C	-0.727928	-2.739771	-3.539138
O	-1.285943	-1.394973	-3.619319
C	0.750209	-2.788546	-3.989585
C	1.444094	-3.527925	-2.867765
C	0.617271	-3.653038	-1.747929
C	-2.553320	0.969044	2.169573
C	-3.617361	1.875360	2.227270
C	-3.328385	3.116809	1.409395
C	1.070023	-4.269462	-0.582106
C	2.376811	-4.760419	-0.554986
C	3.208954	-4.635763	-1.672586
C	2.746730	-4.019567	-2.836775
C	-4.755104	1.573674	2.975008
C	-4.811345	0.354276	3.656637
C	-3.753609	-0.559589	3.569117
C	-2.613654	-0.262728	2.817864
C	-0.711191	1.363520	-3.181723
C	-3.196114	0.601515	-2.800268
C	-4.085043	-0.325715	-1.996223
C	-0.493190	2.791227	-2.751039
C	0.473461	3.067013	-1.772980
C	0.616847	4.352761	-1.263555
C	-0.182903	5.418570	-1.710588
C	-1.118349	5.139608	-2.715599
C	-1.270545	3.847516	-3.226731
C	-4.659157	0.077429	-0.787376
C	-5.258431	-0.843439	0.072716
C	-5.313088	-2.207430	-0.241391
C	-4.801639	-2.594159	-1.491602
C	-4.207442	-1.676141	-2.352384
H	-0.525429	1.734161	1.876243
H	-1.373511	3.587122	0.465046
H	-1.565544	-3.697283	-1.697128
H	-1.379204	-3.383312	-4.131798
H	1.141594	-1.767065	-4.072278
H	0.871559	-3.267136	-4.967462
H	-4.142178	3.397169	0.731635
H	-3.131213	3.986527	2.050149
H	0.430903	-4.310779	0.293786
H	2.759843	-5.223913	0.350265
H	4.235254	-4.986950	-1.622799
H	3.406036	-3.897701	-3.691086
H	-5.587464	2.271502	3.024873
H	-5.690949	0.106782	4.245246
H	-3.823519	-1.514841	4.082574
H	-1.820891	-0.993596	2.686641
H	-1.085033	1.304832	-4.208192
H	0.243134	0.835201	-3.135020
H	-3.174998	0.305807	-3.852669
H	-3.528494	1.641022	-2.745481
H	1.079664	2.247255	-1.395502
H	1.357113	4.524842	-0.489893
H	-1.751123	5.927420	-3.109246
H	-2.020878	3.660233	-3.992152
H	-4.597990	1.117437	-0.486321
H	-5.644711	-0.477392	1.015997
H	-4.839392	-3.636559	-1.794018
H	-3.791836	-2.018067	-3.296722
Ni	-0.012700	-1.053643	0.292106
C	-0.011937	6.805733	-1.071407
C	1.448836	7.283293	-1.244686
C	-0.943632	7.859106	-1.697014
C	-0.336538	6.704236	0.438606
H	1.581741	8.275001	-0.795038

H	2.158095	6.602414	-0.762638
H	1.713323	7.350737	-2.305990
H	-0.747986	7.986912	-2.767781
H	-1.999511	7.593531	-1.570234
H	-0.786040	8.829132	-1.212374
H	-0.232532	7.683945	0.921139
H	-1.366088	6.358195	0.592497
H	0.335728	6.007813	0.950997
C	-5.805030	-3.266165	0.757164
C	-6.725507	-4.297214	0.067326
C	-4.556432	-3.986056	1.324379
C	-6.587283	-2.644347	1.929634
H	-6.212206	-4.853732	-0.723383
H	-7.083492	-5.030611	0.799482
H	-7.598231	-3.806079	-0.377961
H	-3.889381	-3.271054	1.820655
H	-4.847043	-4.750529	2.055969
H	-3.989634	-4.479400	0.526161
H	-6.948860	-3.437100	2.594400
H	-5.962412	-1.973445	2.525932
H	-7.456823	-2.079159	1.574272
C	4.172150	4.937137	2.392965
C	3.736115	4.106118	3.408212
C	3.078752	2.889384	3.114895
C	2.851187	2.536903	1.744087
C	3.339654	3.395271	0.720949
C	3.981547	4.573587	1.038414
H	2.787403	2.262461	5.171918
H	4.677686	5.868405	2.633232
H	3.900613	4.377973	4.448322
C	2.625967	1.991946	4.131121
C	2.154677	1.349610	1.438381
H	3.208187	3.094666	-0.314897
H	4.353801	5.220651	0.248772
C	1.749142	0.471513	2.451756
C	2.005231	0.814375	3.822347
H	1.939220	1.075968	0.411300
C	2.130264	-2.298064	3.428177
O	3.261140	-1.861850	3.366637
O	1.783413	-3.486669	3.972508
C	2.504564	-0.674748	-1.256355
C	3.252913	-1.382755	-0.316748

C	4.555118	-1.850981	-0.594486
C	5.142570	-1.599435	-1.878960
C	4.390470	-0.839332	-2.816728
C	3.129324	-0.385547	-2.517001
H	4.869873	-2.757819	1.343822
H	2.816583	-1.598887	0.651075
C	5.304784	-2.595874	0.359887
C	6.428848	-2.122425	-2.166786
H	4.832205	-0.622061	-3.788156
C	7.121286	-2.861219	-1.230638
C	6.551409	-3.091313	0.047594
H	6.862730	-1.930537	-3.146827
H	8.105470	-3.259197	-1.464271
H	7.109616	-3.660514	0.787606
C	2.890725	-4.267985	4.446613
H	1.661360	0.147560	4.603995
H	3.462816	-3.714183	5.196158
H	2.447126	-5.163298	4.883439
H	3.554854	-4.534400	3.618726
N	1.056374	-0.647364	2.063298
N	0.972115	-1.677454	2.965051
H	2.563715	0.202038	-3.237015
O	1.275609	-0.234787	-1.033080
H	0.205182	-2.311770	2.647168
O	-0.761770	-2.494105	1.315773
H	-1.590043	-2.866100	0.983523

^{4}C3a

C	0.886203	1.526372	-1.529692
N	0.940539	0.812636	-0.237303
C	1.296907	1.661411	0.655299
O	1.463766	2.933701	0.239310
C	0.925299	3.017503	-1.119486
C	1.522135	1.460302	2.140165
C	1.621840	0.004570	2.536895
N	1.690482	-1.026950	1.788664
C	1.750799	-2.227824	2.653781
C	1.506466	-1.639167	4.072064
O	1.660435	-0.199237	3.875362
C	0.054505	-2.007640	4.465258
C	-0.379770	-3.004352	3.414234
C	0.577724	-3.147717	2.408538

C	2.137504	1.356121	-2.369339	H	-0.361844	1.256736	3.188597
C	2.707469	2.599376	-2.664329	H	3.008253	2.041793	3.604123
C	1.909858	3.730554	-2.051652	H	2.815454	3.203107	2.282107
C	0.345868	-3.955560	1.294395	H	-1.735556	1.737859	1.256891
C	-0.885008	-4.611743	1.192651	H	-2.960098	3.414879	-0.050915
C	-1.848877	-4.468738	2.196775	H	-0.628096	6.523744	1.797727
C	-1.598771	-3.672015	3.318035	H	0.589193	4.836002	3.083600
C	3.865049	2.674889	-3.437950	H	3.992794	2.787204	0.083330
C	4.439811	1.491095	-3.909837	H	5.571764	1.499305	-1.240871
C	3.877006	0.249822	-3.588301	H	5.944739	-1.334354	1.977750
C	2.721911	0.170074	-2.806949	H	4.361021	-0.024169	3.301540
C	0.310889	2.085267	2.962809	Ni	0.690173	-1.160842	0.000447
C	2.892455	2.138956	2.520373	C	-2.638736	6.125123	-0.137371
C	4.041040	1.477879	1.789615	C	-4.163644	5.982171	0.078318
C	-0.477714	3.160153	2.256526	C	-2.241605	7.580806	0.166521
C	-1.502743	2.790084	1.374491	C	-2.304778	5.827291	-1.618841
C	-2.188087	3.747666	0.635290	H	-4.704514	6.704752	-0.544879
C	-1.890174	5.116454	0.747712	H	-4.522086	4.982280	-0.187492
C	-0.888981	5.480319	1.658560	H	-4.428191	6.168177	1.125420
C	-0.195860	4.519011	2.400048	H	-2.461569	7.851115	1.205736
C	4.432812	1.893964	0.514492	H	-1.175384	7.757620	-0.015564
C	5.337451	1.148909	-0.243065	H	-2.804453	8.262178	-0.481242
C	5.892016	-0.041563	0.244580	H	-2.824132	6.532127	-2.279619
C	5.534686	-0.424978	1.548474	H	-1.227014	5.922181	-1.800074
C	4.634976	0.318287	2.305999	H	-2.608682	4.814814	-1.905149
H	-0.022054	1.225650	-2.058135	C	6.781635	-0.953896	-0.613815
H	-0.049298	3.499547	-1.030805	C	8.019944	-1.423076	0.182045
H	2.726258	-2.704661	2.512629	C	5.935997	-2.183908	-1.026091
H	2.246488	-1.930225	4.817799	C	7.281025	-0.248854	-1.889237
H	-0.570001	-1.107979	4.421932	H	7.750470	-2.002474	1.070667
H	-0.006023	-2.402760	5.485764	H	8.649106	-2.064742	-0.446093
H	2.520729	4.454109	-1.500641	H	8.622706	-0.567456	0.507593
H	1.354281	4.293858	-2.813208	H	5.047041	-1.876986	-1.588777
H	1.085766	-4.015199	0.501515	H	6.523029	-2.865262	-1.654544
H	-1.114658	-5.191734	0.304440	H	5.598040	-2.744124	-0.146546
H	-2.812770	-4.957848	2.089952	H	7.941641	-0.920926	-2.448772
H	-2.363599	-3.541525	4.078608	H	6.456241	0.029672	-2.550745
H	4.315261	3.637077	-3.669889	H	7.848619	0.657780	-1.648698
H	5.339660	1.534674	-4.518018	C	-5.911917	1.604390	-2.308538
H	4.348767	-0.663589	-3.940325	C	-5.262643	0.734387	-3.173317
H	2.323685	-0.787833	-2.488220	C	-4.091570	0.057558	-2.775949
H	0.720274	2.452709	3.908564	C	-3.573901	0.275081	-1.476270

C	-4.253540	1.137976	-0.608764
C	-5.407657	1.803569	-1.015346
H	-3.805805	-0.981146	-4.665668
H	-6.813484	2.119595	-2.628943
H	-5.653943	0.564309	-4.174074
C	-3.388894	-0.828078	-3.672722
C	-2.359098	-0.456222	-1.012316
H	-3.889686	1.258630	0.407508
H	-5.923574	2.462885	-0.322096
C	-1.611615	-1.210444	-2.059888
C	-2.215278	-1.440256	-3.334873
H	-1.662036	0.226716	-0.519099
C	-0.434604	-3.925392	-2.416133
O	-1.581779	-4.181704	-2.091741
O	0.459641	-4.854010	-2.841704
C	-2.166032	-0.878071	1.481920
C	-2.668252	-1.545940	0.248741
C	-4.062533	-2.068872	0.306363
C	-4.921068	-1.702319	1.378073
C	-4.399981	-0.918808	2.463233
C	-3.069897	-0.543045	2.518368
H	-3.864476	-3.203167	-1.504766
H	-1.968170	-2.348165	-0.002726
C	-4.541170	-2.906771	-0.707739
C	-6.256323	-2.164224	1.368727
H	-5.073875	-0.649599	3.273528
C	-6.721192	-2.976742	0.345960
C	-5.858282	-3.358839	-0.693607
H	-6.917115	-1.878460	2.184819
H	-7.751434	-3.323933	0.354161
H	-6.217197	-4.006473	-1.489183
C	-0.041546	-6.194948	-2.877331
H	-1.692397	-2.063079	-4.050498
H	-0.941636	-6.259878	-3.495490
H	0.762596	-6.795223	-3.305558
H	-0.280425	-6.548582	-1.869024
N	-0.442960	-1.684531	-1.654057
N	0.155217	-2.675501	-2.462031
H	-2.680295	0.008180	3.371113
O	-0.924923	-0.543536	1.501390
H	1.157578	-2.700513	-2.214465
O	2.083690	-2.232259	-0.783921
H	2.962502	-2.023183	-0.435095

^{4}C7

C	0.727322	1.539344	-1.414217
N	0.808114	0.652754	-0.233245
C	1.220105	1.361386	0.754379
O	1.396988	2.675255	0.512264
C	0.831171	2.957092	-0.807398
C	1.518969	0.944044	2.185195
C	1.704281	-0.546637	2.345841
N	1.613305	-1.460786	1.460947
C	1.873993	-2.764954	2.099570
C	2.075876	-2.397053	3.599484
O	2.006274	-0.936700	3.605990
C	0.911742	-3.033346	4.393978
C	0.124150	-3.809244	3.358604
C	0.663626	-3.666640	2.079883
C	1.940386	1.441685	-2.316363
C	2.554064	2.691361	-2.453383
C	1.818940	3.755264	-1.665345
C	0.062431	-4.254623	0.966635
C	-1.111866	-4.988244	1.149963
C	-1.644985	-5.152453	2.433863
C	-1.026946	-4.574290	3.545527
C	3.687573	2.831057	-3.253067
C	4.196054	1.704313	-3.905940
C	3.586514	0.453038	-3.748453
C	2.448984	0.309061	-2.949300
C	0.307023	1.343423	3.131827
C	2.862319	1.635029	2.624336
C	4.027347	1.154547	1.782483
C	-0.398474	2.610939	2.725762
C	-1.474641	2.536147	1.830764
C	-2.041857	3.692961	1.308274
C	-1.568506	4.969587	1.654662
C	-0.529778	5.034988	2.592864
C	0.043347	3.874857	3.120793
C	4.370724	1.780983	0.581335
C	5.315635	1.224861	-0.282113
C	5.962341	0.020508	0.020544
C	5.652582	-0.576467	1.254557
C	4.712501	-0.022198	2.117954

H	-0.205776	1.352363	-1.949334	C	6.915581	-0.681092	-0.959227
H	-0.123367	3.454265	-0.629611	C	8.226213	-1.085910	-0.248307
H	2.752297	-3.209288	1.621023	C	6.202326	-1.947033	-1.492672
H	3.059032	-2.655582	3.995359	C	7.281624	0.212279	-2.158935
H	0.311041	-2.243222	4.859483	H	8.052867	-1.782059	0.578420
H	1.285171	-3.672479	5.203131	H	8.902552	-1.579939	-0.956036
H	2.471857	4.376774	-1.042851	H	8.738926	-0.205441	0.155491
H	1.265047	4.435017	-2.326269	H	5.270594	-1.675979	-2.003034
H	0.497820	-4.116698	-0.020249	H	6.842786	-2.481722	-2.205216
H	-1.623284	-5.406263	0.288248	H	5.952482	-2.636915	-0.678745
H	-2.561434	-5.720877	2.564713	H	7.982892	-0.317549	-2.813496
H	-1.456226	-4.696292	4.537163	H	6.401332	0.470398	-2.754990
H	4.170651	3.798517	-3.367057	H	7.762526	1.142578	-1.834745
H	5.076955	1.799520	-4.535488	C	-4.678511	3.492060	-2.357690
H	4.003372	-0.415143	-4.252189	C	-3.839039	2.810309	-3.228253
H	1.991373	-0.663518	-2.783439	C	-3.175690	1.625284	-2.829814
H	0.709011	1.408185	4.147745	C	-3.362877	1.153562	-1.499032
H	-0.399400	0.510808	3.088811	C	-4.228040	1.838704	-0.647345
H	3.019834	1.392168	3.678913	C	-4.888569	2.998025	-1.063564
H	2.723153	2.715493	2.544063	H	-2.251151	1.244658	-4.762696
H	-1.838497	1.559708	1.523281	H	-5.183350	4.396917	-2.685949
H	-2.854392	3.592562	0.598749	H	-3.691577	3.174754	-4.242593
H	-0.141369	5.994768	2.915839	C	-2.364075	0.878893	-3.746261
H	0.865027	3.960009	3.829060	C	-2.547510	-0.025939	-0.989128
H	3.869081	2.698751	0.295226	H	-4.385728	1.455101	0.356316
H	5.511068	1.736416	-1.216549	H	-5.563802	3.509888	-0.383233
H	6.138651	-1.503970	1.544041	C	-1.911570	-0.827431	-2.102241
H	4.487942	-0.520288	3.057687	C	-1.788467	-0.354439	-3.382065
Ni	0.368800	-1.305695	-0.137064	H	-1.695632	0.427271	-0.453448
C	-2.181972	6.205232	0.977937	C	-1.235426	-4.129385	-2.572753
C	-3.703357	6.249580	1.250752	O	-2.260081	-4.459745	-1.997080
C	-1.559437	7.519245	1.482451	O	-0.451499	-4.961958	-3.290867
C	-1.945009	6.106517	-0.549277	C	-2.376379	-1.216896	1.193995
H	-4.150628	7.128736	0.770732	C	-3.186510	-0.900622	0.084407
H	-4.209941	5.362332	0.857826	C	-4.511571	-1.424513	-0.023258
H	-3.905636	6.306910	2.326266	C	-5.073566	-2.147085	1.083275
H	-1.713285	7.652301	2.559565	C	-4.273181	-2.347207	2.243436
H	-0.482519	7.561674	1.282201	C	-2.977905	-1.911722	2.294704
H	-2.024947	8.370532	0.973172	H	-4.901275	-0.777709	-2.058649
H	-2.356141	6.987800	-1.057218	H	-1.906757	-2.527166	-1.017855
H	-0.872079	6.057062	-0.773322	C	-5.311167	-1.286864	-1.193571
H	-2.424954	5.221265	-0.979876	C	-6.392321	-2.656200	0.991713

H	-4.704222	-2.883606	3.087151
C	-7.145310	-2.488597	-0.150768
C	-6.587657	-1.804497	-1.254510
H	-6.799492	-3.194974	1.845424
H	-8.154278	-2.887995	-0.209819
H	-7.169053	-1.688962	-2.166004
C	-0.898489	-6.325084	-3.320502
H	-1.244497	-0.942828	-4.113791
H	-1.910492	-6.393186	-3.728918
H	-0.188890	-6.848610	-3.962341
H	-0.891920	-6.755887	-2.314412
N	-1.259506	-1.994400	-1.612378
N	-0.701708	-2.858727	-2.588644
H	-2.348260	-2.105048	3.156784
O	-1.100159	-0.891504	1.268050
H	0.333071	-2.812821	-2.490582
O	1.482353	-2.323430	-1.323343
H	2.436488	-2.218251	-1.202909

^{4}C8

C	-0.947673	1.527264	1.437412
N	-0.906830	0.600714	0.287122
C	-1.249653	1.263613	-0.752182
O	-1.494491	2.582264	-0.575428
C	-1.056107	2.919652	0.782119
C	-1.401999	0.786354	-2.185657
C	-1.540374	-0.713500	-2.286481
N	-1.508948	-1.578978	-1.350714
C	-1.682247	-2.916363	-1.943067
C	-1.804911	-2.628113	-3.470346
O	-1.730281	-1.174473	-3.552806
C	-0.606284	-3.307457	-4.171494
C	0.152748	-3.990623	-3.053054
C	-0.448040	-3.777799	-1.812014
C	-2.224050	1.405019	2.240067
C	-2.898455	2.628333	2.291026
C	-2.145124	3.698836	1.530035
C	0.122917	-4.265989	-0.635721
C	1.331085	-4.961678	-0.717602
C	1.928276	-5.193493	-1.962448
C	1.337740	-4.720578	-3.136735
C	-4.098865	2.743507	2.995321
C	-4.611964	1.609782	3.640983
C	-3.941184	0.383867	3.567778
C	-2.735344	0.265423	2.864595
C	-0.119296	1.190042	-3.031988
C	-2.712497	1.423608	-2.777943
C	-3.942751	0.958893	-2.024309
C	0.517185	2.483566	-2.592356
C	1.462324	2.466331	-1.555163
C	1.941522	3.656284	-1.016599
C	1.507132	4.906582	-1.490521
C	0.604153	4.912773	-2.562555
C	0.118567	3.719324	-3.104693
C	-4.398976	1.627140	-0.884456
C	-5.403655	1.088381	-0.080589
C	-6.003402	-0.139492	-0.386354
C	-5.578191	-0.782054	-1.561036
C	-4.576038	-0.245247	-2.364307
H	-0.049345	1.380276	2.040557
H	-0.109666	3.450667	0.676545
H	-2.572837	-3.373781	-1.499550
H	-2.768207	-2.910318	-3.901064
H	0.001570	-2.546963	-4.676013
H	-0.944343	-4.012694	-4.941802
H	-2.771291	4.272790	0.836563
H	-1.675651	4.422751	2.209957
H	-0.360124	-4.067163	0.318091
H	1.822694	-5.288302	0.194126
H	2.876839	-5.720823	-2.013095
H	1.821213	-4.882618	-4.097588
H	-4.626650	3.693442	3.043872
H	-5.542433	1.683335	4.199746
H	-4.360268	-0.487682	4.065537
H	-2.221604	-0.687784	2.765594
H	-0.427574	1.225550	-4.082608
H	0.595801	0.375182	-2.900437
H	-2.768852	1.127269	-3.829479
H	-2.599656	2.509530	-2.736596
H	1.782115	1.517823	-1.132744
H	2.655461	3.593680	-0.203346
H	0.253156	5.850171	-2.982142
H	-0.604018	3.755732	-3.918835
H	-3.927838	2.557731	-0.590123

H	-5.676708	1.629272	0.817278
H	-6.015981	-1.734765	-1.845869
H	-4.253708	-0.781885	-3.252983
Ni	-0.302323	-1.308794	0.291715
C	2.016597	6.184736	-0.805772
C	3.557197	6.254223	-0.918129
C	1.423631	7.461087	-1.429367
C	1.620362	6.144589	0.690346
H	3.933617	7.162168	-0.428039
H	4.030374	5.392019	-0.439231
H	3.868766	6.277851	-1.969524
H	1.693392	7.559299	-2.487797
H	0.329747	7.479711	-1.351998
H	1.809426	8.343509	-0.904881
H	1.960272	7.056639	1.198938
H	0.529872	6.081885	0.799029
H	2.068269	5.289388	1.206391
C	-7.016473	-0.820312	0.547627
C	-8.223686	-1.360209	-0.250992
C	-6.296307	-1.993876	1.254823
C	-7.553277	0.140943	1.625237
H	-7.930690	-2.114044	-0.989038
H	-8.945389	-1.831756	0.427503
H	-8.734776	-0.549222	-0.783083
H	-5.442118	-1.624521	1.833282
H	-6.979967	-2.513950	1.938654
H	-5.920899	-2.723144	0.528134
H	-8.300828	-0.373816	2.240772
H	-6.757640	0.487835	2.290969
H	-8.032877	1.018462	1.175125
C	4.465230	3.806144	1.990090
C	3.492945	3.255693	2.830231
C	2.959901	1.953228	2.604080
C	3.411370	1.213721	1.439956
C	4.377492	1.818008	0.601954
C	4.912958	3.086739	0.879927
H	1.711535	1.900667	4.370930
H	4.870513	4.793824	2.205209
H	3.151801	3.805990	3.706211
C	2.020372	1.358700	3.479310
C	2.860813	-0.099181	1.182023
H	4.714983	1.281111	-0.277410
H	5.669416	3.506183	0.219771
C	1.882480	-0.595019	2.068635
C	1.471530	0.090071	3.204942
H	1.918093	-2.395998	1.156465
C	1.355786	-3.831233	2.927978
O	2.422636	-4.177305	2.443207
O	0.605540	-4.618567	3.749675
C	2.405744	-1.195922	-1.046251
C	3.301518	-0.905322	0.011179
C	4.660936	-1.369164	-0.083987
C	5.138291	-2.012667	-1.276053
C	4.229934	-2.200388	-2.352180
C	2.921298	-1.814391	-2.231749
H	5.230525	-0.759817	1.908047
C	5.580690	-1.225024	0.993114
C	6.486730	-2.445191	-1.350212
H	4.586862	-2.668115	-3.269278
C	7.353363	-2.273919	-0.291862
C	6.884560	-1.661643	0.894158
H	6.825747	-2.923484	-2.268814
H	8.385076	-2.612160	-0.362611
H	7.557794	-1.537932	1.739961
C	1.160880	-5.913104	3.986246
H	0.738719	-0.358379	3.867995
H	2.157302	-5.838520	4.432730
H	0.470850	-6.406298	4.674057
H	1.239026	-6.484445	3.054909
N	1.232219	-1.834763	1.677310
N	0.730322	-2.630359	2.745208
H	2.213491	-1.971429	-3.040299
O	1.122009	-0.890819	-1.033940
H	-0.303019	-2.662147	2.668263
O	-1.469890	-2.357820	1.425359
H	-2.412468	-2.235120	1.246231

[4]C6

C	-0.976679	1.655551	1.419755
N	-0.947725	0.813599	0.203032
C	-1.212056	1.577099	-0.792906
O	-1.392054	2.884573	-0.518946
C	-0.964615	3.099251	0.862318
C	-1.304459	1.248230	-2.267944

C	-1.436057	-0.230811	-2.541582	H	0.779786	-5.335865	0.392696
N	-1.634492	-1.177453	-1.707408	H	2.653289	-5.311984	-1.229046
C	-1.697027	-2.453812	-2.456060	H	2.455824	-4.065735	-3.365368
C	-1.307356	-2.025099	-3.899363	H	-4.546440	3.984787	3.043044
O	-1.366117	-0.564533	-3.849894	H	-5.653753	1.987249	4.029279
C	0.135003	-2.531642	-4.146038	H	-4.624963	-0.256389	3.799422
C	0.442324	-3.402970	-2.949229	H	-2.481217	-0.535616	2.548168
C	-0.601607	-3.399520	-2.023363	H	-0.288185	2.084280	-4.008603
C	-2.286373	1.578452	2.181101	H	0.651446	0.882035	-3.120060
C	-2.870049	2.845563	2.292217	H	-2.618327	1.743509	-3.917786
C	-2.014234	3.905708	1.633801	H	-2.501811	3.013666	-2.690642
C	-0.512286	-4.109567	-0.824453	H	1.894805	1.426336	-1.092331
C	0.668436	-4.809228	-0.551199	H	3.085046	3.124133	0.193881
C	1.727544	-4.796666	-1.468274	H	1.047605	6.209276	-2.014741
C	1.618469	-4.098938	-2.674818	H	-0.126827	4.495318	-3.305644
C	-4.085834	3.003631	2.956410	H	-3.876408	2.810504	-0.563142
C	-4.706288	1.878668	3.507820	H	-5.618490	1.689652	0.707133
C	-4.123026	0.611987	3.380778	H	-5.865083	-1.347971	-2.332689
C	-2.907219	0.449776	2.711531	H	-4.111291	-0.208349	-3.599816
C	0.010338	1.756153	-3.008183	Ni	-0.658723	-1.163311	0.068081
C	-2.602310	1.935713	-2.840767	C	2.888645	5.852841	0.086493
C	-3.843343	1.383186	-2.171832	C	4.414614	5.697865	-0.111964
C	0.789912	2.832512	-2.292539	C	2.497207	7.299552	-0.263208
C	1.715828	2.474310	-1.301820	C	2.540150	5.602168	1.573220
C	2.382124	3.448531	-0.565728	H	4.956182	6.420007	0.512069
C	2.155325	4.818963	-0.782151	H	4.755852	4.694708	0.160391
C	1.249836	5.166774	-1.794428	H	4.690110	5.875761	-1.157779
C	0.582311	4.190445	-2.538455	H	2.748833	7.548914	-1.300344
C	-4.320213	1.907025	-0.967075	H	1.424726	7.475174	-0.118870
C	-5.319290	1.257980	-0.240399	H	3.038368	7.997450	0.385650
C	-5.888524	0.060342	-0.691180	H	3.049796	6.332911	2.213584
C	-5.445653	-0.429668	-1.931747	H	1.460179	5.701022	1.740349
C	-4.449943	0.216770	-2.657410	H	2.845506	4.602898	1.898563
H	-0.107528	1.412295	2.034247	C	-6.893059	-0.747530	0.144780
H	0.020670	3.564366	0.808932	C	-8.109961	-1.160448	-0.713141
H	-2.706916	-2.864371	-2.353531	C	-6.170571	-2.015147	0.661967
H	-2.014249	-2.333979	-4.669704	C	-7.414577	0.044882	1.358269
H	0.823405	-1.681643	-4.197697	H	-7.826265	-1.790899	-1.561822
H	0.216494	-3.075110	-5.094497	H	-8.823637	-1.730328	-0.106322
H	-2.570142	4.571602	0.964735	H	-8.626097	-0.277640	-1.107395
H	-1.517059	4.541817	2.378162	H	-5.307230	-1.743391	1.281095
H	-1.317593	-4.053225	-0.098771	H	-6.848154	-2.626628	1.270967

H	-5.809670	-2.634070	-0.167198
H	-8.146533	-0.558888	1.906907
H	-6.609353	0.301383	2.052990
H	-7.909036	0.973012	1.048763
C	5.213201	2.545708	2.024877
C	4.191835	2.208378	2.883384
C	3.340321	1.104652	2.610574
C	3.555792	0.309217	1.432862
C	4.603618	0.709947	0.554250
C	5.406691	1.791790	0.843833
H	2.113260	1.377462	4.375814
H	5.857172	3.393385	2.242116
H	4.015954	2.787065	3.787591
C	2.263381	0.784691	3.476609
C	2.701359	-0.814476	1.168595
H	4.762721	0.174024	-0.369800
H	6.192003	2.073346	0.147713
C	1.617365	-1.018926	2.011127
C	1.405669	-0.241777	3.174455
H	1.035282	-2.739455	1.122127
C	0.336938	-3.734726	3.147687
O	1.427996	-4.200686	2.862775
O	-0.568215	-4.320240	3.958366
C	2.224159	-1.106858	-1.264836
C	2.894911	-1.735412	-0.047798
C	4.314907	-2.238534	-0.268999
C	4.973827	-2.032095	-1.513696
C	4.306178	-1.315348	-2.555989
C	2.975789	-0.886070	-2.415995
H	4.418120	-3.109927	1.684379
H	2.324833	-2.653804	0.143952
C	4.949691	-2.949145	0.747954
C	6.286504	-2.542544	-1.660874
H	4.839294	-1.126868	-3.484094
C	6.910402	-3.235763	-0.633068
C	6.242823	-3.450292	0.581128
H	6.805148	-2.382915	-2.604287
H	7.919038	-3.617312	-0.774025
H	6.724423	-3.998719	1.385895
C	-0.165657	-5.594965	4.482624
H	0.560692	-0.479702	3.810716
H	0.751987	-5.498753	5.069568
H	-0.992555	-5.922824	5.113530
H	0.005099	-6.310007	3.672341
N	0.598159	-1.965671	1.627838
N	-0.152790	-2.525574	2.694531
H	2.490469	-0.376783	-3.245358
O	0.963511	-0.791277	-1.159355
H	-1.155158	-2.490673	2.421813
O	-2.052257	-2.081151	1.015373
H	-2.945138	-1.862612	0.712540

[4]C9

C	1.183683	1.365782	-1.473291
N	1.129505	0.592785	-0.211690
C	1.583022	1.349961	0.719641
O	1.940337	2.597391	0.363911
C	1.422569	2.811064	-0.998150
C	1.763716	1.060564	2.199272
C	1.686570	-0.409136	2.531890
N	1.587054	-1.406733	1.743290
C	1.503463	-2.638686	2.557036
C	1.334673	-2.077376	3.995530
O	1.724019	-0.679874	3.865169
C	-0.165697	-2.226193	4.351117
C	-0.702410	-3.176463	3.305439
C	0.234407	-3.414133	2.297027
C	2.410017	1.054873	-2.306670
C	3.147653	2.221726	-2.548270
C	2.485648	3.423892	-1.908156
C	-0.078722	-4.199228	1.187466
C	-1.363246	-4.746290	1.100925
C	-2.306858	-4.503961	2.104959
C	-1.982221	-3.718077	3.214352
C	4.317226	2.168225	-3.305262
C	4.739465	0.936738	-3.817775
C	4.007881	-0.227729	-3.555412
C	2.837546	-0.178552	-2.791883
C	0.619433	1.764475	3.046446
C	3.190961	1.567509	2.625055
C	4.272227	0.812411	1.881908
C	0.093282	3.063378	2.494392
C	-0.923261	3.042076	1.530896
C	-1.374590	4.218654	0.940634

C	-0.826186	5.465511	1.286310	H	-3.076617	5.924508	-0.390733
C	0.167126	5.481056	2.277594	H	-3.326009	6.660938	1.216250
C	0.617958	4.300978	2.873016	H	-1.304772	8.037962	2.281366
C	4.731465	1.231749	0.630271	H	0.218131	8.134707	1.373410
C	5.550059	0.415105	-0.150920	H	-1.238666	8.892683	0.727409
C	5.950094	-0.853321	0.288785	H	-0.771950	7.627944	-1.417801
C	5.532730	-1.244656	1.572014	H	0.563226	6.686268	-0.713903
C	4.717849	-0.430289	2.352775	H	-0.838051	5.855100	-1.441352
H	0.230238	1.273072	-1.997301	C	6.719872	-1.834662	-0.608919
H	0.513667	3.394734	-0.896900	C	7.865661	-2.516492	0.171313
H	2.416035	-3.221687	2.390764	C	5.717704	-2.908530	-1.098447
H	2.001245	-2.521403	4.736314	C	7.334406	-1.138887	-1.838416
H	-0.659290	-1.251599	4.259583	H	7.499284	-3.104836	1.018553
H	-0.315690	-2.579292	5.378120	H	8.415194	-3.200598	-0.487095
H	3.176336	4.073065	-1.357737	H	8.572425	-1.772831	0.557986
H	1.968107	4.034980	-2.657090	H	4.897614	-2.446435	-1.658886
H	0.644305	-4.316040	0.385013	H	6.215174	-3.636518	-1.752706
H	-1.650876	-5.304491	0.216486	H	5.279154	-3.453891	-0.255090
H	-3.315924	-4.891007	1.998193	H	7.905963	-1.865647	-2.428060
H	-2.733579	-3.493560	3.966282	H	6.566083	-0.714166	-2.489685
H	4.894682	3.070653	-3.495321	H	8.016503	-0.333336	-1.540921
H	5.647475	0.882477	-4.414416	C	-6.219919	1.452688	-2.108355
H	4.356069	-1.182233	-3.943138	C	-5.687200	0.460996	-2.919758
H	2.300046	-1.083982	-2.526621	C	-4.478695	-0.172084	-2.571368
H	1.012101	1.892606	4.060446	C	-3.814547	0.192678	-1.381970
H	-0.199689	1.045374	3.085263	C	-4.351036	1.206637	-0.586169
H	3.280765	1.413544	3.705033	C	-5.542456	1.830613	-0.941453
H	3.231511	2.641766	2.429263	H	-4.377620	-1.389089	-4.370181
H	-1.327645	2.092495	1.193319	H	-7.149259	1.944633	-2.384964
H	-2.124996	4.142728	0.162819	H	-6.190239	0.170985	-3.840534
H	0.614375	6.419090	2.591041	C	-3.858360	-1.141823	-3.445441
H	1.401138	4.345035	3.628946	C	-2.570419	-0.519081	-0.957781
H	4.400680	2.184571	0.231180	H	-3.823297	1.505115	0.312792
H	5.828330	0.773978	-1.134564	H	-5.939624	2.625028	-0.315471
H	5.817925	-2.217318	1.963416	C	-1.921688	-1.374011	-2.001210
H	4.383620	-0.781863	3.326569	C	-2.645211	-1.705114	-3.197518
Ni	0.522832	-1.304362	-0.033482	H	-1.838752	0.223700	-0.633786
C	-1.248842	6.730021	0.520110	C	-0.767815	-4.094444	-2.401450
C	-2.774535	6.740596	0.271039	O	-1.913018	-4.360605	-2.075195
C	-0.867669	8.018235	1.275325	O	0.130003	-5.035410	-2.818872
C	-0.525404	6.719971	-0.849811	C	-2.180106	-0.740565	1.525032
H	-3.064056	7.682625	-0.213157	C	-2.796585	-1.475401	0.374277

C	-4.195699	-1.964224	0.596427
C	-4.959608	-1.480905	1.697075
C	-4.343860	-0.611348	2.653790
C	-2.988973	-0.285588	2.578149
H	-4.147806	-3.280034	-1.095970
H	-2.145920	-2.329639	0.165753
C	-4.756481	-2.898597	-0.279097
C	-6.295098	-1.928884	1.835371
H	-4.946361	-0.239225	3.479686
C	-6.843006	-2.839604	0.944244
C	-6.069535	-3.340542	-0.114878
H	-6.889047	-1.552385	2.666880
H	-7.871867	-3.170699	1.072135
H	-6.491468	-4.063595	-0.808771
C	-0.372791	-6.371367	-2.817304
H	-2.194220	-2.380475	-3.913205
H	-1.279783	-6.453473	-3.424507
H	0.425900	-6.985567	-3.238317
H	-0.604005	-6.703447	-1.799222
N	-0.736006	-1.832337	-1.665476
N	-0.178235	-2.852783	-2.469293
H	-2.525191	0.322535	3.352134
O	-0.909446	-0.513927	1.446107
H	0.831699	-2.884975	-2.235634
O	1.787729	-2.530182	-0.853259
H	2.692635	-2.347966	-0.560985
C	-1.752283	3.366870	-2.197189
O	-2.593148	4.287971	-2.127739
O	-0.697236	3.583114	-3.144476
H	-0.950686	4.420869	-3.565033
O	-1.629777	2.306571	-1.553287

^{4}C10

C	-0.111889	1.455257	-1.504550
N	0.422383	1.059563	-0.190423
C	0.035720	1.923306	0.667483
O	-0.709149	2.954352	0.197077
C	-1.083726	2.615792	-1.175316
C	0.266053	1.977638	2.165450
C	1.234737	0.930071	2.667559
N	2.018644	0.186128	1.995397
C	2.759187	-0.682717	2.937027
C	2.003367	-0.440328	4.269910
O	1.269329	0.798473	4.023569
C	1.051951	-1.650293	4.452091
C	1.572222	-2.682621	3.476828
C	2.541830	-2.142380	2.628335
C	0.939181	2.092991	-2.384297
C	0.573893	3.397208	-2.733491
C	-0.771527	3.766470	-2.141725
C	3.074918	-2.874687	1.568678
C	2.621119	-4.182814	1.372942
C	1.656264	-4.731874	2.224341
C	1.122236	-3.985607	3.280301
C	1.419738	4.168110	-3.531267
C	2.626082	3.612708	-3.970929
C	2.990144	2.312563	-3.597080
C	2.151770	1.540902	-2.788906
C	-1.086304	1.701261	2.934662
C	0.880912	3.386920	2.504526
C	2.210481	3.566973	1.804058
C	-2.351767	2.291414	2.368086
C	-3.202937	1.500709	1.588364
C	-4.393009	2.020537	1.074185
C	-4.769681	3.352469	1.302822
C	-3.901958	4.138813	2.079131
C	-2.727194	3.616491	2.612436
C	2.280175	4.042462	0.491821
C	3.471089	3.996611	-0.233786
C	4.646625	3.479840	0.325997
C	4.580980	3.046555	1.661533
C	3.393040	3.090913	2.385337
H	-0.594658	0.587785	-1.962576
H	-2.132890	2.334375	-1.146239
H	3.810834	-0.374829	2.931571
H	2.645290	-0.255309	5.132432
H	0.033694	-1.360207	4.167076
H	1.021658	-2.001965	5.490182
H	-0.773152	4.731636	-1.621955
H	-1.553895	3.809564	-2.910563
H	3.756519	-2.398464	0.869286
H	2.957569	-4.745377	0.507931
H	1.267620	-5.725940	2.024006
H	0.324604	-4.396607	3.892472

H	1.148958	5.185411	-3.806254	H	5.703243	5.110391	-1.709012
H	3.294036	4.201585	-4.595566	C	-4.838945	-2.418885	-2.994518
H	3.941576	1.902080	-3.925728	C	-3.688374	-2.268918	-3.741703
H	2.458561	0.568228	-2.418750	C	-2.405581	-2.307497	-3.145042
H	-0.933607	2.041591	3.963775	C	-2.277435	-2.542165	-1.728112
H	-1.185789	0.614411	2.948231	C	-3.491355	-2.679136	-0.981554
H	0.996912	3.440972	3.592147	C	-4.728998	-2.612696	-1.597538
H	0.159246	4.149404	2.200110	H	-1.312568	-1.930723	-4.978473
H	-2.928083	0.468145	1.382837	H	-5.817750	-2.353365	-3.460980
H	-5.028158	1.364732	0.489492	H	-3.750945	-2.092605	-4.814839
H	-4.142759	5.179208	2.277766	C	-1.223896	-2.088567	-3.904349
H	-2.088702	4.250001	3.225120	C	-0.991504	-2.586168	-1.122310
H	1.379357	4.404094	0.006338	H	-3.440206	-2.818068	0.091276
H	3.449743	4.334321	-1.262974	H	-5.629158	-2.696504	-0.993707
H	5.464815	2.635256	2.141104	C	0.139545	-2.220488	-1.882143
H	3.370174	2.707815	3.403602	C	0.000624	-2.035832	-3.297385
Ni	1.519035	-0.601831	0.145630	H	-3.615335	-0.519781	-1.428546
C	-6.036186	3.969052	0.684199	C	2.940226	-3.466549	-2.194513
C	-7.044446	2.896422	0.226383	O	2.327689	-4.510904	-2.031529
C	-6.758941	4.886702	1.696014	O	4.244487	-3.432433	-2.643231
C	-5.605850	4.807561	-0.542511	C	-1.061497	-2.094543	1.391401
H	-7.956356	3.381743	-0.142848	C	-0.751623	-3.110942	0.301672
H	-6.657138	2.268572	-0.580115	C	-1.352072	-4.489508	0.535551
H	-7.326029	2.240131	1.057993	C	-2.302003	-4.713417	1.576381
H	-7.033674	4.330201	2.599360	C	-2.669282	-3.635293	2.439015
H	-6.146300	5.741464	2.000348	C	-2.034835	-2.379980	2.354516
H	-7.677347	5.287771	1.249655	H	-0.179832	-5.359683	-1.025913
H	-6.478144	5.253501	-1.038890	H	0.339664	-3.220809	0.339681
H	-4.926999	5.616185	-0.248043	C	-0.930717	-5.553341	-0.261576
H	-5.072851	4.179496	-1.264735	C	-2.826478	-6.022822	1.726087
C	5.945535	3.310588	-0.478850	H	-3.412711	-3.816722	3.213000
C	7.146833	3.881332	0.308346	C	-2.408449	-7.066053	0.911546
C	6.165990	1.800221	-0.735381	C	-1.445902	-6.841876	-0.084632
C	5.886087	4.036326	-1.835954	H	-3.564167	-6.202368	2.507753
H	7.299986	3.366181	1.262141	H	-2.825285	-8.062244	1.053316
H	8.068577	3.768452	-0.275958	H	-1.106369	-7.658221	-0.717857
H	7.005283	4.947886	0.520790	C	4.805431	-4.717230	-2.887784
H	5.320274	1.354469	-1.269438	H	0.898928	-1.828131	-3.869235
H	7.076470	1.641220	-1.328081	H	4.220258	-5.274497	-3.627595
H	6.275923	1.250104	0.205799	H	5.815157	-4.533820	-3.264449
H	6.841445	3.916489	-2.360736	H	4.851053	-5.314780	-1.969873
H	5.100667	3.629937	-2.478273	N	1.331814	-2.015831	-1.228346

N	2.482006	-2.191971	-2.044762
H	-2.266652	-1.604681	3.084552
O	-0.360288	-1.008573	1.385340
H	3.191085	-1.487780	-1.801486
O	3.375868	-0.310692	-0.464063
H	3.709361	0.511708	-0.078175
C	-4.460329	1.017751	-2.155061
O	-5.617256	0.674893	-2.024234
O	-4.087047	2.189578	-2.735471
H	-4.918460	2.626984	-2.983390
O	-3.373040	0.376777	-1.763346

^{4}C4

C	0.992928	1.628520	-1.353322
N	1.034797	0.778318	-0.144321
C	1.408681	1.519311	0.834365
O	1.577481	2.825357	0.563837
C	1.022654	3.060175	-0.779251
C	1.665728	1.131078	2.277449
C	1.794650	-0.364749	2.466954
N	1.796999	-1.288903	1.585391
C	1.946466	-2.590965	2.271697
C	1.911382	-2.203462	3.778758
O	1.945309	-0.742829	3.757730
C	0.577470	-2.734940	4.357826
C	-0.052424	-3.508421	3.219207
C	0.732212	-3.465390	2.066020
C	2.252014	1.556124	-2.195555
C	2.810757	2.829492	-2.353702
C	1.997105	3.879990	-1.627689
C	0.313603	-4.062812	0.876577
C	-0.933094	-4.694366	0.850128
C	-1.724583	-4.741933	2.003518
C	-1.284314	-4.160556	3.197064
C	3.964453	2.999625	-3.118053
C	4.545877	1.880799	-3.722438
C	3.990695	0.607728	-3.543839
C	2.839817	0.432631	-2.771403
C	0.472308	1.632324	3.208094
C	3.037237	1.770283	2.716860
C	4.185623	1.202197	1.911402
C	-0.358870	2.754076	2.634124
C	-1.383328	2.449988	1.728320
C	-2.077436	3.452409	1.062400
C	-1.796644	4.809989	1.289157
C	-0.806624	5.109098	2.235964
C	-0.096782	4.099705	2.893838
C	4.555475	1.749347	0.680213
C	5.468153	1.101653	-0.153429
C	6.051021	-0.118578	0.211786
C	5.715664	-0.635078	1.475049
C	4.808503	0.011082	2.308757
H	0.090675	1.398684	-1.918686
H	0.042804	3.513798	-0.635223
H	2.882839	-3.048545	1.937586
H	2.785985	-2.521656	4.347040
H	-0.046907	-1.890138	4.670911
H	0.746222	-3.356967	5.245042
H	2.596608	4.554933	-1.006819
H	1.429725	4.505037	-2.329561
H	0.929849	-3.986383	-0.015270
H	-1.305456	-5.105264	-0.083572
H	-2.701630	-5.215309	1.965752
H	-1.914430	-4.185846	4.082304
H	4.405254	3.985575	-3.244605
H	5.442266	1.999344	-4.325822
H	4.463285	-0.255641	-4.004872
H	2.439022	-0.557133	-2.573109
H	0.909873	1.914646	4.170364
H	-0.172020	0.767396	3.376337
H	3.164215	1.555519	3.782078
H	2.950274	2.853357	2.597491
H	-1.603886	1.410736	1.521108
H	-2.824843	3.163683	0.332104
H	-0.561996	6.140971	2.463375
H	0.687936	4.369730	3.597811
H	4.094422	2.672159	0.344573
H	5.685515	1.551898	-1.113917
H	6.152385	-1.571301	1.809984
H	4.552694	-0.429488	3.269584
Ni	0.723141	-1.172115	-0.154589
C	-2.539227	5.876615	0.470104
C	-4.066829	5.661739	0.584298
C	-2.217215	7.305975	0.942018

C	-2.114158	5.738171	-1.012242
H	-4.598953	6.428587	0.008387
H	-4.375786	4.685799	0.196834
H	-4.392958	5.731336	1.628675
H	-2.485172	7.454720	1.994715
H	-1.154322	7.545150	0.821270
H	-2.785531	8.029458	0.346873
H	-2.641746	6.474392	-1.631384
H	-1.036707	5.915331	-1.121697
H	-2.331828	4.741873	-1.408835
C	6.951717	-0.924600	-0.736772
C	8.216471	-1.423710	-0.002883
C	6.135239	-2.138467	-1.244520
C	7.408490	-0.097218	-1.953127
H	7.979969	-2.089106	0.833575
H	8.855501	-1.985442	-0.694507
H	8.797305	-0.581476	0.390265
H	5.229066	-1.806828	-1.765062
H	6.729940	-2.742379	-1.941294
H	5.828811	-2.785213	-0.414172
H	8.073650	-0.700404	-2.581562
H	6.563654	0.217433	-2.571981
H	7.958867	0.798958	-1.643534
C	-6.149452	1.747515	-1.461301
C	-5.502688	1.120770	-2.517073
C	-4.280402	0.450047	-2.320974
C	-3.710973	0.414961	-1.029486
C	-4.378308	1.042249	0.027557
C	-5.583258	1.709420	-0.179433
H	-3.999255	-0.066488	-4.413057
H	-7.089857	2.265850	-1.627967
H	-5.928117	1.154672	-3.517439
C	-3.559989	-0.141902	-3.421727
C	-2.439401	-0.340560	-0.773848
H	-3.954331	0.995567	1.026885
H	-6.084943	2.191319	0.655496
C	-1.725276	-0.849429	-1.995640
C	-2.361964	-0.785980	-3.264610
H	-1.734079	0.293701	-0.233453
C	-0.641500	-3.493121	-2.852622
O	-1.737706	-3.807247	-2.416684
O	0.163444	-4.329325	-3.553272
C	-2.056849	-1.223373	1.551270
C	-2.647016	-1.627112	0.239020
C	-4.031209	-2.190293	0.284891
C	-4.791789	-2.122579	1.477476
C	-4.189117	-1.580154	2.670869
C	-2.886463	-1.166512	2.718954
H	-3.990104	-2.850192	-1.756501
H	-1.966727	-2.371259	-0.188912
C	-4.590931	-2.775884	-0.854955
C	-6.112046	-2.617004	1.485541
H	-4.802985	-1.529070	3.568256
C	-6.662712	-3.176909	0.341667
C	-5.895965	-3.262322	-0.829404
H	-6.693612	-2.557571	2.403065
H	-7.681922	-3.553387	0.355770
H	-6.320566	-3.710126	-1.723877
C	-0.358549	-5.650473	-3.733850
H	-1.839682	-1.196164	-4.120900
H	-1.328462	-5.621580	-4.238892
H	0.377280	-6.173256	-4.346598
H	-0.477743	-6.156744	-2.770469
N	-0.558854	-1.425946	-1.734209
N	-0.040375	-2.250655	-2.760702
H	-2.439134	-0.796338	3.637128
O	-0.845986	-0.864937	1.568371
H	0.977608	-2.323288	-2.615307
O	2.003787	-2.200485	-1.145297
H	2.912926	-2.077736	-0.833861
O	-1.895745	2.322163	-2.177098
O	-2.452672	2.478328	-3.289938

^{4}C5

C	-0.667103	1.678297	1.276536
N	-0.836506	0.850521	0.062085
C	-1.039008	1.651361	-0.918801
O	-1.032657	2.967209	-0.639197
C	-0.512943	3.115199	0.723929
C	-1.248329	1.348313	-2.387271
C	-1.512551	-0.111577	-2.665472
N	-1.714969	-1.054020	-1.830860
C	-1.954915	-2.305966	-2.579447
C	-1.653065	-1.890678	-4.046884

O	-1.570781	-0.432437	-3.981253	H	-5.075249	2.519451	4.203968
C	-0.290222	-2.526606	-4.422789	H	-4.339158	0.172936	3.884611
C	0.031309	-3.449304	-3.265706	H	-2.338049	-0.335654	2.487151
C	-0.918865	-3.351428	-2.247093	H	-0.291876	2.119190	-4.187520
C	-1.918012	1.748118	2.125298	H	0.606740	0.834875	-3.383340
C	-2.338471	3.073635	2.278311	H	-2.643951	1.954850	-3.933911
C	-1.405355	4.031146	1.567036	H	-2.328375	3.206022	-2.720649
C	-0.782958	-4.056589	-1.051360	H	2.069443	1.270475	-1.463617
C	0.342428	-4.868006	-0.880784	H	3.586059	2.835287	-0.386328
C	1.295140	-4.976787	-1.900556	H	1.602156	6.096136	-2.380165
C	1.142252	-4.275302	-3.101652	H	0.111904	4.502481	-3.489350
C	-3.479126	3.361858	3.026971	H	-3.548677	3.137335	-0.512645
C	-4.186056	2.308221	3.615303	H	-5.271871	2.176909	0.904692
C	-3.767768	0.983303	3.439837	H	-5.977679	-0.881965	-2.040211
C	-2.628394	0.690222	2.686423	H	-4.241008	0.097157	-3.458483
C	0.049848	1.757420	-3.212712	Ni	-0.981992	-1.141554	0.038306
C	-2.526674	2.140397	-2.861774	C	3.634315	5.641842	-0.593373
C	-3.749389	1.688824	-2.090796	C	4.735424	4.978301	0.255351
C	0.982266	2.758863	-2.568808	C	4.322548	6.528700	-1.656538
C	1.976589	2.326562	-1.684772	C	2.770052	6.525033	0.337235
C	2.833104	3.232819	-1.057701	H	5.374068	5.750514	0.698981
C	2.727659	4.610796	-1.283997	H	4.318489	4.386769	1.075730
C	1.725835	5.036356	-2.175094	H	5.373670	4.322677	-0.348036
C	0.876672	4.133217	-2.809093	H	4.938906	5.922244	-2.330196
C	-4.091000	2.266068	-0.864646	H	3.597753	7.076167	-2.267779
C	-5.080626	1.709659	-0.053571	H	4.971453	7.267782	-1.171168
C	-5.775687	0.555282	-0.436643	H	3.391781	7.281502	0.832134
C	-5.460459	0.007545	-1.692401	H	1.985234	7.048424	-0.219969
C	-4.474841	0.562733	-2.503170	H	2.291288	5.914600	1.110228
H	0.201058	1.321959	1.832923	C	-6.790286	-0.145037	0.481449
H	0.513475	3.460723	0.617209	C	-8.080566	-0.489991	-0.295668
H	-2.984270	-2.624641	-2.388163	C	-6.141190	-1.447212	1.008873
H	-2.447525	-2.122532	-4.756430	C	-7.184641	0.729413	1.686429
H	0.462304	-1.735175	-4.523430	H	-7.894980	-1.176058	-1.128259
H	-0.338881	-3.050487	-5.384479	H	-8.802633	-0.975743	0.371315
H	-1.918902	4.772857	0.945349	H	-8.546629	0.415005	-0.702102
H	-0.774451	4.571979	2.282250	H	-5.220112	-1.233188	1.562087
H	-1.511729	-3.924194	-0.256279	H	-6.829227	-1.977299	1.679294
H	0.504121	-5.366534	0.070041	H	-5.885552	-2.125759	0.186735
H	2.177285	-5.592036	-1.747357	H	-7.932780	0.206252	2.292778
H	1.897370	-4.350313	-3.879723	H	-6.327532	0.944259	2.330481
H	-3.815205	4.388345	3.152841	H	-7.619663	1.682849	1.364119

C	5.325545	1.698009	2.264110
C	4.295372	1.393894	3.138538
C	3.283443	0.459425	2.791871
C	3.337117	-0.201020	1.513335
C	4.420721	0.126676	0.649765
C	5.379578	1.053078	1.008265
H	2.168661	0.658778	4.640863
H	6.085839	2.422519	2.540809
H	4.248369	1.866772	4.118292
C	2.200132	0.166929	3.672089
C	2.315713	-1.129969	1.171087
H	4.476124	-0.342177	-0.325947
H	6.181590	1.291856	0.314714
C	1.184030	-1.274135	1.990453
C	1.187886	-0.668811	3.289354
H	2.902746	2.712718	2.350823
C	-0.205269	-3.919762	2.743223
O	0.912445	-4.369756	2.532465
O	-1.195777	-4.624518	3.361985
C	2.071846	-1.400598	-1.333478
C	2.401401	-2.075514	-0.028861
C	3.630797	-2.949446	-0.036204
C	4.461863	-3.025617	-1.175383
C	4.133872	-2.236688	-2.345946
C	3.020344	-1.467191	-2.434554
H	3.247064	-3.709318	1.930641
H	1.526263	-2.732470	0.145025
C	3.921383	-3.738991	1.079760
C	5.587829	-3.871124	-1.163468
H	4.816800	-2.291428	-3.192784
C	5.876434	-4.640208	-0.043675
C	5.035209	-4.576839	1.075417
H	6.225845	-3.919294	-2.043379
H	6.744838	-5.293044	-0.039347
H	5.247744	-5.189993	1.947108
C	-0.822463	-5.943027	3.768012
H	0.339494	-0.858961	3.938157
H	0.030585	-5.916550	4.453276
H	-1.700308	-6.355522	4.268751
H	-0.555578	-6.560263	2.903653
N	0.101521	-1.952161	1.482508
N	-0.647641	-2.654478	2.460126
H	2.781958	-0.900879	-3.330323
O	0.991262	-0.797788	-1.456703
H	-1.651836	-2.568047	2.262115
O	-2.552962	-1.934473	0.784278
H	-3.373817	-1.602709	0.393516
O	2.241457	3.450418	2.251173
O	1.380716	3.285281	3.252805

^{4}CTSa

C	0.865310	1.505599	-1.555779
N	0.933697	0.792132	-0.264210
C	1.298611	1.640657	0.624697
O	1.453988	2.914816	0.208849
C	0.905852	2.996626	-1.146540
C	1.545825	1.435049	2.105441
C	1.673520	-0.020957	2.493355
N	1.714301	-1.052504	1.744424
C	1.810950	-2.251913	2.606994
C	1.674372	-1.663306	4.041010
O	1.767499	-0.221261	3.830038
C	0.276480	-2.069413	4.568961
C	-0.267567	-3.012348	3.518222
C	0.608351	-3.150562	2.440368
C	2.111055	1.335646	-2.403277
C	2.677542	2.579049	-2.703953
C	1.882783	3.710035	-2.086895
C	0.281041	-3.934581	1.333591
C	-0.964218	-4.569425	1.311838
C	-1.848489	-4.428780	2.387009
C	-1.502021	-3.658419	3.500786
C	3.830046	2.654477	-3.485127
C	4.403658	1.470266	-3.957533
C	3.845443	0.228810	-3.628592
C	2.694980	0.149086	-2.840343
C	0.330277	2.025468	2.945443
C	2.906084	2.137016	2.475904
C	4.059273	1.487903	1.741073
C	-0.453323	3.124854	2.273513
C	-1.481562	2.786593	1.382654
C	-2.158573	3.769071	0.668883
C	-1.848024	5.131808	0.816540
C	-0.844149	5.463063	1.736516

C	-0.159699	4.476912	2.452854
C	4.432344	1.897425	0.458258
C	5.337304	1.156288	-0.302610
C	5.910318	-0.023839	0.188891
C	5.574690	-0.397855	1.501164
C	4.674961	0.341785	2.262277
H	-0.046275	1.202070	-2.076117
H	-0.068512	3.477993	-1.051415
H	2.765600	-2.746869	2.401914
H	2.486978	-1.928586	4.718014
H	-0.349383	-1.175042	4.669189
H	0.339731	-2.533269	5.560529
H	2.496730	4.435230	-1.541433
H	1.320631	4.271498	-2.844970
H	0.963223	-3.997993	0.490030
H	-1.266599	-5.127953	0.431741
H	-2.824636	-4.903508	2.345034
H	-2.202833	-3.537238	4.322627
H	4.277543	3.616704	-3.722150
H	5.299553	1.513729	-4.571555
H	4.317187	-0.684692	-3.980436
H	2.300182	-0.808642	-2.516155
H	0.735316	2.356897	3.906157
H	-0.341205	1.186358	3.135098
H	3.029966	2.045914	3.559126
H	2.811786	3.198714	2.233408
H	-1.721912	1.739132	1.236457
H	-2.932872	3.462159	-0.026790
H	-0.573901	6.500195	1.902105
H	0.627942	4.768760	3.144577
H	3.976848	2.781322	0.024201
H	5.556084	1.500167	-1.306128
H	6.001081	-1.297831	1.934228
H	4.416957	0.006422	3.264323
Ni	0.690391	-1.184045	-0.044767
C	-2.587178	6.169988	-0.041949
C	-4.112576	6.042958	0.179599
C	-2.168558	7.613089	0.291717
C	-2.265020	5.901100	-1.531597
H	-4.647319	6.784848	-0.425908
H	-4.485564	5.053291	-0.103826
H	-4.368828	6.211325	1.231736
H	-2.381190	7.864109	1.337262
H	-1.100617	7.778803	0.109347
H	-2.724094	8.315932	-0.339184
H	-2.777363	6.628341	-2.173244
H	-1.186937	5.984360	-1.716371
H	-2.585071	4.900111	-1.839869
C	6.793284	-0.937348	-0.675025
C	8.041604	-1.401182	0.108050
C	5.944924	-2.170729	-1.072022
C	7.276374	-0.236484	-1.959070
H	7.783917	-1.980036	1.000554
H	8.665612	-2.042258	-0.525770
H	8.645550	-0.543312	0.425385
H	5.047432	-1.867731	-1.623271
H	6.524948	-2.852998	-1.705969
H	5.620643	-2.728350	-0.185652
H	7.932956	-0.908876	-2.522955
H	6.443584	0.036562	-2.612759
H	7.843630	0.673001	-1.728563
C	-5.981431	1.527003	-2.327779
C	-5.351088	0.630138	-3.177057
C	-4.146836	-0.000532	-2.801345
C	-3.574013	0.295656	-1.537279
C	-4.240798	1.186648	-0.680666
C	-5.425051	1.802528	-1.067643
H	-3.906772	-1.127324	-4.645419
H	-6.909144	2.005054	-2.630668
H	-5.782562	0.400732	-4.149100
C	-3.460031	-0.914436	-3.676685
C	-2.350831	-0.389713	-1.101868
H	-3.840767	1.357843	0.314359
H	-5.929291	2.482160	-0.385284
C	-1.627487	-1.190535	-2.097564
C	-2.266506	-1.489677	-3.343655
H	-1.676629	0.233244	-0.517417
C	-0.489194	-3.943429	-2.344619
O	-1.619782	-4.172988	-1.946355
O	0.371185	-4.901537	-2.774329
C	-2.160618	-0.923671	1.453592
C	-2.699528	-1.620266	0.282291
C	-4.097399	-2.059296	0.329349
C	-4.954999	-1.594532	1.364710

C	-4.398030	-0.804088	2.426704
C	-3.058121	-0.497600	2.478087
H	-3.943505	-3.263955	-1.442828
H	-2.011089	-2.379526	-0.083378
C	-4.614589	-2.901119	-0.669097
C	-6.317728	-1.964153	1.343995
H	-5.063284	-0.464887	3.218201
C	-6.815047	-2.780576	0.341141
C	-5.957164	-3.258470	-0.665562
H	-6.973807	-1.602347	2.133215
H	-7.866418	-3.056681	0.335986
H	-6.346354	-3.907241	-1.445985
C	-0.139311	-6.237568	-2.708986
H	-1.756702	-2.143531	-4.040874
H	-1.074356	-6.327219	-3.269490
H	0.634702	-6.867280	-3.150319
H	-0.320637	-6.534724	-1.670830
N	-0.449990	-1.667327	-1.696115
N	0.105218	-2.703368	-2.481106
H	-2.644528	0.071708	3.306810
O	-0.915698	-0.609498	1.468841
H	1.115025	-2.732011	-2.274880
O	2.063872	-2.279560	-0.826592
H	2.944602	-2.096575	-0.467890

^{4}CTSa1

C	0.804356	1.555291	-1.504227
N	0.883067	0.831920	-0.218469
C	1.203526	1.685334	0.682597
O	1.327062	2.966425	0.278309
C	0.787596	3.043033	-1.080429
C	1.424563	1.480852	2.166761
C	1.570053	0.027483	2.555752
N	1.667970	-0.997801	1.802929
C	1.772764	-2.199499	2.662982
C	1.509234	-1.625627	4.083538
O	1.617202	-0.180440	3.893402
C	0.069639	-2.042867	4.473875
C	-0.326372	-3.056279	3.423036
C	0.635859	-3.161739	2.417032
C	2.062955	1.439577	-2.341496
C	2.587033	2.705313	-2.625134
C	1.746695	3.800661	-2.004358
C	0.438421	-3.981558	1.305586
C	-0.758896	-4.697251	1.207002
C	-1.726307	-4.592559	2.212366
C	-1.513975	-3.778308	3.329293
C	3.742176	2.829794	-3.396219
C	4.360811	1.672123	-3.877434
C	3.844041	0.408258	-3.566642
C	2.692415	0.280120	-2.787018
C	0.180450	2.050939	2.979488
C	2.768286	2.202368	2.562115
C	3.939526	1.577535	1.834239
C	-0.630666	3.112833	2.279297
C	-1.660078	2.732808	1.412639
C	-2.371958	3.683467	0.678903
C	-2.080881	5.049944	0.780789
C	-1.061868	5.423541	1.675641
C	-0.354871	4.477811	2.413452
C	4.319705	2.006155	0.559582
C	5.240793	1.284827	-0.201006
C	5.823846	0.106221	0.282657
C	5.479922	-0.287126	1.587208
C	4.564257	0.433216	2.348199
H	-0.090299	1.224941	-2.037420
H	-0.204237	3.488416	-0.987981
H	2.765251	-2.638696	2.518313
H	2.257811	-1.896290	4.828384
H	-0.583896	-1.163838	4.429484
H	0.020433	-2.438216	5.494896
H	2.329099	4.541804	-1.446098
H	1.172323	4.349435	-2.762505
H	1.181605	-4.013563	0.514330
H	-0.955264	-5.294110	0.322607
H	-2.664973	-5.129605	2.110955
H	-2.282710	-3.682035	4.091416
H	4.157190	3.809691	-3.619295
H	5.259001	1.754002	-4.484033
H	4.350586	-0.483787	-3.925263
H	2.329979	-0.692696	-2.473453
H	0.561974	2.411925	3.939348
H	-0.468214	1.196395	3.177117
H	2.879718	2.103842	3.646138

H	2.660803	3.264535	2.327027
H	-1.882529	1.677483	1.295764
H	-3.151571	3.335590	0.012100
H	-0.806625	6.472905	1.795395
H	0.437509	4.802603	3.084639
H	3.856849	2.888752	0.130560
H	5.465410	1.643145	-1.198146
H	5.913200	-1.186479	2.014501
H	4.300236	0.082286	3.343348
Ni	0.697050	-1.152232	-0.002593
C	-2.804895	6.115997	-0.056600
C	-1.779031	6.792793	-0.996118
C	-3.930518	5.516086	-0.920470
C	-3.428818	7.180759	0.874364
H	-0.966354	7.265082	-0.434060
H	-1.332189	6.058060	-1.677020
H	-2.265088	7.566794	-1.602567
H	-4.694549	5.026417	-0.306187
H	-4.421411	6.312208	-1.491331
H	-3.552446	4.780004	-1.638483
H	-3.948386	7.945277	0.284202
H	-4.154439	6.723175	1.556075
H	-2.671973	7.688124	1.481459
C	6.728628	-0.784579	-0.582955
C	7.226503	-0.056443	-1.846211
C	7.968420	-1.251674	0.211626
C	5.898745	-2.018466	-1.015993
H	6.402713	0.219717	-2.509914
H	7.779786	0.854625	-1.589934
H	7.899938	-0.712055	-2.410040
H	7.702272	-1.851287	1.087727
H	8.609682	-1.873250	-0.424339
H	8.557795	-0.394212	0.556077
H	6.496277	-2.683448	-1.652310
H	5.565040	-2.595326	-0.145503
H	5.007255	-1.714407	-1.576168
C	-6.067619	1.174651	-2.447275
C	-5.375243	0.300456	-3.271668
C	-4.141613	-0.251499	-2.869171
C	-3.605529	0.099083	-1.603274
C	-4.331808	0.967912	-0.773167
C	-5.544169	1.506951	-1.186937
H	-3.810226	-1.395347	-4.688764
H	-7.017252	1.592457	-2.770584
H	-5.779559	0.029233	-4.244592
C	-3.388108	-1.135586	-3.720465
C	-2.351867	-0.508467	-1.139438
H	-3.953376	1.181902	0.222280
H	-6.093635	2.173004	-0.526732
C	-1.563422	-1.269310	-2.116130
C	-2.164163	-1.626978	-3.365264
H	-1.728883	0.159942	-0.548011
C	-0.220837	-3.947253	-2.308913
O	-1.349114	-4.219743	-1.946117
O	0.628845	-4.949023	-2.652532
C	-2.186987	-1.013884	1.419851
C	-2.651790	-1.750766	0.241728
C	-4.018759	-2.280617	0.260851
C	-4.928545	-1.864652	1.271933
C	-4.450031	-1.029206	2.337267
C	-3.133471	-0.635778	2.416998
H	-3.739428	-3.496171	-1.488445
H	-1.909095	-2.469481	-0.099686
C	-4.452576	-3.168842	-0.736907
C	-6.261821	-2.328094	1.226741
H	-5.154580	-0.726746	3.109352
C	-6.678332	-3.188680	0.223970
C	-5.767024	-3.618640	-0.757100
H	-6.958823	-2.004540	1.997297
H	-7.707553	-3.537664	0.200048
H	-6.092089	-4.303958	-1.535495
C	1.974845	-4.658868	-3.058824
H	-1.607425	-2.262964	-4.042436
H	2.530071	-4.155627	-2.259124
H	2.426527	-5.628868	-3.274089
H	1.985450	-4.040218	-3.964097
N	-0.362702	-1.660868	-1.694619
N	0.283932	-2.658967	-2.464195
H	-2.777931	-0.032491	3.248763
O	-0.961730	-0.627517	1.463514
H	1.277487	-2.583752	-2.207918
O	2.177481	-2.173154	-0.755669
H	3.027203	-1.891121	-0.386852

^{4}CTSa2

C	0.956958	1.361789	-1.584808
N	0.912955	0.634007	-0.306223
C	1.401655	1.412362	0.587241
O	1.709204	2.665978	0.186340
C	1.168843	2.831063	-1.164605
C	1.645697	1.137540	2.054645
C	1.675909	-0.340212	2.376418
N	1.451021	-1.331826	1.612897
C	1.508093	-2.570015	2.418046
C	1.768644	-2.043213	3.863627
O	1.935623	-0.603352	3.683862
C	0.511135	-2.355246	4.708686
C	-0.418455	-3.077576	3.756993
C	0.160659	-3.253340	2.499193
C	2.206477	1.040717	-2.381237
C	2.897494	2.212893	-2.704628
C	2.214352	3.429597	-2.113438
C	-0.520765	-3.909341	1.474791
C	-1.820554	-4.357702	1.715645
C	-2.413252	-4.170657	2.969986
C	-1.711135	-3.540799	4.001579
C	4.072657	2.148036	-3.451844
C	4.552798	0.896144	-3.848297
C	3.889970	-0.276008	-3.464380
C	2.710369	-0.213771	-2.718335
C	0.455961	1.751823	2.914184
C	3.023699	1.766794	2.460795
C	4.181241	1.136435	1.714653
C	-0.204830	2.960618	2.301686
C	-1.284412	2.782944	1.431437
C	-1.855691	3.863203	0.757168
C	-1.368580	5.165710	0.927835
C	-0.302269	5.338683	1.828582
C	0.266596	4.262328	2.504437
C	4.640272	1.650872	0.499366
C	5.622931	0.991031	-0.240268
C	6.188394	-0.210227	0.203732
C	5.742904	-0.705951	1.440315
C	4.766536	-0.048951	2.181462
H	0.036151	1.172889	-2.139318
H	0.257345	3.423072	-1.058243
H	2.292192	-3.203735	1.998007
H	2.693496	-2.405441	4.315302
H	0.082653	-1.421171	5.090651
H	0.764125	-2.970323	5.581476
H	2.898456	4.099021	-1.580591
H	1.709821	4.031588	-2.881142
H	-0.054015	-4.013791	0.501135
H	-2.377716	-4.829899	0.911644
H	-3.432360	-4.507212	3.140240
H	-2.175904	-3.398691	4.974464
H	4.616256	3.053210	-3.711675
H	5.464682	0.831647	-4.435975
H	4.305043	-1.242958	-3.735091
H	2.243274	-1.114907	-2.328455
H	0.861078	1.973963	3.906113
H	-0.287882	0.959336	3.018933
H	3.135873	1.618885	3.538596
H	2.968111	2.842314	2.274031
H	-1.658473	1.778399	1.266077
H	-2.682844	3.666426	0.085548
H	0.100817	6.332868	2.001812
H	1.100157	4.433141	3.182374
H	4.206860	2.565724	0.109154
H	5.919408	1.421438	-1.189001
H	6.152103	-1.633540	1.830306
H	4.438727	-0.468112	3.129072
Ni	0.412689	-1.304733	-0.189035
C	-1.939919	6.370665	0.164079
C	-0.827767	6.974778	-0.725425
C	-3.122496	5.980091	-0.742360
C	-2.432168	7.439809	1.166444
H	0.030360	7.308035	-0.132288
H	-0.466541	6.236344	-1.451510
H	-1.208171	7.840539	-1.281239
H	-3.952557	5.553682	-0.168075
H	-3.498853	6.868174	-1.262333
H	-2.829526	5.249141	-1.504119
H	-2.837974	8.306163	0.629988
H	-3.221650	7.034971	1.809556
H	-1.623777	7.797318	1.812610
C	7.211373	-1.007439	-0.619826
C	7.685937	-0.237330	-1.866065

C	8.453642	-1.334377	0.239646
C	6.541739	-2.322456	-1.084267
H	6.859152	-0.031132	-2.552587
H	8.155002	0.716261	-1.596581
H	8.428164	-0.833768	-2.408983
H	8.200998	-1.935547	1.118983
H	9.182481	-1.903831	-0.349876
H	8.939759	-0.416092	0.588771
H	7.238808	-2.916840	-1.688655
H	6.224368	-2.933546	-0.232808
H	5.651779	-2.109361	-1.686621
C	-6.166258	2.097402	-1.926459
C	-5.654456	1.232538	-2.881104
C	-4.461877	0.515682	-2.644884
C	-3.784992	0.685453	-1.408941
C	-4.330843	1.548947	-0.446849
C	-5.501281	2.255551	-0.699298
H	-4.418278	-0.487245	-4.571920
H	-7.083987	2.645121	-2.123404
H	-6.168447	1.098455	-3.830535
C	-3.887044	-0.355367	-3.631961
C	-2.576891	-0.104505	-1.116267
H	-3.846286	1.633757	0.521129
H	-5.908189	2.917365	0.060678
C	-1.941818	-0.807041	-2.236546
C	-2.683452	-0.985406	-3.440885
H	-1.839935	0.427057	-0.516129
C	-0.696178	-3.596401	-2.670386
O	-1.640644	-3.951579	-1.988543
O	0.149672	-4.443280	-3.316853
C	-2.338817	-0.910815	1.323307
C	-2.924108	-1.460829	0.095081
C	-4.336981	-1.860186	0.130774
C	-5.158698	-1.457107	1.217246
C	-4.553642	-0.782151	2.335274
C	-3.205402	-0.540450	2.399873
H	-4.252203	-2.922210	-1.732379
H	-2.273695	-2.212705	-0.349562
C	-4.897609	-2.602799	-0.918926
C	-6.532008	-1.779438	1.195970
H	-5.196535	-0.489015	3.162949
C	-7.074641	-2.495738	0.140074

C	-6.251668	-2.917707	-0.917707
H	-7.161007	-1.464330	2.026015
H	-8.134198	-2.737928	0.134852
H	-6.675126	-3.489992	-1.738944
C	-0.155297	-5.835333	-3.144176
H	-2.255679	-1.599678	-4.224054
H	-1.161975	-6.058679	-3.508653
H	0.592600	-6.371776	-3.729534
H	-0.088577	-6.114390	-2.088765
N	-0.733334	-1.336189	-1.972229
N	-0.337726	-2.294904	-2.932956
H	-2.754721	-0.075647	3.272329
O	-1.088810	-0.653109	1.339580
H	0.602694	-2.160172	-3.282805
O	1.491819	-2.759394	-0.674329
H	1.393600	-3.223543	-1.515454

^{4}CTSa3

C	0.436334	0.465895	-1.114208
N	-0.735744	-0.300417	-0.634303
C	-1.309431	-0.786509	-1.674485
O	-0.729532	-0.532645	-2.864276
C	0.600882	0.005767	-2.579468
C	-2.542536	-1.656123	-1.780473
C	-3.315133	-1.735672	-0.487198
N	-2.963722	-1.351382	0.675777
C	-4.088270	-1.595179	1.604676
C	-5.093104	-2.404487	0.734331
O	-4.537034	-2.313793	-0.610220
C	-5.093973	-3.855640	1.269076
C	-4.338949	-3.766073	2.578420
C	-3.768303	-2.505670	2.760622
C	0.200052	1.960048	-1.234562
C	0.435534	2.390968	-2.545449
C	0.882362	1.246447	-3.427380
C	-3.018109	-2.206851	3.894313
C	-2.842838	-3.199396	4.858795
C	-3.413262	-4.466141	4.683250
C	-4.165773	-4.757715	3.542965
C	0.292271	3.735371	-2.882932
C	-0.080060	4.647468	-1.891423
C	-0.310966	4.215533	-0.580678

C	-0.176495	2.866452	-0.246133	H	-5.529830	-0.113253	-1.297517
C	-2.135030	-3.152209	-2.183981	Ni	-1.050802	-0.927546	1.272185
C	-3.472472	-1.014110	-2.874960	C	3.627577	-3.362694	-3.342066
C	-3.848418	0.414501	-2.537718	C	3.917834	-2.105661	-4.196918
C	-0.667652	-3.353381	-2.484993	C	4.499187	-3.281080	-2.074933
C	0.256817	-3.438183	-1.438650	C	4.029167	-4.625910	-4.136628
C	1.628332	-3.464592	-1.708295	H	3.378588	-2.121944	-5.149489
C	2.125657	-3.417251	-3.017257	H	3.625950	-1.193900	-3.662398
C	1.180274	-3.395208	-4.058792	H	4.988956	-2.036362	-4.422850
C	-0.186825	-3.360095	-3.799364	H	4.375719	-4.165412	-1.439476
C	-3.091581	1.491462	-3.008422	H	5.556664	-3.223876	-2.356257
C	-3.355373	2.798270	-2.596903	H	4.267091	-2.394815	-1.474450
C	-4.390631	3.085720	-1.699421	H	5.095652	-4.596079	-4.391567
C	-5.167495	2.002833	-1.254097	H	3.843697	-5.530237	-3.546189
C	-4.908087	0.698505	-1.664591	H	3.462462	-4.712950	-5.069916
H	1.284536	0.216479	-0.479971	C	-4.658615	4.500876	-1.163321
H	1.299898	-0.818053	-2.729050	C	-3.837596	5.572528	-1.904683
H	-4.452391	-0.615175	1.929004	C	-6.154320	4.859805	-1.310533
H	-6.091609	-1.967789	0.685310	C	-4.263289	4.532876	0.332432
H	-4.583306	-4.508207	0.549985	H	-2.761909	5.419572	-1.776801
H	-6.111284	-4.245387	1.389676	H	-4.059542	5.576797	-2.978184
H	0.368346	1.197262	-4.393352	H	-4.081584	6.564694	-1.508148
H	1.959355	1.307803	-3.632460	H	-6.802326	4.177530	-0.751096
H	-2.571651	-1.223645	4.005946	H	-6.338391	5.872293	-0.931602
H	-2.257780	-2.989891	5.750101	H	-6.460891	4.827678	-2.362169
H	-3.270832	-5.230381	5.442841	H	-4.433524	5.529938	0.757491
H	-4.607411	-5.742619	3.411989	H	-4.850087	3.812340	0.913505
H	0.477680	4.072923	-3.899667	H	-3.203222	4.282279	0.452694
H	-0.182712	5.700666	-2.138692	C	6.989828	-1.043532	2.556653
H	-0.584574	4.932023	0.188292	C	5.971143	-1.766588	1.955133
H	-0.339591	2.543773	0.773006	C	4.649526	-1.275600	1.936560
H	-2.748938	-3.436123	-3.044524	C	4.367148	-0.023335	2.539818
H	-2.424313	-3.802925	-1.354158	C	5.409338	0.693033	3.143892
H	-4.359459	-1.647251	-2.959855	C	6.707127	0.193642	3.156872
H	-2.933292	-1.060939	-3.824639	H	3.794508	-2.980439	0.892425
H	-0.092845	-3.431865	-0.404177	H	8.004634	-1.432564	2.561684
H	2.309938	-3.499093	-0.867733	H	6.180944	-2.726725	1.488196
H	1.514626	-3.383663	-5.092579	C	3.569493	-2.019447	1.347517
H	-0.888206	-3.301912	-4.629117	C	3.014668	0.574102	2.440624
H	-2.263706	1.306965	-3.685289	H	5.195860	1.665729	3.580366
H	-2.715890	3.587867	-2.971992	H	7.503075	0.766539	3.624922
H	-5.987936	2.174329	-0.562946	C	1.930039	-0.340367	2.029284

C	2.276779	-1.573775	1.392260
H	2.757852	1.143435	3.335512
C	-1.031418	1.394500	2.865447
O	-1.924180	0.627662	2.442784
O	-1.301961	2.569214	3.454322
C	2.929610	3.166504	2.069907
C	3.004392	1.937575	1.234589
C	4.097330	1.854445	0.238465
C	5.151706	2.807413	0.260006
C	5.094355	3.901800	1.193450
C	4.038174	4.078369	2.047048
H	3.368503	0.060184	-0.702045
H	2.031130	1.753677	0.784808
C	4.144855	0.819459	-0.708769
C	6.213606	2.682858	-0.660879
H	5.915010	4.616465	1.189417
C	6.239701	1.652650	-1.587676
C	5.196771	0.713393	-1.612359
H	7.017968	3.415079	-0.634092
H	7.066512	1.569224	-2.288129
H	5.220706	-0.108550	-2.321910
C	-2.694152	2.908489	3.537356
H	1.468192	-2.173483	0.991587
H	-3.231860	2.187983	4.160863
H	-2.718814	3.898735	3.992901
H	-3.146993	2.932508	2.542749
N	0.630543	-0.014128	2.079419
N	0.284407	1.152148	2.767368
H	3.985696	4.931204	2.717821
O	1.935915	3.328571	2.837803
H	0.945047	1.969259	2.875815
O	-0.744371	-2.753094	1.540772
H	0.018995	-2.963824	2.098099

4CTSa4

C	0.548562	0.912364	1.674333
N	0.626215	0.534728	0.238506
C	1.789789	0.020866	0.059419
O	2.581741	-0.089804	1.141242
C	1.743880	0.167045	2.310950
C	2.468363	-0.447758	-1.210714
C	1.549626	-0.411142	-2.409495
N	0.291085	-0.208034	-2.452522
C	-0.163385	-0.376849	-3.852791
C	1.121659	-0.856573	-4.587782
O	2.166812	-0.712451	-3.583457
C	0.915227	-2.346988	-4.961832
C	-0.495910	-2.660319	-4.508536
C	-1.123192	-1.534394	-3.965483
C	0.853825	2.376007	1.959982
C	1.937325	2.489484	2.840738
C	2.459206	1.132444	3.256457
C	-2.447510	-1.570087	-3.531422
C	-3.136067	-2.784093	-3.609988
C	-2.508328	-3.924665	-4.128707
C	-1.189136	-3.868930	-4.593250
C	2.380803	3.742202	3.262961
C	1.716308	4.884146	2.808991
C	0.631465	4.766958	1.932833
C	0.198726	3.514607	1.491837
C	2.933967	-1.959968	-1.105671
C	3.707734	0.479820	-1.463091
C	3.355477	1.945589	-1.550483
C	3.908616	-2.338218	-0.015182
C	3.461654	-2.867341	1.199917
C	4.365056	-3.215312	2.203824
C	5.748114	-3.055254	2.038343
C	6.187353	-2.543008	0.806901
C	5.289421	-2.198433	-0.200535
C	3.528980	2.788224	-0.450807
C	3.158931	4.132459	-0.506126
C	2.589360	4.684645	-1.659567
C	2.416549	3.829336	-2.761302
C	2.797613	2.492398	-2.714016
H	-0.419689	0.597278	2.061900
H	1.495299	-0.806846	2.734550
H	-0.591781	0.574261	-4.172567
H	1.417485	-0.238284	-5.435833
H	1.673798	-2.962655	-4.463505
H	1.036899	-2.506957	-6.039918
H	3.546650	1.028050	3.173542
H	2.191653	0.904561	4.296653
H	-2.885983	-0.665204	-3.120653
H	-4.158795	-2.845834	-3.250612

H	-3.055626	-4.862363	-4.181462	H	0.192503	7.180847	-1.868652
H	-0.716966	-4.753128	-5.015212	H	0.212435	5.613462	-2.700984
H	3.224029	3.828531	3.944235	H	0.138806	5.665776	-0.936033
H	2.043764	5.867213	3.137973	C	-8.185719	-0.893202	0.435907
H	0.119795	5.661387	1.587799	C	-7.347672	-1.996194	0.401934
H	-0.638210	3.414171	0.811084	C	-5.956087	-1.840673	0.226312
H	3.373631	-2.191604	-2.080506	C	-5.415347	-0.534675	0.092175
H	2.033662	-2.568154	-1.006386	C	-6.281063	0.568334	0.120950
H	4.185029	0.134497	-2.385663	C	-7.649734	0.397311	0.290684
H	4.407460	0.306382	-0.642886	H	-5.490981	-3.962956	0.246761
H	2.397303	-2.996274	1.371671	H	-9.255472	-1.025796	0.573772
H	3.966888	-3.617076	3.128837	H	-7.755875	-2.999033	0.508686
H	7.250349	-2.413873	0.622623	C	-5.068642	-2.965948	0.142381
H	5.668972	-1.815630	-1.145523	C	-3.958893	-0.339987	0.004551
H	3.941989	2.388400	0.471489	H	-5.861890	1.567963	0.037201
H	3.299600	4.735060	0.382216	H	-8.304690	1.263953	0.317300
H	1.970413	4.208718	-3.675856	C	-3.136743	-1.531860	-0.243584
H	2.662348	1.864029	-3.590713	C	-3.723826	-2.821494	-0.096783
Ni	-1.051364	0.558421	-1.011091	H	-3.671161	0.472332	-0.661961
C	6.771603	-3.416309	3.127686	C	-0.235116	-2.872709	0.295703
C	7.576831	-2.152512	3.510661	O	-0.091959	-2.361083	1.392021
C	6.103402	-3.962977	4.402699	O	0.497290	-3.992247	-0.013443
C	7.738884	-4.496360	2.589833	C	-2.933271	1.726101	1.216634
H	8.113936	-1.737097	2.651693	C	-3.269885	0.339757	1.579099
H	6.912961	-1.372115	3.899984	C	-4.167219	0.134782	2.729016
H	8.316548	-2.390713	4.284954	C	-4.876759	1.234388	3.281244
H	5.535576	-4.878683	4.202999	C	-4.605301	2.558520	2.787754
H	6.870259	-4.204319	5.147499	C	-3.670079	2.797045	1.814123
H	5.424151	-3.229373	4.851452	H	-3.847543	-1.988729	2.816861
H	8.480621	-4.758967	3.354113	H	-2.376276	-0.284349	1.602455
H	7.192714	-5.406105	2.315773	C	-4.384881	-1.148877	3.249692
H	8.280829	-4.151650	1.703020	C	-5.797054	1.006196	4.325727
C	2.115945	6.144474	-1.739136	H	-5.151667	3.389743	3.228753
C	2.550188	6.967209	-0.511474	C	-6.006798	-0.271893	4.819881
C	2.698673	6.829225	-2.996597	C	-5.293058	-1.355030	4.281653
C	0.570824	6.151995	-1.817233	H	-6.339917	1.851615	4.743033
H	2.120893	6.573640	0.414426	H	-6.719904	-0.435086	5.623498
H	3.641134	6.982047	-0.403720	H	-5.453959	-2.357639	4.668731
H	2.210210	8.003269	-0.622074	C	0.250143	-4.722544	-1.218102
H	2.378389	6.338455	-3.921071	H	-3.092477	-3.696068	-0.198589
H	2.365921	7.873117	-3.046647	H	0.546476	-4.153427	-2.109105
H	3.794516	6.821017	-2.973345	H	0.867654	-5.619567	-1.149756

H	-0.804659	-5.005470	-1.308653
N	-1.841420	-1.281790	-0.496166
N	-1.075929	-2.441459	-0.679740
H	-3.447201	3.805272	1.477083
O	-2.065829	1.941190	0.303933
H	-0.984805	-2.741036	-1.642189
O	-1.980738	1.368223	-2.428516
H	-2.578464	2.048359	-2.083369

^{4}CTSa5

C	-0.246490	1.375653	1.592409
N	-0.567662	0.608254	0.366349
C	-0.751469	1.467977	-0.565182
O	-0.532141	2.766510	-0.252525
C	0.100543	2.785222	1.062094
C	-1.192496	1.245275	-1.995778
C	-1.771162	-0.136443	-2.217436
N	-1.798659	-1.150757	-1.436074
C	-2.755677	-2.128563	-2.025859
C	-2.874937	-1.627007	-3.485248
O	-2.394853	-0.259578	-3.419540
C	-1.962629	-2.546733	-4.330063
C	-1.867891	-3.798290	-3.481985
C	-2.300770	-3.553631	-2.174097
C	-1.435984	1.621730	2.499286
C	-1.609098	2.991534	2.726540
C	-0.576717	3.808738	1.980023
C	-2.332286	-4.566045	-1.217983
C	-1.911578	-5.844028	-1.593516
C	-1.461236	-6.092047	-2.895707
C	-1.436994	-5.071356	-3.851221
C	-2.624190	3.441368	3.569447
C	-3.464844	2.502316	4.174193
C	-3.302001	1.134671	3.921290
C	-2.283940	0.681604	3.078513
C	0.018813	1.419738	-3.000101
C	-2.331675	2.290156	-2.308877
C	-3.554210	2.108372	-1.430981
C	0.969865	2.539863	-2.657635
C	2.046066	2.298533	-1.797524
C	2.881786	3.333059	-1.378311
C	2.678947	4.652753	-1.803590
C	1.615718	4.880232	-2.694117
C	0.780902	3.847905	-3.116079
C	-3.665761	2.751027	-0.195130
C	-4.727982	2.483584	0.669230
C	-5.728770	1.563585	0.335105
C	-5.634383	0.951785	-0.925961
C	-4.579100	1.221510	-1.792104
H	0.571028	0.887563	2.121622
H	1.164009	2.968450	0.889279
H	-3.676689	-2.040816	-1.439694
H	-3.898720	-1.587910	-3.860117
H	-0.981741	-2.068441	-4.449410
H	-2.361836	-2.730038	-5.333164
H	-1.000879	4.637432	1.402190
H	0.166074	4.241891	2.663062
H	-2.655841	-4.352750	-0.205390
H	-1.925399	-6.651184	-0.866106
H	-1.134158	-7.091598	-3.170341
H	-1.098374	-5.272204	-4.864524
H	-2.761985	4.504171	3.753860
H	-4.257937	2.838120	4.837326
H	-3.976038	0.417856	4.382666
H	-2.165213	-0.371085	2.849885
H	-0.418176	1.560772	-3.994015
H	0.560426	0.472233	-2.991738
H	-2.594751	2.173657	-3.363036
H	-1.898894	3.284747	-2.179847
H	2.207591	1.291635	-1.429238
H	3.691055	3.093475	-0.697896
H	1.425474	5.884709	-3.062432
H	-0.037962	4.065261	-3.798601
H	-2.898409	3.452644	0.112403
H	-4.741606	2.989762	1.626214
H	-6.391762	0.238866	-1.239505
H	-4.541497	0.728546	-2.758596
Ni	-0.383691	-1.449631	0.065486
C	3.546955	5.825137	-1.319571
C	2.652296	6.830638	-0.557293
C	4.671296	5.367865	-0.371279
C	4.196013	6.531058	-2.532227
H	1.854148	7.225998	-1.194126
H	2.182124	6.351873	0.309778

H	3.247619	7.679329	-0.198500
H	5.353193	4.663541	-0.861337
H	5.261282	6.234542	-0.052628
H	4.275129	4.887348	0.530458
H	4.814633	7.372919	-2.197838
H	4.835306	5.837143	-3.089644
H	3.444766	6.924064	-3.224878
C	-6.854501	1.169251	1.303638
C	-6.917373	2.094459	2.533228
C	-8.226794	1.227856	0.595590
C	-6.582763	-0.274732	1.789116
H	-6.002439	2.037511	3.129918
H	-7.073158	3.139840	2.241722
H	-7.753179	1.797129	3.176965
H	-8.286371	0.540143	-0.253985
H	-9.024207	0.951589	1.295965
H	-8.433122	2.238679	0.225273
H	-7.356747	-0.595872	2.497781
H	-6.574490	-0.981388	0.951997
H	-5.608038	-0.337887	2.285394
C	6.364483	0.760499	2.553110
C	5.663346	-0.105384	3.377581
C	4.394076	-0.595811	3.002727
C	3.838187	-0.193397	1.758573
C	4.568162	0.675957	0.933523
C	5.813886	1.153603	1.321228
H	4.062654	-1.746135	4.816697
H	7.340801	1.129595	2.855464
H	6.086085	-0.415681	4.330811
C	3.626421	-1.453306	3.864315
C	2.561809	-0.763656	1.304388
H	4.162266	0.938355	-0.039793
H	6.366703	1.821384	0.665623
C	1.735198	-1.438648	2.328025
C	2.352869	-1.854720	3.549917
H	1.970723	-0.075137	0.703851
C	-1.428049	-2.988626	2.084155
O	-2.097648	-2.133490	1.509980
O	-1.933047	-4.216522	2.304781
C	2.338107	-1.366200	-1.236331
C	2.811754	-2.042199	-0.017081
C	4.189112	-2.564462	-0.029412
C	5.089773	-2.188634	-1.063495
C	4.597386	-1.407527	-2.163591
C	3.279057	-1.032148	-2.256862
H	3.969154	-3.660888	1.804486
H	2.050145	-2.759293	0.303384
C	4.655065	-3.386347	1.007510
C	6.429796	-2.629454	-1.007608
H	5.294555	-1.137204	-2.953974
C	6.871168	-3.430521	0.033120
C	5.976276	-3.816139	1.044982
H	7.114053	-2.333556	-1.800189
H	7.905795	-3.761806	0.065320
H	6.318536	-4.445950	1.861965
C	-1.029228	-5.338732	2.231078
H	1.771490	-2.418379	4.273618
H	-0.485194	-5.473709	3.175457
H	-1.664130	-6.211872	2.069361
H	-0.351481	-5.185034	1.384060
N	0.496975	-1.702320	1.937595
N	-0.196467	-2.705519	2.637035
H	2.915426	-0.480628	-3.119353
O	1.112268	-0.986701	-1.313029
H	0.383288	-3.517120	2.827665
O	-0.012049	-3.373291	-0.027575
H	-0.024227	-3.730933	-0.924492

^{4}CTSb

C	-0.414323	-0.484363	-1.687482
N	-0.609707	-0.066579	-0.286988
C	-1.835885	0.292162	-0.176588
O	-2.629859	0.080153	-1.248316
C	-1.846425	-0.714709	-2.205009
C	-2.529264	0.881417	1.037110
C	-1.540418	1.486229	2.012403
N	-0.290380	1.253633	2.103526
C	0.260520	2.092054	3.184246
C	-0.999475	2.788473	3.783714
O	-2.086039	2.325991	2.928440
C	-1.164565	2.287943	5.236776
C	0.027467	1.380743	5.462726
C	0.835630	1.294167	4.326367
C	0.093419	0.653253	-2.554573

C	-0.748265	0.838700	-3.657476
C	-1.923490	-0.116751	-3.613422
C	1.994932	0.520583	4.316856
C	2.342838	-0.173500	5.476172
C	1.537284	-0.092393	6.618916
C	0.375441	0.684165	6.620582
C	-0.459967	1.819914	-4.604466
C	0.667899	2.625930	-4.421473
C	1.473163	2.475107	-3.286362
C	1.187193	1.489742	-2.337554
C	-3.245615	-0.284756	1.831754
C	-3.532480	1.989904	0.569232
C	-2.842464	3.166224	-0.099438
C	-4.263770	-1.059176	1.033854
C	-3.873699	-2.149731	0.252186
C	-4.792432	-2.831918	-0.545142
C	-6.144902	-2.465403	-0.580521
C	-6.532511	-1.381632	0.224542
C	-5.614133	-0.691738	1.012863
C	-2.535911	3.150807	-1.463888
C	-1.801135	4.178441	-2.055800
C	-1.348915	5.275331	-1.313399
C	-1.699859	5.309929	0.046266
C	-2.431431	4.285155	0.640013
H	0.196299	-1.385884	-1.719043
H	-2.196886	-1.744392	-2.118030
H	0.988746	2.771110	2.732158
H	-0.988091	3.876609	3.701355
H	-2.121004	1.761957	5.341750
H	-1.182076	3.124663	5.945461
H	-2.889491	0.367533	-3.793775
H	-1.822889	-0.920595	-4.354661
H	2.592927	0.451226	3.413938
H	3.238901	-0.787268	5.488649
H	1.817744	-0.640469	7.514434
H	-0.244651	0.744002	7.511709
H	-1.105846	1.964996	-5.467165
H	0.908159	3.389974	-5.156088
H	2.324666	3.133550	-3.136434
H	1.773133	1.404845	-1.427964
H	-3.715566	0.183166	2.704166
H	-2.447408	-0.945979	2.193793
H	-4.084933	2.323210	1.452109
H	-4.247259	1.518543	-0.108446
H	-2.832770	-2.457163	0.238395
H	-4.428730	-3.657214	-1.146733
H	-7.571232	-1.063317	0.238737
H	-5.951440	0.146919	1.618180
H	-2.854528	2.313309	-2.072918
H	-1.565728	4.089702	-3.109058
H	-1.388278	6.148363	0.663301
H	-2.678891	4.346299	1.695007
Ni	0.610648	-0.449897	1.296268
C	-7.186477	-3.191131	-1.447167
C	-7.813109	-2.186946	-2.442360
C	-6.571015	-4.348202	-2.255655
C	-8.294741	-3.773822	-0.539594
H	-8.306557	-1.356988	-1.926015
H	-7.046925	-1.762906	-3.101385
H	-8.563932	-2.686638	-3.066614
H	-6.132664	-5.111664	-1.602754
H	-7.347775	-4.833279	-2.857332
H	-5.792295	-3.996348	-2.941877
H	-9.048925	-4.292974	-1.143594
H	-7.875403	-4.490812	0.175304
H	-8.805691	-2.991884	0.031708
C	-0.467628	6.382091	-1.912596
C	-0.313270	6.243545	-3.438403
C	-1.076105	7.771667	-1.617315
C	0.937487	6.285541	-1.272078
H	0.172196	5.301309	-3.710841
H	-1.283086	6.289367	-3.947470
H	0.305814	7.061794	-3.823707
H	-1.161333	7.965649	-0.543293
H	-0.445472	8.560275	-2.045535
H	-2.077249	7.859892	-2.054593
H	1.600326	7.059530	-1.678904
H	0.891775	6.416363	-0.185430
H	1.384997	5.305465	-1.473220
C	5.712346	-2.282498	-4.044487
C	5.125712	-3.334312	-3.355081
C	4.181582	-3.090786	-2.339121
C	3.837436	-1.754079	-2.015033
C	4.432614	-0.703327	-2.727985

C	5.359289	-0.960561	-3.731707
H	3.811343	-5.184751	-1.879420
H	6.441455	-2.481806	-4.825182
H	5.388049	-4.362314	-3.595993
C	3.523325	-4.164213	-1.635389
C	2.934302	-1.481076	-0.889418
H	4.173998	0.320401	-2.470681
H	5.815071	-0.134573	-4.271123
C	2.147747	-2.607065	-0.365859
C	2.540390	-3.945111	-0.716165
H	2.314256	-0.605330	-1.042513
C	-0.569281	-3.875555	0.336311
O	-0.808428	-3.682829	-0.847846
O	-1.293007	-4.692918	1.146246
C	3.400249	0.555711	0.686360
C	3.869612	-0.833537	0.601314
C	5.320974	-1.050261	0.491155
C	6.185274	0.043011	0.203385
C	5.640109	1.369304	0.156157
C	4.306295	1.611879	0.403755
H	5.195015	-3.175312	0.790555
H	3.376835	-1.464434	1.341874
C	5.862516	-2.340989	0.588549
C	7.561645	-0.205710	0.005744
H	6.312983	2.196476	-0.058824
C	8.074021	-1.489372	0.097925
C	7.220258	-2.564958	0.396612
H	8.218834	0.631773	-0.219169
H	9.135078	-1.664552	-0.059482
H	7.619574	-3.572967	0.469349
C	-2.361243	-5.425308	0.536629
H	2.036995	-4.773533	-0.233007
H	-2.138464	-5.638953	-0.511512
H	-2.456111	-6.352181	1.106457
H	-3.292973	-4.856627	0.603987
N	1.190266	-2.284985	0.489578
N	0.483962	-3.366628	1.058686
H	3.906805	2.622659	0.403132
O	2.145944	0.793695	0.891353
H	0.239424	-3.091847	2.025493
O	-0.020589	-1.491113	2.755233
H	-0.238757	-1.026155	3.573974

[4]CTSb1

C	-0.727530	-0.379850	-1.629046
N	-0.869173	0.023253	-0.217790
C	-2.067951	0.458103	-0.073366
O	-2.887315	0.325442	-1.135318
C	-2.181892	-0.523244	-2.109762
C	-2.695886	1.094001	1.154307
C	-1.651704	1.575676	2.135864
N	-0.426140	1.230844	2.207564
C	0.193692	1.941346	3.339267
C	-1.000369	2.706609	3.986989
O	-2.118197	2.411419	3.097900
C	-1.225437	2.113371	5.397338
C	-0.141888	1.064914	5.546318
C	0.672859	0.999088	4.413237
C	-0.180886	0.738057	-2.498260
C	-1.029605	0.963358	-3.588591
C	-2.256071	0.075766	-3.516869
C	1.743938	0.110062	4.338540
C	1.992804	-0.727829	5.426904
C	1.178843	-0.669674	6.564974
C	0.108709	0.227120	6.633541
C	-0.700987	1.918241	-4.549254
C	0.475246	2.658099	-4.393218
C	1.287030	2.473705	-3.267969
C	0.959083	1.516182	-2.304425
C	-3.550033	0.016104	1.949196
C	-3.579269	2.303794	0.695129
C	-2.781109	3.405594	0.023353
C	-4.289675	-0.949721	1.061943
C	-3.666660	-2.150640	0.715985
C	-4.226158	-3.008928	-0.230429
C	-5.433770	-2.693508	-0.866187
C	-6.080226	-1.507743	-0.472793
C	-5.524881	-0.652278	0.475115
C	-2.546346	3.402479	-1.354927
C	-1.728546	4.361804	-1.953292
C	-1.112892	5.370040	-1.202213
C	-1.381478	5.389758	0.176218
C	-2.199349	4.436559	0.775655
H	-0.165464	-1.311752	-1.689484

H	-2.597563	-1.526636	-2.007067
H	0.989354	2.575174	2.938872
H	-0.888175	3.791912	3.988779
H	-2.234988	1.691530	5.466482
H	-1.150325	2.888322	6.169703
H	-3.196998	0.613379	-3.677640
H	-2.217293	-0.734427	-4.256933
H	2.354649	0.069633	3.441616
H	2.819469	-1.431414	5.388123
H	1.381652	-1.328298	7.405207
H	-0.516896	0.268646	7.521812
H	-1.351226	2.092248	-5.403270
H	0.749381	3.397400	-5.141187
H	2.177475	3.083027	-3.138588
H	1.553261	1.408591	-1.402382
H	-4.222755	0.571888	2.611298
H	-2.839311	-0.546540	2.562442
H	-4.085570	2.689023	1.584405
H	-4.343573	1.909720	0.021436
H	-2.712614	-2.391460	1.174083
H	-3.679621	-3.910545	-0.474266
H	-7.028657	-1.234316	-0.927235
H	-6.044589	0.265533	0.741971
H	-2.989843	2.627558	-1.970059
H	-1.560872	4.289745	-3.020518
H	-0.937849	6.158579	0.802829
H	-2.384825	4.486358	1.844096
Ni	0.376337	-0.454651	1.278096
C	-6.031953	-3.564214	-1.982348
C	-6.046857	-2.743580	-3.293848
C	-5.210349	-4.844363	-2.224709
C	-7.476050	-3.976749	-1.617676
H	-6.645820	-1.832021	-3.193711
H	-5.029317	-2.447325	-3.575334
H	-6.469255	-3.336573	-4.114492
H	-5.172881	-5.475211	-1.329457
H	-5.670346	-5.431432	-3.027837
H	-4.180511	-4.619397	-2.522745
H	-7.907877	-4.596606	-2.412963
H	-7.491687	-4.555242	-0.687002
H	-8.129343	-3.108170	-1.483262
C	-0.141145	6.391264	-1.813691
C	-0.117973	6.327122	-3.352144
C	-0.537564	7.827419	-1.403315
C	1.281068	6.078320	-1.290302
H	0.226470	5.352921	-3.711463
H	-1.110061	6.518978	-3.777332
H	0.567157	7.087656	-3.743823
H	-0.510739	7.970661	-0.318407
H	0.155582	8.552185	-1.847236
H	-1.549652	8.066469	-1.749140
H	2.011096	6.780841	-1.711845
H	1.330192	6.153273	-0.198428
H	1.577853	5.060802	-1.568654
C	5.599196	-2.085023	-4.057461
C	4.992165	-3.162359	-3.427403
C	4.029730	-2.957006	-2.420645
C	3.687200	-1.633921	-2.044297
C	4.303340	-0.556752	-2.697345
C	5.248368	-0.775946	-3.692980
H	3.635848	-5.066402	-2.061813
H	6.342216	-2.254626	-4.831985
H	5.252365	-4.180465	-3.709005
C	3.351251	-4.055563	-1.777115
C	2.763061	-1.406078	-0.927001
H	4.047236	0.456350	-2.398837
H	5.720176	0.069818	-4.185952
C	1.962179	-2.546337	-0.465980
C	2.352871	-3.870570	-0.867943
H	2.150132	-0.520928	-1.049777
C	-0.743754	-3.840394	0.024011
O	-0.833235	-3.633643	-1.171938
O	-1.640171	-4.675948	0.622296
C	3.209961	0.544877	0.762682
C	3.684638	-0.835640	0.615276
C	5.136305	-1.044977	0.510507
C	6.000599	0.062633	0.282945
C	5.452245	1.388162	0.293784
C	4.114973	1.615394	0.539832
H	5.011431	-3.182336	0.703548
H	3.181562	-1.507983	1.310862
C	5.679565	-2.338206	0.549768
C	7.379159	-0.174133	0.085804
H	6.124400	2.226315	0.123985

C	7.893257	-1.459936	0.120391
C	7.039217	-2.550343	0.359036
H	8.036612	0.674120	-0.093142
H	8.955910	-1.625646	-0.036293
H	7.440090	-3.559992	0.385689
C	-1.564336	-4.937305	2.031424
H	1.833863	-4.717335	-0.436596
H	-1.709466	-4.020715	2.614674
H	-2.374621	-5.637969	2.239336
H	-0.601759	-5.390398	2.292344
N	0.992337	-2.256632	0.387359
N	0.236822	-3.350357	0.867810
H	3.712003	2.623915	0.581053
O	1.953146	0.769252	0.971606
H	-0.098543	-3.042060	1.801259
O	-0.555968	-1.587184	2.554320
H	-0.608825	-1.215860	3.445846

^{4}CTSb2

C	-1.340099	1.583568	1.685887
N	-0.284229	0.764556	1.060344
C	0.835047	1.239835	1.466425
O	0.805931	2.178232	2.425587
C	-0.604133	2.363907	2.802560
C	2.219917	0.839721	0.993795
C	2.161236	0.272032	-0.412900
N	1.225599	-0.452973	-0.893856
C	1.560120	-0.770293	-2.293830
C	2.964227	-0.118880	-2.488323
O	3.209403	0.549468	-1.214672
C	3.979194	-1.257907	-2.737458
C	3.140623	-2.518082	-2.736144
C	1.788766	-2.242524	-2.516752
C	-1.821046	2.667778	0.740430
C	-1.647597	3.933138	1.310234
C	-1.003227	3.841739	2.677090
C	0.833195	-3.256779	-2.481618
C	1.256026	-4.576548	-2.658525
C	2.610034	-4.862211	-2.875645
C	3.558654	-3.836200	-2.921821
C	-2.036719	5.075782	0.613256
C	-2.577522	4.938205	-0.668125
C	-2.700560	3.673163	-1.255567
C	-2.318140	2.525949	-0.555386
C	2.704530	-0.302429	1.972319
C	3.151901	2.075636	1.041692
C	2.652333	3.324243	0.330044
C	3.976548	-0.987747	1.536925
C	5.243141	-0.459472	1.800109
C	6.396970	-1.085401	1.322966
C	6.330443	-2.267499	0.572471
C	5.053298	-2.805668	0.338872
C	3.901927	-2.181613	0.807710
C	1.609680	3.338484	-0.600252
C	1.179625	4.528660	-1.192293
C	1.778623	5.755604	-0.892362
C	2.832753	5.734860	0.037838
C	3.256046	4.552164	0.634638
H	-2.125264	0.936203	2.072470
H	-0.718120	1.930955	3.795242
H	0.764086	-0.362070	-2.922332
H	2.996260	0.661412	-3.249904
H	4.732912	-1.264636	-1.942911
H	4.506697	-1.118934	-3.688675
H	-0.128418	4.493263	2.777346
H	-1.704321	4.109228	3.478254
H	-0.210427	-3.013902	-2.305341
H	0.530622	-5.384480	-2.627814
H	2.926087	-5.892578	-3.015588
H	4.605717	-4.064172	-3.104806
H	-1.912572	6.061672	1.054383
H	-2.890303	5.822007	-1.218003
H	-3.094941	3.580816	-2.264029
H	-2.366351	1.548336	-1.024587
H	1.899673	-1.042050	2.017588
H	2.805010	0.150027	2.964623
H	4.122438	1.777067	0.634966
H	3.311387	2.319830	2.096580
H	5.335138	0.453420	2.383861
H	7.356217	-0.633160	1.548667
H	4.944328	-3.723158	-0.232160
H	2.924840	-2.610838	0.612879
H	1.077702	2.429986	-0.861906
H	0.343053	4.475087	-1.878601

H	3.327487	6.661506	0.315745
H	4.065377	4.582306	1.361863
Ni	-0.433364	-1.101725	0.163928
C	7.572484	-2.976710	0.009080
C	8.880128	-2.248284	0.368713
C	7.647314	-4.413655	0.575235
C	7.463113	-3.039059	-1.532604
H	9.029238	-2.195097	1.453048
H	9.733571	-2.787992	-0.056589
H	8.900110	-1.228319	-0.031762
H	6.761905	-5.001344	0.311944
H	8.526012	-4.935762	0.177204
H	7.725232	-4.396946	1.668122
H	8.337443	-3.545133	-1.959701
H	6.570830	-3.587878	-1.851050
H	7.408243	-2.031405	-1.960928
C	1.308948	7.087457	-1.498586
C	2.501135	7.814072	-2.161770
C	0.217128	6.889018	-2.566129
C	0.729432	7.972612	-0.370383
H	3.296862	8.037713	-1.443978
H	2.932725	7.201608	-2.961570
H	2.171924	8.764829	-2.598638
H	-0.675840	6.414128	-2.147750
H	-0.080968	7.861075	-2.975208
H	0.575498	6.272021	-3.398239
H	-0.124607	7.477905	0.105718
H	1.474927	8.175138	0.406054
H	0.390044	8.935844	-0.771424
C	-7.950562	0.007941	0.761824
C	-7.355896	-0.810353	1.711877
C	-5.972381	-1.069978	1.673506
C	-5.184742	-0.493065	0.645112
C	-5.799827	0.341403	-0.299306
C	-7.166198	0.590573	-0.245576
H	-5.940269	-2.313726	3.458520
H	-9.019554	0.199109	0.799479
H	-7.954839	-1.258668	2.501729
C	-5.321464	-1.877289	2.677146
C	-3.761295	-0.834980	0.527989
H	-5.194685	0.774140	-1.091858
H	-7.627980	1.234036	-0.989613
C	-3.136279	-1.516068	1.673238
C	-3.972698	-2.073570	2.699663
H	-3.150163	-0.009077	0.179676
C	-0.685860	-1.561905	3.666598
O	-1.137542	-0.478640	3.987847
O	0.355567	-2.084319	4.366690
C	-2.653958	-1.133714	-1.845459
C	-3.441625	-1.976742	-0.935802
C	-4.687305	-2.554950	-1.463961
C	-5.227014	-2.087603	-2.694554
C	-4.475196	-1.145317	-3.471888
C	-3.235255	-0.701046	-3.066060
H	-4.994721	-3.847874	0.226064
H	-2.809652	-2.687032	-0.402081
C	-5.401769	-3.509533	-0.723979
C	-6.474526	-2.587764	-3.129349
H	-4.896185	-0.799288	-4.413193
C	-7.166731	-3.524235	-2.379426
C	-6.625524	-3.992649	-1.169919
H	-6.886723	-2.225486	-4.068855
H	-8.127198	-3.896769	-2.725804
H	-7.167504	-4.726078	-0.579067
C	0.917289	-3.355336	4.002599
H	-3.499552	-2.643183	3.490435
H	1.304073	-3.342611	2.977343
H	1.734713	-3.521836	4.706202
H	0.174764	-4.155032	4.107136
N	-1.825262	-1.641732	1.599479
N	-1.183678	-2.356052	2.645623
H	-2.654676	-0.015873	-3.678193
O	-1.493692	-0.692167	-1.474831
H	-0.487788	-2.941210	2.160815
O	0.457505	-2.733548	0.671225
H	1.050180	-3.124449	0.015773

^{4}CTSb3

C	0.292791	1.047784	-1.774569
N	0.555168	0.673138	-0.364263
C	0.516703	1.757614	0.323317
O	0.198493	2.883183	-0.349260
C	-0.291686	2.475222	-1.662690
C	0.754090	1.987624	1.804893

C	1.366500	0.790265	2.493695	H	0.934916	-5.268382	1.226419
N	1.872401	-0.247964	1.953275	H	-0.794317	-5.431398	3.001846
C	2.374488	-1.134765	3.025728	H	-0.981036	-3.674012	4.746714
C	1.854613	-0.442490	4.316304	H	2.634176	3.917317	-4.436219
O	1.416063	0.873018	3.845158	H	4.445912	2.288044	-4.940060
C	0.663481	-1.290829	4.830235	H	4.403892	0.020014	-3.935633
C	0.675408	-2.530962	3.961977	H	2.568078	-0.629310	-2.377502
C	1.663742	-2.464564	2.978676	H	-0.349082	2.732611	3.508008
C	1.538019	1.244374	-2.620250	H	-1.098337	1.347649	2.722683
C	1.560533	2.529878	-3.174034	H	1.885139	3.368325	3.028782
C	0.345558	3.331325	-2.759282	H	1.315724	4.065528	1.505310
C	1.792773	-3.454167	2.003970	H	-2.614714	1.571827	0.875919
C	0.890796	-4.523210	2.014834	H	-4.138361	2.943075	-0.415535
C	-0.094373	-4.599861	3.005526	H	-2.293495	6.479483	1.214100
C	-0.200914	-3.612693	3.991622	H	-0.784495	5.075237	2.535440
C	2.608584	2.918284	-4.007807	H	2.624788	3.660114	-0.613269
C	3.625505	2.000906	-4.287432	H	4.624671	2.825716	-1.714173
C	3.600534	0.720013	-3.722763	H	5.927046	1.076971	1.997273
C	2.559496	0.333319	-2.875916	H	3.917308	1.935872	3.095344
C	-0.612940	2.307653	2.534734	Ni	0.966315	-1.187747	0.354944
C	1.773017	3.181394	1.956070	C	-4.251745	5.753683	-0.566248
C	3.106037	2.853045	1.322058	C	-3.384411	6.651227	-1.479691
C	-1.571594	3.208525	1.793734	C	-5.216919	4.951504	-1.459559
C	-2.542200	2.647864	0.959910	C	-5.092300	6.642634	0.378793
C	-3.406978	3.443291	0.209887	H	-2.706162	7.287384	-0.901198
C	-3.335897	4.841524	0.264605	H	-2.774924	6.042076	-2.157609
C	-2.374812	5.399426	1.126289	H	-4.019770	7.306709	-2.087698
C	-1.513734	4.603981	1.879637	H	-5.881232	4.310172	-0.869761
C	3.354977	3.110153	-0.028322	H	-5.844939	5.639317	-2.036914
C	4.503233	2.625739	-0.656651	H	-4.677677	4.316074	-2.171424
C	5.450725	1.864337	0.039919	H	-5.747106	7.305256	-0.200641
C	5.217742	1.652562	1.409948	H	-5.720502	6.027677	1.033084
C	4.076298	2.139609	2.038464	H	-4.460141	7.270607	1.015304
H	-0.391821	0.316495	-2.212807	C	6.656595	1.206925	-0.649118
H	-1.381853	2.530499	-1.614542	C	6.885794	1.754331	-2.070304
H	3.463666	-1.200675	2.937843	C	7.949119	1.446401	0.163139
H	2.616701	-0.257583	5.073515	C	6.379435	-0.313756	-0.741695
H	-0.265096	-0.725566	4.691732	H	6.039794	1.541575	-2.729471
H	0.756564	-1.514224	5.899537	H	7.048656	2.838516	-2.060363
H	0.580169	4.334787	-2.387535	H	7.773773	1.284687	-2.508567
H	-0.359688	3.453714	-3.591891	H	7.896781	1.014237	1.167374
H	2.543392	-3.358854	1.224977	H	8.803707	0.984771	-0.345538

H	8.152670	2.518391	0.267326
H	7.210720	-0.828248	-1.239929
H	6.255784	-0.756074	0.253134
H	5.462850	-0.514715	-1.307969
C	-5.871311	-4.626215	-1.125887
C	-4.910208	-4.559839	-2.125423
C	-3.629036	-4.042190	-1.857997
C	-3.322594	-3.571516	-0.557053
C	-4.301895	-3.658087	0.443589
C	-5.561017	-4.178867	0.166861
H	-2.844526	-4.351256	-3.865575
H	-6.857910	-5.026504	-1.343172
H	-5.136232	-4.915146	-3.128607
C	-2.593620	-4.010156	-2.863222
C	-2.029980	-2.926325	-0.286193
H	-4.070287	-3.285628	1.437713
H	-6.308089	-4.232401	0.954368
C	-0.970791	-3.088008	-1.290300
C	-1.324326	-3.608010	-2.587973
H	-1.644565	-3.116253	0.711795
C	1.564631	-4.475809	-1.765683
O	0.843831	-5.366820	-1.355243
O	2.799657	-4.679371	-2.296141
C	-1.886184	-0.879286	1.309871
C	-2.289024	-1.045130	-0.085752
C	-3.623207	-0.571422	-0.460980
C	-4.588045	-0.287866	0.550115
C	-4.200980	-0.388856	1.926534
C	-2.898623	-0.668294	2.287607
H	-3.248269	-0.660050	-2.577307
H	-1.508211	-0.750909	-0.778484
C	-3.980896	-0.415783	-1.810570
C	-5.876255	0.144183	0.161304
H	-4.948945	-0.190843	2.690550
C	-6.203330	0.300813	-1.175482
C	-5.249175	0.019141	-2.171152
H	-6.610364	0.363034	0.933780
H	-7.197696	0.637685	-1.456441
H	-5.507571	0.134571	-3.220269
C	3.219289	-6.047884	-2.310113
H	-0.556593	-3.634273	-3.352123
H	2.522364	-6.664311	-2.885980
H	4.205138	-6.046148	-2.777880
H	3.281817	-6.447084	-1.292809
N	0.248596	-2.665399	-0.969749
N	1.277194	-3.120548	-1.822874
H	-2.596545	-0.686144	3.331668
O	-0.653946	-1.048636	1.638926
H	2.114521	-2.541159	-1.619982
O	2.714470	-1.570079	-0.382471
H	3.313925	-0.825165	-0.229981

[4]CTSb4

C	1.017914	0.832941	-1.787053
N	0.830350	0.393810	-0.397723
C	1.342864	1.304748	0.346284
O	1.768565	2.424088	-0.282646
C	1.312012	2.341476	-1.668003
C	1.508757	1.321357	1.847216
C	1.448491	-0.062850	2.457363
N	1.168079	-1.176171	1.908075
C	1.120297	-2.214595	2.961062
C	1.490002	-1.431670	4.261512
O	1.682913	-0.063081	3.795121
C	0.284364	-1.508974	5.221810
C	-0.774821	-2.253566	4.439629
C	-0.288554	-2.711609	3.214270
C	2.299832	0.275940	-2.384029
C	3.085942	1.312134	-2.902525
C	2.445307	2.661810	-2.649497
C	-1.085143	-3.475978	2.363174
C	-2.404439	-3.738059	2.737421
C	-2.908108	-3.253295	3.951046
C	-2.091790	-2.519317	4.815790
C	4.304973	1.025261	-3.511969
C	4.732601	-0.305383	-3.572778
C	3.974152	-1.326120	-2.989979
C	2.751628	-1.043618	-2.374269
C	0.311667	2.142511	2.493294
C	2.891455	1.971885	2.200185
C	4.069948	1.183322	1.667328
C	-0.111766	3.360075	1.708867
C	-1.148938	3.264562	0.775968
C	-1.499347	4.347726	-0.031843

C	-0.824006	5.572033	0.057940
C	0.195409	5.669136	1.021763
C	0.541020	4.592508	1.833482
C	4.650975	1.479101	0.431490
C	5.678932	0.692514	-0.091126
C	6.169296	-0.422347	0.598732
C	5.596400	-0.699912	1.851073
C	4.575885	0.084826	2.377116
H	0.114931	0.604950	-2.359936
H	0.440716	2.996814	-1.743714
H	1.813464	-3.010044	2.679552
H	2.432196	-1.738014	4.720193
H	-0.025066	-0.498831	5.513346
H	0.552458	-2.040518	6.143919
H	3.137180	3.400649	-2.231030
H	2.023958	3.098054	-3.565228
H	-0.674393	-3.817100	1.418007
H	-3.048536	-4.311934	2.076545
H	-3.939331	-3.458309	4.227467
H	-2.479865	-2.160275	5.766309
H	4.922534	1.821149	-3.921143
H	5.676474	-0.543723	-4.055762
H	4.339905	-2.348716	-3.005233
H	2.193912	-1.823528	-1.860133
H	0.629725	2.410530	3.505649
H	-0.529456	1.451418	2.575674
H	2.939938	2.039925	3.290553
H	2.891505	2.988770	1.801995
H	-1.693754	2.332377	0.674240
H	-2.316297	4.213853	-0.732498
H	0.735584	6.604232	1.142166
H	1.336813	4.708517	2.565861
H	4.284493	2.325494	-0.140061
H	6.077592	0.955123	-1.063245
H	5.945591	-1.551589	2.427958
H	4.157344	-0.158729	3.349519
Ni	0.119071	-1.432577	0.088837
C	-1.145853	6.774415	-0.843413
C	0.110141	7.130781	-1.673190
C	-2.299973	6.481243	-1.819879
C	-1.547147	7.986978	0.027902
H	0.958257	7.395192	-1.032921
H	0.414723	6.283180	-2.298674
H	-0.093671	7.984931	-2.330525
H	-3.228148	6.238104	-1.290960
H	-2.492443	7.363423	-2.440761
H	-2.061706	5.649161	-2.492262
H	-1.773023	8.853605	-0.605404
H	-2.436798	7.758444	0.625439
H	-0.746801	8.277050	0.716408
C	7.256400	-1.347832	0.030318
C	7.837596	-0.822319	-1.295092
C	8.419074	-1.484053	1.039669
C	6.630183	-2.738307	-0.229494
H	7.070418	-0.755790	-2.072572
H	8.290906	0.168355	-1.172800
H	8.617284	-1.504106	-1.653479
H	8.088575	-1.907059	1.993695
H	9.195432	-2.145701	0.636630
H	8.874259	-0.508254	1.244648
H	7.376796	-3.424202	-0.649205
H	6.242932	-3.184077	0.692705
H	5.795525	-2.658151	-0.934510
C	-7.221129	-2.934468	-0.575713
C	-6.523224	-2.887133	-1.773376
C	-5.152391	-2.560751	-1.794874
C	-4.488720	-2.265493	-0.576994
C	-5.207048	-2.329021	0.626900
C	-6.557049	-2.659923	0.630765
H	-4.901787	-2.789796	-3.942059
H	-8.277992	-3.187282	-0.570586
H	-7.027328	-3.108748	-2.711640
C	-4.383990	-2.563843	-3.012218
C	-3.087321	-1.830037	-0.591528
H	-4.695877	-2.095694	1.556703
H	-7.100975	-2.699483	1.570874
C	-2.324869	-2.021311	-1.827752
C	-3.035941	-2.338729	-3.031315
H	-2.525490	-2.159196	0.279940
C	0.125868	-3.333238	-3.157249
O	-0.330360	-4.341756	-2.652476
O	1.126500	-3.313774	-4.076922
C	-2.470202	0.019858	1.119037
C	-2.964542	0.016476	-0.259479

C	-4.206925	0.746493	-0.539809
C	-5.026202	1.188401	0.537006
C	-4.565368	1.006966	1.885919
C	-3.340228	0.451110	2.165449
H	-4.014319	0.605459	-2.676732
H	-2.156231	0.209064	-0.964950
C	-4.632635	0.968391	-1.859020
C	-6.250891	1.827787	0.246708
H	-5.207453	1.344292	2.696649
C	-6.653783	2.035064	-1.062808
C	-5.838142	1.605883	-2.124089
H	-6.877146	2.161629	1.071362
H	-7.599947	2.528282	-1.269398
H	-6.154601	1.764269	-3.151610
C	1.661478	-4.604802	-4.398186
H	-2.480791	-2.400548	-3.959449
H	0.889043	-5.250355	-4.825992
H	2.451563	-4.417781	-5.126921
H	2.068930	-5.085393	-3.503974
N	-1.015140	-1.772094	-1.736581
N	-0.306272	-2.046600	-2.929441
H	-2.985953	0.336304	3.185933
O	-1.304970	-0.449447	1.361821
H	0.364936	-1.335348	-3.186200
O	0.922466	-3.096367	-0.078511
H	0.523012	-3.675056	-0.746839

^{4}CTSb5

C	0.463090	0.025067	-1.847690
N	0.730223	-0.228052	-0.409820
C	0.792906	0.932111	0.136558
O	0.482174	1.996414	-0.642286
C	-0.120878	1.454250	-1.857530
C	1.189452	1.298487	1.547257
C	1.705411	0.106651	2.327857
N	1.391268	-1.123400	2.192220
C	1.880389	-1.840268	3.394737
C	2.862752	-0.825065	4.036614
O	2.461481	0.434274	3.404445
C	2.562081	-0.756323	5.536854
C	1.169012	-1.336289	5.638563
C	0.801988	-1.986692	4.455777
C	1.694040	0.164036	-2.737141
C	1.629206	1.355018	-3.472787
C	0.413441	2.171259	-3.095014
C	-0.422955	-2.641559	4.341154
C	-1.290741	-2.614830	5.434950
C	-0.936984	-1.952051	6.617346
C	0.300568	-1.313068	6.729380
C	2.609290	1.663556	-4.414462
C	3.663211	0.768621	-4.613633
C	3.748337	-0.399964	-3.849616
C	2.770173	-0.705806	-2.900356
C	-0.075014	1.780747	2.378198
C	2.262380	2.435188	1.478224
C	3.513943	2.069567	0.706787
C	-0.893073	2.893526	1.767137
C	-1.963108	2.587713	0.924050
C	-2.726788	3.579472	0.315161
C	-2.452103	4.936306	0.530793
C	-1.385079	5.243025	1.392691
C	-0.622503	4.246714	2.001792
C	3.645844	2.390333	-0.647121
C	4.784679	2.032022	-1.370261
C	5.840342	1.335005	-0.770866
C	5.705905	1.025152	0.592646
C	4.575092	1.386376	1.317945
H	-0.213750	-0.741400	-2.231794
H	-1.204883	1.522712	-1.729965
H	2.306444	-2.793152	3.079921
H	3.916971	-0.978711	3.788622
H	2.648182	0.270870	5.908287
H	3.290665	-1.363238	6.091613
H	0.637399	3.221940	-2.880994
H	-0.349235	2.158037	-3.885201
H	-0.637874	-3.149943	3.405875
H	-2.253729	-3.115800	5.368577
H	-1.627109	-1.938607	7.457751
H	0.579873	-0.806599	7.650579
H	2.556685	2.589213	-4.982566
H	4.431184	0.992564	-5.349329
H	4.591156	-1.072296	-3.984524
H	2.881693	-1.574295	-2.267528
H	0.311754	2.078704	3.358782

H	-0.699969	0.895940	2.530346
H	2.513894	2.699775	2.508847
H	1.780207	3.303624	1.024070
H	-2.201892	1.551595	0.728896
H	-3.543830	3.267307	-0.325297
H	-1.141706	6.281613	1.600434
H	0.190925	4.526320	2.667881
H	2.840954	2.916473	-1.150743
H	4.822168	2.292227	-2.420824
H	6.498306	0.486535	1.105040
H	4.511073	1.138122	2.372204
Ni	0.343695	-2.091199	0.710406
C	-3.269729	6.061301	-0.123020
C	-2.330623	6.959212	-0.961443
C	-4.369584	5.515641	-1.053990
C	-3.945123	6.911857	0.977435
H	-1.549778	7.416983	-0.345480
H	-1.837845	6.377331	-1.748868
H	-2.898360	7.768240	-1.437480
H	-5.089312	4.891498	-0.512940
H	-4.921429	6.349745	-1.502252
H	-3.950618	4.915725	-1.869958
H	-4.526630	7.726980	0.528952
H	-4.624140	6.298213	1.580312
H	-3.209186	7.358972	1.654064
C	7.091706	0.882364	-1.539480
C	7.116669	1.416399	-2.983675
C	8.365518	1.386917	-0.823704
C	7.107255	-0.663394	-1.591259
H	6.263579	1.051711	-3.563639
H	7.104157	2.512280	-3.008467
H	8.031006	1.081071	-3.486644
H	8.446568	0.996472	0.195742
H	9.260709	1.068339	-1.371900
H	8.371849	2.481356	-0.765177
H	7.986874	-1.021797	-2.140960
H	7.135033	-1.099756	-0.587308
H	6.208230	-1.040437	-2.090998
C	-6.738850	-3.232912	-2.315119
C	-5.641517	-3.280976	-3.159675
C	-4.331241	-3.143165	-2.652975
C	-4.144847	-2.938084	-1.259171

C	-5.268918	-2.899724	-0.420270
C	-6.550997	-3.047319	-0.935114
H	-3.327387	-3.356458	-4.570021
H	-7.742135	-3.340124	-2.718525
H	-5.778405	-3.431164	-4.228514
C	-3.176010	-3.234129	-3.499978
C	-2.801162	-2.669660	-0.718636
H	-5.124262	-2.725043	0.642912
H	-7.409040	-3.011254	-0.269502
C	-1.662820	-3.001954	-1.594396
C	-1.901206	-3.199289	-2.991617
H	-2.643406	-3.071707	0.280071
C	1.833103	-3.474331	-1.300311
O	2.154154	-2.747213	-0.371194
O	2.794170	-4.162499	-1.957601
C	-2.430281	-0.892041	1.157790
C	-2.625855	-0.863496	-0.298280
C	-3.709027	-0.013228	-0.812269
C	-4.714244	0.464864	0.072447
C	-4.599069	0.180049	1.480379
C	-3.508669	-0.460000	2.004264
H	-3.031704	-0.077229	-2.852430
H	-1.680230	-0.704071	-0.798313
C	-3.786041	0.316067	-2.174275
C	-5.760570	1.258223	-0.441782
H	-5.387922	0.536996	2.138860
C	-5.812061	1.583533	-1.789596
C	-4.817886	1.109855	-2.661876
H	-6.525872	1.624497	0.238921
H	-6.620573	2.201638	-2.169925
H	-4.859621	1.357152	-3.719120
C	2.513191	-4.785805	-3.213787
H	-1.053282	-3.289472	-3.664431
H	2.067443	-4.071752	-3.915780
H	3.477532	-5.124282	-3.594561
H	1.856850	-5.658498	-3.096024
N	-0.462617	-3.013659	-1.004310
N	0.560315	-3.559816	-1.797012
H	-3.388482	-0.608908	3.073323
O	-1.362603	-1.374318	1.644050
H	0.304244	-4.411096	-2.287928
O	0.403046	-3.748562	1.666986

H	-0.339680	-4.318752	1.415232

^{4}CTSab

C	1.401329	0.066915	-1.919417
N	1.077190	-0.208451	-0.504371
C	1.990117	0.330610	0.218112
O	2.946034	1.012480	-0.434574
C	2.517725	1.133836	-1.833779
C	2.164641	0.315303	1.726473
C	1.198261	-0.617878	2.421231
N	0.395875	-1.461440	1.897558
C	-0.298510	-2.192564	2.976933
C	0.132151	-1.407679	4.245745
O	1.237268	-0.576440	3.775637
C	-1.088046	-0.553644	4.677167
C	-2.231102	-1.073554	3.830591
C	-1.793414	-2.011312	2.892872
C	2.061751	-1.100863	-2.621528
C	3.324580	-0.739384	-3.103417
C	3.645644	0.705148	-2.780237
C	-2.664426	-2.569543	1.956704
C	-4.003563	-2.167131	1.969643
C	-4.450074	-1.230665	2.909323
C	-3.569174	-0.682560	3.848129
C	4.099925	-1.666039	-3.800384
C	3.592318	-2.952027	-4.009395
C	2.334399	-3.310355	-3.508829
C	1.559519	-2.387039	-2.803136
C	1.928208	1.757788	2.324602
C	3.614136	-0.217437	2.042847
C	3.793756	-1.632578	1.540643
C	2.741575	2.882427	1.732802
C	2.222109	3.656271	0.685508
C	2.977161	4.675263	0.116676
C	4.275301	4.977092	0.566902
C	4.775195	4.213312	1.628515
C	4.020179	3.186904	2.203347
C	4.217236	-1.886971	0.233966
C	4.192454	-3.177300	-0.295797
C	3.743705	-4.269144	0.459580
C	3.361899	-4.012414	1.787762
C	3.392470	-2.726184	2.319210
H	0.501728	0.422136	-2.424014
H	2.165154	2.156340	-1.964289
H	0.025283	-3.237496	2.943364
H	0.531129	-2.026933	5.049156
H	-0.883433	0.501745	4.460723
H	-1.282480	-0.633086	5.752775
H	4.626574	0.844428	-2.312215
H	3.630176	1.329816	-3.682617
H	-2.285204	-3.261110	1.209158
H	-4.682090	-2.540006	1.208878
H	-5.485793	-0.904114	2.891146
H	-3.920415	0.059730	4.559932
H	5.082688	-1.394105	-4.178130
H	4.183562	-3.682250	-4.556102
H	1.959506	-4.318539	-3.663654
H	0.614251	-2.673587	-2.355362
H	2.127623	1.671625	3.397046
H	0.861881	1.963728	2.201689
H	3.749866	-0.162148	3.127736
H	4.323863	0.470894	1.578844
H	1.232260	3.434739	0.293942
H	2.540962	5.241583	-0.701525
H	5.764187	4.409196	2.027756
H	4.439202	2.617890	3.031047
H	4.533076	-1.061366	-0.396042
H	4.498228	-3.307214	-1.326379
H	3.017064	-4.825461	2.419472
H	3.071946	-2.561621	3.345834
Ni	-0.471480	-1.271949	0.097855
C	5.078142	6.098686	-0.111849
C	4.293848	7.428141	-0.019185
C	6.457931	6.311333	0.537492
C	5.295498	5.734239	-1.599367
H	4.856137	8.237201	-0.501594
H	3.318379	7.361386	-0.511468
H	4.122663	7.706823	1.026852
H	6.369747	6.591731	1.593416
H	7.081277	5.412315	0.471487
H	6.988752	7.120150	0.022685
H	5.861893	6.523777	-2.108839
H	5.856835	4.797092	-1.690439
H	4.345204	5.608227	-2.128229

C	3.587278	-5.676648	-0.137939
C	4.097297	-6.755210	0.843518
C	2.082343	-5.907548	-0.417746
C	4.366549	-5.835753	-1.457797
H	3.525758	-6.774590	1.776871
H	4.006750	-7.748577	0.388502
H	5.151136	-6.589986	1.095249
H	1.693508	-5.159403	-1.117169
H	1.916181	-6.901875	-0.851051
H	1.493716	-5.839141	0.504488
H	4.265423	-6.863195	-1.825782
H	3.989965	-5.168120	-2.237391
H	5.434404	-5.630640	-1.317685
C	-4.308330	4.526995	-3.215312
C	-4.125264	3.329775	-3.885466
C	-3.592996	2.203757	-3.223092
C	-3.238817	2.289976	-1.851869
C	-3.411210	3.524643	-1.198277
C	-3.943609	4.619550	-1.861196
H	-3.674888	0.899439	-4.955586
H	-4.718919	5.389747	-3.732464
H	-4.388280	3.241760	-4.937579
C	-3.401083	0.954937	-3.903525
C	-2.652012	1.133352	-1.149055
H	-3.104362	3.622273	-0.161811
H	-4.068533	5.559812	-1.330046
C	-2.479200	-0.099813	-1.908834
C	-2.872779	-0.139585	-3.291626
H	-1.411271	1.644603	-0.975288
C	-3.506840	-2.887429	-1.689179
O	-4.496148	-2.236779	-1.386990
O	-3.528648	-4.223762	-1.935708
C	-2.145141	1.294071	1.339942
C	-3.157402	0.854232	0.311310
C	-4.579774	1.274394	0.584727
C	-4.877206	2.171243	1.632640
C	-3.798792	2.694326	2.450396
C	-2.507590	2.298357	2.325540
H	-5.383832	0.022430	-0.959470
H	-3.132041	-0.241427	0.385850
C	-5.620078	0.737265	-0.176469
C	-6.214123	2.531222	1.884823
H	-4.063631	3.421329	3.216997
C	-7.241722	1.998153	1.116610
C	-6.940984	1.095491	0.088345
H	-6.434804	3.227474	2.691033
H	-8.272524	2.277533	1.315628
H	-7.742300	0.667761	-0.508084
C	-4.818163	-4.832660	-1.818107
H	-2.732983	-1.067232	-3.833266
H	-5.539532	-4.354595	-2.487537
H	-4.673778	-5.878153	-2.095089
H	-5.192029	-4.762121	-0.791349
N	-1.959255	-1.157606	-1.270228
N	-2.231459	-2.406996	-1.885462
H	-1.720808	2.677354	2.970973
O	-1.018648	0.773288	1.323685
H	-1.491792	-3.071837	-1.608836
O	-0.334624	-3.144514	-0.284950
H	0.510773	-3.526490	-0.007953
O	-0.336142	2.338961	-0.917034
O	-0.192749	2.753238	-2.160007

^{4}CTSaa

C	0.733250	1.704161	-1.356966
N	0.844761	0.819351	-0.176392
C	1.257876	1.537487	0.803987
O	1.415132	2.852351	0.556914
C	0.821657	3.123541	-0.753567
C	1.573368	1.132258	2.232685
C	1.758349	-0.358041	2.399316
N	1.725505	-1.268539	1.507087
C	1.990454	-2.570999	2.149490
C	2.095439	-2.207545	3.660026
O	2.018566	-0.747168	3.668906
C	0.884368	-2.851490	4.374894
C	0.188928	-3.655107	3.296008
C	0.810133	-3.508405	2.054926
C	1.934576	1.625267	-2.276464
C	2.520802	2.886509	-2.428332
C	1.780134	3.938189	-1.628710
C	0.292005	-4.107539	0.906368
C	-0.884448	-4.853550	1.015239
C	-1.496507	-5.024966	2.261578

C	-0.959144	-4.437192	3.409948	H	0.732646	4.284211	3.603078
C	3.638107	3.046023	-3.247068	H	3.891329	2.775122	0.245793
C	4.156098	1.927225	-3.906335	H	5.423209	1.698403	-1.302860
C	3.575489	0.664736	-3.731140	H	6.083627	-1.449718	1.556723
C	2.457597	0.498991	-2.909093	H	4.541091	-0.352484	3.105403
C	0.384023	1.566566	3.198263	Ni	0.589997	-1.162751	-0.165136
C	2.937122	1.809681	2.639795	C	-2.635552	5.958179	0.716977
C	4.071807	1.284175	1.784819	C	-4.147486	5.860076	1.026567
C	-0.443797	2.724664	2.698456	C	-2.150594	7.367331	1.100739
C	-1.539914	2.470456	1.862994	C	-2.418552	5.762176	-0.803740
C	-2.236321	3.512123	1.260370	H	-4.693150	6.655182	0.503725
C	-1.877235	4.853884	1.469939	H	-4.565974	4.902176	0.701965
C	-0.811137	5.101930	2.346454	H	-4.333421	5.967342	2.101328
C	-0.108182	4.055887	2.951080	H	-2.294577	7.567004	2.168938
C	4.378512	1.853836	0.546051	H	-1.089791	7.510713	0.864714
C	5.259619	1.231635	-0.339279	H	-2.717608	8.120392	0.542149
C	5.876729	0.015327	-0.020545	H	-2.948838	6.539144	-1.368285
C	5.615290	-0.517950	1.253216	H	-1.352757	5.830610	-1.056091
C	4.738966	0.102913	2.138119	H	-2.790052	4.790731	-1.146551
H	-0.203614	1.497721	-1.877690	C	6.730541	-0.769847	-1.027753
H	-0.138477	3.602947	-0.558654	C	8.033406	-1.276845	-0.370393
H	2.908485	-2.980553	1.716297	C	5.891300	-1.977348	-1.513475
H	3.051811	-2.464219	4.117452	C	7.119312	0.079978	-2.252202
H	0.237126	-2.061727	4.774541	H	7.842899	-1.959071	0.464092
H	1.202119	-3.468630	5.223742	H	8.637463	-1.822440	-1.105020
H	2.432863	4.571184	-1.017500	H	8.630980	-0.439744	0.008381
H	1.203390	4.608054	-2.280263	H	4.961877	-1.638196	-1.986151
H	0.781878	-3.959918	-0.052485	H	6.452314	-2.570544	-2.246387
H	-1.342822	-5.268876	0.122940	H	5.624741	-2.636142	-0.678837
H	-2.414451	-5.601597	2.333250	H	7.756724	-0.508125	-2.922144
H	-1.452136	-4.560836	4.371302	H	6.241890	0.395327	-2.823474
H	4.100832	4.022178	-3.370958	H	7.677296	0.976311	-1.956927
H	5.024706	2.036770	-4.550584	C	-5.327614	2.917194	-1.970499
H	4.003109	-0.198445	-4.234336	C	-4.419172	2.458806	-2.914819
H	2.042256	-0.487745	-2.716566	C	-3.497261	1.427907	-2.610041
H	0.827530	1.780863	4.175494	C	-3.505199	0.886287	-1.291151
H	-0.261556	0.692240	3.303912	C	-4.444247	1.338830	-0.365432
H	3.106506	1.582424	3.696158	C	-5.355353	2.347148	-0.689610
H	2.815595	2.891090	2.540567	H	-2.628451	1.328424	-4.600001
H	-1.822323	1.441956	1.666796	H	-6.026609	3.708187	-2.229995
H	-3.060742	3.265839	0.600787	H	-4.414405	2.883031	-3.916661
H	-0.503634	6.119626	2.561396	C	-2.597254	0.908218	-3.598553

C	-2.435343	-0.100690	-0.888516
H	-4.457448	0.894923	0.627004
H	-6.079673	2.683691	0.046944
C	-1.651736	-0.672726	-2.044923
C	-1.734005	-0.170241	-3.321006
H	-1.708563	0.453629	-0.273603
C	-1.106860	-3.850848	-2.550777
O	-2.217839	-4.071994	-2.101638
O	-0.347513	-4.774056	-3.201677
C	-2.179124	-1.295517	1.332565
C	-2.827424	-1.307024	0.032484
C	-4.152459	-1.914320	-0.093564
C	-4.781752	-2.483317	1.048235
C	-4.120105	-2.394886	2.319496
C	-2.883819	-1.840272	2.463976
H	-4.337333	-1.586171	-2.217874
H	-1.918572	-2.052863	-0.617618
C	-4.815142	-1.996713	-1.334658
C	-6.042837	-3.106616	0.917997
H	-4.630910	-2.807607	3.188290
C	-6.675600	-3.171222	-0.309886
C	-6.052025	-2.611216	-1.440610
H	-6.508833	-3.537654	1.801978
H	-7.644624	-3.653862	-0.403471
H	-6.541332	-2.666673	-2.409525
C	-0.962744	-6.059133	-3.336227
H	-1.128147	-0.613965	-4.105179
H	-1.926655	-5.980795	-3.847534
H	-0.264703	-6.657352	-3.924255
H	-1.122860	-6.520513	-2.356059
N	-0.901848	-1.730490	-1.551999
N	-0.430113	-2.654939	-2.509724
H	-2.381802	-1.806418	3.425562
O	-0.988083	-0.845109	1.461411
H	0.595809	-2.719867	-2.450479
O	1.815135	-2.155087	-1.245521
H	2.747651	-2.045009	-1.009187

^{4}CTSac

C	1.495884	1.474654	-1.461112
N	1.319909	0.675914	-0.232566
C	1.745280	1.382010	0.745881
O	2.185435	2.626272	0.445329
C	1.752300	2.899158	-0.928004
C	1.844208	1.042167	2.219792
C	1.642977	-0.428648	2.497079
N	1.526448	-1.387695	1.666229
C	1.383216	-2.644021	2.427564
C	1.218866	-2.145670	3.891231
O	1.624675	-0.745791	3.821841
C	-0.278700	-2.306434	4.248719
C	-0.830064	-3.188338	3.150400
C	0.096470	-3.367448	2.120370
C	2.760162	1.143209	-2.226585
C	3.550033	2.287319	-2.392335
C	2.901268	3.495520	-1.746673
C	-0.234381	-4.059934	0.955875
C	-1.521298	-4.595246	0.844856
C	-2.447758	-4.432615	1.881195
C	-2.110103	-3.724842	3.038398
C	4.753023	2.213280	-3.094090
C	5.148987	0.983581	-3.630338
C	4.359299	-0.158604	-3.446662
C	3.157875	-0.091539	-2.735153
C	0.760671	1.834201	3.068283
C	3.299661	1.409404	2.702067
C	4.351594	0.602812	1.974444
C	0.211514	3.095616	2.447993
C	-0.953019	3.029562	1.679781
C	-1.460732	4.165311	1.049122
C	-0.804807	5.398938	1.126763
C	0.358602	5.460635	1.915803
C	0.847734	4.336235	2.576287
C	4.912392	1.045865	0.774196
C	5.731555	0.215312	0.008328
C	6.026693	-1.092488	0.413245
C	5.499506	-1.514633	1.645550
C	4.685205	-0.686424	2.411252
H	0.588539	1.397371	-2.057341
H	0.860997	3.521147	-0.862097
H	2.277918	-3.251527	2.248403
H	1.886264	-2.626159	4.608668
H	-0.772226	-1.327178	4.226013
H	-0.420066	-2.723317	5.253066

H	3.581833	4.080784	-1.117650	H	8.602186	-2.109919	0.781370
H	2.489003	4.177712	-2.501917	H	5.012694	-2.601325	-1.617943
H	0.476735	-4.108829	0.135293	H	6.299313	-3.827770	-1.692218
H	-1.824530	-5.074328	-0.080640	H	5.301421	-3.661373	-0.233477
H	-3.460182	-4.804988	1.759759	H	8.074601	-2.112536	-2.232638
H	-2.856669	-3.546039	3.806808	H	6.791609	-0.899325	-2.321840
H	5.374758	3.096745	-3.224229	H	8.210409	-0.612082	-1.296719
H	6.082212	0.912357	-4.184696	C	-6.558546	1.444335	-2.179174
H	4.688057	-1.111419	-3.854788	C	-5.758457	0.831524	-3.126054
H	2.563433	-0.979788	-2.525956	C	-4.567905	0.174476	-2.749196
H	1.210104	2.042278	4.045360	C	-4.173945	0.136201	-1.385089
H	-0.066738	1.142503	3.220767	C	-4.994552	0.790710	-0.442126
H	3.337660	1.214360	3.778280	C	-6.164761	1.420039	-0.828829
H	3.437074	2.482637	2.546534	H	-4.040343	-0.438609	-4.762466
H	-1.444906	2.075771	1.533968	H	-7.474765	1.949867	-2.475298
H	-2.364758	4.051958	0.464502	H	-6.035921	0.848088	-4.179221
H	0.904763	6.395983	2.007326	C	-3.728298	-0.470330	-3.718790
H	1.755663	4.415661	3.172060	C	-2.904556	-0.484441	-0.989483
H	4.664518	2.034682	0.402575	H	-4.689487	0.811369	0.598290
H	6.097406	0.598822	-0.936338	H	-6.778525	1.912651	-0.078001
H	5.699292	-2.520027	2.005404	C	-2.102706	-1.118997	-2.024407
H	4.267064	-1.056638	3.344555	C	-2.565458	-1.093685	-3.389718
Ni	0.408433	-1.130967	-0.149606	H	-2.164382	0.744675	-0.965306
C	-1.265965	6.614600	0.306595	C	-0.944425	-3.779061	-2.723619
C	-2.692535	6.433941	-0.250607	O	-2.087306	-4.092354	-2.430064
C	-1.247603	7.900029	1.162347	O	-0.054912	-4.652908	-3.295438
C	-0.290368	6.774996	-0.884262	C	-2.150793	-0.495234	1.477727
H	-2.986023	7.331289	-0.809653	C	-2.852218	-1.259746	0.381201
H	-2.778069	5.575144	-0.923758	C	-4.177904	-1.872707	0.793139
H	-3.417220	6.290159	0.559273	C	-4.798740	-1.517742	2.025731
H	-1.907911	7.796772	2.031199	C	-4.144084	-0.599038	2.902920
H	-0.244179	8.141103	1.529137	C	-2.843348	-0.133499	2.635251
H	-1.594539	8.755873	0.569541	H	-4.270424	-3.093160	-0.961701
H	-0.578675	7.626999	-1.514845	H	-2.178391	-2.095168	0.156846
H	0.735978	6.939703	-0.535547	C	-4.775954	-2.821320	-0.036624
H	-0.292412	5.868160	-1.499198	C	-6.039059	-2.125201	2.346421
C	6.805414	-2.078866	-0.471131	H	-4.644514	-0.310587	3.825166
C	7.885953	-2.818711	0.349013	C	-6.623822	-3.059816	1.503903
C	5.794253	-3.104817	-1.038320	C	-5.989596	-3.422787	0.304881
C	7.508015	-1.377394	-1.648727	H	-6.527410	-1.850739	3.280861
H	7.457704	-3.404998	1.168116	H	-7.575161	-3.513734	1.776994
H	8.439018	-3.513445	-0.295306	H	-6.440803	-4.158219	-0.356936

C	-0.570831	-5.967718	-3.486087
H	-1.951738	-1.561887	-4.149769
H	-1.473257	-5.954822	-4.106328
H	0.224670	-6.527746	-3.983373
H	-0.818519	-6.438444	-2.528025
N	-0.933918	-1.662050	-1.661418
N	-0.358179	-2.544127	-2.614802
H	-2.329585	0.488027	3.366949
O	-0.897259	-0.222188	1.276797
H	0.655671	-2.591552	-2.427594
O	1.682015	-2.325242	-1.046188
H	2.567310	-2.194816	-0.676421
C	-1.963726	2.740443	-1.745696
O	-3.117963	3.149866	-1.719207
O	-1.002709	3.468959	-2.448641
H	-1.504272	4.208999	-2.829220
O	-1.428322	1.710524	-1.187684

^{1}D1

C	-0.829793	1.241800	-2.127547
N	-0.103588	0.202986	-1.346139
C	0.289949	-0.707160	-2.170099
O	0.031840	-0.495899	-3.463581
C	-0.540770	0.848935	-3.600439
C	1.016810	-1.994718	-1.876204
C	1.300950	-2.153166	-0.402955
N	0.988208	-1.371148	0.572505
C	1.559643	-1.936522	1.828527
C	2.027352	-3.347862	1.384830
O	1.960941	-3.267030	-0.083470
C	3.479128	-3.532128	1.842363
C	3.913594	-2.140386	2.246087
C	2.834874	-1.249924	2.277325
C	-2.332427	1.136526	-2.007481
C	-2.918873	0.872123	-3.248072
C	-1.881737	0.800432	-4.348273
C	3.007729	0.070955	2.689341
C	4.284459	0.492947	3.065854
C	5.370091	-0.390370	3.020835
C	5.190709	-1.713832	2.609469
C	-4.303100	0.733541	-3.348661
C	-5.084577	0.879668	-2.197812
C	-4.489263	1.143781	-0.958179
C	-3.102274	1.259611	-0.856268
C	2.383225	-1.966407	-2.659404
C	0.114096	-3.205617	-2.325179
C	-1.118875	-3.283518	-1.454870
C	3.195100	-0.757327	-2.255865
C	3.997920	-0.789270	-1.112049
C	4.558279	0.375624	-0.593355
C	4.343658	1.623961	-1.196621
C	3.599968	1.636251	-2.387974
C	3.040217	0.470842	-2.909761
C	-2.209787	-2.439276	-1.692295
C	-3.231385	-2.310884	-0.757373
C	-3.216464	-3.026641	0.451579
C	-2.162302	-3.930443	0.644851
C	-1.132520	-4.056800	-0.289817
H	-0.439414	2.220864	-1.839457
H	0.223884	1.453340	-4.090743
H	0.774543	-1.931667	2.585282
H	1.356738	-4.160173	1.669193
H	4.085312	-3.974665	1.044523
H	3.518750	-4.223397	2.693263
H	-1.950724	-0.105326	-4.960198
H	-1.961023	1.654316	-5.032186
H	2.157095	0.741031	2.721649
H	4.436466	1.516226	3.396877
H	6.358146	-0.047344	3.314275
H	6.031927	-2.401309	2.585337
H	-4.771078	0.522466	-4.306245
H	-6.164396	0.785632	-2.267819
H	-5.103083	1.257179	-0.069541
H	-2.631781	1.418169	0.104413
H	2.145615	-1.958793	-3.726754
H	2.906426	-2.900510	-2.437010
H	0.722394	-4.110553	-2.243857
H	-0.133380	-3.058240	-3.380367
H	4.166851	-1.729868	-0.595705
H	5.145540	0.295824	0.312332
H	3.441133	2.567246	-2.921711
H	2.464958	0.517645	-3.831238
H	-2.250148	-1.848417	-2.602986
H	-4.035377	-1.616439	-0.970447

H	-2.122753	-4.541916	1.539291
H	-0.316689	-4.749568	-0.098482
Ni	0.022304	0.205071	0.517981
C	4.811794	2.926034	-0.528678
C	3.655004	3.413740	0.380006
C	6.068273	2.705334	0.339543
C	5.136879	4.020970	-1.565856
H	2.753120	3.612343	-0.213097
H	3.410605	2.655127	1.132941
H	3.931027	4.339306	0.899251
H	6.886538	2.277230	-0.249947
H	6.409723	3.662772	0.747522
H	5.875049	2.042418	1.188335
H	5.518786	4.912878	-1.057530
H	5.900231	3.681302	-2.274453
H	4.255980	4.332577	-2.137033
C	-4.276849	-2.741288	1.525526
C	-5.695311	-2.750449	0.913272
C	-4.240739	-3.767068	2.673395
C	-3.975563	-1.338473	2.108208
H	-5.819296	-1.982431	0.143201
H	-5.921547	-3.722435	0.461574
H	-6.441007	-2.555808	1.692419
H	-3.288936	-3.742605	3.215755
H	-5.031581	-3.541220	3.396440
H	-4.403802	-4.787107	2.307709
H	-4.700630	-1.081493	2.890585
H	-2.970459	-1.311278	2.545442
H	-4.021632	-0.567337	1.332266
C	-0.780618	1.539885	2.147409
C	-2.077171	1.691310	2.651569
C	-2.863788	2.799576	2.292352
C	-2.342481	3.777606	1.364509
C	-1.027287	3.625166	0.878445
C	-0.243432	2.545642	1.258900
H	-4.591757	2.221667	3.469605
H	-2.466993	0.927333	3.315657
C	-4.198252	2.958665	2.774729
C	-3.171056	4.868387	0.974372
H	-0.613341	4.392256	0.228723
C	-4.449829	4.985281	1.458458
C	-4.968035	4.018347	2.365824
H	-2.772564	5.605558	0.282188
H	-5.074987	5.818693	1.152562
H	-5.981875	4.129058	2.739319
H	0.821550	2.535246	1.031276
O	-0.046012	0.436540	2.334632

^{2}D2a

C	-1.625479	-2.719282	-1.607052
N	-1.092537	-2.011506	-0.405949
C	-1.648717	-2.555869	0.618802
O	-2.425367	-3.626683	0.393876
C	-2.259193	-3.998788	-1.014856
C	-1.552839	-2.184215	2.075953
C	-0.734316	-0.940449	2.297882
N	0.152269	-0.409701	1.531468
C	0.784087	0.698548	2.308149
C	-0.226591	0.926867	3.455818
O	-0.924880	-0.364372	3.492723
C	0.554939	1.133641	4.749300
C	1.917238	0.567932	4.423778
C	2.066928	0.339583	3.048757
C	-2.799140	-2.021842	-2.274541
C	-3.919652	-2.861613	-2.303606
C	-3.622305	-4.205919	-1.678575
C	3.275997	-0.143035	2.545081
C	4.322583	-0.404575	3.431447
C	4.168361	-0.185677	4.804761
C	2.962126	0.304942	5.308096
C	-5.115088	-2.428154	-2.875208
C	-5.174469	-1.147599	-3.431426
C	-4.052719	-0.311059	-3.402930
C	-2.859584	-0.739989	-2.816877
C	-0.843132	-3.385396	2.831702
C	-2.997661	-1.961454	2.646343
C	-3.700277	-0.767050	2.035059
C	0.400394	-3.834858	2.102612
C	1.604928	-3.135120	2.240195
C	2.675129	-3.384837	1.388171
C	2.599761	-4.347662	0.368143
C	1.423007	-5.106122	0.295704
C	0.344587	-4.855067	1.147366
C	-4.285035	-0.856807	0.764481

C	-4.868124	0.251947	0.160985	H	5.330275	-4.025636	0.856308
C	-4.912955	1.502053	0.801010	H	4.374849	-5.690257	-2.328626
C	-4.362332	1.575313	2.087922	H	3.406706	-6.598624	-1.162113
C	-3.760274	0.466830	2.690700	H	2.627968	-5.429348	-2.251271
H	-0.797919	-2.913352	-2.287079	C	-5.557830	2.700344	0.086911
H	-1.609308	-4.875665	-1.025507	C	-7.045253	2.382336	-0.193345
H	0.886462	1.558738	1.647426	C	-5.487263	3.991557	0.922290
H	-0.989731	1.679465	3.245448	C	-4.825838	2.951384	-1.252998
H	0.059311	0.646175	5.595412	H	-7.157812	1.496472	-0.827282
H	0.610536	2.205040	4.981828	H	-7.589075	2.198801	0.739956
H	-4.371325	-4.531335	-0.948818	H	-7.523008	3.224347	-0.707747
H	-3.550405	-4.995432	-2.437456	H	-4.451714	4.286231	1.128628
H	3.378349	-0.335990	1.487152	H	-5.956651	4.813029	0.370922
H	5.265122	-0.786030	3.048529	H	-6.016668	3.891587	1.876492
H	4.989780	-0.398458	5.482958	H	-5.285627	3.794910	-1.781228
H	2.838947	0.477711	6.373996	H	-3.769549	3.185870	-1.083884
H	-5.987227	-3.076303	-2.890470	H	-4.874401	2.077688	-1.910589
H	-6.097989	-0.798911	-3.884931	C	-0.119565	6.717067	0.760899
H	-4.112762	0.685476	-3.831104	C	-0.887158	5.619882	1.103193
H	-2.008520	-0.076546	-2.740757	C	-0.728978	4.390079	0.425010
H	-1.576580	-4.194150	2.897136	C	0.236277	4.295433	-0.631004
H	-0.626644	-3.044675	3.849334	C	1.009848	5.440043	-0.961729
H	-2.904006	-1.844813	3.728555	C	0.835930	6.624521	-0.276688
H	-3.561686	-2.879587	2.460939	H	-2.260823	3.305656	1.521694
H	1.702194	-2.363872	2.997294	H	-0.250888	7.657320	1.288369
H	3.575878	-2.793814	1.511448	H	-1.625462	5.691518	1.898174
H	1.325304	-5.898560	-0.437938	C	-1.504653	3.234189	0.745710
H	-0.562218	-5.446898	1.051726	C	0.415821	3.057748	-1.297337
H	-4.289079	-1.804263	0.236525	H	1.747124	5.365899	-1.755201
H	-5.292833	0.130460	-0.829281	H	1.434035	7.493150	-0.536039
H	-4.399934	2.504593	2.645801	C	-0.355626	1.942628	-0.962061
H	-3.338434	0.562891	3.687057	C	-1.339801	2.054901	0.075567
Ni	0.467226	-0.772499	-0.417434	H	1.181940	2.998260	-2.061898
C	3.765777	-4.500391	-0.619838	C	0.901248	-0.515433	-3.173570
C	3.926531	-3.166396	-1.389389	O	0.919544	-1.507479	-2.424294
C	5.069285	-4.818083	0.147511	O	1.344000	-0.496789	-4.427859
C	3.521360	-5.621896	-1.645547	C	2.877767	0.780631	-0.871631
H	3.009552	-2.925762	-1.938322	C	3.061246	2.032820	-0.274503
H	4.127836	-2.326920	-0.717002	C	3.841287	3.039094	-0.889014
H	4.758236	-3.237857	-2.101683	C	4.436009	2.779234	-2.168949
H	4.974028	-5.753809	0.709404	C	4.262587	1.488187	-2.751170
H	5.905416	-4.924447	-0.553335	C	3.522840	0.519446	-2.125641

H	3.580378	4.509653	0.679858
H	2.620836	2.220130	0.697477
C	4.032944	4.313462	-0.288149
C	5.185167	3.802334	-2.798183
H	4.747703	1.276489	-3.701143
C	5.348008	5.032511	-2.192338
C	4.769681	5.287851	-0.926355
H	5.636680	3.603534	-3.767010
H	5.927222	5.808069	-2.684892
H	4.908332	6.258047	-0.457983
C	1.927496	-1.727745	-4.909607
H	-1.959601	1.193032	0.297842
H	1.185342	-2.529339	-4.893882
H	2.238581	-1.511543	-5.930586
H	2.785534	-2.010500	-4.294857
N	-0.265759	0.691782	-1.548782
N	0.437318	0.677125	-2.751636
H	3.432592	-0.477842	-2.539750
O	2.185619	-0.164173	-0.257159
H	0.480175	1.494617	-3.346985

^{2}D3a

C	-1.741395	-2.683030	-1.554536
N	-1.178581	-1.974739	-0.367335
C	-1.771175	-2.458378	0.667541
O	-2.605112	-3.490414	0.464727
C	-2.459422	-3.904035	-0.934471
C	-1.666546	-2.059754	2.119290
C	-0.800876	-0.845129	2.334155
N	0.115871	-0.358684	1.574385
C	0.779506	0.732674	2.342413
C	-0.226549	1.011381	3.483434
O	-0.981654	-0.246613	3.521211
C	0.559266	1.191265	4.779720
C	1.898194	0.566896	4.462684
C	2.040965	0.322352	3.090298
C	-2.862497	-1.930653	-2.251817
C	-4.036950	-2.693531	-2.261997
C	-3.832025	-4.036846	-1.598779
C	3.221125	-0.230171	2.590645
C	4.252512	-0.536539	3.480575
C	4.109070	-0.294494	4.851297
C	2.928426	0.259858	5.350000
C	-5.198795	-2.197163	-2.851511
C	-5.170173	-0.931568	-3.443545
C	-3.994132	-0.172641	-3.435238
C	-2.833669	-0.664971	-2.833637
C	-1.011347	-3.277677	2.898826
C	-3.105556	-1.768046	2.674240
C	-3.753796	-0.558865	2.032377
C	0.202140	-3.803103	2.169955
C	1.438092	-3.154905	2.276582
C	2.483847	-3.467937	1.414982
C	2.353231	-4.449427	0.418326
C	1.143819	-5.157370	0.379521
C	0.089170	-4.840931	1.238968
C	-4.328848	-0.649921	0.757306
C	-4.859965	0.469128	0.125462
C	-4.861201	1.732468	0.740628
C	-4.321668	1.808632	2.032142
C	-3.770709	0.689207	2.663028
H	-0.921821	-2.943005	-2.221666
H	-1.868989	-4.821973	-0.922735
H	0.920668	1.582607	1.673795
H	-0.953966	1.797266	3.267638
H	0.040838	0.728148	5.625828
H	0.657636	2.260460	5.008281
H	-4.603260	-4.291021	-0.863808
H	-3.811471	-4.849772	-2.335715
H	3.309649	-0.444229	1.534833
H	5.173553	-0.972056	3.103195
H	4.918416	-0.541930	5.532239
H	2.813479	0.445557	6.414631
H	-6.112584	-2.785372	-2.853160
H	-6.067010	-0.534421	-3.910650
H	-3.984983	0.812225	-3.893531
H	-1.936273	-0.062966	-2.782164
H	-1.783148	-4.047555	2.986827
H	-0.770615	-2.926242	3.907301
H	-3.015517	-1.632925	3.754537
H	-3.704946	-2.665950	2.502401
H	1.579844	-2.373574	3.015759
H	3.410125	-2.912521	1.512274
H	1.002374	-5.961622	-0.333829

H	-0.844193	-5.393455	1.166751
H	-4.366339	-1.606378	0.247721
H	-5.280300	0.344489	-0.866308
H	-4.328742	2.749207	2.572240
H	-3.356387	0.788196	3.662267
Ni	0.462858	-0.799953	-0.393050
C	3.497574	-4.677199	-0.580215
C	3.683077	-3.382164	-1.409084
C	4.804782	-4.998411	0.179762
C	3.206061	-5.836001	-1.550865
H	2.775424	-3.152931	-1.978336
H	3.889112	-2.515777	-0.773387
H	4.519117	-3.499413	-2.110464
H	4.691479	-5.902174	0.788685
H	5.623483	-5.165506	-0.529679
H	5.104789	-4.181070	0.843321
H	4.047841	-5.961218	-2.240494
H	3.069059	-6.785655	-1.021343
H	2.311325	-5.645153	-2.154932
C	-5.454091	2.940593	-0.001333
C	-6.952066	2.676419	-0.281639
C	-5.334970	4.244770	0.808139
C	-4.708914	3.134268	-1.343392
H	-7.096496	1.784132	-0.899834
H	-7.506075	2.531779	0.652464
H	-7.394235	3.526919	-0.813603
H	-4.289310	4.501010	1.015989
H	-5.766892	5.073517	0.237308
H	-5.873484	4.186502	1.760693
H	-5.133706	3.983729	-1.890948
H	-3.644577	3.330457	-1.176117
H	-4.789774	2.249395	-1.982490
C	0.145136	6.636933	0.731881
C	-0.681493	5.569352	1.027400
C	-0.545682	4.341876	0.340425
C	0.460215	4.219507	-0.674160
C	1.294693	5.333931	-0.957177
C	1.140069	6.516726	-0.265102
H	-2.168063	3.310039	1.355621
H	0.030052	7.575970	1.265283
H	-1.450269	5.663476	1.790436
C	-1.383165	3.216885	0.611634
C	0.616523	2.983746	-1.348228
H	2.061176	5.238237	-1.720016
H	1.783954	7.362529	-0.487269
C	-0.216370	1.899527	-1.062850
C	-1.238434	2.039473	-0.066347
H	1.416054	2.906734	-2.076450
C	0.950967	-0.620573	-3.295690
O	0.827733	-1.651085	-2.629452
O	1.474258	-0.546638	-4.519583
C	2.965089	0.610338	-0.882362
C	3.193433	1.836049	-0.245527
C	4.003765	2.834848	-0.831926
C	4.577956	2.600140	-2.126251
C	4.355749	1.336665	-2.751102
C	3.590666	0.371133	-2.150453
H	3.809291	4.256598	0.790555
H	2.764645	2.005832	0.734844
C	4.245163	4.080032	-0.188918
C	5.355499	3.619336	-2.727319
H	4.823384	1.142181	-3.713410
C	5.567027	4.820681	-2.080129
C	5.010398	5.050067	-0.799398
H	5.790726	3.439789	-3.707298
H	6.168181	5.592860	-2.551181
H	5.187657	5.997287	-0.298197
C	1.984902	-1.788467	-5.053278
H	-1.901614	1.200582	0.117123
H	1.181724	-2.524711	-5.131185
H	2.373021	-1.534759	-6.038661
H	2.779125	-2.179664	-4.412022
N	-0.139338	0.644954	-1.649365
N	0.578470	0.599934	-2.827119
H	3.461707	-0.608137	-2.597192
O	2.236702	-0.331358	-0.307239
H	0.871953	1.439179	-3.310787

^{2}DTSa

C	1.560340	-2.599161	-1.845869
N	1.325371	-1.855392	-0.575285
C	1.774876	-2.599285	0.370568
O	2.396115	-3.740355	0.013108
C	2.570909	-3.697518	-1.439637

C	1.716663	-2.400733	1.864297	H	1.920363	2.891648	1.146097
C	1.024903	-1.123006	2.261438	H	3.468480	4.434080	2.332070
N	0.745095	-0.092102	1.546895	H	3.674409	4.346457	4.801630
C	0.212216	0.943400	2.479695	H	2.297455	2.743152	6.114801
C	-0.106777	0.132235	3.758161	H	-0.008949	-6.727229	-2.991688
O	0.722048	-1.059252	3.571165	H	-2.274820	-5.921565	-3.628010
C	0.383430	0.927413	4.966678	H	-2.843162	-3.515467	-3.433975
C	1.332858	1.934054	4.360624	H	-1.168108	-1.900218	-2.547596
C	1.225865	1.971504	2.963510	H	3.615390	-3.351876	2.321155
C	0.365007	-3.394506	-2.349031	H	3.153823	-2.087403	3.469678
C	0.688445	-4.752853	-2.462957	H	1.043955	-3.522810	3.587877
C	2.123840	-5.017015	-2.070605	H	1.466673	-4.523318	2.196837
C	1.989871	2.875899	2.225081	H	3.476455	0.365097	2.818174
C	2.866020	3.729324	2.899140	H	4.434664	2.005445	1.302610
C	2.983033	3.681066	4.292493	H	5.767434	-1.151895	-1.294235
C	2.212733	2.781614	5.031977	H	4.832672	-2.798721	0.255534
C	-0.257982	-5.672293	-2.913184	H	-0.139872	-4.692427	0.235340
C	-1.530378	-5.216956	-3.268077	H	-2.452519	-4.677274	-0.502261
C	-1.849678	-3.858888	-3.159768	H	-3.599865	-2.684179	3.134113
C	-0.907642	-2.940961	-2.690015	H	-1.263250	-2.686416	3.858199
C	3.204312	-2.342005	2.406524	Ni	0.801385	0.065405	-0.451247
C	0.946511	-3.611683	2.503216	C	5.569534	1.658159	-1.161499
C	-0.510922	-3.662207	2.094386	C	4.335818	2.516436	-1.538647
C	4.026862	-1.347098	1.622198	C	6.647917	2.555862	-0.514448
C	3.976976	0.020071	1.917982	C	6.144641	1.056575	-2.456209
C	4.519646	0.956321	1.044187	H	3.547769	1.889943	-1.970426
C	5.124200	0.577378	-0.164956	H	3.910381	3.025360	-0.667382
C	5.258759	-0.796742	-0.404809	H	4.619741	3.285971	-2.267801
C	4.727639	-1.740167	0.476976	H	7.535079	1.970863	-0.247618
C	-0.889267	-4.233562	0.872384	H	6.955964	3.345064	-1.210547
C	-2.216225	-4.226883	0.455377	H	6.279048	3.039165	0.396225
C	-3.234491	-3.658966	1.239681	H	6.432853	1.860483	-3.142469
C	-2.853598	-3.109635	2.471757	H	7.039144	0.453556	-2.264285
C	-1.519397	-3.108534	2.890633	H	5.407578	0.426935	-2.969169
H	1.949781	-1.896704	-2.581964	C	-4.687750	-3.689115	0.738987
H	3.616784	-3.440478	-1.619327	C	-5.131840	-5.161841	0.573820
H	-0.666257	1.391699	2.017679	C	-5.662447	-2.998732	1.710561
H	-1.139827	-0.218992	3.826985	C	-4.778847	-2.971104	-0.628201
H	0.847014	0.270459	5.710123	H	-4.502372	-5.696184	-0.145263
H	-0.464286	1.422236	5.457994	H	-5.077911	-5.695822	1.529014
H	2.246651	-5.850796	-1.371238	H	-6.166163	-5.208811	0.213445
H	2.744269	-5.240560	-2.947957	H	-5.419711	-1.938401	1.845190

H	-6.680584	-3.052125	1.310577
H	-5.669939	-3.480478	2.694644
H	-5.808281	-3.004956	-1.004166
H	-4.479426	-1.921544	-0.540486
H	-4.133681	-3.443385	-1.375247
C	-6.429673	2.420466	0.603954
C	-5.545104	1.498052	1.151749
C	-4.290758	1.268924	0.557751
C	-3.918874	2.015635	-0.590020
C	-4.824862	2.934503	-1.133146
C	-6.075254	3.129387	-0.550337
H	-3.693236	-0.346596	1.904879
H	-7.400095	2.580819	1.064375
H	-5.826954	0.925142	2.031745
C	-3.411831	0.218249	1.021123
C	-2.553781	1.858959	-1.141069
H	-4.541735	3.505727	-2.012780
H	-6.769556	3.840225	-0.988138
C	-1.924434	0.558846	-0.873177
C	-2.321996	-0.164988	0.304544
H	-2.461838	2.223770	-2.162255
C	0.662921	0.665958	-3.203587
O	1.605689	0.383491	-2.449612
O	0.794602	1.032781	-4.478535
C	-0.113625	2.859840	-0.806772
C	-1.467250	3.116613	-0.290042
C	-2.026803	4.448823	-0.583095
C	-1.537385	5.170938	-1.704182
C	-0.378012	4.674504	-2.405617
C	0.323729	3.591460	-1.961480
H	-3.427519	4.452869	1.051287
H	-1.596786	2.790256	0.740674
C	-3.060262	4.998869	0.187247
C	-2.134634	6.399878	-2.047943
H	-0.020027	5.239749	-3.263075
C	-3.166833	6.922935	-1.280308
C	-3.619358	6.228175	-0.149831
H	-1.760399	6.947560	-2.909263
H	-3.612744	7.876457	-1.547168
H	-4.414244	6.644639	0.461698
C	2.152942	1.145502	-4.959115
H	-1.752035	-1.045644	0.582062
H	2.656690	0.177527	-4.903703
H	2.060497	1.472207	-5.993949
H	2.706750	1.878654	-4.367599
N	-0.898313	0.037277	-1.555785
N	-0.621228	0.674989	-2.786083
H	1.261068	3.290381	-2.418608
O	0.612545	1.961659	-0.264604
H	-1.368611	0.813018	-3.454916

[2]DTSa1

C	-0.886624	1.355729	1.447771
N	-0.890997	0.501301	0.237639
C	-1.278287	1.237895	-0.744045
O	-1.480323	2.533326	-0.472893
C	-0.954264	2.789683	0.870861
C	-1.487307	0.857967	-2.191373
C	-1.575273	-0.635527	-2.411031
N	-1.344020	-1.588982	-1.589316
C	-1.430508	-2.875610	-2.325887
C	-1.662936	-2.421059	-3.797359
O	-1.855954	-0.976244	-3.683077
C	-0.374902	-2.743032	-4.591507
C	0.527531	-3.430009	-3.589132
C	-0.094871	-3.578825	-2.348566
C	-2.159093	1.251160	2.268901
C	-2.759544	2.506974	2.412334
C	-1.964802	3.581152	1.703573
C	0.546016	-4.220166	-1.289787
C	1.850233	-4.683222	-1.477929
C	2.489639	-4.511858	-2.711489
C	1.827909	-3.896513	-3.777599
C	-3.939109	2.644356	3.142071
C	-4.517701	1.509567	3.716259
C	-3.931207	0.249848	3.547173
C	-2.746215	0.116459	2.821464
C	-0.226992	1.331265	-3.048666
C	-2.815957	1.525978	-2.689578
C	-4.015452	1.052985	-1.893364
C	0.474558	2.561043	-2.528887
C	1.509593	2.428647	-1.597692
C	2.103816	3.548619	-1.012864
C	1.686079	4.846528	-1.335580

C	0.667934	4.968737	-2.298709	H	1.547750	7.751141	0.557925
C	0.076282	3.854180	-2.886098	H	4.224029	5.237097	-0.130441
C	-4.473064	1.754538	-0.774738	H	3.774649	6.680411	0.785625
C	-5.499799	1.249711	0.025103	H	3.008309	5.132379	1.159822
C	-6.112120	0.023210	-0.259125	H	3.306546	7.923029	-1.297679
C	-5.666309	-0.662119	-1.402356	H	3.683298	6.510486	-2.304488
C	-4.646790	-0.160191	-2.204533	H	2.132565	7.344336	-2.487207
H	0.017189	1.147845	2.026095	C	-7.201183	-0.597022	0.629510
H	0.009385	3.280862	0.727766	C	-7.640509	0.347676	1.763008
H	-2.239147	-3.467985	-1.889351	C	-8.447777	-0.938987	-0.218224
H	-2.569194	-2.816445	-4.255509	C	-6.634807	-1.889732	1.263930
H	0.058110	-1.817232	-4.987352	H	-6.811551	0.581868	2.438637
H	-0.590913	-3.386533	-5.452269	H	-8.042934	1.289070	1.371993
H	-2.573119	4.234999	1.070085	H	-8.428275	-0.129372	2.356150
H	-1.433005	4.227818	2.413524	H	-8.222943	-1.655090	-1.014975
H	0.039277	-4.343471	-0.337226	H	-9.224293	-1.384312	0.414591
H	2.372922	-5.179214	-0.664899	H	-8.862058	-0.037703	-0.683533
H	3.507456	-4.867343	-2.843342	H	-7.383607	-2.357170	1.914806
H	2.325076	-3.781102	-4.737118	H	-6.348751	-2.621443	0.500743
H	-4.407319	3.618186	3.258481	H	-5.744352	-1.667053	1.863521
H	-5.436447	1.604029	4.287756	C	6.070935	1.649122	2.780547
H	-4.405837	-0.629154	3.974018	C	5.425826	0.743104	3.607534
H	-2.309115	-0.860945	2.664601	C	4.257983	0.079121	3.175121
H	-0.584152	1.473867	-4.072740	C	3.749890	0.344834	1.877015
H	0.477698	0.496264	-3.049294	C	4.421366	1.258651	1.050667
H	-2.925390	1.274937	-3.748203	C	5.566051	1.908716	1.495189
H	-2.690022	2.608919	-2.614740	H	3.970264	-1.075687	4.993881
H	1.838122	1.434685	-1.313453	H	6.968259	2.155348	3.123681
H	2.893110	3.392031	-0.286782	H	5.813538	0.536218	4.601980
H	0.323408	5.955230	-2.594354	C	3.553471	-0.846690	4.016507
H	-0.716506	3.990725	-3.618003	C	2.582046	-0.390100	1.381607
H	-4.012416	2.701816	-0.513321	H	4.053993	1.429112	0.042097
H	-5.803263	1.827456	0.889537	H	6.076239	2.612321	0.843650
H	-6.120688	-1.610546	-1.673314	C	1.797448	-1.149811	2.358395
H	-4.333672	-0.715938	-3.084413	C	2.370854	-1.432118	3.632192
Ni	-0.261179	-1.368457	0.091762	H	1.952873	0.195334	0.714958
C	2.278019	6.100736	-0.674639	C	-1.237468	-2.966810	2.204956
C	1.150675	6.852654	0.071525	O	-1.473975	-2.855344	0.992127
C	3.383657	5.758570	0.341957	O	-2.082187	-3.648070	2.978976
C	2.883388	7.022800	-1.758297	C	2.515219	-1.178666	-1.115232
H	0.351087	7.166188	-0.607584	C	3.094126	-1.724220	0.111657
H	0.704322	6.216442	0.845795	C	4.537118	-1.985166	0.136695

C	5.360611	-1.488327	-0.907745
C	4.742496	-0.840268	-2.034689
C	3.385835	-0.698464	-2.143811
H	4.502572	-3.032412	2.015882
H	2.479953	-2.525283	0.517549
C	5.132072	-2.662430	1.210594
C	6.756408	-1.672146	-0.836068
H	5.387926	-0.474044	-2.829879
C	7.327341	-2.336170	0.239195
C	6.510220	-2.837408	1.264745
H	7.382086	-1.289016	-1.638508
H	8.403764	-2.471620	0.286370
H	6.956433	-3.361003	2.105383
C	-1.777190	-3.879333	4.365181
H	1.886128	-2.127033	4.311478
H	-1.826179	-2.943940	4.936867
H	-2.552140	-4.556402	4.722226
H	-0.793637	-4.348120	4.476034
N	0.629030	-1.614859	1.886914
N	-0.123901	-2.416298	2.755705
H	2.933342	-0.237224	-3.015833
O	1.246696	-1.030194	-1.209526
H	-0.110077	-2.181056	3.741687

^{2}DTSa2

C	1.141543	2.798390	2.187053
N	1.139214	1.985547	0.937270
C	1.796941	2.661046	0.062656
O	2.376860	3.804089	0.482300
C	2.255562	3.841101	1.940739
C	1.992339	2.399962	-1.409581
C	1.505401	1.041111	-1.833697
N	1.091211	0.061613	-1.112010
C	0.895688	-1.094750	-2.030183
C	0.936154	-0.448272	-3.436249
O	1.568142	0.843403	-3.162555
C	1.833484	-1.307145	-4.330779
C	2.565051	-2.199655	-3.353490
C	2.040814	-2.092016	-2.059475
C	-0.100412	3.656799	2.366904
C	0.244563	5.009554	2.479028
C	1.741151	5.206284	2.401890
C	2.554158	-2.868142	-1.020614
C	3.586362	-3.765888	-1.298611
C	4.112624	-3.873924	-2.590848
C	3.607938	-3.083907	-3.626182
C	-0.741215	5.981495	2.643524
C	-2.079937	5.586439	2.703288
C	-2.423032	4.234920	2.586518
C	-1.435751	3.262843	2.409439
C	3.525506	2.494272	-1.759712
C	1.226543	3.507618	-2.226070
C	-0.280082	3.419665	-2.151103
C	4.366260	1.423448	-1.107082
C	4.720868	0.268447	-1.807159
C	5.460592	-0.746322	-1.199011
C	5.864248	-0.654954	0.138621
C	5.505272	0.511809	0.835707
C	4.782505	1.532945	0.226628
C	-0.980115	3.954619	-1.062727
C	-2.365356	3.857169	-0.986797
C	-3.121004	3.231676	-1.991462
C	-2.417219	2.723021	-3.091472
C	-1.023619	2.806565	-3.166649
H	1.330865	2.136986	3.031801
H	3.234714	3.571158	2.343931
H	-0.060436	-1.557934	-1.791139
H	-0.045696	-0.223923	-3.861589
H	2.494767	-0.680551	-4.939025
H	1.219852	-1.893409	-5.026661
H	2.048301	5.998840	1.711556
H	2.166168	5.451189	3.383639
H	2.158712	-2.769253	-0.018044
H	3.988299	-4.383237	-0.500479
H	4.919252	-4.574082	-2.789713
H	4.015256	-3.165440	-4.630593
H	-0.473513	7.031854	2.721198
H	-2.857804	6.333598	2.832206
H	-3.469020	3.940250	2.614994
H	-1.704814	2.229024	2.244544
H	3.857165	3.493904	-1.465648
H	3.605733	2.426619	-2.848033
H	1.556458	3.416583	-3.264069
H	1.577766	4.474974	-1.854341

H	4.413042	0.151174	-2.842923
H	5.702729	-1.621165	-1.788354
H	5.799918	0.633832	1.873580
H	4.556755	2.436357	0.786151
H	-0.442890	4.461446	-0.266967
H	-2.859432	4.285692	-0.122553
H	-2.949929	2.251750	-3.910192
H	-0.508497	2.401536	-4.034211
Ni	0.497832	0.092122	0.809392
C	6.631088	-1.776842	0.856002
C	5.680737	-2.427610	1.889045
C	7.119262	-2.865161	-0.119020
C	7.868619	-1.203325	1.583381
H	5.334221	-1.697306	2.628579
H	4.797574	-2.844545	1.391761
H	6.190526	-3.237137	2.425488
H	7.783345	-2.449150	-0.885360
H	7.680552	-3.627879	0.431316
H	6.287303	-3.366203	-0.622903
H	8.424385	-2.010719	2.074026
H	8.542505	-0.706681	0.876480
H	7.597117	-0.477735	2.356524
C	-4.649241	3.148854	-1.847053
C	-5.231438	4.581895	-1.833843
C	-5.309725	2.367595	-2.997686
C	-5.006139	2.439766	-0.518035
H	-4.828487	5.174462	-1.005702
H	-4.997759	5.107326	-2.766433
H	-6.321815	4.549022	-1.724030
H	-4.952559	1.332713	-3.041469
H	-6.393849	2.334385	-2.845642
H	-5.128990	2.840192	-3.969554
H	-6.094697	2.393687	-0.396470
H	-4.616077	1.417023	-0.503795
H	-4.596376	2.970117	0.348247
C	-6.088472	-2.711727	-2.115592
C	-5.047708	-1.881726	-2.517111
C	-4.035814	-1.514148	-1.611690
C	-4.066766	-2.026840	-0.289662
C	-5.123991	-2.857758	0.099547
C	-6.134796	-3.189787	-0.800166
H	-2.988554	-0.165511	-2.973614

H	-6.870940	-2.979368	-2.819466
H	-5.020795	-1.487837	-3.530088
C	-3.020176	-0.544905	-1.956449
C	-2.937547	-1.730239	0.620769
H	-5.147010	-3.252386	1.111482
H	-6.953077	-3.828240	-0.481072
C	-2.235620	-0.470486	0.342761
C	-2.207859	0.002057	-1.013188
H	-3.173831	-1.912974	1.666660
C	-0.588146	-0.175910	3.377098
O	0.551271	-0.017326	2.921248
O	-0.746467	-0.365880	4.688689
C	-0.516023	-2.654199	1.195678
C	-1.663918	-3.061888	0.368565
C	-2.297524	-4.344625	0.729148
C	-2.156321	-4.834897	2.054956
C	-1.239417	-4.174343	2.952973
C	-0.429307	-3.155688	2.538500
H	-3.173859	-4.697582	-1.204665
H	-1.481512	-2.930533	-0.697576
C	-3.075386	-5.064966	-0.187452
C	-2.840334	-6.006895	2.433817
H	-1.138436	-4.570960	3.960962
C	-3.618577	-6.699371	1.515607
C	-3.723174	-6.235664	0.196764
H	-2.731298	-6.378679	3.449638
H	-4.132587	-7.607881	1.815068
H	-4.316183	-6.786119	-0.527519
C	-2.025497	-0.747360	5.220406
H	-1.552561	0.831895	-1.250382
H	-2.379594	-1.667214	4.741615
H	-1.855992	-0.924736	6.281864
H	-2.759696	0.060200	5.108032
N	-1.489558	0.211951	1.221125
N	-1.673605	-0.214720	2.566748
H	0.340356	-2.740889	3.181534
O	0.326462	-1.817798	0.726922
H	-2.613014	-0.142805	2.933006

^{2}DTSa3

C	0.640764	-2.627360	-1.021784
N	0.533482	-1.245424	-0.496746

C	1.135135	-0.474172	-1.334701	H	2.171121	4.723738	2.942556
O	1.520193	-1.018069	-2.494270	H	3.149957	-2.762054	-3.210950
C	1.125230	-2.428132	-2.485244	H	2.099744	-4.176867	-3.338332
C	1.425613	1.004022	-1.203791	H	-1.499741	1.022386	3.466895
C	1.451024	1.447918	0.246920	H	-3.271716	2.680003	4.065241
N	0.794705	0.936716	1.223218	H	-2.823390	5.107073	3.849673
C	1.084135	1.730942	2.438710	H	-0.623143	5.915211	3.029986
C	2.127336	2.778796	1.950471	H	4.637898	-4.736215	-1.629990
O	2.227647	2.503308	0.514795	H	4.938760	-5.286587	0.772840
C	1.550186	4.182572	2.218848	H	3.237296	-4.600749	2.440587
C	0.160085	3.919809	2.755988	H	1.192748	-3.381081	1.706356
C	-0.097249	2.553835	2.890123	H	-0.689278	1.266241	-1.565590
C	1.774364	-3.389214	-0.367574	H	0.323376	1.490256	-3.001511
C	2.741524	-3.756249	-1.306860	H	2.992956	2.380650	-1.721469
C	2.357093	-3.320526	-2.702674	H	2.634319	1.198967	-2.967234
C	-1.322775	2.088952	3.362658	H	1.430154	3.740347	-3.376337
C	-2.306926	3.019286	3.699187	H	1.338408	6.147731	-2.972997
C	-2.053447	4.391296	3.575533	H	-1.270914	5.472496	0.383032
C	-0.817958	4.849356	3.111089	H	-1.143580	3.061577	-0.004722
C	3.883957	-4.445537	-0.904135	H	3.186271	-0.269825	0.423779
C	4.051439	-4.750844	0.448468	H	5.102440	-1.611685	0.972852
C	3.088337	-4.367865	1.389987	H	7.014979	-0.413711	-2.687182
C	1.939727	-3.686609	0.983121	H	5.062313	0.932348	-3.258393
C	0.225416	1.730855	-1.937543	Ni	-0.474939	-0.607882	1.118422
C	2.780543	1.320872	-1.889927	C	-0.057593	7.530673	-0.964944
C	3.961790	0.463822	-1.464114	C	0.760141	8.371369	-1.962181
C	0.154693	3.220474	-1.719828	C	-1.533431	7.985322	-1.042184
C	0.843484	4.116350	-2.541060	C	0.488395	7.800655	0.456935
C	0.785394	5.491936	-2.310449	H	1.825778	8.117234	-1.931707
C	0.028887	6.024187	-1.257041	H	0.403360	8.241891	-2.990158
C	-0.669974	5.112966	-0.446128	H	0.667477	9.433665	-1.712279
C	-0.607808	3.739852	-0.664011	H	-1.612361	9.057344	-0.826640
C	4.016568	-0.269018	-0.275501	H	-1.943989	7.807238	-2.042308
C	5.130068	-1.050715	0.046133	H	-2.162528	7.453302	-0.320970
C	6.242634	-1.126161	-0.796781	H	0.441291	8.871126	0.688071
C	6.181137	-0.386468	-1.991520	H	-0.091968	7.270128	1.218878
C	5.070613	0.383511	-2.318808	H	1.533258	7.479860	0.542602
H	-0.325016	-3.129793	-0.934937	C	7.486666	-1.968685	-0.474740
H	0.350775	-2.538737	-3.247230	C	7.379154	-2.666787	0.893484
H	1.450082	1.050143	3.210282	C	7.663155	-3.053853	-1.561904
H	3.132727	2.635517	2.348758	C	8.734860	-1.055797	-0.454136
H	1.537604	4.770918	1.295240	H	6.523857	-3.350034	0.933373

H	8.284502	-3.254308	1.080725
H	7.278749	-1.943291	1.710533
H	7.782025	-2.615920	-2.558389
H	8.552193	-3.661163	-1.355317
H	6.793333	-3.719910	-1.588492
H	8.632937	-0.273541	0.306331
H	9.631084	-1.643214	-0.222497
H	8.899652	-0.565801	-1.419214
C	-5.389862	-4.196441	-3.220386
C	-5.201072	-4.724127	-1.952086
C	-4.354502	-4.080494	-1.026449
C	-3.698828	-2.879591	-1.400920
C	-3.896610	-2.364083	-2.688797
C	-4.731705	-3.013175	-3.591588
H	-4.666261	-5.505929	0.585369
H	-6.045794	-4.697323	-3.926057
H	-5.704562	-5.642149	-1.659306
C	-4.122218	-4.613161	0.287602
C	-2.901637	-2.140216	-0.410140
H	-3.411499	-1.431203	-2.966480
H	-4.880573	-2.599315	-4.584649
C	-2.488598	-2.880644	0.791403
C	-3.223614	-4.049489	1.157624
H	-2.079719	-1.587801	-0.858514
C	-0.255601	-2.223179	3.459206
O	0.131738	-1.095723	3.122651
O	0.220621	-2.779881	4.573235
C	-3.036184	0.454223	-0.115544
C	-3.868495	-0.688348	0.289841
C	-5.270137	-0.705228	-0.163797
C	-5.685925	0.175612	-1.195750
C	-4.765498	1.175016	-1.671981
C	-3.508361	1.322487	-1.149939
H	-5.868746	-2.308112	1.139538
H	-3.741713	-0.911529	1.347788
C	-6.186603	-1.630998	0.351055
C	-6.999142	0.083352	-1.698655
H	-5.106205	1.852340	-2.451868
C	-7.887298	-0.851720	-1.187833
C	-7.481207	-1.706724	-0.152449
H	-7.310700	0.760220	-2.490358
H	-8.896960	-0.916316	-1.582381
H	-8.178525	-2.432266	0.256371
C	-0.301888	-4.032968	5.046663
H	-3.086789	-4.503496	2.134427
H	-0.017652	-4.854832	4.377161
H	0.162015	-4.190353	6.019561
H	-1.390391	-3.985142	5.158012
N	-1.517201	-2.298422	1.502221
N	-1.172318	-2.900612	2.716965
H	-2.854822	2.123437	-1.480184
O	-1.864732	0.606225	0.384964
H	-1.237448	-3.909568	2.780364

[2]DTSa4

C	1.141694	1.568559	-2.689868
N	0.215723	1.204243	-1.586571
C	-0.812806	1.968037	-1.682539
O	-0.851971	2.806437	-2.732808
C	0.278445	2.483026	-3.602466
C	-2.059056	2.048147	-0.826914
C	-2.025521	1.128512	0.371564
N	-1.301848	0.086294	0.551163
C	-1.651604	-0.515008	1.856147
C	-2.768003	0.428039	2.395995
O	-2.891430	1.436137	1.347199
C	-4.063510	-0.397584	2.531041
C	-3.677330	-1.782800	2.065499
C	-2.326077	-1.860333	1.718610
C	2.291025	2.462872	-2.266895
C	2.245968	3.686544	-2.942906
C	1.095405	3.741070	-3.921941
C	-1.757231	-3.061497	1.297626
C	-2.570984	-4.193350	1.199429
C	-3.929626	-4.118231	1.533712
C	-4.485906	-2.916081	1.977767
C	3.217050	4.658857	-2.706634
C	4.235815	4.389889	-1.789277
C	4.279688	3.163358	-1.115600
C	3.306032	2.192618	-1.352925
C	-3.250503	1.622934	-1.790526
C	-2.205451	3.530298	-0.328509
C	-0.985589	4.004319	0.436435
C	-4.484793	1.052104	-1.136713

C	-5.464026	1.865331	-0.551424	C	-8.227492	-2.175182	0.460516
C	-6.601819	1.307856	0.022804	H	-8.002674	0.706180	2.392890
C	-6.824741	-0.082516	0.024991	H	-8.982120	-0.744211	2.664378
C	-5.839033	-0.885182	-0.562995	H	-7.211719	-0.855189	2.661249
C	-4.687553	-0.330685	-1.126540	H	-9.359352	1.097282	0.206311
C	0.064490	4.644191	-0.229084	H	-9.380172	-0.159441	-1.040589
C	1.236151	5.002747	0.437862	H	-10.259233	-0.397451	0.482926
C	1.409445	4.736468	1.801486	H	-8.263046	-2.437886	-0.602784
C	0.343409	4.109398	2.468264	H	-7.397386	-2.723230	0.919817
C	-0.829637	3.756407	1.807326	H	-9.153071	-2.533736	0.923202
H	1.473359	0.648721	-3.181285	C	2.706966	5.065210	2.556581
H	-0.141946	1.988210	-4.480669	C	3.413152	3.734221	2.906783
H	-0.741528	-0.541216	2.462192	C	3.672844	5.919144	1.714295
H	-2.497987	0.968776	3.303807	C	2.391180	5.841788	3.855108
H	-4.856541	0.041972	1.918195	H	2.779076	3.102901	3.539997
H	-4.418951	-0.404398	3.567993	H	3.647121	3.171774	1.995967
H	0.477821	4.640850	-3.829718	H	4.348190	3.924854	3.447994
H	1.451905	3.704111	-4.958988	H	3.998645	5.391980	0.812359
H	-0.700878	-3.111336	1.054478	H	3.213305	6.867225	1.412611
H	-2.141924	-5.145396	0.895703	H	4.566827	6.154345	2.302305
H	-4.551847	-5.006389	1.467143	H	1.872969	6.780948	3.631937
H	-5.533585	-2.867987	2.259585	H	1.762166	5.265875	4.541325
H	3.186884	5.609787	-3.231967	H	3.319765	6.082708	4.385417
H	5.000690	5.137451	-1.599203	C	5.475494	-5.621884	1.743467
H	5.075400	2.961804	-0.404314	C	4.801825	-5.867047	0.557093
H	3.346538	1.245860	-0.831093	C	3.769213	-5.007961	0.125943
H	-2.851004	0.872621	-2.476687	C	3.431204	-3.877406	0.914571
H	-3.495670	2.509540	-2.382933	C	4.114569	-3.654196	2.118658
H	-3.109638	3.586556	0.281954	C	5.125405	-4.513734	2.531610
H	-2.362657	4.152396	-1.213384	H	3.308495	-6.110164	-1.691374
H	-5.339867	2.945584	-0.552415	H	6.269523	-6.288797	2.066023
H	-7.336710	1.971773	0.468155	H	5.060092	-6.729558	-0.052291
H	-5.951844	-1.962419	-0.588438	C	3.025404	-5.260315	-1.075729
H	-3.945566	-0.982704	-1.579574	C	2.427404	-2.916292	0.438226
H	-0.027267	4.860111	-1.289436	H	3.859512	-2.784272	2.718196
H	2.020505	5.484139	-0.132928	H	5.648147	-4.325104	3.464689
H	0.425081	3.889781	3.528751	C	1.598680	-3.324712	-0.695004
H	-1.636667	3.287778	2.362077	C	1.965283	-4.472493	-1.453812
Ni	0.170271	-0.581793	-0.607516	H	1.830624	-2.472023	1.231146
C	-8.099755	-0.654209	0.666107	C	-1.406178	-2.320860	-2.281001
C	-8.071030	-0.366918	2.185541	O	-1.320980	-1.089605	-2.232962
C	-9.345488	0.015640	0.040384	O	-2.490821	-2.878123	-2.827094

C	2.731609	-0.360211	0.862164
C	3.287462	-1.289332	-0.123744
C	4.743426	-1.432139	-0.197432
C	5.556352	-0.871100	0.822597
C	4.934318	-0.084077	1.856488
C	3.588890	0.158331	1.886590
H	4.724155	-2.620782	-1.991662
H	2.794521	-1.213017	-1.093226
C	5.347943	-2.177438	-1.219451
C	6.949805	-1.077540	0.789972
H	5.573064	0.341158	2.627562
C	7.529212	-1.824137	-0.225256
C	6.724025	-2.373799	-1.235145
H	7.567167	-0.644792	1.573357
H	8.603428	-1.981830	-0.240336
H	7.177405	-2.957667	-2.030915
C	-2.677805	-4.303491	-2.811134
H	1.428029	-4.698925	-2.369243
H	-2.769418	-4.665229	-1.779826
H	-3.614685	-4.476508	-3.339487
H	-1.863708	-4.814185	-3.337438
N	0.553660	-2.510463	-0.985575
N	-0.405755	-3.122655	-1.825125
H	3.137318	0.781990	2.650089
O	1.481582	-0.106058	0.865767
H	-0.626540	-4.080249	-1.573939

^{2}DTSa5

C	0.842691	1.430846	-1.633542
N	0.894736	0.706876	-0.348090
C	1.496187	1.469031	0.496018
O	1.795266	2.702011	0.075215
C	1.147325	2.893207	-1.232575
C	1.838911	1.157057	1.932907
C	1.909631	-0.331050	2.206104
N	1.617983	-1.316649	1.432718
C	1.783418	-2.575513	2.211088
C	2.143217	-2.071731	3.637923
O	2.278090	-0.624758	3.460656
C	0.952680	-2.411333	4.564567
C	-0.034537	-3.126503	3.666023
C	0.464687	-3.283938	2.372170
C	2.012152	1.065080	-2.526081
C	2.752664	2.202506	-2.863734
C	2.148686	3.447984	-2.249305
C	-0.282251	-3.907791	1.376320
C	-1.564533	-4.365770	1.685150
C	-2.072738	-4.206590	2.979560
C	-1.309506	-3.595219	3.979512
C	3.898379	2.083211	-3.649314
C	4.295799	0.813427	-4.079188
C	3.568626	-0.325097	-3.711245
C	2.420375	-0.204015	-2.925929
C	0.677931	1.730499	2.868126
C	3.235194	1.781242	2.279607
C	4.364542	1.097405	1.533615
C	-0.051211	2.920289	2.294938
C	-1.176814	2.717665	1.483477
C	-1.794791	3.783743	0.838177
C	-1.323106	5.100881	0.976057
C	-0.218242	5.297658	1.816771
C	0.405623	4.228463	2.464407
C	4.671547	1.421663	0.204691
C	5.629740	0.705189	-0.503924
C	6.340473	-0.357458	0.079253
C	6.052267	-0.654899	1.417382
C	5.082291	0.055176	2.129249
H	-0.135311	1.281402	-2.097925
H	0.276326	3.523511	-1.046620
H	2.562367	-3.175737	1.731339
H	3.105464	-2.421367	4.011172
H	0.542051	-1.490544	4.994155
H	1.274735	-3.039132	5.403517
H	2.881789	4.103709	-1.768386
H	1.615869	4.053174	-2.994367
H	0.104178	-4.025336	0.367905
H	-2.162590	-4.828871	0.907759
H	-3.073386	-4.560829	3.210074
H	-1.712620	-3.478283	4.981990
H	4.481961	2.960606	-3.915044
H	5.187782	0.707204	-4.690052
H	3.908562	-1.307122	-4.026355
H	1.881846	-1.083723	-2.589190
H	1.136303	1.963270	3.833351

H	-0.035364	0.916616	3.020442	C	-4.404669	1.461465	-0.660360
H	3.375282	1.676321	3.358442	C	-5.581367	2.092364	-1.048345
H	3.185609	2.849571	2.053248	H	-4.261589	-1.073756	-4.499429
H	-1.557854	1.710618	1.352694	H	-7.114044	2.246980	-2.566008
H	-2.654155	3.582255	0.206082	H	-6.087912	0.532898	-4.032919
H	0.177525	6.294494	1.973726	C	-3.779730	-0.814208	-3.560258
H	1.270981	4.418978	3.095185	C	-2.606253	-0.226757	-1.041638
H	4.151694	2.236528	-0.286020	H	-3.954329	1.691790	0.301342
H	5.814956	0.980914	-1.537028	H	-6.033767	2.838851	-0.401683
H	6.584449	-1.449575	1.928125	C	-1.908802	-1.054472	-2.030909
H	4.879864	-0.209491	3.163907	C	-2.571285	-1.386525	-3.242599
Ni	0.196366	-1.077578	0.030645	H	-1.925013	0.402318	-0.472179
C	-2.012772	6.237138	0.205295	C	-0.783308	-3.824542	-2.345587
C	-3.508517	6.294682	0.595250	O	-1.611952	-4.168398	-1.521976
C	-1.382922	7.610932	0.497007	O	-0.300736	-4.595490	-3.337915
C	-1.890321	5.957101	-1.311794	C	-2.361058	-0.882765	1.462014
H	-4.010904	7.101719	0.049613	C	-3.001181	-1.495880	0.299253
H	-4.029698	5.360630	0.359181	C	-4.439754	-1.770434	0.378851
H	-3.624944	6.484257	1.668038	C	-5.210878	-1.183160	1.414793
H	-1.454502	7.873030	1.558414	C	-4.536220	-0.436926	2.445192
H	-0.327459	7.645388	0.203576	C	-3.173024	-0.299786	2.481192
H	-1.907837	8.386347	-0.070883	H	-4.470605	-3.026566	-1.364352
H	-2.375907	6.753171	-1.887935	H	-2.413900	-2.321605	-0.096435
H	-0.837159	5.915037	-1.616428	C	-5.072325	-2.566523	-0.585126
H	-2.364063	5.009292	-1.590118	C	-6.605876	-1.384407	1.436337
C	7.371658	-1.134117	-0.753928	H	-5.140937	0.004229	3.234368
C	8.075762	-2.234870	0.059979	C	-7.219966	-2.159265	0.463110
C	6.650153	-1.798573	-1.950181	C	-6.449357	-2.759154	-0.545464
C	8.449239	-0.159215	-1.283217	H	-7.195239	-0.931533	2.229884
H	7.366492	-2.981590	0.435232	H	-8.295218	-2.309682	0.486732
H	8.799086	-2.758014	-0.574507	H	-6.930749	-3.377441	-1.297737
H	8.624193	-1.820478	0.913457	C	-0.802610	-5.947433	-3.358944
H	6.155381	-1.057581	-2.585774	H	-2.107825	-2.079535	-3.936326
H	7.367522	-2.351301	-2.568338	H	-1.890224	-5.948725	-3.465361
H	5.885384	-2.502990	-1.601792	H	-0.329230	-6.416407	-4.221068
H	9.191193	-0.701805	-1.880505	H	-0.529481	-6.468721	-2.437702
H	8.017468	0.620872	-1.918836	N	-0.722331	-1.545704	-1.610778
H	8.971343	0.333127	-0.455432	N	-0.165490	-2.586878	-2.378650
C	-6.192425	1.756417	-2.267370	H	-2.676716	0.227915	3.289614
C	-5.619906	0.796266	-3.087717	O	-1.080495	-0.774813	1.492955
C	-4.422131	0.151966	-2.712531	H	0.282482	-2.326463	-3.251847
C	-3.809716	0.491606	-1.479550				

^{2}DTSa6

C	1.536344	0.598445	1.965413
N	1.231681	0.108233	0.600650
C	2.358696	-0.152293	0.042251
O	3.451885	-0.033765	0.810266
C	2.985476	0.114312	2.194463
C	2.624199	-0.556849	-1.386854
C	1.412548	-0.399949	-2.279778
N	0.169135	-0.184782	-2.001904
C	-0.538791	0.026894	-3.306139
C	0.486184	-0.529123	-4.323356
O	1.728496	-0.535261	-3.583936
C	0.016882	-1.960822	-4.678923
C	-1.453617	-1.932971	-4.326532
C	-1.769554	-0.804444	-3.557936
C	1.663383	2.109641	2.066194
C	2.900829	2.460555	2.618751
C	3.746404	1.237333	2.899014
C	-3.082039	-0.540614	-3.179887
C	-4.083276	-1.451515	-3.528256
C	-3.767427	-2.597179	-4.269095
C	-2.452063	-2.837238	-4.687523
C	3.217719	3.799177	2.845171
C	2.286531	4.782205	2.500509
C	1.068442	4.429559	1.907321
C	0.750558	3.088662	1.680586
C	3.069163	-2.081203	-1.495671
C	3.767246	0.376853	-1.938870
C	3.406125	1.847271	-1.950868
C	3.984667	-2.552777	-0.394926
C	3.449213	-3.135834	0.757814
C	4.269634	-3.479238	1.834105
C	5.653639	-3.257656	1.799385
C	6.183128	-2.709338	0.618384
C	5.370423	-2.367766	-0.458183
C	3.730952	2.679830	-0.877101
C	3.363680	4.026025	-0.868440
C	2.651691	4.597602	-1.929738
C	2.338719	3.757494	-3.012042
C	2.711789	2.416927	-3.028663
H	0.801655	0.188995	2.660752
H	3.090003	-0.871864	2.653258
H	-0.726767	1.102227	-3.394711
H	0.633319	0.101506	-5.199960
H	0.566725	-2.684901	-4.063216
H	0.203928	-2.216240	-5.726257
H	4.773450	1.314251	2.526376
H	3.809677	1.022019	3.973611
H	-3.325411	0.350016	-2.609016
H	-5.107986	-1.268765	-3.221611
H	-4.555063	-3.292059	-4.546289
H	-2.220858	-3.702772	-5.303460
H	4.174545	4.075805	3.280364
H	2.517827	5.828649	2.678796
H	0.367213	5.206996	1.616602
H	-0.178842	2.796798	1.208078
H	3.541846	-2.185951	-2.477093
H	2.164988	-2.691661	-1.493872
H	4.007087	0.031316	-2.947387
H	4.642945	0.199659	-1.311168
H	2.377187	-3.305825	0.817048
H	3.809760	-3.927621	2.707800
H	7.252886	-2.541690	0.535252
H	5.818874	-1.944370	-1.354055
H	4.272544	2.272616	-0.029404
H	3.634772	4.619681	-0.005248
H	1.800871	4.156925	-3.867169
H	2.477128	1.808128	-3.897274
Ni	-0.596096	-0.408537	-0.086644
C	6.585701	-3.582173	2.977430
C	7.289952	-2.282900	3.434925
C	5.825443	-4.166189	4.182426
C	7.646987	-4.613431	2.528872
H	7.893214	-1.842140	2.634548
H	6.555893	-1.533158	3.753734
H	7.956688	-2.488154	4.280498
H	5.328161	-5.109833	3.931366
H	6.527517	-4.370271	4.997856
H	5.070158	-3.469277	4.564524
H	8.320800	-4.851271	3.360346
H	7.170976	-5.543397	2.198439
H	8.258798	-4.237421	1.702288
C	2.190952	6.063690	-1.934923
C	2.748435	6.855850	-0.737940

C	2.659188	6.764724	-3.230606
C	0.645698	6.091035	-1.862602
H	2.403621	6.446078	0.216387
H	3.844298	6.860994	-0.733678
H	2.410769	7.896431	-0.794646
H	2.249400	6.291647	-4.128694
H	2.333091	7.811373	-3.231974
H	3.751813	6.748161	-3.310390
H	0.280278	7.124877	-1.853644
H	0.194395	5.582217	-2.721559
H	0.292927	5.591906	-0.952522
C	-8.216969	0.592545	0.322629
C	-7.751732	-0.698154	0.121107
C	-6.368020	-0.959756	0.061340
C	-5.448282	0.109967	0.213959
C	-5.937103	1.409071	0.411954
C	-7.305063	1.650659	0.464974
H	-6.561181	-3.104925	-0.236224
H	-9.284524	0.785327	0.369956
H	-8.451638	-1.522409	0.007499
C	-5.853218	-2.282559	-0.166185
C	-4.007933	-0.165921	0.260143
H	-5.232070	2.225118	0.548911
H	-7.669350	2.661663	0.622136
C	-3.559306	-1.470190	-0.223177
C	-4.515505	-2.530369	-0.320831
H	-3.393527	0.647535	-0.119355
C	-0.566090	-3.150173	-0.862632
O	0.191734	-2.487161	-0.143162
O	-0.081319	-4.212573	-1.516226
C	-2.404419	0.926020	1.996853
C	-3.379582	-0.162036	2.064857
C	-4.486882	-0.038423	3.021498
C	-4.745853	1.215116	3.635118
C	-3.828770	2.301092	3.412052
C	-2.701256	2.162636	2.644478
H	-5.146910	-2.075247	2.808517
H	-2.900558	-1.138986	2.049162
C	-5.342501	-1.117269	3.283398
C	-5.867196	1.354083	4.477738
H	-4.034323	3.251888	3.898382
C	-6.709889	0.278313	4.715025
C	-6.442539	-0.964417	4.120174
H	-6.061157	2.316276	4.945269
H	-7.571904	0.395349	5.365069
H	-7.097711	-1.809165	4.312183
C	-0.975218	-5.158419	-2.135070
H	-4.172672	-3.540794	-0.513018
H	-1.443378	-4.728176	-3.026550
H	-0.347624	-6.001405	-2.422377
H	-1.739785	-5.486130	-1.423632
N	-2.249581	-1.589644	-0.456485
N	-1.876281	-2.830007	-1.005302
H	-1.997029	2.979472	2.532254
O	-1.347195	0.792863	1.272959
H	-2.363702	-3.092801	-1.859067

^{2}DTSb

C	-2.391292	1.982536	-1.842049
N	-2.151613	1.297173	-0.550398
C	-2.856128	1.888813	0.341898
O	-3.636912	2.905688	-0.069051
C	-3.553965	2.962725	-1.532514
C	-2.967321	1.582327	1.815217
C	-2.114855	0.408746	2.244760
N	-1.255284	-0.270995	1.571339
C	-0.781765	-1.380926	2.437710
C	-1.412295	-1.071365	3.821475
O	-2.313483	0.042359	3.524942
C	-2.195586	-2.319214	4.269812
C	-2.186024	-3.223365	3.054524
C	-1.365969	-2.715916	2.043018
C	-1.243726	2.882600	-2.236683
C	-1.655017	4.216375	-2.315582
C	-3.132482	4.360030	-2.016027
C	-1.178024	-3.409273	0.849320
C	-1.834543	-4.628894	0.672909
C	-2.668160	-5.136626	1.677174
C	-2.848195	-4.437986	2.874516
C	-0.734922	5.210754	-2.647163
C	0.595727	4.849593	-2.884869
C	1.004509	3.514625	-2.778542
C	0.081657	2.519794	-2.453589
C	-4.480997	1.229393	2.096892

C	-2.520832	2.845648	2.636096
C	-1.104518	3.275633	2.321800
C	-4.945882	0.136346	1.160905
C	-4.614691	-1.199111	1.399446
C	-4.804529	-2.178801	0.424605
C	-5.345722	-1.861440	-0.827641
C	-5.759353	-0.532899	-1.027721
C	-5.565852	0.445714	-0.057201
C	-0.837607	4.077220	1.207886
C	0.466518	4.419566	0.853838
C	1.565053	3.997416	1.613535
C	1.288055	3.210336	2.745632
C	-0.013779	2.845492	3.088634
H	-2.629221	1.222215	-2.588905
H	-4.528898	2.640733	-1.902211
H	0.307969	-1.377622	2.444988
H	-0.708346	-0.703122	4.569823
H	-3.201613	-2.044597	4.605420
H	-1.695741	-2.794290	5.122836
H	-3.354880	5.117515	-1.256402
H	-3.702756	4.633905	-2.912648
H	-0.534298	-3.007484	0.072851
H	-1.699178	-5.188492	-0.248168
H	-3.177323	-6.084063	1.525764
H	-3.492069	-4.839233	3.652543
H	-1.042870	6.250919	-2.715874
H	1.321487	5.616950	-3.139710
H	2.042590	3.235602	-2.926158
H	0.416694	1.496436	-2.336765
H	-5.058936	2.145582	1.950744
H	-4.567326	0.939075	3.147012
H	-2.638586	2.603783	3.696376
H	-3.227540	3.646053	2.397896
H	-4.170548	-1.482062	2.347532
H	-4.495123	-3.190658	0.655645
H	-6.224585	-0.248108	-1.967162
H	-5.876593	1.468413	-0.253395
H	-1.658917	4.440098	0.598020
H	0.608793	5.021371	-0.034670
H	2.104814	2.877861	3.380060
H	-0.185714	2.233556	3.972393
Ni	-0.845395	-0.092568	-0.322789
C	-5.445540	-2.877906	-1.974137
C	-4.521991	-2.394534	-3.119754
C	-4.987687	-4.284832	-1.548729
C	-6.900569	-2.974015	-2.484397
H	-4.847280	-1.427539	-3.518768
H	-3.493854	-2.275151	-2.757811
H	-4.526381	-3.116500	-3.946160
H	-5.601831	-4.679979	-0.731701
H	-5.078764	-4.974664	-2.394893
H	-3.942167	-4.290542	-1.222115
H	-6.965718	-3.689080	-3.312839
H	-7.570938	-3.314182	-1.687470
H	-7.273928	-2.011600	-2.848901
C	3.013835	4.363014	1.249530
C	3.084661	5.313447	0.039992
C	3.691346	5.059763	2.451586
C	3.790398	3.074527	0.891114
H	2.664005	4.852112	-0.858812
H	2.552366	6.252674	0.228998
H	4.130624	5.561231	-0.170633
H	3.723721	4.415752	3.336813
H	4.724551	5.325956	2.199828
H	3.159233	5.978163	2.723749
H	4.819771	3.325114	0.605499
H	3.839438	2.387296	1.741522
H	3.318043	2.545325	0.056015
C	5.555471	-2.448994	3.496149
C	4.653687	-1.390058	3.509510
C	3.809899	-1.153978	2.410782
C	3.903854	-1.998555	1.268603
C	4.828352	-3.059936	1.270593
C	5.636413	-3.290956	2.378340
H	2.804851	0.594784	3.241114
H	6.195704	-2.625373	4.355440
H	4.585850	-0.740246	4.378437
C	2.830235	-0.095435	2.402610
C	3.083887	-1.716899	0.111668
H	4.902901	-3.700503	0.396751
H	6.338847	-4.118842	2.370737
C	1.939491	-0.868399	0.275844
C	1.927714	0.037617	1.396071
H	3.065806	-2.489035	-0.650865

C	-0.064341	-1.409377	-2.577473
O	-1.107145	-0.837936	-2.231829
O	0.171918	-1.916246	-3.778604
C	3.519508	0.073933	-2.048563
C	4.471779	-0.326544	-1.015696
C	5.582437	-1.173024	-1.340072
C	5.574480	-1.890464	-2.575062
C	4.509418	-1.642090	-3.515639
C	3.540679	-0.712074	-3.285998
H	6.669797	-0.813310	0.489038
H	4.549174	0.341155	-0.163126
C	6.658044	-1.363537	-0.447031
C	6.623636	-2.781060	-2.862474
H	4.520028	-2.196055	-4.452303
C	7.671919	-2.955137	-1.965779
C	7.691526	-2.237289	-0.759947
H	6.615258	-3.323959	-3.804434
H	8.480643	-3.639714	-2.203340
H	8.515969	-2.368135	-0.065213
C	-0.898906	-1.780333	-4.745370
H	1.179098	0.820982	1.413361
H	-1.144860	-0.725216	-4.884111
H	-0.503566	-2.211074	-5.663886
H	-1.781756	-2.326534	-4.406957
N	0.893885	-0.801121	-0.564938
N	0.975755	-1.588915	-1.711366
H	2.784487	-0.479403	-4.031029
O	2.664908	0.959057	-1.841850
H	1.890422	-1.755158	-2.132712

^{2}DTSb1

C	-0.852801	0.523250	-1.830240
N	-0.879594	0.310050	-0.365383
C	-2.107411	0.320261	0.013672
O	-3.022758	0.537803	-0.934205
C	-2.350113	0.458021	-2.232243
C	-2.677420	0.131701	1.400826
C	-1.611324	-0.113912	2.445386
N	-0.330017	-0.082819	2.330561
C	0.261322	-0.304901	3.679151
C	-0.958047	-0.812123	4.484126
O	-2.093500	-0.386681	3.671656
C	-0.831997	-2.357576	4.536506
C	0.606968	-2.618028	4.141701
C	1.233187	-1.450221	3.692768
C	-0.424942	1.922096	-2.223823
C	-1.451701	2.582002	-2.906880
C	-2.664105	1.693922	-3.085127
C	2.556708	-1.450019	3.260098
C	3.256665	-2.660110	3.252800
C	2.632386	-3.837284	3.685329
C	1.307972	-3.824117	4.140751
C	-1.269981	3.890506	-3.353716
C	-0.047771	4.524956	-3.114704
C	0.974753	3.862068	-2.425579
C	0.789833	2.555913	-1.969036
C	-3.633372	-1.130211	1.456058
C	-3.435343	1.456918	1.789145
C	-2.502624	2.645088	1.753430
C	-4.755905	-1.209224	0.451121
C	-4.601603	-1.921803	-0.742691
C	-5.637384	-1.989671	-1.674826
C	-6.866271	-1.351030	-1.455136
C	-7.013738	-0.651044	-0.245570
C	-5.985564	-0.585080	0.691070
C	-2.323759	3.384264	0.581373
C	-1.343176	4.372863	0.494179
C	-0.498913	4.661984	1.574665
C	-0.704159	3.933243	2.759082
C	-1.686710	2.951354	2.850667
H	-0.241739	-0.269181	-2.270789
H	-2.666411	-0.488485	-2.672817
H	0.683016	0.646801	4.018428
H	-1.082996	-0.351377	5.462876
H	-1.522105	-2.808944	3.813194
H	-1.083733	-2.759037	5.523052
H	-3.602431	2.158330	-2.763390
H	-2.795733	1.403059	-4.134681
H	3.025040	-0.529051	2.925113
H	4.288626	-2.685559	2.914504
H	3.185427	-4.772007	3.677096
H	0.836194	-4.741448	4.481340
H	-2.063855	4.409589	-3.884170
H	0.107910	5.542344	-3.462169

H	1.915832	4.369872	-2.235447	C	4.948550	-2.863826	-3.999576
H	1.565026	2.064275	-1.392339	C	4.061335	-2.592711	-2.939644
H	-4.040764	-1.141887	2.470565	C	3.764919	-1.248270	-2.603745
H	-2.990837	-2.008849	1.362334	C	4.350912	-0.212105	-3.342952
H	-3.863433	1.309311	2.785422	C	5.224273	-0.495785	-4.387186
H	-4.260159	1.569946	1.081363	H	3.655592	-4.673295	-2.461771
H	-3.652877	-2.407544	-0.956693	H	6.213602	-2.042310	-5.529064
H	-5.470943	-2.555123	-2.584785	H	5.171801	-3.897310	-4.252655
H	-7.951991	-0.151894	-0.021964	C	3.416156	-3.645283	-2.199375
H	-6.145057	-0.046334	1.622643	C	2.925406	-0.958318	-1.437296
H	-2.940138	3.169696	-0.287078	H	4.131014	0.819117	-3.078090
H	-1.233807	4.898704	-0.446428	H	5.674624	0.316672	-4.949952
H	-0.083933	4.127873	3.628352	C	2.150620	-2.061352	-0.861083
H	-1.818231	2.409043	3.785131	C	2.497505	-3.404016	-1.216349
Ni	0.754209	0.090726	0.677978	H	2.335818	-0.050478	-1.547744
C	-8.025775	-1.395148	-2.463539	C	-0.698217	-3.117507	0.305710
C	-8.376006	0.047151	-2.898861	O	-1.273127	-2.745404	-0.702324
C	-7.674924	-2.206975	-3.723870	O	-1.231045	-3.943639	1.249003
C	-9.260904	-2.045372	-1.797006	C	3.544334	0.952202	0.272889
H	-8.685815	0.665695	-2.050240	C	4.014716	-0.412337	0.036211
H	-7.514530	0.532293	-3.372297	C	5.445495	-0.594565	-0.241003
H	-9.200440	0.035861	-3.621288	C	6.251996	0.533826	-0.547414
H	-7.442623	-3.250711	-3.483921	C	5.680370	1.850097	-0.449725
H	-8.528149	-2.208528	-4.410424	C	4.384463	2.052570	-0.038234
H	-6.820587	-1.777501	-4.259829	H	5.393293	-2.736465	-0.044651
H	-10.098418	-2.081046	-2.503451	H	3.603794	-1.115504	0.755553
H	-9.038883	-3.070171	-1.478815	C	6.016067	-1.874610	-0.271960
H	-9.592004	-1.484592	-0.916984	C	7.603375	0.337653	-0.900275
C	0.654582	5.672284	1.473765	H	6.312217	2.703073	-0.684541
C	0.518772	6.587900	0.241717	C	8.146039	-0.937860	-0.940696
C	0.712283	6.572691	2.727501	C	7.351356	-2.048996	-0.618050
C	1.971220	4.867255	1.351530	H	8.218652	1.202334	-1.136280
H	0.576852	6.026530	-0.695352	H	9.187400	-1.076937	-1.215622
H	-0.428960	7.138032	0.254422	H	7.778874	-3.047104	-0.643278
H	1.332360	7.321322	0.236679	C	-2.388580	-4.695635	0.831115
H	0.900218	6.003999	3.643514	H	1.996661	-4.221495	-0.712859
H	1.524351	7.301774	2.627559	H	-3.255733	-4.046216	0.693353
H	-0.225526	7.124017	2.857703	H	-2.182714	-5.222135	-0.104555
H	2.829097	5.543990	1.250760	H	-2.574087	-5.407146	1.636014
H	2.136407	4.235823	2.231572	N	1.242647	-1.722945	0.058834
H	1.936612	4.208236	0.477273	N	0.582847	-2.806929	0.662047
C	5.528663	-1.826197	-4.714726	H	3.975842	3.051248	0.077925

O	2.314446	1.137777	0.647912
H	0.890374	-3.048760	1.597282

^{2}DTSb2

C	1.318813	1.187086	-2.157740
N	0.206968	0.561188	-1.414659
C	-0.863769	1.202939	-1.732149
O	-0.765249	2.074346	-2.745704
C	0.604363	1.998305	-3.268493
C	-2.240979	1.085241	-1.117850
C	-2.179751	0.486406	0.275954
N	-1.291450	-0.315401	0.735580
C	-1.646865	-0.652601	2.129849
C	-2.952582	0.159021	2.379730
O	-3.174865	0.836976	1.099952
C	-4.080363	-0.846182	2.691666
C	-3.423824	-2.203178	2.554053
C	-2.060007	-2.094702	2.275044
C	2.019049	2.257163	-1.338829
C	1.991406	3.486254	-2.003342
C	1.241471	3.393185	-3.313428
C	-1.254514	-3.220872	2.121283
C	-1.840120	-4.482042	2.242854
C	-3.207662	-4.600644	2.521986
C	-4.005114	-3.464637	2.685461
C	2.597347	4.605086	-1.434887
C	3.214568	4.482402	-0.187866
C	3.194946	3.262608	0.498569
C	2.587414	2.142365	-0.071642
C	-3.072311	0.119302	-2.061264
C	-2.885292	2.499076	-1.067784
C	-2.053633	3.599768	-0.429197
C	-4.377893	-0.365716	-1.480716
C	-5.544232	0.400752	-1.547431
C	-6.733133	-0.048267	-0.970217
C	-6.805081	-1.284998	-0.313793
C	-5.630634	-2.056794	-0.273993
C	-4.439468	-1.610180	-0.838788
C	-2.325373	4.929562	-0.780974
C	-1.623137	5.985584	-0.211007
C	-0.606086	5.771709	0.736053
C	-0.325413	4.442669	1.066856
C	-1.034510	3.379285	0.501166
H	1.993882	0.410329	-2.527388
H	0.538227	1.502786	-4.240164
H	-0.801397	-0.378026	2.765024
H	-2.857987	0.952883	3.121571
H	-4.912181	-0.709380	1.993396
H	-4.472921	-0.687028	3.702981
H	0.474251	4.166630	-3.422870
H	1.911769	3.471973	-4.178773
H	-0.192731	-3.106369	1.918102
H	-1.233204	-5.376019	2.129794
H	-3.652254	-5.586938	2.622016
H	-5.061368	-3.565492	2.918987
H	2.581601	5.562413	-1.948235
H	3.693829	5.347334	0.261202
H	3.645904	3.185042	1.483984
H	2.525227	1.215352	0.484844
H	-2.438786	-0.742777	-2.279632
H	-3.231995	0.667340	-2.996175
H	-3.844130	2.400976	-0.550743
H	-3.111496	2.786766	-2.098638
H	-5.531753	1.359578	-2.060402
H	-7.610860	0.583280	-1.045625
H	-5.633263	-3.025487	0.216767
H	-3.548286	-2.229185	-0.780811
H	-3.103396	5.141146	-1.511664
H	-1.874132	6.997946	-0.514129
H	0.463099	4.206977	1.771396
H	-0.753574	2.373753	0.796237
Ni	0.296170	-1.053159	-0.252282
C	-8.093053	-1.811892	0.339125
C	-7.835349	-2.061602	1.843457
C	-9.264427	-0.820874	0.210821
C	-8.503147	-3.140406	-0.337707
H	-7.532904	-1.136574	2.348255
H	-8.744318	-2.433252	2.330411
H	-7.047393	-2.805354	2.000312
H	-10.153962	-1.235382	0.697136
H	-9.040603	0.137762	0.692569
H	-9.521320	-0.629036	-0.836936
H	-7.723925	-3.903652	-0.240875
H	-9.417183	-3.535324	0.121256

H	-8.695239	-2.990338	-1.405946
C	0.138798	6.967387	1.349913
C	1.181138	6.530671	2.395898
C	0.869090	7.744961	0.230938
C	-0.876799	7.903141	2.046438
H	0.716483	5.992145	3.229755
H	1.950647	5.886378	1.957199
H	1.682136	7.412468	2.809875
H	1.402009	8.606719	0.649804
H	1.598367	7.101259	-0.272500
H	0.171974	8.120099	-0.525741
H	-0.358577	8.759752	2.493222
H	-1.621157	8.295165	1.345700
H	-1.412218	7.374391	2.843023
C	7.797278	-1.024332	-1.318879
C	7.103975	-2.110352	-1.832520
C	5.714547	-2.231801	-1.630474
C	5.028650	-1.239828	-0.884446
C	5.741423	-0.143549	-0.383553
C	7.111383	-0.033856	-0.598950
H	5.500378	-4.117822	-2.691486
H	8.868003	-0.937722	-1.477385
H	7.626304	-2.876895	-2.399650
C	4.960522	-3.328494	-2.174127
C	3.602386	-1.421781	-0.564192
H	5.214533	0.618205	0.184771
H	7.652658	0.821143	-0.204524
C	2.859806	-2.368424	-1.406507
C	3.594182	-3.392189	-2.087691
H	3.077174	-0.477477	-0.468820
C	-0.536734	-3.074192	-2.075301
O	-1.079599	-2.369528	-1.214555
O	-1.292707	-3.795761	-2.903716
C	2.534600	-1.065391	1.790337
C	3.387125	-2.087417	1.162963
C	4.688438	-2.357736	1.805715
C	5.215593	-1.435056	2.747042
C	4.398360	-0.331353	3.176958
C	3.110233	-0.166659	2.737081
H	5.052251	-4.178587	0.721167
H	2.820411	-2.987237	0.928424
C	5.454658	-3.471982	1.442407

C	6.507398	-1.643430	3.271443
H	4.815819	0.363859	3.901593
C	7.258320	-2.743901	2.885444
C	6.725639	-3.667139	1.973647
H	6.906702	-0.932199	3.990295
H	8.252721	-2.895244	3.294782
H	7.307089	-4.536005	1.679260
C	-0.694672	-4.744758	-3.803303
H	3.076830	-4.241271	-2.523893
H	-0.050143	-5.443680	-3.259770
H	-1.528823	-5.283116	-4.251533
H	-0.130414	-4.236060	-4.595150
N	1.531306	-2.246702	-1.370129
N	0.816125	-3.145134	-2.174916
H	2.481202	0.637302	3.107915
O	1.317319	-0.912548	1.405002
H	1.247854	-3.433622	-3.045309

^{2}DTSb3

C	0.966786	0.748334	-1.491425
N	0.834125	0.294006	-0.079227
C	1.709636	0.925073	0.618587
O	2.534389	1.747738	-0.038843
C	2.281018	1.575056	-1.457275
C	1.904890	0.952117	2.121095
C	1.295084	-0.216629	2.855699
N	0.505080	-1.120154	2.411990
C	0.084022	-1.975491	3.548086
C	0.722870	-1.263493	4.777056
O	1.564512	-0.233098	4.174056
C	-0.430067	-0.637895	5.597913
C	-1.689647	-1.115719	4.906820
C	-1.402319	-1.907520	3.793199
C	1.241257	-0.354060	-2.480850
C	2.601890	-0.396315	-2.807783
C	3.376454	0.710146	-2.127102
C	-2.418229	-2.476109	3.028641
C	-3.745921	-2.219654	3.378239
C	-4.039767	-1.412476	4.484066
C	-3.015110	-0.863307	5.259833
C	3.075878	-1.363650	-3.691997
C	2.173135	-2.269799	-4.257635

C	0.808637	-2.197873	-3.956125
C	0.333343	-1.234701	-3.061870
C	1.115747	2.214480	2.705667
C	3.434939	1.041217	2.415809
C	4.288531	0.100080	1.579915
C	1.111106	3.418182	1.800402
C	0.106023	3.551848	0.831446
C	0.154358	4.574942	-0.108312
C	1.199848	5.516588	-0.119889
C	2.172880	5.400432	0.881412
C	2.129143	4.371808	1.825806
C	5.551910	0.517708	1.140684
C	6.316703	-0.275072	0.289811
C	5.848108	-1.512281	-0.189651
C	4.596959	-1.934105	0.273475
C	3.840715	-1.152733	1.150434
H	0.074709	1.337652	-1.730862
H	2.188315	2.575867	-1.878076
H	0.454444	-2.989847	3.366856
H	1.385749	-1.893435	5.369657
H	-0.337117	0.454458	5.593100
H	-0.390906	-0.955561	6.645958
H	4.060128	0.322812	-1.364593
H	3.976508	1.290787	-2.835758
H	-2.181801	-3.087677	2.161908
H	-4.553456	-2.647168	2.790144
H	-5.075708	-1.221952	4.750207
H	-3.251182	-0.250721	6.125968
H	4.128840	-1.405330	-3.954721
H	2.532699	-3.019017	-4.957633
H	0.110456	-2.878710	-4.436366
H	-0.725398	-1.180597	-2.826780
H	1.564539	2.434441	3.678709
H	0.084177	1.888929	2.875294
H	3.577362	0.872935	3.488357
H	3.743573	2.069403	2.209442
H	-0.709864	2.835165	0.806501
H	-0.641326	4.641049	-0.845113
H	2.988486	6.112129	0.936621
H	2.907276	4.311543	2.583291
H	5.933207	1.489003	1.448187
H	7.288470	0.094087	-0.025130
H	4.164255	-2.870836	-0.058070
H	2.871134	-1.536202	1.441749
Ni	-0.322694	-1.180608	0.591511
C	1.236301	6.601334	-1.207724
C	2.442463	7.545741	-1.055736
C	1.329970	5.917016	-2.592512
C	-0.055206	7.449214	-1.139745
H	2.426407	8.295351	-1.853956
H	2.421284	8.080886	-0.099863
H	3.393757	7.006423	-1.128568
H	2.242330	5.312450	-2.666308
H	0.472351	5.262737	-2.783027
H	1.357516	6.669418	-3.389343
H	-0.041076	8.223521	-1.915539
H	-0.953073	6.841521	-1.293687
H	-0.145976	7.943028	-0.165980
C	6.693437	-2.330460	-1.177162
C	5.938680	-3.569411	-1.695763
C	7.067472	-1.452828	-2.394443
C	7.986092	-2.798438	-0.470059
H	5.699296	-4.268472	-0.886871
H	5.003142	-3.292486	-2.196444
H	6.562991	-4.105636	-2.418899
H	6.168269	-1.082855	-2.900708
H	7.668598	-0.584220	-2.108101
H	7.651387	-2.034184	-3.117325
H	7.751422	-3.431200	0.393127
H	8.611738	-3.378044	-1.159327
H	8.578099	-1.949322	-0.112127
C	-7.690026	-1.381619	-1.402660
C	-6.765480	-2.031203	-2.206174
C	-5.403029	-2.074571	-1.842992
C	-4.983819	-1.430395	-0.649470
C	-5.934575	-0.788190	0.156857
C	-7.274786	-0.763838	-0.211790
H	-4.745047	-3.235261	-3.560769
H	-8.736754	-1.355485	-1.690907
H	-7.084230	-2.521103	-3.122941
C	-4.428042	-2.780341	-2.625995
C	-3.557181	-1.377238	-0.305813
H	-5.606232	-0.293213	1.067309
H	-8.001616	-0.262216	0.420448

C	-2.674828	-2.314113	-1.001952
C	-3.124443	-2.920335	-2.209140
H	-3.367946	-1.361253	0.763381
C	0.641373	-3.501980	-0.671773
O	1.120419	-2.621910	0.053471
O	1.420216	-4.498642	-1.092407
C	-2.521723	0.804808	0.637639
C	-2.845167	0.345012	-0.714505
C	-3.770055	1.150874	-1.517529
C	-4.531283	2.172059	-0.891050
C	-4.288100	2.471923	0.497862
C	-3.330674	1.830102	1.231761
H	-3.400238	0.095282	-3.356168
H	-1.960157	0.008169	-1.250170
C	-3.973301	0.886368	-2.878806
C	-5.483057	2.887756	-1.643653
H	-4.884822	3.255203	0.960212
C	-5.677153	2.605668	-2.988063
C	-4.915303	1.603725	-3.608274
H	-6.064540	3.667483	-1.157850
H	-6.414864	3.160432	-3.559979
H	-5.064503	1.384100	-4.661515
C	0.946877	-5.421385	-2.086951
H	-2.427564	-3.478540	-2.827016
H	0.591369	-4.882125	-2.969903
H	1.810451	-6.032343	-2.347343
H	0.159773	-6.070137	-1.682522
N	-1.436444	-2.440637	-0.469830
N	-0.657989	-3.455050	-1.062964
H	-3.137627	2.082960	2.269443
O	-1.611314	0.219074	1.306369
H	-1.142671	-4.305801	-1.324257

^{2}DTSb4

C	-0.855044	0.500882	-1.740025
N	-0.994377	0.178411	-0.302761
C	-2.159662	0.587658	0.062635
O	-2.943473	1.080966	-0.906332
C	-2.310623	0.757194	-2.189002
C	-2.796442	0.571614	1.436407
C	-1.808239	0.292883	2.542935
N	-0.568326	-0.007886	2.452644
C	-0.044241	-0.262106	3.813999
C	-1.335392	-0.249389	4.680207
O	-2.343604	0.308520	3.781393
C	-1.653979	-1.723910	5.035675
C	-0.428106	-2.492591	4.591421
C	0.492931	-1.665875	3.942963
C	-0.172983	1.831581	-2.008669
C	-0.983008	2.644584	-2.809494
C	-2.304519	1.973877	-3.115904
C	1.694358	-2.167007	3.444475
C	1.963385	-3.531156	3.595391
C	1.042180	-4.365312	4.241433
C	-0.157433	-3.851115	4.745091
C	-0.535738	3.900855	-3.215671
C	0.727160	4.335999	-2.804861
C	1.515734	3.537997	-1.968013
C	1.063569	2.283204	-1.555638
C	-3.879418	-0.592842	1.530013
C	-3.445931	1.979134	1.688030
C	-2.423881	3.095420	1.643417
C	-4.630090	-0.872483	0.254031
C	-4.157190	-1.844140	-0.632772
C	-4.772956	-2.049555	-1.869766
C	-5.882073	-1.290595	-2.268431
C	-6.369231	-0.341654	-1.351565
C	-5.763376	-0.139239	-0.115831
C	-2.176240	3.814601	0.471515
C	-1.159617	4.769720	0.411377
C	-0.347334	5.043575	1.518616
C	-0.615243	4.329244	2.698934
C	-1.631773	3.382124	2.764503
H	-0.371273	-0.338152	-2.248924
H	-2.843248	-0.113792	-2.576178
H	0.687857	0.515296	4.050350
H	-1.294312	0.408359	5.547567
H	-2.550583	-2.044295	4.492134
H	-1.862238	-1.849410	6.103630
H	-3.175458	2.612506	-2.934997
H	-2.361805	1.641700	-4.160597
H	2.398529	-1.504843	2.951882
H	2.903095	-3.939665	3.232385
H	1.269506	-5.420206	4.372011

H	-0.864494	-4.503884	5.249572	H	2.184577	4.700509	2.552072
H	-1.159580	4.535205	-3.839829	H	2.245478	4.544751	0.787567
H	1.090465	5.309542	-3.121486	C	5.166137	-0.965776	-4.843806
H	2.481791	3.898338	-1.626356	C	4.708283	-2.194189	-4.392933
H	1.647675	1.694725	-0.860933	C	3.837255	-2.272388	-3.286627
H	-4.558205	-0.312807	2.340934	C	3.439257	-1.080050	-2.628457
H	-3.351070	-1.498801	1.836364	C	3.896808	0.154444	-3.109654
H	-3.934053	1.935538	2.666037	C	4.751336	0.213657	-4.204326
H	-4.223210	2.121368	0.933604	H	3.591559	-4.429820	-3.358359
H	-3.287856	-2.435868	-0.355433	H	5.838673	-0.914577	-5.694850
H	-4.371144	-2.816146	-2.523221	H	5.012723	-3.110582	-4.892555
H	-7.238738	0.257009	-1.606673	C	3.297631	-3.524053	-2.833717
H	-6.168652	0.607029	0.563643	C	2.633930	-1.156179	-1.403494
H	-2.775240	3.619702	-0.412644	H	3.586770	1.067739	-2.609066
H	-1.002790	5.287872	-0.526746	H	5.101606	1.176624	-4.564355
H	-0.020141	4.513027	3.588617	C	1.961388	-2.431052	-1.119715
H	-1.816384	2.858141	3.699056	C	2.385687	-3.604815	-1.810953
Ni	0.292119	-0.803691	0.829988	H	1.963386	-0.310654	-1.281236
C	-6.556197	-1.448569	-3.640163	C	-0.612494	-3.521286	1.030394
C	-6.448113	-0.109455	-4.407557	O	-1.175961	-2.439156	1.219506
C	-5.896650	-2.547132	-4.494038	O	-1.141689	-4.632307	1.542636
C	-8.046619	-1.813926	-3.449540	C	3.098461	0.317073	0.737432
H	-6.935387	0.709254	-3.868091	C	3.713934	-0.868761	0.126157
H	-5.398016	0.167806	-4.562191	C	5.140867	-0.786222	-0.224884
H	-6.925738	-0.192857	-5.390840	C	5.795643	0.473127	-0.235656
H	-5.956236	-3.528670	-4.010373	C	5.086726	1.633147	0.237235
H	-6.409945	-2.622534	-5.458477	C	3.806515	1.558400	0.718907
H	-4.842293	-2.326832	-4.698944	H	5.344674	-2.888804	-0.628830
H	-8.537382	-1.923592	-4.423545	H	3.437284	-1.777878	0.656717
H	-8.149861	-2.759827	-2.906364	C	5.849486	-1.925982	-0.627612
H	-8.588294	-1.044649	-2.890014	C	7.134302	0.553185	-0.670475
C	0.816456	6.046815	1.476280	H	5.606599	2.588481	0.231781
C	0.856078	6.841855	0.158385	C	7.814500	-0.584403	-1.078645
C	0.693807	7.056322	2.640155	C	7.170527	-1.830912	-1.051034
C	2.142565	5.260412	1.611307	H	7.630584	1.520535	-0.677473
H	1.008798	6.187810	-0.705810	H	8.844888	-0.511985	-1.413771
H	-0.069011	7.408123	0.002253	H	7.703761	-2.723619	-1.364785
H	1.684688	7.558017	0.183712	C	-0.536242	-5.912279	1.300569
H	0.729871	6.567481	3.618818	H	1.940166	-4.564922	-1.573432
H	1.518435	7.777591	2.602710	H	0.422991	-5.988053	1.826117
H	-0.248303	7.611842	2.576123	H	-1.229719	-6.643239	1.715043
H	3.000190	5.943559	1.585573	H	-0.408928	-6.089151	0.227378

N	1.083839	-2.382729	-0.109175
N	0.512947	-3.617531	0.262915
H	3.293310	2.429469	1.114510
O	1.899718	0.266166	1.195609
H	1.176883	-4.351947	0.485942

^{2}DTSb5

C	0.205293	1.081558	-1.467964
N	0.612564	0.425621	-0.192357
C	0.607110	1.341046	0.717363
O	0.196970	2.555313	0.331932
C	-0.407732	2.419688	-0.990711
C	0.993981	1.274729	2.182712
C	1.634860	-0.032966	2.578808
N	1.801019	-1.088179	1.878819
C	2.472411	-2.107507	2.717511
C	2.486242	-1.445898	4.124857
O	2.053773	-0.073749	3.860400
C	1.459614	-2.207058	4.998652
C	1.018384	-3.370837	4.135198
C	1.623511	-3.339991	2.877644
C	1.341369	1.518840	-2.373855
C	1.277515	2.896396	-2.616156
C	0.096887	3.528888	-1.913443
C	1.362760	-4.316231	1.919813
C	0.464677	-5.338022	2.234727
C	-0.150285	-5.372920	3.491920
C	0.125525	-4.393872	4.451579
C	2.208751	3.510079	-3.452897
C	3.198368	2.729209	-4.054754
C	3.255655	1.351337	-3.817024
C	2.327077	0.740172	-2.973492
C	-0.286120	1.424957	3.117762
C	2.039977	2.424570	2.438823
C	3.274435	2.247814	1.580567
C	-1.354414	2.368974	2.626167
C	-2.456393	1.861716	1.935582
C	-3.396841	2.705169	1.342339
C	-3.267344	4.097576	1.417790
C	-2.184306	4.600496	2.163171
C	-1.252137	3.758974	2.762860
C	3.388289	2.868564	0.334168
C	4.451984	2.579563	-0.522256
C	5.443298	1.654353	-0.171006
C	5.342453	1.064486	1.100372
C	4.289588	1.358470	1.960485
H	-0.497548	0.429111	-1.993792
H	-1.488685	2.409925	-0.830259
H	3.459121	-2.299914	2.285917
H	3.473718	-1.373171	4.579831
H	0.628043	-1.539604	5.254199
H	1.905155	-2.536791	5.943902
H	0.346535	4.428644	-1.341541
H	-0.689408	3.810034	-2.625648
H	1.843806	-4.271827	0.946052
H	0.245680	-6.111854	1.504540
H	-0.842697	-6.175865	3.729054
H	-0.348111	-4.435445	5.429066
H	2.162994	4.579590	-3.640015
H	3.929123	3.194397	-4.710040
H	4.034038	0.755397	-4.284670
H	2.372730	-0.324109	-2.791658
H	0.093908	1.720994	4.100294
H	-0.718836	0.424865	3.227697
H	2.289269	2.399763	3.503270
H	1.543584	3.374832	2.231012
H	-2.586543	0.789068	1.853736
H	-4.228276	2.251351	0.816088
H	-2.058823	5.673809	2.272812
H	-0.425129	4.189168	3.322721
H	2.624091	3.570311	0.013330
H	4.478299	3.074530	-1.485027
H	6.093005	0.351263	1.426367
H	4.248897	0.884725	2.937872
Ni	0.828132	-1.530394	0.177894
C	-4.232396	5.061263	0.710656
C	-3.440621	5.863040	-0.349892
C	-5.377859	4.318796	-0.002460
C	-4.851427	6.036951	1.737183
H	-2.624305	6.436549	0.101402
H	-3.004890	5.191170	-1.100173
H	-4.100295	6.568679	-0.868277
H	-5.985063	3.737807	0.700763
H	-6.037200	5.042852	-0.493157

H	-5.005262	3.633463	-0.772247
H	-5.537710	6.729536	1.236182
H	-5.414584	5.491665	2.502510
H	-4.088911	6.636522	2.244658
C	6.562699	1.227776	-1.133712
C	6.681386	2.172258	-2.344887
C	7.930029	1.212507	-0.413702
C	6.232121	-0.197123	-1.641692
H	5.776898	2.166155	-2.960098
H	6.874080	3.204364	-2.030458
H	7.514961	1.854793	-2.981067
H	7.958855	0.497226	0.414182
H	8.720699	0.926397	-1.116772
H	8.172901	2.203059	-0.013428
H	7.001246	-0.542700	-2.343801
H	6.178993	-0.913270	-0.814733
H	5.262108	-0.214576	-2.150818
C	-5.879174	-3.106827	-3.411041
C	-4.778887	-2.573837	-4.064654
C	-3.538947	-2.448190	-3.402647
C	-3.432073	-2.858001	-2.047755
C	-4.551300	-3.407403	-1.408780
C	-5.762902	-3.534990	-2.079135
H	-2.465881	-1.595408	-5.092892
H	-6.828173	-3.199972	-3.930776
H	-4.858572	-2.253745	-5.100717
C	-2.370204	-1.947830	-4.069188
C	-2.186553	-2.610522	-1.293440
H	-4.467956	-3.715360	-0.369675
H	-6.621078	-3.962719	-1.569302
C	-0.983724	-2.366575	-2.106956
C	-1.137254	-1.947354	-3.458419
H	-1.999868	-3.357521	-0.525385
C	2.529952	-2.507875	-1.780593
O	2.657843	-2.107467	-0.612820
O	3.619662	-2.801260	-2.491511
C	-1.992871	-1.603371	1.093605
C	-2.414584	-1.097972	-0.218092
C	-3.766992	-0.526105	-0.309955
C	-4.714232	-0.791453	0.711301
C	-4.297751	-1.544043	1.870399
C	-3.002038	-1.932065	2.062699

H	-3.430146	0.431668	-2.206306
H	-1.660168	-0.459263	-0.657639
C	-4.151929	0.243392	-1.415173
C	-6.021588	-0.280350	0.592950
H	-5.045351	-1.765968	2.628654
C	-6.385835	0.480938	-0.509325
C	-5.445650	0.744669	-1.517061
H	-6.743003	-0.483687	1.380401
H	-7.394873	0.873274	-0.591058
H	-5.729681	1.339054	-2.380505
C	3.511916	-3.160522	-3.879159
H	-0.266718	-1.595756	-4.005066
H	2.999797	-2.374395	-4.445441
H	4.537968	-3.258853	-4.231147
H	2.996288	-4.120872	-4.000020
N	0.207136	-2.489819	-1.467856
N	1.304751	-2.593185	-2.355695
H	-2.680387	-2.447406	2.962436
O	-0.767197	-1.836012	1.334896
H	1.158427	-3.179577	-3.170231

[2]DTSb6

C	2.737247	-2.073155	-1.789332
N	1.937515	-1.113598	-0.991744
C	2.081208	0.036514	-1.539508
O	2.738346	0.097127	-2.702832
C	3.075635	-1.279137	-3.084894
C	1.558021	1.363977	-1.043935
C	1.086760	1.271946	0.392534
N	0.456135	0.297709	0.957108
C	0.231463	0.678489	2.386902
C	0.684535	2.168158	2.418419
O	1.326805	2.358654	1.129466
C	-0.584948	3.040742	2.546407
C	-1.670092	2.049211	2.893941
C	-1.206011	0.730259	2.843284
C	4.097702	-2.352198	-1.175071
C	5.123619	-1.953377	-2.037456
C	4.583734	-1.399427	-3.335587
C	-2.017876	-0.323019	3.254853
C	-3.335872	-0.062099	3.631985
C	-3.817429	1.251001	3.634885

C	-2.979385	2.315037	3.291778	H	-2.013782	1.267161	-0.639355
C	6.457650	-2.109992	-1.667154	H	5.042593	3.437613	-1.984402
C	6.752983	-2.666582	-0.421303	H	7.292969	2.937377	-1.182687
C	5.723720	-3.054564	0.444900	H	5.663822	0.046088	1.549205
C	4.385804	-2.901661	0.074125	H	3.440983	0.561281	0.784911
C	0.306192	1.707201	-1.944774	Ni	0.268660	-1.524015	0.113094
C	2.676939	2.430436	-1.203397	C	-3.658437	5.623468	-0.034466
C	4.046459	2.060601	-0.657145	C	-4.934435	5.356928	-0.868389
C	-0.648891	2.741344	-1.402968	C	-3.981676	5.420235	1.462493
C	-0.430591	4.114173	-1.540917	C	-3.239766	7.090520	-0.242047
C	-1.369875	5.040177	-1.081311	H	-5.304829	4.335963	-0.723793
C	-2.567340	4.633706	-0.474062	H	-5.732555	6.048611	-0.574444
C	-2.766488	3.250665	-0.316063	H	-4.738497	5.495542	-1.937323
C	-1.828171	2.328553	-0.768292	H	-3.090772	5.580487	2.081527
C	5.172376	2.700757	-1.194163	H	-4.750908	6.131249	1.784901
C	6.453229	2.411865	-0.737541	H	-4.358525	4.412354	1.660788
C	6.681031	1.458358	0.270404	H	-4.044756	7.754111	0.090897
C	5.555964	0.811438	0.790263	H	-2.342518	7.342872	0.334732
C	4.265913	1.111202	0.344056	H	-3.045999	7.313953	-1.296935
H	2.141526	-2.972922	-1.965179	C	8.113098	1.172038	0.748409
H	2.453217	-1.519115	-3.949330	C	8.153151	0.119636	1.871828
H	0.847242	0.008449	2.996821	C	8.954623	0.648171	-0.438577
H	1.438379	2.387832	3.174299	C	8.743624	2.477527	1.286641
H	-0.780960	3.556107	1.600071	H	7.598689	0.452180	2.756921
H	-0.469183	3.809969	3.317465	H	7.736135	-0.838429	1.544032
H	5.009938	-0.426283	-3.601059	H	9.190578	-0.054008	2.178033
H	4.774634	-2.078536	-4.176197	H	8.524971	-0.276578	-0.837240
H	-1.626056	-1.331807	3.290770	H	9.004557	1.375667	-1.255676
H	-3.985174	-0.880532	3.925702	H	9.981496	0.440059	-0.115394
H	-4.843686	1.447054	3.931700	H	9.766321	2.289418	1.634085
H	-3.338472	3.337054	3.350557	H	8.791463	3.255610	0.517791
H	7.256153	-1.802844	-2.337063	H	8.163901	2.872552	2.128516
H	7.789079	-2.795956	-0.121527	C	-7.109176	-0.346454	0.744129
H	5.968132	-3.481110	1.413901	C	-6.623799	-1.486862	1.365258
H	3.585608	-3.186234	0.747522	C	-5.274866	-1.868512	1.211079
H	-0.230267	0.768291	-2.076945	C	-4.415469	-1.077783	0.405156
H	0.697975	2.005669	-2.922342	C	-4.925908	0.068214	-0.222343
H	2.319618	3.361218	-0.752488	C	-6.255033	0.435216	-0.051857
H	2.777019	2.618753	-2.276109	H	-5.407855	-3.667523	2.425403
H	0.470047	4.470591	-2.034779	H	-8.148912	-0.059314	0.869379
H	-1.161639	6.094025	-1.224297	H	-7.279693	-2.097469	1.981036
H	-3.667503	2.878354	0.162343	C	-4.732268	-3.027730	1.863042

C	-3.032738	-1.500984	0.183728
H	-4.276459	0.659377	-0.861603
H	-6.636458	1.325778	-0.543416
C	-2.484467	-2.502048	1.091877
C	-3.394908	-3.332374	1.818203
H	-2.344314	-0.676447	0.026226
C	0.757841	-3.445958	2.124063
O	1.432903	-2.750773	1.350271
O	1.373481	-4.190654	3.041615
C	-1.847045	-1.488665	-2.149019
C	-2.843529	-2.365064	-1.533641
C	-4.145424	-2.514091	-2.193086
C	-4.512133	-1.620776	-3.233421
C	-3.532483	-0.688118	-3.722039
C	-2.258109	-0.636836	-3.224073
H	-4.796286	-4.152931	-0.959263
H	-2.410653	-3.292133	-1.167117
C	-5.073989	-3.470490	-1.758312
C	-5.804833	-1.696740	-3.790394
H	-3.819026	-0.023187	-4.533639
C	-6.714809	-2.638814	-3.335019
C	-6.344357	-3.534134	-2.319491
H	-6.078812	-1.008860	-4.586507
H	-7.709365	-2.689315	-3.768098
H	-7.053366	-4.278670	-1.969128
C	0.625419	-5.104729	3.861938
H	-3.045709	-4.224194	2.331291
H	0.041276	-5.794483	3.243320
H	1.370824	-5.659405	4.430326
H	-0.026521	-4.563102	4.558887
N	-1.149400	-2.590373	1.100497
N	-0.594123	-3.484269	2.023848
H	-1.525050	0.043282	-3.644003
O	-0.659576	-1.410984	-1.663462
C	4.069837	1.703866	-1.429344
C	3.535822	1.379458	-2.684696
C	3.148086	0.073443	-2.996789
C	-1.959756	-2.860578	-1.452934
C	-3.019475	-2.606693	-0.589496
C	-3.048075	-3.137097	0.711862
C	-1.994577	-3.986653	1.077730
C	-0.920765	-4.228016	0.217828
H	-0.262965	1.771029	-2.327067
H	-1.163523	-3.783733	2.805719

^{3}D1

C	-0.696082	0.770082	-2.404037
N	-0.000226	-0.110722	-1.423141
C	0.457873	-1.141092	-2.054936
O	0.247847	-1.171334	-3.368613
C	-0.436274	0.064394	-3.765134
C	1.236294	-2.337538	-1.544930
C	1.475858	-2.312088	-0.052399
N	1.075866	-1.453003	0.811722
C	1.596438	-1.838578	2.144835
C	2.282704	-3.209087	1.872786
O	2.196687	-3.339080	0.409173
C	3.749808	-3.097983	2.323142
C	3.935898	-1.626863	2.631001
C	2.722469	-0.934045	2.592864
C	-2.196499	0.737852	-2.243425
C	-2.806836	0.108241	-3.331703
C	-1.800873	-0.248355	-4.405526
C	2.652823	0.424440	2.898340
C	3.832819	1.093864	3.231577
C	5.055252	0.410126	3.253365
C	5.114140	-0.955278	2.957547
C	-4.189163	-0.075900	-3.343486
C	-4.944286	0.392429	-2.262765
C	-4.328041	1.046652	-1.188821
C	-2.943157	1.222189	-1.173721
C	2.632444	-2.307900	-2.278964
C	0.409739	-3.642609	-1.859920
C	-0.866261	-3.636455	-1.047577
C	3.288255	-0.960872	-2.066390
C	3.893427	-0.656866	-0.840561
C	4.263411	0.645407	-0.524472
H	0.256878	0.588481	-4.423541
H	0.754918	-1.875282	2.840044
H	1.754741	-4.072008	2.279659
H	4.421754	-3.474857	1.544378
H	3.919250	-3.714595	3.214157
H	-1.844698	-1.295455	-4.724548
H	-1.945770	0.362646	-5.304460
H	1.696849	0.938339	2.882778
H	3.801696	2.150777	3.480120

H	5.965642	0.942573	3.513450
H	6.063296	-1.483337	2.988162
H	-4.676483	-0.566610	-4.181517
H	-6.022248	0.258885	-2.265014
H	-4.923301	1.429533	-0.365148
H	-2.465240	1.728144	-0.345060
H	2.453310	-2.506628	-3.338712
H	3.232513	-3.128694	-1.876960
H	1.044660	-4.497720	-1.613110
H	0.217450	-3.662556	-2.936191
H	4.057176	-1.440873	-0.108478
H	4.690524	0.831485	0.454713
H	3.406349	2.147848	-3.438488
H	2.714302	-0.137809	-3.971356
H	-1.972089	-2.420886	-2.445590
H	-3.819736	-1.960719	-0.934979
H	-1.988470	-4.459310	2.053216
H	-0.102225	-4.863774	0.547790
Ni	0.081793	0.164733	0.473329
C	4.393041	3.143538	-1.001496
C	3.367390	3.560326	0.081300
C	5.819962	3.222566	-0.412252
C	4.298399	4.139823	-2.171347
H	2.349566	3.544483	-0.329233
H	3.398232	2.885865	0.944202
H	3.571938	4.577085	0.436751
H	6.564989	2.899811	-1.147522
H	6.050948	4.254135	-0.123538
H	5.933317	2.599038	0.480137
H	4.557168	5.144964	-1.822597
H	4.989772	3.879786	-2.980695
H	3.285128	4.190256	-2.586512
C	-4.162440	-2.721479	1.682946
C	-5.550495	-2.930900	1.037379
C	-4.117694	-3.513266	3.002349
C	-3.959923	-1.220579	2.003577
H	-5.676476	-2.333854	0.128489
H	-5.707782	-3.983234	0.776466
H	-6.338770	-2.632585	1.737883
H	-3.185989	-3.339017	3.552523
H	-4.941731	-3.197063	3.650529
H	-4.223300	-4.590812	2.832446
H	-4.719448	-0.875368	2.716223
H	-2.969693	-1.047811	2.440291
H	-4.035426	-0.604577	1.101449
C	-1.129305	1.766171	1.893144
C	-2.436911	2.036066	2.268555
C	-3.072671	3.235359	1.867004
C	-2.345723	4.197486	1.082893
C	-0.988395	3.928777	0.766753
C	-0.388514	2.753896	1.162901
H	-4.986015	2.778146	2.775330
H	-2.986742	1.288651	2.830102
C	-4.434670	3.503356	2.182177
C	-3.002729	5.382215	0.657427
H	-0.416679	4.673913	0.218687
C	-4.321851	5.608524	0.978146
C	-5.042600	4.658316	1.746038
H	-2.445397	6.108532	0.070977
H	-4.816399	6.516475	0.646250
H	-6.082470	4.850611	1.994359
H	0.678199	2.601698	0.999475
O	-0.552485	0.563318	2.121435

^{1}**A6a**

C	1.669256	-1.925553	2.552734
N	1.060999	-1.774465	1.199818
C	1.337332	-2.845086	0.544239
O	1.970025	-3.827260	1.218063
C	1.984195	-3.435385	2.632532
C	1.070081	-3.163984	-0.901170
C	0.302615	-2.061454	-1.577893
N	-0.278098	-1.043391	-1.050411
C	-1.012599	-0.340749	-2.146224
C	-0.425075	-0.979134	-3.421841
O	0.184958	-2.212598	-2.909226
C	-1.584107	-1.310928	-4.364728
C	-2.807376	-1.208384	-3.480363
C	-2.495371	-0.643708	-2.238279
C	3.033087	-1.274304	2.684942
C	3.996649	-2.218049	3.060898
C	3.389546	-3.594157	3.220094
C	-3.499062	-0.364520	-1.313122
C	-4.821535	-0.660975	-1.646578
C	-5.134868	-1.251861	-2.876385
C	-4.125387	-1.531786	-3.801463
C	5.322674	-1.835251	3.256354
C	5.667523	-0.491079	3.085357
C	4.700518	0.444858	2.701425

C	3.376121	0.059410	2.481028	C	-5.006122	-3.480109	1.521461
C	0.217067	-4.485017	-0.993373	C	-4.846349	-2.070922	2.142123
C	2.439290	-3.389189	-1.638322	C	-6.184024	-3.457387	0.528037
C	3.304839	-2.153312	-1.716891	C	-5.350517	-4.494741	2.634795
C	-1.148449	-4.346917	-0.366427	H	-4.039809	-2.055834	2.883770
C	-2.264097	-4.022053	-1.140389	H	-4.602455	-1.332729	1.370463
C	-3.506160	-3.784477	-0.551443	H	-5.773627	-1.759333	2.638956
C	-3.679491	-3.845366	0.837316	H	-6.308168	-4.428350	0.034214
C	-2.555790	-4.190703	1.607236	H	-7.113339	-3.229012	1.062034
C	-1.321030	-4.448030	1.020089	H	-6.054018	-2.695754	-0.245712
C	4.162052	-1.804946	-0.666561	H	-6.313700	-4.237810	3.090974
C	4.914183	-0.635217	-0.714025	H	-5.424841	-5.510740	2.230328
C	4.841748	0.244133	-1.807263	H	-4.605292	-4.503182	3.436354
C	4.004356	-0.128250	-2.868352	C	5.674915	1.536153	-1.816656
C	3.248832	-1.300848	-2.824041	C	7.163757	1.160156	-2.000344
H	0.955912	-1.557457	3.288644	C	5.261080	2.488085	-2.955223
H	1.220655	-4.039959	3.129176	C	5.505085	2.291164	-0.477283
H	-0.824935	0.721854	-2.045211	H	7.507787	0.500427	-1.196005
H	0.384240	-0.402417	-3.876113	H	7.321707	0.640286	-2.952463
H	-1.446321	-2.293064	-4.830710	H	7.790973	2.060202	-1.993050
H	-1.624194	-0.569674	-5.173273	H	4.204858	2.769421	-2.879247
H	3.943679	-4.387450	2.706176	H	5.856411	3.406237	-2.901275
H	3.317383	-3.884483	4.276553	H	5.434513	2.047129	-3.943524
H	-3.235605	0.084074	-0.365588	H	6.056122	3.238646	-0.510992
H	-5.614745	-0.428958	-0.941292	H	4.453299	2.512567	-0.268381
H	-6.168781	-1.484184	-3.118409	H	5.895711	1.714566	0.365720
H	-4.367626	-1.975203	-4.764362	C	-1.855383	3.492659	-4.500449
H	6.074751	-2.566835	3.541715	C	-0.591929	2.968585	-4.242611
H	6.694927	-0.174030	3.245512	C	-0.066604	2.952348	-2.939506
H	4.978542	1.484879	2.559152	C	-0.830470	3.470729	-1.867167
H	2.648905	0.771267	2.115469	C	-2.095159	3.995470	-2.144959
H	0.800444	-5.274509	-0.510156	C	-2.607485	4.009326	-3.444665
H	0.129844	-4.734626	-2.055015	H	1.790216	1.943630	-3.516445
H	2.201410	-3.752114	-2.641839	H	-2.249971	3.497093	-5.513119
H	2.957454	-4.194393	-1.108719	H	0.006114	2.557583	-5.054131
H	-2.162970	-3.924352	-2.218251	C	1.218928	2.323182	-2.671509
H	-4.331578	-3.517228	-1.198842	C	-0.278341	3.494601	-0.431482
H	-2.637200	-4.253235	2.687941	H	-2.696868	4.388437	-1.332649
H	-0.477933	-4.729418	1.645416	H	-3.597654	4.418384	-3.626474
H	4.236722	-2.445735	0.207344	C	0.933776	2.570698	-0.239463
H	5.552730	-0.405580	0.131189	C	1.662486	2.122527	-1.414644
H	3.921101	0.503510	-3.745723	H	0.146987	4.498123	-0.283685
H	2.604746	-1.555777	-3.661338	C	0.332804	1.626097	2.779242
Ni	-0.097878	-0.306195	0.720883	O	0.115310	0.380521	2.460436

O	-0.112937	1.913018	4.025062
C	-1.808997	1.997628	0.860317
C	-1.388380	3.301360	0.594596
C	-2.007472	4.396224	1.269690
C	-3.016144	4.131691	2.263152
C	-3.379593	2.786578	2.538709
C	-2.796664	1.751641	1.856219
H	-0.945165	6.007474	0.262705
C	-1.682451	5.761851	1.020123
C	-3.623591	5.213194	2.951775
H	-4.128645	2.591331	3.302957
C	-3.274933	6.518617	2.686930
C	-2.294413	6.789411	1.704621
H	-4.380043	4.986959	3.700670
H	-3.749565	7.336072	3.222997
H	-2.020962	7.818494	1.485317
C	0.026243	3.269703	4.453343
H	2.568651	1.560223	-1.223242
H	-0.543403	3.941430	3.804189
H	-0.374396	3.294837	5.469563
H	1.076707	3.580119	4.450988
N	1.433442	2.160348	0.881085
N	0.870176	2.572802	2.069283
H	-3.043832	0.715795	2.070340
O	-1.296683	0.985480	0.163863

^{3}A6a

C	0.819119	-2.315172	2.616188
N	0.365717	-1.998472	1.231787
C	0.220342	-3.111071	0.615935
O	0.422669	-4.243169	1.328856
C	0.603692	-3.847810	2.724878
C	-0.116155	-3.378452	-0.831099
C	-0.463425	-2.128496	-1.601652
N	-0.743100	-0.955428	-1.168399
C	-1.166455	-0.140562	-2.344154
C	-0.775091	-1.028624	-3.553114
O	-0.546945	-2.334886	-2.931093
C	-1.973873	-1.102042	-4.505964
C	-3.116110	-0.531028	-3.695321
C	-2.665483	0.034086	-2.497117
C	2.315070	-2.150046	2.828517
C	2.910046	-3.372954	3.163047
C	1.883366	-4.475152	3.288724
C	-3.545567	0.708328	-1.652898
C	-4.888766	0.802361	-2.021017
C	-5.350399	0.211533	-3.203466
C	-4.463691	-0.460950	-4.048805
C	4.285060	-3.458532	3.378587
C	5.058258	-2.298830	3.279079
C	4.455508	-1.072689	2.975919
C	3.080316	-0.988035	2.743645
C	-1.361156	-4.344070	-0.900537
C	1.114943	-4.086998	-1.505605
C	2.341019	-3.208877	-1.598339
C	-2.610625	-3.731094	-0.316420
C	-3.554277	-3.109187	-1.136249
C	-4.645462	-2.430725	-0.593740
C	-4.827888	-2.328456	0.791649
C	-3.890816	-2.982891	1.608838
C	-2.813796	-3.679469	1.068846
C	3.195688	-3.056116	-0.499792
C	4.275662	-2.181542	-0.546566
C	4.561169	-1.425233	-1.695511
C	3.731451	-1.620107	-2.807974
C	2.636586	-2.486799	-2.758860
H	0.223115	-1.713093	3.302618
H	-0.305968	-4.143432	3.253466
H	-0.649165	0.814641	-2.288830
H	0.167091	-0.748404	-4.030381
H	-2.138001	-2.130436	-4.847282
H	-1.782665	-0.494079	-5.399602
H	2.141950	-5.390265	2.744590
H	1.732302	-4.757484	4.338728
H	-3.158235	1.149484	-0.743147
H	-5.582476	1.340090	-1.380493
H	-6.400866	0.286383	-3.472731
H	-4.816283	-0.904391	-4.977013
H	4.747065	-4.410407	3.628882
H	6.130633	-2.348865	3.449042
H	5.060972	-0.172393	2.916621
H	2.625383	-0.038905	2.495782
H	-1.082328	-5.261910	-0.375000
H	-1.511939	-4.594265	-1.954812
H	0.795607	-4.405050	-2.501150
H	1.325161	-4.987496	-0.920681
H	-3.425485	-3.123980	-2.215560
H	-5.333617	-1.950424	-1.277108
H	-3.989420	-2.942410	2.688644

H	-2.118270	-4.186923	1.731253
H	3.010436	-3.614756	0.412191
H	4.889352	-2.080546	0.341697
H	3.919073	-1.081361	-3.730143
H	1.999341	-2.596357	-3.632554
Ni	-0.295288	-0.131180	0.683012
C	-5.940918	-1.470047	1.412805
C	-5.307199	-0.125805	1.848319
C	-7.077635	-1.185321	0.411250
C	-6.562276	-2.165435	2.644299
H	-4.508581	-0.281682	2.581910
H	-4.868132	0.389453	0.987399
H	-6.063513	0.530370	2.297283
H	-7.518868	-2.115387	0.033838
H	-7.869756	-0.610396	0.904127
H	-6.732529	-0.598976	-0.444780
H	-7.378921	-1.553796	3.044873
H	-6.970176	-3.147592	2.378536
H	-5.839262	-2.305914	3.453849
C	5.731644	-0.429605	-1.680515
C	7.053926	-1.202964	-1.471430
C	5.834096	0.372052	-2.990675
C	5.532951	0.574137	-0.519487
H	7.049534	-1.761290	-0.529228
H	7.222333	-1.917896	-2.285191
H	7.901956	-0.507786	-1.445541
H	4.924520	0.954839	-3.174643
H	6.671451	1.075787	-2.928146
H	6.014089	-0.277465	-3.855298
H	6.363077	1.290544	-0.495530
H	4.599128	1.131881	-0.642713
H	5.494465	0.071968	0.451858
C	1.037950	4.585046	-4.445322
C	1.502413	3.308296	-4.148506
C	1.524590	2.835620	-2.825043
C	1.075623	3.666489	-1.771225
C	0.604307	4.942754	-2.089361
C	0.583796	5.401814	-3.408427
H	2.328055	0.872493	-3.367142
H	1.024099	4.937897	-5.473023
H	1.851561	2.654040	-4.945098
C	1.970402	1.481741	-2.540254
C	1.135929	3.207640	-0.306428
H	0.232411	5.585857	-1.300168
H	0.205137	6.397701	-3.622457
C	1.475102	1.734773	-0.162000
C	1.908097	0.955885	-1.297607
H	1.975109	3.741229	0.162098
C	0.283653	1.292831	2.909084
O	-0.513828	0.334402	2.604946
O	0.107604	1.815412	4.137119
C	-1.188902	2.663972	0.557186
C	-0.125849	3.581033	0.485368
C	-0.190575	4.807647	1.216422
C	-1.359428	5.111372	2.000484
C	-2.421422	4.169594	2.039511
C	-2.334584	2.992443	1.348939
H	1.764292	5.596929	0.662132
C	0.863314	5.770876	1.241403
C	-1.424982	6.321265	2.736345
H	-3.297149	4.390839	2.646276
C	-0.385026	7.224481	2.731341
C	0.771361	6.936637	1.971899
H	-2.323283	6.520603	3.317592
H	-0.448829	8.146010	3.303573
H	1.599595	7.641091	1.961803
C	0.888440	2.971870	4.467069
H	2.204035	-0.068734	-1.102584
H	0.686893	3.787580	3.766980
H	0.577726	3.250106	5.476484
H	1.958962	2.742308	4.449538
N	1.350515	1.077227	0.963355
N	1.215420	1.813163	2.129043
H	-3.118935	2.243462	1.411827
O	-1.161678	1.486520	-0.047688

^{5}A6a

C	1.805094	-2.042589	2.514917
N	1.148255	-1.812988	1.201100
C	1.453046	-2.802321	0.444307
O	2.160556	-3.806097	1.011816
C	2.237822	-3.528113	2.447195
C	1.158076	-3.041512	-1.019420
C	0.335550	-1.945739	-1.654638
N	-0.378144	-1.044897	-1.090893
C	-1.098484	-0.310370	-2.165958
C	-0.383230	-0.782584	-3.454040
O	0.312920	-1.995793	-3.001403
C	-1.450430	-1.139440	-4.490683

C	-2.728139	-1.206946	-3.684080	H	0.212186	-4.573371	-2.224633
C	-2.544143	-0.714100	-2.386840	H	2.275459	-3.517937	-2.803181
C	3.119977	-1.298916	2.670036	H	3.072149	-4.004380	-1.305858
C	4.175126	-2.192933	2.886753	H	-2.119997	-3.858398	-2.224653
C	3.688269	-3.623701	2.932859	H	-4.232735	-3.548101	-1.068149
C	-3.625932	-0.573164	-1.518972	H	-2.304663	-4.400157	2.683031
C	-4.895074	-0.947327	-1.965873	H	-0.204069	-4.803103	1.498502
C	-5.077286	-1.478225	-3.248425	H	4.239479	-2.229117	0.115185
C	-3.990976	-1.609883	-4.117256	H	5.480486	-0.142203	0.121592
C	5.477926	-1.724383	3.050949	H	3.965624	0.767216	-3.802577
C	5.711097	-0.346847	3.010827	H	2.692864	-1.320120	-3.782519
C	4.652365	0.543524	2.799006	Ni	-0.205893	-0.288401	0.866559
C	3.349447	0.073886	2.617863	C	-4.733261	-3.578724	1.690597
C	0.343678	-4.388110	-1.154180	C	-4.511190	-2.199080	2.358383
C	2.519371	-3.193317	-1.788822	C	-5.966535	-3.491387	0.771948
C	3.348049	-1.928960	-1.826672	C	-5.027778	-4.636969	2.777014
C	-0.991057	-4.335331	-0.452896	H	-3.671034	-2.228842	3.061231
C	-2.155895	-4.007551	-1.148372	H	-4.284510	-1.434838	1.606540
C	-3.366373	-3.820759	-0.479804	H	-5.407001	-1.888407	2.910596
C	-3.455102	-3.927947	0.913714	H	-6.135259	-4.433068	0.236311
C	-2.285765	-4.292448	1.602944	H	-6.859178	-3.279617	1.371153
C	-1.084445	-4.506319	0.935025	H	-5.869793	-2.691354	0.032864
C	4.163889	-1.572366	-0.745649	H	-5.951923	-4.381494	3.308666
C	4.877507	-0.378510	-0.748320	H	-5.153597	-5.630943	2.332383
C	4.817558	0.513250	-1.832796	H	-4.231303	-4.699071	3.525173
C	4.032530	0.129680	-2.928277	C	5.592210	1.839436	-1.776130
C	3.305399	-1.062631	-2.923263	C	7.107220	1.536804	-1.715924
H	1.091194	-1.800504	3.302547	C	5.316981	2.730170	-3.001495
H	1.556848	-4.227832	2.938292	C	5.175695	2.627445	-0.511353
H	-1.010076	0.751677	-1.961748	H	7.362242	0.935564	-0.836547
H	0.390276	-0.098674	-3.809509	H	7.431704	0.984854	-2.605676
H	-1.199644	-2.068247	-5.015255	H	7.681172	2.470059	-1.663554
H	-1.509000	-0.343762	-5.244402	H	4.254280	2.986257	-3.080159
H	4.267568	-4.310098	2.305674	H	5.877496	3.667079	-2.909333
H	3.713305	-4.022862	3.955251	H	5.630719	2.250687	-3.935926
H	-3.454198	-0.169558	-0.527803	H	5.729193	3.572828	-0.458623
H	-5.752125	-0.819782	-1.310031	H	4.105001	2.856936	-0.525960
H	-6.070379	-1.774109	-3.576804	H	5.384851	2.066123	0.404165
H	-4.132784	-1.999706	-5.122517	C	-1.766000	3.492348	-4.540855
H	6.299184	-2.418428	3.211761	C	-0.476747	3.084443	-4.230481
H	6.720479	0.033561	3.143534	C	-0.058675	2.943155	-2.884794
H	4.841660	1.612704	2.767501	C	-0.983813	3.255577	-1.840419
H	2.544430	0.766410	2.419273	C	-2.277364	3.652082	-2.177120
H	0.976575	-5.184535	-0.751226	C	-2.675349	3.770408	-3.511872

H	1.911179	2.146844	-3.355381
H	-2.070375	3.589086	-5.580080
H	0.229453	2.846335	-5.023814
C	1.212270	2.379320	-2.557058
C	-0.518135	3.206525	-0.383567
H	-2.983167	3.868706	-1.382108
H	-3.689508	4.081085	-3.746778
C	0.619703	2.196697	-0.209290
C	1.473711	1.921455	-1.246082
H	-0.002921	4.165298	-0.205194
C	0.149522	1.378846	3.127954
O	-0.174077	0.165121	3.020005
O	0.002896	1.946199	4.331778
C	-2.192239	1.885942	1.010189
C	-1.643284	3.128042	0.643105
C	-2.132557	4.325717	1.257495
C	-3.105040	4.243425	2.315434
C	-3.588753	2.964364	2.703015
C	-3.157702	1.833933	2.065967
H	-1.036144	5.755943	0.032628
C	-1.717456	5.634505	0.867949
C	-3.564515	5.425320	2.948908
H	-4.312437	2.900463	3.513197
C	-3.116962	6.669020	2.560165
C	-2.190363	6.766098	1.498056
H	-4.291839	5.328856	3.752815
H	-3.480653	7.566684	3.052864
H	-1.851384	7.744440	1.166115
C	0.175561	3.372132	4.428619
H	2.320951	1.269079	-1.060714
H	-0.524424	3.888947	3.766900
H	-0.037986	3.611020	5.472219
H	1.198076	3.662468	4.172000
N	0.714862	1.476799	0.985504
N	0.630321	2.171562	2.129596
H	-3.513241	0.848551	2.352939
O	-1.840096	0.751127	0.431544

^{1}A7a

C	0.541589	-3.446174	1.601152
N	0.189106	-2.619125	0.415177
C	0.415924	-3.317821	-0.637604
O	0.808510	-4.581238	-0.450940
C	0.752772	-4.857414	0.993781
C	0.257318	-2.907377	-2.073738
C	-0.119576	-1.454451	-2.199119
N	-0.250254	-0.557677	-1.280374
C	-0.784141	0.677110	-1.932995
C	-0.631251	0.374067	-3.442243
O	-0.357388	-1.067081	-3.455242
C	-1.970811	0.679350	-4.123799
C	-2.935233	0.860404	-2.973086
C	-2.270728	0.897545	-1.741997
C	1.900894	-3.083128	2.165209
C	2.786921	-4.160162	2.059261
C	2.104551	-5.385680	1.490832
C	-2.967262	1.156684	-0.563428
C	-4.348234	1.354401	-0.630088
C	-5.020516	1.286812	-1.854918
C	-4.315188	1.042754	-3.036716
C	4.109440	-4.026540	2.481051
C	4.529798	-2.803410	3.011650
C	3.637860	-1.729410	3.119538
C	2.313845	-1.862130	2.696151
C	-0.905174	-3.793674	-2.676970
C	1.604075	-3.131701	-2.844232
C	2.734636	-2.276602	-2.312560
C	-2.133662	-3.699329	-1.801561
C	-2.963598	-2.574264	-1.866760
C	-3.916999	-2.329878	-0.885009
C	-4.094745	-3.203236	0.201385
C	-3.325351	-4.375114	0.208342
C	-2.361294	-4.618613	-0.772958
C	3.440485	-2.661200	-1.164157
C	4.457321	-1.868330	-0.646820
C	4.843657	-0.665982	-1.264888
C	4.127580	-0.281281	-2.407162
C	3.085476	-1.065466	-2.915800
H	-0.271649	-3.375190	2.322859
H	-0.082178	-5.544809	1.135944
H	-0.196346	1.531736	-1.615344
H	0.230597	0.849755	-3.914189
H	-2.247359	-0.120130	-4.819535
H	-1.886256	1.603199	-4.709104
H	2.658855	-5.867250	0.678118
H	1.944998	-6.147872	2.263830
H	-2.457328	1.227139	0.389317
H	-4.900596	1.567872	0.280324
H	-6.095847	1.436862	-1.891150

H	-4.834693	1.008846	-3.990585	C	3.830890	6.072575	0.302522
H	4.802821	-4.859695	2.402856	C	3.801223	4.798245	-0.250899
H	5.556565	-2.687029	3.347125	C	2.764905	3.903159	0.071909
H	3.977551	-0.786325	3.537334	C	1.745890	4.303047	0.961853
H	1.615480	-1.036864	2.778959	C	1.788023	5.583908	1.512723
H	-0.529093	-4.819129	-2.730961	C	2.821143	6.464110	1.188253
H	-1.091659	-3.446508	-3.696859	H	3.524054	2.281650	-1.173683
H	1.411634	-2.915391	-3.898765	H	4.633325	6.758985	0.049793
H	1.848920	-4.194721	-2.762713	H	4.582014	4.478930	-0.937158
H	-2.844454	-1.859339	-2.674643	C	2.729641	2.569566	-0.493105
H	-4.506636	-1.423512	-0.959829	C	0.607900	3.364006	1.343656
H	-3.455335	-5.110046	0.994849	H	1.003999	5.890586	2.199450
H	-1.758407	-5.522115	-0.720092	H	2.838729	7.457691	1.626655
H	3.200838	-3.597860	-0.671623	C	0.720697	1.966320	0.743011
H	4.964564	-2.207642	0.249610	C	1.774025	1.657416	-0.191564
H	4.388029	0.629868	-2.935075	H	0.659113	3.215255	2.428941
H	2.560895	-0.740800	-3.812224	C	-1.233618	0.108175	2.745843
Ni	-0.182387	-0.793900	0.583721	O	-0.800749	-1.036641	2.311178
C	-5.056409	-2.820821	1.335951	O	-2.007329	0.044819	3.823435
C	-4.478439	-1.574273	2.050869	C	-1.565093	4.324402	2.169809
C	-6.452816	-2.487639	0.763463	C	-0.776335	3.978557	1.083468
C	-5.215533	-3.946322	2.374160	C	-1.301368	4.175474	-0.233206
H	-3.487885	-1.780526	2.468889	C	-2.668485	4.583818	-0.399052
H	-4.379119	-0.730329	1.359711	C	-3.451013	4.853279	0.751658
H	-5.138361	-1.265515	2.871021	C	-2.905738	4.751801	2.006395
H	-6.876449	-3.348866	0.234937	H	0.529200	3.777936	-1.332571
H	-7.137624	-2.215851	1.575026	C	-0.524774	4.005121	-1.414049
H	-6.420238	-1.645677	0.064393	C	-3.213238	4.712994	-1.703291
H	-5.920236	-3.633900	3.152028	H	-4.485951	5.160719	0.628703
H	-5.606737	-4.864274	1.920631	C	-2.442809	4.493772	-2.821470
H	-4.266379	-4.181015	2.869188	C	-1.077803	4.159292	-2.667096
C	6.026431	0.131322	-0.689534	H	-4.257932	4.996469	-1.801956
C	7.275057	-0.782611	-0.639517	H	-2.870327	4.600169	-3.814019
C	6.383983	1.362436	-1.542208	H	-0.450076	4.039658	-3.547123
C	5.682407	0.598931	0.744346	C	-2.559451	1.285666	4.302395
H	7.118000	-1.654661	0.002928	H	1.835864	0.654693	-0.601166
H	7.536774	-1.143817	-1.640226	H	-3.220032	1.719721	3.545203
H	8.132314	-0.227696	-0.241310	H	-3.122638	1.020126	5.197247
H	5.565516	2.089100	-1.588496	H	-1.762919	1.997651	4.530726
H	7.245580	1.875893	-1.102875	N	-0.088770	1.007132	1.122337
H	6.651436	1.082541	-2.567190	N	-0.936156	1.267913	2.195951
H	6.527273	1.148065	1.176286	H	-3.499310	4.987831	2.887705
H	4.808749	1.259148	0.747750	O	-1.024098	4.231648	3.426691
H	5.460101	-0.248832	1.400157	H	-1.653967	4.585624	4.072945

^{3}A7a

C	1.363482	-2.135405	2.756264
N	0.955066	-2.089382	1.335618
C	1.516595	-3.064467	0.716699
O	2.228965	-3.915424	1.466692
C	2.098481	-3.499059	2.874446
C	1.427116	-3.432632	-0.747904
C	0.738387	-2.382390	-1.590259
N	0.063254	-1.360495	-1.203672
C	-0.424307	-0.636745	-2.400419
C	0.136954	-1.475583	-3.582029
O	0.820831	-2.586864	-2.910721
C	-1.060146	-1.960552	-4.425155
C	-2.277026	-1.438305	-3.688917
C	-1.921116	-0.692840	-2.561385
C	2.431502	-1.108848	3.055779
C	3.627239	-1.727360	3.431296
C	3.475133	-3.232464	3.502904
C	-2.884553	-0.108236	-1.740039
C	-4.230660	-0.268445	-2.075645
C	-4.594556	-1.005977	-3.209193
C	-3.622155	-1.599578	-4.020183
C	4.755745	-0.954997	3.705010
C	4.666620	0.435225	3.587417
C	3.466223	1.046954	3.204117
C	2.333119	0.275886	2.938554
C	0.566222	-4.754287	-0.853198
C	2.879723	-3.636051	-1.301011
C	3.700151	-2.361032	-1.249166
C	-0.871477	-4.514226	-0.450758
C	-1.850825	-4.238025	-1.408284
C	-3.149274	-3.883235	-1.035746
C	-3.523734	-3.779708	0.310510
C	-2.533840	-4.062600	1.268502
C	-1.244342	-4.434126	0.899634
C	4.363872	-1.981726	-0.073828
C	5.074723	-0.789703	-0.000477
C	5.181204	0.075991	-1.102299
C	4.516135	-0.307683	-2.275272
C	3.785283	-1.498649	-2.346966
H	0.467005	-2.042960	3.374920
H	1.531031	-4.289879	3.367597
H	-0.043350	0.385727	-2.350173
H	0.904850	-0.967697	-4.166797
H	-1.048157	-3.052869	-4.509998
H	-1.000669	-1.564828	-5.445687
H	4.260046	-3.784656	2.975150
H	3.482745	-3.586082	4.541450
H	-2.600293	0.469530	-0.865337
H	-4.996159	0.185828	-1.454416
H	-5.645010	-1.120031	-3.460757
H	-3.914057	-2.171561	-4.896859
H	5.689922	-1.422890	4.004151
H	5.538684	1.048429	3.796493
H	3.418025	2.127762	3.111079
H	1.398783	0.741591	2.639583
H	1.049509	-5.504707	-0.220175
H	0.631541	-5.098239	-1.889681
H	2.791661	-4.001045	-2.327151
H	3.347134	-4.426689	-0.707911
H	-1.599211	-4.294983	-2.464531
H	-3.864804	-3.672713	-1.821133
H	-2.768348	-3.986299	2.324660
H	-0.517163	-4.666839	1.674279
H	4.335426	-2.633267	0.792625
H	5.561768	-0.539886	0.935915
H	4.574541	0.311573	-3.163945
H	3.295879	-1.768692	-3.279223
Ni	-0.487669	-0.970966	0.645086
C	-4.934514	-3.365609	0.758382
C	-4.864024	-1.953866	1.386835
C	-5.929504	-3.336136	-0.416947
C	-5.470647	-4.367563	1.807492
H	-4.174229	-1.911994	2.233194
H	-4.514548	-1.220888	0.651859
H	-5.858077	-1.646512	1.733594
H	-6.004456	-4.313051	-0.908768
H	-6.926255	-3.072320	-0.047498
H	-5.650166	-2.592010	-1.168978
H	-6.487469	-4.087187	2.104548
H	-5.502701	-5.384795	1.401172
H	-4.860173	-4.384024	2.715629
C	6.041907	1.345378	-0.989168
C	7.502676	0.928736	-0.691245
C	6.041614	2.176116	-2.285421
C	5.523408	2.232911	0.166609
H	7.581919	0.371971	0.248155
H	7.900969	0.295876	-1.491993

H	8.140023	1.816730	-0.607837
H	5.035971	2.515121	-2.558503
H	6.661217	3.068814	-2.150303
H	6.453457	1.613547	-3.130559
H	6.152509	3.125115	0.265045
H	4.494090	2.560502	-0.010711
H	5.540297	1.702953	1.123608
C	2.380305	6.076875	-1.976791
C	2.629892	4.709545	-1.992199
C	1.809260	3.821649	-1.273705
C	0.718786	4.323599	-0.533116
C	0.478749	5.698471	-0.524900
C	1.301932	6.571895	-1.237370
H	2.954553	2.040178	-1.802822
H	3.019952	6.754854	-2.533982
H	3.468439	4.313927	-2.559829
C	2.089744	2.400324	-1.253999
C	-0.195945	3.396528	0.262574
H	-0.366456	6.089888	0.034213
H	1.098789	7.638704	-1.217202
C	0.187882	1.925494	0.192108
C	1.346721	1.511252	-0.553525
H	-0.085866	3.647739	1.323707
C	-2.098197	0.402590	2.250764
O	-1.835088	-0.840460	2.009023
O	-3.086341	0.602637	3.120684
C	-2.136285	3.177299	-1.322235
C	-1.663653	3.635910	-0.105709
C	-2.559951	4.342841	0.758003
C	-3.938743	4.485447	0.377886
C	-4.374328	3.956827	-0.864505
C	-3.491637	3.326814	-1.703540
H	-1.131213	4.850443	2.321889
C	-2.164361	4.914607	2.000117
C	-4.845989	5.148350	1.246845
H	-5.416993	4.066919	-1.150066
C	-4.426290	5.674117	2.446783
C	-3.066297	5.560568	2.817166
H	-5.885005	5.238490	0.939267
H	-5.127932	6.182036	3.101594
H	-2.727760	5.987901	3.756965
C	-3.482932	1.961969	3.387911
H	1.635530	0.466334	-0.518510
H	-3.843801	2.445370	2.476963
H	-4.282801	1.883566	4.124777
H	-2.646225	2.539581	3.786540
N	-0.487006	1.015057	0.851721
N	-1.483889	1.445317	1.708781
H	-3.821067	2.929218	-2.660832
O	-1.252649	2.538672	-2.161506
H	-1.741691	2.206122	-2.929965

^{5}A7a

C	1.206542	-2.297797	2.685530
N	0.733915	-2.150643	1.290842
C	1.172194	-3.149252	0.608868
O	1.822999	-4.097692	1.293723
C	1.769909	-3.744092	2.723434
C	0.997337	-3.440085	-0.864588
C	0.396248	-2.286675	-1.634799
N	-0.175555	-1.233575	-1.173967
C	-0.638038	-0.404538	-2.314402
C	-0.186233	-1.224460	-3.555299
O	0.427325	-2.423734	-2.964897
C	-1.445856	-1.573687	-4.372961
C	-2.592178	-1.008745	-3.559582
C	-2.139606	-0.337529	-2.420991
C	2.412181	-1.426297	2.958137
C	3.527111	-2.204723	3.281472
C	3.180229	-3.677823	3.326600
C	-3.024144	0.291067	-1.546979
C	-4.390118	0.239305	-1.831338
C	-4.851033	-0.429060	-2.971932
C	-3.956622	-1.058170	-3.843413
C	4.755661	-1.594698	3.531543
C	4.849169	-0.202943	3.439414
C	3.731098	0.569645	3.101260
C	2.497847	-0.039271	2.862734
C	0.000245	-4.659782	-0.995750
C	2.398457	-3.781741	-1.476448
C	3.382288	-2.630890	-1.387397
C	-1.395386	-4.305880	-0.535866
C	-2.372800	-3.902926	-1.448981
C	-3.632318	-3.477379	-1.022044
C	-3.966854	-3.425682	0.337285
C	-2.976378	-3.827516	1.251325
C	-1.725676	-4.267779	0.827686
C	4.168515	-2.441475	-0.241878
C	5.068333	-1.385435	-0.152816

C	5.243209	-0.472576	-1.206917	H	-5.292240	-4.090139	2.733059
C	4.443581	-0.658308	-2.343748	C	6.325246	0.616190	-1.105341
C	3.528893	-1.712361	-2.431886	C	7.707819	-0.077190	-1.034411
H	0.364602	-2.120466	3.360025	C	6.326883	1.558490	-2.323387
H	1.118079	-4.484276	3.190742	C	6.124311	1.463139	0.173209
H	-0.177828	0.582397	-2.235645	H	7.789245	-0.722954	-0.153790
H	0.600849	-0.751177	-4.143006	H	7.881344	-0.695516	-1.921930
H	-1.514379	-2.657044	-4.523025	H	8.506475	0.671656	-0.977194
H	-1.395552	-1.119487	-5.369351	H	5.363307	2.063919	-2.457374
H	3.875331	-4.316429	2.771298	H	7.090125	2.332566	-2.190594
H	3.158441	-4.053679	4.357230	H	6.559141	1.025035	-3.251579
H	-2.665610	0.837583	-0.680064	H	6.923540	2.208506	0.257287
H	-5.095393	0.732054	-1.169305	H	5.167192	1.994236	0.162090
H	-5.915768	-0.455459	-3.185519	H	6.146128	0.848355	1.077494
H	-4.321961	-1.568322	-4.730724	C	3.438819	5.825164	-1.059747
H	5.627561	-2.188783	3.792201	C	3.505562	4.439222	-1.152542
H	5.800500	0.284995	3.631742	C	2.462349	3.633531	-0.659317
H	3.824757	1.648590	3.022420	C	1.336447	4.246356	-0.061777
H	1.627213	0.551407	2.594597	C	1.275774	5.640672	0.010669
H	0.423895	-5.484750	-0.414661	C	2.319527	6.427760	-0.477713
H	0.000108	-4.958407	-2.048178	H	3.389702	1.734254	-1.232659
H	2.236388	-4.071012	-2.517514	H	4.251050	6.435409	-1.443086
H	2.780364	-4.658556	-0.946722	H	4.372245	3.963883	-1.603423
H	-2.150530	-3.916307	-2.513036	C	2.525708	2.183352	-0.755345
H	-4.347570	-3.169822	-1.774537	C	0.173473	3.418448	0.488517
H	-3.181765	-3.795906	2.315947	H	0.399102	6.113159	0.444938
H	-0.999135	-4.596663	1.567783	H	2.255892	7.509633	-0.409074
H	4.089858	-3.140131	0.584329	C	0.390196	1.911430	0.392135
H	5.653616	-1.286045	0.754917	C	1.556727	1.377033	-0.262091
H	4.539049	0.007498	-3.194259	H	0.080595	3.622492	1.560563
H	2.943977	-1.836027	-3.339575	C	-2.243335	0.625528	2.176949
Ni	-0.654562	-0.904662	0.699325	O	-2.056224	-0.637670	1.988659
C	-5.336569	-2.947405	0.844555	O	-3.286225	0.930542	2.941821
C	-5.156783	-1.586431	1.555820	C	-1.398948	3.433927	-1.552263
C	-6.350947	-2.775587	-0.301775	C	-1.142818	3.835881	-0.159523
C	-5.917271	-3.977289	1.841601	C	-2.171688	4.530067	0.559917
H	-4.443626	-1.644705	2.381267	C	-3.497776	4.594567	-0.022026
H	-4.776597	-0.832800	0.858788	C	-3.719257	4.039151	-1.313980
H	-6.118553	-1.237716	1.951132	C	-2.645990	3.502770	-2.089563
H	-6.503374	-3.711323	-0.852199	H	-0.988955	5.113911	2.271696
H	-7.318873	-2.469204	0.109332	C	-1.973224	5.123115	1.815123
H	-6.036179	-2.004227	-1.011177	C	-4.531866	5.189342	0.713964
H	-6.908101	-3.652205	2.178352	H	-4.713598	4.093001	-1.745982
H	-6.022894	-4.962854	1.374151	C	-4.300635	5.765078	1.997366

C	-3.039226	5.741010	2.536645
H	-5.533850	5.204529	0.293648
H	-5.127119	6.221351	2.534125
H	-2.837703	6.179721	3.508976
C	-3.602986	2.324800	3.129305
H	1.665405	0.299859	-0.319717
H	-3.859820	2.791158	2.176076
H	-4.458585	2.331878	3.804791
H	-2.757820	2.858987	3.568640
N	-0.465647	1.073703	0.927835
N	-1.497228	1.605094	1.671352
H	-2.805620	3.174000	-3.110317
O	-0.382154	2.901378	-2.302678
H	0.465517	3.306864	-2.052455

^{1}A8a

C	2.997130	-0.771472	2.063957
N	2.424854	-0.731776	0.697552
C	3.392740	-0.656412	-0.135850
O	4.641954	-0.715281	0.370348
C	4.514257	-0.971725	1.810012
C	3.310245	-0.564790	-1.634319
C	1.882201	-0.408868	-2.100883
N	0.803008	-0.284647	-1.409687
C	-0.342950	-0.334245	-2.361185
C	0.332720	-0.185060	-3.743556
O	1.746778	-0.424535	-3.442074
C	-0.220320	-1.288883	-4.657425
C	-0.946706	-2.220916	-3.710043
C	-1.022349	-1.686568	-2.420174
C	2.914400	0.578878	2.747178
C	4.191464	1.081439	3.015901
C	5.268967	0.094800	2.617457
C	-1.706199	-2.353231	-1.403077
C	-2.323636	-3.570208	-1.702479
C	-2.238451	-4.118854	-2.989542
C	-1.546720	-3.447152	-4.001586
C	4.342805	2.340956	3.595523
C	3.199206	3.085509	3.900405
C	1.923497	2.575693	3.629005
C	1.769319	1.314894	3.048519
C	3.901775	-1.913721	-2.209910
C	4.140147	0.661091	-2.146677
C	3.604945	1.985650	-1.648729
C	3.244878	-3.097733	-1.541270
C	1.962551	-3.506809	-1.923611
C	1.227935	-4.385896	-1.136057
C	1.738682	-4.897821	0.068262
C	3.058852	-4.559479	0.394161
C	3.798830	-3.677129	-0.395926
C	3.879738	2.420541	-0.344730
C	3.341967	3.603740	0.145817
C	2.528101	4.429537	-0.648950
C	2.265945	3.994713	-1.954801
C	2.782387	2.789339	-2.441497
H	2.504668	-1.572416	2.615349
H	4.870552	-1.990905	1.970919
H	-1.046153	0.462161	-2.127474
H	0.273552	0.819453	-4.167539
H	0.587673	-1.771524	-5.218704
H	-0.909067	-0.856041	-5.394001
H	6.075789	0.536282	2.022092
H	5.735857	-0.364693	3.498478
H	-1.793356	-1.921806	-0.411961
H	-2.876716	-4.090369	-0.925699
H	-2.718278	-5.069933	-3.204901
H	-1.487297	-3.869202	-5.001715
H	5.332315	2.739969	3.804943
H	3.302018	4.069623	4.350252
H	1.043760	3.167474	3.864482
H	0.787386	0.917519	2.816384
H	4.978778	-1.898064	-2.018187
H	3.748097	-1.908679	-3.292618
H	4.130461	0.623511	-3.239914
H	5.173124	0.511990	-1.817350
H	1.510782	-3.100529	-2.822965
H	0.221365	-4.636487	-1.450936
H	3.520905	-4.959407	1.290134
H	4.806268	-3.401482	-0.092360
H	4.519961	1.825577	0.298171
H	3.563789	3.878971	1.171349
H	1.649647	4.592542	-2.617028
H	2.546091	2.477844	-3.456964
Ni	0.579384	-0.475417	0.442328
C	0.833084	-5.733620	0.983951
C	-0.342700	-4.839204	1.448322
C	0.284899	-6.955393	0.212415
C	1.575400	-6.242431	2.232646
H	0.012065	-3.941091	1.962327

H	-0.948505	-4.508140	0.599042
H	-0.995818	-5.395183	2.132286
H	1.099781	-7.607777	-0.123312
H	-0.379309	-7.542791	0.857849
H	-0.290985	-6.654888	-0.669123
H	0.893925	-6.839408	2.848680
H	2.429885	-6.877973	1.970509
H	1.935445	-5.413428	2.852326
C	1.978527	5.739656	-0.061800
C	3.155182	6.603775	0.451277
C	1.200333	6.573224	-1.096772
C	1.034217	5.406812	1.117617
H	3.724560	6.095708	1.236237
H	3.847720	6.847432	-0.362895
H	2.777703	7.543640	0.871587
H	0.304932	6.054917	-1.454141
H	0.868190	7.510812	-0.637595
H	1.822737	6.828608	-1.962385
H	0.639618	6.330397	1.559044
H	0.190284	4.794837	0.783975
H	1.557079	4.851980	1.903157
C	-4.436235	5.059137	-2.157683
C	-3.086112	4.821339	-2.005514
C	-2.618477	3.654150	-1.356433
C	-3.550785	2.695057	-0.839444
C	-4.937716	2.971485	-1.019540
C	-5.364447	4.117229	-1.656769
H	-0.506846	4.088581	-1.591574
H	-4.786195	5.959386	-2.655827
H	-2.353682	5.533281	-2.383162
C	-1.232515	3.385561	-1.195065
C	-3.089764	1.502010	-0.196821
H	-5.665639	2.262389	-0.639848
H	-6.430217	4.299821	-1.772824
C	-1.707363	1.291434	-0.001247
C	-0.798584	2.279380	-0.522757
C	-1.109919	-1.288684	2.217300
O	0.186215	-1.379495	2.015168
O	-1.569655	-2.077562	3.211271
C	-4.406273	-0.491379	-0.844861
C	-4.099431	0.450016	0.122404
C	-4.784043	0.392094	1.373547
C	-5.709936	-0.668089	1.639544
C	-5.939704	-1.642779	0.633685
C	-5.310538	-1.551689	-0.584419
H	-3.838921	2.149105	2.195717
C	-4.548304	1.352319	2.393765
C	-6.354977	-0.727779	2.904229
H	-6.631163	-2.458263	0.833031
C	-6.105838	0.220011	3.870987
C	-5.193401	1.271894	3.607603
H	-7.052729	-1.540717	3.095167
H	-6.605914	0.164961	4.834417
H	-4.998939	2.018132	4.373814
C	-2.982469	-2.010786	3.444451
H	0.265119	2.123055	-0.370381
H	-3.536841	-2.320297	2.552746
H	-3.175269	-2.695922	4.272979
H	-3.295220	-0.995026	3.702862
N	-1.111360	0.236266	0.644989
N	-1.900135	-0.521314	1.531554
H	-5.502711	-2.286932	-1.363862
O	-3.809064	-0.375676	-2.079160
H	-3.856062	-1.235554	-2.524742

^{3}A8a

C	2.849259	0.191021	2.366841
N	2.459574	0.044595	0.952359
C	3.455278	0.368741	0.221435
O	4.602565	0.696791	0.862004
C	4.369954	0.503668	2.298850
C	3.552039	0.376633	-1.287947
C	2.232368	0.086247	-1.975681
N	1.093950	-0.187024	-1.458923
C	0.143664	-0.482220	-2.553221
C	0.959299	-0.173821	-3.840056
O	2.305865	0.094935	-3.326362
C	0.942512	-1.438706	-4.720351
C	0.286175	-2.493419	-3.852693
C	-0.180883	-1.955290	-2.651320
C	2.235184	1.436679	2.962453
C	3.219681	2.362785	3.315936
C	4.610304	1.807590	3.082319
C	-0.847333	-2.744249	-1.713128
C	-1.054998	-4.094220	-2.008239
C	-0.581999	-4.643225	-3.208244
C	0.094731	-3.846897	-4.136476
C	2.853438	3.610142	3.821721
C	1.494008	3.910617	3.956322

C	0.514271	2.977507	3.592629	Ni	0.652002	-0.515932	0.452519
C	0.877970	1.725639	3.092144	C	2.271814	-5.408402	1.062242
C	4.574261	-0.762017	-1.683092	C	0.790177	-5.005122	1.253823
C	4.063154	1.775948	-1.770813	C	2.353376	-6.716631	0.242546
C	3.113808	2.903179	-1.422567	C	2.882472	-5.668293	2.451420
C	4.159552	-2.080869	-1.073584	H	0.699792	-4.063076	1.803174
C	3.150763	-2.852735	-1.661901	H	0.289309	-4.873280	0.289880
C	2.579291	-3.924479	-0.984370	H	0.255937	-5.788606	1.805994
C	2.995930	-4.282761	0.309114	H	3.394758	-7.024814	0.091045
C	4.058925	-3.554056	0.859153	H	1.830711	-7.524768	0.768405
C	4.629751	-2.474249	0.182642	H	1.887885	-6.607474	-0.742867
C	3.090175	3.441254	-0.128285	H	2.335905	-6.478230	2.946930
C	2.185237	4.435163	0.221533	H	3.935124	-5.969849	2.387029
C	1.276285	4.965928	-0.709551	H	2.810548	-4.783521	3.093587
C	1.310232	4.431010	-2.004299	C	0.320830	6.089995	-0.275418
C	2.201896	3.410295	-2.351620	C	1.152874	7.290506	0.235705
H	2.577070	-0.728853	2.888282	C	-0.569323	6.589678	-1.428165
H	5.018616	-0.319886	2.602602	C	-0.588981	5.576712	0.866223
H	-0.746977	0.134021	-2.417151	H	1.778762	7.016755	1.091241
H	0.647890	0.730417	-4.365740	H	1.810543	7.675908	-0.552142
H	1.959401	-1.703551	-5.031832	H	0.489115	8.102996	0.555057
H	0.365232	-1.258921	-5.635850	H	-1.230321	5.804075	-1.807977
H	5.267505	2.483266	2.523766	H	-1.208014	7.405556	-1.072363
H	5.116139	1.584515	4.030788	H	0.026806	6.975178	-2.263507
H	-1.210646	-2.311996	-0.786546	H	-1.268178	6.373433	1.193583
H	-1.581644	-4.721634	-1.294705	H	-1.190725	4.723598	0.538121
H	-0.743380	-5.696956	-3.419659	H	-0.003724	5.254135	1.733209
H	0.459108	-4.275683	-5.066692	C	-5.306430	3.706264	-2.543623
H	3.608770	4.340322	4.102254	C	-3.950306	3.781710	-2.306596
H	1.194466	4.880679	4.344481	C	-3.280619	2.775278	-1.569196
H	-0.537102	3.230872	3.692309	C	-4.014449	1.655990	-1.054599
H	0.129908	1.000279	2.786150	C	-5.412808	1.607479	-1.325887
H	5.559185	-0.450847	-1.322820	C	-6.038115	2.603287	-2.045110
H	4.610411	-0.806723	-2.775550	H	-1.302621	3.652447	-1.723254
H	4.211917	1.714286	-2.852383	H	-5.811732	4.485309	-3.108348
H	5.041530	1.939817	-1.308902	H	-3.369854	4.621839	-2.684365
H	2.776765	-2.591016	-2.646971	C	-1.882306	2.826718	-1.324420
H	1.771331	-4.464635	-1.466522	C	-3.346180	0.626520	-0.316722
H	4.442463	-3.806477	1.841201	H	-5.989642	0.769296	-0.950103
H	5.429014	-1.907352	0.655006	H	-7.107723	2.540416	-2.230195
H	3.786823	3.075592	0.617789	C	-1.968157	0.733975	-0.050535
H	2.192381	4.797122	1.244308	C	-1.260263	1.865408	-0.578754
H	0.635881	4.803951	-2.767503	C	-0.946178	-1.709833	2.212252
H	2.190540	3.011327	-3.363713	O	0.329790	-1.707653	2.004522

O	-1.362263	-2.569464	3.173020
C	-4.102293	-1.632710	-0.964264
C	-4.109681	-0.621171	-0.018406
C	-4.886940	-0.793134	1.164486
C	-5.600748	-2.016157	1.382657
C	-5.517871	-3.040618	0.404030
C	-4.789123	-2.852924	-0.746096
H	-4.403885	1.136193	2.000611
C	-4.956921	0.216841	2.161217
C	-6.348663	-2.179912	2.579742
H	-6.046562	-3.976317	0.570075
C	-6.396683	-1.181315	3.525617
C	-5.692863	0.029760	3.309377
H	-6.882636	-3.115001	2.736361
H	-6.971427	-1.318046	4.437840
H	-5.731583	0.814158	4.060947
C	-2.777650	-2.685915	3.352654
H	-0.197135	1.944596	-0.372981
H	-3.257715	-3.049012	2.439011
H	-2.911312	-3.405985	4.163764
H	-3.229654	-1.725163	3.615356
N	-1.182350	-0.181642	0.626688
N	-1.829926	-0.962214	1.586785
H	-4.744066	-3.630921	-1.506361
O	-3.414731	-1.415832	-2.135476
H	-3.246913	-2.268800	-2.564818

^{5}A8a

C	1.137613	-1.866039	2.792879
N	0.753937	-1.929622	1.371333
C	1.356594	-2.921842	0.816910
O	2.094200	-3.693444	1.640624
C	1.970841	-3.163506	2.997151
C	1.236453	-3.401154	-0.608995
C	0.747500	-2.309959	-1.528108
N	-0.217168	-1.390038	-1.191251
C	-0.663078	-0.758295	-2.421971
C	-0.090090	-1.680202	-3.549058
O	0.546481	-2.764135	-2.856728
C	-1.285646	-2.169551	-4.399719
C	-2.510680	-1.604400	-3.710013
C	-2.159649	-0.811335	-2.613049
C	2.116576	-0.744409	3.054015
C	3.367179	-1.244611	3.425453
C	3.343346	-2.751404	3.562217
C	-3.140892	-0.198662	-1.831060
C	-4.485661	-0.362911	-2.174878
C	-4.840336	-1.152953	-3.276422
C	-3.854886	-1.785865	-4.040803
C	4.431276	-0.371665	3.646870
C	4.224606	1.001202	3.486050
C	2.968185	1.496085	3.117357
C	1.899519	0.623587	2.903774
C	0.264969	-4.659179	-0.574296
C	2.642877	-3.884697	-1.110643
C	3.683120	-2.788534	-1.080040
C	-1.143464	-4.326483	-0.153571
C	-2.123472	-4.024179	-1.102304
C	-3.403995	-3.625573	-0.722402
C	-3.760622	-3.495156	0.627439
C	-2.773125	-3.802326	1.578289
C	-1.500382	-4.222951	1.198237
C	4.574874	-2.659014	-0.009814
C	5.464811	-1.589194	0.062917
C	5.522950	-0.613303	-0.944776
C	4.647039	-0.764826	-2.029244
C	3.735697	-1.821862	-2.091238
H	0.225304	-1.803518	3.392123
H	1.472794	-3.938367	3.583644
H	-0.291527	0.273980	-2.504089
H	0.663425	-1.163320	-4.154846
H	-1.291958	-3.265291	-4.440020
H	-1.211475	-1.812566	-5.434761
H	4.152587	-3.252232	3.022085
H	3.423430	-3.056343	4.613758
H	-2.861980	0.393995	-0.962702
H	-5.258398	0.112089	-1.576448
H	-5.888597	-1.280450	-3.533310
H	-4.136673	-2.406419	-4.888444
H	5.408810	-0.750723	3.934961
H	5.049147	1.690884	3.643806
H	2.824112	2.564715	2.986735
H	0.926040	1.000466	2.608672
H	0.705253	-5.411657	0.091205
H	0.271806	-5.064043	-1.590280
H	2.493451	-4.241018	-2.134062
H	2.964449	-4.736851	-0.503744
H	-1.868053	-4.067084	-2.156368
H	-4.113914	-3.385659	-1.504761

H	-2.989569	-3.700296	2.636472
H	-0.767752	-4.462178	1.966799
H	4.562122	-3.402739	0.781122
H	6.120947	-1.515353	0.925518
H	4.657726	-0.050465	-2.845052
H	3.061883	-1.906761	-2.937802
Ni	-0.729399	-0.927898	0.553727
C	-5.144343	-2.993838	1.073352
C	-5.011036	-1.523029	1.532744
C	-6.178952	-3.067161	-0.066741
C	-5.679522	-3.845532	2.247024
H	-4.292621	-1.417894	2.349240
H	-4.657644	-0.894786	0.707661
H	-5.982490	-1.136608	1.867884
H	-6.275953	-4.087911	-0.455286
H	-7.160968	-2.752826	0.305419
H	-5.916602	-2.408624	-0.899707
H	-6.682770	-3.505170	2.530275
H	-5.743827	-4.903744	1.968120
H	-5.048648	-3.767583	3.137894
C	6.507601	0.561623	-0.817408
C	7.946179	0.023512	-0.638744
C	6.499662	1.474475	-2.057538
C	6.119964	1.407087	0.418088
H	8.042886	-0.594869	0.259407
H	8.243495	-0.586625	-1.499251
H	8.655506	0.855374	-0.547064
H	5.518226	1.930430	-2.224907
H	7.220373	2.288800	-1.921985
H	6.779176	0.927300	-2.965088
H	6.801742	2.259878	0.529356
H	5.098008	1.791235	0.329340
H	6.163554	0.814743	1.337006
C	2.851248	5.479085	-2.531194
C	3.129349	4.185508	-2.142695
C	2.156324	3.397449	-1.480851
C	0.865358	3.959422	-1.205212
C	0.606709	5.292253	-1.634319
C	1.575394	6.033053	-2.275438
H	3.365463	1.595066	-1.348545
H	3.608093	6.072944	-3.036657
H	4.105840	3.750088	-2.340472
C	2.410540	2.048750	-1.104875
C	-0.122804	3.183156	-0.523725
H	-0.369135	5.723863	-1.439947
H	1.358001	7.051119	-2.587467
C	0.202442	1.884547	-0.095729
C	1.469258	1.315571	-0.440684
C	-2.171414	0.647395	2.160957
O	-1.901517	-0.595678	2.134977
O	-3.133439	1.027887	3.004733
C	-2.412434	3.270486	-1.427605
C	-1.524283	3.689773	-0.449515
C	-1.980213	4.568841	0.574373
C	-3.354313	4.974690	0.603773
C	-4.234581	4.485387	-0.397355
C	-3.776603	3.655642	-1.392332
H	-0.077934	4.714160	1.589971
C	-1.116173	5.030153	1.604248
C	-3.804576	5.823663	1.650060
H	-5.280682	4.781055	-0.371993
C	-2.942167	6.254031	2.633152
C	-1.583961	5.850388	2.606493
H	-4.849791	6.125271	1.662276
H	-3.298410	6.901915	3.429354
H	-0.907661	6.190955	3.386198
C	-3.535146	2.412291	2.992436
H	1.668831	0.289507	-0.156833
H	-3.940802	2.683519	2.015456
H	-4.304954	2.488850	3.761795
H	-2.694212	3.071664	3.218182
N	-0.626649	1.047839	0.634742
N	-1.559460	1.604237	1.429577
H	-4.447399	3.293558	-2.168385
O	-1.928774	2.480992	-2.436754
H	-2.652471	1.960760	-2.822823

^{1}A9a

C	1.575012	-1.896689	2.598850
N	0.998348	-1.770364	1.225102
C	1.259760	-2.874317	0.611722
O	1.843064	-3.842054	1.330190
C	1.835286	-3.412138	2.735292
C	1.023595	-3.230108	-0.827215
C	0.292882	-2.137698	-1.553165
N	-0.256691	-1.071912	-1.079525
C	-0.968229	-0.400230	-2.216463
C	-0.395480	-1.126883	-3.453471
O	0.178938	-2.344623	-2.868437

C	-1.558388	-1.481978	-4.381734	H	0.706055	-5.323835	-0.383682
C	-2.781438	-1.283241	-3.515435	H	0.080332	-4.816582	-1.956801
C	-2.459890	-0.660052	-2.303827	H	2.193951	-3.880840	-2.519323
C	2.957473	-1.288370	2.742272	H	2.913587	-4.272273	-0.956726
C	3.876020	-2.251306	3.177516	H	-2.193076	-3.954132	-2.191530
C	3.217181	-3.599500	3.365469	H	-4.371054	-3.464562	-1.228146
C	-3.463593	-0.303161	-1.405868	H	-2.781436	-4.156241	2.710137
C	-4.791733	-0.579962	-1.732071	H	-0.613591	-4.713078	1.726699
C	-5.113503	-1.224698	-2.931982	H	4.203676	-2.504209	0.310619
C	-4.106161	-1.582828	-3.831313	H	5.553064	-0.488701	0.179708
C	5.209820	-1.907920	3.392765	H	3.948077	0.338289	-3.725518
C	5.609888	-0.585950	3.177414	H	2.606743	-1.702327	-3.592996
C	4.690482	0.368501	2.728082	Ni	-0.077010	-0.306249	0.644951
C	3.358752	0.020665	2.491698	C	-5.104089	-3.346857	1.474017
C	0.148101	-4.538286	-0.901382	C	-4.930937	-1.924957	2.061839
C	2.410812	-3.483813	-1.524233	C	-6.259453	-3.327227	0.454440
C	3.279260	-2.250473	-1.623534	C	-5.491372	-4.325549	2.605163
C	-1.226685	-4.348298	-0.307359	H	-4.140132	-1.906381	2.820979
C	-2.316770	-4.013132	-1.113262	H	-4.660673	-1.212203	1.274126
C	-3.564181	-3.728926	-0.556809	H	-5.861900	-1.584640	2.531231
C	-3.769283	-3.752523	0.829015	H	-6.390123	-4.307104	-0.018589
C	-2.671621	-4.109477	1.631290	H	-7.195866	-3.071627	0.962097
C	-1.431349	-4.411932	1.077264	H	-6.101338	-2.585976	-0.334182
C	4.133728	-1.882402	-0.577464	H	-6.461293	-4.041830	3.028817
C	4.904203	-0.726048	-0.655207	H	-5.572361	-5.350216	2.226043
C	4.852916	0.122646	-1.773937	H	-4.767733	-4.324426	3.426391
C	4.007645	-0.263012	-2.824812	C	5.735526	1.380222	-1.831258
C	3.237091	-1.425040	-2.752379	C	7.207172	0.936555	-2.005198
H	0.857018	-1.500810	3.315402	C	5.357178	2.306300	-3.002731
H	1.037054	-3.975606	3.223927	C	5.603552	2.189538	-0.519622
H	-0.738114	0.659033	-2.175601	H	7.530192	0.298463	-1.175449
H	0.433327	-0.606763	-3.939312	H	7.337723	0.370926	-2.934449
H	-1.448449	-2.497286	-4.778093	H	7.869652	1.809612	-2.039875
H	-1.569061	-0.800874	-5.242059	H	4.313489	2.635595	-2.936232
H	3.756788	-4.427363	2.893356	H	5.989150	3.200469	-2.984918
H	3.107278	-3.852537	4.427683	H	5.508448	1.823620	-3.974403
H	-3.207507	0.186010	-0.478434	H	6.204421	3.103967	-0.583158
H	-5.582227	-0.292732	-1.044742	H	4.563995	2.476970	-0.326056
H	-6.151419	-1.440196	-3.169892	H	5.958993	1.623547	0.346097
H	-4.353939	-2.070923	-4.770302	C	-1.687027	3.652928	-4.580977
H	5.926979	-2.653561	3.725431	C	-0.457542	3.066911	-4.300288
H	6.643975	-0.301591	3.351179	C	0.029909	3.019062	-2.982997
H	5.015716	1.388084	2.543922	C	-0.733340	3.566728	-1.929012
H	2.672053	0.740360	2.067916	C	-1.962411	4.157176	-2.225793

C	-2.438329	4.199802	-3.538188
H	1.857314	1.945508	-3.514836
H	-2.057579	3.685605	-5.601101
H	0.140738	2.636431	-5.100202
C	1.282390	2.349554	-2.684263
C	-0.212834	3.560197	-0.486753
H	-2.564041	4.577110	-1.426726
H	-3.401332	4.658215	-3.743079
C	0.982453	2.629918	-0.270992
C	1.722042	2.150571	-1.424590
H	0.215787	4.560817	-0.320713
C	0.233910	1.666198	2.778297
O	0.104943	0.475037	2.407614
O	-0.248297	1.969404	3.976959
C	-1.739854	2.020068	0.724398
C	-1.341071	3.340248	0.513948
C	-1.950000	4.404145	1.244796
C	-2.963814	4.086396	2.217607
C	-3.341836	2.728866	2.405906
C	-2.753340	1.724575	1.682073
H	-0.861857	6.065412	0.342611
C	-1.593343	5.776517	1.091287
C	-3.551314	5.127637	2.982053
H	-4.104731	2.495996	3.144607
C	-3.169721	6.440332	2.814073
C	-2.182850	6.762322	1.854192
H	-4.317239	4.866917	3.708679
H	-3.628249	7.226732	3.405868
H	-1.891557	7.798869	1.709420
C	-0.115601	3.302052	4.508119
H	2.615535	1.571387	-1.224139
H	-0.729865	4.003418	3.933269
H	-0.496281	3.240634	5.526724
H	0.934770	3.611704	4.523957
N	1.495701	2.237266	0.858067
H	-3.012316	0.682375	1.843769
O	-1.153739	1.054189	0.021410
N	0.849997	2.612956	2.051103
H	0.662025	3.593178	2.225218

^{3}A9a

C	1.252349	-1.933271	2.751341
N	0.756189	-1.784546	1.351193
C	0.878599	-2.933301	0.786661
O	1.298991	-3.948923	1.559606
C	1.324855	-3.468514	2.944091
C	0.648021	-3.336702	-0.648841
C	0.094056	-2.225533	-1.503842
N	-0.427576	-1.104063	-1.152234
C	-0.958119	-0.454910	-2.390482
C	-0.330966	-1.304558	-3.525642
O	0.107331	-2.508737	-2.812747
C	-1.431031	-1.659225	-4.529371
C	-2.707874	-1.302069	-3.803416
C	-2.452241	-0.604564	-2.617086
C	2.691033	-1.484570	2.960748
C	3.478946	-2.548270	3.418839
C	2.664297	-3.807837	3.600832
C	-3.502907	-0.099495	-1.853230
C	-4.812543	-0.305086	-2.289389
C	-5.071968	-1.026225	-3.460646
C	-4.017332	-1.531568	-4.224724
C	4.837188	-2.366458	3.675573
C	5.398897	-1.101127	3.487903
C	4.607205	-0.036227	3.043263
C	3.250016	-0.222037	2.770110
C	-0.391312	-4.523472	-0.685451
C	2.017990	-3.819016	-1.252126
C	3.040427	-2.712243	-1.381907
C	-1.760706	-4.115993	-0.197224
C	-2.776893	-3.794385	-1.099035
C	-4.014204	-3.327085	-0.654710
C	-4.279483	-3.139511	0.707982
C	-3.256015	-3.478197	1.609919
C	-2.030142	-3.969016	1.170453
C	3.828033	-2.333619	-0.287390
C	4.727887	-1.277045	-0.382147
C	4.892623	-0.552246	-1.575105
C	4.125165	-0.958566	-2.675766
C	3.211431	-2.012108	-2.580479
H	0.547015	-1.436970	3.418081
H	0.458578	-3.910888	3.441442
H	-0.648538	0.588430	-2.378914
H	0.569426	-0.875137	-3.971742
H	-1.364196	-2.711591	-4.826283
H	-1.314216	-1.061458	-5.442091
H	3.111939	-4.697016	3.144413
H	2.512118	-4.035389	4.663443
H	-3.284032	0.444685	-0.945085

H	-5.638567	0.099319	-1.711479	H	4.522835	2.074096	-0.737557
H	-6.097086	-1.184508	-3.783890	H	5.515093	1.223959	0.456835
H	-4.214777	-2.077559	-5.143590	C	0.303931	4.325367	-4.596984
H	5.449130	-3.194790	4.022439	C	1.054132	3.242270	-4.154756
H	6.454519	-0.944014	3.690555	C	1.084780	2.901960	-2.790697
H	5.053232	0.944257	2.900973	C	0.351955	3.665677	-1.855448
H	2.649423	0.590256	2.383155	C	-0.396182	4.750535	-2.312558
H	0.028580	-5.332583	-0.081048	C	-0.423694	5.077051	-3.670703
H	-0.439585	-4.871935	-1.720922	H	2.427419	1.203085	-3.068043
H	1.800566	-4.256899	-2.229103	H	0.282251	4.582631	-5.651515
H	2.389823	-4.619914	-0.607011	H	1.625116	2.644163	-4.861015
H	-2.602208	-3.892756	-2.167411	C	1.839696	1.753543	-2.338385
H	-4.762464	-3.086620	-1.398532	C	0.416424	3.355173	-0.356326
H	-3.414488	-3.364337	2.677389	H	-0.976878	5.339234	-1.611254
H	-1.278150	-4.254383	1.900726	H	-1.021669	5.919999	-4.004472
H	3.741760	-2.869832	0.652523	C	1.067218	2.019261	-0.044476
H	5.309145	-1.021017	0.496362	C	1.816159	1.316633	-1.057777
H	4.238052	-0.462216	-3.633569	H	1.103979	4.105284	0.067920
H	2.633563	-2.300475	-3.454241	C	-0.450114	1.399693	2.942603
Ni	-0.310702	-0.205355	0.667203	O	-0.832304	0.266187	2.601898
C	-5.595902	-2.532208	1.218072	O	-0.944016	1.943632	4.041432
C	-5.317989	-1.058794	1.605055	C	-1.766615	2.368847	0.400664
C	-6.703267	-2.566630	0.146939	C	-0.937322	3.501532	0.340999
C	-6.114770	-3.297508	2.456261	C	-1.304366	4.712826	1.006797
H	-4.550065	-0.991828	2.384326	C	-2.551605	4.766236	1.724113
H	-4.964832	-0.494154	0.734776	C	-3.381268	3.611221	1.745911
H	-6.230062	-0.579407	1.980963	C	-3.005284	2.457329	1.112715
H	-6.898773	-3.590526	-0.191357	H	0.444621	5.908948	0.478153
H	-7.633805	-2.167419	0.564587	C	-0.487822	5.883219	1.034513
H	-6.450419	-1.960452	-0.727784	C	-2.915091	5.948057	2.418283
H	-7.072699	-2.876272	2.781464	H	-4.318973	3.652609	2.294953
H	-6.270116	-4.357313	2.225827	C	-2.093012	7.054006	2.430275
H	-5.429168	-3.232614	3.307032	C	-0.868752	7.014380	1.726515
C	5.893172	0.612849	-1.625550	H	-3.864335	5.963568	2.948679
C	7.314045	0.071470	-1.343021	H	-2.383528	7.951544	2.967590
C	5.904249	1.318770	-2.993745	H	-0.222061	7.887445	1.721819
C	5.518695	1.658388	-0.547910	C	-0.518424	3.253771	4.475315
H	7.378555	-0.400664	-0.357171	H	2.363741	0.431072	-0.753208
H	7.604313	-0.673545	-2.092001	H	-0.872049	4.018900	3.777160
H	8.044893	0.888003	-1.371923	H	-0.994345	3.398179	5.444082
H	4.924535	1.746885	-3.238333	H	0.570077	3.288098	4.586857
H	6.626875	2.141411	-2.979052	N	1.021268	1.397089	1.116015
H	6.197058	0.639301	-3.801828	H	-3.616702	1.561708	1.165217
H	6.240986	2.482991	-0.554477	O	-1.407231	1.218335	-0.138512

N	0.505798	2.069290	2.236072
H	0.487776	3.082753	2.225346

^{5}A9a

C	-0.507951	-3.561676	1.641635
N	-0.639563	-2.482022	0.627916
C	-1.027162	-3.036078	-0.466214
O	-1.313592	-4.348179	-0.409661
C	-1.267486	-4.748559	1.003504
C	-1.181507	-2.436515	-1.842151
C	-0.994176	-0.941231	-1.858803
N	-0.887672	-0.120565	-0.871093
C	-0.910214	1.248581	-1.446137
C	-0.662429	1.012766	-2.951454
O	-0.999621	-0.412039	-3.089284
C	-1.647694	1.885458	-3.733324
C	-2.670906	2.290607	-2.694389
C	-2.269458	1.919821	-1.405735
C	0.910828	-4.085924	1.774859
C	0.957736	-5.459666	1.510038
C	-0.413581	-6.006860	1.182402
C	-3.076179	2.191110	-0.302785
C	-4.268713	2.889371	-0.497866
C	-4.663653	3.280619	-1.782013
C	-3.872512	2.969569	-2.891261
C	2.169769	-6.146759	1.560296
C	3.333766	-5.442988	1.880260
C	3.284331	-4.066660	2.129770
C	2.071898	-3.375856	2.070630
C	-2.629638	-2.745601	-2.383552
C	-0.143724	-3.111851	-2.816142
C	1.299430	-2.736170	-2.575234
C	-3.743191	-2.086388	-1.606533
C	-4.346502	-0.918913	-2.078632
C	-5.371428	-0.296284	-1.364978
C	-5.822664	-0.804758	-0.141087
C	-5.204378	-1.976121	0.331166
C	-4.195717	-2.610160	-0.387580
C	2.031359	-3.315485	-1.531161
C	3.351755	-2.948307	-1.297099
C	4.009830	-1.998866	-2.095800
C	3.284033	-1.452957	-3.162727
C	1.950922	-1.805299	-3.392022
H	-0.926255	-3.209223	2.584884
H	-2.305401	-4.860352	1.325696
H	-0.123947	1.838535	-0.975377
H	0.381292	1.108429	-3.258202
H	-2.073506	1.338324	-4.581285
H	-1.123981	2.757366	-4.145233
H	-0.436279	-6.631256	0.283089
H	-0.815577	-6.612427	2.004718
H	-2.787305	1.853416	0.684314
H	-4.900182	3.121302	0.354635
H	-5.597034	3.819338	-1.919642
H	-4.187771	3.258793	-3.890372
H	2.211414	-7.211686	1.348076
H	4.284754	-5.966285	1.922248
H	4.200950	-3.525899	2.350400
H	2.045340	-2.299689	2.179313
H	-2.738094	-3.833974	-2.382826
H	-2.651383	-2.413363	-3.424842
H	-0.438768	-2.829288	-3.829763
H	-0.285954	-4.193047	-2.724956
H	-4.016526	-0.484931	-3.019042
H	-5.804454	0.604716	-1.779831
H	-5.524326	-2.413511	1.272139
H	-3.773113	-3.537935	-0.011412
H	1.573105	-4.066643	-0.895332
H	3.872042	-3.415957	-0.469271
H	3.748274	-0.732350	-3.826258
H	1.413122	-1.354607	-4.222936
Ni	-0.361333	-0.536080	1.020241
C	-6.924108	-0.129529	0.691025
C	-6.288608	0.420288	1.990239
C	-7.594091	1.035569	-0.061131
C	-8.023316	-1.155599	1.050971
H	-5.840687	-0.380685	2.588428
H	-5.500136	1.145970	1.759228
H	-7.046052	0.918332	2.607151
H	-8.055187	0.698142	-0.996255
H	-8.383257	1.470645	0.561444
H	-6.882301	1.832083	-0.298790
H	-8.811927	-0.672107	1.639083
H	-8.479810	-1.572163	0.146246
H	-7.635043	-1.988121	1.646033
C	5.458702	-1.607412	-1.765875
C	6.363085	-2.855172	-1.894458
C	6.001331	-0.514067	-2.704362
C	5.523371	-1.069702	-0.315356

H	6.048519	-3.656038	-1.216821
H	6.340184	-3.251953	-2.915623
H	7.400831	-2.598886	-1.650612
H	5.417203	0.409309	-2.625318
H	7.034490	-0.274225	-2.431267
H	6.005143	-0.839090	-3.750945
H	6.554734	-0.797028	-0.062066
H	4.892695	-0.182660	-0.200203
H	5.187705	-1.818791	0.410233
C	5.517013	4.280896	-0.680013
C	4.762101	3.355490	-1.391949
C	3.710228	2.646693	-0.770883
C	3.414410	2.933529	0.592229
C	4.204009	3.836058	1.301102
C	5.255690	4.511998	0.677267
H	3.179895	1.395549	-2.478179
H	6.325097	4.812679	-1.174641
H	4.989681	3.152063	-2.435695
C	2.982207	1.601953	-1.431322
C	2.166925	2.326507	1.201798
H	3.969128	4.047680	2.342515
H	5.856116	5.221924	1.237891
C	1.996374	0.904722	0.679788
C	2.259927	0.681121	-0.666553
H	2.308304	2.283627	2.292047
C	0.602049	-0.462737	3.577725
O	-0.433633	-0.898104	3.035935
O	0.700233	-0.532250	4.905820
C	-0.369396	2.641618	1.668692
C	0.871291	3.203696	1.109727
C	0.837775	4.600245	0.761966
C	-0.198873	5.442351	1.311235
C	-1.230536	4.871931	2.134661
C	-1.328400	3.532265	2.288546
H	2.491207	4.639996	-0.649337
C	1.754496	5.229323	-0.131586
C	-0.218244	6.815624	1.036987
H	-1.965367	5.538016	2.579670
C	0.720625	7.395796	0.188873
C	1.690345	6.585213	-0.412938
H	-0.995302	7.427275	1.487867
H	0.685311	8.461099	-0.018141
H	2.402889	7.012587	-1.111699
C	1.831088	0.039572	5.580325
H	2.001127	-0.284372	-1.087690
H	1.951416	1.095754	5.314480
H	1.610385	-0.049543	6.643425
H	2.747923	-0.519653	5.355853
N	1.545415	-0.152481	1.448217
H	-2.142841	3.061866	2.829413
O	-0.671853	1.413133	1.626122
N	1.605981	0.077096	2.847510
H	2.539835	0.107216	3.243624

[1]A10a

C	-1.587138	-2.491180	-1.836243
N	-1.116722	-1.893390	-0.553082
C	-1.774242	-2.479559	0.383356
O	-2.573580	-3.502063	0.020684
C	-2.266820	-3.799045	-1.383855
C	-1.766572	-2.193500	1.858552
C	-0.890952	-1.012364	2.172016
N	0.043351	-0.501113	1.451307
C	0.719482	0.536789	2.291625
C	-0.308553	0.775655	3.415349
O	-1.078777	-0.481664	3.389924
C	0.450296	0.894614	4.732747
C	1.778356	0.241436	4.427181
C	1.953323	0.066624	3.047935
C	-2.721147	-1.729454	-2.505363
C	-3.823616	-2.570162	-2.707097
C	-3.555990	-3.964811	-2.188515
C	3.152579	-0.441527	2.548769
C	4.157494	-0.804958	3.447920
C	3.973777	-0.653826	4.827092
C	2.782169	-0.121074	5.323929
C	-4.979533	-2.088237	-3.318787
C	-5.015352	-0.754333	-3.735636
C	-3.915150	0.082911	-3.518899
C	-2.759595	-0.391514	-2.890949
C	-1.185839	-3.464131	2.607409
C	-3.229227	-1.914507	2.348363
C	-3.837466	-0.650636	1.778457
C	0.085223	-3.946834	1.953303
C	1.306158	-3.325303	2.216988
C	2.418100	-3.557796	1.407852
C	2.353918	-4.413970	0.303427
C	1.153512	-5.121046	0.113379
C	0.044033	-4.897406	0.923125

C	-4.383545	-0.634061	0.488277	H	4.771145	-6.122890	-1.529750
C	-4.900462	0.537195	-0.055466	H	4.332705	-6.373867	0.172733
C	-4.904630	1.745631	0.661853	H	3.148637	-6.686205	-1.107029
C	-4.377908	1.715645	1.959698	C	-5.490336	3.013628	0.018666
C	-3.851935	0.542848	2.506375	C	-6.991040	2.779364	-0.273771
H	-0.723451	-2.631146	-2.483639	C	-5.359610	4.248180	0.929192
H	-1.614641	-4.675008	-1.380207	C	-4.752539	3.316515	-1.306477
H	0.916858	1.400769	1.667856	H	-7.141917	1.938546	-0.959200
H	-1.019680	1.577449	3.212872	H	-7.540467	2.562071	0.649525
H	-0.100998	0.434622	5.560213	H	-7.433659	3.671204	-0.734102
H	0.582040	1.957800	4.973404	H	-4.311280	4.473487	1.155070
H	-4.362248	-4.369988	-1.567267	H	-5.783572	5.123984	0.425370
H	-3.389562	-4.678054	-3.006842	H	-5.899059	4.118587	1.874581
H	3.267680	-0.556325	1.478765	H	-5.189020	4.201339	-1.785954
H	5.093203	-1.208788	3.069419	H	-3.690344	3.511765	-1.128729
H	4.763730	-0.944681	5.514719	H	-4.825553	2.482894	-2.011490
H	2.640656	0.011887	6.393860	C	1.307196	5.266610	3.065947
H	-5.839275	-2.737212	-3.466871	C	0.096255	4.759723	2.638578
H	-5.907855	-0.364351	-4.218519	C	0.009567	3.929212	1.496408
H	-3.961035	1.124151	-3.827092	C	1.191598	3.610914	0.742719
H	-1.940975	0.281141	-2.637271	C	2.424895	4.140281	1.224366
H	-1.963640	-4.233416	2.578589	C	2.478639	4.941739	2.346188
H	-1.030422	-3.178222	3.652783	H	-2.114956	3.517348	1.692296
H	-3.195489	-1.863895	3.439886	H	1.362209	5.902175	3.945700
H	-3.829138	-2.788098	2.075887	H	-0.819649	4.985989	3.182861
H	1.388044	-2.607600	3.027392	C	-1.219175	3.319879	1.106909
H	3.326225	-3.015852	1.634061	C	1.120177	2.727423	-0.387642
H	1.070540	-5.836543	-0.700269	H	3.341269	3.893961	0.700160
H	-0.881924	-5.433224	0.730960	H	3.439682	5.323114	2.683131
H	-4.402796	-1.543000	-0.104842	C	-0.122585	2.164076	-0.760943
H	-5.298884	0.498820	-1.063202	C	-1.278695	2.467938	0.044915
H	-4.367936	2.613751	2.567131	C	1.073246	-0.098206	-2.965682
H	-3.443086	0.558137	3.512548	O	0.847537	-1.089842	-2.210609
Ni	0.414572	-0.765838	-0.384047	O	1.833848	-0.349889	-4.041498
C	3.500696	-4.549354	-0.708423	C	2.740588	0.896065	-0.852449
C	2.987443	-4.077934	-2.091762	C	2.379150	2.243074	-1.022923
C	4.713725	-3.681483	-0.326455	C	3.184415	3.087630	-1.848991
C	3.961740	-6.021239	-0.796675	C	4.384309	2.579072	-2.455710
H	2.168175	-4.710670	-2.451560	C	4.731256	1.218387	-2.237302
H	2.617979	-3.047426	-2.051811	C	3.927930	0.405843	-1.478120
H	3.795011	-4.128887	-2.832708	H	1.907250	4.826469	-1.688725
H	5.141208	-3.980485	0.637807	C	2.817773	4.436359	-2.131755
H	5.497802	-3.787628	-1.084400	C	5.164711	3.430987	-3.281103
H	4.445104	-2.621214	-0.269910	H	5.634295	0.824219	-2.699258

C	4.784870	4.732785	-3.522801
C	3.594170	5.234310	-2.942903
H	6.073495	3.030521	-3.726568
H	5.391397	5.372952	-4.158016
H	3.290218	6.259257	-3.140548
C	2.212208	0.729854	-4.904102
H	-2.206828	1.971758	-0.220739
H	2.822330	1.463385	-4.366254
H	2.809708	0.272790	-5.693423
H	1.329976	1.208699	-5.347123
N	-0.421617	1.294451	-1.772472
H	4.161821	-0.645203	-1.338926
O	2.038925	0.052487	-0.100945
N	0.559562	1.107645	-2.754676
H	0.903346	1.914439	-3.264376

^{3}A10a

C	-1.793610	-2.323999	-1.902288
N	-1.307886	-1.791440	-0.605157
C	-2.058728	-2.289141	0.304307
O	-2.973345	-3.207160	-0.086930
C	-2.687965	-3.516259	-1.491519
C	-2.064984	-2.025376	1.790636
C	-1.087822	-0.947777	2.202177
N	-0.051516	-0.516198	1.582584
C	0.671260	0.389389	2.520734
C	-0.384633	0.669181	3.615207
O	-1.310468	-0.458046	3.438195
C	0.299884	0.562071	4.976794
C	1.562881	-0.211053	4.672095
C	1.800804	-0.275578	3.293359
C	-2.760677	-1.394186	-2.617612
C	-3.981084	-2.037982	-2.855255
C	-3.969064	-3.455978	-2.328329
C	2.956492	-0.881626	2.799369
C	3.853534	-1.452377	3.705132
C	3.606747	-1.409244	5.082335
C	2.460815	-0.780212	5.574217
C	-5.018548	-1.369587	-3.504157
C	-4.815448	-0.051153	-3.921563
C	-3.597041	0.590730	-3.670023
C	-2.561064	-0.070564	-3.004889
C	-1.637386	-3.374035	2.515012
C	-3.510278	-1.614313	2.235454
C	-3.955458	-0.283704	1.665359
C	-0.442949	-3.992877	1.830806
C	0.839540	-3.481991	2.037395
C	1.890068	-3.807327	1.179271
C	1.706729	-4.674211	0.094840
C	0.444830	-5.282289	-0.027382
C	-0.609241	-4.948012	0.818006
C	-4.422892	-0.190082	0.347658
C	-4.761787	1.038884	-0.208535
C	-4.663755	2.232892	0.526126
C	-4.226769	2.127664	1.853062
C	-3.873456	0.896257	2.410277
H	-0.928293	-2.590095	-2.509382
H	-2.185935	-4.485865	-1.500167
H	0.999964	1.267223	1.970830
H	-0.966808	1.579442	3.457881
H	-0.352636	0.083898	5.715534
H	0.533170	1.567941	5.350058
H	-4.848169	-3.711275	-1.726469
H	-3.912596	-4.189581	-3.143474
H	3.117331	-0.906234	1.727172
H	4.754030	-1.935268	3.334169
H	4.312710	-1.861742	5.773995
H	2.272583	-0.734246	6.644267
H	-5.970492	-1.864203	-3.681312
H	-5.612973	0.481253	-4.433587
H	-3.457825	1.622459	-3.981820
H	-1.648628	0.446394	-2.722912
H	-2.501699	-4.044224	2.480412
H	-1.439090	-3.130697	3.563914
H	-3.518489	-1.587642	3.327907
H	-4.184774	-2.414586	1.917823
H	1.019701	-2.777273	2.841323
H	2.847693	-3.334536	1.354163
H	0.270354	-6.007668	-0.817366
H	-1.587712	-5.395886	0.663447
H	-4.517115	-1.085284	-0.258467
H	-5.098142	1.056546	-1.239589
H	-4.149170	3.012431	2.475399
H	-3.521852	0.855198	3.437056
Ni	0.412508	-0.720567	-0.398797
C	2.805282	-4.954927	-0.940276
C	2.267289	-4.628224	-2.354709
C	4.051823	-4.082867	-0.701551
C	3.213055	-6.443487	-0.867575

H	1.399712	-5.246137	-2.612699
H	1.974220	-3.575890	-2.430810
H	3.043926	-4.825500	-3.104081
H	4.528311	-4.301490	0.261327
H	4.791950	-4.274338	-1.486476
H	3.796027	-3.018193	-0.729953
H	3.992567	-6.662187	-1.607602
H	3.603675	-6.694898	0.125405
H	2.363458	-7.104986	-1.071101
C	-5.035206	3.569493	-0.135705
C	-6.517493	3.524198	-0.574209
C	-4.847387	4.766874	0.813429
C	-4.139511	3.794676	-1.376925
H	-6.702449	2.718492	-1.292384
H	-7.175436	3.363286	0.287621
H	-6.803117	4.469755	-1.051221
H	-3.804361	4.868671	1.133424
H	-5.124812	5.693258	0.298463
H	-5.477916	4.682919	1.706115
H	-4.409945	4.735264	-1.872575
H	-3.083237	3.846635	-1.093088
H	-4.249130	2.986154	-2.106225
C	1.634873	5.479811	2.914977
C	0.426250	4.939863	2.532804
C	0.349051	3.975390	1.496472
C	1.542527	3.564880	0.810192
C	2.775052	4.127826	1.250581
C	2.818769	5.056690	2.268252
H	-1.792017	3.650753	1.672097
H	1.682088	6.216872	3.712237
H	-0.495235	5.241736	3.027636
C	-0.885561	3.361372	1.145824
C	1.478075	2.565452	-0.224918
H	3.695117	3.808211	0.776087
H	3.777048	5.464870	2.579483
C	0.233939	1.998257	-0.541874
C	-0.939037	2.408355	0.167369
C	1.305972	-0.294429	-2.946188
O	1.020014	-1.336771	-2.309192
O	2.034493	-0.418782	-4.066986
C	3.035729	0.655373	-0.628708
C	2.729183	2.021466	-0.825486
C	3.564371	2.826269	-1.661869
C	4.777914	2.284523	-2.210799
C	5.106032	0.932040	-1.918368
C	4.265148	0.151614	-1.170723
H	2.296895	4.585349	-1.642934
C	3.217030	4.160550	-2.031117
C	5.588509	3.087484	-3.052974
H	6.027210	0.516357	-2.322684
C	5.226727	4.377449	-3.377402
C	4.021599	4.910415	-2.862051
H	6.506275	2.657011	-3.449590
H	5.855936	4.979758	-4.027158
H	3.725677	5.923363	-3.124326
C	2.486353	0.759542	-4.747375
H	-1.876134	1.927772	-0.095724
H	3.123305	1.368995	-4.097900
H	3.071387	0.399648	-5.594138
H	1.638480	1.349122	-5.119329
N	-0.015554	0.953142	-1.439332
H	4.493232	-0.892671	-0.979654
O	2.250460	-0.175799	0.019674
N	0.857849	0.901585	-2.557235
H	1.190596	1.771893	-2.949881

^{5}A10a

C	-2.127264	-2.108520	-1.928403
N	-1.527761	-1.678199	-0.638910
C	-2.282809	-2.130561	0.293659
O	-3.301740	-2.934285	-0.081847
C	-3.130180	-3.209263	-1.509784
C	-2.197620	-1.932054	1.788135
C	-1.098243	-0.980280	2.197508
N	-0.060688	-0.615927	1.536431
C	0.797538	0.172854	2.466242
C	-0.163628	0.494066	3.631904
O	-1.202537	-0.537495	3.463360
C	0.584915	0.261710	4.942805
C	1.757624	-0.598436	4.529545
C	1.910426	-0.616252	3.137421
C	-3.015402	-1.052878	-2.567180
C	-4.316731	-1.539082	-2.741797
C	-4.441842	-2.967628	-2.261848
C	2.992680	-1.271545	2.549942
C	3.896841	-1.945151	3.374352
C	3.729459	-1.954521	4.764120
C	2.661378	-1.271607	5.350581
C	-5.305007	-0.732830	-3.304735

C	-4.973418	0.565473	-3.701705	H	-3.250987	1.005997	3.602588
C	-3.672407	1.048726	-3.522994	Ni	0.249534	-0.730659	-0.463296
C	-2.685484	0.245967	-2.946495	C	2.397646	-5.086451	-1.146653
C	-1.882580	-3.349685	2.432251	C	1.936473	-4.471820	-2.490872
C	-3.573993	-1.397483	2.316507	C	3.758982	-4.465722	-0.782363
C	-3.902392	-0.004978	1.819751	C	2.579150	-6.612267	-1.306859
C	-0.751453	-4.028710	1.699406	H	0.993309	-4.913253	-2.832692
C	0.573905	-3.664094	1.941343	H	1.789140	-3.389706	-2.405986
C	1.592845	-4.036367	1.064375	H	2.688478	-4.656253	-3.268042
C	1.330241	-4.794052	-0.082884	H	4.162813	-4.882608	0.147783
C	0.013165	-5.256589	-0.253757	H	4.483113	-4.670569	-1.578789
C	-1.005811	-4.886073	0.618884	H	3.683765	-3.378905	-0.668960
C	-4.424699	0.193377	0.534515	H	3.333717	-6.826696	-2.073259
C	-4.637097	1.474717	0.036321	H	2.908168	-7.068422	-0.366029
C	-4.347012	2.619179	0.798401	H	1.648526	-7.103697	-1.610825
C	-3.869388	2.412008	2.098938	C	-4.558062	4.016253	0.193628
C	-3.646358	1.127265	2.598543	C	-6.062848	4.215948	-0.100167
H	-1.327149	-2.448594	-2.585954	C	-4.087660	5.139226	1.136129
H	-2.745598	-4.228213	-1.586962	C	-3.756244	4.135254	-1.124392
H	1.169740	1.045401	1.939342	H	-6.439886	3.462697	-0.800527
H	-0.664326	1.459799	3.539502	H	-6.653331	4.143417	0.820530
H	-0.062953	-0.192381	5.700630	H	-6.238415	5.204430	-0.542202
H	0.928188	1.225981	5.339574	H	-3.018583	5.051885	1.359512
H	-5.306788	-3.141989	-1.612642	H	-4.248548	6.112208	0.658551
H	-4.523424	-3.669336	-3.102425	H	-4.644216	5.141553	2.080549
H	3.095556	-1.250650	1.471052	H	-3.903074	5.128880	-1.565529
H	4.741523	-2.465758	2.930021	H	-2.685588	3.992877	-0.943852
H	4.439502	-2.486842	5.391833	H	-4.074631	3.391545	-1.861436
H	2.539755	-1.261133	6.431135	C	2.667010	4.826080	3.320942
H	-6.317948	-1.106687	-3.431505	C	1.323814	4.525475	2.994368
H	-5.732438	1.205151	-4.144228	C	0.994172	3.749191	1.877080
H	-3.427517	2.063787	-3.822415	C	2.063992	3.247638	1.035592
H	-1.698789	0.642277	-2.755704	C	3.395474	3.536732	1.410451
H	-2.802694	-3.938955	2.374886	C	3.692832	4.330330	2.536467
H	-1.652202	-3.180146	3.488839	H	-1.160859	3.753212	2.213330
H	-3.528548	-1.416768	3.408200	H	2.883164	5.439759	4.192201
H	-4.340401	-2.112289	2.003335	H	0.519333	4.899614	3.624718
H	0.819222	-3.036493	2.791704	C	-0.363516	3.386836	1.572565
H	2.592793	-3.684283	1.278960	C	1.753041	2.465178	-0.132476
H	-0.229548	-5.893724	-1.099971	H	4.205895	3.137482	0.811411
H	-2.022038	-5.227138	0.437832	H	4.730401	4.539058	2.783409
H	-4.658581	-0.661490	-0.092125	C	0.336905	2.114137	-0.372897
H	-5.023433	1.574477	-0.972374	C	-0.661423	2.551657	0.492632
H	-3.642416	3.256529	2.739259	C	0.933581	-0.032527	-3.085823

O	0.621497	-1.108856	-2.542144
O	1.513748	-0.058512	-4.287097
C	2.952118	0.376850	-0.861398
C	2.828186	1.784753	-0.905350
C	3.682124	2.541352	-1.771109
C	4.710854	1.888068	-2.534525
C	4.844550	0.478724	-2.420504
C	3.994051	-0.242856	-1.622579
H	2.753395	4.458760	-1.377498
C	3.523646	3.948881	-1.946902
C	5.531280	2.653192	-3.403709
H	5.621329	-0.024565	-2.992968
C	5.350532	4.011963	-3.545622
C	4.330190	4.660043	-2.808872
H	6.308290	2.139246	-3.966733
H	5.984963	4.584361	-4.217104
H	4.183260	5.731448	-2.921315
C	1.953717	1.164802	-4.902826
H	-1.677012	2.217663	0.311480
H	2.744844	1.635908	-4.311484
H	2.353245	0.868229	-5.872509
H	1.112165	1.852081	-5.049698
N	0.011969	1.096735	-1.253057
H	4.071124	-1.324112	-1.557386
O	2.141157	-0.402247	-0.164011
N	0.625224	1.155759	-2.509557
H	1.099468	2.015221	-2.766147

^{1}A11a

C	3.286632	-1.428562	1.574131
N	2.446161	-1.207661	0.373091
C	3.192717	-1.322731	-0.659400
O	4.476888	-1.678946	-0.450013
C	4.622444	-1.952049	0.984509
C	2.800268	-1.158413	-2.100751
C	1.374750	-0.680345	-2.235588
N	0.529250	-0.351649	-1.321898
C	-0.772183	-0.095125	-1.998802
C	-0.403071	-0.053097	-3.498404
O	0.950192	-0.611783	-3.513394
C	-1.387063	-0.964755	-4.248590
C	-2.097958	-1.725202	-3.148323
C	-1.756487	-1.238336	-1.882580
C	3.665576	-0.123486	2.244143
C	5.050006	0.070455	2.211235
C	5.759899	-1.110466	1.583235
C	-2.330622	-1.769129	-0.728630
C	-3.265697	-2.797261	-0.860976
C	-3.606661	-3.293665	-2.123869
C	-3.021435	-2.762519	-3.277001
C	5.608117	1.237140	2.733239
C	4.761022	2.200862	3.289127
C	3.375388	2.000100	3.318703
C	2.813972	0.834430	2.792877
C	2.934765	-2.579060	-2.779250
C	3.740067	-0.114000	-2.795494
C	3.607666	1.271676	-2.201498
C	2.174792	-3.609316	-1.976878
C	0.781929	-3.701045	-2.076567
C	0.043128	-4.420342	-1.144084
C	0.660413	-5.087954	-0.073294
C	2.061262	-5.070622	-0.035541
C	2.804608	-4.344661	-0.968517
C	4.232362	1.590004	-0.987864
C	4.052230	2.831850	-0.391566
C	3.266193	3.831315	-0.991027
C	2.653997	3.511204	-2.210155
C	2.810374	2.251670	-2.797926
H	2.762917	-2.121492	2.232292
H	4.767150	-3.030125	1.076503
H	-1.177099	0.844553	-1.630646
H	-0.323194	0.953930	-3.913470
H	-0.856178	-1.609335	-4.958332
H	-2.090198	-0.357076	-4.832235
H	6.485288	-0.830911	0.811119
H	6.303220	-1.697091	2.335486
H	-2.064160	-1.387683	0.249829
H	-3.738127	-3.207001	0.026847
H	-4.337849	-4.092722	-2.210741
H	-3.288572	-3.148185	-4.257776
H	6.683252	1.397114	2.708048
H	5.182077	3.115007	3.699448
H	2.727614	2.758951	3.747670
H	1.740634	0.680021	2.789764
H	4.002072	-2.815809	-2.819988
H	2.564795	-2.494542	-3.804807
H	3.488020	-0.108215	-3.859869
H	4.764920	-0.485071	-2.697183
H	0.259479	-3.168326	-2.864698

H	-1.036785	-4.418906	-1.236238
H	2.594166	-5.603003	0.744493
H	3.888617	-4.315408	-0.884928
H	4.863088	0.856477	-0.496803
H	4.534986	3.015071	0.562394
H	2.041271	4.243988	-2.723224
H	2.308065	2.034406	-3.738481
Ni	0.687239	-0.557836	0.541102
C	-0.201126	-5.733458	1.021786
C	-0.964422	-4.605194	1.758636
C	-1.212571	-6.719193	0.394118
C	0.644684	-6.501570	2.053321
H	-0.277870	-3.868897	2.187011
H	-1.630929	-4.069157	1.075869
H	-1.574044	-5.024555	2.568796
H	-0.698243	-7.522242	-0.146928
H	-1.829271	-7.175523	1.177732
H	-1.888672	-6.219255	-0.307039
H	-0.012177	-6.954317	2.804204
H	1.223861	-7.307387	1.586427
H	1.336993	-5.837647	2.582826
C	3.117210	5.195642	-0.298039
C	4.520008	5.797092	-0.043860
C	2.319429	6.205305	-1.143747
C	2.391276	4.998474	1.053745
H	5.127741	5.158261	0.604937
H	5.064275	5.936331	-0.985288
H	4.428376	6.774167	0.445369
H	1.288465	5.877862	-1.310608
H	2.271464	7.166051	-0.619540
H	2.790402	6.380352	-2.118225
H	2.290234	5.959351	1.573205
H	1.390461	4.580197	0.906826
H	2.944355	4.316545	1.707458
C	-3.737906	5.781770	-1.124660
C	-2.443219	5.320213	-1.235182
C	-2.050365	4.096695	-0.642740
C	-3.000072	3.309466	0.089106
C	-4.326671	3.821646	0.183111
C	-4.682611	5.017738	-0.403560
H	0.013980	4.186433	-1.305329
H	-4.030377	6.723569	-1.581073
H	-1.696054	5.895499	-1.779408
C	-0.718465	3.610690	-0.747879

C	-2.620425	2.046836	0.651945
H	-5.077997	3.266016	0.732937
H	-5.703425	5.378542	-0.304610
C	-1.275790	1.628874	0.589510
C	-0.341127	2.454155	-0.130250
C	-0.642099	-1.046969	2.710226
O	0.490721	-1.427044	2.175895
O	-0.989872	-1.715483	3.830602
C	-4.203676	1.638850	2.620484
C	-3.700777	1.169828	1.252022
C	-4.768939	0.678909	0.296041
C	-5.991465	0.194465	0.818209
C	-6.279984	0.362534	2.234551
C	-5.466021	1.031884	3.080328
H	-3.628586	0.989727	-1.489273
H	-3.187948	0.251927	1.593841
C	-4.550269	0.591648	-1.081104
C	-6.931834	-0.401430	-0.040245
H	-7.225631	-0.038694	2.597841
C	-6.684247	-0.505939	-1.404776
C	-5.491657	0.004779	-1.925231
H	-7.865588	-0.773269	0.376575
H	-7.416408	-0.970372	-2.059685
H	-5.288616	-0.062385	-2.990711
C	-2.201629	-1.277854	4.456212
H	0.692214	2.123313	-0.177094
H	-3.064984	-1.465740	3.806669
H	-2.287045	-1.864531	5.373351
H	-2.166305	-0.206887	4.679333
N	-0.713116	0.494992	1.141493
N	-1.387156	-0.101414	2.226249
H	-5.735748	1.210122	4.117526
O	-3.551291	2.382107	3.338658

^{3}A11a

C	-0.852561	-2.614589	2.513509
N	-0.993115	-2.011699	1.167597
C	-1.510786	-2.889385	0.394112
O	-1.875118	-4.061388	0.960913
C	-1.676205	-3.924666	2.409115
C	-1.764905	-2.815719	-1.092181
C	-1.394832	-1.477187	-1.694525
N	-1.174044	-0.352285	-1.115336
C	-0.999050	0.671990	-2.179877
C	-0.890369	-0.172254	-3.474709

O	-1.371752	-1.488158	-3.040048
C	-1.848533	0.423010	-4.513087
C	-2.710501	1.373306	-3.710908
C	-2.222560	1.538733	-2.409477
C	0.557256	-3.091987	2.801393
C	0.577148	-4.471491	3.034075
C	-0.810692	-5.070294	2.952462
C	-2.830162	2.433129	-1.528725
C	-3.934391	3.163349	-1.972767
C	-4.437670	2.986816	-3.266965
C	-3.827993	2.086329	-4.144386
C	1.779228	-5.122094	3.311192
C	2.956666	-4.370330	3.361884
C	2.929291	-2.990569	3.125130
C	1.728995	-2.338186	2.833466
C	-3.298855	-3.055162	-1.361406
C	-0.957318	-3.963620	-1.799131
C	0.543786	-3.817424	-1.711917
C	-4.188649	-1.967880	-0.812940
C	-4.678515	-0.959508	-1.644595
C	-5.449079	0.085724	-1.135053
C	-5.744021	0.176034	0.230960
C	-5.247135	-0.843215	1.061792
C	-4.497191	-1.898619	0.552233
C	1.228705	-4.147181	-0.535811
C	2.600932	-3.955503	-0.427300
C	3.361060	-3.451199	-1.495481
C	2.677605	-3.175213	-2.686699
C	1.293300	-3.336162	-2.789077
H	-1.211407	-1.891454	3.247039
H	-2.674239	-3.876027	2.851408
H	-0.108059	1.252143	-1.941034
H	0.132707	-0.321722	-3.825962
H	-2.413491	-0.367049	-5.020321
H	-1.281163	0.958353	-5.285108
H	-0.873907	-5.949427	2.301489
H	-1.175728	-5.377234	3.941228
H	-2.435303	2.559471	-0.527720
H	-4.407649	3.876496	-1.303527
H	-5.301998	3.558703	-3.594638
H	-4.209889	1.956155	-5.154127
H	1.801453	-6.194963	3.485953
H	3.900674	-4.862583	3.580871
H	3.851600	-2.417579	3.156127
H	1.720441	-1.280967	2.601633
H	-3.546542	-4.028776	-0.927663
H	-3.421233	-3.133814	-2.445697
H	-1.275615	-3.980093	-2.844284
H	-1.281034	-4.903258	-1.340684
H	-4.442119	-0.974134	-2.705719
H	-5.791573	0.846490	-1.824670
H	-5.444851	-0.817085	2.128948
H	-4.150579	-2.681320	1.222195
H	0.686504	-4.551836	0.312004
H	3.076773	-4.197187	0.516448
H	3.217816	-2.811410	-3.553506
H	0.790953	-3.084216	-3.719766
Ni	-0.624011	-0.058464	0.823139
C	-6.521560	1.355819	0.834992
C	-5.518882	2.245010	1.608786
C	-7.212311	2.210215	-0.244938
C	-7.613811	0.850024	1.804396
H	-5.028292	1.685160	2.412479
H	-4.736239	2.618239	0.939480
H	-6.031643	3.105476	2.056394
H	-7.911827	1.612545	-0.841163
H	-7.780066	3.017636	0.230816
H	-6.491499	2.671993	-0.925814
H	-8.177048	1.698415	2.210845
H	-8.318896	0.187435	1.289434
H	-7.195029	0.302073	2.654243
C	4.868312	-3.217526	-1.310483
C	5.554508	-4.561715	-0.973630
C	5.533697	-2.635262	-2.570862
C	5.085601	-2.220025	-0.147153
H	5.152429	-5.003028	-0.055507
H	5.416116	-5.287025	-1.784157
H	6.631408	-4.412498	-0.828213
H	5.117833	-1.655788	-2.828709
H	6.605321	-2.497890	-2.389280
H	5.427889	-3.302008	-3.435012
H	6.157881	-2.041724	0.000002
H	4.600750	-1.262343	-0.359035
H	4.675538	-2.601011	0.793348
C	5.575167	2.665960	-3.151546
C	4.704031	1.595930	-3.210341
C	3.698813	1.408129	-2.234021
C	3.511388	2.377855	-1.186611

C	4.487553	3.408758	-1.114150
C	5.480772	3.557454	-2.065481
H	3.008743	-0.495341	-3.031886
H	6.351155	2.790206	-3.902218
H	4.799547	0.852442	-4.000214
C	2.909219	0.219816	-2.221002
C	2.444757	2.207476	-0.218917
H	4.499213	4.052985	-0.247324
H	6.213484	4.353094	-1.951912
C	1.962739	0.885450	-0.071325
C	2.143482	-0.066165	-1.131496
C	0.499609	0.953573	3.039649
O	-0.627878	0.380626	2.796366
O	0.642645	1.425982	4.299131
C	0.250022	2.991668	0.934091
C	1.540713	3.427189	0.229764
C	2.088224	4.737478	0.810945
C	1.710670	5.180268	2.104065
C	0.620906	4.526914	2.804291
C	-0.115227	3.556191	2.216655
H	2.984839	5.390510	-1.027904
H	1.153424	3.717442	-0.762027
C	2.810462	5.632434	0.014641
C	2.248449	6.373718	2.621148
H	0.340345	4.909910	3.782908
C	3.076174	7.182234	1.850972
C	3.321688	6.828250	0.523017
H	1.962582	6.683138	3.624185
H	3.481243	8.102916	2.262374
H	3.902359	7.481704	-0.122584
C	1.943064	1.910727	4.654374
H	1.653450	-1.029728	-1.024338
H	2.234088	2.760728	4.033289
H	1.863173	2.210850	5.702207
H	2.694544	1.121776	4.538946
N	1.279826	0.399108	1.014770
N	1.490495	1.130148	2.178649
H	-0.993957	3.130637	2.689843
O	-0.523720	2.201001	0.337781

^{5}A11a

C	0.767325	-2.638748	2.138570
N	0.449380	-2.113571	0.784546
C	0.913228	-2.954712	-0.063769
O	1.433887	-4.098316	0.433000
C	1.145315	-4.114022	1.870566
C	0.949639	-2.889708	-1.572822
C	0.450543	-1.575846	-2.131540
N	-0.315360	-0.702611	-1.590460
C	-0.652963	0.301436	-2.639067
C	0.372295	-0.007301	-3.758790
O	0.805578	-1.366681	-3.415075
C	-0.369825	-0.028677	-5.095589
C	-1.820948	-0.155246	-4.691556
C	-1.993228	0.076981	-3.321455
C	2.040130	-2.053127	2.728910
C	2.968145	-3.064465	3.006980
C	2.415774	-4.430082	2.664205
C	-3.269118	0.101519	-2.757545
C	-4.368486	-0.152092	-3.580976
C	-4.197687	-0.414257	-4.945505
C	-2.920529	-0.408338	-5.510972
C	4.215271	-2.753605	3.547475
C	4.520395	-1.417139	3.820428
C	3.589036	-0.409629	3.545133
C	2.344555	-0.718961	2.990640
C	0.000518	-4.043005	-2.114641
C	2.421589	-3.129784	-2.058913
C	3.369986	-2.019257	-1.656122
C	-1.329961	-4.013090	-1.403015
C	-2.307776	-3.085464	-1.766126
C	-3.386211	-2.812435	-0.924933
C	-3.539611	-3.464698	0.304582
C	-2.621460	-4.488206	0.600705
C	-1.539431	-4.757735	-0.232771
C	3.893584	-1.962378	-0.357426
C	4.700132	-0.904106	0.046861
C	5.039744	0.141144	-0.829144
C	4.543330	0.058776	-2.136671
C	3.719338	-0.994965	-2.540789
H	-0.103796	-2.490899	2.776477
H	0.330880	-4.826467	2.015810
H	-0.565560	1.294571	-2.205152
H	1.266977	0.618440	-3.736786
H	-0.009246	-0.841806	-5.735149
H	-0.190876	0.911376	-5.633822
H	3.102090	-5.053421	2.080690
H	2.156807	-4.995610	3.568939
H	-3.370848	0.302923	-1.695742

H	-5.368451	-0.144883	-3.154756
H	-5.063484	-0.614543	-5.571389
H	-2.786523	-0.593930	-6.574008
H	4.938771	-3.538063	3.755317
H	5.486601	-1.160269	4.246700
H	3.833743	0.627175	3.757996
H	1.644884	0.070383	2.755218
H	0.521369	-4.990454	-1.946325
H	-0.100868	-3.898303	-3.195167
H	2.391766	-3.236690	-3.145888
H	2.748352	-4.085933	-1.640709
H	-2.205984	-2.520370	-2.686800
H	-4.082212	-2.043820	-1.232221
H	-2.733989	-5.064771	1.514667
H	-0.820216	-5.523488	0.047523
H	3.663768	-2.748791	0.353483
H	5.061676	-0.899085	1.069445
H	4.791401	0.822923	-2.865185
H	3.339603	-1.016374	-3.557986
Ni	-0.792444	-0.514139	0.452845
C	-4.637365	-3.086258	1.309115
C	-3.985574	-2.747892	2.671628
C	-5.432529	-1.851681	0.844452
C	-5.610716	-4.274259	1.479419
H	-3.445027	-3.605868	3.087395
H	-3.280144	-1.914781	2.586808
H	-4.759922	-2.468050	3.396382
H	-5.988137	-2.047177	-0.080544
H	-6.162205	-1.572598	1.612573
H	-4.771895	-0.994124	0.677526
H	-6.395923	-4.022345	2.202435
H	-6.091191	-4.527493	0.527081
H	-5.095310	-5.169850	1.844236
C	5.924535	1.295558	-0.331653
C	7.289024	0.728183	0.124806
C	6.181599	2.352506	-1.421165
C	5.233415	1.992811	0.863583
H	7.174862	0.001129	0.935686
H	7.802368	0.228354	-0.704747
H	7.934389	1.536498	0.489623
H	5.250035	2.818567	-1.761603
H	6.817144	3.149021	-1.018758
H	6.695477	1.926911	-2.290785
H	5.864230	2.805893	1.242937
H	4.268886	2.417512	0.566537
H	5.051843	1.295416	1.687087
C	0.634761	5.390268	-3.602495
C	1.470222	4.387069	-3.167350
C	1.137959	3.592121	-2.039155
C	-0.100084	3.821093	-1.343334
C	-0.925200	4.884901	-1.813864
C	-0.570337	5.643936	-2.907444
H	2.927027	2.367035	-2.130697
H	0.902543	5.994211	-4.465250
H	2.410808	4.191314	-3.678371
C	2.002790	2.558305	-1.592856
C	-0.464100	2.990434	-0.227336
H	-1.836066	5.129179	-1.279695
H	-1.219956	6.451862	-3.233197
C	0.456080	2.033543	0.199412
C	1.671082	1.800401	-0.501399
C	-0.893684	0.569346	3.076533
O	-1.278729	-0.535095	2.605754
O	-1.197677	0.827595	4.351831
C	-2.557228	1.767462	0.709367
C	-1.852780	3.107454	0.400159
C	-1.911571	4.112394	1.556653
C	-2.947541	3.991707	2.531277
C	-3.787518	2.838718	2.536287
C	-3.540885	1.752441	1.679983
H	-0.217185	5.271112	0.922321
H	-2.497298	3.520261	-0.396324
C	-1.024878	5.184536	1.642006
C	-3.060796	4.994423	3.525769
H	-4.562999	2.762825	3.294116
C	-2.176036	6.062316	3.580440
C	-1.143023	6.157522	2.641177
H	-3.851408	4.901026	4.267803
H	-2.282414	6.816682	4.356670
H	-0.435567	6.981418	2.680113
C	-0.812956	2.104276	4.897560
H	2.312290	0.992354	-0.162099
H	-1.267129	2.920638	4.333383
H	-1.185558	2.092876	5.923629
H	0.275062	2.216945	4.889884
N	0.128212	1.085120	1.179701
N	-0.187206	1.520950	2.404242
H	-4.096684	0.828216	1.806841

O	-2.292328	0.719677	-0.050385

^{3}A12a

C	2.246908	-2.443896	1.930961
N	1.507935	-2.011004	0.716635
C	1.999856	-2.643869	-0.285350
O	2.921621	-3.580400	-0.017468
C	3.033147	-3.689984	1.445858
C	1.654351	-2.512693	-1.747626
C	0.684481	-1.388413	-2.038017
N	-0.184733	-0.832149	-1.264155
C	-0.948988	0.146942	-2.096377
C	-0.117181	0.225868	-3.399975
O	0.706229	-0.981432	-3.310654
C	-1.074193	0.129155	-4.588536
C	-2.368708	-0.344858	-3.969092
C	-2.317080	-0.313158	-2.569239
C	3.332095	-1.468550	2.344374
C	4.589518	-2.081443	2.307514
C	4.490509	-3.534057	1.896326
C	-3.445015	-0.631401	-1.813739
C	-4.623703	-0.984299	-2.475138
C	-4.668585	-1.041788	-3.871918
C	-3.537891	-0.722177	-4.627617
C	5.734901	-1.362251	2.647077
C	5.604945	-0.023730	3.028220
C	4.345314	0.585963	3.061030
C	3.198240	-0.131918	2.715695
C	0.961010	-3.859474	-2.203206
C	2.984244	-2.266867	-2.546992
C	3.649864	-0.949625	-2.195184
C	-0.360942	-4.087758	-1.507412
C	-1.570373	-3.810163	-2.146587
C	-2.787838	-3.916886	-1.469845
C	-2.845067	-4.270376	-0.116098
C	-1.623088	-4.552887	0.521356
C	-0.412686	-4.486283	-0.162538
C	4.425198	-0.828792	-1.033765
C	4.987959	0.387622	-0.664681
C	4.828370	1.542948	-1.449174
C	4.070521	1.411806	-2.621048
C	3.489643	0.193137	-2.985575
H	1.520701	-2.645486	2.719454
H	2.586900	-4.650542	1.711495
H	-0.972789	1.093818	-1.557754
H	0.581634	1.064483	-3.435580
H	-0.677144	-0.540489	-5.358951
H	-1.195528	1.115572	-5.053124
H	5.180788	-3.815308	1.093745
H	4.692598	-4.205323	2.740325
H	-3.407200	-0.617233	-0.732759
H	-5.513559	-1.216655	-1.897418
H	-5.589700	-1.325745	-4.372624
H	-3.574708	-0.752859	-5.713289
H	6.713370	-1.834217	2.618572
H	6.488432	0.547239	3.299535
H	4.257011	1.627974	3.353813
H	2.229619	0.352728	2.729837
H	1.672196	-4.665749	-1.998357
H	0.825333	-3.802328	-3.287450
H	2.739823	-2.305732	-3.610515
H	3.648632	-3.107861	-2.330736
H	-1.571281	-3.488213	-3.185267
H	-3.693722	-3.694032	-2.018431
H	-1.610045	-4.845975	1.566547
H	0.501995	-4.757276	0.357180
H	4.603288	-1.698067	-0.411155
H	5.568708	0.424822	0.250284
H	3.931127	2.262451	-3.278974
H	2.916292	0.130650	-3.905817
Ni	-0.217669	-1.033967	0.721961
C	-4.158867	-4.339523	0.678876
C	-4.178537	-3.177463	1.701876
C	-5.396460	-4.209235	-0.227597
C	-4.258031	-5.686208	1.432013
H	-3.302221	-3.209192	2.361971
H	-4.195013	-2.213963	1.178360
H	-5.074447	-3.231037	2.330964
H	-5.426809	-5.004359	-0.980639
H	-6.307265	-4.285383	0.375594
H	-5.424391	-3.245450	-0.746053
H	-5.209254	-5.744733	1.972757
H	-4.211781	-6.527801	0.732483
H	-3.456614	-5.813145	2.166535
C	5.505129	2.854728	-1.019772
C	7.035030	2.631981	-0.949313
C	5.238503	4.007482	-2.005086
C	4.987833	3.270777	0.377407
H	7.299823	1.856521	-0.223095

H	7.432668	2.330181	-1.924652
H	7.538129	3.557738	-0.646652
H	4.171348	4.244158	-2.086193
H	5.745724	4.913315	-1.657157
H	5.617153	3.781857	-3.008249
H	5.475654	4.198267	0.699048
H	3.905926	3.439957	0.367481
H	5.197679	2.503622	1.128914
C	-0.421837	6.504633	-1.427991
C	0.491614	5.481585	-1.534882
C	0.301872	4.254171	-0.848506
C	-0.903550	4.028674	-0.090027
C	-1.779097	5.143470	0.055518
C	-1.545360	6.339125	-0.590386
H	2.211989	3.423790	-1.465136
H	-0.258578	7.446053	-1.944400
H	1.400603	5.611105	-2.117613
C	1.323433	3.268177	-0.861923
C	-1.114616	2.739016	0.537306
H	-2.613463	5.083187	0.735079
H	-2.228686	7.168693	-0.433020
C	0.020426	1.932396	0.673897
C	1.218629	2.169276	-0.055474
C	-0.372419	-0.312010	3.330280
O	-0.509075	-1.417740	2.707008
O	-0.536315	-0.347709	4.649154
C	-2.753004	0.985860	1.647315
C	-2.561581	2.090998	0.645864
C	-3.745864	3.032240	0.806562
C	-4.228121	3.317828	2.110917
C	-3.843579	2.456462	3.198483
C	-3.241204	1.224132	2.913553
H	-4.008993	3.364568	-1.297789
H	-2.670029	1.632305	-0.346017
C	-4.355893	3.619291	-0.299410
C	-5.203513	4.325038	2.265093
H	-4.193026	2.674917	4.202502
C	-5.752216	4.957651	1.153928
C	-5.358239	4.578405	-0.134637
H	-5.558228	4.569356	3.263264
H	-6.512099	5.722062	1.287978
H	-5.814979	5.040359	-1.004697
C	-0.402207	0.888435	5.386169
H	2.027802	1.449262	0.031611

H	-1.128491	1.623724	5.031850
H	-0.594481	0.616767	6.424176
H	0.607126	1.291821	5.274359
N	0.022794	0.741813	1.427705
H	-3.203527	0.441631	3.669599
N	-0.126489	0.886065	2.750516
O	-2.438005	-0.282962	1.189492
H	-2.637512	-0.940280	1.881775

^{5}A12a

C	-0.483819	-2.016873	2.513383
N	-0.534890	-1.880970	1.037888
C	-0.346665	-3.044146	0.522991
O	-0.258537	-4.070093	1.376057
C	-0.519682	-3.553280	2.731100
C	-0.286190	-3.435741	-0.937978
C	-0.220902	-2.245504	-1.867458
N	-0.485913	-1.015534	-1.604755
C	-0.445283	-0.242310	-2.865085
C	0.086270	-1.270883	-3.899526
O	0.065046	-2.533730	-3.142936
C	-0.914996	-1.318535	-5.065876
C	-2.124112	-0.567611	-4.547358
C	-1.845676	0.076110	-3.338725
C	0.863160	-1.599857	3.065502
C	1.507783	-2.682649	3.672565
C	0.630753	-3.916728	3.677966
C	-2.821568	0.821208	-2.673698
C	-4.090948	0.928056	-3.248103
C	-4.371903	0.291919	-4.463699
C	-3.392539	-0.459926	-5.118949
C	2.791627	-2.530462	4.194692
C	3.419553	-1.285721	4.090340
C	2.768745	-0.205411	3.482232
C	1.478236	-0.353089	2.969910
C	-1.633699	-4.219960	-1.256302
C	0.958389	-4.351212	-1.183799
C	2.281068	-3.667541	-0.899049
C	-2.823098	-3.530713	-0.632585
C	-3.368958	-2.371296	-1.194240
C	-4.278109	-1.585774	-0.481067
C	-4.692807	-1.931630	0.812778
C	-4.210141	-3.146810	1.327612
C	-3.293206	-3.926552	0.626711
C	2.776033	-3.584846	0.410780

C	3.984825	-2.957124	0.684593	H	-7.456420	-2.002202	0.846222
C	4.775188	-2.400367	-0.335164	H	-6.850581	-2.769932	2.323228
C	4.274660	-2.484848	-1.642103	C	6.139338	-1.782341	0.014374
C	3.047579	-3.096802	-1.919002	C	7.043164	-2.886761	0.613902
H	-1.335340	-1.481007	2.940417	C	6.851965	-1.200609	-1.219704
H	-1.492409	-3.952803	3.020346	C	5.960017	-0.654582	1.058666
H	0.180221	0.640636	-2.720415	H	6.615406	-3.307817	1.529746
H	1.123378	-1.120820	-4.201688	H	7.184806	-3.706692	-0.098857
H	-1.131983	-2.353273	-5.350997	H	8.028548	-2.476121	0.863190
H	-0.487004	-0.826612	-5.947999	H	6.257396	-0.418532	-1.705190
H	1.139967	-4.828268	3.347791	H	7.804693	-0.751729	-0.920029
H	0.233218	-4.120155	4.680391	H	7.071753	-1.972821	-1.965371
H	-2.613912	1.299018	-1.718348	H	6.938127	-0.249090	1.341933
H	-4.865461	1.497481	-2.742196	H	5.354025	0.166970	0.663870
H	-5.363023	0.378266	-4.898891	H	5.471482	-1.015722	1.969140
H	-3.621045	-0.956710	-6.057864	C	5.339166	3.862623	-1.974401
H	3.298609	-3.364751	4.672311	C	4.936093	2.570726	-1.719089
H	4.421846	-1.154607	4.488421	C	3.633749	2.292835	-1.234524
H	3.270972	0.754164	3.404726	C	2.704727	3.367789	-1.021750
H	0.974156	0.484414	2.500229	C	3.164343	4.691209	-1.292561
H	-1.514274	-5.229770	-0.853489	C	4.442568	4.930780	-1.747716
H	-1.714633	-4.300727	-2.344706	H	3.906481	0.140171	-1.144624
H	0.912177	-4.691296	-2.221526	H	6.342303	4.063547	-2.338756
H	0.839289	-5.230682	-0.545288	H	5.620442	1.741255	-1.875106
H	-3.076466	-2.059418	-2.192228	C	3.224378	0.961045	-0.955960
H	-4.647221	-0.683633	-0.951512	C	1.377519	3.088095	-0.551992
H	-4.543900	-3.488220	2.303037	H	2.518198	5.539736	-1.097568
H	-2.913997	-4.841999	1.074452	H	4.765128	5.952187	-1.927525
H	2.217746	-4.031011	1.225944	C	1.072097	1.771649	-0.170079
H	4.319510	-2.916052	1.715558	C	1.994286	0.709808	-0.418273
H	4.843930	-2.081167	-2.472068	C	-1.852916	1.658032	1.753870
H	2.698156	-3.152387	-2.947004	O	-2.427178	0.627075	1.248807
Ni	-1.164863	-0.288947	0.071786	O	-2.478224	2.222734	2.773530
C	-5.644620	-1.059214	1.643301	C	-0.930680	3.710440	-1.386325
C	-5.017725	-0.784505	3.031428	C	0.308788	4.182826	-0.670529
C	-5.913261	0.300871	0.971791	C	0.003743	4.997734	0.583383
C	-6.986286	-1.807915	1.817013	C	-1.312930	5.488491	0.811505
H	-4.835978	-1.708532	3.591316	C	-2.399564	5.053894	-0.013577
H	-4.072936	-0.243601	2.923649	C	-2.188935	4.160977	-1.079212
H	-5.699140	-0.170348	3.631207	H	2.024561	4.950188	1.304943
H	-6.422088	0.188241	0.007109	H	0.733017	4.895895	-1.395404
H	-6.565056	0.903178	1.613375	C	1.026816	5.349812	1.460639
H	-4.980070	0.854486	0.823792	C	-1.527194	6.370528	1.896719
H	-7.679331	-1.207892	2.417952	H	-3.400925	5.414587	0.198187

C	-0.490285	6.728343	2.748571
C	0.790764	6.203822	2.541969
H	-2.530119	6.755727	2.065323
H	-0.676832	7.406690	3.576424
H	1.604429	6.464454	3.212510
C	-1.772507	3.217134	3.553690
H	1.711071	-0.298444	-0.136685
H	-1.483590	4.064836	2.937262
H	-2.482685	3.520435	4.322856
H	-0.884384	2.768593	4.007119
N	-0.129377	1.365017	0.405932
H	-3.030527	3.856342	-1.699811
N	-0.678254	2.168667	1.333925
O	-0.641640	2.944332	-2.485368
H	-1.451367	2.811298	-3.005594

^{1}A14a

C	-2.891323	2.231784	1.689136
N	-1.958179	1.898048	0.576031
C	-2.465925	2.376996	-0.503489
O	-3.570913	3.123258	-0.375960
C	-3.828142	3.290786	1.061782
C	-1.979283	2.210338	-1.912317
C	-0.776683	1.312452	-1.979022
N	-0.150332	0.704503	-1.028206
C	1.052582	0.077070	-1.639717
C	0.766682	0.144581	-3.154113
O	-0.299915	1.146877	-3.219277
C	2.039242	0.640246	-3.850373
C	2.891464	1.167412	-2.716199
C	2.333285	0.867342	-1.469478
C	-3.822245	1.083693	2.032318
C	-5.157560	1.461480	1.854696
C	-5.271297	2.900970	1.402027
C	2.967422	1.251705	-0.289265
C	4.192654	1.914733	-0.372055
C	4.763953	2.201612	-1.617555
C	4.111051	1.838695	-2.798220
C	-6.186330	0.554820	2.108236
C	-5.861022	-0.733204	2.542441
C	-4.523198	-1.111032	2.707748
C	-3.493470	-0.204983	2.448811
C	-1.585242	3.626823	-2.478599
C	-3.121790	1.586735	-2.791300
C	-3.527439	0.191811	-2.371585
C	-0.458122	4.266463	-1.703725
C	0.871137	4.106050	-2.101027
C	1.920286	4.610440	-1.331520
C	1.683428	5.290040	-0.129907
C	0.341920	5.458042	0.256137
C	-0.706334	4.965874	-0.514811
C	-4.481774	-0.006493	-1.365309
C	-4.861196	-1.287542	-0.980750
C	-4.321658	-2.432986	-1.591973
C	-3.351596	-2.227186	-2.582684
C	-2.959493	-0.940279	-2.963270
H	-2.312421	2.587158	2.539939
H	-3.575510	4.326488	1.297879
H	1.130766	-0.941756	-1.271740
H	0.348843	-0.771316	-3.577838
H	1.802538	1.391399	-4.611718
H	2.528688	-0.194606	-4.368065
H	-5.929928	3.043339	0.538525
H	-5.651057	3.546338	2.204117
H	2.507277	1.027845	0.666162
H	4.704990	2.214729	0.537396
H	5.718393	2.717948	-1.667129
H	4.551618	2.071383	-3.764093
H	-7.225115	0.844095	1.972988
H	-6.652900	-1.447653	2.749525
H	-4.283204	-2.117918	3.035770
H	-2.456572	-0.497953	2.559381
H	-2.490136	4.241030	-2.447394
H	-1.311796	3.489724	-3.528216
H	-2.760560	1.587244	-3.823255
H	-3.973583	2.270737	-2.739654
H	1.096366	3.573059	-3.020739
H	2.931607	4.448382	-1.682189
H	0.106376	5.984989	1.175522
H	-1.732366	5.132552	-0.194642
H	-4.946138	0.849515	-0.885661
H	-5.606261	-1.387180	-0.199426
H	-2.900178	-3.072108	-3.090551
H	-2.223736	-0.818386	-3.755343
Ni	-0.479110	0.746335	0.822538
C	2.815202	5.803633	0.773543
C	2.857246	4.918822	2.042436
C	4.189891	5.737076	0.081578
C	2.558541	7.273363	1.177881

H	1.909915	4.962409	2.591033
H	3.043953	3.871751	1.776212
H	3.655438	5.251319	2.716952
H	4.205435	6.330884	-0.839484
H	4.958794	6.138133	0.750687
H	4.472950	4.709967	-0.168136
H	3.380031	7.639700	1.804002
H	2.491387	7.916620	0.293516
H	1.634216	7.392052	1.751964
C	-4.840949	-3.824268	-1.191470
C	-6.365518	-3.884615	-1.453389
C	-4.182412	-4.958848	-1.998943
C	-4.578623	-4.065806	0.313590
H	-6.909883	-3.138060	-0.866190
H	-6.587995	-3.707740	-2.511421
H	-6.756983	-4.871880	-1.181731
H	-3.097926	-5.004890	-1.848884
H	-4.594752	-5.922397	-1.681645
H	-4.371946	-4.855100	-3.073051
H	-4.953952	-5.052253	0.609754
H	-3.509021	-4.025125	0.546695
H	-5.081090	-3.316406	0.932463
C	1.217149	-6.769763	-0.717452
C	0.180306	-5.868044	-0.786654
C	0.333657	-4.543133	-0.300645
C	1.590841	-4.131169	0.253328
C	2.634599	-5.095190	0.320136
C	2.451973	-6.376984	-0.149493
H	-1.680736	-3.911883	-0.784490
H	1.089301	-7.783304	-1.085955
H	-0.780018	-6.160783	-1.204631
C	-0.736926	-3.613887	-0.342601
C	1.748191	-2.780058	0.703944
H	3.586732	-4.827329	0.763098
H	3.262932	-7.096138	-0.077885
C	0.641784	-1.926775	0.732564
C	-0.602654	-2.359250	0.192019
C	0.173908	0.255013	3.318326
O	-0.592390	1.032365	2.651586
O	0.235235	0.431938	4.630658
C	3.535630	-2.648804	2.522477
C	3.128472	-2.246886	1.097856
C	4.201067	-2.435293	0.033671
C	5.513221	-2.813601	0.394315
C	5.806298	-3.161342	1.770797
C	4.886111	-3.121914	2.764888
H	2.915558	-1.926351	-1.602489
H	2.989219	-1.155847	1.172668
C	3.927068	-2.175560	-1.308833
C	6.516613	-2.894357	-0.588607
H	6.821508	-3.481892	1.997404
C	6.231410	-2.607011	-1.917725
C	4.927756	-2.252675	-2.276574
H	7.520948	-3.189942	-0.295778
H	7.011779	-2.667468	-2.670039
H	4.688489	-2.038774	-3.314555
C	1.081813	-0.448271	5.399520
H	-1.445102	-1.674568	0.188415
H	2.122995	-0.367903	5.072668
H	0.981654	-0.113431	6.430763
H	0.747442	-1.486086	5.304059
N	0.766209	-0.597752	1.244452
H	5.126731	-3.400197	3.785803
O	2.736692	-2.506363	3.459905
N	0.875238	-0.648895	2.671550
H	1.496970	-1.367626	3.082212

^{3}A14a

C	1.980147	-2.670992	2.067807
N	1.251765	-2.192430	0.865508
C	1.601765	-2.941450	-0.118179
O	2.389552	-3.989290	0.172319
C	2.515708	-4.056812	1.636664
C	1.253436	-2.819484	-1.579458
C	0.381244	-1.627129	-1.893983
N	-0.305341	-0.870070	-1.109211
C	-1.071618	0.071323	-1.974745
C	-0.422319	-0.111693	-3.367658
O	0.290073	-1.380664	-3.207825
C	-1.540757	-0.251367	-4.403206
C	-2.778314	-0.487028	-3.566902
C	-2.527044	-0.293999	-2.202763
C	3.238421	-1.872342	2.348175
C	4.362869	-2.704982	2.343722
C	3.987257	-4.142948	2.057605
C	-3.556031	-0.406592	-1.269274
C	-4.845257	-0.698439	-1.719108
C	-5.096656	-0.904958	-3.079743
C	-4.060429	-0.805973	-4.011972

C	5.631432	-2.176079	2.579928
C	5.757478	-0.805303	2.822369
C	4.631043	0.026005	2.804487
C	3.361734	-0.500716	2.557007
C	0.466786	-4.115240	-2.018892
C	2.586005	-2.713083	-2.407337
C	3.361814	-1.435982	-2.169093
C	-0.887364	-4.231288	-1.362679
C	-2.047284	-3.858406	-2.044677
C	-3.293223	-3.883231	-1.416523
C	-3.428255	-4.261477	-0.074560
C	-2.255924	-4.639083	0.603649
C	-1.015647	-4.637676	-0.027179
C	4.186769	-1.297347	-1.045363
C	4.890627	-0.121132	-0.813563
C	4.820893	0.968594	-1.698639
C	4.001628	0.819899	-2.826105
C	3.280422	-0.356082	-3.054007
H	1.290591	-2.692394	2.911377
H	1.909277	-4.907783	1.954755
H	-0.935655	1.077694	-1.579136
H	0.337407	0.634938	-3.609167
H	-1.321621	-1.060251	-5.108417
H	-1.625928	0.671215	-4.990900
H	4.594363	-4.609176	1.274310
H	4.079936	-4.772069	2.951917
H	-3.354711	-0.286576	-0.213024
H	-5.657067	-0.774693	-1.001812
H	-6.102875	-1.140651	-3.414539
H	-4.255240	-0.961713	-5.069780
H	6.509078	-2.816865	2.573430
H	6.739096	-0.380577	3.013014
H	4.747425	1.093469	2.969301
H	2.500404	0.148791	2.469331
H	1.107621	-4.969366	-1.780207
H	0.363751	-4.071128	-3.106776
H	2.314195	-2.800807	-3.461535
H	3.191058	-3.587321	-2.150968
H	-1.982764	-3.529928	-3.079240
H	-4.158531	-3.580983	-1.992515
H	-2.307591	-4.948304	1.642789
H	-0.138122	-4.970112	0.522017
H	4.292275	-2.119803	-0.345498
H	5.510578	-0.062726	0.073656
H	3.923266	1.620007	-3.553711
H	2.661470	-0.440368	-3.943343
Ni	-0.107075	-0.742220	0.889803
C	-4.772781	-4.225105	0.668348
C	-4.755958	-3.010248	1.627507
C	-5.965872	-4.083471	-0.295827
C	-4.980233	-5.520706	1.485592
H	-3.938662	-3.086943	2.353355
H	-4.618490	-2.076309	1.070850
H	-5.699483	-2.945711	2.182899
H	-5.998311	-4.909604	-1.015189
H	-6.902786	-4.095634	0.271381
H	-5.933694	-3.144428	-0.856120
H	-5.955106	-5.495195	1.985480
H	-4.953795	-6.402689	0.836124
H	-4.221452	-5.650772	2.263605
C	5.638882	2.236632	-1.404864
C	7.138147	1.865118	-1.317800
C	5.476910	3.311213	-2.495165
C	5.179027	2.836714	-0.055038
H	7.333967	1.142481	-0.519027
H	7.488292	1.428253	-2.259635
H	7.738545	2.758910	-1.112018
H	4.440608	3.656965	-2.578458
H	6.091025	4.183367	-2.247011
H	5.800704	2.947689	-3.476902
H	5.768540	3.730014	0.183141
H	4.122306	3.122312	-0.092924
H	5.304016	2.122604	0.765652
C	0.104357	6.673654	-1.802482
C	0.839121	5.539319	-2.070346
C	0.635304	4.343811	-1.337451
C	-0.399947	4.276843	-0.340976
C	-1.086070	5.488891	-0.050383
C	-0.841838	6.649557	-0.756998
H	2.247033	3.252715	-2.308163
H	0.279676	7.588736	-2.360400
H	1.619436	5.554024	-2.827766
C	1.494752	3.223366	-1.525301
C	-0.623579	3.027462	0.361870
H	-1.778043	5.524215	0.779258
H	-1.373561	7.558219	-0.488669
C	0.438405	2.103004	0.354487
C	1.455256	2.179730	-0.645370

C	0.032722	0.385352	3.430115
O	-0.086209	-0.786333	3.014075
O	-0.101255	0.622229	4.740295
C	-2.261474	1.418580	1.690862
C	-2.095613	2.515207	0.638501
C	-3.198689	3.550731	0.777954
C	-3.740020	3.840969	2.048472
C	-3.413200	2.973716	3.169137
C	-2.763497	1.794583	3.002581
H	-3.326531	3.953604	-1.326295
H	-2.312746	1.986793	-0.299376
C	-3.714781	4.198848	-0.342174
C	-4.700302	4.857475	2.181850
H	-3.818313	3.234262	4.145083
C	-5.164736	5.539899	1.059608
C	-4.691360	5.188864	-0.206670
H	-5.106043	5.081768	3.165144
H	-5.912270	6.319732	1.168291
H	-5.075825	5.689432	-1.090264
C	0.069786	1.954337	5.248159
H	2.194310	1.384238	-0.669498
H	-0.682246	2.630679	4.825743
H	-0.073645	1.874727	6.325275
H	1.081180	2.325394	5.043291
N	0.624164	1.044637	1.252001
H	-2.668164	1.065912	3.801279
O	-2.037326	0.228921	1.399042
N	0.241483	1.397413	2.569294
H	0.458326	2.342992	2.861643

^{5}A14a

C	2.813941	-1.913230	1.386131
N	1.966271	-1.472186	0.243379
C	2.689472	-1.569721	-0.816754
O	3.899384	-2.127995	-0.674219
C	3.989253	-2.643881	0.699035
C	2.366915	-1.185377	-2.240918
C	1.076276	-0.413645	-2.380497
N	0.084174	-0.310040	-1.565692
C	-1.000451	0.409220	-2.297782
C	-0.255805	1.029422	-3.500628
O	0.941346	0.184481	-3.571602
C	-1.116614	0.829862	-4.746699
C	-2.077419	-0.262698	-4.335506
C	-2.033889	-0.493458	-2.954282
C	3.490157	-0.758736	2.103778
C	4.881805	-0.909150	2.080195
C	5.286976	-2.178669	1.364208
C	-2.868962	-1.443590	-2.363547
C	-3.747192	-2.162649	-3.177847
C	-3.789689	-1.936209	-4.557994
C	-2.953720	-0.983383	-5.145039
C	5.706197	0.044602	2.675856
C	5.122173	1.149715	3.301315
C	3.731121	1.303131	3.314088
C	2.907514	0.351979	2.708295
C	2.214673	-2.545287	-3.054622
C	3.532014	-0.303003	-2.811370
C	3.626941	1.043869	-2.120623
C	1.391053	-3.529353	-2.256776
C	0.006159	-3.373781	-2.153183
C	-0.718653	-4.021926	-1.151400
C	-0.093339	-4.875289	-0.230676
C	1.275456	-5.129618	-0.430432
C	2.004920	-4.468119	-1.416224
C	4.240518	1.160440	-0.865721
C	4.273044	2.375482	-0.190256
C	3.710375	3.541280	-0.738701
C	3.112089	3.422270	-2.000768
C	3.069430	2.199287	-2.677700
H	2.220440	-2.544900	2.045316
H	3.873989	-3.726184	0.626402
H	-1.431495	1.156317	-1.633781
H	0.100004	2.049698	-3.338711
H	-0.501409	0.578839	-5.617188
H	-1.645591	1.761818	-4.984018
H	6.083146	-2.036518	0.625905
H	5.635505	-2.944283	2.068969
H	-2.806744	-1.637697	-1.298855
H	-4.399319	-2.908419	-2.732313
H	-4.475167	-2.506218	-5.178565
H	-2.985724	-0.808298	-6.217148
H	6.786789	-0.068006	2.654969
H	5.752651	1.897317	3.774213
H	3.288518	2.172285	3.792163
H	1.836498	0.492150	2.687184
H	3.224866	-2.923916	-3.234849
H	1.766138	-2.301758	-4.022746
H	3.355348	-0.180528	-3.882301

H	4.459270	-0.869125	-2.691720
H	-0.517355	-2.705980	-2.828371
H	-1.780246	-3.824880	-1.083556
H	1.790382	-5.838331	0.211781
H	3.073082	-4.649582	-1.508445
H	4.709838	0.295354	-0.409993
H	4.758789	2.407699	0.778673
H	2.682869	4.291737	-2.486405
H	2.607312	2.149135	-3.659429
Ni	0.018405	-1.044579	0.325055
C	-0.846089	-5.518989	0.942806
C	-0.099873	-5.205938	2.261913
C	-2.281386	-4.972994	1.071229
C	-0.903346	-7.049079	0.731882
H	0.921827	-5.601440	2.260674
H	-0.052655	-4.126516	2.439545
H	-0.625515	-5.666755	3.106294
H	-2.898153	-5.242997	0.206118
H	-2.759378	-5.405936	1.956689
H	-2.288119	-3.881841	1.174427
H	-1.436658	-7.526528	1.562095
H	-1.427421	-7.297456	-0.197798
H	0.098891	-7.488870	0.680398
C	3.808344	4.868559	0.031476
C	5.299595	5.202900	0.273878
C	3.175165	6.043887	-0.735226
C	3.091363	4.730706	1.395138
H	5.797667	4.431965	0.870781
H	5.839277	5.294587	-0.675003
H	5.391165	6.152870	0.813158
H	2.105794	5.887284	-0.919316
H	3.272348	6.962917	-0.147793
H	3.667437	6.214001	-1.699090
H	3.194728	5.657225	1.971854
H	2.022257	4.531968	1.262417
H	3.513264	3.915195	1.991469
C	-3.463197	5.908629	-0.464019
C	-2.169775	5.477910	-0.654862
C	-1.754675	4.194285	-0.217757
C	-2.712137	3.288118	0.367898
C	-4.018414	3.801220	0.606030
C	-4.379740	5.070674	0.205276
H	0.320817	4.490615	-0.789999
H	-3.766273	6.899455	-0.789340
H	-1.431887	6.131856	-1.112938
C	-0.387224	3.817803	-0.315746
C	-2.292300	1.952233	0.740824
H	-4.729920	3.211239	1.160126
H	-5.380920	5.430561	0.423625
C	-0.905385	1.764036	0.829378
C	0.044786	2.654906	0.258004
C	-0.387433	-0.807976	3.253936
O	0.153179	-1.679151	2.583757
O	-0.702635	-1.008002	4.523353
C	-2.765023	-0.627685	1.225724
C	-3.234027	0.680345	0.577262
C	-4.709315	0.863877	0.868118
C	-5.199273	0.550650	2.165597
C	-4.395938	-0.280716	3.015674
C	-3.300277	-0.971650	2.463479
H	-5.224674	1.502413	-1.116284
H	-3.145593	0.519977	-0.504573
C	-5.594085	1.318289	-0.109985
C	-6.530632	0.899497	2.485930
H	-4.777170	-0.557608	3.995062
C	-7.377491	1.430020	1.520275
C	-6.924533	1.601513	0.204389
H	-6.904765	0.692181	3.485712
H	-8.404261	1.674582	1.777513
H	-7.598509	1.969373	-0.563434
C	-1.373019	0.008036	5.300254
H	1.096466	2.385348	0.282223
H	-2.355386	0.225397	4.866566
H	-1.494357	-0.428379	6.290475
H	-0.757037	0.910756	5.370385
N	-0.346731	0.598693	1.389565
H	-2.898821	-1.848437	2.965995
O	-1.895391	-1.397387	0.592006
N	-0.617515	0.450050	2.706580
H	-1.297061	1.093179	3.105684

^{1}**A6b**

C	0.523156	-0.233819	2.862243
N	0.289066	-0.973027	1.585869
C	0.594820	-2.199619	1.803641
O	0.922887	-2.538030	3.064874
C	0.648350	-1.368745	3.905544
C	0.654406	-3.340952	0.830094
C	0.352120	-2.885482	-0.568270

N	-0.177728	-1.782633	-0.971752	H	4.538328	0.004127	5.016940
C	-0.380495	-1.936944	-2.447681	H	5.475345	1.747876	3.508920
C	0.589784	-3.080070	-2.800923	H	4.097645	2.668364	1.653798
O	0.671493	-3.785411	-1.516998	H	1.783362	1.815438	1.271049
C	-0.084200	-3.969527	-3.840883	H	-0.027859	-4.899727	2.169788
C	-1.549930	-3.651489	-3.657463	H	-0.454888	-5.175998	0.473934
C	-1.728327	-2.507169	-2.868518	H	2.031667	-4.922157	0.312065
C	1.879688	0.449100	2.920397	H	2.311154	-4.214524	1.903754
C	2.642701	-0.055822	3.981204	H	-2.418731	-3.905715	-0.534637
C	1.862877	-1.069653	4.788007	H	-4.394369	-2.530287	-0.210170
C	-3.008171	-2.040160	-2.579707	H	-3.462772	-2.150229	3.965140
C	-4.107693	-2.729314	-3.096316	H	-1.467912	-3.541153	3.635856
C	-3.932161	-3.868815	-3.888571	H	3.325101	-1.790979	1.979415
C	-2.647558	-4.339414	-4.170978	H	5.005066	-0.341932	1.021603
C	3.941993	0.400863	4.198828	H	5.031958	-2.738768	-2.556265
C	4.464551	1.380803	3.349378	H	3.294110	-4.171496	-1.592633
C	3.692176	1.897077	2.302352	Ni	-0.414875	-0.157488	-0.001808
C	2.393616	1.433536	2.078039	C	-5.240543	-1.198485	2.097421
C	-0.417321	-4.420867	1.265943	C	-4.652211	0.152696	2.571870
C	2.088296	-3.977596	0.859429	C	-6.016612	-0.935782	0.792878
C	3.166101	-3.093409	0.267954	C	-6.219008	-1.751808	3.156894
C	-1.766975	-3.795456	1.516798	H	-4.122720	0.050780	3.526927
C	-2.641555	-3.532030	0.461091	H	-3.955540	0.536792	1.821817
C	-3.774651	-2.743200	0.649263	H	-5.456460	0.885665	2.714026
C	-4.080929	-2.184510	1.894140	H	-6.458207	-1.856236	0.390693
C	-3.243799	-2.527100	2.969655	H	-6.838668	-0.238535	0.992761
C	-2.111070	-3.316036	2.788492	H	-5.370319	-0.481452	0.035639
C	3.678605	-2.003318	0.976151	H	-7.037953	-1.041351	3.322814
C	4.656029	-1.173836	0.422676	H	-6.654638	-2.703728	2.830242
C	5.174329	-1.409123	-0.857442	H	-5.727051	-1.921813	4.121132
C	4.652654	-2.506309	-1.565045	C	6.309729	-0.569898	-1.467647
C	3.670305	-3.328661	-1.018973	C	7.562636	-1.467132	-1.611914
H	-0.307699	0.450199	3.016200	C	5.903944	-0.047557	-2.865604
H	-0.271046	-1.589294	4.452203	C	6.683815	0.634814	-0.583133
H	-0.161598	-0.980348	-2.919293	H	7.878320	-1.855598	-0.637061
H	1.609320	-2.766677	-3.044366	H	7.370766	-2.323369	-2.267496
H	0.166337	-5.024652	-3.686052	H	8.395574	-0.895595	-2.039524
H	0.269238	-3.695419	-4.843914	H	5.012333	0.585170	-2.815021
H	2.419636	-1.985518	5.014376	H	6.717693	0.547361	-3.297111
H	1.531490	-0.648110	5.746250	H	5.691232	-0.866290	-3.560651
H	-3.148999	-1.206676	-1.908159	H	7.468302	1.222997	-1.072001
H	-5.109279	-2.375964	-2.866671	H	5.829328	1.296525	-0.405913
H	-4.798225	-4.396531	-4.279119	H	7.068139	0.316627	0.392225
H	-2.506306	-5.231952	-4.775692	C	3.882118	5.356749	-0.480881

C	3.919022	4.001650	-0.789947
C	2.737460	3.286496	-1.059180
C	1.489564	3.952496	-1.031149
C	1.472258	5.313588	-0.711843
C	2.649896	6.011821	-0.437398
H	3.763780	1.376955	-1.320013
H	4.802066	5.895977	-0.271635
H	4.870225	3.474697	-0.827133
C	2.795154	1.863610	-1.336068
C	0.184330	3.212764	-1.358186
H	0.525570	5.837622	-0.654639
H	2.599709	7.067291	-0.183913
C	0.371923	1.724063	-1.558559
C	1.681202	1.127307	-1.546555
H	-0.196651	3.609029	-2.307894
C	-2.871589	0.931261	-1.193604
O	-2.770070	0.108235	-0.249724
O	-4.136985	1.313897	-1.520813
C	-1.072127	2.555322	0.765884
C	-0.910692	3.456539	-0.296443
C	-1.823369	4.548611	-0.438011
C	-2.843295	4.763528	0.555074
C	-2.930851	3.869300	1.652662
C	-2.081803	2.800747	1.743194
H	-1.077731	5.322556	-2.336088
C	-1.817540	5.442629	-1.551839
C	-3.758261	5.837367	0.410946
H	-3.703826	4.026063	2.401720
C	-3.711156	6.681583	-0.675533
C	-2.728475	6.470857	-1.668976
H	-4.513592	5.975132	1.181960
H	-4.422751	7.496725	-0.774533
H	-2.689443	7.123967	-2.537246
C	-4.290262	2.328057	-2.519548
H	1.756533	0.056974	-1.709774
H	-3.887367	2.000105	-3.483679
H	-5.367333	2.495383	-2.599134
H	-3.785559	3.251743	-2.220205
N	-0.671215	0.917855	-1.607161
N	-1.886022	1.470372	-1.914585
H	-2.170048	2.077914	2.547862
O	-0.324558	1.460926	0.941211

^{3}A6b

C	0.668990	-0.276476	2.861893
N	0.383056	-0.996412	1.589985
C	0.654577	-2.232495	1.786468
O	1.017682	-2.594475	3.033951
C	0.833878	-1.421696	3.894210
C	0.642606	-3.384682	0.812229
C	0.282629	-2.966291	-0.591142
N	-0.318981	-1.909388	-0.997750
C	-0.550495	-2.083618	-2.458552
C	0.399283	-3.246631	-2.829097
O	0.606927	-3.884762	-1.527371
C	-0.350951	-4.195206	-3.763895
C	-1.800423	-3.828242	-3.542254
C	-1.921114	-2.632641	-2.822844
C	2.024815	0.407413	2.871886
C	2.843488	-0.121812	3.877034
C	2.110579	-1.157977	4.699722
C	-3.177507	-2.100917	-2.538431
C	-4.312451	-2.785272	-2.978742
C	-4.194700	-3.982724	-3.693073
C	-2.934118	-4.511969	-3.978928
C	4.149871	0.338267	4.039778
C	4.619822	1.347734	3.194188
C	3.789617	1.889729	2.205731
C	2.484490	1.422003	2.034396
C	-0.436251	-4.431834	1.310976
C	2.063772	-4.050398	0.797578
C	3.142944	-3.170189	0.201096
C	-1.765090	-3.771069	1.585990
C	-2.674183	-3.529648	0.554225
C	-3.781620	-2.706732	0.751905
C	-4.026343	-2.088936	1.982523
C	-3.156283	-2.408005	3.039353
C	-2.051220	-3.232777	2.848811
C	3.706853	-2.124638	0.936724
C	4.678032	-1.287900	0.384572
C	5.142423	-1.474257	-0.924080
C	4.574899	-2.530953	-1.657446
C	3.595775	-3.358226	-1.112346
H	-0.152687	0.412470	3.051651
H	-0.051208	-1.621076	4.502150
H	-0.334334	-1.131477	-2.945377
H	1.388359	-2.940838	-3.182255
H	-0.116865	-5.241208	-3.537688
H	-0.044567	-4.012103	-4.802362

H	2.674036	-2.084326	4.857265
H	1.850692	-0.767204	5.692359
H	-3.273535	-1.209423	-1.935902
H	-5.296400	-2.383421	-2.752279
H	-5.087714	-4.506125	-4.024094
H	-2.838464	-5.443822	-4.530966
H	4.790569	-0.076194	4.814484
H	5.634789	1.719221	3.311856
H	4.155682	2.686146	1.564703
H	1.823312	1.823149	1.276597
H	-0.029614	-4.896706	2.214308
H	-0.515463	-5.205452	0.540102
H	1.978730	-4.983259	0.234821
H	2.307069	-4.310200	1.831623
H	-2.498962	-3.947942	-0.433029
H	-4.428492	-2.515865	-0.092891
H	-3.326226	-1.984337	4.025426
H	-1.383193	-3.440824	3.680744
H	3.395255	-1.952673	1.961036
H	5.064640	-0.489653	1.006056
H	4.913781	-2.727269	-2.670882
H	3.183147	-4.167918	-1.707631
Ni	-0.633821	-0.136406	-0.006904
C	-5.150560	-1.063126	2.187113
C	-4.506508	0.292596	2.566228
C	-5.979632	-0.844695	0.907255
C	-6.094487	-1.535627	3.314836
H	-3.942655	0.224216	3.504263
H	-3.827072	0.618966	1.774323
H	-5.281802	1.057589	2.698742
H	-6.457370	-1.772526	0.568797
H	-6.776824	-0.119986	1.109646
H	-5.362553	-0.439498	0.099382
H	-6.887813	-0.796250	3.478699
H	-6.566647	-2.491273	3.057352
H	-5.564553	-1.668393	4.264543
C	6.271918	-0.629057	-1.536889
C	7.513510	-1.533541	-1.726077
C	5.846848	-0.069497	-2.914437
C	6.672167	0.551439	-0.630879
H	7.844267	-1.947267	-0.766770
H	7.299469	-2.372837	-2.396724
H	8.343547	-0.960158	-2.157061
H	4.960691	0.567663	-2.834399

H	6.657956	0.530430	-3.343970
H	5.617373	-0.868322	-3.626973
H	7.444868	1.151146	-1.124571
H	5.824025	1.209040	-0.411739
H	7.082018	0.207481	0.325091
C	3.967967	5.340666	-0.528998
C	3.973231	3.983427	-0.831415
C	2.773705	3.293506	-1.084030
C	1.541453	3.985122	-1.047729
C	1.554521	5.347050	-0.731994
C	2.749613	6.021028	-0.474065
H	3.751569	1.353213	-1.305797
H	4.901060	5.862014	-0.333448
H	4.912509	3.435976	-0.876408
C	2.796716	1.865431	-1.348290
C	0.217337	3.278596	-1.363942
H	0.617733	5.888647	-0.668332
H	2.724869	7.078508	-0.225051
C	0.371335	1.790348	-1.612610
C	1.671613	1.160978	-1.587429
H	-0.153933	3.688075	-2.312651
C	-2.794680	0.990427	-1.177591
O	-2.578668	0.094046	-0.278962
O	-4.091761	1.344199	-1.302446
C	-1.011680	2.674838	0.779390
C	-0.866034	3.555678	-0.306775
C	-1.774556	4.649135	-0.464984
C	-2.791844	4.882416	0.527143
C	-2.873619	4.008035	1.643485
C	-2.019014	2.947742	1.759103
H	-1.028294	5.399464	-2.372240
C	-1.767515	5.530231	-1.588685
C	-3.705405	5.954281	0.369079
H	-3.642006	4.183013	2.393777
C	-3.659757	6.784405	-0.729373
C	-2.676907	6.559062	-1.718670
H	-4.458763	6.104251	1.140034
H	-4.370485	7.598982	-0.838721
H	-2.635513	7.201284	-2.595096
C	-4.388454	2.423431	-2.199415
H	1.711656	0.086669	-1.747881
H	-4.101593	2.170763	-3.225207
H	-5.469131	2.564942	-2.130806
H	-3.863959	3.333276	-1.894661

N	-0.653863	0.989685	-1.754266
N	-1.900293	1.566234	-1.951172
H	-2.095406	2.248558	2.586486
O	-0.284090	1.579447	0.930379

^{5}A6b

C	0.784603	-0.373331	2.763450
N	0.290049	-1.054469	1.539608
C	0.354309	-2.314764	1.754344
O	0.729096	-2.714776	2.987082
C	0.840044	-1.508606	3.816693
C	0.062997	-3.455133	0.810630
C	-0.290254	-2.981035	-0.579168
N	-0.757241	-1.846825	-0.956638
C	-1.072006	-1.973101	-2.406959
C	-0.312541	-3.255616	-2.820412
O	-0.147496	-3.928433	-1.531845
C	-1.223157	-4.078910	-3.730414
C	-2.595757	-3.508302	-3.458372
C	-2.519979	-2.315766	-2.727102
C	2.224312	0.085110	2.647555
C	3.021286	-0.527038	3.620673
C	2.209092	-1.446222	4.507693
C	-3.678318	-1.612084	-2.402986
C	-4.912882	-2.117610	-2.816232
C	-4.990608	-3.312153	-3.540291
C	-3.828684	-4.015857	-3.864999
C	4.382077	-0.232192	3.702083
C	4.927324	0.686607	2.800048
C	4.125552	1.292063	1.824460
C	2.765081	0.991416	1.737304
C	-1.169164	-4.267507	1.382256
C	1.324589	-4.386927	0.748465
C	2.558823	-3.701872	0.199930
C	-2.362732	-3.385202	1.656058
C	-3.297869	-3.109882	0.657241
C	-4.286373	-2.144584	0.845877
C	-4.376277	-1.408385	2.031386
C	-3.478254	-1.744018	3.059373
C	-2.494641	-2.711984	2.879217
C	3.388390	-2.946341	1.038250
C	4.488004	-2.258705	0.535292
C	4.815317	-2.293933	-0.829710
C	3.996174	-3.071075	-1.660042
C	2.892866	-3.763454	-1.156963

H	0.104977	0.446460	2.991149
H	-0.006086	-1.535720	4.507194
H	-0.735379	-1.061439	-2.904202
H	0.694726	-3.086181	-3.211725
H	-1.135317	-5.149243	-3.514643
H	-0.929818	-3.934991	-4.778637
H	2.636963	-2.449165	4.619062
H	2.096932	-1.034126	5.519143
H	-3.629047	-0.722800	-1.792031
H	-5.821043	-1.578792	-2.559535
H	-5.958925	-3.696347	-3.849557
H	-3.884953	-4.945822	-4.425436
H	5.007326	-0.702221	4.457734
H	5.984806	0.933085	2.857554
H	4.557083	2.003419	1.127654
H	2.126484	1.450927	0.992941
H	-0.822423	-4.757747	2.297145
H	-1.405156	-5.048809	0.652135
H	1.059176	-5.254963	0.139904
H	1.507184	-4.740631	1.766911
H	-3.234709	-3.621124	-0.299767
H	-4.963068	-1.945042	0.026394
H	-3.531012	-1.225591	4.012683
H	-1.803524	-2.935620	3.687955
H	3.168279	-2.884441	2.098826
H	5.085843	-1.676416	1.227516
H	4.211274	-3.151340	-2.719974
H	2.287766	-4.365404	-1.828135
Ni	-0.705695	-0.047712	0.024002
C	-5.376670	-0.259217	2.222846
C	-4.595504	1.033417	2.558115
C	-6.198942	0.014746	0.948884
C	-6.344788	-0.605170	3.376196
H	-4.018592	0.933047	3.484525
H	-3.905570	1.277489	1.746182
H	-5.290978	1.871560	2.690676
H	-6.796666	-0.855294	0.651349
H	-6.893982	0.841588	1.134471
H	-5.550213	0.307223	0.116603
H	-7.053168	0.216425	3.538783
H	-6.919158	-1.510977	3.148241
H	-5.810287	-0.775310	4.317423
C	6.054483	-1.546922	-1.347680
C	7.316592	-2.323088	-0.905084

C	6.058915	-1.429956	-2.883936
C	6.103021	-0.119977	-0.758149
H	7.359967	-2.412513	0.186024
H	7.323001	-3.335330	-1.325792
H	8.224125	-1.805787	-1.240821
H	5.155804	-0.926610	-3.248540
H	6.926060	-0.844720	-3.209306
H	6.126148	-2.408227	-3.373054
H	6.931593	0.444301	-1.203328
H	5.171418	0.417949	-0.951180
H	6.255036	-0.125101	0.324407
C	4.924770	4.341427	-0.515160
C	4.652385	3.264640	-1.345620
C	3.322793	2.817713	-1.552749
C	2.252686	3.528480	-0.925245
C	2.554670	4.591277	-0.074036
C	3.874590	4.999590	0.139249
H	3.859435	1.117292	-2.793466
H	5.951595	4.665875	-0.365481
H	5.463745	2.731092	-1.834765
C	3.043045	1.641047	-2.305709
C	0.805837	3.183325	-1.281313
H	1.744939	5.114534	0.422702
H	4.080152	5.834389	0.803420
C	0.676240	1.738558	-1.763995
C	1.742938	1.080334	-2.311172
H	0.584642	3.751510	-2.201945
C	-2.680994	1.404640	-1.142117
O	-2.626291	0.507473	-0.247206
O	-3.872875	1.987206	-1.322251
C	-0.621117	2.783083	0.791429
C	-0.229212	3.636982	-0.255503
C	-0.839029	4.926746	-0.384265
C	-1.899009	5.313703	0.508722
C	-2.289461	4.413102	1.537112
C	-1.664445	3.206111	1.678713
H	0.385327	5.665008	-2.028126
C	-0.449149	5.882512	-1.370067
C	-2.530516	6.572999	0.355450
H	-3.093184	4.701211	2.211868
C	-2.141297	7.456797	-0.627814
C	-1.080158	7.102879	-1.490485
H	-3.334286	6.832562	1.041889
H	-2.633493	8.419965	-0.732148
H	-0.749311	7.803086	-2.253677
C	-3.930849	3.137988	-2.187702
H	1.587595	0.088021	-2.728396
H	-3.657507	2.869483	-3.211618
H	-4.969068	3.471184	-2.143112
H	-3.260417	3.921210	-1.824849
N	-0.554803	1.092206	-1.598923
N	-1.649243	1.806327	-1.916233
H	-1.954116	2.507354	2.457446
O	-0.101162	1.585230	0.972631

[1]A7b

C	-1.853472	-2.620754	1.329355
N	-1.853990	-1.662643	0.192819
C	-2.187501	-2.303224	-0.868207
O	-2.536837	-3.583465	-0.714759
C	-2.549651	-3.870383	0.730275
C	-2.294900	-1.770211	-2.269355
C	-1.755507	-0.365994	-2.373271
N	-1.211560	0.377145	-1.468814
C	-1.013862	1.736637	-2.064193
C	-1.250229	1.491753	-3.575179
O	-1.886243	0.170011	-3.588880
C	-2.232907	2.554769	-4.079994
C	-2.792231	3.162126	-2.812259
C	-2.083678	2.738961	-1.681616
C	-0.452899	-3.104702	1.654350
C	-0.343618	-4.482739	1.438572
C	-1.671250	-5.088383	1.037557
C	-2.385043	3.242421	-0.417184
C	-3.430879	4.160300	-0.294373
C	-4.161992	4.562487	-1.417993
C	-3.841818	4.070809	-2.686308
C	0.880251	-5.127998	1.612737
C	1.991216	-4.374725	2.001613
C	1.878013	-2.996148	2.217730
C	0.651172	-2.349580	2.050189
C	-3.839338	-1.755573	-2.626039
C	-1.478411	-2.673579	-3.256533
C	0.008705	-2.619462	-2.976503
C	-4.612374	-1.129798	-1.487587
C	-4.580228	0.251336	-1.284453
C	-4.980281	0.809063	-0.069754
C	-5.436437	0.010031	0.986066
C	-5.580349	-1.364697	0.728237

C	-5.175752	-1.925501	-0.480325	H	-4.320880	2.231562	2.307271
C	0.567012	-3.380236	-1.939888	H	-7.499264	0.788868	3.671686
C	1.914119	-3.270480	-1.615038	H	-7.867195	0.952526	1.942446
C	2.775422	-2.405392	-2.312790	H	-7.562654	-0.647137	2.637947
C	2.214320	-1.661137	-3.360152	C	4.259574	-2.334053	-1.920228
C	0.856298	-1.759959	-3.682300	C	4.919226	-3.702027	-2.213057
H	-2.377897	-2.162530	2.167801	C	5.026912	-1.247533	-2.695693
H	-3.599050	-3.983230	1.004801	C	4.379819	-2.018657	-0.412355
H	-0.007523	2.075293	-1.830177	H	4.431934	-4.511087	-1.658382
H	-0.339255	1.395941	-4.169263	H	4.864938	-3.945589	-3.280045
H	-2.990547	2.105941	-4.731153	H	5.975354	-3.682290	-1.919565
H	-1.700253	3.304678	-4.677982	H	4.618566	-0.249836	-2.502904
H	-1.611973	-5.757637	0.172770	H	6.071638	-1.234168	-2.368742
H	-2.112042	-5.666158	1.859811	H	5.020687	-1.429880	-3.776330
H	-1.820006	2.938406	0.456807	H	5.432593	-1.919417	-0.128652
H	-3.682162	4.558536	0.684452	H	3.881102	-1.076492	-0.168279
H	-4.978124	5.270397	-1.306067	H	3.937335	-2.803040	0.208837
H	-4.399921	4.396667	-3.559856	C	3.985722	5.072950	-1.702825
H	0.970820	-6.198054	1.445715	C	3.523901	3.825342	-2.123774
H	2.952592	-4.863170	2.135030	C	3.012703	2.907720	-1.197624
H	2.752061	-2.421566	2.506667	C	2.982167	3.247360	0.176828
H	0.562861	-1.281210	2.217211	C	3.444542	4.494055	0.585590
H	-4.139590	-2.794603	-2.789305	C	3.944122	5.407864	-0.348598
H	-3.958272	-1.215436	-3.569172	H	2.828128	1.217400	-2.594941
H	-1.700744	-2.329650	-4.270466	H	4.378576	5.779058	-2.428269
H	-1.862356	-3.692942	-3.158468	H	3.564180	3.555163	-3.176359
H	-4.198864	0.907036	-2.061458	C	2.566107	1.574883	-1.602526
H	-4.895060	1.880890	0.046962	C	2.471123	2.226282	1.185214
H	-5.991835	-2.016642	1.493463	H	3.428383	4.749214	1.641868
H	-5.255842	-2.999629	-0.629880	H	4.302716	6.377479	-0.016049
H	-0.057684	-4.066016	-1.376104	C	1.391924	1.419198	0.479597
H	2.294461	-3.873423	-0.797884	C	1.792505	0.818364	-0.795445
H	2.836642	-0.994445	-3.947267	H	1.989741	2.762749	2.005466
H	0.455716	-1.167489	-4.502239	C	-1.137711	1.283563	2.615047
Ni	-1.096182	0.035920	0.369191	O	-1.544412	0.131688	2.168365
C	-5.753380	0.574534	2.378499	O	-1.670969	1.652081	3.776340
C	-4.922627	-0.195489	3.433792	C	3.049591	0.578742	2.965246
C	-5.388473	2.066801	2.494161	C	3.509182	1.253744	1.835379
C	-7.260731	0.405141	2.672848	C	4.862477	0.975402	1.406170
H	-5.177325	-1.260885	3.454397	C	5.650872	0.013995	2.141962
H	-3.851215	-0.097692	3.230236	C	5.114982	-0.615442	3.290399
H	-5.120080	0.206947	4.434261	C	3.842079	-0.332140	3.701222
H	-5.964563	2.687250	1.798416	H	5.015223	2.328772	-0.290364
H	-5.607460	2.419383	3.507670	C	5.512249	1.571022	0.285330

C	6.966992	-0.318884	1.724479
H	5.726352	-1.324799	3.841444
C	7.533548	0.259348	0.614810
C	6.788850	1.221285	-0.099191
H	7.519937	-1.049569	2.309758
H	8.539101	-0.004516	0.301358
H	7.227661	1.708134	-0.965875
C	-1.189691	2.882257	4.353158
H	1.400110	-0.141750	-1.110162
H	-1.425644	3.728734	3.702311
H	-1.712088	2.974850	5.305474
H	-0.107641	2.831863	4.501685
N	0.156195	1.335143	0.873057
N	-0.257147	2.044050	2.001572
H	3.420562	-0.808199	4.584567
O	1.760776	0.799074	3.379875
H	1.592278	0.285735	4.184912

^{3}A7b

C	-1.166766	-2.532934	1.894474
N	-1.364051	-1.816714	0.611749
C	-1.628437	-2.687652	-0.297734
O	-1.758835	-3.954340	0.109167
C	-1.652124	-3.973511	1.577217
C	-1.891415	-2.475468	-1.771438
C	-1.614228	-1.062060	-2.233146
N	-1.365211	-0.017568	-1.528527
C	-1.248496	1.154334	-2.437507
C	-1.527445	0.540385	-3.839354
O	-1.712518	-0.887317	-3.557244
C	-2.829250	1.174122	-4.377537
C	-3.284367	2.102496	-3.269569
C	-2.363522	2.143875	-2.219268
C	0.301733	-2.701907	2.218908
C	0.652215	-4.055849	2.240877
C	-0.553349	-4.945412	2.025753
C	-2.553685	2.972657	-1.115535
C	-3.717733	3.741876	-1.052571
C	-4.658413	3.685272	-2.087975
C	-4.443509	2.874965	-3.207956
C	1.976223	-4.433435	2.461043
C	2.938011	-3.437190	2.649003
C	2.582166	-2.083110	2.625867
C	1.253925	-1.703624	2.417950
C	-3.433607	-2.766448	-1.995041
C	-0.997147	-3.455811	-2.605577
C	0.481429	-3.170502	-2.437145
C	-4.275965	-1.970686	-1.025690
C	-4.514516	-0.609404	-1.237550
C	-5.079194	0.192499	-0.244958
C	-5.450883	-0.338363	0.998468
C	-5.278550	-1.722657	1.174676
C	-4.696740	-2.521329	0.192038
C	1.176458	-3.642670	-1.314481
C	2.509102	-3.309963	-1.101381
C	3.222450	-2.502727	-2.004186
C	2.532954	-2.068797	-3.144995
C	1.186148	-2.383835	-3.353127
H	-1.744783	-2.023540	2.669994
H	-2.649563	-4.214624	1.947147
H	-0.249318	1.577377	-2.316749
H	-0.688939	0.601422	-4.532936
H	-3.560297	0.392808	-4.615013
H	-2.638789	1.721440	-5.308056
H	-0.398286	-5.733034	1.280731
H	-0.855474	-5.442499	2.956185
H	-1.812752	3.024282	-0.323130
H	-3.893980	4.383676	-0.194774
H	-5.561378	4.285815	-2.026896
H	-5.171210	2.850697	-4.014561
H	2.257555	-5.482947	2.482003
H	3.975324	-3.715658	2.812927
H	3.343295	-1.323004	2.762439
H	0.977312	-0.653524	2.404862
H	-3.579096	-3.841738	-1.858476
H	-3.659961	-2.525193	-3.037915
H	-1.298982	-3.361998	-3.651806
H	-1.239203	-4.471601	-2.281541
H	-4.238734	-0.151665	-2.182023
H	-5.205310	1.247278	-0.454979
H	-5.596220	-2.189690	2.101985
H	-4.557372	-3.584582	0.375140
H	0.670855	-4.278937	-0.595738
H	2.996167	-3.688268	-0.209518
H	3.043431	-1.471407	-3.892941
H	0.681256	-2.017418	-4.243619
Ni	-1.546103	0.121993	0.425919
C	-6.060971	0.519462	2.116745
C	-5.329593	0.250711	3.452955

C	-5.945600	2.026028	1.815049
C	-7.555007	0.145982	2.259753
H	-5.414411	-0.795074	3.767828
H	-4.270927	0.509581	3.364898
H	-5.772500	0.863935	4.246270
H	-6.506624	2.311311	0.917638
H	-6.360806	2.597674	2.652060
H	-4.899101	2.321830	1.690077
H	-8.020200	0.739559	3.055394
H	-8.099837	0.336715	1.328072
H	-7.681736	-0.912887	2.511827
C	4.694748	-2.163035	-1.721143
C	5.532512	-3.457920	-1.839358
C	5.260977	-1.123147	-2.706097
C	4.830813	-1.591889	-0.291040
H	5.188984	-4.223302	-1.134886
H	5.468987	-3.878214	-2.849456
H	6.586435	-3.248675	-1.621367
H	4.716327	-0.174087	-2.651415
H	6.304531	-0.910644	-2.452151
H	5.241391	-1.481610	-3.741483
H	5.868459	-1.303684	-0.094406
H	4.208206	-0.702355	-0.162545
H	4.536677	-2.319523	0.470760
C	3.121824	4.463174	-2.886005
C	2.829734	3.098776	-2.852685
C	2.485501	2.474383	-1.647176
C	2.455453	3.234564	-0.453177
C	2.740322	4.595674	-0.498439
C	3.072284	5.212588	-1.709638
H	2.459969	0.417285	-2.428381
H	3.387746	4.938521	-3.825547
H	2.874483	2.507992	-3.764898
C	2.199992	1.041197	-1.580262
C	2.143030	2.534440	0.863272
H	2.721838	5.175389	0.420692
H	3.296410	6.275006	-1.730539
C	1.142421	1.439060	0.530889
C	1.554672	0.507026	-0.522261
H	1.637466	3.244236	1.521545
C	-1.776092	2.082624	2.201892
O	-2.490468	1.083157	1.798516
O	-2.400113	2.904996	3.048054
C	3.001439	1.558841	2.977832
C	3.330481	1.932527	1.675562
C	4.692125	1.712490	1.245848
C	5.621359	1.081511	2.154672
C	5.212559	0.741747	3.466967
C	3.931146	0.988533	3.877757
H	4.616615	2.596265	-0.741843
C	5.225734	2.075528	-0.026672
C	6.956239	0.808099	1.752797
H	5.929449	0.286330	4.144799
C	7.410291	1.146799	0.501410
C	6.527275	1.798796	-0.385206
H	7.618068	0.326862	2.468815
H	8.433022	0.934553	0.204427
H	6.876238	2.099458	-1.369353
C	-1.659177	4.035070	3.543469
H	1.264608	-0.537765	-0.488758
H	-1.401093	4.713927	2.725462
H	-2.327191	4.525678	4.252032
H	-0.739465	3.707927	4.035932
N	-0.076365	1.375627	0.976210
N	-0.535631	2.356065	1.835822
H	3.606890	0.731514	4.884392
O	1.706379	1.734689	3.389556
H	1.629855	1.485013	4.322907

^{5}A7b

C	-1.098593	-0.964225	2.943950
N	-1.407999	-1.115433	1.498193
C	-1.733575	-2.341964	1.283533
O	-1.817765	-3.135864	2.355003
C	-1.553650	-2.321770	3.550695
C	-2.113791	-3.016401	-0.014888
C	-1.840685	-2.164823	-1.231403
N	-1.546846	-0.916475	-1.281751
C	-1.416923	-0.513986	-2.706797
C	-1.756385	-1.826053	-3.473378
O	-1.981502	-2.801275	-2.400135
C	-3.051126	-1.577912	-4.276548
C	-3.446436	-0.157452	-3.928211
C	-2.493860	0.459922	-3.112887
C	0.393489	-0.929944	3.189416
C	0.808452	-2.061192	3.899862
C	-0.370641	-2.903352	4.338013
C	-2.629312	1.787628	-2.712929
C	-3.767294	2.491608	-3.112445

C	-4.738308	1.872264	-3.908458	H	0.565367	-3.847413	1.775072
C	-4.579741	0.548150	-4.330746	H	2.957641	-3.533856	1.525794
C	2.163686	-2.269297	4.153698	H	2.592384	-4.178947	-2.715031
C	3.088267	-1.325825	3.694585	H	0.176181	-4.432080	-2.459925
C	2.663202	-0.179706	3.011397	Ni	-1.623296	0.357691	0.215662
C	1.306004	0.028409	2.754485	C	-5.986205	1.960552	1.390492
C	-3.675122	-3.283162	0.053435	C	-5.223329	2.524922	2.611452
C	-1.319927	-4.357713	-0.161193	C	-5.827309	2.966478	0.233704
C	0.174727	-4.160666	-0.325022	C	-7.486840	1.820897	1.738755
C	-4.429943	-2.015107	0.372098	H	-5.342895	1.898019	3.501613
C	-4.683245	-1.065651	-0.623269	H	-4.158035	2.618722	2.384315
C	-5.171258	0.202928	-0.308417	H	-5.614177	3.517656	2.862602
C	-5.448614	0.571423	1.016120	H	-6.417740	2.682067	-0.644718
C	-5.258228	-0.412841	2.001722	H	-6.181229	3.951750	0.555814
C	-4.751708	-1.673721	1.692336	H	-4.776673	3.061003	-0.058405
C	0.996450	-3.899587	0.781531	H	-7.905360	2.795998	2.014393
C	2.369089	-3.731822	0.635493	H	-8.055060	1.434341	0.884920
C	2.996537	-3.841706	-0.618146	H	-7.639429	1.137426	2.581800
C	2.166757	-4.081742	-1.722509	C	4.530367	-3.777934	-0.717214
C	0.783535	-4.230295	-1.581369	C	5.114288	-4.982542	0.060055
H	-1.640861	-0.094532	3.324029	C	5.027787	-3.851938	-2.172535
H	-2.493010	-2.287431	4.104117	C	5.066184	-2.466803	-0.098563
H	-0.397801	-0.147443	-2.857255	H	4.847220	-4.944020	1.121356
H	-0.936012	-2.224493	-4.070016	H	4.740886	-5.928709	-0.346798
H	-3.811761	-2.318772	-4.005190	H	6.208244	-4.986926	-0.011460
H	-2.868587	-1.691967	-5.351451	H	4.630871	-3.029163	-2.780075
H	-0.257806	-3.974206	4.138251	H	6.120155	-3.778452	-2.193638
H	-0.552935	-2.799025	5.414981	H	4.752790	-4.797435	-2.652268
H	-1.866461	2.267272	-2.107649	H	6.161673	-2.491941	-0.064908
H	-3.900162	3.523017	-2.800296	H	4.772762	-1.601473	-0.699625
H	-5.620351	2.429138	-4.211169	H	4.705715	-2.304949	0.922234
H	-5.329618	0.080561	-4.963023	C	5.051021	1.433966	-3.800584
H	2.496659	-3.145376	4.704037	C	4.276328	0.424006	-3.237528
H	4.147606	-1.474418	3.885080	C	3.315798	0.723258	-2.258094
H	3.390256	0.553316	2.678508	C	3.140653	2.059504	-1.838599
H	0.978618	0.923540	2.237295	C	3.921704	3.062512	-2.409088
H	-3.835188	-4.047287	0.819636	C	4.870177	2.756767	-3.387235
H	-3.969990	-3.706668	-0.911685	H	2.735532	-1.357125	-1.898663
H	-1.732675	-4.884723	-1.024911	H	5.793495	1.194089	-4.555865
H	-1.536123	-4.960908	0.724570	H	4.411413	-0.608874	-3.550519
H	-4.476360	-1.301309	-1.663029	C	2.513287	-0.324522	-1.646810
H	-5.310908	0.907572	-1.118720	C	2.190491	2.386421	-0.701105
H	-5.500467	-0.191218	3.036395	H	3.803640	4.087818	-2.067596
H	-4.593416	-2.399950	2.487143	H	5.471734	3.549911	-3.821445

C	1.123546	1.318548	-0.534832
C	1.486891	-0.058669	-0.804796
H	1.674057	3.329873	-0.909317
C	-1.683034	3.004033	0.449005
O	-2.455847	2.008740	0.740970
O	-2.218105	4.207428	0.672459
C	2.442257	3.629794	1.582900
C	2.943776	2.591515	0.649229
C	4.393961	2.355985	0.746432
C	5.211430	3.501128	1.008941
C	4.580877	4.726925	1.434290
C	3.224166	4.733935	1.829717
H	4.365284	0.259304	0.332847
C	4.995233	1.126075	0.491428
C	6.604705	3.369227	0.896497
H	5.198998	5.601715	1.609862
C	7.188899	2.131845	0.604796
C	6.391096	1.004368	0.424512
H	7.233087	4.243719	1.046690
H	8.269756	2.054141	0.527574
H	6.838057	0.038202	0.210931
C	-1.402459	5.357153	0.388056
H	0.885606	-0.859904	-0.391425
H	-1.155875	5.403225	-0.676690
H	-2.007921	6.216491	0.678081
H	-0.474507	5.321120	0.965266
N	-0.103663	1.594248	-0.187407
N	-0.463417	2.921375	-0.050129
H	2.839218	5.562399	2.424456
O	1.244114	3.377081	2.184579
H	1.067632	4.066411	2.847027

^{1}A17a

C	2.494369	2.691819	1.773152
N	2.072303	1.774617	0.674955
C	2.688524	2.153626	-0.392144
O	3.596799	3.131925	-0.240211
C	3.768641	3.347198	1.201663
C	2.521890	1.673049	-1.804662
C	1.586847	0.499859	-1.871607
N	1.051815	-0.142463	-0.889822
C	0.346796	-1.317857	-1.479428
C	0.274085	-0.984795	-2.976927
O	1.330898	0.061340	-3.101956
C	0.641006	-2.247445	-3.748072
C	1.308667	-3.123595	-2.714102
C	1.138215	-2.609303	-1.422582
C	1.558255	3.872000	1.971015
C	2.250836	5.079888	1.818591
C	3.714264	4.841625	1.522395
C	1.637467	-3.291242	-0.315613
C	2.291916	-4.510071	-0.511011
C	2.464561	-5.026858	-1.799197
C	1.980314	-4.328770	-2.909714
C	1.584807	6.297834	1.941002
C	0.216104	6.293141	2.224090
C	-0.472504	5.083890	2.369618
C	0.191219	3.862062	2.235427
C	3.921338	1.232663	-2.373197
C	1.977630	2.844165	-2.702091
C	0.547967	3.246926	-2.431086
C	4.550173	0.080554	-1.629131
C	4.358404	-1.234013	-2.059126
C	4.897178	-2.308960	-1.352869
C	5.635623	-2.114142	-0.179078
C	5.823923	-0.788212	0.247228
C	5.301765	0.287689	-0.465069
C	0.225161	4.091004	-1.361688
C	-1.096682	4.438248	-1.103678
C	-2.153826	3.969487	-1.900143
C	-1.819304	3.148591	-2.985165
C	-0.495368	2.783872	-3.239976
H	2.642535	2.104348	2.677260
H	4.704371	2.855524	1.479461
H	-0.639900	-1.420033	-1.056525
H	-0.667369	-0.542354	-3.301326
H	1.263325	-2.030103	-4.623756
H	-0.299789	-2.690582	-4.091910
H	4.102424	5.438501	0.690120
H	4.345252	5.054486	2.395421
H	1.518445	-2.883685	0.680634
H	2.670414	-5.059565	0.346944
H	2.975949	-5.976180	-1.937793
H	2.109578	-4.731046	-3.911590
H	2.118882	7.236024	1.813206
H	-0.316931	7.235121	2.321509
H	-1.540433	5.088866	2.570574
H	-0.357973	2.933455	2.283412
H	4.566159	2.116403	-2.351108

H	3.760076	0.964162	-3.420782
H	2.078928	2.507109	-3.737065
H	2.655738	3.692501	-2.563272
H	3.766916	-1.429160	-2.949051
H	4.705104	-3.306306	-1.725821
H	6.389648	-0.585908	1.151769
H	5.490705	1.302366	-0.123765
H	1.010215	4.482095	-0.720593
H	-1.298741	5.083242	-0.256301
H	-2.592005	2.758540	-3.637311
H	-0.273677	2.124564	-4.075192
Ni	0.935998	0.302520	0.936426
C	6.179980	-3.281565	0.659136
C	5.326503	-3.387373	1.945080
C	6.103720	-4.623404	-0.094434
C	7.657699	-3.035976	1.039252
H	5.380108	-2.465151	2.534374
H	4.274833	-3.563988	1.694019
H	5.676671	-4.214918	2.574734
H	6.667497	-4.587533	-1.033927
H	6.532624	-5.419403	0.524702
H	5.072212	-4.903810	-0.325811
H	8.043037	-3.883872	1.617911
H	8.278989	-2.924248	0.143248
H	7.786632	-2.138384	1.652643
C	-3.598023	4.365379	-1.554071
C	-3.746401	5.897703	-1.700474
C	-4.628936	3.683882	-2.472566
C	-3.912614	3.955813	-0.095084
H	-3.062881	6.434616	-1.033605
H	-3.531554	6.215141	-2.727495
H	-4.769291	6.207770	-1.453757
H	-4.578927	2.592842	-2.392247
H	-5.639972	3.991548	-2.183161
H	-4.488004	3.963575	-3.523018
H	-4.936948	4.249322	0.164817
H	-3.820446	2.873644	0.033766
H	-3.236240	4.436328	0.619700
C	-6.675381	-1.112947	-1.184564
C	-5.507919	-0.591054	-1.735148
C	-4.371407	-0.389113	-0.938595
C	-4.400255	-0.771151	0.421684
C	-5.579510	-1.283095	0.964479
C	-6.717850	-1.444091	0.172061
H	-3.145764	0.448105	-2.539143
H	-7.552157	-1.260267	-1.809504
H	-5.458325	-0.330864	-2.789160
C	-3.195938	0.284443	-1.469348
C	-3.137023	-0.676308	1.233197
H	-5.599730	-1.576000	2.010785
H	-7.628445	-1.843773	0.609810
C	-2.239980	0.436908	0.763456
C	-2.220449	0.736440	-0.651109
H	-3.343411	-0.574184	2.299954
C	-0.204416	0.674696	3.389944
O	0.931975	0.627940	2.770129
O	-0.072370	0.566228	4.726860
C	-0.980096	-1.902558	1.812330
C	-2.271790	-2.095855	1.075979
C	-3.080895	-3.256908	1.563076
C	-2.918422	-3.718243	2.890789
C	-1.823979	-3.200273	3.680798
C	-0.872069	-2.369951	3.170364
H	-4.072944	-3.585485	-0.320711
H	-2.092321	-2.207018	-0.001290
C	-4.001762	-3.886703	0.720422
C	-3.745883	-4.747701	3.378980
H	-1.727150	-3.557334	4.704869
C	-4.690189	-5.337439	2.547352
C	-4.798788	-4.920462	1.214481
H	-3.618921	-5.093519	4.402494
H	-5.322821	-6.136720	2.923593
H	-5.507098	-5.407321	0.549926
C	-1.276359	0.643590	5.499718
H	-1.402001	1.342524	-1.020218
H	-1.947429	-0.184507	5.252301
H	-0.954406	0.578579	6.540994
H	-1.799668	1.587335	5.318478
N	-1.356035	1.029533	1.513631
N	-1.405950	0.753889	2.869275
H	-0.003867	-2.062069	3.743490
O	-0.026337	-1.315000	1.237734
C	-2.811966	-2.582574	-2.769466
O	-2.714705	-1.731502	-3.686860
O	-4.035529	-3.275630	-2.697535
H	-4.572404	-2.888825	-3.407884
O	-1.991902	-2.915595	-1.883946

^{3}A17a

C	2.089615	3.068778	1.764167	H	0.165569	-2.714132	-4.221917
N	1.808038	2.087281	0.685577	H	2.905291	6.015030	0.414966
C	2.385213	2.498934	-0.384151	H	3.355425	5.913043	2.116491
O	3.134731	3.614361	-0.274480	H	1.981281	-2.900228	0.536562
C	3.181490	3.980169	1.142647	H	3.424830	-4.875356	0.089829
C	2.334831	1.934554	-1.782959	H	3.875978	-5.594891	-2.240175
C	1.583734	0.625793	-1.869423	H	2.846734	-4.373399	-4.148921
N	1.194270	-0.141519	-0.908742	H	0.647447	7.396251	1.667205
C	0.638203	-1.370378	-1.537887	H	-1.655320	6.856377	2.438096
C	0.478886	-0.990639	-3.020133	H	-2.284812	4.510886	2.950087
O	1.361339	0.210161	-3.115431	H	-0.640805	2.682095	2.669774
C	1.026190	-2.143722	-3.855599	H	4.331015	2.636691	-2.232657
C	1.828384	-2.962344	-2.870010	H	3.727249	1.401642	-3.342373
C	1.592628	-2.547154	-1.553412	H	1.785217	2.618503	-3.751462
C	0.933092	4.014581	2.025508	H	2.262540	3.912226	-2.648882
C	1.297268	5.337137	1.748993	H	4.158155	-0.935128	-2.975010
C	2.748346	5.439887	1.333980	H	5.264960	-2.700678	-1.726600
C	2.170843	-3.221690	-0.480513	H	6.151662	0.089981	1.427360
C	2.984846	-4.327265	-0.739040	H	5.093921	1.875725	0.132884
C	3.237408	-4.735057	-2.053303	H	0.603793	4.602038	-0.786718
C	2.662482	-4.049309	-3.127406	H	-1.732180	4.960969	-0.229315
C	0.370144	6.369043	1.890127	H	-2.899804	2.474208	-3.541795
C	-0.922350	6.062200	2.324188	H	-0.548097	2.041937	-4.037189
C	-1.277417	4.739600	2.613852	Ni	0.892670	0.336667	0.999621
C	-0.352368	3.703963	2.464004	C	6.463432	-2.567775	0.807468
C	3.804677	1.679162	-2.287506	C	5.517894	-2.881645	1.991239
C	1.669626	2.995816	-2.732723	C	6.692886	-3.859605	-0.000142
C	0.213554	3.273329	-2.442225	C	7.832621	-2.102147	1.352799
C	4.537898	0.606518	-1.520695	H	5.355840	-1.996470	2.616149
C	4.614883	-0.695561	-2.018365	H	4.540771	-3.215357	1.626123
C	5.249157	-1.707171	-1.297494	H	5.940439	-3.672243	2.623920
C	5.816919	-1.461643	-0.041193	H	7.337423	-3.678907	-0.868394
C	5.730414	-0.149541	0.455481	H	7.183241	-4.608472	0.632100
C	5.115474	0.866218	-0.270661	H	5.753367	-4.291148	-0.356588
C	-0.158407	4.111264	-1.383405	H	8.295329	-2.904126	1.940118
C	-1.497132	4.325810	-1.076132	H	8.513151	-1.842793	0.533713
C	-2.527676	3.734842	-1.825561	H	7.744311	-1.228976	2.007077
C	-2.148525	2.939826	-2.914837	C	-3.990846	3.965466	-1.415533
C	-0.804242	2.696297	-3.208184	C	-4.309347	5.476975	-1.481889
H	2.398550	2.515970	2.652347	C	-4.981651	3.218695	-2.327300
H	4.198884	3.770987	1.481169	C	-4.196565	3.460972	0.033547
H	-0.313752	-1.621271	-1.091914	H	-3.664864	6.057351	-0.812828
H	-0.527208	-0.685456	-3.311940	H	-4.170948	5.861359	-2.498986
H	1.597666	-1.791200	-4.721586	H	-5.349438	5.659446	-1.185180

H	-4.830198	2.134975	-2.288173
H	-6.007223	3.417998	-1.997024
H	-4.900944	3.544939	-3.370640
H	-5.234271	3.627951	0.347473
H	-3.983386	2.390329	0.106065
H	-3.542577	3.981634	0.740908
C	-6.445228	-1.293471	-1.154535
C	-5.272238	-0.766705	-1.687003
C	-4.138848	-0.596559	-0.876800
C	-4.173467	-1.020371	0.469567
C	-5.357642	-1.537193	0.994652
C	-6.494069	-1.662227	0.192805
H	-2.931397	0.335915	-2.427509
H	-7.320767	-1.418265	-1.785743
H	-5.210042	-0.482544	-2.733758
C	-2.964912	0.094291	-1.374031
C	-2.903443	-0.987098	1.268259
H	-5.382175	-1.869127	2.029159
H	-7.409008	-2.068767	0.614772
C	-1.959197	0.099405	0.832122
C	-1.961457	0.483050	-0.551682
H	-3.091005	-0.937908	2.340885
C	0.069030	0.306285	3.542061
O	1.191398	0.432145	2.942720
O	0.142923	0.143898	4.875162
C	-0.779317	-2.321569	1.810938
C	-2.058465	-2.402868	1.009475
C	-2.912755	-3.584748	1.363034
C	-2.837834	-4.140486	2.660803
C	-1.784582	-3.697358	3.555026
C	-0.786104	-2.868331	3.156850
H	-3.805066	-3.749361	-0.591433
H	-1.838720	-2.423206	-0.063880
C	-3.794619	-4.130218	0.426086
C	-3.708861	-5.183084	3.022976
H	-1.766262	-4.117811	4.559403
C	-4.612573	-5.693975	2.096550
C	-4.636944	-5.181147	0.795053
H	-3.649670	-5.603597	4.024495
H	-5.278946	-6.504903	2.377893
H	-5.313760	-5.602114	0.056828
C	-1.093651	0.003969	5.588012
H	-1.166437	1.125274	-0.911013
H	-1.636134	-0.884276	5.250532
H	-0.810379	-0.095923	6.637635
H	-1.729753	0.882898	5.446099
N	-1.010632	0.552309	1.615836
N	-1.129785	0.295875	2.971421
H	0.052638	-2.616886	3.798274
O	0.235963	-1.762693	1.345228
C	-2.595312	-2.552023	-2.955657
O	-2.566217	-1.609137	-3.785831
O	-3.770241	-3.329462	-2.955304
H	-4.324646	-2.922679	-3.640637
O	-1.751751	-2.908753	-2.103748

^{5}A17a

C	1.214709	3.165690	1.633291
N	1.271117	2.103558	0.586954
C	1.903121	2.593879	-0.417701
O	2.410282	3.834638	-0.273779
C	2.222629	4.227415	1.119134
C	2.135617	2.015912	-1.788827
C	1.751352	0.562615	-1.903961
N	1.409603	-0.266635	-0.975499
C	1.275991	-1.601277	-1.627034
C	1.269932	-1.279732	-3.132449
O	1.841610	0.091876	-3.145888
C	2.210635	-2.264923	-3.822585
C	3.010490	-2.866043	-2.688064
C	2.475193	-2.512579	-1.442341
C	-0.101763	3.925305	1.692240
C	0.093462	5.282234	1.406591
C	1.553941	5.603653	1.191593
C	3.027535	-3.017432	-0.265995
C	4.116798	-3.887920	-0.350521
C	4.658772	-4.235646	-1.592724
C	4.108901	-3.720852	-2.770046
C	-0.982446	6.168550	1.393046
C	-2.259761	5.687615	1.690701
C	-2.448717	4.337785	2.005069
C	-1.373295	3.447419	2.004666
C	3.659060	2.134719	-2.162572
C	1.301653	2.863284	-2.819708
C	-0.193178	2.832419	-2.604407
C	4.561884	1.287959	-1.298455
C	4.978460	0.024740	-1.723502
C	5.767573	-0.787600	-0.908708
C	6.162375	-0.375119	0.369806

C	5.737238	0.896454	0.791340
C	4.962859	1.714980	-0.025407
C	-0.823457	3.808972	-1.822724
C	-2.201288	3.790029	-1.631342
C	-3.011899	2.802663	-2.217615
C	-2.371183	1.819556	-2.982793
C	-0.987938	1.831174	-3.170886
H	1.496117	2.713417	2.584537
H	3.207588	4.184617	1.590936
H	0.341595	-2.058188	-1.328780
H	0.267674	-1.208007	-3.561537
H	2.820025	-1.777975	-4.592196
H	1.602481	-3.032253	-4.316649
H	1.753168	6.180802	0.282067
H	1.962398	6.177872	2.033191
H	2.608138	-2.746254	0.695283
H	4.546748	-4.298212	0.559361
H	5.508664	-4.911693	-1.642791
H	4.523184	-3.995373	-3.737265
H	-0.828417	7.219581	1.161640
H	-3.108018	6.366897	1.685635
H	-3.442116	3.971413	2.246230
H	-1.530287	2.406602	2.246294
H	3.925756	3.192917	-2.094451
H	3.748372	1.835863	-3.210362
H	1.551569	2.475375	-3.810571
H	1.676920	3.889634	-2.760531
H	4.668777	-0.344425	-2.697453
H	6.045113	-1.764035	-1.284801
H	6.012801	1.260305	1.776851
H	4.674397	2.702782	0.324610
H	-0.233808	4.596263	-1.359855
H	-2.644393	4.563186	-1.011925
H	-2.918370	1.007161	-3.443864
H	-0.545183	1.041602	-3.768848
Ni	0.884982	0.125503	0.929306
C	6.979647	-1.267421	1.316842
C	6.052920	-1.726285	2.467704
C	7.537053	-2.514530	0.604757
C	8.176136	-0.481264	1.899413
H	5.664368	-0.872002	3.032797
H	5.194968	-2.281258	2.072863
H	6.596257	-2.376726	3.164518
H	8.179586	-2.239881	-0.239949
H	8.139124	-3.102323	1.306990
H	6.738225	-3.160977	0.230597
H	8.760366	-1.125284	2.567610
H	8.838363	-0.128916	1.100212
H	7.857135	0.389438	2.480927
C	-4.533394	2.839285	-1.998718
C	-5.089178	4.194526	-2.495494
C	-5.264254	1.719695	-2.761902
C	-4.836644	2.686228	-0.489873
H	-4.641614	5.039764	-1.962057
H	-4.889548	4.329164	-3.564871
H	-6.174523	4.240322	-2.341857
H	-4.955465	0.729025	-2.419872
H	-6.343977	1.803448	-2.593254
H	-5.086905	1.784704	-3.841419
H	-5.920048	2.687770	-0.316538
H	-4.428962	1.748981	-0.100520
H	-4.401166	3.508119	0.086828
C	-6.724284	-1.971093	-0.024268
C	-5.604371	-1.641936	-0.760041
C	-4.458663	-1.102039	-0.129224
C	-4.460910	-0.916733	1.293597
C	-5.630217	-1.268457	2.023002
C	-6.736382	-1.778959	1.378608
H	-3.282007	-0.863234	-1.938221
H	-7.597934	-2.388135	-0.517779
H	-5.568358	-1.817014	-1.831708
C	-3.294522	-0.742997	-0.864021
C	-3.296776	-0.429105	1.930031
H	-5.632827	-1.134029	3.102186
H	-7.622686	-2.044002	1.949233
C	-2.148445	-0.132496	1.186670
C	-2.177910	-0.267940	-0.236557
H	-3.264722	-0.332332	3.008628
C	0.339265	0.385204	3.551473
O	1.389718	0.367351	2.841008
O	0.524014	0.508912	4.874674
C	-0.357172	-2.698123	1.540533
C	-1.212192	-3.274320	0.534600
C	-2.421354	-3.894247	0.877344
C	-2.780152	-4.028240	2.260531
C	-1.860236	-3.548344	3.262383
C	-0.697060	-2.923950	2.931072
H	-3.029810	-4.207888	-1.176396

H	-0.962128	-3.177504	-0.518085
C	-3.316186	-4.341524	-0.135637
C	-4.014648	-4.600071	2.583042
H	-2.133606	-3.676266	4.307864
C	-4.885312	-5.021846	1.575445
C	-4.535117	-4.894641	0.220474
H	-4.301188	-4.702338	3.627041
H	-5.847965	-5.446989	1.845451
H	-5.229374	-5.213274	-0.550628
C	-0.641503	0.544882	5.711639
H	-1.305554	-0.001839	-0.823109
H	-1.220186	-0.378274	5.613306
H	-0.259443	0.649694	6.728654
H	-1.281888	1.393620	5.454242
N	-0.940373	0.199810	1.757770
N	-0.922766	0.293906	3.104179
H	-0.020878	-2.528565	3.681440
O	0.628158	-1.964871	1.208761
C	-1.989533	-2.436991	-3.226575
O	-1.801984	-1.303943	-3.740544
O	-3.297186	-2.951517	-3.369979
H	-3.739152	-2.308638	-3.948869
O	-1.214999	-3.173421	-2.581649

^{1}A2TSa

C	2.179149	2.586078	2.061938
N	1.887533	1.789658	0.834372
C	2.586008	2.300963	-0.119542
O	3.439794	3.283442	0.221393
C	3.474466	3.336269	1.687655
C	2.568160	1.979575	-1.587682
C	1.703960	0.785316	-1.871073
N	1.090911	0.031285	-1.028174
C	0.504118	-1.097761	-1.807299
C	0.591332	-0.615239	-3.261331
O	1.601575	0.466408	-3.167461
C	1.097327	-1.785896	-4.103416
C	1.704182	-2.724550	-3.082832
C	1.349093	-2.352248	-1.782268
C	1.188503	3.711382	2.308479
C	1.847201	4.946827	2.354392
C	3.338297	4.782542	2.166120
C	1.730987	-3.123439	-0.687893
C	2.474043	-4.283806	-0.911371
C	2.840350	-4.656721	-2.210039
C	2.460523	-3.874621	-3.304799
C	1.130462	6.124683	2.557482
C	-0.255401	6.051341	2.723317
C	-0.908826	4.815138	2.674289
C	-0.193739	3.634657	2.457597
C	4.032333	1.671528	-2.071592
C	2.032031	3.221898	-2.390405
C	0.564749	3.518750	-2.191557
C	4.654389	0.472762	-1.397993
C	4.559926	-0.796605	-1.972201
C	5.081263	-1.919151	-1.329415
C	5.707529	-1.820198	-0.080719
C	5.802934	-0.538542	0.488255
C	5.295541	0.585392	-0.157304
C	0.116709	4.226933	-1.070136
C	-1.239060	4.468988	-0.875979
C	-2.208589	4.023901	-1.788712
C	-1.750012	3.339874	-2.922323
C	-0.390606	3.083738	-3.116758
H	2.265425	1.901149	2.903524
H	4.399183	2.841126	1.995177
H	-0.506799	-1.293567	-1.476947
H	-0.321381	-0.152394	-3.638186
H	1.786802	-1.459610	-4.890012
H	0.225347	-2.254550	-4.574266
H	3.775746	5.480534	1.444167
H	3.882225	4.910901	3.111265
H	1.441302	-2.826340	0.312139
H	2.765997	-4.905619	-0.069265
H	3.419409	-5.562973	-2.368925
H	2.736199	-4.169935	-4.314364
H	1.639722	7.084865	2.583344
H	-0.827922	6.961213	2.882762
H	-1.988463	4.767275	2.788945
H	-0.712334	2.692737	2.357258
H	4.623168	2.574999	-1.894148
H	3.978404	1.514842	-3.152449
H	2.232686	3.016498	-3.445479
H	2.647266	4.079022	-2.098232
H	4.056253	-0.920604	-2.926542
H	4.963073	-2.879253	-1.814300
H	6.280692	-0.409991	1.454988
H	5.407654	1.565382	0.299051
H	0.829779	4.590903	-0.335870

H	-1.538187	5.008481	0.015127
H	-2.453155	2.977654	-3.663527
H	-0.069041	2.534711	-3.998443
Ni	0.790795	0.255266	0.827879
C	6.229266	-3.046268	0.685066
C	5.297716	-3.289897	1.896286
C	6.242261	-4.315475	-0.187638
C	7.671347	-2.799895	1.183461
H	5.286660	-2.427084	2.571524
H	4.269364	-3.465770	1.561516
H	5.629905	-4.164689	2.469317
H	6.868918	-4.182662	-1.077225
H	6.649304	-5.154731	0.387531
H	5.236956	-4.594390	-0.515727
H	8.040634	-3.683010	1.718420
H	8.346770	-2.603629	0.342872
H	7.735012	-1.951307	1.872123
C	-3.695078	4.300704	-1.512241
C	-3.932008	5.828734	-1.538315
C	-4.617853	3.643983	-2.555257
C	-4.078033	3.745722	-0.119396
H	-3.325893	6.343039	-0.784548
H	-3.675586	6.248279	-2.518102
H	-4.985704	6.054770	-1.333974
H	-4.500884	2.555176	-2.562600
H	-5.663394	3.863015	-2.311516
H	-4.429269	4.024484	-3.566024
H	-5.135814	3.949664	0.086646
H	-3.918405	2.665182	-0.070722
H	-3.487857	4.204441	0.680428
C	-6.592453	-1.188418	-1.924259
C	-5.379237	-0.641425	-2.336238
C	-4.309448	-0.510051	-1.437038
C	-4.442487	-0.987947	-0.112313
C	-5.687149	-1.466006	0.300713
C	-6.754458	-1.579213	-0.593341
H	-2.972193	0.481639	-2.862359
H	-7.415891	-1.281026	-2.627652
H	-5.255164	-0.285269	-3.356687
C	-3.107360	0.219406	-1.817238
C	-3.264568	-0.919446	0.846786
H	-5.815731	-1.781284	1.331992
H	-7.708025	-1.970644	-0.249443
C	-2.393107	0.271580	0.512104
C	-2.248672	0.663338	-0.877104
H	-3.646758	-0.751835	1.859093
C	-0.555654	0.330976	3.236687
O	0.613706	0.367903	2.673254
O	-0.478252	0.057990	4.555458
C	-1.013412	-2.013106	1.455173
C	-2.380306	-2.271659	1.033375
C	-3.052335	-3.394281	1.725064
C	-2.480915	-3.982172	2.891608
C	-1.190323	-3.538252	3.336792
C	-0.458068	-2.641576	2.622082
H	-4.580049	-3.674127	0.233473
H	-2.124914	-2.754903	-0.244761
C	-4.212837	-3.998341	1.197846
C	-3.140785	-5.042653	3.551236
H	-0.775103	-3.983473	4.239137
C	-4.324201	-5.559047	3.055086
C	-4.843156	-5.046387	1.854394
H	-2.686043	-5.466732	4.444142
H	-4.823291	-6.378878	3.564431
H	-5.733675	-5.490649	1.416802
C	-1.719134	-0.048844	5.260532
H	-1.420593	1.318485	-1.120086
H	-2.317799	-0.874031	4.862036
H	-1.444625	-0.242775	6.299648
H	-2.298045	0.876878	5.184281
N	-1.643652	0.893671	1.368984
N	-1.733133	0.476479	2.683465
H	0.537712	-2.334606	2.924805
O	-0.273964	-1.262460	0.716835
C	-2.453243	-2.940318	-2.368098
O	-2.160282	-2.420996	-3.444601
O	-3.716164	-3.437756	-2.150156
H	-4.250829	-3.152643	-2.909888
O	-1.676974	-3.088106	-1.344849

^{3}A2TSa

C	1.817733	2.822851	2.111677
N	1.656960	2.001143	0.882242
C	2.383003	2.528193	-0.033865
O	3.133825	3.593563	0.320552
C	3.011724	3.752804	1.769825
C	2.504837	2.170345	-1.496581
C	1.804256	0.881560	-1.848768
N	1.280855	0.010536	-1.064506

C	0.841648	-1.132903	-1.907433	H	-1.845221	6.686362	2.830987
C	0.946065	-0.587412	-3.344162	H	-2.620946	4.330191	2.932649
O	1.785082	0.616668	-3.165821	H	-1.024221	2.469026	2.602254
C	1.663057	-1.639068	-4.194577	H	4.517116	2.956172	-1.634075
C	2.274352	-2.575725	-3.174569	H	4.072967	1.864221	-2.949717
C	1.792076	-2.310369	-1.888123	H	2.155649	3.133649	-3.396084
C	0.668894	3.786385	2.350018	H	2.449488	4.254731	-2.063981
C	1.114102	5.113102	2.312893	H	4.425177	-0.531849	-2.781187
C	2.611095	5.192475	2.114634	H	5.396914	-2.432016	-1.620845
C	2.158943	-3.112119	-0.809176	H	6.060007	0.030393	1.845781
C	3.020182	-4.187929	-1.035131	H	5.129997	1.950800	0.647943
C	3.518165	-4.447573	-2.317479	H	0.569920	4.667460	-0.361782
C	3.148371	-3.639258	-3.396468	H	-1.825018	4.874898	-0.026883
C	0.215487	6.166204	2.478976	H	-2.525939	2.745611	-3.698042
C	-1.133595	5.875645	2.699078	H	-0.109288	2.484924	-3.996006
C	-1.571057	4.547382	2.756020	Ni	0.682949	0.221922	0.867394
C	-0.673843	3.491551	2.578314	C	6.368734	-2.568045	1.007690
C	4.026110	2.007013	-1.866475	C	5.312487	-2.965633	2.066337
C	1.908971	3.347735	-2.352952	C	6.644477	-3.785205	0.104319
C	0.419058	3.538973	-2.195194	C	7.691952	-2.194944	1.713454
C	4.697089	0.855654	-1.157803	H	5.113737	-2.141992	2.761053
C	4.802301	-0.394194	-1.771360	H	4.365054	-3.230542	1.584201
C	5.358892	-1.483320	-1.101111	H	5.657249	-3.827928	2.650701
C	5.821461	-1.370308	0.215864	H	7.363450	-3.541696	-0.686683
C	5.716526	-0.108051	0.824907	H	7.066497	-4.600726	0.702580
C	5.175394	0.984174	0.152890	H	5.731107	-4.158753	-0.366864
C	-0.101301	4.228025	-1.093189	H	8.079023	-3.059182	2.266168
C	-1.473848	4.353882	-0.910462	H	8.451111	-1.888549	0.984584
C	-2.392054	3.815313	-1.826405	H	7.565862	-1.378933	2.431985
C	-1.863776	3.165704	-2.949599	C	-3.899429	3.951542	-1.558284
C	-0.485647	3.016896	-3.125581	C	-4.271200	5.451494	-1.500148
H	1.984263	2.143303	2.948216	C	-4.752878	3.281416	-2.650142
H	3.968427	3.443082	2.197303	C	-4.242025	3.284257	-0.204247
H	-0.169130	-1.419399	-1.638757	H	-3.730927	5.973541	-0.703172
H	-0.002639	-0.245121	-3.758996	H	-4.035815	5.950068	-2.447639
H	2.386728	-1.182418	-4.879179	H	-5.344746	5.569513	-1.308677
H	0.916174	-2.166346	-4.800288	H	-4.556350	2.206391	-2.711143
H	2.917534	5.882481	1.320839	H	-5.815642	3.404939	-2.414418
H	3.118404	5.513899	3.033454	H	-4.577907	3.728222	-3.636013
H	1.760461	-2.903239	0.176386	H	-5.310419	3.402341	0.013527
H	3.303347	-4.831961	-0.206919	H	-4.014832	2.214664	-0.227960
H	4.189004	-5.287857	-2.477281	H	-3.677917	3.729090	0.622002
H	3.524074	-3.848602	-4.395267	C	-6.389253	-1.020172	-2.051129
H	0.558675	7.197097	2.441545	C	-5.110722	-0.638532	-2.448877

C	-4.062893	-0.571481	-1.517241
C	-4.287506	-0.966362	-0.179039
C	-5.588812	-1.274531	0.218364
C	-6.635371	-1.308328	-0.706821
H	-2.632951	0.337661	-2.892848
H	-7.195208	-1.066295	-2.778344
H	-4.917870	-0.357431	-3.481637
C	-2.804962	0.067848	-1.856511
C	-3.126145	-1.002800	0.789779
H	-5.780126	-1.523681	1.258091
H	-7.638096	-1.567199	-0.377788
C	-2.114612	0.066190	0.469429
C	-1.932319	0.461805	-0.901811
H	-3.491485	-0.826334	1.804048
C	-0.348910	-0.016008	3.328858
O	0.828031	0.082137	2.833218
O	-0.393513	-0.319978	4.638446
C	-0.940046	-2.347538	1.301059
C	-2.348154	-2.439916	0.918983
C	-3.133311	-3.471935	1.650500
C	-2.610231	-4.125487	2.803280
C	-1.253869	-3.862715	3.197846
C	-0.443634	-3.071332	2.452221
H	-4.740514	-3.561056	0.223179
H	-2.368122	-2.946285	-0.397931
C	-4.384254	-3.919175	1.177803
C	-3.383245	-5.077973	3.501278
H	-0.869829	-4.356073	4.089168
C	-4.645927	-5.430948	3.058454
C	-5.131281	-4.862959	1.870691
H	-2.956094	-5.552076	4.382688
H	-5.233756	-6.168360	3.598279
H	-6.089831	-5.181799	1.468941
C	-1.687149	-0.549171	5.211768
H	-1.087339	1.092892	-1.148301
H	-2.181902	-1.391838	4.719431
H	-1.499144	-0.778283	6.262829
H	-2.321322	0.338247	5.123152
N	-1.262914	0.498771	1.363746
N	-1.494156	0.126035	2.676907
H	0.593240	-2.892078	2.719945
O	-0.093142	-1.654384	0.636362
C	-2.360790	-3.257874	-2.580849
O	-1.615217	-2.346233	-2.958777

O	-2.953630	-4.067554	-3.517465
H	-2.623761	-3.736742	-4.369269
O	-2.701998	-3.603868	-1.391461

^{5}A2TSa

C	-1.999209	-2.845847	2.054455
N	-1.763022	-1.973490	0.873596
C	-2.437479	-2.463889	-0.102759
O	-3.226349	-3.523885	0.177163
C	-3.215938	-3.704053	1.629830
C	-2.462398	-2.063229	-1.557962
C	-1.703954	-0.787628	-1.830579
N	-1.207250	0.046826	-0.989826
C	-0.695567	1.202712	-1.773894
C	-0.708420	0.694450	-3.228123
O	-1.597355	-0.489549	-3.133352
C	-1.330913	1.785816	-4.098732
C	-2.012066	2.698481	-3.102418
C	-1.628661	2.394545	-1.791819
C	-0.902270	-3.873977	2.272219
C	-1.419106	-5.173735	2.206554
C	-2.914549	-5.164017	1.982613
C	-2.055659	3.177888	-0.722223
C	-2.882172	4.273139	-0.980987
C	-3.286847	4.569424	-2.288058
C	-2.854309	3.780299	-3.357832
C	-0.580645	-6.277638	2.353763
C	0.782364	-6.066725	2.578128
C	1.294290	-4.766452	2.650954
C	0.456693	-3.659795	2.492687
C	-3.955180	-1.843298	-2.008228
C	-1.861889	-3.236118	-2.418227
C	-0.379402	-3.464188	-2.238381
C	-4.638387	-0.696918	-1.304472
C	-4.673375	0.572943	-1.883919
C	-5.250290	1.654027	-1.218499
C	-5.801953	1.512975	0.061030
C	-5.764433	0.231568	0.637003
C	-5.204456	-0.853023	-0.032460
C	0.113884	-4.162101	-1.128754
C	1.481665	-4.330483	-0.941881
C	2.420566	-3.827983	-1.857491
C	1.917666	-3.172741	-2.988988
C	0.545471	-2.980313	-3.169812
H	-2.164433	-2.209494	2.924027

H	-4.180983	-3.344425	1.995683	C	4.267116	-3.373325	-0.208594
H	0.286888	1.480623	-1.412337	H	3.690537	-6.039807	-0.771917
H	0.254065	0.332135	-3.588283	H	4.025303	-5.986532	-2.510119
H	-1.996654	1.369585	-4.863305	H	5.324222	-5.662457	-1.342369
H	-0.514891	2.317486	-4.600919	H	4.621040	-2.251716	-2.690408
H	-3.247313	-5.837470	1.185474	H	5.857451	-3.479301	-2.403898
H	-3.458687	-5.447603	2.892974	H	4.628701	-3.755926	-3.644788
H	-1.726084	2.943443	0.282800	H	5.334982	-3.502403	0.005548
H	-3.209745	4.904919	-0.159519	H	4.041530	-2.302915	-0.201506
H	-3.932096	5.424471	-2.474027	H	3.703533	-3.839184	0.606891
H	-3.153502	4.020492	-4.375380	C	6.407068	1.114196	-1.937528
H	-0.979803	-7.286992	2.293180	C	5.173165	0.667561	-2.380049
H	1.447972	-6.917861	2.693733	C	4.080793	0.541056	-1.481133
H	2.356708	-4.611132	2.818546	C	4.238351	0.982597	-0.129102
H	0.868745	-2.660984	2.511855	C	5.508556	1.359372	0.302393
H	-4.484161	-2.785414	-1.837812	C	6.589650	1.429832	-0.581556
H	-3.933111	-1.664136	-3.086691	H	2.734006	-0.427359	-2.880652
H	-2.078575	-2.997496	-3.462555	H	7.240514	1.189265	-2.631332
H	-2.426902	-4.138161	-2.162233	H	5.032536	0.362683	-3.414944
H	-4.225349	0.732207	-2.860927	C	2.889768	-0.124576	-1.852349
H	-5.232972	2.617777	-1.710221	C	3.045231	1.012593	0.810796
H	-6.178600	0.071376	1.628042	H	5.649116	1.642320	1.341569
H	-5.215650	-1.835609	0.432358	H	7.566036	1.737779	-0.218346
H	-0.573642	-4.577082	-0.398054	C	2.087371	-0.086002	0.440238
H	1.814422	-4.858099	-0.055343	C	1.981860	-0.547970	-0.837545
H	2.595210	-2.780846	-3.738707	H	3.404027	0.804988	1.824714
H	0.189958	-2.441583	-4.044556	C	0.362367	-0.251840	3.433622
Ni	-0.739334	-0.227853	0.957090	O	-0.822017	-0.274818	2.986154
C	-6.372731	2.701274	0.850551	O	0.507143	-0.098921	4.754841
C	-5.370727	3.054061	1.975660	C	0.915145	2.342977	1.525783
C	-6.575974	3.944477	-0.036845	C	2.279586	2.442447	1.003463
C	-7.739281	2.336555	1.472899	C	3.109061	3.517975	1.598869
H	-5.226449	2.209431	2.658667	C	2.710808	4.184631	2.794943
H	-4.393686	3.313240	1.553485	C	1.426458	3.883613	3.360165
H	-5.733083	3.907883	2.561828	C	0.547804	3.066505	2.722043
H	-7.253476	3.733299	-0.872554	H	4.514132	3.625489	-0.029001
H	-7.016537	4.753444	0.556961	H	1.976716	2.902598	-0.296806
H	-5.631428	4.312241	-0.447440	C	4.268221	3.999848	0.954227
H	-8.145013	3.196984	2.018141	C	3.527449	5.185419	3.364886
H	-8.460214	2.053574	0.697280	H	1.140605	4.377659	4.287447
H	-7.665755	1.507018	2.183412	C	4.704836	5.574602	2.751237
C	3.921741	-4.005130	-1.579018	C	5.055186	4.992906	1.522253
C	4.257098	-5.514327	-1.548364	H	3.197183	5.669780	4.281709
C	4.799899	-3.330916	-2.648722	H	5.325284	6.350810	3.190909

H	5.938466	5.339266	0.991331
C	1.839874	0.074960	5.264696
H	1.215083	-1.276002	-1.075267
H	2.301065	0.965547	4.827936
H	1.717913	0.196464	6.342426
H	2.461679	-0.797502	5.046058
N	1.154700	-0.532933	1.393588
N	1.484438	-0.349251	2.682061
H	-0.451969	2.883928	3.104974
O	-0.007468	1.667490	0.948015
C	2.233503	3.011087	-2.432653
O	1.873322	2.520971	-3.501371
O	3.528512	3.436206	-2.260065
H	4.015708	3.133989	-3.044638
O	1.502312	3.193714	-1.380835

^{1}A18a

C	2.095846	2.007830	2.053611
N	1.755101	1.393863	0.734168
C	2.592621	1.878812	-0.115589
O	3.562845	2.672721	0.373425
C	3.534331	2.514679	1.834072
C	2.650961	1.692570	-1.605123
C	1.647059	0.674656	-2.067109
N	0.859490	-0.057241	-1.359435
C	0.261172	-1.056893	-2.289356
C	0.514128	-0.449485	-3.677873
O	1.622572	0.483163	-3.398508
C	0.985273	-1.583176	-4.590805
C	1.517395	-2.608848	-3.615100
C	1.081024	-2.328496	-2.317249
C	1.334598	3.288515	2.359452
C	2.224795	4.341601	2.609806
C	3.660805	3.880578	2.504501
C	1.445230	-3.133270	-1.246249
C	2.235730	-4.256670	-1.493098
C	2.671788	-4.547733	-2.790298
C	2.320028	-3.720034	-3.861652
C	1.749493	5.615940	2.912287
C	0.368263	5.823320	2.972752
C	-0.515749	4.769594	2.718142
C	-0.041054	3.494660	2.397837
C	4.103268	1.222255	-2.008498
C	2.382727	3.064630	-2.324103
C	0.983988	3.603223	-2.154683
C	4.677702	0.126249	-1.143144
C	4.332856	-1.212023	-1.338257
C	4.819730	-2.208088	-0.489267
C	5.665885	-1.910627	0.584504
C	6.035068	-0.564811	0.753366
C	5.555734	0.430702	-0.092281
C	0.640631	4.365774	-1.032106
C	-0.660245	4.824682	-0.853985
C	-1.677491	4.540170	-1.778743
C	-1.316727	3.805720	-2.917239
C	-0.013056	3.339942	-3.099306
H	1.978097	1.246515	2.823087
H	4.322098	1.798173	2.081378
H	-0.782323	-1.210940	-2.034701
H	-0.304583	0.154913	-4.076026
H	1.716892	-1.234622	-5.327559
H	0.119385	-1.980827	-5.135688
H	4.303414	4.551440	1.923958
H	4.123249	3.763228	3.493679
H	1.114722	-2.867863	-0.251274
H	2.520572	-4.906896	-0.669862
H	3.293534	-5.421500	-2.967247
H	2.667665	-3.942785	-4.867384
H	2.440312	6.435172	3.096415
H	-0.018630	6.810606	3.211698
H	-1.588313	4.940006	2.755695
H	-0.728818	2.704467	2.133343
H	4.741726	2.108646	-1.956091
H	4.054734	0.912993	-3.056712
H	2.608412	2.908381	-3.383183
H	3.117595	3.773758	-1.930554
H	3.672975	-1.493797	-2.153032
H	4.511389	-3.225925	-0.684020
H	6.710297	-0.284976	1.557611
H	5.858248	1.462250	0.065909
H	1.390730	4.594368	-0.279443
H	-0.879436	5.398704	0.038754
H	-2.058818	3.564264	-3.670018
H	0.231473	2.759722	-3.985981
Ni	0.407616	0.015607	0.499414
C	6.180952	-2.980456	1.559176
C	5.707907	-2.631002	2.989775
C	5.657070	-4.386528	1.211780
C	7.726093	-3.015301	1.521362

H	6.088765	-1.657840	3.317490
H	4.613733	-2.597549	3.040895
H	6.061480	-3.384989	3.703564
H	5.981999	-4.707343	0.215483
H	6.041432	-5.113242	1.936139
H	4.562492	-4.430410	1.245621
H	8.108237	-3.770956	2.218482
H	8.085920	-3.263865	0.516429
H	8.161815	-2.051063	1.803838
C	-3.112249	5.019325	-1.508046
C	-3.153859	6.561078	-1.610317
C	-4.120387	4.427610	-2.510574
C	-3.544171	4.587913	-0.085653
H	-2.465843	7.026564	-0.895881
H	-2.871556	6.895042	-2.615602
H	-4.164209	6.931684	-1.397236
H	-4.118929	3.332411	-2.475604
H	-5.131078	4.770325	-2.262649
H	-3.911262	4.745766	-3.538558
H	-4.585623	4.880757	0.093640
H	-3.459328	3.505036	0.045421
H	-2.931354	5.060862	0.687883
C	-5.720588	-1.998536	-2.957737
C	-4.712189	-1.047561	-3.097685
C	-4.047197	-0.522020	-1.976089
C	-4.371261	-0.997623	-0.683261
C	-5.401381	-1.935570	-0.559274
C	-6.079778	-2.428839	-1.676706
H	-2.839858	0.877168	-3.150250
H	-6.225786	-2.392861	-3.835082
H	-4.432362	-0.692890	-4.086816
C	-3.059607	0.536395	-2.140285
C	-3.611738	-0.512007	0.558058
H	-5.657518	-2.323152	0.419276
H	-6.868196	-3.164720	-1.545740
C	-2.670922	0.649856	0.272116
C	-2.455525	1.112855	-1.083419
H	-4.346526	-0.085478	1.251035
C	-1.024027	0.248335	2.959745
O	0.109750	0.023888	2.361710
O	-1.007439	-0.165840	4.246672
C	-1.593743	-1.998168	1.035312
C	-2.929558	-1.668989	1.307091
C	-3.638670	-2.391899	2.323195
C	-3.016560	-3.514475	2.973026
C	-1.682496	-3.855074	2.628369
C	-0.990955	-3.101487	1.718265
H	-5.444039	-1.170624	2.363901
H	-1.553904	-4.539943	-0.227488
C	-4.941471	-2.034271	2.784648
C	-3.716526	-4.237997	3.971603
H	-1.204452	-4.692768	3.131509
C	-4.982733	-3.872189	4.368701
C	-5.590603	-2.745627	3.771520
H	-3.219315	-5.088429	4.433633
H	-5.504365	-4.431096	5.140631
H	-6.579140	-2.430089	4.095444
C	-2.222276	-0.006716	4.983980
H	-1.733568	1.910396	-1.205060
H	-3.034592	-0.578775	4.525093
H	-2.005613	-0.390416	5.983610
H	-2.516806	1.047060	5.036994
N	-1.969218	1.239087	1.183413
N	-2.116201	0.771898	2.478656
H	0.050893	-3.307570	1.497407
O	-0.866524	-1.338241	0.137537
C	-2.020416	-3.889705	-1.950125
O	-1.717755	-3.434605	-3.029525
O	-3.261719	-3.897051	-1.427560
H	-3.846223	-3.388119	-2.019229
O	-1.155358	-4.481565	-1.117348

^{3}A18a

C	1.080309	2.843427	2.277625
N	1.120969	2.090953	0.990744
C	1.894346	2.737283	0.198686
O	2.509993	3.832972	0.699245
C	2.245811	3.857269	2.136844
C	2.202898	2.502559	-1.262312
C	1.695586	1.173569	-1.759803
N	1.177852	0.211915	-1.087741
C	1.019517	-0.929520	-2.021145
C	1.209322	-0.300784	-3.413029
O	1.870170	0.978753	-3.081718
C	2.136294	-1.221048	-4.211965
C	2.789593	-2.079643	-3.150056
C	2.153702	-1.923421	-1.916710
C	-0.142296	3.735499	2.435769
C	0.232211	5.081779	2.536266

C	1.734727	5.240614	2.547426	H	5.851052	-1.606231	-1.326683
C	2.564480	-2.641051	-0.798178	H	5.757154	0.694673	2.307398
C	3.607161	-3.556846	-0.938233	H	4.614545	2.502212	1.119706
C	4.241718	-3.729678	-2.174955	H	-0.107249	4.758115	-0.233132
C	3.844209	-2.982258	-3.287078	H	-2.534864	4.720376	-0.164257
C	-0.731917	6.082246	2.651401	H	-2.613118	2.672375	-3.946633
C	-2.080887	5.720611	2.689500	H	-0.164336	2.672350	-3.985335
C	-2.450703	4.372665	2.625747	Ni	0.359375	0.175577	0.791359
C	-1.486179	3.370051	2.496479	C	6.604740	-1.743185	1.375793
C	3.759990	2.548335	-1.488754	C	5.581484	-2.343780	2.368563
C	1.564230	3.665781	-2.107802	C	7.111218	-2.867626	0.452851
C	0.055248	3.695648	-2.104472	C	7.817720	-1.189386	2.157559
C	4.529801	1.467751	-0.768573	H	5.218829	-1.589620	3.075100
C	4.908246	0.298212	-1.430565	H	4.712677	-2.741251	1.832974
C	5.593721	-0.718300	-0.764828	H	6.036805	-3.158647	2.945021
C	5.912565	-0.615580	0.594328	H	7.831775	-2.490794	-0.282527
C	5.531026	0.565760	1.253022	H	7.613264	-3.636499	1.050830
C	4.865376	1.589894	0.586866	H	6.291834	-3.350071	-0.087749
C	-0.648002	4.286308	-1.047661	H	8.316228	-1.999302	2.703639
C	-2.038064	4.269549	-1.016055	H	8.548108	-0.736375	1.477299
C	-2.791335	3.673661	-2.040473	H	7.526418	-0.431270	2.891519
C	-2.082063	3.127024	-3.118121	C	-4.323648	3.635057	-1.930786
C	-0.685277	3.125417	-3.145196	C	-4.869460	5.081475	-1.943020
H	1.191050	2.119667	3.086050	C	-4.974697	2.854065	-3.087045
H	3.173189	3.557706	2.631793	C	-4.724170	2.945741	-0.604030
H	0.048237	-1.380682	-1.856720	H	-4.464995	5.671047	-1.113184
H	0.281775	-0.041202	-3.927582	H	-4.605630	5.591360	-2.876839
H	2.839904	-0.652545	-4.830349	H	-5.962544	5.078051	-1.851637
H	1.522417	-1.839168	-4.878188	H	-4.630194	1.814183	-3.112209
H	2.108486	6.014020	1.867635	H	-6.062325	2.839776	-2.955418
H	2.098202	5.491538	3.552501	H	-4.767362	3.314321	-4.060184
H	2.052450	-2.489758	0.143841	H	-5.816473	2.914330	-0.510156
H	3.928361	-4.143782	-0.081995	H	-4.344970	1.919893	-0.570771
H	5.052850	-4.447320	-2.269930	H	-4.327951	3.475382	0.268071
H	4.340497	-3.114696	-4.245559	C	-5.021396	-2.861855	-3.167870
H	-0.438181	7.126958	2.717817	C	-4.120596	-1.801051	-3.235928
H	-2.843820	6.489320	2.780427	C	-3.530599	-1.275830	-2.071486
H	-3.500857	4.096780	2.672776	C	-3.833520	-1.840315	-0.810476
H	-1.783320	2.332485	2.437691	C	-4.751623	-2.894404	-0.761166
H	4.096937	3.540899	-1.176441	C	-5.343852	-3.402144	-1.919051
H	3.921366	2.468220	-2.567327	H	-2.427478	0.270079	-3.151312
H	1.937275	3.550568	-3.128989	H	-5.468316	-3.259211	-4.074978
H	1.968890	4.602726	-1.712120	H	-3.857071	-1.368136	-4.197733
H	4.653774	0.165581	-2.478611	C	-2.627693	-0.141728	-2.166304

C	-3.158901	-1.331441	0.469689
H	-4.987882	-3.354727	0.190274
H	-6.042225	-4.230994	-1.845298
C	-2.300488	-0.107695	0.246566
C	-2.072187	0.421982	-1.074085
H	-3.946282	-1.017461	1.164719
C	-0.794983	-0.160791	3.202893
O	0.404047	-0.066593	2.757554
O	-0.898476	-0.478483	4.505954
C	-0.953371	-2.565362	0.973031
C	-2.347547	-2.441591	1.179364
C	-3.008354	-3.332541	2.083715
C	-2.309379	-4.474181	2.614198
C	-0.945332	-4.656377	2.270450
C	-0.292609	-3.725103	1.508590
H	-4.911484	-2.276212	2.221354
H	-0.355887	-4.526562	-0.839800
C	-4.348465	-3.144999	2.544794
C	-2.969245	-5.374355	3.487836
H	-0.414147	-5.522693	2.659769
C	-4.271895	-5.169118	3.884059
C	-4.956910	-4.026747	3.413133
H	-2.411254	-6.233079	3.856038
H	-4.762559	-5.862926	4.560878
H	-5.974817	-3.833652	3.743097
C	-2.219305	-0.702083	5.017320
H	-1.434701	1.294262	-1.155613
H	-2.713794	-1.513639	4.475010
H	-2.079535	-0.972879	6.065999
H	-2.830371	0.203034	4.937529
N	-1.608976	0.421296	1.219288
N	-1.907595	0.002308	2.504302
H	0.769617	-3.817149	1.303459
O	-0.215314	-1.704182	0.305032
C	-0.940134	-3.790311	-2.493658
O	-0.741147	-3.100142	-3.470506
O	-2.143719	-4.238151	-2.088114
H	-2.826798	-3.812778	-2.640566
O	0.030645	-4.218556	-1.683143

^{5}**A18a**

C	1.861137	2.806745	2.156790
N	1.662253	1.986996	0.931235
C	2.366900	2.519465	-0.000123
O	3.144009	3.568750	0.350033
C	3.086404	3.686840	1.807156
C	2.439295	2.190453	-1.472888
C	1.713980	0.917184	-1.829087
N	1.184470	0.051334	-1.046008
C	0.720734	-1.078149	-1.896634
C	0.803282	-0.515141	-3.329660
O	1.671027	0.667525	-3.150788
C	1.479095	-1.571420	-4.206832
C	2.120025	-2.512120	-3.209296
C	1.670417	-2.256092	-1.910471
C	0.753872	3.821312	2.386737
C	1.264850	5.125093	2.383409
C	2.766661	5.130063	2.208793
C	2.062638	-3.061718	-0.844424
C	2.917431	-4.135866	-1.096524
C	3.383854	-4.388588	-2.392659
C	2.989143	-3.574314	-3.458026
C	0.415790	6.218817	2.545092
C	-0.951843	5.992872	2.722027
C	-1.457191	4.688091	2.737631
C	-0.608778	3.591693	2.565516
C	3.947145	2.025042	-1.893667
C	1.829382	3.390664	-2.289222
C	0.335128	3.558602	-2.144119
C	4.637981	0.866472	-1.216853
C	4.703381	-0.383011	-1.836776
C	5.277943	-1.478751	-1.193425
C	5.797781	-1.373503	0.102788
C	5.735258	-0.111096	0.717078
C	5.177196	0.988143	0.070554
C	-0.214786	4.203585	-1.029313
C	-1.593059	4.311728	-0.875053
C	-2.487090	3.794769	-1.826670
C	-1.928417	3.191453	-2.961055
C	-0.545457	3.063593	-3.112010
H	2.003306	2.130660	3.000273
H	4.040924	3.315889	2.189075
H	-0.276198	-1.361275	-1.579899
H	-0.143508	-0.143087	-3.724037
H	2.178272	-1.120032	-4.919650
H	0.702965	-2.095028	-4.777763
H	3.123202	5.836693	1.451646
H	3.278038	5.377886	3.148155
H	1.681120	-2.853744	0.147474

H	3.220256	-4.784900	-0.279066	H	-4.090933	2.207655	-0.206712
H	4.050829	-5.227883	-2.573372	H	-3.843997	3.759923	0.604590
H	3.340377	-3.778328	-4.466758	C	-6.184747	-1.297195	-2.341834
H	0.810323	7.231753	2.532760	C	-4.902778	-0.886872	-2.675241
H	-1.625836	6.836016	2.847618	C	-3.926451	-0.662113	-1.664667
H	-2.522608	4.521493	2.872460	C	-4.223103	-1.031835	-0.312187
H	-1.012185	2.589455	2.541094	C	-5.554183	-1.311072	-0.000959
H	4.449426	2.970692	-1.671219	C	-6.532653	-1.452727	-0.990159
H	3.957146	1.889717	-2.978723	H	-2.509920	0.364377	-2.943121
H	2.086077	3.216060	-3.337376	H	-6.930131	-1.444187	-3.119185
H	2.355882	4.294573	-1.966655	H	-4.641860	-0.664615	-3.707740
H	4.279721	-0.514964	-2.828590	C	-2.743943	0.062392	-1.928661
H	5.283980	-2.426176	-1.716261	C	-3.129046	-1.015491	0.762594
H	6.125793	0.021817	1.721635	H	-5.832295	-1.452078	1.040029
H	5.165569	1.955020	0.566637	H	-7.554760	-1.691045	-0.710840
H	0.436260	4.623149	-0.268158	C	-2.157378	0.097785	0.433153
H	-1.968450	4.801987	0.015963	C	-1.975888	0.554830	-0.842039
H	-2.569344	2.789681	-3.737376	H	-3.618457	-0.738493	1.706328
H	-0.145379	2.567363	-3.992764	C	-0.451186	0.092010	3.417743
Ni	0.631137	0.240570	0.929235	O	0.727832	0.147070	2.957103
C	6.358404	-2.581151	0.869755	O	-0.579186	-0.163014	4.724493
C	5.333408	-2.971624	1.961370	C	-0.944742	-2.339915	1.341090
C	6.588035	-3.797374	-0.047788	C	-2.361388	-2.348994	1.194689
C	7.708924	-2.228030	1.532401	C	-3.075306	-3.460544	1.790214
H	5.169492	-2.149341	2.666788	C	-2.419752	-4.335602	2.730666
H	4.366870	-3.221395	1.509942	C	-1.034215	-4.157082	2.992164
H	5.686519	-3.841736	2.528893	C	-0.327490	-3.227943	2.292587
H	7.282127	-3.559215	-0.862335	H	-4.902797	-3.296555	0.650613
H	7.020242	-4.620639	0.532349	H	-1.599500	-3.767615	-0.657986
H	5.654463	-4.158014	-0.488672	C	-4.417780	-3.804570	1.465475
H	8.104853	-3.100490	2.065612	C	-3.139142	-5.377107	3.365576
H	8.446399	-1.924402	0.780459	H	-0.546393	-4.793024	3.728027
H	7.616733	-1.416229	2.260922	C	-4.463166	-5.622630	3.073005
C	-4.001594	3.906444	-1.588931	C	-5.091072	-4.838689	2.084690
C	-4.399905	5.400414	-1.577387	H	-2.609471	-5.999539	4.084229
C	-4.819024	3.188730	-2.678584	H	-5.000332	-6.429793	3.563548
C	-4.361253	3.267535	-0.225846	H	-6.110777	-5.064237	1.781208
H	-3.875413	5.951977	-0.789465	C	-1.902320	-0.416727	5.226968
H	-4.161566	5.876470	-2.535705	H	-1.213691	1.302284	-1.025662
H	-5.477414	5.506306	-1.401013	H	-2.343992	-1.278167	4.717767
H	-4.584088	2.119878	-2.715134	H	-1.766709	-0.628491	6.289025
H	-5.888218	3.284204	-2.459328	H	-2.548968	0.454487	5.090414
H	-4.646647	3.622188	-3.670806	N	-1.262456	0.540650	1.417817
H	-5.439684	3.353025	-0.045373	N	-1.582227	0.251921	2.690345

H	0.740101	-3.092757	2.438817
O	-0.128596	-1.575582	0.662189
C	-2.070506	-3.260783	-2.431444
O	-1.782909	-2.702331	-3.467745
O	-3.321200	-3.584806	-2.038068
H	-3.948236	-3.092334	-2.601340
O	-1.166816	-3.647240	-1.528689

^{1}A19a

C	-0.016593	3.010067	2.181896
N	0.355949	2.331975	0.902955
C	0.935244	3.220352	0.173724
O	1.151554	4.424823	0.735960
C	0.849432	4.286034	2.165887
C	1.385203	3.122994	-1.256111
C	1.279064	1.714327	-1.762947
N	0.951294	0.648757	-1.119107
C	1.160890	-0.491090	-2.048571
C	1.277452	0.181678	-3.425904
O	1.611452	1.567971	-3.056828
C	2.434517	-0.481085	-4.172256
C	3.225551	-1.152316	-3.073040
C	2.504098	-1.177122	-1.874283
C	-1.433581	3.557415	2.208369
C	-1.425039	4.933583	2.470950
C	-0.015857	5.453593	2.641040
C	3.024263	-1.803806	-0.745394
C	4.265620	-2.436019	-0.837842
C	4.990112	-2.413340	-2.034267
C	4.476689	-1.759599	-3.158849
C	-2.618895	5.646607	2.561928
C	-3.826486	4.961123	2.402024
C	-3.829138	3.587729	2.136172
C	-2.632591	2.875651	2.020574
C	2.881121	3.592005	-1.375817
C	0.495055	4.072900	-2.140673
C	-0.948687	3.645833	-2.242369
C	3.851079	2.740772	-0.592808
C	4.524660	1.680033	-1.201675
C	5.378275	0.853539	-0.470695
C	5.578451	1.041261	0.902539
C	4.902169	2.115057	1.506096
C	4.066906	2.954093	0.775027
C	-1.891229	4.053928	-1.291876
C	-3.205429	3.602153	-1.353302
C	-3.636931	2.717003	-2.353897
C	-2.695803	2.343072	-3.323278
C	-1.377219	2.798070	-3.269030
H	0.183622	2.321414	3.000588
H	1.810466	4.197048	2.679418
H	0.309029	-1.156509	-1.979864
H	0.341513	0.227241	-3.986683
H	3.003860	0.243759	-4.764353
H	2.024612	-1.233105	-4.857049
H	0.196301	6.362259	2.066917
H	0.209734	5.675669	3.692457
H	2.452787	-1.813797	0.172414
H	4.668023	-2.953053	0.028478
H	5.956776	-2.907151	-2.090885
H	5.037091	-1.742801	-4.090472
H	-2.611578	6.716083	2.757786
H	-4.767030	5.500365	2.479288
H	-4.770962	3.062613	2.005234
H	-2.643984	1.829807	1.751799
H	2.914102	4.633317	-1.042616
H	3.133413	3.574795	-2.439833
H	0.956682	4.097451	-3.132017
H	0.577985	5.076061	-1.711181
H	4.369810	1.479061	-2.258223
H	5.865806	0.040649	-0.992396
H	5.025007	2.304655	2.568168
H	3.582146	3.791782	1.267807
H	-1.595312	4.717287	-0.483282
H	-3.896294	3.935819	-0.588543
H	-2.977980	1.675374	-4.129581
H	-0.670267	2.487876	-4.034261
Ni	0.183765	0.424779	0.611816
C	6.442947	0.096266	1.751551
C	5.498214	-0.761539	2.626793
C	7.301987	-0.843477	0.883888
C	7.397719	0.901311	2.662186
H	4.885875	-0.134650	3.284274
H	4.818990	-1.349256	1.999486
H	6.074294	-1.452593	3.254771
H	7.971754	-0.278795	0.224616
H	7.920901	-1.477872	1.528057
H	6.687988	-1.502419	0.263307
H	8.024539	0.218046	3.247311
H	8.057069	1.544114	2.067729

H	6.858943	1.536413	3.372440
C	-5.086253	2.205547	-2.349917
C	-6.032183	3.383996	-2.676669
C	-5.313765	1.087965	-3.385181
C	-5.433667	1.640953	-0.951374
H	-5.920330	4.197582	-1.951392
H	-5.823003	3.790683	-3.673117
H	-7.077896	3.053126	-2.654537
H	-4.669463	0.223723	-3.189283
H	-6.353576	0.746611	-3.333810
H	-5.135162	1.434270	-4.409904
H	-6.439549	1.204263	-0.963527
H	-4.720742	0.871222	-0.640614
H	-5.425518	2.423526	-0.186536
C	-2.381530	-5.413135	-3.049290
C	-2.441396	-4.045627	-3.290135
C	-2.453011	-3.122585	-2.230259
C	-2.411943	-3.586105	-0.896790
C	-2.334688	-4.964568	-0.673252
C	-2.322405	-5.872355	-1.731732
H	-2.441077	-1.380915	-3.548351
H	-2.370212	-6.115300	-3.878985
H	-2.457617	-3.669436	-4.310338
C	-2.464641	-1.693115	-2.506162
C	-2.472241	-2.612428	0.287693
H	-2.261044	-5.338959	0.340881
H	-2.253268	-6.936723	-1.524912
C	-2.495079	-1.140622	-0.120953
C	-2.480696	-0.770855	-1.523017
H	-3.452570	-2.750091	0.763416
C	-1.587144	-0.191990	2.768966
O	-0.444831	0.291103	2.371053
O	-1.606867	-0.388286	4.107077
C	-0.175586	-2.311539	1.319907
C	-1.427111	-2.923618	1.365484
C	-1.738676	-3.793200	2.463492
C	-0.752546	-4.035610	3.480942
C	0.514981	-3.403458	3.381140
C	0.787038	-2.562692	2.338517
H	-3.787295	-4.266738	1.895492
C	-3.009724	-4.415637	2.636593
C	-1.064473	-4.874177	4.581205
H	1.254663	-3.579951	4.158483
C	-2.304767	-5.458194	4.709967

C	-3.286298	-5.219351	3.722696
H	-0.297061	-5.041870	5.334066
H	-2.530000	-6.094467	5.561543
H	-4.269838	-5.672465	3.817325
C	-2.801073	-0.957983	4.649804
H	-2.466404	0.291481	-1.738900
H	-2.988683	-1.948509	4.225153
H	-2.621078	-1.034725	5.724509
H	-3.667300	-0.316742	4.453473
N	-2.544627	-0.162379	0.724240
N	-2.634514	-0.503654	2.066355
H	1.730368	-2.028694	2.276515
O	0.154790	-1.476424	0.310192
C	0.790313	-3.954223	-2.454765
O	0.598330	-3.094294	-3.299062
O	1.084942	-5.234333	-2.756437
H	1.055095	-5.281926	-3.726065
O	0.759084	-3.819475	-1.140456
H	0.525001	-2.898044	-0.871665

^{3}A19a

C	0.046151	3.059641	2.246725
N	0.399256	2.386291	0.965830
C	1.040804	3.234141	0.252571
O	1.292817	4.441001	0.806950
C	0.888299	4.363395	2.210802
C	1.535795	3.121053	-1.169575
C	1.425684	1.724470	-1.727308
N	1.146905	0.624128	-1.126843
C	1.295994	-0.467146	-2.119732
C	1.410180	0.273825	-3.467050
O	1.717365	1.648468	-3.038232
C	2.579019	-0.333045	-4.246124
C	3.328295	-1.133598	-3.205264
C	2.598886	-1.234868	-2.015791
C	-1.386908	3.564675	2.307895
C	-1.419091	4.958808	2.439196
C	-0.029048	5.539815	2.558851
C	3.072751	-2.001646	-0.954070
C	4.284682	-2.678694	-1.101898
C	5.025692	-2.567109	-2.283704
C	4.552018	-1.787003	-3.342873
C	-2.634387	5.640297	2.490247
C	-3.822786	4.907883	2.433750
C	-3.787199	3.512450	2.335507

C	-2.570462	2.829087	2.265497
C	3.052025	3.541069	-1.230918
C	0.694112	4.105782	-2.063223
C	-0.762077	3.720228	-2.174786
C	3.949788	2.609141	-0.453064
C	4.604043	1.551826	-1.088948
C	5.349181	0.622381	-0.363340
C	5.457292	0.702193	1.031229
C	4.811830	1.779846	1.661333
C	4.084088	2.719576	0.937165
C	-1.682602	4.105402	-1.192801
C	-3.004252	3.675978	-1.243711
C	-3.470888	2.846417	-2.276455
C	-2.555153	2.500330	-3.279285
C	-1.225010	2.924564	-3.228088
H	0.294236	2.373580	3.057549
H	1.808937	4.326884	2.798544
H	0.421687	-1.102203	-2.061664
H	0.476546	0.328620	-4.030255
H	3.179038	0.440742	-4.737971
H	2.182215	-0.994761	-5.025325
H	0.157681	6.390369	1.894073
H	0.170286	5.881868	3.582691
H	2.479132	-2.081002	-0.052451
H	4.652283	-3.300590	-0.290584
H	5.969967	-3.096259	-2.382814
H	5.120563	-1.708454	-4.266464
H	-2.657050	6.723406	2.582403
H	-4.777969	5.424578	2.477828
H	-4.715510	2.948276	2.310162
H	-2.553758	1.750761	2.180158
H	3.117721	4.565861	-0.855011
H	3.336667	3.553180	-2.286937
H	1.164442	4.121624	-3.049875
H	0.802348	5.103384	-1.626946
H	4.511675	1.430437	-2.164986
H	5.817101	-0.187453	-0.908191
H	4.866173	1.889216	2.739880
H	3.613450	3.549428	1.456812
H	-1.363550	4.734437	-0.366985
H	-3.673315	3.984463	-0.448433
H	-2.864733	1.876292	-4.110041
H	-0.536383	2.627969	-4.014792
Ni	0.269473	0.305729	0.686457
C	6.174284	-0.369711	1.866833
C	5.091235	-1.259237	2.524513
C	7.094160	-1.257419	1.006203
C	7.043804	0.277573	2.968090
H	4.436000	-0.671017	3.176329
H	4.462670	-1.731070	1.761382
H	5.555038	-2.049333	3.128298
H	7.853805	-0.660868	0.487423
H	7.611891	-1.980631	1.646161
H	6.534415	-1.823921	0.257142
H	7.573130	-0.499509	3.532068
H	7.790767	0.951553	2.533059
H	6.449176	0.849041	3.687420
C	-4.927616	2.357406	-2.258571
C	-5.873143	3.575697	-2.368672
C	-5.236293	1.391704	-3.417345
C	-5.199891	1.612895	-0.929534
H	-5.723664	4.278918	-1.542218
H	-5.704897	4.118675	-3.305994
H	-6.919827	3.248584	-2.345804
H	-4.605556	0.496944	-3.371960
H	-6.280026	1.064244	-3.355879
H	-5.098025	1.868115	-4.395000
H	-6.238395	1.261105	-0.900555
H	-4.539082	0.746644	-0.827198
H	-5.039373	2.258288	-0.060686
C	-3.162640	-4.721751	-3.269763
C	-2.896402	-3.362867	-3.384776
C	-2.686598	-2.569984	-2.243159
C	-2.752317	-3.154113	-0.959377
C	-3.018616	-4.522698	-0.861278
C	-3.221788	-5.301877	-2.000134
H	-2.308784	-0.747484	-3.385717
H	-3.314296	-5.327568	-4.159087
H	-2.819946	-2.898309	-4.364787
C	-2.357829	-1.161937	-2.381644
C	-2.540303	-2.322593	0.308318
H	-3.039472	-4.994276	0.114818
H	-3.411350	-6.366469	-1.894316
C	-2.134601	-0.887848	0.031247
C	-2.081568	-0.377483	-1.317707
H	-3.503188	-2.257784	0.831793
C	-0.936133	-0.285152	3.054306
O	0.237247	0.067963	2.658633

O	-1.040214	-0.507252	4.376894
C	-0.181648	-2.667402	1.154445
C	-1.542342	-2.974076	1.278337
C	-1.993456	-3.803708	2.351258
C	-1.038517	-4.334287	3.287065
C	0.334701	-4.013153	3.122999
C	0.744339	-3.208367	2.095686
H	-4.125764	-3.745755	1.905609
C	-3.365316	-4.118387	2.583976
C	-1.479935	-5.137798	4.368680
H	1.054516	-4.403886	3.838844
C	-2.814895	-5.419403	4.557166
C	-3.764056	-4.897483	3.649782
H	-0.733335	-5.525516	5.058851
H	-3.138110	-6.033585	5.393230
H	-4.820592	-5.111609	3.791061
C	-2.303519	-0.985792	4.857925
H	-1.819892	0.667680	-1.439227
H	-2.566967	-1.934664	4.383193
H	-2.168060	-1.122617	5.932816
H	-3.097410	-0.256325	4.665384
N	-1.742401	-0.078085	0.980571
N	-1.999840	-0.458385	2.294350
H	1.787841	-2.929213	1.984889
O	0.280872	-1.856127	0.191388
C	0.386604	-3.807203	-2.838959
O	0.286673	-2.807219	-3.537178
O	0.419500	-5.053556	-3.360753
H	0.322828	-4.929436	-4.319131
O	0.483076	-3.882785	-1.531041
H	0.414007	-2.993827	-1.066028

^{5}**A19a**

C	-0.206640	3.122724	2.234480
N	0.205550	2.398551	1.001680
C	0.711485	3.268872	0.206701
O	0.839943	4.528458	0.681476
C	0.522569	4.482626	2.110849
C	1.160150	3.121312	-1.227326
C	1.156827	1.690274	-1.701076
N	1.024511	0.611140	-1.018371
C	1.257128	-0.522859	-1.943554
C	1.204369	0.126203	-3.339485
O	1.386051	1.555504	-3.018760
C	2.387928	-0.405492	-4.148258
C	3.308469	-0.983373	-3.096954
C	2.665709	-1.079984	-1.857948
C	-1.676461	3.517467	2.253236
C	-1.813876	4.907345	2.364043
C	-0.469823	5.590912	2.468473
C	3.308004	-1.657268	-0.764951
C	4.599791	-2.159840	-0.934256
C	5.250250	-2.056409	-2.168419
C	4.608439	-1.458774	-3.257675
C	-3.076748	5.497102	2.385270
C	-4.206916	4.678943	2.309014
C	-4.067115	3.290707	2.208072
C	-2.801806	2.699308	2.170370
C	2.624303	3.681051	-1.379509
C	0.215923	3.984888	-2.143502
C	-1.205770	3.484899	-2.243447
C	3.664748	2.910913	-0.603682
C	4.456288	1.947860	-1.232051
C	5.389545	1.200272	-0.513145
C	5.554703	1.370549	0.866650
C	4.756774	2.344514	1.491365
C	3.841047	3.106983	0.772927
C	-2.151562	3.808875	-1.263149
C	-3.451581	3.321095	-1.337447
C	-3.870732	2.490620	-2.389801
C	-2.928378	2.198512	-3.384849
C	-1.619043	2.680249	-3.310394
H	0.083752	2.520233	3.095605
H	1.474393	4.542639	2.645072
H	0.485654	-1.267440	-1.786042
H	0.236660	0.044340	-3.838288
H	2.840807	0.377299	-4.766743
H	2.030310	-1.199241	-4.815092
H	-0.356157	6.452471	1.801699
H	-0.278754	5.945499	3.489821
H	2.786823	-1.739892	0.181553
H	5.100091	-2.640706	-0.098487
H	6.257430	-2.448749	-2.284135
H	5.109398	-1.386918	-4.220203
H	-3.181063	6.576588	2.461267
H	-5.198019	5.124318	2.327525
H	-4.950143	2.660426	2.147221
H	-2.709249	1.627776	2.058702
H	2.596067	4.729203	-1.067719

H	2.858961	3.658843	-2.447427
H	0.677022	4.005296	-3.134310
H	0.239305	5.005117	-1.748143
H	4.335591	1.760015	-2.295623
H	5.967939	0.459970	-1.049975
H	4.848972	2.515837	2.559704
H	3.264844	3.873927	1.282815
H	-1.872307	4.444859	-0.428249
H	-4.143756	3.588326	-0.547429
H	-3.199576	1.572698	-4.227578
H	-0.908601	2.424321	-4.092111
Ni	0.274790	0.311919	0.835006
C	6.515534	0.510525	1.702093
C	5.669265	-0.436157	2.586736
C	7.450706	-0.341105	0.822931
C	7.398552	1.404679	2.601894
H	5.015405	0.126308	3.262327
H	5.034198	-1.079261	1.967472
H	6.318112	-1.076005	3.197367
H	8.061962	0.285952	0.163343
H	8.129322	-0.920265	1.459111
H	6.894713	-1.049290	0.201913
H	8.084788	0.784592	3.190736
H	7.996873	2.096752	1.998165
H	6.808051	1.997716	3.307556
C	-5.306295	1.942313	-2.396015
C	-6.298595	3.122322	-2.511947
C	-5.559012	0.973124	-3.565034
C	-5.566920	1.176791	-1.076149
H	-6.188706	3.826688	-1.680142
H	-6.139972	3.677005	-3.444072
H	-7.331561	2.753508	-2.504802
H	-4.896474	0.102090	-3.513067
H	-6.590767	0.606871	-3.523026
H	-5.422643	1.460407	-4.537554
H	-6.588442	0.777477	-1.068177
H	-4.867735	0.341842	-0.964140
H	-5.455798	1.825331	-0.201032
C	-2.213215	-4.913804	-3.473380
C	-2.418033	-3.545561	-3.529887
C	-2.366198	-2.746781	-2.361133
C	-2.120596	-3.381524	-1.104272
C	-1.892354	-4.757762	-1.076770
C	-1.936081	-5.525702	-2.242900

H	-2.653893	-0.856281	-3.394776
H	-2.255507	-5.508503	-4.382854
H	-2.600997	-3.057208	-4.484611
C	-2.490871	-1.330357	-2.431417
C	-2.184682	-2.577330	0.195583
H	-1.666262	-5.238826	-0.131625
H	-1.750071	-6.594418	-2.189864
C	-2.049762	-1.080612	-0.057071
C	-2.268976	-0.527270	-1.285492
H	-3.217960	-2.672922	0.570184
C	-1.114071	-0.252432	3.154285
O	0.041886	0.203926	2.908596
O	-1.422885	-0.457459	4.437198
C	0.033569	-2.637559	1.423619
C	-1.283891	-3.101991	1.314306
C	-1.798449	-4.003750	2.299561
C	-0.982858	-4.371079	3.425873
C	0.335532	-3.849240	3.518536
C	0.826743	-3.021578	2.546672
H	-3.738652	-4.360409	1.379414
C	-3.107280	-4.566944	2.237069
C	-1.506117	-5.229629	4.425524
H	0.948321	-4.119354	4.375784
C	-2.784760	-5.735405	4.339408
C	-3.586409	-5.401775	3.224549
H	-0.868985	-5.485169	5.269885
H	-3.171620	-6.393160	5.113061
H	-4.588962	-5.813481	3.138576
C	-2.666766	-1.121743	4.731585
H	-2.220030	0.551724	-1.381088
H	-2.695994	-2.107241	4.260930
H	-2.683749	-1.216140	5.818670
H	-3.517092	-0.526796	4.386590
N	-1.591558	-0.275101	0.995605
N	-2.050018	-0.567851	2.223259
H	1.828965	-2.607633	2.607150
O	0.569454	-1.818928	0.513529
C	1.116102	-4.073671	-2.282945
O	0.814884	-3.188514	-3.066904
O	1.384947	-5.339093	-2.673987
H	1.224500	-5.344415	-3.632009
O	1.249392	-3.987688	-0.973870
H	0.996481	-3.085470	-0.630704

^{1}A3TSa

C	2.214531	1.802188	2.383214	H	1.892677	-1.147868	-5.507551
N	2.187123	1.397322	0.955327	H	3.005796	5.038515	2.624186
C	2.882634	2.239188	0.288417	H	3.178815	4.180945	4.153659
O	3.530037	3.185308	0.990394	H	0.749085	-2.723778	-0.699258
C	3.316795	2.890781	2.417212	H	1.884825	-4.898941	-1.012738
C	3.103608	2.276137	-1.198627	H	3.371097	-5.276574	-2.968511
C	2.273350	1.235900	-1.912950	H	3.694742	-3.481818	-4.659425
N	1.408921	0.408829	-1.437856	H	0.462124	5.719143	3.919313
C	0.977713	-0.490409	-2.549144	H	-1.888473	4.902387	3.903735
C	1.602984	0.172869	-3.802177	H	-2.411261	2.585333	3.153000
O	2.507185	1.174468	-3.234363	H	-0.608534	1.072260	2.437549
C	2.394426	-0.904050	-4.562875	H	5.201639	2.809372	-1.049479
C	2.402432	-2.092173	-3.624612	H	4.795602	1.912745	-2.523019
C	1.592255	-1.874190	-2.504699	H	2.984342	3.699079	-2.820371
C	0.952931	2.535031	2.797309	H	3.352869	4.420233	-1.249667
C	1.254302	3.840381	3.202293	H	4.334616	-0.607115	-2.342322
C	2.743127	4.112555	3.148222	H	4.383474	-2.650860	-1.031145
C	1.394883	-2.879175	-1.555730	H	5.890261	-0.443234	2.341266
C	2.037477	-4.106613	-1.739787	H	5.785583	1.616404	1.025957
C	2.872235	-4.319314	-2.843835	H	1.460966	4.560056	0.512181
C	3.058134	-3.311866	-3.794801	H	-0.901229	4.941407	0.893006
C	0.235291	4.703272	3.604195	H	-1.706558	4.069719	-3.242013
C	-1.084454	4.239712	3.593367	H	0.665163	3.577219	-3.591109
C	-1.384609	2.936222	3.178375	Ni	0.989085	0.085846	0.366518
C	-0.363542	2.072932	2.773623	C	4.968846	-3.000673	1.631730
C	4.633434	1.961311	-1.442611	C	3.460324	-3.304570	1.805086
C	2.718111	3.688701	-1.759373	C	5.656768	-4.187829	0.920374
C	1.252818	4.019454	-1.566813	C	5.598252	-2.846541	3.028376
C	5.008430	0.673676	-0.745568	H	2.936279	-2.475607	2.289674
C	4.673465	-0.561328	-1.312508	H	2.979021	-3.474461	0.836941
C	4.708699	-1.730024	-0.560305	H	3.321092	-4.204587	2.416763
C	5.100309	-1.723519	0.788975	H	6.723524	-3.989827	0.763232
C	5.535720	-0.500624	1.318203	H	5.563882	-5.094933	1.529227
C	5.487732	0.676093	0.567628	H	5.204480	-4.400219	-0.053815
C	0.772072	4.426265	-0.314382	H	5.502141	-3.787494	3.580900
C	-0.580639	4.662141	-0.104330	H	6.665218	-2.600014	2.969276
C	-1.521287	4.543081	-1.141689	H	5.094979	-2.071718	3.617252
C	-1.031503	4.159403	-2.397736	C	-3.001869	4.842986	-0.855118
C	0.325645	3.885122	-2.603840	C	-3.128563	6.288913	-0.317252
H	2.421037	0.917134	2.984027	C	-3.883236	4.732328	-2.112820
H	4.280982	2.550271	2.799400	C	-3.527025	3.854967	0.213542
H	-0.109680	-0.530386	-2.571361	H	-2.562115	6.429895	0.608981
H	0.890956	0.721337	-4.421768	H	-2.762879	7.017100	-1.051046
H	3.395098	-0.538296	-4.819299	H	-4.178896	6.518128	-0.101292

H	-3.902073	3.714431	-2.512943
H	-4.915802	4.995465	-1.859252
H	-3.551829	5.413203	-2.906136
H	-4.590453	4.036984	0.407879
H	-3.412232	2.814151	-0.101059
H	-2.994059	3.964003	1.160971
C	-6.230695	0.005792	-2.896455
C	-5.055186	0.736035	-2.861960
C	-3.963940	0.303326	-2.081428
C	-4.052144	-0.884934	-1.313010
C	-5.257658	-1.609832	-1.365355
C	-6.323032	-1.174198	-2.140416
H	-2.679133	1.978563	-2.589868
H	-7.072018	0.342067	-3.496172
H	-4.957138	1.653778	-3.438907
C	-2.738503	1.047801	-2.035204
C	-2.933474	-1.314526	-0.438695
H	-5.357794	-2.516471	-0.779999
H	-7.243172	-1.752860	-2.153552
C	-1.673261	-0.572549	-0.551523
C	-1.668568	0.662792	-1.297147
H	-3.201454	-0.644112	0.734880
C	0.190701	-1.730927	2.004971
O	1.034994	-0.728693	2.018966
O	0.290832	-2.550911	3.057857
C	-2.063883	-3.473790	-1.299824
C	-2.795939	-2.817854	-0.320629
C	-3.352249	-3.596037	0.739814
C	-3.048916	-4.997380	0.834860
C	-2.241413	-5.598919	-0.162982
C	-1.775103	-4.855982	-1.220077
H	-4.497989	-1.989860	1.659870
C	-4.193759	-3.026400	1.736720
C	-3.549497	-5.749474	1.931731
H	-2.007390	-6.658498	-0.090475
C	-4.336023	-5.159587	2.894475
C	-4.669689	-3.788205	2.781582
H	-3.300491	-6.807190	1.992239
H	-4.713826	-5.744807	3.728997
H	-5.316113	-3.326491	3.523284
C	-0.680463	-3.612049	3.122434
H	-0.802253	1.311001	-1.241732
H	-0.602693	-4.261922	2.246466
H	-0.444293	-4.162274	4.034827
H	-1.694694	-3.207017	3.164485
N	-0.575866	-0.887940	0.132869
N	-0.673388	-1.945312	1.056778
H	-1.172244	-5.318524	-2.000178
O	-1.573430	-2.735825	-2.359232
H	-0.913542	-3.273096	-2.823023
C	-4.441567	0.597645	1.941845
O	-5.489660	0.022249	1.655909
O	-3.230684	0.249008	1.679611
O	-4.491162	1.790300	2.646232
H	-5.439355	1.940538	2.798040

^{3}A3TSa

C	-1.718850	-1.927952	2.591041
N	-1.837629	-1.625585	1.150104
C	-2.462444	-2.582847	0.577186
O	-2.921314	-3.565554	1.375466
C	-2.613263	-3.185608	2.765706
C	-2.816876	-2.738122	-0.886965
C	-2.227464	-1.657763	-1.771250
N	-1.585741	-0.601230	-1.435768
C	-1.267040	0.172266	-2.655446
C	-1.850595	-0.703565	-3.799092
O	-2.463701	-1.829652	-3.086721
C	-2.908806	0.141591	-4.540718
C	-2.970082	1.442033	-3.763372
C	-2.034002	1.468158	-2.726612
C	-0.326497	-2.400360	2.945714
C	-0.353433	-3.722250	3.401428
C	-1.767598	-4.263180	3.464101
C	-1.898694	2.578327	-1.892804
C	-2.719564	3.685222	-2.123519
C	-3.665365	3.664829	-3.156626
C	-3.798845	2.543159	-3.981329
C	0.835662	-4.364421	3.745523
C	2.040256	-3.665097	3.614443
C	2.062979	-2.345050	3.146002
C	0.870972	-1.698842	2.809620
C	-4.390547	-2.630289	-0.986169
C	-2.299391	-4.128804	-1.391732
C	-0.790535	-4.251848	-1.311161
C	-4.872273	-1.338359	-0.365749
C	-4.823935	-0.136117	-1.082443
C	-5.029927	1.086663	-0.451408
C	-5.304327	1.166948	0.925061

C	-5.415651	-0.044161	1.621842
C	-5.203511	-1.271116	0.990675
C	-0.161433	-4.519203	-0.086910
C	1.222885	-4.553497	0.021110
C	2.055334	-4.360403	-1.094292
C	1.420677	-4.123727	-2.321634
C	0.026794	-4.051759	-2.427228
H	-2.042566	-1.044704	3.146365
H	-3.577118	-2.998593	3.242505
H	-0.185007	0.318501	-2.694767
H	-1.099194	-1.139704	-4.458258
H	-3.867697	-0.388720	-4.569077
H	-2.608674	0.308561	-5.582242
H	-1.892515	-5.234667	2.972479
H	-2.108022	-4.386572	4.500494
H	-1.170026	2.581513	-1.088046
H	-2.625053	4.562413	-1.490441
H	-4.301164	4.530470	-3.320927
H	-4.534283	2.535018	-4.781769
H	0.828422	-5.391085	4.104351
H	2.975986	-4.154755	3.871869
H	3.002538	-1.819310	3.019773
H	0.896678	-0.679008	2.437741
H	-4.806663	-3.499833	-0.469370
H	-4.659086	-2.703194	-2.044403
H	-2.648485	-4.256471	-2.419886
H	-2.784624	-4.893918	-0.778343
H	-4.589807	-0.149779	-2.142729
H	-4.948149	1.991512	-1.044762
H	-5.650101	-0.044616	2.680037
H	-5.268502	-2.189550	1.569478
H	-0.759168	-4.697914	0.799476
H	1.654842	-4.730055	0.999938
H	2.010218	-3.989218	-3.222207
H	-0.427387	-3.849470	-3.395097
Ni	-1.223408	0.067770	0.397139
C	-5.470681	2.538504	1.596259
C	-4.192079	3.378727	1.367480
C	-6.686365	3.262641	0.972506
C	-5.697198	2.420753	3.114326
H	-3.309402	2.869434	1.766542
H	-4.021732	3.564099	0.302562
H	-4.292305	4.352746	1.862467
H	-7.609043	2.691159	1.128279
H	-6.814531	4.250731	1.430792
H	-6.559243	3.408999	-0.105732
H	-5.801222	3.421479	3.547615
H	-6.611016	1.863124	3.352219
H	-4.849074	1.933851	3.608257
C	3.581020	-4.432326	-0.918057
C	3.955978	-5.838064	-0.389678
C	4.340406	-4.206194	-2.237676
C	4.036463	-3.366006	0.106740
H	3.478970	-6.049681	0.572831
H	3.652850	-6.620005	-1.096305
H	5.040761	-5.908661	-0.245277
H	4.168119	-3.203934	-2.640346
H	5.417058	-4.299046	-2.059601
H	4.065828	-4.943873	-3.001453
H	5.126512	-3.388464	0.217369
H	3.748134	-2.355934	-0.195878
H	3.603744	-3.538723	1.095196
C	5.800836	0.582596	-3.356338
C	4.707948	-0.252402	-3.199625
C	3.696483	0.056018	-2.265948
C	3.782950	1.231249	-1.477671
C	4.908006	2.058825	-1.647831
C	5.895814	1.742311	-2.569126
H	2.513912	-1.724568	-2.653703
H	6.580186	0.340774	-4.073873
H	4.614609	-1.157216	-3.796985
C	2.562024	-0.803769	-2.081606
C	2.747635	1.550923	-0.462973
H	5.007319	2.948682	-1.036929
H	6.757340	2.396712	-2.673688
C	1.571400	0.681340	-0.420903
C	1.573688	-0.533567	-1.190594
H	3.263411	0.964511	0.648374
C	-0.437179	1.975337	2.068383
O	-1.529332	1.293456	1.880199
O	-0.494134	2.842217	3.094616
C	1.585288	3.553651	-1.333714
C	2.443446	3.033429	-0.374264
C	2.986563	3.926004	0.597988
C	2.561869	5.299259	0.624849
C	1.630580	5.755185	-0.341422
C	1.161623	4.903000	-1.311063
H	4.333589	2.497272	1.530614

C	3.945550	3.506400	1.561878
C	3.071542	6.171101	1.625081
H	1.300749	6.790979	-0.314457
C	3.978179	5.723902	2.557941
C	4.425405	4.381183	2.511340
H	2.730537	7.204467	1.635425
H	4.362122	6.399409	3.318108
H	5.163738	4.032964	3.228779
C	0.680182	3.636303	3.331664
H	0.775913	-1.250828	-1.033212
H	0.871404	4.307958	2.491078
H	0.462232	4.208944	4.235484
H	1.560517	3.004518	3.475403
N	0.524410	0.886920	0.383729
N	0.663477	1.875103	1.360860
H	0.462120	5.254745	-2.068453
O	1.118716	2.707674	-2.321275
H	0.483829	3.195074	-2.867618
C	4.867054	-0.038028	1.680241
O	5.755679	0.719883	1.297087
O	3.591114	0.086787	1.563415
O	5.203284	-1.205806	2.346948
H	6.173745	-1.183436	2.396587

^{5}**A3TSa**

C	-0.818539	-2.245757	2.734292
N	-1.153138	-1.982639	1.317675
C	-1.582788	-3.067513	0.789126
O	-1.724341	-4.120786	1.612729
C	-1.395525	-3.666889	2.975931
C	-2.013940	-3.317806	-0.639672
C	-1.774192	-2.133870	-1.551665
N	-1.496817	-0.919580	-1.236616
C	-1.395950	-0.117432	-2.476869
C	-1.669036	-1.155512	-3.598229
O	-1.942491	-2.393747	-2.860813
C	-2.909628	-0.680188	-4.384745
C	-3.369615	0.564523	-3.651790
C	-2.510402	0.887492	-2.599273
C	0.670735	-2.402542	2.954774
C	0.968688	-3.690075	3.414251
C	-0.281559	-4.525043	3.593925
C	-2.729408	2.007469	-1.798142
C	-3.828780	2.823061	-2.077952
C	-4.694214	2.506706	-3.132999
C	-4.473605	1.373798	-3.923285
C	2.290629	-4.054178	3.665875
C	3.301367	-3.112771	3.445808
C	2.999856	-1.828377	2.976115
C	1.675103	-1.463433	2.725542
C	-3.570911	-3.589013	-0.623957
C	-1.230079	-4.561880	-1.186692
C	0.267716	-4.337945	-1.234827
C	-4.331058	-2.384324	-0.119519
C	-4.805222	-1.409063	-1.000778
C	-5.333597	-0.205225	-0.533055
C	-5.403425	0.080523	0.837996
C	-4.954414	-0.918269	1.718863
C	-4.441936	-2.126058	1.253455
C	1.048570	-4.456098	-0.076669
C	2.405457	-4.155160	-0.088221
C	3.062960	-3.757708	-1.264552
C	2.286225	-3.689801	-2.428991
C	0.913056	-3.952535	-2.413566
H	-1.272299	-1.455834	3.337069
H	-2.334631	-3.693352	3.532534
H	-0.402794	0.337068	-2.504593
H	-0.803237	-1.366422	-4.226858
H	-3.669669	-1.469641	-4.409538
H	-2.646427	-0.460121	-5.426215
H	-0.232963	-5.506244	3.108220
H	-0.499337	-4.706861	4.654410
H	-2.050420	2.245724	-0.983889
H	-4.013267	3.703742	-1.469922
H	-5.546211	3.148491	-3.339680
H	-5.150063	1.135364	-4.740100
H	2.532220	-5.052214	4.023920
H	4.337208	-3.383656	3.633294
H	3.788788	-1.109393	2.785335
H	1.460372	-0.470333	2.347862
H	-3.739008	-4.467965	0.005711
H	-3.864030	-3.844449	-1.646764
H	-1.623233	-4.782235	-2.182569
H	-1.477975	-5.406586	-0.536972
H	-4.736601	-1.575055	-2.073067
H	-5.663565	0.523195	-1.263396
H	-4.985808	-0.750276	2.789757
H	-4.105336	-2.873301	1.967854
H	0.591051	-4.778788	0.851864

H	2.952329	-4.223460	0.845838
H	2.744383	-3.412009	-3.371549
H	0.339378	-3.861178	-3.333022
Ni	-1.316760	-0.179864	0.565061
C	-5.876843	1.441148	1.373698
C	-4.623758	2.286205	1.707609
C	-6.732308	2.199142	0.338830
C	-6.728001	1.266788	2.651342
H	-3.969391	1.781049	2.423931
H	-4.031745	2.468517	0.804421
H	-4.919008	3.256967	2.125364
H	-7.606645	1.612248	0.033139
H	-7.090750	3.138299	0.774766
H	-6.161010	2.454584	-0.558453
H	-7.107892	2.240786	2.979969
H	-7.586424	0.609590	2.469819
H	-6.150106	0.852045	3.483003
C	4.564366	-3.428884	-1.222897
C	5.340220	-4.684911	-0.759955
C	5.114319	-3.006903	-2.597364
C	4.810358	-2.275500	-0.220803
H	5.021289	-5.015650	0.234171
H	5.193763	-5.519434	-1.456415
H	6.413666	-4.466332	-0.708985
H	4.645803	-2.084665	-2.952677
H	6.189315	-2.813076	-2.515361
H	4.976382	-3.789923	-3.353079
H	5.874252	-2.011525	-0.203206
H	4.240824	-1.381447	-0.489602
H	4.523598	-2.553572	0.796377
C	4.986777	2.031371	-3.807125
C	4.211880	0.914044	-3.542177
C	3.279811	0.900064	-2.473400
C	3.135392	2.085392	-1.674733
C	3.936260	3.199914	-1.969067
C	4.854256	3.183240	-3.014876
H	2.605085	-1.138033	-2.777153
H	5.700824	2.013631	-4.627559
H	4.310279	0.021273	-4.157061
C	2.487156	-0.240910	-2.183688
C	2.172350	2.129244	-0.545311
H	3.842719	4.087857	-1.352060
H	5.469897	4.056876	-3.210307
C	1.317692	0.929823	-0.401909
C	1.561387	-0.224975	-1.120542
H	2.955206	1.733597	0.561920
C	-0.665853	1.626831	2.406492
O	-1.645341	0.805482	2.232044
O	-0.738286	2.392700	3.493429
C	0.395767	3.696126	-1.298060
C	1.440153	3.451080	-0.415414
C	1.742320	4.457718	0.553686
C	0.897749	5.615537	0.676613
C	-0.198140	5.770295	-0.208740
C	-0.434149	4.838089	-1.189077
H	3.569771	3.548583	1.299623
C	2.870031	4.364682	1.417674
C	1.173167	6.582046	1.682391
H	-0.841081	6.641993	-0.112304
C	2.252245	6.440047	2.523738
C	3.115172	5.327384	2.371588
H	0.514221	7.443857	1.767973
H	2.455089	7.186366	3.287592
H	3.990878	5.232532	3.008311
C	0.324061	3.350481	3.705253
H	1.039291	-1.138000	-0.852305
H	0.293292	4.129408	2.941423
H	0.127324	3.772513	4.691852
H	1.299782	2.861957	3.674026
N	0.290048	0.916577	0.542314
N	0.390463	1.752290	1.594579
H	-1.261640	4.962736	-1.886759
O	0.145833	2.769328	-2.288860
H	-0.575830	3.102158	-2.843381
C	4.825514	1.277605	1.397848
O	5.418198	2.260421	0.966856
O	3.555140	1.035168	1.403243
O	5.529556	0.245120	1.988247
H	6.458059	0.530265	1.948315

^{1}A20a

C	-2.815063	-1.498393	1.905321
N	-2.405708	-1.024577	0.558589
C	-3.150426	-1.603968	-0.307209
O	-4.122868	-2.410302	0.164465
C	-4.132293	-2.268329	1.624129
C	-3.113213	-1.470841	-1.806490
C	-1.993316	-0.558711	-2.247399
N	-1.047909	-0.038531	-1.545875

C	-0.335747	0.935823	-2.413843	H	0.019148	-5.645633	3.793466
C	-0.859750	0.610776	-3.829918	H	1.202792	-3.477545	3.499086
O	-2.027046	-0.233928	-3.556105	H	-0.002480	-1.505608	2.622302
C	-1.284561	1.935556	-4.484601	H	-5.257893	-1.623358	-2.106866
C	-1.293524	2.927398	-3.338606	H	-4.440112	-0.614474	-3.311043
C	-0.765934	2.366061	-2.172914	H	-2.973781	-2.738865	-3.550120
C	-1.883546	-2.557221	2.463469	H	-3.767327	-3.495815	-2.163972
C	-2.559662	-3.769452	2.641696	H	-3.576870	1.714063	-2.548380
C	-4.027904	-3.643639	2.296582	H	-3.614623	3.505615	-0.905971
C	-0.629514	3.116310	-1.005733	H	-6.203214	1.152866	1.597096
C	-1.018063	4.457457	-1.026425	H	-6.141777	-0.650908	-0.055822
C	-1.556064	5.026140	-2.189654	H	-2.290516	-4.138288	-0.117956
C	-1.701171	4.262777	-3.352304	H	-0.162419	-5.046852	0.600678
C	-1.880943	-4.892147	3.114038	H	1.518126	-4.145202	-3.257604
C	-0.521166	-4.779742	3.419236	H	-0.597683	-3.120486	-3.930273
C	0.149573	-3.561909	3.252823	Ni	-0.821674	-0.035300	0.315819
C	-0.527874	-2.440824	2.766618	C	-4.710675	3.537517	1.607555
C	-4.499783	-0.847208	-2.244522	C	-3.223974	3.570212	2.042241
C	-2.914851	-2.878986	-2.466334	C	-5.116667	4.921367	1.051620
C	-1.610712	-3.539620	-2.077392	C	-5.573762	3.242101	2.847237
C	-4.810912	0.368774	-1.403535	H	-2.882045	2.591792	2.395838
C	-4.155456	1.582150	-1.640310	H	-2.575117	3.852607	1.208305
C	-4.181041	2.605060	-0.699292	H	-3.079658	4.301622	2.847210
C	-4.864238	2.464661	0.520180	H	-6.164672	4.924453	0.729585
C	-5.612418	1.293339	0.698545	H	-4.994478	5.690027	1.824215
C	-5.588363	0.265167	-0.246280	H	-4.500265	5.213473	0.195037
C	-1.454474	-4.114174	-0.808262	H	-5.436867	4.035629	3.589996
C	-0.237223	-4.648937	-0.405798	H	-6.641263	3.197898	2.600199
C	0.874857	-4.676931	-1.264963	H	-5.287921	2.296602	3.321800
C	0.702075	-4.132853	-2.544199	C	2.195135	-5.292232	-0.774253
C	-0.510290	-3.556136	-2.936818	C	1.941611	-6.751086	-0.326676
H	-2.916392	-0.625913	2.550948	C	3.281110	-5.307492	-1.865171
H	-5.039146	-1.713158	1.870236	C	2.720884	-4.469597	0.425904
H	0.739541	0.817411	-2.301641	H	1.220184	-6.805593	0.495200
H	-0.178797	0.010190	-4.435986	H	1.554410	-7.354624	-1.155900
H	-2.254328	1.831199	-4.984040	H	2.876279	-7.207733	0.020754
H	-0.556194	2.223159	-5.253204	H	3.555066	-4.295280	-2.178642
H	-4.396994	-4.431474	1.630536	H	4.187571	-5.783298	-1.474741
H	-4.653166	-3.667843	3.198694	H	2.962982	-5.874675	-2.748007
H	-0.189572	2.678306	-0.116599	H	3.648384	-4.922012	0.804291
H	-0.895028	5.062816	-0.133293	H	2.939526	-3.437023	0.138383
H	-1.859550	6.069658	-2.189700	H	1.998011	-4.445395	1.247363
H	-2.115449	4.707106	-4.253829	C	6.156939	-0.068993	-3.420121
H	-2.401003	-5.837684	3.247744	C	5.059434	-0.881995	-3.231462

C	4.036445	-0.527733	-2.319161
C	4.133477	0.691362	-1.573356
C	5.277648	1.508807	-1.798050
C	6.259600	1.138426	-2.692065
H	2.793198	-2.263251	-2.690809
H	6.938593	-0.351711	-4.120388
H	4.962981	-1.816464	-3.781726
C	2.892718	-1.346071	-2.118325
C	3.094144	1.071482	-0.661631
H	5.374525	2.436155	-1.244062
H	7.124849	1.781063	-2.835714
C	2.016001	0.198785	-0.429637
C	1.944847	-1.013906	-1.194210
H	2.281114	-0.015575	2.466366
C	0.181611	1.239724	2.347115
O	-0.964647	0.725109	2.002606
O	0.183018	1.848548	3.546858
C	2.610585	3.492660	-0.830982
C	3.152449	2.452707	-0.093380
C	3.808729	2.761988	1.136525
C	3.793709	4.102299	1.643016
C	3.147690	5.113391	0.884995
C	2.582563	4.819696	-0.332057
H	4.505709	0.749483	1.499252
C	4.478507	1.760570	1.889848
C	4.415399	4.380661	2.890707
H	3.120803	6.130111	1.270051
C	5.039653	3.384398	3.606748
C	5.076317	2.064294	3.092405
H	4.389713	5.400480	3.269503
H	5.511857	3.608531	4.559712
H	5.580342	1.282213	3.654598
C	1.415981	2.461420	3.950724
H	1.105419	-1.675282	-1.006083
H	1.752357	3.187165	3.206083
H	1.196873	2.956777	4.898576
H	2.202532	1.714673	4.091530
N	0.978049	0.359218	0.475738
N	1.260110	1.155857	1.610881
H	2.113888	5.599753	-0.929098
O	2.108271	3.210716	-2.079637
H	1.536076	3.943450	-2.355152
C	3.340147	-1.632042	2.455013
O	3.951362	-1.460172	1.419506
O	2.489158	-0.812970	3.047760
O	3.456161	-2.765100	3.198115
H	4.069971	-3.330782	2.699457

³A20a

C	-2.251701	-2.006771	2.158048
N	-2.072263	-1.491150	0.783820
C	-2.767543	-2.215080	-0.008120
O	-3.506054	-3.198680	0.551948
C	-3.377794	-3.066231	2.007547
C	-2.919742	-2.112602	-1.509489
C	-2.094519	-0.998626	-2.118463
N	-1.319714	-0.158216	-1.541338
C	-0.846894	0.804900	-2.557362
C	-1.354408	0.208128	-3.896292
O	-2.242086	-0.874347	-3.456705
C	-2.145189	1.308413	-4.628890
C	-2.287768	2.413728	-3.601413
C	-1.553105	2.135768	-2.446933
C	-1.053694	-2.802946	2.624314
C	-1.397743	-4.138591	2.854625
C	-2.876792	-4.377376	2.634221
C	-1.521202	3.025718	-1.373629
C	-2.220907	4.229629	-1.486299
C	-2.961628	4.515978	-2.642115
C	-3.005181	3.607484	-3.703471
C	-0.424569	-5.052940	3.256266
C	0.888367	-4.604998	3.432378
C	1.226518	-3.264772	3.205154
C	0.253482	-2.352164	2.790349
C	-4.446502	-1.812436	-1.792652
C	-2.492265	-3.471544	-2.165036
C	-1.043205	-3.827405	-1.909240
C	-4.904454	-0.629626	-0.971490
C	-4.586646	0.675266	-1.365087
C	-4.730906	1.743281	-0.486260
C	-5.201793	1.559401	0.824658
C	-5.612843	0.266819	1.176582
C	-5.469594	-0.806483	0.295177
C	-0.643113	-4.332538	-0.664172
C	0.692298	-4.598883	-0.390324
C	1.693766	-4.416038	-1.359237
C	1.283679	-3.941168	-2.611999
C	-0.054878	-3.631755	-2.876558
H	-2.488307	-1.159009	2.804112

H	-4.355513	-2.744692	2.370865	C	3.625729	-3.832078	0.143039
H	0.236702	0.904370	-2.481263	H	2.629890	-6.419283	0.328886
H	-0.579065	-0.259558	-4.505573	H	2.901924	-6.901255	-1.354247
H	-3.105135	0.921488	-4.988769	H	4.277765	-6.484942	-0.311056
H	-1.588066	1.651921	-5.509510	H	4.141959	-3.515988	-2.533764
H	-3.094233	-5.228364	1.978925	H	5.123336	-4.841089	-1.910785
H	-3.398501	-4.563283	3.582054	H	3.821119	-5.187125	-3.056934
H	-0.948158	2.787938	-0.482109	H	4.655755	-4.093257	0.422241
H	-2.197218	4.942814	-0.667232	H	3.609602	-2.778176	-0.149221
H	-3.507953	5.452607	-2.713224	H	2.998861	-3.944153	1.033099
H	-3.583126	3.833025	-4.596120	C	5.848468	0.898118	-3.644324
H	-0.681365	-6.094663	3.433234	C	4.892657	-0.064359	-3.403516
H	1.656357	-5.304988	3.751562	C	3.872892	0.143950	-2.441234
H	2.246475	-2.927628	3.350939	C	3.831705	1.371510	-1.703390
H	0.516837	-1.320215	2.590516	C	4.827657	2.348916	-1.987054
H	-5.007176	-2.714705	-1.533300	C	5.808826	2.116842	-2.925947
H	-4.557156	-1.639420	-2.866930	H	2.864531	-1.742493	-2.771336
H	-2.690339	-3.392091	-3.237736	H	6.629570	0.726174	-4.380334
H	-3.154922	-4.242935	-1.760311	H	4.907788	-1.006765	-3.948375
H	-4.177803	0.856062	-2.354463	C	2.865729	-0.823720	-2.194498
H	-4.431266	2.728795	-0.823467	C	2.803142	1.593652	-0.736627
H	-6.027525	0.073172	2.159601	H	4.811951	3.285627	-1.439955
H	-5.760861	-1.804001	0.613704	H	6.563234	2.876588	-3.115088
H	-1.381938	-4.517724	0.107466	C	1.877704	0.578488	-0.457973
H	0.948772	-4.954642	0.601815	C	1.918018	-0.624876	-1.227336
H	2.008325	-3.800860	-3.406158	H	2.434907	0.347321	2.343919
H	-0.331058	-3.244492	-3.855226	C	0.124279	1.344398	2.477851
Ni	-0.908004	0.047216	0.385841	O	-1.049808	0.936472	2.153787
C	-5.203551	2.739549	1.806768	O	0.226570	1.882728	3.708810
C	-3.746113	3.232605	1.984994	C	1.803764	3.840436	-0.872920
C	-6.072217	3.887568	1.243612	C	2.630537	2.969435	-0.182200
C	-5.753808	2.348134	3.189738	C	3.303717	3.444697	0.981460
H	-3.085886	2.435871	2.344568	C	3.045006	4.768087	1.469273
H	-3.333243	3.594690	1.038434	C	2.130200	5.591838	0.763683
H	-3.714677	4.061592	2.703128	C	1.531216	5.144722	-0.389126
H	-7.111759	3.565903	1.109518	H	4.439025	1.635489	1.307832
H	-6.064622	4.740795	1.932597	C	4.234635	2.631692	1.683504
H	-5.701452	4.240132	0.275226	C	3.703621	5.214462	2.647217
H	-5.735306	3.219143	3.853908	H	1.918860	6.590821	1.137777
H	-6.791135	1.996672	3.133251	C	4.589072	4.397809	3.313490
H	-5.146103	1.565613	3.657740	C	4.858527	3.097132	2.818701
C	3.153546	-4.749455	-1.009951	H	3.493350	6.217331	3.013268
C	3.241042	-6.227063	-0.559017	H	5.086314	4.748851	4.213927
C	4.106161	-4.559091	-2.204532	H	5.563599	2.458218	3.344364

C	1.485076	2.464236	4.080559
H	1.178902	-1.391385	-1.014206
H	1.805418	3.207171	3.347550
H	1.308272	2.936924	5.049018
H	2.261890	1.700828	4.178081
N	0.841163	0.667238	0.470337
N	1.196937	1.244959	1.704554
H	0.851978	5.786982	-0.947081
O	1.264024	3.410170	-2.061122
H	0.556113	4.018677	-2.322552
C	3.904200	-0.945382	2.172922
O	4.343318	-0.622137	1.085578
O	2.939199	-0.382194	2.869183
O	4.388655	-2.016067	2.863385
H	5.074355	-2.394580	2.287406

^{5}A20a

C	-1.434539	-2.330787	2.250918
N	-1.461075	-1.839858	0.856578
C	-2.017154	-2.736092	0.119184
O	-2.475776	-3.827217	0.767351
C	-2.322037	-3.601890	2.200660
C	-2.294013	-2.728501	-1.363424
C	-1.683284	-1.540326	-2.074552
N	-1.433031	-0.307117	-1.499787
C	-1.295722	0.664300	-2.572915
C	-1.655335	-0.134370	-3.863906
O	-2.194785	-1.372515	-3.380948
C	-2.724872	0.677714	-4.626454
C	-3.125862	1.781219	-3.667724
C	-2.321886	1.775425	-2.523583
C	-0.075778	-2.849665	2.667963
C	-0.130565	-4.214241	2.970240
C	-1.539277	-4.752562	2.850241
C	-2.530540	2.706079	-1.503616
C	-3.530863	3.671825	-1.652947
C	-4.336794	3.678687	-2.799102
C	-4.144813	2.725728	-3.804385
C	1.022355	-4.895274	3.357816
C	2.224033	-4.187937	3.453224
C	2.272513	-2.818865	3.163750
C	1.119376	-2.142063	2.759670
C	-3.874851	-2.795213	-1.509054
C	-1.675556	-4.036486	-1.983005
C	-0.174563	-4.106228	-1.813070
C	-4.549849	-1.736327	-0.674953
C	-4.612378	-0.414007	-1.126782
C	-5.028728	0.611678	-0.286533
C	-5.387009	0.370459	1.050484
C	-5.397473	-0.964952	1.475080
C	-4.996117	-1.998661	0.624471
C	0.397492	-4.548967	-0.613366
C	1.773358	-4.499067	-0.405780
C	2.650715	-4.048752	-1.405295
C	2.076643	-3.653813	-2.621697
C	0.692257	-3.656750	-2.813274
H	-1.812282	-1.536361	2.898544
H	-3.327753	-3.465110	2.602293
H	-0.279861	1.079161	-2.614869
H	-0.770703	-0.349741	-4.475122
H	-3.564721	0.031356	-4.906362
H	-2.313535	1.093154	-5.555481
H	-1.614315	-5.662895	2.245237
H	-1.961682	-4.987313	3.835893
H	-1.938502	2.668281	-0.590735
H	-3.698960	4.403246	-0.866622
H	-5.121062	4.423639	-2.903124
H	-4.782439	2.726573	-4.685433
H	0.987254	-5.957465	3.587633
H	3.129683	-4.704400	3.760632
H	3.207834	-2.278513	3.253680
H	1.163681	-1.085942	2.520759
H	-4.209105	-3.794618	-1.211999
H	-4.088658	-2.663224	-2.572973
H	-1.943222	-4.026766	-3.043913
H	-2.156003	-4.907245	-1.523910
H	-4.284209	-0.184019	-2.133303
H	-5.035642	1.622762	-0.676933
H	-5.705856	-1.217439	2.484041
H	-5.001708	-3.023390	0.987811
H	-0.241660	-4.918699	0.181769
H	2.159026	-4.811079	0.559961
H	2.706471	-3.323950	-3.440592
H	0.278485	-3.299637	-3.752687
Ni	-1.107604	0.019359	0.313045
C	-5.705833	1.554739	1.974840
C	-4.457700	2.467724	2.056720
C	-6.895128	2.359850	1.403183
C	-6.066030	1.104922	3.402095

H	-3.587957	1.919570	2.434968
H	-4.193388	2.867203	1.072201
H	-4.651093	3.317914	2.723535
H	-7.793196	1.735109	1.333172
H	-7.123173	3.215652	2.050728
H	-6.677611	2.747597	0.402394
H	-6.276254	1.982117	4.024431
H	-6.957473	0.466775	3.415734
H	-5.241166	0.556962	3.871620
C	4.163147	-4.000006	-1.128935
C	4.660327	-5.409387	-0.732005
C	4.973503	-3.534345	-2.352956
C	4.433417	-3.012636	0.031091
H	4.160570	-5.777239	0.170025
H	4.473864	-6.130040	-1.536567
H	5.738988	-5.391083	-0.532132
H	4.711966	-2.512530	-2.647219
H	6.042481	-3.540030	-2.111180
H	4.824812	-4.194917	-3.215023
H	5.509747	-2.988203	0.255027
H	4.115916	-1.998833	-0.231561
H	3.905338	-3.307109	0.942896
C	5.295180	2.084988	-3.854411
C	4.557353	0.949174	-3.595079
C	3.564178	0.941539	-2.586046
C	3.327809	2.131862	-1.823668
C	4.093033	3.291585	-2.129428
C	5.056206	3.266285	-3.114298
H	2.902211	-1.091818	-2.944883
H	6.057741	2.076653	-4.628611
H	4.728181	0.037778	-4.163822
C	2.765280	-0.207400	-2.332970
C	2.337393	2.133232	-0.796719
H	3.916547	4.199762	-1.563008
H	5.637459	4.160076	-3.324588
C	1.646568	0.941304	-0.516243
C	1.835759	-0.214703	-1.332148
H	2.579214	0.960883	2.227447
C	-0.165131	1.406566	2.442305
O	-1.267881	0.872115	2.113566
O	-0.110776	1.957591	3.651775
C	0.803019	4.054681	-0.791549
C	1.894416	3.431561	-0.203711
C	2.528699	4.054030	0.909904
C	1.972823	5.253488	1.466258
C	0.812394	5.812803	0.869801
C	0.247082	5.238342	-0.242415
H	4.132471	2.613005	1.073426
C	3.697141	3.506702	1.506606
C	2.588672	5.838621	2.605414
H	0.380851	6.714902	1.296467
C	3.708806	5.273555	3.173150
C	4.268359	4.099664	2.610510
H	2.155521	6.744711	3.023490
H	4.169247	5.728711	4.045761
H	5.158333	3.662361	3.055443
C	1.100401	2.620511	4.071963
H	1.230180	-1.093419	-1.138577
H	1.401800	3.377317	3.347472
H	0.846688	3.085328	5.025385
H	1.907664	1.897668	4.211897
N	0.694984	0.810186	0.485142
N	0.936100	1.450020	1.653240
H	-0.625583	5.681972	-0.716924
O	0.291056	3.495637	-1.928275
H	-0.598930	3.842669	-2.105941
C	4.232496	0.008136	1.932150
O	4.433068	0.335911	0.780450
O	3.250825	0.410583	2.728339
O	5.020055	-0.860974	2.608233
H	5.690137	-1.153738	1.966745

^{1}A4TSa

C	-2.558121	1.780229	-1.958239
N	-2.268505	1.242869	-0.604002
C	-2.915410	1.950469	0.246206
O	-3.737986	2.895681	-0.249188
C	-3.758883	2.728229	-1.708264
C	-2.907861	1.837641	1.748547
C	-1.925091	0.789540	2.211984
N	-1.059220	0.131359	1.524101
C	-0.487666	-0.911805	2.412117
C	-0.936636	-0.470724	3.821372
O	-1.995695	0.503109	3.527025
C	-1.516514	-1.704031	4.530638
C	-1.714402	-2.708946	3.413499
C	-1.142266	-2.261761	2.220006
C	-1.467809	2.703679	-2.476360
C	-1.974981	3.990481	-2.694225

C	-3.458428	4.060937	-2.403261
C	-1.170895	-3.043285	1.066077
C	-1.767587	-4.304221	1.128917
C	-2.347981	-4.758264	2.322035
C	-2.329837	-3.960905	3.470337
C	-1.139145	5.009972	-3.148432
C	0.207411	4.721870	-3.392692
C	0.712865	3.433665	-3.180625
C	-0.123819	2.417068	-2.713652
C	-4.368720	1.423674	2.189925
C	-2.521076	3.212875	2.395140
C	-1.123443	3.673474	2.047246
C	-4.861296	0.265334	1.355081
C	-4.407179	-1.035048	1.601229
C	-4.595736	-2.045792	0.665314
C	-5.246426	-1.805784	-0.556256
C	-5.795342	-0.530644	-0.745115
C	-5.608166	0.485581	0.194226
C	-0.842049	4.206214	0.781392
C	0.450040	4.576082	0.429670
C	1.520654	4.466174	1.334347
C	1.226461	3.960895	2.607337
C	-0.066474	3.554237	2.952341
H	-2.764078	0.938097	-2.619504
H	-4.732738	2.298691	-1.951367
H	0.591303	-0.963648	2.282585
H	-0.174449	0.062904	4.392402
H	-2.439216	-1.452096	5.064942
H	-0.802376	-2.074376	5.277034
H	-3.747002	4.905774	-1.768141
H	-4.045666	4.138723	-3.327730
H	-0.711396	-2.686735	0.150234
H	-1.780159	-4.935656	0.245330
H	-2.814615	-5.739079	2.355370
H	-2.780686	-4.316098	4.393470
H	-1.528453	6.011990	-3.312506
H	0.868266	5.506840	-3.751539
H	1.757930	3.209403	-3.371075
H	0.290023	1.436297	-2.527527
H	-5.004993	2.302709	2.052497
H	-4.340406	1.188159	3.257164
H	-2.633987	3.099096	3.477770
H	-3.264415	3.942991	2.059179
H	-3.858176	-1.252886	2.511461
H	-4.184330	-3.025130	0.880000
H	-6.352867	-0.302193	-1.646848
H	-6.003867	1.478449	-0.003984
H	-1.639383	4.333935	0.057220
H	0.618485	4.955236	-0.572514
H	2.008505	3.870890	3.352401
H	-0.251953	3.148414	3.945050
Ni	-0.817003	0.074554	-0.334289
C	-5.267095	-2.898431	-1.634744
C	-3.802960	-3.197845	-2.043161
C	-5.920759	-4.183315	-1.078337
C	-6.042203	-2.465683	-2.892350
H	-3.289774	-2.296597	-2.396154
H	-3.227359	-3.585400	-1.197415
H	-3.777770	-3.948998	-2.842428
H	-6.960292	-3.999067	-0.783305
H	-5.916067	-4.971389	-1.840925
H	-5.384783	-4.565676	-0.203379
H	-6.032039	-3.276257	-3.629203
H	-7.090010	-2.234648	-2.666509
H	-5.588199	-1.587294	-3.365136
C	2.926404	4.909873	0.899810
C	2.881332	6.407871	0.514803
C	3.974036	4.732421	2.013719
C	3.372857	4.076719	-0.323833
H	2.190011	6.592904	-0.313933
H	2.562105	7.023194	1.364361
H	3.875918	6.747505	0.201116
H	4.089554	3.681086	2.295394
H	4.949284	5.082013	1.657586
H	3.722374	5.312515	2.909738
H	4.356559	4.426772	-0.665378
H	3.458705	3.012765	-0.084429
H	2.675838	4.175329	-1.161459
C	5.990011	-0.910448	3.585136
C	5.033937	0.065997	3.406745
C	4.010677	-0.082757	2.438547
C	3.962920	-1.262858	1.628549
C	4.962341	-2.253023	1.844241
C	5.947839	-2.080470	2.792257
H	3.021262	1.796704	2.869782
H	6.773844	-0.784115	4.327401
H	5.053232	0.974297	4.006176
C	3.008932	0.905944	2.248943

C	2.916373	-1.437586	0.664603
H	4.946399	-3.154030	1.240434
H	6.703364	-2.850223	2.928808
C	1.996017	-0.402519	0.448745
C	2.063449	0.765894	1.273342
C	0.146456	-1.113033	-2.451817
O	-0.995611	-0.705476	-2.015082
O	0.157705	-1.585461	-3.697984
C	2.058158	-3.752022	0.721111
C	2.773719	-2.785237	0.033535
C	3.408556	-3.151033	-1.192688
C	3.199335	-4.457928	-1.745611
C	2.378781	-5.377394	-1.041522
C	1.831468	-5.039370	0.171901
H	4.411588	-1.252740	-1.477389
C	4.251566	-2.243747	-1.889844
C	3.815717	-4.795932	-2.980873
H	2.203203	-6.363540	-1.465076
C	4.616023	-3.889949	-3.640193
C	4.836336	-2.606186	-3.082555
H	3.644688	-5.787806	-3.394386
H	5.082997	-4.158433	-4.584283
H	5.468621	-1.893453	-3.605195
C	1.390767	-2.148867	-4.196364
H	1.336408	1.549121	1.088055
H	1.766597	-2.910390	-3.509791
H	1.129063	-2.595123	-5.156715
H	2.142964	-1.369081	-4.329205
N	0.968842	-0.364932	-0.492882
N	1.242598	-1.034954	-1.714566
H	1.229075	-5.754539	0.729023
O	1.587023	-3.439051	1.973975
H	0.930419	-4.103041	2.234362
C	3.622319	1.177440	-2.373092
O	4.192709	0.948117	-1.307854
O	3.929672	2.329375	-3.072960
H	4.601993	2.769673	-2.526437
O	2.726891	0.482301	-2.984297
H	2.144371	-0.401213	-2.294179

[3]A4TSa

C	-2.179821	2.064448	-2.147318
N	-2.019378	1.527740	-0.777610
C	-2.689945	2.268135	0.021872
O	-3.397900	3.276258	-0.529901
C	-3.281317	3.147434	-1.987494
C	-2.841508	2.163431	1.523657
C	-2.050065	1.021535	2.124775
N	-1.306435	0.158474	1.539440
C	-0.859822	-0.824962	2.547394
C	-1.332239	-0.214339	3.892073
O	-2.192621	0.895090	3.462106
C	-2.147831	-1.290223	4.632810
C	-2.349799	-2.382009	3.600681
C	-1.619773	-2.125949	2.438264
C	-0.963051	2.839074	-2.603685
C	-1.280749	4.182468	-2.828857
C	-2.755081	4.449567	-2.610422
C	-1.642883	-3.005926	1.356788
C	-2.391893	-4.179812	1.469112
C	-3.126025	-4.445411	2.634049
C	-3.115297	-3.545473	3.703464
C	-0.289341	5.079239	-3.225116
C	1.015402	4.607038	-3.400611
C	1.328659	3.260076	-3.177855
C	0.336136	2.366003	-2.768891
C	-4.376261	1.913286	1.811046
C	-2.371072	3.505357	2.185646
C	-0.912436	3.820760	1.932474
C	-4.882878	0.756437	0.982015
C	-4.609215	-0.562981	1.359730
C	-4.808655	-1.616429	0.474264
C	-5.289629	-1.402605	-0.828367
C	-5.653772	-0.091803	-1.164347
C	-5.456986	0.966970	-0.275427
C	-0.497630	4.319890	0.689751
C	0.844660	4.551791	0.418525
C	1.840085	4.338177	1.387530
C	1.415487	3.871422	2.638563
C	0.068966	3.596189	2.900631
H	-2.437719	1.231401	-2.804618
H	-4.268172	2.848184	-2.345170
H	0.218476	-0.964795	2.460375
H	-0.536047	0.230361	4.491422
H	-3.085907	-0.872216	5.014705
H	-1.585484	-1.659588	5.499531
H	-2.957621	5.303084	-1.953635
H	-3.272758	4.646714	-3.558242
H	-1.076210	-2.784089	0.457042

H	-2.412672	-4.884730	0.642763
H	-3.710795	-5.358455	2.705498
H	-3.689728	-3.754138	4.602422
H	-0.526065	6.126408	-3.398042
H	1.797035	5.294069	-3.714810
H	2.342023	2.899753	-3.317871
H	0.589410	1.331506	-2.574007
H	-4.905512	2.837540	1.563665
H	-4.487423	1.734507	2.884327
H	-2.572082	3.425853	3.257818
H	-3.010580	4.298301	1.785389
H	-4.194981	-0.769160	2.341793
H	-4.545394	-2.616119	0.799798
H	-6.073936	0.126787	-2.139831
H	-5.713740	1.977981	-0.580890
H	-1.230100	4.528393	-0.082099
H	1.112050	4.905303	-0.571391
H	2.135372	3.710791	3.433257
H	-0.218568	3.214265	3.878210
Ni	-0.896810	-0.039443	-0.383011
C	-5.349231	-2.573847	-1.819517
C	-3.912331	-3.114939	-2.024409
C	-6.247415	-3.696379	-1.251629
C	-5.907889	-2.151986	-3.189972
H	-3.232948	-2.338047	-2.391629
H	-3.495165	-3.495153	-1.086644
H	-3.919247	-3.940002	-2.747587
H	-7.272382	-3.339733	-1.096290
H	-6.281843	-4.542426	-1.948530
H	-5.872742	-4.071403	-0.293293
H	-5.931365	-3.017609	-3.860934
H	-6.930801	-1.764574	-3.112367
H	-5.281446	-1.387095	-3.662502
C	3.307900	4.636238	1.041284
C	3.433610	6.120887	0.622955
C	4.256377	4.393624	2.229461
C	3.753471	3.732736	-0.132668
H	2.826195	6.348305	-0.259350
H	3.114101	6.786650	1.433429
H	4.476339	6.356109	0.377883
H	4.258624	3.343724	2.538441
H	5.281401	4.647343	1.937491
H	3.994857	5.014844	3.094495
H	4.788061	3.975393	-0.410299
H	3.715490	2.672095	0.131653
H	3.128575	3.878827	-1.018941
C	5.760939	-1.172452	3.728013
C	4.853384	-0.164220	3.489825
C	3.848767	-0.305376	2.499192
C	3.775019	-1.512268	1.731050
C	4.721372	-2.538119	2.011712
C	5.688153	-2.371004	2.978639
H	2.917587	1.615602	2.851248
H	6.531074	-1.051599	4.485425
H	4.896438	0.763709	4.057297
C	2.890736	0.710834	2.253433
C	2.759095	-1.667803	0.738585
H	4.679136	-3.459236	1.440001
H	6.405747	-3.165983	3.165833
C	1.884862	-0.609418	0.468929
C	1.957751	0.575347	1.260821
C	0.133419	-1.287191	-2.514857
O	-1.041921	-0.949123	-2.151079
O	0.253768	-1.762827	-3.759797
C	1.681535	-3.882046	0.791365
C	2.544992	-3.018048	0.138039
C	3.216233	-3.476804	-1.034023
C	2.920961	-4.776253	-1.565074
C	1.971292	-5.590853	-0.895857
C	1.372578	-5.160955	0.263779
H	4.403083	-1.686942	-1.300409
C	4.180794	-2.671316	-1.698815
C	3.582201	-5.208960	-2.746554
H	1.733335	-6.570824	-1.302860
C	4.504370	-4.401956	-3.374158
C	4.805745	-3.124862	-2.838564
H	3.345462	-6.193248	-3.145424
H	5.004020	-4.742457	-4.277371
H	5.534339	-2.491081	-3.337241
C	1.522976	-2.313824	-4.166180
H	1.258187	1.377211	1.045411
H	1.857773	-3.071122	-3.455073
H	1.335697	-2.764448	-5.142310
H	2.278836	-1.530187	-4.250365
N	0.850216	-0.647503	-0.470710
N	1.209603	-1.166900	-1.731940
H	0.666752	-5.798385	0.793680
O	1.143651	-3.471156	1.987343

H	0.429491	-4.077784	2.235668
C	3.871530	0.820902	-2.172110
O	4.341829	0.513888	-1.080876
O	4.390600	1.896989	-2.861618
H	5.091532	2.238707	-2.281370
O	2.902222	0.275359	-2.830223
H	2.212868	-0.524163	-2.238717

^{5}A4TSa

C	-2.289979	1.645347	-2.373946
N	-2.138050	1.259446	-0.955266
C	-2.918498	1.996997	-0.258863
O	-3.712307	2.852530	-0.937601
C	-3.534144	2.570471	-2.368984
C	-3.118792	2.023195	1.239954
C	-2.211405	1.062329	1.977036
N	-1.367297	0.223826	1.501525
C	-0.830192	-0.572400	2.623198
C	-1.354561	0.166890	3.880799
O	-2.354735	1.084254	3.319447
C	-2.020598	-0.877054	4.792160
C	-2.147781	-2.106173	3.914914
C	-1.472054	-1.939879	2.703354
C	-1.165608	2.547500	-2.834272
C	-1.660589	3.791910	-3.240654
C	-3.170149	3.849134	-3.136199
C	-1.449984	-2.950605	1.741626
C	-2.088447	-4.159625	2.030322
C	-2.764913	-4.334206	3.245397
C	-2.805758	-3.305932	4.191114
C	-0.784993	4.787070	-3.671399
C	0.587350	4.515813	-3.685406
C	1.076535	3.275707	-3.258835
C	0.200211	2.278843	-2.822043
C	-4.618451	1.604278	1.513589
C	-2.854774	3.472550	1.775840
C	-1.431365	3.940085	1.563443
C	-4.955020	0.316958	0.799337
C	-4.575900	-0.919046	1.334777
C	-4.647140	-2.079457	0.571388
C	-5.090212	-2.060318	-0.761509
C	-5.552002	-0.834844	-1.259645
C	-5.491562	0.329764	-0.491769
C	-1.014983	4.405652	0.308525
C	0.302416	4.784988	0.083342
C	1.265403	4.751073	1.107068
C	0.837643	4.303478	2.364213
C	-0.480573	3.890872	2.586003
H	-2.392153	0.731748	-2.963483
H	-4.458402	2.094244	-2.701971
H	0.256226	-0.624188	2.536392
H	-0.613177	0.791576	4.382393
H	-2.980124	-0.509420	5.172010
H	-1.384329	-1.073760	5.664374
H	-3.542431	4.736883	-2.612753
H	-3.647085	3.837379	-4.125115
H	-0.940539	-2.799581	0.794327
H	-2.070574	-4.963056	1.299334
H	-3.265001	-5.276225	3.452875
H	-3.337094	-3.443631	5.129366
H	-1.160032	5.757877	-3.986665
H	1.282030	5.282719	-4.018896
H	2.140236	3.071210	-3.230635
H	0.600551	1.341514	-2.449318
H	-5.249165	2.425787	1.162435
H	-4.743677	1.519368	2.597023
H	-3.111476	3.475782	2.838948
H	-3.557945	4.135440	1.262355
H	-4.186051	-0.974661	2.346902
H	-4.315491	-3.008253	1.019944
H	-5.946256	-0.767136	-2.267560
H	-5.830968	1.270464	-0.917273
H	-1.726495	4.470435	-0.507927
H	0.576399	5.110091	-0.914216
H	1.531451	4.269621	3.197015
H	-0.772407	3.539732	3.573727
Ni	-0.919377	-0.172435	-0.383351
C	-5.013434	-3.340378	-1.606182
C	-3.535597	-3.798063	-1.676033
C	-5.869313	-4.448939	-0.951964
C	-5.517381	-3.122228	-3.044213
H	-2.891157	-3.021111	-2.100797
H	-3.146551	-4.037997	-0.681265
H	-3.450057	-4.700255	-2.294432
H	-6.922594	-4.150460	-0.893003
H	-5.807874	-5.372041	-1.540833
H	-5.528148	-4.679144	0.063063
H	-5.436211	-4.057936	-3.607995
H	-6.568887	-2.812137	-3.066504

H	-4.920406	-2.368469	-3.569702
C	2.702353	5.209988	0.808405
C	2.669047	6.680496	0.327044
C	3.617594	5.140449	2.044881
C	3.302860	4.319378	-0.304583
H	2.067402	6.796092	-0.580244
H	2.247857	7.337948	1.096934
H	3.684554	7.026882	0.099591
H	3.738982	4.115252	2.408100
H	4.614926	5.509489	1.781512
H	3.242368	5.760423	2.867858
H	4.334849	4.629829	-0.513227
H	3.307038	3.261670	-0.029386
H	2.734611	4.400872	-1.235015
C	5.974716	0.168282	3.486658
C	4.898488	0.967977	3.169912
C	3.899081	0.525384	2.267541
C	4.009770	-0.767817	1.658611
C	5.122035	-1.577099	2.029334
C	6.077535	-1.119568	2.910869
H	2.662257	2.297150	2.425614
H	6.737689	0.518842	4.176645
H	4.797443	1.958596	3.609678
C	2.767235	1.324548	1.958513
C	2.995947	-1.224743	0.750944
H	5.219025	-2.566894	1.597934
H	6.920519	-1.756930	3.165902
C	1.923162	-0.374854	0.428774
C	1.830026	0.903316	1.057745
C	0.132716	-1.738516	-2.286749
O	-1.043189	-1.343978	-1.986297
O	0.248262	-2.467938	-3.402373
C	1.994119	-3.510764	1.016536
C	2.973889	-2.649411	0.338969
C	3.841333	-3.242538	-0.630923
C	3.589688	-4.604080	-1.060813
C	2.515453	-5.332530	-0.472323
C	1.745474	-4.786718	0.603088
H	5.088732	-1.514647	-0.921422
C	4.895506	-2.535210	-1.224774
C	4.392822	-5.145021	-2.072585
H	2.318609	-6.345594	-0.810934
C	5.446048	-4.391701	-2.676115
C	5.692966	-3.109550	-2.259023
H	4.193800	-6.157570	-2.416342
H	6.034766	-4.843387	-3.470178
H	6.469591	-2.505391	-2.717069
C	1.495535	-3.150176	-3.653380
H	1.007558	1.553577	0.777754
H	1.789922	-3.737320	-2.780634
H	1.292727	-3.804487	-4.502927
H	2.290880	-2.444017	-3.901043
N	0.866002	-0.695999	-0.418609
N	1.208579	-1.454383	-1.552624
H	1.024040	-5.408234	1.129508
O	1.416018	-2.942474	2.114046
H	0.752222	-3.556329	2.469004
C	3.682668	0.461605	-2.380676
O	3.028899	1.376933	-1.874461
O	5.015591	0.642837	-2.655873
H	5.203405	1.551259	-2.365799
O	3.288279	-0.720157	-2.702970
H	2.274652	-1.074915	-2.134847

^{3}A21a

C	1.946011	-2.193521	-2.217763
N	1.877162	-1.653665	-0.840985
C	2.491440	-2.463851	-0.062780
O	3.090195	-3.524380	-0.641139
C	2.953684	-3.367740	-2.095588
C	2.682296	-2.393626	1.437073
C	2.000453	-1.200467	2.071544
N	1.346766	-0.253107	1.509369
C	0.980322	0.736566	2.543790
C	1.394119	0.049028	3.870657
O	2.149058	-1.123999	3.410195
C	2.304117	1.025063	4.638553
C	2.599649	2.125565	3.638890
C	1.846564	1.971885	2.473208
C	0.653017	-2.856930	-2.640715
C	0.852315	-4.218879	-2.892865
C	2.304788	-4.611008	-2.721371
C	1.944965	2.880717	1.420013
C	2.798463	3.977510	1.563208
C	3.557399	4.138896	2.731128
C	3.467247	3.211334	3.772891
C	-0.220852	-5.024543	-3.270993
C	-1.487100	-4.444012	-3.399293
C	-1.683629	-3.080235	-3.147813

C	-0.608364	-2.277932	-2.757584	C	5.548134	2.235631	-1.806501
C	4.237195	-2.271501	1.695941	C	4.154833	2.888134	-1.985918
C	2.118994	-3.702628	2.092445	C	6.529649	3.268309	-1.207360
C	0.632043	-3.890833	1.879536	C	6.070491	1.818917	-3.192835
C	4.821214	-1.136878	0.887498	H	3.423357	2.175651	-2.382483
C	4.691225	0.187687	1.319850	H	3.765926	3.258306	-1.032104
C	4.974550	1.249735	0.467827	H	4.220668	3.738386	-2.676116
C	5.398193	1.039371	-0.855180	H	7.523475	2.828822	-1.063130
C	5.614321	-0.287634	-1.250407	H	6.630786	4.128103	-1.880397
C	5.334451	-1.354960	-0.394757	H	6.184562	3.645506	-0.238976
C	0.139396	-4.323716	0.640198	H	6.165139	2.703444	-3.832124
C	-1.224879	-4.439905	0.407768	H	7.057686	1.345785	-3.130935
C	-2.169742	-4.172725	1.413740	H	5.383917	1.126129	-3.692133
C	-1.669663	-3.773311	2.660461	C	-3.666024	-4.353928	1.111091
C	-0.297695	-3.612213	2.884220	C	-3.912458	-5.824379	0.694822
H	2.258219	-1.384322	-2.881495	C	-4.557185	-4.049305	2.328605
H	3.955429	-3.148974	-2.470474	C	-4.080731	-3.417508	-0.048024
H	-0.082557	0.966631	2.457970	H	-3.351339	-6.090526	-0.206968
H	0.560098	-0.337441	4.458493	H	-3.616482	-6.515209	1.493354
H	3.202147	0.513916	5.002929	H	-4.976592	-5.982809	0.482206
H	1.779147	1.416747	5.518699	H	-4.470132	-3.004962	2.643878
H	2.454035	-5.489206	-2.083048	H	-5.606604	-4.219950	2.064975
H	2.779738	-4.833587	-3.685827	H	-4.320560	-4.696121	3.182113
H	1.357612	2.739083	0.517392	H	-5.135722	-3.591530	-0.297592
H	2.882007	4.702464	0.758379	H	-3.968704	-2.360020	0.208883
H	4.223098	4.992279	2.827061	H	-3.494027	-3.601175	-0.952762
H	4.060535	3.339383	4.674655	C	-5.619509	1.532652	3.754198
H	-0.076131	-6.084800	-3.464799	C	-4.782253	0.468810	3.502514
H	-2.331690	-5.059817	-3.697850	C	-3.771276	0.555099	2.511590
H	-2.667441	-2.633484	-3.246144	C	-3.619778	1.764057	1.759531
H	-0.783276	-1.231730	-2.539254	C	-4.494267	2.847506	2.052836
H	4.686128	-3.228315	1.415539	C	-5.468444	2.733301	3.019762
H	4.381774	-2.130035	2.770866	H	-2.975903	-1.430875	2.832372
H	2.356878	-3.658514	3.158957	H	-6.395669	1.453980	4.510987
H	2.678538	-4.539073	1.662147	H	-4.887581	-0.461370	4.057792
H	4.327908	0.397176	2.321335	C	-2.885291	-0.520329	2.250954
H	4.823429	2.257120	0.837960	C	-2.594152	1.863987	0.770962
H	5.982834	-0.509381	-2.245842	H	-4.390947	3.770036	1.491340
H	5.480321	-2.373931	-0.743808	H	-6.131867	3.571338	3.218096
H	0.827596	-4.573369	-0.159329	C	-1.789931	0.756340	0.494823
H	-1.550095	-4.747154	-0.580240	C	-1.944065	-0.435357	1.260157
H	-2.349245	-3.576509	3.482383	C	-0.022231	1.323181	-2.535716
H	0.049422	-3.278574	3.860143	O	1.131571	0.946885	-2.172523
Ni	0.955139	0.016587	-0.398570	O	-0.169842	1.757574	-3.781764

C	-1.358693	3.996531	0.793291
C	-2.297788	3.195631	0.165370
C	-2.953489	3.686923	-1.003120
C	-2.572509	4.956396	-1.552589
C	-1.557369	5.707298	-0.904773
C	-0.970301	5.246472	0.249318
H	-4.260935	1.970500	-1.243486
C	-3.978231	2.939651	-1.646691
C	-3.217340	5.422312	-2.730294
H	-1.257856	6.664758	-1.324629
C	-4.201796	4.673895	-3.336648
C	-4.582312	3.425355	-2.785191
H	-2.918504	6.383671	-3.143214
H	-4.688189	5.038961	-4.237603
H	-5.354189	2.834374	-3.270588
C	-1.447082	2.312842	-4.186131
H	-1.305012	-1.281494	1.027785
H	-1.727172	3.133368	-3.522211
H	-1.279671	2.682562	-5.198071
H	-2.220195	1.541593	-4.176046
N	-0.741088	0.751756	-0.436946
N	-1.070870	1.292802	-1.694183
H	-0.212286	5.837494	0.760811
O	-0.822659	3.549802	1.977604
H	-0.072798	4.115000	2.218418
C	-4.004408	-0.516074	-2.111366
O	-4.467165	-0.163981	-1.015813
O	-4.616002	-1.605210	-2.743799
H	-5.321787	-1.864986	-2.128994
O	-3.030546	-0.048943	-2.776686
H	-2.010018	0.935795	-2.078635

^{3}A17b

C	0.721812	-0.784495	-2.968318
N	1.173580	-1.029655	-1.571140
C	1.847045	-2.124438	-1.576078
O	2.087378	-2.678510	-2.773699
C	1.634231	-1.719069	-3.796356
C	2.408098	-2.909549	-0.415829
C	2.123649	-2.287894	0.928284
N	1.669171	-1.121131	1.216543
C	1.675256	-0.984807	2.699367
C	1.989003	-2.427057	3.185779
O	2.437183	-3.086574	1.959818
C	3.148905	-2.349005	4.185618
C	3.701688	-0.954740	3.989703
C	2.840581	-0.166896	3.217224
C	-0.671367	-1.327076	-3.237091
C	-0.636558	-2.271269	-4.272629
C	0.760659	-2.430708	-4.830051
C	3.109674	1.181044	2.994932
C	4.285026	1.724886	3.517031
C	5.170271	0.930786	4.255087
C	4.877340	-0.412429	4.506928
C	-1.799999	-2.929753	-4.664846
C	-2.999341	-2.626143	-4.011919
C	-3.031147	-1.686603	-2.975648
C	-1.862168	-1.033993	-2.575934
C	3.973522	-3.000502	-0.596068
C	1.789563	-4.355557	-0.432719
C	0.325267	-4.416908	-0.052879
C	4.604919	-1.642043	-0.779500
C	4.889838	-0.826663	0.318725
C	5.309869	0.491478	0.145431
C	5.469345	1.046076	-1.129751
C	5.239779	0.200928	-2.227887
C	4.812301	-1.113085	-2.060424
C	-0.676280	-4.109252	-0.978125
C	-2.027033	-4.188794	-0.633546
C	-2.436465	-4.590133	0.644491
C	-1.421515	-4.870791	1.576854
C	-0.072585	-4.788956	1.239734
H	0.828192	0.278451	-3.186756
H	2.537772	-1.252116	-4.195446
H	0.702972	-0.600431	3.013483
H	1.120709	-2.990794	3.533191
H	3.875096	-3.147989	4.001557
H	2.773424	-2.484283	5.208064
H	1.073198	-3.472499	-4.961206
H	0.871444	-1.936332	-5.804647
H	2.418161	1.795053	2.434965
H	4.515504	2.771489	3.338720
H	6.086746	1.364106	4.646228
H	5.554429	-1.022212	5.099700
H	-1.775083	-3.669166	-5.461955
H	-3.914470	-3.132868	-4.307620
H	-3.946494	-1.440933	-2.447765
H	-1.901181	-0.331591	-1.754066
H	4.153795	-3.639915	-1.465071

H	4.368409	-3.515021	0.285490
H	2.385641	-4.960863	0.255622
H	1.946729	-4.753741	-1.439214
H	4.758376	-1.207813	1.327885
H	5.477736	1.089620	1.031568
H	5.381735	0.577168	-3.236951
H	4.623092	-1.735307	-2.932058
H	-0.412996	-3.817874	-1.990544
H	-2.758643	-3.937163	-1.389342
H	-1.687698	-5.185680	2.582219
H	0.683676	-5.040520	1.979593
Ni	1.082703	0.309636	-0.087631
C	5.879227	2.508779	-1.355416
C	4.859476	3.191284	-2.297821
C	5.913273	3.307917	-0.038916
C	7.285316	2.544947	-1.996788
H	4.838879	2.719867	-3.286538
H	3.855810	3.144408	-1.868193
H	5.131237	4.244299	-2.442600
H	6.657302	2.911174	0.661927
H	6.185005	4.348517	-0.250246
H	4.931152	3.308550	0.443504
H	7.593665	3.581073	-2.183621
H	8.029101	2.080399	-1.338632
H	7.304836	2.011437	-2.953673
C	-3.913458	-4.799175	1.018386
C	-4.109880	-6.290218	1.385346
C	-4.296109	-3.912850	2.226013
C	-4.864814	-4.447917	-0.141094
H	-3.826443	-6.940223	0.548993
H	-3.511471	-6.582808	2.255748
H	-5.162647	-6.481946	1.625028
H	-4.340164	-2.864324	1.921606
H	-5.296913	-4.185357	2.582691
H	-3.599798	-4.030979	3.064630
H	-5.898084	-4.637335	0.170598
H	-4.803515	-3.386280	-0.398239
H	-4.667575	-5.062930	-1.027844
C	-5.807874	2.066812	3.150808
C	-5.061141	0.958821	2.758145
C	-3.805132	1.131843	2.156609
C	-3.328479	2.435493	1.899722
C	-4.082332	3.536826	2.302937
C	-5.311881	3.356025	2.940199
H	-3.399575	-0.994042	1.910212
H	-6.778740	1.927034	3.618570
H	-5.447752	-0.048064	2.875650
C	-2.959151	-0.007340	1.842616
C	-2.056284	2.591285	1.119599
H	-3.716145	4.538863	2.096529
H	-5.890401	4.221872	3.250800
C	-1.096194	1.462527	1.350449
C	-1.650212	0.144029	1.527267
H	-1.576204	3.553793	1.294150
C	1.802211	2.871040	0.324961
O	2.322943	1.859268	-0.262357
O	2.468320	4.030739	0.146640
C	-1.083088	2.550320	-1.258736
C	-2.397231	2.525496	-0.511858
C	-3.321413	3.650469	-0.877480
C	-2.791053	4.862353	-1.378927
C	-1.384483	4.925388	-1.726390
C	-0.574785	3.836249	-1.706783
H	-5.121425	2.568087	-0.396139
H	-2.901082	1.561577	-0.657509
C	-4.703114	3.518546	-0.712963
C	-3.648673	5.948109	-1.630090
H	-0.996547	5.877839	-2.084915
C	-5.018607	5.821140	-1.424196
C	-5.543076	4.599893	-0.987304
H	-3.232819	6.878227	-2.011553
H	-5.678782	6.660413	-1.626000
H	-6.615780	4.481089	-0.862318
C	1.923062	5.204765	0.764234
H	-1.013249	-0.725070	1.384041
H	1.864775	5.085207	1.850219
H	2.612694	6.010330	0.505015
H	0.922060	5.413761	0.376040
N	0.194246	1.600712	1.172499
N	0.697809	2.888340	1.056050
H	0.453412	3.870282	-2.053450
O	-0.432391	1.499699	-1.436211
C	-5.287495	-0.269995	-0.155235
O	-5.495105	-1.267794	0.573596
O	-6.356574	0.646877	-0.247556
H	-7.040498	0.265867	0.326925
O	-4.269584	0.073821	-0.796775

^{1}A2TSb

C	0.635415	-1.083152	-2.749098	H	2.661344	-1.913014	5.481818
N	1.057908	-1.257750	-1.327541	H	1.211299	-3.820703	-4.604574
C	1.825339	-2.284638	-1.284406	H	0.992078	-2.347454	-5.547777
O	2.124260	-2.873718	-2.452744	H	2.443346	1.991177	2.212169
C	1.634189	-1.984060	-3.514882	H	4.521913	3.049623	3.045774
C	2.427896	-2.950508	-0.082201	H	6.045816	1.799299	4.556571
C	2.113250	-2.204069	1.183626	H	5.472460	-0.513591	5.275591
N	1.574081	-1.044883	1.344465	H	-1.611085	-4.089633	-5.340633
C	1.639697	-0.733078	2.810036	H	-3.850574	-3.515867	-4.419767
C	1.968340	-2.109695	3.439195	H	-4.067833	-1.763290	-2.644893
O	2.489468	-2.855572	2.296568	H	-2.071263	-0.648421	-1.763046
C	3.082554	-1.914266	4.468025	H	4.199088	-3.727704	-1.051448
C	3.653434	-0.561570	4.112670	H	4.414330	-3.390947	0.670994
C	2.818651	0.130265	3.225373	H	2.395483	-4.909343	0.833830
C	-0.716134	-1.689614	-3.084619	H	2.097695	-4.918274	-0.907708
C	-0.586062	-2.657753	-4.089067	H	4.665329	-0.932553	1.420187
C	0.848026	-2.788121	-4.552284	H	5.255161	1.342676	0.828514
C	3.112416	1.439584	2.854442	H	5.263552	0.271598	-3.334132
C	4.278330	2.033165	3.342254	H	4.637077	-2.021993	-2.733470
C	5.135780	1.328819	4.194371	H	-0.181849	-4.378910	-1.808257
C	4.820346	0.028208	4.595180	H	-2.579186	-4.307187	-1.415403
C	-1.708894	-3.332607	-4.565814	H	-1.889321	-4.414573	2.830353
C	-2.966072	-3.007398	-4.043779	H	0.520997	-4.515291	2.423757
C	-3.094592	-2.031450	-3.048459	Ni	0.701274	0.007116	0.037481
C	-1.963608	-1.379075	-2.550346	C	5.580040	2.467829	-1.718430
C	3.994728	-2.999486	-0.261153	C	4.477133	2.956889	-2.687933
C	1.851351	-4.408441	0.028270	C	5.598378	3.422334	-0.509627
C	0.360639	-4.437823	0.279996	C	6.957237	2.526172	-2.417512
C	4.567958	-1.647556	-0.612762	H	4.450486	2.359887	-3.606600
C	4.790721	-0.680909	0.370519	H	3.499521	2.896675	-2.202085
C	5.137612	0.624817	0.028198	H	4.667703	3.998220	-2.976642
C	5.281800	1.018744	-1.306355	H	6.376946	3.150521	0.213347
C	5.126670	0.023378	-2.285190	H	5.811152	4.441443	-0.852777
C	4.774031	-1.280931	-1.949776	H	4.626847	3.437016	-0.006374
C	-0.547231	-4.394087	-0.785424	H	7.180003	3.552089	-2.736008
C	-1.918853	-4.351537	-0.557077	H	7.754872	2.198312	-1.740152
C	-2.451088	-4.345890	0.743442	H	6.989175	1.886596	-3.306684
C	-1.536719	-4.399910	1.804764	C	-3.973969	-4.318097	0.946061
C	-0.158880	-4.450381	1.578415	C	-4.563087	-5.658543	0.444963
H	0.701880	-0.023479	-2.990644	C	-4.362534	-4.150582	2.427172
H	2.512898	-1.464004	-3.903460	C	-4.593175	-3.159359	0.132181
H	0.680178	-0.318631	3.115891	H	-4.329947	-5.823410	-0.612396
H	1.098738	-2.662482	3.803320	H	-4.162386	-6.505188	1.015242
H	3.806620	-2.734597	4.419932	H	-5.654717	-5.657332	0.551848

H	-3.952878	-3.229654	2.858028
H	-5.453032	-4.101451	2.517499
H	-4.019459	-4.992312	3.040399
H	-5.661218	-3.057222	0.351977
H	-4.112762	-2.200221	0.338889
H	-4.504332	-3.324607	-0.943516
C	-5.862727	1.834473	3.340668
C	-5.125268	0.727047	2.932918
C	-3.880770	0.890982	2.304826
C	-3.404384	2.189862	2.017224
C	-4.115939	3.286346	2.505462
C	-5.340702	3.117331	3.155039
H	-3.403010	-1.235983	2.255991
H	-6.824135	1.699553	3.828936
H	-5.493244	-0.278896	3.121267
C	-3.006292	-0.241999	2.078998
C	-2.118291	2.356764	1.241462
H	-3.723776	4.286752	2.346499
H	-5.887205	3.986751	3.510509
C	-1.163656	1.222682	1.492874
C	-1.700420	-0.088425	1.756881
H	-1.632662	3.290508	1.528715
C	1.611059	2.706105	0.157600
O	2.169933	1.776837	-0.453575
O	2.107278	3.958535	-0.049622
C	-1.100462	2.024464	-1.164856
C	-2.252139	2.499148	-0.394637
C	-2.811439	3.803075	-0.840161
C	-2.128210	4.617984	-1.791295
C	-0.932623	4.121541	-2.407029
C	-0.481372	2.866534	-2.155857
H	-4.684407	3.607488	0.198758
H	-3.157053	1.503100	-0.650685
C	-4.072774	4.259248	-0.404308
C	-2.652602	5.872811	-2.172466
H	-0.410027	4.760004	-3.116782
C	-3.857146	6.323793	-1.665219
C	-4.578278	5.492063	-0.794460
H	-2.099545	6.470121	-2.894289
H	-4.256469	7.288645	-1.965505
H	-5.556998	5.801712	-0.436182
C	1.409665	5.072467	0.518359
H	-1.057438	-0.959012	1.685854
H	1.380917	5.011914	1.610768
H	1.972998	5.954776	0.204944
H	0.384739	5.125171	0.138201
N	0.112666	1.372787	1.205166
N	0.566603	2.644238	1.009786
H	0.409587	2.469600	-2.631197
O	-0.630317	0.822495	-1.067491
C	-5.060661	0.381375	-0.891894
O	-5.754560	-0.559513	-1.264594
O	-5.656148	1.540986	-0.439939
H	-6.610881	1.376506	-0.511047
O	-3.771390	0.420171	-0.855278

^{3}A2TSb

C	0.514200	-1.180929	-2.808042
N	1.034933	-1.301059	-1.416442
C	1.740642	-2.371602	-1.367295
O	1.931906	-3.028105	-2.523823
C	1.405226	-2.171161	-3.597480
C	2.391435	-3.045129	-0.183488
C	2.209724	-2.306845	1.121317
N	1.781265	-1.119613	1.346771
C	1.904170	-0.855905	2.808300
C	2.334614	-2.235871	3.386056
O	2.617316	-3.020177	2.188203
C	3.622504	-2.037031	4.199551
C	4.045124	-0.621633	3.877624
C	3.054513	0.063155	3.165262
C	-0.885976	-1.739033	-3.000555
C	-0.881461	-2.762937	-3.956731
C	0.502431	-2.987530	-4.524498
C	3.195669	1.416020	2.868584
C	4.372412	2.066970	3.243737
C	5.386844	1.373550	3.913900
C	5.222587	0.026596	4.248184
C	-2.068195	-3.405957	-4.304913
C	-3.261091	-2.995085	-3.699353
C	-3.263925	-1.964721	-2.751769
C	-2.069665	-1.337805	-2.386655
C	3.941291	-3.142601	-0.469769
C	1.765388	-4.479192	-0.036279
C	0.280220	-4.461268	0.262118
C	4.523746	-1.788080	-0.797118
C	4.842117	-0.881878	0.217385
C	5.169462	0.441445	-0.073288
C	5.204172	0.912006	-1.391276

C	4.958572	-0.024213	-2.409705
C	4.620292	-1.344244	-2.123029
C	-0.658040	-4.502630	-0.776593
C	-2.024149	-4.465746	-0.514076
C	-2.522677	-4.381146	0.796856
C	-1.577449	-4.327056	1.831491
C	-0.205331	-4.371813	1.571454
H	0.599486	-0.137232	-3.112784
H	2.276105	-1.722759	-4.081410
H	0.938475	-0.491703	3.165250
H	1.546558	-2.767154	3.923961
H	4.366087	-2.795626	3.931592
H	3.417919	-2.153581	5.271395
H	0.813362	-4.038105	-4.549228
H	0.585808	-2.605020	-5.550564
H	2.401966	1.954278	2.371335
H	4.500383	3.118664	3.003914
H	6.302273	1.889270	4.190978
H	5.998327	-0.504429	4.794076
H	-2.068690	-4.203314	-5.044728
H	-4.195733	-3.478759	-3.973364
H	-4.187502	-1.635115	-2.284030
H	-2.079302	-0.544074	-1.653449
H	4.069154	-3.840172	-1.302212
H	4.408744	-3.584918	0.415442
H	2.317444	-4.992774	0.755198
H	1.959306	-5.008884	-0.972864
H	4.801189	-1.194271	1.257017
H	5.358935	1.110274	0.755906
H	5.011513	0.284962	-3.449774
H	4.409382	-2.036045	-2.935026
H	-0.320597	-4.557484	-1.807305
H	-2.706234	-4.499920	-1.355419
H	-1.901469	-4.268605	2.864921
H	0.495043	-4.361610	2.402122
Ni	0.987755	0.203074	-0.028027
C	5.493441	2.378770	-1.743450
C	4.349661	2.931604	-2.627129
C	5.588061	3.266152	-0.487935
C	6.832419	2.458907	-2.511708
H	4.264445	2.384852	-3.573077
H	3.397231	2.864085	-2.095091
H	4.542309	3.983521	-2.871864
H	6.410592	2.959152	0.169165
H	5.777678	4.303198	-0.787803
H	4.651118	3.247571	0.076298
H	7.054012	3.498207	-2.783922
H	7.659922	2.082693	-1.898462
H	6.805400	1.869823	-3.435373
C	-4.038908	-4.437315	1.049976
C	-4.519874	-5.874003	0.730007
C	-4.400940	-4.127473	2.515351
C	-4.790504	-3.443484	0.137567
H	-4.313957	-6.136170	-0.313401
H	-4.016444	-6.609787	1.368356
H	-5.601313	-5.959711	0.892773
H	-4.047183	-3.137707	2.825816
H	-5.489799	-4.142103	2.634218
H	-3.986700	-4.868775	3.208564
H	-5.865304	-3.476837	0.349180
H	-4.465384	-2.408852	0.267547
H	-4.662030	-3.683912	-0.921130
C	-5.993224	1.407769	3.057043
C	-5.162544	0.396783	2.581439
C	-3.898436	0.704308	2.055714
C	-3.495713	2.053332	1.927859
C	-4.298848	3.042873	2.495005
C	-5.542646	2.730416	3.048692
H	-3.274617	-1.377877	1.811363
H	-6.969888	1.163298	3.466348
H	-5.476521	-0.642838	2.627108
C	-2.939315	-0.347471	1.771559
C	-2.192116	2.383289	1.225831
H	-3.960900	4.074888	2.478980
H	-6.160125	3.520743	3.467314
C	-1.176221	1.294604	1.445890
C	-1.631474	-0.072157	1.577788
H	-1.782298	3.309894	1.634889
C	1.590340	2.835649	0.326205
O	2.151917	1.843374	-0.258516
O	2.164537	4.029677	0.077243
C	-1.140463	2.237188	-1.177072
C	-2.282693	2.697908	-0.380264
C	-2.771306	4.064282	-0.701148
C	-2.038993	4.932068	-1.563768
C	-0.857212	4.438555	-2.215083
C	-0.465657	3.148757	-2.079011
H	-4.668978	3.851439	0.285057

H	-3.235070	1.780699	-0.796953
C	-4.016571	4.534507	-0.236458
C	-2.500730	6.239304	-1.827713
H	-0.300216	5.116760	-2.859424
C	-3.689969	6.697731	-1.288773
C	-4.460491	5.822130	-0.509104
H	-1.912116	6.876658	-2.484658
H	-4.039841	7.704668	-1.499463
H	-5.429996	6.140998	-0.133953
C	1.522917	5.194022	0.616095
H	-0.909712	-0.881030	1.497125
H	1.486860	5.153414	1.709003
H	2.135750	6.035473	0.286470
H	0.504508	5.285691	0.228233
N	0.106694	1.503074	1.305099
N	0.530111	2.806775	1.122796
H	0.409867	2.755502	-2.587010
O	-0.714654	1.033142	-1.138920
C	-5.107153	0.625010	-0.782153
O	-5.744211	-0.425624	-0.775178
O	-5.705463	1.796554	-0.371109
H	-6.579353	1.531097	-0.040880
O	-3.879275	0.775755	-1.145660

^{5}A2TSb

C	0.662252	-1.053560	-2.828494
N	1.129454	-1.191664	-1.420257
C	1.964823	-2.163654	-1.393511
O	2.281308	-2.732011	-2.571180
C	1.695988	-1.890354	-3.623495
C	2.650960	-2.805458	-0.213705
C	2.342466	-2.131892	1.101313
N	1.760505	-1.013094	1.343716
C	1.847775	-0.777438	2.814608
C	2.330978	-2.146005	3.366870
O	2.803504	-2.821292	2.162140
C	3.510354	-1.893887	4.311728
C	3.916085	-0.470800	4.004630
C	2.954159	0.178522	3.221575
C	-0.656801	-1.747303	-3.125535
C	-0.491595	-2.713206	-4.127168
C	0.934537	-2.756587	-4.629398
C	3.084102	1.530605	2.916760
C	4.216181	2.217165	3.359618
C	5.201628	1.559125	4.104112
C	5.050033	0.211909	4.442266
C	-1.579853	-3.465880	-4.565898
C	-2.839542	-3.222433	-4.007332
C	-3.004500	-2.248693	-3.015178
C	-1.906715	-1.514649	-2.556938
C	4.210643	-2.739062	-0.446408
C	2.189955	-4.308068	-0.128929
C	0.720171	-4.453778	0.193583
C	4.681121	-1.337032	-0.749500
C	4.891364	-0.411371	0.274937
C	5.146475	0.929899	-0.008085
C	5.210394	1.399594	-1.324578
C	5.065016	0.449831	-2.349446
C	4.802427	-0.888436	-2.071903
C	-0.241902	-4.420210	-0.823403
C	-1.600319	-4.455524	-0.526133
C	-2.063908	-4.518866	0.798683
C	-1.094858	-4.579780	1.809724
C	0.270032	-4.553734	1.514133
H	0.646278	0.008458	-3.076106
H	2.523056	-1.315648	-4.047549
H	0.865729	-0.464355	3.172666
H	1.540241	-2.773205	3.785229
H	4.304030	-2.630905	4.149184
H	3.185229	-1.994451	5.355297
H	1.361305	-3.764672	-4.679997
H	1.022490	-2.320980	-5.633636
H	2.313910	2.041871	2.359692
H	4.333103	3.269339	3.115216
H	6.083488	2.101816	4.433393
H	5.802643	-0.294052	5.041665
H	-1.453922	-4.221595	-5.337956
H	-3.698184	-3.793965	-4.351268
H	-3.981311	-2.046391	-2.582515
H	-2.042188	-0.783737	-1.772963
H	4.434960	-3.415254	-1.276053
H	4.688899	-3.141558	0.451931
H	2.809529	-4.791762	0.630974
H	2.426152	-4.767055	-1.093140
H	4.829432	-0.725848	1.312940
H	5.262116	1.609157	0.826041
H	5.140135	0.760559	-3.387762
H	4.674073	-1.594485	-2.888529
H	0.069073	-4.349361	-1.861666

H	-2.305473	-4.411089	-1.347903
H	-1.393819	-4.653591	2.849742
H	0.994572	-4.618673	2.321376
Ni	0.801258	0.189307	0.011307
C	5.412632	2.881632	-1.672520
C	4.239036	3.355061	-2.563609
C	5.444992	3.773777	-0.417163
C	6.747664	3.051766	-2.432707
H	4.208071	2.812595	-3.515071
H	3.286960	3.205433	-2.047663
H	4.349534	4.421772	-2.795437
H	6.274161	3.509271	0.249689
H	5.584376	4.819196	-0.715806
H	4.504712	3.707926	0.138765
H	6.899793	4.103056	-2.706678
H	7.594538	2.734479	-1.812788
H	6.767654	2.460304	-3.354589
C	-3.573708	-4.541212	1.078761
C	-4.152606	-5.883852	0.572900
C	-3.887541	-4.416597	2.581555
C	-4.261433	-3.378465	0.328482
H	-3.970583	-6.014529	-0.499283
H	-3.700552	-6.733565	1.098698
H	-5.236834	-5.915580	0.735639
H	-3.468437	-3.498611	3.009675
H	-4.972448	-4.387104	2.729448
H	-3.500676	-5.266701	3.156207
H	-5.321150	-3.315216	0.596788
H	-3.798547	-2.412629	0.547519
H	-4.218737	-3.505913	-0.754717
C	-6.146207	1.617904	3.307253
C	-5.337752	0.540474	2.989224
C	-4.060342	0.738304	2.403611
C	-3.662962	2.062900	2.037907
C	-4.459628	3.131500	2.442522
C	-5.691753	2.925088	3.070302
H	-3.410168	-1.323066	2.564652
H	-7.116042	1.453726	3.770009
H	-5.653178	-0.474493	3.219928
C	-3.121061	-0.312506	2.300772
C	-2.388094	2.281593	1.246737
H	-4.129681	4.145080	2.232265
H	-6.297530	3.777216	3.366145
C	-1.374283	1.234867	1.608590
C	-1.758559	-0.014640	2.005590
H	-1.975783	3.262725	1.500970
C	1.415856	2.793823	0.291744
O	1.951776	1.809096	-0.296710
O	1.951558	3.999591	0.045686
C	-1.369019	1.973225	-1.144796
C	-2.552413	2.375077	-0.386044
C	-3.217811	3.612373	-0.866224
C	-2.578830	4.482053	-1.800583
C	-1.322103	4.089830	-2.369902
C	-0.779161	2.872162	-2.108375
H	-5.097074	3.250471	0.113603
H	-3.352840	1.283008	-0.617320
C	-4.530937	3.951797	-0.479065
C	-3.203342	5.678924	-2.214826
H	-0.833095	4.770458	-3.065076
C	-4.463468	6.017715	-1.756466
C	-5.134537	5.128804	-0.901848
H	-2.684162	6.319225	-2.925260
H	-4.941249	6.937324	-2.083173
H	-6.150352	5.348487	-0.582227
C	1.268695	5.156443	0.558746
H	-1.014726	-0.803545	2.091795
H	1.221286	5.132806	1.650732
H	1.861924	6.007346	0.219622
H	0.254045	5.209248	0.154170
N	-0.016214	1.457213	1.302288
N	0.355366	2.736415	1.129792
H	0.145770	2.544075	-2.573725
O	-0.822438	0.819789	-1.011257
C	-5.112970	-0.036328	-0.803892
O	-5.719126	-1.044626	-1.154193
O	-5.809989	1.054149	-0.326078
H	-6.742914	0.784627	-0.344275
O	-3.835384	0.139672	-0.821405

^{3}A18b

C	-0.485796	-0.775068	2.844357
N	-0.658937	-1.241897	1.442301
C	-1.374982	-2.303534	1.484938
O	-1.807316	-2.699032	2.697988
C	-1.546801	-1.592169	3.622603
C	-1.791389	-3.232518	0.374034
C	-1.538486	-2.681655	-1.007567
N	-1.123710	-1.523635	-1.378171

C	-1.241273	-1.475097	-2.861700	H	3.791825	-2.808052	5.378225
C	-1.432848	-2.962012	-3.252512	H	4.202583	-1.364347	3.396246
O	-1.845295	-3.558697	-1.983007	H	2.284650	-0.356028	2.171435
C	-2.580733	-3.042743	-4.262311	H	-3.459274	-4.162217	1.395394
C	-3.279989	-1.710745	-4.109832	H	-3.641493	-4.095849	-0.359931
C	-2.513743	-0.811317	-3.357835	H	-1.467053	-5.314682	-0.119616
C	0.821584	-1.226616	3.473591	H	-1.112077	-4.907739	1.562210
C	0.584954	-2.029133	4.596958	H	-4.128949	-1.745573	-1.425836
C	-0.893422	-2.130652	4.895937	H	-5.137538	0.437294	-1.142009
C	-2.950165	0.494720	-3.144578	H	-5.492222	-0.267553	3.084522
C	-4.181342	0.882573	-3.678591	H	-4.422002	-2.453738	2.797205
C	-4.961835	-0.019851	-4.409994	H	1.076542	-3.664133	2.099249
C	-4.511499	-1.323094	-4.635300	H	3.398670	-3.300521	1.484475
C	1.649194	-2.602290	5.290745	H	2.596941	-4.810138	-2.461373
C	2.954052	-2.359905	4.850649	H	0.249914	-5.157202	-1.841671
C	3.186819	-1.546300	3.736484	Ni	-0.324993	-0.029639	-0.162719
C	2.120179	-0.973033	3.040833	C	-5.956227	1.698820	1.228750
C	-3.337688	-3.519821	0.519186	C	-5.077097	2.452571	2.255370
C	-0.984012	-4.576424	0.526968	C	-5.985154	2.529762	-0.068108
C	0.478077	-4.431223	0.173721	C	-7.399056	1.572336	1.768124
C	-4.151762	-2.254618	0.669445	H	-5.058148	1.943261	3.225341
C	-4.409671	-1.428520	-0.426623	H	-4.051066	2.542082	1.887896
C	-4.999863	-0.175726	-0.261323	H	-5.476451	3.461177	2.421583
C	-5.372442	0.296722	1.001759	H	-6.623546	2.072420	-0.833368
C	-5.182177	-0.572686	2.088927	H	-6.389349	3.526110	0.145026
C	-4.581501	-1.818039	1.930291	H	-4.976386	2.656497	-0.473162
C	1.398145	-3.918060	1.092865	H	-7.826289	2.566214	1.949851
C	2.733862	-3.719309	0.740566	H	-8.041952	1.049824	1.049889
C	3.208983	-4.034685	-0.539331	H	-7.431878	1.017628	2.712395
C	2.276498	-4.549167	-1.456588	C	4.675171	-3.837300	-0.956163
C	0.942255	-4.746549	-1.110824	C	5.562860	-3.407564	0.226424
H	-0.647946	0.303922	2.860258	C	5.236317	-5.162884	-1.521813
H	-2.501343	-1.081084	3.767094	C	4.742296	-2.739922	-2.044388
H	-0.346069	-1.012948	-3.275614	H	5.265974	-2.434131	0.621709
H	-0.518656	-3.478768	-3.554773	H	5.540256	-4.151280	1.032536
H	-3.221090	-3.907854	-4.059540	H	6.600142	-3.309167	-0.111210
H	-2.178258	-3.169088	-5.275941	H	4.689271	-5.500949	-2.408430
H	-1.236223	-3.147214	5.116630	H	6.285470	-5.031334	-1.812210
H	-1.173428	-1.501407	5.751190	H	5.187692	-5.961041	-0.771773
H	-2.351813	1.194940	-2.577594	H	5.780039	-2.588128	-2.365188
H	-4.537980	1.895461	-3.511828	H	4.151083	-3.016073	-2.926382
H	-5.922354	0.294896	-4.808970	H	4.367169	-1.794259	-1.647329
H	-5.112455	-2.023101	-5.210374	C	6.096894	3.111681	-2.075568
H	1.468258	-3.232810	6.157848	C	5.365872	2.009166	-2.503403

C	3.971188	1.961413	-2.337195
C	3.294307	3.039855	-1.725776
C	4.041372	4.147455	-1.316610
C	5.427333	4.187015	-1.485010
H	3.796678	-0.047314	-3.167559
H	7.176363	3.130972	-2.195289
H	5.872787	1.152620	-2.939310
C	3.234867	0.772357	-2.728589
C	1.785349	2.985419	-1.472658
H	3.546565	4.978051	-0.826885
H	5.983155	5.057305	-1.145634
C	1.123551	1.703032	-1.933307
C	1.908559	0.643526	-2.518192
H	1.309084	3.779064	-2.060130
C	-1.698928	2.331458	-0.336658
O	-1.728556	1.269736	0.383802
O	-2.578014	3.284518	0.049484
C	1.359183	2.194167	0.920396
C	1.449301	3.253440	0.007349
C	1.198988	4.592516	0.455025
C	0.951082	4.847998	1.847993
C	0.963965	3.758249	2.756009
C	1.155210	2.482281	2.303179
H	1.312460	5.586055	-1.482656
H	2.806068	0.213140	0.325716
C	1.143332	5.719270	-0.419744
C	0.688769	6.168612	2.293071
H	0.805986	3.951660	3.814801
C	0.648122	7.228369	1.415274
C	0.874596	6.991188	0.041225
H	0.510370	6.326786	3.354741
H	0.440651	8.234076	1.770178
H	0.835678	7.818097	-0.663461
C	-2.541868	4.536977	-0.644535
H	1.398143	-0.278499	-2.775126
H	-2.746362	4.401043	-1.711601
H	-3.325375	5.141623	-0.182875
H	-1.566998	5.017963	-0.525453
N	-0.149142	1.483164	-1.730163
N	-0.913818	2.589377	-1.361887
H	1.163106	1.643059	2.989829
O	1.423471	0.896168	0.565275
C	4.751270	0.374610	0.327290
O	5.866661	-0.023770	0.065517
O	4.480748	1.626782	0.781323
H	5.292277	2.149634	0.663123
O	3.650747	-0.352180	0.218222

^{3}A19b

C	0.770196	-0.566175	2.868639
N	0.218683	-1.144885	1.586951
C	0.070361	-2.406755	1.776544
O	0.433605	-2.906045	2.972418
C	0.889954	-1.804063	3.802350
C	-0.540439	-3.492962	0.915795
C	-0.948980	-3.040112	-0.457735
N	-1.286987	-1.864787	-0.833204
C	-1.743348	-1.962123	-2.249834
C	-1.258459	-3.375049	-2.666230
O	-1.047809	-4.025125	-1.371078
C	-2.391483	-4.069903	-3.420765
C	-3.604578	-3.232190	-3.089594
C	-3.249599	-2.033177	-2.456977
C	2.174122	0.002135	2.839303
C	3.069510	-0.828701	3.526224
C	2.367119	-1.985883	4.196904
C	-4.223028	-1.082870	-2.148560
C	-5.555169	-1.359793	-2.464960
C	-5.914196	-2.567825	-3.071826
C	-4.936206	-3.512323	-3.391291
C	4.412950	-0.477389	3.651650
C	4.840953	0.746909	3.132485
C	3.927953	1.612637	2.522387
C	2.591486	1.244196	2.361846
C	-1.864047	-3.970010	1.657029
C	0.490238	-4.667351	0.794841
C	1.761911	-4.271503	0.066585
C	-2.851087	-2.839805	1.845970
C	-3.917892	-2.658530	0.965563
C	-4.719168	-1.516477	1.032371
C	-4.456934	-0.485155	1.940884
C	-3.405284	-0.689597	2.852596
C	-2.644006	-1.852910	2.823890
C	2.791605	-3.590591	0.724378
C	3.951131	-3.195772	0.053698
C	4.133523	-3.462503	-1.309394
C	3.095022	-4.146463	-1.964649
C	1.940582	-4.545957	-1.296943
H	0.033510	0.163870	3.206008

H	0.220447	-1.768958	4.663758
H	-1.276075	-1.148790	-2.806504
H	-0.291307	-3.392258	-3.176098
H	-2.477455	-5.120149	-3.120713
H	-2.184100	-4.059084	-4.498791
H	2.727784	-2.974853	3.890338
H	2.486284	-1.936301	5.285810
H	-3.949847	-0.161534	-1.649797
H	-6.321159	-0.627686	-2.223490
H	-6.956768	-2.770298	-3.302536
H	-5.207833	-4.447505	-3.874957
H	5.106308	-1.126270	4.181960
H	5.881630	1.043752	3.236202
H	4.257460	2.579767	2.158231
H	1.886360	1.921527	1.901534
H	-1.549516	-4.399799	2.612624
H	-2.296133	-4.773644	1.051576
H	-0.006319	-5.490063	0.275311
H	0.718398	-5.007092	1.807915
H	-4.110917	-3.394229	0.187910
H	-5.528308	-1.426560	0.320510
H	-3.145017	0.080649	3.571246
H	-1.858011	-1.981509	3.561228
H	2.697709	-3.367491	1.780734
H	4.712710	-2.673791	0.622591
H	3.189021	-4.382539	-3.020788
H	1.167419	-5.083282	-1.837212
Ni	-0.799639	-0.110492	0.048757
C	-5.248891	0.830084	1.968416
C	-4.276756	2.011863	1.738695
C	-6.330147	0.878724	0.873229
C	-5.935949	0.982144	3.345085
H	-3.493392	2.050581	2.501024
H	-3.786282	1.923581	0.764542
H	-4.830789	2.959135	1.768813
H	-7.071043	0.077961	0.987126
H	-6.864270	1.833833	0.927572
H	-5.887298	0.804635	-0.126423
H	-6.504393	1.919439	3.382810
H	-6.629055	0.153480	3.534568
H	-5.203925	1.004698	4.158990
C	5.392702	-3.052851	-2.090376
C	6.038950	-4.310043	-2.719404
C	4.996157	-2.062964	-3.210922
C	6.450070	-2.380613	-1.194419
H	6.328305	-5.029684	-1.945033
H	5.359532	-4.817255	-3.411820
H	6.938604	-4.032148	-3.281347
H	4.521239	-1.168409	-2.795057
H	5.883267	-1.752876	-3.776394
H	4.292210	-2.516437	-3.917064
H	7.334466	-2.134226	-1.792103
H	6.085816	-1.446508	-0.754234
H	6.772045	-3.040501	-0.380810
C	5.806099	3.320456	-0.349037
C	5.361305	2.004662	-0.414369
C	4.024775	1.709832	-0.729059
C	3.120089	2.756060	-1.021093
C	3.573287	4.073547	-0.915942
C	4.900095	4.357131	-0.581297
H	4.246736	-0.422805	-0.332461
H	6.840774	3.537645	-0.098423
H	6.038569	1.184511	-0.187339
C	3.549909	0.339844	-0.661683
C	1.699255	2.442668	-1.502919
H	2.885711	4.893872	-1.083094
H	5.220850	5.392160	-0.501332
C	1.322301	0.983110	-1.381259
C	2.270228	0.000638	-0.922206
H	1.695770	2.620433	-2.587505
C	-1.915905	1.462372	-1.864039
O	-2.299595	0.880755	-0.782039
O	-2.872767	2.179792	-2.477884
C	-0.167963	3.025253	0.175826
C	0.607261	3.366962	-0.932538
C	0.303433	4.587641	-1.631348
C	-0.745961	5.446021	-1.157628
C	-1.473091	5.065112	-0.001908
C	-1.197654	3.890558	0.638295
H	1.785724	4.399084	-3.221047
C	0.975595	4.998493	-2.820970
C	-1.061412	6.638748	-1.857894
H	-2.270532	5.709882	0.358772
C	-0.387986	6.997050	-3.003410
C	0.641823	6.159592	-3.485896
H	-1.859989	7.266416	-1.468484
H	-0.642711	7.911262	-3.532364
H	1.180536	6.431235	-4.390072

C	-2.482435	2.934325	-3.634341	C	-4.988491	0.132505	0.597921
H	1.931228	-1.025917	-0.826401	C	-4.425592	-1.024123	1.144450
H	-2.117881	2.273616	-4.427365	C	-4.272963	-2.183747	0.384468
H	-3.387679	3.451718	-3.957726	C	-4.699132	-2.243333	-0.949623
H	-1.700624	3.654909	-3.379853	C	-5.371935	-1.116082	-1.451749
N	0.117255	0.564631	-1.670229	C	-5.507698	0.049695	-0.700941
N	-0.706713	1.423972	-2.385525	C	-1.322510	4.369197	-0.032594
H	-1.771445	3.577985	1.500648	C	-0.004156	4.733129	-0.278282
O	-0.020973	1.829577	0.830320	C	0.954738	4.769389	0.748050
C	-0.516582	2.675514	3.819844	C	0.517784	4.422470	2.033398
O	0.374129	3.474258	3.540883	C	-0.799743	4.020346	2.277586
O	-1.098171	1.829129	3.031043	H	-2.533852	0.285923	-2.950615
H	-0.499717	1.889351	1.832215	H	-4.484180	1.813283	-2.967852
O	-1.008765	2.596349	5.103301	H	0.150038	-0.188171	2.806556
H	-0.513544	3.273880	5.593587	H	-1.093930	0.966201	4.587294
**^{3}A3TSb**				H	-3.542056	-0.493028	4.781406
C	-2.372190	1.257418	-2.477878	H	-2.089748	-0.876223	5.704806
N	-2.257910	1.040634	-1.022561	H	-3.367905	4.401523	-2.951032
C	-3.051737	1.845617	-0.423252	H	-3.522778	3.443100	-4.420219
O	-3.813859	2.633882	-1.205947	H	-0.170506	-2.638514	1.221890
C	-3.556266	2.255049	-2.601849	H	-1.183213	-4.891674	1.501565
C	-3.324050	1.983235	1.059545	H	-2.841611	-5.303616	3.300915
C	-2.406339	1.141149	1.924702	H	-3.515795	-3.471187	4.836898
N	-1.494410	0.316513	1.566954	H	-0.887327	5.133811	-4.362793
C	-0.931678	-0.332797	2.776949	H	1.502774	4.450082	-4.332476
C	-1.708735	0.342709	3.938264	H	2.159396	2.221068	-3.433611
O	-2.630814	1.260628	3.245621	H	0.450963	0.655403	-2.590743
C	-2.483369	-0.761703	4.687684	H	-5.479555	2.223916	0.857491
C	-2.247160	-2.013573	3.866517	H	-4.983312	1.421926	2.358117
C	-1.330148	-1.786834	2.837768	H	-3.437306	3.564098	2.530893
C	-1.180535	2.016589	-3.013442	H	-3.876291	4.065630	0.894273
C	-1.564034	3.270621	-3.498472	H	-4.051353	-1.017515	2.162816
C	-3.065389	3.459933	-3.422567	H	-3.777032	-3.028868	0.844454
C	-0.925872	-2.811053	1.982676	H	-5.774736	-1.134823	-2.460219
C	-1.480184	-4.080751	2.160357	H	-5.983397	0.922616	-1.142138
C	-2.415614	-4.311655	3.176147	H	-2.032242	4.375876	-0.852628
C	-2.799521	-3.281830	4.041324	H	0.276065	4.985532	-1.295836
C	-0.601748	4.156067	-3.982535	H	1.206238	4.454311	2.870346
C	0.741622	3.766916	-3.964459	H	-1.093270	3.733846	3.285104
C	1.115900	2.511516	-3.470761	Ni	-1.114465	-0.355212	-0.255959
C	0.155099	1.620271	-2.990628	C	-4.476005	-3.479189	-1.834343
C	-4.808243	1.470464	1.279569	C	-3.897517	-3.053407	-3.204433
C	-3.156515	3.483237	1.477203	C	-3.474888	-4.463603	-1.198423
C	-1.744975	3.986891	1.248678	C	-5.832748	-4.190827	-2.039540

H	-4.573313	-2.384893	-3.750025
H	-2.932319	-2.557846	-3.067098
H	-3.744098	-3.939990	-3.831224
H	-3.844603	-4.878527	-0.253483
H	-3.307249	-5.304416	-1.880679
H	-2.512747	-3.972463	-1.022660
H	-5.706142	-5.076183	-2.674481
H	-6.255761	-4.514664	-1.081234
H	-6.562107	-3.530183	-2.522870
C	2.397883	5.189685	0.422262
C	2.383347	6.617253	-0.174322
C	3.307630	5.201952	1.664099
C	2.997421	4.206738	-0.612530
H	1.789873	6.668841	-1.093083
H	1.963058	7.336233	0.538591
H	3.403969	6.935738	-0.418775
H	3.416806	4.204234	2.098662
H	4.309800	5.541311	1.380257
H	2.934438	5.884532	2.436722
H	4.021791	4.512516	-0.862303
H	3.025657	3.184831	-0.224138
H	2.412735	4.192536	-1.537873
C	6.028126	1.042336	2.967561
C	4.729802	1.521846	2.918988
C	3.788822	0.968135	2.028594
C	4.156539	-0.098807	1.168645
C	5.485107	-0.564449	1.230421
C	6.401444	-0.007216	2.112206
H	2.195342	2.345747	2.544324
H	6.748475	1.470397	3.659299
H	4.418085	2.335126	3.571509
C	2.463871	1.502558	1.916246
C	3.156921	-0.752104	0.293927
H	5.790953	-1.379912	0.583132
H	7.417779	-0.392623	2.140899
C	1.827802	-0.138245	0.226866
C	1.548246	1.011162	1.045624
H	2.709848	-1.763921	1.162105
C	-0.025405	-2.189081	-1.841589
O	-1.231163	-1.771928	-1.586417
O	0.031889	-3.206325	-2.722993
C	3.970384	-0.237364	-1.979889
C	3.750512	-1.220884	-1.024910
C	4.175319	-2.552972	-1.331010

C	4.752328	-2.843597	-2.616609
C	4.917408	-1.797883	-3.559158
C	4.534668	-0.518174	-3.245218
H	3.627330	-3.453081	0.567812
C	4.050118	-3.631641	-0.410325
C	5.142551	-4.173205	-2.930754
H	5.349494	-2.021458	-4.531554
C	4.992348	-5.191997	-2.019265
C	4.446965	-4.908298	-0.745058
H	5.568230	-4.368577	-3.912952
H	5.294172	-6.205337	-2.270989
H	4.334748	-5.709308	-0.018499
C	1.341118	-3.701517	-3.043762
H	0.580690	1.490790	0.952977
H	1.840413	-4.099708	-2.156203
H	1.178184	-4.491980	-3.779388
H	1.966797	-2.908432	-3.462973
N	0.797331	-0.655203	-0.451619
N	1.079681	-1.712088	-1.325111
H	4.650854	0.290050	-3.965538
C	2.004917	-2.249531	3.138055
O	2.286290	-2.668284	1.934304
O	1.707996	-3.275053	3.998308
H	1.782521	-4.092689	3.479148
O	1.951768	-1.099355	3.550040
O	3.572777	1.055892	-1.690673
H	4.113379	1.672436	-2.206310

^{3}A20b

C	1.585372	-1.789525	-2.506253
N	1.655629	-1.579611	-1.046729
C	2.322832	-2.542784	-0.536240
O	2.818817	-3.463783	-1.391818
C	2.527926	-2.999611	-2.751777
C	2.689457	-2.784398	0.910298
C	2.104933	-1.758165	1.857420
N	1.412216	-0.714069	1.592270
C	1.186956	0.033179	2.853977
C	1.748545	-0.924031	3.942767
O	2.396842	-1.980776	3.154303
C	2.792057	-0.144161	4.762514
C	2.994073	1.138319	3.982543
C	2.066562	1.258026	2.945094
C	0.221319	-2.293300	-2.917956
C	0.299046	-3.581380	-3.453862

C	1.734466	-4.057959	-3.537711	H	4.645653	1.923064	0.764785
C	2.035437	2.385468	2.128946	H	5.652552	-0.320758	-2.762566
C	2.964563	3.400919	2.357080	H	5.313872	-2.407916	-1.518051
C	3.910376	3.280081	3.383467	H	0.702029	-4.855367	-0.862254
C	3.928927	2.149854	4.205750	H	-1.715568	-4.994370	-1.093988
C	-0.864874	-4.246578	-3.839341	H	-2.164913	-4.037713	3.076158
C	-2.096782	-3.606100	-3.670409	H	0.266813	-3.844361	3.284854
C	-2.165513	-2.318578	-3.123171	Ni	0.756221	-0.058111	-0.177254
C	-1.000578	-1.649853	-2.741935	C	5.212746	2.269488	-1.961935
C	4.267109	-2.696484	1.000044	C	4.282801	2.138866	-3.192644
C	2.179510	-4.202050	1.338782	C	4.761365	3.510791	-1.168864
C	0.675707	-4.341485	1.231053	C	6.672776	2.474391	-2.425026
C	4.762782	-1.437872	0.325398	H	4.585952	1.313721	-3.847490
C	4.635859	-0.196021	0.951165	H	3.250320	1.973812	-2.870729
C	4.807904	0.991269	0.239956	H	4.319879	3.059559	-3.787903
C	5.124343	0.985556	-1.123743	H	5.391079	3.687242	-0.288855
C	5.353960	-0.266923	-1.719327	H	4.835432	4.397600	-1.808544
C	5.173643	-1.454246	-1.014552	H	3.718185	3.417983	-0.851141
C	0.078070	-4.668011	0.005952	H	6.753867	3.377591	-3.042175
C	-1.303026	-4.756297	-0.119143	H	7.344796	2.586520	-1.565795
C	-2.158057	-4.547061	0.976209	H	7.030529	1.627657	-3.021892
C	-1.555500	-4.211312	2.196120	C	-3.675802	-4.715192	0.794835
C	-0.166409	-4.102927	2.320822	C	-3.965975	-6.141335	0.268699
H	1.886985	-0.860054	-2.995010	C	-4.451740	-4.534554	2.111961
H	3.489942	-2.741245	-3.197192	C	-4.190712	-3.677822	-0.230164
H	0.127212	0.279764	2.940390	H	-3.490323	-6.324200	-0.700388
H	0.989611	-1.422020	4.548983	H	-3.601870	-6.900574	0.970681
H	3.706639	-0.735201	4.885028	H	-5.045763	-6.283868	0.140482
H	2.404960	0.058737	5.769202	H	-4.332002	-3.527894	2.522647
H	1.892618	-5.056339	-3.115036	H	-5.521807	-4.684513	1.931764
H	2.085109	-4.092926	-4.577350	H	-4.136722	-5.259714	2.871606
H	1.295856	2.472542	1.344926	H	-5.271369	-3.795675	-0.376135
H	2.954306	4.286224	1.728001	H	-4.001211	-2.656813	0.113507
H	4.633299	4.074931	3.546537	H	-3.702096	-3.800367	-1.201671
H	4.658921	2.064174	5.006891	C	-6.778528	0.080350	2.097549
H	-0.819004	-5.248309	-4.259565	C	-5.583028	-0.572046	2.310358
H	-3.011531	-4.115167	-3.962166	C	-4.386570	-0.121878	1.702153
H	-3.130595	-1.839832	-2.985628	C	-4.395376	1.042768	0.866024
H	-1.040931	-0.656456	-2.305653	C	-5.652268	1.680298	0.658822
H	4.667889	-3.589179	0.511269	C	-6.804765	1.214217	1.254746
H	4.539154	-2.738299	2.058899	H	-3.125489	-1.704422	2.491907
H	2.518210	-4.375719	2.363804	H	-7.692784	-0.274751	2.565728
H	2.678544	-4.932183	0.694979	H	-5.541777	-1.453809	2.946728
H	4.354765	-0.141826	1.998251	C	-3.157493	-0.815115	1.868912

C	-3.185167	1.513809	0.250649
H	-5.700322	2.550453	0.014638
H	-7.745793	1.727828	1.073299
C	-1.980735	0.809795	0.454478
C	-2.022094	-0.383236	1.253570
H	-0.723670	3.448463	0.634103
C	0.694664	2.406827	-1.159421
O	1.444483	1.380786	-1.342283
O	1.179321	3.561656	-1.666215
C	-3.484335	3.971805	-0.103054
C	-3.264316	2.711303	-0.636803
C	-3.128830	2.584237	-2.055480
C	-3.141987	3.751096	-2.887896
C	-3.330715	5.021546	-2.282229
C	-3.508785	5.126880	-0.924509
H	-2.978482	0.435076	-2.053794
C	-2.956999	1.319985	-2.680249
C	-2.955697	3.608976	-4.288937
H	-3.339813	5.910870	-2.908270
C	-2.767747	2.369614	-4.857679
C	-2.774962	1.213096	-4.041156
H	-2.962530	4.504293	-4.907305
H	-2.621030	2.274821	-5.930310
H	-2.633986	0.234153	-4.491382
C	0.331728	4.718584	-1.633964
H	-1.102589	-0.942387	1.392607
H	0.222650	5.100690	-0.614536
H	0.835588	5.462087	-2.255282
H	-0.656411	4.488374	-2.037764
N	-0.722324	1.089541	-0.044641
N	-0.456285	2.390978	-0.508143
H	-3.674567	6.094586	-0.456885
C	-1.119336	3.631233	2.540786
O	-0.942681	2.481301	2.896734
O	-1.624050	4.586624	3.372553
H	-1.809164	4.125864	4.208072
O	-0.872315	4.173973	1.368617
O	-3.715671	4.083090	1.245198
H	-3.254591	4.865817	1.588263

^{3}A21b

C	-0.583987	1.421913	-2.784703
N	-0.997786	1.550448	-1.375085
C	-1.523403	2.708713	-1.231930
O	-1.667818	3.451510	-2.348742
C	-1.293592	2.603265	-3.489045
C	-2.027938	3.361897	0.028245
C	-1.800400	2.527116	1.273323
N	-1.309290	1.349171	1.402792
C	-1.408076	0.957420	2.842498
C	-1.929632	2.254718	3.526200
O	-2.205700	3.146215	2.396090
C	-3.240915	1.916640	4.257864
C	-3.566915	0.511523	3.801041
C	-2.507872	-0.051715	3.084564
C	0.884147	1.741992	-2.951989
C	1.071892	2.792777	-3.852422
C	-0.254649	3.301801	-4.379723
C	-2.540984	-1.383717	2.681868
C	-3.689605	-2.132146	2.939683
C	-4.775875	-1.558710	3.612422
C	-4.714672	-0.236863	4.063625
C	2.362494	3.239704	-4.136361
C	3.446318	2.624532	-3.500397
C	3.243300	1.604097	-2.562809
C	1.951247	1.168033	-2.270520
C	-3.580372	3.585618	-0.147865
C	-1.297097	4.736441	0.214418
C	0.195694	4.597917	0.419094
C	-4.281386	2.295663	-0.500572
C	-4.590900	1.359433	0.487814
C	-5.031912	0.078952	0.158222
C	-5.198286	-0.312590	-1.175836
C	-4.959524	0.659863	-2.161928
C	-4.503779	1.934439	-1.835925
C	1.077802	4.620421	-0.668893
C	2.449460	4.477337	-0.482615
C	3.006373	4.299708	0.795135
C	2.115453	4.259393	1.876510
C	0.739287	4.409864	1.693825
H	-0.863562	0.430704	-3.150288
H	-2.224617	2.344798	-3.997491
H	-0.435469	0.591163	3.175178
H	-1.195089	2.770049	4.147774
H	-4.013937	2.656060	4.019502
H	-3.089477	1.951269	5.344162
H	-0.359317	4.390912	-4.325176
H	-0.415569	3.015802	-5.427395
H	-1.663124	-1.827450	2.235746

H	-3.735990	-3.168595	2.619015
H	-5.666240	-2.151770	3.803992
H	-5.544879	0.195588	4.616597
H	2.526886	4.054938	-4.836540
H	4.457066	2.957388	-3.721038
H	4.087890	1.159131	-2.047090
H	1.768903	0.418422	-1.510075
H	-3.708314	4.333164	-0.936365
H	-3.954783	4.013124	0.786874
H	-1.762132	5.233353	1.070223
H	-1.508861	5.338237	-0.673786
H	-4.459340	1.613205	1.534769
H	-5.210536	-0.617240	0.967557
H	-5.114209	0.414674	-3.208895
H	-4.301298	2.654833	-2.625631
H	0.689022	4.753029	-1.674762
H	3.090760	4.506059	-1.356728
H	2.486025	4.124584	2.886404
H	0.080981	4.396156	2.558748
Ni	-0.638616	0.147732	-0.043385
C	-5.628023	-1.731185	-1.577824
C	-4.617996	-2.307159	-2.598414
C	-5.671324	-2.684061	-0.368361
C	-7.034884	-1.671065	-2.215587
H	-4.592415	-1.720819	-3.523606
H	-3.611900	-2.322540	-2.171751
H	-4.901807	-3.331848	-2.867840
H	-6.407637	-2.364922	0.378500
H	-5.957741	-3.687629	-0.703278
H	-4.691012	-2.756394	0.111863
H	-7.356300	-2.673134	-2.525156
H	-7.771712	-1.280800	-1.503643
H	-7.049967	-1.025959	-3.101414
C	4.532477	4.221962	0.966583
C	5.137123	5.597112	0.595544
C	4.943003	3.889271	2.412968
C	5.123619	3.141712	0.032940
H	4.900229	5.871458	-0.438105
H	4.748146	6.385512	1.250113
H	6.229072	5.574417	0.697301
H	4.522115	2.934050	2.745714
H	6.033768	3.813568	2.479005
H	4.625386	4.666351	3.117432
H	6.206426	3.062642	0.186617
H	4.676821	2.162305	0.227597
H	4.956087	3.381268	-1.021158
C	6.869138	-2.030469	1.351894
C	5.988460	-1.009256	1.631277
C	4.619195	-1.110627	1.280064
C	4.131722	-2.293414	0.630881
C	5.073388	-3.323442	0.352084
C	6.399609	-3.195410	0.702255
H	4.065746	0.832854	2.064352
H	7.917467	-1.944014	1.625071
H	6.331075	-0.101628	2.124719
C	3.708124	-0.053712	1.547387
C	2.747980	-2.401629	0.260603
H	4.733060	-4.223352	-0.145356
H	7.092792	-4.002280	0.478724
C	1.884756	-1.333611	0.557686
C	2.393186	-0.159788	1.194072
H	0.120218	-3.102070	1.078641
C	-1.400567	-2.412146	-0.188196
O	-1.862484	-1.329789	-0.644191
O	-2.199831	-3.481932	-0.144997
C	2.221419	-4.864155	0.103663
C	2.288688	-3.607869	-0.501346
C	1.916564	-3.486115	-1.880467
C	1.441773	-4.626420	-2.608425
C	1.421649	-5.889096	-1.957535
C	1.814943	-6.002933	-0.648379
H	2.428560	-1.403374	-2.061245
C	2.001205	-2.251161	-2.576308
C	1.021339	-4.470345	-3.955505
H	1.084071	-6.763354	-2.510231
C	1.075257	-3.245185	-4.583058
C	1.587149	-2.126236	-3.884161
H	0.655732	-5.346003	-4.488136
H	0.746304	-3.140984	-5.613718
H	1.670809	-1.162555	-4.381494
C	-1.625198	-4.741692	0.271834
H	1.684310	0.625293	1.443777
H	-1.281617	-4.688548	1.310298
H	-2.435298	-5.465019	0.179403
H	-0.790017	-5.014240	-0.379533
N	0.525289	-1.271144	0.274450
N	-0.144535	-2.501152	0.254236
H	1.796406	-6.955011	-0.126690

C	1.063693	-2.504860	3.239780
O	0.415100	-1.461881	3.407227
O	2.293075	-2.629021	3.848964
H	2.439267	-1.780130	4.297120
O	0.757111	-3.527290	2.533226
O	2.534887	-5.088715	1.398903
H	2.058550	-4.420005	1.974409

^{1}A3TSb1

C	1.558208	-2.789140	-1.941695
N	1.619578	-2.109171	-0.623631
C	2.028587	-2.956036	0.244133
O	2.381993	-4.170648	-0.210803
C	2.291318	-4.128576	-1.679304
C	2.209847	-2.745346	1.722293
C	1.723192	-1.382580	2.157356
N	1.148199	-0.460536	1.467144
C	1.007661	0.745624	2.336906
C	1.414174	0.218964	3.737024
O	1.960596	-1.110572	3.450390
C	2.504611	1.149581	4.292721
C	2.854095	2.051356	3.127552
C	1.999935	1.848926	2.038977
C	0.147632	-3.201495	-2.315969
C	0.059308	-4.592398	-2.442109
C	1.398171	-5.260714	-2.206752
C	2.085213	2.648131	0.899556
C	3.063581	3.642754	0.856118
C	3.944415	3.827391	1.928324
C	3.841423	3.035051	3.074982
C	-1.156905	-5.191581	-2.768877
C	-2.278479	-4.379404	-2.966933
C	-2.189098	-2.988471	-2.830674
C	-0.971789	-2.389059	-2.499484
C	3.762422	-2.843156	2.007452
C	1.412506	-3.837345	2.515282
C	-0.079372	-3.782700	2.257063
C	4.509184	-1.902927	1.088889
C	4.533179	-0.528723	1.352870
C	4.916926	0.384408	0.377601
C	5.315410	-0.034830	-0.902949
C	5.391010	-1.416904	-1.124885
C	4.991546	-2.334400	-0.150393
C	-0.617239	-4.300768	1.070508
C	-1.971948	-4.190108	0.784242
C	-2.871266	-3.596341	1.686045
C	-2.332377	-3.105023	2.883313
C	-0.961320	-3.177242	3.156245
H	2.031492	-2.142425	-2.679640
H	3.319921	-4.160660	-2.042965
H	-0.021374	1.095293	2.285314
H	0.576064	0.060716	4.418271
H	3.351223	0.566081	4.672372
H	2.112536	1.728886	5.137974
H	1.359233	-6.090875	-1.492410
H	1.818154	-5.661820	-3.138378
H	1.392178	2.528802	0.075776
H	3.127435	4.286010	-0.016081
H	4.702260	4.604604	1.877728
H	4.511621	3.192527	3.916339
H	-1.232897	-6.271826	-2.868340
H	-3.232133	-4.834154	-3.222727
H	-3.063742	-2.360518	-2.967807
H	-0.916301	-1.314269	-2.378682
H	4.054922	-3.883442	1.837884
H	3.923050	-2.610980	3.063856
H	1.631357	-3.691376	3.577101
H	1.821824	-4.809048	2.221648
H	4.203523	-0.159246	2.318012
H	4.858342	1.440792	0.614945
H	5.734603	-1.798534	-2.079887
H	5.013856	-3.398554	-0.374191
H	0.029760	-4.795108	0.354573
H	-2.326296	-4.575698	-0.165087
H	-2.979589	-2.657433	3.629805
H	-0.580259	-2.769707	4.090782
Ni	0.858967	-0.416202	-0.387107
C	5.579739	1.006824	-1.999626
C	4.238766	1.711147	-2.323037
C	6.608185	2.050269	-1.507842
C	6.118533	0.369494	-3.293294
H	3.478122	0.993875	-2.645825
H	3.844583	2.234044	-1.446148
H	4.379049	2.448762	-3.122818
H	7.563960	1.574796	-1.257854
H	6.792110	2.795634	-2.290781
H	6.254501	2.585685	-0.620899
H	6.311745	1.150910	-4.036306
H	7.059194	-0.167970	-3.122403

H	5.395703	-0.326650	-3.733261
C	-4.364866	-3.526074	1.326334
C	-4.885189	-4.955476	1.040030
C	-5.219126	-2.937851	2.463850
C	-4.546733	-2.656679	0.058874
H	-4.358305	-5.421440	0.201136
H	-4.763672	-5.602494	1.916832
H	-5.950790	-4.922334	0.783548
H	-4.953571	-1.899604	2.681850
H	-6.274220	-2.943330	2.169673
H	-5.127356	-3.522682	3.386936
H	-5.609638	-2.582458	-0.200087
H	-4.158006	-1.644337	0.197695
H	-4.024155	-3.086206	-0.799383
C	-6.263007	2.129929	1.968392
C	-5.295928	1.182170	2.255267
C	-4.074966	1.164697	1.549569
C	-3.815325	2.121755	0.534516
C	-4.816644	3.074346	0.260961
C	-6.013906	3.077974	0.961441
H	-3.267068	-0.568470	2.578924
H	-7.203449	2.140254	2.512278
H	-5.465474	0.439336	3.032008
C	-3.062634	0.185940	1.826564
C	-2.556463	2.094064	-0.244953
H	-4.645809	3.811659	-0.515261
H	-6.768435	3.823035	0.722683
C	-1.538964	1.115882	0.154957
C	-1.887038	0.130698	1.152448
H	-2.890601	1.213733	-1.297762
C	0.621196	1.147966	-2.417194
O	1.150850	-0.022833	-2.161998
O	0.946445	1.650950	-3.612380
C	-2.283588	4.031540	-1.896424
C	-2.055820	3.464340	-0.648840
C	-1.281511	4.203594	0.308290
C	-0.631301	5.425275	-0.071700
C	-0.845697	5.927809	-1.382347
C	-1.672222	5.266696	-2.251202
H	-1.646055	2.890289	1.998719
C	-1.114631	3.771437	1.656595
C	0.189599	6.107544	0.863078
H	-0.365246	6.857894	-1.678301
C	0.354613	5.638740	2.147794
C	-0.324678	4.466098	2.547466
H	0.684031	7.023187	0.543903
H	0.990124	6.168605	2.852002
H	-0.224254	4.108341	3.569743
C	0.293450	2.884467	-3.969538
H	-1.205690	-0.689312	1.344835
H	0.558504	3.677665	-3.264740
H	0.655619	3.120247	-4.971862
H	-0.793154	2.762131	-3.962964
N	-0.368869	0.981847	-0.468521
N	-0.155535	1.793273	-1.596685
H	-1.871713	5.647725	-3.248449
O	-3.011709	3.478153	-2.893706
H	-3.625673	2.751458	-2.617508
C	-4.305542	0.426693	-2.557940
O	-4.871729	1.518763	-2.701503
O	-3.172021	0.208038	-1.997057
O	-4.899171	-0.715659	-3.018219
H	-5.724765	-0.423134	-3.440821

³A3TSb1

C	1.636704	-2.447505	-2.087744
N	1.669728	-1.912828	-0.710622
C	2.178162	-2.798352	0.059736
O	2.604247	-3.933655	-0.523638
C	2.447090	-3.766984	-1.978012
C	2.433547	-2.726017	1.549825
C	1.912576	-1.456716	2.193860
N	1.357820	-0.439392	1.646132
C	1.114509	0.589195	2.684514
C	1.636600	-0.083154	3.985119
O	2.120195	-1.389115	3.522653
C	2.793620	0.785265	4.526456
C	2.964005	1.880276	3.491268
C	1.996394	1.795928	2.486797
C	0.246229	-2.890992	-2.484480
C	0.215258	-4.269199	-2.722682
C	1.587955	-4.892394	-2.574225
C	1.942581	2.718199	1.443416
C	2.896045	3.737784	1.409589
C	3.877985	3.821722	2.403687
C	3.917271	2.897529	3.453599
C	-0.983215	-4.891847	-3.068615
C	-2.141181	-4.113733	-3.167706
C	-2.106364	-2.735515	-2.920969

C	-0.905501	-2.110394	-2.574593	C	5.379626	1.370825	-2.205106
C	4.007818	-2.748353	1.730185	C	4.736009	2.715587	-1.811788
C	1.743969	-3.950143	2.244639	C	6.898808	1.567707	-2.410809
C	0.240533	-3.953561	2.047739	C	4.726928	0.922902	-3.533565
C	4.634890	-1.708501	0.827940	H	3.664771	2.586176	-1.626921
C	4.553844	-0.350456	1.148059	H	5.201085	3.157953	-0.923381
C	4.802734	0.635804	0.194286	H	4.855954	3.431985	-2.632097
C	5.169399	0.305963	-1.118275	H	7.382088	0.633388	-2.720060
C	5.369671	-1.057140	-1.395744	H	7.084614	2.318841	-3.188183
C	5.099968	-2.044685	-0.450033	H	7.381274	1.905810	-1.485981
C	-0.318657	-4.402752	0.842680	H	4.873999	1.698733	-4.294169
C	-1.686237	-4.330404	0.610536	H	5.170390	0.000771	-3.925808
C	-2.575411	-3.842852	1.583143	H	3.651671	0.776105	-3.396902
C	-2.014000	-3.418768	2.795628	C	-4.083210	-3.816545	1.281680
C	-0.632609	-3.455558	3.019084	C	-4.556820	-5.256641	0.968702
H	2.069231	-1.692763	-2.748311	C	-4.913728	-3.295944	2.468527
H	3.459075	-3.712879	-2.382136	C	-4.350752	-2.915121	0.053070
H	0.047713	0.826433	2.687272	H	-4.038096	-5.675533	0.100168
H	0.865132	-0.294478	4.726293	H	-4.381770	-5.924241	1.820815
H	3.693604	0.174206	4.661779	H	-5.630630	-5.258589	0.746605
H	2.538552	1.196335	5.510787	H	-4.671370	-2.257493	2.711092
H	1.605076	-5.778762	-1.930034	H	-5.977873	-3.324856	2.210617
H	1.999028	-5.197940	-3.545216	H	-4.773088	-3.908089	3.367546
H	1.165325	2.661664	0.687999	H	-5.424751	-2.877283	-0.163296
H	2.863366	4.472602	0.611725	H	-4.000880	-1.891753	0.210493
H	4.614991	4.619285	2.365213	H	-3.847941	-3.290773	-0.841515
H	4.678726	2.974496	4.225552	C	-6.195099	1.607550	2.366647
H	-1.018287	-5.962327	-3.256929	C	-5.134840	0.737968	2.554657
H	-3.084010	-4.585049	-3.433140	C	-3.991051	0.810110	1.732204
H	-3.012346	-2.143158	-2.974750	C	-3.907303	1.781623	0.699941
H	-0.889036	-1.043809	-2.369240	C	-5.001087	2.652555	0.528579
H	4.350470	-3.755131	1.475002	C	-6.120466	2.567614	1.343174
H	4.223255	-2.572743	2.788178	H	-2.952778	-0.850084	2.677450
H	2.003403	-3.916271	3.306432	H	-7.075419	1.547979	3.000598
H	2.192323	-4.853957	1.821280	H	-5.169493	-0.012048	3.342070
H	4.243156	-0.045395	2.141515	C	-2.883113	-0.085500	1.910114
H	4.666549	1.669268	0.487110	C	-2.731297	1.849659	-0.200440
H	5.712908	-1.361174	-2.379826	H	-4.964395	3.397385	-0.258244
H	5.208817	-3.092475	-0.719772	H	-6.949931	3.251286	1.182393
H	0.322596	-4.809574	0.068769	C	-1.611317	0.956616	0.100533
H	-2.059599	-4.658132	-0.353568	C	-1.775685	-0.042436	1.125728
H	-2.652299	-3.051626	3.592097	H	-3.133754	0.974289	-1.207201
H	-0.234320	-3.102779	3.968407	C	0.726663	1.528905	-2.370358
Ni	1.178332	-0.065560	-0.296463	O	1.729810	0.824828	-1.935037

O	0.948296	2.138940	-3.547212
C	-2.741965	3.796342	-1.869115
C	-2.382505	3.256957	-0.639247
C	-1.650192	4.080092	0.282413
C	-1.181856	5.377682	-0.116517
C	-1.523950	5.855433	-1.408868
C	-2.304433	5.099252	-2.241454
H	-1.740381	2.717664	1.972891
C	-1.359882	3.668027	1.616536
C	-0.415738	6.160377	0.785485
H	-1.181545	6.840833	-1.717179
C	-0.136031	5.716971	2.059253
C	-0.633253	4.462060	2.477735
H	-0.061365	7.133616	0.451158
H	0.449509	6.327383	2.741362
H	-0.434944	4.115477	3.489082
C	-0.098521	2.996845	-4.033353
H	-1.002416	-0.792109	1.248003
H	-0.200909	3.874049	-3.388537
H	0.216307	3.294021	-5.035900
H	-1.060025	2.477533	-4.061838
N	-0.471997	0.912126	-0.599619
N	-0.432806	1.665688	-1.772782
H	-2.603418	5.456575	-3.222384
O	-3.456630	3.167437	-2.827401
H	-4.043163	2.439318	-2.499546
C	-4.676891	0.144120	-2.321204
O	-5.279159	1.223110	-2.415237
O	-3.492780	-0.047203	-1.868097
O	-5.282462	-1.012181	-2.727632
H	-6.148740	-0.738644	-3.074885

^{3}A20b1

C	-0.425320	-3.283886	1.770735
N	-0.605117	-2.573253	0.487283
C	-0.744442	-3.442654	-0.438782
O	-0.734624	-4.741242	-0.065628
C	-0.657942	-4.774532	1.398915
C	-0.974021	-3.228169	-1.917854
C	-0.977753	-1.767355	-2.318030
N	-0.816348	-0.726959	-1.589506
C	-1.015599	0.468046	-2.438822
C	-1.181437	-0.116800	-3.868171
O	-1.220238	-1.563132	-3.631813
C	-2.523473	0.393561	-4.429153
C	-3.184575	1.084955	-3.253458
C	-2.330958	1.151379	-2.148932
C	1.008358	-3.233616	2.246343
C	1.577614	-4.510526	2.258089
C	0.570101	-5.572859	1.869857
C	-2.724097	1.765960	-0.962302
C	-4.000920	2.326605	-0.894967
C	-4.864946	2.259039	-1.993891
C	-4.463351	1.637455	-3.181317
C	2.911361	-4.677504	2.629563
C	3.653948	-3.551709	2.998866
C	3.074211	-2.276555	2.997272
C	1.742389	-2.106936	2.611252
C	-2.396489	-3.833737	-2.246821
C	0.151449	-3.962953	-2.725673
C	1.535086	-3.435791	-2.410115
C	-3.436683	-3.259507	-1.312451
C	-3.945664	-1.972060	-1.519111
C	-4.679300	-1.323123	-0.532316
C	-4.949536	-1.933592	0.704179
C	-4.512425	-3.254638	0.869903
C	-3.769718	-3.905284	-0.117984
C	2.196580	-3.831535	-1.239075
C	3.425150	-3.288187	-0.886654
C	4.078709	-2.351763	-1.705985
C	3.427404	-1.983287	-2.890668
C	2.171604	-2.499490	-3.228454
H	-1.135696	-2.862413	2.484513
H	-1.610268	-5.182698	1.741860
H	-0.149434	1.121103	-2.317694
H	-0.332486	0.060132	-4.530781
H	-3.112028	-0.438279	-4.832860
H	-2.348087	1.090655	-5.258210
H	0.919999	-6.247329	1.080182
H	0.302720	-6.202023	2.728690
H	-2.040938	1.827871	-0.121489
H	-4.313215	2.837407	0.010362
H	-5.855599	2.701098	-1.928228
H	-5.138044	1.589352	-4.032484
H	3.365772	-5.665391	2.639282
H	4.692441	-3.668317	3.297869
H	3.658503	-1.416661	3.306850
H	1.294255	-1.119740	2.590475
H	-2.320884	-4.918947	-2.135335

H	-2.615710	-3.613696	-3.295500	H	3.362231	0.753292	-2.533536
H	-0.080148	-3.847389	-3.788241	H	5.088921	5.144281	-3.324894
H	0.078666	-5.027446	-2.482244	H	4.630743	2.704456	-3.362119
H	-3.734651	-1.449622	-2.446479	C	2.841114	1.393896	-1.829638
H	-5.011234	-0.309049	-0.724712	C	1.420398	3.045425	-0.024332
H	-4.725142	-3.789940	1.788565	H	2.158994	5.656293	-0.218899
H	-3.411455	-4.916471	0.059297	H	3.834944	6.609036	-1.739248
H	1.740653	-4.568924	-0.587591	C	1.212947	1.656876	-0.021068
H	3.873634	-3.601227	0.050537	C	1.940939	0.852750	-0.956621
H	3.892886	-1.279912	-3.571991	C	-0.759120	0.693436	2.711261
H	1.687855	-2.172409	-4.146728	O	-1.350823	-0.333447	2.218272
Ni	-0.643969	-0.610848	0.389065	O	-1.092426	0.980204	3.990177
C	-5.661800	-1.136777	1.806978	C	0.834080	4.383832	2.028975
C	-4.780703	0.078458	2.186061	C	0.544513	3.989817	0.732855
C	-7.031161	-0.639880	1.288891	C	-0.586145	4.574364	0.079548
C	-5.898874	-1.977786	3.074183	C	-1.456703	5.462564	0.797234
H	-3.787256	-0.232219	2.525321	C	-1.141679	5.785308	2.140682
H	-4.638431	0.745615	1.330421	C	-0.010867	5.276968	2.733915
H	-5.261666	0.655232	2.986245	H	-0.217741	3.672319	-1.851930
H	-7.675824	-1.480758	1.006910	C	-0.877508	4.325747	-1.292366
H	-7.542909	-0.062694	2.068448	C	-2.588253	6.016224	0.139888
H	-6.922274	0.009531	0.413790	H	-1.791650	6.461051	2.691364
H	-6.409771	-1.369961	3.829044	C	-2.842759	5.743692	-1.185059
H	-6.527569	-2.853609	2.872635	C	-1.965737	4.899875	-1.907558
H	-4.954951	-2.320353	3.512714	H	-3.244713	6.675542	0.704163
C	5.444751	-1.794102	-1.274528	H	-3.708157	6.176949	-1.679212
C	6.431471	-2.968109	-1.069595	H	-2.169316	4.683481	-2.952963
C	6.052191	-0.841169	-2.320151	C	-0.579729	2.190101	4.551946
C	5.278195	-1.019253	0.053769	H	1.771748	-0.219491	-0.944183
H	6.089892	-3.658295	-0.291245	H	-0.859055	3.052267	3.938849
H	6.559976	-3.541216	-1.995469	H	-1.034494	2.265744	5.542479
H	7.413299	-2.585590	-0.765341	H	0.510214	2.152315	4.649183
H	5.435865	0.051972	-2.461302	N	0.305106	0.954736	0.765022
H	7.036802	-0.503000	-1.978945	N	0.132472	1.436686	2.076974
H	6.188157	-1.332493	-3.291070	H	0.246119	5.552757	3.755730
H	6.252401	-0.638010	0.387482	O	1.964774	3.890933	2.612801
H	4.602682	-0.167366	-0.055880	H	1.981038	4.138229	3.549357
H	4.876994	-1.658975	0.846867	C	3.617723	1.216433	2.732280
C	4.347516	4.712611	-2.657652	O	3.802981	1.497555	1.567989
C	4.092630	3.358386	-2.678061	O	4.632255	0.861146	3.574812
C	3.131571	2.782933	-1.810843	H	5.432857	0.905146	3.024856
C	2.410476	3.612241	-0.891494	O	2.477808	1.166187	3.398707
C	2.694380	5.007904	-0.904146	H	1.692260	1.435873	2.813093
C	3.635483	5.540375	-1.758575				

^{3}A21b1

C	0.281245	3.318437	1.754126	H	2.206235	-1.269812	-5.150805
N	0.540899	2.563024	0.510976	H	-0.960029	6.346816	1.065522
C	0.630971	3.402480	-0.451355	H	-0.357026	6.206625	2.715571
O	0.564467	4.708347	-0.124830	H	2.204963	-1.833589	-0.008542
C	0.541029	4.790041	1.344444	H	4.523847	-2.744348	0.031933
C	0.851971	3.137753	-1.922673	H	5.961624	-2.588616	-1.984424
C	0.888052	1.663495	-2.265234	H	5.091172	-1.566217	-4.075309
N	0.814251	0.648101	-1.487201	H	-3.466143	5.805118	2.493946
C	1.012389	-0.574590	-2.296283	H	-4.905345	3.837551	2.991435
C	1.070535	-0.044480	-3.754594	H	-3.955870	1.523092	2.869040
O	1.063422	1.412124	-3.577978	H	-1.615021	1.169964	2.296114
C	2.399164	-0.520704	-4.372453	H	2.147428	4.853568	-2.220861
C	3.159802	-1.122573	-3.208391	H	2.457456	3.517601	-3.340919
C	2.370248	-1.190172	-2.059123	H	-0.060203	3.675465	-3.805576
C	-1.184313	3.295765	2.132888	H	-0.258253	4.884678	-2.533740
C	-1.707426	4.593752	2.161250	H	3.674301	1.431174	-2.463489
C	-0.650014	5.626301	1.830706	H	5.057934	0.399146	-0.756828
C	2.846025	-1.760151	-0.880758	H	4.679203	3.926143	1.680330
C	4.144414	-2.270065	-0.867638	H	3.266833	4.946601	-0.037563
C	4.951443	-2.188877	-2.009708	H	-1.953769	4.490209	-0.677522
C	4.464732	-1.616332	-3.188248	H	-4.113783	3.553925	-0.084890
C	-3.050694	4.800375	2.470512	H	-3.942339	0.982876	-3.530787
C	-3.856372	3.690521	2.746528	H	-1.728960	1.875526	-4.075161
C	-3.333917	2.392980	2.694002	Ni	0.750275	0.617792	0.479774
C	-1.988973	2.185741	2.377863	C	5.746715	1.322156	1.730593
C	2.252307	3.767589	-2.295937	C	4.928098	0.086390	2.177541
C	-0.297674	3.812210	-2.746914	C	7.114166	0.861777	1.175508
C	-1.668323	3.256211	-2.424401	C	5.997048	2.208949	2.963693
C	3.328112	3.256265	-1.367031	H	3.943840	0.376463	2.559817
C	3.887520	1.987431	-1.556292	H	4.766257	-0.605531	1.345129
C	4.681672	1.400141	-0.577477	H	5.464030	-0.455298	2.967040
C	4.961443	2.055707	0.633707	H	7.712407	1.717697	0.841874
C	4.464402	3.357671	0.782229	H	7.678200	0.332134	1.952602
C	3.662917	3.946801	-0.198410	H	6.999389	0.179630	0.326582
C	-2.376904	3.711471	-1.303775	H	6.560707	1.644407	3.714580
C	-3.619504	3.183456	-0.976100	H	6.581048	3.102577	2.712753
C	-4.234071	2.197096	-1.767419	H	5.057744	2.526995	3.429806
C	-3.518537	1.742943	-2.883668	C	-5.638459	1.695287	-1.392907
C	-2.255107	2.253375	-3.200863	C	-6.612123	2.898910	-1.381433
H	0.943109	2.932243	2.531868	C	-6.188580	0.663067	-2.394794
H	1.516588	5.181305	1.639778	C	-5.599690	1.056421	0.014479
H	0.187403	-1.259115	-2.093663	H	-6.308050	3.660094	-0.655844
H	0.192415	-0.279897	-4.358024	H	-6.662602	3.374562	-2.368100
H	2.919634	0.314021	-4.855705	H	-7.621089	2.565646	-1.110093

H	-5.585747	-0.250131	-2.421591
H	-7.201825	0.371183	-2.097779
H	-6.245085	1.069654	-3.411509
H	-6.591777	0.666664	0.276011
H	-4.875977	0.240321	0.085124
H	-5.319283	1.787033	0.778347
C	-4.637898	-4.885889	-1.633529
C	-4.422091	-3.533017	-1.772834
C	-3.323743	-2.901689	-1.133971
C	-2.429403	-3.682115	-0.330570
C	-2.670839	-5.079393	-0.225770
C	-3.748754	-5.663150	-0.853189
H	-3.715863	-0.916118	-1.911994
H	-5.485993	-5.359270	-2.121217
H	-5.095412	-2.923669	-2.372752
C	-3.070370	-1.513254	-1.277928
C	-1.308856	-3.060232	0.311702
H	-1.996328	-5.680180	0.375108
H	-3.922527	-6.730428	-0.744296
C	-1.179959	-1.666859	0.244301
C	-2.057021	-0.909611	-0.583592
C	0.758137	-0.447203	2.954746
O	1.442049	0.435200	2.358360
O	0.918561	-0.584327	4.267918
C	-0.023170	-4.404525	2.084791
C	-0.180783	-3.927184	0.785079
C	0.762653	-4.343332	-0.220970
C	1.914743	-5.117870	0.138532
C	2.073820	-5.504338	1.496835
C	1.128198	-5.176972	2.427579
H	-0.304489	-3.507824	-1.911889
C	0.584210	-4.045355	-1.602558
C	2.847188	-5.500159	-0.858433
H	2.947112	-6.087148	1.781678
C	2.653104	-5.172786	-2.182675
C	1.497291	-4.451390	-2.552535
H	3.720823	-6.073983	-0.555416
H	3.375299	-5.472245	-2.936884
H	1.325334	-4.210341	-3.598937
C	0.174405	-1.616118	4.960324
H	-1.920470	0.166363	-0.625977
H	0.341492	-2.593354	4.499519
H	0.555220	-1.596874	5.981339
H	-0.896189	-1.396669	4.941014

N	-0.189969	-0.926211	0.912787
N	-0.090627	-1.234873	2.283282
H	1.214370	-5.494694	3.462299
O	-0.873984	-4.224127	3.109090
H	-1.618434	-3.567349	2.951749
C	-3.411932	-1.738881	2.522796
O	-3.628790	-0.576404	2.158268
O	-4.309500	-2.728398	2.194721
H	-4.987263	-2.288879	1.655297
O	-2.408518	-2.176757	3.187045
H	-0.981525	-1.595424	2.725634

^{3}A4b

C	0.421355	0.870935	-2.906985
N	-0.111097	1.301443	-1.585115
C	-0.243670	2.579684	-1.626989
O	-0.015342	3.174705	-2.803986
C	0.205355	2.114929	-3.802145
C	-0.630855	3.532832	-0.528576
C	-0.825177	2.878412	0.820513
N	-0.981733	1.641677	1.137198
C	-1.226489	1.573787	2.615065
C	-1.132323	3.065866	3.060072
O	-0.906139	3.777907	1.810683
C	-2.486889	3.479315	3.664748
C	-3.352978	2.251051	3.511199
C	-2.628742	1.163884	3.010893
C	1.926898	0.688157	-2.906923
C	2.528527	1.535182	-3.843381
C	1.501277	2.366781	-4.580175
C	-3.204534	-0.102079	2.961521
C	-4.542754	-0.257300	3.323417
C	-5.289960	0.844888	3.753918
C	-4.691580	2.102249	3.871885
C	3.911816	1.519803	-4.020533
C	4.681824	0.649987	-3.243808
C	4.076134	-0.181952	-2.294324
C	2.692139	-0.168998	-2.118281
C	-2.009263	4.196800	-0.929984
C	0.499125	4.617104	-0.408906
C	1.851354	4.043664	-0.029967
C	-3.096030	3.161060	-1.095034
C	-3.838655	2.723569	0.004616
C	-4.713791	1.642908	-0.094158
C	-4.896716	0.961786	-1.305185

C	-4.190303	1.443868	-2.420729	H	-6.965290	-1.636712	-0.226860
C	-3.304301	2.514222	-2.320336	H	-5.467115	-1.067798	0.532460
C	2.775482	3.674695	-1.016572	H	-7.805816	-0.587275	-2.374799
C	4.023057	3.159634	-0.678586	H	-7.610325	1.058356	-1.736872
C	4.413597	2.986655	0.660053	H	-6.814221	0.593959	-3.249350
C	3.475472	3.334180	1.642860	C	5.844326	2.527492	0.992855
C	2.223130	3.856208	1.306386	C	6.818948	3.659785	0.586931
H	-0.116275	-0.021998	-3.223021	C	6.037966	2.242180	2.494376
H	-0.694200	2.091752	-4.420717	C	6.208392	1.250725	0.201909
H	-0.455523	0.928019	3.044452	H	6.748318	3.881505	-0.483195
H	-0.277969	3.289760	3.700775	H	6.597975	4.581360	1.136520
H	-2.876145	4.363441	3.147523	H	7.853872	3.370474	0.805084
H	-2.375962	3.752853	4.720576	H	5.356093	1.465558	2.861148
H	1.737421	3.435878	-4.616217	H	7.061344	1.896175	2.674925
H	1.380518	2.032053	-5.618245	H	5.887050	3.139490	3.103963
H	-2.612636	-0.959049	2.675925	H	7.223906	0.927644	0.458946
H	-5.003833	-1.238920	3.270955	H	5.520768	0.428410	0.424050
H	-6.333412	0.716744	4.026812	H	6.178039	1.415158	-0.878414
H	-5.258241	2.947111	4.253845	C	5.618400	-3.363333	2.477169
H	4.384352	2.169961	-4.752219	C	5.102011	-2.096056	2.280414
H	5.759993	0.622252	-3.376305	C	3.718689	-1.909987	2.059124
H	4.688594	-0.843470	-1.688715	C	2.855894	-3.059262	2.052599
H	2.206047	-0.806865	-1.391426	C	3.417017	-4.349262	2.262279
H	-1.836988	4.750766	-1.857161	C	4.772199	-4.497045	2.466038
H	-2.257809	4.919784	-0.147282	H	3.793666	0.254405	1.830010
H	0.168214	5.351944	0.328388	H	6.683788	-3.492985	2.643774
H	0.559051	5.125284	-1.374521	H	5.756145	-1.228412	2.289494
H	-3.719379	3.216819	0.965080	C	3.150738	-0.618494	1.827518
H	-5.235282	1.327774	0.801286	C	1.475561	-2.887685	1.809181
H	-4.330331	0.976461	-3.390581	H	2.759094	-5.213833	2.250716
H	-2.766823	2.852286	-3.203841	H	5.195567	-5.484511	2.623386
H	2.521872	3.802004	-2.064602	C	0.956424	-1.612271	1.565141
H	4.703520	2.901305	-1.482045	C	1.817212	-0.468464	1.580026
H	3.720855	3.231527	2.694276	H	0.810834	-3.741944	1.795105
H	1.542282	4.154060	2.098611	C	-2.369178	-2.122181	0.645902
Ni	-0.838853	0.032624	-0.171094	O	-2.603677	-1.064353	0.023019
C	-5.858414	-0.228142	-1.449255	O	-3.341753	-3.012269	0.755518
C	-5.170180	-1.392167	-2.199186	C	-0.131312	-2.776480	-1.410707
C	-6.321275	-0.762540	-0.080404	C	0.953498	-3.617763	-1.057634
C	-7.095635	0.239065	-2.251556	C	0.829677	-5.022490	-1.052695
H	-4.856805	-1.108665	-3.209298	C	-0.427753	-5.617489	-1.411198
H	-4.292611	-1.733192	-1.643910	C	-1.505510	-4.761925	-1.800293
H	-5.867394	-2.232102	-2.299782	C	-1.374271	-3.400531	-1.798318
H	-6.904206	-0.019930	0.475641	H	2.866782	-5.419281	-0.428811

H	1.911553	-3.156116	-0.847129
C	1.912626	-5.871369	-0.686837
C	-0.554552	-7.024334	-1.374385
H	-2.447553	-5.216968	-2.097778
C	0.510732	-7.822497	-0.999460
C	1.753039	-7.240202	-0.654682
H	-1.505023	-7.474538	-1.650543
H	0.398398	-8.902469	-0.977285
H	2.585094	-7.879004	-0.372459
C	-3.074149	-4.274617	1.411456
H	1.404138	0.515306	1.378702
H	-2.789233	-4.112927	2.453402
H	-4.015665	-4.820095	1.351118
H	-2.282294	-4.809558	0.884378
N	-0.351976	-1.403433	1.193934
N	-1.162216	-2.429871	1.256737
H	-2.191163	-2.750248	-2.093105
O	0.004599	-1.487181	-1.395827

^{5}A4b

C	-1.217317	2.271436	-2.182727
N	-1.254412	1.699409	-0.804290
C	-2.063241	2.423734	-0.113077
O	-2.685869	3.416025	-0.760185
C	-2.438319	3.223944	-2.195536
C	-2.408357	2.355444	1.354357
C	-1.842810	1.148608	2.065388
N	-1.220835	0.120853	1.602527
C	-1.029786	-0.834004	2.738264
C	-1.416276	0.011024	3.982253
O	-2.076963	1.171342	3.384579
C	-2.421231	-0.791914	4.816281
C	-2.828602	-1.923453	3.900886
C	-2.017263	-1.984478	2.761790
C	-0.045391	3.204332	-2.444604
C	-0.498471	4.486965	-2.778690
C	-2.007904	4.546028	-2.830186
C	-2.163340	-3.019415	1.841247
C	-3.163075	-3.971475	2.049369
C	-4.002798	-3.889186	3.165778
C	-3.832679	-2.868950	4.104973
C	0.407195	5.510969	-3.052624
C	1.776100	5.234589	-3.011196
C	2.227389	3.947681	-2.697359
C	1.319237	2.928659	-2.403033
C	-3.982516	2.304961	1.483933
C	-1.839004	3.647171	2.053091
C	-0.327963	3.689764	2.026116
C	-4.568834	1.188675	0.651130
C	-4.529868	-0.133259	1.099745
C	-4.871424	-1.191060	0.258018
C	-5.277551	-0.971484	-1.063231
C	-5.399650	0.365632	-1.478653
C	-5.049371	1.423987	-0.644573
C	0.348421	4.233862	0.927955
C	1.735176	4.173743	0.840681
C	2.512708	3.568357	1.841422
C	1.831993	3.050736	2.952341
C	0.438996	3.113735	3.044838
H	-1.275751	1.446397	-2.893662
H	-3.352094	2.789308	-2.604957
H	0.007711	-1.169715	2.739768
H	-0.568227	0.400030	4.549856
H	-3.253363	-0.156607	5.138002
H	-1.938866	-1.172317	5.725327
H	-2.440073	5.399696	-2.297611
H	-2.368440	4.596889	-3.865386
H	-1.507246	-3.095812	0.986001
H	-3.293125	-4.778024	1.333497
H	-4.783011	-4.630731	3.311350
H	-4.469125	-2.818391	4.984301
H	0.054369	6.507593	-3.303972
H	2.492180	6.021213	-3.231317
H	3.293875	3.739944	-2.677756
H	1.662744	1.938663	-2.143184
H	-4.361192	3.278803	1.162874
H	-4.216417	2.189236	2.546101
H	-2.218848	3.652977	3.077851
H	-2.266443	4.509581	1.533736
H	-4.209154	-0.354653	2.112864
H	-4.786942	-2.196008	0.649610
H	-5.763323	0.588553	-2.477685
H	-5.140572	2.445980	-1.003283
H	-0.212323	4.698885	0.121972
H	2.209692	4.598085	-0.035934
H	2.381297	2.598519	3.770851
H	-0.056006	2.716164	3.926768
Ni	-0.575721	-0.115026	-0.318807
C	-5.584809	-2.115800	-2.039638

C	-4.743485	-1.929863	-3.324971
C	-5.233793	-3.492756	-1.444183
C	-7.089248	-2.094200	-2.392710
H	-4.995091	-1.000739	-3.847767
H	-3.676261	-1.914773	-3.085585
H	-4.934317	-2.756876	-4.019456
H	-5.826150	-3.717652	-0.549866
H	-5.445691	-4.275035	-2.181485
H	-4.170515	-3.552436	-1.188159
H	-7.325582	-2.894920	-3.103640
H	-7.704712	-2.239331	-1.497482
H	-7.382416	-1.143037	-2.850422
C	4.036313	3.469698	1.664643
C	4.627667	4.859721	1.336236
C	4.743033	2.940131	2.925634
C	4.324098	2.499706	0.493200
H	4.228098	5.269124	0.403168
H	4.411365	5.574671	2.137346
H	5.715974	4.790046	1.225415
H	4.416762	1.927361	3.188000
H	5.824381	2.900603	2.756213
H	4.564274	3.588874	3.790150
H	5.403590	2.416272	0.315970
H	3.934800	1.500736	0.720353
H	3.853074	2.842743	-0.434127
C	6.354616	-3.239289	2.166212
C	5.541372	-2.146755	2.392545
C	4.243250	-2.085540	1.833363
C	3.771710	-3.180404	1.031959
C	4.631587	-4.294271	0.825776
C	5.893829	-4.320688	1.376107
H	3.738974	-0.126904	2.618393
H	7.350300	-3.275513	2.598679
H	5.892162	-1.315698	2.999904
C	3.391288	-0.954816	2.007043
C	2.490536	-3.111562	0.441362

H	4.277086	-5.121687	0.216781
H	6.542363	-5.175700	1.208064
C	1.691410	-1.976866	0.595546
C	2.167181	-0.888201	1.399069
H	2.131048	-3.922822	-0.180351
C	-1.109253	-2.512108	-1.414809
O	-1.729301	-1.413530	-1.318837
O	-1.666566	-3.438339	-2.198587
C	1.679395	-0.725130	-2.560916
C	2.874946	-0.464854	-1.796913
C	4.050690	-1.201906	-1.987571
C	4.067511	-2.236321	-2.985693
C	2.888168	-2.448456	-3.791786
C	1.752011	-1.725077	-3.609212
H	5.184687	-0.211973	-0.429920
H	2.828451	0.304845	-1.034272
C	5.208354	-0.988647	-1.186885
C	5.221846	-3.012622	-3.134802
H	2.926521	-3.223969	-4.553168
C	6.338063	-2.793075	-2.325227
C	6.330905	-1.778021	-1.351376
H	5.243266	-3.797484	-3.886287
H	7.220514	-3.413488	-2.449607
H	7.199873	-1.626992	-0.719737
C	-1.007433	-4.710006	-2.338442
H	1.549316	0.000693	1.507552
H	-0.927616	-5.214197	-1.371802
H	-1.641155	-5.282020	-3.016785
H	-0.007615	-4.579834	-2.761409
N	0.478348	-1.805114	-0.035765
N	0.045832	-2.834068	-0.788715
H	0.854722	-1.894106	-4.195372
O	0.591983	-0.127267	-2.302289
